# Genome-wide identification of long noncoding RNA genes and their potential association with mammary gland development in water buffalo

**DOI:** 10.5713/ab.22.0120

**Published:** 2022-06-30

**Authors:** Yuhan Jin, Yina Ouyang, Xinyang Fan, Jing Huang, Wenbo Guo, Yongwang Miao

**Affiliations:** 1 Faculty of Animal Science and Technology, Yunnan Agricultural University, Kunming 650201, Yunnan, China; 2 Yunnan Animal Science and Veterinary Institute, Kunming 650224, China

**Keywords:** Lactation Stages, lnc-bbug14207, LncRNAs Identification, Mammary Gland Development, RNA-seq, Water Buffalo

## Abstract

**Objective:**

Water buffalo, an important domestic animal in tropical and subtropical regions, play an important role in agricultural economy. It is an important source for milk, meat, horns, skin, and draft power, especially its rich milk that is the great source of cream, butter, yogurt, and many cheeses. In recent years, long noncoding RNAs (lncRNAs) have been reported to play pivotal roles in many biological processes. Previous studies for the mammary gland development of water buffalo mainly focus on protein coding genes. However, lncRNAs of water buffalo remain poorly understood, and the regulation relationship between mammary gland development/milk production traits and lncRNA expression is also unclear.

**Methods:**

Here, we sequenced 22 samples of the milk somatic cells from three lactation stages and integrated the current annotation and identified 7,962 lncRNA genes.

**Results:**

By comparing the lncRNA genes of the water buffalo in the early, peak, and late different lactation stages, we found that lncRNA gene *lnc-bbug14207* displayed significantly different expression between early and late lactation stages. And *lnc-bbug14207* may regulate neighboring milk fat globule-EGF factor 8 (*MFG-E8*) and hyaluronan and proteoglycan link protein 3 (*HAPLN3*) protein coding genes, which are vital for mammary gland development.

**Conclusion:**

This study provides the first genome-wide identification of water buffalo lncRNAs and unveils the potential lncRNAs that impact mammary gland development.

## INTRODUCTION

The human genome contains tens of thousands long non-coding RNA (lncRNA) genes [1]. According to their genomic position, lncRNA can be classified as long intronic non-coding RNA, antisense RNAs and long intergenic non-coding RNAs (lincRNAs). LncRNA was considered as the “noise” produced by transcription, so it has been ignored. Later studies have shown that some lncRNAs play important roles in basic biological processes, including transcriptional regulation [2–4], epigenetic regulation [5,6], genomic imprinting [7] and pluripotency preservation [8]. However, only a small number of lncRNA functions have been determined, and the amount of functional noncoding sequences is still a controversial issue.

Although the identification and functional research of lncRNA are developing rapidly, many lncRNAs have been proved to be important regulators in life, most of them are still focused on mice and humans. Studies such as Xist, GM12371 and Malat1 focus on laboratory animals and human cells, but less on domestic animals. Among domestic animals, there are relatively more studies on pigs as experimental animals, and the number of lncRNAs identified is also more than that of other domestic animals [9], but the research on their functions is still relatively lacking. In cattle, Chinese scientists identified the RNA sequence, lncRNA and protein coding genes from the mammary gland samples of Chinese Holstein cows, and ultimately obtained 1,657 lncRNA transcripts from 1,181 candidate lncRNA sites [10]. This is also the first time to systematically identify lncRNA from RNA-seq data of mammary gland tissues of dairy cows in different lactation periods.

Water buffalo is an important source of meat, horn, skin and draft power in tropical and subtropical regions [11]. Furthermore, water buffalo provides more than 5% of the world's milk. Compared with cattle milk, water buffalo milk contains more fat, lactose, protein and minerals [12]. At present, the research on mammary gland development of water buffalo mainly focuses on protein coding genes, such as milk fat globule-EGF factor 8 (*MFG-E8*) [13], cardiac alpha-actin (*ACTC1*) and gap junction protein delta 2 (*GJD2*) [14]. Whereas, lncRNA related to mammary gland development of water buffalo is still unrevealed.

Genome-wide identification of lncRNAs involved in buffalo mammary gland development and lactation will help to construct the molecular network regulating lactation in buffalo mammary epithelial cells, and identify key functional genes in the process of milk protein synthesis and lipid metabolism, which can provide a basis for the formulation of molecular breeding programs for high-quality dairy buffalo population. In this study, we sequenced 22 samples of milk somatic cell at three different lactation stages in water buffalo, and identified 7,962 lncRNA genes encoded by 10,789 lncRNA transcripts through integrating the known annotation [15]. By comparing the expression level of lncRNA genes in the early, peak and late lactating stages, we proposed that *lnc-bbug14207* may regulate neighboring *MFG-E8* and hyaluronan and proteoglycan link protein 3 (*HAPLN3*) genes and contribute to mammary gland development. These results facilitate the study of lncRNA genes in water buffalo and will provide the new clue for elucidating the mechanism of mammary gland development and lactation traits in water buffalo.

## MATERIALS AND METHODS

### Animals

All the experimental procedures were approved by the Institutional Animal Ethics and Use Committee of Yunnan Agricultural University (No. YNAU2019llwyh020).

The samples of Binglangjiang buffaloes were collected from the farm of Bafule Binglangjiang buffalo elite breeding Co., Ltd, Tengchong County, Yunnan Province, China. In order to reduce errors, this study minimized the differences between samples in different periods and increased the number of samples in each period as much as possible. The sampling water buffaloes with the same parity (the third parity) and similar age were fed on the same diet and environment, and drinking clean water freely and they were divided into three groups according to their lactating stage: early lactating group (15 to 45 days after calving), peak lactating group (46 to 180 days after calving) and late lactating group (180 days after calving). A total of 22 samples of milk somatic cells were obtained, including six from early lactating buffalo, eight from peak lactating buffalo and eight from late lactating buffalo.

### Isolation of milk somatic cells

The methods of separating milk somatic cells mainly refer to the conventional centrifugation method [16] with some modification. The operation steps are as follows: i) Ethylene diamine tetraacetic acid (EDTA) was added in 250 mL fresh buffalo milk samples with a final concentration of 0.5 mM to prevent casein precipitation. The samples were centrifuged at 4°C for 15 min at 2,000×g. ii) The upper fat layer in the centrifugal cup and the skimmed milk in the middle layer were discarded. Then, the milk fat on the cup wall was wiped with an alcohol cotton ball. The cell precipitate at the bottom was resuspended with 4 mL of 1×phosphate-buffered saline solution (pH7.2) containing 0.5 mM EDTA. Cell suspension was transferred into a 5 mL cryogenic tube without RNase, and centrifuged at 4°C at 1,500×g for 10 min. iii) After discarding the upper suspension, the cells were suspended and washed again, and centrifuged for 10 min at 1,500×g at 4°C. Before centrifugation, the milk somatic cell count and activity were measured. iv) After the upper suspension was completely abandoned, the cells were immediately placed in liquid nitrogen, and then stored in a −80°C refrigerator for the preservation. All the above steps should be performed on ice.

### RNA extraction

Total RNA from the milk somatic cells of buffalo was extracted and purified using a combination of TRIZOL (Invitrogen Life Technologies, Carlsbad, CA, USA) and the UNIQ-10 column TRIZOL total RNA extraction kit (Sangon Bioengineering, Shanghai, China) as the recommended protocol.

### RNA sequencing

**Figure 1:**
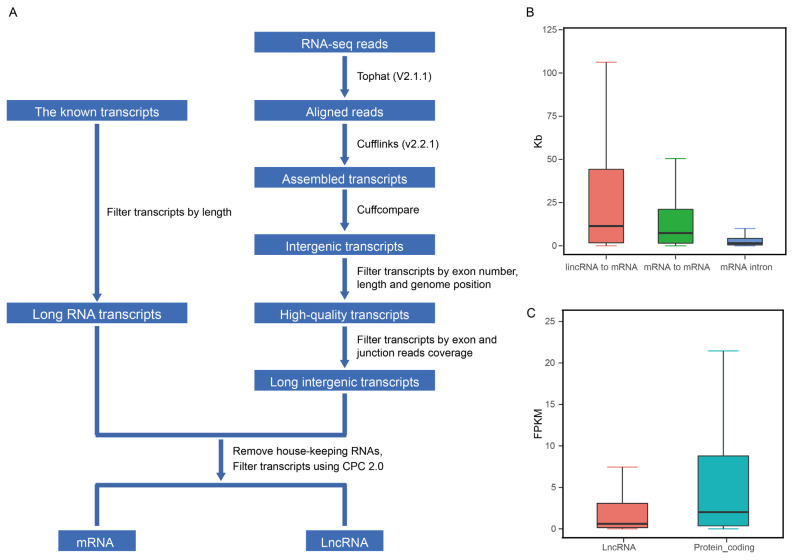
Genome-wide identification and characterization of lncRNA genes in the water buffalo. (A) Pipeline for the identification of lncRNAs. (B) Comparison of the mRNA–lncRNA intervals, mRNA–mRNA intervals, and length of mRNA introns. (C) Expression levels of lncRNA and protein-coding genes.

The RNA-seq was performed by Annoroad Gene Technology (China). Ribosomal RNA was removed by Epicentre Ribo-zero rRNA Removal Kit (Epicentre, Madison, WI, USA) from total RNA, and rRNA free residue was cleaned up by ethanol precipitation. Sequencing libraries were generated using the rRNA-depleted RNA by NEBNext Ultra Directional RNA Library Prep Kit for Illumina (NEB, Ipswich, MA, USA), and products were purified (AMPure XP system) and library quality was assessed on the Agilent Bioanalyzer 2100 system (Agilent Technologies, Palo Alto, CA, USA). The RNA-seq reads were mapped to the water buffalo reference genome (GenBank assembly accession: GCA_003121395.1) [15] using TopHat2 (v2.1.1) (http://ccb.jhu.edu/software/tophat) (Figure 1A) [17]. The mapped reads were assembled using Cufflinks (v2.0.2) (http://cole-trapnell-lab.github.io/cufflinks/releases/v2.0.2) (Figure 1A) [18]. Cuffcompare (http://cole-trapnell-lab.github.io/cufflinks/releases/v2.0.2) was used to obtain the transcripts of the intergenic region for each sample (Figure 1A) [18]. Transcripts of each sample were filtered according to the criteria: i) RNA-seq data must cover at least 80% of the predicted transcript exon nucleotides; ii) For each predicted splicing site, at least three reads support the splicing site (Figure 1A) [9]. Transcripts that meet the above criteria are used for subsequent analysis.

### Identification of lncRNAs

The putative transcripts identified from RNA sequencing should meet the following criteria (Figure 1A): i) The exon number of each transcript is greater than or equal to two; ii) The length of the transcript is greater than 200 nucleotides; iii) The closest protein coding gene to the transcript must be greater than or equal to 500 bp [9]. Further, we combined the transcripts identified from RNA-seq and the known annotation [15], and removed the house-keeping RNAs. We used Coding Potential Calculator (CPC2) to evaluate the coding ability of each transcript [19]. The transcripts labelled with “noncoding” predicted by CPC2 were considered long non-coding RNA and used for further analysis. Gene ontology (GO) enrichment analyses were conducted using DAVID (https://david.ncifcrf.gov) [20] for the set of protein-coding genes adjacent to these lncRNAs identified in this study. And the results were visualized by using R package ggplot2 function.

### Analysis the expression level of lncRNA and protein-coding genes

The RNA-seq reads from three stages were mapped to the reference genome by TopHat using the prarmeter ‘-no-novel-juncs’. SummarizeOverlaps was used to assess reads count for each gene with default mode of “Union”. We used Mann–Whitney test to analysis the distances between lncRNA genes and their closest protein coding genes and the comparison of lncRNA and protein coding gene. In addition, we also used the ‘prcomp’ function of the ‘stats’ R package for principal component analysis (PCA). Deseq2 (v1.28.1) was used to identify the differentially expressed genes for each pairwise comparison of samples [21]. We defined differentially expressed lncRNA genes by using Deseq2 with the parameter padj<0.05 according to their expression levels across various lactating stages. Additionally, we conducted an analysis of the differentially expressed protein and lncRNA genes in the 500-kb window surrounding lncRNA gene.

## RESULTS

Previous studies indicated that the transcriptome of milk somatic cells could be representative of that of mammary gland tissue [22]. Thus, we sequenced 22 samples of milk somatic cells in the early, peak and late lactation of water buffalo. And, from 6.9 to 13.1 Gb reads could be mapped to the water buffalo reference genome (GenBank assembly accession: GCA_003121395.1)[15] using Tophat2 (v2.1.1) with default parameters (Table 1) [17]. The mapped reads were used to assemble the transcripts using Cufflinks (v2.0.2) [18] in water buffalo genome (Figure 1A) [15].

### Identification of water buffalo lncRNAs

**Figure 2:**
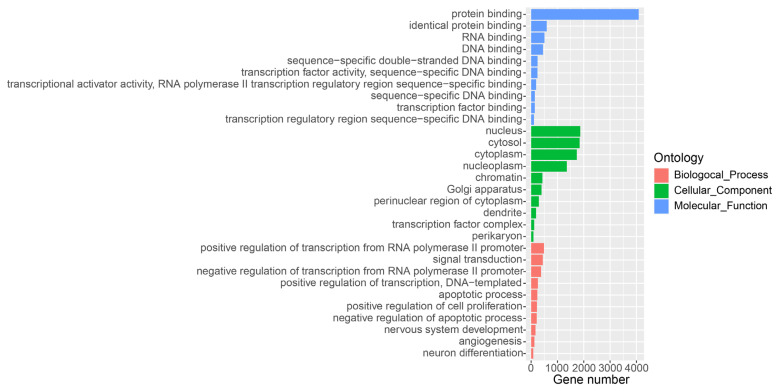
Gene ontology (GO) analysis of protein-coding genes in proximity to lncRNAs. The protein-coding genes adjacent to lncRNAs are divided into the following three biological segments: biological process, cellular components and molecular function. Each term with a p value <0.05 was considered significantly enriched and the top 10 significant enriched terms in three biological segments are listed.

To comprehensively detect lncRNA genes participated the lactation process of water buffalo, we used the mapped RNA-seq data and the known RNA transcripts [15], and performed the identification of lncRNAs using criteria as pervious study in the pig (Figure 1A) [9]. A total of 10,789 lncRNA transcripts encoded by 7,962 genes was identified in the water buffalo genome (Supplementary Table S1). The distances between lncRNA genes and their closest protein coding genes were larger than the lengths of the introns in the protein-coding genes (Mann–Whitney P< 2.2×10^−16^; Figure 1B). This result indicated that the lncRNA transcripts identified from RNA-seq data were independent transcripts and not the exon of protein-coding genes. The expression level of lncRNA is lower than that of protein-coding genes, which are consistent with previous studies (Mann–Whitney P< 2.2×10^−16^; Figure 1C) [9]. Further, we performed GO analysis for the adjacent protein-coding genes of lncRNAs found that they were strikingly enriched in the positive regulation of transcription, signal transduction and apoptotic process of GO terms of the biological processes (Figure 2). According to the cellular component terms, they were principally enriched in nucleus, cytosol, cytoplasm and nucleoplasm (Figure 2). In addition, adjacent protein-coding genes of lncRNAs were enriched in molecular function terms including protein binding, RNA binding and DNA binding (Figure 2).

### Differential expression of lncRNA genes at different lactation stages of water buffalo

**Figure 3:**
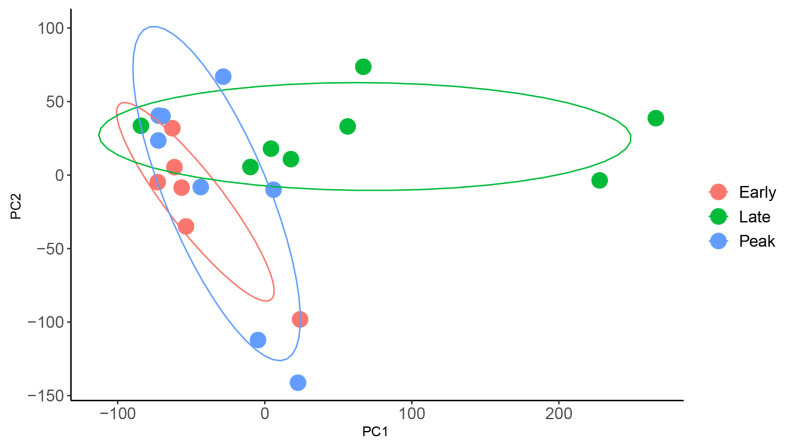
Principal component analysis (PCA) plot analysis of the samples from 3 lactation periods.

**Figure 4:**
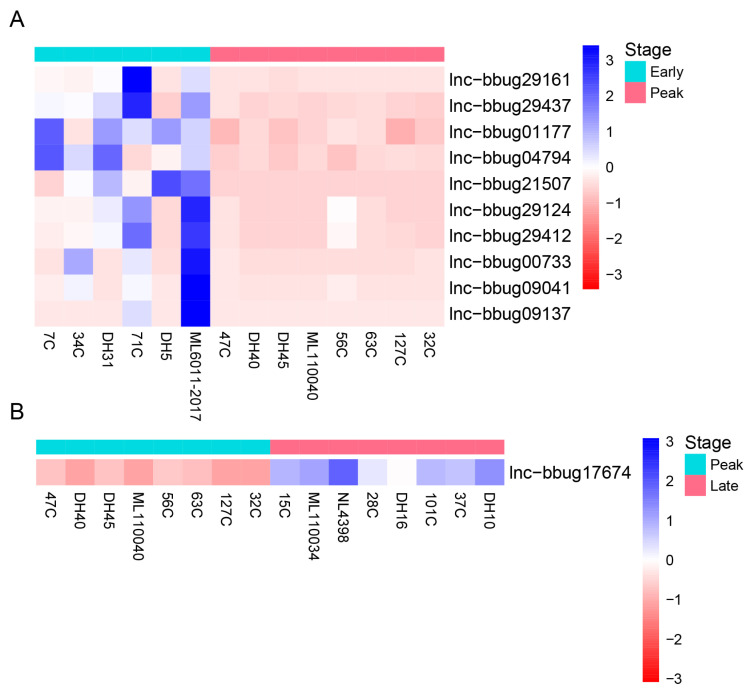
Differential expression of lncRNA genes at different lactation stages of water buffalo. (A) Differential expression of lncRNA genes between early and peak lactation stages (B) Differential expression of lncRNA genes between peak and late lactation stages. Heatmap showing expression abundance of lncRNA genes showing significant differences in expression. Read count of lncRNA genes were measured by RNA-seq. Genes were clustered by hierarchical clustering.

**Figure 5:**
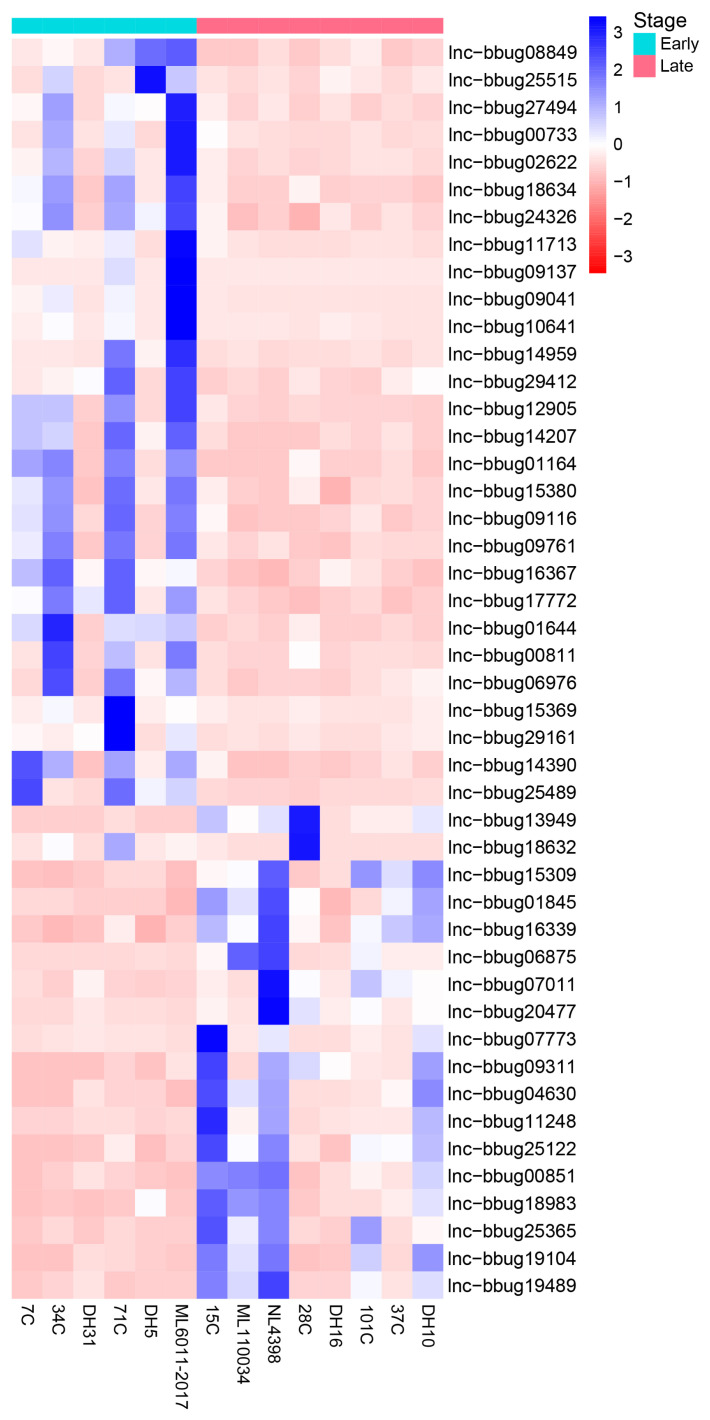
Differential expression of lncRNA genes at early and late lactation stages of water buffalo. Heatmap showing expression abundance of lncRNA genes showing significant differences in expression. Read count of lncRNA genes were measured by RNA-seq. Genes were clustered by hierarchical clustering.

For the understanding of mammary gland development in water buffalo, it is necessary to know the functional genes and their biological network that regulate mammary gland development. The PCA analysis indicated no obvious separation between the 3 lactation periods using the expression level of all protein-coding and lncRNA genes, revealing these samples could be used for the differently expression analysis (Figure 3). Thus, we analyzed the expression level of lncRNAs in milk somatic cells of buffalo at three lactation stages and detected the significantly differential expression genes in different lactating stages. And, we found that 10 lncRNA genes were differentially expressed between the early and peak stages (Figure 4A; Supplementary Table S2) and 46 lncRNA genes were differentially expressed between early and late stages (Figure 5; Supplementary Table S3). One lncRNA gene was found between peak and late stages (Figure 4B; Supplementary Table S4). The results indicate that further analyses need to determine whether the lncRNA genes participate the development of mammary gland, and the different expression of lncRNA genes causes the different milk production traits.

### Potential regulation mechanism of water buffalo lncRNA genes

**Figure 6:**
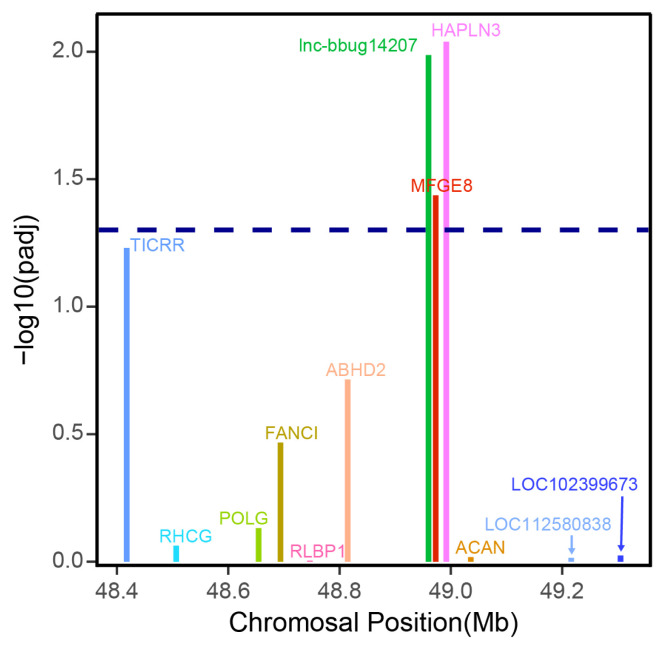
Expression differences of lncRNA lnc–bbug14207 and mRNA genes in the surrounding 500 kb of the lncRNA gene. The x axis shows the genomic positions of these genes. A threshold of padj 0.05 is indicated by the dashed line.

Previous studies suggested that lncRNAs may regulate the expression levels of neighboring protein-coding genes [23]. In this study, we only focused on the lncRNA that may regulate the adjacent protein coding gene. LncRNA gene *lnc-bbug14207* displayed higher expression in early lactation stage compared with late lactation stages (Figure 5; Supplementary Table S3). The analysis of the protein and lncRNA genes in the 500-kb window surrounding this lncRNA gene showed that the lncRNA gene *lnc-bbug14207* may regulate closest neighboring *MFG-E8* protein coding gene (Figure 6), and *MFG-E8* displayed differential expression between early and late lactation stage, with a higher expression in early lactation stage. Previous studies reported that *MFG-E8* is a glycoprotein found in lacteal gland [24]. This protein is critical for the development of the mammary gland and phagocytic clearance of apoptotic cells [25–27]. Another neighboring gene *HAPLN3* also displayed a significantly different expression between early and late stage (Figure 6). *HAPLN3* is associated with cell junction and thus may influence mammary gland morphogenesis in different lactation stages [28,29]. This observation implies that lncRNA gene *lnc-bbug14207* may regulate neighboring *MFG-E8* and *HAPLN3* genes and play roles mammary gland development in water buffalo. Thus, *lnc-bbug14207* gene may could explain the different mammary gland morphogenesis in the different lactation stages. However, the function experiment and the underline regulation relationship need to be further studied.

## DISCUSSION

Milk is one of important food for human diet, which contains all the nutrients required for the newly born human baby. Water buffaloes are one of largest source of milk supply in tropical and subtropical regions [11]. Many studies have focused on the mammary gland development and milk production traits in water buffaloes in regard to *MFG-E8* [13], *ACTC1*, and *GJD2* [14]. However, few study focused on the genome-wide identification of lncRNA genes, and studies on the regulation of lncRNA genes on mammary gland development and milk production traits in buffalo are also rarely reported. In this study, we used high-throughput RNA sequencing data generated from 22 samples of milk somatic cells in the early, peak and late lactation of water buffalo and known annotation to detect lncRNA genes in genome-wide scale. And, we further analyze the transcriptome differences between the early, peak and late lactation of milk somatic cells. We want to understand the molecular mechanism of mammary gland development to help the molecular breeding of water buffalo.

The RNA-seq platform was used for genome-wide identification of lncRNAs. In mammals, 58,648, 19,873 and 14,429 lncRNAs have been identified for human [1], mouse [30], and pig [31] using RNA-seq datasets, respectively. The integrity of the lncRNA transcripts assemblied from RNA-seq data largely depends on the depth of sequencing and the expression levels of lncRNAs in the samples. Sequencing depth varied across the samples, therefore, strict criteria that depend on the coverage of the junctions and exons were defined. Our study identified thousands of lncRNAs for future functional and artificial selection research in water buffalo.

During the development of mammary gland, the cell composition experienced a series of changes. Previous studies indicated that several protein-coding genes and lncRNA genes show differential levels of expression during mammary gland development in Holstein cows [32,33], whereas, no lncRNAs were identified in the water buffalo. LncRNAs, however, have previously been shown to be important in the mammalian milk traits [34,35]. As expected, our analysis identified 46 lncRNA genes that showed significant differences in expression between early and late lactation. Among these lncRNAs that show significant differential expression, one lncRNA, lnc-bbug14207, which is adjacent to *MFG-E8* and *HAPLN3* protein coding gene. Differential expression of lnc-bbug14207, *MFG-E8* and *HAPLN3* suggests that lnc-bbug14207 may be a regulatory element for *MFG-E8* and *HAPLN3*. Further experimental study is required to ascertain the regulatory relationship.

Efferocytosis is a prominent function of MFG-E8, which acts as a bridge between apoptotic and phagocytic cells, thus coordinating the engulfment of apoptotic cells [36,37]. MFG-E8 has anti-inflammatory effects, and also plays roles in wound healing, arterial remodeling, and angiogenesis, enhancing tumorigenicity and tumor metastasis [38,39]. This protein is also a peripheral membrane glycoprotein that is abundantly expressed in lactating mammary glands and secreted together with fat globules [40]. Most importantly, MFG-E8 have demonstrated the importance of development regulation in the activity of mammary gland. It is expressed in both luminal and myoepithelial cells, and promotes mammary gland branching morphogenesis by activating mitogen activated protein kinases in myoepithelial cells [25–27]. A previous study has demonstrated that MFG-E8 can activate signaling pathways in mammary epithelial cells even in the absence of interacting myoepithelial cells, and the expression of MFG-E8 in immortalized mammary epithelial cells promotes their growth and branching in three-dimensional collagen matrices and induces the expression of cyclins D1/D3 and N-cadherin [41]. HAPLN3, belonging to the hyaluronan and proteoglycan link protein family, functions in the aggregation of proteoglycan with hyaluronic acid and cell adhesion [42,43], and it is abundantly and widely expressed in most tissues, including mammary gland tissue. The expression of *HAPLN3* gene has significantly increased in the tissue of breast cancer compared to normal breast tissue, which indicates that up-expression of *HAPLN3* could contribute to the development of breast cancer [44]. *HAPLN3* and *MFG-E8* genes are closely related to the occurrence of breast cancer, which indicates that they are likely important to the development of mammary gland. In this study, we found that lnc-bbug14207 may regulate the adjacent protein coding genes *MFG-E8* and *HAPLN3*. Therefore, we speculated that lnc-bbug14207 may affect mammary gland development and lactation by regulating the expression of *MFG-E8* and *HAPLN3* genes in buffalo mammary gland.

## CONCLUSION

We integrated the known annotation and RNA-seq datasets of 22 milk somatic cell samples from three different lactation stages to identify lncRNA genes in water buffalo, and identified 7,962 lncRNA genes encoded by 10,789 lncRNA transcripts. By comparing the expression level of lncRNA genes in the early, peak and late lactating stages, we proposed that *lnc-bbug14207* may regulate adjacent *MFG-E8* and *HAPLN3* genes and be associated with mammary gland development.

## Figures and Tables

**Table 1:** General statistics of 22 RNA-seq samples in the water buffalo

Stage	Sample name	Raw reads	Mapped reads
Early	34C	29,710,315	25,164,636
	71C	27,327,120	23,528,650
	7C	27,389,814	23,062,223
	DH31	28,120,836	23,621,502
	DH5	28,940,706	24,715,362
	ML6011–2017	28,487,754	24,072,152
Peak	47C	28,536,110	24,455,446
	127C	37,887,227	31,256,962
	32C	27,974,554	23,946,218
	56C	32,792,486	26,889,838
	63C	28,277,918	23,555,505
	DH40	27,916,773	23,757,173
	DH45	52,533,049	45,441,087
	ML110040	29,282,530	24,099,522
Late	101C	30,817,380	25,208,616
	15C	51,071,851	42,287,492
	28C	35,388,567	29,124,790
	37C	51,859,921	44,236,512
	DH10	27,740,779	23,302,254
	DH16	28,183,001	23,871,001
	ML110034	51,173,955	42,934,948
	NL4398	51,564,381	43,829,723

## SUPPLEMENTARY MATERIAL

The dataset supporting the conclusions of this article is included within the article (and its Supplementary Files). The RNA-seq data used in this study were submitted to the Genome Sequence Archive (GSA) with ID CRA004591.

**Supplementary Table S1**. LncRNA transcripts identified in the water buffalo genome **Supplementary Table S2**. Significant differential expression of lncRNAs between early and peak stages **Supplementary Table S3**. Significant differential expression of lncRNAs between early and late stages **Supplementary Table S4**. Significant differential expression of lncRNAs between peak and late stages
