# Supplementary material for: Genome-wide identification of long noncoding RNA genes and their potential association with mammary gland development in water buffalo
Source: Anim Biosci. 2022 Jun 30;35(11):1656–65. doi: 10.5713/ab.22.0120 (PMC9659464; doi:10.5713/ab.22.0120)
Supplement: Supplementary file 1 [file ab-22-0120-suppl.pdf]

Table S1: LncRNA transcripts identified in the water buffalo genome

1	lncRNA	exon	3040676	3043600	.	+	.	gene_id "lnc-bbug00003"	transcript_id "lnc-bbut00000003"	exon_number "1"
1	lncRNA	exon	3043736	3044973	.	+	.	gene_id "lnc-bbug00003"	transcript_id "lnc-bbut00000003"	exon_number "2"
1	lncRNA	exon	4328958	4329149	.	+	.	gene_id "lnc-bbug00008"	transcript_id "lnc-bbut00000013"	exon_number "1"
1	lncRNA	exon	4329795	4330012	.	+	.	gene_id "lnc-bbug00008"	transcript_id "lnc-bbut00000013"	exon_number "2"
1	lncRNA	exon	4349267	4349590	.	+	.	gene_id "lnc-bbug00008"	transcript_id "lnc-bbut00000013"	exon_number "3"
1	lncRNA	exon	5610488	5610602	.	+	.	gene_id "lnc-bbug00011"	transcript_id "lnc-bbut00000027"	exon_number "1"
1	lncRNA	exon	5611571	5611648	.	+	.	gene_id "lnc-bbug00011"	transcript_id "lnc-bbut00000027"	exon_number "2"
1	lncRNA	exon	5613088	5614400	.	+	.	gene_id "lnc-bbug00011"	transcript_id "lnc-bbut00000027"	exon_number "3"
1	lncRNA	exon	5614689	5614758	.	+	.	gene_id "lnc-bbug00011"	transcript_id "lnc-bbut00000027"	exon_number "4"
1	lncRNA	exon	5621141	5621718	.	+	.	gene_id "lnc-bbug00011"	transcript_id "lnc-bbut00000027"	exon_number "5"
1	lncRNA	exon	6652741	6653036	.	+	.	gene_id "lnc-bbug00014"	transcript_id "lnc-bbut00000042"	exon_number "1"
1	lncRNA	exon	6654851	6655001	.	+	.	gene_id "lnc-bbug00014"	transcript_id "lnc-bbut00000042"	exon_number "2"
1	lncRNA	exon	6655262	6655398	.	+	.	gene_id "lnc-bbug00014"	transcript_id "lnc-bbut00000042"	exon_number "3"
1	lncRNA	exon	6653150	6653420	.	+	.	gene_id "lnc-bbug00014"	transcript_id "lnc-bbut00000043"	exon_number "1"
1	lncRNA	exon	6654851	6655001	.	+	.	gene_id "lnc-bbug00014"	transcript_id "lnc-bbut00000043"	exon_number "2"
1	lncRNA	exon	6655262	6655398	.	+	.	gene_id "lnc-bbug00014"	transcript_id "lnc-bbut00000043"	exon_number "3"
1	lncRNA	exon	7988622	7989143	.	+	.	gene_id "lnc-bbug00019"	transcript_id "lnc-bbut00000061"	exon_number "1"
1	lncRNA	exon	8234378	8235615	.	+	.	gene_id "lnc-bbug00023"	transcript_id "lnc-bbut00000067"	exon_number "1"
1	lncRNA	exon	8236748	8238149	.	+	.	gene_id "lnc-bbug00023"	transcript_id "lnc-bbut00000067"	exon_number "2"
1	lncRNA	exon	8316853	8317239	.	+	.	gene_id "lnc-bbug00025"	transcript_id "lnc-bbut00000069"	exon_number "1"
1	lncRNA	exon	8317555	8318491	.	+	.	gene_id "lnc-bbug00025"	transcript_id "lnc-bbut00000069"	exon_number "2"
1	lncRNA	exon	8346565	8347012	.	+	.	gene_id "lnc-bbug00026"	transcript_id "lnc-bbut00000070"	exon_number "1"
1	lncRNA	exon	8353509	8354172	.	+	.	gene_id "lnc-bbug00026"	transcript_id "lnc-bbut00000070"	exon_number "2"
1	lncRNA	exon	8432912	8433234	.	+	.	gene_id "lnc-bbug00028"	transcript_id "lnc-bbut00000072"	exon_number "1"
1	lncRNA	exon	8433381	8433893	.	+	.	gene_id "lnc-bbug00028"	transcript_id "lnc-bbut00000072"	exon_number "2"
1	lncRNA	exon	9307203	9307497	.	+	.	gene_id "lnc-bbug00033"	transcript_id "lnc-bbut00000090"	exon_number "1"
1	lncRNA	exon	9307603	9307951	.	+	.	gene_id "lnc-bbug00033"	transcript_id "lnc-bbut00000090"	exon_number "2"
1	lncRNA	exon	9309449	9309498	.	+	.	gene_id "lnc-bbug00033"	transcript_id "lnc-bbut00000090"	exon_number "3"
1	lncRNA	exon	10023025	10023220	.	+	.	gene_id "lnc-bbug00037"	transcript_id "lnc-bbut00000094"	exon_number "1"
1	lncRNA	exon	10026086	10026153	.	+	.	gene_id "lnc-bbug00037"	transcript_id "lnc-bbut00000094"	exon_number "2"
1	lncRNA	exon	10027866	10029257	.	+	.	gene_id "lnc-bbug00037"	transcript_id "lnc-bbut00000094"	exon_number "3"
1	lncRNA	exon	10951002	10954395	.	+	.	gene_id "lnc-bbug00041"	transcript_id "lnc-bbut00000102"	exon_number "1"
1	lncRNA	exon	11216117	11218242	.	+	.	gene_id "lnc-bbug00042"	transcript_id "lnc-bbut00000103"	exon_number "1"
1	lncRNA	exon	11218349	11218462	.	+	.	gene_id "lnc-bbug00042"	transcript_id "lnc-bbut00000103"	exon_number "2"
1	lncRNA	exon	11523674	11524006	.	+	.	gene_id "lnc-bbug00044"	transcript_id "lnc-bbut00000105"	exon_number "1"
1	lncRNA	exon	11524333	11524871	.	+	.	gene_id "lnc-bbug00044"	transcript_id "lnc-bbut00000105"	exon_number "2"
1	lncRNA	exon	12154003	12154442	.	+	.	gene_id "lnc-bbug00049"	transcript_id "lnc-bbut00000130"	exon_number "1"
1	lncRNA	exon	12157011	12157138	.	+	.	gene_id "lnc-bbug00049"	transcript_id "lnc-bbut00000130"	exon_number "2"
1	lncRNA	exon	12161058	12161150	.	+	.	gene_id "lnc-bbug00049"	transcript_id "lnc-bbut00000130"	exon_number "3"
1	lncRNA	exon	12155597	12155794	.	+	.	gene_id "lnc-bbug00049"	transcript_id "lnc-bbut00000131"	exon_number "1"
1	lncRNA	exon	12157011	12157138	.	+	.	gene_id "lnc-bbug00049"	transcript_id "lnc-bbut00000131"	exon_number "2"
1	lncRNA	exon	12161058	12161150	.	+	.	gene_id "lnc-bbug00049"	transcript_id "lnc-bbut00000131"	exon_number "3"
1	lncRNA	exon	12240957	12243569	.	+	.	gene_id "lnc-bbug00052"	transcript_id "lnc-bbut00000136"	exon_number "1"
1	lncRNA	exon	12244719	12245030	.	+	.	gene_id "lnc-bbug00052"	transcript_id "lnc-bbut00000136"	exon_number "2"
1	lncRNA	exon	12240957	12243559	.	+	.	gene_id "lnc-bbug00052"	transcript_id "lnc-bbut00000135"	exon_number "1"
1	lncRNA	exon	12244719	12245030	.	+	.	gene_id "lnc-bbug00052"	transcript_id "lnc-bbut00000135"	exon_number "2"
1	lncRNA	exon	12240957	12243475	.	+	.	gene_id "lnc-bbug00052"	transcript_id "lnc-bbut00000134"	exon_number "1"
1	lncRNA	exon	12244719	12245030	.	+	.	gene_id "lnc-bbug00052"	transcript_id "lnc-bbut00000134"	exon_number "2"
1	lncRNA	exon	12499447	12499798	.	+	.	gene_id "lnc-bbug00057"	transcript_id "lnc-bbut00000145"	exon_number "1"
1	lncRNA	exon	12500112	12500185	.	+	.	gene_id "lnc-bbug00057"	transcript_id "lnc-bbut00000145"	exon_number "2"

1	lncRNA	exon	12505326	12505618	.	+	.	gene_id "lnc-bbug00057"	transcript_id "lnc-bbut00000145"	exon_number "3"
1	lncRNA	exon	12517879	12517926	.	+	.	gene_id "lnc-bbug00058"	transcript_id "lnc-bbut00000146"	exon_number "1"
1	lncRNA	exon	12519072	12519250	.	+	.	gene_id "lnc-bbug00058"	transcript_id "lnc-bbut00000146"	exon_number "2"
1	lncRNA	exon	12519348	12519396	.	+	.	gene_id "lnc-bbug00058"	transcript_id "lnc-bbut00000146"	exon_number "3"
1	lncRNA	exon	12518423	12518512	.	+	.	gene_id "lnc-bbug00058"	transcript_id "lnc-bbut00000147"	exon_number "1"
1	lncRNA	exon	12519072	12519250	.	+	.	gene_id "lnc-bbug00058"	transcript_id "lnc-bbut00000147"	exon_number "2"
1	lncRNA	exon	12519348	12519396	.	+	.	gene_id "lnc-bbug00058"	transcript_id "lnc-bbut00000147"	exon_number "3"
1	lncRNA	exon	12548985	12549506	.	+	.	gene_id "lnc-bbug00059"	transcript_id "lnc-bbut00000148"	exon_number "1"
1	lncRNA	exon	12550694	12551447	.	+	.	gene_id "lnc-bbug00059"	transcript_id "lnc-bbut00000148"	exon_number "2"
1	lncRNA	exon	12715238	12715398	.	+	.	gene_id "lnc-bbug00060"	transcript_id "lnc-bbut00000149"	exon_number "1"
1	lncRNA	exon	12716405	12716506	.	+	.	gene_id "lnc-bbug00060"	transcript_id "lnc-bbut00000149"	exon_number "2"
1	lncRNA	exon	12722935	12723195	.	+	.	gene_id "lnc-bbug00060"	transcript_id "lnc-bbut00000149"	exon_number "3"
1	lncRNA	exon	14757589	14758518	.	+	.	gene_id "lnc-bbug00062"	transcript_id "lnc-bbut00000151"	exon_number "1"
1	lncRNA	exon	14759407	14760784	.	+	.	gene_id "lnc-bbug00062"	transcript_id "lnc-bbut00000151"	exon_number "2"
1	lncRNA	exon	16545375	16547645	.	+	.	gene_id "lnc-bbug00069"	transcript_id "lnc-bbut00000163"	exon_number "1"
1	lncRNA	exon	18020992	18021193	.	+	.	gene_id "lnc-bbug00070"	transcript_id "lnc-bbut00000164"	exon_number "1"
1	lncRNA	exon	18021478	18021933	.	+	.	gene_id "lnc-bbug00070"	transcript_id "lnc-bbut00000164"	exon_number "2"
1	lncRNA	exon	18635985	18636044	.	+	.	gene_id "lnc-bbug00074"	transcript_id "lnc-bbut00000177"	exon_number "1"
1	lncRNA	exon	18637712	18643273	.	+	.	gene_id "lnc-bbug00074"	transcript_id "lnc-bbut00000177"	exon_number "2"
1	lncRNA	exon	18645785	18645845	.	+	.	gene_id "lnc-bbug00074"	transcript_id "lnc-bbut00000177"	exon_number "3"
1	lncRNA	exon	18647150	18647318	.	+	.	gene_id "lnc-bbug00074"	transcript_id "lnc-bbut00000177"	exon_number "4"
1	lncRNA	exon	19130695	19131874	.	+	.	gene_id "lnc-bbug00078"	transcript_id "lnc-bbut00000181"	exon_number "1"
1	lncRNA	exon	19138165	19138878	.	+	.	gene_id "lnc-bbug00078"	transcript_id "lnc-bbut00000181"	exon_number "2"
1	lncRNA	exon	19225180	19225508	.	+	.	gene_id "lnc-bbug00079"	transcript_id "lnc-bbut00000182"	exon_number "1"
1	lncRNA	exon	19225636	19225817	.	+	.	gene_id "lnc-bbug00079"	transcript_id "lnc-bbut00000182"	exon_number "2"
1	lncRNA	exon	19226660	19226996	.	+	.	gene_id "lnc-bbug00079"	transcript_id "lnc-bbut00000182"	exon_number "3"
1	lncRNA	exon	19326560	19326668	.	+	.	gene_id "lnc-bbug00082"	transcript_id "lnc-bbut00000185"	exon_number "1"
1	lncRNA	exon	19361476	19361539	.	+	.	gene_id "lnc-bbug00082"	transcript_id "lnc-bbut00000185"	exon_number "2"
1	lncRNA	exon	19414783	19414869	.	+	.	gene_id "lnc-bbug00082"	transcript_id "lnc-bbut00000185"	exon_number "3"
1	lncRNA	exon	19424147	19424277	.	+	.	gene_id "lnc-bbug00082"	transcript_id "lnc-bbut00000185"	exon_number "4"
1	lncRNA	exon	19430626	19430729	.	+	.	gene_id "lnc-bbug00082"	transcript_id "lnc-bbut00000185"	exon_number "5"
1	lncRNA	exon	19438402	19439671	.	+	.	gene_id "lnc-bbug00082"	transcript_id "lnc-bbut00000185"	exon_number "6"
1	lncRNA	exon	19326560	19326668	.	+	.	gene_id "lnc-bbug00082"	transcript_id "lnc-bbut00000186"	exon_number "1"
1	lncRNA	exon	19361476	19361539	.	+	.	gene_id "lnc-bbug00082"	transcript_id "lnc-bbut00000186"	exon_number "2"
1	lncRNA	exon	19414783	19414869	.	+	.	gene_id "lnc-bbug00082"	transcript_id "lnc-bbut00000186"	exon_number "3"
1	lncRNA	exon	19424147	19424277	.	+	.	gene_id "lnc-bbug00082"	transcript_id "lnc-bbut00000186"	exon_number "4"
1	lncRNA	exon	19430626	19430729	.	+	.	gene_id "lnc-bbug00082"	transcript_id "lnc-bbut00000186"	exon_number "5"
1	lncRNA	exon	19486605	19488460	.	+	.	gene_id "lnc-bbug00082"	transcript_id "lnc-bbut00000186"	exon_number "6"
1	lncRNA	exon	19326560	19326668	.	+	.	gene_id "lnc-bbug00082"	transcript_id "lnc-bbut00000187"	exon_number "1"
1	lncRNA	exon	19361476	19361539	.	+	.	gene_id "lnc-bbug00082"	transcript_id "lnc-bbut00000187"	exon_number "2"
1	lncRNA	exon	19414783	19414869	.	+	.	gene_id "lnc-bbug00082"	transcript_id "lnc-bbut00000187"	exon_number "3"
1	lncRNA	exon	19424147	19424277	.	+	.	gene_id "lnc-bbug00082"	transcript_id "lnc-bbut00000187"	exon_number "4"
1	lncRNA	exon	19430626	19430729	.	+	.	gene_id "lnc-bbug00082"	transcript_id "lnc-bbut00000187"	exon_number "5"
1	lncRNA	exon	19568714	19569193	.	+	.	gene_id "lnc-bbug00082"	transcript_id "lnc-bbut00000187"	exon_number "6"
1	lncRNA	exon	19344571	19345166	.	+	.	gene_id "lnc-bbug00082"	transcript_id "lnc-bbut00000188"	exon_number "1"
1	lncRNA	exon	19361476	19361539	.	+	.	gene_id "lnc-bbug00082"	transcript_id "lnc-bbut00000188"	exon_number "2"
1	lncRNA	exon	19414783	19414869	.	+	.	gene_id "lnc-bbug00082"	transcript_id "lnc-bbut00000188"	exon_number "3"
1	lncRNA	exon	19424147	19424277	.	+	.	gene_id "lnc-bbug00082"	transcript_id "lnc-bbut00000188"	exon_number "4"
1	lncRNA	exon	19430626	19430729	.	+	.	gene_id "lnc-bbug00082"	transcript_id "lnc-bbut00000188"	exon_number "5"
1	lncRNA	exon	19486605	19488460	.	+	.	gene_id "lnc-bbug00082"	transcript_id "lnc-bbut00000188"	exon_number "6"
1	lncRNA	exon	19442274	19443318	.	+	.	gene_id "lnc-bbug00083"	transcript_id "lnc-bbut00000189"	exon_number "1"
1	lncRNA	exon	19444658	19444886	.	+	.	gene_id "lnc-bbug00083"	transcript_id "lnc-bbut00000189"	exon_number "2"

1	lncRNA	exon	19444978	19445038	.	+	.	gene_id "lnc-bbug00083"	transcript_id "lnc-bbut00000189"	exon_number "3"
1	lncRNA	exon	19445335	19445829	.	+	.	gene_id "lnc-bbug00083"	transcript_id "lnc-bbut00000189"	exon_number "4"
1	lncRNA	exon	19948140	19948480	.	+	.	gene_id "lnc-bbug00085"	transcript_id "lnc-bbut00000192"	exon_number "1"
1	lncRNA	exon	20494071	20494434	.	+	.	gene_id "lnc-bbug00087"	transcript_id "lnc-bbut00000197"	exon_number "1"
1	lncRNA	exon	20495079	20495185	.	+	.	gene_id "lnc-bbug00087"	transcript_id "lnc-bbut00000197"	exon_number "2"
1	lncRNA	exon	20495504	20495623	.	+	.	gene_id "lnc-bbug00087"	transcript_id "lnc-bbut00000197"	exon_number "3"
1	lncRNA	exon	20495774	20495998	.	+	.	gene_id "lnc-bbug00087"	transcript_id "lnc-bbut00000197"	exon_number "4"
1	lncRNA	exon	20496307	20496410	.	+	.	gene_id "lnc-bbug00087"	transcript_id "lnc-bbut00000197"	exon_number "5"
1	lncRNA	exon	20502370	20502556	.	+	.	gene_id "lnc-bbug00087"	transcript_id "lnc-bbut00000197"	exon_number "6"
1	lncRNA	exon	20494071	20494199	.	+	.	gene_id "lnc-bbug00087"	transcript_id "lnc-bbut00000196"	exon_number "1"
1	lncRNA	exon	20495079	20495185	.	+	.	gene_id "lnc-bbug00087"	transcript_id "lnc-bbut00000196"	exon_number "2"
1	lncRNA	exon	20495504	20495623	.	+	.	gene_id "lnc-bbug00087"	transcript_id "lnc-bbut00000196"	exon_number "3"
1	lncRNA	exon	20495774	20495998	.	+	.	gene_id "lnc-bbug00087"	transcript_id "lnc-bbut00000196"	exon_number "4"
1	lncRNA	exon	20496307	20496410	.	+	.	gene_id "lnc-bbug00087"	transcript_id "lnc-bbut00000196"	exon_number "5"
1	lncRNA	exon	20502370	20502556	.	+	.	gene_id "lnc-bbug00087"	transcript_id "lnc-bbut00000196"	exon_number "6"
1	lncRNA	exon	20494071	20495185	.	+	.	gene_id "lnc-bbug00087"	transcript_id "lnc-bbut00000195"	exon_number "1"
1	lncRNA	exon	20495504	20495623	.	+	.	gene_id "lnc-bbug00087"	transcript_id "lnc-bbut00000195"	exon_number "2"
1	lncRNA	exon	20495774	20495998	.	+	.	gene_id "lnc-bbug00087"	transcript_id "lnc-bbut00000195"	exon_number "3"
1	lncRNA	exon	20496307	20496410	.	+	.	gene_id "lnc-bbug00087"	transcript_id "lnc-bbut00000195"	exon_number "4"
1	lncRNA	exon	20502370	20502556	.	+	.	gene_id "lnc-bbug00087"	transcript_id "lnc-bbut00000195"	exon_number "5"
1	lncRNA	exon	20494081	20494199	.	+	.	gene_id "lnc-bbug00087"	transcript_id "lnc-bbut00000198"	exon_number "1"
1	lncRNA	exon	20495504	20495623	.	+	.	gene_id "lnc-bbug00087"	transcript_id "lnc-bbut00000198"	exon_number "2"
1	lncRNA	exon	20495774	20495998	.	+	.	gene_id "lnc-bbug00087"	transcript_id "lnc-bbut00000198"	exon_number "3"
1	lncRNA	exon	20496307	20496410	.	+	.	gene_id "lnc-bbug00087"	transcript_id "lnc-bbut00000198"	exon_number "4"
1	lncRNA	exon	20502370	20502556	.	+	.	gene_id "lnc-bbug00087"	transcript_id "lnc-bbut00000198"	exon_number "5"
1	lncRNA	exon	21522255	21523484	.	+	.	gene_id "lnc-bbug00093"	transcript_id "lnc-bbut00000216"	exon_number "1"
1	lncRNA	exon	21524147	21526240	.	+	.	gene_id "lnc-bbug00093"	transcript_id "lnc-bbut00000216"	exon_number "2"
1	lncRNA	exon	21522255	21523484	.	+	.	gene_id "lnc-bbug00093"	transcript_id "lnc-bbut00000217"	exon_number "1"
1	lncRNA	exon	21524147	21524297	.	+	.	gene_id "lnc-bbug00093"	transcript_id "lnc-bbut00000217"	exon_number "2"
1	lncRNA	exon	21534229	21534636	.	+	.	gene_id "lnc-bbug00093"	transcript_id "lnc-bbut00000217"	exon_number "3"
1	lncRNA	exon	21522255	21523484	.	+	.	gene_id "lnc-bbug00093"	transcript_id "lnc-bbut00000218"	exon_number "1"
1	lncRNA	exon	21524147	21524297	.	+	.	gene_id "lnc-bbug00093"	transcript_id "lnc-bbut00000218"	exon_number "2"
1	lncRNA	exon	21546279	21547021	.	+	.	gene_id "lnc-bbug00093"	transcript_id "lnc-bbut00000218"	exon_number "3"
1	lncRNA	exon	22256378	22256471	.	+	.	gene_id "lnc-bbug00096"	transcript_id "lnc-bbut00000221"	exon_number "1"
1	lncRNA	exon	22396812	22396908	.	+	.	gene_id "lnc-bbug00096"	transcript_id "lnc-bbut00000221"	exon_number "2"
1	lncRNA	exon	22440563	22440638	.	+	.	gene_id "lnc-bbug00096"	transcript_id "lnc-bbut00000221"	exon_number "3"
1	lncRNA	exon	22441726	22441746	.	+	.	gene_id "lnc-bbug00096"	transcript_id "lnc-bbut00000221"	exon_number "4"
1	lncRNA	exon	22441932	22443048	.	+	.	gene_id "lnc-bbug00096"	transcript_id "lnc-bbut00000221"	exon_number "5"
1	lncRNA	exon	25370667	25373441	.	+	.	gene_id "lnc-bbug00102"	transcript_id "lnc-bbut00000231"	exon_number "1"
1	lncRNA	exon	26376340	26376956	.	+	.	gene_id "lnc-bbug00110"	transcript_id "lnc-bbut00000267"	exon_number "1"
1	lncRNA	exon	26390436	26390889	.	+	.	gene_id "lnc-bbug00110"	transcript_id "lnc-bbut00000267"	exon_number "2"
1	lncRNA	exon	27147808	27147860	.	+	.	gene_id "lnc-bbug00112"	transcript_id "lnc-bbut00000269"	exon_number "1"
1	lncRNA	exon	27168214	27168299	.	+	.	gene_id "lnc-bbug00112"	transcript_id "lnc-bbut00000269"	exon_number "2"
1	lncRNA	exon	27196940	27199346	.	+	.	gene_id "lnc-bbug00112"	transcript_id "lnc-bbut00000269"	exon_number "3"
1	lncRNA	exon	29019471	29019761	.	+	.	gene_id "lnc-bbug00115"	transcript_id "lnc-bbut00000273"	exon_number "1"
1	lncRNA	exon	29333280	29333570	.	+	.	gene_id "lnc-bbug00118"	transcript_id "lnc-bbut00000284"	exon_number "1"
1	lncRNA	exon	29334896	29335086	.	+	.	gene_id "lnc-bbug00118"	transcript_id "lnc-bbut00000284"	exon_number "2"
1	lncRNA	exon	29335517	29335651	.	+	.	gene_id "lnc-bbug00118"	transcript_id "lnc-bbut00000284"	exon_number "3"
1	lncRNA	exon	29338242	29338352	.	+	.	gene_id "lnc-bbug00118"	transcript_id "lnc-bbut00000284"	exon_number "4"
1	lncRNA	exon	29339752	29340167	.	+	.	gene_id "lnc-bbug00118"	transcript_id "lnc-bbut00000284"	exon_number "5"
1	lncRNA	exon	29333280	29333570	.	+	.	gene_id "lnc-bbug00118"	transcript_id "lnc-bbut00000285"	exon_number "1"
1	lncRNA	exon	29334966	29335086	.	+	.	gene_id "lnc-bbug00118"	transcript_id "lnc-bbut00000285"	exon_number "2"

1	lncRNA	exon	29335517	29335651	.	+	.	gene_id "lnc-bbug00118"	transcript_id "lnc-bbut00000285"	exon_number "3"
1	lncRNA	exon	29338242	29338352	.	+	.	gene_id "lnc-bbug00118"	transcript_id "lnc-bbut00000285"	exon_number "4"
1	lncRNA	exon	29339752	29340167	.	+	.	gene_id "lnc-bbug00118"	transcript_id "lnc-bbut00000285"	exon_number "5"
1	lncRNA	exon	29333280	29333570	.	+	.	gene_id "lnc-bbug00118"	transcript_id "lnc-bbut00000283"	exon_number "1"
1	lncRNA	exon	29334966	29335651	.	+	.	gene_id "lnc-bbug00118"	transcript_id "lnc-bbut00000283"	exon_number "2"
1	lncRNA	exon	29338242	29338352	.	+	.	gene_id "lnc-bbug00118"	transcript_id "lnc-bbut00000283"	exon_number "3"
1	lncRNA	exon	29339752	29340167	.	+	.	gene_id "lnc-bbug00118"	transcript_id "lnc-bbut00000283"	exon_number "4"
1	lncRNA	exon	29333280	29333570	.	+	.	gene_id "lnc-bbug00118"	transcript_id "lnc-bbut00000282"	exon_number "1"
1	lncRNA	exon	29334896	29335651	.	+	.	gene_id "lnc-bbug00118"	transcript_id "lnc-bbut00000282"	exon_number "2"
1	lncRNA	exon	29338242	29338352	.	+	.	gene_id "lnc-bbug00118"	transcript_id "lnc-bbut00000282"	exon_number "3"
1	lncRNA	exon	29339752	29340167	.	+	.	gene_id "lnc-bbug00118"	transcript_id "lnc-bbut00000282"	exon_number "4"
1	lncRNA	exon	30312431	30312734	.	+	.	gene_id "lnc-bbug00126"	transcript_id "lnc-bbut00000337"	exon_number "1"
1	lncRNA	exon	30313295	30313727	.	+	.	gene_id "lnc-bbug00126"	transcript_id "lnc-bbut00000337"	exon_number "2"
1	lncRNA	exon	30313766	30313926	.	+	.	gene_id "lnc-bbug00126"	transcript_id "lnc-bbut00000337"	exon_number "3"
1	lncRNA	exon	30487445	30488279	.	+	.	gene_id "lnc-bbug00129"	transcript_id "lnc-bbut00000344"	exon_number "1"
1	lncRNA	exon	31204586	31204844	.	+	.	gene_id "lnc-bbug00133"	transcript_id "lnc-bbut00000352"	exon_number "1"
1	lncRNA	exon	31206200	31206280	.	+	.	gene_id "lnc-bbug00133"	transcript_id "lnc-bbut00000352"	exon_number "2"
1	lncRNA	exon	31208306	31208411	.	+	.	gene_id "lnc-bbug00133"	transcript_id "lnc-bbut00000352"	exon_number "3"
1	lncRNA	exon	31208496	31208643	.	+	.	gene_id "lnc-bbug00133"	transcript_id "lnc-bbut00000352"	exon_number "4"
1	lncRNA	exon	31209796	31209913	.	+	.	gene_id "lnc-bbug00133"	transcript_id "lnc-bbut00000352"	exon_number "5"
1	lncRNA	exon	31210782	31210831	.	+	.	gene_id "lnc-bbug00133"	transcript_id "lnc-bbut00000352"	exon_number "6"
1	lncRNA	exon	31210968	31210970	.	+	.	gene_id "lnc-bbug00133"	transcript_id "lnc-bbut00000352"	exon_number "7"
1	lncRNA	exon	31212515	31214332	.	+	.	gene_id "lnc-bbug00133"	transcript_id "lnc-bbut00000352"	exon_number "8"
1	lncRNA	exon	31316834	31317337	.	+	.	gene_id "lnc-bbug00135"	transcript_id "lnc-bbut00000354"	exon_number "1"
1	lncRNA	exon	31317550	31318353	.	+	.	gene_id "lnc-bbug00135"	transcript_id "lnc-bbut00000354"	exon_number "2"
1	lncRNA	exon	31320191	31323786	.	+	.	gene_id "lnc-bbug00136"	transcript_id "lnc-bbut00000355"	exon_number "1"
1	lncRNA	exon	31402536	31402756	.	+	.	gene_id "lnc-bbug00138"	transcript_id "lnc-bbut00000358"	exon_number "1"
1	lncRNA	exon	31405941	31406255	.	+	.	gene_id "lnc-bbug00138"	transcript_id "lnc-bbut00000358"	exon_number "2"
1	lncRNA	exon	33946910	33948900	.	+	.	gene_id "lnc-bbug00141"	transcript_id "lnc-bbut00000363"	exon_number "1"
1	lncRNA	exon	37034324	37034690	.	+	.	gene_id "lnc-bbug00144"	transcript_id "lnc-bbut00000366"	exon_number "1"
1	lncRNA	exon	37038980	37039433	.	+	.	gene_id "lnc-bbug00144"	transcript_id "lnc-bbut00000366"	exon_number "2"
1	lncRNA	exon	37034877	37035054	.	+	.	gene_id "lnc-bbug00144"	transcript_id "lnc-bbut00000367"	exon_number "1"
1	lncRNA	exon	37038980	37039433	.	+	.	gene_id "lnc-bbug00144"	transcript_id "lnc-bbut00000367"	exon_number "2"
1	lncRNA	exon	38373265	38376140	.	+	.	gene_id "lnc-bbug00151"	transcript_id "lnc-bbut00000382"	exon_number "1"
1	lncRNA	exon	38376817	38381908	.	+	.	gene_id "lnc-bbug00151"	transcript_id "lnc-bbut00000382"	exon_number "2"
1	lncRNA	exon	38382070	38382144	.	+	.	gene_id "lnc-bbug00151"	transcript_id "lnc-bbut00000382"	exon_number "3"
1	lncRNA	exon	38383067	38383232	.	+	.	gene_id "lnc-bbug00151"	transcript_id "lnc-bbut00000382"	exon_number "4"
1	lncRNA	exon	38386509	38386594	.	+	.	gene_id "lnc-bbug00151"	transcript_id "lnc-bbut00000382"	exon_number "5"
1	lncRNA	exon	38389439	38389543	.	+	.	gene_id "lnc-bbug00151"	transcript_id "lnc-bbut00000382"	exon_number "6"
1	lncRNA	exon	38373265	38375187	.	+	.	gene_id "lnc-bbug00151"	transcript_id "lnc-bbut00000385"	exon_number "1"
1	lncRNA	exon	38375329	38376140	.	+	.	gene_id "lnc-bbug00151"	transcript_id "lnc-bbut00000385"	exon_number "2"
1	lncRNA	exon	38376817	38376939	.	+	.	gene_id "lnc-bbug00151"	transcript_id "lnc-bbut00000385"	exon_number "3"
1	lncRNA	exon	38377030	38381908	.	+	.	gene_id "lnc-bbug00151"	transcript_id "lnc-bbut00000385"	exon_number "4"
1	lncRNA	exon	38382070	38382144	.	+	.	gene_id "lnc-bbug00151"	transcript_id "lnc-bbut00000385"	exon_number "5"
1	lncRNA	exon	38383067	38383232	.	+	.	gene_id "lnc-bbug00151"	transcript_id "lnc-bbut00000385"	exon_number "6"
1	lncRNA	exon	38386509	38386594	.	+	.	gene_id "lnc-bbug00151"	transcript_id "lnc-bbut00000385"	exon_number "7"
1	lncRNA	exon	38394436	38396722	.	+	.	gene_id "lnc-bbug00151"	transcript_id "lnc-bbut00000385"	exon_number "8"
1	lncRNA	exon	38373265	38376140	.	+	.	gene_id "lnc-bbug00151"	transcript_id "lnc-bbut00000384"	exon_number "1"
1	lncRNA	exon	38376817	38379879	.	+	.	gene_id "lnc-bbug00151"	transcript_id "lnc-bbut00000384"	exon_number "2"
1	lncRNA	exon	38381647	38381908	.	+	.	gene_id "lnc-bbug00151"	transcript_id "lnc-bbut00000384"	exon_number "3"
1	lncRNA	exon	38382070	38382144	.	+	.	gene_id "lnc-bbug00151"	transcript_id "lnc-bbut00000384"	exon_number "4"
1	lncRNA	exon	38383067	38383232	.	+	.	gene_id "lnc-bbug00151"	transcript_id "lnc-bbut00000384"	exon_number "5"

1	lncRNA	exon	38386509	38386594	.	+	.	gene_id "lnc-bbug00151"	transcript_id "lnc-bbut00000384"	exon_number "6"
1	lncRNA	exon	38394436	38396722	.	+	.	gene_id "lnc-bbug00151"	transcript_id "lnc-bbut00000384"	exon_number "7"
1	lncRNA	exon	38373265	38375475	.	+	.	gene_id "lnc-bbug00151"	transcript_id "lnc-bbut00000383"	exon_number "1"
1	lncRNA	exon	38376030	38376140	.	+	.	gene_id "lnc-bbug00151"	transcript_id "lnc-bbut00000383"	exon_number "2"
1	lncRNA	exon	38376817	38381908	.	+	.	gene_id "lnc-bbug00151"	transcript_id "lnc-bbut00000383"	exon_number "3"
1	lncRNA	exon	38382070	38382144	.	+	.	gene_id "lnc-bbug00151"	transcript_id "lnc-bbut00000383"	exon_number "4"
1	lncRNA	exon	38383067	38383232	.	+	.	gene_id "lnc-bbug00151"	transcript_id "lnc-bbut00000383"	exon_number "5"
1	lncRNA	exon	38386509	38386594	.	+	.	gene_id "lnc-bbug00151"	transcript_id "lnc-bbut00000383"	exon_number "6"
1	lncRNA	exon	38394436	38396722	.	+	.	gene_id "lnc-bbug00151"	transcript_id "lnc-bbut00000383"	exon_number "7"
1	lncRNA	exon	38418436	38419434	.	+	.	gene_id "lnc-bbug00152"	transcript_id "lnc-bbut00000386"	exon_number "1"
1	lncRNA	exon	38420124	38420242	.	+	.	gene_id "lnc-bbug00152"	transcript_id "lnc-bbut00000386"	exon_number "2"
1	lncRNA	exon	38420337	38420496	.	+	.	gene_id "lnc-bbug00152"	transcript_id "lnc-bbut00000386"	exon_number "3"
1	lncRNA	exon	38420696	38420797	.	+	.	gene_id "lnc-bbug00152"	transcript_id "lnc-bbut00000386"	exon_number "4"
1	lncRNA	exon	38425619	38425693	.	+	.	gene_id "lnc-bbug00152"	transcript_id "lnc-bbut00000386"	exon_number "5"
1	lncRNA	exon	38426624	38426845	.	+	.	gene_id "lnc-bbug00152"	transcript_id "lnc-bbut00000386"	exon_number "6"
1	lncRNA	exon	38424757	38425457	.	+	.	gene_id "lnc-bbug00152"	transcript_id "lnc-bbut00000387"	exon_number "1"
1	lncRNA	exon	38425619	38425693	.	+	.	gene_id "lnc-bbug00152"	transcript_id "lnc-bbut00000387"	exon_number "2"
1	lncRNA	exon	38426624	38426845	.	+	.	gene_id "lnc-bbug00152"	transcript_id "lnc-bbut00000387"	exon_number "3"
1	lncRNA	exon	38768903	38769654	.	+	.	gene_id "lnc-bbug00155"	transcript_id "lnc-bbut00000390"	exon_number "1"
1	lncRNA	exon	38772424	38773000	.	+	.	gene_id "lnc-bbug00155"	transcript_id "lnc-bbut00000390"	exon_number "2"
1	lncRNA	exon	38790943	38791777	.	+	.	gene_id "lnc-bbug00156"	transcript_id "lnc-bbut00000391"	exon_number "1"
1	lncRNA	exon	38793895	38794086	.	+	.	gene_id "lnc-bbug00156"	transcript_id "lnc-bbut00000391"	exon_number "2"
1	lncRNA	exon	38796670	38797374	.	+	.	gene_id "lnc-bbug00156"	transcript_id "lnc-bbut00000391"	exon_number "3"
1	lncRNA	exon	38816953	38817066	.	+	.	gene_id "lnc-bbug00157"	transcript_id "lnc-bbut00000392"	exon_number "1"
1	lncRNA	exon	38817328	38817491	.	+	.	gene_id "lnc-bbug00157"	transcript_id "lnc-bbut00000392"	exon_number "2"
1	lncRNA	exon	38818138	38818369	.	+	.	gene_id "lnc-bbug00157"	transcript_id "lnc-bbut00000392"	exon_number "3"
1	lncRNA	exon	38817199	38817222	.	+	.	gene_id "lnc-bbug00157"	transcript_id "lnc-bbut00000393"	exon_number "1"
1	lncRNA	exon	38817328	38817491	.	+	.	gene_id "lnc-bbug00157"	transcript_id "lnc-bbut00000393"	exon_number "2"
1	lncRNA	exon	38818138	38818369	.	+	.	gene_id "lnc-bbug00157"	transcript_id "lnc-bbut00000393"	exon_number "3"
1	lncRNA	exon	38836528	38837260	.	+	.	gene_id "lnc-bbug00158"	transcript_id "lnc-bbut00000394"	exon_number "1"
1	lncRNA	exon	38840552	38840755	.	+	.	gene_id "lnc-bbug00158"	transcript_id "lnc-bbut00000394"	exon_number "2"
1	lncRNA	exon	44889349	44890410	.	+	.	gene_id "lnc-bbug00166"	transcript_id "lnc-bbut00000409"	exon_number "1"
1	lncRNA	exon	44912681	44912827	.	+	.	gene_id "lnc-bbug00166"	transcript_id "lnc-bbut00000409"	exon_number "2"
1	lncRNA	exon	44917581	44917733	.	+	.	gene_id "lnc-bbug00166"	transcript_id "lnc-bbut00000409"	exon_number "3"
1	lncRNA	exon	44920250	44920824	.	+	.	gene_id "lnc-bbug00166"	transcript_id "lnc-bbut00000409"	exon_number "4"
1	lncRNA	exon	44896129	44897061	.	+	.	gene_id "lnc-bbug00166"	transcript_id "lnc-bbut00000410"	exon_number "1"
1	lncRNA	exon	44912681	44912827	.	+	.	gene_id "lnc-bbug00166"	transcript_id "lnc-bbut00000410"	exon_number "2"
1	lncRNA	exon	44917581	44917733	.	+	.	gene_id "lnc-bbug00166"	transcript_id "lnc-bbut00000410"	exon_number "3"
1	lncRNA	exon	44920250	44920700	.	+	.	gene_id "lnc-bbug00166"	transcript_id "lnc-bbut00000410"	exon_number "4"
1	lncRNA	exon	44939868	44940551	.	+	.	gene_id "lnc-bbug00166"	transcript_id "lnc-bbut00000410"	exon_number "5"
1	lncRNA	exon	44973051	44973187	.	+	.	gene_id "lnc-bbug00167"	transcript_id "lnc-bbut00000412"	exon_number "1"
1	lncRNA	exon	44978941	44979064	.	+	.	gene_id "lnc-bbug00167"	transcript_id "lnc-bbut00000412"	exon_number "2"
1	lncRNA	exon	44994012	44994553	.	+	.	gene_id "lnc-bbug00167"	transcript_id "lnc-bbut00000412"	exon_number "3"
1	lncRNA	exon	45000144	45000280	.	+	.	gene_id "lnc-bbug00167"	transcript_id "lnc-bbut00000412"	exon_number "4"
1	lncRNA	exon	44973051	44973187	.	+	.	gene_id "lnc-bbug00167"	transcript_id "lnc-bbut00000414"	exon_number "1"
1	lncRNA	exon	44978941	44979064	.	+	.	gene_id "lnc-bbug00167"	transcript_id "lnc-bbut00000414"	exon_number "2"
1	lncRNA	exon	44994012	44994553	.	+	.	gene_id "lnc-bbug00167"	transcript_id "lnc-bbut00000414"	exon_number "3"
1	lncRNA	exon	45033087	45033233	.	+	.	gene_id "lnc-bbug00167"	transcript_id "lnc-bbut00000414"	exon_number "4"
1	lncRNA	exon	45052926	45053029	.	+	.	gene_id "lnc-bbug00167"	transcript_id "lnc-bbut00000414"	exon_number "5"
1	lncRNA	exon	45057778	45063567	.	+	.	gene_id "lnc-bbug00167"	transcript_id "lnc-bbut00000414"	exon_number "6"
1	lncRNA	exon	45068955	45069072	.	+	.	gene_id "lnc-bbug00167"	transcript_id "lnc-bbut00000414"	exon_number "7"
1	lncRNA	exon	45103409	45103479	.	+	.	gene_id "lnc-bbug00167"	transcript_id "lnc-bbut00000414"	exon_number "8"

1	lncRNA	exon	45104244	45104337	.	+	.	gene_id "lnc-bbug00167"	transcript_id "lnc-bbut00000414"	exon_number "9"
1	lncRNA	exon	45105307	45105419	.	+	.	gene_id "lnc-bbug00167"	transcript_id "lnc-bbut00000414"	exon_number "10"
1	lncRNA	exon	45111762	45112052	.	+	.	gene_id "lnc-bbug00167"	transcript_id "lnc-bbut00000414"	exon_number "11"
1	lncRNA	exon	45117905	45117950	.	+	.	gene_id "lnc-bbug00167"	transcript_id "lnc-bbut00000414"	exon_number "12"
1	lncRNA	exon	45119116	45119584	.	+	.	gene_id "lnc-bbug00167"	transcript_id "lnc-bbut00000414"	exon_number "13"
1	lncRNA	exon	45119822	45121142	.	+	.	gene_id "lnc-bbug00169"	transcript_id "lnc-bbut00000416"	exon_number "1"
1	lncRNA	exon	45123818	45123931	.	+	.	gene_id "lnc-bbug00169"	transcript_id "lnc-bbut00000416"	exon_number "2"
1	lncRNA	exon	45124094	45124267	.	+	.	gene_id "lnc-bbug00169"	transcript_id "lnc-bbut00000416"	exon_number "3"
1	lncRNA	exon	45126276	45126349	.	+	.	gene_id "lnc-bbug00169"	transcript_id "lnc-bbut00000416"	exon_number "4"
1	lncRNA	exon	45128578	45128684	.	+	.	gene_id "lnc-bbug00169"	transcript_id "lnc-bbut00000416"	exon_number "5"
1	lncRNA	exon	45130172	45130309	.	+	.	gene_id "lnc-bbug00169"	transcript_id "lnc-bbut00000416"	exon_number "6"
1	lncRNA	exon	45136608	45136641	.	+	.	gene_id "lnc-bbug00169"	transcript_id "lnc-bbut00000416"	exon_number "7"
1	lncRNA	exon	45153464	45155893	.	+	.	gene_id "lnc-bbug00169"	transcript_id "lnc-bbut00000416"	exon_number "8"
1	lncRNA	exon	45185846	45185858	.	+	.	gene_id "lnc-bbug00171"	transcript_id "lnc-bbut00000418"	exon_number "1"
1	lncRNA	exon	45186271	45188425	.	+	.	gene_id "lnc-bbug00171"	transcript_id "lnc-bbut00000418"	exon_number "2"
1	lncRNA	exon	45193524	45193551	.	+	.	gene_id "lnc-bbug00172"	transcript_id "lnc-bbut00000419"	exon_number "1"
1	lncRNA	exon	45196606	45199541	.	+	.	gene_id "lnc-bbug00172"	transcript_id "lnc-bbut00000419"	exon_number "2"
1	lncRNA	exon	45209870	45212043	.	+	.	gene_id "lnc-bbug00173"	transcript_id "lnc-bbut00000420"	exon_number "1"
1	lncRNA	exon	45220886	45222731	.	+	.	gene_id "lnc-bbug00174"	transcript_id "lnc-bbut00000421"	exon_number "1"
1	lncRNA	exon	46753869	46755609	.	+	.	gene_id "lnc-bbug00188"	transcript_id "lnc-bbut00000449"	exon_number "1"
1	lncRNA	exon	46755900	46756064	.	+	.	gene_id "lnc-bbug00188"	transcript_id "lnc-bbut00000449"	exon_number "2"
1	lncRNA	exon	46758261	46758309	.	+	.	gene_id "lnc-bbug00188"	transcript_id "lnc-bbut00000449"	exon_number "3"
1	lncRNA	exon	46753885	46753946	.	+	.	gene_id "lnc-bbug00188"	transcript_id "lnc-bbut00000450"	exon_number "1"
1	lncRNA	exon	46754244	46756064	.	+	.	gene_id "lnc-bbug00188"	transcript_id "lnc-bbut00000450"	exon_number "2"
1	lncRNA	exon	46758261	46758309	.	+	.	gene_id "lnc-bbug00188"	transcript_id "lnc-bbut00000450"	exon_number "3"
1	lncRNA	exon	46844632	46844892	.	+	.	gene_id "lnc-bbug00190"	transcript_id "lnc-bbut00000452"	exon_number "1"
1	lncRNA	exon	46868900	46869971	.	+	.	gene_id "lnc-bbug00190"	transcript_id "lnc-bbut00000452"	exon_number "2"
1	lncRNA	exon	46870639	46870831	.	+	.	gene_id "lnc-bbug00190"	transcript_id "lnc-bbut00000452"	exon_number "3"
1	lncRNA	exon	46874227	46874349	.	+	.	gene_id "lnc-bbug00190"	transcript_id "lnc-bbut00000452"	exon_number "4"
1	lncRNA	exon	46881874	46881933	.	+	.	gene_id "lnc-bbug00190"	transcript_id "lnc-bbut00000452"	exon_number "5"
1	lncRNA	exon	46882524	46882700	.	+	.	gene_id "lnc-bbug00190"	transcript_id "lnc-bbut00000452"	exon_number "6"
1	lncRNA	exon	47246476	47246522	.	+	.	gene_id "lnc-bbug00191"	transcript_id "lnc-bbut00000453"	exon_number "1"
1	lncRNA	exon	47249626	47249768	.	+	.	gene_id "lnc-bbug00191"	transcript_id "lnc-bbut00000453"	exon_number "2"
1	lncRNA	exon	47250904	47251687	.	+	.	gene_id "lnc-bbug00191"	transcript_id "lnc-bbut00000453"	exon_number "3"
1	lncRNA	exon	47246497	47246602	.	+	.	gene_id "lnc-bbug00191"	transcript_id "lnc-bbut00000454"	exon_number "1"
1	lncRNA	exon	47249626	47249768	.	+	.	gene_id "lnc-bbug00191"	transcript_id "lnc-bbut00000454"	exon_number "2"
1	lncRNA	exon	47250904	47251687	.	+	.	gene_id "lnc-bbug00191"	transcript_id "lnc-bbut00000454"	exon_number "3"
1	lncRNA	exon	47246626	47246986	.	+	.	gene_id "lnc-bbug00191"	transcript_id "lnc-bbut00000455"	exon_number "1"
1	lncRNA	exon	47249626	47249768	.	+	.	gene_id "lnc-bbug00191"	transcript_id "lnc-bbut00000455"	exon_number "2"
1	lncRNA	exon	47250904	47251687	.	+	.	gene_id "lnc-bbug00191"	transcript_id "lnc-bbut00000455"	exon_number "3"
1	lncRNA	exon	47246998	47247142	.	+	.	gene_id "lnc-bbug00191"	transcript_id "lnc-bbut00000456"	exon_number "1"
1	lncRNA	exon	47249626	47249768	.	+	.	gene_id "lnc-bbug00191"	transcript_id "lnc-bbut00000456"	exon_number "2"
1	lncRNA	exon	47250904	47251687	.	+	.	gene_id "lnc-bbug00191"	transcript_id "lnc-bbut00000456"	exon_number "3"
1	lncRNA	exon	47247173	47247563	.	+	.	gene_id "lnc-bbug00191"	transcript_id "lnc-bbut00000457"	exon_number "1"
1	lncRNA	exon	47249626	47249768	.	+	.	gene_id "lnc-bbug00191"	transcript_id "lnc-bbut00000457"	exon_number "2"
1	lncRNA	exon	47250904	47251687	.	+	.	gene_id "lnc-bbug00191"	transcript_id "lnc-bbut00000457"	exon_number "3"
1	lncRNA	exon	48175140	48175287	.	+	.	gene_id "lnc-bbug00198"	transcript_id "lnc-bbut00000480"	exon_number "1"
1	lncRNA	exon	48178881	48178973	.	+	.	gene_id "lnc-bbug00198"	transcript_id "lnc-bbut00000480"	exon_number "2"
1	lncRNA	exon	48197697	48197883	.	+	.	gene_id "lnc-bbug00198"	transcript_id "lnc-bbut00000480"	exon_number "3"
1	lncRNA	exon	49353844	49354529	.	+	.	gene_id "lnc-bbug00202"	transcript_id "lnc-bbut00000488"	exon_number "1"
1	lncRNA	exon	49458524	49459116	.	+	.	gene_id "lnc-bbug00204"	transcript_id "lnc-bbut00000490"	exon_number "1"
1	lncRNA	exon	49479681	49480216	.	+	.	gene_id "lnc-bbug00205"	transcript_id "lnc-bbut00000491"	exon_number "1"

1	lncRNA	exon	49500587	49500952	.	+	.	gene_id "lnc-bbug00206"	transcript_id "lnc-bbut00000492"	exon_number "1"
1	lncRNA	exon	49529046	49529360	.	+	.	gene_id "lnc-bbug00207"	transcript_id "lnc-bbut00000493"	exon_number "1"
1	lncRNA	exon	49533755	49534321	.	+	.	gene_id "lnc-bbug00208"	transcript_id "lnc-bbut00000494"	exon_number "1"
1	lncRNA	exon	50004924	50005395	.	+	.	gene_id "lnc-bbug00210"	transcript_id "lnc-bbut00000496"	exon_number "1"
1	lncRNA	exon	50030267	50030771	.	+	.	gene_id "lnc-bbug00211"	transcript_id "lnc-bbut00000497"	exon_number "1"
1	lncRNA	exon	50108809	50109645	.	+	.	gene_id "lnc-bbug00213"	transcript_id "lnc-bbut00000499"	exon_number "1"
1	lncRNA	exon	51704331	51704790	.	+	.	gene_id "lnc-bbug00221"	transcript_id "lnc-bbut00000507"	exon_number "1"
1	lncRNA	exon	54737046	54737439	.	+	.	gene_id "lnc-bbug00230"	transcript_id "lnc-bbut00000522"	exon_number "1"
1	lncRNA	exon	54742885	54743038	.	+	.	gene_id "lnc-bbug00230"	transcript_id "lnc-bbut00000522"	exon_number "2"
1	lncRNA	exon	54743537	54743819	.	+	.	gene_id "lnc-bbug00230"	transcript_id "lnc-bbut00000522"	exon_number "3"
1	lncRNA	exon	54737048	54737439	.	+	.	gene_id "lnc-bbug00230"	transcript_id "lnc-bbut00000523"	exon_number "1"
1	lncRNA	exon	54742885	54743038	.	+	.	gene_id "lnc-bbug00230"	transcript_id "lnc-bbut00000523"	exon_number "2"
1	lncRNA	exon	54746556	54749904	.	+	.	gene_id "lnc-bbug00230"	transcript_id "lnc-bbut00000523"	exon_number "3"
1	lncRNA	exon	55391004	55391350	.	+	.	gene_id "lnc-bbug00233"	transcript_id "lnc-bbut00000533"	exon_number "1"
1	lncRNA	exon	55391449	55391523	.	+	.	gene_id "lnc-bbug00233"	transcript_id "lnc-bbut00000533"	exon_number "2"
1	lncRNA	exon	55391825	55391866	.	+	.	gene_id "lnc-bbug00233"	transcript_id "lnc-bbut00000533"	exon_number "3"
1	lncRNA	exon	55394165	55394220	.	+	.	gene_id "lnc-bbug00233"	transcript_id "lnc-bbut00000533"	exon_number "4"
1	lncRNA	exon	55391004	55391350	.	+	.	gene_id "lnc-bbug00233"	transcript_id "lnc-bbut00000532"	exon_number "1"
1	lncRNA	exon	55391449	55391523	.	+	.	gene_id "lnc-bbug00233"	transcript_id "lnc-bbut00000532"	exon_number "2"
1	lncRNA	exon	55391622	55391866	.	+	.	gene_id "lnc-bbug00233"	transcript_id "lnc-bbut00000532"	exon_number "3"
1	lncRNA	exon	55394165	55394220	.	+	.	gene_id "lnc-bbug00233"	transcript_id "lnc-bbut00000532"	exon_number "4"
1	lncRNA	exon	56318833	56321396	.	+	.	gene_id "lnc-bbug00235"	transcript_id "lnc-bbut00000538"	exon_number "1"
1	lncRNA	exon	66786541	66787182	.	+	.	gene_id "lnc-bbug00243"	transcript_id "lnc-bbut00000550"	exon_number "1"
1	lncRNA	exon	66805959	66806040	.	+	.	gene_id "lnc-bbug00243"	transcript_id "lnc-bbut00000550"	exon_number "2"
1	lncRNA	exon	66832073	66832181	.	+	.	gene_id "lnc-bbug00243"	transcript_id "lnc-bbut00000550"	exon_number "3"
1	lncRNA	exon	66833096	66833206	.	+	.	gene_id "lnc-bbug00243"	transcript_id "lnc-bbut00000550"	exon_number "4"
1	lncRNA	exon	67107293	67107361	.	+	.	gene_id "lnc-bbug00244"	transcript_id "lnc-bbut00000555"	exon_number "1"
1	lncRNA	exon	67109076	67109727	.	+	.	gene_id "lnc-bbug00244"	transcript_id "lnc-bbut00000555"	exon_number "2"
1	lncRNA	exon	82579121	82579211	.	+	.	gene_id "lnc-bbug00257"	transcript_id "lnc-bbut00000573"	exon_number "1"
1	lncRNA	exon	82749973	82750699	.	+	.	gene_id "lnc-bbug00257"	transcript_id "lnc-bbut00000573"	exon_number "2"
1	lncRNA	exon	85634493	85634503	.	+	.	gene_id "lnc-bbug00261"	transcript_id "lnc-bbut00000582"	exon_number "1"
1	lncRNA	exon	85635772	85635773	.	+	.	gene_id "lnc-bbug00261"	transcript_id "lnc-bbut00000582"	exon_number "2"
1	lncRNA	exon	85653400	85653634	.	+	.	gene_id "lnc-bbug00261"	transcript_id "lnc-bbut00000582"	exon_number "3"
1	lncRNA	exon	87145887	87146797	.	+	.	gene_id "lnc-bbug00268"	transcript_id "lnc-bbut00000599"	exon_number "1"
1	lncRNA	exon	87259399	87259505	.	+	.	gene_id "lnc-bbug00273"	transcript_id "lnc-bbut00000604"	exon_number "1"
1	lncRNA	exon	87261238	87261690	.	+	.	gene_id "lnc-bbug00273"	transcript_id "lnc-bbut00000604"	exon_number "2"
1	lncRNA	exon	87261864	87262075	.	+	.	gene_id "lnc-bbug00273"	transcript_id "lnc-bbut00000604"	exon_number "3"
1	lncRNA	exon	87286390	87286449	.	+	.	gene_id "lnc-bbug00276"	transcript_id "lnc-bbut00000607"	exon_number "1"
1	lncRNA	exon	87288231	87288731	.	+	.	gene_id "lnc-bbug00276"	transcript_id "lnc-bbut00000607"	exon_number "2"
1	lncRNA	exon	87292106	87292535	.	+	.	gene_id "lnc-bbug00276"	transcript_id "lnc-bbut00000607"	exon_number "3"
1	lncRNA	exon	87374233	87374717	.	+	.	gene_id "lnc-bbug00281"	transcript_id "lnc-bbut00000612"	exon_number "1"
1	lncRNA	exon	87724024	87724086	.	+	.	gene_id "lnc-bbug00293"	transcript_id "lnc-bbut00000625"	exon_number "1"
1	lncRNA	exon	87754241	87754384	.	+	.	gene_id "lnc-bbug00293"	transcript_id "lnc-bbut00000625"	exon_number "2"
1	lncRNA	exon	87756281	87756406	.	+	.	gene_id "lnc-bbug00293"	transcript_id "lnc-bbut00000625"	exon_number "3"
1	lncRNA	exon	87757478	87757721	.	+	.	gene_id "lnc-bbug00293"	transcript_id "lnc-bbut00000625"	exon_number "4"
1	lncRNA	exon	87763578	87763717	.	+	.	gene_id "lnc-bbug00293"	transcript_id "lnc-bbut00000625"	exon_number "5"
1	lncRNA	exon	88638266	88638347	.	+	.	gene_id "lnc-bbug00296"	transcript_id "lnc-bbut00000641"	exon_number "1"
1	lncRNA	exon	88639400	88639491	.	+	.	gene_id "lnc-bbug00296"	transcript_id "lnc-bbut00000641"	exon_number "2"
1	lncRNA	exon	88646886	88646944	.	+	.	gene_id "lnc-bbug00296"	transcript_id "lnc-bbut00000641"	exon_number "3"
1	lncRNA	exon	88638266	88638347	.	+	.	gene_id "lnc-bbug00296"	transcript_id "lnc-bbut00000642"	exon_number "1"
1	lncRNA	exon	88639400	88639491	.	+	.	gene_id "lnc-bbug00296"	transcript_id "lnc-bbut00000642"	exon_number "2"
1	lncRNA	exon	88656175	88656738	.	+	.	gene_id "lnc-bbug00296"	transcript_id "lnc-bbut00000642"	exon_number "3"

1	lncRNA	exon	90733499	90734732	.	+	.	gene_id "lnc-bbug00309"	transcript_id "lnc-bbut00000674"	exon_number "1"
1	lncRNA	exon	90735291	90735357	.	+	.	gene_id "lnc-bbug00309"	transcript_id "lnc-bbut00000674"	exon_number "2"
1	lncRNA	exon	90737236	90737490	.	+	.	gene_id "lnc-bbug00309"	transcript_id "lnc-bbut00000674"	exon_number "3"
1	lncRNA	exon	90733499	90734223	.	+	.	gene_id "lnc-bbug00309"	transcript_id "lnc-bbut00000673"	exon_number "1"
1	lncRNA	exon	90735291	90735357	.	+	.	gene_id "lnc-bbug00309"	transcript_id "lnc-bbut00000673"	exon_number "2"
1	lncRNA	exon	90737236	90737490	.	+	.	gene_id "lnc-bbug00309"	transcript_id "lnc-bbut00000673"	exon_number "3"
1	lncRNA	exon	91016138	91016355	.	+	.	gene_id "lnc-bbug00316"	transcript_id "lnc-bbut00000695"	exon_number "1"
1	lncRNA	exon	91067504	91067586	.	+	.	gene_id "lnc-bbug00316"	transcript_id "lnc-bbut00000695"	exon_number "2"
1	lncRNA	exon	91071402	91071537	.	+	.	gene_id "lnc-bbug00316"	transcript_id "lnc-bbut00000695"	exon_number "3"
1	lncRNA	exon	91088961	91089148	.	+	.	gene_id "lnc-bbug00316"	transcript_id "lnc-bbut00000695"	exon_number "4"
1	lncRNA	exon	91095319	91095546	.	+	.	gene_id "lnc-bbug00316"	transcript_id "lnc-bbut00000695"	exon_number "5"
1	lncRNA	exon	91106732	91110941	.	+	.	gene_id "lnc-bbug00316"	transcript_id "lnc-bbut00000695"	exon_number "6"
1	lncRNA	exon	91029627	91030341	.	+	.	gene_id "lnc-bbug00316"	transcript_id "lnc-bbut00000697"	exon_number "1"
1	lncRNA	exon	91067504	91067586	.	+	.	gene_id "lnc-bbug00316"	transcript_id "lnc-bbut00000697"	exon_number "2"
1	lncRNA	exon	91071402	91071537	.	+	.	gene_id "lnc-bbug00316"	transcript_id "lnc-bbut00000697"	exon_number "3"
1	lncRNA	exon	91088961	91089148	.	+	.	gene_id "lnc-bbug00316"	transcript_id "lnc-bbut00000697"	exon_number "4"
1	lncRNA	exon	91095319	91095546	.	+	.	gene_id "lnc-bbug00316"	transcript_id "lnc-bbut00000697"	exon_number "5"
1	lncRNA	exon	91106732	91110941	.	+	.	gene_id "lnc-bbug00316"	transcript_id "lnc-bbut00000697"	exon_number "6"
1	lncRNA	exon	91029627	91030341	.	+	.	gene_id "lnc-bbug00316"	transcript_id "lnc-bbut00000696"	exon_number "1"
1	lncRNA	exon	91040499	91040595	.	+	.	gene_id "lnc-bbug00316"	transcript_id "lnc-bbut00000696"	exon_number "2"
1	lncRNA	exon	91067504	91067586	.	+	.	gene_id "lnc-bbug00316"	transcript_id "lnc-bbut00000696"	exon_number "3"
1	lncRNA	exon	91088961	91089148	.	+	.	gene_id "lnc-bbug00316"	transcript_id "lnc-bbut00000696"	exon_number "4"
1	lncRNA	exon	91095319	91095546	.	+	.	gene_id "lnc-bbug00316"	transcript_id "lnc-bbut00000696"	exon_number "5"
1	lncRNA	exon	91106732	91110941	.	+	.	gene_id "lnc-bbug00316"	transcript_id "lnc-bbut00000696"	exon_number "6"
1	lncRNA	exon	91029628	91030341	.	+	.	gene_id "lnc-bbug00316"	transcript_id "lnc-bbut00000698"	exon_number "1"
1	lncRNA	exon	91040499	91040595	.	+	.	gene_id "lnc-bbug00316"	transcript_id "lnc-bbut00000698"	exon_number "2"
1	lncRNA	exon	91067504	91067586	.	+	.	gene_id "lnc-bbug00316"	transcript_id "lnc-bbut00000698"	exon_number "3"
1	lncRNA	exon	91071402	91071537	.	+	.	gene_id "lnc-bbug00316"	transcript_id "lnc-bbut00000698"	exon_number "4"
1	lncRNA	exon	91088961	91089148	.	+	.	gene_id "lnc-bbug00316"	transcript_id "lnc-bbut00000698"	exon_number "5"
1	lncRNA	exon	91106732	91110941	.	+	.	gene_id "lnc-bbug00316"	transcript_id "lnc-bbut00000698"	exon_number "6"
1	lncRNA	exon	91029629	91030341	.	+	.	gene_id "lnc-bbug00316"	transcript_id "lnc-bbut00000699"	exon_number "1"
1	lncRNA	exon	91040499	91040595	.	+	.	gene_id "lnc-bbug00316"	transcript_id "lnc-bbut00000699"	exon_number "2"
1	lncRNA	exon	91067504	91067586	.	+	.	gene_id "lnc-bbug00316"	transcript_id "lnc-bbut00000699"	exon_number "3"
1	lncRNA	exon	91071402	91071537	.	+	.	gene_id "lnc-bbug00316"	transcript_id "lnc-bbut00000699"	exon_number "4"
1	lncRNA	exon	91088961	91089148	.	+	.	gene_id "lnc-bbug00316"	transcript_id "lnc-bbut00000699"	exon_number "5"
1	lncRNA	exon	91095319	91095546	.	+	.	gene_id "lnc-bbug00316"	transcript_id "lnc-bbut00000699"	exon_number "6"
1	lncRNA	exon	91106732	91110941	.	+	.	gene_id "lnc-bbug00316"	transcript_id "lnc-bbut00000699"	exon_number "7"
1	lncRNA	exon	91365541	91366566	.	+	.	gene_id "lnc-bbug00317"	transcript_id "lnc-bbut00000701"	exon_number "1"
1	lncRNA	exon	91370077	91370167	.	+	.	gene_id "lnc-bbug00317"	transcript_id "lnc-bbut00000701"	exon_number "2"
1	lncRNA	exon	91386484	91387031	.	+	.	gene_id "lnc-bbug00317"	transcript_id "lnc-bbut00000701"	exon_number "3"
1	lncRNA	exon	91391344	91392727	.	+	.	gene_id "lnc-bbug00318"	transcript_id "lnc-bbut00000702"	exon_number "1"
1	lncRNA	exon	91392843	91397084	.	+	.	gene_id "lnc-bbug00318"	transcript_id "lnc-bbut00000702"	exon_number "2"
1	lncRNA	exon	94506252	94506277	.	+	.	gene_id "lnc-bbug00320"	transcript_id "lnc-bbut00000704"	exon_number "1"
1	lncRNA	exon	94534395	94534897	.	+	.	gene_id "lnc-bbug00320"	transcript_id "lnc-bbut00000704"	exon_number "2"
1	lncRNA	exon	95803770	95804473	.	+	.	gene_id "lnc-bbug00322"	transcript_id "lnc-bbut00000707"	exon_number "1"
1	lncRNA	exon	96586268	96586877	.	+	.	gene_id "lnc-bbug00323"	transcript_id "lnc-bbut00000708"	exon_number "1"
1	lncRNA	exon	96597133	96597535	.	+	.	gene_id "lnc-bbug00324"	transcript_id "lnc-bbut00000709"	exon_number "1"
1	lncRNA	exon	96601530	96602869	.	+	.	gene_id "lnc-bbug00324"	transcript_id "lnc-bbut00000709"	exon_number "2"
1	lncRNA	exon	96824076	96825548	.	+	.	gene_id "lnc-bbug00326"	transcript_id "lnc-bbut00000711"	exon_number "1"
1	lncRNA	exon	96825994	96826310	.	+	.	gene_id "lnc-bbug00326"	transcript_id "lnc-bbut00000711"	exon_number "2"
1	lncRNA	exon	97478494	97478726	.	+	.	gene_id "lnc-bbug00329"	transcript_id "lnc-bbut00000733"	exon_number "1"
1	lncRNA	exon	97494139	97494244	.	+	.	gene_id "lnc-bbug00329"	transcript_id "lnc-bbut00000733"	exon_number "2"

1	lncRNA	exon	97494911	97495048	.	+	.	gene_id "lnc-bbug00329"	transcript_id "lnc-bbut00000733"	exon_number "3"
1	lncRNA	exon	97498889	97499134	.	+	.	gene_id "lnc-bbug00329"	transcript_id "lnc-bbut00000733"	exon_number "4"
1	lncRNA	exon	97493203	97493314	.	+	.	gene_id "lnc-bbug00329"	transcript_id "lnc-bbut00000735"	exon_number "1"
1	lncRNA	exon	97494139	97494244	.	+	.	gene_id "lnc-bbug00329"	transcript_id "lnc-bbut00000735"	exon_number "2"
1	lncRNA	exon	97494914	97495048	.	+	.	gene_id "lnc-bbug00329"	transcript_id "lnc-bbut00000735"	exon_number "3"
1	lncRNA	exon	97498889	97499134	.	+	.	gene_id "lnc-bbug00329"	transcript_id "lnc-bbut00000735"	exon_number "4"
1	lncRNA	exon	97493203	97493314	.	+	.	gene_id "lnc-bbug00329"	transcript_id "lnc-bbut00000734"	exon_number "1"
1	lncRNA	exon	97494139	97494244	.	+	.	gene_id "lnc-bbug00329"	transcript_id "lnc-bbut00000734"	exon_number "2"
1	lncRNA	exon	97494911	97495048	.	+	.	gene_id "lnc-bbug00329"	transcript_id "lnc-bbut00000734"	exon_number "3"
1	lncRNA	exon	97498889	97499134	.	+	.	gene_id "lnc-bbug00329"	transcript_id "lnc-bbut00000734"	exon_number "4"
1	lncRNA	exon	98746521	98746593	.	+	.	gene_id "lnc-bbug00334"	transcript_id "lnc-bbut00000757"	exon_number "1"
1	lncRNA	exon	98746694	98746746	.	+	.	gene_id "lnc-bbug00334"	transcript_id "lnc-bbut00000757"	exon_number "2"
1	lncRNA	exon	98762181	98762314	.	+	.	gene_id "lnc-bbug00334"	transcript_id "lnc-bbut00000757"	exon_number "3"
1	lncRNA	exon	98763829	98764163	.	+	.	gene_id "lnc-bbug00334"	transcript_id "lnc-bbut00000757"	exon_number "4"
1	lncRNA	exon	100944890	100945040	.	+	.	gene_id "lnc-bbug00338"	transcript_id "lnc-bbut00000762"	exon_number "1"
1	lncRNA	exon	100954476	100954563	.	+	.	gene_id "lnc-bbug00338"	transcript_id "lnc-bbut00000762"	exon_number "2"
1	lncRNA	exon	100956954	100957203	.	+	.	gene_id "lnc-bbug00338"	transcript_id "lnc-bbut00000762"	exon_number "3"
1	lncRNA	exon	100962669	100965925	.	+	.	gene_id "lnc-bbug00339"	transcript_id "lnc-bbut00000763"	exon_number "1"
1	lncRNA	exon	100967702	100967808	.	+	.	gene_id "lnc-bbug00339"	transcript_id "lnc-bbut00000763"	exon_number "2"
1	lncRNA	exon	100969693	100969827	.	+	.	gene_id "lnc-bbug00339"	transcript_id "lnc-bbut00000763"	exon_number "3"
1	lncRNA	exon	101207825	101208549	.	+	.	gene_id "lnc-bbug00341"	transcript_id "lnc-bbut00000769"	exon_number "1"
1	lncRNA	exon	101208581	101209627	.	+	.	gene_id "lnc-bbug00341"	transcript_id "lnc-bbut00000769"	exon_number "2"
1	lncRNA	exon	101765134	101766704	.	+	.	gene_id "lnc-bbug00348"	transcript_id "lnc-bbut00000796"	exon_number "1"
1	lncRNA	exon	101768503	101768624	.	+	.	gene_id "lnc-bbug00348"	transcript_id "lnc-bbut00000796"	exon_number "2"
1	lncRNA	exon	101789900	101790023	.	+	.	gene_id "lnc-bbug00348"	transcript_id "lnc-bbut00000796"	exon_number "3"
1	lncRNA	exon	102388277	102388865	.	+	.	gene_id "lnc-bbug00352"	transcript_id "lnc-bbut00000806"	exon_number "1"
1	lncRNA	exon	103147889	103148420	.	+	.	gene_id "lnc-bbug00357"	transcript_id "lnc-bbut00000813"	exon_number "1"
1	lncRNA	exon	103148656	103148734	.	+	.	gene_id "lnc-bbug00357"	transcript_id "lnc-bbut00000813"	exon_number "2"
1	lncRNA	exon	103152902	103153065	.	+	.	gene_id "lnc-bbug00357"	transcript_id "lnc-bbut00000813"	exon_number "3"
1	lncRNA	exon	103154517	103154687	.	+	.	gene_id "lnc-bbug00357"	transcript_id "lnc-bbut00000813"	exon_number "4"
1	lncRNA	exon	103147928	103148734	.	+	.	gene_id "lnc-bbug00357"	transcript_id "lnc-bbut00000814"	exon_number "1"
1	lncRNA	exon	103152902	103153065	.	+	.	gene_id "lnc-bbug00357"	transcript_id "lnc-bbut00000814"	exon_number "2"
1	lncRNA	exon	103154517	103154687	.	+	.	gene_id "lnc-bbug00357"	transcript_id "lnc-bbut00000814"	exon_number "3"
1	lncRNA	exon	103147947	103148257	.	+	.	gene_id "lnc-bbug00357"	transcript_id "lnc-bbut00000815"	exon_number "1"
1	lncRNA	exon	103152902	103153065	.	+	.	gene_id "lnc-bbug00357"	transcript_id "lnc-bbut00000815"	exon_number "2"
1	lncRNA	exon	103154517	103154687	.	+	.	gene_id "lnc-bbug00357"	transcript_id "lnc-bbut00000815"	exon_number "3"
1	lncRNA	exon	103155193	103156337	.	+	.	gene_id "lnc-bbug00358"	transcript_id "lnc-bbut00000816"	exon_number "1"
1	lncRNA	exon	103156618	103159349	.	+	.	gene_id "lnc-bbug00358"	transcript_id "lnc-bbut00000816"	exon_number "2"
1	lncRNA	exon	104675292	104676024	.	+	.	gene_id "lnc-bbug00365"	transcript_id "lnc-bbut00000830"	exon_number "1"
1	lncRNA	exon	105954238	105955147	.	+	.	gene_id "lnc-bbug00367"	transcript_id "lnc-bbut00000833"	exon_number "1"
1	lncRNA	exon	111725309	111725409	.	+	.	gene_id "lnc-bbug00388"	transcript_id "lnc-bbut00000883"	exon_number "1"
1	lncRNA	exon	111725864	111726079	.	+	.	gene_id "lnc-bbug00388"	transcript_id "lnc-bbut00000883"	exon_number "2"
1	lncRNA	exon	111726706	111727689	.	+	.	gene_id "lnc-bbug00388"	transcript_id "lnc-bbut00000883"	exon_number "3"
1	lncRNA	exon	111725545	111726079	.	+	.	gene_id "lnc-bbug00388"	transcript_id "lnc-bbut00000884"	exon_number "1"
1	lncRNA	exon	111726706	111727689	.	+	.	gene_id "lnc-bbug00388"	transcript_id "lnc-bbut00000884"	exon_number "2"
1	lncRNA	exon	112064317	112065821	.	+	.	gene_id "lnc-bbug00392"	transcript_id "lnc-bbut00000888"	exon_number "1"
1	lncRNA	exon	112066101	112068667	.	+	.	gene_id "lnc-bbug00392"	transcript_id "lnc-bbut00000888"	exon_number "2"
1	lncRNA	exon	112079145	112079321	.	+	.	gene_id "lnc-bbug00392"	transcript_id "lnc-bbut00000888"	exon_number "3"
1	lncRNA	exon	112079481	112079663	.	+	.	gene_id "lnc-bbug00392"	transcript_id "lnc-bbut00000888"	exon_number "4"
1	lncRNA	exon	112065828	112068667	.	+	.	gene_id "lnc-bbug00392"	transcript_id "lnc-bbut00000889"	exon_number "1"
1	lncRNA	exon	112079145	112079321	.	+	.	gene_id "lnc-bbug00392"	transcript_id "lnc-bbut00000889"	exon_number "2"
1	lncRNA	exon	112079481	112079663	.	+	.	gene_id "lnc-bbug00392"	transcript_id "lnc-bbut00000889"	exon_number "3"

1	lncRNA	exon	112897329	112900115	.	+	.	gene_id "lnc-bbug00395"	transcript_id "lnc-bbut00000894"	exon_number "1"
1	lncRNA	exon	113350153	113351135	.	+	.	gene_id "lnc-bbug00398"	transcript_id "lnc-bbut00000916"	exon_number "1"
1	lncRNA	exon	113351408	113353728	.	+	.	gene_id "lnc-bbug00398"	transcript_id "lnc-bbut00000916"	exon_number "2"
1	lncRNA	exon	114675685	114675829	.	+	.	gene_id "lnc-bbug00401"	transcript_id "lnc-bbut00000923"	exon_number "1"
1	lncRNA	exon	114679186	114679280	.	+	.	gene_id "lnc-bbug00401"	transcript_id "lnc-bbut00000923"	exon_number "2"
1	lncRNA	exon	114692976	114693364	.	+	.	gene_id "lnc-bbug00401"	transcript_id "lnc-bbut00000923"	exon_number "3"
1	lncRNA	exon	115607711	115607945	.	+	.	gene_id "lnc-bbug00407"	transcript_id "lnc-bbut00000939"	exon_number "1"
1	lncRNA	exon	115610585	115610829	.	+	.	gene_id "lnc-bbug00407"	transcript_id "lnc-bbut00000939"	exon_number "2"
1	lncRNA	exon	115792664	115792931	.	+	.	gene_id "lnc-bbug00409"	transcript_id "lnc-bbut00000942"	exon_number "1"
1	lncRNA	exon	115813955	115814049	.	+	.	gene_id "lnc-bbug00409"	transcript_id "lnc-bbut00000942"	exon_number "2"
1	lncRNA	exon	115814155	115814788	.	+	.	gene_id "lnc-bbug00409"	transcript_id "lnc-bbut00000942"	exon_number "3"
1	lncRNA	exon	115819306	115820351	.	+	.	gene_id "lnc-bbug00410"	transcript_id "lnc-bbut00000943"	exon_number "1"
1	lncRNA	exon	116221920	116222655	.	+	.	gene_id "lnc-bbug00416"	transcript_id "lnc-bbut00000956"	exon_number "1"
1	lncRNA	exon	116657285	116657378	.	+	.	gene_id "lnc-bbug00417"	transcript_id "lnc-bbut00000957"	exon_number "1"
1	lncRNA	exon	116658188	116658319	.	+	.	gene_id "lnc-bbug00417"	transcript_id "lnc-bbut00000957"	exon_number "2"
1	lncRNA	exon	116658725	116659042	.	+	.	gene_id "lnc-bbug00417"	transcript_id "lnc-bbut00000957"	exon_number "3"
1	lncRNA	exon	116814713	116815053	.	+	.	gene_id "lnc-bbug00418"	transcript_id "lnc-bbut00000958"	exon_number "1"
1	lncRNA	exon	116815451	116815492	.	+	.	gene_id "lnc-bbug00418"	transcript_id "lnc-bbut00000958"	exon_number "2"
1	lncRNA	exon	116815818	116816253	.	+	.	gene_id "lnc-bbug00418"	transcript_id "lnc-bbut00000958"	exon_number "3"
1	lncRNA	exon	116814714	116815053	.	+	.	gene_id "lnc-bbug00418"	transcript_id "lnc-bbut00000959"	exon_number "1"
1	lncRNA	exon	116815451	116815492	.	+	.	gene_id "lnc-bbug00418"	transcript_id "lnc-bbut00000959"	exon_number "2"
1	lncRNA	exon	116815836	116816253	.	+	.	gene_id "lnc-bbug00418"	transcript_id "lnc-bbut00000959"	exon_number "3"
1	lncRNA	exon	116814716	116815053	.	+	.	gene_id "lnc-bbug00418"	transcript_id "lnc-bbut00000960"	exon_number "1"
1	lncRNA	exon	116815836	116816253	.	+	.	gene_id "lnc-bbug00418"	transcript_id "lnc-bbut00000960"	exon_number "2"
1	lncRNA	exon	116818135	116818630	.	+	.	gene_id "lnc-bbug00419"	transcript_id "lnc-bbut00000961"	exon_number "1"
1	lncRNA	exon	116821926	116822320	.	+	.	gene_id "lnc-bbug00419"	transcript_id "lnc-bbut00000961"	exon_number "2"
1	lncRNA	exon	117360358	117361496	.	+	.	gene_id "lnc-bbug00424"	transcript_id "lnc-bbut00000974"	exon_number "1"
1	lncRNA	exon	117362405	117362543	.	+	.	gene_id "lnc-bbug00424"	transcript_id "lnc-bbut00000974"	exon_number "2"
1	lncRNA	exon	117362910	117364941	.	+	.	gene_id "lnc-bbug00424"	transcript_id "lnc-bbut00000974"	exon_number "3"
1	lncRNA	exon	118070975	118072816	.	+	.	gene_id "lnc-bbug00432"	transcript_id "lnc-bbut00000996"	exon_number "1"
1	lncRNA	exon	118072980	118073054	.	+	.	gene_id "lnc-bbug00432"	transcript_id "lnc-bbut00000996"	exon_number "2"
1	lncRNA	exon	118075622	118076547	.	+	.	gene_id "lnc-bbug00432"	transcript_id "lnc-bbut00000996"	exon_number "3"
1	lncRNA	exon	118078601	118079323	.	+	.	gene_id "lnc-bbug00432"	transcript_id "lnc-bbut00000996"	exon_number "4"
1	lncRNA	exon	118201439	118201613	.	+	.	gene_id "lnc-bbug00433"	transcript_id "lnc-bbut00000997"	exon_number "1"
1	lncRNA	exon	118204171	118204494	.	+	.	gene_id "lnc-bbug00433"	transcript_id "lnc-bbut00000997"	exon_number "2"
1	lncRNA	exon	118367981	118373725	.	+	.	gene_id "lnc-bbug00434"	transcript_id "lnc-bbut00000998"	exon_number "1"
1	lncRNA	exon	118373921	118374000	.	+	.	gene_id "lnc-bbug00434"	transcript_id "lnc-bbut00000998"	exon_number "2"
1	lncRNA	exon	118375391	118375481	.	+	.	gene_id "lnc-bbug00434"	transcript_id "lnc-bbut00000998"	exon_number "3"
1	lncRNA	exon	118387171	118387318	.	+	.	gene_id "lnc-bbug00434"	transcript_id "lnc-bbut00000998"	exon_number "4"
1	lncRNA	exon	118388582	118388685	.	+	.	gene_id "lnc-bbug00434"	transcript_id "lnc-bbut00000998"	exon_number "5"
1	lncRNA	exon	118405515	118405794	.	+	.	gene_id "lnc-bbug00435"	transcript_id "lnc-bbut00000999"	exon_number "1"
1	lncRNA	exon	118411689	118411977	.	+	.	gene_id "lnc-bbug00435"	transcript_id "lnc-bbut00000999"	exon_number "2"
1	lncRNA	exon	118422306	118422429	.	+	.	gene_id "lnc-bbug00436"	transcript_id "lnc-bbut00001000"	exon_number "1"
1	lncRNA	exon	118436472	118436648	.	+	.	gene_id "lnc-bbug00436"	transcript_id "lnc-bbut00001000"	exon_number "2"
1	lncRNA	exon	118441539	118442471	.	+	.	gene_id "lnc-bbug00436"	transcript_id "lnc-bbut00001000"	exon_number "3"
1	lncRNA	exon	120926023	120926127	.	+	.	gene_id "lnc-bbug00443"	transcript_id "lnc-bbut00001014"	exon_number "1"
1	lncRNA	exon	120931107	120931312	.	+	.	gene_id "lnc-bbug00443"	transcript_id "lnc-bbut00001014"	exon_number "2"
1	lncRNA	exon	120986027	120986295	.	+	.	gene_id "lnc-bbug00443"	transcript_id "lnc-bbut00001014"	exon_number "3"
1	lncRNA	exon	121078186	121078580	.	+	.	gene_id "lnc-bbug00444"	transcript_id "lnc-bbut00001015"	exon_number "1"
1	lncRNA	exon	121176681	121176768	.	+	.	gene_id "lnc-bbug00444"	transcript_id "lnc-bbut00001015"	exon_number "2"
1	lncRNA	exon	121178300	121178781	.	+	.	gene_id "lnc-bbug00444"	transcript_id "lnc-bbut00001015"	exon_number "3"
1	lncRNA	exon	121128564	121128812	.	+	.	gene_id "lnc-bbug00445"	transcript_id "lnc-bbut00001016"	exon_number "1"

1	lncRNA	exon	121141959	121142300	.	+	.	gene_id "lnc-bbug00445"	transcript_id "lnc-bbut00001016"	exon_number "2"
1	lncRNA	exon	121287715	121288008	.	+	.	gene_id "lnc-bbug00447"	transcript_id "lnc-bbut00001018"	exon_number "1"
1	lncRNA	exon	121296465	121296538	.	+	.	gene_id "lnc-bbug00447"	transcript_id "lnc-bbut00001018"	exon_number "2"
1	lncRNA	exon	121297362	121298440	.	+	.	gene_id "lnc-bbug00447"	transcript_id "lnc-bbut00001018"	exon_number "3"
1	lncRNA	exon	122805390	122805431	.	+	.	gene_id "lnc-bbug00451"	transcript_id "lnc-bbut00001022"	exon_number "1"
1	lncRNA	exon	122805538	122806171	.	+	.	gene_id "lnc-bbug00451"	transcript_id "lnc-bbut00001022"	exon_number "2"
1	lncRNA	exon	122808338	122808379	.	+	.	gene_id "lnc-bbug00451"	transcript_id "lnc-bbut00001022"	exon_number "3"
1	lncRNA	exon	122810745	122810808	.	+	.	gene_id "lnc-bbug00451"	transcript_id "lnc-bbut00001022"	exon_number "4"
1	lncRNA	exon	122814655	122814776	.	+	.	gene_id "lnc-bbug00451"	transcript_id "lnc-bbut00001022"	exon_number "5"
1	lncRNA	exon	122961936	122964202	.	+	.	gene_id "lnc-bbug00452"	transcript_id "lnc-bbut00001023"	exon_number "1"
1	lncRNA	exon	122964582	122964640	.	+	.	gene_id "lnc-bbug00452"	transcript_id "lnc-bbut00001023"	exon_number "2"
1	lncRNA	exon	122967790	122968264	.	+	.	gene_id "lnc-bbug00452"	transcript_id "lnc-bbut00001023"	exon_number "3"
1	lncRNA	exon	123588697	123588774	.	+	.	gene_id "lnc-bbug00453"	transcript_id "lnc-bbut00001024"	exon_number "1"
1	lncRNA	exon	123597640	123597878	.	+	.	gene_id "lnc-bbug00453"	transcript_id "lnc-bbut00001024"	exon_number "2"
1	lncRNA	exon	123602093	123602235	.	+	.	gene_id "lnc-bbug00453"	transcript_id "lnc-bbut00001024"	exon_number "3"
1	lncRNA	exon	124164509	124164713	.	+	.	gene_id "lnc-bbug00454"	transcript_id "lnc-bbut00001025"	exon_number "1"
1	lncRNA	exon	124179308	124179420	.	+	.	gene_id "lnc-bbug00454"	transcript_id "lnc-bbut00001025"	exon_number "2"
1	lncRNA	exon	124190815	124191047	.	+	.	gene_id "lnc-bbug00454"	transcript_id "lnc-bbut00001025"	exon_number "3"
1	lncRNA	exon	124169279	124170164	.	+	.	gene_id "lnc-bbug00454"	transcript_id "lnc-bbut00001026"	exon_number "1"
1	lncRNA	exon	124179308	124179420	.	+	.	gene_id "lnc-bbug00454"	transcript_id "lnc-bbut00001026"	exon_number "2"
1	lncRNA	exon	124190815	124191047	.	+	.	gene_id "lnc-bbug00454"	transcript_id "lnc-bbut00001026"	exon_number "3"
1	lncRNA	exon	124204925	124206365	.	+	.	gene_id "lnc-bbug00455"	transcript_id "lnc-bbut00001027"	exon_number "1"
1	lncRNA	exon	124210849	124210972	.	+	.	gene_id "lnc-bbug00455"	transcript_id "lnc-bbut00001027"	exon_number "2"
1	lncRNA	exon	124228834	124229214	.	+	.	gene_id "lnc-bbug00455"	transcript_id "lnc-bbut00001027"	exon_number "3"
1	lncRNA	exon	124893803	124893940	.	+	.	gene_id "lnc-bbug00460"	transcript_id "lnc-bbut00001044"	exon_number "1"
1	lncRNA	exon	124898912	124899094	.	+	.	gene_id "lnc-bbug00460"	transcript_id "lnc-bbut00001044"	exon_number "2"
1	lncRNA	exon	124921682	124921741	.	+	.	gene_id "lnc-bbug00460"	transcript_id "lnc-bbut00001044"	exon_number "3"
1	lncRNA	exon	124928595	124928893	.	+	.	gene_id "lnc-bbug00460"	transcript_id "lnc-bbut00001044"	exon_number "4"
1	lncRNA	exon	124893803	124893940	.	+	.	gene_id "lnc-bbug00460"	transcript_id "lnc-bbut00001045"	exon_number "1"
1	lncRNA	exon	124898912	124899094	.	+	.	gene_id "lnc-bbug00460"	transcript_id "lnc-bbut00001045"	exon_number "2"
1	lncRNA	exon	124921682	124921741	.	+	.	gene_id "lnc-bbug00460"	transcript_id "lnc-bbut00001045"	exon_number "3"
1	lncRNA	exon	124934352	124936102	.	+	.	gene_id "lnc-bbug00460"	transcript_id "lnc-bbut00001045"	exon_number "4"
1	lncRNA	exon	124940503	124940839	.	+	.	gene_id "lnc-bbug00461"	transcript_id "lnc-bbut00001047"	exon_number "1"
1	lncRNA	exon	124941702	124941785	.	+	.	gene_id "lnc-bbug00461"	transcript_id "lnc-bbut00001047"	exon_number "2"
1	lncRNA	exon	124949882	124950408	.	+	.	gene_id "lnc-bbug00461"	transcript_id "lnc-bbut00001047"	exon_number "3"
1	lncRNA	exon	124960807	124963117	.	+	.	gene_id "lnc-bbug00461"	transcript_id "lnc-bbut00001047"	exon_number "4"
1	lncRNA	exon	124940503	124940839	.	+	.	gene_id "lnc-bbug00461"	transcript_id "lnc-bbut00001046"	exon_number "1"
1	lncRNA	exon	124941702	124941785	.	+	.	gene_id "lnc-bbug00461"	transcript_id "lnc-bbut00001046"	exon_number "2"
1	lncRNA	exon	124949882	124950408	.	+	.	gene_id "lnc-bbug00461"	transcript_id "lnc-bbut00001046"	exon_number "3"
1	lncRNA	exon	124960783	124963117	.	+	.	gene_id "lnc-bbug00461"	transcript_id "lnc-bbut00001046"	exon_number "4"
1	lncRNA	exon	125000381	125000475	.	+	.	gene_id "lnc-bbug00462"	transcript_id "lnc-bbut00001048"	exon_number "1"
1	lncRNA	exon	125001075	125001181	.	+	.	gene_id "lnc-bbug00462"	transcript_id "lnc-bbut00001048"	exon_number "2"
1	lncRNA	exon	125011719	125011808	.	+	.	gene_id "lnc-bbug00462"	transcript_id "lnc-bbut00001048"	exon_number "3"
1	lncRNA	exon	125013612	125013701	.	+	.	gene_id "lnc-bbug00462"	transcript_id "lnc-bbut00001048"	exon_number "4"
1	lncRNA	exon	125017680	125017885	.	+	.	gene_id "lnc-bbug00462"	transcript_id "lnc-bbut00001048"	exon_number "5"
1	lncRNA	exon	125025334	125025672	.	+	.	gene_id "lnc-bbug00462"	transcript_id "lnc-bbut00001048"	exon_number "6"
1	lncRNA	exon	125030185	125030373	.	+	.	gene_id "lnc-bbug00462"	transcript_id "lnc-bbut00001048"	exon_number "7"
1	lncRNA	exon	125031381	125031477	.	+	.	gene_id "lnc-bbug00462"	transcript_id "lnc-bbut00001048"	exon_number "8"
1	lncRNA	exon	125038166	125038442	.	+	.	gene_id "lnc-bbug00462"	transcript_id "lnc-bbut00001048"	exon_number "9"
1	lncRNA	exon	125447399	125451588	.	+	.	gene_id "lnc-bbug00464"	transcript_id "lnc-bbut00001052"	exon_number "1"
1	lncRNA	exon	125455023	125456062	.	+	.	gene_id "lnc-bbug00464"	transcript_id "lnc-bbut00001052"	exon_number "2"
1	lncRNA	exon	127573655	127573916	.	+	.	gene_id "lnc-bbug00478"	transcript_id "lnc-bbut00001085"	exon_number "1"

1	lncRNA	exon	127574199	127574422	.	+	.	gene_id "lnc-bbug00478"	transcript_id "lnc-bbut00001085"	exon_number "2"
1	lncRNA	exon	127761623	127761980	.	+	.	gene_id "lnc-bbug00482"	transcript_id "lnc-bbut00001094"	exon_number "1"
1	lncRNA	exon	127762873	127763901	.	+	.	gene_id "lnc-bbug00482"	transcript_id "lnc-bbut00001094"	exon_number "2"
1	lncRNA	exon	128145756	128146367	.	+	.	gene_id "lnc-bbug00488"	transcript_id "lnc-bbut00001109"	exon_number "1"
1	lncRNA	exon	128151214	128151331	.	+	.	gene_id "lnc-bbug00488"	transcript_id "lnc-bbut00001109"	exon_number "2"
1	lncRNA	exon	128151825	128152043	.	+	.	gene_id "lnc-bbug00488"	transcript_id "lnc-bbut00001109"	exon_number "3"
1	lncRNA	exon	128152157	128154541	.	+	.	gene_id "lnc-bbug00488"	transcript_id "lnc-bbut00001109"	exon_number "4"
1	lncRNA	exon	128155790	128157861	.	+	.	gene_id "lnc-bbug00488"	transcript_id "lnc-bbut00001109"	exon_number "5"
1	lncRNA	exon	130177445	130178160	.	+	.	gene_id "lnc-bbug00495"	transcript_id "lnc-bbut00001133"	exon_number "1"
1	lncRNA	exon	130254008	130254081	.	+	.	gene_id "lnc-bbug00495"	transcript_id "lnc-bbut00001133"	exon_number "2"
1	lncRNA	exon	130258763	130265911	.	+	.	gene_id "lnc-bbug00495"	transcript_id "lnc-bbut00001133"	exon_number "3"
1	lncRNA	exon	132398602	132398638	.	+	.	gene_id "lnc-bbug00500"	transcript_id "lnc-bbut00001147"	exon_number "1"
1	lncRNA	exon	132422214	132422732	.	+	.	gene_id "lnc-bbug00500"	transcript_id "lnc-bbut00001147"	exon_number "2"
1	lncRNA	exon	133450012	133450162	.	+	.	gene_id "lnc-bbug00505"	transcript_id "lnc-bbut00001164"	exon_number "1"
1	lncRNA	exon	133473159	133473247	.	+	.	gene_id "lnc-bbug00505"	transcript_id "lnc-bbut00001164"	exon_number "2"
1	lncRNA	exon	133596183	133596284	.	+	.	gene_id "lnc-bbug00505"	transcript_id "lnc-bbut00001164"	exon_number "3"
1	lncRNA	exon	133596932	133599660	.	+	.	gene_id "lnc-bbug00505"	transcript_id "lnc-bbut00001164"	exon_number "4"
1	lncRNA	exon	134089670	134090153	.	+	.	gene_id "lnc-bbug00507"	transcript_id "lnc-bbut00001166"	exon_number "1"
1	lncRNA	exon	134090260	134090360	.	+	.	gene_id "lnc-bbug00507"	transcript_id "lnc-bbut00001166"	exon_number "2"
1	lncRNA	exon	134097310	134099339	.	+	.	gene_id "lnc-bbug00507"	transcript_id "lnc-bbut00001166"	exon_number "3"
1	lncRNA	exon	134385585	134385634	.	+	.	gene_id "lnc-bbug00508"	transcript_id "lnc-bbut00001168"	exon_number "1"
1	lncRNA	exon	134387822	134387917	.	+	.	gene_id "lnc-bbug00508"	transcript_id "lnc-bbut00001168"	exon_number "2"
1	lncRNA	exon	134390544	134390824	.	+	.	gene_id "lnc-bbug00508"	transcript_id "lnc-bbut00001168"	exon_number "3"
1	lncRNA	exon	134385585	134385634	.	+	.	gene_id "lnc-bbug00508"	transcript_id "lnc-bbut00001167"	exon_number "1"
1	lncRNA	exon	134387822	134387909	.	+	.	gene_id "lnc-bbug00508"	transcript_id "lnc-bbut00001167"	exon_number "2"
1	lncRNA	exon	134390544	134390824	.	+	.	gene_id "lnc-bbug00508"	transcript_id "lnc-bbut00001167"	exon_number "3"
1	lncRNA	exon	134894097	134894277	.	+	.	gene_id "lnc-bbug00510"	transcript_id "lnc-bbut00001180"	exon_number "1"
1	lncRNA	exon	134894841	134894899	.	+	.	gene_id "lnc-bbug00510"	transcript_id "lnc-bbut00001180"	exon_number "2"
1	lncRNA	exon	134899330	134899666	.	+	.	gene_id "lnc-bbug00510"	transcript_id "lnc-bbut00001180"	exon_number "3"
1	lncRNA	exon	135741637	135742412	.	+	.	gene_id "lnc-bbug00511"	transcript_id "lnc-bbut00001181"	exon_number "1"
1	lncRNA	exon	136947848	136947905	.	+	.	gene_id "lnc-bbug00513"	transcript_id "lnc-bbut00001183"	exon_number "1"
1	lncRNA	exon	136999525	136999581	.	+	.	gene_id "lnc-bbug00513"	transcript_id "lnc-bbut00001183"	exon_number "2"
1	lncRNA	exon	137011260	137011421	.	+	.	gene_id "lnc-bbug00513"	transcript_id "lnc-bbut00001183"	exon_number "3"
1	lncRNA	exon	137011900	137011994	.	+	.	gene_id "lnc-bbug00513"	transcript_id "lnc-bbut00001183"	exon_number "4"
1	lncRNA	exon	137015433	137015542	.	+	.	gene_id "lnc-bbug00513"	transcript_id "lnc-bbut00001183"	exon_number "5"
1	lncRNA	exon	137016479	137017250	.	+	.	gene_id "lnc-bbug00513"	transcript_id "lnc-bbut00001183"	exon_number "6"
1	lncRNA	exon	136947848	136947905	.	+	.	gene_id "lnc-bbug00513"	transcript_id "lnc-bbut00001185"	exon_number "1"
1	lncRNA	exon	136999525	136999581	.	+	.	gene_id "lnc-bbug00513"	transcript_id "lnc-bbut00001185"	exon_number "2"
1	lncRNA	exon	137011260	137011421	.	+	.	gene_id "lnc-bbug00513"	transcript_id "lnc-bbut00001185"	exon_number "3"
1	lncRNA	exon	137011900	137011994	.	+	.	gene_id "lnc-bbug00513"	transcript_id "lnc-bbut00001185"	exon_number "4"
1	lncRNA	exon	137015433	137015542	.	+	.	gene_id "lnc-bbug00513"	transcript_id "lnc-bbut00001185"	exon_number "5"
1	lncRNA	exon	137016479	137016562	.	+	.	gene_id "lnc-bbug00513"	transcript_id "lnc-bbut00001185"	exon_number "6"
1	lncRNA	exon	137017946	137018395	.	+	.	gene_id "lnc-bbug00513"	transcript_id "lnc-bbut00001185"	exon_number "7"
1	lncRNA	exon	136947848	136947905	.	+	.	gene_id "lnc-bbug00513"	transcript_id "lnc-bbut00001184"	exon_number "1"
1	lncRNA	exon	136999525	136999581	.	+	.	gene_id "lnc-bbug00513"	transcript_id "lnc-bbut00001184"	exon_number "2"
1	lncRNA	exon	137011260	137011421	.	+	.	gene_id "lnc-bbug00513"	transcript_id "lnc-bbut00001184"	exon_number "3"
1	lncRNA	exon	137011900	137011994	.	+	.	gene_id "lnc-bbug00513"	transcript_id "lnc-bbut00001184"	exon_number "4"
1	lncRNA	exon	137015433	137015542	.	+	.	gene_id "lnc-bbug00513"	transcript_id "lnc-bbut00001184"	exon_number "5"
1	lncRNA	exon	137017946	137018395	.	+	.	gene_id "lnc-bbug00513"	transcript_id "lnc-bbut00001184"	exon_number "6"
1	lncRNA	exon	137981386	137983150	.	+	.	gene_id "lnc-bbug00515"	transcript_id "lnc-bbut00001187"	exon_number "1"
1	lncRNA	exon	138270161	138272511	.	+	.	gene_id "lnc-bbug00516"	transcript_id "lnc-bbut00001188"	exon_number "1"
1	lncRNA	exon	139625339	139625482	.	+	.	gene_id "lnc-bbug00521"	transcript_id "lnc-bbut00001197"	exon_number "1"

1	lncRNA	exon	139626566	139626917	.	+	.	gene_id "lnc-bbug00521"	transcript_id "lnc-bbut00001197"	exon_number "2"
1	lncRNA	exon	139630970	139631147	.	+	.	gene_id "lnc-bbug00521"	transcript_id "lnc-bbut00001197"	exon_number "3"
1	lncRNA	exon	139631821	139631921	.	+	.	gene_id "lnc-bbug00521"	transcript_id "lnc-bbut00001197"	exon_number "4"
1	lncRNA	exon	140394228	140394236	.	+	.	gene_id "lnc-bbug00523"	transcript_id "lnc-bbut00001210"	exon_number "1"
1	lncRNA	exon	140395992	140396773	.	+	.	gene_id "lnc-bbug00523"	transcript_id "lnc-bbut00001210"	exon_number "2"
1	lncRNA	exon	143938053	143941884	.	+	.	gene_id "lnc-bbug00538"	transcript_id "lnc-bbut00001256"	exon_number "1"
1	lncRNA	exon	143942299	143944741	.	+	.	gene_id "lnc-bbug00538"	transcript_id "lnc-bbut00001256"	exon_number "2"
1	lncRNA	exon	144872569	144872608	.	+	.	gene_id "lnc-bbug00543"	transcript_id "lnc-bbut00001270"	exon_number "1"
1	lncRNA	exon	144872711	144872796	.	+	.	gene_id "lnc-bbug00543"	transcript_id "lnc-bbut00001270"	exon_number "2"
1	lncRNA	exon	144876047	144876399	.	+	.	gene_id "lnc-bbug00543"	transcript_id "lnc-bbut00001270"	exon_number "3"
1	lncRNA	exon	146208997	146211026	.	+	.	gene_id "lnc-bbug00545"	transcript_id "lnc-bbut00001272"	exon_number "1"
1	lncRNA	exon	146221116	146221252	.	+	.	gene_id "lnc-bbug00545"	transcript_id "lnc-bbut00001272"	exon_number "2"
1	lncRNA	exon	146224463	146224560	.	+	.	gene_id "lnc-bbug00545"	transcript_id "lnc-bbut00001272"	exon_number "3"
1	lncRNA	exon	146270094	146272480	.	+	.	gene_id "lnc-bbug00545"	transcript_id "lnc-bbut00001272"	exon_number "4"
1	lncRNA	exon	146870243	146871639	.	+	.	gene_id "lnc-bbug00548"	transcript_id "lnc-bbut00001276"	exon_number "1"
1	lncRNA	exon	147745496	147745541	.	+	.	gene_id "lnc-bbug00550"	transcript_id "lnc-bbut00001281"	exon_number "1"
1	lncRNA	exon	147758665	147758848	.	+	.	gene_id "lnc-bbug00550"	transcript_id "lnc-bbut00001281"	exon_number "2"
1	lncRNA	exon	148963856	148964915	.	+	.	gene_id "lnc-bbug00553"	transcript_id "lnc-bbut00001284"	exon_number "1"
1	lncRNA	exon	149891483	149891574	.	+	.	gene_id "lnc-bbug00555"	transcript_id "lnc-bbut00001286"	exon_number "1"
1	lncRNA	exon	149904391	149904584	.	+	.	gene_id "lnc-bbug00555"	transcript_id "lnc-bbut00001286"	exon_number "2"
1	lncRNA	exon	149904675	149905121	.	+	.	gene_id "lnc-bbug00555"	transcript_id "lnc-bbut00001286"	exon_number "3"
1	lncRNA	exon	150621096	150621354	.	+	.	gene_id "lnc-bbug00557"	transcript_id "lnc-bbut00001289"	exon_number "1"
1	lncRNA	exon	150632423	150632516	.	+	.	gene_id "lnc-bbug00557"	transcript_id "lnc-bbut00001289"	exon_number "2"
1	lncRNA	exon	150642106	150642169	.	+	.	gene_id "lnc-bbug00557"	transcript_id "lnc-bbut00001289"	exon_number "3"
1	lncRNA	exon	150648798	150650368	.	+	.	gene_id "lnc-bbug00557"	transcript_id "lnc-bbut00001289"	exon_number "4"
1	lncRNA	exon	150621096	150621354	.	+	.	gene_id "lnc-bbug00557"	transcript_id "lnc-bbut00001288"	exon_number "1"
1	lncRNA	exon	150632423	150632516	.	+	.	gene_id "lnc-bbug00557"	transcript_id "lnc-bbut00001288"	exon_number "2"
1	lncRNA	exon	150648798	150650368	.	+	.	gene_id "lnc-bbug00557"	transcript_id "lnc-bbut00001288"	exon_number "3"
1	lncRNA	exon	150653345	150653413	.	+	.	gene_id "lnc-bbug00558"	transcript_id "lnc-bbut00001290"	exon_number "1"
1	lncRNA	exon	150658689	150658903	.	+	.	gene_id "lnc-bbug00558"	transcript_id "lnc-bbut00001290"	exon_number "2"
1	lncRNA	exon	150659355	150659678	.	+	.	gene_id "lnc-bbug00558"	transcript_id "lnc-bbut00001290"	exon_number "3"
1	lncRNA	exon	151673368	151674055	.	+	.	gene_id "lnc-bbug00565"	transcript_id "lnc-bbut00001322"	exon_number "1"
1	lncRNA	exon	151674849	151675705	.	+	.	gene_id "lnc-bbug00565"	transcript_id "lnc-bbut00001322"	exon_number "2"
1	lncRNA	exon	152019684	152019791	.	+	.	gene_id "lnc-bbug00567"	transcript_id "lnc-bbut00001325"	exon_number "1"
1	lncRNA	exon	152020127	152020350	.	+	.	gene_id "lnc-bbug00567"	transcript_id "lnc-bbut00001325"	exon_number "2"
1	lncRNA	exon	152020475	152021509	.	+	.	gene_id "lnc-bbug00567"	transcript_id "lnc-bbut00001325"	exon_number "3"
1	lncRNA	exon	152090011	152090379	.	+	.	gene_id "lnc-bbug00568"	transcript_id "lnc-bbut00001326"	exon_number "1"
1	lncRNA	exon	152108589	152108749	.	+	.	gene_id "lnc-bbug00568"	transcript_id "lnc-bbut00001326"	exon_number "2"
1	lncRNA	exon	152111208	152111815	.	+	.	gene_id "lnc-bbug00568"	transcript_id "lnc-bbut00001326"	exon_number "3"
1	lncRNA	exon	152180963	152181123	.	+	.	gene_id "lnc-bbug00569"	transcript_id "lnc-bbut00001327"	exon_number "1"
1	lncRNA	exon	152183936	152184077	.	+	.	gene_id "lnc-bbug00569"	transcript_id "lnc-bbut00001327"	exon_number "2"
1	lncRNA	exon	152185001	152186094	.	+	.	gene_id "lnc-bbug00569"	transcript_id "lnc-bbut00001327"	exon_number "3"
1	lncRNA	exon	152481458	152482440	.	+	.	gene_id "lnc-bbug00570"	transcript_id "lnc-bbut00001328"	exon_number "1"
1	lncRNA	exon	152508222	152508289	.	+	.	gene_id "lnc-bbug00570"	transcript_id "lnc-bbut00001328"	exon_number "2"
1	lncRNA	exon	152509766	152510768	.	+	.	gene_id "lnc-bbug00570"	transcript_id "lnc-bbut00001328"	exon_number "3"
1	lncRNA	exon	152481458	152482440	.	+	.	gene_id "lnc-bbug00570"	transcript_id "lnc-bbut00001329"	exon_number "1"
1	lncRNA	exon	152508222	152508289	.	+	.	gene_id "lnc-bbug00570"	transcript_id "lnc-bbut00001329"	exon_number "2"
1	lncRNA	exon	152509766	152509911	.	+	.	gene_id "lnc-bbug00570"	transcript_id "lnc-bbut00001329"	exon_number "3"
1	lncRNA	exon	152526801	152526884	.	+	.	gene_id "lnc-bbug00570"	transcript_id "lnc-bbut00001329"	exon_number "4"
1	lncRNA	exon	153437338	153437835	.	+	.	gene_id "lnc-bbug00573"	transcript_id "lnc-bbut00001332"	exon_number "1"
1	lncRNA	exon	153439486	153441445	.	+	.	gene_id "lnc-bbug00573"	transcript_id "lnc-bbut00001332"	exon_number "2"
1	lncRNA	exon	155816372	155816533	.	+	.	gene_id "lnc-bbug00578"	transcript_id "lnc-bbut00001347"	exon_number "1"

1	lncRNA	exon	155816675	155817728	.	+	.	gene_id "lnc-bbug00578"	transcript_id "lnc-bbut00001347"	exon_number "2"
1	lncRNA	exon	156897735	156897867	.	+	.	gene_id "lnc-bbug00584"	transcript_id "lnc-bbut00001359"	exon_number "1"
1	lncRNA	exon	156900637	156900735	.	+	.	gene_id "lnc-bbug00584"	transcript_id "lnc-bbut00001359"	exon_number "2"
1	lncRNA	exon	156900833	156900963	.	+	.	gene_id "lnc-bbug00584"	transcript_id "lnc-bbut00001359"	exon_number "3"
1	lncRNA	exon	159138414	159138460	.	+	.	gene_id "lnc-bbug00588"	transcript_id "lnc-bbut00001364"	exon_number "1"
1	lncRNA	exon	159165118	159165178	.	+	.	gene_id "lnc-bbug00588"	transcript_id "lnc-bbut00001364"	exon_number "2"
1	lncRNA	exon	159203173	159203355	.	+	.	gene_id "lnc-bbug00588"	transcript_id "lnc-bbut00001364"	exon_number "3"
1	lncRNA	exon	159204239	159204336	.	+	.	gene_id "lnc-bbug00588"	transcript_id "lnc-bbut00001364"	exon_number "4"
1	lncRNA	exon	159204861	159205622	.	+	.	gene_id "lnc-bbug00588"	transcript_id "lnc-bbut00001364"	exon_number "5"
1	lncRNA	exon	159207981	159208039	.	+	.	gene_id "lnc-bbug00588"	transcript_id "lnc-bbut00001364"	exon_number "6"
1	lncRNA	exon	159188244	159188297	.	+	.	gene_id "lnc-bbug00588"	transcript_id "lnc-bbut00001365"	exon_number "1"
1	lncRNA	exon	159203173	159203355	.	+	.	gene_id "lnc-bbug00588"	transcript_id "lnc-bbut00001365"	exon_number "2"
1	lncRNA	exon	159204239	159204336	.	+	.	gene_id "lnc-bbug00588"	transcript_id "lnc-bbut00001365"	exon_number "3"
1	lncRNA	exon	159204861	159205622	.	+	.	gene_id "lnc-bbug00588"	transcript_id "lnc-bbut00001365"	exon_number "4"
1	lncRNA	exon	159207981	159208039	.	+	.	gene_id "lnc-bbug00588"	transcript_id "lnc-bbut00001365"	exon_number "5"
1	lncRNA	exon	159198304	159198624	.	+	.	gene_id "lnc-bbug00588"	transcript_id "lnc-bbut00001366"	exon_number "1"
1	lncRNA	exon	159203173	159203355	.	+	.	gene_id "lnc-bbug00588"	transcript_id "lnc-bbut00001366"	exon_number "2"
1	lncRNA	exon	159204239	159204336	.	+	.	gene_id "lnc-bbug00588"	transcript_id "lnc-bbut00001366"	exon_number "3"
1	lncRNA	exon	159204861	159205622	.	+	.	gene_id "lnc-bbug00588"	transcript_id "lnc-bbut00001366"	exon_number "4"
1	lncRNA	exon	159207981	159208039	.	+	.	gene_id "lnc-bbug00588"	transcript_id "lnc-bbut00001366"	exon_number "5"
1	lncRNA	exon	160089884	160090019	.	+	.	gene_id "lnc-bbug00589"	transcript_id "lnc-bbut00001367"	exon_number "1"
1	lncRNA	exon	160128821	160128903	.	+	.	gene_id "lnc-bbug00589"	transcript_id "lnc-bbut00001367"	exon_number "2"
1	lncRNA	exon	160130349	160131315	.	+	.	gene_id "lnc-bbug00589"	transcript_id "lnc-bbut00001367"	exon_number "3"
1	lncRNA	exon	160402245	160402550	.	+	.	gene_id "lnc-bbug00590"	transcript_id "lnc-bbut00001368"	exon_number "1"
1	lncRNA	exon	162141137	162141630	.	+	.	gene_id "lnc-bbug00600"	transcript_id "lnc-bbut00001396"	exon_number "1"
1	lncRNA	exon	162141909	162141984	.	+	.	gene_id "lnc-bbug00600"	transcript_id "lnc-bbut00001396"	exon_number "2"
1	lncRNA	exon	162143169	162145708	.	+	.	gene_id "lnc-bbug00600"	transcript_id "lnc-bbut00001396"	exon_number "3"
1	lncRNA	exon	162150227	162151468	.	+	.	gene_id "lnc-bbug00601"	transcript_id "lnc-bbut00001397"	exon_number "1"
1	lncRNA	exon	162595946	162596158	.	+	.	gene_id "lnc-bbug00602"	transcript_id "lnc-bbut00001398"	exon_number "1"
1	lncRNA	exon	162654519	162654622	.	+	.	gene_id "lnc-bbug00602"	transcript_id "lnc-bbut00001398"	exon_number "2"
1	lncRNA	exon	162660950	162661120	.	+	.	gene_id "lnc-bbug00602"	transcript_id "lnc-bbut00001398"	exon_number "3"
1	lncRNA	exon	162670298	162670758	.	+	.	gene_id "lnc-bbug00602"	transcript_id "lnc-bbut00001398"	exon_number "4"
1	lncRNA	exon	162674699	162674866	.	+	.	gene_id "lnc-bbug00602"	transcript_id "lnc-bbut00001398"	exon_number "5"
1	lncRNA	exon	162679857	162680398	.	+	.	gene_id "lnc-bbug00602"	transcript_id "lnc-bbut00001398"	exon_number "6"
1	lncRNA	exon	162664970	162665072	.	+	.	gene_id "lnc-bbug00602"	transcript_id "lnc-bbut00001399"	exon_number "1"
1	lncRNA	exon	162670298	162670758	.	+	.	gene_id "lnc-bbug00602"	transcript_id "lnc-bbut00001399"	exon_number "2"
1	lncRNA	exon	162674699	162674866	.	+	.	gene_id "lnc-bbug00602"	transcript_id "lnc-bbut00001399"	exon_number "3"
1	lncRNA	exon	162679857	162680398	.	+	.	gene_id "lnc-bbug00602"	transcript_id "lnc-bbut00001399"	exon_number "4"
1	lncRNA	exon	163568235	163569343	.	+	.	gene_id "lnc-bbug00612"	transcript_id "lnc-bbut00001414"	exon_number "1"
1	lncRNA	exon	164981569	164981996	.	+	.	gene_id "lnc-bbug00614"	transcript_id "lnc-bbut00001417"	exon_number "1"
1	lncRNA	exon	164989929	164990126	.	+	.	gene_id "lnc-bbug00614"	transcript_id "lnc-bbut00001417"	exon_number "2"
1	lncRNA	exon	164990173	164990398	.	+	.	gene_id "lnc-bbug00614"	transcript_id "lnc-bbut00001417"	exon_number "3"
1	lncRNA	exon	165386043	165386661	.	+	.	gene_id "lnc-bbug00615"	transcript_id "lnc-bbut00001418"	exon_number "1"
1	lncRNA	exon	165419940	165420147	.	+	.	gene_id "lnc-bbug00616"	transcript_id "lnc-bbut00001419"	exon_number "1"
1	lncRNA	exon	165423836	165423920	.	+	.	gene_id "lnc-bbug00616"	transcript_id "lnc-bbut00001419"	exon_number "2"
1	lncRNA	exon	165435042	165435360	.	+	.	gene_id "lnc-bbug00616"	transcript_id "lnc-bbut00001419"	exon_number "3"
1	lncRNA	exon	165437474	165440278	.	+	.	gene_id "lnc-bbug00616"	transcript_id "lnc-bbut00001419"	exon_number "4"
1	lncRNA	exon	165422545	165422653	.	+	.	gene_id "lnc-bbug00616"	transcript_id "lnc-bbut00001423"	exon_number "1"
1	lncRNA	exon	165423836	165423920	.	+	.	gene_id "lnc-bbug00616"	transcript_id "lnc-bbut00001423"	exon_number "2"
1	lncRNA	exon	165435042	165435360	.	+	.	gene_id "lnc-bbug00616"	transcript_id "lnc-bbut00001423"	exon_number "3"
1	lncRNA	exon	165437474	165440278	.	+	.	gene_id "lnc-bbug00616"	transcript_id "lnc-bbut00001423"	exon_number "4"
1	lncRNA	exon	165758080	165758193	.	+	.	gene_id "lnc-bbug00617"	transcript_id "lnc-bbut00001426"	exon_number "1"

1	lncRNA	exon	165761410	165761501	.	+	.	gene_id "lnc-bbug00617"	transcript_id "lnc-bbut00001426"	exon_number "2"
1	lncRNA	exon	165852393	165853542	.	+	.	gene_id "lnc-bbug00617"	transcript_id "lnc-bbut00001426"	exon_number "3"
1	lncRNA	exon	169419289	169419442	.	+	.	gene_id "lnc-bbug00625"	transcript_id "lnc-bbut00001448"	exon_number "1"
1	lncRNA	exon	169426972	169427064	.	+	.	gene_id "lnc-bbug00625"	transcript_id "lnc-bbut00001448"	exon_number "2"
1	lncRNA	exon	169427230	169427460	.	+	.	gene_id "lnc-bbug00625"	transcript_id "lnc-bbut00001448"	exon_number "3"
1	lncRNA	exon	169469944	169470058	.	+	.	gene_id "lnc-bbug00625"	transcript_id "lnc-bbut00001448"	exon_number "4"
1	lncRNA	exon	169474603	169474617	.	+	.	gene_id "lnc-bbug00625"	transcript_id "lnc-bbut00001448"	exon_number "5"
1	lncRNA	exon	169419291	169419488	.	+	.	gene_id "lnc-bbug00625"	transcript_id "lnc-bbut00001449"	exon_number "1"
1	lncRNA	exon	169426972	169427064	.	+	.	gene_id "lnc-bbug00625"	transcript_id "lnc-bbut00001449"	exon_number "2"
1	lncRNA	exon	169427230	169427796	.	+	.	gene_id "lnc-bbug00625"	transcript_id "lnc-bbut00001449"	exon_number "3"
1	lncRNA	exon	169419294	169419442	.	+	.	gene_id "lnc-bbug00625"	transcript_id "lnc-bbut00001450"	exon_number "1"
1	lncRNA	exon	169426972	169427064	.	+	.	gene_id "lnc-bbug00625"	transcript_id "lnc-bbut00001450"	exon_number "2"
1	lncRNA	exon	169427230	169427460	.	+	.	gene_id "lnc-bbug00625"	transcript_id "lnc-bbut00001450"	exon_number "3"
1	lncRNA	exon	169428370	169428764	.	+	.	gene_id "lnc-bbug00625"	transcript_id "lnc-bbut00001450"	exon_number "4"
1	lncRNA	exon	169419294	169419442	.	+	.	gene_id "lnc-bbug00625"	transcript_id "lnc-bbut00001451"	exon_number "1"
1	lncRNA	exon	169426972	169427064	.	+	.	gene_id "lnc-bbug00625"	transcript_id "lnc-bbut00001451"	exon_number "2"
1	lncRNA	exon	169427230	169427460	.	+	.	gene_id "lnc-bbug00625"	transcript_id "lnc-bbut00001451"	exon_number "3"
1	lncRNA	exon	169433346	169433721	.	+	.	gene_id "lnc-bbug00625"	transcript_id "lnc-bbut00001451"	exon_number "4"
1	lncRNA	exon	169419294	169419442	.	+	.	gene_id "lnc-bbug00625"	transcript_id "lnc-bbut00001452"	exon_number "1"
1	lncRNA	exon	169426972	169427064	.	+	.	gene_id "lnc-bbug00625"	transcript_id "lnc-bbut00001452"	exon_number "2"
1	lncRNA	exon	169427230	169427460	.	+	.	gene_id "lnc-bbug00625"	transcript_id "lnc-bbut00001452"	exon_number "3"
1	lncRNA	exon	169442316	169442725	.	+	.	gene_id "lnc-bbug00625"	transcript_id "lnc-bbut00001452"	exon_number "4"
1	lncRNA	exon	169419294	169419442	.	+	.	gene_id "lnc-bbug00625"	transcript_id "lnc-bbut00001453"	exon_number "1"
1	lncRNA	exon	169426972	169427064	.	+	.	gene_id "lnc-bbug00625"	transcript_id "lnc-bbut00001453"	exon_number "2"
1	lncRNA	exon	169427230	169427460	.	+	.	gene_id "lnc-bbug00625"	transcript_id "lnc-bbut00001453"	exon_number "3"
1	lncRNA	exon	169464884	169465198	.	+	.	gene_id "lnc-bbug00625"	transcript_id "lnc-bbut00001453"	exon_number "4"
1	lncRNA	exon	169419294	169419442	.	+	.	gene_id "lnc-bbug00625"	transcript_id "lnc-bbut00001454"	exon_number "1"
1	lncRNA	exon	169426972	169427064	.	+	.	gene_id "lnc-bbug00625"	transcript_id "lnc-bbut00001454"	exon_number "2"
1	lncRNA	exon	169427230	169427460	.	+	.	gene_id "lnc-bbug00625"	transcript_id "lnc-bbut00001454"	exon_number "3"
1	lncRNA	exon	169469944	169470368	.	+	.	gene_id "lnc-bbug00625"	transcript_id "lnc-bbut00001454"	exon_number "4"
1	lncRNA	exon	169419295	169419442	.	+	.	gene_id "lnc-bbug00625"	transcript_id "lnc-bbut00001455"	exon_number "1"
1	lncRNA	exon	169426972	169427064	.	+	.	gene_id "lnc-bbug00625"	transcript_id "lnc-bbut00001455"	exon_number "2"
1	lncRNA	exon	169427230	169427796	.	+	.	gene_id "lnc-bbug00625"	transcript_id "lnc-bbut00001455"	exon_number "3"
1	lncRNA	exon	171958932	171959545	.	+	.	gene_id "lnc-bbug00635"	transcript_id "lnc-bbut00001493"	exon_number "1"
1	lncRNA	exon	174769920	174770642	.	+	.	gene_id "lnc-bbug00644"	transcript_id "lnc-bbut00001503"	exon_number "1"
1	lncRNA	exon	175183483	175183557	.	+	.	gene_id "lnc-bbug00648"	transcript_id "lnc-bbut00001510"	exon_number "1"
1	lncRNA	exon	175195648	175196246	.	+	.	gene_id "lnc-bbug00648"	transcript_id "lnc-bbut00001510"	exon_number "2"
1	lncRNA	exon	175283199	175283347	.	+	.	gene_id "lnc-bbug00649"	transcript_id "lnc-bbut00001511"	exon_number "1"
1	lncRNA	exon	175283643	175284312	.	+	.	gene_id "lnc-bbug00649"	transcript_id "lnc-bbut00001511"	exon_number "2"
1	lncRNA	exon	175348698	175350040	.	+	.	gene_id "lnc-bbug00651"	transcript_id "lnc-bbut00001519"	exon_number "1"
1	lncRNA	exon	175351129	175351209	.	+	.	gene_id "lnc-bbug00651"	transcript_id "lnc-bbut00001519"	exon_number "2"
1	lncRNA	exon	175352635	175352908	.	+	.	gene_id "lnc-bbug00651"	transcript_id "lnc-bbut00001519"	exon_number "3"
1	lncRNA	exon	175535022	175535227	.	+	.	gene_id "lnc-bbug00654"	transcript_id "lnc-bbut00001525"	exon_number "1"
1	lncRNA	exon	175538324	175538400	.	+	.	gene_id "lnc-bbug00654"	transcript_id "lnc-bbut00001525"	exon_number "2"
1	lncRNA	exon	175545988	175546028	.	+	.	gene_id "lnc-bbug00654"	transcript_id "lnc-bbut00001525"	exon_number "3"
1	lncRNA	exon	176845283	176846282	.	+	.	gene_id "lnc-bbug00657"	transcript_id "lnc-bbut00001529"	exon_number "1"
1	lncRNA	exon	176856752	176856883	.	+	.	gene_id "lnc-bbug00657"	transcript_id "lnc-bbut00001529"	exon_number "2"
1	lncRNA	exon	176858579	176860208	.	+	.	gene_id "lnc-bbug00657"	transcript_id "lnc-bbut00001529"	exon_number "3"
1	lncRNA	exon	178662669	178663463	.	+	.	gene_id "lnc-bbug00661"	transcript_id "lnc-bbut00001535"	exon_number "1"
1	lncRNA	exon	178666470	178667459	.	+	.	gene_id "lnc-bbug00661"	transcript_id "lnc-bbut00001535"	exon_number "2"
1	lncRNA	exon	178668783	178671204	.	+	.	gene_id "lnc-bbug00661"	transcript_id "lnc-bbut00001535"	exon_number "3"
1	lncRNA	exon	179169862	179169914	.	+	.	gene_id "lnc-bbug00662"	transcript_id "lnc-bbut00001536"	exon_number "1"

1	lncRNA	exon	179172996	179173135	.	+	.	gene_id "lnc-bbug00662"	transcript_id "lnc-bbut00001536"	exon_number "2"
1	lncRNA	exon	179173230	179173940	.	+	.	gene_id "lnc-bbug00662"	transcript_id "lnc-bbut00001536"	exon_number "3"
1	lncRNA	exon	179401822	179401954	.	+	.	gene_id "lnc-bbug00664"	transcript_id "lnc-bbut00001538"	exon_number "1"
1	lncRNA	exon	179403204	179403334	.	+	.	gene_id "lnc-bbug00664"	transcript_id "lnc-bbut00001538"	exon_number "2"
1	lncRNA	exon	179407025	179407427	.	+	.	gene_id "lnc-bbug00664"	transcript_id "lnc-bbut00001538"	exon_number "3"
1	lncRNA	exon	179401992	179402194	.	+	.	gene_id "lnc-bbug00664"	transcript_id "lnc-bbut00001539"	exon_number "1"
1	lncRNA	exon	179403204	179403334	.	+	.	gene_id "lnc-bbug00664"	transcript_id "lnc-bbut00001539"	exon_number "2"
1	lncRNA	exon	179407025	179407427	.	+	.	gene_id "lnc-bbug00664"	transcript_id "lnc-bbut00001539"	exon_number "3"
1	lncRNA	exon	179402008	179402237	.	+	.	gene_id "lnc-bbug00664"	transcript_id "lnc-bbut00001540"	exon_number "1"
1	lncRNA	exon	179403204	179403334	.	+	.	gene_id "lnc-bbug00664"	transcript_id "lnc-bbut00001540"	exon_number "2"
1	lncRNA	exon	179407025	179407427	.	+	.	gene_id "lnc-bbug00664"	transcript_id "lnc-bbut00001540"	exon_number "3"
1	lncRNA	exon	179402385	179402522	.	+	.	gene_id "lnc-bbug00664"	transcript_id "lnc-bbut00001541"	exon_number "1"
1	lncRNA	exon	179403204	179403334	.	+	.	gene_id "lnc-bbug00664"	transcript_id "lnc-bbut00001541"	exon_number "2"
1	lncRNA	exon	179407025	179407427	.	+	.	gene_id "lnc-bbug00664"	transcript_id "lnc-bbut00001541"	exon_number "3"
1	lncRNA	exon	179402547	179403074	.	+	.	gene_id "lnc-bbug00664"	transcript_id "lnc-bbut00001542"	exon_number "1"
1	lncRNA	exon	179403204	179403334	.	+	.	gene_id "lnc-bbug00664"	transcript_id "lnc-bbut00001542"	exon_number "2"
1	lncRNA	exon	179407025	179407427	.	+	.	gene_id "lnc-bbug00664"	transcript_id "lnc-bbut00001542"	exon_number "3"
1	lncRNA	exon	179854352	179854690	.	+	.	gene_id "lnc-bbug00667"	transcript_id "lnc-bbut00001548"	exon_number "1"
1	lncRNA	exon	179855132	179855267	.	+	.	gene_id "lnc-bbug00667"	transcript_id "lnc-bbut00001548"	exon_number "2"
1	lncRNA	exon	179915132	179917801	.	+	.	gene_id "lnc-bbug00669"	transcript_id "lnc-bbut00001550"	exon_number "1"
1	lncRNA	exon	180640822	180643717	.	+	.	gene_id "lnc-bbug00673"	transcript_id "lnc-bbut00001556"	exon_number "1"
1	lncRNA	exon	181786743	181786852	.	+	.	gene_id "lnc-bbug00676"	transcript_id "lnc-bbut00001562"	exon_number "1"
1	lncRNA	exon	181837312	181837439	.	+	.	gene_id "lnc-bbug00676"	transcript_id "lnc-bbut00001562"	exon_number "2"
1	lncRNA	exon	181845697	181845792	.	+	.	gene_id "lnc-bbug00676"	transcript_id "lnc-bbut00001562"	exon_number "3"
1	lncRNA	exon	181848570	181848626	.	+	.	gene_id "lnc-bbug00676"	transcript_id "lnc-bbut00001562"	exon_number "4"
1	lncRNA	exon	181848753	181848848	.	+	.	gene_id "lnc-bbug00676"	transcript_id "lnc-bbut00001562"	exon_number "5"
1	lncRNA	exon	184226779	184227338	.	+	.	gene_id "lnc-bbug00684"	transcript_id "lnc-bbut00001580"	exon_number "1"
1	lncRNA	exon	184255828	184256279	.	+	.	gene_id "lnc-bbug00684"	transcript_id "lnc-bbut00001580"	exon_number "2"
1	lncRNA	exon	184505825	184505949	.	+	.	gene_id "lnc-bbug00687"	transcript_id "lnc-bbut00001588"	exon_number "1"
1	lncRNA	exon	184536493	184536544	.	+	.	gene_id "lnc-bbug00687"	transcript_id "lnc-bbut00001588"	exon_number "2"
1	lncRNA	exon	184572311	184572722	.	+	.	gene_id "lnc-bbug00687"	transcript_id "lnc-bbut00001588"	exon_number "3"
1	lncRNA	exon	186286490	186286607	.	+	.	gene_id "lnc-bbug00692"	transcript_id "lnc-bbut00001602"	exon_number "1"
1	lncRNA	exon	186289778	186289859	.	+	.	gene_id "lnc-bbug00692"	transcript_id "lnc-bbut00001602"	exon_number "2"
1	lncRNA	exon	186290188	186290612	.	+	.	gene_id "lnc-bbug00692"	transcript_id "lnc-bbut00001602"	exon_number "3"
1	lncRNA	exon	187600452	187600559	.	+	.	gene_id "lnc-bbug00700"	transcript_id "lnc-bbut00001623"	exon_number "1"
1	lncRNA	exon	187601079	187601319	.	+	.	gene_id "lnc-bbug00700"	transcript_id "lnc-bbut00001623"	exon_number "2"
1	lncRNA	exon	187602763	187603102	.	+	.	gene_id "lnc-bbug00700"	transcript_id "lnc-bbut00001623"	exon_number "3"
1	lncRNA	exon	187665859	187666016	.	+	.	gene_id "lnc-bbug00701"	transcript_id "lnc-bbut00001625"	exon_number "1"
1	lncRNA	exon	187667851	187667971	.	+	.	gene_id "lnc-bbug00701"	transcript_id "lnc-bbut00001625"	exon_number "2"
1	lncRNA	exon	187676739	187677324	.	+	.	gene_id "lnc-bbug00701"	transcript_id "lnc-bbut00001625"	exon_number "3"
1	lncRNA	exon	187793029	187794723	.	+	.	gene_id "lnc-bbug00703"	transcript_id "lnc-bbut00001628"	exon_number "1"
1	lncRNA	exon	187795662	187795750	.	+	.	gene_id "lnc-bbug00703"	transcript_id "lnc-bbut00001628"	exon_number "2"
1	lncRNA	exon	187798078	187799243	.	+	.	gene_id "lnc-bbug00703"	transcript_id "lnc-bbut00001628"	exon_number "3"
1	lncRNA	exon	187793029	187794723	.	+	.	gene_id "lnc-bbug00703"	transcript_id "lnc-bbut00001630"	exon_number "1"
1	lncRNA	exon	187795662	187795750	.	+	.	gene_id "lnc-bbug00703"	transcript_id "lnc-bbut00001630"	exon_number "2"
1	lncRNA	exon	187811360	187811613	.	+	.	gene_id "lnc-bbug00703"	transcript_id "lnc-bbut00001630"	exon_number "3"
1	lncRNA	exon	187793029	187795750	.	+	.	gene_id "lnc-bbug00703"	transcript_id "lnc-bbut00001629"	exon_number "1"
1	lncRNA	exon	187811360	187811613	.	+	.	gene_id "lnc-bbug00703"	transcript_id "lnc-bbut00001629"	exon_number "2"
1	lncRNA	exon	187874983	187875294	.	+	.	gene_id "lnc-bbug00705"	transcript_id "lnc-bbut00001632"	exon_number "1"
1	lncRNA	exon	187876762	187877746	.	+	.	gene_id "lnc-bbug00705"	transcript_id "lnc-bbut00001632"	exon_number "2"
1	lncRNA	exon	189412290	189412474	.	+	.	gene_id "lnc-bbug00718"	transcript_id "lnc-bbut00001658"	exon_number "1"
1	lncRNA	exon	189414757	189414871	.	+	.	gene_id "lnc-bbug00718"	transcript_id "lnc-bbut00001658"	exon_number "2"

1	lncRNA	exon	189416041	189416236	.	+	.	gene_id "lnc-bbug00718"	transcript_id "lnc-bbut00001658"	exon_number "3"
1	lncRNA	exon	189416757	189418852	.	+	.	gene_id "lnc-bbug00718"	transcript_id "lnc-bbut00001658"	exon_number "4"
1	lncRNA	exon	189412290	189412474	.	+	.	gene_id "lnc-bbug00718"	transcript_id "lnc-bbut00001657"	exon_number "1"
1	lncRNA	exon	189416041	189416236	.	+	.	gene_id "lnc-bbug00718"	transcript_id "lnc-bbut00001657"	exon_number "2"
1	lncRNA	exon	189416757	189418852	.	+	.	gene_id "lnc-bbug00718"	transcript_id "lnc-bbut00001657"	exon_number "3"
1	lncRNA	exon	189888972	189889378	.	+	.	gene_id "lnc-bbug00721"	transcript_id "lnc-bbut00001669"	exon_number "1"
1	lncRNA	exon	189890638	189891365	.	+	.	gene_id "lnc-bbug00721"	transcript_id "lnc-bbut00001669"	exon_number "2"
1	lncRNA	exon	190451069	190451204	.	+	.	gene_id "lnc-bbug00725"	transcript_id "lnc-bbut00001687"	exon_number "1"
1	lncRNA	exon	190452175	190452329	.	+	.	gene_id "lnc-bbug00725"	transcript_id "lnc-bbut00001687"	exon_number "2"
1	lncRNA	exon	190452843	190452941	.	+	.	gene_id "lnc-bbug00725"	transcript_id "lnc-bbut00001687"	exon_number "3"
1	lncRNA	exon	190453365	190453498	.	+	.	gene_id "lnc-bbug00725"	transcript_id "lnc-bbut00001687"	exon_number "4"
1	lncRNA	exon	190451069	190451204	.	+	.	gene_id "lnc-bbug00725"	transcript_id "lnc-bbut00001688"	exon_number "1"
1	lncRNA	exon	190452175	190452329	.	+	.	gene_id "lnc-bbug00725"	transcript_id "lnc-bbut00001688"	exon_number "2"
1	lncRNA	exon	190452843	190452941	.	+	.	gene_id "lnc-bbug00725"	transcript_id "lnc-bbut00001688"	exon_number "3"
1	lncRNA	exon	190456064	190456637	.	+	.	gene_id "lnc-bbug00725"	transcript_id "lnc-bbut00001688"	exon_number "4"
1	lncRNA	exon	190451069	190451204	.	+	.	gene_id "lnc-bbug00725"	transcript_id "lnc-bbut00001689"	exon_number "1"
1	lncRNA	exon	190452175	190452329	.	+	.	gene_id "lnc-bbug00725"	transcript_id "lnc-bbut00001689"	exon_number "2"
1	lncRNA	exon	190452843	190452941	.	+	.	gene_id "lnc-bbug00725"	transcript_id "lnc-bbut00001689"	exon_number "3"
1	lncRNA	exon	190458825	190459090	.	+	.	gene_id "lnc-bbug00725"	transcript_id "lnc-bbut00001689"	exon_number "4"
1	lncRNA	exon	191756559	191756867	.	+	.	gene_id "lnc-bbug00732"	transcript_id "lnc-bbut00001728"	exon_number "1"
1	lncRNA	exon	191763642	191764201	.	+	.	gene_id "lnc-bbug00732"	transcript_id "lnc-bbut00001728"	exon_number "2"
1	lncRNA	exon	192048379	192048685	.	+	.	gene_id "lnc-bbug00733"	transcript_id "lnc-bbut00001729"	exon_number "1"
1	lncRNA	exon	192049294	192049800	.	+	.	gene_id "lnc-bbug00733"	transcript_id "lnc-bbut00001729"	exon_number "2"
1	lncRNA	exon	192053143	192053586	.	+	.	gene_id "lnc-bbug00733"	transcript_id "lnc-bbut00001729"	exon_number "3"
1	lncRNA	exon	192168692	192170851	.	+	.	gene_id "lnc-bbug00734"	transcript_id "lnc-bbut00001730"	exon_number "1"
1	lncRNA	exon	192181028	192181736	.	+	.	gene_id "lnc-bbug00734"	transcript_id "lnc-bbut00001730"	exon_number "2"
1	lncRNA	exon	192168692	192170851	.	+	.	gene_id "lnc-bbug00734"	transcript_id "lnc-bbut00001731"	exon_number "1"
1	lncRNA	exon	192210266	192210332	.	+	.	gene_id "lnc-bbug00734"	transcript_id "lnc-bbut00001731"	exon_number "2"
1	lncRNA	exon	192244601	192245604	.	+	.	gene_id "lnc-bbug00734"	transcript_id "lnc-bbut00001731"	exon_number "3"
1	lncRNA	exon	192551869	192552508	.	+	.	gene_id "lnc-bbug00735"	transcript_id "lnc-bbut00001732"	exon_number "1"
1	lncRNA	exon	192565135	192565842	.	+	.	gene_id "lnc-bbug00735"	transcript_id "lnc-bbut00001732"	exon_number "2"
1	lncRNA	exon	192889117	192890127	.	+	.	gene_id "lnc-bbug00737"	transcript_id "lnc-bbut00001734"	exon_number "1"
1	lncRNA	exon	192890427	192890489	.	+	.	gene_id "lnc-bbug00737"	transcript_id "lnc-bbut00001734"	exon_number "2"
1	lncRNA	exon	192890659	192892653	.	+	.	gene_id "lnc-bbug00737"	transcript_id "lnc-bbut00001734"	exon_number "3"
1	lncRNA	exon	192899417	192900427	.	+	.	gene_id "lnc-bbug00738"	transcript_id "lnc-bbut00001738"	exon_number "1"
1	lncRNA	exon	192900726	192900788	.	+	.	gene_id "lnc-bbug00738"	transcript_id "lnc-bbut00001738"	exon_number "2"
1	lncRNA	exon	192900958	192902961	.	+	.	gene_id "lnc-bbug00738"	transcript_id "lnc-bbut00001738"	exon_number "3"
1	lncRNA	exon	192899417	192900427	.	+	.	gene_id "lnc-bbug00738"	transcript_id "lnc-bbut00001740"	exon_number "1"
1	lncRNA	exon	192900726	192900788	.	+	.	gene_id "lnc-bbug00738"	transcript_id "lnc-bbut00001740"	exon_number "2"
1	lncRNA	exon	192903551	192904166	.	+	.	gene_id "lnc-bbug00738"	transcript_id "lnc-bbut00001740"	exon_number "3"
1	lncRNA	exon	192909711	192910721	.	+	.	gene_id "lnc-bbug00740"	transcript_id "lnc-bbut00001743"	exon_number "1"
1	lncRNA	exon	192911021	192911083	.	+	.	gene_id "lnc-bbug00740"	transcript_id "lnc-bbut00001743"	exon_number "2"
1	lncRNA	exon	192911253	192911462	.	+	.	gene_id "lnc-bbug00740"	transcript_id "lnc-bbut00001743"	exon_number "3"
1	lncRNA	exon	192909711	192910721	.	+	.	gene_id "lnc-bbug00740"	transcript_id "lnc-bbut00001742"	exon_number "1"
1	lncRNA	exon	192911253	192911462	.	+	.	gene_id "lnc-bbug00740"	transcript_id "lnc-bbut00001742"	exon_number "2"
1	lncRNA	exon	192940689	192941006	.	+	.	gene_id "lnc-bbug00742"	transcript_id "lnc-bbut00001746"	exon_number "1"
1	lncRNA	exon	192941370	192941478	.	+	.	gene_id "lnc-bbug00742"	transcript_id "lnc-bbut00001746"	exon_number "2"
1	lncRNA	exon	192962158	192962867	.	+	.	gene_id "lnc-bbug00742"	transcript_id "lnc-bbut00001746"	exon_number "3"
1	lncRNA	exon	193511029	193511151	.	+	.	gene_id "lnc-bbug00748"	transcript_id "lnc-bbut00001755"	exon_number "1"
1	lncRNA	exon	193511191	193511429	.	+	.	gene_id "lnc-bbug00748"	transcript_id "lnc-bbut00001755"	exon_number "2"
1	lncRNA	exon	194498775	194499011	.	+	.	gene_id "lnc-bbug00753"	transcript_id "lnc-bbut00001777"	exon_number "1"
1	lncRNA	exon	194499863	194500105	.	+	.	gene_id "lnc-bbug00753"	transcript_id "lnc-bbut00001777"	exon_number "2"

1	lncRNA	exon	194500762	194500893	.	+	.	gene_id "lnc-bbug00753"	transcript_id "lnc-bbut00001777"	exon_number "3"
1	lncRNA	exon	194501562	194501984	.	+	.	gene_id "lnc-bbug00753"	transcript_id "lnc-bbut00001777"	exon_number "4"
1	lncRNA	exon	194498775	194500105	.	+	.	gene_id "lnc-bbug00753"	transcript_id "lnc-bbut00001776"	exon_number "1"
1	lncRNA	exon	194500762	194500893	.	+	.	gene_id "lnc-bbug00753"	transcript_id "lnc-bbut00001776"	exon_number "2"
1	lncRNA	exon	194501562	194501984	.	+	.	gene_id "lnc-bbug00753"	transcript_id "lnc-bbut00001776"	exon_number "3"
1	lncRNA	exon	195303470	195303750	.	+	.	gene_id "lnc-bbug00755"	transcript_id "lnc-bbut00001787"	exon_number "1"
1	lncRNA	exon	195304205	195304289	.	+	.	gene_id "lnc-bbug00755"	transcript_id "lnc-bbut00001787"	exon_number "2"
1	lncRNA	exon	196886485	196886597	.	+	.	gene_id "lnc-bbug00760"	transcript_id "lnc-bbut00001797"	exon_number "1"
1	lncRNA	exon	196887490	196887701	.	+	.	gene_id "lnc-bbug00760"	transcript_id "lnc-bbut00001797"	exon_number "2"
1	lncRNA	exon	196888242	196888769	.	+	.	gene_id "lnc-bbug00760"	transcript_id "lnc-bbut00001797"	exon_number "3"
1	lncRNA	exon	197097215	197097948	.	+	.	gene_id "lnc-bbug00762"	transcript_id "lnc-bbut00001799"	exon_number "1"
1	lncRNA	exon	197101282	197101449	.	+	.	gene_id "lnc-bbug00762"	transcript_id "lnc-bbut00001799"	exon_number "2"
1	lncRNA	exon	197102302	197106219	.	+	.	gene_id "lnc-bbug00762"	transcript_id "lnc-bbut00001799"	exon_number "3"
1	lncRNA	exon	197107375	197107427	.	+	.	gene_id "lnc-bbug00762"	transcript_id "lnc-bbut00001799"	exon_number "4"
1	lncRNA	exon	197470125	197470173	.	+	.	gene_id "lnc-bbug00764"	transcript_id "lnc-bbut00001804"	exon_number "1"
1	lncRNA	exon	197470516	197470697	.	+	.	gene_id "lnc-bbug00764"	transcript_id "lnc-bbut00001804"	exon_number "2"
1	lncRNA	exon	197471112	197471206	.	+	.	gene_id "lnc-bbug00764"	transcript_id "lnc-bbut00001804"	exon_number "3"
1	lncRNA	exon	197480411	197480585	.	+	.	gene_id "lnc-bbug00764"	transcript_id "lnc-bbut00001804"	exon_number "4"
1	lncRNA	exon	197470193	197470251	.	+	.	gene_id "lnc-bbug00764"	transcript_id "lnc-bbut00001805"	exon_number "1"
1	lncRNA	exon	197470516	197470697	.	+	.	gene_id "lnc-bbug00764"	transcript_id "lnc-bbut00001805"	exon_number "2"
1	lncRNA	exon	197471112	197471206	.	+	.	gene_id "lnc-bbug00764"	transcript_id "lnc-bbut00001805"	exon_number "3"
1	lncRNA	exon	197480411	197480585	.	+	.	gene_id "lnc-bbug00764"	transcript_id "lnc-bbut00001805"	exon_number "4"
1	lncRNA	exon	197470261	197470697	.	+	.	gene_id "lnc-bbug00764"	transcript_id "lnc-bbut00001806"	exon_number "1"
1	lncRNA	exon	197471112	197471206	.	+	.	gene_id "lnc-bbug00764"	transcript_id "lnc-bbut00001806"	exon_number "2"
1	lncRNA	exon	197480411	197480585	.	+	.	gene_id "lnc-bbug00764"	transcript_id "lnc-bbut00001806"	exon_number "3"
1	lncRNA	exon	197529872	197529983	.	+	.	gene_id "lnc-bbug00765"	transcript_id "lnc-bbut00001807"	exon_number "1"
1	lncRNA	exon	197530305	197530446	.	+	.	gene_id "lnc-bbug00765"	transcript_id "lnc-bbut00001807"	exon_number "2"
1	lncRNA	exon	197530769	197530900	.	+	.	gene_id "lnc-bbug00765"	transcript_id "lnc-bbut00001807"	exon_number "3"
1	lncRNA	exon	198104058	198104208	.	+	.	gene_id "lnc-bbug00768"	transcript_id "lnc-bbut00001824"	exon_number "1"
1	lncRNA	exon	198104618	198104848	.	+	.	gene_id "lnc-bbug00768"	transcript_id "lnc-bbut00001824"	exon_number "2"
1	lncRNA	exon	198108255	198108476	.	+	.	gene_id "lnc-bbug00768"	transcript_id "lnc-bbut00001824"	exon_number "3"
1	lncRNA	exon	198110542	198110654	.	+	.	gene_id "lnc-bbug00768"	transcript_id "lnc-bbut00001824"	exon_number "4"
1	lncRNA	exon	198111172	198111816	.	+	.	gene_id "lnc-bbug00768"	transcript_id "lnc-bbut00001824"	exon_number "5"
1	lncRNA	exon	200075968	200076045	.	+	.	gene_id "lnc-bbug00770"	transcript_id "lnc-bbut00001829"	exon_number "1"
1	lncRNA	exon	200079591	200079851	.	+	.	gene_id "lnc-bbug00770"	transcript_id "lnc-bbut00001829"	exon_number "2"
1	lncRNA	exon	200120495	200120594	.	+	.	gene_id "lnc-bbug00770"	transcript_id "lnc-bbut00001829"	exon_number "3"
1	lncRNA	exon	200120950	200121417	.	+	.	gene_id "lnc-bbug00770"	transcript_id "lnc-bbut00001829"	exon_number "4"
1	lncRNA	exon	200075974	200076045	.	+	.	gene_id "lnc-bbug00770"	transcript_id "lnc-bbut00001830"	exon_number "1"
1	lncRNA	exon	200120495	200120594	.	+	.	gene_id "lnc-bbug00770"	transcript_id "lnc-bbut00001830"	exon_number "2"
1	lncRNA	exon	200120950	200121417	.	+	.	gene_id "lnc-bbug00770"	transcript_id "lnc-bbut00001830"	exon_number "3"
1	lncRNA	exon	200076089	200079851	.	+	.	gene_id "lnc-bbug00770"	transcript_id "lnc-bbut00001831"	exon_number "1"
1	lncRNA	exon	200081768	200082759	.	+	.	gene_id "lnc-bbug00770"	transcript_id "lnc-bbut00001831"	exon_number "2"
1	lncRNA	exon	200076093	200078686	.	+	.	gene_id "lnc-bbug00770"	transcript_id "lnc-bbut00001832"	exon_number "1"
1	lncRNA	exon	200079109	200079851	.	+	.	gene_id "lnc-bbug00770"	transcript_id "lnc-bbut00001832"	exon_number "2"
1	lncRNA	exon	200120495	200120594	.	+	.	gene_id "lnc-bbug00770"	transcript_id "lnc-bbut00001832"	exon_number "3"
1	lncRNA	exon	200120950	200121417	.	+	.	gene_id "lnc-bbug00770"	transcript_id "lnc-bbut00001832"	exon_number "4"
1	lncRNA	exon	200076104	200079851	.	+	.	gene_id "lnc-bbug00770"	transcript_id "lnc-bbut00001833"	exon_number "1"
1	lncRNA	exon	200120495	200120594	.	+	.	gene_id "lnc-bbug00770"	transcript_id "lnc-bbut00001833"	exon_number "2"
1	lncRNA	exon	200120950	200121417	.	+	.	gene_id "lnc-bbug00770"	transcript_id "lnc-bbut00001833"	exon_number "3"
1	lncRNA	exon	200083317	200083543	.	+	.	gene_id "lnc-bbug00770"	transcript_id "lnc-bbut00001835"	exon_number "1"
1	lncRNA	exon	200120495	200120594	.	+	.	gene_id "lnc-bbug00770"	transcript_id "lnc-bbut00001835"	exon_number "2"
1	lncRNA	exon	200120950	200121417	.	+	.	gene_id "lnc-bbug00770"	transcript_id "lnc-bbut00001835"	exon_number "3"

1	lncRNA	exon	200419731	200419780	.	+	.	gene_id "lnc-bbug00771"	transcript_id "lnc-bbut00001836"	exon_number "1"
1	lncRNA	exon	200434719	200434753	.	+	.	gene_id "lnc-bbug00771"	transcript_id "lnc-bbut00001836"	exon_number "2"
1	lncRNA	exon	200435237	200436735	.	+	.	gene_id "lnc-bbug00771"	transcript_id "lnc-bbut00001836"	exon_number "3"
1	lncRNA	exon	200679785	200679986	.	+	.	gene_id "lnc-bbug00772"	transcript_id "lnc-bbut00001837"	exon_number "1"
1	lncRNA	exon	200698119	200698362	.	+	.	gene_id "lnc-bbug00772"	transcript_id "lnc-bbut00001837"	exon_number "2"
1	lncRNA	exon	200698932	200699021	.	+	.	gene_id "lnc-bbug00772"	transcript_id "lnc-bbut00001837"	exon_number "3"
1	lncRNA	exon	200702671	200703717	.	+	.	gene_id "lnc-bbug00772"	transcript_id "lnc-bbut00001837"	exon_number "4"
1	lncRNA	exon	200964972	200966666	.	+	.	gene_id "lnc-bbug00774"	transcript_id "lnc-bbut00001839"	exon_number "1"
1	lncRNA	exon	3432922	3433176	.	-	.	gene_id "lnc-bbug00779"	transcript_id "lnc-bbut00001845"	exon_number "1"
1	lncRNA	exon	3433436	3433535	.	-	.	gene_id "lnc-bbug00779"	transcript_id "lnc-bbut00001845"	exon_number "2"
1	lncRNA	exon	3435154	3435567	.	-	.	gene_id "lnc-bbug00779"	transcript_id "lnc-bbut00001845"	exon_number "3"
1	lncRNA	exon	5707123	5707383	.	-	.	gene_id "lnc-bbug00786"	transcript_id "lnc-bbut00001867"	exon_number "1"
1	lncRNA	exon	5709465	5709724	.	-	.	gene_id "lnc-bbug00786"	transcript_id "lnc-bbut00001867"	exon_number "2"
1	lncRNA	exon	5717586	5717881	.	-	.	gene_id "lnc-bbug00786"	transcript_id "lnc-bbut00001867"	exon_number "3"
1	lncRNA	exon	6566415	6566758	.	-	.	gene_id "lnc-bbug00789"	transcript_id "lnc-bbut00001870"	exon_number "1"
1	lncRNA	exon	6567225	6567888	.	-	.	gene_id "lnc-bbug00789"	transcript_id "lnc-bbut00001870"	exon_number "2"
1	lncRNA	exon	8217552	8219273	.	-	.	gene_id "lnc-bbug00799"	transcript_id "lnc-bbut00001900"	exon_number "1"
1	lncRNA	exon	8219307	8219442	.	-	.	gene_id "lnc-bbug00799"	transcript_id "lnc-bbut00001900"	exon_number "2"
1	lncRNA	exon	8224760	8224884	.	-	.	gene_id "lnc-bbug00799"	transcript_id "lnc-bbut00001900"	exon_number "3"
1	lncRNA	exon	8226371	8226508	.	-	.	gene_id "lnc-bbug00799"	transcript_id "lnc-bbut00001900"	exon_number "4"
1	lncRNA	exon	8230593	8230749	.	-	.	gene_id "lnc-bbug00799"	transcript_id "lnc-bbut00001900"	exon_number "5"
1	lncRNA	exon	8217552	8219273	.	-	.	gene_id "lnc-bbug00799"	transcript_id "lnc-bbut00001901"	exon_number "1"
1	lncRNA	exon	8219307	8219442	.	-	.	gene_id "lnc-bbug00799"	transcript_id "lnc-bbut00001901"	exon_number "2"
1	lncRNA	exon	8224760	8224884	.	-	.	gene_id "lnc-bbug00799"	transcript_id "lnc-bbut00001901"	exon_number "3"
1	lncRNA	exon	8226371	8226508	.	-	.	gene_id "lnc-bbug00799"	transcript_id "lnc-bbut00001901"	exon_number "4"
1	lncRNA	exon	8230852	8231004	.	-	.	gene_id "lnc-bbug00799"	transcript_id "lnc-bbut00001901"	exon_number "5"
1	lncRNA	exon	8301756	8302588	.	-	.	gene_id "lnc-bbug00800"	transcript_id "lnc-bbut00001902"	exon_number "1"
1	lncRNA	exon	8304276	8308907	.	-	.	gene_id "lnc-bbug00800"	transcript_id "lnc-bbut00001902"	exon_number "2"
1	lncRNA	exon	8537454	8537831	.	-	.	gene_id "lnc-bbug00805"	transcript_id "lnc-bbut00001912"	exon_number "1"
1	lncRNA	exon	8538507	8539095	.	-	.	gene_id "lnc-bbug00805"	transcript_id "lnc-bbut00001912"	exon_number "2"
1	lncRNA	exon	9441829	9442204	.	-	.	gene_id "lnc-bbug00810"	transcript_id "lnc-bbut00001923"	exon_number "1"
1	lncRNA	exon	10347562	10348890	.	-	.	gene_id "lnc-bbug00811"	transcript_id "lnc-bbut00001924"	exon_number "1"
1	lncRNA	exon	12557855	12558331	.	-	.	gene_id "lnc-bbug00832"	transcript_id "lnc-bbut00001980"	exon_number "1"
1	lncRNA	exon	12558978	12559098	.	-	.	gene_id "lnc-bbug00832"	transcript_id "lnc-bbut00001980"	exon_number "2"
1	lncRNA	exon	12559893	12560205	.	-	.	gene_id "lnc-bbug00832"	transcript_id "lnc-bbut00001980"	exon_number "3"
1	lncRNA	exon	12558522	12559098	.	-	.	gene_id "lnc-bbug00832"	transcript_id "lnc-bbut00001981"	exon_number "1"
1	lncRNA	exon	12559893	12560205	.	-	.	gene_id "lnc-bbug00832"	transcript_id "lnc-bbut00001981"	exon_number "2"
1	lncRNA	exon	13232512	13233156	.	-	.	gene_id "lnc-bbug00834"	transcript_id "lnc-bbut00001983"	exon_number "1"
1	lncRNA	exon	13233518	13233619	.	-	.	gene_id "lnc-bbug00834"	transcript_id "lnc-bbut00001983"	exon_number "2"
1	lncRNA	exon	13233912	13234554	.	-	.	gene_id "lnc-bbug00834"	transcript_id "lnc-bbut00001983"	exon_number "3"
1	lncRNA	exon	13234767	13235203	.	-	.	gene_id "lnc-bbug00834"	transcript_id "lnc-bbut00001983"	exon_number "4"
1	lncRNA	exon	13235426	13235592	.	-	.	gene_id "lnc-bbug00834"	transcript_id "lnc-bbut00001983"	exon_number "5"
1	lncRNA	exon	13235631	13235709	.	-	.	gene_id "lnc-bbug00834"	transcript_id "lnc-bbut00001983"	exon_number "6"
1	lncRNA	exon	13239095	13239184	.	-	.	gene_id "lnc-bbug00834"	transcript_id "lnc-bbut00001983"	exon_number "7"
1	lncRNA	exon	15141608	15141691	.	-	.	gene_id "lnc-bbug00837"	transcript_id "lnc-bbut00001986"	exon_number "1"
1	lncRNA	exon	15151169	15151236	.	-	.	gene_id "lnc-bbug00837"	transcript_id "lnc-bbut00001986"	exon_number "2"
1	lncRNA	exon	15158408	15158620	.	-	.	gene_id "lnc-bbug00837"	transcript_id "lnc-bbut00001986"	exon_number "3"
1	lncRNA	exon	16292627	16294748	.	-	.	gene_id "lnc-bbug00838"	transcript_id "lnc-bbut00001987"	exon_number "1"
1	lncRNA	exon	16307241	16307616	.	-	.	gene_id "lnc-bbug00838"	transcript_id "lnc-bbut00001987"	exon_number "2"
1	lncRNA	exon	16390114	16390864	.	-	.	gene_id "lnc-bbug00840"	transcript_id "lnc-bbut00001989"	exon_number "1"
1	lncRNA	exon	16390978	16391991	.	-	.	gene_id "lnc-bbug00840"	transcript_id "lnc-bbut00001989"	exon_number "2"
1	lncRNA	exon	17206140	17206323	.	-	.	gene_id "lnc-bbug00842"	transcript_id "lnc-bbut00002006"	exon_number "1"

1	lncRNA	exon	17206707	17206782	.	-	.	gene_id "lnc-bbug00842"	transcript_id "lnc-bbut00002006"	exon_number "2"
1	lncRNA	exon	17207127	17211113	.	-	.	gene_id "lnc-bbug00842"	transcript_id "lnc-bbut00002006"	exon_number "3"
1	lncRNA	exon	17206140	17206782	.	-	.	gene_id "lnc-bbug00842"	transcript_id "lnc-bbut00002005"	exon_number "1"
1	lncRNA	exon	17207127	17211113	.	-	.	gene_id "lnc-bbug00842"	transcript_id "lnc-bbut00002005"	exon_number "2"
1	lncRNA	exon	18685298	18687079	.	-	.	gene_id "lnc-bbug00845"	transcript_id "lnc-bbut00002016"	exon_number "1"
1	lncRNA	exon	18774735	18775268	.	-	.	gene_id "lnc-bbug00846"	transcript_id "lnc-bbut00002017"	exon_number "1"
1	lncRNA	exon	18775716	18775761	.	-	.	gene_id "lnc-bbug00846"	transcript_id "lnc-bbut00002017"	exon_number "2"
1	lncRNA	exon	18781613	18781676	.	-	.	gene_id "lnc-bbug00846"	transcript_id "lnc-bbut00002017"	exon_number "3"
1	lncRNA	exon	19093831	19094037	.	-	.	gene_id "lnc-bbug00848"	transcript_id "lnc-bbut00002026"	exon_number "1"
1	lncRNA	exon	19100760	19101050	.	-	.	gene_id "lnc-bbug00848"	transcript_id "lnc-bbut00002026"	exon_number "2"
1	lncRNA	exon	19107360	19107462	.	-	.	gene_id "lnc-bbug00848"	transcript_id "lnc-bbut00002026"	exon_number "3"
1	lncRNA	exon	19597665	19599573	.	-	.	gene_id "lnc-bbug00851"	transcript_id "lnc-bbut00002030"	exon_number "1"
1	lncRNA	exon	19600704	19600800	.	-	.	gene_id "lnc-bbug00851"	transcript_id "lnc-bbut00002030"	exon_number "2"
1	lncRNA	exon	19611596	19611836	.	-	.	gene_id "lnc-bbug00851"	transcript_id "lnc-bbut00002030"	exon_number "3"
1	lncRNA	exon	19612242	19612446	.	-	.	gene_id "lnc-bbug00851"	transcript_id "lnc-bbut00002030"	exon_number "4"
1	lncRNA	exon	19616287	19616485	.	-	.	gene_id "lnc-bbug00851"	transcript_id "lnc-bbut00002030"	exon_number "5"
1	lncRNA	exon	19597665	19599573	.	-	.	gene_id "lnc-bbug00851"	transcript_id "lnc-bbut00002031"	exon_number "1"
1	lncRNA	exon	19600704	19600800	.	-	.	gene_id "lnc-bbug00851"	transcript_id "lnc-bbut00002031"	exon_number "2"
1	lncRNA	exon	19611596	19611836	.	-	.	gene_id "lnc-bbug00851"	transcript_id "lnc-bbut00002031"	exon_number "3"
1	lncRNA	exon	19612242	19612446	.	-	.	gene_id "lnc-bbug00851"	transcript_id "lnc-bbut00002031"	exon_number "4"
1	lncRNA	exon	19619549	19619620	.	-	.	gene_id "lnc-bbug00851"	transcript_id "lnc-bbut00002031"	exon_number "5"
1	lncRNA	exon	19640002	19640975	.	-	.	gene_id "lnc-bbug00852"	transcript_id "lnc-bbut00002032"	exon_number "1"
1	lncRNA	exon	19656695	19656819	.	-	.	gene_id "lnc-bbug00852"	transcript_id "lnc-bbut00002032"	exon_number "2"
1	lncRNA	exon	19676536	19676695	.	-	.	gene_id "lnc-bbug00852"	transcript_id "lnc-bbut00002032"	exon_number "3"
1	lncRNA	exon	19678493	19679349	.	-	.	gene_id "lnc-bbug00852"	transcript_id "lnc-bbut00002032"	exon_number "4"
1	lncRNA	exon	19648526	19649191	.	-	.	gene_id "lnc-bbug00852"	transcript_id "lnc-bbut00002034"	exon_number "1"
1	lncRNA	exon	19656695	19656819	.	-	.	gene_id "lnc-bbug00852"	transcript_id "lnc-bbut00002034"	exon_number "2"
1	lncRNA	exon	19676536	19676695	.	-	.	gene_id "lnc-bbug00852"	transcript_id "lnc-bbut00002034"	exon_number "3"
1	lncRNA	exon	19678493	19679349	.	-	.	gene_id "lnc-bbug00852"	transcript_id "lnc-bbut00002034"	exon_number "4"
1	lncRNA	exon	19648526	19649172	.	-	.	gene_id "lnc-bbug00852"	transcript_id "lnc-bbut00002033"	exon_number "1"
1	lncRNA	exon	19656695	19656819	.	-	.	gene_id "lnc-bbug00852"	transcript_id "lnc-bbut00002033"	exon_number "2"
1	lncRNA	exon	19676536	19676695	.	-	.	gene_id "lnc-bbug00852"	transcript_id "lnc-bbut00002033"	exon_number "3"
1	lncRNA	exon	19678493	19679349	.	-	.	gene_id "lnc-bbug00852"	transcript_id "lnc-bbut00002033"	exon_number "4"
1	lncRNA	exon	19648526	19649191	.	-	.	gene_id "lnc-bbug00852"	transcript_id "lnc-bbut00002035"	exon_number "1"
1	lncRNA	exon	19656695	19656819	.	-	.	gene_id "lnc-bbug00852"	transcript_id "lnc-bbut00002035"	exon_number "2"
1	lncRNA	exon	19676536	19676695	.	-	.	gene_id "lnc-bbug00852"	transcript_id "lnc-bbut00002035"	exon_number "3"
1	lncRNA	exon	19678493	19678623	.	-	.	gene_id "lnc-bbug00852"	transcript_id "lnc-bbut00002035"	exon_number "4"
1	lncRNA	exon	19681343	19681383	.	-	.	gene_id "lnc-bbug00852"	transcript_id "lnc-bbut00002035"	exon_number "5"
1	lncRNA	exon	19682545	19683034	.	-	.	gene_id "lnc-bbug00853"	transcript_id "lnc-bbut00002036"	exon_number "1"
1	lncRNA	exon	19684078	19684148	.	-	.	gene_id "lnc-bbug00853"	transcript_id "lnc-bbut00002036"	exon_number "2"
1	lncRNA	exon	19684245	19684480	.	-	.	gene_id "lnc-bbug00853"	transcript_id "lnc-bbut00002036"	exon_number "3"
1	lncRNA	exon	19701583	19702389	.	-	.	gene_id "lnc-bbug00853"	transcript_id "lnc-bbut00002036"	exon_number "4"
1	lncRNA	exon	19801744	19805673	.	-	.	gene_id "lnc-bbug00854"	transcript_id "lnc-bbut00002037"	exon_number "1"
1	lncRNA	exon	19807139	19807271	.	-	.	gene_id "lnc-bbug00854"	transcript_id "lnc-bbut00002037"	exon_number "2"
1	lncRNA	exon	19824560	19824626	.	-	.	gene_id "lnc-bbug00854"	transcript_id "lnc-bbut00002037"	exon_number "3"
1	lncRNA	exon	19826460	19826780	.	-	.	gene_id "lnc-bbug00854"	transcript_id "lnc-bbut00002037"	exon_number "4"
1	lncRNA	exon	19839206	19844931	.	-	.	gene_id "lnc-bbug00854"	transcript_id "lnc-bbut00002037"	exon_number "5"
1	lncRNA	exon	19933465	19934836	.	-	.	gene_id "lnc-bbug00857"	transcript_id "lnc-bbut00002041"	exon_number "1"
1	lncRNA	exon	20308452	20309313	.	-	.	gene_id "lnc-bbug00859"	transcript_id "lnc-bbut00002043"	exon_number "1"
1	lncRNA	exon	20317264	20317429	.	-	.	gene_id "lnc-bbug00859"	transcript_id "lnc-bbut00002043"	exon_number "2"
1	lncRNA	exon	20368746	20368864	.	-	.	gene_id "lnc-bbug00859"	transcript_id "lnc-bbut00002043"	exon_number "3"
1	lncRNA	exon	20433701	20434183	.	-	.	gene_id "lnc-bbug00860"	transcript_id "lnc-bbut00002044"	exon_number "1"

1	lncRNA	exon	20434397	20434656	.	-	.	gene_id "lnc-bbug00860"	transcript_id "lnc-bbut00002044"	exon_number "2"
1	lncRNA	exon	20476783	20477918	.	-	.	gene_id "lnc-bbug00861"	transcript_id "lnc-bbut00002045"	exon_number "1"
1	lncRNA	exon	20479212	20479326	.	-	.	gene_id "lnc-bbug00861"	transcript_id "lnc-bbut00002045"	exon_number "2"
1	lncRNA	exon	20482096	20482205	.	-	.	gene_id "lnc-bbug00861"	transcript_id "lnc-bbut00002045"	exon_number "3"
1	lncRNA	exon	20483547	20483630	.	-	.	gene_id "lnc-bbug00861"	transcript_id "lnc-bbut00002045"	exon_number "4"
1	lncRNA	exon	20488202	20488413	.	-	.	gene_id "lnc-bbug00861"	transcript_id "lnc-bbut00002045"	exon_number "5"
1	lncRNA	exon	20489636	20489814	.	-	.	gene_id "lnc-bbug00861"	transcript_id "lnc-bbut00002045"	exon_number "6"
1	lncRNA	exon	20493685	20493971	.	-	.	gene_id "lnc-bbug00861"	transcript_id "lnc-bbut00002045"	exon_number "7"
1	lncRNA	exon	21053539	21054917	.	-	.	gene_id "lnc-bbug00863"	transcript_id "lnc-bbut00002047"	exon_number "1"
1	lncRNA	exon	21056528	21056795	.	-	.	gene_id "lnc-bbug00863"	transcript_id "lnc-bbut00002047"	exon_number "2"
1	lncRNA	exon	21058293	21059561	.	-	.	gene_id "lnc-bbug00863"	transcript_id "lnc-bbut00002047"	exon_number "3"
1	lncRNA	exon	21053539	21054139	.	-	.	gene_id "lnc-bbug00863"	transcript_id "lnc-bbut00002048"	exon_number "1"
1	lncRNA	exon	21054789	21054917	.	-	.	gene_id "lnc-bbug00863"	transcript_id "lnc-bbut00002048"	exon_number "2"
1	lncRNA	exon	21056528	21056795	.	-	.	gene_id "lnc-bbug00863"	transcript_id "lnc-bbut00002048"	exon_number "3"
1	lncRNA	exon	21058293	21059563	.	-	.	gene_id "lnc-bbug00863"	transcript_id "lnc-bbut00002048"	exon_number "4"
1	lncRNA	exon	21053539	21054917	.	-	.	gene_id "lnc-bbug00863"	transcript_id "lnc-bbut00002049"	exon_number "1"
1	lncRNA	exon	21056528	21056795	.	-	.	gene_id "lnc-bbug00863"	transcript_id "lnc-bbut00002049"	exon_number "2"
1	lncRNA	exon	21059607	21060147	.	-	.	gene_id "lnc-bbug00863"	transcript_id "lnc-bbut00002049"	exon_number "3"
1	lncRNA	exon	21358495	21358543	.	-	.	gene_id "lnc-bbug00865"	transcript_id "lnc-bbut00002051"	exon_number "1"
1	lncRNA	exon	21359969	21360166	.	-	.	gene_id "lnc-bbug00865"	transcript_id "lnc-bbut00002051"	exon_number "2"
1	lncRNA	exon	21373358	21373464	.	-	.	gene_id "lnc-bbug00865"	transcript_id "lnc-bbut00002051"	exon_number "3"
1	lncRNA	exon	21398106	21398161	.	-	.	gene_id "lnc-bbug00865"	transcript_id "lnc-bbut00002051"	exon_number "4"
1	lncRNA	exon	21407091	21407202	.	-	.	gene_id "lnc-bbug00865"	transcript_id "lnc-bbut00002051"	exon_number "5"
1	lncRNA	exon	21472262	21473071	.	-	.	gene_id "lnc-bbug00865"	transcript_id "lnc-bbut00002051"	exon_number "6"
1	lncRNA	exon	25638565	25639352	.	-	.	gene_id "lnc-bbug00870"	transcript_id "lnc-bbut00002066"	exon_number "1"
1	lncRNA	exon	25889866	25890176	.	-	.	gene_id "lnc-bbug00872"	transcript_id "lnc-bbut00002069"	exon_number "1"
1	lncRNA	exon	25890620	25891756	.	-	.	gene_id "lnc-bbug00872"	transcript_id "lnc-bbut00002069"	exon_number "2"
1	lncRNA	exon	26229978	26231285	.	-	.	gene_id "lnc-bbug00875"	transcript_id "lnc-bbut00002078"	exon_number "1"
1	lncRNA	exon	26247000	26247655	.	-	.	gene_id "lnc-bbug00875"	transcript_id "lnc-bbut00002078"	exon_number "2"
1	lncRNA	exon	28552782	28553059	.	-	.	gene_id "lnc-bbug00882"	transcript_id "lnc-bbut00002086"	exon_number "1"
1	lncRNA	exon	28564622	28564727	.	-	.	gene_id "lnc-bbug00882"	transcript_id "lnc-bbut00002086"	exon_number "2"
1	lncRNA	exon	28566079	28566233	.	-	.	gene_id "lnc-bbug00882"	transcript_id "lnc-bbut00002086"	exon_number "3"
1	lncRNA	exon	28569168	28569289	.	-	.	gene_id "lnc-bbug00882"	transcript_id "lnc-bbut00002086"	exon_number "4"
1	lncRNA	exon	28572221	28572333	.	-	.	gene_id "lnc-bbug00882"	transcript_id "lnc-bbut00002086"	exon_number "5"
1	lncRNA	exon	28577623	28579874	.	-	.	gene_id "lnc-bbug00882"	transcript_id "lnc-bbut00002086"	exon_number "6"
1	lncRNA	exon	28593816	28594978	.	-	.	gene_id "lnc-bbug00883"	transcript_id "lnc-bbut00002089"	exon_number "1"
1	lncRNA	exon	28597769	28597889	.	-	.	gene_id "lnc-bbug00883"	transcript_id "lnc-bbut00002089"	exon_number "2"
1	lncRNA	exon	28603901	28603976	.	-	.	gene_id "lnc-bbug00883"	transcript_id "lnc-bbut00002089"	exon_number "3"
1	lncRNA	exon	28604578	28604774	.	-	.	gene_id "lnc-bbug00883"	transcript_id "lnc-bbut00002089"	exon_number "4"
1	lncRNA	exon	28610961	28612398	.	-	.	gene_id "lnc-bbug00883"	transcript_id "lnc-bbut00002089"	exon_number "5"
1	lncRNA	exon	28615816	28616030	.	-	.	gene_id "lnc-bbug00883"	transcript_id "lnc-bbut00002089"	exon_number "6"
1	lncRNA	exon	28616750	28616873	.	-	.	gene_id "lnc-bbug00883"	transcript_id "lnc-bbut00002089"	exon_number "7"
1	lncRNA	exon	28617236	28617293	.	-	.	gene_id "lnc-bbug00883"	transcript_id "lnc-bbut00002089"	exon_number "8"
1	lncRNA	exon	28621506	28621624	.	-	.	gene_id "lnc-bbug00883"	transcript_id "lnc-bbut00002089"	exon_number "9"
1	lncRNA	exon	28622139	28622227	.	-	.	gene_id "lnc-bbug00883"	transcript_id "lnc-bbut00002089"	exon_number "10"
1	lncRNA	exon	28626547	28627449	.	-	.	gene_id "lnc-bbug00883"	transcript_id "lnc-bbut00002089"	exon_number "11"
1	lncRNA	exon	28593816	28594978	.	-	.	gene_id "lnc-bbug00883"	transcript_id "lnc-bbut00002088"	exon_number "1"
1	lncRNA	exon	28597769	28597889	.	-	.	gene_id "lnc-bbug00883"	transcript_id "lnc-bbut00002088"	exon_number "2"
1	lncRNA	exon	28603901	28603976	.	-	.	gene_id "lnc-bbug00883"	transcript_id "lnc-bbut00002088"	exon_number "3"
1	lncRNA	exon	28604578	28604774	.	-	.	gene_id "lnc-bbug00883"	transcript_id "lnc-bbut00002088"	exon_number "4"
1	lncRNA	exon	28610961	28612398	.	-	.	gene_id "lnc-bbug00883"	transcript_id "lnc-bbut00002088"	exon_number "5"
1	lncRNA	exon	28615816	28615967	.	-	.	gene_id "lnc-bbug00883"	transcript_id "lnc-bbut00002088"	exon_number "6"

1	lncRNA	exon	28616750	28616873	.	-	.	gene_id "lnc-bbug00883"	transcript_id "lnc-bbut00002088"	exon_number "7"
1	lncRNA	exon	28617236	28617293	.	-	.	gene_id "lnc-bbug00883"	transcript_id "lnc-bbut00002088"	exon_number "8"
1	lncRNA	exon	28621506	28621624	.	-	.	gene_id "lnc-bbug00883"	transcript_id "lnc-bbut00002088"	exon_number "9"
1	lncRNA	exon	28622139	28622227	.	-	.	gene_id "lnc-bbug00883"	transcript_id "lnc-bbut00002088"	exon_number "10"
1	lncRNA	exon	28626547	28627449	.	-	.	gene_id "lnc-bbug00883"	transcript_id "lnc-bbut00002088"	exon_number "11"
1	lncRNA	exon	28593816	28594978	.	-	.	gene_id "lnc-bbug00883"	transcript_id "lnc-bbut00002087"	exon_number "1"
1	lncRNA	exon	28603901	28603976	.	-	.	gene_id "lnc-bbug00883"	transcript_id "lnc-bbut00002087"	exon_number "2"
1	lncRNA	exon	28604578	28604774	.	-	.	gene_id "lnc-bbug00883"	transcript_id "lnc-bbut00002087"	exon_number "3"
1	lncRNA	exon	28610961	28612398	.	-	.	gene_id "lnc-bbug00883"	transcript_id "lnc-bbut00002087"	exon_number "4"
1	lncRNA	exon	28615816	28616030	.	-	.	gene_id "lnc-bbug00883"	transcript_id "lnc-bbut00002087"	exon_number "5"
1	lncRNA	exon	28616750	28616873	.	-	.	gene_id "lnc-bbug00883"	transcript_id "lnc-bbut00002087"	exon_number "6"
1	lncRNA	exon	28617236	28617293	.	-	.	gene_id "lnc-bbug00883"	transcript_id "lnc-bbut00002087"	exon_number "7"
1	lncRNA	exon	28621506	28621624	.	-	.	gene_id "lnc-bbug00883"	transcript_id "lnc-bbut00002087"	exon_number "8"
1	lncRNA	exon	28622139	28622227	.	-	.	gene_id "lnc-bbug00883"	transcript_id "lnc-bbut00002087"	exon_number "9"
1	lncRNA	exon	28626547	28627449	.	-	.	gene_id "lnc-bbug00883"	transcript_id "lnc-bbut00002087"	exon_number "10"
1	lncRNA	exon	28656009	28656886	.	-	.	gene_id "lnc-bbug00884"	transcript_id "lnc-bbut00002090"	exon_number "1"
1	lncRNA	exon	28657688	28657766	.	-	.	gene_id "lnc-bbug00884"	transcript_id "lnc-bbut00002090"	exon_number "2"
1	lncRNA	exon	28674897	28675039	.	-	.	gene_id "lnc-bbug00884"	transcript_id "lnc-bbut00002090"	exon_number "3"
1	lncRNA	exon	28675404	28675576	.	-	.	gene_id "lnc-bbug00884"	transcript_id "lnc-bbut00002090"	exon_number "4"
1	lncRNA	exon	28684333	28684572	.	-	.	gene_id "lnc-bbug00884"	transcript_id "lnc-bbut00002090"	exon_number "5"
1	lncRNA	exon	28689661	28689811	.	-	.	gene_id "lnc-bbug00884"	transcript_id "lnc-bbut00002090"	exon_number "6"
1	lncRNA	exon	28691489	28692455	.	-	.	gene_id "lnc-bbug00884"	transcript_id "lnc-bbut00002090"	exon_number "7"
1	lncRNA	exon	28668846	28669006	.	-	.	gene_id "lnc-bbug00884"	transcript_id "lnc-bbut00002091"	exon_number "1"
1	lncRNA	exon	28674897	28675039	.	-	.	gene_id "lnc-bbug00884"	transcript_id "lnc-bbut00002091"	exon_number "2"
1	lncRNA	exon	28675404	28675576	.	-	.	gene_id "lnc-bbug00884"	transcript_id "lnc-bbut00002091"	exon_number "3"
1	lncRNA	exon	28684333	28684572	.	-	.	gene_id "lnc-bbug00884"	transcript_id "lnc-bbut00002091"	exon_number "4"
1	lncRNA	exon	28689661	28689811	.	-	.	gene_id "lnc-bbug00884"	transcript_id "lnc-bbut00002091"	exon_number "5"
1	lncRNA	exon	28691489	28692455	.	-	.	gene_id "lnc-bbug00884"	transcript_id "lnc-bbut00002091"	exon_number "6"
1	lncRNA	exon	28693960	28694795	.	-	.	gene_id "lnc-bbug00885"	transcript_id "lnc-bbut00002092"	exon_number "1"
1	lncRNA	exon	28719002	28719134	.	-	.	gene_id "lnc-bbug00885"	transcript_id "lnc-bbut00002092"	exon_number "2"
1	lncRNA	exon	28764516	28764843	.	-	.	gene_id "lnc-bbug00885"	transcript_id "lnc-bbut00002092"	exon_number "3"
1	lncRNA	exon	30729089	30729997	.	-	.	gene_id "lnc-bbug00898"	transcript_id "lnc-bbut00002122"	exon_number "1"
1	lncRNA	exon	30736153	30736704	.	-	.	gene_id "lnc-bbug00898"	transcript_id "lnc-bbut00002122"	exon_number "2"
1	lncRNA	exon	30729089	30729997	.	-	.	gene_id "lnc-bbug00898"	transcript_id "lnc-bbut00002123"	exon_number "1"
1	lncRNA	exon	30737697	30737756	.	-	.	gene_id "lnc-bbug00898"	transcript_id "lnc-bbut00002123"	exon_number "2"
1	lncRNA	exon	30738489	30738988	.	-	.	gene_id "lnc-bbug00899"	transcript_id "lnc-bbut00002124"	exon_number "1"
1	lncRNA	exon	30739103	30739212	.	-	.	gene_id "lnc-bbug00899"	transcript_id "lnc-bbut00002124"	exon_number "2"
1	lncRNA	exon	30739413	30739521	.	-	.	gene_id "lnc-bbug00899"	transcript_id "lnc-bbut00002124"	exon_number "3"
1	lncRNA	exon	30880868	30882287	.	-	.	gene_id "lnc-bbug00900"	transcript_id "lnc-bbut00002126"	exon_number "1"
1	lncRNA	exon	30884173	30884424	.	-	.	gene_id "lnc-bbug00900"	transcript_id "lnc-bbut00002126"	exon_number "2"
1	lncRNA	exon	30893476	30893576	.	-	.	gene_id "lnc-bbug00900"	transcript_id "lnc-bbut00002126"	exon_number "3"
1	lncRNA	exon	30896448	30896568	.	-	.	gene_id "lnc-bbug00900"	transcript_id "lnc-bbut00002126"	exon_number "4"
1	lncRNA	exon	30898400	30898563	.	-	.	gene_id "lnc-bbug00900"	transcript_id "lnc-bbut00002126"	exon_number "5"
1	lncRNA	exon	30899732	30899798	.	-	.	gene_id "lnc-bbug00900"	transcript_id "lnc-bbut00002126"	exon_number "6"
1	lncRNA	exon	30904008	30905021	.	-	.	gene_id "lnc-bbug00900"	transcript_id "lnc-bbut00002126"	exon_number "7"
1	lncRNA	exon	30880868	30882287	.	-	.	gene_id "lnc-bbug00900"	transcript_id "lnc-bbut00002125"	exon_number "1"
1	lncRNA	exon	30884173	30884424	.	-	.	gene_id "lnc-bbug00900"	transcript_id "lnc-bbut00002125"	exon_number "2"
1	lncRNA	exon	30893476	30893576	.	-	.	gene_id "lnc-bbug00900"	transcript_id "lnc-bbut00002125"	exon_number "3"
1	lncRNA	exon	30898400	30898563	.	-	.	gene_id "lnc-bbug00900"	transcript_id "lnc-bbut00002125"	exon_number "4"
1	lncRNA	exon	30899732	30899798	.	-	.	gene_id "lnc-bbug00900"	transcript_id "lnc-bbut00002125"	exon_number "5"
1	lncRNA	exon	30904008	30905021	.	-	.	gene_id "lnc-bbug00900"	transcript_id "lnc-bbut00002125"	exon_number "6"
1	lncRNA	exon	30880868	30882287	.	-	.	gene_id "lnc-bbug00900"	transcript_id "lnc-bbut00002127"	exon_number "1"

1	lncRNA	exon	30884173	30884424	.	-	.	gene_id "lnc-bbug00900"	transcript_id "lnc-bbut00002127"	exon_number "2"
1	lncRNA	exon	30893476	30893576	.	-	.	gene_id "lnc-bbug00900"	transcript_id "lnc-bbut00002127"	exon_number "3"
1	lncRNA	exon	30896448	30896568	.	-	.	gene_id "lnc-bbug00900"	transcript_id "lnc-bbut00002127"	exon_number "4"
1	lncRNA	exon	30898400	30898563	.	-	.	gene_id "lnc-bbug00900"	transcript_id "lnc-bbut00002127"	exon_number "5"
1	lncRNA	exon	30899732	30899798	.	-	.	gene_id "lnc-bbug00900"	transcript_id "lnc-bbut00002127"	exon_number "6"
1	lncRNA	exon	30913263	30914879	.	-	.	gene_id "lnc-bbug00900"	transcript_id "lnc-bbut00002127"	exon_number "7"
1	lncRNA	exon	32209772	32213535	.	-	.	gene_id "lnc-bbug00907"	transcript_id "lnc-bbut00002141"	exon_number "1"
1	lncRNA	exon	36851509	36854857	.	-	.	gene_id "lnc-bbug00909"	transcript_id "lnc-bbut00002143"	exon_number "1"
1	lncRNA	exon	36881768	36881870	.	-	.	gene_id "lnc-bbug00909"	transcript_id "lnc-bbut00002143"	exon_number "2"
1	lncRNA	exon	36895324	36895505	.	-	.	gene_id "lnc-bbug00909"	transcript_id "lnc-bbut00002143"	exon_number "3"
1	lncRNA	exon	36898270	36898565	.	-	.	gene_id "lnc-bbug00909"	transcript_id "lnc-bbut00002143"	exon_number "4"
1	lncRNA	exon	36899674	36899785	.	-	.	gene_id "lnc-bbug00910"	transcript_id "lnc-bbut00002145"	exon_number "1"
1	lncRNA	exon	36908492	36908732	.	-	.	gene_id "lnc-bbug00910"	transcript_id "lnc-bbut00002145"	exon_number "2"
1	lncRNA	exon	36920531	36920698	.	-	.	gene_id "lnc-bbug00910"	transcript_id "lnc-bbut00002145"	exon_number "3"
1	lncRNA	exon	36921506	36921607	.	-	.	gene_id "lnc-bbug00910"	transcript_id "lnc-bbut00002145"	exon_number "4"
1	lncRNA	exon	36925820	36925918	.	-	.	gene_id "lnc-bbug00910"	transcript_id "lnc-bbut00002145"	exon_number "5"
1	lncRNA	exon	36926491	36926642	.	-	.	gene_id "lnc-bbug00910"	transcript_id "lnc-bbut00002145"	exon_number "6"
1	lncRNA	exon	36899674	36899785	.	-	.	gene_id "lnc-bbug00910"	transcript_id "lnc-bbut00002144"	exon_number "1"
1	lncRNA	exon	36908492	36908732	.	-	.	gene_id "lnc-bbug00910"	transcript_id "lnc-bbut00002144"	exon_number "2"
1	lncRNA	exon	36920531	36920694	.	-	.	gene_id "lnc-bbug00910"	transcript_id "lnc-bbut00002144"	exon_number "3"
1	lncRNA	exon	36921506	36921607	.	-	.	gene_id "lnc-bbug00910"	transcript_id "lnc-bbut00002144"	exon_number "4"
1	lncRNA	exon	36925820	36925918	.	-	.	gene_id "lnc-bbug00910"	transcript_id "lnc-bbut00002144"	exon_number "5"
1	lncRNA	exon	36926491	36926642	.	-	.	gene_id "lnc-bbug00910"	transcript_id "lnc-bbut00002144"	exon_number "6"
1	lncRNA	exon	37180723	37181110	.	-	.	gene_id "lnc-bbug00912"	transcript_id "lnc-bbut00002148"	exon_number "1"
1	lncRNA	exon	37181212	37181850	.	-	.	gene_id "lnc-bbug00912"	transcript_id "lnc-bbut00002148"	exon_number "2"
1	lncRNA	exon	37180723	37181110	.	-	.	gene_id "lnc-bbug00912"	transcript_id "lnc-bbut00002147"	exon_number "1"
1	lncRNA	exon	37181194	37181850	.	-	.	gene_id "lnc-bbug00912"	transcript_id "lnc-bbut00002147"	exon_number "2"
1	lncRNA	exon	38489587	38489675	.	-	.	gene_id "lnc-bbug00917"	transcript_id "lnc-bbut00002156"	exon_number "1"
1	lncRNA	exon	38497749	38498206	.	-	.	gene_id "lnc-bbug00917"	transcript_id "lnc-bbut00002156"	exon_number "2"
1	lncRNA	exon	38498395	38499643	.	-	.	gene_id "lnc-bbug00917"	transcript_id "lnc-bbut00002156"	exon_number "3"
1	lncRNA	exon	38501183	38501436	.	-	.	gene_id "lnc-bbug00917"	transcript_id "lnc-bbut00002156"	exon_number "4"
1	lncRNA	exon	38497404	38497549	.	-	.	gene_id "lnc-bbug00917"	transcript_id "lnc-bbut00002157"	exon_number "1"
1	lncRNA	exon	38498395	38499643	.	-	.	gene_id "lnc-bbug00917"	transcript_id "lnc-bbut00002157"	exon_number "2"
1	lncRNA	exon	38501183	38501428	.	-	.	gene_id "lnc-bbug00917"	transcript_id "lnc-bbut00002157"	exon_number "3"
1	lncRNA	exon	38497404	38497549	.	-	.	gene_id "lnc-bbug00917"	transcript_id "lnc-bbut00002158"	exon_number "1"
1	lncRNA	exon	38497749	38497890	.	-	.	gene_id "lnc-bbug00917"	transcript_id "lnc-bbut00002158"	exon_number "2"
1	lncRNA	exon	38498395	38499643	.	-	.	gene_id "lnc-bbug00917"	transcript_id "lnc-bbut00002158"	exon_number "3"
1	lncRNA	exon	38501183	38501431	.	-	.	gene_id "lnc-bbug00917"	transcript_id "lnc-bbut00002158"	exon_number "4"
1	lncRNA	exon	38497404	38497549	.	-	.	gene_id "lnc-bbug00917"	transcript_id "lnc-bbut00002159"	exon_number "1"
1	lncRNA	exon	38497749	38498115	.	-	.	gene_id "lnc-bbug00917"	transcript_id "lnc-bbut00002159"	exon_number "2"
1	lncRNA	exon	38498395	38499643	.	-	.	gene_id "lnc-bbug00917"	transcript_id "lnc-bbut00002159"	exon_number "3"
1	lncRNA	exon	38501183	38501435	.	-	.	gene_id "lnc-bbug00917"	transcript_id "lnc-bbut00002159"	exon_number "4"
1	lncRNA	exon	38497404	38497549	.	-	.	gene_id "lnc-bbug00917"	transcript_id "lnc-bbut00002160"	exon_number "1"
1	lncRNA	exon	38497749	38498206	.	-	.	gene_id "lnc-bbug00917"	transcript_id "lnc-bbut00002160"	exon_number "2"
1	lncRNA	exon	38498395	38499643	.	-	.	gene_id "lnc-bbug00917"	transcript_id "lnc-bbut00002160"	exon_number "3"
1	lncRNA	exon	38501183	38501436	.	-	.	gene_id "lnc-bbug00917"	transcript_id "lnc-bbut00002160"	exon_number "4"
1	lncRNA	exon	38553591	38553835	.	-	.	gene_id "lnc-bbug00918"	transcript_id "lnc-bbut00002161"	exon_number "1"
1	lncRNA	exon	38555323	38555466	.	-	.	gene_id "lnc-bbug00918"	transcript_id "lnc-bbut00002161"	exon_number "2"
1	lncRNA	exon	38593035	38593291	.	-	.	gene_id "lnc-bbug00919"	transcript_id "lnc-bbut00002162"	exon_number "1"
1	lncRNA	exon	38594782	38595107	.	-	.	gene_id "lnc-bbug00919"	transcript_id "lnc-bbut00002162"	exon_number "2"
1	lncRNA	exon	38602670	38603200	.	-	.	gene_id "lnc-bbug00920"	transcript_id "lnc-bbut00002163"	exon_number "1"
1	lncRNA	exon	38609871	38609928	.	-	.	gene_id "lnc-bbug00920"	transcript_id "lnc-bbut00002163"	exon_number "2"

1	lncRNA	exon	38623139	38623478	.	-	.	gene_id "lnc-bbug00921"	transcript_id "lnc-bbut00002164"	exon_number "1"
1	lncRNA	exon	38626888	38626997	.	-	.	gene_id "lnc-bbug00921"	transcript_id "lnc-bbut00002164"	exon_number "2"
1	lncRNA	exon	38631569	38631774	.	-	.	gene_id "lnc-bbug00922"	transcript_id "lnc-bbut00002165"	exon_number "1"
1	lncRNA	exon	38636166	38636337	.	-	.	gene_id "lnc-bbug00922"	transcript_id "lnc-bbut00002165"	exon_number "2"
1	lncRNA	exon	38677537	38677688	.	-	.	gene_id "lnc-bbug00925"	transcript_id "lnc-bbut00002168"	exon_number "1"
1	lncRNA	exon	38683777	38683914	.	-	.	gene_id "lnc-bbug00925"	transcript_id "lnc-bbut00002168"	exon_number "2"
1	lncRNA	exon	38687916	38688026	.	-	.	gene_id "lnc-bbug00925"	transcript_id "lnc-bbut00002168"	exon_number "3"
1	lncRNA	exon	38710436	38711339	.	-	.	gene_id "lnc-bbug00926"	transcript_id "lnc-bbut00002169"	exon_number "1"
1	lncRNA	exon	38715130	38715243	.	-	.	gene_id "lnc-bbug00926"	transcript_id "lnc-bbut00002169"	exon_number "2"
1	lncRNA	exon	38716660	38717032	.	-	.	gene_id "lnc-bbug00926"	transcript_id "lnc-bbut00002169"	exon_number "3"
1	lncRNA	exon	38746945	38747836	.	-	.	gene_id "lnc-bbug00928"	transcript_id "lnc-bbut00002177"	exon_number "1"
1	lncRNA	exon	38751575	38752592	.	-	.	gene_id "lnc-bbug00928"	transcript_id "lnc-bbut00002177"	exon_number "2"
1	lncRNA	exon	38758273	38758692	.	-	.	gene_id "lnc-bbug00929"	transcript_id "lnc-bbut00002178"	exon_number "1"
1	lncRNA	exon	38759869	38759910	.	-	.	gene_id "lnc-bbug00929"	transcript_id "lnc-bbut00002178"	exon_number "2"
1	lncRNA	exon	38760246	38761277	.	-	.	gene_id "lnc-bbug00929"	transcript_id "lnc-bbut00002178"	exon_number "3"
1	lncRNA	exon	38953728	38957134	.	-	.	gene_id "lnc-bbug00930"	transcript_id "lnc-bbut00002179"	exon_number "1"
1	lncRNA	exon	39992730	39993488	.	-	.	gene_id "lnc-bbug00933"	transcript_id "lnc-bbut00002187"	exon_number "1"
1	lncRNA	exon	40000046	40000067	.	-	.	gene_id "lnc-bbug00933"	transcript_id "lnc-bbut00002187"	exon_number "2"
1	lncRNA	exon	42692539	42692873	.	-	.	gene_id "lnc-bbug00935"	transcript_id "lnc-bbut00002189"	exon_number "1"
1	lncRNA	exon	42696322	42696494	.	-	.	gene_id "lnc-bbug00935"	transcript_id "lnc-bbut00002189"	exon_number "2"
1	lncRNA	exon	42701196	42701308	.	-	.	gene_id "lnc-bbug00935"	transcript_id "lnc-bbut00002189"	exon_number "3"
1	lncRNA	exon	43345128	43345410	.	-	.	gene_id "lnc-bbug00936"	transcript_id "lnc-bbut00002190"	exon_number "1"
1	lncRNA	exon	43353162	43353318	.	-	.	gene_id "lnc-bbug00936"	transcript_id "lnc-bbut00002190"	exon_number "2"
1	lncRNA	exon	44827350	44827358	.	-	.	gene_id "lnc-bbug00947"	transcript_id "lnc-bbut00002220"	exon_number "1"
1	lncRNA	exon	44828956	44829971	.	-	.	gene_id "lnc-bbug00947"	transcript_id "lnc-bbut00002220"	exon_number "2"
1	lncRNA	exon	44872771	44872784	.	-	.	gene_id "lnc-bbug00947"	transcript_id "lnc-bbut00002220"	exon_number "3"
1	lncRNA	exon	45707287	45708756	.	-	.	gene_id "lnc-bbug00951"	transcript_id "lnc-bbut00002228"	exon_number "1"
1	lncRNA	exon	45722579	45722756	.	-	.	gene_id "lnc-bbug00951"	transcript_id "lnc-bbut00002228"	exon_number "2"
1	lncRNA	exon	45729004	45729102	.	-	.	gene_id "lnc-bbug00951"	transcript_id "lnc-bbut00002228"	exon_number "3"
1	lncRNA	exon	45729938	45730055	.	-	.	gene_id "lnc-bbug00951"	transcript_id "lnc-bbut00002228"	exon_number "4"
1	lncRNA	exon	45719895	45720082	.	-	.	gene_id "lnc-bbug00951"	transcript_id "lnc-bbut00002231"	exon_number "1"
1	lncRNA	exon	45722579	45722756	.	-	.	gene_id "lnc-bbug00951"	transcript_id "lnc-bbut00002231"	exon_number "2"
1	lncRNA	exon	45729004	45729102	.	-	.	gene_id "lnc-bbug00951"	transcript_id "lnc-bbut00002231"	exon_number "3"
1	lncRNA	exon	45735109	45735223	.	-	.	gene_id "lnc-bbug00951"	transcript_id "lnc-bbut00002231"	exon_number "4"
1	lncRNA	exon	45806620	45807095	.	-	.	gene_id "lnc-bbug00954"	transcript_id "lnc-bbut00002235"	exon_number "1"
1	lncRNA	exon	45808348	45808444	.	-	.	gene_id "lnc-bbug00954"	transcript_id "lnc-bbut00002235"	exon_number "2"
1	lncRNA	exon	45847124	45847180	.	-	.	gene_id "lnc-bbug00954"	transcript_id "lnc-bbut00002235"	exon_number "3"
1	lncRNA	exon	45889014	45889046	.	-	.	gene_id "lnc-bbug00954"	transcript_id "lnc-bbut00002235"	exon_number "4"
1	lncRNA	exon	45806620	45807095	.	-	.	gene_id "lnc-bbug00954"	transcript_id "lnc-bbut00002234"	exon_number "1"
1	lncRNA	exon	45808348	45808444	.	-	.	gene_id "lnc-bbug00954"	transcript_id "lnc-bbut00002234"	exon_number "2"
1	lncRNA	exon	45889014	45889046	.	-	.	gene_id "lnc-bbug00954"	transcript_id "lnc-bbut00002234"	exon_number "3"
1	lncRNA	exon	46558536	46558799	.	-	.	gene_id "lnc-bbug00957"	transcript_id "lnc-bbut00002250"	exon_number "1"
1	lncRNA	exon	46565445	46565547	.	-	.	gene_id "lnc-bbug00957"	transcript_id "lnc-bbut00002250"	exon_number "2"
1	lncRNA	exon	47037895	47038350	.	-	.	gene_id "lnc-bbug00964"	transcript_id "lnc-bbut00002265"	exon_number "1"
1	lncRNA	exon	47135150	47136148	.	-	.	gene_id "lnc-bbug00966"	transcript_id "lnc-bbut00002267"	exon_number "1"
1	lncRNA	exon	47136302	47136480	.	-	.	gene_id "lnc-bbug00966"	transcript_id "lnc-bbut00002267"	exon_number "2"
1	lncRNA	exon	47137951	47138322	.	-	.	gene_id "lnc-bbug00966"	transcript_id "lnc-bbut00002267"	exon_number "3"
1	lncRNA	exon	47206777	47207428	.	-	.	gene_id "lnc-bbug00968"	transcript_id "lnc-bbut00002269"	exon_number "1"
1	lncRNA	exon	47243421	47245898	.	-	.	gene_id "lnc-bbug00969"	transcript_id "lnc-bbut00002270"	exon_number "1"
1	lncRNA	exon	47246180	47246264	.	-	.	gene_id "lnc-bbug00969"	transcript_id "lnc-bbut00002270"	exon_number "2"
1	lncRNA	exon	47246377	47246521	.	-	.	gene_id "lnc-bbug00969"	transcript_id "lnc-bbut00002270"	exon_number "3"
1	lncRNA	exon	47464970	47465512	.	-	.	gene_id "lnc-bbug00970"	transcript_id "lnc-bbut00002271"	exon_number "1"

1	lncRNA	exon	48706224	48710831	.	-	.	gene_id "lnc-bbug00975"	transcript_id "lnc-bbut00002287"	exon_number "1"
1	lncRNA	exon	49468533	49469121	.	-	.	gene_id "lnc-bbug00977"	transcript_id "lnc-bbut00002289"	exon_number "1"
1	lncRNA	exon	49473278	49473494	.	-	.	gene_id "lnc-bbug00978"	transcript_id "lnc-bbut00002290"	exon_number "1"
1	lncRNA	exon	49473627	49473723	.	-	.	gene_id "lnc-bbug00978"	transcript_id "lnc-bbut00002290"	exon_number "2"
1	lncRNA	exon	49494668	49494931	.	-	.	gene_id "lnc-bbug00980"	transcript_id "lnc-bbut00002292"	exon_number "1"
1	lncRNA	exon	49521335	49521932	.	-	.	gene_id "lnc-bbug00981"	transcript_id "lnc-bbut00002293"	exon_number "1"
1	lncRNA	exon	49543196	49543790	.	-	.	gene_id "lnc-bbug00982"	transcript_id "lnc-bbut00002294"	exon_number "1"
1	lncRNA	exon	55545259	55547208	.	-	.	gene_id "lnc-bbug00997"	transcript_id "lnc-bbut00002323"	exon_number "1"
1	lncRNA	exon	55558780	55558888	.	-	.	gene_id "lnc-bbug00997"	transcript_id "lnc-bbut00002323"	exon_number "2"
1	lncRNA	exon	55839808	55841123	.	-	.	gene_id "lnc-bbug00998"	transcript_id "lnc-bbut00002324"	exon_number "1"
1	lncRNA	exon	55841778	55841991	.	-	.	gene_id "lnc-bbug00998"	transcript_id "lnc-bbut00002324"	exon_number "2"
1	lncRNA	exon	55843328	55843416	.	-	.	gene_id "lnc-bbug00998"	transcript_id "lnc-bbut00002324"	exon_number "3"
1	lncRNA	exon	56115004	56115610	.	-	.	gene_id "lnc-bbug00999"	transcript_id "lnc-bbut00002325"	exon_number "1"
1	lncRNA	exon	56115794	56115939	.	-	.	gene_id "lnc-bbug00999"	transcript_id "lnc-bbut00002325"	exon_number "2"
1	lncRNA	exon	56120532	56120713	.	-	.	gene_id "lnc-bbug00999"	transcript_id "lnc-bbut00002325"	exon_number "3"
1	lncRNA	exon	60804228	60805364	.	-	.	gene_id "lnc-bbug01001"	transcript_id "lnc-bbut00002327"	exon_number "1"
1	lncRNA	exon	60805422	60805716	.	-	.	gene_id "lnc-bbug01001"	transcript_id "lnc-bbut00002327"	exon_number "2"
1	lncRNA	exon	63799301	63799500	.	-	.	gene_id "lnc-bbug01003"	transcript_id "lnc-bbut00002329"	exon_number "1"
1	lncRNA	exon	63819704	63819837	.	-	.	gene_id "lnc-bbug01003"	transcript_id "lnc-bbut00002329"	exon_number "2"
1	lncRNA	exon	63821591	63821694	.	-	.	gene_id "lnc-bbug01003"	transcript_id "lnc-bbut00002329"	exon_number "3"
1	lncRNA	exon	63868464	63868552	.	-	.	gene_id "lnc-bbug01003"	transcript_id "lnc-bbut00002329"	exon_number "4"
1	lncRNA	exon	63886837	63887192	.	-	.	gene_id "lnc-bbug01003"	transcript_id "lnc-bbut00002329"	exon_number "5"
1	lncRNA	exon	63818987	63819837	.	-	.	gene_id "lnc-bbug01003"	transcript_id "lnc-bbut00002330"	exon_number "1"
1	lncRNA	exon	63821591	63821694	.	-	.	gene_id "lnc-bbug01003"	transcript_id "lnc-bbut00002330"	exon_number "2"
1	lncRNA	exon	63868464	63868552	.	-	.	gene_id "lnc-bbug01003"	transcript_id "lnc-bbut00002330"	exon_number "3"
1	lncRNA	exon	63886837	63887192	.	-	.	gene_id "lnc-bbug01003"	transcript_id "lnc-bbut00002330"	exon_number "4"
1	lncRNA	exon	64001043	64001922	.	-	.	gene_id "lnc-bbug01004"	transcript_id "lnc-bbut00002331"	exon_number "1"
1	lncRNA	exon	64010693	64010837	.	-	.	gene_id "lnc-bbug01004"	transcript_id "lnc-bbut00002331"	exon_number "2"
1	lncRNA	exon	64032166	64032252	.	-	.	gene_id "lnc-bbug01004"	transcript_id "lnc-bbut00002331"	exon_number "3"
1	lncRNA	exon	64089194	64089953	.	-	.	gene_id "lnc-bbug01004"	transcript_id "lnc-bbut00002331"	exon_number "4"
1	lncRNA	exon	64275235	64276030	.	-	.	gene_id "lnc-bbug01006"	transcript_id "lnc-bbut00002335"	exon_number "1"
1	lncRNA	exon	64276161	64276624	.	-	.	gene_id "lnc-bbug01006"	transcript_id "lnc-bbut00002335"	exon_number "2"
1	lncRNA	exon	64277174	64278770	.	-	.	gene_id "lnc-bbug01006"	transcript_id "lnc-bbut00002335"	exon_number "3"
1	lncRNA	exon	66785412	66786226	.	-	.	gene_id "lnc-bbug01009"	transcript_id "lnc-bbut00002345"	exon_number "1"
1	lncRNA	exon	66786600	66786726	.	-	.	gene_id "lnc-bbug01009"	transcript_id "lnc-bbut00002345"	exon_number "2"
1	lncRNA	exon	73976709	73982364	.	-	.	gene_id "lnc-bbug01015"	transcript_id "lnc-bbut00002357"	exon_number "1"
1	lncRNA	exon	75690142	75690546	.	-	.	gene_id "lnc-bbug01016"	transcript_id "lnc-bbut00002358"	exon_number "1"
1	lncRNA	exon	75690579	75690923	.	-	.	gene_id "lnc-bbug01016"	transcript_id "lnc-bbut00002358"	exon_number "2"
1	lncRNA	exon	76396345	76397176	.	-	.	gene_id "lnc-bbug01017"	transcript_id "lnc-bbut00002359"	exon_number "1"
1	lncRNA	exon	76397337	76397452	.	-	.	gene_id "lnc-bbug01017"	transcript_id "lnc-bbut00002359"	exon_number "2"
1	lncRNA	exon	76398504	76399407	.	-	.	gene_id "lnc-bbug01017"	transcript_id "lnc-bbut00002359"	exon_number "3"
1	lncRNA	exon	79045379	79046377	.	-	.	gene_id "lnc-bbug01018"	transcript_id "lnc-bbut00002360"	exon_number "1"
1	lncRNA	exon	79759120	79760791	.	-	.	gene_id "lnc-bbug01019"	transcript_id "lnc-bbut00002361"	exon_number "1"
1	lncRNA	exon	79761037	79762081	.	-	.	gene_id "lnc-bbug01019"	transcript_id "lnc-bbut00002361"	exon_number "2"
1	lncRNA	exon	81130777	81131159	.	-	.	gene_id "lnc-bbug01024"	transcript_id "lnc-bbut00002370"	exon_number "1"
1	lncRNA	exon	82375492	82376231	.	-	.	gene_id "lnc-bbug01025"	transcript_id "lnc-bbut00002371"	exon_number "1"
1	lncRNA	exon	82378246	82378315	.	-	.	gene_id "lnc-bbug01025"	transcript_id "lnc-bbut00002371"	exon_number "2"
1	lncRNA	exon	82383662	82384041	.	-	.	gene_id "lnc-bbug01025"	transcript_id "lnc-bbut00002371"	exon_number "3"
1	lncRNA	exon	87646531	87648393	.	-	.	gene_id "lnc-bbug01032"	transcript_id "lnc-bbut00002383"	exon_number "1"
1	lncRNA	exon	87658301	87658668	.	-	.	gene_id "lnc-bbug01032"	transcript_id "lnc-bbut00002383"	exon_number "2"
1	lncRNA	exon	88951001	88964855	.	-	.	gene_id "lnc-bbug01038"	transcript_id "lnc-bbut00002391"	exon_number "1"
1	lncRNA	exon	88965319	88967737	.	-	.	gene_id "lnc-bbug01038"	transcript_id "lnc-bbut00002391"	exon_number "2"

1	lncRNA	exon	91253806	91255046	.	-	.	gene_id "lnc-bbug01047"	transcript_id "lnc-bbut00002455"	exon_number "1"
1	lncRNA	exon	97489221	97490324	.	-	.	gene_id "lnc-bbug01051"	transcript_id "lnc-bbut00002468"	exon_number "1"
1	lncRNA	exon	97491774	97494674	.	-	.	gene_id "lnc-bbug01051"	transcript_id "lnc-bbut00002468"	exon_number "2"
1	lncRNA	exon	97495075	97495674	.	-	.	gene_id "lnc-bbug01051"	transcript_id "lnc-bbut00002468"	exon_number "3"
1	lncRNA	exon	97526261	97532624	.	-	.	gene_id "lnc-bbug01052"	transcript_id "lnc-bbut00002469"	exon_number "1"
1	lncRNA	exon	97532905	97538475	.	-	.	gene_id "lnc-bbug01052"	transcript_id "lnc-bbut00002469"	exon_number "2"
1	lncRNA	exon	98522223	98522404	.	-	.	gene_id "lnc-bbug01059"	transcript_id "lnc-bbut00002478"	exon_number "1"
1	lncRNA	exon	98555485	98555966	.	-	.	gene_id "lnc-bbug01059"	transcript_id "lnc-bbut00002478"	exon_number "2"
1	lncRNA	exon	98907408	98908404	.	-	.	gene_id "lnc-bbug01062"	transcript_id "lnc-bbut00002482"	exon_number "1"
1	lncRNA	exon	99065864	99066282	.	-	.	gene_id "lnc-bbug01064"	transcript_id "lnc-bbut00002484"	exon_number "1"
1	lncRNA	exon	99066362	99066419	.	-	.	gene_id "lnc-bbug01064"	transcript_id "lnc-bbut00002484"	exon_number "2"
1	lncRNA	exon	101098065	101099684	.	-	.	gene_id "lnc-bbug01066"	transcript_id "lnc-bbut00002486"	exon_number "1"
1	lncRNA	exon	101112440	101112510	.	-	.	gene_id "lnc-bbug01066"	transcript_id "lnc-bbut00002486"	exon_number "2"
1	lncRNA	exon	101114242	101114320	.	-	.	gene_id "lnc-bbug01066"	transcript_id "lnc-bbut00002486"	exon_number "3"
1	lncRNA	exon	101119840	101119952	.	-	.	gene_id "lnc-bbug01066"	transcript_id "lnc-bbut00002486"	exon_number "4"
1	lncRNA	exon	101644667	101645041	.	-	.	gene_id "lnc-bbug01067"	transcript_id "lnc-bbut00002487"	exon_number "1"
1	lncRNA	exon	101645428	101645613	.	-	.	gene_id "lnc-bbug01067"	transcript_id "lnc-bbut00002487"	exon_number "2"
1	lncRNA	exon	101647263	101648987	.	-	.	gene_id "lnc-bbug01067"	transcript_id "lnc-bbut00002487"	exon_number "3"
1	lncRNA	exon	102231696	102231880	.	-	.	gene_id "lnc-bbug01073"	transcript_id "lnc-bbut00002497"	exon_number "1"
1	lncRNA	exon	102233303	102233730	.	-	.	gene_id "lnc-bbug01073"	transcript_id "lnc-bbut00002497"	exon_number "2"
1	lncRNA	exon	102235546	102235696	.	-	.	gene_id "lnc-bbug01073"	transcript_id "lnc-bbut00002497"	exon_number "3"
1	lncRNA	exon	102235803	102237731	.	-	.	gene_id "lnc-bbug01073"	transcript_id "lnc-bbut00002497"	exon_number "4"
1	lncRNA	exon	102231696	102231880	.	-	.	gene_id "lnc-bbug01073"	transcript_id "lnc-bbut00002498"	exon_number "1"
1	lncRNA	exon	102233303	102233730	.	-	.	gene_id "lnc-bbug01073"	transcript_id "lnc-bbut00002498"	exon_number "2"
1	lncRNA	exon	102235546	102235696	.	-	.	gene_id "lnc-bbug01073"	transcript_id "lnc-bbut00002498"	exon_number "3"
1	lncRNA	exon	102235803	102235957	.	-	.	gene_id "lnc-bbug01073"	transcript_id "lnc-bbut00002498"	exon_number "4"
1	lncRNA	exon	102259005	102259173	.	-	.	gene_id "lnc-bbug01073"	transcript_id "lnc-bbut00002498"	exon_number "5"
1	lncRNA	exon	103157252	103160273	.	-	.	gene_id "lnc-bbug01080"	transcript_id "lnc-bbut00002523"	exon_number "1"
1	lncRNA	exon	103160970	103161036	.	-	.	gene_id "lnc-bbug01080"	transcript_id "lnc-bbut00002523"	exon_number "2"
1	lncRNA	exon	103161339	103161458	.	-	.	gene_id "lnc-bbug01080"	transcript_id "lnc-bbut00002523"	exon_number "3"
1	lncRNA	exon	103161879	103162015	.	-	.	gene_id "lnc-bbug01080"	transcript_id "lnc-bbut00002523"	exon_number "4"
1	lncRNA	exon	103162140	103162162	.	-	.	gene_id "lnc-bbug01080"	transcript_id "lnc-bbut00002523"	exon_number "5"
1	lncRNA	exon	103157252	103160273	.	-	.	gene_id "lnc-bbug01080"	transcript_id "lnc-bbut00002522"	exon_number "1"
1	lncRNA	exon	103160970	103161036	.	-	.	gene_id "lnc-bbug01080"	transcript_id "lnc-bbut00002522"	exon_number "2"
1	lncRNA	exon	103161339	103161458	.	-	.	gene_id "lnc-bbug01080"	transcript_id "lnc-bbut00002522"	exon_number "3"
1	lncRNA	exon	103161879	103162012	.	-	.	gene_id "lnc-bbug01080"	transcript_id "lnc-bbut00002522"	exon_number "4"
1	lncRNA	exon	103162140	103162162	.	-	.	gene_id "lnc-bbug01080"	transcript_id "lnc-bbut00002522"	exon_number "5"
1	lncRNA	exon	104777366	104777531	.	-	.	gene_id "lnc-bbug01084"	transcript_id "lnc-bbut00002553"	exon_number "1"
1	lncRNA	exon	104778458	104778624	.	-	.	gene_id "lnc-bbug01084"	transcript_id "lnc-bbut00002553"	exon_number "2"
1	lncRNA	exon	104786715	104786806	.	-	.	gene_id "lnc-bbug01084"	transcript_id "lnc-bbut00002553"	exon_number "3"
1	lncRNA	exon	104787204	104787355	.	-	.	gene_id "lnc-bbug01084"	transcript_id "lnc-bbut00002553"	exon_number "4"
1	lncRNA	exon	104777366	104777531	.	-	.	gene_id "lnc-bbug01084"	transcript_id "lnc-bbut00002554"	exon_number "1"
1	lncRNA	exon	104778458	104778624	.	-	.	gene_id "lnc-bbug01084"	transcript_id "lnc-bbut00002554"	exon_number "2"
1	lncRNA	exon	104786715	104786806	.	-	.	gene_id "lnc-bbug01084"	transcript_id "lnc-bbut00002554"	exon_number "3"
1	lncRNA	exon	104787362	104787801	.	-	.	gene_id "lnc-bbug01084"	transcript_id "lnc-bbut00002554"	exon_number "4"
1	lncRNA	exon	109123939	109125690	.	-	.	gene_id "lnc-bbug01090"	transcript_id "lnc-bbut00002570"	exon_number "1"
1	lncRNA	exon	109127223	109127367	.	-	.	gene_id "lnc-bbug01090"	transcript_id "lnc-bbut00002570"	exon_number "2"
1	lncRNA	exon	109128310	109129749	.	-	.	gene_id "lnc-bbug01091"	transcript_id "lnc-bbut00002571"	exon_number "1"
1	lncRNA	exon	109130179	109130392	.	-	.	gene_id "lnc-bbug01091"	transcript_id "lnc-bbut00002571"	exon_number "2"
1	lncRNA	exon	109167779	109167910	.	-	.	gene_id "lnc-bbug01093"	transcript_id "lnc-bbut00002577"	exon_number "1"
1	lncRNA	exon	109173531	109173619	.	-	.	gene_id "lnc-bbug01093"	transcript_id "lnc-bbut00002577"	exon_number "2"
1	lncRNA	exon	109175412	109175623	.	-	.	gene_id "lnc-bbug01093"	transcript_id "lnc-bbut00002577"	exon_number "3"

1	lncRNA	exon	109663576	109663611	.	-	.	gene_id "lnc-bbug01095"	transcript_id "lnc-bbut00002581"	exon_number "1"
1	lncRNA	exon	109674020	109674079	.	-	.	gene_id "lnc-bbug01095"	transcript_id "lnc-bbut00002581"	exon_number "2"
1	lncRNA	exon	109674306	109675837	.	-	.	gene_id "lnc-bbug01095"	transcript_id "lnc-bbut00002581"	exon_number "3"
1	lncRNA	exon	109979100	109984576	.	-	.	gene_id "lnc-bbug01099"	transcript_id "lnc-bbut00002585"	exon_number "1"
1	lncRNA	exon	109986682	109986880	.	-	.	gene_id "lnc-bbug01099"	transcript_id "lnc-bbut00002585"	exon_number "2"
1	lncRNA	exon	110010509	110011717	.	-	.	gene_id "lnc-bbug01099"	transcript_id "lnc-bbut00002585"	exon_number "3"
1	lncRNA	exon	110016090	110017485	.	-	.	gene_id "lnc-bbug01100"	transcript_id "lnc-bbut00002586"	exon_number "1"
1	lncRNA	exon	110024086	110024298	.	-	.	gene_id "lnc-bbug01100"	transcript_id "lnc-bbut00002586"	exon_number "2"
1	lncRNA	exon	110029921	110029970	.	-	.	gene_id "lnc-bbug01100"	transcript_id "lnc-bbut00002586"	exon_number "3"
1	lncRNA	exon	110030450	110030545	.	-	.	gene_id "lnc-bbug01100"	transcript_id "lnc-bbut00002586"	exon_number "4"
1	lncRNA	exon	110033364	110033436	.	-	.	gene_id "lnc-bbug01100"	transcript_id "lnc-bbut00002586"	exon_number "5"
1	lncRNA	exon	110040048	110040268	.	-	.	gene_id "lnc-bbug01100"	transcript_id "lnc-bbut00002586"	exon_number "6"
1	lncRNA	exon	110062554	110062663	.	-	.	gene_id "lnc-bbug01100"	transcript_id "lnc-bbut00002586"	exon_number "7"
1	lncRNA	exon	110828699	110831971	.	-	.	gene_id "lnc-bbug01104"	transcript_id "lnc-bbut00002595"	exon_number "1"
1	lncRNA	exon	112682130	112685976	.	-	.	gene_id "lnc-bbug01118"	transcript_id "lnc-bbut00002637"	exon_number "1"
1	lncRNA	exon	112692155	112694512	.	-	.	gene_id "lnc-bbug01118"	transcript_id "lnc-bbut00002637"	exon_number "2"
1	lncRNA	exon	113924572	113924663	.	-	.	gene_id "lnc-bbug01122"	transcript_id "lnc-bbut00002653"	exon_number "1"
1	lncRNA	exon	113924817	113925148	.	-	.	gene_id "lnc-bbug01122"	transcript_id "lnc-bbut00002653"	exon_number "2"
1	lncRNA	exon	114661548	114662099	.	-	.	gene_id "lnc-bbug01129"	transcript_id "lnc-bbut00002667"	exon_number "1"
1	lncRNA	exon	114664028	114664087	.	-	.	gene_id "lnc-bbug01129"	transcript_id "lnc-bbut00002667"	exon_number "2"
1	lncRNA	exon	114665446	114665751	.	-	.	gene_id "lnc-bbug01129"	transcript_id "lnc-bbut00002667"	exon_number "3"
1	lncRNA	exon	114976838	114976914	.	-	.	gene_id "lnc-bbug01134"	transcript_id "lnc-bbut00002677"	exon_number "1"
1	lncRNA	exon	114978123	114978267	.	-	.	gene_id "lnc-bbug01134"	transcript_id "lnc-bbut00002677"	exon_number "2"
1	lncRNA	exon	114979207	114979359	.	-	.	gene_id "lnc-bbug01134"	transcript_id "lnc-bbut00002677"	exon_number "3"
1	lncRNA	exon	114979576	114979620	.	-	.	gene_id "lnc-bbug01134"	transcript_id "lnc-bbut00002677"	exon_number "4"
1	lncRNA	exon	114980211	114980267	.	-	.	gene_id "lnc-bbug01134"	transcript_id "lnc-bbut00002677"	exon_number "5"
1	lncRNA	exon	114976838	114976914	.	-	.	gene_id "lnc-bbug01134"	transcript_id "lnc-bbut00002678"	exon_number "1"
1	lncRNA	exon	114978123	114978267	.	-	.	gene_id "lnc-bbug01134"	transcript_id "lnc-bbut00002678"	exon_number "2"
1	lncRNA	exon	114979207	114979359	.	-	.	gene_id "lnc-bbug01134"	transcript_id "lnc-bbut00002678"	exon_number "3"
1	lncRNA	exon	114979576	114979620	.	-	.	gene_id "lnc-bbug01134"	transcript_id "lnc-bbut00002678"	exon_number "4"
1	lncRNA	exon	114980256	114980341	.	-	.	gene_id "lnc-bbug01134"	transcript_id "lnc-bbut00002678"	exon_number "5"
1	lncRNA	exon	115954091	115954391	.	-	.	gene_id "lnc-bbug01147"	transcript_id "lnc-bbut00002729"	exon_number "1"
1	lncRNA	exon	116226278	116226512	.	-	.	gene_id "lnc-bbug01151"	transcript_id "lnc-bbut00002737"	exon_number "1"
1	lncRNA	exon	116228013	116228158	.	-	.	gene_id "lnc-bbug01151"	transcript_id "lnc-bbut00002737"	exon_number "2"
1	lncRNA	exon	116236342	116236626	.	-	.	gene_id "lnc-bbug01151"	transcript_id "lnc-bbut00002737"	exon_number "3"
1	lncRNA	exon	116241457	116241561	.	-	.	gene_id "lnc-bbug01151"	transcript_id "lnc-bbut00002737"	exon_number "4"
1	lncRNA	exon	116247794	116248112	.	-	.	gene_id "lnc-bbug01151"	transcript_id "lnc-bbut00002737"	exon_number "5"
1	lncRNA	exon	116857923	116858300	.	-	.	gene_id "lnc-bbug01155"	transcript_id "lnc-bbut00002770"	exon_number "1"
1	lncRNA	exon	116873623	116873713	.	-	.	gene_id "lnc-bbug01155"	transcript_id "lnc-bbut00002770"	exon_number "2"
1	lncRNA	exon	116876426	116877003	.	-	.	gene_id "lnc-bbug01155"	transcript_id "lnc-bbut00002770"	exon_number "3"
1	lncRNA	exon	116870167	116873437	.	-	.	gene_id "lnc-bbug01155"	transcript_id "lnc-bbut00002771"	exon_number "1"
1	lncRNA	exon	116873623	116873713	.	-	.	gene_id "lnc-bbug01155"	transcript_id "lnc-bbut00002771"	exon_number "2"
1	lncRNA	exon	116876426	116877003	.	-	.	gene_id "lnc-bbug01155"	transcript_id "lnc-bbut00002771"	exon_number "3"
1	lncRNA	exon	117127946	117128873	.	-	.	gene_id "lnc-bbug01157"	transcript_id "lnc-bbut00002773"	exon_number "1"
1	lncRNA	exon	117477440	117477600	.	-	.	gene_id "lnc-bbug01158"	transcript_id "lnc-bbut00002774"	exon_number "1"
1	lncRNA	exon	117479053	117479154	.	-	.	gene_id "lnc-bbug01158"	transcript_id "lnc-bbut00002774"	exon_number "2"
1	lncRNA	exon	117479649	117480015	.	-	.	gene_id "lnc-bbug01158"	transcript_id "lnc-bbut00002774"	exon_number "3"
1	lncRNA	exon	117993089	117994677	.	-	.	gene_id "lnc-bbug01160"	transcript_id "lnc-bbut00002776"	exon_number "1"
1	lncRNA	exon	118007588	118007700	.	-	.	gene_id "lnc-bbug01160"	transcript_id "lnc-bbut00002776"	exon_number "2"
1	lncRNA	exon	118025306	118025860	.	-	.	gene_id "lnc-bbug01160"	transcript_id "lnc-bbut00002776"	exon_number "3"
1	lncRNA	exon	118178877	118182493	.	-	.	gene_id "lnc-bbug01161"	transcript_id "lnc-bbut00002777"	exon_number "1"
1	lncRNA	exon	118183253	118183345	.	-	.	gene_id "lnc-bbug01161"	transcript_id "lnc-bbut00002777"	exon_number "2"

1	lncRNA	exon	118185355	118185955	.	-	.	gene_id "lnc-bbug01161"	transcript_id "lnc-bbut00002777"	exon_number "3"
1	lncRNA	exon	118696772	118697295	.	-	.	gene_id "lnc-bbug01164"	transcript_id "lnc-bbut00002783"	exon_number "1"
1	lncRNA	exon	118708368	118708463	.	-	.	gene_id "lnc-bbug01164"	transcript_id "lnc-bbut00002783"	exon_number "2"
1	lncRNA	exon	118714091	118714201	.	-	.	gene_id "lnc-bbug01164"	transcript_id "lnc-bbut00002783"	exon_number "3"
1	lncRNA	exon	118929538	118931073	.	-	.	gene_id "lnc-bbug01165"	transcript_id "lnc-bbut00002784"	exon_number "1"
1	lncRNA	exon	118931630	118931831	.	-	.	gene_id "lnc-bbug01165"	transcript_id "lnc-bbut00002784"	exon_number "2"
1	lncRNA	exon	118936866	118937090	.	-	.	gene_id "lnc-bbug01165"	transcript_id "lnc-bbut00002784"	exon_number "3"
1	lncRNA	exon	118941223	118941881	.	-	.	gene_id "lnc-bbug01165"	transcript_id "lnc-bbut00002784"	exon_number "4"
1	lncRNA	exon	119285452	119285666	.	-	.	gene_id "lnc-bbug01167"	transcript_id "lnc-bbut00002791"	exon_number "1"
1	lncRNA	exon	119286742	119286907	.	-	.	gene_id "lnc-bbug01167"	transcript_id "lnc-bbut00002791"	exon_number "2"
1	lncRNA	exon	119289506	119289613	.	-	.	gene_id "lnc-bbug01167"	transcript_id "lnc-bbut00002791"	exon_number "3"
1	lncRNA	exon	119309156	119309216	.	-	.	gene_id "lnc-bbug01168"	transcript_id "lnc-bbut00002792"	exon_number "1"
1	lncRNA	exon	119313285	119313412	.	-	.	gene_id "lnc-bbug01168"	transcript_id "lnc-bbut00002792"	exon_number "2"
1	lncRNA	exon	119316595	119316820	.	-	.	gene_id "lnc-bbug01168"	transcript_id "lnc-bbut00002792"	exon_number "3"
1	lncRNA	exon	120912986	120913959	.	-	.	gene_id "lnc-bbug01170"	transcript_id "lnc-bbut00002796"	exon_number "1"
1	lncRNA	exon	120917675	120917771	.	-	.	gene_id "lnc-bbug01170"	transcript_id "lnc-bbut00002796"	exon_number "2"
1	lncRNA	exon	120931082	120931296	.	-	.	gene_id "lnc-bbug01170"	transcript_id "lnc-bbut00002796"	exon_number "3"
1	lncRNA	exon	120933778	120933876	.	-	.	gene_id "lnc-bbug01170"	transcript_id "lnc-bbut00002796"	exon_number "4"
1	lncRNA	exon	120949799	120950018	.	-	.	gene_id "lnc-bbug01170"	transcript_id "lnc-bbut00002796"	exon_number "5"
1	lncRNA	exon	124229669	124230952	.	-	.	gene_id "lnc-bbug01177"	transcript_id "lnc-bbut00002826"	exon_number "1"
1	lncRNA	exon	124231659	124231725	.	-	.	gene_id "lnc-bbug01177"	transcript_id "lnc-bbut00002826"	exon_number "2"
1	lncRNA	exon	124234416	124236018	.	-	.	gene_id "lnc-bbug01177"	transcript_id "lnc-bbut00002826"	exon_number "3"
1	lncRNA	exon	124229669	124230952	.	-	.	gene_id "lnc-bbug01177"	transcript_id "lnc-bbut00002827"	exon_number "1"
1	lncRNA	exon	124231659	124231725	.	-	.	gene_id "lnc-bbug01177"	transcript_id "lnc-bbut00002827"	exon_number "2"
1	lncRNA	exon	124234416	124234520	.	-	.	gene_id "lnc-bbug01177"	transcript_id "lnc-bbut00002827"	exon_number "3"
1	lncRNA	exon	124236026	124237510	.	-	.	gene_id "lnc-bbug01177"	transcript_id "lnc-bbut00002827"	exon_number "4"
1	lncRNA	exon	124391282	124391876	.	-	.	gene_id "lnc-bbug01178"	transcript_id "lnc-bbut00002828"	exon_number "1"
1	lncRNA	exon	124393030	124397831	.	-	.	gene_id "lnc-bbug01178"	transcript_id "lnc-bbut00002828"	exon_number "2"
1	lncRNA	exon	124664778	124668571	.	-	.	gene_id "lnc-bbug01179"	transcript_id "lnc-bbut00002829"	exon_number "1"
1	lncRNA	exon	124668654	124668874	.	-	.	gene_id "lnc-bbug01179"	transcript_id "lnc-bbut00002829"	exon_number "2"
1	lncRNA	exon	124672858	124673422	.	-	.	gene_id "lnc-bbug01179"	transcript_id "lnc-bbut00002829"	exon_number "3"
1	lncRNA	exon	124806648	124807789	.	-	.	gene_id "lnc-bbug01181"	transcript_id "lnc-bbut00002832"	exon_number "1"
1	lncRNA	exon	124808547	124816450	.	-	.	gene_id "lnc-bbug01181"	transcript_id "lnc-bbut00002832"	exon_number "2"
1	lncRNA	exon	124818396	124818508	.	-	.	gene_id "lnc-bbug01181"	transcript_id "lnc-bbut00002832"	exon_number "3"
1	lncRNA	exon	124806648	124807789	.	-	.	gene_id "lnc-bbug01181"	transcript_id "lnc-bbut00002833"	exon_number "1"
1	lncRNA	exon	124808547	124816450	.	-	.	gene_id "lnc-bbug01181"	transcript_id "lnc-bbut00002833"	exon_number "2"
1	lncRNA	exon	124817485	124817542	.	-	.	gene_id "lnc-bbug01181"	transcript_id "lnc-bbut00002833"	exon_number "3"
1	lncRNA	exon	124818396	124818514	.	-	.	gene_id "lnc-bbug01181"	transcript_id "lnc-bbut00002833"	exon_number "4"
1	lncRNA	exon	126401156	126401744	.	-	.	gene_id "lnc-bbug01192"	transcript_id "lnc-bbut00002850"	exon_number "1"
1	lncRNA	exon	126402900	126403868	.	-	.	gene_id "lnc-bbug01192"	transcript_id "lnc-bbut00002850"	exon_number "2"
1	lncRNA	exon	126447224	126447446	.	-	.	gene_id "lnc-bbug01194"	transcript_id "lnc-bbut00002854"	exon_number "1"
1	lncRNA	exon	126447872	126448779	.	-	.	gene_id "lnc-bbug01194"	transcript_id "lnc-bbut00002854"	exon_number "2"
1	lncRNA	exon	126453895	126453957	.	-	.	gene_id "lnc-bbug01194"	transcript_id "lnc-bbut00002854"	exon_number "3"
1	lncRNA	exon	126455351	126455536	.	-	.	gene_id "lnc-bbug01194"	transcript_id "lnc-bbut00002854"	exon_number "4"
1	lncRNA	exon	127270466	127270700	.	-	.	gene_id "lnc-bbug01198"	transcript_id "lnc-bbut00002872"	exon_number "1"
1	lncRNA	exon	127270944	127271006	.	-	.	gene_id "lnc-bbug01198"	transcript_id "lnc-bbut00002872"	exon_number "2"
1	lncRNA	exon	127273364	127273516	.	-	.	gene_id "lnc-bbug01198"	transcript_id "lnc-bbut00002872"	exon_number "3"
1	lncRNA	exon	127294283	127294336	.	-	.	gene_id "lnc-bbug01198"	transcript_id "lnc-bbut00002872"	exon_number "4"
1	lncRNA	exon	127295031	127295437	.	-	.	gene_id "lnc-bbug01198"	transcript_id "lnc-bbut00002872"	exon_number "5"
1	lncRNA	exon	127270466	127270700	.	-	.	gene_id "lnc-bbug01198"	transcript_id "lnc-bbut00002873"	exon_number "1"
1	lncRNA	exon	127270944	127271006	.	-	.	gene_id "lnc-bbug01198"	transcript_id "lnc-bbut00002873"	exon_number "2"
1	lncRNA	exon	127273364	127273516	.	-	.	gene_id "lnc-bbug01198"	transcript_id "lnc-bbut00002873"	exon_number "3"

1	lncRNA	exon	127294283	127294336	.	-	.	gene_id "lnc-bbug01198"	transcript_id "lnc-bbut00002873"	exon_number "4"
1	lncRNA	exon	127297097	127297255	.	-	.	gene_id "lnc-bbug01198"	transcript_id "lnc-bbut00002873"	exon_number "5"
1	lncRNA	exon	127657828	127657968	.	-	.	gene_id "lnc-bbug01204"	transcript_id "lnc-bbut00002882"	exon_number "1"
1	lncRNA	exon	127658332	127658422	.	-	.	gene_id "lnc-bbug01204"	transcript_id "lnc-bbut00002882"	exon_number "2"
1	lncRNA	exon	127658587	127658806	.	-	.	gene_id "lnc-bbug01204"	transcript_id "lnc-bbut00002882"	exon_number "3"
1	lncRNA	exon	127792214	127792588	.	-	.	gene_id "lnc-bbug01211"	transcript_id "lnc-bbut00002911"	exon_number "1"
1	lncRNA	exon	127798597	127798739	.	-	.	gene_id "lnc-bbug01211"	transcript_id "lnc-bbut00002911"	exon_number "2"
1	lncRNA	exon	127799033	127801348	.	-	.	gene_id "lnc-bbug01211"	transcript_id "lnc-bbut00002911"	exon_number "3"
1	lncRNA	exon	128385149	128385731	.	-	.	gene_id "lnc-bbug01223"	transcript_id "lnc-bbut00002945"	exon_number "1"
1	lncRNA	exon	128480280	128480884	.	-	.	gene_id "lnc-bbug01224"	transcript_id "lnc-bbut00002946"	exon_number "1"
1	lncRNA	exon	128482759	128482807	.	-	.	gene_id "lnc-bbug01224"	transcript_id "lnc-bbut00002946"	exon_number "2"
1	lncRNA	exon	128483604	128483709	.	-	.	gene_id "lnc-bbug01224"	transcript_id "lnc-bbut00002946"	exon_number "3"
1	lncRNA	exon	128484935	128485104	.	-	.	gene_id "lnc-bbug01224"	transcript_id "lnc-bbut00002946"	exon_number "4"
1	lncRNA	exon	128487898	128488105	.	-	.	gene_id "lnc-bbug01224"	transcript_id "lnc-bbut00002946"	exon_number "5"
1	lncRNA	exon	128480280	128480884	.	-	.	gene_id "lnc-bbug01224"	transcript_id "lnc-bbut00002947"	exon_number "1"
1	lncRNA	exon	128482759	128482807	.	-	.	gene_id "lnc-bbug01224"	transcript_id "lnc-bbut00002947"	exon_number "2"
1	lncRNA	exon	128483604	128484474	.	-	.	gene_id "lnc-bbug01224"	transcript_id "lnc-bbut00002947"	exon_number "3"
1	lncRNA	exon	128484935	128485104	.	-	.	gene_id "lnc-bbug01224"	transcript_id "lnc-bbut00002947"	exon_number "4"
1	lncRNA	exon	128487898	128488115	.	-	.	gene_id "lnc-bbug01224"	transcript_id "lnc-bbut00002947"	exon_number "5"
1	lncRNA	exon	128480280	128480884	.	-	.	gene_id "lnc-bbug01224"	transcript_id "lnc-bbut00002948"	exon_number "1"
1	lncRNA	exon	128482759	128482807	.	-	.	gene_id "lnc-bbug01224"	transcript_id "lnc-bbut00002948"	exon_number "2"
1	lncRNA	exon	128483604	128485104	.	-	.	gene_id "lnc-bbug01224"	transcript_id "lnc-bbut00002948"	exon_number "3"
1	lncRNA	exon	128487898	128488120	.	-	.	gene_id "lnc-bbug01224"	transcript_id "lnc-bbut00002948"	exon_number "4"
1	lncRNA	exon	128480280	128480884	.	-	.	gene_id "lnc-bbug01224"	transcript_id "lnc-bbut00002949"	exon_number "1"
1	lncRNA	exon	128482759	128482807	.	-	.	gene_id "lnc-bbug01224"	transcript_id "lnc-bbut00002949"	exon_number "2"
1	lncRNA	exon	128483604	128483709	.	-	.	gene_id "lnc-bbug01224"	transcript_id "lnc-bbut00002949"	exon_number "3"
1	lncRNA	exon	128484248	128485104	.	-	.	gene_id "lnc-bbug01224"	transcript_id "lnc-bbut00002949"	exon_number "4"
1	lncRNA	exon	128487898	128488123	.	-	.	gene_id "lnc-bbug01224"	transcript_id "lnc-bbut00002949"	exon_number "5"
1	lncRNA	exon	129137403	129137939	.	-	.	gene_id "lnc-bbug01227"	transcript_id "lnc-bbut00002960"	exon_number "1"
1	lncRNA	exon	130067267	130067511	.	-	.	gene_id "lnc-bbug01228"	transcript_id "lnc-bbut00002961"	exon_number "1"
1	lncRNA	exon	130083586	130083753	.	-	.	gene_id "lnc-bbug01228"	transcript_id "lnc-bbut00002961"	exon_number "2"
1	lncRNA	exon	130104220	130104926	.	-	.	gene_id "lnc-bbug01228"	transcript_id "lnc-bbut00002961"	exon_number "3"
1	lncRNA	exon	130129210	130134678	.	-	.	gene_id "lnc-bbug01229"	transcript_id "lnc-bbut00002962"	exon_number "1"
1	lncRNA	exon	130136652	130136803	.	-	.	gene_id "lnc-bbug01229"	transcript_id "lnc-bbut00002962"	exon_number "2"
1	lncRNA	exon	130138628	130138714	.	-	.	gene_id "lnc-bbug01229"	transcript_id "lnc-bbut00002962"	exon_number "3"
1	lncRNA	exon	130158145	130158252	.	-	.	gene_id "lnc-bbug01229"	transcript_id "lnc-bbut00002962"	exon_number "4"
1	lncRNA	exon	130173769	130174054	.	-	.	gene_id "lnc-bbug01229"	transcript_id "lnc-bbut00002962"	exon_number "5"
1	lncRNA	exon	130265846	130265914	.	-	.	gene_id "lnc-bbug01229"	transcript_id "lnc-bbut00002962"	exon_number "6"
1	lncRNA	exon	130266033	130268266	.	-	.	gene_id "lnc-bbug01229"	transcript_id "lnc-bbut00002962"	exon_number "7"
1	lncRNA	exon	130129210	130134678	.	-	.	gene_id "lnc-bbug01229"	transcript_id "lnc-bbut00002963"	exon_number "1"
1	lncRNA	exon	130136652	130136803	.	-	.	gene_id "lnc-bbug01229"	transcript_id "lnc-bbut00002963"	exon_number "2"
1	lncRNA	exon	130138628	130138714	.	-	.	gene_id "lnc-bbug01229"	transcript_id "lnc-bbut00002963"	exon_number "3"
1	lncRNA	exon	130158145	130158252	.	-	.	gene_id "lnc-bbug01229"	transcript_id "lnc-bbut00002963"	exon_number "4"
1	lncRNA	exon	130173769	130174054	.	-	.	gene_id "lnc-bbug01229"	transcript_id "lnc-bbut00002963"	exon_number "5"
1	lncRNA	exon	130265846	130265918	.	-	.	gene_id "lnc-bbug01229"	transcript_id "lnc-bbut00002963"	exon_number "6"
1	lncRNA	exon	130266033	130268267	.	-	.	gene_id "lnc-bbug01229"	transcript_id "lnc-bbut00002963"	exon_number "7"
1	lncRNA	exon	130244946	130245105	.	-	.	gene_id "lnc-bbug01231"	transcript_id "lnc-bbut00002965"	exon_number "1"
1	lncRNA	exon	130250525	130252964	.	-	.	gene_id "lnc-bbug01231"	transcript_id "lnc-bbut00002965"	exon_number "2"
1	lncRNA	exon	134090377	134090581	.	-	.	gene_id "lnc-bbug01242"	transcript_id "lnc-bbut00002990"	exon_number "1"
1	lncRNA	exon	134126217	134126274	.	-	.	gene_id "lnc-bbug01242"	transcript_id "lnc-bbut00002990"	exon_number "2"
1	lncRNA	exon	134153676	134153739	.	-	.	gene_id "lnc-bbug01242"	transcript_id "lnc-bbut00002990"	exon_number "3"
1	lncRNA	exon	134326081	134326222	.	-	.	gene_id "lnc-bbug01242"	transcript_id "lnc-bbut00002990"	exon_number "4"

1	lncRNA	exon	134538055	134538383	.	-	.	gene_id "lnc-bbug01242"	transcript_id "lnc-bbut00002990"	exon_number "5"
1	lncRNA	exon	134565780	134567057	.	-	.	gene_id "lnc-bbug01242"	transcript_id "lnc-bbut00002990"	exon_number "6"
1	lncRNA	exon	140063072	140063131	.	-	.	gene_id "lnc-bbug01247"	transcript_id "lnc-bbut00003011"	exon_number "1"
1	lncRNA	exon	140085629	140085728	.	-	.	gene_id "lnc-bbug01247"	transcript_id "lnc-bbut00003011"	exon_number "2"
1	lncRNA	exon	140123151	140123575	.	-	.	gene_id "lnc-bbug01247"	transcript_id "lnc-bbut00003011"	exon_number "3"
1	lncRNA	exon	140138750	140138797	.	-	.	gene_id "lnc-bbug01248"	transcript_id "lnc-bbut00003012"	exon_number "1"
1	lncRNA	exon	140149784	140149964	.	-	.	gene_id "lnc-bbug01248"	transcript_id "lnc-bbut00003012"	exon_number "2"
1	lncRNA	exon	140169597	140170036	.	-	.	gene_id "lnc-bbug01248"	transcript_id "lnc-bbut00003012"	exon_number "3"
1	lncRNA	exon	140486161	140486831	.	-	.	gene_id "lnc-bbug01251"	transcript_id "lnc-bbut00003016"	exon_number "1"
1	lncRNA	exon	140587474	140587588	.	-	.	gene_id "lnc-bbug01251"	transcript_id "lnc-bbut00003016"	exon_number "2"
1	lncRNA	exon	140593419	140594127	.	-	.	gene_id "lnc-bbug01251"	transcript_id "lnc-bbut00003016"	exon_number "3"
1	lncRNA	exon	140537101	140538723	.	-	.	gene_id "lnc-bbug01251"	transcript_id "lnc-bbut00003017"	exon_number "1"
1	lncRNA	exon	140587474	140587588	.	-	.	gene_id "lnc-bbug01251"	transcript_id "lnc-bbut00003017"	exon_number "2"
1	lncRNA	exon	140593419	140594127	.	-	.	gene_id "lnc-bbug01251"	transcript_id "lnc-bbut00003017"	exon_number "3"
1	lncRNA	exon	140573168	140574962	.	-	.	gene_id "lnc-bbug01251"	transcript_id "lnc-bbut00003018"	exon_number "1"
1	lncRNA	exon	140587474	140587588	.	-	.	gene_id "lnc-bbug01251"	transcript_id "lnc-bbut00003018"	exon_number "2"
1	lncRNA	exon	140593419	140594127	.	-	.	gene_id "lnc-bbug01251"	transcript_id "lnc-bbut00003018"	exon_number "3"
1	lncRNA	exon	141799459	141800059	.	-	.	gene_id "lnc-bbug01255"	transcript_id "lnc-bbut00003024"	exon_number "1"
1	lncRNA	exon	141802299	141802340	.	-	.	gene_id "lnc-bbug01255"	transcript_id "lnc-bbut00003024"	exon_number "2"
1	lncRNA	exon	141912222	141912640	.	-	.	gene_id "lnc-bbug01257"	transcript_id "lnc-bbut00003030"	exon_number "1"
1	lncRNA	exon	141920818	141920859	.	-	.	gene_id "lnc-bbug01257"	transcript_id "lnc-bbut00003030"	exon_number "2"
1	lncRNA	exon	141922380	141924357	.	-	.	gene_id "lnc-bbug01257"	transcript_id "lnc-bbut00003030"	exon_number "3"
1	lncRNA	exon	141912966	141914955	.	-	.	gene_id "lnc-bbug01257"	transcript_id "lnc-bbut00003031"	exon_number "1"
1	lncRNA	exon	141920818	141920859	.	-	.	gene_id "lnc-bbug01257"	transcript_id "lnc-bbut00003031"	exon_number "2"
1	lncRNA	exon	141922380	141924357	.	-	.	gene_id "lnc-bbug01257"	transcript_id "lnc-bbut00003031"	exon_number "3"
1	lncRNA	exon	142139061	142140344	.	-	.	gene_id "lnc-bbug01262"	transcript_id "lnc-bbut00003037"	exon_number "1"
1	lncRNA	exon	142141586	142142224	.	-	.	gene_id "lnc-bbug01262"	transcript_id "lnc-bbut00003037"	exon_number "2"
1	lncRNA	exon	142252950	142253097	.	-	.	gene_id "lnc-bbug01266"	transcript_id "lnc-bbut00003043"	exon_number "1"
1	lncRNA	exon	142255038	142255124	.	-	.	gene_id "lnc-bbug01266"	transcript_id "lnc-bbut00003043"	exon_number "2"
1	lncRNA	exon	142255491	142255553	.	-	.	gene_id "lnc-bbug01266"	transcript_id "lnc-bbut00003043"	exon_number "3"
1	lncRNA	exon	142255625	142255798	.	-	.	gene_id "lnc-bbug01266"	transcript_id "lnc-bbut00003043"	exon_number "4"
1	lncRNA	exon	142256696	142257019	.	-	.	gene_id "lnc-bbug01266"	transcript_id "lnc-bbut00003043"	exon_number "5"
1	lncRNA	exon	142261073	142261299	.	-	.	gene_id "lnc-bbug01267"	transcript_id "lnc-bbut00003044"	exon_number "1"
1	lncRNA	exon	142262348	142262612	.	-	.	gene_id "lnc-bbug01267"	transcript_id "lnc-bbut00003044"	exon_number "2"
1	lncRNA	exon	142547166	142550745	.	-	.	gene_id "lnc-bbug01268"	transcript_id "lnc-bbut00003045"	exon_number "1"
1	lncRNA	exon	142552234	142552320	.	-	.	gene_id "lnc-bbug01268"	transcript_id "lnc-bbut00003045"	exon_number "2"
1	lncRNA	exon	142552585	142552759	.	-	.	gene_id "lnc-bbug01268"	transcript_id "lnc-bbut00003045"	exon_number "3"
1	lncRNA	exon	142569038	142569413	.	-	.	gene_id "lnc-bbug01268"	transcript_id "lnc-bbut00003045"	exon_number "4"
1	lncRNA	exon	142919659	142919893	.	-	.	gene_id "lnc-bbug01269"	transcript_id "lnc-bbut00003046"	exon_number "1"
1	lncRNA	exon	142920006	142920115	.	-	.	gene_id "lnc-bbug01269"	transcript_id "lnc-bbut00003046"	exon_number "2"
1	lncRNA	exon	142941450	142941716	.	-	.	gene_id "lnc-bbug01269"	transcript_id "lnc-bbut00003046"	exon_number "3"
1	lncRNA	exon	145276897	145277413	.	-	.	gene_id "lnc-bbug01273"	transcript_id "lnc-bbut00003062"	exon_number "1"
1	lncRNA	exon	145278696	145278883	.	-	.	gene_id "lnc-bbug01273"	transcript_id "lnc-bbut00003062"	exon_number "2"
1	lncRNA	exon	145279322	145279440	.	-	.	gene_id "lnc-bbug01273"	transcript_id "lnc-bbut00003062"	exon_number "3"
1	lncRNA	exon	145285926	145286044	.	-	.	gene_id "lnc-bbug01273"	transcript_id "lnc-bbut00003062"	exon_number "4"
1	lncRNA	exon	145534987	145535798	.	-	.	gene_id "lnc-bbug01275"	transcript_id "lnc-bbut00003064"	exon_number "1"
1	lncRNA	exon	146225023	146228651	.	-	.	gene_id "lnc-bbug01276"	transcript_id "lnc-bbut00003065"	exon_number "1"
1	lncRNA	exon	150532486	150532559	.	-	.	gene_id "lnc-bbug01278"	transcript_id "lnc-bbut00003067"	exon_number "1"
1	lncRNA	exon	150538581	150538655	.	-	.	gene_id "lnc-bbug01278"	transcript_id "lnc-bbut00003067"	exon_number "2"
1	lncRNA	exon	150571181	150571383	.	-	.	gene_id "lnc-bbug01278"	transcript_id "lnc-bbut00003067"	exon_number "3"
1	lncRNA	exon	150881088	150881723	.	-	.	gene_id "lnc-bbug01280"	transcript_id "lnc-bbut00003069"	exon_number "1"
1	lncRNA	exon	150883665	150883818	.	-	.	gene_id "lnc-bbug01280"	transcript_id "lnc-bbut00003069"	exon_number "2"

1	lncRNA	exon	150901116	150901245	.	-	.	gene_id "lnc-bbug01280"	transcript_id "lnc-bbut00003069"	exon_number "3"
1	lncRNA	exon	151745904	151746637	.	-	.	gene_id "lnc-bbug01285"	transcript_id "lnc-bbut00003077"	exon_number "1"
1	lncRNA	exon	151796854	151796900	.	-	.	gene_id "lnc-bbug01286"	transcript_id "lnc-bbut00003078"	exon_number "1"
1	lncRNA	exon	151797906	151799045	.	-	.	gene_id "lnc-bbug01286"	transcript_id "lnc-bbut00003078"	exon_number "2"
1	lncRNA	exon	151801319	151801817	.	-	.	gene_id "lnc-bbug01286"	transcript_id "lnc-bbut00003078"	exon_number "3"
1	lncRNA	exon	152305855	152308808	.	-	.	gene_id "lnc-bbug01290"	transcript_id "lnc-bbut00003088"	exon_number "1"
1	lncRNA	exon	153773463	153775395	.	-	.	gene_id "lnc-bbug01295"	transcript_id "lnc-bbut00003096"	exon_number "1"
1	lncRNA	exon	153776524	153778865	.	-	.	gene_id "lnc-bbug01295"	transcript_id "lnc-bbut00003096"	exon_number "2"
1	lncRNA	exon	154985159	154986015	.	-	.	gene_id "lnc-bbug01297"	transcript_id "lnc-bbut00003098"	exon_number "1"
1	lncRNA	exon	154995760	154995889	.	-	.	gene_id "lnc-bbug01297"	transcript_id "lnc-bbut00003098"	exon_number "2"
1	lncRNA	exon	155058598	155059265	.	-	.	gene_id "lnc-bbug01299"	transcript_id "lnc-bbut00003100"	exon_number "1"
1	lncRNA	exon	155063716	155063814	.	-	.	gene_id "lnc-bbug01299"	transcript_id "lnc-bbut00003100"	exon_number "2"
1	lncRNA	exon	155066313	155066822	.	-	.	gene_id "lnc-bbug01299"	transcript_id "lnc-bbut00003100"	exon_number "3"
1	lncRNA	exon	155082779	155083907	.	-	.	gene_id "lnc-bbug01300"	transcript_id "lnc-bbut00003101"	exon_number "1"
1	lncRNA	exon	155091636	155091974	.	-	.	gene_id "lnc-bbug01300"	transcript_id "lnc-bbut00003101"	exon_number "2"
1	lncRNA	exon	155095682	155095820	.	-	.	gene_id "lnc-bbug01300"	transcript_id "lnc-bbut00003101"	exon_number "3"
1	lncRNA	exon	155097080	155097177	.	-	.	gene_id "lnc-bbug01300"	transcript_id "lnc-bbut00003101"	exon_number "4"
1	lncRNA	exon	155098256	155099201	.	-	.	gene_id "lnc-bbug01300"	transcript_id "lnc-bbut00003101"	exon_number "5"
1	lncRNA	exon	155082779	155083907	.	-	.	gene_id "lnc-bbug01300"	transcript_id "lnc-bbut00003102"	exon_number "1"
1	lncRNA	exon	155091636	155091974	.	-	.	gene_id "lnc-bbug01300"	transcript_id "lnc-bbut00003102"	exon_number "2"
1	lncRNA	exon	155095682	155097177	.	-	.	gene_id "lnc-bbug01300"	transcript_id "lnc-bbut00003102"	exon_number "3"
1	lncRNA	exon	155098256	155099216	.	-	.	gene_id "lnc-bbug01300"	transcript_id "lnc-bbut00003102"	exon_number "4"
1	lncRNA	exon	155685205	155686351	.	-	.	gene_id "lnc-bbug01305"	transcript_id "lnc-bbut00003114"	exon_number "1"
1	lncRNA	exon	157217844	157218282	.	-	.	gene_id "lnc-bbug01310"	transcript_id "lnc-bbut00003126"	exon_number "1"
1	lncRNA	exon	158221042	158222654	.	-	.	gene_id "lnc-bbug01312"	transcript_id "lnc-bbut00003128"	exon_number "1"
1	lncRNA	exon	158340008	158340820	.	-	.	gene_id "lnc-bbug01313"	transcript_id "lnc-bbut00003129"	exon_number "1"
1	lncRNA	exon	158387440	158387545	.	-	.	gene_id "lnc-bbug01313"	transcript_id "lnc-bbut00003129"	exon_number "2"
1	lncRNA	exon	159087610	159087960	.	-	.	gene_id "lnc-bbug01316"	transcript_id "lnc-bbut00003132"	exon_number "1"
1	lncRNA	exon	159091065	159091230	.	-	.	gene_id "lnc-bbug01316"	transcript_id "lnc-bbut00003132"	exon_number "2"
1	lncRNA	exon	159572642	159572691	.	-	.	gene_id "lnc-bbug01319"	transcript_id "lnc-bbut00003135"	exon_number "1"
1	lncRNA	exon	159596367	159596504	.	-	.	gene_id "lnc-bbug01319"	transcript_id "lnc-bbut00003135"	exon_number "2"
1	lncRNA	exon	159610545	159610762	.	-	.	gene_id "lnc-bbug01319"	transcript_id "lnc-bbut00003135"	exon_number "3"
1	lncRNA	exon	159734569	159734681	.	-	.	gene_id "lnc-bbug01319"	transcript_id "lnc-bbut00003135"	exon_number "4"
1	lncRNA	exon	160525352	160526537	.	-	.	gene_id "lnc-bbug01322"	transcript_id "lnc-bbut00003162"	exon_number "1"
1	lncRNA	exon	160534818	160534989	.	-	.	gene_id "lnc-bbug01322"	transcript_id "lnc-bbut00003162"	exon_number "2"
1	lncRNA	exon	160536493	160536562	.	-	.	gene_id "lnc-bbug01322"	transcript_id "lnc-bbut00003162"	exon_number "3"
1	lncRNA	exon	160543454	160543675	.	-	.	gene_id "lnc-bbug01322"	transcript_id "lnc-bbut00003162"	exon_number "4"
1	lncRNA	exon	160552401	160555391	.	-	.	gene_id "lnc-bbug01322"	transcript_id "lnc-bbut00003162"	exon_number "5"
1	lncRNA	exon	160585456	160587192	.	-	.	gene_id "lnc-bbug01324"	transcript_id "lnc-bbut00003167"	exon_number "1"
1	lncRNA	exon	160587944	160587950	.	-	.	gene_id "lnc-bbug01324"	transcript_id "lnc-bbut00003167"	exon_number "2"
1	lncRNA	exon	160585456	160590471	.	-	.	gene_id "lnc-bbug01324"	transcript_id "lnc-bbut00003168"	exon_number "1"
1	lncRNA	exon	160590851	160592137	.	-	.	gene_id "lnc-bbug01325"	transcript_id "lnc-bbut00003169"	exon_number "1"
1	lncRNA	exon	160614681	160614746	.	-	.	gene_id "lnc-bbug01325"	transcript_id "lnc-bbut00003169"	exon_number "2"
1	lncRNA	exon	160624720	160625095	.	-	.	gene_id "lnc-bbug01325"	transcript_id "lnc-bbut00003169"	exon_number "3"
1	lncRNA	exon	160783213	160783813	.	-	.	gene_id "lnc-bbug01328"	transcript_id "lnc-bbut00003172"	exon_number "1"
1	lncRNA	exon	160785557	160785728	.	-	.	gene_id "lnc-bbug01328"	transcript_id "lnc-bbut00003172"	exon_number "2"
1	lncRNA	exon	160787038	160787117	.	-	.	gene_id "lnc-bbug01328"	transcript_id "lnc-bbut00003172"	exon_number "3"
1	lncRNA	exon	160826312	160826914	.	-	.	gene_id "lnc-bbug01329"	transcript_id "lnc-bbut00003173"	exon_number "1"
1	lncRNA	exon	160829883	160830007	.	-	.	gene_id "lnc-bbug01329"	transcript_id "lnc-bbut00003173"	exon_number "2"
1	lncRNA	exon	160836247	160836412	.	-	.	gene_id "lnc-bbug01329"	transcript_id "lnc-bbut00003173"	exon_number "3"
1	lncRNA	exon	161040228	161040713	.	-	.	gene_id "lnc-bbug01331"	transcript_id "lnc-bbut00003175"	exon_number "1"
1	lncRNA	exon	161901886	161902436	.	-	.	gene_id "lnc-bbug01333"	transcript_id "lnc-bbut00003185"	exon_number "1"

1	lncRNA	exon	161902861	161902989	.	-	.	gene_id "lnc-bbug01333"	transcript_id "lnc-bbut00003185"	exon_number "2"
1	lncRNA	exon	162927808	162928615	.	-	.	gene_id "lnc-bbug01338"	transcript_id "lnc-bbut00003199"	exon_number "1"
1	lncRNA	exon	162943253	162943409	.	-	.	gene_id "lnc-bbug01338"	transcript_id "lnc-bbut00003199"	exon_number "2"
1	lncRNA	exon	162943560	162943747	.	-	.	gene_id "lnc-bbug01338"	transcript_id "lnc-bbut00003199"	exon_number "3"
1	lncRNA	exon	163033134	163034974	.	-	.	gene_id "lnc-bbug01339"	transcript_id "lnc-bbut00003200"	exon_number "1"
1	lncRNA	exon	163036018	163036088	.	-	.	gene_id "lnc-bbug01339"	transcript_id "lnc-bbut00003200"	exon_number "2"
1	lncRNA	exon	163036441	163036581	.	-	.	gene_id "lnc-bbug01339"	transcript_id "lnc-bbut00003200"	exon_number "3"
1	lncRNA	exon	164240647	164240768	.	-	.	gene_id "lnc-bbug01346"	transcript_id "lnc-bbut00003216"	exon_number "1"
1	lncRNA	exon	164242008	164242589	.	-	.	gene_id "lnc-bbug01346"	transcript_id "lnc-bbut00003216"	exon_number "2"
1	lncRNA	exon	164246262	164246371	.	-	.	gene_id "lnc-bbug01346"	transcript_id "lnc-bbut00003216"	exon_number "3"
1	lncRNA	exon	164847698	164848076	.	-	.	gene_id "lnc-bbug01348"	transcript_id "lnc-bbut00003219"	exon_number "1"
1	lncRNA	exon	164848460	164848679	.	-	.	gene_id "lnc-bbug01348"	transcript_id "lnc-bbut00003219"	exon_number "2"
1	lncRNA	exon	164852080	164852200	.	-	.	gene_id "lnc-bbug01348"	transcript_id "lnc-bbut00003219"	exon_number "3"
1	lncRNA	exon	164854010	164854046	.	-	.	gene_id "lnc-bbug01348"	transcript_id "lnc-bbut00003219"	exon_number "4"
1	lncRNA	exon	164874522	164874615	.	-	.	gene_id "lnc-bbug01348"	transcript_id "lnc-bbut00003219"	exon_number "5"
1	lncRNA	exon	164847698	164848076	.	-	.	gene_id "lnc-bbug01348"	transcript_id "lnc-bbut00003218"	exon_number "1"
1	lncRNA	exon	164848460	164848679	.	-	.	gene_id "lnc-bbug01348"	transcript_id "lnc-bbut00003218"	exon_number "2"
1	lncRNA	exon	164852080	164852200	.	-	.	gene_id "lnc-bbug01348"	transcript_id "lnc-bbut00003218"	exon_number "3"
1	lncRNA	exon	164853875	164854046	.	-	.	gene_id "lnc-bbug01348"	transcript_id "lnc-bbut00003218"	exon_number "4"
1	lncRNA	exon	164874522	164874615	.	-	.	gene_id "lnc-bbug01348"	transcript_id "lnc-bbut00003218"	exon_number "5"
1	lncRNA	exon	165703764	165703943	.	-	.	gene_id "lnc-bbug01350"	transcript_id "lnc-bbut00003221"	exon_number "1"
1	lncRNA	exon	165705129	165705420	.	-	.	gene_id "lnc-bbug01350"	transcript_id "lnc-bbut00003221"	exon_number "2"
1	lncRNA	exon	166495358	166497808	.	-	.	gene_id "lnc-bbug01351"	transcript_id "lnc-bbut00003222"	exon_number "1"
1	lncRNA	exon	166498210	166498292	.	-	.	gene_id "lnc-bbug01351"	transcript_id "lnc-bbut00003222"	exon_number "2"
1	lncRNA	exon	166498549	166498595	.	-	.	gene_id "lnc-bbug01351"	transcript_id "lnc-bbut00003222"	exon_number "3"
1	lncRNA	exon	166501042	166501161	.	-	.	gene_id "lnc-bbug01351"	transcript_id "lnc-bbut00003222"	exon_number "4"
1	lncRNA	exon	166501251	166501483	.	-	.	gene_id "lnc-bbug01351"	transcript_id "lnc-bbut00003222"	exon_number "5"
1	lncRNA	exon	169075483	169075607	.	-	.	gene_id "lnc-bbug01352"	transcript_id "lnc-bbut00003226"	exon_number "1"
1	lncRNA	exon	169089036	169089172	.	-	.	gene_id "lnc-bbug01352"	transcript_id "lnc-bbut00003226"	exon_number "2"
1	lncRNA	exon	169092631	169092759	.	-	.	gene_id "lnc-bbug01352"	transcript_id "lnc-bbut00003226"	exon_number "3"
1	lncRNA	exon	169093275	169093583	.	-	.	gene_id "lnc-bbug01352"	transcript_id "lnc-bbut00003226"	exon_number "4"
1	lncRNA	exon	169266655	169269321	.	-	.	gene_id "lnc-bbug01353"	transcript_id "lnc-bbut00003227"	exon_number "1"
1	lncRNA	exon	169302202	169302233	.	-	.	gene_id "lnc-bbug01354"	transcript_id "lnc-bbut00003228"	exon_number "1"
1	lncRNA	exon	169311121	169311182	.	-	.	gene_id "lnc-bbug01354"	transcript_id "lnc-bbut00003228"	exon_number "2"
1	lncRNA	exon	169335777	169335899	.	-	.	gene_id "lnc-bbug01354"	transcript_id "lnc-bbut00003228"	exon_number "3"
1	lncRNA	exon	170105511	170105636	.	-	.	gene_id "lnc-bbug01356"	transcript_id "lnc-bbut00003231"	exon_number "1"
1	lncRNA	exon	170124345	170124379	.	-	.	gene_id "lnc-bbug01356"	transcript_id "lnc-bbut00003231"	exon_number "2"
1	lncRNA	exon	170140728	170140965	.	-	.	gene_id "lnc-bbug01356"	transcript_id "lnc-bbut00003231"	exon_number "3"
1	lncRNA	exon	170141077	170141230	.	-	.	gene_id "lnc-bbug01356"	transcript_id "lnc-bbut00003231"	exon_number "4"
1	lncRNA	exon	171694824	171695743	.	-	.	gene_id "lnc-bbug01366"	transcript_id "lnc-bbut00003252"	exon_number "1"
1	lncRNA	exon	171698067	171698114	.	-	.	gene_id "lnc-bbug01366"	transcript_id "lnc-bbut00003252"	exon_number "2"
1	lncRNA	exon	171701597	171701856	.	-	.	gene_id "lnc-bbug01366"	transcript_id "lnc-bbut00003252"	exon_number "3"
1	lncRNA	exon	171694824	171695743	.	-	.	gene_id "lnc-bbug01366"	transcript_id "lnc-bbut00003253"	exon_number "1"
1	lncRNA	exon	171698067	171698114	.	-	.	gene_id "lnc-bbug01366"	transcript_id "lnc-bbut00003253"	exon_number "2"
1	lncRNA	exon	171701604	171701864	.	-	.	gene_id "lnc-bbug01366"	transcript_id "lnc-bbut00003253"	exon_number "3"
1	lncRNA	exon	171695750	171696757	.	-	.	gene_id "lnc-bbug01366"	transcript_id "lnc-bbut00003254"	exon_number "1"
1	lncRNA	exon	171698067	171698114	.	-	.	gene_id "lnc-bbug01366"	transcript_id "lnc-bbut00003254"	exon_number "2"
1	lncRNA	exon	171701604	171701873	.	-	.	gene_id "lnc-bbug01366"	transcript_id "lnc-bbut00003254"	exon_number "3"
1	lncRNA	exon	172058194	172058878	.	-	.	gene_id "lnc-bbug01369"	transcript_id "lnc-bbut00003275"	exon_number "1"
1	lncRNA	exon	172061575	172061679	.	-	.	gene_id "lnc-bbug01369"	transcript_id "lnc-bbut00003275"	exon_number "2"
1	lncRNA	exon	172063099	172063253	.	-	.	gene_id "lnc-bbug01369"	transcript_id "lnc-bbut00003275"	exon_number "3"
1	lncRNA	exon	172058194	172058878	.	-	.	gene_id "lnc-bbug01369"	transcript_id "lnc-bbut00003276"	exon_number "1"

1	lncRNA	exon	172061575	172061679	.	-	.	gene_id "lnc-bbug01369"	transcript_id "lnc-bbut00003276"	exon_number "2"
1	lncRNA	exon	172076853	172076918	.	-	.	gene_id "lnc-bbug01369"	transcript_id "lnc-bbut00003276"	exon_number "3"
1	lncRNA	exon	172095054	172095128	.	-	.	gene_id "lnc-bbug01369"	transcript_id "lnc-bbut00003276"	exon_number "4"
1	lncRNA	exon	172096253	172096396	.	-	.	gene_id "lnc-bbug01369"	transcript_id "lnc-bbut00003276"	exon_number "5"
1	lncRNA	exon	172158618	172158924	.	-	.	gene_id "lnc-bbug01369"	transcript_id "lnc-bbut00003276"	exon_number "6"
1	lncRNA	exon	172081008	172081409	.	-	.	gene_id "lnc-bbug01369"	transcript_id "lnc-bbut00003277"	exon_number "1"
1	lncRNA	exon	172095054	172095128	.	-	.	gene_id "lnc-bbug01369"	transcript_id "lnc-bbut00003277"	exon_number "2"
1	lncRNA	exon	172096253	172096396	.	-	.	gene_id "lnc-bbug01369"	transcript_id "lnc-bbut00003277"	exon_number "3"
1	lncRNA	exon	172158618	172158922	.	-	.	gene_id "lnc-bbug01369"	transcript_id "lnc-bbut00003277"	exon_number "4"
1	lncRNA	exon	172082003	172085492	.	-	.	gene_id "lnc-bbug01369"	transcript_id "lnc-bbut00003278"	exon_number "1"
1	lncRNA	exon	172095054	172095128	.	-	.	gene_id "lnc-bbug01369"	transcript_id "lnc-bbut00003278"	exon_number "2"
1	lncRNA	exon	172096253	172096396	.	-	.	gene_id "lnc-bbug01369"	transcript_id "lnc-bbut00003278"	exon_number "3"
1	lncRNA	exon	172158618	172158923	.	-	.	gene_id "lnc-bbug01369"	transcript_id "lnc-bbut00003278"	exon_number "4"
1	lncRNA	exon	172086620	172088035	.	-	.	gene_id "lnc-bbug01369"	transcript_id "lnc-bbut00003280"	exon_number "1"
1	lncRNA	exon	172095054	172095128	.	-	.	gene_id "lnc-bbug01369"	transcript_id "lnc-bbut00003280"	exon_number "2"
1	lncRNA	exon	172096253	172096396	.	-	.	gene_id "lnc-bbug01369"	transcript_id "lnc-bbut00003280"	exon_number "3"
1	lncRNA	exon	172158618	172158923	.	-	.	gene_id "lnc-bbug01369"	transcript_id "lnc-bbut00003280"	exon_number "4"
1	lncRNA	exon	172086620	172087904	.	-	.	gene_id "lnc-bbug01369"	transcript_id "lnc-bbut00003279"	exon_number "1"
1	lncRNA	exon	172095054	172095128	.	-	.	gene_id "lnc-bbug01369"	transcript_id "lnc-bbut00003279"	exon_number "2"
1	lncRNA	exon	172096253	172096396	.	-	.	gene_id "lnc-bbug01369"	transcript_id "lnc-bbut00003279"	exon_number "3"
1	lncRNA	exon	172158618	172158923	.	-	.	gene_id "lnc-bbug01369"	transcript_id "lnc-bbut00003279"	exon_number "4"
1	lncRNA	exon	172426354	172426549	.	-	.	gene_id "lnc-bbug01372"	transcript_id "lnc-bbut00003288"	exon_number "1"
1	lncRNA	exon	172427126	172427200	.	-	.	gene_id "lnc-bbug01372"	transcript_id "lnc-bbut00003288"	exon_number "2"
1	lncRNA	exon	172446223	172446354	.	-	.	gene_id "lnc-bbug01372"	transcript_id "lnc-bbut00003288"	exon_number "3"
1	lncRNA	exon	172426354	172426549	.	-	.	gene_id "lnc-bbug01372"	transcript_id "lnc-bbut00003289"	exon_number "1"
1	lncRNA	exon	172427126	172427200	.	-	.	gene_id "lnc-bbug01372"	transcript_id "lnc-bbut00003289"	exon_number "2"
1	lncRNA	exon	172443657	172443743	.	-	.	gene_id "lnc-bbug01372"	transcript_id "lnc-bbut00003289"	exon_number "3"
1	lncRNA	exon	172446223	172446361	.	-	.	gene_id "lnc-bbug01372"	transcript_id "lnc-bbut00003289"	exon_number "4"
1	lncRNA	exon	172569862	172569951	.	-	.	gene_id "lnc-bbug01374"	transcript_id "lnc-bbut00003292"	exon_number "1"
1	lncRNA	exon	172570151	172570400	.	-	.	gene_id "lnc-bbug01374"	transcript_id "lnc-bbut00003292"	exon_number "2"
1	lncRNA	exon	174063929	174064279	.	-	.	gene_id "lnc-bbug01377"	transcript_id "lnc-bbut00003295"	exon_number "1"
1	lncRNA	exon	174163721	174164081	.	-	.	gene_id "lnc-bbug01377"	transcript_id "lnc-bbut00003295"	exon_number "2"
1	lncRNA	exon	174616783	174617245	.	-	.	gene_id "lnc-bbug01381"	transcript_id "lnc-bbut00003301"	exon_number "1"
1	lncRNA	exon	174813632	174813996	.	-	.	gene_id "lnc-bbug01382"	transcript_id "lnc-bbut00003302"	exon_number "1"
1	lncRNA	exon	177935142	177935399	.	-	.	gene_id "lnc-bbug01395"	transcript_id "lnc-bbut00003352"	exon_number "1"
1	lncRNA	exon	177937636	177937700	.	-	.	gene_id "lnc-bbug01395"	transcript_id "lnc-bbut00003352"	exon_number "2"
1	lncRNA	exon	177938411	177938604	.	-	.	gene_id "lnc-bbug01395"	transcript_id "lnc-bbut00003352"	exon_number "3"
1	lncRNA	exon	179739239	179739849	.	-	.	gene_id "lnc-bbug01398"	transcript_id "lnc-bbut00003362"	exon_number "1"
1	lncRNA	exon	179741595	179741688	.	-	.	gene_id "lnc-bbug01398"	transcript_id "lnc-bbut00003362"	exon_number "2"
1	lncRNA	exon	180563851	180564242	.	-	.	gene_id "lnc-bbug01405"	transcript_id "lnc-bbut00003377"	exon_number "1"
1	lncRNA	exon	181145677	181146501	.	-	.	gene_id "lnc-bbug01407"	transcript_id "lnc-bbut00003381"	exon_number "1"
1	lncRNA	exon	182981904	182982379	.	-	.	gene_id "lnc-bbug01412"	transcript_id "lnc-bbut00003391"	exon_number "1"
1	lncRNA	exon	184232895	184232937	.	-	.	gene_id "lnc-bbug01421"	transcript_id "lnc-bbut00003422"	exon_number "1"
1	lncRNA	exon	184235518	184235631	.	-	.	gene_id "lnc-bbug01421"	transcript_id "lnc-bbut00003422"	exon_number "2"
1	lncRNA	exon	184239524	184240093	.	-	.	gene_id "lnc-bbug01421"	transcript_id "lnc-bbut00003422"	exon_number "3"
1	lncRNA	exon	185318022	185318304	.	-	.	gene_id "lnc-bbug01423"	transcript_id "lnc-bbut00003425"	exon_number "1"
1	lncRNA	exon	185326331	185327243	.	-	.	gene_id "lnc-bbug01423"	transcript_id "lnc-bbut00003425"	exon_number "2"
1	lncRNA	exon	186310083	186310413	.	-	.	gene_id "lnc-bbug01426"	transcript_id "lnc-bbut00003429"	exon_number "1"
1	lncRNA	exon	186311123	186311345	.	-	.	gene_id "lnc-bbug01426"	transcript_id "lnc-bbut00003429"	exon_number "2"
1	lncRNA	exon	186320530	186321091	.	-	.	gene_id "lnc-bbug01426"	transcript_id "lnc-bbut00003429"	exon_number "3"
1	lncRNA	exon	186325037	186326203	.	-	.	gene_id "lnc-bbug01427"	transcript_id "lnc-bbut00003430"	exon_number "1"
1	lncRNA	exon	186327161	186327398	.	-	.	gene_id "lnc-bbug01427"	transcript_id "lnc-bbut00003430"	exon_number "2"

1	lncRNA	exon	186331470	186331581	.	-	.	gene_id "lnc-bbug01427"	transcript_id "lnc-bbut00003430"	exon_number "3"
1	lncRNA	exon	186502427	186503203	.	-	.	gene_id "lnc-bbug01429"	transcript_id "lnc-bbut00003432"	exon_number "1"
1	lncRNA	exon	186504391	186504619	.	-	.	gene_id "lnc-bbug01429"	transcript_id "lnc-bbut00003432"	exon_number "2"
1	lncRNA	exon	186507740	186508089	.	-	.	gene_id "lnc-bbug01429"	transcript_id "lnc-bbut00003432"	exon_number "3"
1	lncRNA	exon	187176247	187176438	.	-	.	gene_id "lnc-bbug01436"	transcript_id "lnc-bbut00003445"	exon_number "1"
1	lncRNA	exon	187176871	187177017	.	-	.	gene_id "lnc-bbug01436"	transcript_id "lnc-bbut00003445"	exon_number "2"
1	lncRNA	exon	187180551	187180703	.	-	.	gene_id "lnc-bbug01436"	transcript_id "lnc-bbut00003445"	exon_number "3"
1	lncRNA	exon	188145533	188145846	.	-	.	gene_id "lnc-bbug01444"	transcript_id "lnc-bbut00003461"	exon_number "1"
1	lncRNA	exon	188149003	188149157	.	-	.	gene_id "lnc-bbug01444"	transcript_id "lnc-bbut00003461"	exon_number "2"
1	lncRNA	exon	188149929	188150672	.	-	.	gene_id "lnc-bbug01445"	transcript_id "lnc-bbut00003462"	exon_number "1"
1	lncRNA	exon	188151703	188152198	.	-	.	gene_id "lnc-bbug01445"	transcript_id "lnc-bbut00003462"	exon_number "2"
1	lncRNA	exon	188393810	188394149	.	-	.	gene_id "lnc-bbug01448"	transcript_id "lnc-bbut00003467"	exon_number "1"
1	lncRNA	exon	188394575	188394676	.	-	.	gene_id "lnc-bbug01448"	transcript_id "lnc-bbut00003467"	exon_number "2"
1	lncRNA	exon	188397119	188397339	.	-	.	gene_id "lnc-bbug01448"	transcript_id "lnc-bbut00003467"	exon_number "3"
1	lncRNA	exon	188736096	188736202	.	-	.	gene_id "lnc-bbug01449"	transcript_id "lnc-bbut00003468"	exon_number "1"
1	lncRNA	exon	188737538	188737669	.	-	.	gene_id "lnc-bbug01449"	transcript_id "lnc-bbut00003468"	exon_number "2"
1	lncRNA	exon	188737892	188742227	.	-	.	gene_id "lnc-bbug01449"	transcript_id "lnc-bbut00003468"	exon_number "3"
1	lncRNA	exon	189156625	189156931	.	-	.	gene_id "lnc-bbug01462"	transcript_id "lnc-bbut00003488"	exon_number "1"
1	lncRNA	exon	189250864	189252160	.	-	.	gene_id "lnc-bbug01464"	transcript_id "lnc-bbut00003494"	exon_number "1"
1	lncRNA	exon	189253474	189253545	.	-	.	gene_id "lnc-bbug01464"	transcript_id "lnc-bbut00003494"	exon_number "2"
1	lncRNA	exon	189255648	189255776	.	-	.	gene_id "lnc-bbug01464"	transcript_id "lnc-bbut00003494"	exon_number "3"
1	lncRNA	exon	189261334	189261489	.	-	.	gene_id "lnc-bbug01464"	transcript_id "lnc-bbut00003494"	exon_number "4"
1	lncRNA	exon	189504563	189504752	.	-	.	gene_id "lnc-bbug01467"	transcript_id "lnc-bbut00003498"	exon_number "1"
1	lncRNA	exon	189505115	189505648	.	-	.	gene_id "lnc-bbug01467"	transcript_id "lnc-bbut00003498"	exon_number "2"
1	lncRNA	exon	190851422	190852711	.	-	.	gene_id "lnc-bbug01478"	transcript_id "lnc-bbut00003526"	exon_number "1"
1	lncRNA	exon	190855509	190855646	.	-	.	gene_id "lnc-bbug01478"	transcript_id "lnc-bbut00003526"	exon_number "2"
1	lncRNA	exon	190858752	190858856	.	-	.	gene_id "lnc-bbug01478"	transcript_id "lnc-bbut00003526"	exon_number "3"
1	lncRNA	exon	192464246	192466015	.	-	.	gene_id "lnc-bbug01483"	transcript_id "lnc-bbut00003536"	exon_number "1"
1	lncRNA	exon	192478222	192478408	.	-	.	gene_id "lnc-bbug01483"	transcript_id "lnc-bbut00003536"	exon_number "2"
1	lncRNA	exon	192479515	192479562	.	-	.	gene_id "lnc-bbug01483"	transcript_id "lnc-bbut00003536"	exon_number "3"
1	lncRNA	exon	192479743	192479889	.	-	.	gene_id "lnc-bbug01484"	transcript_id "lnc-bbut00003537"	exon_number "1"
1	lncRNA	exon	192481425	192481538	.	-	.	gene_id "lnc-bbug01484"	transcript_id "lnc-bbut00003537"	exon_number "2"
1	lncRNA	exon	192484842	192485308	.	-	.	gene_id "lnc-bbug01484"	transcript_id "lnc-bbut00003537"	exon_number "3"
1	lncRNA	exon	193159105	193160021	.	-	.	gene_id "lnc-bbug01487"	transcript_id "lnc-bbut00003545"	exon_number "1"
1	lncRNA	exon	193161391	193161554	.	-	.	gene_id "lnc-bbug01487"	transcript_id "lnc-bbut00003545"	exon_number "2"
1	lncRNA	exon	193291841	193293230	.	-	.	gene_id "lnc-bbug01488"	transcript_id "lnc-bbut00003546"	exon_number "1"
1	lncRNA	exon	193346073	193346208	.	-	.	gene_id "lnc-bbug01488"	transcript_id "lnc-bbut00003546"	exon_number "2"
1	lncRNA	exon	193357838	193357968	.	-	.	gene_id "lnc-bbug01488"	transcript_id "lnc-bbut00003546"	exon_number "3"
1	lncRNA	exon	193375334	193375597	.	-	.	gene_id "lnc-bbug01488"	transcript_id "lnc-bbut00003546"	exon_number "4"
1	lncRNA	exon	193341854	193344469	.	-	.	gene_id "lnc-bbug01488"	transcript_id "lnc-bbut00003555"	exon_number "1"
1	lncRNA	exon	193346073	193346208	.	-	.	gene_id "lnc-bbug01488"	transcript_id "lnc-bbut00003555"	exon_number "2"
1	lncRNA	exon	193357838	193357968	.	-	.	gene_id "lnc-bbug01488"	transcript_id "lnc-bbut00003555"	exon_number "3"
1	lncRNA	exon	193375334	193375597	.	-	.	gene_id "lnc-bbug01488"	transcript_id "lnc-bbut00003555"	exon_number "4"
1	lncRNA	exon	193486506	193487164	.	-	.	gene_id "lnc-bbug01489"	transcript_id "lnc-bbut00003556"	exon_number "1"
1	lncRNA	exon	193514944	193515115	.	-	.	gene_id "lnc-bbug01489"	transcript_id "lnc-bbut00003556"	exon_number "2"
1	lncRNA	exon	193527129	193527401	.	-	.	gene_id "lnc-bbug01489"	transcript_id "lnc-bbut00003556"	exon_number "3"
1	lncRNA	exon	193527722	193529907	.	-	.	gene_id "lnc-bbug01489"	transcript_id "lnc-bbut00003556"	exon_number "4"
1	lncRNA	exon	193508306	193508653	.	-	.	gene_id "lnc-bbug01489"	transcript_id "lnc-bbut00003557"	exon_number "1"
1	lncRNA	exon	193510154	193510256	.	-	.	gene_id "lnc-bbug01489"	transcript_id "lnc-bbut00003557"	exon_number "2"
1	lncRNA	exon	193514487	193514615	.	-	.	gene_id "lnc-bbug01489"	transcript_id "lnc-bbut00003557"	exon_number "3"
1	lncRNA	exon	193514944	193515115	.	-	.	gene_id "lnc-bbug01489"	transcript_id "lnc-bbut00003557"	exon_number "4"
1	lncRNA	exon	193516011	193516158	.	-	.	gene_id "lnc-bbug01489"	transcript_id "lnc-bbut00003557"	exon_number "5"

1	lncRNA	exon	193508306	193508653	.	-	.	gene_id "lnc-bbug01489"	transcript_id "lnc-bbut00003558"	exon_number "1"
1	lncRNA	exon	193510154	193510256	.	-	.	gene_id "lnc-bbug01489"	transcript_id "lnc-bbut00003558"	exon_number "2"
1	lncRNA	exon	193514487	193514615	.	-	.	gene_id "lnc-bbug01489"	transcript_id "lnc-bbut00003558"	exon_number "3"
1	lncRNA	exon	193514944	193515115	.	-	.	gene_id "lnc-bbug01489"	transcript_id "lnc-bbut00003558"	exon_number "4"
1	lncRNA	exon	193524822	193526126	.	-	.	gene_id "lnc-bbug01489"	transcript_id "lnc-bbut00003558"	exon_number "5"
1	lncRNA	exon	193508306	193508653	.	-	.	gene_id "lnc-bbug01489"	transcript_id "lnc-bbut00003559"	exon_number "1"
1	lncRNA	exon	193510154	193510256	.	-	.	gene_id "lnc-bbug01489"	transcript_id "lnc-bbut00003559"	exon_number "2"
1	lncRNA	exon	193514487	193514615	.	-	.	gene_id "lnc-bbug01489"	transcript_id "lnc-bbut00003559"	exon_number "3"
1	lncRNA	exon	193514944	193515115	.	-	.	gene_id "lnc-bbug01489"	transcript_id "lnc-bbut00003559"	exon_number "4"
1	lncRNA	exon	193527129	193527524	.	-	.	gene_id "lnc-bbug01489"	transcript_id "lnc-bbut00003559"	exon_number "5"
1	lncRNA	exon	193508306	193508653	.	-	.	gene_id "lnc-bbug01489"	transcript_id "lnc-bbut00003560"	exon_number "1"
1	lncRNA	exon	193514944	193515115	.	-	.	gene_id "lnc-bbug01489"	transcript_id "lnc-bbut00003560"	exon_number "2"
1	lncRNA	exon	193527129	193527401	.	-	.	gene_id "lnc-bbug01489"	transcript_id "lnc-bbut00003560"	exon_number "3"
1	lncRNA	exon	193527722	193529907	.	-	.	gene_id "lnc-bbug01489"	transcript_id "lnc-bbut00003560"	exon_number "4"
1	lncRNA	exon	194287663	194288085	.	-	.	gene_id "lnc-bbug01494"	transcript_id "lnc-bbut00003569"	exon_number "1"
1	lncRNA	exon	194289244	194289290	.	-	.	gene_id "lnc-bbug01494"	transcript_id "lnc-bbut00003569"	exon_number "2"
1	lncRNA	exon	195128923	195128972	.	-	.	gene_id "lnc-bbug01498"	transcript_id "lnc-bbut00003573"	exon_number "1"
1	lncRNA	exon	195129101	195129184	.	-	.	gene_id "lnc-bbug01498"	transcript_id "lnc-bbut00003573"	exon_number "2"
1	lncRNA	exon	195129520	195129705	.	-	.	gene_id "lnc-bbug01498"	transcript_id "lnc-bbut00003573"	exon_number "3"
1	lncRNA	exon	195129815	195129864	.	-	.	gene_id "lnc-bbug01498"	transcript_id "lnc-bbut00003573"	exon_number "4"
1	lncRNA	exon	195130800	195130897	.	-	.	gene_id "lnc-bbug01498"	transcript_id "lnc-bbut00003573"	exon_number "5"
1	lncRNA	exon	195131406	195131566	.	-	.	gene_id "lnc-bbug01498"	transcript_id "lnc-bbut00003573"	exon_number "6"
1	lncRNA	exon	195132770	195133072	.	-	.	gene_id "lnc-bbug01498"	transcript_id "lnc-bbut00003573"	exon_number "7"
1	lncRNA	exon	195128923	195128972	.	-	.	gene_id "lnc-bbug01498"	transcript_id "lnc-bbut00003574"	exon_number "1"
1	lncRNA	exon	195129101	195129184	.	-	.	gene_id "lnc-bbug01498"	transcript_id "lnc-bbut00003574"	exon_number "2"
1	lncRNA	exon	195129520	195129633	.	-	.	gene_id "lnc-bbug01498"	transcript_id "lnc-bbut00003574"	exon_number "3"
1	lncRNA	exon	195129815	195129864	.	-	.	gene_id "lnc-bbug01498"	transcript_id "lnc-bbut00003574"	exon_number "4"
1	lncRNA	exon	195130800	195130897	.	-	.	gene_id "lnc-bbug01498"	transcript_id "lnc-bbut00003574"	exon_number "5"
1	lncRNA	exon	195131406	195131566	.	-	.	gene_id "lnc-bbug01498"	transcript_id "lnc-bbut00003574"	exon_number "6"
1	lncRNA	exon	195132770	195133073	.	-	.	gene_id "lnc-bbug01498"	transcript_id "lnc-bbut00003574"	exon_number "7"
1	lncRNA	exon	195128923	195128972	.	-	.	gene_id "lnc-bbug01498"	transcript_id "lnc-bbut00003575"	exon_number "1"
1	lncRNA	exon	195129101	195129184	.	-	.	gene_id "lnc-bbug01498"	transcript_id "lnc-bbut00003575"	exon_number "2"
1	lncRNA	exon	195129520	195129705	.	-	.	gene_id "lnc-bbug01498"	transcript_id "lnc-bbut00003575"	exon_number "3"
1	lncRNA	exon	195129815	195130897	.	-	.	gene_id "lnc-bbug01498"	transcript_id "lnc-bbut00003575"	exon_number "4"
1	lncRNA	exon	195131406	195131566	.	-	.	gene_id "lnc-bbug01498"	transcript_id "lnc-bbut00003575"	exon_number "5"
1	lncRNA	exon	195132770	195133083	.	-	.	gene_id "lnc-bbug01498"	transcript_id "lnc-bbut00003575"	exon_number "6"
1	lncRNA	exon	195220961	195221209	.	-	.	gene_id "lnc-bbug01499"	transcript_id "lnc-bbut00003576"	exon_number "1"
1	lncRNA	exon	195223052	195223188	.	-	.	gene_id "lnc-bbug01499"	transcript_id "lnc-bbut00003576"	exon_number "2"
1	lncRNA	exon	195231713	195231926	.	-	.	gene_id "lnc-bbug01499"	transcript_id "lnc-bbut00003576"	exon_number "3"
1	lncRNA	exon	196017884	196018299	.	-	.	gene_id "lnc-bbug01501"	transcript_id "lnc-bbut00003582"	exon_number "1"
1	lncRNA	exon	196018390	196018599	.	-	.	gene_id "lnc-bbug01501"	transcript_id "lnc-bbut00003582"	exon_number "2"
1	lncRNA	exon	196033359	196034528	.	-	.	gene_id "lnc-bbug01501"	transcript_id "lnc-bbut00003582"	exon_number "3"
1	lncRNA	exon	196660059	196660103	.	-	.	gene_id "lnc-bbug01505"	transcript_id "lnc-bbut00003588"	exon_number "1"
1	lncRNA	exon	196660250	196660315	.	-	.	gene_id "lnc-bbug01505"	transcript_id "lnc-bbut00003588"	exon_number "2"
1	lncRNA	exon	196663142	196663768	.	-	.	gene_id "lnc-bbug01505"	transcript_id "lnc-bbut00003588"	exon_number "3"
1	lncRNA	exon	196727614	196727840	.	-	.	gene_id "lnc-bbug01506"	transcript_id "lnc-bbut00003589"	exon_number "1"
1	lncRNA	exon	196742648	196742789	.	-	.	gene_id "lnc-bbug01506"	transcript_id "lnc-bbut00003589"	exon_number "2"
1	lncRNA	exon	197561671	197561991	.	-	.	gene_id "lnc-bbug01512"	transcript_id "lnc-bbut00003608"	exon_number "1"
1	lncRNA	exon	197564949	197565394	.	-	.	gene_id "lnc-bbug01512"	transcript_id "lnc-bbut00003608"	exon_number "2"
1	lncRNA	exon	197567893	197568030	.	-	.	gene_id "lnc-bbug01512"	transcript_id "lnc-bbut00003608"	exon_number "3"
1	lncRNA	exon	197570189	197571542	.	-	.	gene_id "lnc-bbug01512"	transcript_id "lnc-bbut00003608"	exon_number "4"
1	lncRNA	exon	197807910	197810580	.	-	.	gene_id "lnc-bbug01513"	transcript_id "lnc-bbut00003609"	exon_number "1"

1	lncRNA	exon	197812778	197812852	.	-	.	gene_id "lnc-bbug01513"	transcript_id "lnc-bbut00003609"	exon_number "2"
1	lncRNA	exon	197813830	197814086	.	-	.	gene_id "lnc-bbug01513"	transcript_id "lnc-bbut00003609"	exon_number "3"
1	lncRNA	exon	197807910	197810580	.	-	.	gene_id "lnc-bbug01513"	transcript_id "lnc-bbut00003610"	exon_number "1"
1	lncRNA	exon	197813830	197814088	.	-	.	gene_id "lnc-bbug01513"	transcript_id "lnc-bbut00003610"	exon_number "2"
1	lncRNA	exon	200849992	200850371	.	-	.	gene_id "lnc-bbug01518"	transcript_id "lnc-bbut00003639"	exon_number "1"
1	lncRNA	exon	200851261	200851538	.	-	.	gene_id "lnc-bbug01518"	transcript_id "lnc-bbut00003639"	exon_number "2"
1	lncRNA	exon	200851782	200852026	.	-	.	gene_id "lnc-bbug01518"	transcript_id "lnc-bbut00003639"	exon_number "3"
1	lncRNA	exon	201525033	201525296	.	-	.	gene_id "lnc-bbug01519"	transcript_id "lnc-bbut00003640"	exon_number "1"
1	lncRNA	exon	201531394	201531496	.	-	.	gene_id "lnc-bbug01519"	transcript_id "lnc-bbut00003640"	exon_number "2"
1	lncRNA	exon	201537329	201537457	.	-	.	gene_id "lnc-bbug01519"	transcript_id "lnc-bbut00003640"	exon_number "3"
1	lncRNA	exon	201720019	201721204	.	-	.	gene_id "lnc-bbug01521"	transcript_id "lnc-bbut00003646"	exon_number "1"
1	lncRNA	exon	201721498	201721692	.	-	.	gene_id "lnc-bbug01521"	transcript_id "lnc-bbut00003646"	exon_number "2"
1	lncRNA	exon	201953551	201954566	.	-	.	gene_id "lnc-bbug01524"	transcript_id "lnc-bbut00003651"	exon_number "1"
1	lncRNA	exon	201962306	201962410	.	-	.	gene_id "lnc-bbug01524"	transcript_id "lnc-bbut00003651"	exon_number "2"
10	lncRNA	exon	177229	177369	.	+	.	gene_id "lnc-bbug01530"	transcript_id "lnc-bbut00003658"	exon_number "1"
10	lncRNA	exon	179230	179800	.	+	.	gene_id "lnc-bbug01530"	transcript_id "lnc-bbut00003658"	exon_number "2"
10	lncRNA	exon	226313	226655	.	+	.	gene_id "lnc-bbug01532"	transcript_id "lnc-bbut00003660"	exon_number "1"
10	lncRNA	exon	244221	244429	.	+	.	gene_id "lnc-bbug01532"	transcript_id "lnc-bbut00003660"	exon_number "2"
10	lncRNA	exon	244718	248318	.	+	.	gene_id "lnc-bbug01532"	transcript_id "lnc-bbut00003660"	exon_number "3"
10	lncRNA	exon	233114	233688	.	+	.	gene_id "lnc-bbug01533"	transcript_id "lnc-bbut00003661"	exon_number "1"
10	lncRNA	exon	235170	238833	.	+	.	gene_id "lnc-bbug01533"	transcript_id "lnc-bbut00003661"	exon_number "2"
10	lncRNA	exon	248391	248927	.	+	.	gene_id "lnc-bbug01534"	transcript_id "lnc-bbut00003662"	exon_number "1"
10	lncRNA	exon	249926	252193	.	+	.	gene_id "lnc-bbug01534"	transcript_id "lnc-bbut00003662"	exon_number "2"
10	lncRNA	exon	345426	345538	.	+	.	gene_id "lnc-bbug01535"	transcript_id "lnc-bbut00003663"	exon_number "1"
10	lncRNA	exon	346217	346799	.	+	.	gene_id "lnc-bbug01535"	transcript_id "lnc-bbut00003663"	exon_number "2"
10	lncRNA	exon	346994	347599	.	+	.	gene_id "lnc-bbug01535"	transcript_id "lnc-bbut00003663"	exon_number "3"
10	lncRNA	exon	392814	392951	.	+	.	gene_id "lnc-bbug01536"	transcript_id "lnc-bbut00003664"	exon_number "1"
10	lncRNA	exon	394094	394226	.	+	.	gene_id "lnc-bbug01536"	transcript_id "lnc-bbut00003664"	exon_number "2"
10	lncRNA	exon	399298	400759	.	+	.	gene_id "lnc-bbug01536"	transcript_id "lnc-bbut00003664"	exon_number "3"
10	lncRNA	exon	570425	571244	.	+	.	gene_id "lnc-bbug01537"	transcript_id "lnc-bbut00003667"	exon_number "1"
10	lncRNA	exon	585673	585786	.	+	.	gene_id "lnc-bbug01537"	transcript_id "lnc-bbut00003667"	exon_number "2"
10	lncRNA	exon	661446	661595	.	+	.	gene_id "lnc-bbug01540"	transcript_id "lnc-bbut00003671"	exon_number "1"
10	lncRNA	exon	663012	663101	.	+	.	gene_id "lnc-bbug01540"	transcript_id "lnc-bbut00003671"	exon_number "2"
10	lncRNA	exon	666128	666140	.	+	.	gene_id "lnc-bbug01540"	transcript_id "lnc-bbut00003671"	exon_number "3"
10	lncRNA	exon	674507	674562	.	+	.	gene_id "lnc-bbug01541"	transcript_id "lnc-bbut00003672"	exon_number "1"
10	lncRNA	exon	687127	688551	.	+	.	gene_id "lnc-bbug01541"	transcript_id "lnc-bbut00003672"	exon_number "2"
10	lncRNA	exon	753373	756814	.	+	.	gene_id "lnc-bbug01542"	transcript_id "lnc-bbut00003674"	exon_number "1"
10	lncRNA	exon	760160	760776	.	+	.	gene_id "lnc-bbug01542"	transcript_id "lnc-bbut00003674"	exon_number "2"
10	lncRNA	exon	2020690	2020864	.	+	.	gene_id "lnc-bbug01549"	transcript_id "lnc-bbut00003690"	exon_number "1"
10	lncRNA	exon	2021053	2021929	.	+	.	gene_id "lnc-bbug01549"	transcript_id "lnc-bbut00003690"	exon_number "2"
10	lncRNA	exon	2106551	2106890	.	+	.	gene_id "lnc-bbug01551"	transcript_id "lnc-bbut00003693"	exon_number "1"
10	lncRNA	exon	2109315	2109560	.	+	.	gene_id "lnc-bbug01551"	transcript_id "lnc-bbut00003693"	exon_number "2"
10	lncRNA	exon	2109913	2110432	.	+	.	gene_id "lnc-bbug01551"	transcript_id "lnc-bbut00003693"	exon_number "3"
10	lncRNA	exon	2597278	2597456	.	+	.	gene_id "lnc-bbug01555"	transcript_id "lnc-bbut00003704"	exon_number "1"
10	lncRNA	exon	2605382	2605385	.	+	.	gene_id "lnc-bbug01555"	transcript_id "lnc-bbut00003704"	exon_number "2"
10	lncRNA	exon	2607551	2607647	.	+	.	gene_id "lnc-bbug01555"	transcript_id "lnc-bbut00003704"	exon_number "3"
10	lncRNA	exon	2608516	2608648	.	+	.	gene_id "lnc-bbug01555"	transcript_id "lnc-bbut00003704"	exon_number "4"
10	lncRNA	exon	2609000	2609530	.	+	.	gene_id "lnc-bbug01555"	transcript_id "lnc-bbut00003704"	exon_number "5"
10	lncRNA	exon	2715685	2715877	.	+	.	gene_id "lnc-bbug01558"	transcript_id "lnc-bbut00003708"	exon_number "1"
10	lncRNA	exon	2755063	2755210	.	+	.	gene_id "lnc-bbug01558"	transcript_id "lnc-bbut00003708"	exon_number "2"
10	lncRNA	exon	2756341	2756542	.	+	.	gene_id "lnc-bbug01558"	transcript_id "lnc-bbut00003708"	exon_number "3"
10	lncRNA	exon	2773630	2774061	.	+	.	gene_id "lnc-bbug01559"	transcript_id "lnc-bbut00003709"	exon_number "1"

10	lncRNA	exon	2776575	2776675	.	+	.	gene_id "lnc-bbug01559"	transcript_id "lnc-bbut00003709"	exon_number "2"
10	lncRNA	exon	2785334	2785394	.	+	.	gene_id "lnc-bbug01559"	transcript_id "lnc-bbut00003709"	exon_number "3"
10	lncRNA	exon	2785511	2786233	.	+	.	gene_id "lnc-bbug01559"	transcript_id "lnc-bbut00003709"	exon_number "4"
10	lncRNA	exon	2788487	2788751	.	+	.	gene_id "lnc-bbug01559"	transcript_id "lnc-bbut00003709"	exon_number "5"
10	lncRNA	exon	2809476	2809738	.	+	.	gene_id "lnc-bbug01560"	transcript_id "lnc-bbut00003712"	exon_number "1"
10	lncRNA	exon	2810420	2810596	.	+	.	gene_id "lnc-bbug01560"	transcript_id "lnc-bbut00003712"	exon_number "2"
10	lncRNA	exon	3452065	3453493	.	+	.	gene_id "lnc-bbug01562"	transcript_id "lnc-bbut00003720"	exon_number "1"
10	lncRNA	exon	3453557	3456331	.	+	.	gene_id "lnc-bbug01562"	transcript_id "lnc-bbut00003720"	exon_number "2"
10	lncRNA	exon	5235376	5235908	.	+	.	gene_id "lnc-bbug01564"	transcript_id "lnc-bbut00003725"	exon_number "1"
10	lncRNA	exon	5236074	5238060	.	+	.	gene_id "lnc-bbug01564"	transcript_id "lnc-bbut00003725"	exon_number "2"
10	lncRNA	exon	5239447	5241007	.	+	.	gene_id "lnc-bbug01564"	transcript_id "lnc-bbut00003725"	exon_number "3"
10	lncRNA	exon	7544660	7544910	.	+	.	gene_id "lnc-bbug01569"	transcript_id "lnc-bbut00003739"	exon_number "1"
10	lncRNA	exon	7545206	7545553	.	+	.	gene_id "lnc-bbug01569"	transcript_id "lnc-bbut00003739"	exon_number "2"
10	lncRNA	exon	7547529	7547577	.	+	.	gene_id "lnc-bbug01569"	transcript_id "lnc-bbut00003739"	exon_number "3"
10	lncRNA	exon	7840559	7841067	.	+	.	gene_id "lnc-bbug01571"	transcript_id "lnc-bbut00003744"	exon_number "1"
10	lncRNA	exon	7851327	7851524	.	+	.	gene_id "lnc-bbug01571"	transcript_id "lnc-bbut00003744"	exon_number "2"
10	lncRNA	exon	7853274	7853734	.	+	.	gene_id "lnc-bbug01571"	transcript_id "lnc-bbut00003744"	exon_number "3"
10	lncRNA	exon	9022809	9023388	.	+	.	gene_id "lnc-bbug01581"	transcript_id "lnc-bbut00003756"	exon_number "1"
10	lncRNA	exon	9023776	9024044	.	+	.	gene_id "lnc-bbug01581"	transcript_id "lnc-bbut00003756"	exon_number "2"
10	lncRNA	exon	9252400	9252639	.	+	.	gene_id "lnc-bbug01582"	transcript_id "lnc-bbut00003757"	exon_number "1"
10	lncRNA	exon	9480481	9481702	.	+	.	gene_id "lnc-bbug01584"	transcript_id "lnc-bbut00003762"	exon_number "1"
10	lncRNA	exon	9481923	9482040	.	+	.	gene_id "lnc-bbug01584"	transcript_id "lnc-bbut00003762"	exon_number "2"
10	lncRNA	exon	9493141	9493244	.	+	.	gene_id "lnc-bbug01584"	transcript_id "lnc-bbut00003762"	exon_number "3"
10	lncRNA	exon	9496826	9497070	.	+	.	gene_id "lnc-bbug01584"	transcript_id "lnc-bbut00003762"	exon_number "4"
10	lncRNA	exon	9497203	9497379	.	+	.	gene_id "lnc-bbug01585"	transcript_id "lnc-bbut00003763"	exon_number "1"
10	lncRNA	exon	9501927	9502188	.	+	.	gene_id "lnc-bbug01585"	transcript_id "lnc-bbut00003763"	exon_number "2"
10	lncRNA	exon	10167975	10169078	.	+	.	gene_id "lnc-bbug01588"	transcript_id "lnc-bbut00003766"	exon_number "1"
10	lncRNA	exon	10169563	10169942	.	+	.	gene_id "lnc-bbug01588"	transcript_id "lnc-bbut00003766"	exon_number "2"
10	lncRNA	exon	10659930	10660041	.	+	.	gene_id "lnc-bbug01589"	transcript_id "lnc-bbut00003767"	exon_number "1"
10	lncRNA	exon	10660218	10661801	.	+	.	gene_id "lnc-bbug01589"	transcript_id "lnc-bbut00003767"	exon_number "2"
10	lncRNA	exon	10856116	10856580	.	+	.	gene_id "lnc-bbug01590"	transcript_id "lnc-bbut00003768"	exon_number "1"
10	lncRNA	exon	10862067	10862198	.	+	.	gene_id "lnc-bbug01590"	transcript_id "lnc-bbut00003768"	exon_number "2"
10	lncRNA	exon	11013600	11014357	.	+	.	gene_id "lnc-bbug01590"	transcript_id "lnc-bbut00003768"	exon_number "3"
10	lncRNA	exon	11067538	11067589	.	+	.	gene_id "lnc-bbug01591"	transcript_id "lnc-bbut00003769"	exon_number "1"
10	lncRNA	exon	11074109	11074450	.	+	.	gene_id "lnc-bbug01591"	transcript_id "lnc-bbut00003769"	exon_number "2"
10	lncRNA	exon	11081104	11081212	.	+	.	gene_id "lnc-bbug01591"	transcript_id "lnc-bbut00003769"	exon_number "3"
10	lncRNA	exon	11094794	11094887	.	+	.	gene_id "lnc-bbug01591"	transcript_id "lnc-bbut00003769"	exon_number "4"
10	lncRNA	exon	11139320	11139512	.	+	.	gene_id "lnc-bbug01591"	transcript_id "lnc-bbut00003769"	exon_number "5"
10	lncRNA	exon	11141911	11145237	.	+	.	gene_id "lnc-bbug01591"	transcript_id "lnc-bbut00003769"	exon_number "6"
10	lncRNA	exon	11069150	11069273	.	+	.	gene_id "lnc-bbug01591"	transcript_id "lnc-bbut00003770"	exon_number "1"
10	lncRNA	exon	11074109	11074450	.	+	.	gene_id "lnc-bbug01591"	transcript_id "lnc-bbut00003770"	exon_number "2"
10	lncRNA	exon	11081104	11081212	.	+	.	gene_id "lnc-bbug01591"	transcript_id "lnc-bbut00003770"	exon_number "3"
10	lncRNA	exon	11094794	11094887	.	+	.	gene_id "lnc-bbug01591"	transcript_id "lnc-bbut00003770"	exon_number "4"
10	lncRNA	exon	11097717	11097761	.	+	.	gene_id "lnc-bbug01591"	transcript_id "lnc-bbut00003770"	exon_number "5"
10	lncRNA	exon	11069150	11069273	.	+	.	gene_id "lnc-bbug01591"	transcript_id "lnc-bbut00003772"	exon_number "1"
10	lncRNA	exon	11074109	11074450	.	+	.	gene_id "lnc-bbug01591"	transcript_id "lnc-bbut00003772"	exon_number "2"
10	lncRNA	exon	11081104	11081212	.	+	.	gene_id "lnc-bbug01591"	transcript_id "lnc-bbut00003772"	exon_number "3"
10	lncRNA	exon	11094794	11094887	.	+	.	gene_id "lnc-bbug01591"	transcript_id "lnc-bbut00003772"	exon_number "4"
10	lncRNA	exon	11139320	11139512	.	+	.	gene_id "lnc-bbug01591"	transcript_id "lnc-bbut00003772"	exon_number "5"
10	lncRNA	exon	11141911	11145237	.	+	.	gene_id "lnc-bbug01591"	transcript_id "lnc-bbut00003772"	exon_number "6"
10	lncRNA	exon	11069150	11069273	.	+	.	gene_id "lnc-bbug01591"	transcript_id "lnc-bbut00003771"	exon_number "1"
10	lncRNA	exon	11074109	11074450	.	+	.	gene_id "lnc-bbug01591"	transcript_id "lnc-bbut00003771"	exon_number "2"

10	lncRNA	exon	11081104	11081212	.	+	.	gene_id "lnc-bbug01591"	transcript_id "lnc-bbut00003771"	exon_number "3"
10	lncRNA	exon	11094794	11094887	.	+	.	gene_id "lnc-bbug01591"	transcript_id "lnc-bbut00003771"	exon_number "4"
10	lncRNA	exon	11141911	11145237	.	+	.	gene_id "lnc-bbug01591"	transcript_id "lnc-bbut00003771"	exon_number "5"
10	lncRNA	exon	11069150	11069273	.	+	.	gene_id "lnc-bbug01591"	transcript_id "lnc-bbut00003773"	exon_number "1"
10	lncRNA	exon	11074109	11074450	.	+	.	gene_id "lnc-bbug01591"	transcript_id "lnc-bbut00003773"	exon_number "2"
10	lncRNA	exon	11081104	11081212	.	+	.	gene_id "lnc-bbug01591"	transcript_id "lnc-bbut00003773"	exon_number "3"
10	lncRNA	exon	11094794	11094887	.	+	.	gene_id "lnc-bbug01591"	transcript_id "lnc-bbut00003773"	exon_number "4"
10	lncRNA	exon	11190281	11191582	.	+	.	gene_id "lnc-bbug01591"	transcript_id "lnc-bbut00003773"	exon_number "5"
10	lncRNA	exon	12650196	12650463	.	+	.	gene_id "lnc-bbug01596"	transcript_id "lnc-bbut00003780"	exon_number "1"
10	lncRNA	exon	12653926	12654057	.	+	.	gene_id "lnc-bbug01596"	transcript_id "lnc-bbut00003780"	exon_number "2"
10	lncRNA	exon	12669726	12670990	.	+	.	gene_id "lnc-bbug01596"	transcript_id "lnc-bbut00003780"	exon_number "3"
10	lncRNA	exon	12671645	12672229	.	+	.	gene_id "lnc-bbug01596"	transcript_id "lnc-bbut00003780"	exon_number "4"
10	lncRNA	exon	12650197	12650463	.	+	.	gene_id "lnc-bbug01596"	transcript_id "lnc-bbut00003781"	exon_number "1"
10	lncRNA	exon	12653926	12654034	.	+	.	gene_id "lnc-bbug01596"	transcript_id "lnc-bbut00003781"	exon_number "2"
10	lncRNA	exon	12669726	12670990	.	+	.	gene_id "lnc-bbug01596"	transcript_id "lnc-bbut00003781"	exon_number "3"
10	lncRNA	exon	12671645	12672229	.	+	.	gene_id "lnc-bbug01596"	transcript_id "lnc-bbut00003781"	exon_number "4"
10	lncRNA	exon	12650206	12650463	.	+	.	gene_id "lnc-bbug01596"	transcript_id "lnc-bbut00003782"	exon_number "1"
10	lncRNA	exon	12653926	12654057	.	+	.	gene_id "lnc-bbug01596"	transcript_id "lnc-bbut00003782"	exon_number "2"
10	lncRNA	exon	12669726	12669951	.	+	.	gene_id "lnc-bbug01596"	transcript_id "lnc-bbut00003782"	exon_number "3"
10	lncRNA	exon	12670932	12670990	.	+	.	gene_id "lnc-bbug01596"	transcript_id "lnc-bbut00003782"	exon_number "4"
10	lncRNA	exon	12671645	12672229	.	+	.	gene_id "lnc-bbug01596"	transcript_id "lnc-bbut00003782"	exon_number "5"
10	lncRNA	exon	12753216	12753455	.	+	.	gene_id "lnc-bbug01597"	transcript_id "lnc-bbut00003783"	exon_number "1"
10	lncRNA	exon	12753572	12753671	.	+	.	gene_id "lnc-bbug01597"	transcript_id "lnc-bbut00003783"	exon_number "2"
10	lncRNA	exon	12783329	12783462	.	+	.	gene_id "lnc-bbug01597"	transcript_id "lnc-bbut00003783"	exon_number "3"
10	lncRNA	exon	12783621	12784475	.	+	.	gene_id "lnc-bbug01597"	transcript_id "lnc-bbut00003783"	exon_number "4"
10	lncRNA	exon	13845396	13847468	.	+	.	gene_id "lnc-bbug01601"	transcript_id "lnc-bbut00003796"	exon_number "1"
10	lncRNA	exon	16031214	16031261	.	+	.	gene_id "lnc-bbug01609"	transcript_id "lnc-bbut00003816"	exon_number "1"
10	lncRNA	exon	16032022	16032136	.	+	.	gene_id "lnc-bbug01609"	transcript_id "lnc-bbut00003816"	exon_number "2"
10	lncRNA	exon	16032282	16032673	.	+	.	gene_id "lnc-bbug01609"	transcript_id "lnc-bbut00003816"	exon_number "3"
10	lncRNA	exon	16561075	16561438	.	+	.	gene_id "lnc-bbug01610"	transcript_id "lnc-bbut00003817"	exon_number "1"
10	lncRNA	exon	16566248	16566407	.	+	.	gene_id "lnc-bbug01610"	transcript_id "lnc-bbut00003817"	exon_number "2"
10	lncRNA	exon	16566750	16566995	.	+	.	gene_id "lnc-bbug01610"	transcript_id "lnc-bbut00003817"	exon_number "3"
10	lncRNA	exon	16716566	16716828	.	+	.	gene_id "lnc-bbug01611"	transcript_id "lnc-bbut00003818"	exon_number "1"
10	lncRNA	exon	16717019	16717123	.	+	.	gene_id "lnc-bbug01611"	transcript_id "lnc-bbut00003818"	exon_number "2"
10	lncRNA	exon	16718779	16719040	.	+	.	gene_id "lnc-bbug01611"	transcript_id "lnc-bbut00003818"	exon_number "3"
10	lncRNA	exon	16911325	16915557	.	+	.	gene_id "lnc-bbug01613"	transcript_id "lnc-bbut00003822"	exon_number "1"
10	lncRNA	exon	16999122	16999849	.	+	.	gene_id "lnc-bbug01616"	transcript_id "lnc-bbut00003826"	exon_number "1"
10	lncRNA	exon	17000082	17000344	.	+	.	gene_id "lnc-bbug01616"	transcript_id "lnc-bbut00003826"	exon_number "2"
10	lncRNA	exon	17000563	17001256	.	+	.	gene_id "lnc-bbug01616"	transcript_id "lnc-bbut00003826"	exon_number "3"
10	lncRNA	exon	17071269	17071379	.	+	.	gene_id "lnc-bbug01617"	transcript_id "lnc-bbut00003827"	exon_number "1"
10	lncRNA	exon	17080257	17080347	.	+	.	gene_id "lnc-bbug01617"	transcript_id "lnc-bbut00003827"	exon_number "2"
10	lncRNA	exon	17081765	17081906	.	+	.	gene_id "lnc-bbug01617"	transcript_id "lnc-bbut00003827"	exon_number "3"
10	lncRNA	exon	19575550	19575655	.	+	.	gene_id "lnc-bbug01630"	transcript_id "lnc-bbut00003858"	exon_number "1"
10	lncRNA	exon	19578189	19578235	.	+	.	gene_id "lnc-bbug01630"	transcript_id "lnc-bbut00003858"	exon_number "2"
10	lncRNA	exon	19579895	19581957	.	+	.	gene_id "lnc-bbug01630"	transcript_id "lnc-bbut00003858"	exon_number "3"
10	lncRNA	exon	19575550	19575655	.	+	.	gene_id "lnc-bbug01630"	transcript_id "lnc-bbut00003859"	exon_number "1"
10	lncRNA	exon	19578189	19578235	.	+	.	gene_id "lnc-bbug01630"	transcript_id "lnc-bbut00003859"	exon_number "2"
10	lncRNA	exon	19590458	19590989	.	+	.	gene_id "lnc-bbug01630"	transcript_id "lnc-bbut00003859"	exon_number "3"
10	lncRNA	exon	21575468	21575730	.	+	.	gene_id "lnc-bbug01636"	transcript_id "lnc-bbut00003867"	exon_number "1"
10	lncRNA	exon	21576143	21578702	.	+	.	gene_id "lnc-bbug01636"	transcript_id "lnc-bbut00003867"	exon_number "2"
10	lncRNA	exon	22644809	22646523	.	+	.	gene_id "lnc-bbug01637"	transcript_id "lnc-bbut00003868"	exon_number "1"
10	lncRNA	exon	22646867	22647794	.	+	.	gene_id "lnc-bbug01637"	transcript_id "lnc-bbut00003868"	exon_number "2"

10	lncRNA	exon	22752778	22753175	.	+	.	gene_id "lnc-bbug01638"	transcript_id "lnc-bbut00003869"	exon_number "1"
10	lncRNA	exon	22753278	22753508	.	+	.	gene_id "lnc-bbug01638"	transcript_id "lnc-bbut00003869"	exon_number "2"
10	lncRNA	exon	22752779	22753171	.	+	.	gene_id "lnc-bbug01638"	transcript_id "lnc-bbut00003870"	exon_number "1"
10	lncRNA	exon	22753278	22753508	.	+	.	gene_id "lnc-bbug01638"	transcript_id "lnc-bbut00003870"	exon_number "2"
10	lncRNA	exon	26862694	26864206	.	+	.	gene_id "lnc-bbug01644"	transcript_id "lnc-bbut00003901"	exon_number "1"
10	lncRNA	exon	26866533	26866763	.	+	.	gene_id "lnc-bbug01644"	transcript_id "lnc-bbut00003901"	exon_number "2"
10	lncRNA	exon	26912048	26913065	.	+	.	gene_id "lnc-bbug01645"	transcript_id "lnc-bbut00003902"	exon_number "1"
10	lncRNA	exon	26936692	26936768	.	+	.	gene_id "lnc-bbug01645"	transcript_id "lnc-bbut00003902"	exon_number "2"
10	lncRNA	exon	26965375	26965876	.	+	.	gene_id "lnc-bbug01645"	transcript_id "lnc-bbut00003902"	exon_number "3"
10	lncRNA	exon	27037525	27038999	.	+	.	gene_id "lnc-bbug01646"	transcript_id "lnc-bbut00003903"	exon_number "1"
10	lncRNA	exon	27821160	27821263	.	+	.	gene_id "lnc-bbug01650"	transcript_id "lnc-bbut00003915"	exon_number "1"
10	lncRNA	exon	27833499	27834419	.	+	.	gene_id "lnc-bbug01650"	transcript_id "lnc-bbut00003915"	exon_number "2"
10	lncRNA	exon	27821160	27821263	.	+	.	gene_id "lnc-bbug01650"	transcript_id "lnc-bbut00003916"	exon_number "1"
10	lncRNA	exon	27833499	27833629	.	+	.	gene_id "lnc-bbug01650"	transcript_id "lnc-bbut00003916"	exon_number "2"
10	lncRNA	exon	27840794	27840899	.	+	.	gene_id "lnc-bbug01650"	transcript_id "lnc-bbut00003916"	exon_number "3"
10	lncRNA	exon	27845980	27846730	.	+	.	gene_id "lnc-bbug01650"	transcript_id "lnc-bbut00003916"	exon_number "4"
10	lncRNA	exon	28343365	28344053	.	+	.	gene_id "lnc-bbug01653"	transcript_id "lnc-bbut00003919"	exon_number "1"
10	lncRNA	exon	28348273	28348383	.	+	.	gene_id "lnc-bbug01653"	transcript_id "lnc-bbut00003919"	exon_number "2"
10	lncRNA	exon	28350163	28350287	.	+	.	gene_id "lnc-bbug01653"	transcript_id "lnc-bbut00003919"	exon_number "3"
10	lncRNA	exon	28352259	28353310	.	+	.	gene_id "lnc-bbug01653"	transcript_id "lnc-bbut00003919"	exon_number "4"
10	lncRNA	exon	28343372	28344053	.	+	.	gene_id "lnc-bbug01653"	transcript_id "lnc-bbut00003920"	exon_number "1"
10	lncRNA	exon	28348273	28348383	.	+	.	gene_id "lnc-bbug01653"	transcript_id "lnc-bbut00003920"	exon_number "2"
10	lncRNA	exon	28352259	28353310	.	+	.	gene_id "lnc-bbug01653"	transcript_id "lnc-bbut00003920"	exon_number "3"
10	lncRNA	exon	28540977	28542070	.	+	.	gene_id "lnc-bbug01655"	transcript_id "lnc-bbut00003922"	exon_number "1"
10	lncRNA	exon	28544122	28544150	.	+	.	gene_id "lnc-bbug01655"	transcript_id "lnc-bbut00003922"	exon_number "2"
10	lncRNA	exon	28568852	28569222	.	+	.	gene_id "lnc-bbug01655"	transcript_id "lnc-bbut00003922"	exon_number "3"
10	lncRNA	exon	28662814	28662900	.	+	.	gene_id "lnc-bbug01656"	transcript_id "lnc-bbut00003923"	exon_number "1"
10	lncRNA	exon	28686599	28686740	.	+	.	gene_id "lnc-bbug01656"	transcript_id "lnc-bbut00003923"	exon_number "2"
10	lncRNA	exon	28693661	28695106	.	+	.	gene_id "lnc-bbug01656"	transcript_id "lnc-bbut00003923"	exon_number "3"
10	lncRNA	exon	28831971	28832726	.	+	.	gene_id "lnc-bbug01658"	transcript_id "lnc-bbut00003925"	exon_number "1"
10	lncRNA	exon	28832853	28833273	.	+	.	gene_id "lnc-bbug01658"	transcript_id "lnc-bbut00003925"	exon_number "2"
10	lncRNA	exon	31221455	31223217	.	+	.	gene_id "lnc-bbug01670"	transcript_id "lnc-bbut00003979"	exon_number "1"
10	lncRNA	exon	31256877	31256980	.	+	.	gene_id "lnc-bbug01670"	transcript_id "lnc-bbut00003979"	exon_number "2"
10	lncRNA	exon	31275660	31275785	.	+	.	gene_id "lnc-bbug01670"	transcript_id "lnc-bbut00003979"	exon_number "3"
10	lncRNA	exon	31277505	31277575	.	+	.	gene_id "lnc-bbug01670"	transcript_id "lnc-bbut00003979"	exon_number "4"
10	lncRNA	exon	31285016	31285097	.	+	.	gene_id "lnc-bbug01670"	transcript_id "lnc-bbut00003979"	exon_number "5"
10	lncRNA	exon	31285804	31287120	.	+	.	gene_id "lnc-bbug01670"	transcript_id "lnc-bbut00003979"	exon_number "6"
10	lncRNA	exon	31221459	31223217	.	+	.	gene_id "lnc-bbug01670"	transcript_id "lnc-bbut00003980"	exon_number "1"
10	lncRNA	exon	31256877	31256980	.	+	.	gene_id "lnc-bbug01670"	transcript_id "lnc-bbut00003980"	exon_number "2"
10	lncRNA	exon	31275660	31275785	.	+	.	gene_id "lnc-bbug01670"	transcript_id "lnc-bbut00003980"	exon_number "3"
10	lncRNA	exon	31277505	31277575	.	+	.	gene_id "lnc-bbug01670"	transcript_id "lnc-bbut00003980"	exon_number "4"
10	lncRNA	exon	31285016	31287120	.	+	.	gene_id "lnc-bbug01670"	transcript_id "lnc-bbut00003980"	exon_number "5"
10	lncRNA	exon	32532384	32532452	.	+	.	gene_id "lnc-bbug01675"	transcript_id "lnc-bbut00003990"	exon_number "1"
10	lncRNA	exon	32533620	32533750	.	+	.	gene_id "lnc-bbug01675"	transcript_id "lnc-bbut00003990"	exon_number "2"
10	lncRNA	exon	32537702	32537810	.	+	.	gene_id "lnc-bbug01675"	transcript_id "lnc-bbut00003990"	exon_number "3"
10	lncRNA	exon	34633364	34633488	.	+	.	gene_id "lnc-bbug01699"	transcript_id "lnc-bbut00004025"	exon_number "1"
10	lncRNA	exon	34703732	34704798	.	+	.	gene_id "lnc-bbug01699"	transcript_id "lnc-bbut00004025"	exon_number "2"
10	lncRNA	exon	34713516	34713668	.	+	.	gene_id "lnc-bbug01699"	transcript_id "lnc-bbut00004025"	exon_number "3"
10	lncRNA	exon	34714187	34715945	.	+	.	gene_id "lnc-bbug01699"	transcript_id "lnc-bbut00004025"	exon_number "4"
10	lncRNA	exon	34633386	34633488	.	+	.	gene_id "lnc-bbug01699"	transcript_id "lnc-bbut00004027"	exon_number "1"
10	lncRNA	exon	34700916	34701028	.	+	.	gene_id "lnc-bbug01699"	transcript_id "lnc-bbut00004027"	exon_number "2"
10	lncRNA	exon	34703732	34703850	.	+	.	gene_id "lnc-bbug01699"	transcript_id "lnc-bbut00004027"	exon_number "3"

10	lncRNA	exon	34704716	34704798	.	+	.	gene_id "lnc-bbug01699"	transcript_id "lnc-bbut00004027"	exon_number "4"
10	lncRNA	exon	34713516	34713668	.	+	.	gene_id "lnc-bbug01699"	transcript_id "lnc-bbut00004027"	exon_number "5"
10	lncRNA	exon	34714187	34715945	.	+	.	gene_id "lnc-bbug01699"	transcript_id "lnc-bbut00004027"	exon_number "6"
10	lncRNA	exon	34633386	34633488	.	+	.	gene_id "lnc-bbug01699"	transcript_id "lnc-bbut00004026"	exon_number "1"
10	lncRNA	exon	34700916	34701028	.	+	.	gene_id "lnc-bbug01699"	transcript_id "lnc-bbut00004026"	exon_number "2"
10	lncRNA	exon	34703732	34703850	.	+	.	gene_id "lnc-bbug01699"	transcript_id "lnc-bbut00004026"	exon_number "3"
10	lncRNA	exon	34713516	34713668	.	+	.	gene_id "lnc-bbug01699"	transcript_id "lnc-bbut00004026"	exon_number "4"
10	lncRNA	exon	34714187	34715945	.	+	.	gene_id "lnc-bbug01699"	transcript_id "lnc-bbut00004026"	exon_number "5"
10	lncRNA	exon	36149890	36150969	.	+	.	gene_id "lnc-bbug01703"	transcript_id "lnc-bbut00004050"	exon_number "1"
10	lncRNA	exon	36176345	36176430	.	+	.	gene_id "lnc-bbug01703"	transcript_id "lnc-bbut00004050"	exon_number "2"
10	lncRNA	exon	36178291	36178364	.	+	.	gene_id "lnc-bbug01703"	transcript_id "lnc-bbut00004050"	exon_number "3"
10	lncRNA	exon	36181576	36181794	.	+	.	gene_id "lnc-bbug01703"	transcript_id "lnc-bbut00004050"	exon_number "4"
10	lncRNA	exon	36174272	36174350	.	+	.	gene_id "lnc-bbug01703"	transcript_id "lnc-bbut00004051"	exon_number "1"
10	lncRNA	exon	36176345	36176430	.	+	.	gene_id "lnc-bbug01703"	transcript_id "lnc-bbut00004051"	exon_number "2"
10	lncRNA	exon	36178291	36178364	.	+	.	gene_id "lnc-bbug01703"	transcript_id "lnc-bbut00004051"	exon_number "3"
10	lncRNA	exon	36181576	36181794	.	+	.	gene_id "lnc-bbug01703"	transcript_id "lnc-bbut00004051"	exon_number "4"
10	lncRNA	exon	36655358	36655531	.	+	.	gene_id "lnc-bbug01705"	transcript_id "lnc-bbut00004054"	exon_number "1"
10	lncRNA	exon	36657129	36657242	.	+	.	gene_id "lnc-bbug01705"	transcript_id "lnc-bbut00004054"	exon_number "2"
10	lncRNA	exon	36677238	36677344	.	+	.	gene_id "lnc-bbug01705"	transcript_id "lnc-bbut00004054"	exon_number "3"
10	lncRNA	exon	39023626	39023734	.	+	.	gene_id "lnc-bbug01713"	transcript_id "lnc-bbut00004080"	exon_number "1"
10	lncRNA	exon	39024215	39024403	.	+	.	gene_id "lnc-bbug01713"	transcript_id "lnc-bbut00004080"	exon_number "2"
10	lncRNA	exon	39043976	39044001	.	+	.	gene_id "lnc-bbug01713"	transcript_id "lnc-bbut00004080"	exon_number "3"
10	lncRNA	exon	39044748	39045418	.	+	.	gene_id "lnc-bbug01713"	transcript_id "lnc-bbut00004080"	exon_number "4"
10	lncRNA	exon	39047255	39048772	.	+	.	gene_id "lnc-bbug01713"	transcript_id "lnc-bbut00004080"	exon_number "5"
10	lncRNA	exon	39023637	39023734	.	+	.	gene_id "lnc-bbug01713"	transcript_id "lnc-bbut00004082"	exon_number "1"
10	lncRNA	exon	39024206	39024403	.	+	.	gene_id "lnc-bbug01713"	transcript_id "lnc-bbut00004082"	exon_number "2"
10	lncRNA	exon	39043976	39044001	.	+	.	gene_id "lnc-bbug01713"	transcript_id "lnc-bbut00004082"	exon_number "3"
10	lncRNA	exon	39044748	39045418	.	+	.	gene_id "lnc-bbug01713"	transcript_id "lnc-bbut00004082"	exon_number "4"
10	lncRNA	exon	39047255	39048772	.	+	.	gene_id "lnc-bbug01713"	transcript_id "lnc-bbut00004082"	exon_number "5"
10	lncRNA	exon	39023637	39023734	.	+	.	gene_id "lnc-bbug01713"	transcript_id "lnc-bbut00004081"	exon_number "1"
10	lncRNA	exon	39043976	39044001	.	+	.	gene_id "lnc-bbug01713"	transcript_id "lnc-bbut00004081"	exon_number "2"
10	lncRNA	exon	39044748	39045418	.	+	.	gene_id "lnc-bbug01713"	transcript_id "lnc-bbut00004081"	exon_number "3"
10	lncRNA	exon	39047255	39048772	.	+	.	gene_id "lnc-bbug01713"	transcript_id "lnc-bbut00004081"	exon_number "4"
10	lncRNA	exon	41612318	41618438	.	+	.	gene_id "lnc-bbug01720"	transcript_id "lnc-bbut00004093"	exon_number "1"
10	lncRNA	exon	41618542	41618602	.	+	.	gene_id "lnc-bbug01720"	transcript_id "lnc-bbut00004093"	exon_number "2"
10	lncRNA	exon	41618793	41619112	.	+	.	gene_id "lnc-bbug01720"	transcript_id "lnc-bbut00004093"	exon_number "3"
10	lncRNA	exon	41612318	41616120	.	+	.	gene_id "lnc-bbug01720"	transcript_id "lnc-bbut00004092"	exon_number "1"
10	lncRNA	exon	41618395	41618438	.	+	.	gene_id "lnc-bbug01720"	transcript_id "lnc-bbut00004092"	exon_number "2"
10	lncRNA	exon	41618542	41619112	.	+	.	gene_id "lnc-bbug01720"	transcript_id "lnc-bbut00004092"	exon_number "3"
10	lncRNA	exon	41647809	41647900	.	+	.	gene_id "lnc-bbug01721"	transcript_id "lnc-bbut00004094"	exon_number "1"
10	lncRNA	exon	41656840	41656997	.	+	.	gene_id "lnc-bbug01721"	transcript_id "lnc-bbut00004094"	exon_number "2"
10	lncRNA	exon	41658642	41659785	.	+	.	gene_id "lnc-bbug01721"	transcript_id "lnc-bbut00004094"	exon_number "3"
10	lncRNA	exon	41647881	41648011	.	+	.	gene_id "lnc-bbug01721"	transcript_id "lnc-bbut00004095"	exon_number "1"
10	lncRNA	exon	41656840	41656997	.	+	.	gene_id "lnc-bbug01721"	transcript_id "lnc-bbut00004095"	exon_number "2"
10	lncRNA	exon	41658642	41659785	.	+	.	gene_id "lnc-bbug01721"	transcript_id "lnc-bbut00004095"	exon_number "3"
10	lncRNA	exon	41655161	41655238	.	+	.	gene_id "lnc-bbug01721"	transcript_id "lnc-bbut00004096"	exon_number "1"
10	lncRNA	exon	41656840	41656997	.	+	.	gene_id "lnc-bbug01721"	transcript_id "lnc-bbut00004096"	exon_number "2"
10	lncRNA	exon	41658642	41659785	.	+	.	gene_id "lnc-bbug01721"	transcript_id "lnc-bbut00004096"	exon_number "3"
10	lncRNA	exon	41655173	41655195	.	+	.	gene_id "lnc-bbug01721"	transcript_id "lnc-bbut00004097"	exon_number "1"
10	lncRNA	exon	41656840	41656997	.	+	.	gene_id "lnc-bbug01721"	transcript_id "lnc-bbut00004097"	exon_number "2"
10	lncRNA	exon	41658642	41659785	.	+	.	gene_id "lnc-bbug01721"	transcript_id "lnc-bbut00004097"	exon_number "3"
10	lncRNA	exon	41980420	41980582	.	+	.	gene_id "lnc-bbug01726"	transcript_id "lnc-bbut00004111"	exon_number "1"

10	lncRNA	exon	42044762	42047636	.	+	.	gene_id "lnc-bbug01726"	transcript_id "lnc-bbut00004111"	exon_number "2"
10	lncRNA	exon	42022126	42022181	.	+	.	gene_id "lnc-bbug01726"	transcript_id "lnc-bbut00004116"	exon_number "1"
10	lncRNA	exon	42024909	42025097	.	+	.	gene_id "lnc-bbug01726"	transcript_id "lnc-bbut00004116"	exon_number "2"
10	lncRNA	exon	42044762	42047636	.	+	.	gene_id "lnc-bbug01726"	transcript_id "lnc-bbut00004116"	exon_number "3"
10	lncRNA	exon	42027232	42037047	.	+	.	gene_id "lnc-bbug01726"	transcript_id "lnc-bbut00004117"	exon_number "1"
10	lncRNA	exon	42037150	42038956	.	+	.	gene_id "lnc-bbug01726"	transcript_id "lnc-bbut00004117"	exon_number "2"
10	lncRNA	exon	42036998	42037047	.	+	.	gene_id "lnc-bbug01726"	transcript_id "lnc-bbut00004118"	exon_number "1"
10	lncRNA	exon	42057198	42057355	.	+	.	gene_id "lnc-bbug01726"	transcript_id "lnc-bbut00004118"	exon_number "2"
10	lncRNA	exon	42058214	42058701	.	+	.	gene_id "lnc-bbug01726"	transcript_id "lnc-bbut00004118"	exon_number "3"
10	lncRNA	exon	42042364	42045215	.	+	.	gene_id "lnc-bbug01726"	transcript_id "lnc-bbut00004119"	exon_number "1"
10	lncRNA	exon	42057198	42057355	.	+	.	gene_id "lnc-bbug01726"	transcript_id "lnc-bbut00004119"	exon_number "2"
10	lncRNA	exon	42058214	42058478	.	+	.	gene_id "lnc-bbug01726"	transcript_id "lnc-bbut00004119"	exon_number "3"
10	lncRNA	exon	42059335	42059818	.	+	.	gene_id "lnc-bbug01726"	transcript_id "lnc-bbut00004119"	exon_number "4"
10	lncRNA	exon	41993878	41994805	.	+	.	gene_id "lnc-bbug01727"	transcript_id "lnc-bbut00004112"	exon_number "1"
10	lncRNA	exon	41996035	41996150	.	+	.	gene_id "lnc-bbug01727"	transcript_id "lnc-bbut00004112"	exon_number "2"
10	lncRNA	exon	41995392	41996286	.	+	.	gene_id "lnc-bbug01727"	transcript_id "lnc-bbut00004113"	exon_number "1"
10	lncRNA	exon	42006946	42007232	.	+	.	gene_id "lnc-bbug01727"	transcript_id "lnc-bbut00004113"	exon_number "2"
10	lncRNA	exon	42009537	42011601	.	+	.	gene_id "lnc-bbug01727"	transcript_id "lnc-bbut00004113"	exon_number "3"
10	lncRNA	exon	42007079	42007103	.	+	.	gene_id "lnc-bbug01727"	transcript_id "lnc-bbut00004114"	exon_number "1"
10	lncRNA	exon	42009537	42011601	.	+	.	gene_id "lnc-bbug01727"	transcript_id "lnc-bbut00004114"	exon_number "2"
10	lncRNA	exon	42008800	42009304	.	+	.	gene_id "lnc-bbug01727"	transcript_id "lnc-bbut00004115"	exon_number "1"
10	lncRNA	exon	42009537	42011601	.	+	.	gene_id "lnc-bbug01727"	transcript_id "lnc-bbut00004115"	exon_number "2"
10	lncRNA	exon	43184390	43184756	.	+	.	gene_id "lnc-bbug01730"	transcript_id "lnc-bbut00004123"	exon_number "1"
10	lncRNA	exon	43204630	43205126	.	+	.	gene_id "lnc-bbug01730"	transcript_id "lnc-bbut00004123"	exon_number "2"
10	lncRNA	exon	43207614	43207711	.	+	.	gene_id "lnc-bbug01730"	transcript_id "lnc-bbut00004123"	exon_number "3"
10	lncRNA	exon	43208018	43210160	.	+	.	gene_id "lnc-bbug01730"	transcript_id "lnc-bbut00004123"	exon_number "4"
10	lncRNA	exon	43184390	43184756	.	+	.	gene_id "lnc-bbug01730"	transcript_id "lnc-bbut00004122"	exon_number "1"
10	lncRNA	exon	43204630	43204768	.	+	.	gene_id "lnc-bbug01730"	transcript_id "lnc-bbut00004122"	exon_number "2"
10	lncRNA	exon	43204897	43205126	.	+	.	gene_id "lnc-bbug01730"	transcript_id "lnc-bbut00004122"	exon_number "3"
10	lncRNA	exon	43207614	43210160	.	+	.	gene_id "lnc-bbug01730"	transcript_id "lnc-bbut00004122"	exon_number "4"
10	lncRNA	exon	43283766	43286311	.	+	.	gene_id "lnc-bbug01732"	transcript_id "lnc-bbut00004125"	exon_number "1"
10	lncRNA	exon	43287649	43287740	.	+	.	gene_id "lnc-bbug01732"	transcript_id "lnc-bbut00004125"	exon_number "2"
10	lncRNA	exon	43288293	43288449	.	+	.	gene_id "lnc-bbug01732"	transcript_id "lnc-bbut00004125"	exon_number "3"
10	lncRNA	exon	43448419	43448814	.	+	.	gene_id "lnc-bbug01734"	transcript_id "lnc-bbut00004127"	exon_number "1"
10	lncRNA	exon	43448918	43449032	.	+	.	gene_id "lnc-bbug01734"	transcript_id "lnc-bbut00004127"	exon_number "2"
10	lncRNA	exon	43478795	43479209	.	+	.	gene_id "lnc-bbug01734"	transcript_id "lnc-bbut00004127"	exon_number "3"
10	lncRNA	exon	43514879	43515751	.	+	.	gene_id "lnc-bbug01735"	transcript_id "lnc-bbut00004128"	exon_number "1"
10	lncRNA	exon	43775349	43775483	.	+	.	gene_id "lnc-bbug01737"	transcript_id "lnc-bbut00004133"	exon_number "1"
10	lncRNA	exon	43775797	43775910	.	+	.	gene_id "lnc-bbug01737"	transcript_id "lnc-bbut00004133"	exon_number "2"
10	lncRNA	exon	43776775	43777067	.	+	.	gene_id "lnc-bbug01737"	transcript_id "lnc-bbut00004133"	exon_number "3"
10	lncRNA	exon	44339426	44339539	.	+	.	gene_id "lnc-bbug01740"	transcript_id "lnc-bbut00004144"	exon_number "1"
10	lncRNA	exon	44340605	44340808	.	+	.	gene_id "lnc-bbug01740"	transcript_id "lnc-bbut00004144"	exon_number "2"
10	lncRNA	exon	44373699	44374032	.	+	.	gene_id "lnc-bbug01740"	transcript_id "lnc-bbut00004144"	exon_number "3"
10	lncRNA	exon	45217576	45217628	.	+	.	gene_id "lnc-bbug01742"	transcript_id "lnc-bbut00004146"	exon_number "1"
10	lncRNA	exon	45220763	45220899	.	+	.	gene_id "lnc-bbug01742"	transcript_id "lnc-bbut00004146"	exon_number "2"
10	lncRNA	exon	45221196	45221308	.	+	.	gene_id "lnc-bbug01742"	transcript_id "lnc-bbut00004146"	exon_number "3"
10	lncRNA	exon	50101427	50101718	.	+	.	gene_id "lnc-bbug01744"	transcript_id "lnc-bbut00004148"	exon_number "1"
10	lncRNA	exon	52645849	52646603	.	+	.	gene_id "lnc-bbug01750"	transcript_id "lnc-bbut00004165"	exon_number "1"
10	lncRNA	exon	52652760	52652999	.	+	.	gene_id "lnc-bbug01750"	transcript_id "lnc-bbut00004165"	exon_number "2"
10	lncRNA	exon	52661778	52661856	.	+	.	gene_id "lnc-bbug01750"	transcript_id "lnc-bbut00004165"	exon_number "3"
10	lncRNA	exon	52729623	52729754	.	+	.	gene_id "lnc-bbug01750"	transcript_id "lnc-bbut00004165"	exon_number "4"
10	lncRNA	exon	58276324	58276837	.	+	.	gene_id "lnc-bbug01756"	transcript_id "lnc-bbut00004177"	exon_number "1"

10	lncRNA	exon	58277704	58277914	.	+	.	gene_id "lnc-bbug01756"	transcript_id "lnc-bbut00004177"	exon_number "2"
10	lncRNA	exon	59263544	59263971	.	+	.	gene_id "lnc-bbug01758"	transcript_id "lnc-bbut00004179"	exon_number "1"
10	lncRNA	exon	59264173	59264302	.	+	.	gene_id "lnc-bbug01758"	transcript_id "lnc-bbut00004179"	exon_number "2"
10	lncRNA	exon	59268088	59268279	.	+	.	gene_id "lnc-bbug01758"	transcript_id "lnc-bbut00004179"	exon_number "3"
10	lncRNA	exon	59288279	59288489	.	+	.	gene_id "lnc-bbug01759"	transcript_id "lnc-bbut00004180"	exon_number "1"
10	lncRNA	exon	59297431	59297479	.	+	.	gene_id "lnc-bbug01759"	transcript_id "lnc-bbut00004180"	exon_number "2"
10	lncRNA	exon	59298914	59299216	.	+	.	gene_id "lnc-bbug01759"	transcript_id "lnc-bbut00004180"	exon_number "3"
10	lncRNA	exon	59289021	59289387	.	+	.	gene_id "lnc-bbug01759"	transcript_id "lnc-bbut00004181"	exon_number "1"
10	lncRNA	exon	59297431	59297479	.	+	.	gene_id "lnc-bbug01759"	transcript_id "lnc-bbut00004181"	exon_number "2"
10	lncRNA	exon	59298914	59299216	.	+	.	gene_id "lnc-bbug01759"	transcript_id "lnc-bbut00004181"	exon_number "3"
10	lncRNA	exon	60376833	60377677	.	+	.	gene_id "lnc-bbug01763"	transcript_id "lnc-bbut00004188"	exon_number "1"
10	lncRNA	exon	60626930	60628911	.	+	.	gene_id "lnc-bbug01765"	transcript_id "lnc-bbut00004195"	exon_number "1"
10	lncRNA	exon	61334479	61334539	.	+	.	gene_id "lnc-bbug01768"	transcript_id "lnc-bbut00004199"	exon_number "1"
10	lncRNA	exon	61334885	61334963	.	+	.	gene_id "lnc-bbug01768"	transcript_id "lnc-bbut00004199"	exon_number "2"
10	lncRNA	exon	61344590	61344664	.	+	.	gene_id "lnc-bbug01768"	transcript_id "lnc-bbut00004199"	exon_number "3"
10	lncRNA	exon	61345666	61345784	.	+	.	gene_id "lnc-bbug01769"	transcript_id "lnc-bbut00004200"	exon_number "1"
10	lncRNA	exon	61346660	61347253	.	+	.	gene_id "lnc-bbug01769"	transcript_id "lnc-bbut00004200"	exon_number "2"
10	lncRNA	exon	61425767	61427330	.	+	.	gene_id "lnc-bbug01771"	transcript_id "lnc-bbut00004202"	exon_number "1"
10	lncRNA	exon	62393782	62395786	.	+	.	gene_id "lnc-bbug01775"	transcript_id "lnc-bbut00004210"	exon_number "1"
10	lncRNA	exon	63023438	63025281	.	+	.	gene_id "lnc-bbug01780"	transcript_id "lnc-bbut00004221"	exon_number "1"
10	lncRNA	exon	63030651	63030776	.	+	.	gene_id "lnc-bbug01780"	transcript_id "lnc-bbut00004221"	exon_number "2"
10	lncRNA	exon	63049150	63049329	.	+	.	gene_id "lnc-bbug01780"	transcript_id "lnc-bbut00004221"	exon_number "3"
10	lncRNA	exon	63057389	63057583	.	+	.	gene_id "lnc-bbug01780"	transcript_id "lnc-bbut00004221"	exon_number "4"
10	lncRNA	exon	63058003	63058966	.	+	.	gene_id "lnc-bbug01780"	transcript_id "lnc-bbut00004221"	exon_number "5"
10	lncRNA	exon	63023438	63025281	.	+	.	gene_id "lnc-bbug01780"	transcript_id "lnc-bbut00004220"	exon_number "1"
10	lncRNA	exon	63030651	63030776	.	+	.	gene_id "lnc-bbug01780"	transcript_id "lnc-bbut00004220"	exon_number "2"
10	lncRNA	exon	63049150	63049329	.	+	.	gene_id "lnc-bbug01780"	transcript_id "lnc-bbut00004220"	exon_number "3"
10	lncRNA	exon	63057389	63058966	.	+	.	gene_id "lnc-bbug01780"	transcript_id "lnc-bbut00004220"	exon_number "4"
10	lncRNA	exon	63023439	63025281	.	+	.	gene_id "lnc-bbug01780"	transcript_id "lnc-bbut00004222"	exon_number "1"
10	lncRNA	exon	63030651	63030776	.	+	.	gene_id "lnc-bbug01780"	transcript_id "lnc-bbut00004222"	exon_number "2"
10	lncRNA	exon	63057389	63058966	.	+	.	gene_id "lnc-bbug01780"	transcript_id "lnc-bbut00004222"	exon_number "3"
10	lncRNA	exon	64707585	64707660	.	+	.	gene_id "lnc-bbug01788"	transcript_id "lnc-bbut00004246"	exon_number "1"
10	lncRNA	exon	64715037	64715145	.	+	.	gene_id "lnc-bbug01788"	transcript_id "lnc-bbut00004246"	exon_number "2"
10	lncRNA	exon	64715606	64715738	.	+	.	gene_id "lnc-bbug01788"	transcript_id "lnc-bbut00004246"	exon_number "3"
10	lncRNA	exon	64707585	64707660	.	+	.	gene_id "lnc-bbug01788"	transcript_id "lnc-bbut00004247"	exon_number "1"
10	lncRNA	exon	64715037	64715145	.	+	.	gene_id "lnc-bbug01788"	transcript_id "lnc-bbut00004247"	exon_number "2"
10	lncRNA	exon	64716362	64716515	.	+	.	gene_id "lnc-bbug01788"	transcript_id "lnc-bbut00004247"	exon_number "3"
10	lncRNA	exon	65428122	65428326	.	+	.	gene_id "lnc-bbug01794"	transcript_id "lnc-bbut00004257"	exon_number "1"
10	lncRNA	exon	65445119	65445180	.	+	.	gene_id "lnc-bbug01794"	transcript_id "lnc-bbut00004257"	exon_number "2"
10	lncRNA	exon	65446122	65446423	.	+	.	gene_id "lnc-bbug01794"	transcript_id "lnc-bbut00004257"	exon_number "3"
10	lncRNA	exon	65428122	65428326	.	+	.	gene_id "lnc-bbug01794"	transcript_id "lnc-bbut00004256"	exon_number "1"
10	lncRNA	exon	65440774	65440851	.	+	.	gene_id "lnc-bbug01794"	transcript_id "lnc-bbut00004256"	exon_number "2"
10	lncRNA	exon	65445119	65445180	.	+	.	gene_id "lnc-bbug01794"	transcript_id "lnc-bbut00004256"	exon_number "3"
10	lncRNA	exon	65446122	65446423	.	+	.	gene_id "lnc-bbug01794"	transcript_id "lnc-bbut00004256"	exon_number "4"
10	lncRNA	exon	65428122	65428326	.	+	.	gene_id "lnc-bbug01794"	transcript_id "lnc-bbut00004262"	exon_number "1"
10	lncRNA	exon	65440774	65440851	.	+	.	gene_id "lnc-bbug01794"	transcript_id "lnc-bbut00004262"	exon_number "2"
10	lncRNA	exon	65445119	65445180	.	+	.	gene_id "lnc-bbug01794"	transcript_id "lnc-bbut00004262"	exon_number "3"
10	lncRNA	exon	65446466	65446536	.	+	.	gene_id "lnc-bbug01794"	transcript_id "lnc-bbut00004262"	exon_number "4"
10	lncRNA	exon	65467291	65467347	.	+	.	gene_id "lnc-bbug01794"	transcript_id "lnc-bbut00004262"	exon_number "5"
10	lncRNA	exon	65467455	65469067	.	+	.	gene_id "lnc-bbug01794"	transcript_id "lnc-bbut00004262"	exon_number "6"
10	lncRNA	exon	65428122	65428326	.	+	.	gene_id "lnc-bbug01794"	transcript_id "lnc-bbut00004264"	exon_number "1"
10	lncRNA	exon	65440774	65440851	.	+	.	gene_id "lnc-bbug01794"	transcript_id "lnc-bbut00004264"	exon_number "2"

10	lncRNA	exon	65445119	65445180	.	+	.	gene_id "lnc-bbug01794"	transcript_id "lnc-bbut00004264"	exon_number "3"
10	lncRNA	exon	65446466	65446536	.	+	.	gene_id "lnc-bbug01794"	transcript_id "lnc-bbut00004264"	exon_number "4"
10	lncRNA	exon	65448808	65449081	.	+	.	gene_id "lnc-bbug01794"	transcript_id "lnc-bbut00004264"	exon_number "5"
10	lncRNA	exon	65467291	65467347	.	+	.	gene_id "lnc-bbug01794"	transcript_id "lnc-bbut00004264"	exon_number "6"
10	lncRNA	exon	65467455	65469067	.	+	.	gene_id "lnc-bbug01794"	transcript_id "lnc-bbut00004264"	exon_number "7"
10	lncRNA	exon	65428122	65428326	.	+	.	gene_id "lnc-bbug01794"	transcript_id "lnc-bbut00004263"	exon_number "1"
10	lncRNA	exon	65440774	65440851	.	+	.	gene_id "lnc-bbug01794"	transcript_id "lnc-bbut00004263"	exon_number "2"
10	lncRNA	exon	65445119	65445180	.	+	.	gene_id "lnc-bbug01794"	transcript_id "lnc-bbut00004263"	exon_number "3"
10	lncRNA	exon	65446466	65446536	.	+	.	gene_id "lnc-bbug01794"	transcript_id "lnc-bbut00004263"	exon_number "4"
10	lncRNA	exon	65467291	65467347	.	+	.	gene_id "lnc-bbug01794"	transcript_id "lnc-bbut00004263"	exon_number "5"
10	lncRNA	exon	65468403	65469067	.	+	.	gene_id "lnc-bbug01794"	transcript_id "lnc-bbut00004263"	exon_number "6"
10	lncRNA	exon	65428122	65428326	.	+	.	gene_id "lnc-bbug01794"	transcript_id "lnc-bbut00004261"	exon_number "1"
10	lncRNA	exon	65440774	65440851	.	+	.	gene_id "lnc-bbug01794"	transcript_id "lnc-bbut00004261"	exon_number "2"
10	lncRNA	exon	65445119	65445180	.	+	.	gene_id "lnc-bbug01794"	transcript_id "lnc-bbut00004261"	exon_number "3"
10	lncRNA	exon	65467291	65467347	.	+	.	gene_id "lnc-bbug01794"	transcript_id "lnc-bbut00004261"	exon_number "4"
10	lncRNA	exon	65468403	65469067	.	+	.	gene_id "lnc-bbug01794"	transcript_id "lnc-bbut00004261"	exon_number "5"
10	lncRNA	exon	65428122	65428326	.	+	.	gene_id "lnc-bbug01794"	transcript_id "lnc-bbut00004260"	exon_number "1"
10	lncRNA	exon	65440774	65440851	.	+	.	gene_id "lnc-bbug01794"	transcript_id "lnc-bbut00004260"	exon_number "2"
10	lncRNA	exon	65445119	65445180	.	+	.	gene_id "lnc-bbug01794"	transcript_id "lnc-bbut00004260"	exon_number "3"
10	lncRNA	exon	65467291	65467347	.	+	.	gene_id "lnc-bbug01794"	transcript_id "lnc-bbut00004260"	exon_number "4"
10	lncRNA	exon	65467455	65469067	.	+	.	gene_id "lnc-bbug01794"	transcript_id "lnc-bbut00004260"	exon_number "5"
10	lncRNA	exon	65428122	65428326	.	+	.	gene_id "lnc-bbug01794"	transcript_id "lnc-bbut00004259"	exon_number "1"
10	lncRNA	exon	65440774	65440851	.	+	.	gene_id "lnc-bbug01794"	transcript_id "lnc-bbut00004259"	exon_number "2"
10	lncRNA	exon	65467291	65467347	.	+	.	gene_id "lnc-bbug01794"	transcript_id "lnc-bbut00004259"	exon_number "3"
10	lncRNA	exon	65467455	65469067	.	+	.	gene_id "lnc-bbug01794"	transcript_id "lnc-bbut00004259"	exon_number "4"
10	lncRNA	exon	65428122	65428326	.	+	.	gene_id "lnc-bbug01794"	transcript_id "lnc-bbut00004258"	exon_number "1"
10	lncRNA	exon	65440774	65440851	.	+	.	gene_id "lnc-bbug01794"	transcript_id "lnc-bbut00004258"	exon_number "2"
10	lncRNA	exon	65445119	65445180	.	+	.	gene_id "lnc-bbug01794"	transcript_id "lnc-bbut00004258"	exon_number "3"
10	lncRNA	exon	65446466	65446536	.	+	.	gene_id "lnc-bbug01794"	transcript_id "lnc-bbut00004258"	exon_number "4"
10	lncRNA	exon	65448808	65449081	.	+	.	gene_id "lnc-bbug01794"	transcript_id "lnc-bbut00004258"	exon_number "5"
10	lncRNA	exon	65467291	65467347	.	+	.	gene_id "lnc-bbug01794"	transcript_id "lnc-bbut00004258"	exon_number "6"
10	lncRNA	exon	65468403	65469067	.	+	.	gene_id "lnc-bbug01794"	transcript_id "lnc-bbut00004258"	exon_number "7"
10	lncRNA	exon	65428122	65428326	.	+	.	gene_id "lnc-bbug01794"	transcript_id "lnc-bbut00004276"	exon_number "1"
10	lncRNA	exon	65440774	65440851	.	+	.	gene_id "lnc-bbug01794"	transcript_id "lnc-bbut00004276"	exon_number "2"
10	lncRNA	exon	65445119	65445180	.	+	.	gene_id "lnc-bbug01794"	transcript_id "lnc-bbut00004276"	exon_number "3"
10	lncRNA	exon	65446466	65446536	.	+	.	gene_id "lnc-bbug01794"	transcript_id "lnc-bbut00004276"	exon_number "4"
10	lncRNA	exon	65447879	65448024	.	+	.	gene_id "lnc-bbug01794"	transcript_id "lnc-bbut00004276"	exon_number "5"
10	lncRNA	exon	65448954	65449081	.	+	.	gene_id "lnc-bbug01794"	transcript_id "lnc-bbut00004276"	exon_number "6"
10	lncRNA	exon	65467291	65467347	.	+	.	gene_id "lnc-bbug01794"	transcript_id "lnc-bbut00004276"	exon_number "7"
10	lncRNA	exon	65479275	65483033	.	+	.	gene_id "lnc-bbug01794"	transcript_id "lnc-bbut00004276"	exon_number "8"
10	lncRNA	exon	65428122	65428326	.	+	.	gene_id "lnc-bbug01794"	transcript_id "lnc-bbut00004278"	exon_number "1"
10	lncRNA	exon	65440774	65440851	.	+	.	gene_id "lnc-bbug01794"	transcript_id "lnc-bbut00004278"	exon_number "2"
10	lncRNA	exon	65445119	65445180	.	+	.	gene_id "lnc-bbug01794"	transcript_id "lnc-bbut00004278"	exon_number "3"
10	lncRNA	exon	65446466	65446536	.	+	.	gene_id "lnc-bbug01794"	transcript_id "lnc-bbut00004278"	exon_number "4"
10	lncRNA	exon	65467291	65467347	.	+	.	gene_id "lnc-bbug01794"	transcript_id "lnc-bbut00004278"	exon_number "5"
10	lncRNA	exon	65479275	65483033	.	+	.	gene_id "lnc-bbug01794"	transcript_id "lnc-bbut00004278"	exon_number "6"
10	lncRNA	exon	65428122	65428326	.	+	.	gene_id "lnc-bbug01794"	transcript_id "lnc-bbut00004277"	exon_number "1"
10	lncRNA	exon	65440774	65440851	.	+	.	gene_id "lnc-bbug01794"	transcript_id "lnc-bbut00004277"	exon_number "2"
10	lncRNA	exon	65445119	65445180	.	+	.	gene_id "lnc-bbug01794"	transcript_id "lnc-bbut00004277"	exon_number "3"
10	lncRNA	exon	65446466	65446536	.	+	.	gene_id "lnc-bbug01794"	transcript_id "lnc-bbut00004277"	exon_number "4"
10	lncRNA	exon	65447879	65448024	.	+	.	gene_id "lnc-bbug01794"	transcript_id "lnc-bbut00004277"	exon_number "5"
10	lncRNA	exon	65448808	65448870	.	+	.	gene_id "lnc-bbug01794"	transcript_id "lnc-bbut00004277"	exon_number "6"

10	lncRNA	exon	65448954	65449081	.	+	.	gene_id "lnc-bbug01794"	transcript_id "lnc-bbut00004277"	exon_number "7"
10	lncRNA	exon	65467291	65467347	.	+	.	gene_id "lnc-bbug01794"	transcript_id "lnc-bbut00004277"	exon_number "8"
10	lncRNA	exon	65479275	65483033	.	+	.	gene_id "lnc-bbug01794"	transcript_id "lnc-bbut00004277"	exon_number "9"
10	lncRNA	exon	65428122	65428326	.	+	.	gene_id "lnc-bbug01794"	transcript_id "lnc-bbut00004275"	exon_number "1"
10	lncRNA	exon	65440774	65440851	.	+	.	gene_id "lnc-bbug01794"	transcript_id "lnc-bbut00004275"	exon_number "2"
10	lncRNA	exon	65445119	65445180	.	+	.	gene_id "lnc-bbug01794"	transcript_id "lnc-bbut00004275"	exon_number "3"
10	lncRNA	exon	65446466	65446536	.	+	.	gene_id "lnc-bbug01794"	transcript_id "lnc-bbut00004275"	exon_number "4"
10	lncRNA	exon	65482318	65483033	.	+	.	gene_id "lnc-bbug01794"	transcript_id "lnc-bbut00004275"	exon_number "5"
10	lncRNA	exon	65428122	65428326	.	+	.	gene_id "lnc-bbug01794"	transcript_id "lnc-bbut00004269"	exon_number "1"
10	lncRNA	exon	65440774	65440851	.	+	.	gene_id "lnc-bbug01794"	transcript_id "lnc-bbut00004269"	exon_number "2"
10	lncRNA	exon	65445119	65445180	.	+	.	gene_id "lnc-bbug01794"	transcript_id "lnc-bbut00004269"	exon_number "3"
10	lncRNA	exon	65446466	65446536	.	+	.	gene_id "lnc-bbug01794"	transcript_id "lnc-bbut00004269"	exon_number "4"
10	lncRNA	exon	65448808	65449081	.	+	.	gene_id "lnc-bbug01794"	transcript_id "lnc-bbut00004269"	exon_number "5"
10	lncRNA	exon	65467291	65467347	.	+	.	gene_id "lnc-bbug01794"	transcript_id "lnc-bbut00004269"	exon_number "6"
10	lncRNA	exon	65482318	65483033	.	+	.	gene_id "lnc-bbug01794"	transcript_id "lnc-bbut00004269"	exon_number "7"
10	lncRNA	exon	65428122	65428326	.	+	.	gene_id "lnc-bbug01794"	transcript_id "lnc-bbut00004270"	exon_number "1"
10	lncRNA	exon	65467291	65467347	.	+	.	gene_id "lnc-bbug01794"	transcript_id "lnc-bbut00004270"	exon_number "2"
10	lncRNA	exon	65479275	65480883	.	+	.	gene_id "lnc-bbug01794"	transcript_id "lnc-bbut00004270"	exon_number "3"
10	lncRNA	exon	65482318	65483033	.	+	.	gene_id "lnc-bbug01794"	transcript_id "lnc-bbut00004270"	exon_number "4"
10	lncRNA	exon	65428122	65428326	.	+	.	gene_id "lnc-bbug01794"	transcript_id "lnc-bbut00004272"	exon_number "1"
10	lncRNA	exon	65445119	65445180	.	+	.	gene_id "lnc-bbug01794"	transcript_id "lnc-bbut00004272"	exon_number "2"
10	lncRNA	exon	65467291	65467347	.	+	.	gene_id "lnc-bbug01794"	transcript_id "lnc-bbut00004272"	exon_number "3"
10	lncRNA	exon	65479275	65483033	.	+	.	gene_id "lnc-bbug01794"	transcript_id "lnc-bbut00004272"	exon_number "4"
10	lncRNA	exon	65428122	65428326	.	+	.	gene_id "lnc-bbug01794"	transcript_id "lnc-bbut00004273"	exon_number "1"
10	lncRNA	exon	65440774	65440851	.	+	.	gene_id "lnc-bbug01794"	transcript_id "lnc-bbut00004273"	exon_number "2"
10	lncRNA	exon	65467291	65467347	.	+	.	gene_id "lnc-bbug01794"	transcript_id "lnc-bbut00004273"	exon_number "3"
10	lncRNA	exon	65479275	65483033	.	+	.	gene_id "lnc-bbug01794"	transcript_id "lnc-bbut00004273"	exon_number "4"
10	lncRNA	exon	65428122	65428326	.	+	.	gene_id "lnc-bbug01794"	transcript_id "lnc-bbut00004274"	exon_number "1"
10	lncRNA	exon	65440774	65440851	.	+	.	gene_id "lnc-bbug01794"	transcript_id "lnc-bbut00004274"	exon_number "2"
10	lncRNA	exon	65445119	65445180	.	+	.	gene_id "lnc-bbug01794"	transcript_id "lnc-bbut00004274"	exon_number "3"
10	lncRNA	exon	65446466	65446536	.	+	.	gene_id "lnc-bbug01794"	transcript_id "lnc-bbut00004274"	exon_number "4"
10	lncRNA	exon	65448954	65449081	.	+	.	gene_id "lnc-bbug01794"	transcript_id "lnc-bbut00004274"	exon_number "5"
10	lncRNA	exon	65467291	65467347	.	+	.	gene_id "lnc-bbug01794"	transcript_id "lnc-bbut00004274"	exon_number "6"
10	lncRNA	exon	65479275	65483033	.	+	.	gene_id "lnc-bbug01794"	transcript_id "lnc-bbut00004274"	exon_number "7"
10	lncRNA	exon	65428122	65428326	.	+	.	gene_id "lnc-bbug01794"	transcript_id "lnc-bbut00004267"	exon_number "1"
10	lncRNA	exon	65440774	65440851	.	+	.	gene_id "lnc-bbug01794"	transcript_id "lnc-bbut00004267"	exon_number "2"
10	lncRNA	exon	65445119	65445180	.	+	.	gene_id "lnc-bbug01794"	transcript_id "lnc-bbut00004267"	exon_number "3"
10	lncRNA	exon	65446466	65446536	.	+	.	gene_id "lnc-bbug01794"	transcript_id "lnc-bbut00004267"	exon_number "4"
10	lncRNA	exon	65448808	65449081	.	+	.	gene_id "lnc-bbug01794"	transcript_id "lnc-bbut00004267"	exon_number "5"
10	lncRNA	exon	65482318	65483033	.	+	.	gene_id "lnc-bbug01794"	transcript_id "lnc-bbut00004267"	exon_number "6"
10	lncRNA	exon	65428122	65428326	.	+	.	gene_id "lnc-bbug01794"	transcript_id "lnc-bbut00004268"	exon_number "1"
10	lncRNA	exon	65440774	65440851	.	+	.	gene_id "lnc-bbug01794"	transcript_id "lnc-bbut00004268"	exon_number "2"
10	lncRNA	exon	65445119	65445180	.	+	.	gene_id "lnc-bbug01794"	transcript_id "lnc-bbut00004268"	exon_number "3"
10	lncRNA	exon	65446466	65446536	.	+	.	gene_id "lnc-bbug01794"	transcript_id "lnc-bbut00004268"	exon_number "4"
10	lncRNA	exon	65467291	65467347	.	+	.	gene_id "lnc-bbug01794"	transcript_id "lnc-bbut00004268"	exon_number "5"
10	lncRNA	exon	65482318	65483033	.	+	.	gene_id "lnc-bbug01794"	transcript_id "lnc-bbut00004268"	exon_number "6"
10	lncRNA	exon	65428122	65428326	.	+	.	gene_id "lnc-bbug01794"	transcript_id "lnc-bbut00004266"	exon_number "1"
10	lncRNA	exon	65440774	65440851	.	+	.	gene_id "lnc-bbug01794"	transcript_id "lnc-bbut00004266"	exon_number "2"
10	lncRNA	exon	65445119	65445180	.	+	.	gene_id "lnc-bbug01794"	transcript_id "lnc-bbut00004266"	exon_number "3"
10	lncRNA	exon	65467291	65467347	.	+	.	gene_id "lnc-bbug01794"	transcript_id "lnc-bbut00004266"	exon_number "4"
10	lncRNA	exon	65482318	65483033	.	+	.	gene_id "lnc-bbug01794"	transcript_id "lnc-bbut00004266"	exon_number "5"
10	lncRNA	exon	65428122	65428326	.	+	.	gene_id "lnc-bbug01794"	transcript_id "lnc-bbut00004265"	exon_number "1"

10	lncRNA	exon	65440774	65440851	.	+	.	gene_id "lnc-bbug01794"	transcript_id "lnc-bbut00004265"	exon_number "2"
10	lncRNA	exon	65445119	65445180	.	+	.	gene_id "lnc-bbug01794"	transcript_id "lnc-bbut00004265"	exon_number "3"
10	lncRNA	exon	65467291	65467347	.	+	.	gene_id "lnc-bbug01794"	transcript_id "lnc-bbut00004265"	exon_number "4"
10	lncRNA	exon	65479275	65483033	.	+	.	gene_id "lnc-bbug01794"	transcript_id "lnc-bbut00004265"	exon_number "5"
10	lncRNA	exon	65428122	65428326	.	+	.	gene_id "lnc-bbug01794"	transcript_id "lnc-bbut00004281"	exon_number "1"
10	lncRNA	exon	65494612	65494761	.	+	.	gene_id "lnc-bbug01794"	transcript_id "lnc-bbut00004281"	exon_number "2"
10	lncRNA	exon	65525390	65525474	.	+	.	gene_id "lnc-bbug01794"	transcript_id "lnc-bbut00004281"	exon_number "3"
10	lncRNA	exon	65526754	65527013	.	+	.	gene_id "lnc-bbug01794"	transcript_id "lnc-bbut00004281"	exon_number "4"
10	lncRNA	exon	65528787	65531280	.	+	.	gene_id "lnc-bbug01794"	transcript_id "lnc-bbut00004281"	exon_number "5"
10	lncRNA	exon	65535462	65538277	.	+	.	gene_id "lnc-bbug01794"	transcript_id "lnc-bbut00004281"	exon_number "6"
10	lncRNA	exon	65428122	65428326	.	+	.	gene_id "lnc-bbug01794"	transcript_id "lnc-bbut00004282"	exon_number "1"
10	lncRNA	exon	65494612	65494761	.	+	.	gene_id "lnc-bbug01794"	transcript_id "lnc-bbut00004282"	exon_number "2"
10	lncRNA	exon	65528787	65531280	.	+	.	gene_id "lnc-bbug01794"	transcript_id "lnc-bbut00004282"	exon_number "3"
10	lncRNA	exon	65535462	65538277	.	+	.	gene_id "lnc-bbug01794"	transcript_id "lnc-bbut00004282"	exon_number "4"
10	lncRNA	exon	65428122	65428326	.	+	.	gene_id "lnc-bbug01794"	transcript_id "lnc-bbut00004280"	exon_number "1"
10	lncRNA	exon	65494612	65494761	.	+	.	gene_id "lnc-bbug01794"	transcript_id "lnc-bbut00004280"	exon_number "2"
10	lncRNA	exon	65525390	65525474	.	+	.	gene_id "lnc-bbug01794"	transcript_id "lnc-bbut00004280"	exon_number "3"
10	lncRNA	exon	65528787	65531280	.	+	.	gene_id "lnc-bbug01794"	transcript_id "lnc-bbut00004280"	exon_number "4"
10	lncRNA	exon	65535462	65538277	.	+	.	gene_id "lnc-bbug01794"	transcript_id "lnc-bbut00004280"	exon_number "5"
10	lncRNA	exon	65428122	65428326	.	+	.	gene_id "lnc-bbug01794"	transcript_id "lnc-bbut00004279"	exon_number "1"
10	lncRNA	exon	65494612	65494761	.	+	.	gene_id "lnc-bbug01794"	transcript_id "lnc-bbut00004279"	exon_number "2"
10	lncRNA	exon	65525390	65525474	.	+	.	gene_id "lnc-bbug01794"	transcript_id "lnc-bbut00004279"	exon_number "3"
10	lncRNA	exon	65526754	65527013	.	+	.	gene_id "lnc-bbug01794"	transcript_id "lnc-bbut00004279"	exon_number "4"
10	lncRNA	exon	65528787	65531280	.	+	.	gene_id "lnc-bbug01794"	transcript_id "lnc-bbut00004279"	exon_number "5"
10	lncRNA	exon	65535462	65535595	.	+	.	gene_id "lnc-bbug01794"	transcript_id "lnc-bbut00004279"	exon_number "6"
10	lncRNA	exon	65536726	65538277	.	+	.	gene_id "lnc-bbug01794"	transcript_id "lnc-bbut00004279"	exon_number "7"
10	lncRNA	exon	65428337	65428437	.	+	.	gene_id "lnc-bbug01794"	transcript_id "lnc-bbut00004284"	exon_number "1"
10	lncRNA	exon	65440774	65440851	.	+	.	gene_id "lnc-bbug01794"	transcript_id "lnc-bbut00004284"	exon_number "2"
10	lncRNA	exon	65445119	65445180	.	+	.	gene_id "lnc-bbug01794"	transcript_id "lnc-bbut00004284"	exon_number "3"
10	lncRNA	exon	65446466	65446536	.	+	.	gene_id "lnc-bbug01794"	transcript_id "lnc-bbut00004284"	exon_number "4"
10	lncRNA	exon	65448808	65449081	.	+	.	gene_id "lnc-bbug01794"	transcript_id "lnc-bbut00004284"	exon_number "5"
10	lncRNA	exon	65467291	65467347	.	+	.	gene_id "lnc-bbug01794"	transcript_id "lnc-bbut00004284"	exon_number "6"
10	lncRNA	exon	65479275	65483033	.	+	.	gene_id "lnc-bbug01794"	transcript_id "lnc-bbut00004284"	exon_number "7"
10	lncRNA	exon	65428337	65428437	.	+	.	gene_id "lnc-bbug01794"	transcript_id "lnc-bbut00004283"	exon_number "1"
10	lncRNA	exon	65440774	65440851	.	+	.	gene_id "lnc-bbug01794"	transcript_id "lnc-bbut00004283"	exon_number "2"
10	lncRNA	exon	65467291	65467347	.	+	.	gene_id "lnc-bbug01794"	transcript_id "lnc-bbut00004283"	exon_number "3"
10	lncRNA	exon	65479275	65483033	.	+	.	gene_id "lnc-bbug01794"	transcript_id "lnc-bbut00004283"	exon_number "4"
10	lncRNA	exon	65526558	65526622	.	+	.	gene_id "lnc-bbug01794"	transcript_id "lnc-bbut00004287"	exon_number "1"
10	lncRNA	exon	65526754	65527013	.	+	.	gene_id "lnc-bbug01794"	transcript_id "lnc-bbut00004287"	exon_number "2"
10	lncRNA	exon	65528787	65531280	.	+	.	gene_id "lnc-bbug01794"	transcript_id "lnc-bbut00004287"	exon_number "3"
10	lncRNA	exon	65535462	65538277	.	+	.	gene_id "lnc-bbug01794"	transcript_id "lnc-bbut00004287"	exon_number "4"
10	lncRNA	exon	65681975	65682175	.	+	.	gene_id "lnc-bbug01795"	transcript_id "lnc-bbut00004288"	exon_number "1"
10	lncRNA	exon	65682711	65682798	.	+	.	gene_id "lnc-bbug01795"	transcript_id "lnc-bbut00004288"	exon_number "2"
10	lncRNA	exon	65684615	65684666	.	+	.	gene_id "lnc-bbug01795"	transcript_id "lnc-bbut00004288"	exon_number "3"
10	lncRNA	exon	66014823	66014973	.	+	.	gene_id "lnc-bbug01797"	transcript_id "lnc-bbut00004291"	exon_number "1"
10	lncRNA	exon	66020566	66020725	.	+	.	gene_id "lnc-bbug01797"	transcript_id "lnc-bbut00004291"	exon_number "2"
10	lncRNA	exon	66022826	66022964	.	+	.	gene_id "lnc-bbug01797"	transcript_id "lnc-bbut00004291"	exon_number "3"
10	lncRNA	exon	66024353	66027748	.	+	.	gene_id "lnc-bbug01797"	transcript_id "lnc-bbut00004291"	exon_number "4"
10	lncRNA	exon	66014823	66014973	.	+	.	gene_id "lnc-bbug01797"	transcript_id "lnc-bbut00004290"	exon_number "1"
10	lncRNA	exon	66020566	66020668	.	+	.	gene_id "lnc-bbug01797"	transcript_id "lnc-bbut00004290"	exon_number "2"
10	lncRNA	exon	66022826	66022964	.	+	.	gene_id "lnc-bbug01797"	transcript_id "lnc-bbut00004290"	exon_number "3"
10	lncRNA	exon	66024353	66027748	.	+	.	gene_id "lnc-bbug01797"	transcript_id "lnc-bbut00004290"	exon_number "4"

10	lncRNA	exon	66014830	66014973	.	+	.	gene_id "lnc-bbug01797"	transcript_id "lnc-bbut00004292"	exon_number "1"
10	lncRNA	exon	66020569	66020668	.	+	.	gene_id "lnc-bbug01797"	transcript_id "lnc-bbut00004292"	exon_number "2"
10	lncRNA	exon	66022826	66022964	.	+	.	gene_id "lnc-bbug01797"	transcript_id "lnc-bbut00004292"	exon_number "3"
10	lncRNA	exon	66024353	66027748	.	+	.	gene_id "lnc-bbug01797"	transcript_id "lnc-bbut00004292"	exon_number "4"
10	lncRNA	exon	66014832	66014973	.	+	.	gene_id "lnc-bbug01797"	transcript_id "lnc-bbut00004293"	exon_number "1"
10	lncRNA	exon	66020569	66020725	.	+	.	gene_id "lnc-bbug01797"	transcript_id "lnc-bbut00004293"	exon_number "2"
10	lncRNA	exon	66022826	66022964	.	+	.	gene_id "lnc-bbug01797"	transcript_id "lnc-bbut00004293"	exon_number "3"
10	lncRNA	exon	66024353	66027748	.	+	.	gene_id "lnc-bbug01797"	transcript_id "lnc-bbut00004293"	exon_number "4"
10	lncRNA	exon	66014835	66014984	.	+	.	gene_id "lnc-bbug01797"	transcript_id "lnc-bbut00004294"	exon_number "1"
10	lncRNA	exon	66020566	66020668	.	+	.	gene_id "lnc-bbug01797"	transcript_id "lnc-bbut00004294"	exon_number "2"
10	lncRNA	exon	66022826	66022964	.	+	.	gene_id "lnc-bbug01797"	transcript_id "lnc-bbut00004294"	exon_number "3"
10	lncRNA	exon	66024353	66027748	.	+	.	gene_id "lnc-bbug01797"	transcript_id "lnc-bbut00004294"	exon_number "4"
10	lncRNA	exon	66014837	66014984	.	+	.	gene_id "lnc-bbug01797"	transcript_id "lnc-bbut00004295"	exon_number "1"
10	lncRNA	exon	66020566	66020725	.	+	.	gene_id "lnc-bbug01797"	transcript_id "lnc-bbut00004295"	exon_number "2"
10	lncRNA	exon	66022826	66022964	.	+	.	gene_id "lnc-bbug01797"	transcript_id "lnc-bbut00004295"	exon_number "3"
10	lncRNA	exon	66024353	66027748	.	+	.	gene_id "lnc-bbug01797"	transcript_id "lnc-bbut00004295"	exon_number "4"
10	lncRNA	exon	66014839	66014984	.	+	.	gene_id "lnc-bbug01797"	transcript_id "lnc-bbut00004296"	exon_number "1"
10	lncRNA	exon	66020569	66020668	.	+	.	gene_id "lnc-bbug01797"	transcript_id "lnc-bbut00004296"	exon_number "2"
10	lncRNA	exon	66022826	66022964	.	+	.	gene_id "lnc-bbug01797"	transcript_id "lnc-bbut00004296"	exon_number "3"
10	lncRNA	exon	66024353	66027748	.	+	.	gene_id "lnc-bbug01797"	transcript_id "lnc-bbut00004296"	exon_number "4"
10	lncRNA	exon	66014844	66014984	.	+	.	gene_id "lnc-bbug01797"	transcript_id "lnc-bbut00004297"	exon_number "1"
10	lncRNA	exon	66020569	66020725	.	+	.	gene_id "lnc-bbug01797"	transcript_id "lnc-bbut00004297"	exon_number "2"
10	lncRNA	exon	66022826	66022964	.	+	.	gene_id "lnc-bbug01797"	transcript_id "lnc-bbut00004297"	exon_number "3"
10	lncRNA	exon	66024353	66027748	.	+	.	gene_id "lnc-bbug01797"	transcript_id "lnc-bbut00004297"	exon_number "4"
10	lncRNA	exon	66020075	66020096	.	+	.	gene_id "lnc-bbug01797"	transcript_id "lnc-bbut00004298"	exon_number "1"
10	lncRNA	exon	66020569	66020725	.	+	.	gene_id "lnc-bbug01797"	transcript_id "lnc-bbut00004298"	exon_number "2"
10	lncRNA	exon	66022826	66022964	.	+	.	gene_id "lnc-bbug01797"	transcript_id "lnc-bbut00004298"	exon_number "3"
10	lncRNA	exon	66024353	66027748	.	+	.	gene_id "lnc-bbug01797"	transcript_id "lnc-bbut00004298"	exon_number "4"
10	lncRNA	exon	66020077	66020096	.	+	.	gene_id "lnc-bbug01797"	transcript_id "lnc-bbut00004299"	exon_number "1"
10	lncRNA	exon	66020566	66020725	.	+	.	gene_id "lnc-bbug01797"	transcript_id "lnc-bbut00004299"	exon_number "2"
10	lncRNA	exon	66022826	66022964	.	+	.	gene_id "lnc-bbug01797"	transcript_id "lnc-bbut00004299"	exon_number "3"
10	lncRNA	exon	66024353	66027748	.	+	.	gene_id "lnc-bbug01797"	transcript_id "lnc-bbut00004299"	exon_number "4"
10	lncRNA	exon	67410252	67410406	.	+	.	gene_id "lnc-bbug01798"	transcript_id "lnc-bbut00004300"	exon_number "1"
10	lncRNA	exon	67411440	67411737	.	+	.	gene_id "lnc-bbug01798"	transcript_id "lnc-bbut00004300"	exon_number "2"
10	lncRNA	exon	67443648	67451802	.	+	.	gene_id "lnc-bbug01799"	transcript_id "lnc-bbut00004301"	exon_number "1"
10	lncRNA	exon	67453057	67458628	.	+	.	gene_id "lnc-bbug01799"	transcript_id "lnc-bbut00004301"	exon_number "2"
10	lncRNA	exon	67451341	67451427	.	+	.	gene_id "lnc-bbug01799"	transcript_id "lnc-bbut00004302"	exon_number "1"
10	lncRNA	exon	67451693	67451802	.	+	.	gene_id "lnc-bbug01799"	transcript_id "lnc-bbut00004302"	exon_number "2"
10	lncRNA	exon	67453057	67458628	.	+	.	gene_id "lnc-bbug01799"	transcript_id "lnc-bbut00004302"	exon_number "3"
10	lncRNA	exon	67458923	67464139	.	+	.	gene_id "lnc-bbug01800"	transcript_id "lnc-bbut00004303"	exon_number "1"
10	lncRNA	exon	67470691	67472452	.	+	.	gene_id "lnc-bbug01800"	transcript_id "lnc-bbut00004303"	exon_number "2"
10	lncRNA	exon	67775594	67775659	.	+	.	gene_id "lnc-bbug01801"	transcript_id "lnc-bbut00004304"	exon_number "1"
10	lncRNA	exon	67776342	67776669	.	+	.	gene_id "lnc-bbug01801"	transcript_id "lnc-bbut00004304"	exon_number "2"
10	lncRNA	exon	68195302	68195885	.	+	.	gene_id "lnc-bbug01803"	transcript_id "lnc-bbut00004308"	exon_number "1"
10	lncRNA	exon	68200182	68200250	.	+	.	gene_id "lnc-bbug01803"	transcript_id "lnc-bbut00004308"	exon_number "2"
10	lncRNA	exon	68204159	68204266	.	+	.	gene_id "lnc-bbug01803"	transcript_id "lnc-bbut00004308"	exon_number "3"
10	lncRNA	exon	68206448	68206666	.	+	.	gene_id "lnc-bbug01803"	transcript_id "lnc-bbut00004308"	exon_number "4"
10	lncRNA	exon	68195302	68195676	.	+	.	gene_id "lnc-bbug01803"	transcript_id "lnc-bbut00004307"	exon_number "1"
10	lncRNA	exon	68200182	68200250	.	+	.	gene_id "lnc-bbug01803"	transcript_id "lnc-bbut00004307"	exon_number "2"
10	lncRNA	exon	68204159	68204266	.	+	.	gene_id "lnc-bbug01803"	transcript_id "lnc-bbut00004307"	exon_number "3"
10	lncRNA	exon	68206448	68206666	.	+	.	gene_id "lnc-bbug01803"	transcript_id "lnc-bbut00004307"	exon_number "4"
10	lncRNA	exon	68195302	68195645	.	+	.	gene_id "lnc-bbug01803"	transcript_id "lnc-bbut00004306"	exon_number "1"

10	lncRNA	exon	68200182	68200250	.	+	.	gene_id "lnc-bbug01803"	transcript_id "lnc-bbut00004306"	exon_number "2"
10	lncRNA	exon	68204159	68204266	.	+	.	gene_id "lnc-bbug01803"	transcript_id "lnc-bbut00004306"	exon_number "3"
10	lncRNA	exon	68206448	68206666	.	+	.	gene_id "lnc-bbug01803"	transcript_id "lnc-bbut00004306"	exon_number "4"
10	lncRNA	exon	69888814	69890314	.	+	.	gene_id "lnc-bbug01805"	transcript_id "lnc-bbut00004312"	exon_number "1"
10	lncRNA	exon	69890892	69893027	.	+	.	gene_id "lnc-bbug01805"	transcript_id "lnc-bbut00004312"	exon_number "2"
10	lncRNA	exon	70000017	70000149	.	+	.	gene_id "lnc-bbug01806"	transcript_id "lnc-bbut00004313"	exon_number "1"
10	lncRNA	exon	70001564	70001695	.	+	.	gene_id "lnc-bbug01806"	transcript_id "lnc-bbut00004313"	exon_number "2"
10	lncRNA	exon	70004179	70004266	.	+	.	gene_id "lnc-bbug01806"	transcript_id "lnc-bbut00004313"	exon_number "3"
10	lncRNA	exon	70012045	70012219	.	+	.	gene_id "lnc-bbug01806"	transcript_id "lnc-bbut00004313"	exon_number "4"
10	lncRNA	exon	70026033	70026080	.	+	.	gene_id "lnc-bbug01806"	transcript_id "lnc-bbut00004313"	exon_number "5"
10	lncRNA	exon	70346857	70346998	.	+	.	gene_id "lnc-bbug01813"	transcript_id "lnc-bbut00004321"	exon_number "1"
10	lncRNA	exon	70355143	70355239	.	+	.	gene_id "lnc-bbug01813"	transcript_id "lnc-bbut00004321"	exon_number "2"
10	lncRNA	exon	70361536	70361637	.	+	.	gene_id "lnc-bbug01813"	transcript_id "lnc-bbut00004321"	exon_number "3"
10	lncRNA	exon	70370884	70370977	.	+	.	gene_id "lnc-bbug01813"	transcript_id "lnc-bbut00004321"	exon_number "4"
10	lncRNA	exon	70376167	70376289	.	+	.	gene_id "lnc-bbug01813"	transcript_id "lnc-bbut00004321"	exon_number "5"
10	lncRNA	exon	70378109	70378203	.	+	.	gene_id "lnc-bbug01813"	transcript_id "lnc-bbut00004321"	exon_number "6"
10	lncRNA	exon	70381734	70381919	.	+	.	gene_id "lnc-bbug01813"	transcript_id "lnc-bbut00004321"	exon_number "7"
10	lncRNA	exon	70383968	70384522	.	+	.	gene_id "lnc-bbug01813"	transcript_id "lnc-bbut00004321"	exon_number "8"
10	lncRNA	exon	71837005	71837056	.	+	.	gene_id "lnc-bbug01824"	transcript_id "lnc-bbut00004335"	exon_number "1"
10	lncRNA	exon	71839848	71840022	.	+	.	gene_id "lnc-bbug01824"	transcript_id "lnc-bbut00004335"	exon_number "2"
10	lncRNA	exon	71948871	71949005	.	+	.	gene_id "lnc-bbug01825"	transcript_id "lnc-bbut00004336"	exon_number "1"
10	lncRNA	exon	71957167	71959705	.	+	.	gene_id "lnc-bbug01825"	transcript_id "lnc-bbut00004336"	exon_number "2"
10	lncRNA	exon	71948871	71949005	.	+	.	gene_id "lnc-bbug01825"	transcript_id "lnc-bbut00004337"	exon_number "1"
10	lncRNA	exon	71957167	71957648	.	+	.	gene_id "lnc-bbug01825"	transcript_id "lnc-bbut00004337"	exon_number "2"
10	lncRNA	exon	71960366	71962050	.	+	.	gene_id "lnc-bbug01825"	transcript_id "lnc-bbut00004337"	exon_number "3"
10	lncRNA	exon	73022733	73023294	.	+	.	gene_id "lnc-bbug01827"	transcript_id "lnc-bbut00004339"	exon_number "1"
10	lncRNA	exon	73026610	73027160	.	+	.	gene_id "lnc-bbug01827"	transcript_id "lnc-bbut00004339"	exon_number "2"
10	lncRNA	exon	73041879	73042031	.	+	.	gene_id "lnc-bbug01827"	transcript_id "lnc-bbut00004339"	exon_number "3"
10	lncRNA	exon	73044738	73044792	.	+	.	gene_id "lnc-bbug01827"	transcript_id "lnc-bbut00004339"	exon_number "4"
10	lncRNA	exon	74931674	74931780	.	+	.	gene_id "lnc-bbug01829"	transcript_id "lnc-bbut00004341"	exon_number "1"
10	lncRNA	exon	74936258	74936566	.	+	.	gene_id "lnc-bbug01829"	transcript_id "lnc-bbut00004341"	exon_number "2"
10	lncRNA	exon	75693889	75694027	.	+	.	gene_id "lnc-bbug01831"	transcript_id "lnc-bbut00004346"	exon_number "1"
10	lncRNA	exon	75721693	75721791	.	+	.	gene_id "lnc-bbug01831"	transcript_id "lnc-bbut00004346"	exon_number "2"
10	lncRNA	exon	75805128	75805307	.	+	.	gene_id "lnc-bbug01831"	transcript_id "lnc-bbut00004346"	exon_number "3"
10	lncRNA	exon	75813508	75814759	.	+	.	gene_id "lnc-bbug01831"	transcript_id "lnc-bbut00004346"	exon_number "4"
10	lncRNA	exon	75693891	75694027	.	+	.	gene_id "lnc-bbug01831"	transcript_id "lnc-bbut00004348"	exon_number "1"
10	lncRNA	exon	75721693	75721791	.	+	.	gene_id "lnc-bbug01831"	transcript_id "lnc-bbut00004348"	exon_number "2"
10	lncRNA	exon	75766398	75766467	.	+	.	gene_id "lnc-bbug01831"	transcript_id "lnc-bbut00004348"	exon_number "3"
10	lncRNA	exon	75805128	75805307	.	+	.	gene_id "lnc-bbug01831"	transcript_id "lnc-bbut00004348"	exon_number "4"
10	lncRNA	exon	75813508	75814759	.	+	.	gene_id "lnc-bbug01831"	transcript_id "lnc-bbut00004348"	exon_number "5"
10	lncRNA	exon	75693898	75694027	.	+	.	gene_id "lnc-bbug01831"	transcript_id "lnc-bbut00004349"	exon_number "1"
10	lncRNA	exon	75766398	75766467	.	+	.	gene_id "lnc-bbug01831"	transcript_id "lnc-bbut00004349"	exon_number "2"
10	lncRNA	exon	75805128	75805307	.	+	.	gene_id "lnc-bbug01831"	transcript_id "lnc-bbut00004349"	exon_number "3"
10	lncRNA	exon	75813508	75814759	.	+	.	gene_id "lnc-bbug01831"	transcript_id "lnc-bbut00004349"	exon_number "4"
10	lncRNA	exon	75693906	75694027	.	+	.	gene_id "lnc-bbug01831"	transcript_id "lnc-bbut00004350"	exon_number "1"
10	lncRNA	exon	75805128	75805307	.	+	.	gene_id "lnc-bbug01831"	transcript_id "lnc-bbut00004350"	exon_number "2"
10	lncRNA	exon	75813508	75814759	.	+	.	gene_id "lnc-bbug01831"	transcript_id "lnc-bbut00004350"	exon_number "3"
10	lncRNA	exon	75743059	75743206	.	+	.	gene_id "lnc-bbug01831"	transcript_id "lnc-bbut00004351"	exon_number "1"
10	lncRNA	exon	75766398	75766467	.	+	.	gene_id "lnc-bbug01831"	transcript_id "lnc-bbut00004351"	exon_number "2"
10	lncRNA	exon	75805128	75805307	.	+	.	gene_id "lnc-bbug01831"	transcript_id "lnc-bbut00004351"	exon_number "3"
10	lncRNA	exon	75813508	75814759	.	+	.	gene_id "lnc-bbug01831"	transcript_id "lnc-bbut00004351"	exon_number "4"
10	lncRNA	exon	75765737	75765947	.	+	.	gene_id "lnc-bbug01831"	transcript_id "lnc-bbut00004352"	exon_number "1"

10	lncRNA	exon	75766398	75766467	.	+	.	gene_id "lnc-bbug01831"	transcript_id "lnc-bbut00004352"	exon_number "2"
10	lncRNA	exon	75805128	75805307	.	+	.	gene_id "lnc-bbug01831"	transcript_id "lnc-bbut00004352"	exon_number "3"
10	lncRNA	exon	75813508	75814759	.	+	.	gene_id "lnc-bbug01831"	transcript_id "lnc-bbut00004352"	exon_number "4"
10	lncRNA	exon	76419068	76419270	.	+	.	gene_id "lnc-bbug01835"	transcript_id "lnc-bbut00004359"	exon_number "1"
10	lncRNA	exon	76432495	76432560	.	+	.	gene_id "lnc-bbug01835"	transcript_id "lnc-bbut00004359"	exon_number "2"
10	lncRNA	exon	76433998	76434103	.	+	.	gene_id "lnc-bbug01835"	transcript_id "lnc-bbut00004359"	exon_number "3"
10	lncRNA	exon	76466858	76468770	.	+	.	gene_id "lnc-bbug01835"	transcript_id "lnc-bbut00004359"	exon_number "4"
10	lncRNA	exon	76470194	76470327	.	+	.	gene_id "lnc-bbug01835"	transcript_id "lnc-bbut00004359"	exon_number "5"
10	lncRNA	exon	76470647	76470800	.	+	.	gene_id "lnc-bbug01835"	transcript_id "lnc-bbut00004359"	exon_number "6"
10	lncRNA	exon	76471504	76471554	.	+	.	gene_id "lnc-bbug01835"	transcript_id "lnc-bbut00004359"	exon_number "7"
10	lncRNA	exon	76471779	76471827	.	+	.	gene_id "lnc-bbug01835"	transcript_id "lnc-bbut00004359"	exon_number "8"
10	lncRNA	exon	76419068	76419270	.	+	.	gene_id "lnc-bbug01835"	transcript_id "lnc-bbut00004360"	exon_number "1"
10	lncRNA	exon	76432495	76432560	.	+	.	gene_id "lnc-bbug01835"	transcript_id "lnc-bbut00004360"	exon_number "2"
10	lncRNA	exon	76433998	76434103	.	+	.	gene_id "lnc-bbug01835"	transcript_id "lnc-bbut00004360"	exon_number "3"
10	lncRNA	exon	76466858	76468770	.	+	.	gene_id "lnc-bbug01835"	transcript_id "lnc-bbut00004360"	exon_number "4"
10	lncRNA	exon	76470194	76470327	.	+	.	gene_id "lnc-bbug01835"	transcript_id "lnc-bbut00004360"	exon_number "5"
10	lncRNA	exon	76470647	76470800	.	+	.	gene_id "lnc-bbug01835"	transcript_id "lnc-bbut00004360"	exon_number "6"
10	lncRNA	exon	76471504	76471554	.	+	.	gene_id "lnc-bbug01835"	transcript_id "lnc-bbut00004360"	exon_number "7"
10	lncRNA	exon	76473834	76473882	.	+	.	gene_id "lnc-bbug01835"	transcript_id "lnc-bbut00004360"	exon_number "8"
10	lncRNA	exon	76419068	76419270	.	+	.	gene_id "lnc-bbug01835"	transcript_id "lnc-bbut00004362"	exon_number "1"
10	lncRNA	exon	76432495	76432560	.	+	.	gene_id "lnc-bbug01835"	transcript_id "lnc-bbut00004362"	exon_number "2"
10	lncRNA	exon	76433998	76434103	.	+	.	gene_id "lnc-bbug01835"	transcript_id "lnc-bbut00004362"	exon_number "3"
10	lncRNA	exon	76466858	76467056	.	+	.	gene_id "lnc-bbug01835"	transcript_id "lnc-bbut00004362"	exon_number "4"
10	lncRNA	exon	76468651	76468770	.	+	.	gene_id "lnc-bbug01835"	transcript_id "lnc-bbut00004362"	exon_number "5"
10	lncRNA	exon	76470194	76470327	.	+	.	gene_id "lnc-bbug01835"	transcript_id "lnc-bbut00004362"	exon_number "6"
10	lncRNA	exon	76470647	76470800	.	+	.	gene_id "lnc-bbug01835"	transcript_id "lnc-bbut00004362"	exon_number "7"
10	lncRNA	exon	76471504	76471554	.	+	.	gene_id "lnc-bbug01835"	transcript_id "lnc-bbut00004362"	exon_number "8"
10	lncRNA	exon	76478445	76479695	.	+	.	gene_id "lnc-bbug01835"	transcript_id "lnc-bbut00004362"	exon_number "9"
10	lncRNA	exon	76419068	76419270	.	+	.	gene_id "lnc-bbug01835"	transcript_id "lnc-bbut00004361"	exon_number "1"
10	lncRNA	exon	76432495	76432560	.	+	.	gene_id "lnc-bbug01835"	transcript_id "lnc-bbut00004361"	exon_number "2"
10	lncRNA	exon	76433998	76434103	.	+	.	gene_id "lnc-bbug01835"	transcript_id "lnc-bbut00004361"	exon_number "3"
10	lncRNA	exon	76466858	76468770	.	+	.	gene_id "lnc-bbug01835"	transcript_id "lnc-bbut00004361"	exon_number "4"
10	lncRNA	exon	76470194	76470327	.	+	.	gene_id "lnc-bbug01835"	transcript_id "lnc-bbut00004361"	exon_number "5"
10	lncRNA	exon	76470647	76470800	.	+	.	gene_id "lnc-bbug01835"	transcript_id "lnc-bbut00004361"	exon_number "6"
10	lncRNA	exon	76471504	76471554	.	+	.	gene_id "lnc-bbug01835"	transcript_id "lnc-bbut00004361"	exon_number "7"
10	lncRNA	exon	76478445	76479695	.	+	.	gene_id "lnc-bbug01835"	transcript_id "lnc-bbut00004361"	exon_number "8"
10	lncRNA	exon	76424466	76424632	.	+	.	gene_id "lnc-bbug01835"	transcript_id "lnc-bbut00004363"	exon_number "1"
10	lncRNA	exon	76432495	76432560	.	+	.	gene_id "lnc-bbug01835"	transcript_id "lnc-bbut00004363"	exon_number "2"
10	lncRNA	exon	76433998	76434103	.	+	.	gene_id "lnc-bbug01835"	transcript_id "lnc-bbut00004363"	exon_number "3"
10	lncRNA	exon	76466858	76468770	.	+	.	gene_id "lnc-bbug01835"	transcript_id "lnc-bbut00004363"	exon_number "4"
10	lncRNA	exon	76470194	76470327	.	+	.	gene_id "lnc-bbug01835"	transcript_id "lnc-bbut00004363"	exon_number "5"
10	lncRNA	exon	76470647	76470800	.	+	.	gene_id "lnc-bbug01835"	transcript_id "lnc-bbut00004363"	exon_number "6"
10	lncRNA	exon	76471504	76471554	.	+	.	gene_id "lnc-bbug01835"	transcript_id "lnc-bbut00004363"	exon_number "7"
10	lncRNA	exon	76478445	76479695	.	+	.	gene_id "lnc-bbug01835"	transcript_id "lnc-bbut00004363"	exon_number "8"
10	lncRNA	exon	76429447	76429490	.	+	.	gene_id "lnc-bbug01835"	transcript_id "lnc-bbut00004364"	exon_number "1"
10	lncRNA	exon	76432495	76432560	.	+	.	gene_id "lnc-bbug01835"	transcript_id "lnc-bbut00004364"	exon_number "2"
10	lncRNA	exon	76433998	76434103	.	+	.	gene_id "lnc-bbug01835"	transcript_id "lnc-bbut00004364"	exon_number "3"
10	lncRNA	exon	76466858	76468770	.	+	.	gene_id "lnc-bbug01835"	transcript_id "lnc-bbut00004364"	exon_number "4"
10	lncRNA	exon	76470194	76470327	.	+	.	gene_id "lnc-bbug01835"	transcript_id "lnc-bbut00004364"	exon_number "5"
10	lncRNA	exon	76470647	76470800	.	+	.	gene_id "lnc-bbug01835"	transcript_id "lnc-bbut00004364"	exon_number "6"
10	lncRNA	exon	76471504	76471554	.	+	.	gene_id "lnc-bbug01835"	transcript_id "lnc-bbut00004364"	exon_number "7"
10	lncRNA	exon	76478445	76479695	.	+	.	gene_id "lnc-bbug01835"	transcript_id "lnc-bbut00004364"	exon_number "8"

10	lncRNA	exon	78400449	78400626	.	+	.	gene_id "lnc-bbug01839"	transcript_id "lnc-bbut00004377"	exon_number "1"
10	lncRNA	exon	78412269	78412341	.	+	.	gene_id "lnc-bbug01839"	transcript_id "lnc-bbut00004377"	exon_number "2"
10	lncRNA	exon	78416469	78418457	.	+	.	gene_id "lnc-bbug01839"	transcript_id "lnc-bbut00004377"	exon_number "3"
10	lncRNA	exon	78425258	78426228	.	+	.	gene_id "lnc-bbug01840"	transcript_id "lnc-bbut00004378"	exon_number "1"
10	lncRNA	exon	78436580	78438376	.	+	.	gene_id "lnc-bbug01840"	transcript_id "lnc-bbut00004378"	exon_number "2"
10	lncRNA	exon	78439102	78439598	.	+	.	gene_id "lnc-bbug01840"	transcript_id "lnc-bbut00004378"	exon_number "3"
10	lncRNA	exon	79409225	79409537	.	+	.	gene_id "lnc-bbug01845"	transcript_id "lnc-bbut00004395"	exon_number "1"
10	lncRNA	exon	79415041	79415154	.	+	.	gene_id "lnc-bbug01845"	transcript_id "lnc-bbut00004395"	exon_number "2"
10	lncRNA	exon	79417073	79417657	.	+	.	gene_id "lnc-bbug01845"	transcript_id "lnc-bbut00004395"	exon_number "3"
10	lncRNA	exon	81287491	81287936	.	+	.	gene_id "lnc-bbug01854"	transcript_id "lnc-bbut00004417"	exon_number "1"
10	lncRNA	exon	81288731	81289066	.	+	.	gene_id "lnc-bbug01854"	transcript_id "lnc-bbut00004417"	exon_number "2"
10	lncRNA	exon	81538538	81539303	.	+	.	gene_id "lnc-bbug01856"	transcript_id "lnc-bbut00004421"	exon_number "1"
10	lncRNA	exon	82449480	82450172	.	+	.	gene_id "lnc-bbug01858"	transcript_id "lnc-bbut00004425"	exon_number "1"
10	lncRNA	exon	82450270	82450608	.	+	.	gene_id "lnc-bbug01858"	transcript_id "lnc-bbut00004425"	exon_number "2"
10	lncRNA	exon	84563015	84563195	.	+	.	gene_id "lnc-bbug01862"	transcript_id "lnc-bbut00004430"	exon_number "1"
10	lncRNA	exon	84565987	84566269	.	+	.	gene_id "lnc-bbug01862"	transcript_id "lnc-bbut00004430"	exon_number "2"
10	lncRNA	exon	84573635	84574000	.	+	.	gene_id "lnc-bbug01862"	transcript_id "lnc-bbut00004430"	exon_number "3"
10	lncRNA	exon	85164218	85164852	.	+	.	gene_id "lnc-bbug01864"	transcript_id "lnc-bbut00004434"	exon_number "1"
10	lncRNA	exon	85165060	85165332	.	+	.	gene_id "lnc-bbug01864"	transcript_id "lnc-bbut00004434"	exon_number "2"
10	lncRNA	exon	85567494	85567686	.	+	.	gene_id "lnc-bbug01866"	transcript_id "lnc-bbut00004440"	exon_number "1"
10	lncRNA	exon	85588052	85588102	.	+	.	gene_id "lnc-bbug01866"	transcript_id "lnc-bbut00004440"	exon_number "2"
10	lncRNA	exon	85594013	85594042	.	+	.	gene_id "lnc-bbug01866"	transcript_id "lnc-bbut00004440"	exon_number "3"
10	lncRNA	exon	85597291	85597341	.	+	.	gene_id "lnc-bbug01866"	transcript_id "lnc-bbut00004440"	exon_number "4"
10	lncRNA	exon	85599246	85599537	.	+	.	gene_id "lnc-bbug01866"	transcript_id "lnc-bbut00004440"	exon_number "5"
10	lncRNA	exon	85567501	85567686	.	+	.	gene_id "lnc-bbug01866"	transcript_id "lnc-bbut00004442"	exon_number "1"
10	lncRNA	exon	85588052	85588102	.	+	.	gene_id "lnc-bbug01866"	transcript_id "lnc-bbut00004442"	exon_number "2"
10	lncRNA	exon	85594013	85594042	.	+	.	gene_id "lnc-bbug01866"	transcript_id "lnc-bbut00004442"	exon_number "3"
10	lncRNA	exon	85597291	85597341	.	+	.	gene_id "lnc-bbug01866"	transcript_id "lnc-bbut00004442"	exon_number "4"
10	lncRNA	exon	85599544	85599663	.	+	.	gene_id "lnc-bbug01866"	transcript_id "lnc-bbut00004442"	exon_number "5"
10	lncRNA	exon	85599784	85599883	.	+	.	gene_id "lnc-bbug01866"	transcript_id "lnc-bbut00004442"	exon_number "6"
10	lncRNA	exon	85600211	85600694	.	+	.	gene_id "lnc-bbug01866"	transcript_id "lnc-bbut00004442"	exon_number "7"
10	lncRNA	exon	85911432	85911559	.	+	.	gene_id "lnc-bbug01868"	transcript_id "lnc-bbut00004451"	exon_number "1"
10	lncRNA	exon	85917229	85917474	.	+	.	gene_id "lnc-bbug01868"	transcript_id "lnc-bbut00004451"	exon_number "2"
10	lncRNA	exon	85935088	85935138	.	+	.	gene_id "lnc-bbug01868"	transcript_id "lnc-bbut00004451"	exon_number "3"
10	lncRNA	exon	86054702	86055603	.	+	.	gene_id "lnc-bbug01869"	transcript_id "lnc-bbut00004452"	exon_number "1"
10	lncRNA	exon	86057067	86057147	.	+	.	gene_id "lnc-bbug01869"	transcript_id "lnc-bbut00004452"	exon_number "2"
10	lncRNA	exon	86078181	86078333	.	+	.	gene_id "lnc-bbug01869"	transcript_id "lnc-bbut00004452"	exon_number "3"
10	lncRNA	exon	86163724	86164029	.	+	.	gene_id "lnc-bbug01869"	transcript_id "lnc-bbut00004452"	exon_number "4"
10	lncRNA	exon	86214805	86215091	.	+	.	gene_id "lnc-bbug01870"	transcript_id "lnc-bbut00004453"	exon_number "1"
10	lncRNA	exon	86215589	86215810	.	+	.	gene_id "lnc-bbug01870"	transcript_id "lnc-bbut00004453"	exon_number "2"
10	lncRNA	exon	86217170	86219039	.	+	.	gene_id "lnc-bbug01870"	transcript_id "lnc-bbut00004453"	exon_number "3"
10	lncRNA	exon	88530396	88530895	.	+	.	gene_id "lnc-bbug01872"	transcript_id "lnc-bbut00004455"	exon_number "1"
10	lncRNA	exon	89146966	89148425	.	+	.	gene_id "lnc-bbug01875"	transcript_id "lnc-bbut00004458"	exon_number "1"
10	lncRNA	exon	89151584	89151634	.	+	.	gene_id "lnc-bbug01875"	transcript_id "lnc-bbut00004458"	exon_number "2"
10	lncRNA	exon	89530867	89531023	.	+	.	gene_id "lnc-bbug01878"	transcript_id "lnc-bbut00004463"	exon_number "1"
10	lncRNA	exon	89532186	89532275	.	+	.	gene_id "lnc-bbug01878"	transcript_id "lnc-bbut00004463"	exon_number "2"
10	lncRNA	exon	89534376	89534460	.	+	.	gene_id "lnc-bbug01878"	transcript_id "lnc-bbut00004463"	exon_number "3"
10	lncRNA	exon	89536045	89536247	.	+	.	gene_id "lnc-bbug01878"	transcript_id "lnc-bbut00004463"	exon_number "4"
10	lncRNA	exon	90269067	90269497	.	+	.	gene_id "lnc-bbug01880"	transcript_id "lnc-bbut00004467"	exon_number "1"
10	lncRNA	exon	90269563	90270523	.	+	.	gene_id "lnc-bbug01880"	transcript_id "lnc-bbut00004467"	exon_number "2"
10	lncRNA	exon	90270761	90270939	.	+	.	gene_id "lnc-bbug01880"	transcript_id "lnc-bbut00004467"	exon_number "3"
10	lncRNA	exon	90573090	90573224	.	+	.	gene_id "lnc-bbug01881"	transcript_id "lnc-bbut00004468"	exon_number "1"

10	lncRNA	exon	90575829	90575898	.	+	.	gene_id "lnc-bbug01881"	transcript_id "lnc-bbut00004468"	exon_number "2"
10	lncRNA	exon	90701973	90702453	.	+	.	gene_id "lnc-bbug01881"	transcript_id "lnc-bbut00004468"	exon_number "3"
10	lncRNA	exon	91311001	91311111	.	+	.	gene_id "lnc-bbug01884"	transcript_id "lnc-bbut00004475"	exon_number "1"
10	lncRNA	exon	91325412	91325541	.	+	.	gene_id "lnc-bbug01884"	transcript_id "lnc-bbut00004475"	exon_number "2"
10	lncRNA	exon	91326117	91328670	.	+	.	gene_id "lnc-bbug01884"	transcript_id "lnc-bbut00004475"	exon_number "3"
10	lncRNA	exon	93809036	93813209	.	+	.	gene_id "lnc-bbug01890"	transcript_id "lnc-bbut00004484"	exon_number "1"
10	lncRNA	exon	93813937	93815160	.	+	.	gene_id "lnc-bbug01890"	transcript_id "lnc-bbut00004484"	exon_number "2"
10	lncRNA	exon	93949532	93949572	.	+	.	gene_id "lnc-bbug01891"	transcript_id "lnc-bbut00004485"	exon_number "1"
10	lncRNA	exon	93949742	93949789	.	+	.	gene_id "lnc-bbug01891"	transcript_id "lnc-bbut00004485"	exon_number "2"
10	lncRNA	exon	93950639	93950966	.	+	.	gene_id "lnc-bbug01891"	transcript_id "lnc-bbut00004485"	exon_number "3"
10	lncRNA	exon	94819224	94819400	.	+	.	gene_id "lnc-bbug01894"	transcript_id "lnc-bbut00004488"	exon_number "1"
10	lncRNA	exon	94836097	94836522	.	+	.	gene_id "lnc-bbug01894"	transcript_id "lnc-bbut00004488"	exon_number "2"
10	lncRNA	exon	94836632	94841784	.	+	.	gene_id "lnc-bbug01894"	transcript_id "lnc-bbut00004488"	exon_number "3"
10	lncRNA	exon	94819224	94819400	.	+	.	gene_id "lnc-bbug01894"	transcript_id "lnc-bbut00004489"	exon_number "1"
10	lncRNA	exon	94836097	94836154	.	+	.	gene_id "lnc-bbug01894"	transcript_id "lnc-bbut00004489"	exon_number "2"
10	lncRNA	exon	94873435	94873520	.	+	.	gene_id "lnc-bbug01894"	transcript_id "lnc-bbut00004489"	exon_number "3"
10	lncRNA	exon	94875539	94875686	.	+	.	gene_id "lnc-bbug01894"	transcript_id "lnc-bbut00004489"	exon_number "4"
10	lncRNA	exon	94819246	94819400	.	+	.	gene_id "lnc-bbug01894"	transcript_id "lnc-bbut00004490"	exon_number "1"
10	lncRNA	exon	94873435	94873520	.	+	.	gene_id "lnc-bbug01894"	transcript_id "lnc-bbut00004490"	exon_number "2"
10	lncRNA	exon	94875539	94875686	.	+	.	gene_id "lnc-bbug01894"	transcript_id "lnc-bbut00004490"	exon_number "3"
10	lncRNA	exon	94819247	94819400	.	+	.	gene_id "lnc-bbug01894"	transcript_id "lnc-bbut00004491"	exon_number "1"
10	lncRNA	exon	94836097	94836154	.	+	.	gene_id "lnc-bbug01894"	transcript_id "lnc-bbut00004491"	exon_number "2"
10	lncRNA	exon	94836632	94841784	.	+	.	gene_id "lnc-bbug01894"	transcript_id "lnc-bbut00004491"	exon_number "3"
10	lncRNA	exon	94863857	94864143	.	+	.	gene_id "lnc-bbug01894"	transcript_id "lnc-bbut00004492"	exon_number "1"
10	lncRNA	exon	94873435	94873520	.	+	.	gene_id "lnc-bbug01894"	transcript_id "lnc-bbut00004492"	exon_number "2"
10	lncRNA	exon	94875539	94875686	.	+	.	gene_id "lnc-bbug01894"	transcript_id "lnc-bbut00004492"	exon_number "3"
10	lncRNA	exon	95895470	95895565	.	+	.	gene_id "lnc-bbug01897"	transcript_id "lnc-bbut00004496"	exon_number "1"
10	lncRNA	exon	95899899	95900480	.	+	.	gene_id "lnc-bbug01897"	transcript_id "lnc-bbut00004496"	exon_number "2"
10	lncRNA	exon	96114873	96114979	.	+	.	gene_id "lnc-bbug01898"	transcript_id "lnc-bbut00004497"	exon_number "1"
10	lncRNA	exon	96121844	96122041	.	+	.	gene_id "lnc-bbug01898"	transcript_id "lnc-bbut00004497"	exon_number "2"
10	lncRNA	exon	96135502	96136461	.	+	.	gene_id "lnc-bbug01898"	transcript_id "lnc-bbut00004497"	exon_number "3"
10	lncRNA	exon	98164516	98164627	.	+	.	gene_id "lnc-bbug01899"	transcript_id "lnc-bbut00004498"	exon_number "1"
10	lncRNA	exon	98344870	98346089	.	+	.	gene_id "lnc-bbug01899"	transcript_id "lnc-bbut00004498"	exon_number "2"
10	lncRNA	exon	103831003	103831529	.	+	.	gene_id "lnc-bbug01904"	transcript_id "lnc-bbut00004504"	exon_number "1"
10	lncRNA	exon	103831622	103831981	.	+	.	gene_id "lnc-bbug01904"	transcript_id "lnc-bbut00004504"	exon_number "2"
10	lncRNA	exon	392284	394234	.	-	.	gene_id "lnc-bbug01912"	transcript_id "lnc-bbut00004519"	exon_number "1"
10	lncRNA	exon	395609	398472	.	-	.	gene_id "lnc-bbug01912"	transcript_id "lnc-bbut00004519"	exon_number "2"
10	lncRNA	exon	392284	394234	.	-	.	gene_id "lnc-bbug01912"	transcript_id "lnc-bbut00004518"	exon_number "1"
10	lncRNA	exon	395326	398472	.	-	.	gene_id "lnc-bbug01912"	transcript_id "lnc-bbut00004518"	exon_number "2"
10	lncRNA	exon	575577	577293	.	-	.	gene_id "lnc-bbug01914"	transcript_id "lnc-bbut00004531"	exon_number "1"
10	lncRNA	exon	582016	582118	.	-	.	gene_id "lnc-bbug01914"	transcript_id "lnc-bbut00004531"	exon_number "2"
10	lncRNA	exon	595317	596542	.	-	.	gene_id "lnc-bbug01914"	transcript_id "lnc-bbut00004531"	exon_number "3"
10	lncRNA	exon	1648047	1648400	.	-	.	gene_id "lnc-bbug01917"	transcript_id "lnc-bbut00004536"	exon_number "1"
10	lncRNA	exon	1648749	1649131	.	-	.	gene_id "lnc-bbug01917"	transcript_id "lnc-bbut00004536"	exon_number "2"
10	lncRNA	exon	2099541	2100502	.	-	.	gene_id "lnc-bbug01922"	transcript_id "lnc-bbut00004543"	exon_number "1"
10	lncRNA	exon	2115567	2116914	.	-	.	gene_id "lnc-bbug01922"	transcript_id "lnc-bbut00004543"	exon_number "2"
10	lncRNA	exon	2101316	2102110	.	-	.	gene_id "lnc-bbug01923"	transcript_id "lnc-bbut00004544"	exon_number "1"
10	lncRNA	exon	2106319	2106627	.	-	.	gene_id "lnc-bbug01923"	transcript_id "lnc-bbut00004544"	exon_number "2"
10	lncRNA	exon	2110553	2114781	.	-	.	gene_id "lnc-bbug01923"	transcript_id "lnc-bbut00004544"	exon_number "3"
10	lncRNA	exon	2574677	2575980	.	-	.	gene_id "lnc-bbug01925"	transcript_id "lnc-bbut00004547"	exon_number "1"
10	lncRNA	exon	2576840	2576978	.	-	.	gene_id "lnc-bbug01925"	transcript_id "lnc-bbut00004547"	exon_number "2"
10	lncRNA	exon	2577859	2578990	.	-	.	gene_id "lnc-bbug01925"	transcript_id "lnc-bbut00004547"	exon_number "3"

10	lncRNA	exon	2583396	2583489	.	-	.	gene_id "lnc-bbug01925"	transcript_id "lnc-bbut00004547"	exon_number "4"
10	lncRNA	exon	2583584	2584374	.	-	.	gene_id "lnc-bbug01925"	transcript_id "lnc-bbut00004547"	exon_number "5"
10	lncRNA	exon	2574677	2575188	.	-	.	gene_id "lnc-bbug01925"	transcript_id "lnc-bbut00004546"	exon_number "1"
10	lncRNA	exon	2575245	2576978	.	-	.	gene_id "lnc-bbug01925"	transcript_id "lnc-bbut00004546"	exon_number "2"
10	lncRNA	exon	2577859	2578990	.	-	.	gene_id "lnc-bbug01925"	transcript_id "lnc-bbut00004546"	exon_number "3"
10	lncRNA	exon	2583396	2584374	.	-	.	gene_id "lnc-bbug01925"	transcript_id "lnc-bbut00004546"	exon_number "4"
10	lncRNA	exon	2577661	2577986	.	-	.	gene_id "lnc-bbug01925"	transcript_id "lnc-bbut00004548"	exon_number "1"
10	lncRNA	exon	2580060	2580153	.	-	.	gene_id "lnc-bbug01925"	transcript_id "lnc-bbut00004548"	exon_number "2"
10	lncRNA	exon	2580589	2580799	.	-	.	gene_id "lnc-bbug01925"	transcript_id "lnc-bbut00004548"	exon_number "3"
10	lncRNA	exon	2583396	2584374	.	-	.	gene_id "lnc-bbug01925"	transcript_id "lnc-bbut00004548"	exon_number "4"
10	lncRNA	exon	2579436	2580153	.	-	.	gene_id "lnc-bbug01925"	transcript_id "lnc-bbut00004551"	exon_number "1"
10	lncRNA	exon	2580589	2580827	.	-	.	gene_id "lnc-bbug01925"	transcript_id "lnc-bbut00004551"	exon_number "2"
10	lncRNA	exon	2583396	2583503	.	-	.	gene_id "lnc-bbug01925"	transcript_id "lnc-bbut00004551"	exon_number "3"
10	lncRNA	exon	2583584	2584374	.	-	.	gene_id "lnc-bbug01925"	transcript_id "lnc-bbut00004551"	exon_number "4"
10	lncRNA	exon	2579436	2580153	.	-	.	gene_id "lnc-bbug01925"	transcript_id "lnc-bbut00004552"	exon_number "1"
10	lncRNA	exon	2580589	2580799	.	-	.	gene_id "lnc-bbug01925"	transcript_id "lnc-bbut00004552"	exon_number "2"
10	lncRNA	exon	2583396	2583503	.	-	.	gene_id "lnc-bbug01925"	transcript_id "lnc-bbut00004552"	exon_number "3"
10	lncRNA	exon	2583584	2583728	.	-	.	gene_id "lnc-bbug01925"	transcript_id "lnc-bbut00004552"	exon_number "4"
10	lncRNA	exon	2583839	2584374	.	-	.	gene_id "lnc-bbug01925"	transcript_id "lnc-bbut00004552"	exon_number "5"
10	lncRNA	exon	2579436	2580153	.	-	.	gene_id "lnc-bbug01925"	transcript_id "lnc-bbut00004550"	exon_number "1"
10	lncRNA	exon	2580589	2580827	.	-	.	gene_id "lnc-bbug01925"	transcript_id "lnc-bbut00004550"	exon_number "2"
10	lncRNA	exon	2583396	2583489	.	-	.	gene_id "lnc-bbug01925"	transcript_id "lnc-bbut00004550"	exon_number "3"
10	lncRNA	exon	2583584	2584374	.	-	.	gene_id "lnc-bbug01925"	transcript_id "lnc-bbut00004550"	exon_number "4"
10	lncRNA	exon	2579436	2580153	.	-	.	gene_id "lnc-bbug01925"	transcript_id "lnc-bbut00004549"	exon_number "1"
10	lncRNA	exon	2580589	2580799	.	-	.	gene_id "lnc-bbug01925"	transcript_id "lnc-bbut00004549"	exon_number "2"
10	lncRNA	exon	2583396	2583489	.	-	.	gene_id "lnc-bbug01925"	transcript_id "lnc-bbut00004549"	exon_number "3"
10	lncRNA	exon	2583584	2584374	.	-	.	gene_id "lnc-bbug01925"	transcript_id "lnc-bbut00004549"	exon_number "4"
10	lncRNA	exon	2700908	2701148	.	-	.	gene_id "lnc-bbug01927"	transcript_id "lnc-bbut00004554"	exon_number "1"
10	lncRNA	exon	2701265	2701310	.	-	.	gene_id "lnc-bbug01927"	transcript_id "lnc-bbut00004554"	exon_number "2"
10	lncRNA	exon	3115056	3115735	.	-	.	gene_id "lnc-bbug01929"	transcript_id "lnc-bbut00004556"	exon_number "1"
10	lncRNA	exon	3117844	3117974	.	-	.	gene_id "lnc-bbug01929"	transcript_id "lnc-bbut00004556"	exon_number "2"
10	lncRNA	exon	3118212	3118390	.	-	.	gene_id "lnc-bbug01929"	transcript_id "lnc-bbut00004556"	exon_number "3"
10	lncRNA	exon	4450915	4454283	.	-	.	gene_id "lnc-bbug01931"	transcript_id "lnc-bbut00004558"	exon_number "1"
10	lncRNA	exon	4460391	4461966	.	-	.	gene_id "lnc-bbug01931"	transcript_id "lnc-bbut00004558"	exon_number "2"
10	lncRNA	exon	4545875	4547051	.	-	.	gene_id "lnc-bbug01932"	transcript_id "lnc-bbut00004559"	exon_number "1"
10	lncRNA	exon	4547917	4548033	.	-	.	gene_id "lnc-bbug01932"	transcript_id "lnc-bbut00004559"	exon_number "2"
10	lncRNA	exon	4545875	4547051	.	-	.	gene_id "lnc-bbug01932"	transcript_id "lnc-bbut00004560"	exon_number "1"
10	lncRNA	exon	4547952	4548067	.	-	.	gene_id "lnc-bbug01932"	transcript_id "lnc-bbut00004560"	exon_number "2"
10	lncRNA	exon	6571568	6571689	.	-	.	gene_id "lnc-bbug01935"	transcript_id "lnc-bbut00004571"	exon_number "1"
10	lncRNA	exon	6573663	6573745	.	-	.	gene_id "lnc-bbug01935"	transcript_id "lnc-bbut00004571"	exon_number "2"
10	lncRNA	exon	6574355	6575062	.	-	.	gene_id "lnc-bbug01935"	transcript_id "lnc-bbut00004571"	exon_number "3"
10	lncRNA	exon	7380642	7380761	.	-	.	gene_id "lnc-bbug01937"	transcript_id "lnc-bbut00004578"	exon_number "1"
10	lncRNA	exon	7384809	7384929	.	-	.	gene_id "lnc-bbug01937"	transcript_id "lnc-bbut00004578"	exon_number "2"
10	lncRNA	exon	7386155	7386822	.	-	.	gene_id "lnc-bbug01937"	transcript_id "lnc-bbut00004578"	exon_number "3"
10	lncRNA	exon	8031464	8035103	.	-	.	gene_id "lnc-bbug01946"	transcript_id "lnc-bbut00004601"	exon_number "1"
10	lncRNA	exon	8035934	8036802	.	-	.	gene_id "lnc-bbug01946"	transcript_id "lnc-bbut00004601"	exon_number "2"
10	lncRNA	exon	8682586	8683837	.	-	.	gene_id "lnc-bbug01950"	transcript_id "lnc-bbut00004605"	exon_number "1"
10	lncRNA	exon	8683929	8684076	.	-	.	gene_id "lnc-bbug01950"	transcript_id "lnc-bbut00004605"	exon_number "2"
10	lncRNA	exon	9020109	9021518	.	-	.	gene_id "lnc-bbug01953"	transcript_id "lnc-bbut00004613"	exon_number "1"
10	lncRNA	exon	9293607	9294018	.	-	.	gene_id "lnc-bbug01955"	transcript_id "lnc-bbut00004616"	exon_number "1"
10	lncRNA	exon	9304937	9305019	.	-	.	gene_id "lnc-bbug01955"	transcript_id "lnc-bbut00004616"	exon_number "2"
10	lncRNA	exon	9305844	9305963	.	-	.	gene_id "lnc-bbug01955"	transcript_id "lnc-bbut00004616"	exon_number "3"

10	lncRNA	exon	9626931	9627181	.	-	.	gene_id "lnc-bbug01958"	transcript_id "lnc-bbut00004620"	exon_number "1"
10	lncRNA	exon	9641591	9641732	.	-	.	gene_id "lnc-bbug01958"	transcript_id "lnc-bbut00004620"	exon_number "2"
10	lncRNA	exon	10094734	10094845	.	-	.	gene_id "lnc-bbug01960"	transcript_id "lnc-bbut00004626"	exon_number "1"
10	lncRNA	exon	10096120	10096277	.	-	.	gene_id "lnc-bbug01960"	transcript_id "lnc-bbut00004626"	exon_number "2"
10	lncRNA	exon	10097809	10097938	.	-	.	gene_id "lnc-bbug01960"	transcript_id "lnc-bbut00004626"	exon_number "3"
10	lncRNA	exon	10121281	10121439	.	-	.	gene_id "lnc-bbug01960"	transcript_id "lnc-bbut00004626"	exon_number "4"
10	lncRNA	exon	10125709	10125905	.	-	.	gene_id "lnc-bbug01960"	transcript_id "lnc-bbut00004626"	exon_number "5"
10	lncRNA	exon	12676386	12678922	.	-	.	gene_id "lnc-bbug01965"	transcript_id "lnc-bbut00004655"	exon_number "1"
10	lncRNA	exon	12806156	12809461	.	-	.	gene_id "lnc-bbug01966"	transcript_id "lnc-bbut00004656"	exon_number "1"
10	lncRNA	exon	14352280	14353308	.	-	.	gene_id "lnc-bbug01970"	transcript_id "lnc-bbut00004661"	exon_number "1"
10	lncRNA	exon	14354442	14354580	.	-	.	gene_id "lnc-bbug01970"	transcript_id "lnc-bbut00004661"	exon_number "2"
10	lncRNA	exon	14356853	14356921	.	-	.	gene_id "lnc-bbug01970"	transcript_id "lnc-bbut00004661"	exon_number "3"
10	lncRNA	exon	15513888	15514129	.	-	.	gene_id "lnc-bbug01975"	transcript_id "lnc-bbut00004674"	exon_number "1"
10	lncRNA	exon	15514403	15515112	.	-	.	gene_id "lnc-bbug01975"	transcript_id "lnc-bbut00004674"	exon_number "2"
10	lncRNA	exon	15787537	15787581	.	-	.	gene_id "lnc-bbug01978"	transcript_id "lnc-bbut00004679"	exon_number "1"
10	lncRNA	exon	15788079	15788114	.	-	.	gene_id "lnc-bbug01978"	transcript_id "lnc-bbut00004679"	exon_number "2"
10	lncRNA	exon	15793690	15793910	.	-	.	gene_id "lnc-bbug01978"	transcript_id "lnc-bbut00004679"	exon_number "3"
10	lncRNA	exon	16693160	16694696	.	-	.	gene_id "lnc-bbug01983"	transcript_id "lnc-bbut00004692"	exon_number "1"
10	lncRNA	exon	16695194	16695815	.	-	.	gene_id "lnc-bbug01983"	transcript_id "lnc-bbut00004692"	exon_number "2"
10	lncRNA	exon	16824085	16824389	.	-	.	gene_id "lnc-bbug01985"	transcript_id "lnc-bbut00004694"	exon_number "1"
10	lncRNA	exon	16824460	16826475	.	-	.	gene_id "lnc-bbug01985"	transcript_id "lnc-bbut00004694"	exon_number "2"
10	lncRNA	exon	17040607	17041110	.	-	.	gene_id "lnc-bbug01989"	transcript_id "lnc-bbut00004700"	exon_number "1"
10	lncRNA	exon	17042271	17042403	.	-	.	gene_id "lnc-bbug01989"	transcript_id "lnc-bbut00004700"	exon_number "2"
10	lncRNA	exon	17043541	17043816	.	-	.	gene_id "lnc-bbug01989"	transcript_id "lnc-bbut00004700"	exon_number "3"
10	lncRNA	exon	17044193	17044456	.	-	.	gene_id "lnc-bbug01989"	transcript_id "lnc-bbut00004700"	exon_number "4"
10	lncRNA	exon	17071257	17071433	.	-	.	gene_id "lnc-bbug01989"	transcript_id "lnc-bbut00004700"	exon_number "5"
10	lncRNA	exon	17065645	17069028	.	-	.	gene_id "lnc-bbug01991"	transcript_id "lnc-bbut00004702"	exon_number "1"
10	lncRNA	exon	17069119	17069248	.	-	.	gene_id "lnc-bbug01991"	transcript_id "lnc-bbut00004702"	exon_number "2"
10	lncRNA	exon	17071207	17071244	.	-	.	gene_id "lnc-bbug01991"	transcript_id "lnc-bbut00004702"	exon_number "3"
10	lncRNA	exon	17129432	17134126	.	-	.	gene_id "lnc-bbug01993"	transcript_id "lnc-bbut00004708"	exon_number "1"
10	lncRNA	exon	17134217	17134346	.	-	.	gene_id "lnc-bbug01993"	transcript_id "lnc-bbut00004708"	exon_number "2"
10	lncRNA	exon	17136306	17136343	.	-	.	gene_id "lnc-bbug01993"	transcript_id "lnc-bbut00004708"	exon_number "3"
10	lncRNA	exon	17160810	17162092	.	-	.	gene_id "lnc-bbug01995"	transcript_id "lnc-bbut00004710"	exon_number "1"
10	lncRNA	exon	17162715	17162965	.	-	.	gene_id "lnc-bbug01995"	transcript_id "lnc-bbut00004710"	exon_number "2"
10	lncRNA	exon	17166010	17166411	.	-	.	gene_id "lnc-bbug01996"	transcript_id "lnc-bbut00004711"	exon_number "1"
10	lncRNA	exon	17166529	17167673	.	-	.	gene_id "lnc-bbug01996"	transcript_id "lnc-bbut00004711"	exon_number "2"
10	lncRNA	exon	17169839	17170002	.	-	.	gene_id "lnc-bbug01996"	transcript_id "lnc-bbut00004711"	exon_number "3"
10	lncRNA	exon	17394679	17394939	.	-	.	gene_id "lnc-bbug01998"	transcript_id "lnc-bbut00004715"	exon_number "1"
10	lncRNA	exon	17405477	17405826	.	-	.	gene_id "lnc-bbug01998"	transcript_id "lnc-bbut00004715"	exon_number "2"
10	lncRNA	exon	17400184	17400482	.	-	.	gene_id "lnc-bbug01998"	transcript_id "lnc-bbut00004716"	exon_number "1"
10	lncRNA	exon	17405477	17405826	.	-	.	gene_id "lnc-bbug01998"	transcript_id "lnc-bbut00004716"	exon_number "2"
10	lncRNA	exon	18072400	18077850	.	-	.	gene_id "lnc-bbug02002"	transcript_id "lnc-bbut00004726"	exon_number "1"
10	lncRNA	exon	18078988	18079910	.	-	.	gene_id "lnc-bbug02002"	transcript_id "lnc-bbut00004726"	exon_number "2"
10	lncRNA	exon	18433537	18435511	.	-	.	gene_id "lnc-bbug02004"	transcript_id "lnc-bbut00004730"	exon_number "1"
10	lncRNA	exon	18435857	18435872	.	-	.	gene_id "lnc-bbug02004"	transcript_id "lnc-bbut00004730"	exon_number "2"
10	lncRNA	exon	18649562	18650759	.	-	.	gene_id "lnc-bbug02005"	transcript_id "lnc-bbut00004731"	exon_number "1"
10	lncRNA	exon	18809119	18810821	.	-	.	gene_id "lnc-bbug02006"	transcript_id "lnc-bbut00004732"	exon_number "1"
10	lncRNA	exon	18813317	18813743	.	-	.	gene_id "lnc-bbug02006"	transcript_id "lnc-bbut00004732"	exon_number "2"
10	lncRNA	exon	19475612	19475743	.	-	.	gene_id "lnc-bbug02009"	transcript_id "lnc-bbut00004736"	exon_number "1"
10	lncRNA	exon	19477783	19478247	.	-	.	gene_id "lnc-bbug02009"	transcript_id "lnc-bbut00004736"	exon_number "2"
10	lncRNA	exon	19478354	19479227	.	-	.	gene_id "lnc-bbug02009"	transcript_id "lnc-bbut00004736"	exon_number "3"
10	lncRNA	exon	19774611	19774899	.	-	.	gene_id "lnc-bbug02011"	transcript_id "lnc-bbut00004744"	exon_number "1"

10	lncRNA	exon	19775764	19775969	.	-	.	gene_id "lnc-bbug02011" transcript_id "lnc-bbut00004744" exon_number "2"
10	lncRNA	exon	19784601	19784782	.	-	.	gene_id "lnc-bbug02011" transcript_id "lnc-bbut00004744" exon_number "3"
10	lncRNA	exon	20177301	20181271	.	-	.	gene_id "lnc-bbug02013" transcript_id "lnc-bbut00004749" exon_number "1"
10	lncRNA	exon	20181916	20182029	.	-	.	gene_id "lnc-bbug02013" transcript_id "lnc-bbut00004749" exon_number "2"
10	lncRNA	exon	22873409	22874459	.	-	.	gene_id "lnc-bbug02019" transcript_id "lnc-bbut00004759" exon_number "1"
10	lncRNA	exon	22874697	22874839	.	-	.	gene_id "lnc-bbug02019" transcript_id "lnc-bbut00004759" exon_number "2"
10	lncRNA	exon	22873409	22874459	.	-	.	gene_id "lnc-bbug02019" transcript_id "lnc-bbut00004760" exon_number "1"
10	lncRNA	exon	22875083	22875193	.	-	.	gene_id "lnc-bbug02019" transcript_id "lnc-bbut00004760" exon_number "2"
10	lncRNA	exon	25917995	25918599	.	-	.	gene_id "lnc-bbug02028" transcript_id "lnc-bbut00004786" exon_number "1"
10	lncRNA	exon	25918673	25918773	.	-	.	gene_id "lnc-bbug02028" transcript_id "lnc-bbut00004786" exon_number "2"
10	lncRNA	exon	26920397	26924557	.	-	.	gene_id "lnc-bbug02030" transcript_id "lnc-bbut00004788" exon_number "1"
10	lncRNA	exon	26938386	26938477	.	-	.	gene_id "lnc-bbug02030" transcript_id "lnc-bbut00004788" exon_number "2"
10	lncRNA	exon	27035948	27037254	.	-	.	gene_id "lnc-bbug02030" transcript_id "lnc-bbut00004788" exon_number "3"
10	lncRNA	exon	27394413	27395003	.	-	.	gene_id "lnc-bbug02033" transcript_id "lnc-bbut00004793" exon_number "1"
10	lncRNA	exon	27404203	27404269	.	-	.	gene_id "lnc-bbug02033" transcript_id "lnc-bbut00004793" exon_number "2"
10	lncRNA	exon	27409475	27409614	.	-	.	gene_id "lnc-bbug02033" transcript_id "lnc-bbut00004793" exon_number "3"
10	lncRNA	exon	27394413	27395003	.	-	.	gene_id "lnc-bbug02033" transcript_id "lnc-bbut00004794" exon_number "1"
10	lncRNA	exon	27404203	27404269	.	-	.	gene_id "lnc-bbug02033" transcript_id "lnc-bbut00004794" exon_number "2"
10	lncRNA	exon	27409467	27409615	.	-	.	gene_id "lnc-bbug02033" transcript_id "lnc-bbut00004794" exon_number "3"
10	lncRNA	exon	29866157	29867066	.	-	.	gene_id "lnc-bbug02044" transcript_id "lnc-bbut00004817" exon_number "1"
10	lncRNA	exon	29867485	29868711	.	-	.	gene_id "lnc-bbug02044" transcript_id "lnc-bbut00004817" exon_number "2"
10	lncRNA	exon	29871144	29871324	.	-	.	gene_id "lnc-bbug02044" transcript_id "lnc-bbut00004817" exon_number "3"
10	lncRNA	exon	31501355	31501604	.	-	.	gene_id "lnc-bbug02048" transcript_id "lnc-bbut00004825" exon_number "1"
10	lncRNA	exon	31511901	31511972	.	-	.	gene_id "lnc-bbug02048" transcript_id "lnc-bbut00004825" exon_number "2"
10	lncRNA	exon	31516809	31517238	.	-	.	gene_id "lnc-bbug02049" transcript_id "lnc-bbut00004826" exon_number "1"
10	lncRNA	exon	31706068	31706245	.	-	.	gene_id "lnc-bbug02050" transcript_id "lnc-bbut00004827" exon_number "1"
10	lncRNA	exon	31716992	31717131	.	-	.	gene_id "lnc-bbug02050" transcript_id "lnc-bbut00004827" exon_number "2"
10	lncRNA	exon	31717209	31717339	.	-	.	gene_id "lnc-bbug02050" transcript_id "lnc-bbut00004827" exon_number "3"
10	lncRNA	exon	31755422	31757448	.	-	.	gene_id "lnc-bbug02051" transcript_id "lnc-bbut00004828" exon_number "1"
10	lncRNA	exon	31758018	31758097	.	-	.	gene_id "lnc-bbug02051" transcript_id "lnc-bbut00004828" exon_number "2"
10	lncRNA	exon	31764542	31764654	.	-	.	gene_id "lnc-bbug02051" transcript_id "lnc-bbut00004828" exon_number "3"
10	lncRNA	exon	33219128	33220987	.	-	.	gene_id "lnc-bbug02056" transcript_id "lnc-bbut00004845" exon_number "1"
10	lncRNA	exon	33223958	33224108	.	-	.	gene_id "lnc-bbug02056" transcript_id "lnc-bbut00004845" exon_number "2"
10	lncRNA	exon	33224208	33224527	.	-	.	gene_id "lnc-bbug02056" transcript_id "lnc-bbut00004845" exon_number "3"
10	lncRNA	exon	34784238	34786551	.	-	.	gene_id "lnc-bbug02074" transcript_id "lnc-bbut00004866" exon_number "1"
10	lncRNA	exon	34799172	34799346	.	-	.	gene_id "lnc-bbug02074" transcript_id "lnc-bbut00004866" exon_number "2"
10	lncRNA	exon	34800793	34801589	.	-	.	gene_id "lnc-bbug02074" transcript_id "lnc-bbut00004866" exon_number "3"
10	lncRNA	exon	34784238	34786551	.	-	.	gene_id "lnc-bbug02074" transcript_id "lnc-bbut00004867" exon_number "1"
10	lncRNA	exon	34799172	34799346	.	-	.	gene_id "lnc-bbug02074" transcript_id "lnc-bbut00004867" exon_number "2"
10	lncRNA	exon	34827855	34831290	.	-	.	gene_id "lnc-bbug02074" transcript_id "lnc-bbut00004867" exon_number "3"
10	lncRNA	exon	34833853	34835880	.	-	.	gene_id "lnc-bbug02075" transcript_id "lnc-bbut00004868" exon_number "1"
10	lncRNA	exon	34864871	34864939	.	-	.	gene_id "lnc-bbug02075" transcript_id "lnc-bbut00004868" exon_number "2"
10	lncRNA	exon	34867278	34867377	.	-	.	gene_id "lnc-bbug02075" transcript_id "lnc-bbut00004868" exon_number "3"
10	lncRNA	exon	34833853	34835880	.	-	.	gene_id "lnc-bbug02075" transcript_id "lnc-bbut00004869" exon_number "1"
10	lncRNA	exon	34867278	34867386	.	-	.	gene_id "lnc-bbug02075" transcript_id "lnc-bbut00004869" exon_number "2"
10	lncRNA	exon	35224333	35226648	.	-	.	gene_id "lnc-bbug02076" transcript_id "lnc-bbut00004872" exon_number "1"
10	lncRNA	exon	35234046	35234317	.	-	.	gene_id "lnc-bbug02076" transcript_id "lnc-bbut00004872" exon_number "2"
10	lncRNA	exon	35235036	35235137	.	-	.	gene_id "lnc-bbug02076" transcript_id "lnc-bbut00004872" exon_number "3"
10	lncRNA	exon	36152127	36153816	.	-	.	gene_id "lnc-bbug02079" transcript_id "lnc-bbut00004883" exon_number "1"
10	lncRNA	exon	36154389	36154530	.	-	.	gene_id "lnc-bbug02079" transcript_id "lnc-bbut00004883" exon_number "2"
10	lncRNA	exon	36154639	36154980	.	-	.	gene_id "lnc-bbug02079" transcript_id "lnc-bbut00004883" exon_number "3"
10	lncRNA	exon	38271281	38272065	.	-	.	gene_id "lnc-bbug02081" transcript_id "lnc-bbut00004887" exon_number "1"

10	lncRNA	exon	38282078	38282192	.	-	.	gene_id "lnc-bbug02081"	transcript_id "lnc-bbut00004887"	exon_number "2"
10	lncRNA	exon	38282312	38282436	.	-	.	gene_id "lnc-bbug02081"	transcript_id "lnc-bbut00004887"	exon_number "3"
10	lncRNA	exon	40395702	40396515	.	-	.	gene_id "lnc-bbug02088"	transcript_id "lnc-bbut00004904"	exon_number "1"
10	lncRNA	exon	40400097	40400739	.	-	.	gene_id "lnc-bbug02088"	transcript_id "lnc-bbut00004904"	exon_number "2"
10	lncRNA	exon	40406119	40406414	.	-	.	gene_id "lnc-bbug02089"	transcript_id "lnc-bbut00004905"	exon_number "1"
10	lncRNA	exon	40406633	40406700	.	-	.	gene_id "lnc-bbug02089"	transcript_id "lnc-bbut00004905"	exon_number "2"
10	lncRNA	exon	40406966	40407027	.	-	.	gene_id "lnc-bbug02089"	transcript_id "lnc-bbut00004905"	exon_number "3"
10	lncRNA	exon	40407113	40407190	.	-	.	gene_id "lnc-bbug02089"	transcript_id "lnc-bbut00004905"	exon_number "4"
10	lncRNA	exon	40407299	40407369	.	-	.	gene_id "lnc-bbug02089"	transcript_id "lnc-bbut00004905"	exon_number "5"
10	lncRNA	exon	40407892	40407998	.	-	.	gene_id "lnc-bbug02089"	transcript_id "lnc-bbut00004905"	exon_number "6"
10	lncRNA	exon	41432225	41432577	.	-	.	gene_id "lnc-bbug02091"	transcript_id "lnc-bbut00004907"	exon_number "1"
10	lncRNA	exon	41433206	41433390	.	-	.	gene_id "lnc-bbug02091"	transcript_id "lnc-bbut00004907"	exon_number "2"
10	lncRNA	exon	41434501	41434607	.	-	.	gene_id "lnc-bbug02091"	transcript_id "lnc-bbut00004907"	exon_number "3"
10	lncRNA	exon	41448105	41448211	.	-	.	gene_id "lnc-bbug02091"	transcript_id "lnc-bbut00004907"	exon_number "4"
10	lncRNA	exon	41452199	41452275	.	-	.	gene_id "lnc-bbug02091"	transcript_id "lnc-bbut00004907"	exon_number "5"
10	lncRNA	exon	41432225	41432577	.	-	.	gene_id "lnc-bbug02091"	transcript_id "lnc-bbut00004908"	exon_number "1"
10	lncRNA	exon	41433206	41433390	.	-	.	gene_id "lnc-bbug02091"	transcript_id "lnc-bbut00004908"	exon_number "2"
10	lncRNA	exon	41434501	41434607	.	-	.	gene_id "lnc-bbug02091"	transcript_id "lnc-bbut00004908"	exon_number "3"
10	lncRNA	exon	41452199	41452329	.	-	.	gene_id "lnc-bbug02091"	transcript_id "lnc-bbut00004908"	exon_number "4"
10	lncRNA	exon	42027232	42037039	.	-	.	gene_id "lnc-bbug02098"	transcript_id "lnc-bbut00004918"	exon_number "1"
10	lncRNA	exon	42044754	42047711	.	-	.	gene_id "lnc-bbug02098"	transcript_id "lnc-bbut00004918"	exon_number "2"
10	lncRNA	exon	43518492	43518737	.	-	.	gene_id "lnc-bbug02106"	transcript_id "lnc-bbut00004933"	exon_number "1"
10	lncRNA	exon	43518924	43519043	.	-	.	gene_id "lnc-bbug02106"	transcript_id "lnc-bbut00004933"	exon_number "2"
10	lncRNA	exon	43519161	43519459	.	-	.	gene_id "lnc-bbug02106"	transcript_id "lnc-bbut00004933"	exon_number "3"
10	lncRNA	exon	43713325	43714206	.	-	.	gene_id "lnc-bbug02107"	transcript_id "lnc-bbut00004934"	exon_number "1"
10	lncRNA	exon	43714891	43715031	.	-	.	gene_id "lnc-bbug02107"	transcript_id "lnc-bbut00004934"	exon_number "2"
10	lncRNA	exon	43715516	43715639	.	-	.	gene_id "lnc-bbug02107"	transcript_id "lnc-bbut00004934"	exon_number "3"
10	lncRNA	exon	43799792	43801461	.	-	.	gene_id "lnc-bbug02109"	transcript_id "lnc-bbut00004939"	exon_number "1"
10	lncRNA	exon	44324263	44326080	.	-	.	gene_id "lnc-bbug02112"	transcript_id "lnc-bbut00004947"	exon_number "1"
10	lncRNA	exon	44327423	44328565	.	-	.	gene_id "lnc-bbug02112"	transcript_id "lnc-bbut00004947"	exon_number "2"
10	lncRNA	exon	51419971	51421186	.	-	.	gene_id "lnc-bbug02120"	transcript_id "lnc-bbut00004963"	exon_number "1"
10	lncRNA	exon	51424704	51424802	.	-	.	gene_id "lnc-bbug02120"	transcript_id "lnc-bbut00004963"	exon_number "2"
10	lncRNA	exon	51656100	51656302	.	-	.	gene_id "lnc-bbug02121"	transcript_id "lnc-bbut00004964"	exon_number "1"
10	lncRNA	exon	51664891	51665296	.	-	.	gene_id "lnc-bbug02121"	transcript_id "lnc-bbut00004964"	exon_number "2"
10	lncRNA	exon	52927305	52927904	.	-	.	gene_id "lnc-bbug02123"	transcript_id "lnc-bbut00004966"	exon_number "1"
10	lncRNA	exon	53706794	53707003	.	-	.	gene_id "lnc-bbug02125"	transcript_id "lnc-bbut00004970"	exon_number "1"
10	lncRNA	exon	53773279	53773353	.	-	.	gene_id "lnc-bbug02125"	transcript_id "lnc-bbut00004970"	exon_number "2"
10	lncRNA	exon	53773448	53773907	.	-	.	gene_id "lnc-bbug02125"	transcript_id "lnc-bbut00004970"	exon_number "3"
10	lncRNA	exon	53941953	53942289	.	-	.	gene_id "lnc-bbug02128"	transcript_id "lnc-bbut00004981"	exon_number "1"
10	lncRNA	exon	53942523	53942711	.	-	.	gene_id "lnc-bbug02128"	transcript_id "lnc-bbut00004981"	exon_number "2"
10	lncRNA	exon	53944331	53944449	.	-	.	gene_id "lnc-bbug02128"	transcript_id "lnc-bbut00004981"	exon_number "3"
10	lncRNA	exon	53946591	53946671	.	-	.	gene_id "lnc-bbug02128"	transcript_id "lnc-bbut00004981"	exon_number "4"
10	lncRNA	exon	53955931	53956694	.	-	.	gene_id "lnc-bbug02128"	transcript_id "lnc-bbut00004981"	exon_number "5"
10	lncRNA	exon	54462888	54463774	.	-	.	gene_id "lnc-bbug02133"	transcript_id "lnc-bbut00004993"	exon_number "1"
10	lncRNA	exon	54560389	54561458	.	-	.	gene_id "lnc-bbug02134"	transcript_id "lnc-bbut00004994"	exon_number "1"
10	lncRNA	exon	54682215	54682237	.	-	.	gene_id "lnc-bbug02134"	transcript_id "lnc-bbut00004994"	exon_number "2"
10	lncRNA	exon	54682537	54682730	.	-	.	gene_id "lnc-bbug02134"	transcript_id "lnc-bbut00004994"	exon_number "3"
10	lncRNA	exon	54663899	54665058	.	-	.	gene_id "lnc-bbug02134"	transcript_id "lnc-bbut00004995"	exon_number "1"
10	lncRNA	exon	54682215	54682237	.	-	.	gene_id "lnc-bbug02134"	transcript_id "lnc-bbut00004995"	exon_number "2"
10	lncRNA	exon	54682537	54682730	.	-	.	gene_id "lnc-bbug02134"	transcript_id "lnc-bbut00004995"	exon_number "3"
10	lncRNA	exon	55170537	55179411	.	-	.	gene_id "lnc-bbug02137"	transcript_id "lnc-bbut00005000"	exon_number "1"
10	lncRNA	exon	55179471	55180575	.	-	.	gene_id "lnc-bbug02137"	transcript_id "lnc-bbut00005000"	exon_number "2"

10	lncRNA	exon	55694043	55694887	.	-	.	gene_id "lnc-bbug02139"	transcript_id "lnc-bbut00005002"	exon_number "1"
10	lncRNA	exon	55707188	55707281	.	-	.	gene_id "lnc-bbug02139"	transcript_id "lnc-bbut00005002"	exon_number "2"
10	lncRNA	exon	55857955	55858508	.	-	.	gene_id "lnc-bbug02140"	transcript_id "lnc-bbut00005003"	exon_number "1"
10	lncRNA	exon	58603774	58605794	.	-	.	gene_id "lnc-bbug02141"	transcript_id "lnc-bbut00005004"	exon_number "1"
10	lncRNA	exon	58607883	58608012	.	-	.	gene_id "lnc-bbug02141"	transcript_id "lnc-bbut00005004"	exon_number "2"
10	lncRNA	exon	58609463	58609942	.	-	.	gene_id "lnc-bbug02141"	transcript_id "lnc-bbut00005004"	exon_number "3"
10	lncRNA	exon	58997410	58999466	.	-	.	gene_id "lnc-bbug02143"	transcript_id "lnc-bbut00005006"	exon_number "1"
10	lncRNA	exon	59025542	59025619	.	-	.	gene_id "lnc-bbug02144"	transcript_id "lnc-bbut00005007"	exon_number "1"
10	lncRNA	exon	59040185	59040282	.	-	.	gene_id "lnc-bbug02144"	transcript_id "lnc-bbut00005007"	exon_number "2"
10	lncRNA	exon	59043363	59043789	.	-	.	gene_id "lnc-bbug02144"	transcript_id "lnc-bbut00005007"	exon_number "3"
10	lncRNA	exon	59269506	59270579	.	-	.	gene_id "lnc-bbug02147"	transcript_id "lnc-bbut00005018"	exon_number "1"
10	lncRNA	exon	59275955	59276124	.	-	.	gene_id "lnc-bbug02147"	transcript_id "lnc-bbut00005018"	exon_number "2"
10	lncRNA	exon	59276674	59276839	.	-	.	gene_id "lnc-bbug02147"	transcript_id "lnc-bbut00005018"	exon_number "3"
10	lncRNA	exon	59278746	59278979	.	-	.	gene_id "lnc-bbug02147"	transcript_id "lnc-bbut00005018"	exon_number "4"
10	lncRNA	exon	59287757	59287970	.	-	.	gene_id "lnc-bbug02147"	transcript_id "lnc-bbut00005018"	exon_number "5"
10	lncRNA	exon	59269506	59270579	.	-	.	gene_id "lnc-bbug02147"	transcript_id "lnc-bbut00005017"	exon_number "1"
10	lncRNA	exon	59275955	59276124	.	-	.	gene_id "lnc-bbug02147"	transcript_id "lnc-bbut00005017"	exon_number "2"
10	lncRNA	exon	59276674	59276839	.	-	.	gene_id "lnc-bbug02147"	transcript_id "lnc-bbut00005017"	exon_number "3"
10	lncRNA	exon	59278746	59278860	.	-	.	gene_id "lnc-bbug02147"	transcript_id "lnc-bbut00005017"	exon_number "4"
10	lncRNA	exon	59287757	59287970	.	-	.	gene_id "lnc-bbug02147"	transcript_id "lnc-bbut00005017"	exon_number "5"
10	lncRNA	exon	59271980	59274249	.	-	.	gene_id "lnc-bbug02147"	transcript_id "lnc-bbut00005019"	exon_number "1"
10	lncRNA	exon	59275955	59276124	.	-	.	gene_id "lnc-bbug02147"	transcript_id "lnc-bbut00005019"	exon_number "2"
10	lncRNA	exon	59276674	59276839	.	-	.	gene_id "lnc-bbug02147"	transcript_id "lnc-bbut00005019"	exon_number "3"
10	lncRNA	exon	59278746	59278979	.	-	.	gene_id "lnc-bbug02147"	transcript_id "lnc-bbut00005019"	exon_number "4"
10	lncRNA	exon	59287757	59287971	.	-	.	gene_id "lnc-bbug02147"	transcript_id "lnc-bbut00005019"	exon_number "5"
10	lncRNA	exon	60460645	60462860	.	-	.	gene_id "lnc-bbug02153"	transcript_id "lnc-bbut00005031"	exon_number "1"
10	lncRNA	exon	60464229	60464796	.	-	.	gene_id "lnc-bbug02153"	transcript_id "lnc-bbut00005031"	exon_number "2"
10	lncRNA	exon	60607183	60610926	.	-	.	gene_id "lnc-bbug02154"	transcript_id "lnc-bbut00005032"	exon_number "1"
10	lncRNA	exon	61401473	61402563	.	-	.	gene_id "lnc-bbug02158"	transcript_id "lnc-bbut00005042"	exon_number "1"
10	lncRNA	exon	61403606	61403839	.	-	.	gene_id "lnc-bbug02158"	transcript_id "lnc-bbut00005042"	exon_number "2"
10	lncRNA	exon	61768346	61769700	.	-	.	gene_id "lnc-bbug02161"	transcript_id "lnc-bbut00005049"	exon_number "1"
10	lncRNA	exon	61770042	61771558	.	-	.	gene_id "lnc-bbug02161"	transcript_id "lnc-bbut00005049"	exon_number "2"
10	lncRNA	exon	62367690	62367752	.	-	.	gene_id "lnc-bbug02166"	transcript_id "lnc-bbut00005065"	exon_number "1"
10	lncRNA	exon	62368705	62369369	.	-	.	gene_id "lnc-bbug02166"	transcript_id "lnc-bbut00005065"	exon_number "2"
10	lncRNA	exon	62760318	62761563	.	-	.	gene_id "lnc-bbug02168"	transcript_id "lnc-bbut00005067"	exon_number "1"
10	lncRNA	exon	62762140	62762268	.	-	.	gene_id "lnc-bbug02168"	transcript_id "lnc-bbut00005067"	exon_number "2"
10	lncRNA	exon	63437078	63437255	.	-	.	gene_id "lnc-bbug02170"	transcript_id "lnc-bbut00005071"	exon_number "1"
10	lncRNA	exon	63441879	63441965	.	-	.	gene_id "lnc-bbug02170"	transcript_id "lnc-bbut00005071"	exon_number "2"
10	lncRNA	exon	63458012	63458767	.	-	.	gene_id "lnc-bbug02170"	transcript_id "lnc-bbut00005071"	exon_number "3"
10	lncRNA	exon	65969429	65969950	.	-	.	gene_id "lnc-bbug02185"	transcript_id "lnc-bbut00005126"	exon_number "1"
10	lncRNA	exon	65971266	65971419	.	-	.	gene_id "lnc-bbug02185"	transcript_id "lnc-bbut00005126"	exon_number "2"
10	lncRNA	exon	66202742	66204311	.	-	.	gene_id "lnc-bbug02188"	transcript_id "lnc-bbut00005132"	exon_number "1"
10	lncRNA	exon	67120020	67120043	.	-	.	gene_id "lnc-bbug02190"	transcript_id "lnc-bbut00005134"	exon_number "1"
10	lncRNA	exon	67217131	67217200	.	-	.	gene_id "lnc-bbug02190"	transcript_id "lnc-bbut00005134"	exon_number "2"
10	lncRNA	exon	67325411	67325758	.	-	.	gene_id "lnc-bbug02190"	transcript_id "lnc-bbut00005134"	exon_number "3"
10	lncRNA	exon	67379745	67380064	.	-	.	gene_id "lnc-bbug02191"	transcript_id "lnc-bbut00005135"	exon_number "1"
10	lncRNA	exon	67387993	67388086	.	-	.	gene_id "lnc-bbug02191"	transcript_id "lnc-bbut00005135"	exon_number "2"
10	lncRNA	exon	67391341	67391462	.	-	.	gene_id "lnc-bbug02191"	transcript_id "lnc-bbut00005135"	exon_number "3"
10	lncRNA	exon	67392212	67392290	.	-	.	gene_id "lnc-bbug02191"	transcript_id "lnc-bbut00005135"	exon_number "4"
10	lncRNA	exon	67412153	67414672	.	-	.	gene_id "lnc-bbug02191"	transcript_id "lnc-bbut00005135"	exon_number "5"
10	lncRNA	exon	67379745	67380064	.	-	.	gene_id "lnc-bbug02191"	transcript_id "lnc-bbut00005136"	exon_number "1"
10	lncRNA	exon	67385691	67385810	.	-	.	gene_id "lnc-bbug02191"	transcript_id "lnc-bbut00005136"	exon_number "2"

10	lncRNA	exon	67387993	67388086	.	-	.	gene_id "lnc-bbug02191"	transcript_id "lnc-bbut00005136"	exon_number "3"
10	lncRNA	exon	67391341	67391462	.	-	.	gene_id "lnc-bbug02191"	transcript_id "lnc-bbut00005136"	exon_number "4"
10	lncRNA	exon	67392212	67392290	.	-	.	gene_id "lnc-bbug02191"	transcript_id "lnc-bbut00005136"	exon_number "5"
10	lncRNA	exon	67412153	67414676	.	-	.	gene_id "lnc-bbug02191"	transcript_id "lnc-bbut00005136"	exon_number "6"
10	lncRNA	exon	67379745	67380064	.	-	.	gene_id "lnc-bbug02191"	transcript_id "lnc-bbut00005138"	exon_number "1"
10	lncRNA	exon	67385691	67386621	.	-	.	gene_id "lnc-bbug02191"	transcript_id "lnc-bbut00005138"	exon_number "2"
10	lncRNA	exon	67387993	67388086	.	-	.	gene_id "lnc-bbug02191"	transcript_id "lnc-bbut00005138"	exon_number "3"
10	lncRNA	exon	67388762	67388811	.	-	.	gene_id "lnc-bbug02191"	transcript_id "lnc-bbut00005138"	exon_number "4"
10	lncRNA	exon	67391341	67391462	.	-	.	gene_id "lnc-bbug02191"	transcript_id "lnc-bbut00005138"	exon_number "5"
10	lncRNA	exon	67392212	67392290	.	-	.	gene_id "lnc-bbug02191"	transcript_id "lnc-bbut00005138"	exon_number "6"
10	lncRNA	exon	67412153	67414683	.	-	.	gene_id "lnc-bbug02191"	transcript_id "lnc-bbut00005138"	exon_number "7"
10	lncRNA	exon	67379745	67380064	.	-	.	gene_id "lnc-bbug02191"	transcript_id "lnc-bbut00005137"	exon_number "1"
10	lncRNA	exon	67385691	67386621	.	-	.	gene_id "lnc-bbug02191"	transcript_id "lnc-bbut00005137"	exon_number "2"
10	lncRNA	exon	67387993	67388086	.	-	.	gene_id "lnc-bbug02191"	transcript_id "lnc-bbut00005137"	exon_number "3"
10	lncRNA	exon	67391341	67391462	.	-	.	gene_id "lnc-bbug02191"	transcript_id "lnc-bbut00005137"	exon_number "4"
10	lncRNA	exon	67392212	67392290	.	-	.	gene_id "lnc-bbug02191"	transcript_id "lnc-bbut00005137"	exon_number "5"
10	lncRNA	exon	67412153	67414683	.	-	.	gene_id "lnc-bbug02191"	transcript_id "lnc-bbut00005137"	exon_number "6"
10	lncRNA	exon	67858578	67859459	.	-	.	gene_id "lnc-bbug02192"	transcript_id "lnc-bbut00005139"	exon_number "1"
10	lncRNA	exon	67868900	67868951	.	-	.	gene_id "lnc-bbug02192"	transcript_id "lnc-bbut00005139"	exon_number "2"
10	lncRNA	exon	67875117	67875603	.	-	.	gene_id "lnc-bbug02192"	transcript_id "lnc-bbut00005139"	exon_number "3"
10	lncRNA	exon	69272669	69273522	.	-	.	gene_id "lnc-bbug02197"	transcript_id "lnc-bbut00005152"	exon_number "1"
10	lncRNA	exon	70508990	70510685	.	-	.	gene_id "lnc-bbug02204"	transcript_id "lnc-bbut00005164"	exon_number "1"
10	lncRNA	exon	70518482	70518590	.	-	.	gene_id "lnc-bbug02204"	transcript_id "lnc-bbut00005164"	exon_number "2"
10	lncRNA	exon	70520923	70521034	.	-	.	gene_id "lnc-bbug02204"	transcript_id "lnc-bbut00005164"	exon_number "3"
10	lncRNA	exon	70521171	70521394	.	-	.	gene_id "lnc-bbug02204"	transcript_id "lnc-bbut00005164"	exon_number "4"
10	lncRNA	exon	70542826	70544167	.	-	.	gene_id "lnc-bbug02205"	transcript_id "lnc-bbut00005165"	exon_number "1"
10	lncRNA	exon	70545004	70545127	.	-	.	gene_id "lnc-bbug02205"	transcript_id "lnc-bbut00005165"	exon_number "2"
10	lncRNA	exon	70547947	70548118	.	-	.	gene_id "lnc-bbug02205"	transcript_id "lnc-bbut00005165"	exon_number "3"
10	lncRNA	exon	70548847	70548886	.	-	.	gene_id "lnc-bbug02205"	transcript_id "lnc-bbut00005165"	exon_number "4"
10	lncRNA	exon	70562170	70562977	.	-	.	gene_id "lnc-bbug02205"	transcript_id "lnc-bbut00005165"	exon_number "5"
10	lncRNA	exon	71120325	71120379	.	-	.	gene_id "lnc-bbug02207"	transcript_id "lnc-bbut00005174"	exon_number "1"
10	lncRNA	exon	71122846	71124691	.	-	.	gene_id "lnc-bbug02207"	transcript_id "lnc-bbut00005174"	exon_number "2"
10	lncRNA	exon	71139772	71140688	.	-	.	gene_id "lnc-bbug02207"	transcript_id "lnc-bbut00005174"	exon_number "3"
10	lncRNA	exon	72433196	72433652	.	-	.	gene_id "lnc-bbug02214"	transcript_id "lnc-bbut00005195"	exon_number "1"
10	lncRNA	exon	72438077	72438277	.	-	.	gene_id "lnc-bbug02214"	transcript_id "lnc-bbut00005195"	exon_number "2"
10	lncRNA	exon	74574198	74574695	.	-	.	gene_id "lnc-bbug02218"	transcript_id "lnc-bbut00005203"	exon_number "1"
10	lncRNA	exon	74578924	74579143	.	-	.	gene_id "lnc-bbug02218"	transcript_id "lnc-bbut00005203"	exon_number "2"
10	lncRNA	exon	74581033	74581239	.	-	.	gene_id "lnc-bbug02218"	transcript_id "lnc-bbut00005203"	exon_number "3"
10	lncRNA	exon	75625856	75626530	.	-	.	gene_id "lnc-bbug02220"	transcript_id "lnc-bbut00005205"	exon_number "1"
10	lncRNA	exon	75627000	75627059	.	-	.	gene_id "lnc-bbug02220"	transcript_id "lnc-bbut00005205"	exon_number "2"
10	lncRNA	exon	75634150	75634307	.	-	.	gene_id "lnc-bbug02220"	transcript_id "lnc-bbut00005205"	exon_number "3"
10	lncRNA	exon	75683525	75683809	.	-	.	gene_id "lnc-bbug02221"	transcript_id "lnc-bbut00005206"	exon_number "1"
10	lncRNA	exon	75840926	75843285	.	-	.	gene_id "lnc-bbug02222"	transcript_id "lnc-bbut00005207"	exon_number "1"
10	lncRNA	exon	75864460	75864506	.	-	.	gene_id "lnc-bbug02222"	transcript_id "lnc-bbut00005207"	exon_number "2"
10	lncRNA	exon	75850814	75851880	.	-	.	gene_id "lnc-bbug02223"	transcript_id "lnc-bbut00005208"	exon_number "1"
10	lncRNA	exon	75855386	75857255	.	-	.	gene_id "lnc-bbug02223"	transcript_id "lnc-bbut00005208"	exon_number "2"
10	lncRNA	exon	81553654	81553934	.	-	.	gene_id "lnc-bbug02233"	transcript_id "lnc-bbut00005237"	exon_number "1"
10	lncRNA	exon	81579700	81579771	.	-	.	gene_id "lnc-bbug02233"	transcript_id "lnc-bbut00005237"	exon_number "2"
10	lncRNA	exon	81580020	81580151	.	-	.	gene_id "lnc-bbug02233"	transcript_id "lnc-bbut00005237"	exon_number "3"
10	lncRNA	exon	81553654	81553934	.	-	.	gene_id "lnc-bbug02233"	transcript_id "lnc-bbut00005238"	exon_number "1"
10	lncRNA	exon	81579700	81579771	.	-	.	gene_id "lnc-bbug02233"	transcript_id "lnc-bbut00005238"	exon_number "2"
10	lncRNA	exon	81580080	81580194	.	-	.	gene_id "lnc-bbug02233"	transcript_id "lnc-bbut00005238"	exon_number "3"

10	lncRNA	exon	83112857	83113010	.	-	.	gene_id "lnc-bbug02236"	transcript_id "lnc-bbut00005241"	exon_number "1"
10	lncRNA	exon	83126102	83126188	.	-	.	gene_id "lnc-bbug02236"	transcript_id "lnc-bbut00005241"	exon_number "2"
10	lncRNA	exon	83129406	83129881	.	-	.	gene_id "lnc-bbug02236"	transcript_id "lnc-bbut00005241"	exon_number "3"
10	lncRNA	exon	84429777	84430733	.	-	.	gene_id "lnc-bbug02238"	transcript_id "lnc-bbut00005244"	exon_number "1"
10	lncRNA	exon	84444048	84444051	.	-	.	gene_id "lnc-bbug02238"	transcript_id "lnc-bbut00005244"	exon_number "2"
10	lncRNA	exon	84448710	84448722	.	-	.	gene_id "lnc-bbug02238"	transcript_id "lnc-bbut00005244"	exon_number "3"
10	lncRNA	exon	84869213	84870125	.	-	.	gene_id "lnc-bbug02240"	transcript_id "lnc-bbut00005250"	exon_number "1"
10	lncRNA	exon	84881427	84881505	.	-	.	gene_id "lnc-bbug02240"	transcript_id "lnc-bbut00005250"	exon_number "2"
10	lncRNA	exon	84882703	84882753	.	-	.	gene_id "lnc-bbug02240"	transcript_id "lnc-bbut00005250"	exon_number "3"
10	lncRNA	exon	84918951	84919159	.	-	.	gene_id "lnc-bbug02241"	transcript_id "lnc-bbut00005251"	exon_number "1"
10	lncRNA	exon	84919976	84920196	.	-	.	gene_id "lnc-bbug02241"	transcript_id "lnc-bbut00005251"	exon_number "2"
10	lncRNA	exon	84920711	84920876	.	-	.	gene_id "lnc-bbug02241"	transcript_id "lnc-bbut00005251"	exon_number "3"
10	lncRNA	exon	85119905	85123862	.	-	.	gene_id "lnc-bbug02243"	transcript_id "lnc-bbut00005253"	exon_number "1"
10	lncRNA	exon	85123962	85124043	.	-	.	gene_id "lnc-bbug02243"	transcript_id "lnc-bbut00005253"	exon_number "2"
10	lncRNA	exon	85124294	85124683	.	-	.	gene_id "lnc-bbug02243"	transcript_id "lnc-bbut00005253"	exon_number "3"
10	lncRNA	exon	85638368	85640218	.	-	.	gene_id "lnc-bbug02244"	transcript_id "lnc-bbut00005255"	exon_number "1"
10	lncRNA	exon	85652334	85652440	.	-	.	gene_id "lnc-bbug02244"	transcript_id "lnc-bbut00005255"	exon_number "2"
10	lncRNA	exon	85672138	85672251	.	-	.	gene_id "lnc-bbug02244"	transcript_id "lnc-bbut00005255"	exon_number "3"
10	lncRNA	exon	85672634	85672970	.	-	.	gene_id "lnc-bbug02244"	transcript_id "lnc-bbut00005255"	exon_number "4"
10	lncRNA	exon	85638368	85640218	.	-	.	gene_id "lnc-bbug02244"	transcript_id "lnc-bbut00005254"	exon_number "1"
10	lncRNA	exon	85672138	85672251	.	-	.	gene_id "lnc-bbug02244"	transcript_id "lnc-bbut00005254"	exon_number "2"
10	lncRNA	exon	85672634	85672970	.	-	.	gene_id "lnc-bbug02244"	transcript_id "lnc-bbut00005254"	exon_number "3"
10	lncRNA	exon	89445962	89446218	.	-	.	gene_id "lnc-bbug02251"	transcript_id "lnc-bbut00005279"	exon_number "1"
10	lncRNA	exon	89446775	89446823	.	-	.	gene_id "lnc-bbug02251"	transcript_id "lnc-bbut00005279"	exon_number "2"
10	lncRNA	exon	89462079	89463459	.	-	.	gene_id "lnc-bbug02251"	transcript_id "lnc-bbut00005279"	exon_number "3"
10	lncRNA	exon	91277338	91280000	.	-	.	gene_id "lnc-bbug02257"	transcript_id "lnc-bbut00005285"	exon_number "1"
10	lncRNA	exon	91304979	91305091	.	-	.	gene_id "lnc-bbug02257"	transcript_id "lnc-bbut00005285"	exon_number "2"
10	lncRNA	exon	91310969	91311509	.	-	.	gene_id "lnc-bbug02257"	transcript_id "lnc-bbut00005285"	exon_number "3"
10	lncRNA	exon	92273038	92275094	.	-	.	gene_id "lnc-bbug02261"	transcript_id "lnc-bbut00005290"	exon_number "1"
10	lncRNA	exon	92277406	92282335	.	-	.	gene_id "lnc-bbug02261"	transcript_id "lnc-bbut00005290"	exon_number "2"
10	lncRNA	exon	93667422	93668244	.	-	.	gene_id "lnc-bbug02263"	transcript_id "lnc-bbut00005337"	exon_number "1"
10	lncRNA	exon	93675265	93675324	.	-	.	gene_id "lnc-bbug02263"	transcript_id "lnc-bbut00005337"	exon_number "2"
10	lncRNA	exon	93676461	93676528	.	-	.	gene_id "lnc-bbug02263"	transcript_id "lnc-bbut00005337"	exon_number "3"
10	lncRNA	exon	93690223	93690275	.	-	.	gene_id "lnc-bbug02263"	transcript_id "lnc-bbut00005337"	exon_number "4"
10	lncRNA	exon	93698499	93698569	.	-	.	gene_id "lnc-bbug02263"	transcript_id "lnc-bbut00005337"	exon_number "5"
10	lncRNA	exon	93698689	93703313	.	-	.	gene_id "lnc-bbug02263"	transcript_id "lnc-bbut00005337"	exon_number "6"
10	lncRNA	exon	93711700	93711907	.	-	.	gene_id "lnc-bbug02264"	transcript_id "lnc-bbut00005339"	exon_number "1"
10	lncRNA	exon	93714483	93714559	.	-	.	gene_id "lnc-bbug02264"	transcript_id "lnc-bbut00005339"	exon_number "2"
10	lncRNA	exon	93719264	93723201	.	-	.	gene_id "lnc-bbug02264"	transcript_id "lnc-bbut00005339"	exon_number "3"
10	lncRNA	exon	93736499	93736554	.	-	.	gene_id "lnc-bbug02264"	transcript_id "lnc-bbut00005339"	exon_number "4"
10	lncRNA	exon	93736849	93738017	.	-	.	gene_id "lnc-bbug02264"	transcript_id "lnc-bbut00005339"	exon_number "5"
10	lncRNA	exon	93738647	93738736	.	-	.	gene_id "lnc-bbug02264"	transcript_id "lnc-bbut00005339"	exon_number "6"
10	lncRNA	exon	93746381	93747017	.	-	.	gene_id "lnc-bbug02264"	transcript_id "lnc-bbut00005339"	exon_number "7"
10	lncRNA	exon	93711700	93711907	.	-	.	gene_id "lnc-bbug02264"	transcript_id "lnc-bbut00005338"	exon_number "1"
10	lncRNA	exon	93719264	93723201	.	-	.	gene_id "lnc-bbug02264"	transcript_id "lnc-bbut00005338"	exon_number "2"
10	lncRNA	exon	93736499	93736554	.	-	.	gene_id "lnc-bbug02264"	transcript_id "lnc-bbut00005338"	exon_number "3"
10	lncRNA	exon	93736849	93738017	.	-	.	gene_id "lnc-bbug02264"	transcript_id "lnc-bbut00005338"	exon_number "4"
10	lncRNA	exon	93738647	93738736	.	-	.	gene_id "lnc-bbug02264"	transcript_id "lnc-bbut00005338"	exon_number "5"
10	lncRNA	exon	93746381	93747017	.	-	.	gene_id "lnc-bbug02264"	transcript_id "lnc-bbut00005338"	exon_number "6"
10	lncRNA	exon	93712506	93714559	.	-	.	gene_id "lnc-bbug02264"	transcript_id "lnc-bbut00005340"	exon_number "1"
10	lncRNA	exon	93719264	93723201	.	-	.	gene_id "lnc-bbug02264"	transcript_id "lnc-bbut00005340"	exon_number "2"
10	lncRNA	exon	93736499	93736554	.	-	.	gene_id "lnc-bbug02264"	transcript_id "lnc-bbut00005340"	exon_number "3"

10	lncRNA	exon	93736849	93738017	.	-	.	gene_id "lnc-bbug02264"	transcript_id "lnc-bbut00005340"	exon_number "4"
10	lncRNA	exon	93738647	93738736	.	-	.	gene_id "lnc-bbug02264"	transcript_id "lnc-bbut00005340"	exon_number "5"
10	lncRNA	exon	93746381	93747017	.	-	.	gene_id "lnc-bbug02264"	transcript_id "lnc-bbut00005340"	exon_number "6"
10	lncRNA	exon	93716897	93723201	.	-	.	gene_id "lnc-bbug02264"	transcript_id "lnc-bbut00005341"	exon_number "1"
10	lncRNA	exon	93736499	93736554	.	-	.	gene_id "lnc-bbug02264"	transcript_id "lnc-bbut00005341"	exon_number "2"
10	lncRNA	exon	93736849	93738017	.	-	.	gene_id "lnc-bbug02264"	transcript_id "lnc-bbut00005341"	exon_number "3"
10	lncRNA	exon	93738647	93738736	.	-	.	gene_id "lnc-bbug02264"	transcript_id "lnc-bbut00005341"	exon_number "4"
10	lncRNA	exon	93746381	93747017	.	-	.	gene_id "lnc-bbug02264"	transcript_id "lnc-bbut00005341"	exon_number "5"
10	lncRNA	exon	93880730	93883589	.	-	.	gene_id "lnc-bbug02265"	transcript_id "lnc-bbut00005342"	exon_number "1"
10	lncRNA	exon	93897448	93897577	.	-	.	gene_id "lnc-bbug02265"	transcript_id "lnc-bbut00005342"	exon_number "2"
10	lncRNA	exon	93894137	93896798	.	-	.	gene_id "lnc-bbug02265"	transcript_id "lnc-bbut00005343"	exon_number "1"
10	lncRNA	exon	93897448	93897549	.	-	.	gene_id "lnc-bbug02265"	transcript_id "lnc-bbut00005343"	exon_number "2"
10	lncRNA	exon	93939116	93939530	.	-	.	gene_id "lnc-bbug02267"	transcript_id "lnc-bbut00005348"	exon_number "1"
10	lncRNA	exon	93939902	93943674	.	-	.	gene_id "lnc-bbug02267"	transcript_id "lnc-bbut00005348"	exon_number "2"
10	lncRNA	exon	93955410	93955580	.	-	.	gene_id "lnc-bbug02267"	transcript_id "lnc-bbut00005348"	exon_number "3"
10	lncRNA	exon	93957103	93957154	.	-	.	gene_id "lnc-bbug02267"	transcript_id "lnc-bbut00005348"	exon_number "4"
10	lncRNA	exon	93957359	93958390	.	-	.	gene_id "lnc-bbug02267"	transcript_id "lnc-bbut00005348"	exon_number "5"
10	lncRNA	exon	93939116	93943674	.	-	.	gene_id "lnc-bbug02267"	transcript_id "lnc-bbut00005347"	exon_number "1"
10	lncRNA	exon	93955410	93955580	.	-	.	gene_id "lnc-bbug02267"	transcript_id "lnc-bbut00005347"	exon_number "2"
10	lncRNA	exon	93957103	93957154	.	-	.	gene_id "lnc-bbug02267"	transcript_id "lnc-bbut00005347"	exon_number "3"
10	lncRNA	exon	93957359	93958390	.	-	.	gene_id "lnc-bbug02267"	transcript_id "lnc-bbut00005347"	exon_number "4"
10	lncRNA	exon	93939116	93943674	.	-	.	gene_id "lnc-bbug02267"	transcript_id "lnc-bbut00005349"	exon_number "1"
10	lncRNA	exon	93955410	93955580	.	-	.	gene_id "lnc-bbug02267"	transcript_id "lnc-bbut00005349"	exon_number "2"
10	lncRNA	exon	93957103	93957154	.	-	.	gene_id "lnc-bbug02267"	transcript_id "lnc-bbut00005349"	exon_number "3"
10	lncRNA	exon	93963440	93964123	.	-	.	gene_id "lnc-bbug02267"	transcript_id "lnc-bbut00005349"	exon_number "4"
10	lncRNA	exon	94571344	94572174	.	-	.	gene_id "lnc-bbug02269"	transcript_id "lnc-bbut00005355"	exon_number "1"
10	lncRNA	exon	94580322	94580346	.	-	.	gene_id "lnc-bbug02269"	transcript_id "lnc-bbut00005355"	exon_number "2"
10	lncRNA	exon	100517695	100518303	.	-	.	gene_id "lnc-bbug02277"	transcript_id "lnc-bbut00005369"	exon_number "1"
10	lncRNA	exon	104030726	104031447	.	-	.	gene_id "lnc-bbug02282"	transcript_id "lnc-bbut00005383"	exon_number "1"
11	lncRNA	exon	497183	497777	.	+	.	gene_id "lnc-bbug02289"	transcript_id "lnc-bbut00005398"	exon_number "1"
11	lncRNA	exon	499067	499174	.	+	.	gene_id "lnc-bbug02289"	transcript_id "lnc-bbut00005398"	exon_number "2"
11	lncRNA	exon	2995288	2995529	.	+	.	gene_id "lnc-bbug02297"	transcript_id "lnc-bbut00005450"	exon_number "1"
11	lncRNA	exon	2998360	3000092	.	+	.	gene_id "lnc-bbug02297"	transcript_id "lnc-bbut00005450"	exon_number "2"
11	lncRNA	exon	3351385	3351663	.	+	.	gene_id "lnc-bbug02300"	transcript_id "lnc-bbut00005457"	exon_number "1"
11	lncRNA	exon	3352256	3352394	.	+	.	gene_id "lnc-bbug02300"	transcript_id "lnc-bbut00005457"	exon_number "2"
11	lncRNA	exon	3352962	3353217	.	+	.	gene_id "lnc-bbug02300"	transcript_id "lnc-bbut00005457"	exon_number "3"
11	lncRNA	exon	3738036	3738148	.	+	.	gene_id "lnc-bbug02302"	transcript_id "lnc-bbut00005461"	exon_number "1"
11	lncRNA	exon	3738531	3738706	.	+	.	gene_id "lnc-bbug02302"	transcript_id "lnc-bbut00005461"	exon_number "2"
11	lncRNA	exon	3739401	3740276	.	+	.	gene_id "lnc-bbug02302"	transcript_id "lnc-bbut00005461"	exon_number "3"
11	lncRNA	exon	3742491	3742820	.	+	.	gene_id "lnc-bbug02303"	transcript_id "lnc-bbut00005462"	exon_number "1"
11	lncRNA	exon	3749094	3756033	.	+	.	gene_id "lnc-bbug02303"	transcript_id "lnc-bbut00005462"	exon_number "2"
11	lncRNA	exon	6429425	6429527	.	+	.	gene_id "lnc-bbug02305"	transcript_id "lnc-bbut00005464"	exon_number "1"
11	lncRNA	exon	6430171	6430258	.	+	.	gene_id "lnc-bbug02305"	transcript_id "lnc-bbut00005464"	exon_number "2"
11	lncRNA	exon	6431094	6431322	.	+	.	gene_id "lnc-bbug02305"	transcript_id "lnc-bbut00005464"	exon_number "3"
11	lncRNA	exon	6433228	6433333	.	+	.	gene_id "lnc-bbug02305"	transcript_id "lnc-bbut00005464"	exon_number "4"
11	lncRNA	exon	6438796	6438983	.	+	.	gene_id "lnc-bbug02305"	transcript_id "lnc-bbut00005464"	exon_number "5"
11	lncRNA	exon	8318869	8319236	.	+	.	gene_id "lnc-bbug02307"	transcript_id "lnc-bbut00005466"	exon_number "1"
11	lncRNA	exon	10432871	10433890	.	+	.	gene_id "lnc-bbug02310"	transcript_id "lnc-bbut00005470"	exon_number "1"
11	lncRNA	exon	10440331	10441529	.	+	.	gene_id "lnc-bbug02310"	transcript_id "lnc-bbut00005470"	exon_number "2"
11	lncRNA	exon	13042436	13042600	.	+	.	gene_id "lnc-bbug02316"	transcript_id "lnc-bbut00005478"	exon_number "1"
11	lncRNA	exon	13044238	13044392	.	+	.	gene_id "lnc-bbug02316"	transcript_id "lnc-bbut00005478"	exon_number "2"
11	lncRNA	exon	13049579	13049882	.	+	.	gene_id "lnc-bbug02316"	transcript_id "lnc-bbut00005478"	exon_number "3"

11	lncRNA	exon	14811987	14812107	.	+	.	gene_id "lnc-bbug02328"	transcript_id "lnc-bbut00005494"	exon_number "1"
11	lncRNA	exon	14815622	14816671	.	+	.	gene_id "lnc-bbug02328"	transcript_id "lnc-bbut00005494"	exon_number "2"
11	lncRNA	exon	14966090	14966139	.	+	.	gene_id "lnc-bbug02332"	transcript_id "lnc-bbut00005499"	exon_number "1"
11	lncRNA	exon	14967835	14967932	.	+	.	gene_id "lnc-bbug02332"	transcript_id "lnc-bbut00005499"	exon_number "2"
11	lncRNA	exon	14968981	14969046	.	+	.	gene_id "lnc-bbug02332"	transcript_id "lnc-bbut00005499"	exon_number "3"
11	lncRNA	exon	14969449	14969590	.	+	.	gene_id "lnc-bbug02332"	transcript_id "lnc-bbut00005499"	exon_number "4"
11	lncRNA	exon	14966090	14966139	.	+	.	gene_id "lnc-bbug02332"	transcript_id "lnc-bbut00005498"	exon_number "1"
11	lncRNA	exon	14967835	14967932	.	+	.	gene_id "lnc-bbug02332"	transcript_id "lnc-bbut00005498"	exon_number "2"
11	lncRNA	exon	14968981	14969046	.	+	.	gene_id "lnc-bbug02332"	transcript_id "lnc-bbut00005498"	exon_number "3"
11	lncRNA	exon	14969446	14969590	.	+	.	gene_id "lnc-bbug02332"	transcript_id "lnc-bbut00005498"	exon_number "4"
11	lncRNA	exon	15027150	15027929	.	+	.	gene_id "lnc-bbug02333"	transcript_id "lnc-bbut00005500"	exon_number "1"
11	lncRNA	exon	15032825	15033086	.	+	.	gene_id "lnc-bbug02333"	transcript_id "lnc-bbut00005500"	exon_number "2"
11	lncRNA	exon	15170979	15171498	.	+	.	gene_id "lnc-bbug02335"	transcript_id "lnc-bbut00005509"	exon_number "1"
11	lncRNA	exon	15173033	15173356	.	+	.	gene_id "lnc-bbug02335"	transcript_id "lnc-bbut00005509"	exon_number "2"
11	lncRNA	exon	15229150	15229288	.	+	.	gene_id "lnc-bbug02335"	transcript_id "lnc-bbut00005509"	exon_number "3"
11	lncRNA	exon	15262961	15263042	.	+	.	gene_id "lnc-bbug02335"	transcript_id "lnc-bbut00005509"	exon_number "4"
11	lncRNA	exon	15283932	15284224	.	+	.	gene_id "lnc-bbug02335"	transcript_id "lnc-bbut00005509"	exon_number "5"
11	lncRNA	exon	15171037	15171498	.	+	.	gene_id "lnc-bbug02335"	transcript_id "lnc-bbut00005510"	exon_number "1"
11	lncRNA	exon	15173033	15173356	.	+	.	gene_id "lnc-bbug02335"	transcript_id "lnc-bbut00005510"	exon_number "2"
11	lncRNA	exon	15229150	15229288	.	+	.	gene_id "lnc-bbug02335"	transcript_id "lnc-bbut00005510"	exon_number "3"
11	lncRNA	exon	15232012	15232062	.	+	.	gene_id "lnc-bbug02335"	transcript_id "lnc-bbut00005510"	exon_number "4"
11	lncRNA	exon	15171038	15171498	.	+	.	gene_id "lnc-bbug02335"	transcript_id "lnc-bbut00005511"	exon_number "1"
11	lncRNA	exon	15173033	15173356	.	+	.	gene_id "lnc-bbug02335"	transcript_id "lnc-bbut00005511"	exon_number "2"
11	lncRNA	exon	15229150	15229288	.	+	.	gene_id "lnc-bbug02335"	transcript_id "lnc-bbut00005511"	exon_number "3"
11	lncRNA	exon	15244066	15245563	.	+	.	gene_id "lnc-bbug02335"	transcript_id "lnc-bbut00005511"	exon_number "4"
11	lncRNA	exon	15732703	15732764	.	+	.	gene_id "lnc-bbug02336"	transcript_id "lnc-bbut00005512"	exon_number "1"
11	lncRNA	exon	15734109	15735708	.	+	.	gene_id "lnc-bbug02336"	transcript_id "lnc-bbut00005512"	exon_number "2"
11	lncRNA	exon	16228590	16228698	.	+	.	gene_id "lnc-bbug02338"	transcript_id "lnc-bbut00005515"	exon_number "1"
11	lncRNA	exon	16233421	16233511	.	+	.	gene_id "lnc-bbug02338"	transcript_id "lnc-bbut00005515"	exon_number "2"
11	lncRNA	exon	16245352	16246033	.	+	.	gene_id "lnc-bbug02338"	transcript_id "lnc-bbut00005515"	exon_number "3"
11	lncRNA	exon	16438462	16438575	.	+	.	gene_id "lnc-bbug02341"	transcript_id "lnc-bbut00005518"	exon_number "1"
11	lncRNA	exon	16441190	16441323	.	+	.	gene_id "lnc-bbug02341"	transcript_id "lnc-bbut00005518"	exon_number "2"
11	lncRNA	exon	16443033	16443198	.	+	.	gene_id "lnc-bbug02342"	transcript_id "lnc-bbut00005522"	exon_number "1"
11	lncRNA	exon	16443373	16443478	.	+	.	gene_id "lnc-bbug02342"	transcript_id "lnc-bbut00005522"	exon_number "2"
11	lncRNA	exon	16443627	16443738	.	+	.	gene_id "lnc-bbug02342"	transcript_id "lnc-bbut00005522"	exon_number "3"
11	lncRNA	exon	16444420	16444475	.	+	.	gene_id "lnc-bbug02342"	transcript_id "lnc-bbut00005522"	exon_number "4"
11	lncRNA	exon	16449050	16449664	.	+	.	gene_id "lnc-bbug02342"	transcript_id "lnc-bbut00005522"	exon_number "5"
11	lncRNA	exon	16465775	16465863	.	+	.	gene_id "lnc-bbug02343"	transcript_id "lnc-bbut00005523"	exon_number "1"
11	lncRNA	exon	16468480	16468644	.	+	.	gene_id "lnc-bbug02343"	transcript_id "lnc-bbut00005523"	exon_number "2"
11	lncRNA	exon	16470956	16471046	.	+	.	gene_id "lnc-bbug02343"	transcript_id "lnc-bbut00005523"	exon_number "3"
11	lncRNA	exon	16477563	16477681	.	+	.	gene_id "lnc-bbug02343"	transcript_id "lnc-bbut00005523"	exon_number "4"
11	lncRNA	exon	16477808	16479490	.	+	.	gene_id "lnc-bbug02343"	transcript_id "lnc-bbut00005523"	exon_number "5"
11	lncRNA	exon	16570805	16570902	.	+	.	gene_id "lnc-bbug02344"	transcript_id "lnc-bbut00005526"	exon_number "1"
11	lncRNA	exon	16572291	16572610	.	+	.	gene_id "lnc-bbug02344"	transcript_id "lnc-bbut00005526"	exon_number "2"
11	lncRNA	exon	16736640	16736730	.	+	.	gene_id "lnc-bbug02345"	transcript_id "lnc-bbut00005527"	exon_number "1"
11	lncRNA	exon	16737410	16737515	.	+	.	gene_id "lnc-bbug02345"	transcript_id "lnc-bbut00005527"	exon_number "2"
11	lncRNA	exon	16748027	16749012	.	+	.	gene_id "lnc-bbug02345"	transcript_id "lnc-bbut00005527"	exon_number "3"
11	lncRNA	exon	16749205	16749266	.	+	.	gene_id "lnc-bbug02345"	transcript_id "lnc-bbut00005527"	exon_number "4"
11	lncRNA	exon	16990277	16990407	.	+	.	gene_id "lnc-bbug02348"	transcript_id "lnc-bbut00005531"	exon_number "1"
11	lncRNA	exon	16990819	16990916	.	+	.	gene_id "lnc-bbug02348"	transcript_id "lnc-bbut00005531"	exon_number "2"
11	lncRNA	exon	16996155	16996584	.	+	.	gene_id "lnc-bbug02348"	transcript_id "lnc-bbut00005531"	exon_number "3"
11	lncRNA	exon	16990322	16990439	.	+	.	gene_id "lnc-bbug02348"	transcript_id "lnc-bbut00005532"	exon_number "1"

11	lncRNA	exon	16990819	16990916	.	+	.	gene_id "lnc-bbug02348"	transcript_id "lnc-bbut00005532"	exon_number "2"
11	lncRNA	exon	16996155	16996584	.	+	.	gene_id "lnc-bbug02348"	transcript_id "lnc-bbut00005532"	exon_number "3"
11	lncRNA	exon	17518719	17518881	.	+	.	gene_id "lnc-bbug02355"	transcript_id "lnc-bbut00005561"	exon_number "1"
11	lncRNA	exon	17522437	17522544	.	+	.	gene_id "lnc-bbug02355"	transcript_id "lnc-bbut00005561"	exon_number "2"
11	lncRNA	exon	17524102	17524274	.	+	.	gene_id "lnc-bbug02355"	transcript_id "lnc-bbut00005561"	exon_number "3"
11	lncRNA	exon	17526699	17527554	.	+	.	gene_id "lnc-bbug02355"	transcript_id "lnc-bbut00005561"	exon_number "4"
11	lncRNA	exon	18019486	18019596	.	+	.	gene_id "lnc-bbug02362"	transcript_id "lnc-bbut00005586"	exon_number "1"
11	lncRNA	exon	18024516	18024858	.	+	.	gene_id "lnc-bbug02362"	transcript_id "lnc-bbut00005586"	exon_number "2"
11	lncRNA	exon	18042810	18042908	.	+	.	gene_id "lnc-bbug02363"	transcript_id "lnc-bbut00005587"	exon_number "1"
11	lncRNA	exon	18044018	18044137	.	+	.	gene_id "lnc-bbug02363"	transcript_id "lnc-bbut00005587"	exon_number "2"
11	lncRNA	exon	18047278	18047327	.	+	.	gene_id "lnc-bbug02363"	transcript_id "lnc-bbut00005587"	exon_number "3"
11	lncRNA	exon	18602439	18602707	.	+	.	gene_id "lnc-bbug02368"	transcript_id "lnc-bbut00005603"	exon_number "1"
11	lncRNA	exon	18607032	18607184	.	+	.	gene_id "lnc-bbug02368"	transcript_id "lnc-bbut00005603"	exon_number "2"
11	lncRNA	exon	18612660	18613034	.	+	.	gene_id "lnc-bbug02368"	transcript_id "lnc-bbut00005603"	exon_number "3"
11	lncRNA	exon	19921222	19921880	.	+	.	gene_id "lnc-bbug02371"	transcript_id "lnc-bbut00005612"	exon_number "1"
11	lncRNA	exon	19922502	19923214	.	+	.	gene_id "lnc-bbug02371"	transcript_id "lnc-bbut00005612"	exon_number "2"
11	lncRNA	exon	20002583	20002663	.	+	.	gene_id "lnc-bbug02372"	transcript_id "lnc-bbut00005613"	exon_number "1"
11	lncRNA	exon	20004097	20004699	.	+	.	gene_id "lnc-bbug02372"	transcript_id "lnc-bbut00005613"	exon_number "2"
11	lncRNA	exon	20566937	20567522	.	+	.	gene_id "lnc-bbug02373"	transcript_id "lnc-bbut00005614"	exon_number "1"
11	lncRNA	exon	20568297	20568956	.	+	.	gene_id "lnc-bbug02373"	transcript_id "lnc-bbut00005614"	exon_number "2"
11	lncRNA	exon	20963848	20964448	.	+	.	gene_id "lnc-bbug02375"	transcript_id "lnc-bbut00005617"	exon_number "1"
11	lncRNA	exon	20979446	20979504	.	+	.	gene_id "lnc-bbug02375"	transcript_id "lnc-bbut00005617"	exon_number "2"
11	lncRNA	exon	20983926	20984055	.	+	.	gene_id "lnc-bbug02375"	transcript_id "lnc-bbut00005617"	exon_number "3"
11	lncRNA	exon	21130142	21130179	.	+	.	gene_id "lnc-bbug02379"	transcript_id "lnc-bbut00005624"	exon_number "1"
11	lncRNA	exon	21134408	21135719	.	+	.	gene_id "lnc-bbug02379"	transcript_id "lnc-bbut00005624"	exon_number "2"
11	lncRNA	exon	21332278	21334285	.	+	.	gene_id "lnc-bbug02380"	transcript_id "lnc-bbut00005625"	exon_number "1"
11	lncRNA	exon	21483199	21487058	.	+	.	gene_id "lnc-bbug02383"	transcript_id "lnc-bbut00005634"	exon_number "1"
11	lncRNA	exon	21489738	21491006	.	+	.	gene_id "lnc-bbug02383"	transcript_id "lnc-bbut00005634"	exon_number "2"
11	lncRNA	exon	23058186	23058296	.	+	.	gene_id "lnc-bbug02392"	transcript_id "lnc-bbut00005648"	exon_number "1"
11	lncRNA	exon	23062584	23062809	.	+	.	gene_id "lnc-bbug02392"	transcript_id "lnc-bbut00005648"	exon_number "2"
11	lncRNA	exon	23111252	23111868	.	+	.	gene_id "lnc-bbug02393"	transcript_id "lnc-bbut00005650"	exon_number "1"
11	lncRNA	exon	23148991	23149104	.	+	.	gene_id "lnc-bbug02393"	transcript_id "lnc-bbut00005650"	exon_number "2"
11	lncRNA	exon	23158862	23159566	.	+	.	gene_id "lnc-bbug02393"	transcript_id "lnc-bbut00005650"	exon_number "3"
11	lncRNA	exon	23160059	23160202	.	+	.	gene_id "lnc-bbug02393"	transcript_id "lnc-bbut00005650"	exon_number "4"
11	lncRNA	exon	23160723	23160814	.	+	.	gene_id "lnc-bbug02393"	transcript_id "lnc-bbut00005650"	exon_number "5"
11	lncRNA	exon	23165940	23165992	.	+	.	gene_id "lnc-bbug02393"	transcript_id "lnc-bbut00005650"	exon_number "6"
11	lncRNA	exon	23168721	23171895	.	+	.	gene_id "lnc-bbug02393"	transcript_id "lnc-bbut00005650"	exon_number "7"
11	lncRNA	exon	23111252	23111868	.	+	.	gene_id "lnc-bbug02393"	transcript_id "lnc-bbut00005649"	exon_number "1"
11	lncRNA	exon	23158862	23159566	.	+	.	gene_id "lnc-bbug02393"	transcript_id "lnc-bbut00005649"	exon_number "2"
11	lncRNA	exon	23160059	23160202	.	+	.	gene_id "lnc-bbug02393"	transcript_id "lnc-bbut00005649"	exon_number "3"
11	lncRNA	exon	23160723	23160814	.	+	.	gene_id "lnc-bbug02393"	transcript_id "lnc-bbut00005649"	exon_number "4"
11	lncRNA	exon	23165940	23165992	.	+	.	gene_id "lnc-bbug02393"	transcript_id "lnc-bbut00005649"	exon_number "5"
11	lncRNA	exon	23168721	23171895	.	+	.	gene_id "lnc-bbug02393"	transcript_id "lnc-bbut00005649"	exon_number "6"
11	lncRNA	exon	23899498	23899936	.	+	.	gene_id "lnc-bbug02397"	transcript_id "lnc-bbut00005657"	exon_number "1"
11	lncRNA	exon	23904884	23905023	.	+	.	gene_id "lnc-bbug02397"	transcript_id "lnc-bbut00005657"	exon_number "2"
11	lncRNA	exon	23905359	23906073	.	+	.	gene_id "lnc-bbug02397"	transcript_id "lnc-bbut00005657"	exon_number "3"
11	lncRNA	exon	23899498	23899875	.	+	.	gene_id "lnc-bbug02397"	transcript_id "lnc-bbut00005656"	exon_number "1"
11	lncRNA	exon	23904884	23905023	.	+	.	gene_id "lnc-bbug02397"	transcript_id "lnc-bbut00005656"	exon_number "2"
11	lncRNA	exon	23905359	23906073	.	+	.	gene_id "lnc-bbug02397"	transcript_id "lnc-bbut00005656"	exon_number "3"
11	lncRNA	exon	24010351	24010637	.	+	.	gene_id "lnc-bbug02399"	transcript_id "lnc-bbut00005662"	exon_number "1"
11	lncRNA	exon	24038842	24039023	.	+	.	gene_id "lnc-bbug02399"	transcript_id "lnc-bbut00005662"	exon_number "2"
11	lncRNA	exon	24039317	24041085	.	+	.	gene_id "lnc-bbug02399"	transcript_id "lnc-bbut00005662"	exon_number "3"

11	lncRNA	exon	24054343	24057884	.	+	.	gene_id "lnc-bbug02400"	transcript_id "lnc-bbut00005678"	exon_number "1"
11	lncRNA	exon	24057979	24058518	.	+	.	gene_id "lnc-bbug02400"	transcript_id "lnc-bbut00005678"	exon_number "2"
11	lncRNA	exon	25026187	25026335	.	+	.	gene_id "lnc-bbug02403"	transcript_id "lnc-bbut00005681"	exon_number "1"
11	lncRNA	exon	25072465	25072644	.	+	.	gene_id "lnc-bbug02403"	transcript_id "lnc-bbut00005681"	exon_number "2"
11	lncRNA	exon	25073485	25073548	.	+	.	gene_id "lnc-bbug02403"	transcript_id "lnc-bbut00005681"	exon_number "3"
11	lncRNA	exon	25088453	25088624	.	+	.	gene_id "lnc-bbug02403"	transcript_id "lnc-bbut00005681"	exon_number "4"
11	lncRNA	exon	26117396	26117582	.	+	.	gene_id "lnc-bbug02405"	transcript_id "lnc-bbut00005683"	exon_number "1"
11	lncRNA	exon	26117743	26118241	.	+	.	gene_id "lnc-bbug02405"	transcript_id "lnc-bbut00005683"	exon_number "2"
11	lncRNA	exon	26119023	26119902	.	+	.	gene_id "lnc-bbug02405"	transcript_id "lnc-bbut00005683"	exon_number "3"
11	lncRNA	exon	26184131	26184287	.	+	.	gene_id "lnc-bbug02406"	transcript_id "lnc-bbut00005688"	exon_number "1"
11	lncRNA	exon	26184474	26184597	.	+	.	gene_id "lnc-bbug02406"	transcript_id "lnc-bbut00005688"	exon_number "2"
11	lncRNA	exon	26185071	26185155	.	+	.	gene_id "lnc-bbug02406"	transcript_id "lnc-bbut00005688"	exon_number "3"
11	lncRNA	exon	26185843	26187359	.	+	.	gene_id "lnc-bbug02406"	transcript_id "lnc-bbut00005688"	exon_number "4"
11	lncRNA	exon	26291815	26292838	.	+	.	gene_id "lnc-bbug02409"	transcript_id "lnc-bbut00005691"	exon_number "1"
11	lncRNA	exon	26293591	26293856	.	+	.	gene_id "lnc-bbug02409"	transcript_id "lnc-bbut00005691"	exon_number "2"
11	lncRNA	exon	26314876	26315278	.	+	.	gene_id "lnc-bbug02410"	transcript_id "lnc-bbut00005692"	exon_number "1"
11	lncRNA	exon	26317765	26318436	.	+	.	gene_id "lnc-bbug02410"	transcript_id "lnc-bbut00005692"	exon_number "2"
11	lncRNA	exon	27101290	27101598	.	+	.	gene_id "lnc-bbug02416"	transcript_id "lnc-bbut00005717"	exon_number "1"
11	lncRNA	exon	27114011	27115245	.	+	.	gene_id "lnc-bbug02416"	transcript_id "lnc-bbut00005717"	exon_number "2"
11	lncRNA	exon	27498007	27498124	.	+	.	gene_id "lnc-bbug02418"	transcript_id "lnc-bbut00005719"	exon_number "1"
11	lncRNA	exon	27517111	27517235	.	+	.	gene_id "lnc-bbug02418"	transcript_id "lnc-bbut00005719"	exon_number "2"
11	lncRNA	exon	27575650	27576086	.	+	.	gene_id "lnc-bbug02418"	transcript_id "lnc-bbut00005719"	exon_number "3"
11	lncRNA	exon	27798649	27799677	.	+	.	gene_id "lnc-bbug02420"	transcript_id "lnc-bbut00005728"	exon_number "1"
11	lncRNA	exon	27803097	27804638	.	+	.	gene_id "lnc-bbug02420"	transcript_id "lnc-bbut00005728"	exon_number "2"
11	lncRNA	exon	29614390	29614603	.	+	.	gene_id "lnc-bbug02422"	transcript_id "lnc-bbut00005732"	exon_number "1"
11	lncRNA	exon	29668081	29668203	.	+	.	gene_id "lnc-bbug02422"	transcript_id "lnc-bbut00005732"	exon_number "2"
11	lncRNA	exon	29670450	29671004	.	+	.	gene_id "lnc-bbug02422"	transcript_id "lnc-bbut00005732"	exon_number "3"
11	lncRNA	exon	29700415	29700948	.	+	.	gene_id "lnc-bbug02423"	transcript_id "lnc-bbut00005733"	exon_number "1"
11	lncRNA	exon	29701855	29702092	.	+	.	gene_id "lnc-bbug02423"	transcript_id "lnc-bbut00005733"	exon_number "2"
11	lncRNA	exon	29923230	29924023	.	+	.	gene_id "lnc-bbug02424"	transcript_id "lnc-bbut00005735"	exon_number "1"
11	lncRNA	exon	29931109	29934261	.	+	.	gene_id "lnc-bbug02424"	transcript_id "lnc-bbut00005735"	exon_number "2"
11	lncRNA	exon	29934747	29935028	.	+	.	gene_id "lnc-bbug02424"	transcript_id "lnc-bbut00005735"	exon_number "3"
11	lncRNA	exon	29923230	29924023	.	+	.	gene_id "lnc-bbug02424"	transcript_id "lnc-bbut00005734"	exon_number "1"
11	lncRNA	exon	29931109	29931326	.	+	.	gene_id "lnc-bbug02424"	transcript_id "lnc-bbut00005734"	exon_number "2"
11	lncRNA	exon	29934133	29935028	.	+	.	gene_id "lnc-bbug02424"	transcript_id "lnc-bbut00005734"	exon_number "3"
11	lncRNA	exon	29923230	29924023	.	+	.	gene_id "lnc-bbug02424"	transcript_id "lnc-bbut00005736"	exon_number "1"
11	lncRNA	exon	29931109	29934261	.	+	.	gene_id "lnc-bbug02424"	transcript_id "lnc-bbut00005736"	exon_number "2"
11	lncRNA	exon	29939222	29941958	.	+	.	gene_id "lnc-bbug02424"	transcript_id "lnc-bbut00005736"	exon_number "3"
11	lncRNA	exon	30556506	30556737	.	+	.	gene_id "lnc-bbug02429"	transcript_id "lnc-bbut00005742"	exon_number "1"
11	lncRNA	exon	30557073	30557129	.	+	.	gene_id "lnc-bbug02430"	transcript_id "lnc-bbut00005743"	exon_number "1"
11	lncRNA	exon	30558773	30559335	.	+	.	gene_id "lnc-bbug02430"	transcript_id "lnc-bbut00005743"	exon_number "2"
11	lncRNA	exon	31708819	31708938	.	+	.	gene_id "lnc-bbug02439"	transcript_id "lnc-bbut00005758"	exon_number "1"
11	lncRNA	exon	31709377	31711061	.	+	.	gene_id "lnc-bbug02439"	transcript_id "lnc-bbut00005758"	exon_number "2"
11	lncRNA	exon	31923818	31924171	.	+	.	gene_id "lnc-bbug02440"	transcript_id "lnc-bbut00005759"	exon_number "1"
11	lncRNA	exon	31926129	31929629	.	+	.	gene_id "lnc-bbug02440"	transcript_id "lnc-bbut00005759"	exon_number "2"
11	lncRNA	exon	32695329	32695477	.	+	.	gene_id "lnc-bbug02442"	transcript_id "lnc-bbut00005762"	exon_number "1"
11	lncRNA	exon	32697504	32697592	.	+	.	gene_id "lnc-bbug02442"	transcript_id "lnc-bbut00005762"	exon_number "2"
11	lncRNA	exon	32697716	32697794	.	+	.	gene_id "lnc-bbug02442"	transcript_id "lnc-bbut00005762"	exon_number "3"
11	lncRNA	exon	32708234	32708297	.	+	.	gene_id "lnc-bbug02442"	transcript_id "lnc-bbut00005762"	exon_number "4"
11	lncRNA	exon	32708637	32708732	.	+	.	gene_id "lnc-bbug02442"	transcript_id "lnc-bbut00005762"	exon_number "5"
11	lncRNA	exon	32710906	32712171	.	+	.	gene_id "lnc-bbug02442"	transcript_id "lnc-bbut00005762"	exon_number "6"
11	lncRNA	exon	32695329	32695477	.	+	.	gene_id "lnc-bbug02442"	transcript_id "lnc-bbut00005761"	exon_number "1"

11	lncRNA	exon	32697504	32697592	.	+	.	gene_id "lnc-bbug02442"	transcript_id "lnc-bbut00005761"	exon_number "2"
11	lncRNA	exon	32708234	32708297	.	+	.	gene_id "lnc-bbug02442"	transcript_id "lnc-bbut00005761"	exon_number "3"
11	lncRNA	exon	32708637	32708732	.	+	.	gene_id "lnc-bbug02442"	transcript_id "lnc-bbut00005761"	exon_number "4"
11	lncRNA	exon	32710906	32712171	.	+	.	gene_id "lnc-bbug02442"	transcript_id "lnc-bbut00005761"	exon_number "5"
11	lncRNA	exon	32695516	32696539	.	+	.	gene_id "lnc-bbug02442"	transcript_id "lnc-bbut00005763"	exon_number "1"
11	lncRNA	exon	32697504	32697592	.	+	.	gene_id "lnc-bbug02442"	transcript_id "lnc-bbut00005763"	exon_number "2"
11	lncRNA	exon	32708637	32708732	.	+	.	gene_id "lnc-bbug02442"	transcript_id "lnc-bbut00005763"	exon_number "3"
11	lncRNA	exon	32710906	32712171	.	+	.	gene_id "lnc-bbug02442"	transcript_id "lnc-bbut00005763"	exon_number "4"
11	lncRNA	exon	32805195	32805255	.	+	.	gene_id "lnc-bbug02443"	transcript_id "lnc-bbut00005764"	exon_number "1"
11	lncRNA	exon	32806413	32806495	.	+	.	gene_id "lnc-bbug02443"	transcript_id "lnc-bbut00005764"	exon_number "2"
11	lncRNA	exon	32815807	32816586	.	+	.	gene_id "lnc-bbug02443"	transcript_id "lnc-bbut00005764"	exon_number "3"
11	lncRNA	exon	32895916	32896100	.	+	.	gene_id "lnc-bbug02445"	transcript_id "lnc-bbut00005766"	exon_number "1"
11	lncRNA	exon	32896189	32896324	.	+	.	gene_id "lnc-bbug02445"	transcript_id "lnc-bbut00005766"	exon_number "2"
11	lncRNA	exon	32896553	32897042	.	+	.	gene_id "lnc-bbug02445"	transcript_id "lnc-bbut00005766"	exon_number "3"
11	lncRNA	exon	32898327	32898369	.	+	.	gene_id "lnc-bbug02446"	transcript_id "lnc-bbut00005767"	exon_number "1"
11	lncRNA	exon	32900880	32900967	.	+	.	gene_id "lnc-bbug02446"	transcript_id "lnc-bbut00005767"	exon_number "2"
11	lncRNA	exon	32901345	32901639	.	+	.	gene_id "lnc-bbug02446"	transcript_id "lnc-bbut00005767"	exon_number "3"
11	lncRNA	exon	32911497	32912336	.	+	.	gene_id "lnc-bbug02447"	transcript_id "lnc-bbut00005768"	exon_number "1"
11	lncRNA	exon	35851756	35851878	.	+	.	gene_id "lnc-bbug02460"	transcript_id "lnc-bbut00005789"	exon_number "1"
11	lncRNA	exon	35886061	35886272	.	+	.	gene_id "lnc-bbug02460"	transcript_id "lnc-bbut00005789"	exon_number "2"
11	lncRNA	exon	36273010	36273265	.	+	.	gene_id "lnc-bbug02463"	transcript_id "lnc-bbut00005792"	exon_number "1"
11	lncRNA	exon	36280431	36280656	.	+	.	gene_id "lnc-bbug02463"	transcript_id "lnc-bbut00005792"	exon_number "2"
11	lncRNA	exon	36485354	36485890	.	+	.	gene_id "lnc-bbug02466"	transcript_id "lnc-bbut00005796"	exon_number "1"
11	lncRNA	exon	36498148	36502326	.	+	.	gene_id "lnc-bbug02466"	transcript_id "lnc-bbut00005796"	exon_number "2"
11	lncRNA	exon	38082881	38083018	.	+	.	gene_id "lnc-bbug02472"	transcript_id "lnc-bbut00005820"	exon_number "1"
11	lncRNA	exon	38086070	38086238	.	+	.	gene_id "lnc-bbug02472"	transcript_id "lnc-bbut00005820"	exon_number "2"
11	lncRNA	exon	38087269	38087483	.	+	.	gene_id "lnc-bbug02472"	transcript_id "lnc-bbut00005820"	exon_number "3"
11	lncRNA	exon	38087733	38088946	.	+	.	gene_id "lnc-bbug02472"	transcript_id "lnc-bbut00005820"	exon_number "4"
11	lncRNA	exon	38374268	38374404	.	+	.	gene_id "lnc-bbug02478"	transcript_id "lnc-bbut00005827"	exon_number "1"
11	lncRNA	exon	38374543	38374603	.	+	.	gene_id "lnc-bbug02478"	transcript_id "lnc-bbut00005827"	exon_number "2"
11	lncRNA	exon	38374758	38374843	.	+	.	gene_id "lnc-bbug02478"	transcript_id "lnc-bbut00005827"	exon_number "3"
11	lncRNA	exon	38376037	38376097	.	+	.	gene_id "lnc-bbug02478"	transcript_id "lnc-bbut00005827"	exon_number "4"
11	lncRNA	exon	38377642	38378008	.	+	.	gene_id "lnc-bbug02478"	transcript_id "lnc-bbut00005827"	exon_number "5"
11	lncRNA	exon	38390313	38390627	.	+	.	gene_id "lnc-bbug02479"	transcript_id "lnc-bbut00005828"	exon_number "1"
11	lncRNA	exon	38400258	38400328	.	+	.	gene_id "lnc-bbug02479"	transcript_id "lnc-bbut00005828"	exon_number "2"
11	lncRNA	exon	38411419	38413285	.	+	.	gene_id "lnc-bbug02479"	transcript_id "lnc-bbut00005828"	exon_number "3"
11	lncRNA	exon	38390313	38390627	.	+	.	gene_id "lnc-bbug02479"	transcript_id "lnc-bbut00005829"	exon_number "1"
11	lncRNA	exon	38400258	38400328	.	+	.	gene_id "lnc-bbug02479"	transcript_id "lnc-bbut00005829"	exon_number "2"
11	lncRNA	exon	38423561	38423594	.	+	.	gene_id "lnc-bbug02479"	transcript_id "lnc-bbut00005829"	exon_number "3"
11	lncRNA	exon	38430118	38430524	.	+	.	gene_id "lnc-bbug02479"	transcript_id "lnc-bbut00005829"	exon_number "4"
11	lncRNA	exon	38390313	38390627	.	+	.	gene_id "lnc-bbug02479"	transcript_id "lnc-bbut00005830"	exon_number "1"
11	lncRNA	exon	38400258	38400328	.	+	.	gene_id "lnc-bbug02479"	transcript_id "lnc-bbut00005830"	exon_number "2"
11	lncRNA	exon	38423561	38423594	.	+	.	gene_id "lnc-bbug02479"	transcript_id "lnc-bbut00005830"	exon_number "3"
11	lncRNA	exon	38430838	38432279	.	+	.	gene_id "lnc-bbug02479"	transcript_id "lnc-bbut00005830"	exon_number "4"
11	lncRNA	exon	38473310	38474395	.	+	.	gene_id "lnc-bbug02480"	transcript_id "lnc-bbut00005831"	exon_number "1"
11	lncRNA	exon	38474565	38474820	.	+	.	gene_id "lnc-bbug02480"	transcript_id "lnc-bbut00005831"	exon_number "2"
11	lncRNA	exon	38477846	38482440	.	+	.	gene_id "lnc-bbug02480"	transcript_id "lnc-bbut00005831"	exon_number "3"
11	lncRNA	exon	39388972	39389345	.	+	.	gene_id "lnc-bbug02483"	transcript_id "lnc-bbut00005837"	exon_number "1"
11	lncRNA	exon	39389406	39389858	.	+	.	gene_id "lnc-bbug02483"	transcript_id "lnc-bbut00005837"	exon_number "2"
11	lncRNA	exon	40984117	40984140	.	+	.	gene_id "lnc-bbug02485"	transcript_id "lnc-bbut00005849"	exon_number "1"
11	lncRNA	exon	41078609	41079267	.	+	.	gene_id "lnc-bbug02485"	transcript_id "lnc-bbut00005849"	exon_number "2"
11	lncRNA	exon	41187531	41187872	.	+	.	gene_id "lnc-bbug02487"	transcript_id "lnc-bbut00005851"	exon_number "1"

11	lncRNA	exon	41193269	41193762	.	+	.	gene_id "lnc-bbug02487"	transcript_id "lnc-bbut00005851"	exon_number "2"
11	lncRNA	exon	43830917	43833696	.	+	.	gene_id "lnc-bbug02495"	transcript_id "lnc-bbut00005875"	exon_number "1"
11	lncRNA	exon	44329142	44334349	.	+	.	gene_id "lnc-bbug02497"	transcript_id "lnc-bbut00005879"	exon_number "1"
11	lncRNA	exon	44336200	44336273	.	+	.	gene_id "lnc-bbug02497"	transcript_id "lnc-bbut00005879"	exon_number "2"
11	lncRNA	exon	44337517	44337642	.	+	.	gene_id "lnc-bbug02497"	transcript_id "lnc-bbut00005879"	exon_number "3"
11	lncRNA	exon	44434754	44434764	.	+	.	gene_id "lnc-bbug02499"	transcript_id "lnc-bbut00005881"	exon_number "1"
11	lncRNA	exon	44435675	44436140	.	+	.	gene_id "lnc-bbug02499"	transcript_id "lnc-bbut00005881"	exon_number "2"
11	lncRNA	exon	44436308	44437131	.	+	.	gene_id "lnc-bbug02499"	transcript_id "lnc-bbut00005881"	exon_number "3"
11	lncRNA	exon	44657207	44657210	.	+	.	gene_id "lnc-bbug02500"	transcript_id "lnc-bbut00005882"	exon_number "1"
11	lncRNA	exon	44658355	44658906	.	+	.	gene_id "lnc-bbug02500"	transcript_id "lnc-bbut00005882"	exon_number "2"
11	lncRNA	exon	45091304	45091864	.	+	.	gene_id "lnc-bbug02505"	transcript_id "lnc-bbut00005895"	exon_number "1"
11	lncRNA	exon	45111261	45111321	.	+	.	gene_id "lnc-bbug02505"	transcript_id "lnc-bbut00005895"	exon_number "2"
11	lncRNA	exon	45112078	45112266	.	+	.	gene_id "lnc-bbug02505"	transcript_id "lnc-bbut00005895"	exon_number "3"
11	lncRNA	exon	45112697	45112873	.	+	.	gene_id "lnc-bbug02505"	transcript_id "lnc-bbut00005895"	exon_number "4"
11	lncRNA	exon	45114497	45114600	.	+	.	gene_id "lnc-bbug02505"	transcript_id "lnc-bbut00005895"	exon_number "5"
11	lncRNA	exon	45114866	45114975	.	+	.	gene_id "lnc-bbug02505"	transcript_id "lnc-bbut00005895"	exon_number "6"
11	lncRNA	exon	45109093	45109134	.	+	.	gene_id "lnc-bbug02505"	transcript_id "lnc-bbut00005896"	exon_number "1"
11	lncRNA	exon	45111261	45111321	.	+	.	gene_id "lnc-bbug02505"	transcript_id "lnc-bbut00005896"	exon_number "2"
11	lncRNA	exon	45112078	45112266	.	+	.	gene_id "lnc-bbug02505"	transcript_id "lnc-bbut00005896"	exon_number "3"
11	lncRNA	exon	45112657	45112873	.	+	.	gene_id "lnc-bbug02505"	transcript_id "lnc-bbut00005896"	exon_number "4"
11	lncRNA	exon	45114497	45114600	.	+	.	gene_id "lnc-bbug02505"	transcript_id "lnc-bbut00005896"	exon_number "5"
11	lncRNA	exon	45114866	45114975	.	+	.	gene_id "lnc-bbug02505"	transcript_id "lnc-bbut00005896"	exon_number "6"
11	lncRNA	exon	45109093	45109134	.	+	.	gene_id "lnc-bbug02505"	transcript_id "lnc-bbut00005897"	exon_number "1"
11	lncRNA	exon	45111261	45111321	.	+	.	gene_id "lnc-bbug02505"	transcript_id "lnc-bbut00005897"	exon_number "2"
11	lncRNA	exon	45112078	45112266	.	+	.	gene_id "lnc-bbug02505"	transcript_id "lnc-bbut00005897"	exon_number "3"
11	lncRNA	exon	45112697	45112873	.	+	.	gene_id "lnc-bbug02505"	transcript_id "lnc-bbut00005897"	exon_number "4"
11	lncRNA	exon	45114497	45114600	.	+	.	gene_id "lnc-bbug02505"	transcript_id "lnc-bbut00005897"	exon_number "5"
11	lncRNA	exon	45114866	45114974	.	+	.	gene_id "lnc-bbug02505"	transcript_id "lnc-bbut00005897"	exon_number "6"
11	lncRNA	exon	45131228	45133117	.	+	.	gene_id "lnc-bbug02505"	transcript_id "lnc-bbut00005897"	exon_number "7"
11	lncRNA	exon	48109739	48109896	.	+	.	gene_id "lnc-bbug02509"	transcript_id "lnc-bbut00005901"	exon_number "1"
11	lncRNA	exon	48128889	48129006	.	+	.	gene_id "lnc-bbug02509"	transcript_id "lnc-bbut00005901"	exon_number "2"
11	lncRNA	exon	48166804	48167459	.	+	.	gene_id "lnc-bbug02509"	transcript_id "lnc-bbut00005901"	exon_number "3"
11	lncRNA	exon	48802681	48803277	.	+	.	gene_id "lnc-bbug02512"	transcript_id "lnc-bbut00005905"	exon_number "1"
11	lncRNA	exon	48813844	48815102	.	+	.	gene_id "lnc-bbug02512"	transcript_id "lnc-bbut00005905"	exon_number "2"
11	lncRNA	exon	50223289	50223725	.	+	.	gene_id "lnc-bbug02516"	transcript_id "lnc-bbut00005922"	exon_number "1"
11	lncRNA	exon	50224655	50224775	.	+	.	gene_id "lnc-bbug02516"	transcript_id "lnc-bbut00005922"	exon_number "2"
11	lncRNA	exon	50602143	50606163	.	+	.	gene_id "lnc-bbug02518"	transcript_id "lnc-bbut00005929"	exon_number "1"
11	lncRNA	exon	50630039	50630151	.	+	.	gene_id "lnc-bbug02518"	transcript_id "lnc-bbut00005929"	exon_number "2"
11	lncRNA	exon	50636057	50636141	.	+	.	gene_id "lnc-bbug02518"	transcript_id "lnc-bbut00005929"	exon_number "3"
11	lncRNA	exon	50644368	50644930	.	+	.	gene_id "lnc-bbug02518"	transcript_id "lnc-bbut00005929"	exon_number "4"
11	lncRNA	exon	50988742	50988909	.	+	.	gene_id "lnc-bbug02521"	transcript_id "lnc-bbut00005933"	exon_number "1"
11	lncRNA	exon	50993016	50993295	.	+	.	gene_id "lnc-bbug02521"	transcript_id "lnc-bbut00005933"	exon_number "2"
11	lncRNA	exon	51360103	51360350	.	+	.	gene_id "lnc-bbug02523"	transcript_id "lnc-bbut00005936"	exon_number "1"
11	lncRNA	exon	51374730	51374863	.	+	.	gene_id "lnc-bbug02523"	transcript_id "lnc-bbut00005936"	exon_number "2"
11	lncRNA	exon	51375167	51377684	.	+	.	gene_id "lnc-bbug02523"	transcript_id "lnc-bbut00005936"	exon_number "3"
11	lncRNA	exon	51372839	51374863	.	+	.	gene_id "lnc-bbug02523"	transcript_id "lnc-bbut00005937"	exon_number "1"
11	lncRNA	exon	51375167	51377684	.	+	.	gene_id "lnc-bbug02523"	transcript_id "lnc-bbut00005937"	exon_number "2"
11	lncRNA	exon	52302144	52302456	.	+	.	gene_id "lnc-bbug02528"	transcript_id "lnc-bbut00005949"	exon_number "1"
11	lncRNA	exon	52307125	52307251	.	+	.	gene_id "lnc-bbug02528"	transcript_id "lnc-bbut00005949"	exon_number "2"
11	lncRNA	exon	52308171	52308379	.	+	.	gene_id "lnc-bbug02528"	transcript_id "lnc-bbut00005949"	exon_number "3"
11	lncRNA	exon	52390096	52391474	.	+	.	gene_id "lnc-bbug02529"	transcript_id "lnc-bbut00005950"	exon_number "1"
11	lncRNA	exon	52396377	52398186	.	+	.	gene_id "lnc-bbug02529"	transcript_id "lnc-bbut00005950"	exon_number "2"

11	lncRNA	exon	52707832	52708802	.	+	.	gene_id "lnc-bbug02532"	transcript_id "lnc-bbut00005953"	exon_number "1"
11	lncRNA	exon	52723532	52724811	.	+	.	gene_id "lnc-bbug02532"	transcript_id "lnc-bbut00005953"	exon_number "2"
11	lncRNA	exon	52726034	52727360	.	+	.	gene_id "lnc-bbug02532"	transcript_id "lnc-bbut00005953"	exon_number "3"
11	lncRNA	exon	53374750	53375623	.	+	.	gene_id "lnc-bbug02534"	transcript_id "lnc-bbut00005955"	exon_number "1"
11	lncRNA	exon	53388614	53388696	.	+	.	gene_id "lnc-bbug02534"	transcript_id "lnc-bbut00005955"	exon_number "2"
11	lncRNA	exon	53398691	53399433	.	+	.	gene_id "lnc-bbug02534"	transcript_id "lnc-bbut00005955"	exon_number "3"
11	lncRNA	exon	53491104	53493160	.	+	.	gene_id "lnc-bbug02535"	transcript_id "lnc-bbut00005956"	exon_number "1"
11	lncRNA	exon	53798721	53798919	.	+	.	gene_id "lnc-bbug02536"	transcript_id "lnc-bbut00005957"	exon_number "1"
11	lncRNA	exon	53868734	53869154	.	+	.	gene_id "lnc-bbug02536"	transcript_id "lnc-bbut00005957"	exon_number "2"
11	lncRNA	exon	56704634	56704727	.	+	.	gene_id "lnc-bbug02543"	transcript_id "lnc-bbut00005993"	exon_number "1"
11	lncRNA	exon	56723690	56723797	.	+	.	gene_id "lnc-bbug02543"	transcript_id "lnc-bbut00005993"	exon_number "2"
11	lncRNA	exon	56729568	56730030	.	+	.	gene_id "lnc-bbug02543"	transcript_id "lnc-bbut00005993"	exon_number "3"
11	lncRNA	exon	57717012	57717088	.	+	.	gene_id "lnc-bbug02549"	transcript_id "lnc-bbut00006012"	exon_number "1"
11	lncRNA	exon	57717621	57718017	.	+	.	gene_id "lnc-bbug02549"	transcript_id "lnc-bbut00006012"	exon_number "2"
11	lncRNA	exon	58055735	58056240	.	+	.	gene_id "lnc-bbug02552"	transcript_id "lnc-bbut00006026"	exon_number "1"
11	lncRNA	exon	58063103	58063228	.	+	.	gene_id "lnc-bbug02552"	transcript_id "lnc-bbut00006026"	exon_number "2"
11	lncRNA	exon	58080718	58080887	.	+	.	gene_id "lnc-bbug02552"	transcript_id "lnc-bbut00006026"	exon_number "3"
11	lncRNA	exon	58082985	58083162	.	+	.	gene_id "lnc-bbug02552"	transcript_id "lnc-bbut00006026"	exon_number "4"
11	lncRNA	exon	58084117	58086479	.	+	.	gene_id "lnc-bbug02552"	transcript_id "lnc-bbut00006026"	exon_number "5"
11	lncRNA	exon	58079502	58080887	.	+	.	gene_id "lnc-bbug02552"	transcript_id "lnc-bbut00006027"	exon_number "1"
11	lncRNA	exon	58082985	58083162	.	+	.	gene_id "lnc-bbug02552"	transcript_id "lnc-bbut00006027"	exon_number "2"
11	lncRNA	exon	58084117	58086479	.	+	.	gene_id "lnc-bbug02552"	transcript_id "lnc-bbut00006027"	exon_number "3"
11	lncRNA	exon	58724562	58724747	.	+	.	gene_id "lnc-bbug02556"	transcript_id "lnc-bbut00006032"	exon_number "1"
11	lncRNA	exon	58731240	58732468	.	+	.	gene_id "lnc-bbug02556"	transcript_id "lnc-bbut00006032"	exon_number "2"
11	lncRNA	exon	59112979	59113064	.	+	.	gene_id "lnc-bbug02557"	transcript_id "lnc-bbut00006033"	exon_number "1"
11	lncRNA	exon	59115708	59115878	.	+	.	gene_id "lnc-bbug02557"	transcript_id "lnc-bbut00006033"	exon_number "2"
11	lncRNA	exon	59129708	59130013	.	+	.	gene_id "lnc-bbug02557"	transcript_id "lnc-bbut00006033"	exon_number "3"
11	lncRNA	exon	59647561	59655348	.	+	.	gene_id "lnc-bbug02560"	transcript_id "lnc-bbut00006048"	exon_number "1"
11	lncRNA	exon	59656108	59656179	.	+	.	gene_id "lnc-bbug02560"	transcript_id "lnc-bbut00006048"	exon_number "2"
11	lncRNA	exon	59656301	59656423	.	+	.	gene_id "lnc-bbug02560"	transcript_id "lnc-bbut00006048"	exon_number "3"
11	lncRNA	exon	59668791	59669323	.	+	.	gene_id "lnc-bbug02560"	transcript_id "lnc-bbut00006048"	exon_number "4"
11	lncRNA	exon	59647561	59656179	.	+	.	gene_id "lnc-bbug02560"	transcript_id "lnc-bbut00006047"	exon_number "1"
11	lncRNA	exon	59656301	59656423	.	+	.	gene_id "lnc-bbug02560"	transcript_id "lnc-bbut00006047"	exon_number "2"
11	lncRNA	exon	59668791	59669323	.	+	.	gene_id "lnc-bbug02560"	transcript_id "lnc-bbut00006047"	exon_number "3"
11	lncRNA	exon	60096366	60096670	.	+	.	gene_id "lnc-bbug02565"	transcript_id "lnc-bbut00006068"	exon_number "1"
11	lncRNA	exon	60097210	60097496	.	+	.	gene_id "lnc-bbug02565"	transcript_id "lnc-bbut00006068"	exon_number "2"
11	lncRNA	exon	60122613	60122734	.	+	.	gene_id "lnc-bbug02565"	transcript_id "lnc-bbut00006068"	exon_number "3"
11	lncRNA	exon	60134933	60135004	.	+	.	gene_id "lnc-bbug02565"	transcript_id "lnc-bbut00006068"	exon_number "4"
11	lncRNA	exon	60138252	60138342	.	+	.	gene_id "lnc-bbug02565"	transcript_id "lnc-bbut00006068"	exon_number "5"
11	lncRNA	exon	60139901	60140102	.	+	.	gene_id "lnc-bbug02565"	transcript_id "lnc-bbut00006068"	exon_number "6"
11	lncRNA	exon	60140500	60140582	.	+	.	gene_id "lnc-bbug02565"	transcript_id "lnc-bbut00006068"	exon_number "7"
11	lncRNA	exon	60143465	60143521	.	+	.	gene_id "lnc-bbug02565"	transcript_id "lnc-bbut00006068"	exon_number "8"
11	lncRNA	exon	60144681	60144836	.	+	.	gene_id "lnc-bbug02565"	transcript_id "lnc-bbut00006068"	exon_number "9"
11	lncRNA	exon	60146677	60148968	.	+	.	gene_id "lnc-bbug02565"	transcript_id "lnc-bbut00006068"	exon_number "10"
11	lncRNA	exon	60717700	60717830	.	+	.	gene_id "lnc-bbug02575"	transcript_id "lnc-bbut00006087"	exon_number "1"
11	lncRNA	exon	60719010	60719460	.	+	.	gene_id "lnc-bbug02575"	transcript_id "lnc-bbut00006087"	exon_number "2"
11	lncRNA	exon	60747159	60747319	.	+	.	gene_id "lnc-bbug02576"	transcript_id "lnc-bbut00006088"	exon_number "1"
11	lncRNA	exon	60747548	60747647	.	+	.	gene_id "lnc-bbug02576"	transcript_id "lnc-bbut00006088"	exon_number "2"
11	lncRNA	exon	60748976	60749079	.	+	.	gene_id "lnc-bbug02576"	transcript_id "lnc-bbut00006088"	exon_number "3"
11	lncRNA	exon	61213511	61213566	.	+	.	gene_id "lnc-bbug02577"	transcript_id "lnc-bbut00006090"	exon_number "1"
11	lncRNA	exon	61223766	61223940	.	+	.	gene_id "lnc-bbug02577"	transcript_id "lnc-bbut00006090"	exon_number "2"
11	lncRNA	exon	61225581	61225677	.	+	.	gene_id "lnc-bbug02577"	transcript_id "lnc-bbut00006090"	exon_number "3"

11	lncRNA	exon	61509558	61509750	.	+	.	gene_id "lnc-bbug02577"	transcript_id "lnc-bbut00006090"	exon_number "4"
11	lncRNA	exon	61560305	61560887	.	+	.	gene_id "lnc-bbug02577"	transcript_id "lnc-bbut00006090"	exon_number "5"
11	lncRNA	exon	61213511	61213566	.	+	.	gene_id "lnc-bbug02577"	transcript_id "lnc-bbut00006089"	exon_number "1"
11	lncRNA	exon	61223766	61223940	.	+	.	gene_id "lnc-bbug02577"	transcript_id "lnc-bbut00006089"	exon_number "2"
11	lncRNA	exon	61225502	61225677	.	+	.	gene_id "lnc-bbug02577"	transcript_id "lnc-bbut00006089"	exon_number "3"
11	lncRNA	exon	61509558	61509750	.	+	.	gene_id "lnc-bbug02577"	transcript_id "lnc-bbut00006089"	exon_number "4"
11	lncRNA	exon	61560305	61560887	.	+	.	gene_id "lnc-bbug02577"	transcript_id "lnc-bbut00006089"	exon_number "5"
11	lncRNA	exon	62648849	62651169	.	+	.	gene_id "lnc-bbug02578"	transcript_id "lnc-bbut00006091"	exon_number "1"
11	lncRNA	exon	63309979	63309993	.	+	.	gene_id "lnc-bbug02579"	transcript_id "lnc-bbut00006092"	exon_number "1"
11	lncRNA	exon	63310107	63310205	.	+	.	gene_id "lnc-bbug02579"	transcript_id "lnc-bbut00006092"	exon_number "2"
11	lncRNA	exon	63339162	63339870	.	+	.	gene_id "lnc-bbug02579"	transcript_id "lnc-bbut00006092"	exon_number "3"
11	lncRNA	exon	64279807	64279878	.	+	.	gene_id "lnc-bbug02581"	transcript_id "lnc-bbut00006094"	exon_number "1"
11	lncRNA	exon	64289204	64290137	.	+	.	gene_id "lnc-bbug02581"	transcript_id "lnc-bbut00006094"	exon_number "2"
11	lncRNA	exon	64596771	64597068	.	+	.	gene_id "lnc-bbug02584"	transcript_id "lnc-bbut00006097"	exon_number "1"
11	lncRNA	exon	65109600	65113266	.	+	.	gene_id "lnc-bbug02587"	transcript_id "lnc-bbut00006102"	exon_number "1"
11	lncRNA	exon	66352199	66352460	.	+	.	gene_id "lnc-bbug02599"	transcript_id "lnc-bbut00006134"	exon_number "1"
11	lncRNA	exon	66352623	66352832	.	+	.	gene_id "lnc-bbug02599"	transcript_id "lnc-bbut00006134"	exon_number "2"
11	lncRNA	exon	66975400	66975530	.	+	.	gene_id "lnc-bbug02607"	transcript_id "lnc-bbut00006156"	exon_number "1"
11	lncRNA	exon	66976406	66979245	.	+	.	gene_id "lnc-bbug02607"	transcript_id "lnc-bbut00006156"	exon_number "2"
11	lncRNA	exon	66987392	66987650	.	+	.	gene_id "lnc-bbug02607"	transcript_id "lnc-bbut00006156"	exon_number "3"
11	lncRNA	exon	66997597	66997629	.	+	.	gene_id "lnc-bbug02607"	transcript_id "lnc-bbut00006156"	exon_number "4"
11	lncRNA	exon	66997838	66998153	.	+	.	gene_id "lnc-bbug02607"	transcript_id "lnc-bbut00006156"	exon_number "5"
11	lncRNA	exon	67501469	67501654	.	+	.	gene_id "lnc-bbug02616"	transcript_id "lnc-bbut00006183"	exon_number "1"
11	lncRNA	exon	67507092	67507310	.	+	.	gene_id "lnc-bbug02616"	transcript_id "lnc-bbut00006183"	exon_number "2"
11	lncRNA	exon	67508455	67508530	.	+	.	gene_id "lnc-bbug02616"	transcript_id "lnc-bbut00006183"	exon_number "3"
11	lncRNA	exon	67509301	67509783	.	+	.	gene_id "lnc-bbug02616"	transcript_id "lnc-bbut00006183"	exon_number "4"
11	lncRNA	exon	68134704	68134810	.	+	.	gene_id "lnc-bbug02622"	transcript_id "lnc-bbut00006198"	exon_number "1"
11	lncRNA	exon	68136851	68137395	.	+	.	gene_id "lnc-bbug02622"	transcript_id "lnc-bbut00006198"	exon_number "2"
11	lncRNA	exon	69718077	69721303	.	+	.	gene_id "lnc-bbug02625"	transcript_id "lnc-bbut00006207"	exon_number "1"
11	lncRNA	exon	69722294	69722483	.	+	.	gene_id "lnc-bbug02625"	transcript_id "lnc-bbut00006207"	exon_number "2"
11	lncRNA	exon	69725870	69725915	.	+	.	gene_id "lnc-bbug02625"	transcript_id "lnc-bbut00006207"	exon_number "3"
11	lncRNA	exon	69725967	69726024	.	+	.	gene_id "lnc-bbug02626"	transcript_id "lnc-bbut00006208"	exon_number "1"
11	lncRNA	exon	69733138	69733344	.	+	.	gene_id "lnc-bbug02626"	transcript_id "lnc-bbut00006208"	exon_number "2"
11	lncRNA	exon	69734011	69734133	.	+	.	gene_id "lnc-bbug02626"	transcript_id "lnc-bbut00006208"	exon_number "3"
11	lncRNA	exon	69739422	69739470	.	+	.	gene_id "lnc-bbug02626"	transcript_id "lnc-bbut00006208"	exon_number "4"
11	lncRNA	exon	70824800	70825602	.	+	.	gene_id "lnc-bbug02628"	transcript_id "lnc-bbut00006218"	exon_number "1"
11	lncRNA	exon	70826305	70826473	.	+	.	gene_id "lnc-bbug02628"	transcript_id "lnc-bbut00006218"	exon_number "2"
11	lncRNA	exon	70842503	70842610	.	+	.	gene_id "lnc-bbug02628"	transcript_id "lnc-bbut00006218"	exon_number "3"
11	lncRNA	exon	71186974	71187252	.	+	.	gene_id "lnc-bbug02629"	transcript_id "lnc-bbut00006220"	exon_number "1"
11	lncRNA	exon	71188998	71189136	.	+	.	gene_id "lnc-bbug02629"	transcript_id "lnc-bbut00006220"	exon_number "2"
11	lncRNA	exon	71217710	71218128	.	+	.	gene_id "lnc-bbug02629"	transcript_id "lnc-bbut00006220"	exon_number "3"
11	lncRNA	exon	71250891	71251824	.	+	.	gene_id "lnc-bbug02629"	transcript_id "lnc-bbut00006220"	exon_number "4"
11	lncRNA	exon	71186974	71187252	.	+	.	gene_id "lnc-bbug02629"	transcript_id "lnc-bbut00006219"	exon_number "1"
11	lncRNA	exon	71188998	71189136	.	+	.	gene_id "lnc-bbug02629"	transcript_id "lnc-bbut00006219"	exon_number "2"
11	lncRNA	exon	71250891	71251824	.	+	.	gene_id "lnc-bbug02629"	transcript_id "lnc-bbut00006219"	exon_number "3"
11	lncRNA	exon	71366284	71367675	.	+	.	gene_id "lnc-bbug02630"	transcript_id "lnc-bbut00006221"	exon_number "1"
11	lncRNA	exon	71440361	71440438	.	+	.	gene_id "lnc-bbug02630"	transcript_id "lnc-bbut00006221"	exon_number "2"
11	lncRNA	exon	71570915	71571015	.	+	.	gene_id "lnc-bbug02630"	transcript_id "lnc-bbut00006221"	exon_number "3"
11	lncRNA	exon	71578061	71578234	.	+	.	gene_id "lnc-bbug02630"	transcript_id "lnc-bbut00006221"	exon_number "4"
11	lncRNA	exon	71582618	71582689	.	+	.	gene_id "lnc-bbug02630"	transcript_id "lnc-bbut00006221"	exon_number "5"
11	lncRNA	exon	71583300	71583375	.	+	.	gene_id "lnc-bbug02630"	transcript_id "lnc-bbut00006221"	exon_number "6"
11	lncRNA	exon	71584917	71585003	.	+	.	gene_id "lnc-bbug02630"	transcript_id "lnc-bbut00006221"	exon_number "7"

11	lncRNA	exon	71587070	71588538	.	+	.	gene_id "lnc-bbug02630"	transcript_id "lnc-bbut00006221"	exon_number "8"
11	lncRNA	exon	71429485	71429672	.	+	.	gene_id "lnc-bbug02630"	transcript_id "lnc-bbut00006222"	exon_number "1"
11	lncRNA	exon	71440361	71440438	.	+	.	gene_id "lnc-bbug02630"	transcript_id "lnc-bbut00006222"	exon_number "2"
11	lncRNA	exon	71570915	71571015	.	+	.	gene_id "lnc-bbug02630"	transcript_id "lnc-bbut00006222"	exon_number "3"
11	lncRNA	exon	71578061	71578234	.	+	.	gene_id "lnc-bbug02630"	transcript_id "lnc-bbut00006222"	exon_number "4"
11	lncRNA	exon	71582618	71582689	.	+	.	gene_id "lnc-bbug02630"	transcript_id "lnc-bbut00006222"	exon_number "5"
11	lncRNA	exon	71583300	71583375	.	+	.	gene_id "lnc-bbug02630"	transcript_id "lnc-bbut00006222"	exon_number "6"
11	lncRNA	exon	71584917	71585003	.	+	.	gene_id "lnc-bbug02630"	transcript_id "lnc-bbut00006222"	exon_number "7"
11	lncRNA	exon	71587070	71588538	.	+	.	gene_id "lnc-bbug02630"	transcript_id "lnc-bbut00006222"	exon_number "8"
11	lncRNA	exon	73951946	73952074	.	+	.	gene_id "lnc-bbug02640"	transcript_id "lnc-bbut00006238"	exon_number "1"
11	lncRNA	exon	73956425	73956529	.	+	.	gene_id "lnc-bbug02640"	transcript_id "lnc-bbut00006238"	exon_number "2"
11	lncRNA	exon	73960514	73961884	.	+	.	gene_id "lnc-bbug02640"	transcript_id "lnc-bbut00006238"	exon_number "3"
11	lncRNA	exon	75589630	75590569	.	+	.	gene_id "lnc-bbug02654"	transcript_id "lnc-bbut00006254"	exon_number "1"
11	lncRNA	exon	76653780	76654014	.	+	.	gene_id "lnc-bbug02682"	transcript_id "lnc-bbut00006282"	exon_number "1"
11	lncRNA	exon	76768255	76770198	.	+	.	gene_id "lnc-bbug02685"	transcript_id "lnc-bbut00006288"	exon_number "1"
11	lncRNA	exon	76809101	76809399	.	+	.	gene_id "lnc-bbug02687"	transcript_id "lnc-bbut00006290"	exon_number "1"
11	lncRNA	exon	76810224	76810255	.	+	.	gene_id "lnc-bbug02687"	transcript_id "lnc-bbut00006290"	exon_number "2"
11	lncRNA	exon	76810890	76811017	.	+	.	gene_id "lnc-bbug02687"	transcript_id "lnc-bbut00006290"	exon_number "3"
11	lncRNA	exon	76897467	76897769	.	+	.	gene_id "lnc-bbug02689"	transcript_id "lnc-bbut00006297"	exon_number "1"
11	lncRNA	exon	77280930	77281713	.	+	.	gene_id "lnc-bbug02705"	transcript_id "lnc-bbut00006322"	exon_number "1"
11	lncRNA	exon	77281904	77282034	.	+	.	gene_id "lnc-bbug02705"	transcript_id "lnc-bbut00006322"	exon_number "2"
11	lncRNA	exon	77282264	77282309	.	+	.	gene_id "lnc-bbug02705"	transcript_id "lnc-bbut00006322"	exon_number "3"
11	lncRNA	exon	77282603	77282793	.	+	.	gene_id "lnc-bbug02705"	transcript_id "lnc-bbut00006322"	exon_number "4"
11	lncRNA	exon	77337845	77338913	.	+	.	gene_id "lnc-bbug02708"	transcript_id "lnc-bbut00006333"	exon_number "1"
11	lncRNA	exon	77639724	77640059	.	+	.	gene_id "lnc-bbug02712"	transcript_id "lnc-bbut00006341"	exon_number "1"
11	lncRNA	exon	77727655	77727790	.	+	.	gene_id "lnc-bbug02714"	transcript_id "lnc-bbut00006345"	exon_number "1"
11	lncRNA	exon	77730749	77730852	.	+	.	gene_id "lnc-bbug02714"	transcript_id "lnc-bbut00006345"	exon_number "2"
11	lncRNA	exon	77738237	77738405	.	+	.	gene_id "lnc-bbug02714"	transcript_id "lnc-bbut00006345"	exon_number "3"
11	lncRNA	exon	77983154	77983573	.	+	.	gene_id "lnc-bbug02722"	transcript_id "lnc-bbut00006354"	exon_number "1"
11	lncRNA	exon	77983682	77984182	.	+	.	gene_id "lnc-bbug02722"	transcript_id "lnc-bbut00006354"	exon_number "2"
11	lncRNA	exon	78062617	78062814	.	+	.	gene_id "lnc-bbug02726"	transcript_id "lnc-bbut00006358"	exon_number "1"
11	lncRNA	exon	78062907	78063382	.	+	.	gene_id "lnc-bbug02726"	transcript_id "lnc-bbut00006358"	exon_number "2"
11	lncRNA	exon	78140686	78141607	.	+	.	gene_id "lnc-bbug02730"	transcript_id "lnc-bbut00006363"	exon_number "1"
11	lncRNA	exon	78154310	78154520	.	+	.	gene_id "lnc-bbug02731"	transcript_id "lnc-bbut00006365"	exon_number "1"
11	lncRNA	exon	78154712	78155527	.	+	.	gene_id "lnc-bbug02731"	transcript_id "lnc-bbut00006365"	exon_number "2"
11	lncRNA	exon	78159921	78160884	.	+	.	gene_id "lnc-bbug02732"	transcript_id "lnc-bbut00006366"	exon_number "1"
11	lncRNA	exon	78177911	78177962	.	+	.	gene_id "lnc-bbug02733"	transcript_id "lnc-bbut00006368"	exon_number "1"
11	lncRNA	exon	78178186	78178637	.	+	.	gene_id "lnc-bbug02733"	transcript_id "lnc-bbut00006368"	exon_number "2"
11	lncRNA	exon	78190544	78191472	.	+	.	gene_id "lnc-bbug02734"	transcript_id "lnc-bbut00006369"	exon_number "1"
11	lncRNA	exon	78215839	78215961	.	+	.	gene_id "lnc-bbug02735"	transcript_id "lnc-bbut00006371"	exon_number "1"
11	lncRNA	exon	78216218	78216462	.	+	.	gene_id "lnc-bbug02735"	transcript_id "lnc-bbut00006371"	exon_number "2"
11	lncRNA	exon	78239294	78240192	.	+	.	gene_id "lnc-bbug02737"	transcript_id "lnc-bbut00006373"	exon_number "1"
11	lncRNA	exon	78262102	78262152	.	+	.	gene_id "lnc-bbug02740"	transcript_id "lnc-bbut00006377"	exon_number "1"
11	lncRNA	exon	78262345	78262669	.	+	.	gene_id "lnc-bbug02740"	transcript_id "lnc-bbut00006377"	exon_number "2"
11	lncRNA	exon	78276115	78276978	.	+	.	gene_id "lnc-bbug02741"	transcript_id "lnc-bbut00006378"	exon_number "1"
11	lncRNA	exon	78325908	78327642	.	+	.	gene_id "lnc-bbug02743"	transcript_id "lnc-bbut00006382"	exon_number "1"
11	lncRNA	exon	78338565	78341868	.	+	.	gene_id "lnc-bbug02744"	transcript_id "lnc-bbut00006384"	exon_number "1"
11	lncRNA	exon	78381908	78382670	.	+	.	gene_id "lnc-bbug02746"	transcript_id "lnc-bbut00006388"	exon_number "1"
11	lncRNA	exon	78405212	78405263	.	+	.	gene_id "lnc-bbug02747"	transcript_id "lnc-bbut00006390"	exon_number "1"
11	lncRNA	exon	78405630	78406002	.	+	.	gene_id "lnc-bbug02747"	transcript_id "lnc-bbut00006390"	exon_number "2"
11	lncRNA	exon	78407035	78407062	.	+	.	gene_id "lnc-bbug02747"	transcript_id "lnc-bbut00006390"	exon_number "3"
11	lncRNA	exon	78415658	78417614	.	+	.	gene_id "lnc-bbug02749"	transcript_id "lnc-bbut00006393"	exon_number "1"

11	lncRNA	exon	78423316	78426175	.	+	.	gene_id "lnc-bbug02750"	transcript_id "lnc-bbut00006395"	exon_number "1"
11	lncRNA	exon	78438381	78438769	.	+	.	gene_id "lnc-bbug02751"	transcript_id "lnc-bbut00006397"	exon_number "1"
11	lncRNA	exon	78438939	78439820	.	+	.	gene_id "lnc-bbug02751"	transcript_id "lnc-bbut00006397"	exon_number "2"
11	lncRNA	exon	78458447	78458673	.	+	.	gene_id "lnc-bbug02752"	transcript_id "lnc-bbut00006398"	exon_number "1"
11	lncRNA	exon	78458842	78459285	.	+	.	gene_id "lnc-bbug02752"	transcript_id "lnc-bbut00006398"	exon_number "2"
11	lncRNA	exon	78464044	78464343	.	+	.	gene_id "lnc-bbug02753"	transcript_id "lnc-bbut00006399"	exon_number "1"
11	lncRNA	exon	78465070	78465388	.	+	.	gene_id "lnc-bbug02753"	transcript_id "lnc-bbut00006399"	exon_number "2"
11	lncRNA	exon	78486763	78487749	.	+	.	gene_id "lnc-bbug02754"	transcript_id "lnc-bbut00006400"	exon_number "1"
11	lncRNA	exon	78538398	78539141	.	+	.	gene_id "lnc-bbug02758"	transcript_id "lnc-bbut00006405"	exon_number "1"
11	lncRNA	exon	78548264	78549672	.	+	.	gene_id "lnc-bbug02759"	transcript_id "lnc-bbut00006407"	exon_number "1"
11	lncRNA	exon	78551572	78551687	.	+	.	gene_id "lnc-bbug02760"	transcript_id "lnc-bbut00006409"	exon_number "1"
11	lncRNA	exon	78551880	78552494	.	+	.	gene_id "lnc-bbug02760"	transcript_id "lnc-bbut00006409"	exon_number "2"
11	lncRNA	exon	78568900	78579565	.	+	.	gene_id "lnc-bbug02762"	transcript_id "lnc-bbut00006411"	exon_number "1"
11	lncRNA	exon	78586949	78588453	.	+	.	gene_id "lnc-bbug02763"	transcript_id "lnc-bbut00006413"	exon_number "1"
11	lncRNA	exon	78629013	78629205	.	+	.	gene_id "lnc-bbug02767"	transcript_id "lnc-bbut00006419"	exon_number "1"
11	lncRNA	exon	78629401	78629982	.	+	.	gene_id "lnc-bbug02767"	transcript_id "lnc-bbut00006419"	exon_number "2"
11	lncRNA	exon	78652001	78652162	.	+	.	gene_id "lnc-bbug02769"	transcript_id "lnc-bbut00006422"	exon_number "1"
11	lncRNA	exon	78652673	78653194	.	+	.	gene_id "lnc-bbug02769"	transcript_id "lnc-bbut00006422"	exon_number "2"
11	lncRNA	exon	78662836	78664292	.	+	.	gene_id "lnc-bbug02770"	transcript_id "lnc-bbut00006423"	exon_number "1"
11	lncRNA	exon	78664462	78664800	.	+	.	gene_id "lnc-bbug02770"	transcript_id "lnc-bbut00006423"	exon_number "2"
11	lncRNA	exon	78745036	78745607	.	+	.	gene_id "lnc-bbug02773"	transcript_id "lnc-bbut00006426"	exon_number "1"
11	lncRNA	exon	78745766	78746246	.	+	.	gene_id "lnc-bbug02773"	transcript_id "lnc-bbut00006426"	exon_number "2"
11	lncRNA	exon	78774681	78775594	.	+	.	gene_id "lnc-bbug02774"	transcript_id "lnc-bbut00006427"	exon_number "1"
11	lncRNA	exon	78795873	78796172	.	+	.	gene_id "lnc-bbug02775"	transcript_id "lnc-bbut00006429"	exon_number "1"
11	lncRNA	exon	78798037	78798219	.	+	.	gene_id "lnc-bbug02775"	transcript_id "lnc-bbut00006429"	exon_number "2"
11	lncRNA	exon	78798418	78798535	.	+	.	gene_id "lnc-bbug02775"	transcript_id "lnc-bbut00006429"	exon_number "3"
11	lncRNA	exon	78806372	78806576	.	+	.	gene_id "lnc-bbug02775"	transcript_id "lnc-bbut00006429"	exon_number "4"
11	lncRNA	exon	78809991	78810098	.	+	.	gene_id "lnc-bbug02775"	transcript_id "lnc-bbut00006429"	exon_number "5"
11	lncRNA	exon	78828185	78828765	.	+	.	gene_id "lnc-bbug02777"	transcript_id "lnc-bbut00006432"	exon_number "1"
11	lncRNA	exon	78828921	78829573	.	+	.	gene_id "lnc-bbug02777"	transcript_id "lnc-bbut00006432"	exon_number "2"
11	lncRNA	exon	78836786	78837053	.	+	.	gene_id "lnc-bbug02778"	transcript_id "lnc-bbut00006433"	exon_number "1"
11	lncRNA	exon	78837169	78837529	.	+	.	gene_id "lnc-bbug02778"	transcript_id "lnc-bbut00006433"	exon_number "2"
11	lncRNA	exon	78865152	78865521	.	+	.	gene_id "lnc-bbug02782"	transcript_id "lnc-bbut00006438"	exon_number "1"
11	lncRNA	exon	78865647	78866773	.	+	.	gene_id "lnc-bbug02782"	transcript_id "lnc-bbut00006438"	exon_number "2"
11	lncRNA	exon	78912507	78913162	.	+	.	gene_id "lnc-bbug02784"	transcript_id "lnc-bbut00006441"	exon_number "1"
11	lncRNA	exon	78967406	78968836	.	+	.	gene_id "lnc-bbug02787"	transcript_id "lnc-bbut00006445"	exon_number "1"
11	lncRNA	exon	78999537	78999689	.	+	.	gene_id "lnc-bbug02790"	transcript_id "lnc-bbut00006449"	exon_number "1"
11	lncRNA	exon	78999858	79000515	.	+	.	gene_id "lnc-bbug02790"	transcript_id "lnc-bbut00006449"	exon_number "2"
11	lncRNA	exon	79268978	79270798	.	+	.	gene_id "lnc-bbug02792"	transcript_id "lnc-bbut00006451"	exon_number "1"
11	lncRNA	exon	79280007	79281399	.	+	.	gene_id "lnc-bbug02793"	transcript_id "lnc-bbut00006453"	exon_number "1"
11	lncRNA	exon	79281598	79282173	.	+	.	gene_id "lnc-bbug02793"	transcript_id "lnc-bbut00006453"	exon_number "2"
11	lncRNA	exon	79293000	79293338	.	+	.	gene_id "lnc-bbug02794"	transcript_id "lnc-bbut00006454"	exon_number "1"
11	lncRNA	exon	79293556	79293891	.	+	.	gene_id "lnc-bbug02794"	transcript_id "lnc-bbut00006454"	exon_number "2"
11	lncRNA	exon	79343559	79344908	.	+	.	gene_id "lnc-bbug02796"	transcript_id "lnc-bbut00006457"	exon_number "1"
11	lncRNA	exon	79364397	79365060	.	+	.	gene_id "lnc-bbug02797"	transcript_id "lnc-bbut00006459"	exon_number "1"
11	lncRNA	exon	79380130	79380267	.	+	.	gene_id "lnc-bbug02798"	transcript_id "lnc-bbut00006461"	exon_number "1"
11	lncRNA	exon	79380711	79381123	.	+	.	gene_id "lnc-bbug02798"	transcript_id "lnc-bbut00006461"	exon_number "2"
11	lncRNA	exon	79397754	79398146	.	+	.	gene_id "lnc-bbug02799"	transcript_id "lnc-bbut00006462"	exon_number "1"
11	lncRNA	exon	79398289	79398582	.	+	.	gene_id "lnc-bbug02799"	transcript_id "lnc-bbut00006462"	exon_number "2"
11	lncRNA	exon	79403491	79404346	.	+	.	gene_id "lnc-bbug02800"	transcript_id "lnc-bbut00006463"	exon_number "1"
11	lncRNA	exon	79774605	79774764	.	+	.	gene_id "lnc-bbug02807"	transcript_id "lnc-bbut00006473"	exon_number "1"
11	lncRNA	exon	79774933	79775267	.	+	.	gene_id "lnc-bbug02807"	transcript_id "lnc-bbut00006473"	exon_number "2"

11	lncRNA	exon	79819897	79833605	.	+	.	gene_id "lnc-bbug02810"	transcript_id "lnc-bbut00006476"	exon_number "1"
11	lncRNA	exon	79852204	79852410	.	+	.	gene_id "lnc-bbug02812"	transcript_id "lnc-bbut00006480"	exon_number "1"
11	lncRNA	exon	79852551	79852834	.	+	.	gene_id "lnc-bbug02812"	transcript_id "lnc-bbut00006480"	exon_number "2"
11	lncRNA	exon	79857708	79864544	.	+	.	gene_id "lnc-bbug02813"	transcript_id "lnc-bbut00006481"	exon_number "1"
11	lncRNA	exon	79888748	79891034	.	+	.	gene_id "lnc-bbug02817"	transcript_id "lnc-bbut00006486"	exon_number "1"
11	lncRNA	exon	79933654	79935919	.	+	.	gene_id "lnc-bbug02822"	transcript_id "lnc-bbut00006492"	exon_number "1"
11	lncRNA	exon	79941336	79942487	.	+	.	gene_id "lnc-bbug02823"	transcript_id "lnc-bbut00006494"	exon_number "1"
11	lncRNA	exon	79948924	79950116	.	+	.	gene_id "lnc-bbug02824"	transcript_id "lnc-bbut00006496"	exon_number "1"
11	lncRNA	exon	80014550	80015670	.	+	.	gene_id "lnc-bbug02826"	transcript_id "lnc-bbut00006499"	exon_number "1"
11	lncRNA	exon	80024148	80024821	.	+	.	gene_id "lnc-bbug02827"	transcript_id "lnc-bbut00006502"	exon_number "1"
11	lncRNA	exon	80025049	80025735	.	+	.	gene_id "lnc-bbug02827"	transcript_id "lnc-bbut00006502"	exon_number "2"
11	lncRNA	exon	80024148	80025735	.	+	.	gene_id "lnc-bbug02827"	transcript_id "lnc-bbut00006501"	exon_number "1"
11	lncRNA	exon	80080995	80082035	.	+	.	gene_id "lnc-bbug02831"	transcript_id "lnc-bbut00006507"	exon_number "1"
11	lncRNA	exon	80103191	80106222	.	+	.	gene_id "lnc-bbug02832"	transcript_id "lnc-bbut00006509"	exon_number "1"
11	lncRNA	exon	80165657	80167060	.	+	.	gene_id "lnc-bbug02835"	transcript_id "lnc-bbut00006514"	exon_number "1"
11	lncRNA	exon	80172229	80177492	.	+	.	gene_id "lnc-bbug02836"	transcript_id "lnc-bbut00006516"	exon_number "1"
11	lncRNA	exon	80177908	80180252	.	+	.	gene_id "lnc-bbug02837"	transcript_id "lnc-bbut00006519"	exon_number "1"
11	lncRNA	exon	80457780	80466475	.	+	.	gene_id "lnc-bbug02842"	transcript_id "lnc-bbut00006526"	exon_number "1"
11	lncRNA	exon	80496064	80496181	.	+	.	gene_id "lnc-bbug02843"	transcript_id "lnc-bbut00006529"	exon_number "1"
11	lncRNA	exon	80549333	80549605	.	+	.	gene_id "lnc-bbug02843"	transcript_id "lnc-bbut00006529"	exon_number "2"
11	lncRNA	exon	80551623	80551667	.	+	.	gene_id "lnc-bbug02843"	transcript_id "lnc-bbut00006529"	exon_number "3"
11	lncRNA	exon	80553104	80553211	.	+	.	gene_id "lnc-bbug02843"	transcript_id "lnc-bbut00006529"	exon_number "4"
11	lncRNA	exon	80553867	80554433	.	+	.	gene_id "lnc-bbug02843"	transcript_id "lnc-bbut00006529"	exon_number "5"
11	lncRNA	exon	80496064	80554433	.	+	.	gene_id "lnc-bbug02843"	transcript_id "lnc-bbut00006528"	exon_number "1"
11	lncRNA	exon	80619665	80619694	.	+	.	gene_id "lnc-bbug02845"	transcript_id "lnc-bbut00006533"	exon_number "1"
11	lncRNA	exon	80622834	80623632	.	+	.	gene_id "lnc-bbug02845"	transcript_id "lnc-bbut00006533"	exon_number "2"
11	lncRNA	exon	80652668	80653222	.	+	.	gene_id "lnc-bbug02846"	transcript_id "lnc-bbut00006534"	exon_number "1"
11	lncRNA	exon	80754235	80755713	.	+	.	gene_id "lnc-bbug02852"	transcript_id "lnc-bbut00006540"	exon_number "1"
11	lncRNA	exon	80847588	80848245	.	+	.	gene_id "lnc-bbug02854"	transcript_id "lnc-bbut00006542"	exon_number "1"
11	lncRNA	exon	80848350	80848410	.	+	.	gene_id "lnc-bbug02854"	transcript_id "lnc-bbut00006542"	exon_number "2"
11	lncRNA	exon	80848487	80848554	.	+	.	gene_id "lnc-bbug02854"	transcript_id "lnc-bbut00006542"	exon_number "3"
11	lncRNA	exon	80852518	80852697	.	+	.	gene_id "lnc-bbug02854"	transcript_id "lnc-bbut00006542"	exon_number "4"
11	lncRNA	exon	80848157	80848245	.	+	.	gene_id "lnc-bbug02854"	transcript_id "lnc-bbut00006543"	exon_number "1"
11	lncRNA	exon	80848353	80848410	.	+	.	gene_id "lnc-bbug02854"	transcript_id "lnc-bbut00006543"	exon_number "2"
11	lncRNA	exon	80848487	80848554	.	+	.	gene_id "lnc-bbug02854"	transcript_id "lnc-bbut00006543"	exon_number "3"
11	lncRNA	exon	80852088	80852159	.	+	.	gene_id "lnc-bbug02854"	transcript_id "lnc-bbut00006543"	exon_number "4"
11	lncRNA	exon	80852518	80852697	.	+	.	gene_id "lnc-bbug02854"	transcript_id "lnc-bbut00006543"	exon_number "5"
11	lncRNA	exon	80848167	80848245	.	+	.	gene_id "lnc-bbug02854"	transcript_id "lnc-bbut00006544"	exon_number "1"
11	lncRNA	exon	80848350	80848410	.	+	.	gene_id "lnc-bbug02854"	transcript_id "lnc-bbut00006544"	exon_number "2"
11	lncRNA	exon	80848487	80848554	.	+	.	gene_id "lnc-bbug02854"	transcript_id "lnc-bbut00006544"	exon_number "3"
11	lncRNA	exon	80852088	80852152	.	+	.	gene_id "lnc-bbug02854"	transcript_id "lnc-bbut00006544"	exon_number "4"
11	lncRNA	exon	80852518	80852697	.	+	.	gene_id "lnc-bbug02854"	transcript_id "lnc-bbut00006544"	exon_number "5"
11	lncRNA	exon	80848168	80848230	.	+	.	gene_id "lnc-bbug02854"	transcript_id "lnc-bbut00006545"	exon_number "1"
11	lncRNA	exon	80848350	80848410	.	+	.	gene_id "lnc-bbug02854"	transcript_id "lnc-bbut00006545"	exon_number "2"
11	lncRNA	exon	80848487	80848554	.	+	.	gene_id "lnc-bbug02854"	transcript_id "lnc-bbut00006545"	exon_number "3"
11	lncRNA	exon	80852088	80852152	.	+	.	gene_id "lnc-bbug02854"	transcript_id "lnc-bbut00006545"	exon_number "4"
11	lncRNA	exon	80852518	80852697	.	+	.	gene_id "lnc-bbug02854"	transcript_id "lnc-bbut00006545"	exon_number "5"
11	lncRNA	exon	80848174	80848245	.	+	.	gene_id "lnc-bbug02854"	transcript_id "lnc-bbut00006546"	exon_number "1"
11	lncRNA	exon	80848353	80848410	.	+	.	gene_id "lnc-bbug02854"	transcript_id "lnc-bbut00006546"	exon_number "2"
11	lncRNA	exon	80848487	80848554	.	+	.	gene_id "lnc-bbug02854"	transcript_id "lnc-bbut00006546"	exon_number "3"
11	lncRNA	exon	80852088	80852152	.	+	.	gene_id "lnc-bbug02854"	transcript_id "lnc-bbut00006546"	exon_number "4"
11	lncRNA	exon	80852518	80852697	.	+	.	gene_id "lnc-bbug02854"	transcript_id "lnc-bbut00006546"	exon_number "5"

11	lncRNA	exon	80848203	80848230	.	+	.	gene_id "lnc-bbug02854"	transcript_id "lnc-bbut00006547"	exon_number "1"
11	lncRNA	exon	80848353	80848410	.	+	.	gene_id "lnc-bbug02854"	transcript_id "lnc-bbut00006547"	exon_number "2"
11	lncRNA	exon	80848487	80848554	.	+	.	gene_id "lnc-bbug02854"	transcript_id "lnc-bbut00006547"	exon_number "3"
11	lncRNA	exon	80852088	80852152	.	+	.	gene_id "lnc-bbug02854"	transcript_id "lnc-bbut00006547"	exon_number "4"
11	lncRNA	exon	80852518	80852697	.	+	.	gene_id "lnc-bbug02854"	transcript_id "lnc-bbut00006547"	exon_number "5"
11	lncRNA	exon	80848205	80848245	.	+	.	gene_id "lnc-bbug02854"	transcript_id "lnc-bbut00006548"	exon_number "1"
11	lncRNA	exon	80848350	80848410	.	+	.	gene_id "lnc-bbug02854"	transcript_id "lnc-bbut00006548"	exon_number "2"
11	lncRNA	exon	80852088	80852152	.	+	.	gene_id "lnc-bbug02854"	transcript_id "lnc-bbut00006548"	exon_number "3"
11	lncRNA	exon	80852518	80852697	.	+	.	gene_id "lnc-bbug02854"	transcript_id "lnc-bbut00006548"	exon_number "4"
11	lncRNA	exon	80848522	80848661	.	+	.	gene_id "lnc-bbug02854"	transcript_id "lnc-bbut00006549"	exon_number "1"
11	lncRNA	exon	80852088	80852152	.	+	.	gene_id "lnc-bbug02854"	transcript_id "lnc-bbut00006549"	exon_number "2"
11	lncRNA	exon	80852518	80852697	.	+	.	gene_id "lnc-bbug02854"	transcript_id "lnc-bbut00006549"	exon_number "3"
11	lncRNA	exon	80970465	80970596	.	+	.	gene_id "lnc-bbug02859"	transcript_id "lnc-bbut00006559"	exon_number "1"
11	lncRNA	exon	80980691	80980792	.	+	.	gene_id "lnc-bbug02859"	transcript_id "lnc-bbut00006559"	exon_number "2"
11	lncRNA	exon	80988987	80989504	.	+	.	gene_id "lnc-bbug02859"	transcript_id "lnc-bbut00006559"	exon_number "3"
11	lncRNA	exon	80979913	80980037	.	+	.	gene_id "lnc-bbug02859"	transcript_id "lnc-bbut00006560"	exon_number "1"
11	lncRNA	exon	80980691	80980792	.	+	.	gene_id "lnc-bbug02859"	transcript_id "lnc-bbut00006560"	exon_number "2"
11	lncRNA	exon	80988987	80989504	.	+	.	gene_id "lnc-bbug02859"	transcript_id "lnc-bbut00006560"	exon_number "3"
11	lncRNA	exon	81213533	81213601	.	+	.	gene_id "lnc-bbug02863"	transcript_id "lnc-bbut00006566"	exon_number "1"
11	lncRNA	exon	81214627	81214707	.	+	.	gene_id "lnc-bbug02863"	transcript_id "lnc-bbut00006566"	exon_number "2"
11	lncRNA	exon	81216997	81217107	.	+	.	gene_id "lnc-bbug02863"	transcript_id "lnc-bbut00006566"	exon_number "3"
11	lncRNA	exon	81496028	81496948	.	+	.	gene_id "lnc-bbug02870"	transcript_id "lnc-bbut00006578"	exon_number "1"
11	lncRNA	exon	81502024	81502618	.	+	.	gene_id "lnc-bbug02870"	transcript_id "lnc-bbut00006578"	exon_number "2"
11	lncRNA	exon	81672928	81674888	.	+	.	gene_id "lnc-bbug02872"	transcript_id "lnc-bbut00006580"	exon_number "1"
11	lncRNA	exon	81676129	81676889	.	+	.	gene_id "lnc-bbug02873"	transcript_id "lnc-bbut00006583"	exon_number "1"
11	lncRNA	exon	81685159	81685202	.	+	.	gene_id "lnc-bbug02873"	transcript_id "lnc-bbut00006583"	exon_number "2"
11	lncRNA	exon	81690967	81691042	.	+	.	gene_id "lnc-bbug02873"	transcript_id "lnc-bbut00006583"	exon_number "3"
11	lncRNA	exon	81692112	81693715	.	+	.	gene_id "lnc-bbug02873"	transcript_id "lnc-bbut00006583"	exon_number "4"
11	lncRNA	exon	81676129	81676889	.	+	.	gene_id "lnc-bbug02873"	transcript_id "lnc-bbut00006585"	exon_number "1"
11	lncRNA	exon	81683593	81683763	.	+	.	gene_id "lnc-bbug02873"	transcript_id "lnc-bbut00006585"	exon_number "2"
11	lncRNA	exon	81685159	81685202	.	+	.	gene_id "lnc-bbug02873"	transcript_id "lnc-bbut00006585"	exon_number "3"
11	lncRNA	exon	81688807	81688988	.	+	.	gene_id "lnc-bbug02873"	transcript_id "lnc-bbut00006585"	exon_number "4"
11	lncRNA	exon	81690967	81691042	.	+	.	gene_id "lnc-bbug02873"	transcript_id "lnc-bbut00006585"	exon_number "5"
11	lncRNA	exon	81692112	81693715	.	+	.	gene_id "lnc-bbug02873"	transcript_id "lnc-bbut00006585"	exon_number "6"
11	lncRNA	exon	81676129	81676889	.	+	.	gene_id "lnc-bbug02873"	transcript_id "lnc-bbut00006584"	exon_number "1"
11	lncRNA	exon	81683593	81683763	.	+	.	gene_id "lnc-bbug02873"	transcript_id "lnc-bbut00006584"	exon_number "2"
11	lncRNA	exon	81685159	81685202	.	+	.	gene_id "lnc-bbug02873"	transcript_id "lnc-bbut00006584"	exon_number "3"
11	lncRNA	exon	81690967	81691042	.	+	.	gene_id "lnc-bbug02873"	transcript_id "lnc-bbut00006584"	exon_number "4"
11	lncRNA	exon	81692112	81693715	.	+	.	gene_id "lnc-bbug02873"	transcript_id "lnc-bbut00006584"	exon_number "5"
11	lncRNA	exon	81676129	81676889	.	+	.	gene_id "lnc-bbug02873"	transcript_id "lnc-bbut00006582"	exon_number "1"
11	lncRNA	exon	81690967	81691042	.	+	.	gene_id "lnc-bbug02873"	transcript_id "lnc-bbut00006582"	exon_number "2"
11	lncRNA	exon	81692112	81693715	.	+	.	gene_id "lnc-bbug02873"	transcript_id "lnc-bbut00006582"	exon_number "3"
11	lncRNA	exon	81706320	81707408	.	+	.	gene_id "lnc-bbug02874"	transcript_id "lnc-bbut00006586"	exon_number "1"
11	lncRNA	exon	83504839	83504848	.	+	.	gene_id "lnc-bbug02897"	transcript_id "lnc-bbut00006646"	exon_number "1"
11	lncRNA	exon	83504986	83505248	.	+	.	gene_id "lnc-bbug02897"	transcript_id "lnc-bbut00006646"	exon_number "2"
11	lncRNA	exon	83505365	83505610	.	+	.	gene_id "lnc-bbug02897"	transcript_id "lnc-bbut00006646"	exon_number "3"
11	lncRNA	exon	83505872	83506182	.	+	.	gene_id "lnc-bbug02897"	transcript_id "lnc-bbut00006646"	exon_number "4"
11	lncRNA	exon	85332667	85332760	.	+	.	gene_id "lnc-bbug02907"	transcript_id "lnc-bbut00006676"	exon_number "1"
11	lncRNA	exon	85333029	85333744	.	+	.	gene_id "lnc-bbug02907"	transcript_id "lnc-bbut00006676"	exon_number "2"
11	lncRNA	exon	85347791	85347861	.	+	.	gene_id "lnc-bbug02908"	transcript_id "lnc-bbut00006679"	exon_number "1"
11	lncRNA	exon	85372016	85372600	.	+	.	gene_id "lnc-bbug02908"	transcript_id "lnc-bbut00006679"	exon_number "2"
11	lncRNA	exon	85372660	85375748	.	+	.	gene_id "lnc-bbug02909"	transcript_id "lnc-bbut00006680"	exon_number "1"

11	lncRNA	exon	85377207	85377537	.	+	.	gene_id "lnc-bbug02909"	transcript_id "lnc-bbut00006680"	exon_number "2"
11	lncRNA	exon	86038830	86041988	.	+	.	gene_id "lnc-bbug02911"	transcript_id "lnc-bbut00006695"	exon_number "1"
11	lncRNA	exon	86042837	86043136	.	+	.	gene_id "lnc-bbug02911"	transcript_id "lnc-bbut00006695"	exon_number "2"
11	lncRNA	exon	86329931	86330346	.	+	.	gene_id "lnc-bbug02912"	transcript_id "lnc-bbut00006696"	exon_number "1"
11	lncRNA	exon	86351186	86351508	.	+	.	gene_id "lnc-bbug02912"	transcript_id "lnc-bbut00006696"	exon_number "2"
11	lncRNA	exon	86769623	86770078	.	+	.	gene_id "lnc-bbug02913"	transcript_id "lnc-bbut00006697"	exon_number "1"
11	lncRNA	exon	86770119	86770668	.	+	.	gene_id "lnc-bbug02913"	transcript_id "lnc-bbut00006697"	exon_number "2"
11	lncRNA	exon	87040966	87043221	.	+	.	gene_id "lnc-bbug02915"	transcript_id "lnc-bbut00006703"	exon_number "1"
11	lncRNA	exon	87043533	87046676	.	+	.	gene_id "lnc-bbug02915"	transcript_id "lnc-bbut00006703"	exon_number "2"
11	lncRNA	exon	88042428	88042975	.	+	.	gene_id "lnc-bbug02921"	transcript_id "lnc-bbut00006711"	exon_number "1"
11	lncRNA	exon	88043377	88043462	.	+	.	gene_id "lnc-bbug02921"	transcript_id "lnc-bbut00006711"	exon_number "2"
11	lncRNA	exon	88043768	88044209	.	+	.	gene_id "lnc-bbug02921"	transcript_id "lnc-bbut00006711"	exon_number "3"
11	lncRNA	exon	88042428	88042975	.	+	.	gene_id "lnc-bbug02921"	transcript_id "lnc-bbut00006712"	exon_number "1"
11	lncRNA	exon	88043377	88043462	.	+	.	gene_id "lnc-bbug02921"	transcript_id "lnc-bbut00006712"	exon_number "2"
11	lncRNA	exon	88050572	88050787	.	+	.	gene_id "lnc-bbug02921"	transcript_id "lnc-bbut00006712"	exon_number "3"
11	lncRNA	exon	88630288	88630448	.	+	.	gene_id "lnc-bbug02924"	transcript_id "lnc-bbut00006719"	exon_number "1"
11	lncRNA	exon	88631313	88633063	.	+	.	gene_id "lnc-bbug02924"	transcript_id "lnc-bbut00006719"	exon_number "2"
11	lncRNA	exon	88927470	88928637	.	+	.	gene_id "lnc-bbug02925"	transcript_id "lnc-bbut00006720"	exon_number "1"
11	lncRNA	exon	88940076	88940274	.	+	.	gene_id "lnc-bbug02925"	transcript_id "lnc-bbut00006720"	exon_number "2"
11	lncRNA	exon	88941248	88941336	.	+	.	gene_id "lnc-bbug02925"	transcript_id "lnc-bbut00006720"	exon_number "3"
11	lncRNA	exon	88941629	88942509	.	+	.	gene_id "lnc-bbug02925"	transcript_id "lnc-bbut00006720"	exon_number "4"
11	lncRNA	exon	89294427	89295325	.	+	.	gene_id "lnc-bbug02928"	transcript_id "lnc-bbut00006723"	exon_number "1"
11	lncRNA	exon	89297615	89297748	.	+	.	gene_id "lnc-bbug02928"	transcript_id "lnc-bbut00006723"	exon_number "2"
11	lncRNA	exon	89299977	89300849	.	+	.	gene_id "lnc-bbug02928"	transcript_id "lnc-bbut00006723"	exon_number "3"
11	lncRNA	exon	89294444	89295325	.	+	.	gene_id "lnc-bbug02928"	transcript_id "lnc-bbut00006724"	exon_number "1"
11	lncRNA	exon	89296752	89296841	.	+	.	gene_id "lnc-bbug02928"	transcript_id "lnc-bbut00006724"	exon_number "2"
11	lncRNA	exon	89297612	89297748	.	+	.	gene_id "lnc-bbug02928"	transcript_id "lnc-bbut00006724"	exon_number "3"
11	lncRNA	exon	89313248	89313864	.	+	.	gene_id "lnc-bbug02928"	transcript_id "lnc-bbut00006724"	exon_number "4"
11	lncRNA	exon	89974810	89975161	.	+	.	gene_id "lnc-bbug02932"	transcript_id "lnc-bbut00006743"	exon_number "1"
11	lncRNA	exon	89978043	89978140	.	+	.	gene_id "lnc-bbug02932"	transcript_id "lnc-bbut00006743"	exon_number "2"
11	lncRNA	exon	89982801	89982959	.	+	.	gene_id "lnc-bbug02932"	transcript_id "lnc-bbut00006743"	exon_number "3"
11	lncRNA	exon	89974812	89975161	.	+	.	gene_id "lnc-bbug02932"	transcript_id "lnc-bbut00006744"	exon_number "1"
11	lncRNA	exon	89977135	89977423	.	+	.	gene_id "lnc-bbug02932"	transcript_id "lnc-bbut00006744"	exon_number "2"
11	lncRNA	exon	90604749	90605455	.	+	.	gene_id "lnc-bbug02943"	transcript_id "lnc-bbut00006778"	exon_number "1"
11	lncRNA	exon	90611457	90612124	.	+	.	gene_id "lnc-bbug02943"	transcript_id "lnc-bbut00006778"	exon_number "2"
11	lncRNA	exon	90614910	90615116	.	+	.	gene_id "lnc-bbug02943"	transcript_id "lnc-bbut00006778"	exon_number "3"
11	lncRNA	exon	90622036	90622137	.	+	.	gene_id "lnc-bbug02943"	transcript_id "lnc-bbut00006778"	exon_number "4"
11	lncRNA	exon	90623284	90624252	.	+	.	gene_id "lnc-bbug02943"	transcript_id "lnc-bbut00006778"	exon_number "5"
11	lncRNA	exon	91607047	91607175	.	+	.	gene_id "lnc-bbug02952"	transcript_id "lnc-bbut00006796"	exon_number "1"
11	lncRNA	exon	91607442	91608351	.	+	.	gene_id "lnc-bbug02952"	transcript_id "lnc-bbut00006796"	exon_number "2"
11	lncRNA	exon	91713275	91713397	.	+	.	gene_id "lnc-bbug02954"	transcript_id "lnc-bbut00006802"	exon_number "1"
11	lncRNA	exon	91713490	91718351	.	+	.	gene_id "lnc-bbug02954"	transcript_id "lnc-bbut00006802"	exon_number "2"
11	lncRNA	exon	92471771	92472048	.	+	.	gene_id "lnc-bbug02956"	transcript_id "lnc-bbut00006805"	exon_number "1"
11	lncRNA	exon	92472322	92473295	.	+	.	gene_id "lnc-bbug02956"	transcript_id "lnc-bbut00006805"	exon_number "2"
11	lncRNA	exon	94407424	94407541	.	+	.	gene_id "lnc-bbug02966"	transcript_id "lnc-bbut00006829"	exon_number "1"
11	lncRNA	exon	94408200	94408869	.	+	.	gene_id "lnc-bbug02966"	transcript_id "lnc-bbut00006829"	exon_number "2"
11	lncRNA	exon	94534865	94534955	.	+	.	gene_id "lnc-bbug02967"	transcript_id "lnc-bbut00006830"	exon_number "1"
11	lncRNA	exon	94557993	94558013	.	+	.	gene_id "lnc-bbug02967"	transcript_id "lnc-bbut00006830"	exon_number "2"
11	lncRNA	exon	94558691	94559638	.	+	.	gene_id "lnc-bbug02967"	transcript_id "lnc-bbut00006830"	exon_number "3"
11	lncRNA	exon	94559854	94560184	.	+	.	gene_id "lnc-bbug02967"	transcript_id "lnc-bbut00006830"	exon_number "4"
11	lncRNA	exon	94626109	94626277	.	+	.	gene_id "lnc-bbug02968"	transcript_id "lnc-bbut00006831"	exon_number "1"
11	lncRNA	exon	94628245	94628340	.	+	.	gene_id "lnc-bbug02968"	transcript_id "lnc-bbut00006831"	exon_number "2"

11	lncRNA	exon	94628477	94628525	.	+	.	gene_id "lnc-bbug02968"	transcript_id "lnc-bbut00006831"	exon_number "3"
11	lncRNA	exon	94640518	94640784	.	+	.	gene_id "lnc-bbug02969"	transcript_id "lnc-bbut00006832"	exon_number "1"
11	lncRNA	exon	94641991	94642078	.	+	.	gene_id "lnc-bbug02969"	transcript_id "lnc-bbut00006832"	exon_number "2"
11	lncRNA	exon	94643044	94643091	.	+	.	gene_id "lnc-bbug02969"	transcript_id "lnc-bbut00006832"	exon_number "3"
11	lncRNA	exon	95050875	95052606	.	+	.	gene_id "lnc-bbug02971"	transcript_id "lnc-bbut00006834"	exon_number "1"
11	lncRNA	exon	95053306	95053495	.	+	.	gene_id "lnc-bbug02971"	transcript_id "lnc-bbut00006834"	exon_number "2"
11	lncRNA	exon	95058015	95058236	.	+	.	gene_id "lnc-bbug02971"	transcript_id "lnc-bbut00006834"	exon_number "3"
11	lncRNA	exon	95059273	95061269	.	+	.	gene_id "lnc-bbug02971"	transcript_id "lnc-bbut00006834"	exon_number "4"
11	lncRNA	exon	95072570	95074073	.	+	.	gene_id "lnc-bbug02972"	transcript_id "lnc-bbut00006835"	exon_number "1"
11	lncRNA	exon	95076875	95077182	.	+	.	gene_id "lnc-bbug02972"	transcript_id "lnc-bbut00006835"	exon_number "2"
11	lncRNA	exon	97072580	97072710	.	+	.	gene_id "lnc-bbug02979"	transcript_id "lnc-bbut00006854"	exon_number "1"
11	lncRNA	exon	97077541	97078213	.	+	.	gene_id "lnc-bbug02979"	transcript_id "lnc-bbut00006854"	exon_number "2"
11	lncRNA	exon	97223110	97223360	.	+	.	gene_id "lnc-bbug02981"	transcript_id "lnc-bbut00006866"	exon_number "1"
11	lncRNA	exon	97308794	97308913	.	+	.	gene_id "lnc-bbug02981"	transcript_id "lnc-bbut00006866"	exon_number "2"
11	lncRNA	exon	97309059	97309234	.	+	.	gene_id "lnc-bbug02981"	transcript_id "lnc-bbut00006866"	exon_number "3"
11	lncRNA	exon	97309987	97314115	.	+	.	gene_id "lnc-bbug02981"	transcript_id "lnc-bbut00006866"	exon_number "4"
11	lncRNA	exon	97299328	97299456	.	+	.	gene_id "lnc-bbug02981"	transcript_id "lnc-bbut00006867"	exon_number "1"
11	lncRNA	exon	97308794	97308913	.	+	.	gene_id "lnc-bbug02981"	transcript_id "lnc-bbut00006867"	exon_number "2"
11	lncRNA	exon	97309059	97309234	.	+	.	gene_id "lnc-bbug02981"	transcript_id "lnc-bbut00006867"	exon_number "3"
11	lncRNA	exon	97309987	97314115	.	+	.	gene_id "lnc-bbug02981"	transcript_id "lnc-bbut00006867"	exon_number "4"
11	lncRNA	exon	97301126	97301362	.	+	.	gene_id "lnc-bbug02981"	transcript_id "lnc-bbut00006868"	exon_number "1"
11	lncRNA	exon	97308794	97308913	.	+	.	gene_id "lnc-bbug02981"	transcript_id "lnc-bbut00006868"	exon_number "2"
11	lncRNA	exon	97309059	97309234	.	+	.	gene_id "lnc-bbug02981"	transcript_id "lnc-bbut00006868"	exon_number "3"
11	lncRNA	exon	97309987	97314115	.	+	.	gene_id "lnc-bbug02981"	transcript_id "lnc-bbut00006868"	exon_number "4"
11	lncRNA	exon	97907728	97907749	.	+	.	gene_id "lnc-bbug02984"	transcript_id "lnc-bbut00006874"	exon_number "1"
11	lncRNA	exon	97908352	97908429	.	+	.	gene_id "lnc-bbug02984"	transcript_id "lnc-bbut00006874"	exon_number "2"
11	lncRNA	exon	97909968	97910122	.	+	.	gene_id "lnc-bbug02984"	transcript_id "lnc-bbut00006874"	exon_number "3"
11	lncRNA	exon	97915110	97915279	.	+	.	gene_id "lnc-bbug02984"	transcript_id "lnc-bbut00006874"	exon_number "4"
11	lncRNA	exon	97916121	97916851	.	+	.	gene_id "lnc-bbug02984"	transcript_id "lnc-bbut00006874"	exon_number "5"
11	lncRNA	exon	98397064	98397107	.	+	.	gene_id "lnc-bbug02988"	transcript_id "lnc-bbut00006882"	exon_number "1"
11	lncRNA	exon	98405127	98409441	.	+	.	gene_id "lnc-bbug02988"	transcript_id "lnc-bbut00006882"	exon_number "2"
11	lncRNA	exon	98450345	98451715	.	+	.	gene_id "lnc-bbug02990"	transcript_id "lnc-bbut00006885"	exon_number "1"
11	lncRNA	exon	99309205	99309725	.	+	.	gene_id "lnc-bbug02993"	transcript_id "lnc-bbut00006891"	exon_number "1"
11	lncRNA	exon	99310267	99310522	.	+	.	gene_id "lnc-bbug02993"	transcript_id "lnc-bbut00006891"	exon_number "2"
11	lncRNA	exon	99458064	99458147	.	+	.	gene_id "lnc-bbug02994"	transcript_id "lnc-bbut00006892"	exon_number "1"
11	lncRNA	exon	99459278	99459347	.	+	.	gene_id "lnc-bbug02994"	transcript_id "lnc-bbut00006892"	exon_number "2"
11	lncRNA	exon	99522829	99525703	.	+	.	gene_id "lnc-bbug02994"	transcript_id "lnc-bbut00006892"	exon_number "3"
11	lncRNA	exon	100754664	100755169	.	+	.	gene_id "lnc-bbug02995"	transcript_id "lnc-bbut00006893"	exon_number "1"
11	lncRNA	exon	100755600	100755676	.	+	.	gene_id "lnc-bbug02995"	transcript_id "lnc-bbut00006893"	exon_number "2"
11	lncRNA	exon	100756060	100756215	.	+	.	gene_id "lnc-bbug02995"	transcript_id "lnc-bbut00006893"	exon_number "3"
11	lncRNA	exon	101012765	101012874	.	+	.	gene_id "lnc-bbug02996"	transcript_id "lnc-bbut00006894"	exon_number "1"
11	lncRNA	exon	101027337	101027678	.	+	.	gene_id "lnc-bbug02996"	transcript_id "lnc-bbut00006894"	exon_number "2"
11	lncRNA	exon	101053827	101055664	.	+	.	gene_id "lnc-bbug02997"	transcript_id "lnc-bbut00006895"	exon_number "1"
11	lncRNA	exon	101065199	101065311	.	+	.	gene_id "lnc-bbug02997"	transcript_id "lnc-bbut00006895"	exon_number "2"
11	lncRNA	exon	101162951	101164166	.	+	.	gene_id "lnc-bbug02997"	transcript_id "lnc-bbut00006895"	exon_number "3"
11	lncRNA	exon	102096375	102096514	.	+	.	gene_id "lnc-bbug03001"	transcript_id "lnc-bbut00006910"	exon_number "1"
11	lncRNA	exon	102096689	102096778	.	+	.	gene_id "lnc-bbug03001"	transcript_id "lnc-bbut00006910"	exon_number "2"
11	lncRNA	exon	102097223	102097570	.	+	.	gene_id "lnc-bbug03001"	transcript_id "lnc-bbut00006910"	exon_number "3"
11	lncRNA	exon	102135499	102135641	.	+	.	gene_id "lnc-bbug03003"	transcript_id "lnc-bbut00006912"	exon_number "1"
11	lncRNA	exon	102136166	102136290	.	+	.	gene_id "lnc-bbug03003"	transcript_id "lnc-bbut00006912"	exon_number "2"
11	lncRNA	exon	102149729	102150069	.	+	.	gene_id "lnc-bbug03003"	transcript_id "lnc-bbut00006912"	exon_number "3"
11	lncRNA	exon	102198025	102198344	.	+	.	gene_id "lnc-bbug03004"	transcript_id "lnc-bbut00006914"	exon_number "1"

11	lncRNA	exon	102206480	102206611	.	+	.	gene_id "lnc-bbug03004"	transcript_id "lnc-bbut00006914"	exon_number "2"
11	lncRNA	exon	102210631	102210762	.	+	.	gene_id "lnc-bbug03004"	transcript_id "lnc-bbut00006914"	exon_number "3"
11	lncRNA	exon	102212035	102214205	.	+	.	gene_id "lnc-bbug03004"	transcript_id "lnc-bbut00006914"	exon_number "4"
11	lncRNA	exon	102198025	102198344	.	+	.	gene_id "lnc-bbug03004"	transcript_id "lnc-bbut00006915"	exon_number "1"
11	lncRNA	exon	102206480	102206611	.	+	.	gene_id "lnc-bbug03004"	transcript_id "lnc-bbut00006915"	exon_number "2"
11	lncRNA	exon	102210631	102210762	.	+	.	gene_id "lnc-bbug03004"	transcript_id "lnc-bbut00006915"	exon_number "3"
11	lncRNA	exon	102230800	102231309	.	+	.	gene_id "lnc-bbug03004"	transcript_id "lnc-bbut00006915"	exon_number "4"
11	lncRNA	exon	524604	526102	.	-	.	gene_id "lnc-bbug03013"	transcript_id "lnc-bbut00006933"	exon_number "1"
11	lncRNA	exon	532165	532246	.	-	.	gene_id "lnc-bbug03013"	transcript_id "lnc-bbut00006933"	exon_number "2"
11	lncRNA	exon	558857	559066	.	-	.	gene_id "lnc-bbug03013"	transcript_id "lnc-bbut00006933"	exon_number "3"
11	lncRNA	exon	557293	558318	.	-	.	gene_id "lnc-bbug03014"	transcript_id "lnc-bbut00006934"	exon_number "1"
11	lncRNA	exon	558578	558824	.	-	.	gene_id "lnc-bbug03014"	transcript_id "lnc-bbut00006934"	exon_number "2"
11	lncRNA	exon	655701	657188	.	-	.	gene_id "lnc-bbug03015"	transcript_id "lnc-bbut00006935"	exon_number "1"
11	lncRNA	exon	659735	659868	.	-	.	gene_id "lnc-bbug03015"	transcript_id "lnc-bbut00006935"	exon_number "2"
11	lncRNA	exon	660791	660938	.	-	.	gene_id "lnc-bbug03015"	transcript_id "lnc-bbut00006935"	exon_number "3"
11	lncRNA	exon	661957	662755	.	-	.	gene_id "lnc-bbug03015"	transcript_id "lnc-bbut00006935"	exon_number "4"
11	lncRNA	exon	1200482	1200883	.	-	.	gene_id "lnc-bbug03017"	transcript_id "lnc-bbut00006938"	exon_number "1"
11	lncRNA	exon	1201790	1201933	.	-	.	gene_id "lnc-bbug03017"	transcript_id "lnc-bbut00006938"	exon_number "2"
11	lncRNA	exon	1248856	1249114	.	-	.	gene_id "lnc-bbug03019"	transcript_id "lnc-bbut00006941"	exon_number "1"
11	lncRNA	exon	1250622	1250945	.	-	.	gene_id "lnc-bbug03019"	transcript_id "lnc-bbut00006941"	exon_number "2"
11	lncRNA	exon	1254125	1255574	.	-	.	gene_id "lnc-bbug03019"	transcript_id "lnc-bbut00006941"	exon_number "3"
11	lncRNA	exon	2343556	2344139	.	-	.	gene_id "lnc-bbug03024"	transcript_id "lnc-bbut00006947"	exon_number "1"
11	lncRNA	exon	2354812	2354996	.	-	.	gene_id "lnc-bbug03024"	transcript_id "lnc-bbut00006947"	exon_number "2"
11	lncRNA	exon	4246296	4246585	.	-	.	gene_id "lnc-bbug03030"	transcript_id "lnc-bbut00006972"	exon_number "1"
11	lncRNA	exon	5176851	5177705	.	-	.	gene_id "lnc-bbug03031"	transcript_id "lnc-bbut00006973"	exon_number "1"
11	lncRNA	exon	8871503	8871712	.	-	.	gene_id "lnc-bbug03033"	transcript_id "lnc-bbut00006978"	exon_number "1"
11	lncRNA	exon	8908132	8908242	.	-	.	gene_id "lnc-bbug03033"	transcript_id "lnc-bbut00006978"	exon_number "2"
11	lncRNA	exon	9767143	9767808	.	-	.	gene_id "lnc-bbug03034"	transcript_id "lnc-bbut00006979"	exon_number "1"
11	lncRNA	exon	9768319	9769511	.	-	.	gene_id "lnc-bbug03034"	transcript_id "lnc-bbut00006979"	exon_number "2"
11	lncRNA	exon	9869428	9875361	.	-	.	gene_id "lnc-bbug03035"	transcript_id "lnc-bbut00006980"	exon_number "1"
11	lncRNA	exon	9876620	9876743	.	-	.	gene_id "lnc-bbug03035"	transcript_id "lnc-bbut00006980"	exon_number "2"
11	lncRNA	exon	9890037	9890159	.	-	.	gene_id "lnc-bbug03035"	transcript_id "lnc-bbut00006980"	exon_number "3"
11	lncRNA	exon	9890365	9891587	.	-	.	gene_id "lnc-bbug03035"	transcript_id "lnc-bbut00006980"	exon_number "4"
11	lncRNA	exon	9869428	9875361	.	-	.	gene_id "lnc-bbug03035"	transcript_id "lnc-bbut00006981"	exon_number "1"
11	lncRNA	exon	9876620	9876743	.	-	.	gene_id "lnc-bbug03035"	transcript_id "lnc-bbut00006981"	exon_number "2"
11	lncRNA	exon	9890037	9890159	.	-	.	gene_id "lnc-bbug03035"	transcript_id "lnc-bbut00006981"	exon_number "3"
11	lncRNA	exon	9996835	9996989	.	-	.	gene_id "lnc-bbug03035"	transcript_id "lnc-bbut00006981"	exon_number "4"
11	lncRNA	exon	9997193	9997317	.	-	.	gene_id "lnc-bbug03036"	transcript_id "lnc-bbut00006982"	exon_number "1"
11	lncRNA	exon	9999343	9999413	.	-	.	gene_id "lnc-bbug03036"	transcript_id "lnc-bbut00006982"	exon_number "2"
11	lncRNA	exon	10010520	10010584	.	-	.	gene_id "lnc-bbug03036"	transcript_id "lnc-bbut00006982"	exon_number "3"
11	lncRNA	exon	10016829	10017199	.	-	.	gene_id "lnc-bbug03036"	transcript_id "lnc-bbut00006982"	exon_number "4"
11	lncRNA	exon	10622778	10623287	.	-	.	gene_id "lnc-bbug03037"	transcript_id "lnc-bbut00006983"	exon_number "1"
11	lncRNA	exon	10623832	10624292	.	-	.	gene_id "lnc-bbug03037"	transcript_id "lnc-bbut00006983"	exon_number "2"
11	lncRNA	exon	10663969	10664142	.	-	.	gene_id "lnc-bbug03038"	transcript_id "lnc-bbut00006984"	exon_number "1"
11	lncRNA	exon	10664371	10665274	.	-	.	gene_id "lnc-bbug03038"	transcript_id "lnc-bbut00006984"	exon_number "2"
11	lncRNA	exon	11415094	11417229	.	-	.	gene_id "lnc-bbug03040"	transcript_id "lnc-bbut00006987"	exon_number "1"
11	lncRNA	exon	11417347	11417512	.	-	.	gene_id "lnc-bbug03040"	transcript_id "lnc-bbut00006987"	exon_number "2"
11	lncRNA	exon	11437882	11438085	.	-	.	gene_id "lnc-bbug03040"	transcript_id "lnc-bbut00006987"	exon_number "3"
11	lncRNA	exon	11415094	11417229	.	-	.	gene_id "lnc-bbug03040"	transcript_id "lnc-bbut00006986"	exon_number "1"
11	lncRNA	exon	11417347	11417512	.	-	.	gene_id "lnc-bbug03040"	transcript_id "lnc-bbut00006986"	exon_number "2"
11	lncRNA	exon	11437859	11438085	.	-	.	gene_id "lnc-bbug03040"	transcript_id "lnc-bbut00006986"	exon_number "3"
11	lncRNA	exon	11683561	11683895	.	-	.	gene_id "lnc-bbug03041"	transcript_id "lnc-bbut00006988"	exon_number "1"

11	lncRNA	exon	11687929	11688042	.	-	.	gene_id "lnc-bbug03041"	transcript_id "lnc-bbut00006988"	exon_number "2"
11	lncRNA	exon	11763649	11763794	.	-	.	gene_id "lnc-bbug03042"	transcript_id "lnc-bbut00006989"	exon_number "1"
11	lncRNA	exon	11764294	11764395	.	-	.	gene_id "lnc-bbug03042"	transcript_id "lnc-bbut00006989"	exon_number "2"
11	lncRNA	exon	11766210	11766979	.	-	.	gene_id "lnc-bbug03042"	transcript_id "lnc-bbut00006989"	exon_number "3"
11	lncRNA	exon	14476810	14476850	.	-	.	gene_id "lnc-bbug03045"	transcript_id "lnc-bbut00006994"	exon_number "1"
11	lncRNA	exon	14479164	14479490	.	-	.	gene_id "lnc-bbug03045"	transcript_id "lnc-bbut00006994"	exon_number "2"
11	lncRNA	exon	14946625	14948256	.	-	.	gene_id "lnc-bbug03052"	transcript_id "lnc-bbut00007009"	exon_number "1"
11	lncRNA	exon	14948385	14949193	.	-	.	gene_id "lnc-bbug03052"	transcript_id "lnc-bbut00007009"	exon_number "2"
11	lncRNA	exon	15668407	15669173	.	-	.	gene_id "lnc-bbug03056"	transcript_id "lnc-bbut00007015"	exon_number "1"
11	lncRNA	exon	15670486	15671380	.	-	.	gene_id "lnc-bbug03056"	transcript_id "lnc-bbut00007015"	exon_number "2"
11	lncRNA	exon	15858848	15859245	.	-	.	gene_id "lnc-bbug03059"	transcript_id "lnc-bbut00007029"	exon_number "1"
11	lncRNA	exon	15861772	15865154	.	-	.	gene_id "lnc-bbug03059"	transcript_id "lnc-bbut00007029"	exon_number "2"
11	lncRNA	exon	16332960	16333194	.	-	.	gene_id "lnc-bbug03062"	transcript_id "lnc-bbut00007045"	exon_number "1"
11	lncRNA	exon	16339430	16339593	.	-	.	gene_id "lnc-bbug03062"	transcript_id "lnc-bbut00007045"	exon_number "2"
11	lncRNA	exon	16348463	16348631	.	-	.	gene_id "lnc-bbug03062"	transcript_id "lnc-bbut00007045"	exon_number "3"
11	lncRNA	exon	16357614	16357963	.	-	.	gene_id "lnc-bbug03062"	transcript_id "lnc-bbut00007045"	exon_number "4"
11	lncRNA	exon	16358180	16358234	.	-	.	gene_id "lnc-bbug03062"	transcript_id "lnc-bbut00007045"	exon_number "5"
11	lncRNA	exon	16336954	16337486	.	-	.	gene_id "lnc-bbug03062"	transcript_id "lnc-bbut00007047"	exon_number "1"
11	lncRNA	exon	16339430	16339593	.	-	.	gene_id "lnc-bbug03062"	transcript_id "lnc-bbut00007047"	exon_number "2"
11	lncRNA	exon	16348463	16348631	.	-	.	gene_id "lnc-bbug03062"	transcript_id "lnc-bbut00007047"	exon_number "3"
11	lncRNA	exon	16357614	16357963	.	-	.	gene_id "lnc-bbug03062"	transcript_id "lnc-bbut00007047"	exon_number "4"
11	lncRNA	exon	16358180	16358234	.	-	.	gene_id "lnc-bbug03062"	transcript_id "lnc-bbut00007047"	exon_number "5"
11	lncRNA	exon	16336954	16337458	.	-	.	gene_id "lnc-bbug03062"	transcript_id "lnc-bbut00007046"	exon_number "1"
11	lncRNA	exon	16339430	16339593	.	-	.	gene_id "lnc-bbug03062"	transcript_id "lnc-bbut00007046"	exon_number "2"
11	lncRNA	exon	16348463	16348631	.	-	.	gene_id "lnc-bbug03062"	transcript_id "lnc-bbut00007046"	exon_number "3"
11	lncRNA	exon	16357614	16357963	.	-	.	gene_id "lnc-bbug03062"	transcript_id "lnc-bbut00007046"	exon_number "4"
11	lncRNA	exon	16358180	16358234	.	-	.	gene_id "lnc-bbug03062"	transcript_id "lnc-bbut00007046"	exon_number "5"
11	lncRNA	exon	16455061	16457170	.	-	.	gene_id "lnc-bbug03063"	transcript_id "lnc-bbut00007049"	exon_number "1"
11	lncRNA	exon	16457305	16457445	.	-	.	gene_id "lnc-bbug03063"	transcript_id "lnc-bbut00007049"	exon_number "2"
11	lncRNA	exon	16465429	16465688	.	-	.	gene_id "lnc-bbug03063"	transcript_id "lnc-bbut00007049"	exon_number "3"
11	lncRNA	exon	16711201	16711485	.	-	.	gene_id "lnc-bbug03067"	transcript_id "lnc-bbut00007056"	exon_number "1"
11	lncRNA	exon	16712403	16712450	.	-	.	gene_id "lnc-bbug03067"	transcript_id "lnc-bbut00007056"	exon_number "2"
11	lncRNA	exon	16772987	16774417	.	-	.	gene_id "lnc-bbug03068"	transcript_id "lnc-bbut00007057"	exon_number "1"
11	lncRNA	exon	16775330	16776143	.	-	.	gene_id "lnc-bbug03068"	transcript_id "lnc-bbut00007057"	exon_number "2"
11	lncRNA	exon	16772987	16774417	.	-	.	gene_id "lnc-bbug03068"	transcript_id "lnc-bbut00007058"	exon_number "1"
11	lncRNA	exon	16775330	16775654	.	-	.	gene_id "lnc-bbug03068"	transcript_id "lnc-bbut00007058"	exon_number "2"
11	lncRNA	exon	16781645	16787005	.	-	.	gene_id "lnc-bbug03068"	transcript_id "lnc-bbut00007058"	exon_number "3"
11	lncRNA	exon	16798437	16803969	.	-	.	gene_id "lnc-bbug03068"	transcript_id "lnc-bbut00007058"	exon_number "4"
11	lncRNA	exon	16781089	16787005	.	-	.	gene_id "lnc-bbug03068"	transcript_id "lnc-bbut00007059"	exon_number "1"
11	lncRNA	exon	16798437	16798471	.	-	.	gene_id "lnc-bbug03068"	transcript_id "lnc-bbut00007059"	exon_number "2"
11	lncRNA	exon	16796133	16801335	.	-	.	gene_id "lnc-bbug03068"	transcript_id "lnc-bbut00007061"	exon_number "1"
11	lncRNA	exon	16801419	16803969	.	-	.	gene_id "lnc-bbug03068"	transcript_id "lnc-bbut00007061"	exon_number "2"
11	lncRNA	exon	16796133	16800337	.	-	.	gene_id "lnc-bbug03068"	transcript_id "lnc-bbut00007060"	exon_number "1"
11	lncRNA	exon	16801212	16803969	.	-	.	gene_id "lnc-bbug03068"	transcript_id "lnc-bbut00007060"	exon_number "2"
11	lncRNA	exon	17190504	17194289	.	-	.	gene_id "lnc-bbug03073"	transcript_id "lnc-bbut00007067"	exon_number "1"
11	lncRNA	exon	17197827	17197947	.	-	.	gene_id "lnc-bbug03073"	transcript_id "lnc-bbut00007067"	exon_number "2"
11	lncRNA	exon	17422766	17423072	.	-	.	gene_id "lnc-bbug03077"	transcript_id "lnc-bbut00007073"	exon_number "1"
11	lncRNA	exon	17424475	17424663	.	-	.	gene_id "lnc-bbug03077"	transcript_id "lnc-bbut00007073"	exon_number "2"
11	lncRNA	exon	17748753	17750805	.	-	.	gene_id "lnc-bbug03081"	transcript_id "lnc-bbut00007080"	exon_number "1"
11	lncRNA	exon	17851603	17851640	.	-	.	gene_id "lnc-bbug03081"	transcript_id "lnc-bbut00007080"	exon_number "2"
11	lncRNA	exon	17854074	17854140	.	-	.	gene_id "lnc-bbug03081"	transcript_id "lnc-bbut00007080"	exon_number "3"
11	lncRNA	exon	17857473	17857510	.	-	.	gene_id "lnc-bbug03081"	transcript_id "lnc-bbut00007080"	exon_number "4"

11	lncRNA	exon	17858223	17858297	.	-	.	gene_id "lnc-bbug03081"	transcript_id "lnc-bbut00007080"	exon_number "5"
11	lncRNA	exon	17858400	17858487	.	-	.	gene_id "lnc-bbug03081"	transcript_id "lnc-bbut00007080"	exon_number "6"
11	lncRNA	exon	17863315	17863699	.	-	.	gene_id "lnc-bbug03081"	transcript_id "lnc-bbut00007080"	exon_number "7"
11	lncRNA	exon	17818529	17818587	.	-	.	gene_id "lnc-bbug03081"	transcript_id "lnc-bbut00007081"	exon_number "1"
11	lncRNA	exon	17851557	17851640	.	-	.	gene_id "lnc-bbug03081"	transcript_id "lnc-bbut00007081"	exon_number "2"
11	lncRNA	exon	17854074	17854140	.	-	.	gene_id "lnc-bbug03081"	transcript_id "lnc-bbut00007081"	exon_number "3"
11	lncRNA	exon	17857473	17857510	.	-	.	gene_id "lnc-bbug03081"	transcript_id "lnc-bbut00007081"	exon_number "4"
11	lncRNA	exon	17858223	17858297	.	-	.	gene_id "lnc-bbug03081"	transcript_id "lnc-bbut00007081"	exon_number "5"
11	lncRNA	exon	17858400	17858487	.	-	.	gene_id "lnc-bbug03081"	transcript_id "lnc-bbut00007081"	exon_number "6"
11	lncRNA	exon	17863315	17863694	.	-	.	gene_id "lnc-bbug03081"	transcript_id "lnc-bbut00007081"	exon_number "7"
11	lncRNA	exon	19873668	19873989	.	-	.	gene_id "lnc-bbug03099"	transcript_id "lnc-bbut00007125"	exon_number "1"
11	lncRNA	exon	19886629	19886890	.	-	.	gene_id "lnc-bbug03099"	transcript_id "lnc-bbut00007125"	exon_number "2"
11	lncRNA	exon	19888145	19889170	.	-	.	gene_id "lnc-bbug03099"	transcript_id "lnc-bbut00007125"	exon_number "3"
11	lncRNA	exon	19881383	19881513	.	-	.	gene_id "lnc-bbug03099"	transcript_id "lnc-bbut00007126"	exon_number "1"
11	lncRNA	exon	19886629	19886890	.	-	.	gene_id "lnc-bbug03099"	transcript_id "lnc-bbut00007126"	exon_number "2"
11	lncRNA	exon	19888145	19888526	.	-	.	gene_id "lnc-bbug03099"	transcript_id "lnc-bbut00007126"	exon_number "3"
11	lncRNA	exon	19890144	19890246	.	-	.	gene_id "lnc-bbug03099"	transcript_id "lnc-bbut00007126"	exon_number "4"
11	lncRNA	exon	19893522	19893551	.	-	.	gene_id "lnc-bbug03099"	transcript_id "lnc-bbut00007126"	exon_number "5"
11	lncRNA	exon	19895844	19895930	.	-	.	gene_id "lnc-bbug03099"	transcript_id "lnc-bbut00007126"	exon_number "6"
11	lncRNA	exon	19885715	19886890	.	-	.	gene_id "lnc-bbug03099"	transcript_id "lnc-bbut00007127"	exon_number "1"
11	lncRNA	exon	19888145	19889939	.	-	.	gene_id "lnc-bbug03099"	transcript_id "lnc-bbut00007127"	exon_number "2"
11	lncRNA	exon	19890175	19890246	.	-	.	gene_id "lnc-bbug03099"	transcript_id "lnc-bbut00007128"	exon_number "1"
11	lncRNA	exon	19904184	19907324	.	-	.	gene_id "lnc-bbug03099"	transcript_id "lnc-bbut00007128"	exon_number "2"
11	lncRNA	exon	19951961	19952630	.	-	.	gene_id "lnc-bbug03100"	transcript_id "lnc-bbut00007129"	exon_number "1"
11	lncRNA	exon	19954598	19954708	.	-	.	gene_id "lnc-bbug03100"	transcript_id "lnc-bbut00007129"	exon_number "2"
11	lncRNA	exon	19961441	19961792	.	-	.	gene_id "lnc-bbug03100"	transcript_id "lnc-bbut00007129"	exon_number "3"
11	lncRNA	exon	20129524	20130369	.	-	.	gene_id "lnc-bbug03102"	transcript_id "lnc-bbut00007131"	exon_number "1"
11	lncRNA	exon	20130422	20131387	.	-	.	gene_id "lnc-bbug03102"	transcript_id "lnc-bbut00007131"	exon_number "2"
11	lncRNA	exon	20216592	20216641	.	-	.	gene_id "lnc-bbug03103"	transcript_id "lnc-bbut00007132"	exon_number "1"
11	lncRNA	exon	20230532	20230593	.	-	.	gene_id "lnc-bbug03103"	transcript_id "lnc-bbut00007132"	exon_number "2"
11	lncRNA	exon	20329436	20329496	.	-	.	gene_id "lnc-bbug03103"	transcript_id "lnc-bbut00007132"	exon_number "3"
11	lncRNA	exon	20417917	20418272	.	-	.	gene_id "lnc-bbug03103"	transcript_id "lnc-bbut00007132"	exon_number "4"
11	lncRNA	exon	20787266	20787521	.	-	.	gene_id "lnc-bbug03105"	transcript_id "lnc-bbut00007139"	exon_number "1"
11	lncRNA	exon	20789074	20789203	.	-	.	gene_id "lnc-bbug03105"	transcript_id "lnc-bbut00007139"	exon_number "2"
11	lncRNA	exon	20790612	20791487	.	-	.	gene_id "lnc-bbug03105"	transcript_id "lnc-bbut00007139"	exon_number "3"
11	lncRNA	exon	20795093	20795171	.	-	.	gene_id "lnc-bbug03105"	transcript_id "lnc-bbut00007139"	exon_number "4"
11	lncRNA	exon	21254585	21254759	.	-	.	gene_id "lnc-bbug03110"	transcript_id "lnc-bbut00007144"	exon_number "1"
11	lncRNA	exon	21306349	21306411	.	-	.	gene_id "lnc-bbug03110"	transcript_id "lnc-bbut00007144"	exon_number "2"
11	lncRNA	exon	21310936	21311007	.	-	.	gene_id "lnc-bbug03110"	transcript_id "lnc-bbut00007144"	exon_number "3"
11	lncRNA	exon	21316760	21316985	.	-	.	gene_id "lnc-bbug03110"	transcript_id "lnc-bbut00007144"	exon_number "4"
11	lncRNA	exon	21302889	21303271	.	-	.	gene_id "lnc-bbug03110"	transcript_id "lnc-bbut00007145"	exon_number "1"
11	lncRNA	exon	21306349	21306411	.	-	.	gene_id "lnc-bbug03110"	transcript_id "lnc-bbut00007145"	exon_number "2"
11	lncRNA	exon	21310936	21311007	.	-	.	gene_id "lnc-bbug03110"	transcript_id "lnc-bbut00007145"	exon_number "3"
11	lncRNA	exon	21316760	21316981	.	-	.	gene_id "lnc-bbug03110"	transcript_id "lnc-bbut00007145"	exon_number "4"
11	lncRNA	exon	22704609	22705349	.	-	.	gene_id "lnc-bbug03118"	transcript_id "lnc-bbut00007167"	exon_number "1"
11	lncRNA	exon	22732905	22740531	.	-	.	gene_id "lnc-bbug03119"	transcript_id "lnc-bbut00007168"	exon_number "1"
11	lncRNA	exon	22748789	22749987	.	-	.	gene_id "lnc-bbug03119"	transcript_id "lnc-bbut00007168"	exon_number "2"
11	lncRNA	exon	23777152	23777665	.	-	.	gene_id "lnc-bbug03121"	transcript_id "lnc-bbut00007171"	exon_number "1"
11	lncRNA	exon	23919176	23919526	.	-	.	gene_id "lnc-bbug03124"	transcript_id "lnc-bbut00007175"	exon_number "1"
11	lncRNA	exon	25498774	25499541	.	-	.	gene_id "lnc-bbug03131"	transcript_id "lnc-bbut00007203"	exon_number "1"
11	lncRNA	exon	25500252	25500452	.	-	.	gene_id "lnc-bbug03131"	transcript_id "lnc-bbut00007203"	exon_number "2"
11	lncRNA	exon	25510879	25511601	.	-	.	gene_id "lnc-bbug03131"	transcript_id "lnc-bbut00007203"	exon_number "3"

11	lncRNA	exon	25933854	25934253	.	-	.	gene_id "lnc-bbug03133"	transcript_id "lnc-bbut00007211"	exon_number "1"
11	lncRNA	exon	25943290	25943346	.	-	.	gene_id "lnc-bbug03133"	transcript_id "lnc-bbut00007211"	exon_number "2"
11	lncRNA	exon	25943813	25944065	.	-	.	gene_id "lnc-bbug03133"	transcript_id "lnc-bbut00007211"	exon_number "3"
11	lncRNA	exon	26079229	26080131	.	-	.	gene_id "lnc-bbug03134"	transcript_id "lnc-bbut00007212"	exon_number "1"
11	lncRNA	exon	26117934	26118139	.	-	.	gene_id "lnc-bbug03134"	transcript_id "lnc-bbut00007212"	exon_number "2"
11	lncRNA	exon	26126240	26128089	.	-	.	gene_id "lnc-bbug03134"	transcript_id "lnc-bbut00007212"	exon_number "3"
11	lncRNA	exon	26113879	26114806	.	-	.	gene_id "lnc-bbug03134"	transcript_id "lnc-bbut00007213"	exon_number "1"
11	lncRNA	exon	26117934	26118139	.	-	.	gene_id "lnc-bbug03134"	transcript_id "lnc-bbut00007213"	exon_number "2"
11	lncRNA	exon	26126240	26128089	.	-	.	gene_id "lnc-bbug03134"	transcript_id "lnc-bbut00007213"	exon_number "3"
11	lncRNA	exon	26318430	26319409	.	-	.	gene_id "lnc-bbug03136"	transcript_id "lnc-bbut00007218"	exon_number "1"
11	lncRNA	exon	26323946	26324056	.	-	.	gene_id "lnc-bbug03136"	transcript_id "lnc-bbut00007218"	exon_number "2"
11	lncRNA	exon	26329423	26329496	.	-	.	gene_id "lnc-bbug03136"	transcript_id "lnc-bbut00007218"	exon_number "3"
11	lncRNA	exon	26331485	26331620	.	-	.	gene_id "lnc-bbug03136"	transcript_id "lnc-bbut00007218"	exon_number "4"
11	lncRNA	exon	26339816	26339967	.	-	.	gene_id "lnc-bbug03136"	transcript_id "lnc-bbut00007218"	exon_number "5"
11	lncRNA	exon	26318430	26319409	.	-	.	gene_id "lnc-bbug03136"	transcript_id "lnc-bbut00007217"	exon_number "1"
11	lncRNA	exon	26323946	26324056	.	-	.	gene_id "lnc-bbug03136"	transcript_id "lnc-bbut00007217"	exon_number "2"
11	lncRNA	exon	26329423	26329493	.	-	.	gene_id "lnc-bbug03136"	transcript_id "lnc-bbut00007217"	exon_number "3"
11	lncRNA	exon	26331485	26331620	.	-	.	gene_id "lnc-bbug03136"	transcript_id "lnc-bbut00007217"	exon_number "4"
11	lncRNA	exon	26339816	26339967	.	-	.	gene_id "lnc-bbug03136"	transcript_id "lnc-bbut00007217"	exon_number "5"
11	lncRNA	exon	26321429	26323918	.	-	.	gene_id "lnc-bbug03136"	transcript_id "lnc-bbut00007219"	exon_number "1"
11	lncRNA	exon	26331485	26331620	.	-	.	gene_id "lnc-bbug03136"	transcript_id "lnc-bbut00007219"	exon_number "2"
11	lncRNA	exon	26339816	26339967	.	-	.	gene_id "lnc-bbug03136"	transcript_id "lnc-bbut00007219"	exon_number "3"
11	lncRNA	exon	27471876	27474548	.	-	.	gene_id "lnc-bbug03148"	transcript_id "lnc-bbut00007249"	exon_number "1"
11	lncRNA	exon	27481669	27481692	.	-	.	gene_id "lnc-bbug03148"	transcript_id "lnc-bbut00007249"	exon_number "2"
11	lncRNA	exon	27471876	27474548	.	-	.	gene_id "lnc-bbug03148"	transcript_id "lnc-bbut00007250"	exon_number "1"
11	lncRNA	exon	27497616	27497782	.	-	.	gene_id "lnc-bbug03148"	transcript_id "lnc-bbut00007250"	exon_number "2"
11	lncRNA	exon	29122309	29128385	.	-	.	gene_id "lnc-bbug03151"	transcript_id "lnc-bbut00007253"	exon_number "1"
11	lncRNA	exon	29128491	29128606	.	-	.	gene_id "lnc-bbug03151"	transcript_id "lnc-bbut00007253"	exon_number "2"
11	lncRNA	exon	29139439	29139555	.	-	.	gene_id "lnc-bbug03151"	transcript_id "lnc-bbut00007253"	exon_number "3"
11	lncRNA	exon	29140639	29141520	.	-	.	gene_id "lnc-bbug03151"	transcript_id "lnc-bbut00007253"	exon_number "4"
11	lncRNA	exon	29122309	29128385	.	-	.	gene_id "lnc-bbug03151"	transcript_id "lnc-bbut00007254"	exon_number "1"
11	lncRNA	exon	29128491	29128606	.	-	.	gene_id "lnc-bbug03151"	transcript_id "lnc-bbut00007254"	exon_number "2"
11	lncRNA	exon	29139439	29139555	.	-	.	gene_id "lnc-bbug03151"	transcript_id "lnc-bbut00007254"	exon_number "3"
11	lncRNA	exon	29140661	29141528	.	-	.	gene_id "lnc-bbug03151"	transcript_id "lnc-bbut00007254"	exon_number "4"
11	lncRNA	exon	30570935	30572067	.	-	.	gene_id "lnc-bbug03158"	transcript_id "lnc-bbut00007273"	exon_number "1"
11	lncRNA	exon	30573590	30573694	.	-	.	gene_id "lnc-bbug03158"	transcript_id "lnc-bbut00007273"	exon_number "2"
11	lncRNA	exon	31436562	31438503	.	-	.	gene_id "lnc-bbug03163"	transcript_id "lnc-bbut00007294"	exon_number "1"
11	lncRNA	exon	33315702	33316812	.	-	.	gene_id "lnc-bbug03173"	transcript_id "lnc-bbut00007319"	exon_number "1"
11	lncRNA	exon	33349678	33349800	.	-	.	gene_id "lnc-bbug03173"	transcript_id "lnc-bbut00007319"	exon_number "2"
11	lncRNA	exon	33351615	33351826	.	-	.	gene_id "lnc-bbug03173"	transcript_id "lnc-bbut00007319"	exon_number "3"
11	lncRNA	exon	35247266	35247505	.	-	.	gene_id "lnc-bbug03179"	transcript_id "lnc-bbut00007328"	exon_number "1"
11	lncRNA	exon	35252075	35252296	.	-	.	gene_id "lnc-bbug03179"	transcript_id "lnc-bbut00007328"	exon_number "2"
11	lncRNA	exon	35819107	35819345	.	-	.	gene_id "lnc-bbug03183"	transcript_id "lnc-bbut00007345"	exon_number "1"
11	lncRNA	exon	35829963	35831406	.	-	.	gene_id "lnc-bbug03183"	transcript_id "lnc-bbut00007345"	exon_number "2"
11	lncRNA	exon	36071796	36072895	.	-	.	gene_id "lnc-bbug03186"	transcript_id "lnc-bbut00007348"	exon_number "1"
11	lncRNA	exon	37817111	37817354	.	-	.	gene_id "lnc-bbug03190"	transcript_id "lnc-bbut00007366"	exon_number "1"
11	lncRNA	exon	37829799	37829887	.	-	.	gene_id "lnc-bbug03190"	transcript_id "lnc-bbut00007366"	exon_number "2"
11	lncRNA	exon	37863012	37864856	.	-	.	gene_id "lnc-bbug03190"	transcript_id "lnc-bbut00007366"	exon_number "3"
11	lncRNA	exon	38166096	38167239	.	-	.	gene_id "lnc-bbug03196"	transcript_id "lnc-bbut00007375"	exon_number "1"
11	lncRNA	exon	38168905	38169231	.	-	.	gene_id "lnc-bbug03196"	transcript_id "lnc-bbut00007375"	exon_number "2"
11	lncRNA	exon	38463538	38464363	.	-	.	gene_id "lnc-bbug03200"	transcript_id "lnc-bbut00007379"	exon_number "1"
11	lncRNA	exon	38464693	38464702	.	-	.	gene_id "lnc-bbug03200"	transcript_id "lnc-bbut00007379"	exon_number "2"

11	lncRNA	exon	39000613	39001275	.	-	.	gene_id "lnc-bbug03201"	transcript_id "lnc-bbut00007380"	exon_number "1"
11	lncRNA	exon	41259276	41259325	.	-	.	gene_id "lnc-bbug03204"	transcript_id "lnc-bbut00007386"	exon_number "1"
11	lncRNA	exon	41280625	41280887	.	-	.	gene_id "lnc-bbug03204"	transcript_id "lnc-bbut00007386"	exon_number "2"
11	lncRNA	exon	41296554	41296675	.	-	.	gene_id "lnc-bbug03204"	transcript_id "lnc-bbut00007386"	exon_number "3"
11	lncRNA	exon	41297930	41298037	.	-	.	gene_id "lnc-bbug03204"	transcript_id "lnc-bbut00007386"	exon_number "4"
11	lncRNA	exon	41298535	41298661	.	-	.	gene_id "lnc-bbug03204"	transcript_id "lnc-bbut00007386"	exon_number "5"
11	lncRNA	exon	41309981	41310259	.	-	.	gene_id "lnc-bbug03204"	transcript_id "lnc-bbut00007386"	exon_number "6"
11	lncRNA	exon	41280118	41280887	.	-	.	gene_id "lnc-bbug03204"	transcript_id "lnc-bbut00007387"	exon_number "1"
11	lncRNA	exon	41296554	41296675	.	-	.	gene_id "lnc-bbug03204"	transcript_id "lnc-bbut00007387"	exon_number "2"
11	lncRNA	exon	41297930	41298037	.	-	.	gene_id "lnc-bbug03204"	transcript_id "lnc-bbut00007387"	exon_number "3"
11	lncRNA	exon	41298535	41298661	.	-	.	gene_id "lnc-bbug03204"	transcript_id "lnc-bbut00007387"	exon_number "4"
11	lncRNA	exon	41309981	41310259	.	-	.	gene_id "lnc-bbug03204"	transcript_id "lnc-bbut00007387"	exon_number "5"
11	lncRNA	exon	43499834	43500444	.	-	.	gene_id "lnc-bbug03216"	transcript_id "lnc-bbut00007432"	exon_number "1"
11	lncRNA	exon	43500624	43500634	.	-	.	gene_id "lnc-bbug03216"	transcript_id "lnc-bbut00007432"	exon_number "2"
11	lncRNA	exon	43606815	43607977	.	-	.	gene_id "lnc-bbug03218"	transcript_id "lnc-bbut00007436"	exon_number "1"
11	lncRNA	exon	45144720	45147579	.	-	.	gene_id "lnc-bbug03229"	transcript_id "lnc-bbut00007463"	exon_number "1"
11	lncRNA	exon	45148444	45148466	.	-	.	gene_id "lnc-bbug03229"	transcript_id "lnc-bbut00007463"	exon_number "2"
11	lncRNA	exon	45549450	45553709	.	-	.	gene_id "lnc-bbug03234"	transcript_id "lnc-bbut00007477"	exon_number "1"
11	lncRNA	exon	45557788	45557910	.	-	.	gene_id "lnc-bbug03234"	transcript_id "lnc-bbut00007477"	exon_number "2"
11	lncRNA	exon	45566527	45566654	.	-	.	gene_id "lnc-bbug03234"	transcript_id "lnc-bbut00007477"	exon_number "3"
11	lncRNA	exon	45549450	45553709	.	-	.	gene_id "lnc-bbug03234"	transcript_id "lnc-bbut00007478"	exon_number "1"
11	lncRNA	exon	45557788	45557910	.	-	.	gene_id "lnc-bbug03234"	transcript_id "lnc-bbut00007478"	exon_number "2"
11	lncRNA	exon	45567069	45567123	.	-	.	gene_id "lnc-bbug03234"	transcript_id "lnc-bbut00007478"	exon_number "3"
11	lncRNA	exon	45567369	45567454	.	-	.	gene_id "lnc-bbug03234"	transcript_id "lnc-bbut00007478"	exon_number "4"
11	lncRNA	exon	45786310	45787353	.	-	.	gene_id "lnc-bbug03237"	transcript_id "lnc-bbut00007492"	exon_number "1"
11	lncRNA	exon	45790644	45790732	.	-	.	gene_id "lnc-bbug03237"	transcript_id "lnc-bbut00007492"	exon_number "2"
11	lncRNA	exon	45790947	45791267	.	-	.	gene_id "lnc-bbug03237"	transcript_id "lnc-bbut00007492"	exon_number "3"
11	lncRNA	exon	45792269	45794776	.	-	.	gene_id "lnc-bbug03237"	transcript_id "lnc-bbut00007492"	exon_number "4"
11	lncRNA	exon	45845032	45845599	.	-	.	gene_id "lnc-bbug03238"	transcript_id "lnc-bbut00007493"	exon_number "1"
11	lncRNA	exon	45845887	45845910	.	-	.	gene_id "lnc-bbug03238"	transcript_id "lnc-bbut00007493"	exon_number "2"
11	lncRNA	exon	46910146	46910488	.	-	.	gene_id "lnc-bbug03240"	transcript_id "lnc-bbut00007495"	exon_number "1"
11	lncRNA	exon	48385240	48387291	.	-	.	gene_id "lnc-bbug03243"	transcript_id "lnc-bbut00007504"	exon_number "1"
11	lncRNA	exon	48415776	48415866	.	-	.	gene_id "lnc-bbug03243"	transcript_id "lnc-bbut00007504"	exon_number "2"
11	lncRNA	exon	49022958	49028091	.	-	.	gene_id "lnc-bbug03248"	transcript_id "lnc-bbut00007521"	exon_number "1"
11	lncRNA	exon	49028391	49029041	.	-	.	gene_id "lnc-bbug03248"	transcript_id "lnc-bbut00007521"	exon_number "2"
11	lncRNA	exon	49201863	49204140	.	-	.	gene_id "lnc-bbug03250"	transcript_id "lnc-bbut00007527"	exon_number "1"
11	lncRNA	exon	49223924	49223983	.	-	.	gene_id "lnc-bbug03250"	transcript_id "lnc-bbut00007527"	exon_number "2"
11	lncRNA	exon	50748358	50748634	.	-	.	gene_id "lnc-bbug03253"	transcript_id "lnc-bbut00007547"	exon_number "1"
11	lncRNA	exon	50766419	50766565	.	-	.	gene_id "lnc-bbug03253"	transcript_id "lnc-bbut00007547"	exon_number "2"
11	lncRNA	exon	50777732	50778113	.	-	.	gene_id "lnc-bbug03253"	transcript_id "lnc-bbut00007547"	exon_number "3"
11	lncRNA	exon	51634840	51636237	.	-	.	gene_id "lnc-bbug03255"	transcript_id "lnc-bbut00007549"	exon_number "1"
11	lncRNA	exon	51663521	51663640	.	-	.	gene_id "lnc-bbug03255"	transcript_id "lnc-bbut00007549"	exon_number "2"
11	lncRNA	exon	51666443	51666864	.	-	.	gene_id "lnc-bbug03255"	transcript_id "lnc-bbut00007549"	exon_number "3"
11	lncRNA	exon	51660528	51661019	.	-	.	gene_id "lnc-bbug03255"	transcript_id "lnc-bbut00007550"	exon_number "1"
11	lncRNA	exon	51663521	51663640	.	-	.	gene_id "lnc-bbug03255"	transcript_id "lnc-bbut00007550"	exon_number "2"
11	lncRNA	exon	51666443	51666865	.	-	.	gene_id "lnc-bbug03255"	transcript_id "lnc-bbut00007550"	exon_number "3"
11	lncRNA	exon	51667645	51668488	.	-	.	gene_id "lnc-bbug03256"	transcript_id "lnc-bbut00007551"	exon_number "1"
11	lncRNA	exon	51668683	51673096	.	-	.	gene_id "lnc-bbug03256"	transcript_id "lnc-bbut00007551"	exon_number "2"
11	lncRNA	exon	52022495	52023036	.	-	.	gene_id "lnc-bbug03261"	transcript_id "lnc-bbut00007567"	exon_number "1"
11	lncRNA	exon	52023147	52023614	.	-	.	gene_id "lnc-bbug03261"	transcript_id "lnc-bbut00007567"	exon_number "2"
11	lncRNA	exon	52035792	52035970	.	-	.	gene_id "lnc-bbug03261"	transcript_id "lnc-bbut00007567"	exon_number "3"
11	lncRNA	exon	52722704	52723000	.	-	.	gene_id "lnc-bbug03264"	transcript_id "lnc-bbut00007573"	exon_number "1"

11	lncRNA	exon	52725416	52725542	.	-	.	gene_id "lnc-bbug03264"	transcript_id "lnc-bbut00007573"	exon_number "2"
11	lncRNA	exon	52729177	52729281	.	-	.	gene_id "lnc-bbug03264"	transcript_id "lnc-bbut00007573"	exon_number "3"
11	lncRNA	exon	52729915	52730035	.	-	.	gene_id "lnc-bbug03264"	transcript_id "lnc-bbut00007573"	exon_number "4"
11	lncRNA	exon	52756347	52756497	.	-	.	gene_id "lnc-bbug03264"	transcript_id "lnc-bbut00007573"	exon_number "5"
11	lncRNA	exon	52722704	52723000	.	-	.	gene_id "lnc-bbug03264"	transcript_id "lnc-bbut00007574"	exon_number "1"
11	lncRNA	exon	52725416	52725542	.	-	.	gene_id "lnc-bbug03264"	transcript_id "lnc-bbut00007574"	exon_number "2"
11	lncRNA	exon	52729177	52729281	.	-	.	gene_id "lnc-bbug03264"	transcript_id "lnc-bbut00007574"	exon_number "3"
11	lncRNA	exon	52729915	52730035	.	-	.	gene_id "lnc-bbug03264"	transcript_id "lnc-bbut00007574"	exon_number "4"
11	lncRNA	exon	52756417	52756506	.	-	.	gene_id "lnc-bbug03264"	transcript_id "lnc-bbut00007574"	exon_number "5"
11	lncRNA	exon	53018668	53020278	.	-	.	gene_id "lnc-bbug03266"	transcript_id "lnc-bbut00007576"	exon_number "1"
11	lncRNA	exon	53022866	53022961	.	-	.	gene_id "lnc-bbug03266"	transcript_id "lnc-bbut00007576"	exon_number "2"
11	lncRNA	exon	53282424	53282734	.	-	.	gene_id "lnc-bbug03267"	transcript_id "lnc-bbut00007577"	exon_number "1"
11	lncRNA	exon	53477135	53479013	.	-	.	gene_id "lnc-bbug03268"	transcript_id "lnc-bbut00007578"	exon_number "1"
11	lncRNA	exon	53480102	53480212	.	-	.	gene_id "lnc-bbug03268"	transcript_id "lnc-bbut00007578"	exon_number "2"
11	lncRNA	exon	53481239	53484408	.	-	.	gene_id "lnc-bbug03268"	transcript_id "lnc-bbut00007578"	exon_number "3"
11	lncRNA	exon	55019916	55020123	.	-	.	gene_id "lnc-bbug03272"	transcript_id "lnc-bbut00007586"	exon_number "1"
11	lncRNA	exon	55023956	55024024	.	-	.	gene_id "lnc-bbug03272"	transcript_id "lnc-bbut00007586"	exon_number "2"
11	lncRNA	exon	55025057	55025137	.	-	.	gene_id "lnc-bbug03272"	transcript_id "lnc-bbut00007586"	exon_number "3"
11	lncRNA	exon	55043887	55044127	.	-	.	gene_id "lnc-bbug03272"	transcript_id "lnc-bbut00007586"	exon_number "4"
11	lncRNA	exon	55451841	55456581	.	-	.	gene_id "lnc-bbug03276"	transcript_id "lnc-bbut00007593"	exon_number "1"
11	lncRNA	exon	55458263	55458334	.	-	.	gene_id "lnc-bbug03276"	transcript_id "lnc-bbut00007593"	exon_number "2"
11	lncRNA	exon	55461338	55465654	.	-	.	gene_id "lnc-bbug03276"	transcript_id "lnc-bbut00007593"	exon_number "3"
11	lncRNA	exon	56154179	56154531	.	-	.	gene_id "lnc-bbug03277"	transcript_id "lnc-bbut00007594"	exon_number "1"
11	lncRNA	exon	56156897	56157141	.	-	.	gene_id "lnc-bbug03277"	transcript_id "lnc-bbut00007594"	exon_number "2"
11	lncRNA	exon	56157649	56157770	.	-	.	gene_id "lnc-bbug03277"	transcript_id "lnc-bbut00007594"	exon_number "3"
11	lncRNA	exon	56357033	56357514	.	-	.	gene_id "lnc-bbug03278"	transcript_id "lnc-bbut00007595"	exon_number "1"
11	lncRNA	exon	56364364	56364462	.	-	.	gene_id "lnc-bbug03278"	transcript_id "lnc-bbut00007595"	exon_number "2"
11	lncRNA	exon	56364836	56365041	.	-	.	gene_id "lnc-bbug03278"	transcript_id "lnc-bbut00007595"	exon_number "3"
11	lncRNA	exon	56477595	56479029	.	-	.	gene_id "lnc-bbug03280"	transcript_id "lnc-bbut00007598"	exon_number "1"
11	lncRNA	exon	56479707	56481423	.	-	.	gene_id "lnc-bbug03280"	transcript_id "lnc-bbut00007598"	exon_number "2"
11	lncRNA	exon	56477595	56479029	.	-	.	gene_id "lnc-bbug03280"	transcript_id "lnc-bbut00007597"	exon_number "1"
11	lncRNA	exon	56479700	56481423	.	-	.	gene_id "lnc-bbug03280"	transcript_id "lnc-bbut00007597"	exon_number "2"
11	lncRNA	exon	57285565	57285598	.	-	.	gene_id "lnc-bbug03283"	transcript_id "lnc-bbut00007618"	exon_number "1"
11	lncRNA	exon	57285720	57285809	.	-	.	gene_id "lnc-bbug03283"	transcript_id "lnc-bbut00007618"	exon_number "2"
11	lncRNA	exon	57287792	57293426	.	-	.	gene_id "lnc-bbug03283"	transcript_id "lnc-bbut00007618"	exon_number "3"
11	lncRNA	exon	57474202	57474669	.	-	.	gene_id "lnc-bbug03285"	transcript_id "lnc-bbut00007622"	exon_number "1"
11	lncRNA	exon	57475501	57475546	.	-	.	gene_id "lnc-bbug03285"	transcript_id "lnc-bbut00007622"	exon_number "2"
11	lncRNA	exon	57485441	57485605	.	-	.	gene_id "lnc-bbug03285"	transcript_id "lnc-bbut00007622"	exon_number "3"
11	lncRNA	exon	57489526	57489832	.	-	.	gene_id "lnc-bbug03285"	transcript_id "lnc-bbut00007622"	exon_number "4"
11	lncRNA	exon	57712397	57713407	.	-	.	gene_id "lnc-bbug03288"	transcript_id "lnc-bbut00007640"	exon_number "1"
11	lncRNA	exon	57718782	57718944	.	-	.	gene_id "lnc-bbug03288"	transcript_id "lnc-bbut00007640"	exon_number "2"
11	lncRNA	exon	57720098	57720178	.	-	.	gene_id "lnc-bbug03288"	transcript_id "lnc-bbut00007640"	exon_number "3"
11	lncRNA	exon	57720480	57720638	.	-	.	gene_id "lnc-bbug03288"	transcript_id "lnc-bbut00007640"	exon_number "4"
11	lncRNA	exon	57927032	57927619	.	-	.	gene_id "lnc-bbug03289"	transcript_id "lnc-bbut00007641"	exon_number "1"
11	lncRNA	exon	57941185	57941321	.	-	.	gene_id "lnc-bbug03289"	transcript_id "lnc-bbut00007641"	exon_number "2"
11	lncRNA	exon	57946229	57946495	.	-	.	gene_id "lnc-bbug03289"	transcript_id "lnc-bbut00007641"	exon_number "3"
11	lncRNA	exon	57990109	57991349	.	-	.	gene_id "lnc-bbug03290"	transcript_id "lnc-bbut00007642"	exon_number "1"
11	lncRNA	exon	57991574	57991659	.	-	.	gene_id "lnc-bbug03290"	transcript_id "lnc-bbut00007642"	exon_number "2"
11	lncRNA	exon	57992523	57992698	.	-	.	gene_id "lnc-bbug03290"	transcript_id "lnc-bbut00007642"	exon_number "3"
11	lncRNA	exon	57993845	57994002	.	-	.	gene_id "lnc-bbug03290"	transcript_id "lnc-bbut00007642"	exon_number "4"
11	lncRNA	exon	58002697	58002923	.	-	.	gene_id "lnc-bbug03290"	transcript_id "lnc-bbut00007642"	exon_number "5"
11	lncRNA	exon	58030133	58031223	.	-	.	gene_id "lnc-bbug03291"	transcript_id "lnc-bbut00007643"	exon_number "1"

11	lncRNA	exon	58031479	58031548	.	-	.	gene_id "lnc-bbug03291"	transcript_id "lnc-bbut00007643"	exon_number "2"
11	lncRNA	exon	58038181	58038329	.	-	.	gene_id "lnc-bbug03291"	transcript_id "lnc-bbut00007643"	exon_number "3"
11	lncRNA	exon	58038808	58038958	.	-	.	gene_id "lnc-bbug03291"	transcript_id "lnc-bbut00007643"	exon_number "4"
11	lncRNA	exon	58039272	58039334	.	-	.	gene_id "lnc-bbug03291"	transcript_id "lnc-bbut00007643"	exon_number "5"
11	lncRNA	exon	58040065	58040103	.	-	.	gene_id "lnc-bbug03291"	transcript_id "lnc-bbut00007643"	exon_number "6"
11	lncRNA	exon	58040673	58040736	.	-	.	gene_id "lnc-bbug03291"	transcript_id "lnc-bbut00007643"	exon_number "7"
11	lncRNA	exon	58201965	58204175	.	-	.	gene_id "lnc-bbug03294"	transcript_id "lnc-bbut00007648"	exon_number "1"
11	lncRNA	exon	58204418	58204802	.	-	.	gene_id "lnc-bbug03294"	transcript_id "lnc-bbut00007648"	exon_number "2"
11	lncRNA	exon	58589054	58592283	.	-	.	gene_id "lnc-bbug03296"	transcript_id "lnc-bbut00007651"	exon_number "1"
11	lncRNA	exon	58611637	58611750	.	-	.	gene_id "lnc-bbug03296"	transcript_id "lnc-bbut00007651"	exon_number "2"
11	lncRNA	exon	58698741	58698781	.	-	.	gene_id "lnc-bbug03296"	transcript_id "lnc-bbut00007651"	exon_number "3"
11	lncRNA	exon	58699608	58699721	.	-	.	gene_id "lnc-bbug03296"	transcript_id "lnc-bbut00007651"	exon_number "4"
11	lncRNA	exon	58589054	58592283	.	-	.	gene_id "lnc-bbug03296"	transcript_id "lnc-bbut00007652"	exon_number "1"
11	lncRNA	exon	58611637	58611750	.	-	.	gene_id "lnc-bbug03296"	transcript_id "lnc-bbut00007652"	exon_number "2"
11	lncRNA	exon	58698741	58698781	.	-	.	gene_id "lnc-bbug03296"	transcript_id "lnc-bbut00007652"	exon_number "3"
11	lncRNA	exon	58702804	58702906	.	-	.	gene_id "lnc-bbug03296"	transcript_id "lnc-bbut00007652"	exon_number "4"
11	lncRNA	exon	58589054	58592283	.	-	.	gene_id "lnc-bbug03296"	transcript_id "lnc-bbut00007653"	exon_number "1"
11	lncRNA	exon	58611637	58611750	.	-	.	gene_id "lnc-bbug03296"	transcript_id "lnc-bbut00007653"	exon_number "2"
11	lncRNA	exon	58698741	58698781	.	-	.	gene_id "lnc-bbug03296"	transcript_id "lnc-bbut00007653"	exon_number "3"
11	lncRNA	exon	58717567	58717641	.	-	.	gene_id "lnc-bbug03296"	transcript_id "lnc-bbut00007653"	exon_number "4"
11	lncRNA	exon	58589054	58592283	.	-	.	gene_id "lnc-bbug03296"	transcript_id "lnc-bbut00007654"	exon_number "1"
11	lncRNA	exon	58611637	58611750	.	-	.	gene_id "lnc-bbug03296"	transcript_id "lnc-bbut00007654"	exon_number "2"
11	lncRNA	exon	58698741	58698781	.	-	.	gene_id "lnc-bbug03296"	transcript_id "lnc-bbut00007654"	exon_number "3"
11	lncRNA	exon	58717921	58718081	.	-	.	gene_id "lnc-bbug03296"	transcript_id "lnc-bbut00007654"	exon_number "4"
11	lncRNA	exon	58592290	58593281	.	-	.	gene_id "lnc-bbug03297"	transcript_id "lnc-bbut00007655"	exon_number "1"
11	lncRNA	exon	58598823	58598973	.	-	.	gene_id "lnc-bbug03297"	transcript_id "lnc-bbut00007655"	exon_number "2"
11	lncRNA	exon	58602679	58604644	.	-	.	gene_id "lnc-bbug03297"	transcript_id "lnc-bbut00007655"	exon_number "3"
11	lncRNA	exon	59390565	59393737	.	-	.	gene_id "lnc-bbug03300"	transcript_id "lnc-bbut00007660"	exon_number "1"
11	lncRNA	exon	59486063	59488906	.	-	.	gene_id "lnc-bbug03301"	transcript_id "lnc-bbut00007661"	exon_number "1"
11	lncRNA	exon	59489194	59489241	.	-	.	gene_id "lnc-bbug03301"	transcript_id "lnc-bbut00007661"	exon_number "2"
11	lncRNA	exon	59504031	59506351	.	-	.	gene_id "lnc-bbug03301"	transcript_id "lnc-bbut00007661"	exon_number "3"
11	lncRNA	exon	60364006	60364622	.	-	.	gene_id "lnc-bbug03304"	transcript_id "lnc-bbut00007665"	exon_number "1"
11	lncRNA	exon	60388935	60389854	.	-	.	gene_id "lnc-bbug03305"	transcript_id "lnc-bbut00007666"	exon_number "1"
11	lncRNA	exon	60391309	60393769	.	-	.	gene_id "lnc-bbug03305"	transcript_id "lnc-bbut00007666"	exon_number "2"
11	lncRNA	exon	60388935	60389854	.	-	.	gene_id "lnc-bbug03305"	transcript_id "lnc-bbut00007667"	exon_number "1"
11	lncRNA	exon	60391043	60393778	.	-	.	gene_id "lnc-bbug03305"	transcript_id "lnc-bbut00007667"	exon_number "2"
11	lncRNA	exon	60406112	60408090	.	-	.	gene_id "lnc-bbug03306"	transcript_id "lnc-bbut00007668"	exon_number "1"
11	lncRNA	exon	60408467	60408843	.	-	.	gene_id "lnc-bbug03306"	transcript_id "lnc-bbut00007668"	exon_number "2"
11	lncRNA	exon	60471158	60472675	.	-	.	gene_id "lnc-bbug03308"	transcript_id "lnc-bbut00007670"	exon_number "1"
11	lncRNA	exon	60473350	60473425	.	-	.	gene_id "lnc-bbug03308"	transcript_id "lnc-bbut00007670"	exon_number "2"
11	lncRNA	exon	60478770	60483024	.	-	.	gene_id "lnc-bbug03308"	transcript_id "lnc-bbut00007670"	exon_number "3"
11	lncRNA	exon	60726730	60726741	.	-	.	gene_id "lnc-bbug03313"	transcript_id "lnc-bbut00007681"	exon_number "1"
11	lncRNA	exon	60729327	60729425	.	-	.	gene_id "lnc-bbug03313"	transcript_id "lnc-bbut00007681"	exon_number "2"
11	lncRNA	exon	60731312	60731370	.	-	.	gene_id "lnc-bbug03313"	transcript_id "lnc-bbut00007681"	exon_number "3"
11	lncRNA	exon	60732573	60733085	.	-	.	gene_id "lnc-bbug03313"	transcript_id "lnc-bbut00007681"	exon_number "4"
11	lncRNA	exon	60731377	60732186	.	-	.	gene_id "lnc-bbug03313"	transcript_id "lnc-bbut00007682"	exon_number "1"
11	lncRNA	exon	60732573	60733098	.	-	.	gene_id "lnc-bbug03313"	transcript_id "lnc-bbut00007682"	exon_number "2"
11	lncRNA	exon	65183910	65184423	.	-	.	gene_id "lnc-bbug03317"	transcript_id "lnc-bbut00007690"	exon_number "1"
11	lncRNA	exon	65235690	65236209	.	-	.	gene_id "lnc-bbug03318"	transcript_id "lnc-bbut00007691"	exon_number "1"
11	lncRNA	exon	65447015	65447684	.	-	.	gene_id "lnc-bbug03320"	transcript_id "lnc-bbut00007701"	exon_number "1"
11	lncRNA	exon	65448818	65449194	.	-	.	gene_id "lnc-bbug03320"	transcript_id "lnc-bbut00007701"	exon_number "2"
11	lncRNA	exon	65473132	65474579	.	-	.	gene_id "lnc-bbug03321"	transcript_id "lnc-bbut00007702"	exon_number "1"

11	lncRNA	exon	65476313	65476421	.	-	.	gene_id "lnc-bbug03321"	transcript_id "lnc-bbut00007702"	exon_number "2"
11	lncRNA	exon	65480031	65480100	.	-	.	gene_id "lnc-bbug03321"	transcript_id "lnc-bbut00007702"	exon_number "3"
11	lncRNA	exon	65480430	65480465	.	-	.	gene_id "lnc-bbug03321"	transcript_id "lnc-bbut00007702"	exon_number "4"
11	lncRNA	exon	65936091	65936640	.	-	.	gene_id "lnc-bbug03326"	transcript_id "lnc-bbut00007725"	exon_number "1"
11	lncRNA	exon	65939692	65939974	.	-	.	gene_id "lnc-bbug03326"	transcript_id "lnc-bbut00007725"	exon_number "2"
11	lncRNA	exon	65940653	65941700	.	-	.	gene_id "lnc-bbug03326"	transcript_id "lnc-bbut00007725"	exon_number "3"
11	lncRNA	exon	66576534	66584162	.	-	.	gene_id "lnc-bbug03334"	transcript_id "lnc-bbut00007751"	exon_number "1"
11	lncRNA	exon	66587299	66587435	.	-	.	gene_id "lnc-bbug03334"	transcript_id "lnc-bbut00007751"	exon_number "2"
11	lncRNA	exon	66590943	66591023	.	-	.	gene_id "lnc-bbug03334"	transcript_id "lnc-bbut00007751"	exon_number "3"
11	lncRNA	exon	66592532	66592688	.	-	.	gene_id "lnc-bbug03334"	transcript_id "lnc-bbut00007751"	exon_number "4"
11	lncRNA	exon	66576534	66584162	.	-	.	gene_id "lnc-bbug03334"	transcript_id "lnc-bbut00007750"	exon_number "1"
11	lncRNA	exon	66590943	66591023	.	-	.	gene_id "lnc-bbug03334"	transcript_id "lnc-bbut00007750"	exon_number "2"
11	lncRNA	exon	66592532	66592688	.	-	.	gene_id "lnc-bbug03334"	transcript_id "lnc-bbut00007750"	exon_number "3"
11	lncRNA	exon	67035938	67036450	.	-	.	gene_id "lnc-bbug03342"	transcript_id "lnc-bbut00007763"	exon_number "1"
11	lncRNA	exon	67037689	67037783	.	-	.	gene_id "lnc-bbug03342"	transcript_id "lnc-bbut00007763"	exon_number "2"
11	lncRNA	exon	67039513	67039963	.	-	.	gene_id "lnc-bbug03342"	transcript_id "lnc-bbut00007763"	exon_number "3"
11	lncRNA	exon	69553396	69553743	.	-	.	gene_id "lnc-bbug03359"	transcript_id "lnc-bbut00007798"	exon_number "1"
11	lncRNA	exon	69556948	69556989	.	-	.	gene_id "lnc-bbug03359"	transcript_id "lnc-bbut00007798"	exon_number "2"
11	lncRNA	exon	69563136	69563263	.	-	.	gene_id "lnc-bbug03359"	transcript_id "lnc-bbut00007798"	exon_number "3"
11	lncRNA	exon	69566421	69568846	.	-	.	gene_id "lnc-bbug03359"	transcript_id "lnc-bbut00007798"	exon_number "4"
11	lncRNA	exon	69553396	69553743	.	-	.	gene_id "lnc-bbug03359"	transcript_id "lnc-bbut00007797"	exon_number "1"
11	lncRNA	exon	69563136	69563263	.	-	.	gene_id "lnc-bbug03359"	transcript_id "lnc-bbut00007797"	exon_number "2"
11	lncRNA	exon	69566421	69568846	.	-	.	gene_id "lnc-bbug03359"	transcript_id "lnc-bbut00007797"	exon_number "3"
11	lncRNA	exon	70583041	70583811	.	-	.	gene_id "lnc-bbug03361"	transcript_id "lnc-bbut00007804"	exon_number "1"
11	lncRNA	exon	70589926	70591085	.	-	.	gene_id "lnc-bbug03361"	transcript_id "lnc-bbut00007804"	exon_number "2"
11	lncRNA	exon	72305857	72306730	.	-	.	gene_id "lnc-bbug03364"	transcript_id "lnc-bbut00007815"	exon_number "1"
11	lncRNA	exon	72345308	72345407	.	-	.	gene_id "lnc-bbug03364"	transcript_id "lnc-bbut00007815"	exon_number "2"
11	lncRNA	exon	72353191	72353512	.	-	.	gene_id "lnc-bbug03364"	transcript_id "lnc-bbut00007815"	exon_number "3"
11	lncRNA	exon	72784511	72790088	.	-	.	gene_id "lnc-bbug03365"	transcript_id "lnc-bbut00007816"	exon_number "1"
11	lncRNA	exon	72814448	72814571	.	-	.	gene_id "lnc-bbug03365"	transcript_id "lnc-bbut00007816"	exon_number "2"
11	lncRNA	exon	72820719	72820890	.	-	.	gene_id "lnc-bbug03365"	transcript_id "lnc-bbut00007816"	exon_number "3"
11	lncRNA	exon	72835588	72835735	.	-	.	gene_id "lnc-bbug03365"	transcript_id "lnc-bbut00007816"	exon_number "4"
11	lncRNA	exon	72926909	72926978	.	-	.	gene_id "lnc-bbug03365"	transcript_id "lnc-bbut00007816"	exon_number "5"
11	lncRNA	exon	72927675	72927943	.	-	.	gene_id "lnc-bbug03365"	transcript_id "lnc-bbut00007816"	exon_number "6"
11	lncRNA	exon	72784511	72790088	.	-	.	gene_id "lnc-bbug03365"	transcript_id "lnc-bbut00007817"	exon_number "1"
11	lncRNA	exon	72797559	72797644	.	-	.	gene_id "lnc-bbug03365"	transcript_id "lnc-bbut00007817"	exon_number "2"
11	lncRNA	exon	72814448	72814571	.	-	.	gene_id "lnc-bbug03365"	transcript_id "lnc-bbut00007817"	exon_number "3"
11	lncRNA	exon	72820719	72820890	.	-	.	gene_id "lnc-bbug03365"	transcript_id "lnc-bbut00007817"	exon_number "4"
11	lncRNA	exon	72926909	72926978	.	-	.	gene_id "lnc-bbug03365"	transcript_id "lnc-bbut00007817"	exon_number "5"
11	lncRNA	exon	72927675	72927944	.	-	.	gene_id "lnc-bbug03365"	transcript_id "lnc-bbut00007817"	exon_number "6"
11	lncRNA	exon	72784511	72790088	.	-	.	gene_id "lnc-bbug03365"	transcript_id "lnc-bbut00007818"	exon_number "1"
11	lncRNA	exon	72797559	72797644	.	-	.	gene_id "lnc-bbug03365"	transcript_id "lnc-bbut00007818"	exon_number "2"
11	lncRNA	exon	72814448	72814571	.	-	.	gene_id "lnc-bbug03365"	transcript_id "lnc-bbut00007818"	exon_number "3"
11	lncRNA	exon	72820719	72820890	.	-	.	gene_id "lnc-bbug03365"	transcript_id "lnc-bbut00007818"	exon_number "4"
11	lncRNA	exon	72835588	72835735	.	-	.	gene_id "lnc-bbug03365"	transcript_id "lnc-bbut00007818"	exon_number "5"
11	lncRNA	exon	72926909	72926978	.	-	.	gene_id "lnc-bbug03365"	transcript_id "lnc-bbut00007818"	exon_number "6"
11	lncRNA	exon	72927675	72927946	.	-	.	gene_id "lnc-bbug03365"	transcript_id "lnc-bbut00007818"	exon_number "7"
11	lncRNA	exon	72799129	72802053	.	-	.	gene_id "lnc-bbug03365"	transcript_id "lnc-bbut00007819"	exon_number "1"
11	lncRNA	exon	72814448	72814571	.	-	.	gene_id "lnc-bbug03365"	transcript_id "lnc-bbut00007819"	exon_number "2"
11	lncRNA	exon	72820719	72820890	.	-	.	gene_id "lnc-bbug03365"	transcript_id "lnc-bbut00007819"	exon_number "3"
11	lncRNA	exon	72835588	72835735	.	-	.	gene_id "lnc-bbug03365"	transcript_id "lnc-bbut00007819"	exon_number "4"
11	lncRNA	exon	72926909	72926978	.	-	.	gene_id "lnc-bbug03365"	transcript_id "lnc-bbut00007819"	exon_number "5"

11	lncRNA	exon	72927675	72927944	.	-	.	gene_id "lnc-bbug03365"	transcript_id "lnc-bbut00007819"	exon_number "6"
11	lncRNA	exon	73305847	73306413	.	-	.	gene_id "lnc-bbug03366"	transcript_id "lnc-bbut00007820"	exon_number "1"
11	lncRNA	exon	73306849	73306942	.	-	.	gene_id "lnc-bbug03366"	transcript_id "lnc-bbut00007820"	exon_number "2"
11	lncRNA	exon	74895801	74896301	.	-	.	gene_id "lnc-bbug03370"	transcript_id "lnc-bbut00007837"	exon_number "1"
11	lncRNA	exon	74899994	74900183	.	-	.	gene_id "lnc-bbug03370"	transcript_id "lnc-bbut00007837"	exon_number "2"
11	lncRNA	exon	75118120	75118506	.	-	.	gene_id "lnc-bbug03374"	transcript_id "lnc-bbut00007843"	exon_number "1"
11	lncRNA	exon	75119075	75119229	.	-	.	gene_id "lnc-bbug03374"	transcript_id "lnc-bbut00007843"	exon_number "2"
11	lncRNA	exon	75144753	75145227	.	-	.	gene_id "lnc-bbug03376"	transcript_id "lnc-bbut00007845"	exon_number "1"
11	lncRNA	exon	75831213	75831422	.	-	.	gene_id "lnc-bbug03398"	transcript_id "lnc-bbut00007867"	exon_number "1"
11	lncRNA	exon	75833795	75833805	.	-	.	gene_id "lnc-bbug03398"	transcript_id "lnc-bbut00007867"	exon_number "2"
11	lncRNA	exon	75839542	75839560	.	-	.	gene_id "lnc-bbug03398"	transcript_id "lnc-bbut00007867"	exon_number "3"
11	lncRNA	exon	75839799	75840449	.	-	.	gene_id "lnc-bbug03398"	transcript_id "lnc-bbut00007867"	exon_number "4"
11	lncRNA	exon	75842151	75842170	.	-	.	gene_id "lnc-bbug03398"	transcript_id "lnc-bbut00007867"	exon_number "5"
11	lncRNA	exon	76562886	76563586	.	-	.	gene_id "lnc-bbug03427"	transcript_id "lnc-bbut00007896"	exon_number "1"
11	lncRNA	exon	76563906	76564603	.	-	.	gene_id "lnc-bbug03427"	transcript_id "lnc-bbut00007896"	exon_number "2"
11	lncRNA	exon	76564737	76564771	.	-	.	gene_id "lnc-bbug03427"	transcript_id "lnc-bbut00007896"	exon_number "3"
11	lncRNA	exon	76642685	76642800	.	-	.	gene_id "lnc-bbug03431"	transcript_id "lnc-bbut00007903"	exon_number "1"
11	lncRNA	exon	76644069	76644193	.	-	.	gene_id "lnc-bbug03431"	transcript_id "lnc-bbut00007903"	exon_number "2"
11	lncRNA	exon	76645364	76645442	.	-	.	gene_id "lnc-bbug03431"	transcript_id "lnc-bbut00007903"	exon_number "3"
11	lncRNA	exon	76642685	76642800	.	-	.	gene_id "lnc-bbug03431"	transcript_id "lnc-bbut00007902"	exon_number "1"
11	lncRNA	exon	76644069	76644178	.	-	.	gene_id "lnc-bbug03431"	transcript_id "lnc-bbut00007902"	exon_number "2"
11	lncRNA	exon	76645364	76645442	.	-	.	gene_id "lnc-bbug03431"	transcript_id "lnc-bbut00007902"	exon_number "3"
11	lncRNA	exon	76888854	76889469	.	-	.	gene_id "lnc-bbug03438"	transcript_id "lnc-bbut00007918"	exon_number "1"
11	lncRNA	exon	76889893	76889898	.	-	.	gene_id "lnc-bbug03438"	transcript_id "lnc-bbut00007918"	exon_number "2"
11	lncRNA	exon	77208429	77209497	.	-	.	gene_id "lnc-bbug03456"	transcript_id "lnc-bbut00007941"	exon_number "1"
11	lncRNA	exon	77210366	77210564	.	-	.	gene_id "lnc-bbug03456"	transcript_id "lnc-bbut00007941"	exon_number "2"
11	lncRNA	exon	77326540	77327055	.	-	.	gene_id "lnc-bbug03459"	transcript_id "lnc-bbut00007951"	exon_number "1"
11	lncRNA	exon	77327736	77327870	.	-	.	gene_id "lnc-bbug03459"	transcript_id "lnc-bbut00007951"	exon_number "2"
11	lncRNA	exon	77329122	77329263	.	-	.	gene_id "lnc-bbug03459"	transcript_id "lnc-bbut00007951"	exon_number "3"
11	lncRNA	exon	77333938	77334031	.	-	.	gene_id "lnc-bbug03459"	transcript_id "lnc-bbut00007951"	exon_number "4"
11	lncRNA	exon	77353728	77354758	.	-	.	gene_id "lnc-bbug03459"	transcript_id "lnc-bbut00007951"	exon_number "5"
11	lncRNA	exon	77326540	77327055	.	-	.	gene_id "lnc-bbug03459"	transcript_id "lnc-bbut00007952"	exon_number "1"
11	lncRNA	exon	77327736	77327870	.	-	.	gene_id "lnc-bbug03459"	transcript_id "lnc-bbut00007952"	exon_number "2"
11	lncRNA	exon	77329122	77329263	.	-	.	gene_id "lnc-bbug03459"	transcript_id "lnc-bbut00007952"	exon_number "3"
11	lncRNA	exon	77333938	77334031	.	-	.	gene_id "lnc-bbug03459"	transcript_id "lnc-bbut00007952"	exon_number "4"
11	lncRNA	exon	77354787	77355323	.	-	.	gene_id "lnc-bbug03459"	transcript_id "lnc-bbut00007952"	exon_number "5"
11	lncRNA	exon	77511044	77512502	.	-	.	gene_id "lnc-bbug03463"	transcript_id "lnc-bbut00007971"	exon_number "1"
11	lncRNA	exon	77763825	77764647	.	-	.	gene_id "lnc-bbug03469"	transcript_id "lnc-bbut00007985"	exon_number "1"
11	lncRNA	exon	79046349	79047041	.	-	.	gene_id "lnc-bbug03474"	transcript_id "lnc-bbut00007990"	exon_number "1"
11	lncRNA	exon	79047210	79047384	.	-	.	gene_id "lnc-bbug03474"	transcript_id "lnc-bbut00007990"	exon_number "2"
11	lncRNA	exon	79078873	79079302	.	-	.	gene_id "lnc-bbug03478"	transcript_id "lnc-bbut00007994"	exon_number "1"
11	lncRNA	exon	79079471	79079929	.	-	.	gene_id "lnc-bbug03478"	transcript_id "lnc-bbut00007994"	exon_number "2"
11	lncRNA	exon	79091873	79092284	.	-	.	gene_id "lnc-bbug03480"	transcript_id "lnc-bbut00007996"	exon_number "1"
11	lncRNA	exon	79092456	79092726	.	-	.	gene_id "lnc-bbug03480"	transcript_id "lnc-bbut00007996"	exon_number "2"
11	lncRNA	exon	79108672	79109006	.	-	.	gene_id "lnc-bbug03481"	transcript_id "lnc-bbut00007997"	exon_number "1"
11	lncRNA	exon	79109175	79109334	.	-	.	gene_id "lnc-bbug03481"	transcript_id "lnc-bbut00007997"	exon_number "2"
11	lncRNA	exon	79143173	79143586	.	-	.	gene_id "lnc-bbug03483"	transcript_id "lnc-bbut00007999"	exon_number "1"
11	lncRNA	exon	79143786	79144006	.	-	.	gene_id "lnc-bbug03483"	transcript_id "lnc-bbut00007999"	exon_number "2"
11	lncRNA	exon	79188192	79188228	.	-	.	gene_id "lnc-bbug03488"	transcript_id "lnc-bbut00008006"	exon_number "1"
11	lncRNA	exon	79191759	79192131	.	-	.	gene_id "lnc-bbug03488"	transcript_id "lnc-bbut00008006"	exon_number "2"
11	lncRNA	exon	79192504	79192555	.	-	.	gene_id "lnc-bbug03488"	transcript_id "lnc-bbut00008006"	exon_number "3"
11	lncRNA	exon	79226104	79226988	.	-	.	gene_id "lnc-bbug03491"	transcript_id "lnc-bbut00008010"	exon_number "1"

11	lncRNA	exon	79253703	79254381	.	-	.	gene_id "lnc-bbug03493"	transcript_id "lnc-bbut00008014"	exon_number "1"
11	lncRNA	exon	79457380	79458344	.	-	.	gene_id "lnc-bbug03494"	transcript_id "lnc-bbut00008016"	exon_number "1"
11	lncRNA	exon	79458554	79458711	.	-	.	gene_id "lnc-bbug03494"	transcript_id "lnc-bbut00008016"	exon_number "2"
11	lncRNA	exon	79457380	79465400	.	-	.	gene_id "lnc-bbug03494"	transcript_id "lnc-bbut00008017"	exon_number "1"
11	lncRNA	exon	79488220	79490120	.	-	.	gene_id "lnc-bbug03496"	transcript_id "lnc-bbut00008019"	exon_number "1"
11	lncRNA	exon	79497180	79497918	.	-	.	gene_id "lnc-bbug03497"	transcript_id "lnc-bbut00008021"	exon_number "1"
11	lncRNA	exon	79498137	79498581	.	-	.	gene_id "lnc-bbug03497"	transcript_id "lnc-bbut00008021"	exon_number "2"
11	lncRNA	exon	79540555	79541234	.	-	.	gene_id "lnc-bbug03502"	transcript_id "lnc-bbut00008026"	exon_number "1"
11	lncRNA	exon	79551098	79552257	.	-	.	gene_id "lnc-bbug03504"	transcript_id "lnc-bbut00008030"	exon_number "1"
11	lncRNA	exon	79552422	79553100	.	-	.	gene_id "lnc-bbug03504"	transcript_id "lnc-bbut00008030"	exon_number "2"
11	lncRNA	exon	79626515	79627731	.	-	.	gene_id "lnc-bbug03508"	transcript_id "lnc-bbut00008037"	exon_number "1"
11	lncRNA	exon	79632341	79633389	.	-	.	gene_id "lnc-bbug03509"	transcript_id "lnc-bbut00008039"	exon_number "1"
11	lncRNA	exon	79655409	79657183	.	-	.	gene_id "lnc-bbug03511"	transcript_id "lnc-bbut00008042"	exon_number "1"
11	lncRNA	exon	80116294	80116574	.	-	.	gene_id "lnc-bbug03518"	transcript_id "lnc-bbut00008053"	exon_number "1"
11	lncRNA	exon	80116945	80117242	.	-	.	gene_id "lnc-bbug03518"	transcript_id "lnc-bbut00008053"	exon_number "2"
11	lncRNA	exon	80266211	80266586	.	-	.	gene_id "lnc-bbug03520"	transcript_id "lnc-bbut00008055"	exon_number "1"
11	lncRNA	exon	80267950	80268043	.	-	.	gene_id "lnc-bbug03520"	transcript_id "lnc-bbut00008055"	exon_number "2"
11	lncRNA	exon	80268747	80268982	.	-	.	gene_id "lnc-bbug03520"	transcript_id "lnc-bbut00008055"	exon_number "3"
11	lncRNA	exon	80301277	80301527	.	-	.	gene_id "lnc-bbug03521"	transcript_id "lnc-bbut00008057"	exon_number "1"
11	lncRNA	exon	80301751	80301817	.	-	.	gene_id "lnc-bbug03521"	transcript_id "lnc-bbut00008057"	exon_number "2"
11	lncRNA	exon	80302249	80302470	.	-	.	gene_id "lnc-bbug03521"	transcript_id "lnc-bbut00008057"	exon_number "3"
11	lncRNA	exon	80303423	80303532	.	-	.	gene_id "lnc-bbug03521"	transcript_id "lnc-bbut00008057"	exon_number "4"
11	lncRNA	exon	80304010	80305457	.	-	.	gene_id "lnc-bbug03521"	transcript_id "lnc-bbut00008057"	exon_number "5"
11	lncRNA	exon	80305572	80308157	.	-	.	gene_id "lnc-bbug03521"	transcript_id "lnc-bbut00008057"	exon_number "6"
11	lncRNA	exon	80301277	80301527	.	-	.	gene_id "lnc-bbug03521"	transcript_id "lnc-bbut00008056"	exon_number "1"
11	lncRNA	exon	80301751	80301817	.	-	.	gene_id "lnc-bbug03521"	transcript_id "lnc-bbut00008056"	exon_number "2"
11	lncRNA	exon	80302249	80302470	.	-	.	gene_id "lnc-bbug03521"	transcript_id "lnc-bbut00008056"	exon_number "3"
11	lncRNA	exon	80303423	80305457	.	-	.	gene_id "lnc-bbug03521"	transcript_id "lnc-bbut00008056"	exon_number "4"
11	lncRNA	exon	80305572	80308157	.	-	.	gene_id "lnc-bbug03521"	transcript_id "lnc-bbut00008056"	exon_number "5"
11	lncRNA	exon	80327311	80331806	.	-	.	gene_id "lnc-bbug03522"	transcript_id "lnc-bbut00008060"	exon_number "1"
11	lncRNA	exon	80333217	80333983	.	-	.	gene_id "lnc-bbug03522"	transcript_id "lnc-bbut00008060"	exon_number "2"
11	lncRNA	exon	80343798	80344054	.	-	.	gene_id "lnc-bbug03522"	transcript_id "lnc-bbut00008060"	exon_number "3"
11	lncRNA	exon	80353681	80354678	.	-	.	gene_id "lnc-bbug03522"	transcript_id "lnc-bbut00008060"	exon_number "4"
11	lncRNA	exon	80327311	80331806	.	-	.	gene_id "lnc-bbug03522"	transcript_id "lnc-bbut00008059"	exon_number "1"
11	lncRNA	exon	80333217	80333983	.	-	.	gene_id "lnc-bbug03522"	transcript_id "lnc-bbut00008059"	exon_number "2"
11	lncRNA	exon	80343798	80344054	.	-	.	gene_id "lnc-bbug03522"	transcript_id "lnc-bbut00008059"	exon_number "3"
11	lncRNA	exon	80352753	80354678	.	-	.	gene_id "lnc-bbug03522"	transcript_id "lnc-bbut00008059"	exon_number "4"
11	lncRNA	exon	80327311	80331806	.	-	.	gene_id "lnc-bbug03522"	transcript_id "lnc-bbut00008058"	exon_number "1"
11	lncRNA	exon	80333217	80333983	.	-	.	gene_id "lnc-bbug03522"	transcript_id "lnc-bbut00008058"	exon_number "2"
11	lncRNA	exon	80352753	80354678	.	-	.	gene_id "lnc-bbug03522"	transcript_id "lnc-bbut00008058"	exon_number "3"
11	lncRNA	exon	80327311	80331806	.	-	.	gene_id "lnc-bbug03522"	transcript_id "lnc-bbut00008061"	exon_number "1"
11	lncRNA	exon	80333217	80333983	.	-	.	gene_id "lnc-bbug03522"	transcript_id "lnc-bbut00008061"	exon_number "2"
11	lncRNA	exon	80343798	80344054	.	-	.	gene_id "lnc-bbug03522"	transcript_id "lnc-bbut00008061"	exon_number "3"
11	lncRNA	exon	80354685	80355310	.	-	.	gene_id "lnc-bbug03522"	transcript_id "lnc-bbut00008061"	exon_number "4"
11	lncRNA	exon	80347554	80348007	.	-	.	gene_id "lnc-bbug03523"	transcript_id "lnc-bbut00008062"	exon_number "1"
11	lncRNA	exon	80348223	80348969	.	-	.	gene_id "lnc-bbug03523"	transcript_id "lnc-bbut00008062"	exon_number "2"
11	lncRNA	exon	80468825	80469141	.	-	.	gene_id "lnc-bbug03524"	transcript_id "lnc-bbut00008064"	exon_number "1"
11	lncRNA	exon	80469363	80469479	.	-	.	gene_id "lnc-bbug03524"	transcript_id "lnc-bbut00008064"	exon_number "2"
11	lncRNA	exon	80468825	80469479	.	-	.	gene_id "lnc-bbug03524"	transcript_id "lnc-bbut00008063"	exon_number "1"
11	lncRNA	exon	81533788	81534355	.	-	.	gene_id "lnc-bbug03547"	transcript_id "lnc-bbut00008130"	exon_number "1"
11	lncRNA	exon	81543256	81544310	.	-	.	gene_id "lnc-bbug03547"	transcript_id "lnc-bbut00008130"	exon_number "2"
11	lncRNA	exon	81555230	81556278	.	-	.	gene_id "lnc-bbug03548"	transcript_id "lnc-bbut00008131"	exon_number "1"

11	lncRNA	exon	81657915	81658518	.	-	.	gene_id "lnc-bbug03549"	transcript_id "lnc-bbut00008132"	exon_number "1"
11	lncRNA	exon	81723560	81726074	.	-	.	gene_id "lnc-bbug03550"	transcript_id "lnc-bbut00008133"	exon_number "1"
11	lncRNA	exon	81730468	81730545	.	-	.	gene_id "lnc-bbug03550"	transcript_id "lnc-bbut00008133"	exon_number "2"
11	lncRNA	exon	81886530	81886899	.	-	.	gene_id "lnc-bbug03558"	transcript_id "lnc-bbut00008145"	exon_number "1"
11	lncRNA	exon	81887465	81887547	.	-	.	gene_id "lnc-bbug03558"	transcript_id "lnc-bbut00008145"	exon_number "2"
11	lncRNA	exon	81887716	81887763	.	-	.	gene_id "lnc-bbug03558"	transcript_id "lnc-bbut00008145"	exon_number "3"
11	lncRNA	exon	81895173	81895326	.	-	.	gene_id "lnc-bbug03558"	transcript_id "lnc-bbut00008145"	exon_number "4"
11	lncRNA	exon	82019755	82021164	.	-	.	gene_id "lnc-bbug03566"	transcript_id "lnc-bbut00008168"	exon_number "1"
11	lncRNA	exon	82021247	82021858	.	-	.	gene_id "lnc-bbug03566"	transcript_id "lnc-bbut00008168"	exon_number "2"
11	lncRNA	exon	82054441	82054723	.	-	.	gene_id "lnc-bbug03567"	transcript_id "lnc-bbut00008169"	exon_number "1"
11	lncRNA	exon	82055406	82056329	.	-	.	gene_id "lnc-bbug03567"	transcript_id "lnc-bbut00008169"	exon_number "2"
11	lncRNA	exon	82056473	82056790	.	-	.	gene_id "lnc-bbug03567"	transcript_id "lnc-bbut00008169"	exon_number "3"
11	lncRNA	exon	85096196	85096736	.	-	.	gene_id "lnc-bbug03582"	transcript_id "lnc-bbut00008217"	exon_number "1"
11	lncRNA	exon	85296232	85297915	.	-	.	gene_id "lnc-bbug03583"	transcript_id "lnc-bbut00008218"	exon_number "1"
11	lncRNA	exon	85298631	85298739	.	-	.	gene_id "lnc-bbug03583"	transcript_id "lnc-bbut00008218"	exon_number "2"
11	lncRNA	exon	85302439	85302642	.	-	.	gene_id "lnc-bbug03583"	transcript_id "lnc-bbut00008218"	exon_number "3"
11	lncRNA	exon	85312477	85313241	.	-	.	gene_id "lnc-bbug03584"	transcript_id "lnc-bbut00008220"	exon_number "1"
11	lncRNA	exon	85315075	85315249	.	-	.	gene_id "lnc-bbug03584"	transcript_id "lnc-bbut00008220"	exon_number "2"
11	lncRNA	exon	85315436	85316298	.	-	.	gene_id "lnc-bbug03584"	transcript_id "lnc-bbut00008220"	exon_number "3"
11	lncRNA	exon	85312477	85313241	.	-	.	gene_id "lnc-bbug03584"	transcript_id "lnc-bbut00008219"	exon_number "1"
11	lncRNA	exon	85315075	85315240	.	-	.	gene_id "lnc-bbug03584"	transcript_id "lnc-bbut00008219"	exon_number "2"
11	lncRNA	exon	85315436	85316298	.	-	.	gene_id "lnc-bbug03584"	transcript_id "lnc-bbut00008219"	exon_number "3"
11	lncRNA	exon	85316449	85317028	.	-	.	gene_id "lnc-bbug03585"	transcript_id "lnc-bbut00008221"	exon_number "1"
11	lncRNA	exon	85317130	85317542	.	-	.	gene_id "lnc-bbug03585"	transcript_id "lnc-bbut00008221"	exon_number "2"
11	lncRNA	exon	85318543	85318653	.	-	.	gene_id "lnc-bbug03585"	transcript_id "lnc-bbut00008221"	exon_number "3"
11	lncRNA	exon	85318965	85319213	.	-	.	gene_id "lnc-bbug03585"	transcript_id "lnc-bbut00008221"	exon_number "4"
11	lncRNA	exon	85316449	85317028	.	-	.	gene_id "lnc-bbug03585"	transcript_id "lnc-bbut00008222"	exon_number "1"
11	lncRNA	exon	85317130	85317542	.	-	.	gene_id "lnc-bbug03585"	transcript_id "lnc-bbut00008222"	exon_number "2"
11	lncRNA	exon	85318543	85319396	.	-	.	gene_id "lnc-bbug03585"	transcript_id "lnc-bbut00008222"	exon_number "3"
11	lncRNA	exon	85340587	85349890	.	-	.	gene_id "lnc-bbug03586"	transcript_id "lnc-bbut00008224"	exon_number "1"
11	lncRNA	exon	85350502	85357536	.	-	.	gene_id "lnc-bbug03586"	transcript_id "lnc-bbut00008224"	exon_number "2"
11	lncRNA	exon	85340587	85349870	.	-	.	gene_id "lnc-bbug03586"	transcript_id "lnc-bbut00008223"	exon_number "1"
11	lncRNA	exon	85350502	85357536	.	-	.	gene_id "lnc-bbug03586"	transcript_id "lnc-bbut00008223"	exon_number "2"
11	lncRNA	exon	85574916	85574984	.	-	.	gene_id "lnc-bbug03587"	transcript_id "lnc-bbut00008225"	exon_number "1"
11	lncRNA	exon	85576288	85576780	.	-	.	gene_id "lnc-bbug03587"	transcript_id "lnc-bbut00008225"	exon_number "2"
11	lncRNA	exon	85624323	85624382	.	-	.	gene_id "lnc-bbug03587"	transcript_id "lnc-bbut00008225"	exon_number "3"
11	lncRNA	exon	85624441	85624525	.	-	.	gene_id "lnc-bbug03587"	transcript_id "lnc-bbut00008225"	exon_number "4"
11	lncRNA	exon	85625475	85625498	.	-	.	gene_id "lnc-bbug03587"	transcript_id "lnc-bbut00008225"	exon_number "5"
11	lncRNA	exon	86051842	86055010	.	-	.	gene_id "lnc-bbug03588"	transcript_id "lnc-bbut00008226"	exon_number "1"
11	lncRNA	exon	86056064	86057640	.	-	.	gene_id "lnc-bbug03588"	transcript_id "lnc-bbut00008226"	exon_number "2"
11	lncRNA	exon	86051842	86055661	.	-	.	gene_id "lnc-bbug03588"	transcript_id "lnc-bbut00008227"	exon_number "1"
11	lncRNA	exon	86056064	86057649	.	-	.	gene_id "lnc-bbug03588"	transcript_id "lnc-bbut00008227"	exon_number "2"
11	lncRNA	exon	86077946	86084329	.	-	.	gene_id "lnc-bbug03589"	transcript_id "lnc-bbut00008228"	exon_number "1"
11	lncRNA	exon	86087795	86088334	.	-	.	gene_id "lnc-bbug03589"	transcript_id "lnc-bbut00008228"	exon_number "2"
11	lncRNA	exon	86127271	86127386	.	-	.	gene_id "lnc-bbug03589"	transcript_id "lnc-bbut00008228"	exon_number "3"
11	lncRNA	exon	86129649	86129744	.	-	.	gene_id "lnc-bbug03589"	transcript_id "lnc-bbut00008228"	exon_number "4"
11	lncRNA	exon	86130292	86130486	.	-	.	gene_id "lnc-bbug03589"	transcript_id "lnc-bbut00008228"	exon_number "5"
11	lncRNA	exon	86133603	86133715	.	-	.	gene_id "lnc-bbug03589"	transcript_id "lnc-bbut00008228"	exon_number "6"
11	lncRNA	exon	86216311	86216506	.	-	.	gene_id "lnc-bbug03589"	transcript_id "lnc-bbut00008228"	exon_number "7"
11	lncRNA	exon	86216912	86219294	.	-	.	gene_id "lnc-bbug03589"	transcript_id "lnc-bbut00008228"	exon_number "8"
11	lncRNA	exon	86077946	86079466	.	-	.	gene_id "lnc-bbug03589"	transcript_id "lnc-bbut00008232"	exon_number "1"
11	lncRNA	exon	86081203	86084329	.	-	.	gene_id "lnc-bbug03589"	transcript_id "lnc-bbut00008232"	exon_number "2"

11	lncRNA	exon	86087795	86088334	.	-	.	gene_id "lnc-bbug03589"	transcript_id "lnc-bbut00008232"	exon_number "3"
11	lncRNA	exon	86127271	86127386	.	-	.	gene_id "lnc-bbug03589"	transcript_id "lnc-bbut00008232"	exon_number "4"
11	lncRNA	exon	86129649	86129744	.	-	.	gene_id "lnc-bbug03589"	transcript_id "lnc-bbut00008232"	exon_number "5"
11	lncRNA	exon	86130292	86130486	.	-	.	gene_id "lnc-bbug03589"	transcript_id "lnc-bbut00008232"	exon_number "6"
11	lncRNA	exon	86133603	86133715	.	-	.	gene_id "lnc-bbug03589"	transcript_id "lnc-bbut00008232"	exon_number "7"
11	lncRNA	exon	86216311	86216506	.	-	.	gene_id "lnc-bbug03589"	transcript_id "lnc-bbut00008232"	exon_number "8"
11	lncRNA	exon	86315836	86316035	.	-	.	gene_id "lnc-bbug03589"	transcript_id "lnc-bbut00008232"	exon_number "9"
11	lncRNA	exon	86317447	86317621	.	-	.	gene_id "lnc-bbug03589"	transcript_id "lnc-bbut00008232"	exon_number "10"
11	lncRNA	exon	86077946	86081283	.	-	.	gene_id "lnc-bbug03589"	transcript_id "lnc-bbut00008234"	exon_number "1"
11	lncRNA	exon	86081585	86084329	.	-	.	gene_id "lnc-bbug03589"	transcript_id "lnc-bbut00008234"	exon_number "2"
11	lncRNA	exon	86087795	86088334	.	-	.	gene_id "lnc-bbug03589"	transcript_id "lnc-bbut00008234"	exon_number "3"
11	lncRNA	exon	86127271	86127386	.	-	.	gene_id "lnc-bbug03589"	transcript_id "lnc-bbut00008234"	exon_number "4"
11	lncRNA	exon	86129649	86129744	.	-	.	gene_id "lnc-bbug03589"	transcript_id "lnc-bbut00008234"	exon_number "5"
11	lncRNA	exon	86130292	86130486	.	-	.	gene_id "lnc-bbug03589"	transcript_id "lnc-bbut00008234"	exon_number "6"
11	lncRNA	exon	86133603	86133715	.	-	.	gene_id "lnc-bbug03589"	transcript_id "lnc-bbut00008234"	exon_number "7"
11	lncRNA	exon	86216311	86216506	.	-	.	gene_id "lnc-bbug03589"	transcript_id "lnc-bbut00008234"	exon_number "8"
11	lncRNA	exon	86315836	86316035	.	-	.	gene_id "lnc-bbug03589"	transcript_id "lnc-bbut00008234"	exon_number "9"
11	lncRNA	exon	86317447	86317621	.	-	.	gene_id "lnc-bbug03589"	transcript_id "lnc-bbut00008234"	exon_number "10"
11	lncRNA	exon	86077946	86081688	.	-	.	gene_id "lnc-bbug03589"	transcript_id "lnc-bbut00008235"	exon_number "1"
11	lncRNA	exon	86084130	86084329	.	-	.	gene_id "lnc-bbug03589"	transcript_id "lnc-bbut00008235"	exon_number "2"
11	lncRNA	exon	86087795	86088334	.	-	.	gene_id "lnc-bbug03589"	transcript_id "lnc-bbut00008235"	exon_number "3"
11	lncRNA	exon	86127271	86127386	.	-	.	gene_id "lnc-bbug03589"	transcript_id "lnc-bbut00008235"	exon_number "4"
11	lncRNA	exon	86129649	86129744	.	-	.	gene_id "lnc-bbug03589"	transcript_id "lnc-bbut00008235"	exon_number "5"
11	lncRNA	exon	86130292	86130486	.	-	.	gene_id "lnc-bbug03589"	transcript_id "lnc-bbut00008235"	exon_number "6"
11	lncRNA	exon	86133603	86133715	.	-	.	gene_id "lnc-bbug03589"	transcript_id "lnc-bbut00008235"	exon_number "7"
11	lncRNA	exon	86216311	86216506	.	-	.	gene_id "lnc-bbug03589"	transcript_id "lnc-bbut00008235"	exon_number "8"
11	lncRNA	exon	86315836	86316035	.	-	.	gene_id "lnc-bbug03589"	transcript_id "lnc-bbut00008235"	exon_number "9"
11	lncRNA	exon	86317447	86317621	.	-	.	gene_id "lnc-bbug03589"	transcript_id "lnc-bbut00008235"	exon_number "10"
11	lncRNA	exon	86077946	86081283	.	-	.	gene_id "lnc-bbug03589"	transcript_id "lnc-bbut00008233"	exon_number "1"
11	lncRNA	exon	86081509	86084329	.	-	.	gene_id "lnc-bbug03589"	transcript_id "lnc-bbut00008233"	exon_number "2"
11	lncRNA	exon	86087795	86088334	.	-	.	gene_id "lnc-bbug03589"	transcript_id "lnc-bbut00008233"	exon_number "3"
11	lncRNA	exon	86127271	86127386	.	-	.	gene_id "lnc-bbug03589"	transcript_id "lnc-bbut00008233"	exon_number "4"
11	lncRNA	exon	86129649	86129744	.	-	.	gene_id "lnc-bbug03589"	transcript_id "lnc-bbut00008233"	exon_number "5"
11	lncRNA	exon	86130292	86130486	.	-	.	gene_id "lnc-bbug03589"	transcript_id "lnc-bbut00008233"	exon_number "6"
11	lncRNA	exon	86133603	86133715	.	-	.	gene_id "lnc-bbug03589"	transcript_id "lnc-bbut00008233"	exon_number "7"
11	lncRNA	exon	86216311	86216506	.	-	.	gene_id "lnc-bbug03589"	transcript_id "lnc-bbut00008233"	exon_number "8"
11	lncRNA	exon	86315836	86316035	.	-	.	gene_id "lnc-bbug03589"	transcript_id "lnc-bbut00008233"	exon_number "9"
11	lncRNA	exon	86317447	86317621	.	-	.	gene_id "lnc-bbug03589"	transcript_id "lnc-bbut00008233"	exon_number "10"
11	lncRNA	exon	86077946	86084329	.	-	.	gene_id "lnc-bbug03589"	transcript_id "lnc-bbut00008231"	exon_number "1"
11	lncRNA	exon	86087795	86088334	.	-	.	gene_id "lnc-bbug03589"	transcript_id "lnc-bbut00008231"	exon_number "2"
11	lncRNA	exon	86127271	86127386	.	-	.	gene_id "lnc-bbug03589"	transcript_id "lnc-bbut00008231"	exon_number "3"
11	lncRNA	exon	86129649	86129744	.	-	.	gene_id "lnc-bbug03589"	transcript_id "lnc-bbut00008231"	exon_number "4"
11	lncRNA	exon	86130292	86130486	.	-	.	gene_id "lnc-bbug03589"	transcript_id "lnc-bbut00008231"	exon_number "5"
11	lncRNA	exon	86133603	86133715	.	-	.	gene_id "lnc-bbug03589"	transcript_id "lnc-bbut00008231"	exon_number "6"
11	lncRNA	exon	86216311	86216506	.	-	.	gene_id "lnc-bbug03589"	transcript_id "lnc-bbut00008231"	exon_number "7"
11	lncRNA	exon	86315836	86316035	.	-	.	gene_id "lnc-bbug03589"	transcript_id "lnc-bbut00008231"	exon_number "8"
11	lncRNA	exon	86317447	86317621	.	-	.	gene_id "lnc-bbug03589"	transcript_id "lnc-bbut00008231"	exon_number "9"
11	lncRNA	exon	86077946	86084329	.	-	.	gene_id "lnc-bbug03589"	transcript_id "lnc-bbut00008230"	exon_number "1"
11	lncRNA	exon	86087795	86088334	.	-	.	gene_id "lnc-bbug03589"	transcript_id "lnc-bbut00008230"	exon_number "2"
11	lncRNA	exon	86127271	86127386	.	-	.	gene_id "lnc-bbug03589"	transcript_id "lnc-bbut00008230"	exon_number "3"
11	lncRNA	exon	86129649	86129744	.	-	.	gene_id "lnc-bbug03589"	transcript_id "lnc-bbut00008230"	exon_number "4"
11	lncRNA	exon	86130292	86130439	.	-	.	gene_id "lnc-bbug03589"	transcript_id "lnc-bbut00008230"	exon_number "5"

11	lncRNA	exon	86133603	86133715	.	-	.	gene_id "lnc-bbug03589"	transcript_id "lnc-bbut00008230"	exon_number "6"
11	lncRNA	exon	86216311	86216506	.	-	.	gene_id "lnc-bbug03589"	transcript_id "lnc-bbut00008230"	exon_number "7"
11	lncRNA	exon	86315836	86316035	.	-	.	gene_id "lnc-bbug03589"	transcript_id "lnc-bbut00008230"	exon_number "8"
11	lncRNA	exon	86317447	86317621	.	-	.	gene_id "lnc-bbug03589"	transcript_id "lnc-bbut00008230"	exon_number "9"
11	lncRNA	exon	86077946	86084329	.	-	.	gene_id "lnc-bbug03589"	transcript_id "lnc-bbut00008229"	exon_number "1"
11	lncRNA	exon	86087795	86088334	.	-	.	gene_id "lnc-bbug03589"	transcript_id "lnc-bbut00008229"	exon_number "2"
11	lncRNA	exon	86127271	86127386	.	-	.	gene_id "lnc-bbug03589"	transcript_id "lnc-bbut00008229"	exon_number "3"
11	lncRNA	exon	86129649	86129744	.	-	.	gene_id "lnc-bbug03589"	transcript_id "lnc-bbut00008229"	exon_number "4"
11	lncRNA	exon	86130292	86130486	.	-	.	gene_id "lnc-bbug03589"	transcript_id "lnc-bbut00008229"	exon_number "5"
11	lncRNA	exon	86133603	86133715	.	-	.	gene_id "lnc-bbug03589"	transcript_id "lnc-bbut00008229"	exon_number "6"
11	lncRNA	exon	86315836	86316035	.	-	.	gene_id "lnc-bbug03589"	transcript_id "lnc-bbut00008229"	exon_number "7"
11	lncRNA	exon	86317447	86317621	.	-	.	gene_id "lnc-bbug03589"	transcript_id "lnc-bbut00008229"	exon_number "8"
11	lncRNA	exon	86930421	86930945	.	-	.	gene_id "lnc-bbug03596"	transcript_id "lnc-bbut00008259"	exon_number "1"
11	lncRNA	exon	86934711	86934741	.	-	.	gene_id "lnc-bbug03596"	transcript_id "lnc-bbut00008259"	exon_number "2"
11	lncRNA	exon	88305394	88305619	.	-	.	gene_id "lnc-bbug03603"	transcript_id "lnc-bbut00008272"	exon_number "1"
11	lncRNA	exon	88306320	88306336	.	-	.	gene_id "lnc-bbug03603"	transcript_id "lnc-bbut00008272"	exon_number "2"
11	lncRNA	exon	88646150	88647061	.	-	.	gene_id "lnc-bbug03606"	transcript_id "lnc-bbut00008277"	exon_number "1"
11	lncRNA	exon	88647100	88652915	.	-	.	gene_id "lnc-bbug03606"	transcript_id "lnc-bbut00008277"	exon_number "2"
11	lncRNA	exon	89172022	89175003	.	-	.	gene_id "lnc-bbug03608"	transcript_id "lnc-bbut00008281"	exon_number "1"
11	lncRNA	exon	89175233	89175287	.	-	.	gene_id "lnc-bbug03608"	transcript_id "lnc-bbut00008281"	exon_number "2"
11	lncRNA	exon	89184272	89184399	.	-	.	gene_id "lnc-bbug03608"	transcript_id "lnc-bbut00008281"	exon_number "3"
11	lncRNA	exon	89196157	89196267	.	-	.	gene_id "lnc-bbug03608"	transcript_id "lnc-bbut00008281"	exon_number "4"
11	lncRNA	exon	89208742	89208867	.	-	.	gene_id "lnc-bbug03608"	transcript_id "lnc-bbut00008281"	exon_number "5"
11	lncRNA	exon	89211433	89211508	.	-	.	gene_id "lnc-bbug03608"	transcript_id "lnc-bbut00008281"	exon_number "6"
11	lncRNA	exon	89224909	89225053	.	-	.	gene_id "lnc-bbug03608"	transcript_id "lnc-bbut00008281"	exon_number "7"
11	lncRNA	exon	89863810	89866538	.	-	.	gene_id "lnc-bbug03612"	transcript_id "lnc-bbut00008289"	exon_number "1"
11	lncRNA	exon	89889675	89889765	.	-	.	gene_id "lnc-bbug03612"	transcript_id "lnc-bbut00008289"	exon_number "2"
11	lncRNA	exon	89926755	89928289	.	-	.	gene_id "lnc-bbug03614"	transcript_id "lnc-bbut00008291"	exon_number "1"
11	lncRNA	exon	89929888	89929993	.	-	.	gene_id "lnc-bbug03614"	transcript_id "lnc-bbut00008291"	exon_number "2"
11	lncRNA	exon	89952131	89952963	.	-	.	gene_id "lnc-bbug03614"	transcript_id "lnc-bbut00008291"	exon_number "3"
11	lncRNA	exon	89929169	89929219	.	-	.	gene_id "lnc-bbug03614"	transcript_id "lnc-bbut00008292"	exon_number "1"
11	lncRNA	exon	89929888	89929993	.	-	.	gene_id "lnc-bbug03614"	transcript_id "lnc-bbut00008292"	exon_number "2"
11	lncRNA	exon	89952131	89952962	.	-	.	gene_id "lnc-bbug03614"	transcript_id "lnc-bbut00008292"	exon_number "3"
11	lncRNA	exon	90579550	90580232	.	-	.	gene_id "lnc-bbug03619"	transcript_id "lnc-bbut00008306"	exon_number "1"
11	lncRNA	exon	90580852	90581859	.	-	.	gene_id "lnc-bbug03619"	transcript_id "lnc-bbut00008306"	exon_number "2"
11	lncRNA	exon	90581972	90582070	.	-	.	gene_id "lnc-bbug03619"	transcript_id "lnc-bbut00008306"	exon_number "3"
11	lncRNA	exon	94253928	94254495	.	-	.	gene_id "lnc-bbug03634"	transcript_id "lnc-bbut00008346"	exon_number "1"
11	lncRNA	exon	94256640	94256773	.	-	.	gene_id "lnc-bbug03634"	transcript_id "lnc-bbut00008346"	exon_number "2"
11	lncRNA	exon	94258312	94259767	.	-	.	gene_id "lnc-bbug03634"	transcript_id "lnc-bbut00008346"	exon_number "3"
11	lncRNA	exon	94253928	94254495	.	-	.	gene_id "lnc-bbug03634"	transcript_id "lnc-bbut00008345"	exon_number "1"
11	lncRNA	exon	94256640	94256751	.	-	.	gene_id "lnc-bbug03634"	transcript_id "lnc-bbut00008345"	exon_number "2"
11	lncRNA	exon	94258312	94259767	.	-	.	gene_id "lnc-bbug03634"	transcript_id "lnc-bbut00008345"	exon_number "3"
11	lncRNA	exon	94253928	94254458	.	-	.	gene_id "lnc-bbug03634"	transcript_id "lnc-bbut00008344"	exon_number "1"
11	lncRNA	exon	94256640	94256773	.	-	.	gene_id "lnc-bbug03634"	transcript_id "lnc-bbut00008344"	exon_number "2"
11	lncRNA	exon	94258312	94259767	.	-	.	gene_id "lnc-bbug03634"	transcript_id "lnc-bbut00008344"	exon_number "3"
11	lncRNA	exon	94337247	94337528	.	-	.	gene_id "lnc-bbug03635"	transcript_id "lnc-bbut00008347"	exon_number "1"
11	lncRNA	exon	94337639	94337847	.	-	.	gene_id "lnc-bbug03635"	transcript_id "lnc-bbut00008347"	exon_number "2"
11	lncRNA	exon	94341268	94341361	.	-	.	gene_id "lnc-bbug03635"	transcript_id "lnc-bbut00008347"	exon_number "3"
11	lncRNA	exon	94383952	94384052	.	-	.	gene_id "lnc-bbug03635"	transcript_id "lnc-bbut00008347"	exon_number "4"
11	lncRNA	exon	95031875	95032337	.	-	.	gene_id "lnc-bbug03642"	transcript_id "lnc-bbut00008356"	exon_number "1"
11	lncRNA	exon	95032518	95033558	.	-	.	gene_id "lnc-bbug03642"	transcript_id "lnc-bbut00008356"	exon_number "2"
11	lncRNA	exon	95453662	95455089	.	-	.	gene_id "lnc-bbug03644"	transcript_id "lnc-bbut00008358"	exon_number "1"

11	lncRNA	exon	95455694	95455812	.	-	.	gene_id "lnc-bbug03644"	transcript_id "lnc-bbut00008358"	exon_number "2"
11	lncRNA	exon	95463855	95465158	.	-	.	gene_id "lnc-bbug03644"	transcript_id "lnc-bbut00008358"	exon_number "3"
11	lncRNA	exon	95453662	95455089	.	-	.	gene_id "lnc-bbug03644"	transcript_id "lnc-bbut00008359"	exon_number "1"
11	lncRNA	exon	95455694	95455812	.	-	.	gene_id "lnc-bbug03644"	transcript_id "lnc-bbut00008359"	exon_number "2"
11	lncRNA	exon	95524972	95525098	.	-	.	gene_id "lnc-bbug03644"	transcript_id "lnc-bbut00008359"	exon_number "3"
11	lncRNA	exon	96357800	96358848	.	-	.	gene_id "lnc-bbug03650"	transcript_id "lnc-bbut00008371"	exon_number "1"
11	lncRNA	exon	98122593	98123749	.	-	.	gene_id "lnc-bbug03658"	transcript_id "lnc-bbut00008395"	exon_number "1"
11	lncRNA	exon	98156512	98160789	.	-	.	gene_id "lnc-bbug03659"	transcript_id "lnc-bbut00008396"	exon_number "1"
11	lncRNA	exon	98161214	98162597	.	-	.	gene_id "lnc-bbug03660"	transcript_id "lnc-bbut00008397"	exon_number "1"
11	lncRNA	exon	99583481	99583999	.	-	.	gene_id "lnc-bbug03665"	transcript_id "lnc-bbut00008404"	exon_number "1"
11	lncRNA	exon	99585253	99586297	.	-	.	gene_id "lnc-bbug03665"	transcript_id "lnc-bbut00008404"	exon_number "2"
11	lncRNA	exon	100263382	100266712	.	-	.	gene_id "lnc-bbug03667"	transcript_id "lnc-bbut00008408"	exon_number "1"
11	lncRNA	exon	100276691	100277100	.	-	.	gene_id "lnc-bbug03667"	transcript_id "lnc-bbut00008408"	exon_number "2"
11	lncRNA	exon	100277553	100277599	.	-	.	gene_id "lnc-bbug03667"	transcript_id "lnc-bbut00008408"	exon_number "3"
11	lncRNA	exon	100327306	100329065	.	-	.	gene_id "lnc-bbug03668"	transcript_id "lnc-bbut00008409"	exon_number "1"
11	lncRNA	exon	100333993	100334070	.	-	.	gene_id "lnc-bbug03668"	transcript_id "lnc-bbut00008409"	exon_number "2"
11	lncRNA	exon	100341098	100341173	.	-	.	gene_id "lnc-bbug03668"	transcript_id "lnc-bbut00008409"	exon_number "3"
11	lncRNA	exon	100342451	100343597	.	-	.	gene_id "lnc-bbug03668"	transcript_id "lnc-bbut00008409"	exon_number "4"
11	lncRNA	exon	100327306	100329065	.	-	.	gene_id "lnc-bbug03668"	transcript_id "lnc-bbut00008410"	exon_number "1"
11	lncRNA	exon	100333993	100334070	.	-	.	gene_id "lnc-bbug03668"	transcript_id "lnc-bbut00008410"	exon_number "2"
11	lncRNA	exon	100358377	100358445	.	-	.	gene_id "lnc-bbug03668"	transcript_id "lnc-bbut00008410"	exon_number "3"
11	lncRNA	exon	100359736	100359777	.	-	.	gene_id "lnc-bbug03668"	transcript_id "lnc-bbut00008410"	exon_number "4"
11	lncRNA	exon	100360146	100360226	.	-	.	gene_id "lnc-bbug03668"	transcript_id "lnc-bbut00008410"	exon_number "5"
11	lncRNA	exon	100426127	100426639	.	-	.	gene_id "lnc-bbug03669"	transcript_id "lnc-bbut00008411"	exon_number "1"
11	lncRNA	exon	100645162	100645430	.	-	.	gene_id "lnc-bbug03669"	transcript_id "lnc-bbut00008411"	exon_number "2"
11	lncRNA	exon	100674277	100674326	.	-	.	gene_id "lnc-bbug03669"	transcript_id "lnc-bbut00008411"	exon_number "3"
11	lncRNA	exon	100541020	100543358	.	-	.	gene_id "lnc-bbug03669"	transcript_id "lnc-bbut00008412"	exon_number "1"
11	lncRNA	exon	100645162	100645430	.	-	.	gene_id "lnc-bbug03669"	transcript_id "lnc-bbut00008412"	exon_number "2"
11	lncRNA	exon	100674277	100674326	.	-	.	gene_id "lnc-bbug03669"	transcript_id "lnc-bbut00008412"	exon_number "3"
11	lncRNA	exon	100541020	100543358	.	-	.	gene_id "lnc-bbug03669"	transcript_id "lnc-bbut00008413"	exon_number "1"
11	lncRNA	exon	100645162	100645430	.	-	.	gene_id "lnc-bbug03669"	transcript_id "lnc-bbut00008413"	exon_number "2"
11	lncRNA	exon	100754274	100754508	.	-	.	gene_id "lnc-bbug03669"	transcript_id "lnc-bbut00008413"	exon_number "3"
11	lncRNA	exon	101173142	101174250	.	-	.	gene_id "lnc-bbug03671"	transcript_id "lnc-bbut00008422"	exon_number "1"
11	lncRNA	exon	101187368	101187447	.	-	.	gene_id "lnc-bbug03671"	transcript_id "lnc-bbut00008422"	exon_number "2"
11	lncRNA	exon	101187732	101187923	.	-	.	gene_id "lnc-bbug03671"	transcript_id "lnc-bbut00008422"	exon_number "3"
12	lncRNA	exon	779052	779267	.	+	.	gene_id "lnc-bbug03678"	transcript_id "lnc-bbut00008436"	exon_number "1"
12	lncRNA	exon	840825	841101	.	+	.	gene_id "lnc-bbug03678"	transcript_id "lnc-bbut00008436"	exon_number "2"
12	lncRNA	exon	888842	889693	.	+	.	gene_id "lnc-bbug03679"	transcript_id "lnc-bbut00008437"	exon_number "1"
12	lncRNA	exon	903678	903785	.	+	.	gene_id "lnc-bbug03679"	transcript_id "lnc-bbut00008437"	exon_number "2"
12	lncRNA	exon	928443	932706	.	+	.	gene_id "lnc-bbug03679"	transcript_id "lnc-bbut00008437"	exon_number "3"
12	lncRNA	exon	900661	900796	.	+	.	gene_id "lnc-bbug03679"	transcript_id "lnc-bbut00008439"	exon_number "1"
12	lncRNA	exon	901924	902171	.	+	.	gene_id "lnc-bbug03679"	transcript_id "lnc-bbut00008439"	exon_number "2"
12	lncRNA	exon	903678	903785	.	+	.	gene_id "lnc-bbug03679"	transcript_id "lnc-bbut00008439"	exon_number "3"
12	lncRNA	exon	928443	932706	.	+	.	gene_id "lnc-bbug03679"	transcript_id "lnc-bbut00008439"	exon_number "4"
12	lncRNA	exon	900661	900796	.	+	.	gene_id "lnc-bbug03679"	transcript_id "lnc-bbut00008438"	exon_number "1"
12	lncRNA	exon	903678	903785	.	+	.	gene_id "lnc-bbug03679"	transcript_id "lnc-bbut00008438"	exon_number "2"
12	lncRNA	exon	928443	932706	.	+	.	gene_id "lnc-bbug03679"	transcript_id "lnc-bbut00008438"	exon_number "3"
12	lncRNA	exon	900804	902171	.	+	.	gene_id "lnc-bbug03679"	transcript_id "lnc-bbut00008440"	exon_number "1"
12	lncRNA	exon	903678	903785	.	+	.	gene_id "lnc-bbug03679"	transcript_id "lnc-bbut00008440"	exon_number "2"
12	lncRNA	exon	928443	932706	.	+	.	gene_id "lnc-bbug03679"	transcript_id "lnc-bbut00008440"	exon_number "3"
12	lncRNA	exon	907901	909154	.	+	.	gene_id "lnc-bbug03680"	transcript_id "lnc-bbut00008441"	exon_number "1"
12	lncRNA	exon	912124	913923	.	+	.	gene_id "lnc-bbug03680"	transcript_id "lnc-bbut00008441"	exon_number "2"

12	lncRNA	exon	1962415	1962551	.	+	.	gene_id "lnc-bbug03689"	transcript_id "lnc-bbut00008457"	exon_number "1"
12	lncRNA	exon	1962802	1963086	.	+	.	gene_id "lnc-bbug03689"	transcript_id "lnc-bbut00008457"	exon_number "2"
12	lncRNA	exon	2292020	2292032	.	+	.	gene_id "lnc-bbug03693"	transcript_id "lnc-bbut00008466"	exon_number "1"
12	lncRNA	exon	2300877	2300880	.	+	.	gene_id "lnc-bbug03693"	transcript_id "lnc-bbut00008466"	exon_number "2"
12	lncRNA	exon	2308926	2309385	.	+	.	gene_id "lnc-bbug03693"	transcript_id "lnc-bbut00008466"	exon_number "3"
12	lncRNA	exon	2612972	2614080	.	+	.	gene_id "lnc-bbug03699"	transcript_id "lnc-bbut00008478"	exon_number "1"
12	lncRNA	exon	2615534	2616004	.	+	.	gene_id "lnc-bbug03699"	transcript_id "lnc-bbut00008478"	exon_number "2"
12	lncRNA	exon	4178824	4178877	.	+	.	gene_id "lnc-bbug03708"	transcript_id "lnc-bbut00008501"	exon_number "1"
12	lncRNA	exon	4179708	4180322	.	+	.	gene_id "lnc-bbug03708"	transcript_id "lnc-bbut00008501"	exon_number "2"
12	lncRNA	exon	6101421	6101745	.	+	.	gene_id "lnc-bbug03717"	transcript_id "lnc-bbut00008513"	exon_number "1"
12	lncRNA	exon	6141620	6141788	.	+	.	gene_id "lnc-bbug03717"	transcript_id "lnc-bbut00008513"	exon_number "2"
12	lncRNA	exon	6333407	6334594	.	+	.	gene_id "lnc-bbug03718"	transcript_id "lnc-bbut00008514"	exon_number "1"
12	lncRNA	exon	6572101	6575064	.	+	.	gene_id "lnc-bbug03720"	transcript_id "lnc-bbut00008541"	exon_number "1"
12	lncRNA	exon	6575956	6576055	.	+	.	gene_id "lnc-bbug03720"	transcript_id "lnc-bbut00008541"	exon_number "2"
12	lncRNA	exon	6578667	6579431	.	+	.	gene_id "lnc-bbug03720"	transcript_id "lnc-bbut00008541"	exon_number "3"
12	lncRNA	exon	6580566	6581709	.	+	.	gene_id "lnc-bbug03720"	transcript_id "lnc-bbut00008541"	exon_number "4"
12	lncRNA	exon	6572101	6575064	.	+	.	gene_id "lnc-bbug03720"	transcript_id "lnc-bbut00008540"	exon_number "1"
12	lncRNA	exon	6575956	6576055	.	+	.	gene_id "lnc-bbug03720"	transcript_id "lnc-bbut00008540"	exon_number "2"
12	lncRNA	exon	6578667	6579431	.	+	.	gene_id "lnc-bbug03720"	transcript_id "lnc-bbut00008540"	exon_number "3"
12	lncRNA	exon	6580403	6581709	.	+	.	gene_id "lnc-bbug03720"	transcript_id "lnc-bbut00008540"	exon_number "4"
12	lncRNA	exon	6875304	6876545	.	+	.	gene_id "lnc-bbug03723"	transcript_id "lnc-bbut00008554"	exon_number "1"
12	lncRNA	exon	7666403	7666509	.	+	.	gene_id "lnc-bbug03731"	transcript_id "lnc-bbut00008581"	exon_number "1"
12	lncRNA	exon	7671508	7672070	.	+	.	gene_id "lnc-bbug03731"	transcript_id "lnc-bbut00008581"	exon_number "2"
12	lncRNA	exon	7676076	7677916	.	+	.	gene_id "lnc-bbug03731"	transcript_id "lnc-bbut00008581"	exon_number "3"
12	lncRNA	exon	8319976	8320997	.	+	.	gene_id "lnc-bbug03732"	transcript_id "lnc-bbut00008582"	exon_number "1"
12	lncRNA	exon	9212249	9213277	.	+	.	gene_id "lnc-bbug03737"	transcript_id "lnc-bbut00008587"	exon_number "1"
12	lncRNA	exon	9216387	9220257	.	+	.	gene_id "lnc-bbug03737"	transcript_id "lnc-bbut00008587"	exon_number "2"
12	lncRNA	exon	9212249	9213277	.	+	.	gene_id "lnc-bbug03737"	transcript_id "lnc-bbut00008588"	exon_number "1"
12	lncRNA	exon	9238338	9241210	.	+	.	gene_id "lnc-bbug03737"	transcript_id "lnc-bbut00008588"	exon_number "2"
12	lncRNA	exon	9748483	9748923	.	+	.	gene_id "lnc-bbug03740"	transcript_id "lnc-bbut00008591"	exon_number "1"
12	lncRNA	exon	9749880	9749987	.	+	.	gene_id "lnc-bbug03740"	transcript_id "lnc-bbut00008591"	exon_number "2"
12	lncRNA	exon	9750811	9754643	.	+	.	gene_id "lnc-bbug03740"	transcript_id "lnc-bbut00008591"	exon_number "3"
12	lncRNA	exon	10397257	10397413	.	+	.	gene_id "lnc-bbug03756"	transcript_id "lnc-bbut00008622"	exon_number "1"
12	lncRNA	exon	10399995	10400118	.	+	.	gene_id "lnc-bbug03756"	transcript_id "lnc-bbut00008622"	exon_number "2"
12	lncRNA	exon	10402078	10402166	.	+	.	gene_id "lnc-bbug03756"	transcript_id "lnc-bbut00008622"	exon_number "3"
12	lncRNA	exon	10404842	10405907	.	+	.	gene_id "lnc-bbug03756"	transcript_id "lnc-bbut00008622"	exon_number "4"
12	lncRNA	exon	10530287	10530657	.	+	.	gene_id "lnc-bbug03757"	transcript_id "lnc-bbut00008623"	exon_number "1"
12	lncRNA	exon	10543961	10544282	.	+	.	gene_id "lnc-bbug03757"	transcript_id "lnc-bbut00008623"	exon_number "2"
12	lncRNA	exon	10583252	10583784	.	+	.	gene_id "lnc-bbug03758"	transcript_id "lnc-bbut00008625"	exon_number "1"
12	lncRNA	exon	10584083	10584177	.	+	.	gene_id "lnc-bbug03758"	transcript_id "lnc-bbut00008625"	exon_number "2"
12	lncRNA	exon	10585990	10586096	.	+	.	gene_id "lnc-bbug03758"	transcript_id "lnc-bbut00008625"	exon_number "3"
12	lncRNA	exon	10586323	10589305	.	+	.	gene_id "lnc-bbug03758"	transcript_id "lnc-bbut00008625"	exon_number "4"
12	lncRNA	exon	10583252	10583784	.	+	.	gene_id "lnc-bbug03758"	transcript_id "lnc-bbut00008624"	exon_number "1"
12	lncRNA	exon	10584083	10584177	.	+	.	gene_id "lnc-bbug03758"	transcript_id "lnc-bbut00008624"	exon_number "2"
12	lncRNA	exon	10585990	10589305	.	+	.	gene_id "lnc-bbug03758"	transcript_id "lnc-bbut00008624"	exon_number "3"
12	lncRNA	exon	11130936	11131875	.	+	.	gene_id "lnc-bbug03766"	transcript_id "lnc-bbut00008645"	exon_number "1"
12	lncRNA	exon	11132338	11132528	.	+	.	gene_id "lnc-bbug03766"	transcript_id "lnc-bbut00008645"	exon_number "2"
12	lncRNA	exon	12374150	12374205	.	+	.	gene_id "lnc-bbug03771"	transcript_id "lnc-bbut00008668"	exon_number "1"
12	lncRNA	exon	12374768	12374802	.	+	.	gene_id "lnc-bbug03771"	transcript_id "lnc-bbut00008668"	exon_number "2"
12	lncRNA	exon	12376552	12376748	.	+	.	gene_id "lnc-bbug03771"	transcript_id "lnc-bbut00008668"	exon_number "3"
12	lncRNA	exon	12882742	12883305	.	+	.	gene_id "lnc-bbug03772"	transcript_id "lnc-bbut00008669"	exon_number "1"
12	lncRNA	exon	12883778	12883992	.	+	.	gene_id "lnc-bbug03772"	transcript_id "lnc-bbut00008669"	exon_number "2"

12	lncRNA	exon	12884758	12884878	.	+	.	gene_id "lnc-bbug03772"	transcript_id "lnc-bbut00008669"	exon_number "3"
12	lncRNA	exon	12885175	12885271	.	+	.	gene_id "lnc-bbug03772"	transcript_id "lnc-bbut00008669"	exon_number "4"
12	lncRNA	exon	12885684	12885904	.	+	.	gene_id "lnc-bbug03772"	transcript_id "lnc-bbut00008669"	exon_number "5"
12	lncRNA	exon	12886136	12886431	.	+	.	gene_id "lnc-bbug03772"	transcript_id "lnc-bbut00008669"	exon_number "6"
12	lncRNA	exon	12882742	12883305	.	+	.	gene_id "lnc-bbug03772"	transcript_id "lnc-bbut00008671"	exon_number "1"
12	lncRNA	exon	12883778	12883992	.	+	.	gene_id "lnc-bbug03772"	transcript_id "lnc-bbut00008671"	exon_number "2"
12	lncRNA	exon	12884758	12884878	.	+	.	gene_id "lnc-bbug03772"	transcript_id "lnc-bbut00008671"	exon_number "3"
12	lncRNA	exon	12885175	12885271	.	+	.	gene_id "lnc-bbug03772"	transcript_id "lnc-bbut00008671"	exon_number "4"
12	lncRNA	exon	12885684	12885904	.	+	.	gene_id "lnc-bbug03772"	transcript_id "lnc-bbut00008671"	exon_number "5"
12	lncRNA	exon	12889855	12890438	.	+	.	gene_id "lnc-bbug03772"	transcript_id "lnc-bbut00008671"	exon_number "6"
12	lncRNA	exon	12882742	12883305	.	+	.	gene_id "lnc-bbug03772"	transcript_id "lnc-bbut00008670"	exon_number "1"
12	lncRNA	exon	12883856	12883992	.	+	.	gene_id "lnc-bbug03772"	transcript_id "lnc-bbut00008670"	exon_number "2"
12	lncRNA	exon	12884758	12884878	.	+	.	gene_id "lnc-bbug03772"	transcript_id "lnc-bbut00008670"	exon_number "3"
12	lncRNA	exon	12885175	12885271	.	+	.	gene_id "lnc-bbug03772"	transcript_id "lnc-bbut00008670"	exon_number "4"
12	lncRNA	exon	12885684	12885904	.	+	.	gene_id "lnc-bbug03772"	transcript_id "lnc-bbut00008670"	exon_number "5"
12	lncRNA	exon	12889855	12890438	.	+	.	gene_id "lnc-bbug03772"	transcript_id "lnc-bbut00008670"	exon_number "6"
12	lncRNA	exon	12969464	12969579	.	+	.	gene_id "lnc-bbug03773"	transcript_id "lnc-bbut00008672"	exon_number "1"
12	lncRNA	exon	12969751	12969795	.	+	.	gene_id "lnc-bbug03773"	transcript_id "lnc-bbut00008672"	exon_number "2"
12	lncRNA	exon	12972717	12972823	.	+	.	gene_id "lnc-bbug03773"	transcript_id "lnc-bbut00008672"	exon_number "3"
12	lncRNA	exon	13678235	13678450	.	+	.	gene_id "lnc-bbug03781"	transcript_id "lnc-bbut00008681"	exon_number "1"
12	lncRNA	exon	13679420	13679516	.	+	.	gene_id "lnc-bbug03781"	transcript_id "lnc-bbut00008681"	exon_number "2"
12	lncRNA	exon	13680785	13681131	.	+	.	gene_id "lnc-bbug03781"	transcript_id "lnc-bbut00008681"	exon_number "3"
12	lncRNA	exon	13706814	13706987	.	+	.	gene_id "lnc-bbug03782"	transcript_id "lnc-bbut00008682"	exon_number "1"
12	lncRNA	exon	13712897	13713034	.	+	.	gene_id "lnc-bbug03782"	transcript_id "lnc-bbut00008682"	exon_number "2"
12	lncRNA	exon	13715170	13716313	.	+	.	gene_id "lnc-bbug03782"	transcript_id "lnc-bbut00008682"	exon_number "3"
12	lncRNA	exon	13782754	13782969	.	+	.	gene_id "lnc-bbug03783"	transcript_id "lnc-bbut00008683"	exon_number "1"
12	lncRNA	exon	13818369	13818422	.	+	.	gene_id "lnc-bbug03783"	transcript_id "lnc-bbut00008683"	exon_number "2"
12	lncRNA	exon	13878459	13878579	.	+	.	gene_id "lnc-bbug03783"	transcript_id "lnc-bbut00008683"	exon_number "3"
12	lncRNA	exon	13895317	13899474	.	+	.	gene_id "lnc-bbug03783"	transcript_id "lnc-bbut00008683"	exon_number "4"
12	lncRNA	exon	14347239	14348202	.	+	.	gene_id "lnc-bbug03787"	transcript_id "lnc-bbut00008689"	exon_number "1"
12	lncRNA	exon	14612780	14612827	.	+	.	gene_id "lnc-bbug03788"	transcript_id "lnc-bbut00008690"	exon_number "1"
12	lncRNA	exon	14614643	14615385	.	+	.	gene_id "lnc-bbug03788"	transcript_id "lnc-bbut00008690"	exon_number "2"
12	lncRNA	exon	18091623	18092591	.	+	.	gene_id "lnc-bbug03798"	transcript_id "lnc-bbut00008727"	exon_number "1"
12	lncRNA	exon	19496854	19496935	.	+	.	gene_id "lnc-bbug03803"	transcript_id "lnc-bbut00008736"	exon_number "1"
12	lncRNA	exon	19500062	19500177	.	+	.	gene_id "lnc-bbug03803"	transcript_id "lnc-bbut00008736"	exon_number "2"
12	lncRNA	exon	19501204	19501248	.	+	.	gene_id "lnc-bbug03803"	transcript_id "lnc-bbut00008736"	exon_number "3"
12	lncRNA	exon	19501895	19501979	.	+	.	gene_id "lnc-bbug03803"	transcript_id "lnc-bbut00008736"	exon_number "4"
12	lncRNA	exon	19503230	19503460	.	+	.	gene_id "lnc-bbug03803"	transcript_id "lnc-bbut00008736"	exon_number "5"
12	lncRNA	exon	19498519	19498642	.	+	.	gene_id "lnc-bbug03803"	transcript_id "lnc-bbut00008737"	exon_number "1"
12	lncRNA	exon	19500062	19500177	.	+	.	gene_id "lnc-bbug03803"	transcript_id "lnc-bbut00008737"	exon_number "2"
12	lncRNA	exon	19501204	19501248	.	+	.	gene_id "lnc-bbug03803"	transcript_id "lnc-bbut00008737"	exon_number "3"
12	lncRNA	exon	19501895	19501979	.	+	.	gene_id "lnc-bbug03803"	transcript_id "lnc-bbut00008737"	exon_number "4"
12	lncRNA	exon	19503230	19503460	.	+	.	gene_id "lnc-bbug03803"	transcript_id "lnc-bbut00008737"	exon_number "5"
12	lncRNA	exon	20336660	20337060	.	+	.	gene_id "lnc-bbug03807"	transcript_id "lnc-bbut00008746"	exon_number "1"
12	lncRNA	exon	20337663	20337765	.	+	.	gene_id "lnc-bbug03807"	transcript_id "lnc-bbut00008746"	exon_number "2"
12	lncRNA	exon	20520397	20523261	.	+	.	gene_id "lnc-bbug03808"	transcript_id "lnc-bbut00008747"	exon_number "1"
12	lncRNA	exon	20691542	20692358	.	+	.	gene_id "lnc-bbug03809"	transcript_id "lnc-bbut00008748"	exon_number "1"
12	lncRNA	exon	20735922	20736643	.	+	.	gene_id "lnc-bbug03810"	transcript_id "lnc-bbut00008749"	exon_number "1"
12	lncRNA	exon	20736959	20736981	.	+	.	gene_id "lnc-bbug03810"	transcript_id "lnc-bbut00008749"	exon_number "2"
12	lncRNA	exon	20737063	20737113	.	+	.	gene_id "lnc-bbug03811"	transcript_id "lnc-bbut00008750"	exon_number "1"
12	lncRNA	exon	20737352	20740139	.	+	.	gene_id "lnc-bbug03811"	transcript_id "lnc-bbut00008750"	exon_number "2"
12	lncRNA	exon	20864068	20864253	.	+	.	gene_id "lnc-bbug03813"	transcript_id "lnc-bbut00008752"	exon_number "1"

12	lncRNA	exon	20873400	20873481	.	+	.	gene_id "lnc-bbug03813"	transcript_id "lnc-bbut00008752"	exon_number "2"
12	lncRNA	exon	20874102	20874598	.	+	.	gene_id "lnc-bbug03813"	transcript_id "lnc-bbut00008752"	exon_number "3"
12	lncRNA	exon	20900948	20906827	.	+	.	gene_id "lnc-bbug03815"	transcript_id "lnc-bbut00008754"	exon_number "1"
12	lncRNA	exon	20906913	20907147	.	+	.	gene_id "lnc-bbug03815"	transcript_id "lnc-bbut00008754"	exon_number "2"
12	lncRNA	exon	21066685	21066812	.	+	.	gene_id "lnc-bbug03817"	transcript_id "lnc-bbut00008760"	exon_number "1"
12	lncRNA	exon	21072692	21072828	.	+	.	gene_id "lnc-bbug03817"	transcript_id "lnc-bbut00008760"	exon_number "2"
12	lncRNA	exon	21074487	21074718	.	+	.	gene_id "lnc-bbug03817"	transcript_id "lnc-bbut00008760"	exon_number "3"
12	lncRNA	exon	21066691	21066812	.	+	.	gene_id "lnc-bbug03817"	transcript_id "lnc-bbut00008761"	exon_number "1"
12	lncRNA	exon	21071087	21071137	.	+	.	gene_id "lnc-bbug03817"	transcript_id "lnc-bbut00008761"	exon_number "2"
12	lncRNA	exon	21071575	21071681	.	+	.	gene_id "lnc-bbug03817"	transcript_id "lnc-bbut00008761"	exon_number "3"
12	lncRNA	exon	21072692	21072828	.	+	.	gene_id "lnc-bbug03817"	transcript_id "lnc-bbut00008761"	exon_number "4"
12	lncRNA	exon	21074487	21074718	.	+	.	gene_id "lnc-bbug03817"	transcript_id "lnc-bbut00008761"	exon_number "5"
12	lncRNA	exon	21066704	21066812	.	+	.	gene_id "lnc-bbug03817"	transcript_id "lnc-bbut00008762"	exon_number "1"
12	lncRNA	exon	21071575	21071681	.	+	.	gene_id "lnc-bbug03817"	transcript_id "lnc-bbut00008762"	exon_number "2"
12	lncRNA	exon	21072692	21072828	.	+	.	gene_id "lnc-bbug03817"	transcript_id "lnc-bbut00008762"	exon_number "3"
12	lncRNA	exon	21074487	21074718	.	+	.	gene_id "lnc-bbug03817"	transcript_id "lnc-bbut00008762"	exon_number "4"
12	lncRNA	exon	21678546	21679227	.	+	.	gene_id "lnc-bbug03820"	transcript_id "lnc-bbut00008770"	exon_number "1"
12	lncRNA	exon	21679817	21679944	.	+	.	gene_id "lnc-bbug03820"	transcript_id "lnc-bbut00008770"	exon_number "2"
12	lncRNA	exon	21687148	21687198	.	+	.	gene_id "lnc-bbug03820"	transcript_id "lnc-bbut00008770"	exon_number "3"
12	lncRNA	exon	21678546	21679227	.	+	.	gene_id "lnc-bbug03820"	transcript_id "lnc-bbut00008769"	exon_number "1"
12	lncRNA	exon	21679757	21679944	.	+	.	gene_id "lnc-bbug03820"	transcript_id "lnc-bbut00008769"	exon_number "2"
12	lncRNA	exon	21687148	21687198	.	+	.	gene_id "lnc-bbug03820"	transcript_id "lnc-bbut00008769"	exon_number "3"
12	lncRNA	exon	22083385	22083436	.	+	.	gene_id "lnc-bbug03822"	transcript_id "lnc-bbut00008772"	exon_number "1"
12	lncRNA	exon	22095474	22095528	.	+	.	gene_id "lnc-bbug03822"	transcript_id "lnc-bbut00008772"	exon_number "2"
12	lncRNA	exon	22114805	22114962	.	+	.	gene_id "lnc-bbug03822"	transcript_id "lnc-bbut00008772"	exon_number "3"
12	lncRNA	exon	22143298	22143708	.	+	.	gene_id "lnc-bbug03822"	transcript_id "lnc-bbut00008772"	exon_number "4"
12	lncRNA	exon	22731026	22731172	.	+	.	gene_id "lnc-bbug03823"	transcript_id "lnc-bbut00008773"	exon_number "1"
12	lncRNA	exon	22745793	22746261	.	+	.	gene_id "lnc-bbug03823"	transcript_id "lnc-bbut00008773"	exon_number "2"
12	lncRNA	exon	22749803	22749849	.	+	.	gene_id "lnc-bbug03823"	transcript_id "lnc-bbut00008773"	exon_number "3"
12	lncRNA	exon	22750405	22750556	.	+	.	gene_id "lnc-bbug03824"	transcript_id "lnc-bbut00008774"	exon_number "1"
12	lncRNA	exon	22751589	22751663	.	+	.	gene_id "lnc-bbug03824"	transcript_id "lnc-bbut00008774"	exon_number "2"
12	lncRNA	exon	22765220	22765637	.	+	.	gene_id "lnc-bbug03824"	transcript_id "lnc-bbut00008774"	exon_number "3"
12	lncRNA	exon	23492759	23493132	.	+	.	gene_id "lnc-bbug03825"	transcript_id "lnc-bbut00008775"	exon_number "1"
12	lncRNA	exon	24457023	24458183	.	+	.	gene_id "lnc-bbug03827"	transcript_id "lnc-bbut00008779"	exon_number "1"
12	lncRNA	exon	24458619	24458890	.	+	.	gene_id "lnc-bbug03827"	transcript_id "lnc-bbut00008779"	exon_number "2"
12	lncRNA	exon	24461373	24461556	.	+	.	gene_id "lnc-bbug03827"	transcript_id "lnc-bbut00008779"	exon_number "3"
12	lncRNA	exon	24462113	24462252	.	+	.	gene_id "lnc-bbug03827"	transcript_id "lnc-bbut00008779"	exon_number "4"
12	lncRNA	exon	24463300	24463431	.	+	.	gene_id "lnc-bbug03827"	transcript_id "lnc-bbut00008779"	exon_number "5"
12	lncRNA	exon	24479748	24479802	.	+	.	gene_id "lnc-bbug03827"	transcript_id "lnc-bbut00008779"	exon_number "6"
12	lncRNA	exon	24488938	24489159	.	+	.	gene_id "lnc-bbug03827"	transcript_id "lnc-bbut00008779"	exon_number "7"
12	lncRNA	exon	24491309	24491433	.	+	.	gene_id "lnc-bbug03827"	transcript_id "lnc-bbut00008779"	exon_number "8"
12	lncRNA	exon	24460066	24461203	.	+	.	gene_id "lnc-bbug03827"	transcript_id "lnc-bbut00008780"	exon_number "1"
12	lncRNA	exon	24461373	24461556	.	+	.	gene_id "lnc-bbug03827"	transcript_id "lnc-bbut00008780"	exon_number "2"
12	lncRNA	exon	24462113	24462252	.	+	.	gene_id "lnc-bbug03827"	transcript_id "lnc-bbut00008780"	exon_number "3"
12	lncRNA	exon	24463300	24463431	.	+	.	gene_id "lnc-bbug03827"	transcript_id "lnc-bbut00008780"	exon_number "4"
12	lncRNA	exon	24479748	24479802	.	+	.	gene_id "lnc-bbug03827"	transcript_id "lnc-bbut00008780"	exon_number "5"
12	lncRNA	exon	24488938	24489159	.	+	.	gene_id "lnc-bbug03827"	transcript_id "lnc-bbut00008780"	exon_number "6"
12	lncRNA	exon	24491309	24491433	.	+	.	gene_id "lnc-bbug03827"	transcript_id "lnc-bbut00008780"	exon_number "7"
12	lncRNA	exon	24465284	24467599	.	+	.	gene_id "lnc-bbug03828"	transcript_id "lnc-bbut00008781"	exon_number "1"
12	lncRNA	exon	24470573	24470685	.	+	.	gene_id "lnc-bbug03828"	transcript_id "lnc-bbut00008781"	exon_number "2"
12	lncRNA	exon	25263342	25263905	.	+	.	gene_id "lnc-bbug03834"	transcript_id "lnc-bbut00008801"	exon_number "1"
12	lncRNA	exon	25264885	25265034	.	+	.	gene_id "lnc-bbug03834"	transcript_id "lnc-bbut00008801"	exon_number "2"

12	lncRNA	exon	25265447	25267807	.	+	.	gene_id "lnc-bbug03834"	transcript_id "lnc-bbut00008801"	exon_number "3"
12	lncRNA	exon	25467835	25468002	.	+	.	gene_id "lnc-bbug03835"	transcript_id "lnc-bbut00008802"	exon_number "1"
12	lncRNA	exon	25471808	25471889	.	+	.	gene_id "lnc-bbug03835"	transcript_id "lnc-bbut00008802"	exon_number "2"
12	lncRNA	exon	25479278	25479798	.	+	.	gene_id "lnc-bbug03835"	transcript_id "lnc-bbut00008802"	exon_number "3"
12	lncRNA	exon	25759001	25759428	.	+	.	gene_id "lnc-bbug03836"	transcript_id "lnc-bbut00008803"	exon_number "1"
12	lncRNA	exon	26673524	26674132	.	+	.	gene_id "lnc-bbug03843"	transcript_id "lnc-bbut00008818"	exon_number "1"
12	lncRNA	exon	27451088	27451657	.	+	.	gene_id "lnc-bbug03845"	transcript_id "lnc-bbut00008820"	exon_number "1"
12	lncRNA	exon	27577310	27578182	.	+	.	gene_id "lnc-bbug03846"	transcript_id "lnc-bbut00008821"	exon_number "1"
12	lncRNA	exon	28735481	28735616	.	+	.	gene_id "lnc-bbug03851"	transcript_id "lnc-bbut00008828"	exon_number "1"
12	lncRNA	exon	28737791	28737856	.	+	.	gene_id "lnc-bbug03851"	transcript_id "lnc-bbut00008828"	exon_number "2"
12	lncRNA	exon	28741920	28741974	.	+	.	gene_id "lnc-bbug03851"	transcript_id "lnc-bbut00008828"	exon_number "3"
12	lncRNA	exon	28745117	28745220	.	+	.	gene_id "lnc-bbug03851"	transcript_id "lnc-bbut00008828"	exon_number "4"
12	lncRNA	exon	28745703	28746595	.	+	.	gene_id "lnc-bbug03851"	transcript_id "lnc-bbut00008828"	exon_number "5"
12	lncRNA	exon	28953714	28954077	.	+	.	gene_id "lnc-bbug03853"	transcript_id "lnc-bbut00008833"	exon_number "1"
12	lncRNA	exon	29047361	29047501	.	+	.	gene_id "lnc-bbug03854"	transcript_id "lnc-bbut00008834"	exon_number "1"
12	lncRNA	exon	29047890	29047978	.	+	.	gene_id "lnc-bbug03854"	transcript_id "lnc-bbut00008834"	exon_number "2"
12	lncRNA	exon	29048310	29048587	.	+	.	gene_id "lnc-bbug03854"	transcript_id "lnc-bbut00008834"	exon_number "3"
12	lncRNA	exon	29047361	29047501	.	+	.	gene_id "lnc-bbug03854"	transcript_id "lnc-bbut00008835"	exon_number "1"
12	lncRNA	exon	29047890	29047978	.	+	.	gene_id "lnc-bbug03854"	transcript_id "lnc-bbut00008835"	exon_number "2"
12	lncRNA	exon	29053010	29053329	.	+	.	gene_id "lnc-bbug03854"	transcript_id "lnc-bbut00008835"	exon_number "3"
12	lncRNA	exon	29053466	29053812	.	+	.	gene_id "lnc-bbug03855"	transcript_id "lnc-bbut00008836"	exon_number "1"
12	lncRNA	exon	29054635	29054711	.	+	.	gene_id "lnc-bbug03855"	transcript_id "lnc-bbut00008836"	exon_number "2"
12	lncRNA	exon	29075303	29075394	.	+	.	gene_id "lnc-bbug03855"	transcript_id "lnc-bbut00008836"	exon_number "3"
12	lncRNA	exon	29053466	29053812	.	+	.	gene_id "lnc-bbug03855"	transcript_id "lnc-bbut00008837"	exon_number "1"
12	lncRNA	exon	29054635	29054711	.	+	.	gene_id "lnc-bbug03855"	transcript_id "lnc-bbut00008837"	exon_number "2"
12	lncRNA	exon	29077144	29077872	.	+	.	gene_id "lnc-bbug03855"	transcript_id "lnc-bbut00008837"	exon_number "3"
12	lncRNA	exon	29315233	29315734	.	+	.	gene_id "lnc-bbug03857"	transcript_id "lnc-bbut00008843"	exon_number "1"
12	lncRNA	exon	29320826	29320883	.	+	.	gene_id "lnc-bbug03857"	transcript_id "lnc-bbut00008843"	exon_number "2"
12	lncRNA	exon	29321094	29321140	.	+	.	gene_id "lnc-bbug03857"	transcript_id "lnc-bbut00008843"	exon_number "3"
12	lncRNA	exon	29321996	29322128	.	+	.	gene_id "lnc-bbug03857"	transcript_id "lnc-bbut00008843"	exon_number "4"
12	lncRNA	exon	29330458	29330544	.	+	.	gene_id "lnc-bbug03857"	transcript_id "lnc-bbut00008843"	exon_number "5"
12	lncRNA	exon	29333065	29333504	.	+	.	gene_id "lnc-bbug03857"	transcript_id "lnc-bbut00008843"	exon_number "6"
12	lncRNA	exon	29315233	29315734	.	+	.	gene_id "lnc-bbug03857"	transcript_id "lnc-bbut00008844"	exon_number "1"
12	lncRNA	exon	29316634	29316834	.	+	.	gene_id "lnc-bbug03857"	transcript_id "lnc-bbut00008844"	exon_number "2"
12	lncRNA	exon	29320826	29320883	.	+	.	gene_id "lnc-bbug03857"	transcript_id "lnc-bbut00008844"	exon_number "3"
12	lncRNA	exon	29321094	29321140	.	+	.	gene_id "lnc-bbug03857"	transcript_id "lnc-bbut00008844"	exon_number "4"
12	lncRNA	exon	29321996	29322128	.	+	.	gene_id "lnc-bbug03857"	transcript_id "lnc-bbut00008844"	exon_number "5"
12	lncRNA	exon	29330458	29330544	.	+	.	gene_id "lnc-bbug03857"	transcript_id "lnc-bbut00008844"	exon_number "6"
12	lncRNA	exon	29333065	29333504	.	+	.	gene_id "lnc-bbug03857"	transcript_id "lnc-bbut00008844"	exon_number "7"
12	lncRNA	exon	29315233	29315734	.	+	.	gene_id "lnc-bbug03857"	transcript_id "lnc-bbut00008842"	exon_number "1"
12	lncRNA	exon	29316634	29316834	.	+	.	gene_id "lnc-bbug03857"	transcript_id "lnc-bbut00008842"	exon_number "2"
12	lncRNA	exon	29320826	29320883	.	+	.	gene_id "lnc-bbug03857"	transcript_id "lnc-bbut00008842"	exon_number "3"
12	lncRNA	exon	29321996	29322128	.	+	.	gene_id "lnc-bbug03857"	transcript_id "lnc-bbut00008842"	exon_number "4"
12	lncRNA	exon	29330458	29330544	.	+	.	gene_id "lnc-bbug03857"	transcript_id "lnc-bbut00008842"	exon_number "5"
12	lncRNA	exon	29333065	29333504	.	+	.	gene_id "lnc-bbug03857"	transcript_id "lnc-bbut00008842"	exon_number "6"
12	lncRNA	exon	29315233	29315734	.	+	.	gene_id "lnc-bbug03857"	transcript_id "lnc-bbut00008841"	exon_number "1"
12	lncRNA	exon	29320826	29320883	.	+	.	gene_id "lnc-bbug03857"	transcript_id "lnc-bbut00008841"	exon_number "2"
12	lncRNA	exon	29321996	29322128	.	+	.	gene_id "lnc-bbug03857"	transcript_id "lnc-bbut00008841"	exon_number "3"
12	lncRNA	exon	29330458	29330544	.	+	.	gene_id "lnc-bbug03857"	transcript_id "lnc-bbut00008841"	exon_number "4"
12	lncRNA	exon	29333065	29333504	.	+	.	gene_id "lnc-bbug03857"	transcript_id "lnc-bbut00008841"	exon_number "5"
12	lncRNA	exon	30013360	30013894	.	+	.	gene_id "lnc-bbug03861"	transcript_id "lnc-bbut00008848"	exon_number "1"
12	lncRNA	exon	32847506	32848156	.	+	.	gene_id "lnc-bbug03867"	transcript_id "lnc-bbut00008865"	exon_number "1"

12	lncRNA	exon	36211255	36211778	.	+	.	gene_id "lnc-bbug03871"	transcript_id "lnc-bbut00008871"	exon_number "1"
12	lncRNA	exon	36212403	36213370	.	+	.	gene_id "lnc-bbug03871"	transcript_id "lnc-bbut00008871"	exon_number "2"
12	lncRNA	exon	36496210	36496463	.	+	.	gene_id "lnc-bbug03872"	transcript_id "lnc-bbut00008872"	exon_number "1"
12	lncRNA	exon	36513189	36513205	.	+	.	gene_id "lnc-bbug03872"	transcript_id "lnc-bbut00008872"	exon_number "2"
12	lncRNA	exon	36514869	36514978	.	+	.	gene_id "lnc-bbug03872"	transcript_id "lnc-bbut00008872"	exon_number "3"
12	lncRNA	exon	36515159	36517797	.	+	.	gene_id "lnc-bbug03872"	transcript_id "lnc-bbut00008872"	exon_number "4"
12	lncRNA	exon	36496210	36496463	.	+	.	gene_id "lnc-bbug03872"	transcript_id "lnc-bbut00008876"	exon_number "1"
12	lncRNA	exon	36513189	36513205	.	+	.	gene_id "lnc-bbug03872"	transcript_id "lnc-bbut00008876"	exon_number "2"
12	lncRNA	exon	36514869	36514978	.	+	.	gene_id "lnc-bbug03872"	transcript_id "lnc-bbut00008876"	exon_number "3"
12	lncRNA	exon	36672623	36672704	.	+	.	gene_id "lnc-bbug03872"	transcript_id "lnc-bbut00008876"	exon_number "4"
12	lncRNA	exon	36675741	36676377	.	+	.	gene_id "lnc-bbug03872"	transcript_id "lnc-bbut00008876"	exon_number "5"
12	lncRNA	exon	36704425	36707022	.	+	.	gene_id "lnc-bbug03873"	transcript_id "lnc-bbut00008880"	exon_number "1"
12	lncRNA	exon	38120490	38120944	.	+	.	gene_id "lnc-bbug03879"	transcript_id "lnc-bbut00008902"	exon_number "1"
12	lncRNA	exon	40698080	40698639	.	+	.	gene_id "lnc-bbug03883"	transcript_id "lnc-bbut00008919"	exon_number "1"
12	lncRNA	exon	41132532	41133822	.	+	.	gene_id "lnc-bbug03884"	transcript_id "lnc-bbut00008920"	exon_number "1"
12	lncRNA	exon	41266417	41266582	.	+	.	gene_id "lnc-bbug03884"	transcript_id "lnc-bbut00008920"	exon_number "2"
12	lncRNA	exon	41291840	41292026	.	+	.	gene_id "lnc-bbug03884"	transcript_id "lnc-bbut00008920"	exon_number "3"
12	lncRNA	exon	41294408	41294452	.	+	.	gene_id "lnc-bbug03884"	transcript_id "lnc-bbut00008920"	exon_number "4"
12	lncRNA	exon	41303876	41303991	.	+	.	gene_id "lnc-bbug03884"	transcript_id "lnc-bbut00008920"	exon_number "5"
12	lncRNA	exon	41318586	41318710	.	+	.	gene_id "lnc-bbug03884"	transcript_id "lnc-bbut00008920"	exon_number "6"
12	lncRNA	exon	41352141	41352279	.	+	.	gene_id "lnc-bbug03884"	transcript_id "lnc-bbut00008920"	exon_number "7"
12	lncRNA	exon	41359281	41360530	.	+	.	gene_id "lnc-bbug03884"	transcript_id "lnc-bbut00008920"	exon_number "8"
12	lncRNA	exon	41466229	41467145	.	+	.	gene_id "lnc-bbug03885"	transcript_id "lnc-bbut00008921"	exon_number "1"
12	lncRNA	exon	41485772	41485811	.	+	.	gene_id "lnc-bbug03885"	transcript_id "lnc-bbut00008921"	exon_number "2"
12	lncRNA	exon	41500735	41502657	.	+	.	gene_id "lnc-bbug03885"	transcript_id "lnc-bbut00008921"	exon_number "3"
12	lncRNA	exon	42939357	42939643	.	+	.	gene_id "lnc-bbug03886"	transcript_id "lnc-bbut00008922"	exon_number "1"
12	lncRNA	exon	42943317	42943498	.	+	.	gene_id "lnc-bbug03886"	transcript_id "lnc-bbut00008922"	exon_number "2"
12	lncRNA	exon	43240800	43244991	.	+	.	gene_id "lnc-bbug03888"	transcript_id "lnc-bbut00008928"	exon_number "1"
12	lncRNA	exon	43399319	43402211	.	+	.	gene_id "lnc-bbug03890"	transcript_id "lnc-bbut00008932"	exon_number "1"
12	lncRNA	exon	43404982	43405976	.	+	.	gene_id "lnc-bbug03890"	transcript_id "lnc-bbut00008932"	exon_number "2"
12	lncRNA	exon	43399319	43402211	.	+	.	gene_id "lnc-bbug03890"	transcript_id "lnc-bbut00008933"	exon_number "1"
12	lncRNA	exon	43412231	43413110	.	+	.	gene_id "lnc-bbug03890"	transcript_id "lnc-bbut00008933"	exon_number "2"
12	lncRNA	exon	43777340	43777738	.	+	.	gene_id "lnc-bbug03896"	transcript_id "lnc-bbut00008943"	exon_number "1"
12	lncRNA	exon	44187675	44187989	.	+	.	gene_id "lnc-bbug03900"	transcript_id "lnc-bbut00008949"	exon_number "1"
12	lncRNA	exon	44188460	44188718	.	+	.	gene_id "lnc-bbug03900"	transcript_id "lnc-bbut00008949"	exon_number "2"
12	lncRNA	exon	44260512	44262308	.	+	.	gene_id "lnc-bbug03901"	transcript_id "lnc-bbut00008950"	exon_number "1"
12	lncRNA	exon	44263598	44263990	.	+	.	gene_id "lnc-bbug03901"	transcript_id "lnc-bbut00008950"	exon_number "2"
12	lncRNA	exon	44264610	44266032	.	+	.	gene_id "lnc-bbug03901"	transcript_id "lnc-bbut00008950"	exon_number "3"
12	lncRNA	exon	44655836	44655971	.	+	.	gene_id "lnc-bbug03903"	transcript_id "lnc-bbut00008953"	exon_number "1"
12	lncRNA	exon	44664566	44665071	.	+	.	gene_id "lnc-bbug03903"	transcript_id "lnc-bbut00008953"	exon_number "2"
12	lncRNA	exon	44666628	44666794	.	+	.	gene_id "lnc-bbug03903"	transcript_id "lnc-bbut00008953"	exon_number "3"
12	lncRNA	exon	44667026	44667654	.	+	.	gene_id "lnc-bbug03903"	transcript_id "lnc-bbut00008953"	exon_number "4"
12	lncRNA	exon	44655836	44655971	.	+	.	gene_id "lnc-bbug03903"	transcript_id "lnc-bbut00008954"	exon_number "1"
12	lncRNA	exon	44664566	44665071	.	+	.	gene_id "lnc-bbug03903"	transcript_id "lnc-bbut00008954"	exon_number "2"
12	lncRNA	exon	44666628	44666794	.	+	.	gene_id "lnc-bbug03903"	transcript_id "lnc-bbut00008954"	exon_number "3"
12	lncRNA	exon	44671091	44671137	.	+	.	gene_id "lnc-bbug03903"	transcript_id "lnc-bbut00008954"	exon_number "4"
12	lncRNA	exon	44655836	44655971	.	+	.	gene_id "lnc-bbug03903"	transcript_id "lnc-bbut00008957"	exon_number "1"
12	lncRNA	exon	44664566	44665071	.	+	.	gene_id "lnc-bbug03903"	transcript_id "lnc-bbut00008957"	exon_number "2"
12	lncRNA	exon	44666628	44666794	.	+	.	gene_id "lnc-bbug03903"	transcript_id "lnc-bbut00008957"	exon_number "3"
12	lncRNA	exon	44671535	44671587	.	+	.	gene_id "lnc-bbug03903"	transcript_id "lnc-bbut00008957"	exon_number "4"
12	lncRNA	exon	44673441	44674195	.	+	.	gene_id "lnc-bbug03903"	transcript_id "lnc-bbut00008957"	exon_number "5"
12	lncRNA	exon	44655836	44655971	.	+	.	gene_id "lnc-bbug03903"	transcript_id "lnc-bbut00008956"	exon_number "1"

12	lncRNA	exon	44664566	44665071	.	+	.	gene_id "lnc-bbug03903"	transcript_id "lnc-bbut00008956"	exon_number "2"
12	lncRNA	exon	44666628	44666794	.	+	.	gene_id "lnc-bbug03903"	transcript_id "lnc-bbut00008956"	exon_number "3"
12	lncRNA	exon	44673441	44674195	.	+	.	gene_id "lnc-bbug03903"	transcript_id "lnc-bbut00008956"	exon_number "4"
12	lncRNA	exon	44655836	44655971	.	+	.	gene_id "lnc-bbug03903"	transcript_id "lnc-bbut00008955"	exon_number "1"
12	lncRNA	exon	44664559	44665071	.	+	.	gene_id "lnc-bbug03903"	transcript_id "lnc-bbut00008955"	exon_number "2"
12	lncRNA	exon	44666628	44666794	.	+	.	gene_id "lnc-bbug03903"	transcript_id "lnc-bbut00008955"	exon_number "3"
12	lncRNA	exon	44671535	44674195	.	+	.	gene_id "lnc-bbug03903"	transcript_id "lnc-bbut00008955"	exon_number "4"
12	lncRNA	exon	44655836	44655971	.	+	.	gene_id "lnc-bbug03903"	transcript_id "lnc-bbut00008958"	exon_number "1"
12	lncRNA	exon	44664566	44665071	.	+	.	gene_id "lnc-bbug03903"	transcript_id "lnc-bbut00008958"	exon_number "2"
12	lncRNA	exon	44666628	44666794	.	+	.	gene_id "lnc-bbug03903"	transcript_id "lnc-bbut00008958"	exon_number "3"
12	lncRNA	exon	44671535	44674715	.	+	.	gene_id "lnc-bbug03903"	transcript_id "lnc-bbut00008958"	exon_number "4"
12	lncRNA	exon	44676098	44676439	.	+	.	gene_id "lnc-bbug03903"	transcript_id "lnc-bbut00008958"	exon_number "5"
12	lncRNA	exon	44655836	44655971	.	+	.	gene_id "lnc-bbug03903"	transcript_id "lnc-bbut00008959"	exon_number "1"
12	lncRNA	exon	44664566	44665071	.	+	.	gene_id "lnc-bbug03903"	transcript_id "lnc-bbut00008959"	exon_number "2"
12	lncRNA	exon	44666628	44666794	.	+	.	gene_id "lnc-bbug03903"	transcript_id "lnc-bbut00008959"	exon_number "3"
12	lncRNA	exon	44671535	44676225	.	+	.	gene_id "lnc-bbug03903"	transcript_id "lnc-bbut00008959"	exon_number "4"
12	lncRNA	exon	44676636	44676681	.	+	.	gene_id "lnc-bbug03903"	transcript_id "lnc-bbut00008959"	exon_number "5"
12	lncRNA	exon	44655841	44655971	.	+	.	gene_id "lnc-bbug03903"	transcript_id "lnc-bbut00008960"	exon_number "1"
12	lncRNA	exon	44657764	44657933	.	+	.	gene_id "lnc-bbug03903"	transcript_id "lnc-bbut00008960"	exon_number "2"
12	lncRNA	exon	44664566	44665071	.	+	.	gene_id "lnc-bbug03903"	transcript_id "lnc-bbut00008960"	exon_number "3"
12	lncRNA	exon	44666628	44666794	.	+	.	gene_id "lnc-bbug03903"	transcript_id "lnc-bbut00008960"	exon_number "4"
12	lncRNA	exon	44671535	44674195	.	+	.	gene_id "lnc-bbug03903"	transcript_id "lnc-bbut00008960"	exon_number "5"
12	lncRNA	exon	44768045	44768118	.	+	.	gene_id "lnc-bbug03905"	transcript_id "lnc-bbut00008962"	exon_number "1"
12	lncRNA	exon	44768798	44769163	.	+	.	gene_id "lnc-bbug03905"	transcript_id "lnc-bbut00008962"	exon_number "2"
12	lncRNA	exon	45897694	45899041	.	+	.	gene_id "lnc-bbug03910"	transcript_id "lnc-bbut00008968"	exon_number "1"
12	lncRNA	exon	46026307	46026800	.	+	.	gene_id "lnc-bbug03913"	transcript_id "lnc-bbut00008973"	exon_number "1"
12	lncRNA	exon	46028359	46028495	.	+	.	gene_id "lnc-bbug03913"	transcript_id "lnc-bbut00008973"	exon_number "2"
12	lncRNA	exon	46028578	46028743	.	+	.	gene_id "lnc-bbug03913"	transcript_id "lnc-bbut00008973"	exon_number "3"
12	lncRNA	exon	46033763	46035947	.	+	.	gene_id "lnc-bbug03913"	transcript_id "lnc-bbut00008973"	exon_number "4"
12	lncRNA	exon	46026312	46026800	.	+	.	gene_id "lnc-bbug03913"	transcript_id "lnc-bbut00008977"	exon_number "1"
12	lncRNA	exon	46028355	46028495	.	+	.	gene_id "lnc-bbug03913"	transcript_id "lnc-bbut00008977"	exon_number "2"
12	lncRNA	exon	46028578	46028743	.	+	.	gene_id "lnc-bbug03913"	transcript_id "lnc-bbut00008977"	exon_number "3"
12	lncRNA	exon	46033763	46035947	.	+	.	gene_id "lnc-bbug03913"	transcript_id "lnc-bbut00008977"	exon_number "4"
12	lncRNA	exon	46170681	46171164	.	+	.	gene_id "lnc-bbug03915"	transcript_id "lnc-bbut00008981"	exon_number "1"
12	lncRNA	exon	47265509	47265589	.	+	.	gene_id "lnc-bbug03928"	transcript_id "lnc-bbut00009010"	exon_number "1"
12	lncRNA	exon	47267058	47267187	.	+	.	gene_id "lnc-bbug03928"	transcript_id "lnc-bbut00009010"	exon_number "2"
12	lncRNA	exon	47277571	47277696	.	+	.	gene_id "lnc-bbug03928"	transcript_id "lnc-bbut00009010"	exon_number "3"
12	lncRNA	exon	47265521	47265641	.	+	.	gene_id "lnc-bbug03928"	transcript_id "lnc-bbut00009011"	exon_number "1"
12	lncRNA	exon	47267058	47267187	.	+	.	gene_id "lnc-bbug03928"	transcript_id "lnc-bbut00009011"	exon_number "2"
12	lncRNA	exon	47277571	47277696	.	+	.	gene_id "lnc-bbug03928"	transcript_id "lnc-bbut00009011"	exon_number "3"
12	lncRNA	exon	47265531	47265889	.	+	.	gene_id "lnc-bbug03928"	transcript_id "lnc-bbut00009012"	exon_number "1"
12	lncRNA	exon	47267058	47267187	.	+	.	gene_id "lnc-bbug03928"	transcript_id "lnc-bbut00009012"	exon_number "2"
12	lncRNA	exon	47277571	47277696	.	+	.	gene_id "lnc-bbug03928"	transcript_id "lnc-bbut00009012"	exon_number "3"
12	lncRNA	exon	47687846	47687914	.	+	.	gene_id "lnc-bbug03931"	transcript_id "lnc-bbut00009015"	exon_number "1"
12	lncRNA	exon	47688446	47688583	.	+	.	gene_id "lnc-bbug03931"	transcript_id "lnc-bbut00009015"	exon_number "2"
12	lncRNA	exon	47689696	47689792	.	+	.	gene_id "lnc-bbug03931"	transcript_id "lnc-bbut00009015"	exon_number "3"
12	lncRNA	exon	48911226	48911293	.	+	.	gene_id "lnc-bbug03942"	transcript_id "lnc-bbut00009043"	exon_number "1"
12	lncRNA	exon	48912259	48912543	.	+	.	gene_id "lnc-bbug03942"	transcript_id "lnc-bbut00009043"	exon_number "2"
12	lncRNA	exon	48956359	48956563	.	+	.	gene_id "lnc-bbug03942"	transcript_id "lnc-bbut00009043"	exon_number "3"
12	lncRNA	exon	48956845	48956912	.	+	.	gene_id "lnc-bbug03942"	transcript_id "lnc-bbut00009043"	exon_number "4"
12	lncRNA	exon	48961649	48961754	.	+	.	gene_id "lnc-bbug03942"	transcript_id "lnc-bbut00009043"	exon_number "5"
12	lncRNA	exon	49164376	49164940	.	+	.	gene_id "lnc-bbug03947"	transcript_id "lnc-bbut00009054"	exon_number "1"

12	lncRNA	exon	49165321	49165429	.	+	.	gene_id "lnc-bbug03947"	transcript_id "lnc-bbut00009054"	exon_number "2"
12	lncRNA	exon	49168234	49168830	.	+	.	gene_id "lnc-bbug03947"	transcript_id "lnc-bbut00009054"	exon_number "3"
12	lncRNA	exon	49170707	49170927	.	+	.	gene_id "lnc-bbug03947"	transcript_id "lnc-bbut00009054"	exon_number "4"
12	lncRNA	exon	49507751	49509088	.	+	.	gene_id "lnc-bbug03950"	transcript_id "lnc-bbut00009066"	exon_number "1"
12	lncRNA	exon	49750440	49752546	.	+	.	gene_id "lnc-bbug03951"	transcript_id "lnc-bbut00009067"	exon_number "1"
12	lncRNA	exon	49754105	49754795	.	+	.	gene_id "lnc-bbug03951"	transcript_id "lnc-bbut00009067"	exon_number "2"
12	lncRNA	exon	49817793	49821303	.	+	.	gene_id "lnc-bbug03952"	transcript_id "lnc-bbut00009068"	exon_number "1"
12	lncRNA	exon	49822810	49822976	.	+	.	gene_id "lnc-bbug03952"	transcript_id "lnc-bbut00009068"	exon_number "2"
12	lncRNA	exon	49831540	49831999	.	+	.	gene_id "lnc-bbug03952"	transcript_id "lnc-bbut00009068"	exon_number "3"
12	lncRNA	exon	49835915	49836094	.	+	.	gene_id "lnc-bbug03952"	transcript_id "lnc-bbut00009068"	exon_number "4"
12	lncRNA	exon	49837136	49837279	.	+	.	gene_id "lnc-bbug03952"	transcript_id "lnc-bbut00009068"	exon_number "5"
12	lncRNA	exon	50185543	50185604	.	+	.	gene_id "lnc-bbug03954"	transcript_id "lnc-bbut00009070"	exon_number "1"
12	lncRNA	exon	50187449	50188019	.	+	.	gene_id "lnc-bbug03954"	transcript_id "lnc-bbut00009070"	exon_number "2"
12	lncRNA	exon	56682987	56683264	.	+	.	gene_id "lnc-bbug03962"	transcript_id "lnc-bbut00009082"	exon_number "1"
12	lncRNA	exon	56873654	56874472	.	+	.	gene_id "lnc-bbug03963"	transcript_id "lnc-bbut00009083"	exon_number "1"
12	lncRNA	exon	58286928	58294343	.	+	.	gene_id "lnc-bbug03965"	transcript_id "lnc-bbut00009085"	exon_number "1"
12	lncRNA	exon	60354275	60358546	.	+	.	gene_id "lnc-bbug03971"	transcript_id "lnc-bbut00009094"	exon_number "1"
12	lncRNA	exon	60359638	60359738	.	+	.	gene_id "lnc-bbug03971"	transcript_id "lnc-bbut00009094"	exon_number "2"
12	lncRNA	exon	60363146	60363925	.	+	.	gene_id "lnc-bbug03971"	transcript_id "lnc-bbut00009094"	exon_number "3"
12	lncRNA	exon	60354275	60355248	.	+	.	gene_id "lnc-bbug03971"	transcript_id "lnc-bbut00009093"	exon_number "1"
12	lncRNA	exon	60358471	60358546	.	+	.	gene_id "lnc-bbug03971"	transcript_id "lnc-bbut00009093"	exon_number "2"
12	lncRNA	exon	60363146	60363925	.	+	.	gene_id "lnc-bbug03971"	transcript_id "lnc-bbut00009093"	exon_number "3"
12	lncRNA	exon	62807005	62807437	.	+	.	gene_id "lnc-bbug03978"	transcript_id "lnc-bbut00009112"	exon_number "1"
12	lncRNA	exon	62808666	62809950	.	+	.	gene_id "lnc-bbug03978"	transcript_id "lnc-bbut00009112"	exon_number "2"
12	lncRNA	exon	62841663	62841823	.	+	.	gene_id "lnc-bbug03979"	transcript_id "lnc-bbut00009113"	exon_number "1"
12	lncRNA	exon	62887328	62887590	.	+	.	gene_id "lnc-bbug03979"	transcript_id "lnc-bbut00009113"	exon_number "2"
12	lncRNA	exon	62899225	62899425	.	+	.	gene_id "lnc-bbug03979"	transcript_id "lnc-bbut00009113"	exon_number "3"
12	lncRNA	exon	63069110	63069315	.	+	.	gene_id "lnc-bbug03981"	transcript_id "lnc-bbut00009115"	exon_number "1"
12	lncRNA	exon	63077779	63078074	.	+	.	gene_id "lnc-bbug03981"	transcript_id "lnc-bbut00009115"	exon_number "2"
12	lncRNA	exon	63079632	63080223	.	+	.	gene_id "lnc-bbug03981"	transcript_id "lnc-bbut00009115"	exon_number "3"
12	lncRNA	exon	63272075	63274597	.	+	.	gene_id "lnc-bbug03984"	transcript_id "lnc-bbut00009125"	exon_number "1"
12	lncRNA	exon	63314619	63314799	.	+	.	gene_id "lnc-bbug03984"	transcript_id "lnc-bbut00009125"	exon_number "2"
12	lncRNA	exon	64148624	64148753	.	+	.	gene_id "lnc-bbug03985"	transcript_id "lnc-bbut00009126"	exon_number "1"
12	lncRNA	exon	64153006	64153103	.	+	.	gene_id "lnc-bbug03985"	transcript_id "lnc-bbut00009126"	exon_number "2"
12	lncRNA	exon	64156123	64156875	.	+	.	gene_id "lnc-bbug03985"	transcript_id "lnc-bbut00009126"	exon_number "3"
12	lncRNA	exon	64588493	64589185	.	+	.	gene_id "lnc-bbug03987"	transcript_id "lnc-bbut00009135"	exon_number "1"
12	lncRNA	exon	64607460	64607657	.	+	.	gene_id "lnc-bbug03987"	transcript_id "lnc-bbut00009135"	exon_number "2"
12	lncRNA	exon	64645272	64645368	.	+	.	gene_id "lnc-bbug03987"	transcript_id "lnc-bbut00009135"	exon_number "3"
12	lncRNA	exon	64647210	64647362	.	+	.	gene_id "lnc-bbug03987"	transcript_id "lnc-bbut00009135"	exon_number "4"
12	lncRNA	exon	64678219	64678270	.	+	.	gene_id "lnc-bbug03987"	transcript_id "lnc-bbut00009135"	exon_number "5"
12	lncRNA	exon	64683978	64689356	.	+	.	gene_id "lnc-bbug03987"	transcript_id "lnc-bbut00009135"	exon_number "6"
12	lncRNA	exon	64588493	64589185	.	+	.	gene_id "lnc-bbug03987"	transcript_id "lnc-bbut00009134"	exon_number "1"
12	lncRNA	exon	64645272	64645368	.	+	.	gene_id "lnc-bbug03987"	transcript_id "lnc-bbut00009134"	exon_number "2"
12	lncRNA	exon	64647210	64647362	.	+	.	gene_id "lnc-bbug03987"	transcript_id "lnc-bbut00009134"	exon_number "3"
12	lncRNA	exon	64678219	64678270	.	+	.	gene_id "lnc-bbug03987"	transcript_id "lnc-bbut00009134"	exon_number "4"
12	lncRNA	exon	64683978	64689356	.	+	.	gene_id "lnc-bbug03987"	transcript_id "lnc-bbut00009134"	exon_number "5"
12	lncRNA	exon	64596630	64597082	.	+	.	gene_id "lnc-bbug03987"	transcript_id "lnc-bbut00009137"	exon_number "1"
12	lncRNA	exon	64607460	64607657	.	+	.	gene_id "lnc-bbug03987"	transcript_id "lnc-bbut00009137"	exon_number "2"
12	lncRNA	exon	64639954	64640001	.	+	.	gene_id "lnc-bbug03987"	transcript_id "lnc-bbut00009137"	exon_number "3"
12	lncRNA	exon	64645272	64645368	.	+	.	gene_id "lnc-bbug03987"	transcript_id "lnc-bbut00009137"	exon_number "4"
12	lncRNA	exon	64647210	64647362	.	+	.	gene_id "lnc-bbug03987"	transcript_id "lnc-bbut00009137"	exon_number "5"
12	lncRNA	exon	64678219	64678270	.	+	.	gene_id "lnc-bbug03987"	transcript_id "lnc-bbut00009137"	exon_number "6"

12	lncRNA	exon	64683978	64689356	.	+	.	gene_id "lnc-bbug03987"	transcript_id "lnc-bbut00009137"	exon_number "7"
12	lncRNA	exon	64596630	64597082	.	+	.	gene_id "lnc-bbug03987"	transcript_id "lnc-bbut00009136"	exon_number "1"
12	lncRNA	exon	64607460	64607657	.	+	.	gene_id "lnc-bbug03987"	transcript_id "lnc-bbut00009136"	exon_number "2"
12	lncRNA	exon	64645272	64645368	.	+	.	gene_id "lnc-bbug03987"	transcript_id "lnc-bbut00009136"	exon_number "3"
12	lncRNA	exon	64647210	64647362	.	+	.	gene_id "lnc-bbug03987"	transcript_id "lnc-bbut00009136"	exon_number "4"
12	lncRNA	exon	64678219	64678270	.	+	.	gene_id "lnc-bbug03987"	transcript_id "lnc-bbut00009136"	exon_number "5"
12	lncRNA	exon	64683978	64689356	.	+	.	gene_id "lnc-bbug03987"	transcript_id "lnc-bbut00009136"	exon_number "6"
12	lncRNA	exon	64930425	64930908	.	+	.	gene_id "lnc-bbug03988"	transcript_id "lnc-bbut00009138"	exon_number "1"
12	lncRNA	exon	64951142	64951167	.	+	.	gene_id "lnc-bbug03988"	transcript_id "lnc-bbut00009138"	exon_number "2"
12	lncRNA	exon	64952252	64952311	.	+	.	gene_id "lnc-bbug03988"	transcript_id "lnc-bbut00009138"	exon_number "3"
12	lncRNA	exon	64953577	64953625	.	+	.	gene_id "lnc-bbug03988"	transcript_id "lnc-bbut00009138"	exon_number "4"
12	lncRNA	exon	64954427	64954601	.	+	.	gene_id "lnc-bbug03988"	transcript_id "lnc-bbut00009138"	exon_number "5"
12	lncRNA	exon	66326396	66326490	.	+	.	gene_id "lnc-bbug03991"	transcript_id "lnc-bbut00009143"	exon_number "1"
12	lncRNA	exon	66351824	66352022	.	+	.	gene_id "lnc-bbug03991"	transcript_id "lnc-bbut00009143"	exon_number "2"
12	lncRNA	exon	66362289	66362383	.	+	.	gene_id "lnc-bbug03991"	transcript_id "lnc-bbut00009143"	exon_number "3"
12	lncRNA	exon	66362600	66363430	.	+	.	gene_id "lnc-bbug03991"	transcript_id "lnc-bbut00009143"	exon_number "4"
12	lncRNA	exon	66340578	66342493	.	+	.	gene_id "lnc-bbug03991"	transcript_id "lnc-bbut00009145"	exon_number "1"
12	lncRNA	exon	66351824	66352022	.	+	.	gene_id "lnc-bbug03991"	transcript_id "lnc-bbut00009145"	exon_number "2"
12	lncRNA	exon	66362289	66362383	.	+	.	gene_id "lnc-bbug03991"	transcript_id "lnc-bbut00009145"	exon_number "3"
12	lncRNA	exon	66362600	66363430	.	+	.	gene_id "lnc-bbug03991"	transcript_id "lnc-bbut00009145"	exon_number "4"
12	lncRNA	exon	66327610	66327849	.	+	.	gene_id "lnc-bbug03992"	transcript_id "lnc-bbut00009144"	exon_number "1"
12	lncRNA	exon	66328673	66333156	.	+	.	gene_id "lnc-bbug03992"	transcript_id "lnc-bbut00009144"	exon_number "2"
12	lncRNA	exon	68213407	68216012	.	+	.	gene_id "lnc-bbug04005"	transcript_id "lnc-bbut00009172"	exon_number "1"
12	lncRNA	exon	70555237	70556010	.	+	.	gene_id "lnc-bbug04014"	transcript_id "lnc-bbut00009183"	exon_number "1"
12	lncRNA	exon	70800546	70800639	.	+	.	gene_id "lnc-bbug04016"	transcript_id "lnc-bbut00009185"	exon_number "1"
12	lncRNA	exon	70804367	70804488	.	+	.	gene_id "lnc-bbug04016"	transcript_id "lnc-bbut00009185"	exon_number "2"
12	lncRNA	exon	70804624	70804829	.	+	.	gene_id "lnc-bbug04016"	transcript_id "lnc-bbut00009185"	exon_number "3"
12	lncRNA	exon	70805823	70805948	.	+	.	gene_id "lnc-bbug04016"	transcript_id "lnc-bbut00009185"	exon_number "4"
12	lncRNA	exon	72241472	72241629	.	+	.	gene_id "lnc-bbug04033"	transcript_id "lnc-bbut00009234"	exon_number "1"
12	lncRNA	exon	72241752	72241889	.	+	.	gene_id "lnc-bbug04033"	transcript_id "lnc-bbut00009234"	exon_number "2"
12	lncRNA	exon	72242526	72242761	.	+	.	gene_id "lnc-bbug04033"	transcript_id "lnc-bbut00009234"	exon_number "3"
12	lncRNA	exon	74643362	74643461	.	+	.	gene_id "lnc-bbug04047"	transcript_id "lnc-bbut00009284"	exon_number "1"
12	lncRNA	exon	74644584	74644713	.	+	.	gene_id "lnc-bbug04047"	transcript_id "lnc-bbut00009284"	exon_number "2"
12	lncRNA	exon	74645273	74645410	.	+	.	gene_id "lnc-bbug04047"	transcript_id "lnc-bbut00009284"	exon_number "3"
12	lncRNA	exon	74646210	74646315	.	+	.	gene_id "lnc-bbug04047"	transcript_id "lnc-bbut00009284"	exon_number "4"
12	lncRNA	exon	74647190	74648073	.	+	.	gene_id "lnc-bbug04047"	transcript_id "lnc-bbut00009284"	exon_number "5"
12	lncRNA	exon	74643612	74644045	.	+	.	gene_id "lnc-bbug04047"	transcript_id "lnc-bbut00009285"	exon_number "1"
12	lncRNA	exon	74644584	74644713	.	+	.	gene_id "lnc-bbug04047"	transcript_id "lnc-bbut00009285"	exon_number "2"
12	lncRNA	exon	74645273	74645410	.	+	.	gene_id "lnc-bbug04047"	transcript_id "lnc-bbut00009285"	exon_number "3"
12	lncRNA	exon	74646210	74646315	.	+	.	gene_id "lnc-bbug04047"	transcript_id "lnc-bbut00009285"	exon_number "4"
12	lncRNA	exon	74647190	74648073	.	+	.	gene_id "lnc-bbug04047"	transcript_id "lnc-bbut00009285"	exon_number "5"
12	lncRNA	exon	75253286	75253588	.	+	.	gene_id "lnc-bbug04053"	transcript_id "lnc-bbut00009301"	exon_number "1"
12	lncRNA	exon	75297565	75297972	.	+	.	gene_id "lnc-bbug04053"	transcript_id "lnc-bbut00009301"	exon_number "2"
12	lncRNA	exon	75421052	75421140	.	+	.	gene_id "lnc-bbug04053"	transcript_id "lnc-bbut00009301"	exon_number "3"
12	lncRNA	exon	75521076	75521109	.	+	.	gene_id "lnc-bbug04053"	transcript_id "lnc-bbut00009301"	exon_number "4"
12	lncRNA	exon	75525482	75525584	.	+	.	gene_id "lnc-bbug04053"	transcript_id "lnc-bbut00009301"	exon_number "5"
12	lncRNA	exon	75619668	75619822	.	+	.	gene_id "lnc-bbug04053"	transcript_id "lnc-bbut00009301"	exon_number "6"
12	lncRNA	exon	75646272	75646464	.	+	.	gene_id "lnc-bbug04053"	transcript_id "lnc-bbut00009301"	exon_number "7"
12	lncRNA	exon	75650413	75650528	.	+	.	gene_id "lnc-bbug04053"	transcript_id "lnc-bbut00009301"	exon_number "8"
12	lncRNA	exon	75650750	75650801	.	+	.	gene_id "lnc-bbug04053"	transcript_id "lnc-bbut00009301"	exon_number "9"
12	lncRNA	exon	75253286	75253588	.	+	.	gene_id "lnc-bbug04053"	transcript_id "lnc-bbut00009300"	exon_number "1"
12	lncRNA	exon	75297565	75297972	.	+	.	gene_id "lnc-bbug04053"	transcript_id "lnc-bbut00009300"	exon_number "2"

12	lncRNA	exon	75421052	75421140	.	+	.	gene_id "lnc-bbug04053"	transcript_id "lnc-bbut00009300"	exon_number "3"
12	lncRNA	exon	75525482	75525584	.	+	.	gene_id "lnc-bbug04053"	transcript_id "lnc-bbut00009300"	exon_number "4"
12	lncRNA	exon	75619668	75619822	.	+	.	gene_id "lnc-bbug04053"	transcript_id "lnc-bbut00009300"	exon_number "5"
12	lncRNA	exon	75646272	75646464	.	+	.	gene_id "lnc-bbug04053"	transcript_id "lnc-bbut00009300"	exon_number "6"
12	lncRNA	exon	75650413	75650528	.	+	.	gene_id "lnc-bbug04053"	transcript_id "lnc-bbut00009300"	exon_number "7"
12	lncRNA	exon	75650750	75650801	.	+	.	gene_id "lnc-bbug04053"	transcript_id "lnc-bbut00009300"	exon_number "8"
12	lncRNA	exon	75253287	75253588	.	+	.	gene_id "lnc-bbug04053"	transcript_id "lnc-bbut00009302"	exon_number "1"
12	lncRNA	exon	75297565	75297972	.	+	.	gene_id "lnc-bbug04053"	transcript_id "lnc-bbut00009302"	exon_number "2"
12	lncRNA	exon	75421052	75421140	.	+	.	gene_id "lnc-bbug04053"	transcript_id "lnc-bbut00009302"	exon_number "3"
12	lncRNA	exon	75521076	75521109	.	+	.	gene_id "lnc-bbug04053"	transcript_id "lnc-bbut00009302"	exon_number "4"
12	lncRNA	exon	75525482	75525584	.	+	.	gene_id "lnc-bbug04053"	transcript_id "lnc-bbut00009302"	exon_number "5"
12	lncRNA	exon	75646272	75646464	.	+	.	gene_id "lnc-bbug04053"	transcript_id "lnc-bbut00009302"	exon_number "6"
12	lncRNA	exon	75650413	75650528	.	+	.	gene_id "lnc-bbug04053"	transcript_id "lnc-bbut00009302"	exon_number "7"
12	lncRNA	exon	75650750	75650801	.	+	.	gene_id "lnc-bbug04053"	transcript_id "lnc-bbut00009302"	exon_number "8"
12	lncRNA	exon	75253292	75253588	.	+	.	gene_id "lnc-bbug04053"	transcript_id "lnc-bbut00009303"	exon_number "1"
12	lncRNA	exon	75297565	75297972	.	+	.	gene_id "lnc-bbug04053"	transcript_id "lnc-bbut00009303"	exon_number "2"
12	lncRNA	exon	75421052	75421140	.	+	.	gene_id "lnc-bbug04053"	transcript_id "lnc-bbut00009303"	exon_number "3"
12	lncRNA	exon	75521076	75521109	.	+	.	gene_id "lnc-bbug04053"	transcript_id "lnc-bbut00009303"	exon_number "4"
12	lncRNA	exon	75525482	75525584	.	+	.	gene_id "lnc-bbug04053"	transcript_id "lnc-bbut00009303"	exon_number "5"
12	lncRNA	exon	75561019	75561722	.	+	.	gene_id "lnc-bbug04053"	transcript_id "lnc-bbut00009303"	exon_number "6"
12	lncRNA	exon	75704721	75706003	.	+	.	gene_id "lnc-bbug04054"	transcript_id "lnc-bbut00009304"	exon_number "1"
12	lncRNA	exon	75716447	75716516	.	+	.	gene_id "lnc-bbug04054"	transcript_id "lnc-bbut00009304"	exon_number "2"
12	lncRNA	exon	75718725	75718778	.	+	.	gene_id "lnc-bbug04054"	transcript_id "lnc-bbut00009304"	exon_number "3"
12	lncRNA	exon	75726885	75726976	.	+	.	gene_id "lnc-bbug04054"	transcript_id "lnc-bbut00009304"	exon_number "4"
12	lncRNA	exon	75728749	75729078	.	+	.	gene_id "lnc-bbug04054"	transcript_id "lnc-bbut00009304"	exon_number "5"
12	lncRNA	exon	75732192	75733477	.	+	.	gene_id "lnc-bbug04055"	transcript_id "lnc-bbut00009305"	exon_number "1"
12	lncRNA	exon	75733921	75734304	.	+	.	gene_id "lnc-bbug04055"	transcript_id "lnc-bbut00009305"	exon_number "2"
12	lncRNA	exon	77804779	77804920	.	+	.	gene_id "lnc-bbug04058"	transcript_id "lnc-bbut00009309"	exon_number "1"
12	lncRNA	exon	77809378	77809533	.	+	.	gene_id "lnc-bbug04058"	transcript_id "lnc-bbut00009309"	exon_number "2"
12	lncRNA	exon	77820173	77820316	.	+	.	gene_id "lnc-bbug04058"	transcript_id "lnc-bbut00009309"	exon_number "3"
12	lncRNA	exon	77834521	77834690	.	+	.	gene_id "lnc-bbug04058"	transcript_id "lnc-bbut00009309"	exon_number "4"
12	lncRNA	exon	77877324	77877406	.	+	.	gene_id "lnc-bbug04058"	transcript_id "lnc-bbut00009309"	exon_number "5"
12	lncRNA	exon	77890855	77894524	.	+	.	gene_id "lnc-bbug04058"	transcript_id "lnc-bbut00009309"	exon_number "6"
12	lncRNA	exon	78568128	78568135	.	+	.	gene_id "lnc-bbug04065"	transcript_id "lnc-bbut00009336"	exon_number "1"
12	lncRNA	exon	78569310	78570993	.	+	.	gene_id "lnc-bbug04065"	transcript_id "lnc-bbut00009336"	exon_number "2"
12	lncRNA	exon	78736724	78736941	.	+	.	gene_id "lnc-bbug04067"	transcript_id "lnc-bbut00009338"	exon_number "1"
12	lncRNA	exon	78737478	78737877	.	+	.	gene_id "lnc-bbug04067"	transcript_id "lnc-bbut00009338"	exon_number "2"
12	lncRNA	exon	78738336	78738670	.	+	.	gene_id "lnc-bbug04067"	transcript_id "lnc-bbut00009338"	exon_number "3"
12	lncRNA	exon	80098728	80098920	.	+	.	gene_id "lnc-bbug04071"	transcript_id "lnc-bbut00009343"	exon_number "1"
12	lncRNA	exon	80150548	80151018	.	+	.	gene_id "lnc-bbug04071"	transcript_id "lnc-bbut00009343"	exon_number "2"
12	lncRNA	exon	80151237	80151461	.	+	.	gene_id "lnc-bbug04071"	transcript_id "lnc-bbut00009343"	exon_number "3"
12	lncRNA	exon	81027292	81027381	.	+	.	gene_id "lnc-bbug04074"	transcript_id "lnc-bbut00009350"	exon_number "1"
12	lncRNA	exon	81067071	81067146	.	+	.	gene_id "lnc-bbug04074"	transcript_id "lnc-bbut00009350"	exon_number "2"
12	lncRNA	exon	81068463	81068508	.	+	.	gene_id "lnc-bbug04074"	transcript_id "lnc-bbut00009350"	exon_number "3"
12	lncRNA	exon	84986743	84986848	.	+	.	gene_id "lnc-bbug04078"	transcript_id "lnc-bbut00009358"	exon_number "1"
12	lncRNA	exon	84987583	84987692	.	+	.	gene_id "lnc-bbug04078"	transcript_id "lnc-bbut00009358"	exon_number "2"
12	lncRNA	exon	84996379	84996514	.	+	.	gene_id "lnc-bbug04078"	transcript_id "lnc-bbut00009358"	exon_number "3"
12	lncRNA	exon	85038767	85038826	.	+	.	gene_id "lnc-bbug04079"	transcript_id "lnc-bbut00009359"	exon_number "1"
12	lncRNA	exon	85039352	85039500	.	+	.	gene_id "lnc-bbug04079"	transcript_id "lnc-bbut00009359"	exon_number "2"
12	lncRNA	exon	85040154	85041303	.	+	.	gene_id "lnc-bbug04079"	transcript_id "lnc-bbut00009359"	exon_number "3"
12	lncRNA	exon	85038767	85038826	.	+	.	gene_id "lnc-bbug04079"	transcript_id "lnc-bbut00009360"	exon_number "1"
12	lncRNA	exon	85039352	85039500	.	+	.	gene_id "lnc-bbug04079"	transcript_id "lnc-bbut00009360"	exon_number "2"

12	lncRNA	exon	85040154	85040380	.	+	.	gene_id "lnc-bbug04079"	transcript_id "lnc-bbut00009360"	exon_number "3"
12	lncRNA	exon	85088385	85089290	.	+	.	gene_id "lnc-bbug04079"	transcript_id "lnc-bbut00009360"	exon_number "4"
12	lncRNA	exon	85997161	85998372	.	+	.	gene_id "lnc-bbug04083"	transcript_id "lnc-bbut00009365"	exon_number "1"
12	lncRNA	exon	86014866	86015219	.	+	.	gene_id "lnc-bbug04083"	transcript_id "lnc-bbut00009365"	exon_number "2"
12	lncRNA	exon	86501185	86501505	.	+	.	gene_id "lnc-bbug04087"	transcript_id "lnc-bbut00009375"	exon_number "1"
12	lncRNA	exon	86501894	86502052	.	+	.	gene_id "lnc-bbug04087"	transcript_id "lnc-bbut00009375"	exon_number "2"
12	lncRNA	exon	86505767	86506103	.	+	.	gene_id "lnc-bbug04087"	transcript_id "lnc-bbut00009375"	exon_number "3"
12	lncRNA	exon	87054800	87056276	.	+	.	gene_id "lnc-bbug04090"	transcript_id "lnc-bbut00009381"	exon_number "1"
12	lncRNA	exon	87057032	87057122	.	+	.	gene_id "lnc-bbug04090"	transcript_id "lnc-bbut00009381"	exon_number "2"
12	lncRNA	exon	87061255	87061579	.	+	.	gene_id "lnc-bbug04090"	transcript_id "lnc-bbut00009381"	exon_number "3"
12	lncRNA	exon	87062616	87062667	.	+	.	gene_id "lnc-bbug04090"	transcript_id "lnc-bbut00009381"	exon_number "4"
12	lncRNA	exon	87227872	87229279	.	+	.	gene_id "lnc-bbug04092"	transcript_id "lnc-bbut00009383"	exon_number "1"
12	lncRNA	exon	88172774	88173101	.	+	.	gene_id "lnc-bbug04098"	transcript_id "lnc-bbut00009410"	exon_number "1"
12	lncRNA	exon	88173188	88173401	.	+	.	gene_id "lnc-bbug04098"	transcript_id "lnc-bbut00009410"	exon_number "2"
12	lncRNA	exon	88189507	88190124	.	+	.	gene_id "lnc-bbug04098"	transcript_id "lnc-bbut00009410"	exon_number "3"
12	lncRNA	exon	88172774	88173081	.	+	.	gene_id "lnc-bbug04098"	transcript_id "lnc-bbut00009409"	exon_number "1"
12	lncRNA	exon	88173188	88173401	.	+	.	gene_id "lnc-bbug04098"	transcript_id "lnc-bbut00009409"	exon_number "2"
12	lncRNA	exon	88189507	88190124	.	+	.	gene_id "lnc-bbug04098"	transcript_id "lnc-bbut00009409"	exon_number "3"
12	lncRNA	exon	88289130	88291020	.	+	.	gene_id "lnc-bbug04099"	transcript_id "lnc-bbut00009411"	exon_number "1"
12	lncRNA	exon	88291564	88291653	.	+	.	gene_id "lnc-bbug04099"	transcript_id "lnc-bbut00009411"	exon_number "2"
12	lncRNA	exon	88372171	88372315	.	+	.	gene_id "lnc-bbug04099"	transcript_id "lnc-bbut00009411"	exon_number "3"
12	lncRNA	exon	88372683	88372757	.	+	.	gene_id "lnc-bbug04099"	transcript_id "lnc-bbut00009411"	exon_number "4"
12	lncRNA	exon	88373219	88373420	.	+	.	gene_id "lnc-bbug04099"	transcript_id "lnc-bbut00009411"	exon_number "5"
12	lncRNA	exon	88375675	88377777	.	+	.	gene_id "lnc-bbug04099"	transcript_id "lnc-bbut00009411"	exon_number "6"
12	lncRNA	exon	88368730	88369390	.	+	.	gene_id "lnc-bbug04099"	transcript_id "lnc-bbut00009412"	exon_number "1"
12	lncRNA	exon	88372171	88372315	.	+	.	gene_id "lnc-bbug04099"	transcript_id "lnc-bbut00009412"	exon_number "2"
12	lncRNA	exon	88372683	88372757	.	+	.	gene_id "lnc-bbug04099"	transcript_id "lnc-bbut00009412"	exon_number "3"
12	lncRNA	exon	88373219	88373420	.	+	.	gene_id "lnc-bbug04099"	transcript_id "lnc-bbut00009412"	exon_number "4"
12	lncRNA	exon	88375675	88377777	.	+	.	gene_id "lnc-bbug04099"	transcript_id "lnc-bbut00009412"	exon_number "5"
12	lncRNA	exon	88369401	88371764	.	+	.	gene_id "lnc-bbug04099"	transcript_id "lnc-bbut00009413"	exon_number "1"
12	lncRNA	exon	88372171	88372315	.	+	.	gene_id "lnc-bbug04099"	transcript_id "lnc-bbut00009413"	exon_number "2"
12	lncRNA	exon	88372683	88372757	.	+	.	gene_id "lnc-bbug04099"	transcript_id "lnc-bbut00009413"	exon_number "3"
12	lncRNA	exon	88373219	88373420	.	+	.	gene_id "lnc-bbug04099"	transcript_id "lnc-bbut00009413"	exon_number "4"
12	lncRNA	exon	88375675	88377777	.	+	.	gene_id "lnc-bbug04099"	transcript_id "lnc-bbut00009413"	exon_number "5"
12	lncRNA	exon	88395679	88396639	.	+	.	gene_id "lnc-bbug04100"	transcript_id "lnc-bbut00009414"	exon_number "1"
12	lncRNA	exon	88402539	88403646	.	+	.	gene_id "lnc-bbug04100"	transcript_id "lnc-bbut00009414"	exon_number "2"
12	lncRNA	exon	88406713	88406828	.	+	.	gene_id "lnc-bbug04100"	transcript_id "lnc-bbut00009414"	exon_number "3"
12	lncRNA	exon	88407045	88407796	.	+	.	gene_id "lnc-bbug04100"	transcript_id "lnc-bbut00009414"	exon_number "4"
12	lncRNA	exon	88518920	88519137	.	+	.	gene_id "lnc-bbug04101"	transcript_id "lnc-bbut00009415"	exon_number "1"
12	lncRNA	exon	88525786	88525953	.	+	.	gene_id "lnc-bbug04101"	transcript_id "lnc-bbut00009415"	exon_number "2"
12	lncRNA	exon	88537582	88537671	.	+	.	gene_id "lnc-bbug04101"	transcript_id "lnc-bbut00009415"	exon_number "3"
12	lncRNA	exon	88537845	88537955	.	+	.	gene_id "lnc-bbug04101"	transcript_id "lnc-bbut00009415"	exon_number "4"
12	lncRNA	exon	88612569	88612653	.	+	.	gene_id "lnc-bbug04101"	transcript_id "lnc-bbut00009415"	exon_number "5"
12	lncRNA	exon	88612739	88612835	.	+	.	gene_id "lnc-bbug04101"	transcript_id "lnc-bbut00009415"	exon_number "6"
12	lncRNA	exon	88614757	88614841	.	+	.	gene_id "lnc-bbug04101"	transcript_id "lnc-bbut00009415"	exon_number "7"
12	lncRNA	exon	88616299	88616425	.	+	.	gene_id "lnc-bbug04101"	transcript_id "lnc-bbut00009415"	exon_number "8"
12	lncRNA	exon	88616547	88616723	.	+	.	gene_id "lnc-bbug04101"	transcript_id "lnc-bbut00009415"	exon_number "9"
12	lncRNA	exon	88518921	88519137	.	+	.	gene_id "lnc-bbug04101"	transcript_id "lnc-bbut00009416"	exon_number "1"
12	lncRNA	exon	88537582	88537671	.	+	.	gene_id "lnc-bbug04101"	transcript_id "lnc-bbut00009416"	exon_number "2"
12	lncRNA	exon	88537845	88537955	.	+	.	gene_id "lnc-bbug04101"	transcript_id "lnc-bbut00009416"	exon_number "3"
12	lncRNA	exon	88612569	88612653	.	+	.	gene_id "lnc-bbug04101"	transcript_id "lnc-bbut00009416"	exon_number "4"
12	lncRNA	exon	88612739	88612835	.	+	.	gene_id "lnc-bbug04101"	transcript_id "lnc-bbut00009416"	exon_number "5"

12	lncRNA	exon	88614757	88614841	.	+	.	gene_id "lnc-bbug04101"	transcript_id "lnc-bbut00009416"	exon_number "6"
12	lncRNA	exon	88616299	88616425	.	+	.	gene_id "lnc-bbug04101"	transcript_id "lnc-bbut00009416"	exon_number "7"
12	lncRNA	exon	88616547	88616723	.	+	.	gene_id "lnc-bbug04101"	transcript_id "lnc-bbut00009416"	exon_number "8"
12	lncRNA	exon	88518923	88519137	.	+	.	gene_id "lnc-bbug04101"	transcript_id "lnc-bbut00009417"	exon_number "1"
12	lncRNA	exon	88525786	88525953	.	+	.	gene_id "lnc-bbug04101"	transcript_id "lnc-bbut00009417"	exon_number "2"
12	lncRNA	exon	88537582	88537671	.	+	.	gene_id "lnc-bbug04101"	transcript_id "lnc-bbut00009417"	exon_number "3"
12	lncRNA	exon	88537845	88537955	.	+	.	gene_id "lnc-bbug04101"	transcript_id "lnc-bbut00009417"	exon_number "4"
12	lncRNA	exon	88612569	88612653	.	+	.	gene_id "lnc-bbug04101"	transcript_id "lnc-bbut00009417"	exon_number "5"
12	lncRNA	exon	88614757	88614841	.	+	.	gene_id "lnc-bbug04101"	transcript_id "lnc-bbut00009417"	exon_number "6"
12	lncRNA	exon	88616299	88616425	.	+	.	gene_id "lnc-bbug04101"	transcript_id "lnc-bbut00009417"	exon_number "7"
12	lncRNA	exon	88616547	88616723	.	+	.	gene_id "lnc-bbug04101"	transcript_id "lnc-bbut00009417"	exon_number "8"
12	lncRNA	exon	88583673	88584788	.	+	.	gene_id "lnc-bbug04102"	transcript_id "lnc-bbut00009418"	exon_number "1"
12	lncRNA	exon	88586410	88586764	.	+	.	gene_id "lnc-bbug04102"	transcript_id "lnc-bbut00009418"	exon_number "2"
12	lncRNA	exon	88799997	88800105	.	+	.	gene_id "lnc-bbug04103"	transcript_id "lnc-bbut00009419"	exon_number "1"
12	lncRNA	exon	88842399	88842492	.	+	.	gene_id "lnc-bbug04103"	transcript_id "lnc-bbut00009419"	exon_number "2"
12	lncRNA	exon	88854451	88854548	.	+	.	gene_id "lnc-bbug04103"	transcript_id "lnc-bbut00009419"	exon_number "3"
12	lncRNA	exon	88857399	88857455	.	+	.	gene_id "lnc-bbug04103"	transcript_id "lnc-bbut00009419"	exon_number "4"
12	lncRNA	exon	88860420	88860992	.	+	.	gene_id "lnc-bbug04103"	transcript_id "lnc-bbut00009419"	exon_number "5"
12	lncRNA	exon	88837752	88837925	.	+	.	gene_id "lnc-bbug04103"	transcript_id "lnc-bbut00009420"	exon_number "1"
12	lncRNA	exon	88842399	88842492	.	+	.	gene_id "lnc-bbug04103"	transcript_id "lnc-bbut00009420"	exon_number "2"
12	lncRNA	exon	88854451	88854548	.	+	.	gene_id "lnc-bbug04103"	transcript_id "lnc-bbut00009420"	exon_number "3"
12	lncRNA	exon	88857399	88857455	.	+	.	gene_id "lnc-bbug04103"	transcript_id "lnc-bbut00009420"	exon_number "4"
12	lncRNA	exon	88860420	88860992	.	+	.	gene_id "lnc-bbug04103"	transcript_id "lnc-bbut00009420"	exon_number "5"
12	lncRNA	exon	89186798	89186998	.	+	.	gene_id "lnc-bbug04105"	transcript_id "lnc-bbut00009422"	exon_number "1"
12	lncRNA	exon	89187126	89187364	.	+	.	gene_id "lnc-bbug04105"	transcript_id "lnc-bbut00009422"	exon_number "2"
12	lncRNA	exon	89189784	89190239	.	+	.	gene_id "lnc-bbug04105"	transcript_id "lnc-bbut00009422"	exon_number "3"
12	lncRNA	exon	89186807	89186998	.	+	.	gene_id "lnc-bbug04105"	transcript_id "lnc-bbut00009423"	exon_number "1"
12	lncRNA	exon	89187293	89187364	.	+	.	gene_id "lnc-bbug04105"	transcript_id "lnc-bbut00009423"	exon_number "2"
12	lncRNA	exon	89189784	89190239	.	+	.	gene_id "lnc-bbug04105"	transcript_id "lnc-bbut00009423"	exon_number "3"
12	lncRNA	exon	90100680	90100999	.	+	.	gene_id "lnc-bbug04107"	transcript_id "lnc-bbut00009425"	exon_number "1"
12	lncRNA	exon	91963717	91965197	.	+	.	gene_id "lnc-bbug04108"	transcript_id "lnc-bbut00009426"	exon_number "1"
12	lncRNA	exon	91965339	91965678	.	+	.	gene_id "lnc-bbug04108"	transcript_id "lnc-bbut00009426"	exon_number "2"
12	lncRNA	exon	92432658	92432984	.	+	.	gene_id "lnc-bbug04110"	transcript_id "lnc-bbut00009428"	exon_number "1"
12	lncRNA	exon	92436910	92437069	.	+	.	gene_id "lnc-bbug04110"	transcript_id "lnc-bbut00009428"	exon_number "2"
12	lncRNA	exon	92438355	92438466	.	+	.	gene_id "lnc-bbug04110"	transcript_id "lnc-bbut00009428"	exon_number "3"
12	lncRNA	exon	92844157	92844518	.	+	.	gene_id "lnc-bbug04114"	transcript_id "lnc-bbut00009438"	exon_number "1"
12	lncRNA	exon	92844711	92844930	.	+	.	gene_id "lnc-bbug04114"	transcript_id "lnc-bbut00009438"	exon_number "2"
12	lncRNA	exon	92845320	92845640	.	+	.	gene_id "lnc-bbug04114"	transcript_id "lnc-bbut00009438"	exon_number "3"
12	lncRNA	exon	92959731	92960665	.	+	.	gene_id "lnc-bbug04116"	transcript_id "lnc-bbut00009440"	exon_number "1"
12	lncRNA	exon	95209494	95209813	.	+	.	gene_id "lnc-bbug04144"	transcript_id "lnc-bbut00009478"	exon_number "1"
12	lncRNA	exon	95242720	95242825	.	+	.	gene_id "lnc-bbug04144"	transcript_id "lnc-bbut00009478"	exon_number "2"
12	lncRNA	exon	95244407	95244562	.	+	.	gene_id "lnc-bbug04144"	transcript_id "lnc-bbut00009478"	exon_number "3"
12	lncRNA	exon	95209494	95209813	.	+	.	gene_id "lnc-bbug04144"	transcript_id "lnc-bbut00009479"	exon_number "1"
12	lncRNA	exon	95242720	95242825	.	+	.	gene_id "lnc-bbug04144"	transcript_id "lnc-bbut00009479"	exon_number "2"
12	lncRNA	exon	95270698	95270751	.	+	.	gene_id "lnc-bbug04144"	transcript_id "lnc-bbut00009479"	exon_number "3"
12	lncRNA	exon	95272624	95272725	.	+	.	gene_id "lnc-bbug04144"	transcript_id "lnc-bbut00009479"	exon_number "4"
12	lncRNA	exon	98184775	98184824	.	+	.	gene_id "lnc-bbug04166"	transcript_id "lnc-bbut00009565"	exon_number "1"
12	lncRNA	exon	98185515	98185626	.	+	.	gene_id "lnc-bbug04166"	transcript_id "lnc-bbut00009565"	exon_number "2"
12	lncRNA	exon	98187189	98187237	.	+	.	gene_id "lnc-bbug04166"	transcript_id "lnc-bbut00009565"	exon_number "3"
12	lncRNA	exon	98303994	98304502	.	+	.	gene_id "lnc-bbug04168"	transcript_id "lnc-bbut00009572"	exon_number "1"
12	lncRNA	exon	98305507	98305590	.	+	.	gene_id "lnc-bbug04168"	transcript_id "lnc-bbut00009572"	exon_number "2"
12	lncRNA	exon	98305678	98305933	.	+	.	gene_id "lnc-bbug04168"	transcript_id "lnc-bbut00009572"	exon_number "3"

12	lncRNA	exon	98416059	98416202	.	+	.	gene_id "lnc-bbug04172"	transcript_id "lnc-bbut00009599"	exon_number "1"
12	lncRNA	exon	98416493	98416588	.	+	.	gene_id "lnc-bbug04172"	transcript_id "lnc-bbut00009599"	exon_number "2"
12	lncRNA	exon	98418676	98418797	.	+	.	gene_id "lnc-bbug04172"	transcript_id "lnc-bbut00009599"	exon_number "3"
12	lncRNA	exon	98419870	98419964	.	+	.	gene_id "lnc-bbug04172"	transcript_id "lnc-bbut00009599"	exon_number "4"
12	lncRNA	exon	98420347	98420390	.	+	.	gene_id "lnc-bbug04172"	transcript_id "lnc-bbut00009599"	exon_number "5"
12	lncRNA	exon	98420956	98420993	.	+	.	gene_id "lnc-bbug04172"	transcript_id "lnc-bbut00009599"	exon_number "6"
12	lncRNA	exon	98422028	98422336	.	+	.	gene_id "lnc-bbug04172"	transcript_id "lnc-bbut00009599"	exon_number "7"
12	lncRNA	exon	98503947	98504068	.	+	.	gene_id "lnc-bbug04175"	transcript_id "lnc-bbut00009605"	exon_number "1"
12	lncRNA	exon	98508423	98508493	.	+	.	gene_id "lnc-bbug04175"	transcript_id "lnc-bbut00009605"	exon_number "2"
12	lncRNA	exon	98516680	98516761	.	+	.	gene_id "lnc-bbug04175"	transcript_id "lnc-bbut00009605"	exon_number "3"
12	lncRNA	exon	98518262	98518310	.	+	.	gene_id "lnc-bbug04175"	transcript_id "lnc-bbut00009605"	exon_number "4"
12	lncRNA	exon	98518680	98520401	.	+	.	gene_id "lnc-bbug04175"	transcript_id "lnc-bbut00009605"	exon_number "5"
12	lncRNA	exon	98504083	98504282	.	+	.	gene_id "lnc-bbug04175"	transcript_id "lnc-bbut00009606"	exon_number "1"
12	lncRNA	exon	98508423	98508493	.	+	.	gene_id "lnc-bbug04175"	transcript_id "lnc-bbut00009606"	exon_number "2"
12	lncRNA	exon	98516680	98516761	.	+	.	gene_id "lnc-bbug04175"	transcript_id "lnc-bbut00009606"	exon_number "3"
12	lncRNA	exon	98518262	98518310	.	+	.	gene_id "lnc-bbug04175"	transcript_id "lnc-bbut00009606"	exon_number "4"
12	lncRNA	exon	98518680	98520401	.	+	.	gene_id "lnc-bbug04175"	transcript_id "lnc-bbut00009606"	exon_number "5"
12	lncRNA	exon	98557244	98558645	.	+	.	gene_id "lnc-bbug04178"	transcript_id "lnc-bbut00009611"	exon_number "1"
12	lncRNA	exon	99126145	99128499	.	+	.	gene_id "lnc-bbug04192"	transcript_id "lnc-bbut00009683"	exon_number "1"
12	lncRNA	exon	99129598	99129807	.	+	.	gene_id "lnc-bbug04192"	transcript_id "lnc-bbut00009683"	exon_number "2"
12	lncRNA	exon	99131039	99131088	.	+	.	gene_id "lnc-bbug04192"	transcript_id "lnc-bbut00009683"	exon_number "3"
12	lncRNA	exon	99255189	99255404	.	+	.	gene_id "lnc-bbug04193"	transcript_id "lnc-bbut00009684"	exon_number "1"
12	lncRNA	exon	99262517	99263838	.	+	.	gene_id "lnc-bbug04193"	transcript_id "lnc-bbut00009684"	exon_number "2"
12	lncRNA	exon	99264178	99264316	.	+	.	gene_id "lnc-bbug04193"	transcript_id "lnc-bbut00009684"	exon_number "3"
12	lncRNA	exon	99264407	99265708	.	+	.	gene_id "lnc-bbug04193"	transcript_id "lnc-bbut00009684"	exon_number "4"
12	lncRNA	exon	99305570	99305715	.	+	.	gene_id "lnc-bbug04194"	transcript_id "lnc-bbut00009686"	exon_number "1"
12	lncRNA	exon	99306928	99307346	.	+	.	gene_id "lnc-bbug04194"	transcript_id "lnc-bbut00009686"	exon_number "2"
12	lncRNA	exon	99399429	99401889	.	+	.	gene_id "lnc-bbug04196"	transcript_id "lnc-bbut00009689"	exon_number "1"
12	lncRNA	exon	99408511	99409201	.	+	.	gene_id "lnc-bbug04196"	transcript_id "lnc-bbut00009689"	exon_number "2"
12	lncRNA	exon	99724392	99725355	.	+	.	gene_id "lnc-bbug04201"	transcript_id "lnc-bbut00009705"	exon_number "1"
12	lncRNA	exon	99822614	99822713	.	+	.	gene_id "lnc-bbug04202"	transcript_id "lnc-bbut00009706"	exon_number "1"
12	lncRNA	exon	99825113	99825277	.	+	.	gene_id "lnc-bbug04202"	transcript_id "lnc-bbut00009706"	exon_number "2"
12	lncRNA	exon	99825611	99825971	.	+	.	gene_id "lnc-bbug04202"	transcript_id "lnc-bbut00009706"	exon_number "3"
12	lncRNA	exon	99824910	99825000	.	+	.	gene_id "lnc-bbug04202"	transcript_id "lnc-bbut00009707"	exon_number "1"
12	lncRNA	exon	99825113	99825277	.	+	.	gene_id "lnc-bbug04202"	transcript_id "lnc-bbut00009707"	exon_number "2"
12	lncRNA	exon	99825611	99825971	.	+	.	gene_id "lnc-bbug04202"	transcript_id "lnc-bbut00009707"	exon_number "3"
12	lncRNA	exon	99853041	99853253	.	+	.	gene_id "lnc-bbug04203"	transcript_id "lnc-bbut00009708"	exon_number "1"
12	lncRNA	exon	99855460	99855573	.	+	.	gene_id "lnc-bbug04203"	transcript_id "lnc-bbut00009708"	exon_number "2"
12	lncRNA	exon	99855985	99856371	.	+	.	gene_id "lnc-bbug04203"	transcript_id "lnc-bbut00009708"	exon_number "3"
12	lncRNA	exon	99942404	99943775	.	+	.	gene_id "lnc-bbug04205"	transcript_id "lnc-bbut00009711"	exon_number "1"
12	lncRNA	exon	99945646	99947001	.	+	.	gene_id "lnc-bbug04205"	transcript_id "lnc-bbut00009711"	exon_number "2"
12	lncRNA	exon	101202848	101203029	.	+	.	gene_id "lnc-bbug04220"	transcript_id "lnc-bbut00009757"	exon_number "1"
12	lncRNA	exon	101203423	101203855	.	+	.	gene_id "lnc-bbug04220"	transcript_id "lnc-bbut00009757"	exon_number "2"
12	lncRNA	exon	102679062	102679312	.	+	.	gene_id "lnc-bbug04229"	transcript_id "lnc-bbut00009785"	exon_number "1"
12	lncRNA	exon	102679770	102679957	.	+	.	gene_id "lnc-bbug04229"	transcript_id "lnc-bbut00009785"	exon_number "2"
12	lncRNA	exon	102680465	102680767	.	+	.	gene_id "lnc-bbug04229"	transcript_id "lnc-bbut00009785"	exon_number "3"
12	lncRNA	exon	102689176	102690581	.	+	.	gene_id "lnc-bbug04229"	transcript_id "lnc-bbut00009785"	exon_number "4"
12	lncRNA	exon	102679064	102679312	.	+	.	gene_id "lnc-bbug04229"	transcript_id "lnc-bbut00009786"	exon_number "1"
12	lncRNA	exon	102679770	102679957	.	+	.	gene_id "lnc-bbug04229"	transcript_id "lnc-bbut00009786"	exon_number "2"
12	lncRNA	exon	102680465	102681282	.	+	.	gene_id "lnc-bbug04229"	transcript_id "lnc-bbut00009786"	exon_number "3"
12	lncRNA	exon	103423052	103423969	.	+	.	gene_id "lnc-bbug04237"	transcript_id "lnc-bbut00009811"	exon_number "1"
12	lncRNA	exon	103424121	103424541	.	+	.	gene_id "lnc-bbug04237"	transcript_id "lnc-bbut00009811"	exon_number "2"

12	lncRNA	exon	103497878	103500535	.	+	.	gene_id "lnc-bbug04238"	transcript_id "lnc-bbut00009812"	exon_number "1"
12	lncRNA	exon	103500659	103501056	.	+	.	gene_id "lnc-bbug04238"	transcript_id "lnc-bbut00009812"	exon_number "2"
12	lncRNA	exon	103501372	103504525	.	+	.	gene_id "lnc-bbug04238"	transcript_id "lnc-bbut00009812"	exon_number "3"
12	lncRNA	exon	103497878	103501056	.	+	.	gene_id "lnc-bbug04238"	transcript_id "lnc-bbut00009814"	exon_number "1"
12	lncRNA	exon	103501372	103501514	.	+	.	gene_id "lnc-bbug04238"	transcript_id "lnc-bbut00009814"	exon_number "2"
12	lncRNA	exon	103506023	103506081	.	+	.	gene_id "lnc-bbug04238"	transcript_id "lnc-bbut00009814"	exon_number "3"
12	lncRNA	exon	103497878	103501056	.	+	.	gene_id "lnc-bbug04238"	transcript_id "lnc-bbut00009813"	exon_number "1"
12	lncRNA	exon	103501372	103501427	.	+	.	gene_id "lnc-bbug04238"	transcript_id "lnc-bbut00009813"	exon_number "2"
12	lncRNA	exon	103506023	103506081	.	+	.	gene_id "lnc-bbug04238"	transcript_id "lnc-bbut00009813"	exon_number "3"
12	lncRNA	exon	103567231	103568037	.	+	.	gene_id "lnc-bbug04239"	transcript_id "lnc-bbut00009815"	exon_number "1"
12	lncRNA	exon	103568383	103573595	.	+	.	gene_id "lnc-bbug04239"	transcript_id "lnc-bbut00009815"	exon_number "2"
12	lncRNA	exon	103583634	103583678	.	+	.	gene_id "lnc-bbug04240"	transcript_id "lnc-bbut00009820"	exon_number "1"
12	lncRNA	exon	103585031	103585151	.	+	.	gene_id "lnc-bbug04240"	transcript_id "lnc-bbut00009820"	exon_number "2"
12	lncRNA	exon	103588667	103588788	.	+	.	gene_id "lnc-bbug04240"	transcript_id "lnc-bbut00009820"	exon_number "3"
12	lncRNA	exon	103588998	103589114	.	+	.	gene_id "lnc-bbug04240"	transcript_id "lnc-bbut00009820"	exon_number "4"
12	lncRNA	exon	103590102	103590217	.	+	.	gene_id "lnc-bbug04240"	transcript_id "lnc-bbut00009820"	exon_number "5"
12	lncRNA	exon	103590360	103590455	.	+	.	gene_id "lnc-bbug04240"	transcript_id "lnc-bbut00009820"	exon_number "6"
12	lncRNA	exon	103590590	103590754	.	+	.	gene_id "lnc-bbug04240"	transcript_id "lnc-bbut00009820"	exon_number "7"
12	lncRNA	exon	103591324	103591388	.	+	.	gene_id "lnc-bbug04240"	transcript_id "lnc-bbut00009820"	exon_number "8"
12	lncRNA	exon	103591597	103592076	.	+	.	gene_id "lnc-bbug04240"	transcript_id "lnc-bbut00009820"	exon_number "9"
12	lncRNA	exon	103644287	103645714	.	+	.	gene_id "lnc-bbug04242"	transcript_id "lnc-bbut00009826"	exon_number "1"
12	lncRNA	exon	103652190	103652256	.	+	.	gene_id "lnc-bbug04242"	transcript_id "lnc-bbut00009826"	exon_number "2"
12	lncRNA	exon	103652903	103653095	.	+	.	gene_id "lnc-bbug04242"	transcript_id "lnc-bbut00009826"	exon_number "3"
12	lncRNA	exon	103858777	103858892	.	+	.	gene_id "lnc-bbug04247"	transcript_id "lnc-bbut00009847"	exon_number "1"
12	lncRNA	exon	103860503	103861175	.	+	.	gene_id "lnc-bbug04247"	transcript_id "lnc-bbut00009847"	exon_number "2"
12	lncRNA	exon	103861602	103861727	.	+	.	gene_id "lnc-bbug04247"	transcript_id "lnc-bbut00009847"	exon_number "3"
12	lncRNA	exon	103862065	103862137	.	+	.	gene_id "lnc-bbug04247"	transcript_id "lnc-bbut00009847"	exon_number "4"
12	lncRNA	exon	103862682	103862807	.	+	.	gene_id "lnc-bbug04247"	transcript_id "lnc-bbut00009847"	exon_number "5"
12	lncRNA	exon	103865571	103865678	.	+	.	gene_id "lnc-bbug04247"	transcript_id "lnc-bbut00009847"	exon_number "6"
12	lncRNA	exon	103866209	103866446	.	+	.	gene_id "lnc-bbug04247"	transcript_id "lnc-bbut00009847"	exon_number "7"
12	lncRNA	exon	103858914	103861175	.	+	.	gene_id "lnc-bbug04247"	transcript_id "lnc-bbut00009848"	exon_number "1"
12	lncRNA	exon	103861602	103861727	.	+	.	gene_id "lnc-bbug04247"	transcript_id "lnc-bbut00009848"	exon_number "2"
12	lncRNA	exon	103862065	103862137	.	+	.	gene_id "lnc-bbug04247"	transcript_id "lnc-bbut00009848"	exon_number "3"
12	lncRNA	exon	103862682	103862807	.	+	.	gene_id "lnc-bbug04247"	transcript_id "lnc-bbut00009848"	exon_number "4"
12	lncRNA	exon	103865571	103865678	.	+	.	gene_id "lnc-bbug04247"	transcript_id "lnc-bbut00009848"	exon_number "5"
12	lncRNA	exon	103866209	103866446	.	+	.	gene_id "lnc-bbug04247"	transcript_id "lnc-bbut00009848"	exon_number "6"
12	lncRNA	exon	103881690	103882456	.	+	.	gene_id "lnc-bbug04248"	transcript_id "lnc-bbut00009851"	exon_number "1"
12	lncRNA	exon	103882716	103882954	.	+	.	gene_id "lnc-bbug04248"	transcript_id "lnc-bbut00009851"	exon_number "2"
12	lncRNA	exon	103883186	103884263	.	+	.	gene_id "lnc-bbug04248"	transcript_id "lnc-bbut00009851"	exon_number "3"
12	lncRNA	exon	103881691	103882456	.	+	.	gene_id "lnc-bbug04248"	transcript_id "lnc-bbut00009852"	exon_number "1"
12	lncRNA	exon	103883186	103884263	.	+	.	gene_id "lnc-bbug04248"	transcript_id "lnc-bbut00009852"	exon_number "2"
12	lncRNA	exon	104294823	104295797	.	+	.	gene_id "lnc-bbug04252"	transcript_id "lnc-bbut00009864"	exon_number "1"
12	lncRNA	exon	104300666	104300832	.	+	.	gene_id "lnc-bbug04252"	transcript_id "lnc-bbut00009864"	exon_number "2"
12	lncRNA	exon	104303670	104303714	.	+	.	gene_id "lnc-bbug04252"	transcript_id "lnc-bbut00009864"	exon_number "3"
12	lncRNA	exon	104306988	104307066	.	+	.	gene_id "lnc-bbug04253"	transcript_id "lnc-bbut00009865"	exon_number "1"
12	lncRNA	exon	104307698	104311520	.	+	.	gene_id "lnc-bbug04253"	transcript_id "lnc-bbut00009865"	exon_number "2"
12	lncRNA	exon	104389986	104391326	.	+	.	gene_id "lnc-bbug04255"	transcript_id "lnc-bbut00009872"	exon_number "1"
12	lncRNA	exon	104394666	104394738	.	+	.	gene_id "lnc-bbug04255"	transcript_id "lnc-bbut00009872"	exon_number "2"
12	lncRNA	exon	104397680	104402081	.	+	.	gene_id "lnc-bbug04255"	transcript_id "lnc-bbut00009872"	exon_number "3"
12	lncRNA	exon	104402638	104404554	.	+	.	gene_id "lnc-bbug04256"	transcript_id "lnc-bbut00009873"	exon_number "1"
12	lncRNA	exon	104410327	104410696	.	+	.	gene_id "lnc-bbug04256"	transcript_id "lnc-bbut00009873"	exon_number "2"
12	lncRNA	exon	104423928	104424050	.	+	.	gene_id "lnc-bbug04257"	transcript_id "lnc-bbut00009874"	exon_number "1"

12	lncRNA	exon	104424463	104424976	.	+	.	gene_id "lnc-bbug04257"	transcript_id "lnc-bbut00009874"	exon_number "2"
12	lncRNA	exon	104458895	104459229	.	+	.	gene_id "lnc-bbug04258"	transcript_id "lnc-bbut00009875"	exon_number "1"
12	lncRNA	exon	104460355	104460519	.	+	.	gene_id "lnc-bbug04258"	transcript_id "lnc-bbut00009875"	exon_number "2"
12	lncRNA	exon	104461252	104462426	.	+	.	gene_id "lnc-bbug04258"	transcript_id "lnc-bbut00009875"	exon_number "3"
12	lncRNA	exon	105581632	105581861	.	+	.	gene_id "lnc-bbug04283"	transcript_id "lnc-bbut00009945"	exon_number "1"
12	lncRNA	exon	105582350	105582622	.	+	.	gene_id "lnc-bbug04283"	transcript_id "lnc-bbut00009945"	exon_number "2"
12	lncRNA	exon	105679513	105679900	.	+	.	gene_id "lnc-bbug04286"	transcript_id "lnc-bbut00009948"	exon_number "1"
12	lncRNA	exon	105680053	105680228	.	+	.	gene_id "lnc-bbug04286"	transcript_id "lnc-bbut00009948"	exon_number "2"
12	lncRNA	exon	105690589	105690818	.	+	.	gene_id "lnc-bbug04286"	transcript_id "lnc-bbut00009948"	exon_number "3"
12	lncRNA	exon	105907043	105908366	.	+	.	gene_id "lnc-bbug04293"	transcript_id "lnc-bbut00009960"	exon_number "1"
12	lncRNA	exon	105908727	105908806	.	+	.	gene_id "lnc-bbug04293"	transcript_id "lnc-bbut00009960"	exon_number "2"
12	lncRNA	exon	105908903	105910197	.	+	.	gene_id "lnc-bbug04293"	transcript_id "lnc-bbut00009960"	exon_number "3"
12	lncRNA	exon	106062263	106062510	.	+	.	gene_id "lnc-bbug04294"	transcript_id "lnc-bbut00009961"	exon_number "1"
12	lncRNA	exon	106062878	106066924	.	+	.	gene_id "lnc-bbug04294"	transcript_id "lnc-bbut00009961"	exon_number "2"
12	lncRNA	exon	898502	898812	.	-	.	gene_id "lnc-bbug04302"	transcript_id "lnc-bbut00009980"	exon_number "1"
12	lncRNA	exon	900146	900284	.	-	.	gene_id "lnc-bbug04302"	transcript_id "lnc-bbut00009980"	exon_number "2"
12	lncRNA	exon	911825	912042	.	-	.	gene_id "lnc-bbug04302"	transcript_id "lnc-bbut00009980"	exon_number "3"
12	lncRNA	exon	1107515	1111831	.	-	.	gene_id "lnc-bbug04303"	transcript_id "lnc-bbut00009985"	exon_number "1"
12	lncRNA	exon	1125810	1125919	.	-	.	gene_id "lnc-bbug04303"	transcript_id "lnc-bbut00009985"	exon_number "2"
12	lncRNA	exon	1154072	1154161	.	-	.	gene_id "lnc-bbug04303"	transcript_id "lnc-bbut00009985"	exon_number "3"
12	lncRNA	exon	1154330	1154466	.	-	.	gene_id "lnc-bbug04303"	transcript_id "lnc-bbut00009985"	exon_number "4"
12	lncRNA	exon	1157067	1157193	.	-	.	gene_id "lnc-bbug04303"	transcript_id "lnc-bbut00009985"	exon_number "5"
12	lncRNA	exon	1107515	1111831	.	-	.	gene_id "lnc-bbug04303"	transcript_id "lnc-bbut00009984"	exon_number "1"
12	lncRNA	exon	1154072	1154161	.	-	.	gene_id "lnc-bbug04303"	transcript_id "lnc-bbut00009984"	exon_number "2"
12	lncRNA	exon	1154330	1154466	.	-	.	gene_id "lnc-bbug04303"	transcript_id "lnc-bbut00009984"	exon_number "3"
12	lncRNA	exon	1157067	1157193	.	-	.	gene_id "lnc-bbug04303"	transcript_id "lnc-bbut00009984"	exon_number "4"
12	lncRNA	exon	1107515	1111831	.	-	.	gene_id "lnc-bbug04303"	transcript_id "lnc-bbut00009983"	exon_number "1"
12	lncRNA	exon	1125810	1125919	.	-	.	gene_id "lnc-bbug04303"	transcript_id "lnc-bbut00009983"	exon_number "2"
12	lncRNA	exon	1154330	1154466	.	-	.	gene_id "lnc-bbug04303"	transcript_id "lnc-bbut00009983"	exon_number "3"
12	lncRNA	exon	1157067	1157193	.	-	.	gene_id "lnc-bbug04303"	transcript_id "lnc-bbut00009983"	exon_number "4"
12	lncRNA	exon	1125810	1125919	.	-	.	gene_id "lnc-bbug04303"	transcript_id "lnc-bbut00009989"	exon_number "1"
12	lncRNA	exon	1157067	1157825	.	-	.	gene_id "lnc-bbug04303"	transcript_id "lnc-bbut00009989"	exon_number "2"
12	lncRNA	exon	2287511	2287725	.	-	.	gene_id "lnc-bbug04314"	transcript_id "lnc-bbut00010006"	exon_number "1"
12	lncRNA	exon	2287939	2288086	.	-	.	gene_id "lnc-bbug04314"	transcript_id "lnc-bbut00010006"	exon_number "2"
12	lncRNA	exon	2433781	2434332	.	-	.	gene_id "lnc-bbug04316"	transcript_id "lnc-bbut00010012"	exon_number "1"
12	lncRNA	exon	2435268	2435353	.	-	.	gene_id "lnc-bbug04316"	transcript_id "lnc-bbut00010012"	exon_number "2"
12	lncRNA	exon	2435602	2435728	.	-	.	gene_id "lnc-bbug04316"	transcript_id "lnc-bbut00010012"	exon_number "3"
12	lncRNA	exon	2436410	2436494	.	-	.	gene_id "lnc-bbug04316"	transcript_id "lnc-bbut00010012"	exon_number "4"
12	lncRNA	exon	2438017	2439235	.	-	.	gene_id "lnc-bbug04316"	transcript_id "lnc-bbut00010012"	exon_number "5"
12	lncRNA	exon	3674825	3677041	.	-	.	gene_id "lnc-bbug04326"	transcript_id "lnc-bbut00010052"	exon_number "1"
12	lncRNA	exon	3679115	3679198	.	-	.	gene_id "lnc-bbug04326"	transcript_id "lnc-bbut00010052"	exon_number "2"
12	lncRNA	exon	3710066	3710271	.	-	.	gene_id "lnc-bbug04326"	transcript_id "lnc-bbut00010052"	exon_number "3"
12	lncRNA	exon	4269602	4270014	.	-	.	gene_id "lnc-bbug04333"	transcript_id "lnc-bbut00010075"	exon_number "1"
12	lncRNA	exon	4270207	4270337	.	-	.	gene_id "lnc-bbug04333"	transcript_id "lnc-bbut00010075"	exon_number "2"
12	lncRNA	exon	4272077	4272083	.	-	.	gene_id "lnc-bbug04333"	transcript_id "lnc-bbut00010075"	exon_number "3"
12	lncRNA	exon	4272879	4273066	.	-	.	gene_id "lnc-bbug04333"	transcript_id "lnc-bbut00010075"	exon_number "4"
12	lncRNA	exon	4273939	4274104	.	-	.	gene_id "lnc-bbug04333"	transcript_id "lnc-bbut00010075"	exon_number "5"
12	lncRNA	exon	4551696	4552286	.	-	.	gene_id "lnc-bbug04337"	transcript_id "lnc-bbut00010091"	exon_number "1"
12	lncRNA	exon	6044315	6044617	.	-	.	gene_id "lnc-bbug04341"	transcript_id "lnc-bbut00010099"	exon_number "1"
12	lncRNA	exon	6048843	6048960	.	-	.	gene_id "lnc-bbug04341"	transcript_id "lnc-bbut00010099"	exon_number "2"
12	lncRNA	exon	6051202	6051333	.	-	.	gene_id "lnc-bbug04341"	transcript_id "lnc-bbut00010099"	exon_number "3"
12	lncRNA	exon	6051615	6051672	.	-	.	gene_id "lnc-bbug04341"	transcript_id "lnc-bbut00010099"	exon_number "4"

12	lncRNA	exon	6809035	6809142	.	-	.	gene_id "lnc-bbug04346"	transcript_id "lnc-bbut00010107"	exon_number "1"
12	lncRNA	exon	6809882	6810053	.	-	.	gene_id "lnc-bbug04346"	transcript_id "lnc-bbut00010107"	exon_number "2"
12	lncRNA	exon	6880781	6881566	.	-	.	gene_id "lnc-bbug04346"	transcript_id "lnc-bbut00010107"	exon_number "3"
12	lncRNA	exon	8841341	8841605	.	-	.	gene_id "lnc-bbug04348"	transcript_id "lnc-bbut00010109"	exon_number "1"
12	lncRNA	exon	8844850	8845032	.	-	.	gene_id "lnc-bbug04348"	transcript_id "lnc-bbut00010109"	exon_number "2"
12	lncRNA	exon	8854458	8855036	.	-	.	gene_id "lnc-bbug04348"	transcript_id "lnc-bbut00010109"	exon_number "3"
12	lncRNA	exon	8855481	8855587	.	-	.	gene_id "lnc-bbug04349"	transcript_id "lnc-bbut00010110"	exon_number "1"
12	lncRNA	exon	8858049	8858164	.	-	.	gene_id "lnc-bbug04349"	transcript_id "lnc-bbut00010110"	exon_number "2"
12	lncRNA	exon	8860429	8860578	.	-	.	gene_id "lnc-bbug04349"	transcript_id "lnc-bbut00010110"	exon_number "3"
12	lncRNA	exon	8863188	8863582	.	-	.	gene_id "lnc-bbug04349"	transcript_id "lnc-bbut00010110"	exon_number "4"
12	lncRNA	exon	8901841	8902062	.	-	.	gene_id "lnc-bbug04350"	transcript_id "lnc-bbut00010111"	exon_number "1"
12	lncRNA	exon	8907282	8907516	.	-	.	gene_id "lnc-bbug04350"	transcript_id "lnc-bbut00010111"	exon_number "2"
12	lncRNA	exon	8908346	8909511	.	-	.	gene_id "lnc-bbug04350"	transcript_id "lnc-bbut00010111"	exon_number "3"
12	lncRNA	exon	9148988	9149002	.	-	.	gene_id "lnc-bbug04352"	transcript_id "lnc-bbut00010114"	exon_number "1"
12	lncRNA	exon	9149332	9150000	.	-	.	gene_id "lnc-bbug04352"	transcript_id "lnc-bbut00010114"	exon_number "2"
12	lncRNA	exon	9150392	9150442	.	-	.	gene_id "lnc-bbug04352"	transcript_id "lnc-bbut00010114"	exon_number "3"
12	lncRNA	exon	9151104	9151231	.	-	.	gene_id "lnc-bbug04352"	transcript_id "lnc-bbut00010114"	exon_number "4"
12	lncRNA	exon	9149632	9150022	.	-	.	gene_id "lnc-bbug04352"	transcript_id "lnc-bbut00010115"	exon_number "1"
12	lncRNA	exon	9150392	9150442	.	-	.	gene_id "lnc-bbug04352"	transcript_id "lnc-bbut00010115"	exon_number "2"
12	lncRNA	exon	9151104	9151238	.	-	.	gene_id "lnc-bbug04352"	transcript_id "lnc-bbut00010115"	exon_number "3"
12	lncRNA	exon	10107060	10107362	.	-	.	gene_id "lnc-bbug04365"	transcript_id "lnc-bbut00010138"	exon_number "1"
12	lncRNA	exon	10116537	10116648	.	-	.	gene_id "lnc-bbug04365"	transcript_id "lnc-bbut00010138"	exon_number "2"
12	lncRNA	exon	10117269	10117524	.	-	.	gene_id "lnc-bbug04365"	transcript_id "lnc-bbut00010138"	exon_number "3"
12	lncRNA	exon	10653265	10656796	.	-	.	gene_id "lnc-bbug04371"	transcript_id "lnc-bbut00010152"	exon_number "1"
12	lncRNA	exon	11208619	11209135	.	-	.	gene_id "lnc-bbug04379"	transcript_id "lnc-bbut00010173"	exon_number "1"
12	lncRNA	exon	14190527	14192073	.	-	.	gene_id "lnc-bbug04389"	transcript_id "lnc-bbut00010201"	exon_number "1"
12	lncRNA	exon	14199907	14200621	.	-	.	gene_id "lnc-bbug04389"	transcript_id "lnc-bbut00010201"	exon_number "2"
12	lncRNA	exon	14845584	14845697	.	-	.	gene_id "lnc-bbug04394"	transcript_id "lnc-bbut00010219"	exon_number "1"
12	lncRNA	exon	14871509	14871604	.	-	.	gene_id "lnc-bbug04394"	transcript_id "lnc-bbut00010219"	exon_number "2"
12	lncRNA	exon	14880812	14880912	.	-	.	gene_id "lnc-bbug04394"	transcript_id "lnc-bbut00010219"	exon_number "3"
12	lncRNA	exon	14883225	14883691	.	-	.	gene_id "lnc-bbug04394"	transcript_id "lnc-bbut00010219"	exon_number "4"
12	lncRNA	exon	14845584	14845697	.	-	.	gene_id "lnc-bbug04394"	transcript_id "lnc-bbut00010218"	exon_number "1"
12	lncRNA	exon	14871509	14871604	.	-	.	gene_id "lnc-bbug04394"	transcript_id "lnc-bbut00010218"	exon_number "2"
12	lncRNA	exon	14883225	14883691	.	-	.	gene_id "lnc-bbug04394"	transcript_id "lnc-bbut00010218"	exon_number "3"
12	lncRNA	exon	15320905	15322243	.	-	.	gene_id "lnc-bbug04395"	transcript_id "lnc-bbut00010220"	exon_number "1"
12	lncRNA	exon	18083305	18083416	.	-	.	gene_id "lnc-bbug04397"	transcript_id "lnc-bbut00010222"	exon_number "1"
12	lncRNA	exon	18083587	18083719	.	-	.	gene_id "lnc-bbug04397"	transcript_id "lnc-bbut00010222"	exon_number "2"
12	lncRNA	exon	18089034	18089147	.	-	.	gene_id "lnc-bbug04397"	transcript_id "lnc-bbut00010222"	exon_number "3"
12	lncRNA	exon	18480519	18480659	.	-	.	gene_id "lnc-bbug04398"	transcript_id "lnc-bbut00010223"	exon_number "1"
12	lncRNA	exon	18492337	18493005	.	-	.	gene_id "lnc-bbug04398"	transcript_id "lnc-bbut00010223"	exon_number "2"
12	lncRNA	exon	20128666	20129202	.	-	.	gene_id "lnc-bbug04407"	transcript_id "lnc-bbut00010245"	exon_number "1"
12	lncRNA	exon	20137659	20138416	.	-	.	gene_id "lnc-bbug04407"	transcript_id "lnc-bbut00010245"	exon_number "2"
12	lncRNA	exon	20148296	20148477	.	-	.	gene_id "lnc-bbug04407"	transcript_id "lnc-bbut00010245"	exon_number "3"
12	lncRNA	exon	20204744	20204888	.	-	.	gene_id "lnc-bbug04407"	transcript_id "lnc-bbut00010245"	exon_number "4"
12	lncRNA	exon	23458421	23461082	.	-	.	gene_id "lnc-bbug04420"	transcript_id "lnc-bbut00010307"	exon_number "1"
12	lncRNA	exon	23461392	23461605	.	-	.	gene_id "lnc-bbug04420"	transcript_id "lnc-bbut00010307"	exon_number "2"
12	lncRNA	exon	23471194	23471308	.	-	.	gene_id "lnc-bbug04420"	transcript_id "lnc-bbut00010307"	exon_number "3"
12	lncRNA	exon	23477829	23477969	.	-	.	gene_id "lnc-bbug04420"	transcript_id "lnc-bbut00010307"	exon_number "4"
12	lncRNA	exon	23458421	23461082	.	-	.	gene_id "lnc-bbug04420"	transcript_id "lnc-bbut00010306"	exon_number "1"
12	lncRNA	exon	23461392	23461605	.	-	.	gene_id "lnc-bbug04420"	transcript_id "lnc-bbut00010306"	exon_number "2"
12	lncRNA	exon	23471194	23471308	.	-	.	gene_id "lnc-bbug04420"	transcript_id "lnc-bbut00010306"	exon_number "3"
12	lncRNA	exon	23477565	23477969	.	-	.	gene_id "lnc-bbug04420"	transcript_id "lnc-bbut00010306"	exon_number "4"

12	lncRNA	exon	23494942	23496539	.	-	.	gene_id "lnc-bbug04421"	transcript_id "lnc-bbut00010308"	exon_number "1"
12	lncRNA	exon	23574231	23574324	.	-	.	gene_id "lnc-bbug04421"	transcript_id "lnc-bbut00010308"	exon_number "2"
12	lncRNA	exon	23650672	23650781	.	-	.	gene_id "lnc-bbug04421"	transcript_id "lnc-bbut00010308"	exon_number "3"
12	lncRNA	exon	23849498	23849624	.	-	.	gene_id "lnc-bbug04421"	transcript_id "lnc-bbut00010308"	exon_number "4"
12	lncRNA	exon	23851852	23851994	.	-	.	gene_id "lnc-bbug04421"	transcript_id "lnc-bbut00010308"	exon_number "5"
12	lncRNA	exon	23572208	23574324	.	-	.	gene_id "lnc-bbug04421"	transcript_id "lnc-bbut00010309"	exon_number "1"
12	lncRNA	exon	23650672	23650781	.	-	.	gene_id "lnc-bbug04421"	transcript_id "lnc-bbut00010309"	exon_number "2"
12	lncRNA	exon	23849498	23849624	.	-	.	gene_id "lnc-bbug04421"	transcript_id "lnc-bbut00010309"	exon_number "3"
12	lncRNA	exon	23851852	23851994	.	-	.	gene_id "lnc-bbug04421"	transcript_id "lnc-bbut00010309"	exon_number "4"
12	lncRNA	exon	23852036	23852085	.	-	.	gene_id "lnc-bbug04422"	transcript_id "lnc-bbut00010311"	exon_number "1"
12	lncRNA	exon	23852384	23852573	.	-	.	gene_id "lnc-bbug04422"	transcript_id "lnc-bbut00010311"	exon_number "2"
12	lncRNA	exon	23877927	23878051	.	-	.	gene_id "lnc-bbug04422"	transcript_id "lnc-bbut00010311"	exon_number "3"
12	lncRNA	exon	23879102	23879345	.	-	.	gene_id "lnc-bbug04422"	transcript_id "lnc-bbut00010311"	exon_number "4"
12	lncRNA	exon	23880278	23880418	.	-	.	gene_id "lnc-bbug04422"	transcript_id "lnc-bbut00010311"	exon_number "5"
12	lncRNA	exon	23852036	23852085	.	-	.	gene_id "lnc-bbug04422"	transcript_id "lnc-bbut00010310"	exon_number "1"
12	lncRNA	exon	23852384	23852573	.	-	.	gene_id "lnc-bbug04422"	transcript_id "lnc-bbut00010310"	exon_number "2"
12	lncRNA	exon	23877927	23878051	.	-	.	gene_id "lnc-bbug04422"	transcript_id "lnc-bbut00010310"	exon_number "3"
12	lncRNA	exon	23880278	23880418	.	-	.	gene_id "lnc-bbug04422"	transcript_id "lnc-bbut00010310"	exon_number "4"
12	lncRNA	exon	24658304	24659176	.	-	.	gene_id "lnc-bbug04423"	transcript_id "lnc-bbut00010312"	exon_number "1"
12	lncRNA	exon	24660418	24660549	.	-	.	gene_id "lnc-bbug04423"	transcript_id "lnc-bbut00010312"	exon_number "2"
12	lncRNA	exon	24670209	24670372	.	-	.	gene_id "lnc-bbug04423"	transcript_id "lnc-bbut00010312"	exon_number "3"
12	lncRNA	exon	24689423	24690068	.	-	.	gene_id "lnc-bbug04424"	transcript_id "lnc-bbut00010313"	exon_number "1"
12	lncRNA	exon	24690734	24690863	.	-	.	gene_id "lnc-bbug04424"	transcript_id "lnc-bbut00010313"	exon_number "2"
12	lncRNA	exon	24693749	24696152	.	-	.	gene_id "lnc-bbug04424"	transcript_id "lnc-bbut00010313"	exon_number "3"
12	lncRNA	exon	25038828	25040730	.	-	.	gene_id "lnc-bbug04428"	transcript_id "lnc-bbut00010319"	exon_number "1"
12	lncRNA	exon	25040819	25041501	.	-	.	gene_id "lnc-bbug04428"	transcript_id "lnc-bbut00010319"	exon_number "2"
12	lncRNA	exon	25333673	25336247	.	-	.	gene_id "lnc-bbug04429"	transcript_id "lnc-bbut00010324"	exon_number "1"
12	lncRNA	exon	25337277	25337609	.	-	.	gene_id "lnc-bbug04429"	transcript_id "lnc-bbut00010324"	exon_number "2"
12	lncRNA	exon	25369948	25370021	.	-	.	gene_id "lnc-bbug04429"	transcript_id "lnc-bbut00010324"	exon_number "3"
12	lncRNA	exon	25374993	25375111	.	-	.	gene_id "lnc-bbug04429"	transcript_id "lnc-bbut00010324"	exon_number "4"
12	lncRNA	exon	25379642	25379706	.	-	.	gene_id "lnc-bbug04429"	transcript_id "lnc-bbut00010324"	exon_number "5"
12	lncRNA	exon	25387656	25389946	.	-	.	gene_id "lnc-bbug04429"	transcript_id "lnc-bbut00010324"	exon_number "6"
12	lncRNA	exon	25333673	25336247	.	-	.	gene_id "lnc-bbug04429"	transcript_id "lnc-bbut00010326"	exon_number "1"
12	lncRNA	exon	25337468	25337609	.	-	.	gene_id "lnc-bbug04429"	transcript_id "lnc-bbut00010326"	exon_number "2"
12	lncRNA	exon	25369948	25371917	.	-	.	gene_id "lnc-bbug04429"	transcript_id "lnc-bbut00010326"	exon_number "3"
12	lncRNA	exon	25374993	25375111	.	-	.	gene_id "lnc-bbug04429"	transcript_id "lnc-bbut00010326"	exon_number "4"
12	lncRNA	exon	25379642	25379706	.	-	.	gene_id "lnc-bbug04429"	transcript_id "lnc-bbut00010326"	exon_number "5"
12	lncRNA	exon	25387656	25389946	.	-	.	gene_id "lnc-bbug04429"	transcript_id "lnc-bbut00010326"	exon_number "6"
12	lncRNA	exon	25333673	25336247	.	-	.	gene_id "lnc-bbug04429"	transcript_id "lnc-bbut00010325"	exon_number "1"
12	lncRNA	exon	25337277	25337609	.	-	.	gene_id "lnc-bbug04429"	transcript_id "lnc-bbut00010325"	exon_number "2"
12	lncRNA	exon	25369948	25371917	.	-	.	gene_id "lnc-bbug04429"	transcript_id "lnc-bbut00010325"	exon_number "3"
12	lncRNA	exon	25374993	25375111	.	-	.	gene_id "lnc-bbug04429"	transcript_id "lnc-bbut00010325"	exon_number "4"
12	lncRNA	exon	25379642	25379706	.	-	.	gene_id "lnc-bbug04429"	transcript_id "lnc-bbut00010325"	exon_number "5"
12	lncRNA	exon	25387656	25389946	.	-	.	gene_id "lnc-bbug04429"	transcript_id "lnc-bbut00010325"	exon_number "6"
12	lncRNA	exon	25338248	25342497	.	-	.	gene_id "lnc-bbug04429"	transcript_id "lnc-bbut00010329"	exon_number "1"
12	lncRNA	exon	25369948	25371917	.	-	.	gene_id "lnc-bbug04429"	transcript_id "lnc-bbut00010329"	exon_number "2"
12	lncRNA	exon	25374993	25375111	.	-	.	gene_id "lnc-bbug04429"	transcript_id "lnc-bbut00010329"	exon_number "3"
12	lncRNA	exon	25379642	25379706	.	-	.	gene_id "lnc-bbug04429"	transcript_id "lnc-bbut00010329"	exon_number "4"
12	lncRNA	exon	25387656	25389946	.	-	.	gene_id "lnc-bbug04429"	transcript_id "lnc-bbut00010329"	exon_number "5"
12	lncRNA	exon	25338248	25342497	.	-	.	gene_id "lnc-bbug04429"	transcript_id "lnc-bbut00010328"	exon_number "1"
12	lncRNA	exon	25369948	25370021	.	-	.	gene_id "lnc-bbug04429"	transcript_id "lnc-bbut00010328"	exon_number "2"
12	lncRNA	exon	25374993	25375111	.	-	.	gene_id "lnc-bbug04429"	transcript_id "lnc-bbut00010328"	exon_number "3"

12	lncRNA	exon	25379642	25379706	.	-	.	gene_id "lnc-bbug04429"	transcript_id "lnc-bbut00010328"	exon_number "4"
12	lncRNA	exon	25387656	25389946	.	-	.	gene_id "lnc-bbug04429"	transcript_id "lnc-bbut00010328"	exon_number "5"
12	lncRNA	exon	25338248	25342497	.	-	.	gene_id "lnc-bbug04429"	transcript_id "lnc-bbut00010327"	exon_number "1"
12	lncRNA	exon	25344494	25348488	.	-	.	gene_id "lnc-bbug04429"	transcript_id "lnc-bbut00010327"	exon_number "2"
12	lncRNA	exon	25369948	25371917	.	-	.	gene_id "lnc-bbug04429"	transcript_id "lnc-bbut00010327"	exon_number "3"
12	lncRNA	exon	25374993	25375111	.	-	.	gene_id "lnc-bbug04429"	transcript_id "lnc-bbut00010327"	exon_number "4"
12	lncRNA	exon	25379642	25379706	.	-	.	gene_id "lnc-bbug04429"	transcript_id "lnc-bbut00010327"	exon_number "5"
12	lncRNA	exon	25387656	25389946	.	-	.	gene_id "lnc-bbug04429"	transcript_id "lnc-bbut00010327"	exon_number "6"
12	lncRNA	exon	25370740	25371917	.	-	.	gene_id "lnc-bbug04429"	transcript_id "lnc-bbut00010331"	exon_number "1"
12	lncRNA	exon	25374993	25375111	.	-	.	gene_id "lnc-bbug04429"	transcript_id "lnc-bbut00010331"	exon_number "2"
12	lncRNA	exon	25387656	25389946	.	-	.	gene_id "lnc-bbug04429"	transcript_id "lnc-bbut00010331"	exon_number "3"
12	lncRNA	exon	25370740	25371917	.	-	.	gene_id "lnc-bbug04429"	transcript_id "lnc-bbut00010332"	exon_number "1"
12	lncRNA	exon	25374993	25375111	.	-	.	gene_id "lnc-bbug04429"	transcript_id "lnc-bbut00010332"	exon_number "2"
12	lncRNA	exon	25379642	25379706	.	-	.	gene_id "lnc-bbug04429"	transcript_id "lnc-bbut00010332"	exon_number "3"
12	lncRNA	exon	25389953	25390760	.	-	.	gene_id "lnc-bbug04429"	transcript_id "lnc-bbut00010332"	exon_number "4"
12	lncRNA	exon	26303174	26303619	.	-	.	gene_id "lnc-bbug04434"	transcript_id "lnc-bbut00010346"	exon_number "1"
12	lncRNA	exon	27735461	27735508	.	-	.	gene_id "lnc-bbug04439"	transcript_id "lnc-bbut00010361"	exon_number "1"
12	lncRNA	exon	27737892	27737968	.	-	.	gene_id "lnc-bbug04439"	transcript_id "lnc-bbut00010361"	exon_number "2"
12	lncRNA	exon	27743273	27743342	.	-	.	gene_id "lnc-bbug04439"	transcript_id "lnc-bbut00010361"	exon_number "3"
12	lncRNA	exon	27750035	27750405	.	-	.	gene_id "lnc-bbug04439"	transcript_id "lnc-bbut00010361"	exon_number "4"
12	lncRNA	exon	27735461	27735508	.	-	.	gene_id "lnc-bbug04439"	transcript_id "lnc-bbut00010359"	exon_number "1"
12	lncRNA	exon	27737892	27737943	.	-	.	gene_id "lnc-bbug04439"	transcript_id "lnc-bbut00010359"	exon_number "2"
12	lncRNA	exon	27743273	27743342	.	-	.	gene_id "lnc-bbug04439"	transcript_id "lnc-bbut00010359"	exon_number "3"
12	lncRNA	exon	27750035	27750405	.	-	.	gene_id "lnc-bbug04439"	transcript_id "lnc-bbut00010359"	exon_number "4"
12	lncRNA	exon	29216774	29221546	.	-	.	gene_id "lnc-bbug04444"	transcript_id "lnc-bbut00010384"	exon_number "1"
12	lncRNA	exon	29224582	29224686	.	-	.	gene_id "lnc-bbug04444"	transcript_id "lnc-bbut00010384"	exon_number "2"
12	lncRNA	exon	29253738	29253995	.	-	.	gene_id "lnc-bbug04444"	transcript_id "lnc-bbut00010384"	exon_number "3"
12	lncRNA	exon	29255094	29255153	.	-	.	gene_id "lnc-bbug04444"	transcript_id "lnc-bbut00010384"	exon_number "4"
12	lncRNA	exon	29257725	29258694	.	-	.	gene_id "lnc-bbug04444"	transcript_id "lnc-bbut00010384"	exon_number "5"
12	lncRNA	exon	29418973	29420783	.	-	.	gene_id "lnc-bbug04447"	transcript_id "lnc-bbut00010389"	exon_number "1"
12	lncRNA	exon	29420921	29421015	.	-	.	gene_id "lnc-bbug04447"	transcript_id "lnc-bbut00010389"	exon_number "2"
12	lncRNA	exon	29421521	29421568	.	-	.	gene_id "lnc-bbug04447"	transcript_id "lnc-bbut00010389"	exon_number "3"
12	lncRNA	exon	29753150	29754777	.	-	.	gene_id "lnc-bbug04449"	transcript_id "lnc-bbut00010392"	exon_number "1"
12	lncRNA	exon	29767330	29767502	.	-	.	gene_id "lnc-bbug04449"	transcript_id "lnc-bbut00010392"	exon_number "2"
12	lncRNA	exon	29964135	29964518	.	-	.	gene_id "lnc-bbug04451"	transcript_id "lnc-bbut00010396"	exon_number "1"
12	lncRNA	exon	29965297	29965373	.	-	.	gene_id "lnc-bbug04451"	transcript_id "lnc-bbut00010396"	exon_number "2"
12	lncRNA	exon	29971466	29971559	.	-	.	gene_id "lnc-bbug04451"	transcript_id "lnc-bbut00010396"	exon_number "3"
12	lncRNA	exon	29972393	29972598	.	-	.	gene_id "lnc-bbug04451"	transcript_id "lnc-bbut00010396"	exon_number "4"
12	lncRNA	exon	29983048	29983226	.	-	.	gene_id "lnc-bbug04451"	transcript_id "lnc-bbut00010396"	exon_number "5"
12	lncRNA	exon	34891300	34891531	.	-	.	gene_id "lnc-bbug04457"	transcript_id "lnc-bbut00010420"	exon_number "1"
12	lncRNA	exon	34891688	34891806	.	-	.	gene_id "lnc-bbug04457"	transcript_id "lnc-bbut00010420"	exon_number "2"
12	lncRNA	exon	34892378	34892500	.	-	.	gene_id "lnc-bbug04457"	transcript_id "lnc-bbut00010420"	exon_number "3"
12	lncRNA	exon	35311993	35312429	.	-	.	gene_id "lnc-bbug04458"	transcript_id "lnc-bbut00010421"	exon_number "1"
12	lncRNA	exon	35313923	35314016	.	-	.	gene_id "lnc-bbug04458"	transcript_id "lnc-bbut00010421"	exon_number "2"
12	lncRNA	exon	35319451	35319676	.	-	.	gene_id "lnc-bbug04458"	transcript_id "lnc-bbut00010421"	exon_number "3"
12	lncRNA	exon	35311993	35312429	.	-	.	gene_id "lnc-bbug04458"	transcript_id "lnc-bbut00010422"	exon_number "1"
12	lncRNA	exon	35313923	35314016	.	-	.	gene_id "lnc-bbug04458"	transcript_id "lnc-bbut00010422"	exon_number "2"
12	lncRNA	exon	35332003	35332100	.	-	.	gene_id "lnc-bbug04458"	transcript_id "lnc-bbut00010422"	exon_number "3"
12	lncRNA	exon	35334187	35334913	.	-	.	gene_id "lnc-bbug04458"	transcript_id "lnc-bbut00010422"	exon_number "4"
12	lncRNA	exon	35869378	35870029	.	-	.	gene_id "lnc-bbug04459"	transcript_id "lnc-bbut00010423"	exon_number "1"
12	lncRNA	exon	35947037	35947496	.	-	.	gene_id "lnc-bbug04460"	transcript_id "lnc-bbut00010424"	exon_number "1"
12	lncRNA	exon	35948964	35949066	.	-	.	gene_id "lnc-bbug04460"	transcript_id "lnc-bbut00010424"	exon_number "2"

12	lncRNA	exon	35949352	35949430	.	-	.	gene_id "lnc-bbug04460"	transcript_id "lnc-bbut00010424"	exon_number "3"
12	lncRNA	exon	37543559	37544812	.	-	.	gene_id "lnc-bbug04467"	transcript_id "lnc-bbut00010440"	exon_number "1"
12	lncRNA	exon	37544923	37544998	.	-	.	gene_id "lnc-bbug04467"	transcript_id "lnc-bbut00010440"	exon_number "2"
12	lncRNA	exon	37556990	37557236	.	-	.	gene_id "lnc-bbug04467"	transcript_id "lnc-bbut00010440"	exon_number "3"
12	lncRNA	exon	37574407	37576647	.	-	.	gene_id "lnc-bbug04467"	transcript_id "lnc-bbut00010440"	exon_number "4"
12	lncRNA	exon	37543559	37544812	.	-	.	gene_id "lnc-bbug04467"	transcript_id "lnc-bbut00010441"	exon_number "1"
12	lncRNA	exon	37544923	37544998	.	-	.	gene_id "lnc-bbug04467"	transcript_id "lnc-bbut00010441"	exon_number "2"
12	lncRNA	exon	37574407	37576649	.	-	.	gene_id "lnc-bbug04467"	transcript_id "lnc-bbut00010441"	exon_number "3"
12	lncRNA	exon	38202512	38203379	.	-	.	gene_id "lnc-bbug04474"	transcript_id "lnc-bbut00010470"	exon_number "1"
12	lncRNA	exon	38203597	38204242	.	-	.	gene_id "lnc-bbug04474"	transcript_id "lnc-bbut00010470"	exon_number "2"
12	lncRNA	exon	38205195	38205218	.	-	.	gene_id "lnc-bbug04474"	transcript_id "lnc-bbut00010470"	exon_number "3"
12	lncRNA	exon	38339653	38340860	.	-	.	gene_id "lnc-bbug04475"	transcript_id "lnc-bbut00010471"	exon_number "1"
12	lncRNA	exon	38355507	38355608	.	-	.	gene_id "lnc-bbug04475"	transcript_id "lnc-bbut00010471"	exon_number "2"
12	lncRNA	exon	38395734	38395994	.	-	.	gene_id "lnc-bbug04475"	transcript_id "lnc-bbut00010471"	exon_number "3"
12	lncRNA	exon	38397532	38398809	.	-	.	gene_id "lnc-bbug04475"	transcript_id "lnc-bbut00010471"	exon_number "4"
12	lncRNA	exon	38394171	38394509	.	-	.	gene_id "lnc-bbug04475"	transcript_id "lnc-bbut00010472"	exon_number "1"
12	lncRNA	exon	38395734	38395994	.	-	.	gene_id "lnc-bbug04475"	transcript_id "lnc-bbut00010472"	exon_number "2"
12	lncRNA	exon	38397532	38398806	.	-	.	gene_id "lnc-bbug04475"	transcript_id "lnc-bbut00010472"	exon_number "3"
12	lncRNA	exon	39727550	39727810	.	-	.	gene_id "lnc-bbug04476"	transcript_id "lnc-bbut00010473"	exon_number "1"
12	lncRNA	exon	39728736	39728888	.	-	.	gene_id "lnc-bbug04476"	transcript_id "lnc-bbut00010473"	exon_number "2"
12	lncRNA	exon	42456763	42456923	.	-	.	gene_id "lnc-bbug04479"	transcript_id "lnc-bbut00010480"	exon_number "1"
12	lncRNA	exon	42501794	42501895	.	-	.	gene_id "lnc-bbug04479"	transcript_id "lnc-bbut00010480"	exon_number "2"
12	lncRNA	exon	42518048	42518116	.	-	.	gene_id "lnc-bbug04479"	transcript_id "lnc-bbut00010480"	exon_number "3"
12	lncRNA	exon	42557038	42557093	.	-	.	gene_id "lnc-bbug04479"	transcript_id "lnc-bbut00010480"	exon_number "4"
12	lncRNA	exon	42605229	42605313	.	-	.	gene_id "lnc-bbug04479"	transcript_id "lnc-bbut00010480"	exon_number "5"
12	lncRNA	exon	42605668	42605707	.	-	.	gene_id "lnc-bbug04479"	transcript_id "lnc-bbut00010480"	exon_number "6"
12	lncRNA	exon	42610208	42610330	.	-	.	gene_id "lnc-bbug04479"	transcript_id "lnc-bbut00010480"	exon_number "7"
12	lncRNA	exon	42674216	42674309	.	-	.	gene_id "lnc-bbug04479"	transcript_id "lnc-bbut00010480"	exon_number "8"
12	lncRNA	exon	42456763	42456923	.	-	.	gene_id "lnc-bbug04479"	transcript_id "lnc-bbut00010479"	exon_number "1"
12	lncRNA	exon	42501794	42501895	.	-	.	gene_id "lnc-bbug04479"	transcript_id "lnc-bbut00010479"	exon_number "2"
12	lncRNA	exon	42518048	42518116	.	-	.	gene_id "lnc-bbug04479"	transcript_id "lnc-bbut00010479"	exon_number "3"
12	lncRNA	exon	42557038	42557093	.	-	.	gene_id "lnc-bbug04479"	transcript_id "lnc-bbut00010479"	exon_number "4"
12	lncRNA	exon	42605668	42605707	.	-	.	gene_id "lnc-bbug04479"	transcript_id "lnc-bbut00010479"	exon_number "5"
12	lncRNA	exon	42610208	42610330	.	-	.	gene_id "lnc-bbug04479"	transcript_id "lnc-bbut00010479"	exon_number "6"
12	lncRNA	exon	42674216	42674309	.	-	.	gene_id "lnc-bbug04479"	transcript_id "lnc-bbut00010479"	exon_number "7"
12	lncRNA	exon	42719130	42719464	.	-	.	gene_id "lnc-bbug04480"	transcript_id "lnc-bbut00010481"	exon_number "1"
12	lncRNA	exon	42720055	42720165	.	-	.	gene_id "lnc-bbug04480"	transcript_id "lnc-bbut00010481"	exon_number "2"
12	lncRNA	exon	42828717	42832816	.	-	.	gene_id "lnc-bbug04481"	transcript_id "lnc-bbut00010482"	exon_number "1"
12	lncRNA	exon	42833263	42833887	.	-	.	gene_id "lnc-bbug04481"	transcript_id "lnc-bbut00010482"	exon_number "2"
12	lncRNA	exon	43328303	43329584	.	-	.	gene_id "lnc-bbug04483"	transcript_id "lnc-bbut00010499"	exon_number "1"
12	lncRNA	exon	43345594	43346195	.	-	.	gene_id "lnc-bbug04483"	transcript_id "lnc-bbut00010499"	exon_number "2"
12	lncRNA	exon	43328303	43329581	.	-	.	gene_id "lnc-bbug04483"	transcript_id "lnc-bbut00010498"	exon_number "1"
12	lncRNA	exon	43345594	43346195	.	-	.	gene_id "lnc-bbug04483"	transcript_id "lnc-bbut00010498"	exon_number "2"
12	lncRNA	exon	43431153	43431688	.	-	.	gene_id "lnc-bbug04485"	transcript_id "lnc-bbut00010508"	exon_number "1"
12	lncRNA	exon	43444932	43444937	.	-	.	gene_id "lnc-bbug04485"	transcript_id "lnc-bbut00010508"	exon_number "2"
12	lncRNA	exon	43447459	43447465	.	-	.	gene_id "lnc-bbug04485"	transcript_id "lnc-bbut00010508"	exon_number "3"
12	lncRNA	exon	43529988	43530307	.	-	.	gene_id "lnc-bbug04486"	transcript_id "lnc-bbut00010509"	exon_number "1"
12	lncRNA	exon	43530424	43530488	.	-	.	gene_id "lnc-bbug04486"	transcript_id "lnc-bbut00010509"	exon_number "2"
12	lncRNA	exon	43531422	43531640	.	-	.	gene_id "lnc-bbug04486"	transcript_id "lnc-bbut00010509"	exon_number "3"
12	lncRNA	exon	43730968	43731654	.	-	.	gene_id "lnc-bbug04487"	transcript_id "lnc-bbut00010510"	exon_number "1"
12	lncRNA	exon	43731739	43731757	.	-	.	gene_id "lnc-bbug04487"	transcript_id "lnc-bbut00010510"	exon_number "2"
12	lncRNA	exon	44017187	44017928	.	-	.	gene_id "lnc-bbug04490"	transcript_id "lnc-bbut00010514"	exon_number "1"

12	lncRNA	exon	44018168	44018268	.	-	.	gene_id "lnc-bbug04490"	transcript_id "lnc-bbut00010514"	exon_number "2"
12	lncRNA	exon	44022028	44022920	.	-	.	gene_id "lnc-bbug04490"	transcript_id "lnc-bbut00010514"	exon_number "3"
12	lncRNA	exon	44038299	44038910	.	-	.	gene_id "lnc-bbug04490"	transcript_id "lnc-bbut00010514"	exon_number "4"
12	lncRNA	exon	44017187	44017589	.	-	.	gene_id "lnc-bbug04490"	transcript_id "lnc-bbut00010513"	exon_number "1"
12	lncRNA	exon	44017672	44018268	.	-	.	gene_id "lnc-bbug04490"	transcript_id "lnc-bbut00010513"	exon_number "2"
12	lncRNA	exon	44022028	44022920	.	-	.	gene_id "lnc-bbug04490"	transcript_id "lnc-bbut00010513"	exon_number "3"
12	lncRNA	exon	44038299	44038910	.	-	.	gene_id "lnc-bbug04490"	transcript_id "lnc-bbut00010513"	exon_number "4"
12	lncRNA	exon	45463868	45464582	.	-	.	gene_id "lnc-bbug04504"	transcript_id "lnc-bbut00010549"	exon_number "1"
12	lncRNA	exon	45465618	45465820	.	-	.	gene_id "lnc-bbug04504"	transcript_id "lnc-bbut00010549"	exon_number "2"
12	lncRNA	exon	45466119	45466647	.	-	.	gene_id "lnc-bbug04504"	transcript_id "lnc-bbut00010549"	exon_number "3"
12	lncRNA	exon	46278527	46279397	.	-	.	gene_id "lnc-bbug04512"	transcript_id "lnc-bbut00010559"	exon_number "1"
12	lncRNA	exon	46914541	46915440	.	-	.	gene_id "lnc-bbug04515"	transcript_id "lnc-bbut00010569"	exon_number "1"
12	lncRNA	exon	46916515	46916625	.	-	.	gene_id "lnc-bbug04515"	transcript_id "lnc-bbut00010569"	exon_number "2"
12	lncRNA	exon	47146760	47148597	.	-	.	gene_id "lnc-bbug04517"	transcript_id "lnc-bbut00010571"	exon_number "1"
12	lncRNA	exon	47556338	47556805	.	-	.	gene_id "lnc-bbug04520"	transcript_id "lnc-bbut00010576"	exon_number "1"
12	lncRNA	exon	48691049	48691063	.	-	.	gene_id "lnc-bbug04529"	transcript_id "lnc-bbut00010593"	exon_number "1"
12	lncRNA	exon	48691755	48691896	.	-	.	gene_id "lnc-bbug04529"	transcript_id "lnc-bbut00010593"	exon_number "2"
12	lncRNA	exon	48693874	48694073	.	-	.	gene_id "lnc-bbug04529"	transcript_id "lnc-bbut00010593"	exon_number "3"
12	lncRNA	exon	48698073	48698213	.	-	.	gene_id "lnc-bbug04529"	transcript_id "lnc-bbut00010593"	exon_number "4"
12	lncRNA	exon	49068002	49068444	.	-	.	gene_id "lnc-bbug04536"	transcript_id "lnc-bbut00010606"	exon_number "1"
12	lncRNA	exon	49069684	49069839	.	-	.	gene_id "lnc-bbug04536"	transcript_id "lnc-bbut00010606"	exon_number "2"
12	lncRNA	exon	49071107	49071255	.	-	.	gene_id "lnc-bbug04536"	transcript_id "lnc-bbut00010606"	exon_number "3"
12	lncRNA	exon	49745511	49745797	.	-	.	gene_id "lnc-bbug04543"	transcript_id "lnc-bbut00010619"	exon_number "1"
12	lncRNA	exon	49746078	49746192	.	-	.	gene_id "lnc-bbug04543"	transcript_id "lnc-bbut00010619"	exon_number "2"
12	lncRNA	exon	49746470	49746631	.	-	.	gene_id "lnc-bbug04543"	transcript_id "lnc-bbut00010619"	exon_number "3"
12	lncRNA	exon	60047198	60048978	.	-	.	gene_id "lnc-bbug04557"	transcript_id "lnc-bbut00010641"	exon_number "1"
12	lncRNA	exon	60627113	60627129	.	-	.	gene_id "lnc-bbug04560"	transcript_id "lnc-bbut00010646"	exon_number "1"
12	lncRNA	exon	60627549	60627656	.	-	.	gene_id "lnc-bbug04560"	transcript_id "lnc-bbut00010646"	exon_number "2"
12	lncRNA	exon	60629297	60629476	.	-	.	gene_id "lnc-bbug04560"	transcript_id "lnc-bbut00010646"	exon_number "3"
12	lncRNA	exon	60630351	60630729	.	-	.	gene_id "lnc-bbug04560"	transcript_id "lnc-bbut00010646"	exon_number "4"
12	lncRNA	exon	60632735	60632811	.	-	.	gene_id "lnc-bbug04560"	transcript_id "lnc-bbut00010646"	exon_number "5"
12	lncRNA	exon	60632910	60634183	.	-	.	gene_id "lnc-bbug04560"	transcript_id "lnc-bbut00010646"	exon_number "6"
12	lncRNA	exon	60627275	60627656	.	-	.	gene_id "lnc-bbug04560"	transcript_id "lnc-bbut00010647"	exon_number "1"
12	lncRNA	exon	60629297	60629476	.	-	.	gene_id "lnc-bbug04560"	transcript_id "lnc-bbut00010647"	exon_number "2"
12	lncRNA	exon	60630351	60630729	.	-	.	gene_id "lnc-bbug04560"	transcript_id "lnc-bbut00010647"	exon_number "3"
12	lncRNA	exon	60632735	60632811	.	-	.	gene_id "lnc-bbug04560"	transcript_id "lnc-bbut00010647"	exon_number "4"
12	lncRNA	exon	60632910	60634182	.	-	.	gene_id "lnc-bbug04560"	transcript_id "lnc-bbut00010647"	exon_number "5"
12	lncRNA	exon	60711302	60711438	.	-	.	gene_id "lnc-bbug04562"	transcript_id "lnc-bbut00010649"	exon_number "1"
12	lncRNA	exon	60737527	60737662	.	-	.	gene_id "lnc-bbug04562"	transcript_id "lnc-bbut00010649"	exon_number "2"
12	lncRNA	exon	60737967	60738108	.	-	.	gene_id "lnc-bbug04562"	transcript_id "lnc-bbut00010649"	exon_number "3"
12	lncRNA	exon	60794196	60795126	.	-	.	gene_id "lnc-bbug04563"	transcript_id "lnc-bbut00010651"	exon_number "1"
12	lncRNA	exon	60808275	60808412	.	-	.	gene_id "lnc-bbug04563"	transcript_id "lnc-bbut00010651"	exon_number "2"
12	lncRNA	exon	60819631	60819794	.	-	.	gene_id "lnc-bbug04563"	transcript_id "lnc-bbut00010651"	exon_number "3"
12	lncRNA	exon	60794196	60795123	.	-	.	gene_id "lnc-bbug04563"	transcript_id "lnc-bbut00010650"	exon_number "1"
12	lncRNA	exon	60808275	60808412	.	-	.	gene_id "lnc-bbug04563"	transcript_id "lnc-bbut00010650"	exon_number "2"
12	lncRNA	exon	60819631	60819794	.	-	.	gene_id "lnc-bbug04563"	transcript_id "lnc-bbut00010650"	exon_number "3"
12	lncRNA	exon	61026242	61026622	.	-	.	gene_id "lnc-bbug04564"	transcript_id "lnc-bbut00010652"	exon_number "1"
12	lncRNA	exon	62652903	62654945	.	-	.	gene_id "lnc-bbug04568"	transcript_id "lnc-bbut00010670"	exon_number "1"
12	lncRNA	exon	62659576	62659704	.	-	.	gene_id "lnc-bbug04568"	transcript_id "lnc-bbut00010670"	exon_number "2"
12	lncRNA	exon	62660199	62660258	.	-	.	gene_id "lnc-bbug04568"	transcript_id "lnc-bbut00010670"	exon_number "3"
12	lncRNA	exon	62660750	62660950	.	-	.	gene_id "lnc-bbug04568"	transcript_id "lnc-bbut00010670"	exon_number "4"
12	lncRNA	exon	62652903	62654945	.	-	.	gene_id "lnc-bbug04568"	transcript_id "lnc-bbut00010671"	exon_number "1"

12	lncRNA	exon	62659576	62659704	.	-	.	gene_id "lnc-bbug04568"	transcript_id "lnc-bbut00010671"	exon_number "2"
12	lncRNA	exon	62660199	62660258	.	-	.	gene_id "lnc-bbug04568"	transcript_id "lnc-bbut00010671"	exon_number "3"
12	lncRNA	exon	62661193	62661512	.	-	.	gene_id "lnc-bbug04568"	transcript_id "lnc-bbut00010671"	exon_number "4"
12	lncRNA	exon	62663072	62664246	.	-	.	gene_id "lnc-bbug04568"	transcript_id "lnc-bbut00010671"	exon_number "5"
12	lncRNA	exon	62652903	62654945	.	-	.	gene_id "lnc-bbug04568"	transcript_id "lnc-bbut00010672"	exon_number "1"
12	lncRNA	exon	62658916	62658959	.	-	.	gene_id "lnc-bbug04568"	transcript_id "lnc-bbut00010672"	exon_number "2"
12	lncRNA	exon	62659576	62659704	.	-	.	gene_id "lnc-bbug04568"	transcript_id "lnc-bbut00010672"	exon_number "3"
12	lncRNA	exon	62660199	62660258	.	-	.	gene_id "lnc-bbug04568"	transcript_id "lnc-bbut00010672"	exon_number "4"
12	lncRNA	exon	62661193	62661512	.	-	.	gene_id "lnc-bbug04568"	transcript_id "lnc-bbut00010672"	exon_number "5"
12	lncRNA	exon	62663072	62664254	.	-	.	gene_id "lnc-bbug04568"	transcript_id "lnc-bbut00010672"	exon_number "6"
12	lncRNA	exon	64421461	64421663	.	-	.	gene_id "lnc-bbug04574"	transcript_id "lnc-bbut00010683"	exon_number "1"
12	lncRNA	exon	64432986	64433031	.	-	.	gene_id "lnc-bbug04574"	transcript_id "lnc-bbut00010683"	exon_number "2"
12	lncRNA	exon	64434558	64437141	.	-	.	gene_id "lnc-bbug04574"	transcript_id "lnc-bbut00010683"	exon_number "3"
12	lncRNA	exon	64439796	64441071	.	-	.	gene_id "lnc-bbug04574"	transcript_id "lnc-bbut00010683"	exon_number "4"
12	lncRNA	exon	64588288	64590799	.	-	.	gene_id "lnc-bbug04575"	transcript_id "lnc-bbut00010684"	exon_number "1"
12	lncRNA	exon	64591108	64591796	.	-	.	gene_id "lnc-bbug04575"	transcript_id "lnc-bbut00010684"	exon_number "2"
12	lncRNA	exon	64911714	64912300	.	-	.	gene_id "lnc-bbug04576"	transcript_id "lnc-bbut00010685"	exon_number "1"
12	lncRNA	exon	64948374	64948527	.	-	.	gene_id "lnc-bbug04576"	transcript_id "lnc-bbut00010685"	exon_number "2"
12	lncRNA	exon	64954324	64954601	.	-	.	gene_id "lnc-bbug04576"	transcript_id "lnc-bbut00010685"	exon_number "3"
12	lncRNA	exon	66152688	66156767	.	-	.	gene_id "lnc-bbug04577"	transcript_id "lnc-bbut00010686"	exon_number "1"
12	lncRNA	exon	66159617	66159672	.	-	.	gene_id "lnc-bbug04577"	transcript_id "lnc-bbut00010686"	exon_number "2"
12	lncRNA	exon	66159980	66160046	.	-	.	gene_id "lnc-bbug04577"	transcript_id "lnc-bbut00010686"	exon_number "3"
12	lncRNA	exon	66160792	66160938	.	-	.	gene_id "lnc-bbug04577"	transcript_id "lnc-bbut00010686"	exon_number "4"
12	lncRNA	exon	66166399	66166993	.	-	.	gene_id "lnc-bbug04577"	transcript_id "lnc-bbut00010686"	exon_number "5"
12	lncRNA	exon	66152688	66156767	.	-	.	gene_id "lnc-bbug04577"	transcript_id "lnc-bbut00010687"	exon_number "1"
12	lncRNA	exon	66159617	66159672	.	-	.	gene_id "lnc-bbug04577"	transcript_id "lnc-bbut00010687"	exon_number "2"
12	lncRNA	exon	66159980	66160046	.	-	.	gene_id "lnc-bbug04577"	transcript_id "lnc-bbut00010687"	exon_number "3"
12	lncRNA	exon	66160792	66160938	.	-	.	gene_id "lnc-bbug04577"	transcript_id "lnc-bbut00010687"	exon_number "4"
12	lncRNA	exon	66173148	66173690	.	-	.	gene_id "lnc-bbug04577"	transcript_id "lnc-bbut00010687"	exon_number "5"
12	lncRNA	exon	66152688	66156767	.	-	.	gene_id "lnc-bbug04577"	transcript_id "lnc-bbut00010688"	exon_number "1"
12	lncRNA	exon	66159617	66159672	.	-	.	gene_id "lnc-bbug04577"	transcript_id "lnc-bbut00010688"	exon_number "2"
12	lncRNA	exon	66159980	66160046	.	-	.	gene_id "lnc-bbug04577"	transcript_id "lnc-bbut00010688"	exon_number "3"
12	lncRNA	exon	66160792	66160938	.	-	.	gene_id "lnc-bbug04577"	transcript_id "lnc-bbut00010688"	exon_number "4"
12	lncRNA	exon	66175357	66175421	.	-	.	gene_id "lnc-bbug04577"	transcript_id "lnc-bbut00010688"	exon_number "5"
12	lncRNA	exon	66152688	66156767	.	-	.	gene_id "lnc-bbug04577"	transcript_id "lnc-bbut00010689"	exon_number "1"
12	lncRNA	exon	66159617	66159672	.	-	.	gene_id "lnc-bbug04577"	transcript_id "lnc-bbut00010689"	exon_number "2"
12	lncRNA	exon	66159980	66160046	.	-	.	gene_id "lnc-bbug04577"	transcript_id "lnc-bbut00010689"	exon_number "3"
12	lncRNA	exon	66160792	66160938	.	-	.	gene_id "lnc-bbug04577"	transcript_id "lnc-bbut00010689"	exon_number "4"
12	lncRNA	exon	66175348	66175443	.	-	.	gene_id "lnc-bbug04577"	transcript_id "lnc-bbut00010689"	exon_number "5"
12	lncRNA	exon	66933835	66936485	.	-	.	gene_id "lnc-bbug04584"	transcript_id "lnc-bbut00010701"	exon_number "1"
12	lncRNA	exon	66939743	66939844	.	-	.	gene_id "lnc-bbug04584"	transcript_id "lnc-bbut00010701"	exon_number "2"
12	lncRNA	exon	66942168	66943688	.	-	.	gene_id "lnc-bbug04584"	transcript_id "lnc-bbut00010701"	exon_number "3"
12	lncRNA	exon	68009960	68010797	.	-	.	gene_id "lnc-bbug04591"	transcript_id "lnc-bbut00010727"	exon_number "1"
12	lncRNA	exon	68023722	68023869	.	-	.	gene_id "lnc-bbug04591"	transcript_id "lnc-bbut00010727"	exon_number "2"
12	lncRNA	exon	68040131	68040235	.	-	.	gene_id "lnc-bbug04591"	transcript_id "lnc-bbut00010727"	exon_number "3"
12	lncRNA	exon	68049383	68049441	.	-	.	gene_id "lnc-bbug04591"	transcript_id "lnc-bbut00010727"	exon_number "4"
12	lncRNA	exon	68050557	68050737	.	-	.	gene_id "lnc-bbug04591"	transcript_id "lnc-bbut00010727"	exon_number "5"
12	lncRNA	exon	68066197	68066971	.	-	.	gene_id "lnc-bbug04591"	transcript_id "lnc-bbut00010727"	exon_number "6"
12	lncRNA	exon	68009960	68010797	.	-	.	gene_id "lnc-bbug04591"	transcript_id "lnc-bbut00010728"	exon_number "1"
12	lncRNA	exon	68023722	68023869	.	-	.	gene_id "lnc-bbug04591"	transcript_id "lnc-bbut00010728"	exon_number "2"
12	lncRNA	exon	68040131	68040235	.	-	.	gene_id "lnc-bbug04591"	transcript_id "lnc-bbut00010728"	exon_number "3"
12	lncRNA	exon	68049383	68049441	.	-	.	gene_id "lnc-bbug04591"	transcript_id "lnc-bbut00010728"	exon_number "4"

12	lncRNA	exon	68050557	68050737	.	-	.	gene_id "lnc-bbug04591"	transcript_id "lnc-bbut00010728"	exon_number "5"
12	lncRNA	exon	68053049	68053113	.	-	.	gene_id "lnc-bbug04591"	transcript_id "lnc-bbut00010728"	exon_number "6"
12	lncRNA	exon	68066197	68066973	.	-	.	gene_id "lnc-bbug04591"	transcript_id "lnc-bbut00010728"	exon_number "7"
12	lncRNA	exon	68009960	68010797	.	-	.	gene_id "lnc-bbug04591"	transcript_id "lnc-bbut00010730"	exon_number "1"
12	lncRNA	exon	68023722	68023869	.	-	.	gene_id "lnc-bbug04591"	transcript_id "lnc-bbut00010730"	exon_number "2"
12	lncRNA	exon	68040131	68040235	.	-	.	gene_id "lnc-bbug04591"	transcript_id "lnc-bbut00010730"	exon_number "3"
12	lncRNA	exon	68049383	68049441	.	-	.	gene_id "lnc-bbug04591"	transcript_id "lnc-bbut00010730"	exon_number "4"
12	lncRNA	exon	68050557	68050776	.	-	.	gene_id "lnc-bbug04591"	transcript_id "lnc-bbut00010730"	exon_number "5"
12	lncRNA	exon	68053049	68053113	.	-	.	gene_id "lnc-bbug04591"	transcript_id "lnc-bbut00010730"	exon_number "6"
12	lncRNA	exon	68066197	68066977	.	-	.	gene_id "lnc-bbug04591"	transcript_id "lnc-bbut00010730"	exon_number "7"
12	lncRNA	exon	68009960	68010797	.	-	.	gene_id "lnc-bbug04591"	transcript_id "lnc-bbut00010729"	exon_number "1"
12	lncRNA	exon	68023722	68023869	.	-	.	gene_id "lnc-bbug04591"	transcript_id "lnc-bbut00010729"	exon_number "2"
12	lncRNA	exon	68040131	68040235	.	-	.	gene_id "lnc-bbug04591"	transcript_id "lnc-bbut00010729"	exon_number "3"
12	lncRNA	exon	68049383	68049441	.	-	.	gene_id "lnc-bbug04591"	transcript_id "lnc-bbut00010729"	exon_number "4"
12	lncRNA	exon	68050557	68050737	.	-	.	gene_id "lnc-bbug04591"	transcript_id "lnc-bbut00010729"	exon_number "5"
12	lncRNA	exon	68064177	68064338	.	-	.	gene_id "lnc-bbug04591"	transcript_id "lnc-bbut00010729"	exon_number "6"
12	lncRNA	exon	68066197	68066977	.	-	.	gene_id "lnc-bbug04591"	transcript_id "lnc-bbut00010729"	exon_number "7"
12	lncRNA	exon	68009960	68010797	.	-	.	gene_id "lnc-bbug04591"	transcript_id "lnc-bbut00010731"	exon_number "1"
12	lncRNA	exon	68023722	68023869	.	-	.	gene_id "lnc-bbug04591"	transcript_id "lnc-bbut00010731"	exon_number "2"
12	lncRNA	exon	68040131	68040235	.	-	.	gene_id "lnc-bbug04591"	transcript_id "lnc-bbut00010731"	exon_number "3"
12	lncRNA	exon	68049383	68049441	.	-	.	gene_id "lnc-bbug04591"	transcript_id "lnc-bbut00010731"	exon_number "4"
12	lncRNA	exon	68050557	68050737	.	-	.	gene_id "lnc-bbug04591"	transcript_id "lnc-bbut00010731"	exon_number "5"
12	lncRNA	exon	68053049	68053113	.	-	.	gene_id "lnc-bbug04591"	transcript_id "lnc-bbut00010731"	exon_number "6"
12	lncRNA	exon	68064177	68064338	.	-	.	gene_id "lnc-bbug04591"	transcript_id "lnc-bbut00010731"	exon_number "7"
12	lncRNA	exon	68066197	68066978	.	-	.	gene_id "lnc-bbug04591"	transcript_id "lnc-bbut00010731"	exon_number "8"
12	lncRNA	exon	68009960	68010797	.	-	.	gene_id "lnc-bbug04591"	transcript_id "lnc-bbut00010732"	exon_number "1"
12	lncRNA	exon	68023722	68023869	.	-	.	gene_id "lnc-bbug04591"	transcript_id "lnc-bbut00010732"	exon_number "2"
12	lncRNA	exon	68040131	68040235	.	-	.	gene_id "lnc-bbug04591"	transcript_id "lnc-bbut00010732"	exon_number "3"
12	lncRNA	exon	68049383	68049441	.	-	.	gene_id "lnc-bbug04591"	transcript_id "lnc-bbut00010732"	exon_number "4"
12	lncRNA	exon	68050557	68050737	.	-	.	gene_id "lnc-bbug04591"	transcript_id "lnc-bbut00010732"	exon_number "5"
12	lncRNA	exon	68053049	68053113	.	-	.	gene_id "lnc-bbug04591"	transcript_id "lnc-bbut00010732"	exon_number "6"
12	lncRNA	exon	68066197	68066287	.	-	.	gene_id "lnc-bbug04591"	transcript_id "lnc-bbut00010732"	exon_number "7"
12	lncRNA	exon	68066698	68066979	.	-	.	gene_id "lnc-bbug04591"	transcript_id "lnc-bbut00010732"	exon_number "8"
12	lncRNA	exon	68009960	68010797	.	-	.	gene_id "lnc-bbug04591"	transcript_id "lnc-bbut00010734"	exon_number "1"
12	lncRNA	exon	68023722	68023869	.	-	.	gene_id "lnc-bbug04591"	transcript_id "lnc-bbut00010734"	exon_number "2"
12	lncRNA	exon	68040131	68040235	.	-	.	gene_id "lnc-bbug04591"	transcript_id "lnc-bbut00010734"	exon_number "3"
12	lncRNA	exon	68049383	68049441	.	-	.	gene_id "lnc-bbug04591"	transcript_id "lnc-bbut00010734"	exon_number "4"
12	lncRNA	exon	68050557	68050737	.	-	.	gene_id "lnc-bbug04591"	transcript_id "lnc-bbut00010734"	exon_number "5"
12	lncRNA	exon	68053049	68053113	.	-	.	gene_id "lnc-bbug04591"	transcript_id "lnc-bbut00010734"	exon_number "6"
12	lncRNA	exon	68064177	68064338	.	-	.	gene_id "lnc-bbug04591"	transcript_id "lnc-bbut00010734"	exon_number "7"
12	lncRNA	exon	68066197	68066287	.	-	.	gene_id "lnc-bbug04591"	transcript_id "lnc-bbut00010734"	exon_number "8"
12	lncRNA	exon	68066698	68066981	.	-	.	gene_id "lnc-bbug04591"	transcript_id "lnc-bbut00010734"	exon_number "9"
12	lncRNA	exon	68009960	68010797	.	-	.	gene_id "lnc-bbug04591"	transcript_id "lnc-bbut00010733"	exon_number "1"
12	lncRNA	exon	68023722	68023869	.	-	.	gene_id "lnc-bbug04591"	transcript_id "lnc-bbut00010733"	exon_number "2"
12	lncRNA	exon	68040131	68040235	.	-	.	gene_id "lnc-bbug04591"	transcript_id "lnc-bbut00010733"	exon_number "3"
12	lncRNA	exon	68049383	68049441	.	-	.	gene_id "lnc-bbug04591"	transcript_id "lnc-bbut00010733"	exon_number "4"
12	lncRNA	exon	68050557	68050776	.	-	.	gene_id "lnc-bbug04591"	transcript_id "lnc-bbut00010733"	exon_number "5"
12	lncRNA	exon	68053049	68053113	.	-	.	gene_id "lnc-bbug04591"	transcript_id "lnc-bbut00010733"	exon_number "6"
12	lncRNA	exon	68064177	68064338	.	-	.	gene_id "lnc-bbug04591"	transcript_id "lnc-bbut00010733"	exon_number "7"
12	lncRNA	exon	68066197	68066981	.	-	.	gene_id "lnc-bbug04591"	transcript_id "lnc-bbut00010733"	exon_number "8"
12	lncRNA	exon	68009960	68010797	.	-	.	gene_id "lnc-bbug04591"	transcript_id "lnc-bbut00010735"	exon_number "1"
12	lncRNA	exon	68023722	68023869	.	-	.	gene_id "lnc-bbug04591"	transcript_id "lnc-bbut00010735"	exon_number "2"

12	lncRNA	exon	68040131	68040235	.	-	.	gene_id "lnc-bbug04591"	transcript_id "lnc-bbut00010735"	exon_number "3"
12	lncRNA	exon	68049383	68049441	.	-	.	gene_id "lnc-bbug04591"	transcript_id "lnc-bbut00010735"	exon_number "4"
12	lncRNA	exon	68050557	68050737	.	-	.	gene_id "lnc-bbug04591"	transcript_id "lnc-bbut00010735"	exon_number "5"
12	lncRNA	exon	68053049	68053113	.	-	.	gene_id "lnc-bbug04591"	transcript_id "lnc-bbut00010735"	exon_number "6"
12	lncRNA	exon	68064177	68064338	.	-	.	gene_id "lnc-bbug04591"	transcript_id "lnc-bbut00010735"	exon_number "7"
12	lncRNA	exon	68065173	68065255	.	-	.	gene_id "lnc-bbug04591"	transcript_id "lnc-bbut00010735"	exon_number "8"
12	lncRNA	exon	68066197	68066982	.	-	.	gene_id "lnc-bbug04591"	transcript_id "lnc-bbut00010735"	exon_number "9"
12	lncRNA	exon	68009960	68010797	.	-	.	gene_id "lnc-bbug04591"	transcript_id "lnc-bbut00010736"	exon_number "1"
12	lncRNA	exon	68023722	68023869	.	-	.	gene_id "lnc-bbug04591"	transcript_id "lnc-bbut00010736"	exon_number "2"
12	lncRNA	exon	68040131	68040235	.	-	.	gene_id "lnc-bbug04591"	transcript_id "lnc-bbut00010736"	exon_number "3"
12	lncRNA	exon	68049383	68049441	.	-	.	gene_id "lnc-bbug04591"	transcript_id "lnc-bbut00010736"	exon_number "4"
12	lncRNA	exon	68050557	68050737	.	-	.	gene_id "lnc-bbug04591"	transcript_id "lnc-bbut00010736"	exon_number "5"
12	lncRNA	exon	68053049	68053113	.	-	.	gene_id "lnc-bbug04591"	transcript_id "lnc-bbut00010736"	exon_number "6"
12	lncRNA	exon	68064177	68064338	.	-	.	gene_id "lnc-bbug04591"	transcript_id "lnc-bbut00010736"	exon_number "7"
12	lncRNA	exon	68065173	68065255	.	-	.	gene_id "lnc-bbug04591"	transcript_id "lnc-bbut00010736"	exon_number "8"
12	lncRNA	exon	68066197	68066287	.	-	.	gene_id "lnc-bbug04591"	transcript_id "lnc-bbut00010736"	exon_number "9"
12	lncRNA	exon	68066698	68066985	.	-	.	gene_id "lnc-bbug04591"	transcript_id "lnc-bbut00010736"	exon_number "10"
12	lncRNA	exon	68009960	68010797	.	-	.	gene_id "lnc-bbug04591"	transcript_id "lnc-bbut00010738"	exon_number "1"
12	lncRNA	exon	68023722	68023869	.	-	.	gene_id "lnc-bbug04591"	transcript_id "lnc-bbut00010738"	exon_number "2"
12	lncRNA	exon	68040131	68040235	.	-	.	gene_id "lnc-bbug04591"	transcript_id "lnc-bbut00010738"	exon_number "3"
12	lncRNA	exon	68049383	68049441	.	-	.	gene_id "lnc-bbug04591"	transcript_id "lnc-bbut00010738"	exon_number "4"
12	lncRNA	exon	68050557	68050737	.	-	.	gene_id "lnc-bbug04591"	transcript_id "lnc-bbut00010738"	exon_number "5"
12	lncRNA	exon	68053049	68053113	.	-	.	gene_id "lnc-bbug04591"	transcript_id "lnc-bbut00010738"	exon_number "6"
12	lncRNA	exon	68066197	68066287	.	-	.	gene_id "lnc-bbug04591"	transcript_id "lnc-bbut00010738"	exon_number "7"
12	lncRNA	exon	68067038	68067862	.	-	.	gene_id "lnc-bbug04591"	transcript_id "lnc-bbut00010738"	exon_number "8"
12	lncRNA	exon	68009960	68010797	.	-	.	gene_id "lnc-bbug04591"	transcript_id "lnc-bbut00010737"	exon_number "1"
12	lncRNA	exon	68023722	68023869	.	-	.	gene_id "lnc-bbug04591"	transcript_id "lnc-bbut00010737"	exon_number "2"
12	lncRNA	exon	68040131	68040235	.	-	.	gene_id "lnc-bbug04591"	transcript_id "lnc-bbut00010737"	exon_number "3"
12	lncRNA	exon	68049383	68049441	.	-	.	gene_id "lnc-bbug04591"	transcript_id "lnc-bbut00010737"	exon_number "4"
12	lncRNA	exon	68050557	68050737	.	-	.	gene_id "lnc-bbug04591"	transcript_id "lnc-bbut00010737"	exon_number "5"
12	lncRNA	exon	68053049	68053113	.	-	.	gene_id "lnc-bbug04591"	transcript_id "lnc-bbut00010737"	exon_number "6"
12	lncRNA	exon	68066197	68066287	.	-	.	gene_id "lnc-bbug04591"	transcript_id "lnc-bbut00010737"	exon_number "7"
12	lncRNA	exon	68066999	68067862	.	-	.	gene_id "lnc-bbug04591"	transcript_id "lnc-bbut00010737"	exon_number "8"
12	lncRNA	exon	68009960	68010797	.	-	.	gene_id "lnc-bbug04591"	transcript_id "lnc-bbut00010740"	exon_number "1"
12	lncRNA	exon	68023722	68023869	.	-	.	gene_id "lnc-bbug04591"	transcript_id "lnc-bbut00010740"	exon_number "2"
12	lncRNA	exon	68040131	68040235	.	-	.	gene_id "lnc-bbug04591"	transcript_id "lnc-bbut00010740"	exon_number "3"
12	lncRNA	exon	68049383	68049441	.	-	.	gene_id "lnc-bbug04591"	transcript_id "lnc-bbut00010740"	exon_number "4"
12	lncRNA	exon	68050557	68050737	.	-	.	gene_id "lnc-bbug04591"	transcript_id "lnc-bbut00010740"	exon_number "5"
12	lncRNA	exon	68053049	68053113	.	-	.	gene_id "lnc-bbug04591"	transcript_id "lnc-bbut00010740"	exon_number "6"
12	lncRNA	exon	68064177	68064338	.	-	.	gene_id "lnc-bbug04591"	transcript_id "lnc-bbut00010740"	exon_number "7"
12	lncRNA	exon	68066197	68066287	.	-	.	gene_id "lnc-bbug04591"	transcript_id "lnc-bbut00010740"	exon_number "8"
12	lncRNA	exon	68067038	68067863	.	-	.	gene_id "lnc-bbug04591"	transcript_id "lnc-bbut00010740"	exon_number "9"
12	lncRNA	exon	68009960	68010797	.	-	.	gene_id "lnc-bbug04591"	transcript_id "lnc-bbut00010739"	exon_number "1"
12	lncRNA	exon	68023722	68023869	.	-	.	gene_id "lnc-bbug04591"	transcript_id "lnc-bbut00010739"	exon_number "2"
12	lncRNA	exon	68040131	68040235	.	-	.	gene_id "lnc-bbug04591"	transcript_id "lnc-bbut00010739"	exon_number "3"
12	lncRNA	exon	68049383	68049441	.	-	.	gene_id "lnc-bbug04591"	transcript_id "lnc-bbut00010739"	exon_number "4"
12	lncRNA	exon	68050557	68050737	.	-	.	gene_id "lnc-bbug04591"	transcript_id "lnc-bbut00010739"	exon_number "5"
12	lncRNA	exon	68053049	68053113	.	-	.	gene_id "lnc-bbug04591"	transcript_id "lnc-bbut00010739"	exon_number "6"
12	lncRNA	exon	68064177	68064338	.	-	.	gene_id "lnc-bbug04591"	transcript_id "lnc-bbut00010739"	exon_number "7"
12	lncRNA	exon	68066197	68066287	.	-	.	gene_id "lnc-bbug04591"	transcript_id "lnc-bbut00010739"	exon_number "8"
12	lncRNA	exon	68066999	68067863	.	-	.	gene_id "lnc-bbug04591"	transcript_id "lnc-bbut00010739"	exon_number "9"
12	lncRNA	exon	68009960	68010797	.	-	.	gene_id "lnc-bbug04591"	transcript_id "lnc-bbut00010741"	exon_number "1"

12	lncRNA	exon	68023722	68023869	.	-	.	gene_id "lnc-bbug04591"	transcript_id "lnc-bbut00010741"	exon_number "2"
12	lncRNA	exon	68040131	68040235	.	-	.	gene_id "lnc-bbug04591"	transcript_id "lnc-bbut00010741"	exon_number "3"
12	lncRNA	exon	68049383	68049441	.	-	.	gene_id "lnc-bbug04591"	transcript_id "lnc-bbut00010741"	exon_number "4"
12	lncRNA	exon	68050557	68050737	.	-	.	gene_id "lnc-bbug04591"	transcript_id "lnc-bbut00010741"	exon_number "5"
12	lncRNA	exon	68053049	68053113	.	-	.	gene_id "lnc-bbug04591"	transcript_id "lnc-bbut00010741"	exon_number "6"
12	lncRNA	exon	68064177	68064338	.	-	.	gene_id "lnc-bbug04591"	transcript_id "lnc-bbut00010741"	exon_number "7"
12	lncRNA	exon	68065173	68065255	.	-	.	gene_id "lnc-bbug04591"	transcript_id "lnc-bbut00010741"	exon_number "8"
12	lncRNA	exon	68066197	68066287	.	-	.	gene_id "lnc-bbug04591"	transcript_id "lnc-bbut00010741"	exon_number "9"
12	lncRNA	exon	68067038	68067866	.	-	.	gene_id "lnc-bbug04591"	transcript_id "lnc-bbut00010741"	exon_number "10"
12	lncRNA	exon	68265438	68265638	.	-	.	gene_id "lnc-bbug04595"	transcript_id "lnc-bbut00010762"	exon_number "1"
12	lncRNA	exon	68269629	68269753	.	-	.	gene_id "lnc-bbug04595"	transcript_id "lnc-bbut00010762"	exon_number "2"
12	lncRNA	exon	68270478	68270500	.	-	.	gene_id "lnc-bbug04595"	transcript_id "lnc-bbut00010762"	exon_number "3"
12	lncRNA	exon	68273938	68274077	.	-	.	gene_id "lnc-bbug04595"	transcript_id "lnc-bbut00010762"	exon_number "4"
12	lncRNA	exon	69277846	69280466	.	-	.	gene_id "lnc-bbug04598"	transcript_id "lnc-bbut00010766"	exon_number "1"
12	lncRNA	exon	69301521	69301623	.	-	.	gene_id "lnc-bbug04598"	transcript_id "lnc-bbut00010766"	exon_number "2"
12	lncRNA	exon	69304155	69304380	.	-	.	gene_id "lnc-bbug04598"	transcript_id "lnc-bbut00010766"	exon_number "3"
12	lncRNA	exon	69370726	69372681	.	-	.	gene_id "lnc-bbug04599"	transcript_id "lnc-bbut00010767"	exon_number "1"
12	lncRNA	exon	69374156	69374320	.	-	.	gene_id "lnc-bbug04599"	transcript_id "lnc-bbut00010767"	exon_number "2"
12	lncRNA	exon	69385313	69385465	.	-	.	gene_id "lnc-bbug04599"	transcript_id "lnc-bbut00010767"	exon_number "3"
12	lncRNA	exon	70570068	70570277	.	-	.	gene_id "lnc-bbug04604"	transcript_id "lnc-bbut00010777"	exon_number "1"
12	lncRNA	exon	70571215	70571901	.	-	.	gene_id "lnc-bbug04604"	transcript_id "lnc-bbut00010777"	exon_number "2"
12	lncRNA	exon	71039893	71040262	.	-	.	gene_id "lnc-bbug04609"	transcript_id "lnc-bbut00010793"	exon_number "1"
12	lncRNA	exon	71041340	71041412	.	-	.	gene_id "lnc-bbug04609"	transcript_id "lnc-bbut00010793"	exon_number "2"
12	lncRNA	exon	71043483	71043697	.	-	.	gene_id "lnc-bbug04609"	transcript_id "lnc-bbut00010793"	exon_number "3"
12	lncRNA	exon	71044154	71044258	.	-	.	gene_id "lnc-bbug04609"	transcript_id "lnc-bbut00010793"	exon_number "4"
12	lncRNA	exon	71039893	71040262	.	-	.	gene_id "lnc-bbug04609"	transcript_id "lnc-bbut00010792"	exon_number "1"
12	lncRNA	exon	71043483	71043697	.	-	.	gene_id "lnc-bbug04609"	transcript_id "lnc-bbut00010792"	exon_number "2"
12	lncRNA	exon	71044154	71044258	.	-	.	gene_id "lnc-bbug04609"	transcript_id "lnc-bbut00010792"	exon_number "3"
12	lncRNA	exon	71489142	71491875	.	-	.	gene_id "lnc-bbug04611"	transcript_id "lnc-bbut00010801"	exon_number "1"
12	lncRNA	exon	71492586	71492846	.	-	.	gene_id "lnc-bbug04611"	transcript_id "lnc-bbut00010801"	exon_number "2"
12	lncRNA	exon	71601488	71601791	.	-	.	gene_id "lnc-bbug04612"	transcript_id "lnc-bbut00010802"	exon_number "1"
12	lncRNA	exon	71607254	71607360	.	-	.	gene_id "lnc-bbug04612"	transcript_id "lnc-bbut00010802"	exon_number "2"
12	lncRNA	exon	71608991	71609009	.	-	.	gene_id "lnc-bbug04612"	transcript_id "lnc-bbut00010802"	exon_number "3"
12	lncRNA	exon	71609809	71609902	.	-	.	gene_id "lnc-bbug04612"	transcript_id "lnc-bbut00010802"	exon_number "4"
12	lncRNA	exon	71601488	71601791	.	-	.	gene_id "lnc-bbug04612"	transcript_id "lnc-bbut00010803"	exon_number "1"
12	lncRNA	exon	71607254	71607360	.	-	.	gene_id "lnc-bbug04612"	transcript_id "lnc-bbut00010803"	exon_number "2"
12	lncRNA	exon	71608991	71609009	.	-	.	gene_id "lnc-bbug04612"	transcript_id "lnc-bbut00010803"	exon_number "3"
12	lncRNA	exon	71609866	71609985	.	-	.	gene_id "lnc-bbug04612"	transcript_id "lnc-bbut00010803"	exon_number "4"
12	lncRNA	exon	71602509	71602603	.	-	.	gene_id "lnc-bbug04612"	transcript_id "lnc-bbut00010804"	exon_number "1"
12	lncRNA	exon	71607254	71607360	.	-	.	gene_id "lnc-bbug04612"	transcript_id "lnc-bbut00010804"	exon_number "2"
12	lncRNA	exon	71608991	71609009	.	-	.	gene_id "lnc-bbug04612"	transcript_id "lnc-bbut00010804"	exon_number "3"
12	lncRNA	exon	71609809	71610929	.	-	.	gene_id "lnc-bbug04612"	transcript_id "lnc-bbut00010804"	exon_number "4"
12	lncRNA	exon	71818210	71821045	.	-	.	gene_id "lnc-bbug04617"	transcript_id "lnc-bbut00010817"	exon_number "1"
12	lncRNA	exon	71821638	71821768	.	-	.	gene_id "lnc-bbug04617"	transcript_id "lnc-bbut00010817"	exon_number "2"
12	lncRNA	exon	71825746	71825895	.	-	.	gene_id "lnc-bbug04617"	transcript_id "lnc-bbut00010817"	exon_number "3"
12	lncRNA	exon	71836623	71836706	.	-	.	gene_id "lnc-bbug04617"	transcript_id "lnc-bbut00010817"	exon_number "4"
12	lncRNA	exon	71836799	71836903	.	-	.	gene_id "lnc-bbug04617"	transcript_id "lnc-bbut00010817"	exon_number "5"
12	lncRNA	exon	71837102	71837198	.	-	.	gene_id "lnc-bbug04617"	transcript_id "lnc-bbut00010817"	exon_number "6"
12	lncRNA	exon	71837590	71837668	.	-	.	gene_id "lnc-bbug04617"	transcript_id "lnc-bbut00010817"	exon_number "7"
12	lncRNA	exon	71837996	71838090	.	-	.	gene_id "lnc-bbug04617"	transcript_id "lnc-bbut00010817"	exon_number "8"
12	lncRNA	exon	71838330	71838451	.	-	.	gene_id "lnc-bbug04617"	transcript_id "lnc-bbut00010817"	exon_number "9"
12	lncRNA	exon	71839122	71839222	.	-	.	gene_id "lnc-bbug04617"	transcript_id "lnc-bbut00010817"	exon_number "10"

12	lncRNA	exon	71840560	71840664	.	-	.	gene_id "lnc-bbug04617"	transcript_id "lnc-bbut00010817"	exon_number "11"
12	lncRNA	exon	71842918	71843013	.	-	.	gene_id "lnc-bbug04617"	transcript_id "lnc-bbut00010817"	exon_number "12"
12	lncRNA	exon	71844008	71844009	.	-	.	gene_id "lnc-bbug04617"	transcript_id "lnc-bbut00010817"	exon_number "13"
12	lncRNA	exon	71844493	71844544	.	-	.	gene_id "lnc-bbug04617"	transcript_id "lnc-bbut00010817"	exon_number "14"
12	lncRNA	exon	71844732	71844795	.	-	.	gene_id "lnc-bbug04617"	transcript_id "lnc-bbut00010817"	exon_number "15"
12	lncRNA	exon	71845104	71845167	.	-	.	gene_id "lnc-bbug04617"	transcript_id "lnc-bbut00010817"	exon_number "16"
12	lncRNA	exon	71845551	71845614	.	-	.	gene_id "lnc-bbug04617"	transcript_id "lnc-bbut00010817"	exon_number "17"
12	lncRNA	exon	71846449	71846522	.	-	.	gene_id "lnc-bbug04617"	transcript_id "lnc-bbut00010817"	exon_number "18"
12	lncRNA	exon	71846699	71846838	.	-	.	gene_id "lnc-bbug04617"	transcript_id "lnc-bbut00010817"	exon_number "19"
12	lncRNA	exon	71847152	71848179	.	-	.	gene_id "lnc-bbug04617"	transcript_id "lnc-bbut00010817"	exon_number "20"
12	lncRNA	exon	72468370	72469604	.	-	.	gene_id "lnc-bbug04630"	transcript_id "lnc-bbut00010857"	exon_number "1"
12	lncRNA	exon	72470468	72470627	.	-	.	gene_id "lnc-bbug04630"	transcript_id "lnc-bbut00010857"	exon_number "2"
12	lncRNA	exon	72470912	72470989	.	-	.	gene_id "lnc-bbug04630"	transcript_id "lnc-bbut00010857"	exon_number "3"
12	lncRNA	exon	72471503	72471612	.	-	.	gene_id "lnc-bbug04630"	transcript_id "lnc-bbut00010857"	exon_number "4"
12	lncRNA	exon	72474561	72474819	.	-	.	gene_id "lnc-bbug04630"	transcript_id "lnc-bbut00010857"	exon_number "5"
12	lncRNA	exon	72468370	72469604	.	-	.	gene_id "lnc-bbug04630"	transcript_id "lnc-bbut00010858"	exon_number "1"
12	lncRNA	exon	72470443	72470627	.	-	.	gene_id "lnc-bbug04630"	transcript_id "lnc-bbut00010858"	exon_number "2"
12	lncRNA	exon	72470912	72470989	.	-	.	gene_id "lnc-bbug04630"	transcript_id "lnc-bbut00010858"	exon_number "3"
12	lncRNA	exon	72471503	72471612	.	-	.	gene_id "lnc-bbug04630"	transcript_id "lnc-bbut00010858"	exon_number "4"
12	lncRNA	exon	72474561	72474828	.	-	.	gene_id "lnc-bbug04630"	transcript_id "lnc-bbut00010858"	exon_number "5"
12	lncRNA	exon	73330419	73330587	.	-	.	gene_id "lnc-bbug04639"	transcript_id "lnc-bbut00010871"	exon_number "1"
12	lncRNA	exon	73345430	73345556	.	-	.	gene_id "lnc-bbug04639"	transcript_id "lnc-bbut00010871"	exon_number "2"
12	lncRNA	exon	73353695	73353766	.	-	.	gene_id "lnc-bbug04639"	transcript_id "lnc-bbut00010871"	exon_number "3"
12	lncRNA	exon	73358833	73358967	.	-	.	gene_id "lnc-bbug04639"	transcript_id "lnc-bbut00010871"	exon_number "4"
12	lncRNA	exon	73506877	73507092	.	-	.	gene_id "lnc-bbug04640"	transcript_id "lnc-bbut00010872"	exon_number "1"
12	lncRNA	exon	73507739	73508180	.	-	.	gene_id "lnc-bbug04640"	transcript_id "lnc-bbut00010872"	exon_number "2"
12	lncRNA	exon	73506877	73507092	.	-	.	gene_id "lnc-bbug04640"	transcript_id "lnc-bbut00010873"	exon_number "1"
12	lncRNA	exon	73507739	73507805	.	-	.	gene_id "lnc-bbug04640"	transcript_id "lnc-bbut00010873"	exon_number "2"
12	lncRNA	exon	73519554	73519595	.	-	.	gene_id "lnc-bbug04640"	transcript_id "lnc-bbut00010873"	exon_number "3"
12	lncRNA	exon	73545664	73546485	.	-	.	gene_id "lnc-bbug04641"	transcript_id "lnc-bbut00010875"	exon_number "1"
12	lncRNA	exon	73546664	73546860	.	-	.	gene_id "lnc-bbug04641"	transcript_id "lnc-bbut00010875"	exon_number "2"
12	lncRNA	exon	73547349	73547879	.	-	.	gene_id "lnc-bbug04641"	transcript_id "lnc-bbut00010875"	exon_number "3"
12	lncRNA	exon	73545664	73546485	.	-	.	gene_id "lnc-bbug04641"	transcript_id "lnc-bbut00010874"	exon_number "1"
12	lncRNA	exon	73546664	73546768	.	-	.	gene_id "lnc-bbug04641"	transcript_id "lnc-bbut00010874"	exon_number "2"
12	lncRNA	exon	73547349	73547879	.	-	.	gene_id "lnc-bbug04641"	transcript_id "lnc-bbut00010874"	exon_number "3"
12	lncRNA	exon	73614987	73616474	.	-	.	gene_id "lnc-bbug04642"	transcript_id "lnc-bbut00010876"	exon_number "1"
12	lncRNA	exon	73617839	73617951	.	-	.	gene_id "lnc-bbug04642"	transcript_id "lnc-bbut00010876"	exon_number "2"
12	lncRNA	exon	73618260	73620856	.	-	.	gene_id "lnc-bbug04643"	transcript_id "lnc-bbut00010878"	exon_number "1"
12	lncRNA	exon	73620974	73621062	.	-	.	gene_id "lnc-bbug04643"	transcript_id "lnc-bbut00010878"	exon_number "2"
12	lncRNA	exon	73621166	73621263	.	-	.	gene_id "lnc-bbug04643"	transcript_id "lnc-bbut00010878"	exon_number "3"
12	lncRNA	exon	73621639	73621843	.	-	.	gene_id "lnc-bbug04643"	transcript_id "lnc-bbut00010878"	exon_number "4"
12	lncRNA	exon	73618260	73620856	.	-	.	gene_id "lnc-bbug04643"	transcript_id "lnc-bbut00010877"	exon_number "1"
12	lncRNA	exon	73620974	73621062	.	-	.	gene_id "lnc-bbug04643"	transcript_id "lnc-bbut00010877"	exon_number "2"
12	lncRNA	exon	73621166	73621843	.	-	.	gene_id "lnc-bbug04643"	transcript_id "lnc-bbut00010877"	exon_number "3"
12	lncRNA	exon	73618260	73620856	.	-	.	gene_id "lnc-bbug04643"	transcript_id "lnc-bbut00010879"	exon_number "1"
12	lncRNA	exon	73620974	73621062	.	-	.	gene_id "lnc-bbug04643"	transcript_id "lnc-bbut00010879"	exon_number "2"
12	lncRNA	exon	73621166	73621263	.	-	.	gene_id "lnc-bbug04643"	transcript_id "lnc-bbut00010879"	exon_number "3"
12	lncRNA	exon	73632054	73633527	.	-	.	gene_id "lnc-bbug04643"	transcript_id "lnc-bbut00010879"	exon_number "4"
12	lncRNA	exon	73786631	73786975	.	-	.	gene_id "lnc-bbug04645"	transcript_id "lnc-bbut00010881"	exon_number "1"
12	lncRNA	exon	74083174	74083411	.	-	.	gene_id "lnc-bbug04647"	transcript_id "lnc-bbut00010890"	exon_number "1"
12	lncRNA	exon	74083802	74083928	.	-	.	gene_id "lnc-bbug04647"	transcript_id "lnc-bbut00010890"	exon_number "2"
12	lncRNA	exon	74088672	74088783	.	-	.	gene_id "lnc-bbug04647"	transcript_id "lnc-bbut00010890"	exon_number "3"

12	lncRNA	exon	74672285	74672893	.	-	.	gene_id "lnc-bbug04652"	transcript_id "lnc-bbut00010907"	exon_number "1"
12	lncRNA	exon	74673932	74674044	.	-	.	gene_id "lnc-bbug04652"	transcript_id "lnc-bbut00010907"	exon_number "2"
12	lncRNA	exon	74679040	74679087	.	-	.	gene_id "lnc-bbug04652"	transcript_id "lnc-bbut00010907"	exon_number "3"
12	lncRNA	exon	74679197	74679395	.	-	.	gene_id "lnc-bbug04652"	transcript_id "lnc-bbut00010907"	exon_number "4"
12	lncRNA	exon	74672285	74672938	.	-	.	gene_id "lnc-bbug04652"	transcript_id "lnc-bbut00010909"	exon_number "1"
12	lncRNA	exon	74673932	74674044	.	-	.	gene_id "lnc-bbug04652"	transcript_id "lnc-bbut00010909"	exon_number "2"
12	lncRNA	exon	74679040	74679087	.	-	.	gene_id "lnc-bbug04652"	transcript_id "lnc-bbut00010909"	exon_number "3"
12	lncRNA	exon	74683081	74683277	.	-	.	gene_id "lnc-bbug04652"	transcript_id "lnc-bbut00010909"	exon_number "4"
12	lncRNA	exon	74672285	74672893	.	-	.	gene_id "lnc-bbug04652"	transcript_id "lnc-bbut00010910"	exon_number "1"
12	lncRNA	exon	74673932	74674044	.	-	.	gene_id "lnc-bbug04652"	transcript_id "lnc-bbut00010910"	exon_number "2"
12	lncRNA	exon	74679040	74679087	.	-	.	gene_id "lnc-bbug04652"	transcript_id "lnc-bbut00010910"	exon_number "3"
12	lncRNA	exon	74685337	74685367	.	-	.	gene_id "lnc-bbug04652"	transcript_id "lnc-bbut00010910"	exon_number "4"
12	lncRNA	exon	74672285	74672893	.	-	.	gene_id "lnc-bbug04652"	transcript_id "lnc-bbut00010911"	exon_number "1"
12	lncRNA	exon	74673932	74674044	.	-	.	gene_id "lnc-bbug04652"	transcript_id "lnc-bbut00010911"	exon_number "2"
12	lncRNA	exon	74679040	74679087	.	-	.	gene_id "lnc-bbug04652"	transcript_id "lnc-bbut00010911"	exon_number "3"
12	lncRNA	exon	74685492	74685575	.	-	.	gene_id "lnc-bbug04652"	transcript_id "lnc-bbut00010911"	exon_number "4"
12	lncRNA	exon	78617508	78617863	.	-	.	gene_id "lnc-bbug04664"	transcript_id "lnc-bbut00010928"	exon_number "1"
12	lncRNA	exon	78617980	78618219	.	-	.	gene_id "lnc-bbug04664"	transcript_id "lnc-bbut00010928"	exon_number "2"
12	lncRNA	exon	78621050	78621128	.	-	.	gene_id "lnc-bbug04664"	transcript_id "lnc-bbut00010928"	exon_number "3"
12	lncRNA	exon	78621615	78621746	.	-	.	gene_id "lnc-bbug04664"	transcript_id "lnc-bbut00010928"	exon_number "4"
12	lncRNA	exon	82276227	82276907	.	-	.	gene_id "lnc-bbug04670"	transcript_id "lnc-bbut00010940"	exon_number "1"
12	lncRNA	exon	82277626	82277814	.	-	.	gene_id "lnc-bbug04670"	transcript_id "lnc-bbut00010940"	exon_number "2"
12	lncRNA	exon	82317666	82317786	.	-	.	gene_id "lnc-bbug04670"	transcript_id "lnc-bbut00010940"	exon_number "3"
12	lncRNA	exon	82977657	82977804	.	-	.	gene_id "lnc-bbug04673"	transcript_id "lnc-bbut00010944"	exon_number "1"
12	lncRNA	exon	82978709	82978964	.	-	.	gene_id "lnc-bbug04673"	transcript_id "lnc-bbut00010944"	exon_number "2"
12	lncRNA	exon	84236427	84237768	.	-	.	gene_id "lnc-bbug04676"	transcript_id "lnc-bbut00010948"	exon_number "1"
12	lncRNA	exon	84556142	84556735	.	-	.	gene_id "lnc-bbug04677"	transcript_id "lnc-bbut00010950"	exon_number "1"
12	lncRNA	exon	84557484	84558586	.	-	.	gene_id "lnc-bbug04677"	transcript_id "lnc-bbut00010950"	exon_number "2"
12	lncRNA	exon	84559219	84561539	.	-	.	gene_id "lnc-bbug04677"	transcript_id "lnc-bbut00010950"	exon_number "3"
12	lncRNA	exon	84564175	84564310	.	-	.	gene_id "lnc-bbug04677"	transcript_id "lnc-bbut00010950"	exon_number "4"
12	lncRNA	exon	84564488	84564567	.	-	.	gene_id "lnc-bbug04677"	transcript_id "lnc-bbut00010950"	exon_number "5"
12	lncRNA	exon	84564757	84566458	.	-	.	gene_id "lnc-bbug04677"	transcript_id "lnc-bbut00010950"	exon_number "6"
12	lncRNA	exon	84556142	84558586	.	-	.	gene_id "lnc-bbug04677"	transcript_id "lnc-bbut00010949"	exon_number "1"
12	lncRNA	exon	84559219	84561539	.	-	.	gene_id "lnc-bbug04677"	transcript_id "lnc-bbut00010949"	exon_number "2"
12	lncRNA	exon	84564175	84564310	.	-	.	gene_id "lnc-bbug04677"	transcript_id "lnc-bbut00010949"	exon_number "3"
12	lncRNA	exon	84564488	84564567	.	-	.	gene_id "lnc-bbug04677"	transcript_id "lnc-bbut00010949"	exon_number "4"
12	lncRNA	exon	84564757	84566458	.	-	.	gene_id "lnc-bbug04677"	transcript_id "lnc-bbut00010949"	exon_number "5"
12	lncRNA	exon	84556142	84558586	.	-	.	gene_id "lnc-bbug04677"	transcript_id "lnc-bbut00010951"	exon_number "1"
12	lncRNA	exon	84559219	84561539	.	-	.	gene_id "lnc-bbug04677"	transcript_id "lnc-bbut00010951"	exon_number "2"
12	lncRNA	exon	84564175	84564310	.	-	.	gene_id "lnc-bbug04677"	transcript_id "lnc-bbut00010951"	exon_number "3"
12	lncRNA	exon	84564488	84564567	.	-	.	gene_id "lnc-bbug04677"	transcript_id "lnc-bbut00010951"	exon_number "4"
12	lncRNA	exon	84590326	84590468	.	-	.	gene_id "lnc-bbug04677"	transcript_id "lnc-bbut00010951"	exon_number "5"
12	lncRNA	exon	84850279	84851541	.	-	.	gene_id "lnc-bbug04679"	transcript_id "lnc-bbut00010953"	exon_number "1"
12	lncRNA	exon	84869924	84870067	.	-	.	gene_id "lnc-bbug04679"	transcript_id "lnc-bbut00010953"	exon_number "2"
12	lncRNA	exon	84872954	84873720	.	-	.	gene_id "lnc-bbug04679"	transcript_id "lnc-bbut00010953"	exon_number "3"
12	lncRNA	exon	84854815	84854917	.	-	.	gene_id "lnc-bbug04679"	transcript_id "lnc-bbut00010954"	exon_number "1"
12	lncRNA	exon	84869924	84870067	.	-	.	gene_id "lnc-bbug04679"	transcript_id "lnc-bbut00010954"	exon_number "2"
12	lncRNA	exon	84872954	84873720	.	-	.	gene_id "lnc-bbug04679"	transcript_id "lnc-bbut00010954"	exon_number "3"
12	lncRNA	exon	86357077	86357695	.	-	.	gene_id "lnc-bbug04685"	transcript_id "lnc-bbut00010961"	exon_number "1"
12	lncRNA	exon	86359737	86359898	.	-	.	gene_id "lnc-bbug04685"	transcript_id "lnc-bbut00010961"	exon_number "2"
12	lncRNA	exon	86367954	86368101	.	-	.	gene_id "lnc-bbug04685"	transcript_id "lnc-bbut00010961"	exon_number "3"
12	lncRNA	exon	86369123	86369400	.	-	.	gene_id "lnc-bbug04685"	transcript_id "lnc-bbut00010961"	exon_number "4"

12	lncRNA	exon	87329097	87329330	.	-	.	gene_id "lnc-bbug04692"	transcript_id "lnc-bbut00010982"	exon_number "1"
12	lncRNA	exon	87330015	87330212	.	-	.	gene_id "lnc-bbug04692"	transcript_id "lnc-bbut00010982"	exon_number "2"
12	lncRNA	exon	87330385	87330612	.	-	.	gene_id "lnc-bbug04692"	transcript_id "lnc-bbut00010982"	exon_number "3"
12	lncRNA	exon	87471954	87472510	.	-	.	gene_id "lnc-bbug04694"	transcript_id "lnc-bbut00010984"	exon_number "1"
12	lncRNA	exon	87472986	87473122	.	-	.	gene_id "lnc-bbug04694"	transcript_id "lnc-bbut00010984"	exon_number "2"
12	lncRNA	exon	87919033	87921380	.	-	.	gene_id "lnc-bbug04699"	transcript_id "lnc-bbut00010996"	exon_number "1"
12	lncRNA	exon	87921499	87921858	.	-	.	gene_id "lnc-bbug04699"	transcript_id "lnc-bbut00010996"	exon_number "2"
12	lncRNA	exon	88166113	88166984	.	-	.	gene_id "lnc-bbug04702"	transcript_id "lnc-bbut00010999"	exon_number "1"
12	lncRNA	exon	88168787	88169520	.	-	.	gene_id "lnc-bbug04702"	transcript_id "lnc-bbut00010999"	exon_number "2"
12	lncRNA	exon	88642119	88642518	.	-	.	gene_id "lnc-bbug04705"	transcript_id "lnc-bbut00011002"	exon_number "1"
12	lncRNA	exon	88646448	88646537	.	-	.	gene_id "lnc-bbug04705"	transcript_id "lnc-bbut00011002"	exon_number "2"
12	lncRNA	exon	88646964	88647082	.	-	.	gene_id "lnc-bbug04705"	transcript_id "lnc-bbut00011002"	exon_number "3"
12	lncRNA	exon	89152548	89152954	.	-	.	gene_id "lnc-bbug04706"	transcript_id "lnc-bbut00011003"	exon_number "1"
12	lncRNA	exon	89159709	89159800	.	-	.	gene_id "lnc-bbug04706"	transcript_id "lnc-bbut00011003"	exon_number "2"
12	lncRNA	exon	89199690	89199823	.	-	.	gene_id "lnc-bbug04706"	transcript_id "lnc-bbut00011003"	exon_number "3"
12	lncRNA	exon	90098860	90098998	.	-	.	gene_id "lnc-bbug04709"	transcript_id "lnc-bbut00011011"	exon_number "1"
12	lncRNA	exon	90099372	90099500	.	-	.	gene_id "lnc-bbug04709"	transcript_id "lnc-bbut00011011"	exon_number "2"
12	lncRNA	exon	90099617	90100052	.	-	.	gene_id "lnc-bbug04709"	transcript_id "lnc-bbut00011011"	exon_number "3"
12	lncRNA	exon	90261948	90262984	.	-	.	gene_id "lnc-bbug04711"	transcript_id "lnc-bbut00011013"	exon_number "1"
12	lncRNA	exon	90264970	90265315	.	-	.	gene_id "lnc-bbug04711"	transcript_id "lnc-bbut00011013"	exon_number "2"
12	lncRNA	exon	90268261	90268386	.	-	.	gene_id "lnc-bbug04711"	transcript_id "lnc-bbut00011013"	exon_number "3"
12	lncRNA	exon	90344398	90345008	.	-	.	gene_id "lnc-bbug04711"	transcript_id "lnc-bbut00011013"	exon_number "4"
12	lncRNA	exon	92065271	92065982	.	-	.	gene_id "lnc-bbug04717"	transcript_id "lnc-bbut00011026"	exon_number "1"
12	lncRNA	exon	92067124	92067285	.	-	.	gene_id "lnc-bbug04717"	transcript_id "lnc-bbut00011026"	exon_number "2"
12	lncRNA	exon	92075843	92076391	.	-	.	gene_id "lnc-bbug04717"	transcript_id "lnc-bbut00011026"	exon_number "3"
12	lncRNA	exon	92594647	92594966	.	-	.	gene_id "lnc-bbug04719"	transcript_id "lnc-bbut00011028"	exon_number "1"
12	lncRNA	exon	92598757	92598923	.	-	.	gene_id "lnc-bbug04719"	transcript_id "lnc-bbut00011028"	exon_number "2"
12	lncRNA	exon	92604733	92604896	.	-	.	gene_id "lnc-bbug04719"	transcript_id "lnc-bbut00011028"	exon_number "3"
12	lncRNA	exon	92612346	92612512	.	-	.	gene_id "lnc-bbug04719"	transcript_id "lnc-bbut00011028"	exon_number "4"
12	lncRNA	exon	92794121	92796404	.	-	.	gene_id "lnc-bbug04722"	transcript_id "lnc-bbut00011034"	exon_number "1"
12	lncRNA	exon	92796588	92796740	.	-	.	gene_id "lnc-bbug04722"	transcript_id "lnc-bbut00011034"	exon_number "2"
12	lncRNA	exon	92807123	92807338	.	-	.	gene_id "lnc-bbug04722"	transcript_id "lnc-bbut00011034"	exon_number "3"
12	lncRNA	exon	92852703	92853637	.	-	.	gene_id "lnc-bbug04723"	transcript_id "lnc-bbut00011035"	exon_number "1"
12	lncRNA	exon	93006515	93007448	.	-	.	gene_id "lnc-bbug04730"	transcript_id "lnc-bbut00011042"	exon_number "1"
12	lncRNA	exon	93183265	93184205	.	-	.	gene_id "lnc-bbug04735"	transcript_id "lnc-bbut00011047"	exon_number "1"
12	lncRNA	exon	94524954	94525024	.	-	.	gene_id "lnc-bbug04749"	transcript_id "lnc-bbut00011075"	exon_number "1"
12	lncRNA	exon	94525481	94527706	.	-	.	gene_id "lnc-bbug04749"	transcript_id "lnc-bbut00011075"	exon_number "2"
12	lncRNA	exon	95286406	95290248	.	-	.	gene_id "lnc-bbug04753"	transcript_id "lnc-bbut00011082"	exon_number "1"
12	lncRNA	exon	96064993	96065172	.	-	.	gene_id "lnc-bbug04761"	transcript_id "lnc-bbut00011115"	exon_number "1"
12	lncRNA	exon	96075820	96076021	.	-	.	gene_id "lnc-bbug04761"	transcript_id "lnc-bbut00011115"	exon_number "2"
12	lncRNA	exon	96077968	96078045	.	-	.	gene_id "lnc-bbug04761"	transcript_id "lnc-bbut00011115"	exon_number "3"
12	lncRNA	exon	96083898	96084032	.	-	.	gene_id "lnc-bbug04761"	transcript_id "lnc-bbut00011115"	exon_number "4"
12	lncRNA	exon	96917290	96917334	.	-	.	gene_id "lnc-bbug04762"	transcript_id "lnc-bbut00011116"	exon_number "1"
12	lncRNA	exon	96917552	96917611	.	-	.	gene_id "lnc-bbug04762"	transcript_id "lnc-bbut00011116"	exon_number "2"
12	lncRNA	exon	96942158	96942288	.	-	.	gene_id "lnc-bbug04762"	transcript_id "lnc-bbut00011116"	exon_number "3"
12	lncRNA	exon	96943963	96944150	.	-	.	gene_id "lnc-bbug04762"	transcript_id "lnc-bbut00011116"	exon_number "4"
12	lncRNA	exon	98211224	98211854	.	-	.	gene_id "lnc-bbug04776"	transcript_id "lnc-bbut00011152"	exon_number "1"
12	lncRNA	exon	98211989	98212329	.	-	.	gene_id "lnc-bbug04776"	transcript_id "lnc-bbut00011152"	exon_number "2"
12	lncRNA	exon	98215375	98215635	.	-	.	gene_id "lnc-bbug04776"	transcript_id "lnc-bbut00011152"	exon_number "3"
12	lncRNA	exon	98569571	98570479	.	-	.	gene_id "lnc-bbug04783"	transcript_id "lnc-bbut00011176"	exon_number "1"
12	lncRNA	exon	98571425	98571837	.	-	.	gene_id "lnc-bbug04783"	transcript_id "lnc-bbut00011176"	exon_number "2"
12	lncRNA	exon	98811971	98812215	.	-	.	gene_id "lnc-bbug04787"	transcript_id "lnc-bbut00011185"	exon_number "1"

12	lncRNA	exon	98812605	98812859	.	-	.	gene_id "lnc-bbug04787"	transcript_id "lnc-bbut00011185"	exon_number "2"
12	lncRNA	exon	99350178	99350584	.	-	.	gene_id "lnc-bbug04794"	transcript_id "lnc-bbut00011210"	exon_number "1"
12	lncRNA	exon	99351739	99351826	.	-	.	gene_id "lnc-bbug04794"	transcript_id "lnc-bbut00011210"	exon_number "2"
12	lncRNA	exon	99354428	99355006	.	-	.	gene_id "lnc-bbug04794"	transcript_id "lnc-bbut00011210"	exon_number "3"
12	lncRNA	exon	99350178	99350584	.	-	.	gene_id "lnc-bbug04794"	transcript_id "lnc-bbut00011211"	exon_number "1"
12	lncRNA	exon	99351739	99351826	.	-	.	gene_id "lnc-bbug04794"	transcript_id "lnc-bbut00011211"	exon_number "2"
12	lncRNA	exon	99352002	99352086	.	-	.	gene_id "lnc-bbug04794"	transcript_id "lnc-bbut00011211"	exon_number "3"
12	lncRNA	exon	99354428	99355020	.	-	.	gene_id "lnc-bbug04794"	transcript_id "lnc-bbut00011211"	exon_number "4"
12	lncRNA	exon	99350178	99350584	.	-	.	gene_id "lnc-bbug04794"	transcript_id "lnc-bbut00011213"	exon_number "1"
12	lncRNA	exon	99351739	99351826	.	-	.	gene_id "lnc-bbug04794"	transcript_id "lnc-bbut00011213"	exon_number "2"
12	lncRNA	exon	99352221	99352301	.	-	.	gene_id "lnc-bbug04794"	transcript_id "lnc-bbut00011213"	exon_number "3"
12	lncRNA	exon	99354428	99355033	.	-	.	gene_id "lnc-bbug04794"	transcript_id "lnc-bbut00011213"	exon_number "4"
12	lncRNA	exon	99350178	99350584	.	-	.	gene_id "lnc-bbug04794"	transcript_id "lnc-bbut00011212"	exon_number "1"
12	lncRNA	exon	99351739	99351826	.	-	.	gene_id "lnc-bbug04794"	transcript_id "lnc-bbut00011212"	exon_number "2"
12	lncRNA	exon	99352211	99352301	.	-	.	gene_id "lnc-bbug04794"	transcript_id "lnc-bbut00011212"	exon_number "3"
12	lncRNA	exon	99354428	99355033	.	-	.	gene_id "lnc-bbug04794"	transcript_id "lnc-bbut00011212"	exon_number "4"
12	lncRNA	exon	100635623	100638978	.	-	.	gene_id "lnc-bbug04802"	transcript_id "lnc-bbut00011237"	exon_number "1"
12	lncRNA	exon	102242510	102243002	.	-	.	gene_id "lnc-bbug04811"	transcript_id "lnc-bbut00011281"	exon_number "1"
12	lncRNA	exon	102243998	102244192	.	-	.	gene_id "lnc-bbug04811"	transcript_id "lnc-bbut00011281"	exon_number "2"
12	lncRNA	exon	102244943	102245066	.	-	.	gene_id "lnc-bbug04811"	transcript_id "lnc-bbut00011281"	exon_number "3"
12	lncRNA	exon	102249343	102249665	.	-	.	gene_id "lnc-bbug04811"	transcript_id "lnc-bbut00011281"	exon_number "4"
12	lncRNA	exon	103122763	103125064	.	-	.	gene_id "lnc-bbug04822"	transcript_id "lnc-bbut00011321"	exon_number "1"
12	lncRNA	exon	103126417	103126759	.	-	.	gene_id "lnc-bbug04822"	transcript_id "lnc-bbut00011321"	exon_number "2"
12	lncRNA	exon	103122763	103125064	.	-	.	gene_id "lnc-bbug04822"	transcript_id "lnc-bbut00011322"	exon_number "1"
12	lncRNA	exon	103126807	103126964	.	-	.	gene_id "lnc-bbug04822"	transcript_id "lnc-bbut00011322"	exon_number "2"
12	lncRNA	exon	103655981	103657262	.	-	.	gene_id "lnc-bbug04836"	transcript_id "lnc-bbut00011372"	exon_number "1"
12	lncRNA	exon	103659967	103660252	.	-	.	gene_id "lnc-bbug04836"	transcript_id "lnc-bbut00011372"	exon_number "2"
12	lncRNA	exon	103660759	103660946	.	-	.	gene_id "lnc-bbug04836"	transcript_id "lnc-bbut00011372"	exon_number "3"
12	lncRNA	exon	103661404	103661649	.	-	.	gene_id "lnc-bbug04836"	transcript_id "lnc-bbut00011372"	exon_number "4"
12	lncRNA	exon	103655981	103657262	.	-	.	gene_id "lnc-bbug04836"	transcript_id "lnc-bbut00011374"	exon_number "1"
12	lncRNA	exon	103659572	103659757	.	-	.	gene_id "lnc-bbug04836"	transcript_id "lnc-bbut00011374"	exon_number "2"
12	lncRNA	exon	103659967	103660252	.	-	.	gene_id "lnc-bbug04836"	transcript_id "lnc-bbut00011374"	exon_number "3"
12	lncRNA	exon	103660759	103660946	.	-	.	gene_id "lnc-bbug04836"	transcript_id "lnc-bbut00011374"	exon_number "4"
12	lncRNA	exon	103661404	103661650	.	-	.	gene_id "lnc-bbug04836"	transcript_id "lnc-bbut00011374"	exon_number "5"
12	lncRNA	exon	103655981	103657262	.	-	.	gene_id "lnc-bbug04836"	transcript_id "lnc-bbut00011373"	exon_number "1"
12	lncRNA	exon	103659572	103660252	.	-	.	gene_id "lnc-bbug04836"	transcript_id "lnc-bbut00011373"	exon_number "2"
12	lncRNA	exon	103660759	103660946	.	-	.	gene_id "lnc-bbug04836"	transcript_id "lnc-bbut00011373"	exon_number "3"
12	lncRNA	exon	103661404	103661650	.	-	.	gene_id "lnc-bbug04836"	transcript_id "lnc-bbut00011373"	exon_number "4"
12	lncRNA	exon	103655981	103657262	.	-	.	gene_id "lnc-bbug04836"	transcript_id "lnc-bbut00011375"	exon_number "1"
12	lncRNA	exon	103659572	103660252	.	-	.	gene_id "lnc-bbug04836"	transcript_id "lnc-bbut00011375"	exon_number "2"
12	lncRNA	exon	103660759	103660932	.	-	.	gene_id "lnc-bbug04836"	transcript_id "lnc-bbut00011375"	exon_number "3"
12	lncRNA	exon	103661404	103661651	.	-	.	gene_id "lnc-bbug04836"	transcript_id "lnc-bbut00011375"	exon_number "4"
12	lncRNA	exon	104256519	104256910	.	-	.	gene_id "lnc-bbug04848"	transcript_id "lnc-bbut00011424"	exon_number "1"
12	lncRNA	exon	104258136	104263805	.	-	.	gene_id "lnc-bbug04848"	transcript_id "lnc-bbut00011424"	exon_number "2"
12	lncRNA	exon	104256519	104256910	.	-	.	gene_id "lnc-bbug04848"	transcript_id "lnc-bbut00011425"	exon_number "1"
12	lncRNA	exon	104265916	104267660	.	-	.	gene_id "lnc-bbug04848"	transcript_id "lnc-bbut00011425"	exon_number "2"
12	lncRNA	exon	105155938	105158961	.	-	.	gene_id "lnc-bbug04856"	transcript_id "lnc-bbut00011451"	exon_number "1"
12	lncRNA	exon	105160262	105163170	.	-	.	gene_id "lnc-bbug04856"	transcript_id "lnc-bbut00011451"	exon_number "2"
12	lncRNA	exon	105164871	105166462	.	-	.	gene_id "lnc-bbug04856"	transcript_id "lnc-bbut00011451"	exon_number "3"
12	lncRNA	exon	105155938	105158961	.	-	.	gene_id "lnc-bbug04856"	transcript_id "lnc-bbut00011454"	exon_number "1"
12	lncRNA	exon	105160262	105161075	.	-	.	gene_id "lnc-bbug04856"	transcript_id "lnc-bbut00011454"	exon_number "2"
12	lncRNA	exon	105163041	105163170	.	-	.	gene_id "lnc-bbug04856"	transcript_id "lnc-bbut00011454"	exon_number "3"

12	lncRNA	exon	105173191	105173324	.	-	.	gene_id "lnc-bbug04856"	transcript_id "lnc-bbut00011454"	exon_number "4"
12	lncRNA	exon	105350269	105350941	.	-	.	gene_id "lnc-bbug04864"	transcript_id "lnc-bbut00011471"	exon_number "1"
12	lncRNA	exon	105351243	105352136	.	-	.	gene_id "lnc-bbug04864"	transcript_id "lnc-bbut00011471"	exon_number "2"
12	lncRNA	exon	105350269	105350941	.	-	.	gene_id "lnc-bbug04864"	transcript_id "lnc-bbut00011472"	exon_number "1"
12	lncRNA	exon	105352379	105352547	.	-	.	gene_id "lnc-bbug04864"	transcript_id "lnc-bbut00011472"	exon_number "2"
12	lncRNA	exon	105390799	105392272	.	-	.	gene_id "lnc-bbug04868"	transcript_id "lnc-bbut00011481"	exon_number "1"
12	lncRNA	exon	105392404	105392507	.	-	.	gene_id "lnc-bbug04868"	transcript_id "lnc-bbut00011481"	exon_number "2"
12	lncRNA	exon	105392734	105392842	.	-	.	gene_id "lnc-bbug04868"	transcript_id "lnc-bbut00011481"	exon_number "3"
12	lncRNA	exon	105430393	105430592	.	-	.	gene_id "lnc-bbug04870"	transcript_id "lnc-bbut00011492"	exon_number "1"
12	lncRNA	exon	105431496	105431773	.	-	.	gene_id "lnc-bbug04870"	transcript_id "lnc-bbut00011492"	exon_number "2"
12	lncRNA	exon	105590004	105590127	.	-	.	gene_id "lnc-bbug04876"	transcript_id "lnc-bbut00011501"	exon_number "1"
12	lncRNA	exon	105591258	105591359	.	-	.	gene_id "lnc-bbug04876"	transcript_id "lnc-bbut00011501"	exon_number "2"
12	lncRNA	exon	105591471	105591574	.	-	.	gene_id "lnc-bbug04876"	transcript_id "lnc-bbut00011501"	exon_number "3"
12	lncRNA	exon	105591654	105591733	.	-	.	gene_id "lnc-bbug04876"	transcript_id "lnc-bbut00011501"	exon_number "4"
12	lncRNA	exon	105591904	105592006	.	-	.	gene_id "lnc-bbug04876"	transcript_id "lnc-bbut00011501"	exon_number "5"
12	lncRNA	exon	105691379	105691802	.	-	.	gene_id "lnc-bbug04884"	transcript_id "lnc-bbut00011518"	exon_number "1"
12	lncRNA	exon	105692203	105692310	.	-	.	gene_id "lnc-bbug04884"	transcript_id "lnc-bbut00011518"	exon_number "2"
12	lncRNA	exon	105692449	105692532	.	-	.	gene_id "lnc-bbug04884"	transcript_id "lnc-bbut00011518"	exon_number "3"
12	lncRNA	exon	105692711	105692777	.	-	.	gene_id "lnc-bbug04884"	transcript_id "lnc-bbut00011518"	exon_number "4"
12	lncRNA	exon	105693064	105693194	.	-	.	gene_id "lnc-bbug04884"	transcript_id "lnc-bbut00011518"	exon_number "5"
12	lncRNA	exon	105692203	105692310	.	-	.	gene_id "lnc-bbug04884"	transcript_id "lnc-bbut00011519"	exon_number "1"
12	lncRNA	exon	105692387	105692532	.	-	.	gene_id "lnc-bbug04884"	transcript_id "lnc-bbut00011519"	exon_number "2"
12	lncRNA	exon	105692711	105692777	.	-	.	gene_id "lnc-bbug04884"	transcript_id "lnc-bbut00011519"	exon_number "3"
12	lncRNA	exon	105693064	105693194	.	-	.	gene_id "lnc-bbug04884"	transcript_id "lnc-bbut00011519"	exon_number "4"
12	lncRNA	exon	105724597	105726608	.	-	.	gene_id "lnc-bbug04887"	transcript_id "lnc-bbut00011527"	exon_number "1"
12	lncRNA	exon	105726967	105727102	.	-	.	gene_id "lnc-bbug04887"	transcript_id "lnc-bbut00011527"	exon_number "2"
13	lncRNA	exon	327331	327627	.	+	.	gene_id "lnc-bbug04895"	transcript_id "lnc-bbut00011565"	exon_number "1"
13	lncRNA	exon	328749	329733	.	+	.	gene_id "lnc-bbug04895"	transcript_id "lnc-bbut00011565"	exon_number "2"
13	lncRNA	exon	329813	332130	.	+	.	gene_id "lnc-bbug04895"	transcript_id "lnc-bbut00011565"	exon_number "3"
13	lncRNA	exon	332469	332605	.	+	.	gene_id "lnc-bbug04895"	transcript_id "lnc-bbut00011565"	exon_number "4"
13	lncRNA	exon	530807	531492	.	+	.	gene_id "lnc-bbug04901"	transcript_id "lnc-bbut00011586"	exon_number "1"
13	lncRNA	exon	545625	546370	.	+	.	gene_id "lnc-bbug04901"	transcript_id "lnc-bbut00011586"	exon_number "2"
13	lncRNA	exon	546696	547601	.	+	.	gene_id "lnc-bbug04901"	transcript_id "lnc-bbut00011586"	exon_number "3"
13	lncRNA	exon	544888	546370	.	+	.	gene_id "lnc-bbug04901"	transcript_id "lnc-bbut00011587"	exon_number "1"
13	lncRNA	exon	546696	547601	.	+	.	gene_id "lnc-bbug04901"	transcript_id "lnc-bbut00011587"	exon_number "2"
13	lncRNA	exon	544890	546370	.	+	.	gene_id "lnc-bbug04901"	transcript_id "lnc-bbut00011588"	exon_number "1"
13	lncRNA	exon	546696	546816	.	+	.	gene_id "lnc-bbug04901"	transcript_id "lnc-bbut00011588"	exon_number "2"
13	lncRNA	exon	547450	547601	.	+	.	gene_id "lnc-bbug04901"	transcript_id "lnc-bbut00011588"	exon_number "3"
13	lncRNA	exon	725262	727090	.	+	.	gene_id "lnc-bbug04904"	transcript_id "lnc-bbut00011592"	exon_number "1"
13	lncRNA	exon	729105	731435	.	+	.	gene_id "lnc-bbug04904"	transcript_id "lnc-bbut00011592"	exon_number "2"
13	lncRNA	exon	837477	838475	.	+	.	gene_id "lnc-bbug04905"	transcript_id "lnc-bbut00011593"	exon_number "1"
13	lncRNA	exon	839367	840913	.	+	.	gene_id "lnc-bbug04905"	transcript_id "lnc-bbut00011593"	exon_number "2"
13	lncRNA	exon	877189	877528	.	+	.	gene_id "lnc-bbug04906"	transcript_id "lnc-bbut00011594"	exon_number "1"
13	lncRNA	exon	883856	883996	.	+	.	gene_id "lnc-bbug04906"	transcript_id "lnc-bbut00011594"	exon_number "2"
13	lncRNA	exon	889282	889394	.	+	.	gene_id "lnc-bbug04906"	transcript_id "lnc-bbut00011594"	exon_number "3"
13	lncRNA	exon	893670	894165	.	+	.	gene_id "lnc-bbug04906"	transcript_id "lnc-bbut00011594"	exon_number "4"
13	lncRNA	exon	989209	989367	.	+	.	gene_id "lnc-bbug04908"	transcript_id "lnc-bbut00011597"	exon_number "1"
13	lncRNA	exon	989735	990321	.	+	.	gene_id "lnc-bbug04908"	transcript_id "lnc-bbut00011597"	exon_number "2"
13	lncRNA	exon	990583	990750	.	+	.	gene_id "lnc-bbug04908"	transcript_id "lnc-bbut00011597"	exon_number "3"
13	lncRNA	exon	991918	992112	.	+	.	gene_id "lnc-bbug04908"	transcript_id "lnc-bbut00011597"	exon_number "4"
13	lncRNA	exon	992309	992449	.	+	.	gene_id "lnc-bbug04908"	transcript_id "lnc-bbut00011597"	exon_number "5"
13	lncRNA	exon	990776	991506	.	+	.	gene_id "lnc-bbug04908"	transcript_id "lnc-bbut00011598"	exon_number "1"

13	lncRNA	exon	991918	992112	.	+	.	gene_id "lnc-bbug04908"	transcript_id "lnc-bbut00011598"	exon_number "2"
13	lncRNA	exon	992309	992449	.	+	.	gene_id "lnc-bbug04908"	transcript_id "lnc-bbut00011598"	exon_number "3"
13	lncRNA	exon	990776	992112	.	+	.	gene_id "lnc-bbug04908"	transcript_id "lnc-bbut00011599"	exon_number "1"
13	lncRNA	exon	992740	992827	.	+	.	gene_id "lnc-bbug04908"	transcript_id "lnc-bbut00011599"	exon_number "2"
13	lncRNA	exon	993724	993912	.	+	.	gene_id "lnc-bbug04908"	transcript_id "lnc-bbut00011599"	exon_number "3"
13	lncRNA	exon	994220	994385	.	+	.	gene_id "lnc-bbug04908"	transcript_id "lnc-bbut00011599"	exon_number "4"
13	lncRNA	exon	1594834	1594898	.	+	.	gene_id "lnc-bbug04909"	transcript_id "lnc-bbut00011600"	exon_number "1"
13	lncRNA	exon	1595491	1595643	.	+	.	gene_id "lnc-bbug04909"	transcript_id "lnc-bbut00011600"	exon_number "2"
13	lncRNA	exon	1595744	1595862	.	+	.	gene_id "lnc-bbug04909"	transcript_id "lnc-bbut00011600"	exon_number "3"
13	lncRNA	exon	1595987	1596034	.	+	.	gene_id "lnc-bbug04909"	transcript_id "lnc-bbut00011600"	exon_number "4"
13	lncRNA	exon	1596332	1596383	.	+	.	gene_id "lnc-bbug04909"	transcript_id "lnc-bbut00011600"	exon_number "5"
13	lncRNA	exon	1885567	1885603	.	+	.	gene_id "lnc-bbug04912"	transcript_id "lnc-bbut00011607"	exon_number "1"
13	lncRNA	exon	1886107	1886538	.	+	.	gene_id "lnc-bbug04912"	transcript_id "lnc-bbut00011607"	exon_number "2"
13	lncRNA	exon	2459414	2461908	.	+	.	gene_id "lnc-bbug04915"	transcript_id "lnc-bbut00011618"	exon_number "1"
13	lncRNA	exon	2472987	2475404	.	+	.	gene_id "lnc-bbug04915"	transcript_id "lnc-bbut00011618"	exon_number "2"
13	lncRNA	exon	2536793	2537890	.	+	.	gene_id "lnc-bbug04916"	transcript_id "lnc-bbut00011619"	exon_number "1"
13	lncRNA	exon	2543827	2545765	.	+	.	gene_id "lnc-bbug04916"	transcript_id "lnc-bbut00011619"	exon_number "2"
13	lncRNA	exon	2547784	2551034	.	+	.	gene_id "lnc-bbug04916"	transcript_id "lnc-bbut00011619"	exon_number "3"
13	lncRNA	exon	2560625	2560696	.	+	.	gene_id "lnc-bbug04916"	transcript_id "lnc-bbut00011619"	exon_number "4"
13	lncRNA	exon	2560869	2561924	.	+	.	gene_id "lnc-bbug04916"	transcript_id "lnc-bbut00011619"	exon_number "5"
13	lncRNA	exon	2553570	2554999	.	+	.	gene_id "lnc-bbug04917"	transcript_id "lnc-bbut00011620"	exon_number "1"
13	lncRNA	exon	2555269	2557706	.	+	.	gene_id "lnc-bbug04917"	transcript_id "lnc-bbut00011620"	exon_number "2"
13	lncRNA	exon	2589451	2594206	.	+	.	gene_id "lnc-bbug04918"	transcript_id "lnc-bbut00011621"	exon_number "1"
13	lncRNA	exon	2602322	2603470	.	+	.	gene_id "lnc-bbug04918"	transcript_id "lnc-bbut00011621"	exon_number "2"
13	lncRNA	exon	2607177	2608181	.	+	.	gene_id "lnc-bbug04918"	transcript_id "lnc-bbut00011621"	exon_number "3"
13	lncRNA	exon	2625245	2625355	.	+	.	gene_id "lnc-bbug04919"	transcript_id "lnc-bbut00011624"	exon_number "1"
13	lncRNA	exon	2625738	2627433	.	+	.	gene_id "lnc-bbug04919"	transcript_id "lnc-bbut00011624"	exon_number "2"
13	lncRNA	exon	2627798	2629440	.	+	.	gene_id "lnc-bbug04919"	transcript_id "lnc-bbut00011624"	exon_number "3"
13	lncRNA	exon	4307835	4308219	.	+	.	gene_id "lnc-bbug04921"	transcript_id "lnc-bbut00011631"	exon_number "1"
13	lncRNA	exon	4310683	4310765	.	+	.	gene_id "lnc-bbug04921"	transcript_id "lnc-bbut00011631"	exon_number "2"
13	lncRNA	exon	4311501	4311552	.	+	.	gene_id "lnc-bbug04921"	transcript_id "lnc-bbut00011631"	exon_number "3"
13	lncRNA	exon	4313679	4314085	.	+	.	gene_id "lnc-bbug04921"	transcript_id "lnc-bbut00011631"	exon_number "4"
13	lncRNA	exon	4544780	4545147	.	+	.	gene_id "lnc-bbug04922"	transcript_id "lnc-bbut00011632"	exon_number "1"
13	lncRNA	exon	4545447	4545534	.	+	.	gene_id "lnc-bbug04922"	transcript_id "lnc-bbut00011632"	exon_number "2"
13	lncRNA	exon	4546417	4546960	.	+	.	gene_id "lnc-bbug04922"	transcript_id "lnc-bbut00011632"	exon_number "3"
13	lncRNA	exon	4555681	4555873	.	+	.	gene_id "lnc-bbug04923"	transcript_id "lnc-bbut00011633"	exon_number "1"
13	lncRNA	exon	4558716	4559113	.	+	.	gene_id "lnc-bbug04923"	transcript_id "lnc-bbut00011633"	exon_number "2"
13	lncRNA	exon	4872744	4874510	.	+	.	gene_id "lnc-bbug04926"	transcript_id "lnc-bbut00011637"	exon_number "1"
13	lncRNA	exon	4875444	4875697	.	+	.	gene_id "lnc-bbug04926"	transcript_id "lnc-bbut00011637"	exon_number "2"
13	lncRNA	exon	4876854	4878031	.	+	.	gene_id "lnc-bbug04926"	transcript_id "lnc-bbut00011637"	exon_number "3"
13	lncRNA	exon	8815887	8817469	.	+	.	gene_id "lnc-bbug04932"	transcript_id "lnc-bbut00011646"	exon_number "1"
13	lncRNA	exon	8853833	8853943	.	+	.	gene_id "lnc-bbug04932"	transcript_id "lnc-bbut00011646"	exon_number "2"
13	lncRNA	exon	8861227	8861330	.	+	.	gene_id "lnc-bbug04932"	transcript_id "lnc-bbut00011646"	exon_number "3"
13	lncRNA	exon	8965217	8965415	.	+	.	gene_id "lnc-bbug04932"	transcript_id "lnc-bbut00011646"	exon_number "4"
13	lncRNA	exon	8966626	8968792	.	+	.	gene_id "lnc-bbug04932"	transcript_id "lnc-bbut00011646"	exon_number "5"
13	lncRNA	exon	10296505	10296510	.	+	.	gene_id "lnc-bbug04936"	transcript_id "lnc-bbut00011667"	exon_number "1"
13	lncRNA	exon	10306167	10306854	.	+	.	gene_id "lnc-bbug04936"	transcript_id "lnc-bbut00011667"	exon_number "2"
13	lncRNA	exon	10306919	10306941	.	+	.	gene_id "lnc-bbug04936"	transcript_id "lnc-bbut00011667"	exon_number "3"
13	lncRNA	exon	10307153	10307880	.	+	.	gene_id "lnc-bbug04936"	transcript_id "lnc-bbut00011667"	exon_number "4"
13	lncRNA	exon	10308318	10308566	.	+	.	gene_id "lnc-bbug04936"	transcript_id "lnc-bbut00011667"	exon_number "5"
13	lncRNA	exon	10473075	10473186	.	+	.	gene_id "lnc-bbug04938"	transcript_id "lnc-bbut00011669"	exon_number "1"
13	lncRNA	exon	10473302	10473473	.	+	.	gene_id "lnc-bbug04938"	transcript_id "lnc-bbut00011669"	exon_number "2"

13	lncRNA	exon	10473986	10474205	.	+	.	gene_id "lnc-bbug04938"	transcript_id "lnc-bbut00011669"	exon_number "3"
13	lncRNA	exon	10940364	10940608	.	+	.	gene_id "lnc-bbug04941"	transcript_id "lnc-bbut00011680"	exon_number "1"
13	lncRNA	exon	10942505	10942598	.	+	.	gene_id "lnc-bbug04941"	transcript_id "lnc-bbut00011680"	exon_number "2"
13	lncRNA	exon	10952941	10953355	.	+	.	gene_id "lnc-bbug04941"	transcript_id "lnc-bbut00011680"	exon_number "3"
13	lncRNA	exon	12072990	12073100	.	+	.	gene_id "lnc-bbug04946"	transcript_id "lnc-bbut00011687"	exon_number "1"
13	lncRNA	exon	12076070	12077682	.	+	.	gene_id "lnc-bbug04946"	transcript_id "lnc-bbut00011687"	exon_number "2"
13	lncRNA	exon	12730012	12731408	.	+	.	gene_id "lnc-bbug04947"	transcript_id "lnc-bbut00011688"	exon_number "1"
13	lncRNA	exon	12738938	12739046	.	+	.	gene_id "lnc-bbug04947"	transcript_id "lnc-bbut00011688"	exon_number "2"
13	lncRNA	exon	12764300	12764740	.	+	.	gene_id "lnc-bbug04947"	transcript_id "lnc-bbut00011688"	exon_number "3"
13	lncRNA	exon	12766773	12769767	.	+	.	gene_id "lnc-bbug04947"	transcript_id "lnc-bbut00011688"	exon_number "4"
13	lncRNA	exon	13965620	13965825	.	+	.	gene_id "lnc-bbug04951"	transcript_id "lnc-bbut00011696"	exon_number "1"
13	lncRNA	exon	13968024	13968816	.	+	.	gene_id "lnc-bbug04951"	transcript_id "lnc-bbut00011696"	exon_number "2"
13	lncRNA	exon	14194817	14197726	.	+	.	gene_id "lnc-bbug04953"	transcript_id "lnc-bbut00011701"	exon_number "1"
13	lncRNA	exon	14198109	14198285	.	+	.	gene_id "lnc-bbug04953"	transcript_id "lnc-bbut00011701"	exon_number "2"
13	lncRNA	exon	14198517	14198698	.	+	.	gene_id "lnc-bbug04953"	transcript_id "lnc-bbut00011701"	exon_number "3"
13	lncRNA	exon	15108217	15108675	.	+	.	gene_id "lnc-bbug04956"	transcript_id "lnc-bbut00011704"	exon_number "1"
13	lncRNA	exon	16900690	16902196	.	+	.	gene_id "lnc-bbug04961"	transcript_id "lnc-bbut00011716"	exon_number "1"
13	lncRNA	exon	16902380	16902437	.	+	.	gene_id "lnc-bbug04961"	transcript_id "lnc-bbut00011716"	exon_number "2"
13	lncRNA	exon	16905605	16905775	.	+	.	gene_id "lnc-bbug04961"	transcript_id "lnc-bbut00011716"	exon_number "3"
13	lncRNA	exon	16900690	16902196	.	+	.	gene_id "lnc-bbug04961"	transcript_id "lnc-bbut00011717"	exon_number "1"
13	lncRNA	exon	16902380	16902437	.	+	.	gene_id "lnc-bbug04961"	transcript_id "lnc-bbut00011717"	exon_number "2"
13	lncRNA	exon	16910680	16910714	.	+	.	gene_id "lnc-bbug04961"	transcript_id "lnc-bbut00011717"	exon_number "3"
13	lncRNA	exon	17449649	17449681	.	+	.	gene_id "lnc-bbug04968"	transcript_id "lnc-bbut00011724"	exon_number "1"
13	lncRNA	exon	17453483	17453601	.	+	.	gene_id "lnc-bbug04968"	transcript_id "lnc-bbut00011724"	exon_number "2"
13	lncRNA	exon	17453933	17454645	.	+	.	gene_id "lnc-bbug04968"	transcript_id "lnc-bbut00011724"	exon_number "3"
13	lncRNA	exon	17653903	17657142	.	+	.	gene_id "lnc-bbug04970"	transcript_id "lnc-bbut00011728"	exon_number "1"
13	lncRNA	exon	17661644	17661987	.	+	.	gene_id "lnc-bbug04970"	transcript_id "lnc-bbut00011728"	exon_number "2"
13	lncRNA	exon	17664526	17664821	.	+	.	gene_id "lnc-bbug04970"	transcript_id "lnc-bbut00011728"	exon_number "3"
13	lncRNA	exon	17666456	17666506	.	+	.	gene_id "lnc-bbug04971"	transcript_id "lnc-bbut00011730"	exon_number "1"
13	lncRNA	exon	17689281	17689503	.	+	.	gene_id "lnc-bbug04971"	transcript_id "lnc-bbut00011730"	exon_number "2"
13	lncRNA	exon	17690374	17692253	.	+	.	gene_id "lnc-bbug04971"	transcript_id "lnc-bbut00011730"	exon_number "3"
13	lncRNA	exon	19907263	19907894	.	+	.	gene_id "lnc-bbug04977"	transcript_id "lnc-bbut00011736"	exon_number "1"
13	lncRNA	exon	19910608	19910653	.	+	.	gene_id "lnc-bbug04978"	transcript_id "lnc-bbut00011737"	exon_number "1"
13	lncRNA	exon	19913488	19915144	.	+	.	gene_id "lnc-bbug04978"	transcript_id "lnc-bbut00011737"	exon_number "2"
13	lncRNA	exon	21772989	21773231	.	+	.	gene_id "lnc-bbug04985"	transcript_id "lnc-bbut00011744"	exon_number "1"
13	lncRNA	exon	21876082	21876956	.	+	.	gene_id "lnc-bbug04986"	transcript_id "lnc-bbut00011745"	exon_number "1"
13	lncRNA	exon	21885712	21886158	.	+	.	gene_id "lnc-bbug04986"	transcript_id "lnc-bbut00011745"	exon_number "2"
13	lncRNA	exon	21876082	21876956	.	+	.	gene_id "lnc-bbug04986"	transcript_id "lnc-bbut00011746"	exon_number "1"
13	lncRNA	exon	21888673	21888806	.	+	.	gene_id "lnc-bbug04986"	transcript_id "lnc-bbut00011746"	exon_number "2"
13	lncRNA	exon	22019715	22019958	.	+	.	gene_id "lnc-bbug04987"	transcript_id "lnc-bbut00011747"	exon_number "1"
13	lncRNA	exon	22020163	22020396	.	+	.	gene_id "lnc-bbug04987"	transcript_id "lnc-bbut00011747"	exon_number "2"
13	lncRNA	exon	23172693	23173045	.	+	.	gene_id "lnc-bbug04988"	transcript_id "lnc-bbut00011748"	exon_number "1"
13	lncRNA	exon	23187014	23187074	.	+	.	gene_id "lnc-bbug04988"	transcript_id "lnc-bbut00011748"	exon_number "2"
13	lncRNA	exon	23205207	23205282	.	+	.	gene_id "lnc-bbug04988"	transcript_id "lnc-bbut00011748"	exon_number "3"
13	lncRNA	exon	23212412	23213202	.	+	.	gene_id "lnc-bbug04988"	transcript_id "lnc-bbut00011748"	exon_number "4"
13	lncRNA	exon	25055541	25055585	.	+	.	gene_id "lnc-bbug04989"	transcript_id "lnc-bbut00011749"	exon_number "1"
13	lncRNA	exon	25055954	25056050	.	+	.	gene_id "lnc-bbug04989"	transcript_id "lnc-bbut00011749"	exon_number "2"
13	lncRNA	exon	25058580	25058695	.	+	.	gene_id "lnc-bbug04989"	transcript_id "lnc-bbut00011749"	exon_number "3"
13	lncRNA	exon	25509523	25509791	.	+	.	gene_id "lnc-bbug04990"	transcript_id "lnc-bbut00011750"	exon_number "1"
13	lncRNA	exon	25512136	25512547	.	+	.	gene_id "lnc-bbug04990"	transcript_id "lnc-bbut00011750"	exon_number "2"
13	lncRNA	exon	25704872	25704970	.	+	.	gene_id "lnc-bbug04991"	transcript_id "lnc-bbut00011752"	exon_number "1"
13	lncRNA	exon	25725058	25725142	.	+	.	gene_id "lnc-bbug04991"	transcript_id "lnc-bbut00011752"	exon_number "2"

13	lncRNA	exon	25730040	25730173	.	+	.	gene_id "lnc-bbug04991"	transcript_id "lnc-bbut00011752"	exon_number "3"
13	lncRNA	exon	25739100	25739781	.	+	.	gene_id "lnc-bbug04991"	transcript_id "lnc-bbut00011752"	exon_number "4"
13	lncRNA	exon	25704872	25704970	.	+	.	gene_id "lnc-bbug04991"	transcript_id "lnc-bbut00011751"	exon_number "1"
13	lncRNA	exon	25725058	25725142	.	+	.	gene_id "lnc-bbug04991"	transcript_id "lnc-bbut00011751"	exon_number "2"
13	lncRNA	exon	25730040	25730173	.	+	.	gene_id "lnc-bbug04991"	transcript_id "lnc-bbut00011751"	exon_number "3"
13	lncRNA	exon	25739097	25739781	.	+	.	gene_id "lnc-bbug04991"	transcript_id "lnc-bbut00011751"	exon_number "4"
13	lncRNA	exon	33290139	33290758	.	+	.	gene_id "lnc-bbug04997"	transcript_id "lnc-bbut00011758"	exon_number "1"
13	lncRNA	exon	34365749	34365821	.	+	.	gene_id "lnc-bbug04998"	transcript_id "lnc-bbut00011759"	exon_number "1"
13	lncRNA	exon	34365868	34366104	.	+	.	gene_id "lnc-bbug04998"	transcript_id "lnc-bbut00011759"	exon_number "2"
13	lncRNA	exon	34366876	34368150	.	+	.	gene_id "lnc-bbug04998"	transcript_id "lnc-bbut00011759"	exon_number "3"
13	lncRNA	exon	35859988	35861513	.	+	.	gene_id "lnc-bbug05001"	transcript_id "lnc-bbut00011762"	exon_number "1"
13	lncRNA	exon	35893448	35893986	.	+	.	gene_id "lnc-bbug05001"	transcript_id "lnc-bbut00011762"	exon_number "2"
13	lncRNA	exon	38805190	38808635	.	+	.	gene_id "lnc-bbug05009"	transcript_id "lnc-bbut00011800"	exon_number "1"
13	lncRNA	exon	38809044	38809434	.	+	.	gene_id "lnc-bbug05009"	transcript_id "lnc-bbut00011800"	exon_number "2"
13	lncRNA	exon	38845053	38845132	.	+	.	gene_id "lnc-bbug05010"	transcript_id "lnc-bbut00011801"	exon_number "1"
13	lncRNA	exon	38845659	38846493	.	+	.	gene_id "lnc-bbug05010"	transcript_id "lnc-bbut00011801"	exon_number "2"
13	lncRNA	exon	38867536	38867791	.	+	.	gene_id "lnc-bbug05011"	transcript_id "lnc-bbut00011803"	exon_number "1"
13	lncRNA	exon	38869448	38869592	.	+	.	gene_id "lnc-bbug05011"	transcript_id "lnc-bbut00011803"	exon_number "2"
13	lncRNA	exon	38875859	38876148	.	+	.	gene_id "lnc-bbug05011"	transcript_id "lnc-bbut00011803"	exon_number "3"
13	lncRNA	exon	38878551	38878734	.	+	.	gene_id "lnc-bbug05011"	transcript_id "lnc-bbut00011803"	exon_number "4"
13	lncRNA	exon	38879090	38879320	.	+	.	gene_id "lnc-bbug05011"	transcript_id "lnc-bbut00011803"	exon_number "5"
13	lncRNA	exon	38880579	38880701	.	+	.	gene_id "lnc-bbug05011"	transcript_id "lnc-bbut00011803"	exon_number "6"
13	lncRNA	exon	38867536	38867682	.	+	.	gene_id "lnc-bbug05011"	transcript_id "lnc-bbut00011802"	exon_number "1"
13	lncRNA	exon	38869448	38869592	.	+	.	gene_id "lnc-bbug05011"	transcript_id "lnc-bbut00011802"	exon_number "2"
13	lncRNA	exon	38875859	38876148	.	+	.	gene_id "lnc-bbug05011"	transcript_id "lnc-bbut00011802"	exon_number "3"
13	lncRNA	exon	38878551	38878734	.	+	.	gene_id "lnc-bbug05011"	transcript_id "lnc-bbut00011802"	exon_number "4"
13	lncRNA	exon	38879090	38879320	.	+	.	gene_id "lnc-bbug05011"	transcript_id "lnc-bbut00011802"	exon_number "5"
13	lncRNA	exon	38880579	38880701	.	+	.	gene_id "lnc-bbug05011"	transcript_id "lnc-bbut00011802"	exon_number "6"
13	lncRNA	exon	38869111	38869186	.	+	.	gene_id "lnc-bbug05011"	transcript_id "lnc-bbut00011804"	exon_number "1"
13	lncRNA	exon	38869448	38869592	.	+	.	gene_id "lnc-bbug05011"	transcript_id "lnc-bbut00011804"	exon_number "2"
13	lncRNA	exon	38875859	38876148	.	+	.	gene_id "lnc-bbug05011"	transcript_id "lnc-bbut00011804"	exon_number "3"
13	lncRNA	exon	38878551	38878734	.	+	.	gene_id "lnc-bbug05011"	transcript_id "lnc-bbut00011804"	exon_number "4"
13	lncRNA	exon	38879090	38879320	.	+	.	gene_id "lnc-bbug05011"	transcript_id "lnc-bbut00011804"	exon_number "5"
13	lncRNA	exon	38880579	38880701	.	+	.	gene_id "lnc-bbug05011"	transcript_id "lnc-bbut00011804"	exon_number "6"
13	lncRNA	exon	38883401	38883548	.	+	.	gene_id "lnc-bbug05012"	transcript_id "lnc-bbut00011805"	exon_number "1"
13	lncRNA	exon	38922438	38924582	.	+	.	gene_id "lnc-bbug05012"	transcript_id "lnc-bbut00011805"	exon_number "2"
13	lncRNA	exon	38908234	38909751	.	+	.	gene_id "lnc-bbug05012"	transcript_id "lnc-bbut00011806"	exon_number "1"
13	lncRNA	exon	38922438	38924582	.	+	.	gene_id "lnc-bbug05012"	transcript_id "lnc-bbut00011806"	exon_number "2"
13	lncRNA	exon	38918754	38924241	.	+	.	gene_id "lnc-bbug05012"	transcript_id "lnc-bbut00011807"	exon_number "1"
13	lncRNA	exon	38927868	38928072	.	+	.	gene_id "lnc-bbug05012"	transcript_id "lnc-bbut00011807"	exon_number "2"
13	lncRNA	exon	38929568	38930266	.	+	.	gene_id "lnc-bbug05012"	transcript_id "lnc-bbut00011807"	exon_number "3"
13	lncRNA	exon	38919567	38919985	.	+	.	gene_id "lnc-bbug05012"	transcript_id "lnc-bbut00011808"	exon_number "1"
13	lncRNA	exon	38920541	38920601	.	+	.	gene_id "lnc-bbug05012"	transcript_id "lnc-bbut00011808"	exon_number "2"
13	lncRNA	exon	38922438	38924582	.	+	.	gene_id "lnc-bbug05012"	transcript_id "lnc-bbut00011808"	exon_number "3"
13	lncRNA	exon	38941757	38943504	.	+	.	gene_id "lnc-bbug05013"	transcript_id "lnc-bbut00011809"	exon_number "1"
13	lncRNA	exon	38945512	38945725	.	+	.	gene_id "lnc-bbug05013"	transcript_id "lnc-bbut00011809"	exon_number "2"
13	lncRNA	exon	38946192	38948484	.	+	.	gene_id "lnc-bbug05013"	transcript_id "lnc-bbut00011809"	exon_number "3"
13	lncRNA	exon	40179750	40179887	.	+	.	gene_id "lnc-bbug05015"	transcript_id "lnc-bbut00011812"	exon_number "1"
13	lncRNA	exon	40179972	40179983	.	+	.	gene_id "lnc-bbug05015"	transcript_id "lnc-bbut00011812"	exon_number "2"
13	lncRNA	exon	40193956	40194108	.	+	.	gene_id "lnc-bbug05015"	transcript_id "lnc-bbut00011812"	exon_number "3"
13	lncRNA	exon	40200544	40200828	.	+	.	gene_id "lnc-bbug05015"	transcript_id "lnc-bbut00011812"	exon_number "4"
13	lncRNA	exon	40202436	40202764	.	+	.	gene_id "lnc-bbug05015"	transcript_id "lnc-bbut00011812"	exon_number "5"

13	lncRNA	exon	40179750	40179887	.	+	.	gene_id "lnc-bbug05015"	transcript_id "lnc-bbut00011811"	exon_number "1"
13	lncRNA	exon	40179972	40179983	.	+	.	gene_id "lnc-bbug05015"	transcript_id "lnc-bbut00011811"	exon_number "2"
13	lncRNA	exon	40193956	40194108	.	+	.	gene_id "lnc-bbug05015"	transcript_id "lnc-bbut00011811"	exon_number "3"
13	lncRNA	exon	40200544	40202764	.	+	.	gene_id "lnc-bbug05015"	transcript_id "lnc-bbut00011811"	exon_number "4"
13	lncRNA	exon	40179990	40180124	.	+	.	gene_id "lnc-bbug05015"	transcript_id "lnc-bbut00011813"	exon_number "1"
13	lncRNA	exon	40193956	40194108	.	+	.	gene_id "lnc-bbug05015"	transcript_id "lnc-bbut00011813"	exon_number "2"
13	lncRNA	exon	40200544	40202764	.	+	.	gene_id "lnc-bbug05015"	transcript_id "lnc-bbut00011813"	exon_number "3"
13	lncRNA	exon	40180053	40180191	.	+	.	gene_id "lnc-bbug05015"	transcript_id "lnc-bbut00011814"	exon_number "1"
13	lncRNA	exon	40193956	40194108	.	+	.	gene_id "lnc-bbug05015"	transcript_id "lnc-bbut00011814"	exon_number "2"
13	lncRNA	exon	40200544	40202764	.	+	.	gene_id "lnc-bbug05015"	transcript_id "lnc-bbut00011814"	exon_number "3"
13	lncRNA	exon	43259258	43259451	.	+	.	gene_id "lnc-bbug05019"	transcript_id "lnc-bbut00011838"	exon_number "1"
13	lncRNA	exon	43265134	43265279	.	+	.	gene_id "lnc-bbug05019"	transcript_id "lnc-bbut00011838"	exon_number "2"
13	lncRNA	exon	43272954	43274757	.	+	.	gene_id "lnc-bbug05019"	transcript_id "lnc-bbut00011838"	exon_number "3"
13	lncRNA	exon	43259916	43260161	.	+	.	gene_id "lnc-bbug05019"	transcript_id "lnc-bbut00011839"	exon_number "1"
13	lncRNA	exon	43265134	43265279	.	+	.	gene_id "lnc-bbug05019"	transcript_id "lnc-bbut00011839"	exon_number "2"
13	lncRNA	exon	43272954	43274757	.	+	.	gene_id "lnc-bbug05019"	transcript_id "lnc-bbut00011839"	exon_number "3"
13	lncRNA	exon	48429425	48429464	.	+	.	gene_id "lnc-bbug05023"	transcript_id "lnc-bbut00011845"	exon_number "1"
13	lncRNA	exon	48482068	48482563	.	+	.	gene_id "lnc-bbug05023"	transcript_id "lnc-bbut00011845"	exon_number "2"
13	lncRNA	exon	52712701	52712726	.	+	.	gene_id "lnc-bbug05025"	transcript_id "lnc-bbut00011847"	exon_number "1"
13	lncRNA	exon	52721146	52722272	.	+	.	gene_id "lnc-bbug05025"	transcript_id "lnc-bbut00011847"	exon_number "2"
13	lncRNA	exon	52722384	52723105	.	+	.	gene_id "lnc-bbug05025"	transcript_id "lnc-bbut00011847"	exon_number "3"
13	lncRNA	exon	54275993	54276685	.	+	.	gene_id "lnc-bbug05030"	transcript_id "lnc-bbut00011860"	exon_number "1"
13	lncRNA	exon	54760898	54760962	.	+	.	gene_id "lnc-bbug05034"	transcript_id "lnc-bbut00011878"	exon_number "1"
13	lncRNA	exon	54761807	54763081	.	+	.	gene_id "lnc-bbug05034"	transcript_id "lnc-bbut00011878"	exon_number "2"
13	lncRNA	exon	54853099	54853789	.	+	.	gene_id "lnc-bbug05035"	transcript_id "lnc-bbut00011879"	exon_number "1"
13	lncRNA	exon	54854244	54854445	.	+	.	gene_id "lnc-bbug05035"	transcript_id "lnc-bbut00011879"	exon_number "2"
13	lncRNA	exon	54853099	54853789	.	+	.	gene_id "lnc-bbug05035"	transcript_id "lnc-bbut00011880"	exon_number "1"
13	lncRNA	exon	54860078	54860403	.	+	.	gene_id "lnc-bbug05035"	transcript_id "lnc-bbut00011880"	exon_number "2"
13	lncRNA	exon	54929668	54932839	.	+	.	gene_id "lnc-bbug05037"	transcript_id "lnc-bbut00011891"	exon_number "1"
13	lncRNA	exon	54933294	54933589	.	+	.	gene_id "lnc-bbug05037"	transcript_id "lnc-bbut00011891"	exon_number "2"
13	lncRNA	exon	54933806	54933927	.	+	.	gene_id "lnc-bbug05037"	transcript_id "lnc-bbut00011891"	exon_number "3"
13	lncRNA	exon	54961953	54962371	.	+	.	gene_id "lnc-bbug05037"	transcript_id "lnc-bbut00011891"	exon_number "4"
13	lncRNA	exon	55072076	55072462	.	+	.	gene_id "lnc-bbug05038"	transcript_id "lnc-bbut00011892"	exon_number "1"
13	lncRNA	exon	55076486	55077023	.	+	.	gene_id "lnc-bbug05039"	transcript_id "lnc-bbut00011894"	exon_number "1"
13	lncRNA	exon	55092512	55092585	.	+	.	gene_id "lnc-bbug05039"	transcript_id "lnc-bbut00011894"	exon_number "2"
13	lncRNA	exon	55098408	55098663	.	+	.	gene_id "lnc-bbug05039"	transcript_id "lnc-bbut00011894"	exon_number "3"
13	lncRNA	exon	55076486	55077023	.	+	.	gene_id "lnc-bbug05039"	transcript_id "lnc-bbut00011895"	exon_number "1"
13	lncRNA	exon	55092512	55092585	.	+	.	gene_id "lnc-bbug05039"	transcript_id "lnc-bbut00011895"	exon_number "2"
13	lncRNA	exon	55111219	55111785	.	+	.	gene_id "lnc-bbug05039"	transcript_id "lnc-bbut00011895"	exon_number "3"
13	lncRNA	exon	55131878	55133009	.	+	.	gene_id "lnc-bbug05040"	transcript_id "lnc-bbut00011896"	exon_number "1"
13	lncRNA	exon	55133537	55136457	.	+	.	gene_id "lnc-bbug05040"	transcript_id "lnc-bbut00011896"	exon_number "2"
13	lncRNA	exon	55186902	55187014	.	+	.	gene_id "lnc-bbug05042"	transcript_id "lnc-bbut00011898"	exon_number "1"
13	lncRNA	exon	55187121	55187581	.	+	.	gene_id "lnc-bbug05042"	transcript_id "lnc-bbut00011898"	exon_number "2"
13	lncRNA	exon	55186909	55187014	.	+	.	gene_id "lnc-bbug05042"	transcript_id "lnc-bbut00011899"	exon_number "1"
13	lncRNA	exon	55187089	55187581	.	+	.	gene_id "lnc-bbug05042"	transcript_id "lnc-bbut00011899"	exon_number "2"
13	lncRNA	exon	55186969	55187033	.	+	.	gene_id "lnc-bbug05042"	transcript_id "lnc-bbut00011900"	exon_number "1"
13	lncRNA	exon	55187121	55187581	.	+	.	gene_id "lnc-bbug05042"	transcript_id "lnc-bbut00011900"	exon_number "2"
13	lncRNA	exon	55277010	55277136	.	+	.	gene_id "lnc-bbug05043"	transcript_id "lnc-bbut00011901"	exon_number "1"
13	lncRNA	exon	55277430	55277521	.	+	.	gene_id "lnc-bbug05043"	transcript_id "lnc-bbut00011901"	exon_number "2"
13	lncRNA	exon	55279892	55280090	.	+	.	gene_id "lnc-bbug05043"	transcript_id "lnc-bbut00011901"	exon_number "3"
13	lncRNA	exon	55554595	55557380	.	+	.	gene_id "lnc-bbug05046"	transcript_id "lnc-bbut00011908"	exon_number "1"
13	lncRNA	exon	55557862	55558000	.	+	.	gene_id "lnc-bbug05046"	transcript_id "lnc-bbut00011908"	exon_number "2"

13	lncRNA	exon	55602041	55602216	.	+	.	gene_id "lnc-bbug05046"	transcript_id "lnc-bbut00011908"	exon_number "3"
13	lncRNA	exon	55602296	55604659	.	+	.	gene_id "lnc-bbug05046"	transcript_id "lnc-bbut00011908"	exon_number "4"
13	lncRNA	exon	55610069	55610993	.	+	.	gene_id "lnc-bbug05047"	transcript_id "lnc-bbut00011909"	exon_number "1"
13	lncRNA	exon	55611569	55611709	.	+	.	gene_id "lnc-bbug05047"	transcript_id "lnc-bbut00011909"	exon_number "2"
13	lncRNA	exon	55613089	55613851	.	+	.	gene_id "lnc-bbug05047"	transcript_id "lnc-bbut00011909"	exon_number "3"
13	lncRNA	exon	55738717	55738848	.	+	.	gene_id "lnc-bbug05048"	transcript_id "lnc-bbut00011910"	exon_number "1"
13	lncRNA	exon	55744602	55744858	.	+	.	gene_id "lnc-bbug05048"	transcript_id "lnc-bbut00011910"	exon_number "2"
13	lncRNA	exon	58081779	58082057	.	+	.	gene_id "lnc-bbug05058"	transcript_id "lnc-bbut00011937"	exon_number "1"
13	lncRNA	exon	58082559	58082880	.	+	.	gene_id "lnc-bbug05058"	transcript_id "lnc-bbut00011937"	exon_number "2"
13	lncRNA	exon	58152517	58152615	.	+	.	gene_id "lnc-bbug05061"	transcript_id "lnc-bbut00011942"	exon_number "1"
13	lncRNA	exon	58152852	58153012	.	+	.	gene_id "lnc-bbug05061"	transcript_id "lnc-bbut00011942"	exon_number "2"
13	lncRNA	exon	58156498	58156701	.	+	.	gene_id "lnc-bbug05061"	transcript_id "lnc-bbut00011942"	exon_number "3"
13	lncRNA	exon	58427944	58428023	.	+	.	gene_id "lnc-bbug05063"	transcript_id "lnc-bbut00011944"	exon_number "1"
13	lncRNA	exon	58428561	58428646	.	+	.	gene_id "lnc-bbug05063"	transcript_id "lnc-bbut00011944"	exon_number "2"
13	lncRNA	exon	58428788	58428847	.	+	.	gene_id "lnc-bbug05063"	transcript_id "lnc-bbut00011944"	exon_number "3"
13	lncRNA	exon	58429347	58429473	.	+	.	gene_id "lnc-bbug05064"	transcript_id "lnc-bbut00011946"	exon_number "1"
13	lncRNA	exon	58451311	58451385	.	+	.	gene_id "lnc-bbug05064"	transcript_id "lnc-bbut00011946"	exon_number "2"
13	lncRNA	exon	58461224	58462245	.	+	.	gene_id "lnc-bbug05064"	transcript_id "lnc-bbut00011946"	exon_number "3"
13	lncRNA	exon	58868980	58870377	.	+	.	gene_id "lnc-bbug05068"	transcript_id "lnc-bbut00011962"	exon_number "1"
13	lncRNA	exon	58872565	58876040	.	+	.	gene_id "lnc-bbug05068"	transcript_id "lnc-bbut00011962"	exon_number "2"
13	lncRNA	exon	59277520	59278909	.	+	.	gene_id "lnc-bbug05070"	transcript_id "lnc-bbut00011964"	exon_number "1"
13	lncRNA	exon	59279783	59280041	.	+	.	gene_id "lnc-bbug05070"	transcript_id "lnc-bbut00011964"	exon_number "2"
13	lncRNA	exon	59892857	59893377	.	+	.	gene_id "lnc-bbug05072"	transcript_id "lnc-bbut00011969"	exon_number "1"
13	lncRNA	exon	59895179	59895244	.	+	.	gene_id "lnc-bbug05072"	transcript_id "lnc-bbut00011969"	exon_number "2"
13	lncRNA	exon	59895993	59896279	.	+	.	gene_id "lnc-bbug05072"	transcript_id "lnc-bbut00011969"	exon_number "3"
13	lncRNA	exon	60018128	60018236	.	+	.	gene_id "lnc-bbug05073"	transcript_id "lnc-bbut00011970"	exon_number "1"
13	lncRNA	exon	60018643	60018739	.	+	.	gene_id "lnc-bbug05073"	transcript_id "lnc-bbut00011970"	exon_number "2"
13	lncRNA	exon	60031358	60031522	.	+	.	gene_id "lnc-bbug05073"	transcript_id "lnc-bbut00011970"	exon_number "3"
13	lncRNA	exon	60035729	60041121	.	+	.	gene_id "lnc-bbug05073"	transcript_id "lnc-bbut00011970"	exon_number "4"
13	lncRNA	exon	60061972	60062351	.	+	.	gene_id "lnc-bbug05074"	transcript_id "lnc-bbut00011971"	exon_number "1"
13	lncRNA	exon	60068420	60068610	.	+	.	gene_id "lnc-bbug05074"	transcript_id "lnc-bbut00011971"	exon_number "2"
13	lncRNA	exon	60069386	60070050	.	+	.	gene_id "lnc-bbug05074"	transcript_id "lnc-bbut00011971"	exon_number "3"
13	lncRNA	exon	60162135	60162797	.	+	.	gene_id "lnc-bbug05075"	transcript_id "lnc-bbut00011973"	exon_number "1"
13	lncRNA	exon	60164113	60164289	.	+	.	gene_id "lnc-bbug05075"	transcript_id "lnc-bbut00011973"	exon_number "2"
13	lncRNA	exon	60164658	60164758	.	+	.	gene_id "lnc-bbug05075"	transcript_id "lnc-bbut00011973"	exon_number "3"
13	lncRNA	exon	60167113	60168380	.	+	.	gene_id "lnc-bbug05075"	transcript_id "lnc-bbut00011973"	exon_number "4"
13	lncRNA	exon	60162135	60162797	.	+	.	gene_id "lnc-bbug05075"	transcript_id "lnc-bbut00011972"	exon_number "1"
13	lncRNA	exon	60164113	60164289	.	+	.	gene_id "lnc-bbug05075"	transcript_id "lnc-bbut00011972"	exon_number "2"
13	lncRNA	exon	60164658	60168380	.	+	.	gene_id "lnc-bbug05075"	transcript_id "lnc-bbut00011972"	exon_number "3"
13	lncRNA	exon	60662194	60662645	.	+	.	gene_id "lnc-bbug05079"	transcript_id "lnc-bbut00011981"	exon_number "1"
13	lncRNA	exon	60663158	60663731	.	+	.	gene_id "lnc-bbug05079"	transcript_id "lnc-bbut00011981"	exon_number "2"
13	lncRNA	exon	62796476	62796663	.	+	.	gene_id "lnc-bbug05089"	transcript_id "lnc-bbut00011999"	exon_number "1"
13	lncRNA	exon	62802773	62802849	.	+	.	gene_id "lnc-bbug05089"	transcript_id "lnc-bbut00011999"	exon_number "2"
13	lncRNA	exon	62812117	62812876	.	+	.	gene_id "lnc-bbug05089"	transcript_id "lnc-bbut00011999"	exon_number "3"
13	lncRNA	exon	63126147	63126653	.	+	.	gene_id "lnc-bbug05090"	transcript_id "lnc-bbut00012000"	exon_number "1"
13	lncRNA	exon	63133741	63133828	.	+	.	gene_id "lnc-bbug05090"	transcript_id "lnc-bbut00012000"	exon_number "2"
13	lncRNA	exon	63135233	63137788	.	+	.	gene_id "lnc-bbug05090"	transcript_id "lnc-bbut00012000"	exon_number "3"
13	lncRNA	exon	63164910	63165007	.	+	.	gene_id "lnc-bbug05090"	transcript_id "lnc-bbut00012000"	exon_number "4"
13	lncRNA	exon	63178034	63178144	.	+	.	gene_id "lnc-bbug05090"	transcript_id "lnc-bbut00012000"	exon_number "5"
13	lncRNA	exon	63179545	63183940	.	+	.	gene_id "lnc-bbug05090"	transcript_id "lnc-bbut00012000"	exon_number "6"
13	lncRNA	exon	63126148	63126686	.	+	.	gene_id "lnc-bbug05090"	transcript_id "lnc-bbut00012001"	exon_number "1"
13	lncRNA	exon	63133741	63133828	.	+	.	gene_id "lnc-bbug05090"	transcript_id "lnc-bbut00012001"	exon_number "2"

13	lncRNA	exon	63137656	63137788	.	+	.	gene_id "lnc-bbug05090"	transcript_id "lnc-bbut00012001"	exon_number "3"
13	lncRNA	exon	63164910	63165007	.	+	.	gene_id "lnc-bbug05090"	transcript_id "lnc-bbut00012001"	exon_number "4"
13	lncRNA	exon	63178034	63178144	.	+	.	gene_id "lnc-bbug05090"	transcript_id "lnc-bbut00012001"	exon_number "5"
13	lncRNA	exon	63179545	63183940	.	+	.	gene_id "lnc-bbug05090"	transcript_id "lnc-bbut00012001"	exon_number "6"
13	lncRNA	exon	63126156	63126653	.	+	.	gene_id "lnc-bbug05090"	transcript_id "lnc-bbut00012002"	exon_number "1"
13	lncRNA	exon	63133741	63133828	.	+	.	gene_id "lnc-bbug05090"	transcript_id "lnc-bbut00012002"	exon_number "2"
13	lncRNA	exon	63137656	63137788	.	+	.	gene_id "lnc-bbug05090"	transcript_id "lnc-bbut00012002"	exon_number "3"
13	lncRNA	exon	63164910	63165007	.	+	.	gene_id "lnc-bbug05090"	transcript_id "lnc-bbut00012002"	exon_number "4"
13	lncRNA	exon	63178034	63178144	.	+	.	gene_id "lnc-bbug05090"	transcript_id "lnc-bbut00012002"	exon_number "5"
13	lncRNA	exon	63179545	63183940	.	+	.	gene_id "lnc-bbug05090"	transcript_id "lnc-bbut00012002"	exon_number "6"
13	lncRNA	exon	63126157	63126686	.	+	.	gene_id "lnc-bbug05090"	transcript_id "lnc-bbut00012003"	exon_number "1"
13	lncRNA	exon	63133741	63133828	.	+	.	gene_id "lnc-bbug05090"	transcript_id "lnc-bbut00012003"	exon_number "2"
13	lncRNA	exon	63164910	63165007	.	+	.	gene_id "lnc-bbug05090"	transcript_id "lnc-bbut00012003"	exon_number "3"
13	lncRNA	exon	63178034	63178144	.	+	.	gene_id "lnc-bbug05090"	transcript_id "lnc-bbut00012003"	exon_number "4"
13	lncRNA	exon	63179545	63183940	.	+	.	gene_id "lnc-bbug05090"	transcript_id "lnc-bbut00012003"	exon_number "5"
13	lncRNA	exon	63126158	63126653	.	+	.	gene_id "lnc-bbug05090"	transcript_id "lnc-bbut00012005"	exon_number "1"
13	lncRNA	exon	63133741	63133828	.	+	.	gene_id "lnc-bbug05090"	transcript_id "lnc-bbut00012005"	exon_number "2"
13	lncRNA	exon	63164910	63165007	.	+	.	gene_id "lnc-bbug05090"	transcript_id "lnc-bbut00012005"	exon_number "3"
13	lncRNA	exon	63178034	63178144	.	+	.	gene_id "lnc-bbug05090"	transcript_id "lnc-bbut00012005"	exon_number "4"
13	lncRNA	exon	63179545	63181999	.	+	.	gene_id "lnc-bbug05090"	transcript_id "lnc-bbut00012005"	exon_number "5"
13	lncRNA	exon	63182179	63183940	.	+	.	gene_id "lnc-bbug05090"	transcript_id "lnc-bbut00012005"	exon_number "6"
13	lncRNA	exon	63126158	63126653	.	+	.	gene_id "lnc-bbug05090"	transcript_id "lnc-bbut00012004"	exon_number "1"
13	lncRNA	exon	63133741	63133828	.	+	.	gene_id "lnc-bbug05090"	transcript_id "lnc-bbut00012004"	exon_number "2"
13	lncRNA	exon	63164910	63165007	.	+	.	gene_id "lnc-bbug05090"	transcript_id "lnc-bbut00012004"	exon_number "3"
13	lncRNA	exon	63178034	63178144	.	+	.	gene_id "lnc-bbug05090"	transcript_id "lnc-bbut00012004"	exon_number "4"
13	lncRNA	exon	63179545	63182063	.	+	.	gene_id "lnc-bbug05090"	transcript_id "lnc-bbut00012004"	exon_number "5"
13	lncRNA	exon	63182179	63183940	.	+	.	gene_id "lnc-bbug05090"	transcript_id "lnc-bbut00012004"	exon_number "6"
13	lncRNA	exon	63126225	63126653	.	+	.	gene_id "lnc-bbug05090"	transcript_id "lnc-bbut00012006"	exon_number "1"
13	lncRNA	exon	63133741	63133828	.	+	.	gene_id "lnc-bbug05090"	transcript_id "lnc-bbut00012006"	exon_number "2"
13	lncRNA	exon	63164910	63165007	.	+	.	gene_id "lnc-bbug05090"	transcript_id "lnc-bbut00012006"	exon_number "3"
13	lncRNA	exon	63179545	63183940	.	+	.	gene_id "lnc-bbug05090"	transcript_id "lnc-bbut00012006"	exon_number "4"
13	lncRNA	exon	63126235	63126653	.	+	.	gene_id "lnc-bbug05090"	transcript_id "lnc-bbut00012007"	exon_number "1"
13	lncRNA	exon	63133741	63133828	.	+	.	gene_id "lnc-bbug05090"	transcript_id "lnc-bbut00012007"	exon_number "2"
13	lncRNA	exon	63164910	63165007	.	+	.	gene_id "lnc-bbug05090"	transcript_id "lnc-bbut00012007"	exon_number "3"
13	lncRNA	exon	63182179	63183940	.	+	.	gene_id "lnc-bbug05090"	transcript_id "lnc-bbut00012007"	exon_number "4"
13	lncRNA	exon	66002066	66002373	.	+	.	gene_id "lnc-bbug05096"	transcript_id "lnc-bbut00012034"	exon_number "1"
13	lncRNA	exon	66004387	66004459	.	+	.	gene_id "lnc-bbug05096"	transcript_id "lnc-bbut00012034"	exon_number "2"
13	lncRNA	exon	66014865	66015478	.	+	.	gene_id "lnc-bbug05096"	transcript_id "lnc-bbut00012034"	exon_number "3"
13	lncRNA	exon	66050014	66050574	.	+	.	gene_id "lnc-bbug05098"	transcript_id "lnc-bbut00012036"	exon_number "1"
13	lncRNA	exon	66752039	66752320	.	+	.	gene_id "lnc-bbug05099"	transcript_id "lnc-bbut00012038"	exon_number "1"
13	lncRNA	exon	66806823	66808222	.	+	.	gene_id "lnc-bbug05099"	transcript_id "lnc-bbut00012038"	exon_number "2"
13	lncRNA	exon	66752039	66752320	.	+	.	gene_id "lnc-bbug05099"	transcript_id "lnc-bbut00012037"	exon_number "1"
13	lncRNA	exon	66806818	66808222	.	+	.	gene_id "lnc-bbug05099"	transcript_id "lnc-bbut00012037"	exon_number "2"
13	lncRNA	exon	67024898	67025031	.	+	.	gene_id "lnc-bbug05100"	transcript_id "lnc-bbut00012039"	exon_number "1"
13	lncRNA	exon	67025112	67025168	.	+	.	gene_id "lnc-bbug05100"	transcript_id "lnc-bbut00012039"	exon_number "2"
13	lncRNA	exon	67030707	67030764	.	+	.	gene_id "lnc-bbug05100"	transcript_id "lnc-bbut00012039"	exon_number "3"
13	lncRNA	exon	67034285	67034324	.	+	.	gene_id "lnc-bbug05100"	transcript_id "lnc-bbut00012039"	exon_number "4"
13	lncRNA	exon	67035680	67035712	.	+	.	gene_id "lnc-bbug05100"	transcript_id "lnc-bbut00012039"	exon_number "5"
13	lncRNA	exon	67037244	67039376	.	+	.	gene_id "lnc-bbug05100"	transcript_id "lnc-bbut00012039"	exon_number "6"
13	lncRNA	exon	69329210	69329564	.	+	.	gene_id "lnc-bbug05111"	transcript_id "lnc-bbut00012066"	exon_number "1"
13	lncRNA	exon	69346243	69346385	.	+	.	gene_id "lnc-bbug05111"	transcript_id "lnc-bbut00012066"	exon_number "2"
13	lncRNA	exon	69353668	69353799	.	+	.	gene_id "lnc-bbug05111"	transcript_id "lnc-bbut00012066"	exon_number "3"

13	lncRNA	exon	70347896	70348096	.	+	.	gene_id "lnc-bbug05116"	transcript_id "lnc-bbut00012078"	exon_number "1"
13	lncRNA	exon	70348165	70348684	.	+	.	gene_id "lnc-bbug05116"	transcript_id "lnc-bbut00012078"	exon_number "2"
13	lncRNA	exon	70365095	70365745	.	+	.	gene_id "lnc-bbug05116"	transcript_id "lnc-bbut00012078"	exon_number "3"
13	lncRNA	exon	70384948	70385332	.	+	.	gene_id "lnc-bbug05117"	transcript_id "lnc-bbut00012079"	exon_number "1"
13	lncRNA	exon	70387256	70387350	.	+	.	gene_id "lnc-bbug05117"	transcript_id "lnc-bbut00012079"	exon_number "2"
13	lncRNA	exon	70391044	70392688	.	+	.	gene_id "lnc-bbug05117"	transcript_id "lnc-bbut00012079"	exon_number "3"
13	lncRNA	exon	71103013	71103393	.	+	.	gene_id "lnc-bbug05118"	transcript_id "lnc-bbut00012080"	exon_number "1"
13	lncRNA	exon	71116551	71119555	.	+	.	gene_id "lnc-bbug05118"	transcript_id "lnc-bbut00012080"	exon_number "2"
13	lncRNA	exon	71126637	71128032	.	+	.	gene_id "lnc-bbug05119"	transcript_id "lnc-bbut00012081"	exon_number "1"
13	lncRNA	exon	71131114	71136312	.	+	.	gene_id "lnc-bbug05119"	transcript_id "lnc-bbut00012081"	exon_number "2"
13	lncRNA	exon	71138187	71147390	.	+	.	gene_id "lnc-bbug05120"	transcript_id "lnc-bbut00012082"	exon_number "1"
13	lncRNA	exon	71149403	71149469	.	+	.	gene_id "lnc-bbug05120"	transcript_id "lnc-bbut00012082"	exon_number "2"
13	lncRNA	exon	71150009	71150341	.	+	.	gene_id "lnc-bbug05120"	transcript_id "lnc-bbut00012082"	exon_number "3"
13	lncRNA	exon	71160958	71161634	.	+	.	gene_id "lnc-bbug05121"	transcript_id "lnc-bbut00012083"	exon_number "1"
13	lncRNA	exon	71198415	71199828	.	+	.	gene_id "lnc-bbug05123"	transcript_id "lnc-bbut00012087"	exon_number "1"
13	lncRNA	exon	71200543	71200645	.	+	.	gene_id "lnc-bbug05123"	transcript_id "lnc-bbut00012087"	exon_number "2"
13	lncRNA	exon	71208508	71208724	.	+	.	gene_id "lnc-bbug05123"	transcript_id "lnc-bbut00012087"	exon_number "3"
13	lncRNA	exon	71211619	71211650	.	+	.	gene_id "lnc-bbug05123"	transcript_id "lnc-bbut00012087"	exon_number "4"
13	lncRNA	exon	71212105	71212119	.	+	.	gene_id "lnc-bbug05123"	transcript_id "lnc-bbut00012087"	exon_number "5"
13	lncRNA	exon	71198418	71199828	.	+	.	gene_id "lnc-bbug05123"	transcript_id "lnc-bbut00012088"	exon_number "1"
13	lncRNA	exon	71200543	71200645	.	+	.	gene_id "lnc-bbug05123"	transcript_id "lnc-bbut00012088"	exon_number "2"
13	lncRNA	exon	71208508	71208724	.	+	.	gene_id "lnc-bbug05123"	transcript_id "lnc-bbut00012088"	exon_number "3"
13	lncRNA	exon	71209940	71209988	.	+	.	gene_id "lnc-bbug05123"	transcript_id "lnc-bbut00012088"	exon_number "4"
13	lncRNA	exon	71198419	71199828	.	+	.	gene_id "lnc-bbug05123"	transcript_id "lnc-bbut00012089"	exon_number "1"
13	lncRNA	exon	71200543	71200645	.	+	.	gene_id "lnc-bbug05123"	transcript_id "lnc-bbut00012089"	exon_number "2"
13	lncRNA	exon	71208508	71208724	.	+	.	gene_id "lnc-bbug05123"	transcript_id "lnc-bbut00012089"	exon_number "3"
13	lncRNA	exon	71209121	71209851	.	+	.	gene_id "lnc-bbug05123"	transcript_id "lnc-bbut00012089"	exon_number "4"
13	lncRNA	exon	71391935	71392198	.	+	.	gene_id "lnc-bbug05126"	transcript_id "lnc-bbut00012096"	exon_number "1"
13	lncRNA	exon	71413370	71413439	.	+	.	gene_id "lnc-bbug05126"	transcript_id "lnc-bbut00012096"	exon_number "2"
13	lncRNA	exon	71419007	71419146	.	+	.	gene_id "lnc-bbug05126"	transcript_id "lnc-bbut00012096"	exon_number "3"
13	lncRNA	exon	71420823	71421482	.	+	.	gene_id "lnc-bbug05126"	transcript_id "lnc-bbut00012096"	exon_number "4"
13	lncRNA	exon	71747199	71747898	.	+	.	gene_id "lnc-bbug05128"	transcript_id "lnc-bbut00012119"	exon_number "1"
13	lncRNA	exon	71749641	71749812	.	+	.	gene_id "lnc-bbug05128"	transcript_id "lnc-bbut00012119"	exon_number "2"
13	lncRNA	exon	71752623	71754269	.	+	.	gene_id "lnc-bbug05128"	transcript_id "lnc-bbut00012119"	exon_number "3"
13	lncRNA	exon	71747280	71747898	.	+	.	gene_id "lnc-bbug05128"	transcript_id "lnc-bbut00012120"	exon_number "1"
13	lncRNA	exon	71749641	71751253	.	+	.	gene_id "lnc-bbug05128"	transcript_id "lnc-bbut00012120"	exon_number "2"
13	lncRNA	exon	71994626	71995523	.	+	.	gene_id "lnc-bbug05129"	transcript_id "lnc-bbut00012121"	exon_number "1"
13	lncRNA	exon	71995644	71995713	.	+	.	gene_id "lnc-bbug05129"	transcript_id "lnc-bbut00012121"	exon_number "2"
13	lncRNA	exon	72039978	72040228	.	+	.	gene_id "lnc-bbug05129"	transcript_id "lnc-bbut00012121"	exon_number "3"
13	lncRNA	exon	72362448	72362883	.	+	.	gene_id "lnc-bbug05131"	transcript_id "lnc-bbut00012123"	exon_number "1"
13	lncRNA	exon	72368654	72368902	.	+	.	gene_id "lnc-bbug05131"	transcript_id "lnc-bbut00012123"	exon_number "2"
13	lncRNA	exon	75901323	75901374	.	+	.	gene_id "lnc-bbug05149"	transcript_id "lnc-bbut00012184"	exon_number "1"
13	lncRNA	exon	75902443	75902494	.	+	.	gene_id "lnc-bbug05149"	transcript_id "lnc-bbut00012184"	exon_number "2"
13	lncRNA	exon	75903667	75905060	.	+	.	gene_id "lnc-bbug05149"	transcript_id "lnc-bbut00012184"	exon_number "3"
13	lncRNA	exon	75928548	75928652	.	+	.	gene_id "lnc-bbug05150"	transcript_id "lnc-bbut00012186"	exon_number "1"
13	lncRNA	exon	75930412	75930503	.	+	.	gene_id "lnc-bbug05150"	transcript_id "lnc-bbut00012186"	exon_number "2"
13	lncRNA	exon	75930709	75930956	.	+	.	gene_id "lnc-bbug05150"	transcript_id "lnc-bbut00012186"	exon_number "3"
13	lncRNA	exon	76109844	76110378	.	+	.	gene_id "lnc-bbug05151"	transcript_id "lnc-bbut00012187"	exon_number "1"
13	lncRNA	exon	76110482	76110684	.	+	.	gene_id "lnc-bbug05151"	transcript_id "lnc-bbut00012187"	exon_number "2"
13	lncRNA	exon	76114908	76114992	.	+	.	gene_id "lnc-bbug05151"	transcript_id "lnc-bbut00012187"	exon_number "3"
13	lncRNA	exon	76117195	76117970	.	+	.	gene_id "lnc-bbug05151"	transcript_id "lnc-bbut00012187"	exon_number "4"
13	lncRNA	exon	77426614	77426976	.	+	.	gene_id "lnc-bbug05155"	transcript_id "lnc-bbut00012198"	exon_number "1"

13	lncRNA	exon	77921312	77921647	.	+	.	gene_id "lnc-bbug05157"	transcript_id "lnc-bbut00012204"	exon_number "1"
13	lncRNA	exon	77922679	77922859	.	+	.	gene_id "lnc-bbug05157"	transcript_id "lnc-bbut00012204"	exon_number "2"
13	lncRNA	exon	77923486	77923666	.	+	.	gene_id "lnc-bbug05157"	transcript_id "lnc-bbut00012204"	exon_number "3"
13	lncRNA	exon	79345441	79346434	.	+	.	gene_id "lnc-bbug05164"	transcript_id "lnc-bbut00012216"	exon_number "1"
13	lncRNA	exon	79441312	79451302	.	+	.	gene_id "lnc-bbug05166"	transcript_id "lnc-bbut00012219"	exon_number "1"
13	lncRNA	exon	79452266	79453756	.	+	.	gene_id "lnc-bbug05166"	transcript_id "lnc-bbut00012219"	exon_number "2"
13	lncRNA	exon	80526578	80527418	.	+	.	gene_id "lnc-bbug05170"	transcript_id "lnc-bbut00012223"	exon_number "1"
13	lncRNA	exon	80531229	80531396	.	+	.	gene_id "lnc-bbug05170"	transcript_id "lnc-bbut00012223"	exon_number "2"
13	lncRNA	exon	80532763	80534431	.	+	.	gene_id "lnc-bbug05170"	transcript_id "lnc-bbut00012223"	exon_number "3"
13	lncRNA	exon	83609895	83611298	.	+	.	gene_id "lnc-bbug05172"	transcript_id "lnc-bbut00012225"	exon_number "1"
13	lncRNA	exon	108619	112366	.	-	.	gene_id "lnc-bbug05182"	transcript_id "lnc-bbut00012243"	exon_number "1"
13	lncRNA	exon	127737	128937	.	-	.	gene_id "lnc-bbug05183"	transcript_id "lnc-bbut00012244"	exon_number "1"
13	lncRNA	exon	129896	129991	.	-	.	gene_id "lnc-bbug05183"	transcript_id "lnc-bbut00012244"	exon_number "2"
13	lncRNA	exon	848351	851716	.	-	.	gene_id "lnc-bbug05197"	transcript_id "lnc-bbut00012286"	exon_number "1"
13	lncRNA	exon	853163	853501	.	-	.	gene_id "lnc-bbug05197"	transcript_id "lnc-bbut00012286"	exon_number "2"
13	lncRNA	exon	866223	866651	.	-	.	gene_id "lnc-bbug05198"	transcript_id "lnc-bbut00012287"	exon_number "1"
13	lncRNA	exon	867209	867860	.	-	.	gene_id "lnc-bbug05198"	transcript_id "lnc-bbut00012287"	exon_number "2"
13	lncRNA	exon	866223	866651	.	-	.	gene_id "lnc-bbug05198"	transcript_id "lnc-bbut00012288"	exon_number "1"
13	lncRNA	exon	867706	868652	.	-	.	gene_id "lnc-bbug05198"	transcript_id "lnc-bbut00012288"	exon_number "2"
13	lncRNA	exon	1920669	1920935	.	-	.	gene_id "lnc-bbug05207"	transcript_id "lnc-bbut00012302"	exon_number "1"
13	lncRNA	exon	1921199	1922719	.	-	.	gene_id "lnc-bbug05207"	transcript_id "lnc-bbut00012302"	exon_number "2"
13	lncRNA	exon	1923458	1924280	.	-	.	gene_id "lnc-bbug05207"	transcript_id "lnc-bbut00012302"	exon_number "3"
13	lncRNA	exon	1924672	1924813	.	-	.	gene_id "lnc-bbug05207"	transcript_id "lnc-bbut00012302"	exon_number "4"
13	lncRNA	exon	1920669	1920935	.	-	.	gene_id "lnc-bbug05207"	transcript_id "lnc-bbut00012304"	exon_number "1"
13	lncRNA	exon	1921199	1922748	.	-	.	gene_id "lnc-bbug05207"	transcript_id "lnc-bbut00012304"	exon_number "2"
13	lncRNA	exon	1923458	1924280	.	-	.	gene_id "lnc-bbug05207"	transcript_id "lnc-bbut00012304"	exon_number "3"
13	lncRNA	exon	1924672	1924813	.	-	.	gene_id "lnc-bbug05207"	transcript_id "lnc-bbut00012304"	exon_number "4"
13	lncRNA	exon	1920669	1920935	.	-	.	gene_id "lnc-bbug05207"	transcript_id "lnc-bbut00012306"	exon_number "1"
13	lncRNA	exon	1921199	1923607	.	-	.	gene_id "lnc-bbug05207"	transcript_id "lnc-bbut00012306"	exon_number "2"
13	lncRNA	exon	1924064	1924280	.	-	.	gene_id "lnc-bbug05207"	transcript_id "lnc-bbut00012306"	exon_number "3"
13	lncRNA	exon	1924672	1924813	.	-	.	gene_id "lnc-bbug05207"	transcript_id "lnc-bbut00012306"	exon_number "4"
13	lncRNA	exon	1920669	1920935	.	-	.	gene_id "lnc-bbug05207"	transcript_id "lnc-bbut00012307"	exon_number "1"
13	lncRNA	exon	1921199	1922020	.	-	.	gene_id "lnc-bbug05207"	transcript_id "lnc-bbut00012307"	exon_number "2"
13	lncRNA	exon	1922162	1922393	.	-	.	gene_id "lnc-bbug05207"	transcript_id "lnc-bbut00012307"	exon_number "3"
13	lncRNA	exon	1922547	1924280	.	-	.	gene_id "lnc-bbug05207"	transcript_id "lnc-bbut00012307"	exon_number "4"
13	lncRNA	exon	1924672	1924813	.	-	.	gene_id "lnc-bbug05207"	transcript_id "lnc-bbut00012307"	exon_number "5"
13	lncRNA	exon	1920669	1920935	.	-	.	gene_id "lnc-bbug05207"	transcript_id "lnc-bbut00012305"	exon_number "1"
13	lncRNA	exon	1921199	1923607	.	-	.	gene_id "lnc-bbug05207"	transcript_id "lnc-bbut00012305"	exon_number "2"
13	lncRNA	exon	1923885	1924280	.	-	.	gene_id "lnc-bbug05207"	transcript_id "lnc-bbut00012305"	exon_number "3"
13	lncRNA	exon	1924672	1924813	.	-	.	gene_id "lnc-bbug05207"	transcript_id "lnc-bbut00012305"	exon_number "4"
13	lncRNA	exon	1920669	1920935	.	-	.	gene_id "lnc-bbug05207"	transcript_id "lnc-bbut00012303"	exon_number "1"
13	lncRNA	exon	1921199	1922723	.	-	.	gene_id "lnc-bbug05207"	transcript_id "lnc-bbut00012303"	exon_number "2"
13	lncRNA	exon	1923458	1924280	.	-	.	gene_id "lnc-bbug05207"	transcript_id "lnc-bbut00012303"	exon_number "3"
13	lncRNA	exon	1924672	1924813	.	-	.	gene_id "lnc-bbug05207"	transcript_id "lnc-bbut00012303"	exon_number "4"
13	lncRNA	exon	1920669	1920935	.	-	.	gene_id "lnc-bbug05207"	transcript_id "lnc-bbut00012301"	exon_number "1"
13	lncRNA	exon	1921199	1921245	.	-	.	gene_id "lnc-bbug05207"	transcript_id "lnc-bbut00012301"	exon_number "2"
13	lncRNA	exon	1921627	1924280	.	-	.	gene_id "lnc-bbug05207"	transcript_id "lnc-bbut00012301"	exon_number "3"
13	lncRNA	exon	1924672	1924813	.	-	.	gene_id "lnc-bbug05207"	transcript_id "lnc-bbut00012301"	exon_number "4"
13	lncRNA	exon	1920669	1920935	.	-	.	gene_id "lnc-bbug05207"	transcript_id "lnc-bbut00012300"	exon_number "1"
13	lncRNA	exon	1921199	1924283	.	-	.	gene_id "lnc-bbug05207"	transcript_id "lnc-bbut00012300"	exon_number "2"
13	lncRNA	exon	1924672	1924813	.	-	.	gene_id "lnc-bbug05207"	transcript_id "lnc-bbut00012300"	exon_number "3"
13	lncRNA	exon	2441594	2442671	.	-	.	gene_id "lnc-bbug05209"	transcript_id "lnc-bbut00012309"	exon_number "1"

13	lncRNA	exon	2442880	2443464	.	-	.	gene_id "lnc-bbug05209"	transcript_id "lnc-bbut00012309"	exon_number "2"
13	lncRNA	exon	2511048	2511683	.	-	.	gene_id "lnc-bbug05210"	transcript_id "lnc-bbut00012310"	exon_number "1"
13	lncRNA	exon	2513542	2513721	.	-	.	gene_id "lnc-bbug05210"	transcript_id "lnc-bbut00012310"	exon_number "2"
13	lncRNA	exon	2514628	2516179	.	-	.	gene_id "lnc-bbug05210"	transcript_id "lnc-bbut00012310"	exon_number "3"
13	lncRNA	exon	2511048	2512169	.	-	.	gene_id "lnc-bbug05210"	transcript_id "lnc-bbut00012312"	exon_number "1"
13	lncRNA	exon	2513542	2513721	.	-	.	gene_id "lnc-bbug05210"	transcript_id "lnc-bbut00012312"	exon_number "2"
13	lncRNA	exon	2514628	2516181	.	-	.	gene_id "lnc-bbug05210"	transcript_id "lnc-bbut00012312"	exon_number "3"
13	lncRNA	exon	2511048	2511683	.	-	.	gene_id "lnc-bbug05210"	transcript_id "lnc-bbut00012313"	exon_number "1"
13	lncRNA	exon	2513542	2513721	.	-	.	gene_id "lnc-bbug05210"	transcript_id "lnc-bbut00012313"	exon_number "2"
13	lncRNA	exon	2514628	2515052	.	-	.	gene_id "lnc-bbug05210"	transcript_id "lnc-bbut00012313"	exon_number "3"
13	lncRNA	exon	2519801	2519911	.	-	.	gene_id "lnc-bbug05210"	transcript_id "lnc-bbut00012313"	exon_number "4"
13	lncRNA	exon	2521149	2521286	.	-	.	gene_id "lnc-bbug05210"	transcript_id "lnc-bbut00012313"	exon_number "5"
13	lncRNA	exon	2720750	2720985	.	-	.	gene_id "lnc-bbug05212"	transcript_id "lnc-bbut00012315"	exon_number "1"
13	lncRNA	exon	2734241	2734505	.	-	.	gene_id "lnc-bbug05212"	transcript_id "lnc-bbut00012315"	exon_number "2"
13	lncRNA	exon	4076269	4077190	.	-	.	gene_id "lnc-bbug05216"	transcript_id "lnc-bbut00012327"	exon_number "1"
13	lncRNA	exon	4080408	4080522	.	-	.	gene_id "lnc-bbug05216"	transcript_id "lnc-bbut00012327"	exon_number "2"
13	lncRNA	exon	4080608	4081307	.	-	.	gene_id "lnc-bbug05216"	transcript_id "lnc-bbut00012327"	exon_number "3"
13	lncRNA	exon	4704543	4705524	.	-	.	gene_id "lnc-bbug05217"	transcript_id "lnc-bbut00012328"	exon_number "1"
13	lncRNA	exon	4706806	4706894	.	-	.	gene_id "lnc-bbug05217"	transcript_id "lnc-bbut00012328"	exon_number "2"
13	lncRNA	exon	4722016	4722154	.	-	.	gene_id "lnc-bbug05217"	transcript_id "lnc-bbut00012328"	exon_number "3"
13	lncRNA	exon	4732233	4733368	.	-	.	gene_id "lnc-bbug05217"	transcript_id "lnc-bbut00012328"	exon_number "4"
13	lncRNA	exon	9754362	9754541	.	-	.	gene_id "lnc-bbug05222"	transcript_id "lnc-bbut00012339"	exon_number "1"
13	lncRNA	exon	9754644	9754759	.	-	.	gene_id "lnc-bbug05222"	transcript_id "lnc-bbut00012339"	exon_number "2"
13	lncRNA	exon	9761038	9761266	.	-	.	gene_id "lnc-bbug05222"	transcript_id "lnc-bbut00012339"	exon_number "3"
13	lncRNA	exon	9765466	9765531	.	-	.	gene_id "lnc-bbug05222"	transcript_id "lnc-bbut00012339"	exon_number "4"
13	lncRNA	exon	9784171	9785059	.	-	.	gene_id "lnc-bbug05222"	transcript_id "lnc-bbut00012339"	exon_number "5"
13	lncRNA	exon	10369922	10370263	.	-	.	gene_id "lnc-bbug05225"	transcript_id "lnc-bbut00012346"	exon_number "1"
13	lncRNA	exon	10390883	10390952	.	-	.	gene_id "lnc-bbug05225"	transcript_id "lnc-bbut00012346"	exon_number "2"
13	lncRNA	exon	10391384	10391529	.	-	.	gene_id "lnc-bbug05225"	transcript_id "lnc-bbut00012346"	exon_number "3"
13	lncRNA	exon	10393822	10394015	.	-	.	gene_id "lnc-bbug05225"	transcript_id "lnc-bbut00012346"	exon_number "4"
13	lncRNA	exon	10761820	10762277	.	-	.	gene_id "lnc-bbug05228"	transcript_id "lnc-bbut00012351"	exon_number "1"
13	lncRNA	exon	10764160	10764410	.	-	.	gene_id "lnc-bbug05228"	transcript_id "lnc-bbut00012351"	exon_number "2"
13	lncRNA	exon	10776161	10779954	.	-	.	gene_id "lnc-bbug05229"	transcript_id "lnc-bbut00012352"	exon_number "1"
13	lncRNA	exon	10801901	10801943	.	-	.	gene_id "lnc-bbug05229"	transcript_id "lnc-bbut00012352"	exon_number "2"
13	lncRNA	exon	10809436	10809626	.	-	.	gene_id "lnc-bbug05229"	transcript_id "lnc-bbut00012352"	exon_number "3"
13	lncRNA	exon	10776161	10779954	.	-	.	gene_id "lnc-bbug05229"	transcript_id "lnc-bbut00012353"	exon_number "1"
13	lncRNA	exon	10809436	10810079	.	-	.	gene_id "lnc-bbug05229"	transcript_id "lnc-bbut00012353"	exon_number "2"
13	lncRNA	exon	10776161	10779954	.	-	.	gene_id "lnc-bbug05229"	transcript_id "lnc-bbut00012354"	exon_number "1"
13	lncRNA	exon	10801837	10801943	.	-	.	gene_id "lnc-bbug05229"	transcript_id "lnc-bbut00012354"	exon_number "2"
13	lncRNA	exon	10809436	10810792	.	-	.	gene_id "lnc-bbug05229"	transcript_id "lnc-bbut00012354"	exon_number "3"
13	lncRNA	exon	10784566	10784974	.	-	.	gene_id "lnc-bbug05229"	transcript_id "lnc-bbut00012355"	exon_number "1"
13	lncRNA	exon	10801837	10801943	.	-	.	gene_id "lnc-bbug05229"	transcript_id "lnc-bbut00012355"	exon_number "2"
13	lncRNA	exon	10809436	10809655	.	-	.	gene_id "lnc-bbug05229"	transcript_id "lnc-bbut00012355"	exon_number "3"
13	lncRNA	exon	10799484	10801943	.	-	.	gene_id "lnc-bbug05229"	transcript_id "lnc-bbut00012356"	exon_number "1"
13	lncRNA	exon	10809436	10810079	.	-	.	gene_id "lnc-bbug05229"	transcript_id "lnc-bbut00012356"	exon_number "2"
13	lncRNA	exon	14736439	14736667	.	-	.	gene_id "lnc-bbug05241"	transcript_id "lnc-bbut00012388"	exon_number "1"
13	lncRNA	exon	14737209	14737319	.	-	.	gene_id "lnc-bbug05241"	transcript_id "lnc-bbut00012388"	exon_number "2"
13	lncRNA	exon	14747876	14748287	.	-	.	gene_id "lnc-bbug05241"	transcript_id "lnc-bbut00012388"	exon_number "3"
13	lncRNA	exon	14768018	14768441	.	-	.	gene_id "lnc-bbug05242"	transcript_id "lnc-bbut00012389"	exon_number "1"
13	lncRNA	exon	15195540	15196228	.	-	.	gene_id "lnc-bbug05244"	transcript_id "lnc-bbut00012391"	exon_number "1"
13	lncRNA	exon	15196424	15196601	.	-	.	gene_id "lnc-bbug05244"	transcript_id "lnc-bbut00012391"	exon_number "2"
13	lncRNA	exon	16164705	16167009	.	-	.	gene_id "lnc-bbug05249"	transcript_id "lnc-bbut00012407"	exon_number "1"

13	lncRNA	exon	16715575	16716004	.	-	.	gene_id "lnc-bbug05251"	transcript_id "lnc-bbut00012409"	exon_number "1"
13	lncRNA	exon	16722044	16722506	.	-	.	gene_id "lnc-bbug05251"	transcript_id "lnc-bbut00012409"	exon_number "2"
13	lncRNA	exon	17091597	17091942	.	-	.	gene_id "lnc-bbug05252"	transcript_id "lnc-bbut00012410"	exon_number "1"
13	lncRNA	exon	17092103	17092354	.	-	.	gene_id "lnc-bbug05252"	transcript_id "lnc-bbut00012410"	exon_number "2"
13	lncRNA	exon	17456158	17456507	.	-	.	gene_id "lnc-bbug05253"	transcript_id "lnc-bbut00012411"	exon_number "1"
13	lncRNA	exon	17456668	17456749	.	-	.	gene_id "lnc-bbug05253"	transcript_id "lnc-bbut00012411"	exon_number "2"
13	lncRNA	exon	18623020	18624308	.	-	.	gene_id "lnc-bbug05261"	transcript_id "lnc-bbut00012433"	exon_number "1"
13	lncRNA	exon	18634259	18634364	.	-	.	gene_id "lnc-bbug05261"	transcript_id "lnc-bbut00012433"	exon_number "2"
13	lncRNA	exon	18634752	18634813	.	-	.	gene_id "lnc-bbug05261"	transcript_id "lnc-bbut00012433"	exon_number "3"
13	lncRNA	exon	19764453	19764877	.	-	.	gene_id "lnc-bbug05266"	transcript_id "lnc-bbut00012441"	exon_number "1"
13	lncRNA	exon	19962726	19963156	.	-	.	gene_id "lnc-bbug05267"	transcript_id "lnc-bbut00012442"	exon_number "1"
13	lncRNA	exon	20495009	20495100	.	-	.	gene_id "lnc-bbug05270"	transcript_id "lnc-bbut00012450"	exon_number "1"
13	lncRNA	exon	20508104	20508205	.	-	.	gene_id "lnc-bbug05270"	transcript_id "lnc-bbut00012450"	exon_number "2"
13	lncRNA	exon	20510953	20511202	.	-	.	gene_id "lnc-bbug05270"	transcript_id "lnc-bbut00012450"	exon_number "3"
13	lncRNA	exon	20512152	20512281	.	-	.	gene_id "lnc-bbug05270"	transcript_id "lnc-bbut00012450"	exon_number "4"
13	lncRNA	exon	20514956	20515045	.	-	.	gene_id "lnc-bbug05270"	transcript_id "lnc-bbut00012450"	exon_number "5"
13	lncRNA	exon	20522304	20522529	.	-	.	gene_id "lnc-bbug05270"	transcript_id "lnc-bbut00012450"	exon_number "6"
13	lncRNA	exon	20547917	20548504	.	-	.	gene_id "lnc-bbug05270"	transcript_id "lnc-bbut00012450"	exon_number "7"
13	lncRNA	exon	20549328	20549426	.	-	.	gene_id "lnc-bbug05270"	transcript_id "lnc-bbut00012450"	exon_number "8"
13	lncRNA	exon	20550045	20550373	.	-	.	gene_id "lnc-bbug05270"	transcript_id "lnc-bbut00012450"	exon_number "9"
13	lncRNA	exon	23673919	23679063	.	-	.	gene_id "lnc-bbug05274"	transcript_id "lnc-bbut00012455"	exon_number "1"
13	lncRNA	exon	25000681	25002910	.	-	.	gene_id "lnc-bbug05275"	transcript_id "lnc-bbut00012456"	exon_number "1"
13	lncRNA	exon	25003001	25003751	.	-	.	gene_id "lnc-bbug05275"	transcript_id "lnc-bbut00012456"	exon_number "2"
13	lncRNA	exon	25000681	25001006	.	-	.	gene_id "lnc-bbug05275"	transcript_id "lnc-bbut00012457"	exon_number "1"
13	lncRNA	exon	25007205	25007274	.	-	.	gene_id "lnc-bbug05275"	transcript_id "lnc-bbut00012457"	exon_number "2"
13	lncRNA	exon	25037642	25038031	.	-	.	gene_id "lnc-bbug05275"	transcript_id "lnc-bbut00012457"	exon_number "3"
13	lncRNA	exon	25000681	25002910	.	-	.	gene_id "lnc-bbug05275"	transcript_id "lnc-bbut00012458"	exon_number "1"
13	lncRNA	exon	25007205	25007274	.	-	.	gene_id "lnc-bbug05275"	transcript_id "lnc-bbut00012458"	exon_number "2"
13	lncRNA	exon	25037642	25037714	.	-	.	gene_id "lnc-bbug05275"	transcript_id "lnc-bbut00012458"	exon_number "3"
13	lncRNA	exon	25039469	25039598	.	-	.	gene_id "lnc-bbug05275"	transcript_id "lnc-bbut00012458"	exon_number "4"
13	lncRNA	exon	25032930	25037714	.	-	.	gene_id "lnc-bbug05275"	transcript_id "lnc-bbut00012459"	exon_number "1"
13	lncRNA	exon	25039469	25039635	.	-	.	gene_id "lnc-bbug05275"	transcript_id "lnc-bbut00012459"	exon_number "2"
13	lncRNA	exon	25094382	25095929	.	-	.	gene_id "lnc-bbug05276"	transcript_id "lnc-bbut00012460"	exon_number "1"
13	lncRNA	exon	25097122	25097242	.	-	.	gene_id "lnc-bbug05276"	transcript_id "lnc-bbut00012460"	exon_number "2"
13	lncRNA	exon	25107124	25107205	.	-	.	gene_id "lnc-bbug05276"	transcript_id "lnc-bbut00012460"	exon_number "3"
13	lncRNA	exon	25126051	25126212	.	-	.	gene_id "lnc-bbug05276"	transcript_id "lnc-bbut00012460"	exon_number "4"
13	lncRNA	exon	29680288	29681166	.	-	.	gene_id "lnc-bbug05279"	transcript_id "lnc-bbut00012464"	exon_number "1"
13	lncRNA	exon	29682615	29682635	.	-	.	gene_id "lnc-bbug05279"	transcript_id "lnc-bbut00012464"	exon_number "2"
13	lncRNA	exon	35187397	35189009	.	-	.	gene_id "lnc-bbug05282"	transcript_id "lnc-bbut00012467"	exon_number "1"
13	lncRNA	exon	35194063	35194240	.	-	.	gene_id "lnc-bbug05282"	transcript_id "lnc-bbut00012467"	exon_number "2"
13	lncRNA	exon	35194885	35194939	.	-	.	gene_id "lnc-bbug05282"	transcript_id "lnc-bbut00012467"	exon_number "3"
13	lncRNA	exon	35195098	35195178	.	-	.	gene_id "lnc-bbug05282"	transcript_id "lnc-bbut00012467"	exon_number "4"
13	lncRNA	exon	35201056	35202030	.	-	.	gene_id "lnc-bbug05282"	transcript_id "lnc-bbut00012467"	exon_number "5"
13	lncRNA	exon	35187397	35189009	.	-	.	gene_id "lnc-bbug05282"	transcript_id "lnc-bbut00012468"	exon_number "1"
13	lncRNA	exon	35194063	35194240	.	-	.	gene_id "lnc-bbug05282"	transcript_id "lnc-bbut00012468"	exon_number "2"
13	lncRNA	exon	35194885	35194939	.	-	.	gene_id "lnc-bbug05282"	transcript_id "lnc-bbut00012468"	exon_number "3"
13	lncRNA	exon	35195098	35195178	.	-	.	gene_id "lnc-bbug05282"	transcript_id "lnc-bbut00012468"	exon_number "4"
13	lncRNA	exon	35216802	35217255	.	-	.	gene_id "lnc-bbug05282"	transcript_id "lnc-bbut00012468"	exon_number "5"
13	lncRNA	exon	35187397	35189009	.	-	.	gene_id "lnc-bbug05282"	transcript_id "lnc-bbut00012469"	exon_number "1"
13	lncRNA	exon	35194063	35194240	.	-	.	gene_id "lnc-bbug05282"	transcript_id "lnc-bbut00012469"	exon_number "2"
13	lncRNA	exon	35194885	35194939	.	-	.	gene_id "lnc-bbug05282"	transcript_id "lnc-bbut00012469"	exon_number "3"
13	lncRNA	exon	35195098	35195178	.	-	.	gene_id "lnc-bbug05282"	transcript_id "lnc-bbut00012469"	exon_number "4"

13	lncRNA	exon	35267143	35267244	.	-	.	gene_id "lnc-bbug05282"	transcript_id "lnc-bbut00012469"	exon_number "5"
13	lncRNA	exon	35271100	35271240	.	-	.	gene_id "lnc-bbug05282"	transcript_id "lnc-bbut00012469"	exon_number "6"
13	lncRNA	exon	35383832	35386027	.	-	.	gene_id "lnc-bbug05284"	transcript_id "lnc-bbut00012471"	exon_number "1"
13	lncRNA	exon	35398321	35398458	.	-	.	gene_id "lnc-bbug05284"	transcript_id "lnc-bbut00012471"	exon_number "2"
13	lncRNA	exon	35403668	35403818	.	-	.	gene_id "lnc-bbug05284"	transcript_id "lnc-bbut00012471"	exon_number "3"
13	lncRNA	exon	35409163	35409400	.	-	.	gene_id "lnc-bbug05284"	transcript_id "lnc-bbut00012471"	exon_number "4"
13	lncRNA	exon	35413031	35413244	.	-	.	gene_id "lnc-bbug05284"	transcript_id "lnc-bbut00012471"	exon_number "5"
13	lncRNA	exon	35504189	35504376	.	-	.	gene_id "lnc-bbug05284"	transcript_id "lnc-bbut00012471"	exon_number "6"
13	lncRNA	exon	35510151	35510278	.	-	.	gene_id "lnc-bbug05284"	transcript_id "lnc-bbut00012471"	exon_number "7"
13	lncRNA	exon	35585200	35585293	.	-	.	gene_id "lnc-bbug05284"	transcript_id "lnc-bbut00012471"	exon_number "8"
13	lncRNA	exon	35612466	35612620	.	-	.	gene_id "lnc-bbug05284"	transcript_id "lnc-bbut00012471"	exon_number "9"
13	lncRNA	exon	35613445	35613563	.	-	.	gene_id "lnc-bbug05284"	transcript_id "lnc-bbut00012471"	exon_number "10"
13	lncRNA	exon	35758249	35758311	.	-	.	gene_id "lnc-bbug05284"	transcript_id "lnc-bbut00012471"	exon_number "11"
13	lncRNA	exon	35835146	35835219	.	-	.	gene_id "lnc-bbug05284"	transcript_id "lnc-bbut00012471"	exon_number "12"
13	lncRNA	exon	35837224	35837324	.	-	.	gene_id "lnc-bbug05284"	transcript_id "lnc-bbut00012471"	exon_number "13"
13	lncRNA	exon	35837749	35838017	.	-	.	gene_id "lnc-bbug05284"	transcript_id "lnc-bbut00012471"	exon_number "14"
13	lncRNA	exon	35865077	35866652	.	-	.	gene_id "lnc-bbug05284"	transcript_id "lnc-bbut00012471"	exon_number "15"
13	lncRNA	exon	35556222	35558045	.	-	.	gene_id "lnc-bbug05284"	transcript_id "lnc-bbut00012477"	exon_number "1"
13	lncRNA	exon	35585200	35585293	.	-	.	gene_id "lnc-bbug05284"	transcript_id "lnc-bbut00012477"	exon_number "2"
13	lncRNA	exon	35612466	35612620	.	-	.	gene_id "lnc-bbug05284"	transcript_id "lnc-bbut00012477"	exon_number "3"
13	lncRNA	exon	35613445	35613563	.	-	.	gene_id "lnc-bbug05284"	transcript_id "lnc-bbut00012477"	exon_number "4"
13	lncRNA	exon	35758249	35758311	.	-	.	gene_id "lnc-bbug05284"	transcript_id "lnc-bbut00012477"	exon_number "5"
13	lncRNA	exon	35835146	35835219	.	-	.	gene_id "lnc-bbug05284"	transcript_id "lnc-bbut00012477"	exon_number "6"
13	lncRNA	exon	35837224	35837324	.	-	.	gene_id "lnc-bbug05284"	transcript_id "lnc-bbut00012477"	exon_number "7"
13	lncRNA	exon	35837749	35838017	.	-	.	gene_id "lnc-bbug05284"	transcript_id "lnc-bbut00012477"	exon_number "8"
13	lncRNA	exon	35865077	35866649	.	-	.	gene_id "lnc-bbug05284"	transcript_id "lnc-bbut00012477"	exon_number "9"
13	lncRNA	exon	35556222	35558045	.	-	.	gene_id "lnc-bbug05284"	transcript_id "lnc-bbut00012476"	exon_number "1"
13	lncRNA	exon	35585200	35585293	.	-	.	gene_id "lnc-bbug05284"	transcript_id "lnc-bbut00012476"	exon_number "2"
13	lncRNA	exon	35613445	35613563	.	-	.	gene_id "lnc-bbug05284"	transcript_id "lnc-bbut00012476"	exon_number "3"
13	lncRNA	exon	35758249	35758311	.	-	.	gene_id "lnc-bbug05284"	transcript_id "lnc-bbut00012476"	exon_number "4"
13	lncRNA	exon	35835146	35835219	.	-	.	gene_id "lnc-bbug05284"	transcript_id "lnc-bbut00012476"	exon_number "5"
13	lncRNA	exon	35837224	35837324	.	-	.	gene_id "lnc-bbug05284"	transcript_id "lnc-bbut00012476"	exon_number "6"
13	lncRNA	exon	35837749	35838017	.	-	.	gene_id "lnc-bbug05284"	transcript_id "lnc-bbut00012476"	exon_number "7"
13	lncRNA	exon	35865077	35866649	.	-	.	gene_id "lnc-bbug05284"	transcript_id "lnc-bbut00012476"	exon_number "8"
13	lncRNA	exon	35575496	35577446	.	-	.	gene_id "lnc-bbug05284"	transcript_id "lnc-bbut00012478"	exon_number "1"
13	lncRNA	exon	35585200	35585293	.	-	.	gene_id "lnc-bbug05284"	transcript_id "lnc-bbut00012478"	exon_number "2"
13	lncRNA	exon	35758249	35758311	.	-	.	gene_id "lnc-bbug05284"	transcript_id "lnc-bbut00012478"	exon_number "3"
13	lncRNA	exon	35837893	35838017	.	-	.	gene_id "lnc-bbug05284"	transcript_id "lnc-bbut00012478"	exon_number "4"
13	lncRNA	exon	35838320	35839725	.	-	.	gene_id "lnc-bbug05284"	transcript_id "lnc-bbut00012478"	exon_number "5"
13	lncRNA	exon	35577457	35577977	.	-	.	gene_id "lnc-bbug05284"	transcript_id "lnc-bbut00012482"	exon_number "1"
13	lncRNA	exon	35585200	35585293	.	-	.	gene_id "lnc-bbug05284"	transcript_id "lnc-bbut00012482"	exon_number "2"
13	lncRNA	exon	35613445	35613563	.	-	.	gene_id "lnc-bbug05284"	transcript_id "lnc-bbut00012482"	exon_number "3"
13	lncRNA	exon	35758249	35758311	.	-	.	gene_id "lnc-bbug05284"	transcript_id "lnc-bbut00012482"	exon_number "4"
13	lncRNA	exon	35835146	35835219	.	-	.	gene_id "lnc-bbug05284"	transcript_id "lnc-bbut00012482"	exon_number "5"
13	lncRNA	exon	35837224	35837324	.	-	.	gene_id "lnc-bbug05284"	transcript_id "lnc-bbut00012482"	exon_number "6"
13	lncRNA	exon	35837749	35838017	.	-	.	gene_id "lnc-bbug05284"	transcript_id "lnc-bbut00012482"	exon_number "7"
13	lncRNA	exon	35865077	35866649	.	-	.	gene_id "lnc-bbug05284"	transcript_id "lnc-bbut00012482"	exon_number "8"
13	lncRNA	exon	35778055	35778200	.	-	.	gene_id "lnc-bbug05284"	transcript_id "lnc-bbut00012489"	exon_number "1"
13	lncRNA	exon	35835146	35835219	.	-	.	gene_id "lnc-bbug05284"	transcript_id "lnc-bbut00012489"	exon_number "2"
13	lncRNA	exon	35837224	35837324	.	-	.	gene_id "lnc-bbug05284"	transcript_id "lnc-bbut00012489"	exon_number "3"
13	lncRNA	exon	35837749	35838017	.	-	.	gene_id "lnc-bbug05284"	transcript_id "lnc-bbut00012489"	exon_number "4"
13	lncRNA	exon	35865077	35866638	.	-	.	gene_id "lnc-bbug05284"	transcript_id "lnc-bbut00012489"	exon_number "5"

13	lncRNA	exon	36167147	36167651	.	-	.	gene_id "lnc-bbug05286"	transcript_id "lnc-bbut00012492"	exon_number "1"
13	lncRNA	exon	36168960	36169039	.	-	.	gene_id "lnc-bbug05286"	transcript_id "lnc-bbut00012492"	exon_number "2"
13	lncRNA	exon	36178003	36178151	.	-	.	gene_id "lnc-bbug05286"	transcript_id "lnc-bbut00012492"	exon_number "3"
13	lncRNA	exon	37280487	37280730	.	-	.	gene_id "lnc-bbug05288"	transcript_id "lnc-bbut00012494"	exon_number "1"
13	lncRNA	exon	37301816	37301986	.	-	.	gene_id "lnc-bbug05288"	transcript_id "lnc-bbut00012494"	exon_number "2"
13	lncRNA	exon	37302079	37302273	.	-	.	gene_id "lnc-bbug05288"	transcript_id "lnc-bbut00012494"	exon_number "3"
13	lncRNA	exon	38144110	38144684	.	-	.	gene_id "lnc-bbug05291"	transcript_id "lnc-bbut00012511"	exon_number "1"
13	lncRNA	exon	38151188	38151287	.	-	.	gene_id "lnc-bbug05291"	transcript_id "lnc-bbut00012511"	exon_number "2"
13	lncRNA	exon	38183006	38183302	.	-	.	gene_id "lnc-bbug05291"	transcript_id "lnc-bbut00012511"	exon_number "3"
13	lncRNA	exon	38232236	38233230	.	-	.	gene_id "lnc-bbug05292"	transcript_id "lnc-bbut00012512"	exon_number "1"
13	lncRNA	exon	38242086	38242236	.	-	.	gene_id "lnc-bbug05292"	transcript_id "lnc-bbut00012512"	exon_number "2"
13	lncRNA	exon	38242925	38243002	.	-	.	gene_id "lnc-bbug05292"	transcript_id "lnc-bbut00012512"	exon_number "3"
13	lncRNA	exon	38243810	38244508	.	-	.	gene_id "lnc-bbug05292"	transcript_id "lnc-bbut00012512"	exon_number "4"
13	lncRNA	exon	38618739	38619202	.	-	.	gene_id "lnc-bbug05293"	transcript_id "lnc-bbut00012513"	exon_number "1"
13	lncRNA	exon	38622112	38622188	.	-	.	gene_id "lnc-bbug05293"	transcript_id "lnc-bbut00012513"	exon_number "2"
13	lncRNA	exon	38624818	38624951	.	-	.	gene_id "lnc-bbug05293"	transcript_id "lnc-bbut00012513"	exon_number "3"
13	lncRNA	exon	38932463	38934267	.	-	.	gene_id "lnc-bbug05296"	transcript_id "lnc-bbut00012518"	exon_number "1"
13	lncRNA	exon	38938976	38939154	.	-	.	gene_id "lnc-bbug05296"	transcript_id "lnc-bbut00012518"	exon_number "2"
13	lncRNA	exon	38939963	38940734	.	-	.	gene_id "lnc-bbug05296"	transcript_id "lnc-bbut00012518"	exon_number "3"
13	lncRNA	exon	39401873	39402378	.	-	.	gene_id "lnc-bbug05297"	transcript_id "lnc-bbut00012519"	exon_number "1"
13	lncRNA	exon	39402594	39402856	.	-	.	gene_id "lnc-bbug05297"	transcript_id "lnc-bbut00012519"	exon_number "2"
13	lncRNA	exon	39403098	39403241	.	-	.	gene_id "lnc-bbug05297"	transcript_id "lnc-bbut00012519"	exon_number "3"
13	lncRNA	exon	40205866	40207197	.	-	.	gene_id "lnc-bbug05300"	transcript_id "lnc-bbut00012529"	exon_number "1"
13	lncRNA	exon	40207316	40207522	.	-	.	gene_id "lnc-bbug05300"	transcript_id "lnc-bbut00012529"	exon_number "2"
13	lncRNA	exon	42622570	42622943	.	-	.	gene_id "lnc-bbug05303"	transcript_id "lnc-bbut00012532"	exon_number "1"
13	lncRNA	exon	42625388	42625569	.	-	.	gene_id "lnc-bbug05303"	transcript_id "lnc-bbut00012532"	exon_number "2"
13	lncRNA	exon	42628242	42628325	.	-	.	gene_id "lnc-bbug05303"	transcript_id "lnc-bbut00012532"	exon_number "3"
13	lncRNA	exon	42643656	42643763	.	-	.	gene_id "lnc-bbug05303"	transcript_id "lnc-bbut00012532"	exon_number "4"
13	lncRNA	exon	44166352	44170008	.	-	.	gene_id "lnc-bbug05307"	transcript_id "lnc-bbut00012542"	exon_number "1"
13	lncRNA	exon	44170091	44170227	.	-	.	gene_id "lnc-bbug05307"	transcript_id "lnc-bbut00012542"	exon_number "2"
13	lncRNA	exon	44182123	44182223	.	-	.	gene_id "lnc-bbug05307"	transcript_id "lnc-bbut00012542"	exon_number "3"
13	lncRNA	exon	45044773	45045358	.	-	.	gene_id "lnc-bbug05308"	transcript_id "lnc-bbut00012543"	exon_number "1"
13	lncRNA	exon	45052161	45052183	.	-	.	gene_id "lnc-bbug05308"	transcript_id "lnc-bbut00012543"	exon_number "2"
13	lncRNA	exon	49817591	49818358	.	-	.	gene_id "lnc-bbug05312"	transcript_id "lnc-bbut00012547"	exon_number "1"
13	lncRNA	exon	52265904	52267245	.	-	.	gene_id "lnc-bbug05313"	transcript_id "lnc-bbut00012548"	exon_number "1"
13	lncRNA	exon	52268436	52268460	.	-	.	gene_id "lnc-bbug05313"	transcript_id "lnc-bbut00012548"	exon_number "2"
13	lncRNA	exon	54159697	54160442	.	-	.	gene_id "lnc-bbug05314"	transcript_id "lnc-bbut00012549"	exon_number "1"
13	lncRNA	exon	54319929	54320879	.	-	.	gene_id "lnc-bbug05316"	transcript_id "lnc-bbut00012556"	exon_number "1"
13	lncRNA	exon	54417343	54418439	.	-	.	gene_id "lnc-bbug05317"	transcript_id "lnc-bbut00012557"	exon_number "1"
13	lncRNA	exon	54430699	54431544	.	-	.	gene_id "lnc-bbug05317"	transcript_id "lnc-bbut00012557"	exon_number "2"
13	lncRNA	exon	54501367	54501987	.	-	.	gene_id "lnc-bbug05319"	transcript_id "lnc-bbut00012560"	exon_number "1"
13	lncRNA	exon	54753544	54754601	.	-	.	gene_id "lnc-bbug05324"	transcript_id "lnc-bbut00012579"	exon_number "1"
13	lncRNA	exon	54756694	54757889	.	-	.	gene_id "lnc-bbug05324"	transcript_id "lnc-bbut00012579"	exon_number "2"
13	lncRNA	exon	56633969	56634772	.	-	.	gene_id "lnc-bbug05336"	transcript_id "lnc-bbut00012614"	exon_number "1"
13	lncRNA	exon	56635337	56635503	.	-	.	gene_id "lnc-bbug05336"	transcript_id "lnc-bbut00012614"	exon_number "2"
13	lncRNA	exon	56703836	56703953	.	-	.	gene_id "lnc-bbug05336"	transcript_id "lnc-bbut00012614"	exon_number "3"
13	lncRNA	exon	57983591	57983769	.	-	.	gene_id "lnc-bbug05343"	transcript_id "lnc-bbut00012630"	exon_number "1"
13	lncRNA	exon	57984535	57987693	.	-	.	gene_id "lnc-bbug05343"	transcript_id "lnc-bbut00012630"	exon_number "2"
13	lncRNA	exon	58828100	58829312	.	-	.	gene_id "lnc-bbug05350"	transcript_id "lnc-bbut00012645"	exon_number "1"
13	lncRNA	exon	59213799	59214359	.	-	.	gene_id "lnc-bbug05352"	transcript_id "lnc-bbut00012650"	exon_number "1"
13	lncRNA	exon	59229070	59229129	.	-	.	gene_id "lnc-bbug05352"	transcript_id "lnc-bbut00012650"	exon_number "2"
13	lncRNA	exon	59255825	59259464	.	-	.	gene_id "lnc-bbug05353"	transcript_id "lnc-bbut00012651"	exon_number "1"

13	lncRNA	exon	59260492	59260699	.	-	.	gene_id "lnc-bbug05353"	transcript_id "lnc-bbut00012651"	exon_number "2"
13	lncRNA	exon	59269654	59269786	.	-	.	gene_id "lnc-bbug05353"	transcript_id "lnc-bbut00012651"	exon_number "3"
13	lncRNA	exon	59896853	59899786	.	-	.	gene_id "lnc-bbug05356"	transcript_id "lnc-bbut00012658"	exon_number "1"
13	lncRNA	exon	59900090	59900167	.	-	.	gene_id "lnc-bbug05356"	transcript_id "lnc-bbut00012658"	exon_number "2"
13	lncRNA	exon	59904526	59904612	.	-	.	gene_id "lnc-bbug05356"	transcript_id "lnc-bbut00012658"	exon_number "3"
13	lncRNA	exon	59909055	59909163	.	-	.	gene_id "lnc-bbug05356"	transcript_id "lnc-bbut00012658"	exon_number "4"
13	lncRNA	exon	59944247	59945270	.	-	.	gene_id "lnc-bbug05356"	transcript_id "lnc-bbut00012658"	exon_number "5"
13	lncRNA	exon	60126139	60127526	.	-	.	gene_id "lnc-bbug05357"	transcript_id "lnc-bbut00012659"	exon_number "1"
13	lncRNA	exon	60130035	60130717	.	-	.	gene_id "lnc-bbug05357"	transcript_id "lnc-bbut00012659"	exon_number "2"
13	lncRNA	exon	61898807	61899650	.	-	.	gene_id "lnc-bbug05363"	transcript_id "lnc-bbut00012676"	exon_number "1"
13	lncRNA	exon	61899920	61899925	.	-	.	gene_id "lnc-bbug05363"	transcript_id "lnc-bbut00012676"	exon_number "2"
13	lncRNA	exon	61993427	61993562	.	-	.	gene_id "lnc-bbug05364"	transcript_id "lnc-bbut00012678"	exon_number "1"
13	lncRNA	exon	61994668	61998926	.	-	.	gene_id "lnc-bbug05364"	transcript_id "lnc-bbut00012678"	exon_number "2"
13	lncRNA	exon	62002758	62002922	.	-	.	gene_id "lnc-bbug05364"	transcript_id "lnc-bbut00012678"	exon_number "3"
13	lncRNA	exon	62003061	62003210	.	-	.	gene_id "lnc-bbug05364"	transcript_id "lnc-bbut00012678"	exon_number "4"
13	lncRNA	exon	62006049	62006573	.	-	.	gene_id "lnc-bbug05364"	transcript_id "lnc-bbut00012678"	exon_number "5"
13	lncRNA	exon	62007717	62007899	.	-	.	gene_id "lnc-bbug05364"	transcript_id "lnc-bbut00012678"	exon_number "6"
13	lncRNA	exon	62010007	62014434	.	-	.	gene_id "lnc-bbug05364"	transcript_id "lnc-bbut00012678"	exon_number "7"
13	lncRNA	exon	62015293	62015408	.	-	.	gene_id "lnc-bbug05364"	transcript_id "lnc-bbut00012678"	exon_number "8"
13	lncRNA	exon	62019054	62019150	.	-	.	gene_id "lnc-bbug05364"	transcript_id "lnc-bbut00012678"	exon_number "9"
13	lncRNA	exon	62020319	62020435	.	-	.	gene_id "lnc-bbug05364"	transcript_id "lnc-bbut00012678"	exon_number "10"
13	lncRNA	exon	62022787	62022920	.	-	.	gene_id "lnc-bbug05364"	transcript_id "lnc-bbut00012678"	exon_number "11"
13	lncRNA	exon	61993427	61993562	.	-	.	gene_id "lnc-bbug05364"	transcript_id "lnc-bbut00012677"	exon_number "1"
13	lncRNA	exon	61994668	61998926	.	-	.	gene_id "lnc-bbug05364"	transcript_id "lnc-bbut00012677"	exon_number "2"
13	lncRNA	exon	62002758	62002922	.	-	.	gene_id "lnc-bbug05364"	transcript_id "lnc-bbut00012677"	exon_number "3"
13	lncRNA	exon	62003061	62003210	.	-	.	gene_id "lnc-bbug05364"	transcript_id "lnc-bbut00012677"	exon_number "4"
13	lncRNA	exon	62006049	62006573	.	-	.	gene_id "lnc-bbug05364"	transcript_id "lnc-bbut00012677"	exon_number "5"
13	lncRNA	exon	62007717	62007899	.	-	.	gene_id "lnc-bbug05364"	transcript_id "lnc-bbut00012677"	exon_number "6"
13	lncRNA	exon	62010007	62014434	.	-	.	gene_id "lnc-bbug05364"	transcript_id "lnc-bbut00012677"	exon_number "7"
13	lncRNA	exon	62015293	62015408	.	-	.	gene_id "lnc-bbug05364"	transcript_id "lnc-bbut00012677"	exon_number "8"
13	lncRNA	exon	62019054	62019146	.	-	.	gene_id "lnc-bbug05364"	transcript_id "lnc-bbut00012677"	exon_number "9"
13	lncRNA	exon	62020319	62020435	.	-	.	gene_id "lnc-bbug05364"	transcript_id "lnc-bbut00012677"	exon_number "10"
13	lncRNA	exon	62022787	62022920	.	-	.	gene_id "lnc-bbug05364"	transcript_id "lnc-bbut00012677"	exon_number "11"
13	lncRNA	exon	62126627	62126760	.	-	.	gene_id "lnc-bbug05365"	transcript_id "lnc-bbut00012693"	exon_number "1"
13	lncRNA	exon	62134084	62134229	.	-	.	gene_id "lnc-bbug05365"	transcript_id "lnc-bbut00012693"	exon_number "2"
13	lncRNA	exon	62136481	62136744	.	-	.	gene_id "lnc-bbug05365"	transcript_id "lnc-bbut00012693"	exon_number "3"
13	lncRNA	exon	62139028	62141532	.	-	.	gene_id "lnc-bbug05366"	transcript_id "lnc-bbut00012696"	exon_number "1"
13	lncRNA	exon	62142860	62142928	.	-	.	gene_id "lnc-bbug05366"	transcript_id "lnc-bbut00012696"	exon_number "2"
13	lncRNA	exon	62145361	62145488	.	-	.	gene_id "lnc-bbug05366"	transcript_id "lnc-bbut00012696"	exon_number "3"
13	lncRNA	exon	62163375	62165197	.	-	.	gene_id "lnc-bbug05366"	transcript_id "lnc-bbut00012696"	exon_number "4"
13	lncRNA	exon	62139028	62141532	.	-	.	gene_id "lnc-bbug05366"	transcript_id "lnc-bbut00012695"	exon_number "1"
13	lncRNA	exon	62145361	62145488	.	-	.	gene_id "lnc-bbug05366"	transcript_id "lnc-bbut00012695"	exon_number "2"
13	lncRNA	exon	62163375	62165197	.	-	.	gene_id "lnc-bbug05366"	transcript_id "lnc-bbut00012695"	exon_number "3"
13	lncRNA	exon	63418928	63418970	.	-	.	gene_id "lnc-bbug05370"	transcript_id "lnc-bbut00012718"	exon_number "1"
13	lncRNA	exon	63419626	63419793	.	-	.	gene_id "lnc-bbug05370"	transcript_id "lnc-bbut00012718"	exon_number "2"
13	lncRNA	exon	63424977	63425329	.	-	.	gene_id "lnc-bbug05370"	transcript_id "lnc-bbut00012718"	exon_number "3"
13	lncRNA	exon	63689419	63689475	.	-	.	gene_id "lnc-bbug05371"	transcript_id "lnc-bbut00012719"	exon_number "1"
13	lncRNA	exon	63690545	63690704	.	-	.	gene_id "lnc-bbug05371"	transcript_id "lnc-bbut00012719"	exon_number "2"
13	lncRNA	exon	63756744	63756820	.	-	.	gene_id "lnc-bbug05371"	transcript_id "lnc-bbut00012719"	exon_number "3"
13	lncRNA	exon	63758375	63758580	.	-	.	gene_id "lnc-bbug05371"	transcript_id "lnc-bbut00012719"	exon_number "4"
13	lncRNA	exon	63823074	63823326	.	-	.	gene_id "lnc-bbug05371"	transcript_id "lnc-bbut00012719"	exon_number "5"
13	lncRNA	exon	63971967	63972004	.	-	.	gene_id "lnc-bbug05371"	transcript_id "lnc-bbut00012719"	exon_number "6"

13	lncRNA	exon	64024976	64025069	.	-	.	gene_id "lnc-bbug05371"	transcript_id "lnc-bbut00012719"	exon_number "7"
13	lncRNA	exon	64086839	64086933	.	-	.	gene_id "lnc-bbug05371"	transcript_id "lnc-bbut00012719"	exon_number "8"
13	lncRNA	exon	64180273	64180385	.	-	.	gene_id "lnc-bbug05371"	transcript_id "lnc-bbut00012719"	exon_number "9"
13	lncRNA	exon	64286588	64286672	.	-	.	gene_id "lnc-bbug05371"	transcript_id "lnc-bbut00012719"	exon_number "10"
13	lncRNA	exon	64297258	64297324	.	-	.	gene_id "lnc-bbug05371"	transcript_id "lnc-bbut00012719"	exon_number "11"
13	lncRNA	exon	64297503	64297735	.	-	.	gene_id "lnc-bbug05371"	transcript_id "lnc-bbut00012719"	exon_number "12"
13	lncRNA	exon	63725620	63725918	.	-	.	gene_id "lnc-bbug05371"	transcript_id "lnc-bbut00012720"	exon_number "1"
13	lncRNA	exon	63756744	63756820	.	-	.	gene_id "lnc-bbug05371"	transcript_id "lnc-bbut00012720"	exon_number "2"
13	lncRNA	exon	63758375	63758580	.	-	.	gene_id "lnc-bbug05371"	transcript_id "lnc-bbut00012720"	exon_number "3"
13	lncRNA	exon	63823074	63823326	.	-	.	gene_id "lnc-bbug05371"	transcript_id "lnc-bbut00012720"	exon_number "4"
13	lncRNA	exon	63971967	63972004	.	-	.	gene_id "lnc-bbug05371"	transcript_id "lnc-bbut00012720"	exon_number "5"
13	lncRNA	exon	64024976	64025069	.	-	.	gene_id "lnc-bbug05371"	transcript_id "lnc-bbut00012720"	exon_number "6"
13	lncRNA	exon	64286588	64286672	.	-	.	gene_id "lnc-bbug05371"	transcript_id "lnc-bbut00012720"	exon_number "7"
13	lncRNA	exon	64297258	64297324	.	-	.	gene_id "lnc-bbug05371"	transcript_id "lnc-bbut00012720"	exon_number "8"
13	lncRNA	exon	64297503	64297729	.	-	.	gene_id "lnc-bbug05371"	transcript_id "lnc-bbut00012720"	exon_number "9"
13	lncRNA	exon	63725620	63725918	.	-	.	gene_id "lnc-bbug05371"	transcript_id "lnc-bbut00012722"	exon_number "1"
13	lncRNA	exon	63756744	63756820	.	-	.	gene_id "lnc-bbug05371"	transcript_id "lnc-bbut00012722"	exon_number "2"
13	lncRNA	exon	63758375	63758580	.	-	.	gene_id "lnc-bbug05371"	transcript_id "lnc-bbut00012722"	exon_number "3"
13	lncRNA	exon	63823074	63823326	.	-	.	gene_id "lnc-bbug05371"	transcript_id "lnc-bbut00012722"	exon_number "4"
13	lncRNA	exon	63971967	63972004	.	-	.	gene_id "lnc-bbug05371"	transcript_id "lnc-bbut00012722"	exon_number "5"
13	lncRNA	exon	64024976	64025069	.	-	.	gene_id "lnc-bbug05371"	transcript_id "lnc-bbut00012722"	exon_number "6"
13	lncRNA	exon	64180273	64180385	.	-	.	gene_id "lnc-bbug05371"	transcript_id "lnc-bbut00012722"	exon_number "7"
13	lncRNA	exon	64286588	64286672	.	-	.	gene_id "lnc-bbug05371"	transcript_id "lnc-bbut00012722"	exon_number "8"
13	lncRNA	exon	64297258	64297324	.	-	.	gene_id "lnc-bbug05371"	transcript_id "lnc-bbut00012722"	exon_number "9"
13	lncRNA	exon	64297503	64297731	.	-	.	gene_id "lnc-bbug05371"	transcript_id "lnc-bbut00012722"	exon_number "10"
13	lncRNA	exon	63725620	63725918	.	-	.	gene_id "lnc-bbug05371"	transcript_id "lnc-bbut00012721"	exon_number "1"
13	lncRNA	exon	63756744	63756820	.	-	.	gene_id "lnc-bbug05371"	transcript_id "lnc-bbut00012721"	exon_number "2"
13	lncRNA	exon	63758375	63758580	.	-	.	gene_id "lnc-bbug05371"	transcript_id "lnc-bbut00012721"	exon_number "3"
13	lncRNA	exon	63823074	63823326	.	-	.	gene_id "lnc-bbug05371"	transcript_id "lnc-bbut00012721"	exon_number "4"
13	lncRNA	exon	63971967	63972004	.	-	.	gene_id "lnc-bbug05371"	transcript_id "lnc-bbut00012721"	exon_number "5"
13	lncRNA	exon	64024976	64025069	.	-	.	gene_id "lnc-bbug05371"	transcript_id "lnc-bbut00012721"	exon_number "6"
13	lncRNA	exon	64086839	64086933	.	-	.	gene_id "lnc-bbug05371"	transcript_id "lnc-bbut00012721"	exon_number "7"
13	lncRNA	exon	64286588	64286672	.	-	.	gene_id "lnc-bbug05371"	transcript_id "lnc-bbut00012721"	exon_number "8"
13	lncRNA	exon	64297258	64297324	.	-	.	gene_id "lnc-bbug05371"	transcript_id "lnc-bbut00012721"	exon_number "9"
13	lncRNA	exon	64297503	64297731	.	-	.	gene_id "lnc-bbug05371"	transcript_id "lnc-bbut00012721"	exon_number "10"
13	lncRNA	exon	63725620	63725918	.	-	.	gene_id "lnc-bbug05371"	transcript_id "lnc-bbut00012723"	exon_number "1"
13	lncRNA	exon	63756744	63756820	.	-	.	gene_id "lnc-bbug05371"	transcript_id "lnc-bbut00012723"	exon_number "2"
13	lncRNA	exon	63758375	63758580	.	-	.	gene_id "lnc-bbug05371"	transcript_id "lnc-bbut00012723"	exon_number "3"
13	lncRNA	exon	63823074	63823326	.	-	.	gene_id "lnc-bbug05371"	transcript_id "lnc-bbut00012723"	exon_number "4"
13	lncRNA	exon	64024976	64025069	.	-	.	gene_id "lnc-bbug05371"	transcript_id "lnc-bbut00012723"	exon_number "5"
13	lncRNA	exon	64086839	64086933	.	-	.	gene_id "lnc-bbug05371"	transcript_id "lnc-bbut00012723"	exon_number "6"
13	lncRNA	exon	64180273	64180385	.	-	.	gene_id "lnc-bbug05371"	transcript_id "lnc-bbut00012723"	exon_number "7"
13	lncRNA	exon	64286588	64286672	.	-	.	gene_id "lnc-bbug05371"	transcript_id "lnc-bbut00012723"	exon_number "8"
13	lncRNA	exon	64297258	64297324	.	-	.	gene_id "lnc-bbug05371"	transcript_id "lnc-bbut00012723"	exon_number "9"
13	lncRNA	exon	64297503	64297732	.	-	.	gene_id "lnc-bbug05371"	transcript_id "lnc-bbut00012723"	exon_number "10"
13	lncRNA	exon	63725620	63725918	.	-	.	gene_id "lnc-bbug05371"	transcript_id "lnc-bbut00012725"	exon_number "1"
13	lncRNA	exon	63756744	63756820	.	-	.	gene_id "lnc-bbug05371"	transcript_id "lnc-bbut00012725"	exon_number "2"
13	lncRNA	exon	63758375	63758580	.	-	.	gene_id "lnc-bbug05371"	transcript_id "lnc-bbut00012725"	exon_number "3"
13	lncRNA	exon	63823074	63823326	.	-	.	gene_id "lnc-bbug05371"	transcript_id "lnc-bbut00012725"	exon_number "4"
13	lncRNA	exon	63971967	63972004	.	-	.	gene_id "lnc-bbug05371"	transcript_id "lnc-bbut00012725"	exon_number "5"
13	lncRNA	exon	64024976	64025069	.	-	.	gene_id "lnc-bbug05371"	transcript_id "lnc-bbut00012725"	exon_number "6"
13	lncRNA	exon	64086839	64086933	.	-	.	gene_id "lnc-bbug05371"	transcript_id "lnc-bbut00012725"	exon_number "7"

13	lncRNA	exon	64180273	64180385	.	-	.	gene_id "lnc-bbug05371"	transcript_id "lnc-bbut00012725"	exon_number "8"
13	lncRNA	exon	64286588	64286672	.	-	.	gene_id "lnc-bbug05371"	transcript_id "lnc-bbut00012725"	exon_number "9"
13	lncRNA	exon	64297258	64297324	.	-	.	gene_id "lnc-bbug05371"	transcript_id "lnc-bbut00012725"	exon_number "10"
13	lncRNA	exon	64297503	64297733	.	-	.	gene_id "lnc-bbug05371"	transcript_id "lnc-bbut00012725"	exon_number "11"
13	lncRNA	exon	63725620	63725918	.	-	.	gene_id "lnc-bbug05371"	transcript_id "lnc-bbut00012724"	exon_number "1"
13	lncRNA	exon	63756744	63756820	.	-	.	gene_id "lnc-bbug05371"	transcript_id "lnc-bbut00012724"	exon_number "2"
13	lncRNA	exon	63758375	63758580	.	-	.	gene_id "lnc-bbug05371"	transcript_id "lnc-bbut00012724"	exon_number "3"
13	lncRNA	exon	63823074	63823326	.	-	.	gene_id "lnc-bbug05371"	transcript_id "lnc-bbut00012724"	exon_number "4"
13	lncRNA	exon	63971967	63972004	.	-	.	gene_id "lnc-bbug05371"	transcript_id "lnc-bbut00012724"	exon_number "5"
13	lncRNA	exon	64024976	64025069	.	-	.	gene_id "lnc-bbug05371"	transcript_id "lnc-bbut00012724"	exon_number "6"
13	lncRNA	exon	64086839	64086933	.	-	.	gene_id "lnc-bbug05371"	transcript_id "lnc-bbut00012724"	exon_number "7"
13	lncRNA	exon	64180273	64180385	.	-	.	gene_id "lnc-bbug05371"	transcript_id "lnc-bbut00012724"	exon_number "8"
13	lncRNA	exon	64286588	64286672	.	-	.	gene_id "lnc-bbug05371"	transcript_id "lnc-bbut00012724"	exon_number "9"
13	lncRNA	exon	64297503	64297733	.	-	.	gene_id "lnc-bbug05371"	transcript_id "lnc-bbut00012724"	exon_number "10"
13	lncRNA	exon	63725620	63725918	.	-	.	gene_id "lnc-bbug05371"	transcript_id "lnc-bbut00012727"	exon_number "1"
13	lncRNA	exon	63756744	63756820	.	-	.	gene_id "lnc-bbug05371"	transcript_id "lnc-bbut00012727"	exon_number "2"
13	lncRNA	exon	63758375	63758580	.	-	.	gene_id "lnc-bbug05371"	transcript_id "lnc-bbut00012727"	exon_number "3"
13	lncRNA	exon	63823074	63823326	.	-	.	gene_id "lnc-bbug05371"	transcript_id "lnc-bbut00012727"	exon_number "4"
13	lncRNA	exon	63971967	63972004	.	-	.	gene_id "lnc-bbug05371"	transcript_id "lnc-bbut00012727"	exon_number "5"
13	lncRNA	exon	64024976	64025069	.	-	.	gene_id "lnc-bbug05371"	transcript_id "lnc-bbut00012727"	exon_number "6"
13	lncRNA	exon	64086839	64086933	.	-	.	gene_id "lnc-bbug05371"	transcript_id "lnc-bbut00012727"	exon_number "7"
13	lncRNA	exon	64180273	64180385	.	-	.	gene_id "lnc-bbug05371"	transcript_id "lnc-bbut00012727"	exon_number "8"
13	lncRNA	exon	64286588	64286672	.	-	.	gene_id "lnc-bbug05371"	transcript_id "lnc-bbut00012727"	exon_number "9"
13	lncRNA	exon	64287784	64287871	.	-	.	gene_id "lnc-bbug05371"	transcript_id "lnc-bbut00012727"	exon_number "10"
13	lncRNA	exon	64297258	64297324	.	-	.	gene_id "lnc-bbug05371"	transcript_id "lnc-bbut00012727"	exon_number "11"
13	lncRNA	exon	64297503	64297734	.	-	.	gene_id "lnc-bbug05371"	transcript_id "lnc-bbut00012727"	exon_number "12"
13	lncRNA	exon	63725620	63725918	.	-	.	gene_id "lnc-bbug05371"	transcript_id "lnc-bbut00012726"	exon_number "1"
13	lncRNA	exon	63756744	63756820	.	-	.	gene_id "lnc-bbug05371"	transcript_id "lnc-bbut00012726"	exon_number "2"
13	lncRNA	exon	63758375	63758580	.	-	.	gene_id "lnc-bbug05371"	transcript_id "lnc-bbut00012726"	exon_number "3"
13	lncRNA	exon	63823074	63823326	.	-	.	gene_id "lnc-bbug05371"	transcript_id "lnc-bbut00012726"	exon_number "4"
13	lncRNA	exon	63971967	63972004	.	-	.	gene_id "lnc-bbug05371"	transcript_id "lnc-bbut00012726"	exon_number "5"
13	lncRNA	exon	64024976	64025069	.	-	.	gene_id "lnc-bbug05371"	transcript_id "lnc-bbut00012726"	exon_number "6"
13	lncRNA	exon	64086839	64086933	.	-	.	gene_id "lnc-bbug05371"	transcript_id "lnc-bbut00012726"	exon_number "7"
13	lncRNA	exon	64180273	64180385	.	-	.	gene_id "lnc-bbug05371"	transcript_id "lnc-bbut00012726"	exon_number "8"
13	lncRNA	exon	64286588	64286672	.	-	.	gene_id "lnc-bbug05371"	transcript_id "lnc-bbut00012726"	exon_number "9"
13	lncRNA	exon	64287784	64287871	.	-	.	gene_id "lnc-bbug05371"	transcript_id "lnc-bbut00012726"	exon_number "10"
13	lncRNA	exon	64297503	64297734	.	-	.	gene_id "lnc-bbug05371"	transcript_id "lnc-bbut00012726"	exon_number "11"
13	lncRNA	exon	63725620	63725918	.	-	.	gene_id "lnc-bbug05371"	transcript_id "lnc-bbut00012728"	exon_number "1"
13	lncRNA	exon	63756744	63756820	.	-	.	gene_id "lnc-bbug05371"	transcript_id "lnc-bbut00012728"	exon_number "2"
13	lncRNA	exon	63758375	63758580	.	-	.	gene_id "lnc-bbug05371"	transcript_id "lnc-bbut00012728"	exon_number "3"
13	lncRNA	exon	63787424	63787538	.	-	.	gene_id "lnc-bbug05371"	transcript_id "lnc-bbut00012728"	exon_number "4"
13	lncRNA	exon	63823074	63823326	.	-	.	gene_id "lnc-bbug05371"	transcript_id "lnc-bbut00012728"	exon_number "5"
13	lncRNA	exon	63971967	63972004	.	-	.	gene_id "lnc-bbug05371"	transcript_id "lnc-bbut00012728"	exon_number "6"
13	lncRNA	exon	64024976	64025069	.	-	.	gene_id "lnc-bbug05371"	transcript_id "lnc-bbut00012728"	exon_number "7"
13	lncRNA	exon	64086839	64086933	.	-	.	gene_id "lnc-bbug05371"	transcript_id "lnc-bbut00012728"	exon_number "8"
13	lncRNA	exon	64180273	64180385	.	-	.	gene_id "lnc-bbug05371"	transcript_id "lnc-bbut00012728"	exon_number "9"
13	lncRNA	exon	64286588	64286672	.	-	.	gene_id "lnc-bbug05371"	transcript_id "lnc-bbut00012728"	exon_number "10"
13	lncRNA	exon	64297258	64297324	.	-	.	gene_id "lnc-bbug05371"	transcript_id "lnc-bbut00012728"	exon_number "11"
13	lncRNA	exon	64297503	64297736	.	-	.	gene_id "lnc-bbug05371"	transcript_id "lnc-bbut00012728"	exon_number "12"
13	lncRNA	exon	64728995	64729101	.	-	.	gene_id "lnc-bbug05372"	transcript_id "lnc-bbut00012729"	exon_number "1"
13	lncRNA	exon	64738514	64738633	.	-	.	gene_id "lnc-bbug05372"	transcript_id "lnc-bbut00012729"	exon_number "2"
13	lncRNA	exon	64747328	64747433	.	-	.	gene_id "lnc-bbug05372"	transcript_id "lnc-bbut00012729"	exon_number "3"

13	lncRNA	exon	64752604	64752907	.	-	.	gene_id "lnc-bbug05372"	transcript_id "lnc-bbut00012729"	exon_number "4"
13	lncRNA	exon	65419232	65419534	.	-	.	gene_id "lnc-bbug05374"	transcript_id "lnc-bbut00012734"	exon_number "1"
13	lncRNA	exon	65485664	65488521	.	-	.	gene_id "lnc-bbug05376"	transcript_id "lnc-bbut00012737"	exon_number "1"
13	lncRNA	exon	65494726	65494802	.	-	.	gene_id "lnc-bbug05376"	transcript_id "lnc-bbut00012737"	exon_number "2"
13	lncRNA	exon	65497649	65497762	.	-	.	gene_id "lnc-bbug05376"	transcript_id "lnc-bbut00012737"	exon_number "3"
13	lncRNA	exon	67539926	67540135	.	-	.	gene_id "lnc-bbug05385"	transcript_id "lnc-bbut00012775"	exon_number "1"
13	lncRNA	exon	67546198	67546928	.	-	.	gene_id "lnc-bbug05385"	transcript_id "lnc-bbut00012775"	exon_number "2"
13	lncRNA	exon	68220698	68220756	.	-	.	gene_id "lnc-bbug05387"	transcript_id "lnc-bbut00012778"	exon_number "1"
13	lncRNA	exon	68223633	68225393	.	-	.	gene_id "lnc-bbug05387"	transcript_id "lnc-bbut00012778"	exon_number "2"
13	lncRNA	exon	68227050	68227311	.	-	.	gene_id "lnc-bbug05387"	transcript_id "lnc-bbut00012778"	exon_number "3"
13	lncRNA	exon	68222226	68223435	.	-	.	gene_id "lnc-bbug05387"	transcript_id "lnc-bbut00012779"	exon_number "1"
13	lncRNA	exon	68223633	68224648	.	-	.	gene_id "lnc-bbug05387"	transcript_id "lnc-bbut00012779"	exon_number "2"
13	lncRNA	exon	68224794	68225393	.	-	.	gene_id "lnc-bbug05387"	transcript_id "lnc-bbut00012779"	exon_number "3"
13	lncRNA	exon	68227050	68227311	.	-	.	gene_id "lnc-bbug05387"	transcript_id "lnc-bbut00012779"	exon_number "4"
13	lncRNA	exon	68310628	68312361	.	-	.	gene_id "lnc-bbug05388"	transcript_id "lnc-bbut00012780"	exon_number "1"
13	lncRNA	exon	68313192	68313240	.	-	.	gene_id "lnc-bbug05388"	transcript_id "lnc-bbut00012780"	exon_number "2"
13	lncRNA	exon	68320592	68320668	.	-	.	gene_id "lnc-bbug05388"	transcript_id "lnc-bbut00012780"	exon_number "3"
13	lncRNA	exon	68321257	68321311	.	-	.	gene_id "lnc-bbug05388"	transcript_id "lnc-bbut00012780"	exon_number "4"
13	lncRNA	exon	68310628	68312361	.	-	.	gene_id "lnc-bbug05388"	transcript_id "lnc-bbut00012781"	exon_number "1"
13	lncRNA	exon	68313192	68313240	.	-	.	gene_id "lnc-bbug05388"	transcript_id "lnc-bbut00012781"	exon_number "2"
13	lncRNA	exon	68321257	68321317	.	-	.	gene_id "lnc-bbug05388"	transcript_id "lnc-bbut00012781"	exon_number "3"
13	lncRNA	exon	68310628	68312361	.	-	.	gene_id "lnc-bbug05388"	transcript_id "lnc-bbut00012782"	exon_number "1"
13	lncRNA	exon	68313192	68313240	.	-	.	gene_id "lnc-bbug05388"	transcript_id "lnc-bbut00012782"	exon_number "2"
13	lncRNA	exon	68410063	68410135	.	-	.	gene_id "lnc-bbug05388"	transcript_id "lnc-bbut00012782"	exon_number "3"
13	lncRNA	exon	68417168	68417490	.	-	.	gene_id "lnc-bbug05388"	transcript_id "lnc-bbut00012782"	exon_number "4"
13	lncRNA	exon	68310628	68312361	.	-	.	gene_id "lnc-bbug05388"	transcript_id "lnc-bbut00012783"	exon_number "1"
13	lncRNA	exon	68313192	68313240	.	-	.	gene_id "lnc-bbug05388"	transcript_id "lnc-bbut00012783"	exon_number "2"
13	lncRNA	exon	68410063	68410135	.	-	.	gene_id "lnc-bbug05388"	transcript_id "lnc-bbut00012783"	exon_number "3"
13	lncRNA	exon	68486848	68487971	.	-	.	gene_id "lnc-bbug05388"	transcript_id "lnc-bbut00012783"	exon_number "4"
13	lncRNA	exon	68527645	68527954	.	-	.	gene_id "lnc-bbug05389"	transcript_id "lnc-bbut00012784"	exon_number "1"
13	lncRNA	exon	68591865	68595162	.	-	.	gene_id "lnc-bbug05389"	transcript_id "lnc-bbut00012784"	exon_number "2"
13	lncRNA	exon	68583143	68583612	.	-	.	gene_id "lnc-bbug05390"	transcript_id "lnc-bbut00012785"	exon_number "1"
13	lncRNA	exon	68583952	68589016	.	-	.	gene_id "lnc-bbug05390"	transcript_id "lnc-bbut00012785"	exon_number "2"
13	lncRNA	exon	69504031	69508289	.	-	.	gene_id "lnc-bbug05399"	transcript_id "lnc-bbut00012808"	exon_number "1"
13	lncRNA	exon	69508499	69509561	.	-	.	gene_id "lnc-bbug05399"	transcript_id "lnc-bbut00012808"	exon_number "2"
13	lncRNA	exon	69960593	69961825	.	-	.	gene_id "lnc-bbug05402"	transcript_id "lnc-bbut00012812"	exon_number "1"
13	lncRNA	exon	70316654	70316787	.	-	.	gene_id "lnc-bbug05404"	transcript_id "lnc-bbut00012817"	exon_number "1"
13	lncRNA	exon	70318267	70318357	.	-	.	gene_id "lnc-bbug05404"	transcript_id "lnc-bbut00012817"	exon_number "2"
13	lncRNA	exon	70322209	70322623	.	-	.	gene_id "lnc-bbug05404"	transcript_id "lnc-bbut00012817"	exon_number "3"
13	lncRNA	exon	70493060	70498172	.	-	.	gene_id "lnc-bbug05405"	transcript_id "lnc-bbut00012818"	exon_number "1"
13	lncRNA	exon	70506543	70506676	.	-	.	gene_id "lnc-bbug05405"	transcript_id "lnc-bbut00012818"	exon_number "2"
13	lncRNA	exon	70525615	70525714	.	-	.	gene_id "lnc-bbug05405"	transcript_id "lnc-bbut00012818"	exon_number "3"
13	lncRNA	exon	70560396	70560675	.	-	.	gene_id "lnc-bbug05405"	transcript_id "lnc-bbut00012818"	exon_number "4"
13	lncRNA	exon	70737795	70737997	.	-	.	gene_id "lnc-bbug05405"	transcript_id "lnc-bbut00012818"	exon_number "5"
13	lncRNA	exon	70795111	70795239	.	-	.	gene_id "lnc-bbug05405"	transcript_id "lnc-bbut00012818"	exon_number "6"
13	lncRNA	exon	70799582	70800364	.	-	.	gene_id "lnc-bbug05405"	transcript_id "lnc-bbut00012818"	exon_number "7"
13	lncRNA	exon	70955224	70956865	.	-	.	gene_id "lnc-bbug05406"	transcript_id "lnc-bbut00012819"	exon_number "1"
13	lncRNA	exon	70960467	70960588	.	-	.	gene_id "lnc-bbug05406"	transcript_id "lnc-bbut00012819"	exon_number "2"
13	lncRNA	exon	71038805	71039931	.	-	.	gene_id "lnc-bbug05407"	transcript_id "lnc-bbut00012820"	exon_number "1"
13	lncRNA	exon	71045303	71045408	.	-	.	gene_id "lnc-bbug05407"	transcript_id "lnc-bbut00012820"	exon_number "2"
13	lncRNA	exon	71045719	71045811	.	-	.	gene_id "lnc-bbug05407"	transcript_id "lnc-bbut00012820"	exon_number "3"
13	lncRNA	exon	71050047	71050620	.	-	.	gene_id "lnc-bbug05407"	transcript_id "lnc-bbut00012820"	exon_number "4"

13	lncRNA	exon	71064684	71065449	.	-	.	gene_id "lnc-bbug05408"	transcript_id "lnc-bbut00012821"	exon_number "1"
13	lncRNA	exon	71084292	71084390	.	-	.	gene_id "lnc-bbug05408"	transcript_id "lnc-bbut00012821"	exon_number "2"
13	lncRNA	exon	71085121	71085226	.	-	.	gene_id "lnc-bbug05408"	transcript_id "lnc-bbut00012821"	exon_number "3"
13	lncRNA	exon	71102656	71102789	.	-	.	gene_id "lnc-bbug05408"	transcript_id "lnc-bbut00012821"	exon_number "4"
13	lncRNA	exon	71075116	71075375	.	-	.	gene_id "lnc-bbug05408"	transcript_id "lnc-bbut00012823"	exon_number "1"
13	lncRNA	exon	71084292	71084406	.	-	.	gene_id "lnc-bbug05408"	transcript_id "lnc-bbut00012823"	exon_number "2"
13	lncRNA	exon	71085121	71085226	.	-	.	gene_id "lnc-bbug05408"	transcript_id "lnc-bbut00012823"	exon_number "3"
13	lncRNA	exon	71102656	71102789	.	-	.	gene_id "lnc-bbug05408"	transcript_id "lnc-bbut00012823"	exon_number "4"
13	lncRNA	exon	71075116	71075375	.	-	.	gene_id "lnc-bbug05408"	transcript_id "lnc-bbut00012822"	exon_number "1"
13	lncRNA	exon	71084292	71084390	.	-	.	gene_id "lnc-bbug05408"	transcript_id "lnc-bbut00012822"	exon_number "2"
13	lncRNA	exon	71085121	71085226	.	-	.	gene_id "lnc-bbug05408"	transcript_id "lnc-bbut00012822"	exon_number "3"
13	lncRNA	exon	71102656	71102789	.	-	.	gene_id "lnc-bbug05408"	transcript_id "lnc-bbut00012822"	exon_number "4"
13	lncRNA	exon	72076754	72077977	.	-	.	gene_id "lnc-bbug05417"	transcript_id "lnc-bbut00012852"	exon_number "1"
13	lncRNA	exon	72078068	72078120	.	-	.	gene_id "lnc-bbug05417"	transcript_id "lnc-bbut00012852"	exon_number "2"
13	lncRNA	exon	72080187	72081177	.	-	.	gene_id "lnc-bbug05417"	transcript_id "lnc-bbut00012852"	exon_number "3"
13	lncRNA	exon	72345436	72346133	.	-	.	gene_id "lnc-bbug05419"	transcript_id "lnc-bbut00012857"	exon_number "1"
13	lncRNA	exon	72348402	72349334	.	-	.	gene_id "lnc-bbug05419"	transcript_id "lnc-bbut00012857"	exon_number "2"
13	lncRNA	exon	72688592	72688724	.	-	.	gene_id "lnc-bbug05422"	transcript_id "lnc-bbut00012861"	exon_number "1"
13	lncRNA	exon	72689158	72689658	.	-	.	gene_id "lnc-bbug05422"	transcript_id "lnc-bbut00012861"	exon_number "2"
13	lncRNA	exon	73543712	73544038	.	-	.	gene_id "lnc-bbug05424"	transcript_id "lnc-bbut00012863"	exon_number "1"
13	lncRNA	exon	73544546	73544658	.	-	.	gene_id "lnc-bbug05424"	transcript_id "lnc-bbut00012863"	exon_number "2"
13	lncRNA	exon	73675105	73676243	.	-	.	gene_id "lnc-bbug05424"	transcript_id "lnc-bbut00012863"	exon_number "3"
13	lncRNA	exon	73543712	73544038	.	-	.	gene_id "lnc-bbug05424"	transcript_id "lnc-bbut00012864"	exon_number "1"
13	lncRNA	exon	73544546	73544658	.	-	.	gene_id "lnc-bbug05424"	transcript_id "lnc-bbut00012864"	exon_number "2"
13	lncRNA	exon	73735322	73735365	.	-	.	gene_id "lnc-bbug05424"	transcript_id "lnc-bbut00012864"	exon_number "3"
13	lncRNA	exon	73735498	73735655	.	-	.	gene_id "lnc-bbug05424"	transcript_id "lnc-bbut00012864"	exon_number "4"
13	lncRNA	exon	74297491	74297868	.	-	.	gene_id "lnc-bbug05427"	transcript_id "lnc-bbut00012869"	exon_number "1"
13	lncRNA	exon	74299026	74300558	.	-	.	gene_id "lnc-bbug05427"	transcript_id "lnc-bbut00012869"	exon_number "2"
13	lncRNA	exon	74330004	74330366	.	-	.	gene_id "lnc-bbug05428"	transcript_id "lnc-bbut00012870"	exon_number "1"
13	lncRNA	exon	74331532	74331642	.	-	.	gene_id "lnc-bbug05428"	transcript_id "lnc-bbut00012870"	exon_number "2"
13	lncRNA	exon	75089253	75090127	.	-	.	gene_id "lnc-bbug05432"	transcript_id "lnc-bbut00012879"	exon_number "1"
13	lncRNA	exon	75110103	75110178	.	-	.	gene_id "lnc-bbug05432"	transcript_id "lnc-bbut00012879"	exon_number "2"
13	lncRNA	exon	75112077	75112438	.	-	.	gene_id "lnc-bbug05432"	transcript_id "lnc-bbut00012879"	exon_number "3"
13	lncRNA	exon	75569253	75569580	.	-	.	gene_id "lnc-bbug05435"	transcript_id "lnc-bbut00012887"	exon_number "1"
13	lncRNA	exon	75572999	75573108	.	-	.	gene_id "lnc-bbug05435"	transcript_id "lnc-bbut00012887"	exon_number "2"
13	lncRNA	exon	75579987	75580198	.	-	.	gene_id "lnc-bbug05435"	transcript_id "lnc-bbut00012887"	exon_number "3"
13	lncRNA	exon	75739784	75742310	.	-	.	gene_id "lnc-bbug05436"	transcript_id "lnc-bbut00012888"	exon_number "1"
13	lncRNA	exon	75748451	75748597	.	-	.	gene_id "lnc-bbug05436"	transcript_id "lnc-bbut00012888"	exon_number "2"
13	lncRNA	exon	75757899	75757965	.	-	.	gene_id "lnc-bbug05436"	transcript_id "lnc-bbut00012888"	exon_number "3"
13	lncRNA	exon	75758553	75759050	.	-	.	gene_id "lnc-bbug05436"	transcript_id "lnc-bbut00012888"	exon_number "4"
13	lncRNA	exon	75937631	75938085	.	-	.	gene_id "lnc-bbug05437"	transcript_id "lnc-bbut00012891"	exon_number "1"
13	lncRNA	exon	75944980	75945227	.	-	.	gene_id "lnc-bbug05437"	transcript_id "lnc-bbut00012891"	exon_number "2"
13	lncRNA	exon	75945343	75945397	.	-	.	gene_id "lnc-bbug05437"	transcript_id "lnc-bbut00012891"	exon_number "3"
13	lncRNA	exon	75955986	75956058	.	-	.	gene_id "lnc-bbug05437"	transcript_id "lnc-bbut00012891"	exon_number "4"
13	lncRNA	exon	75962881	75962998	.	-	.	gene_id "lnc-bbug05437"	transcript_id "lnc-bbut00012891"	exon_number "5"
13	lncRNA	exon	75963222	75963329	.	-	.	gene_id "lnc-bbug05437"	transcript_id "lnc-bbut00012891"	exon_number "6"
13	lncRNA	exon	75937631	75938085	.	-	.	gene_id "lnc-bbug05437"	transcript_id "lnc-bbut00012890"	exon_number "1"
13	lncRNA	exon	75944980	75945227	.	-	.	gene_id "lnc-bbug05437"	transcript_id "lnc-bbut00012890"	exon_number "2"
13	lncRNA	exon	75955986	75956058	.	-	.	gene_id "lnc-bbug05437"	transcript_id "lnc-bbut00012890"	exon_number "3"
13	lncRNA	exon	75962881	75962998	.	-	.	gene_id "lnc-bbug05437"	transcript_id "lnc-bbut00012890"	exon_number "4"
13	lncRNA	exon	75963222	75963329	.	-	.	gene_id "lnc-bbug05437"	transcript_id "lnc-bbut00012890"	exon_number "5"
13	lncRNA	exon	75937631	75938085	.	-	.	gene_id "lnc-bbug05437"	transcript_id "lnc-bbut00012889"	exon_number "1"

13	lncRNA	exon	75955986	75956058	.	-	.	gene_id "lnc-bbug05437"	transcript_id "lnc-bbut00012889"	exon_number "2"
13	lncRNA	exon	75962881	75962998	.	-	.	gene_id "lnc-bbug05437"	transcript_id "lnc-bbut00012889"	exon_number "3"
13	lncRNA	exon	75963222	75963329	.	-	.	gene_id "lnc-bbug05437"	transcript_id "lnc-bbut00012889"	exon_number "4"
13	lncRNA	exon	75949269	75949848	.	-	.	gene_id "lnc-bbug05437"	transcript_id "lnc-bbut00012892"	exon_number "1"
13	lncRNA	exon	75955986	75956058	.	-	.	gene_id "lnc-bbug05437"	transcript_id "lnc-bbut00012892"	exon_number "2"
13	lncRNA	exon	75962881	75962998	.	-	.	gene_id "lnc-bbug05437"	transcript_id "lnc-bbut00012892"	exon_number "3"
13	lncRNA	exon	75963222	75963329	.	-	.	gene_id "lnc-bbug05437"	transcript_id "lnc-bbut00012892"	exon_number "4"
13	lncRNA	exon	75949269	75949848	.	-	.	gene_id "lnc-bbug05437"	transcript_id "lnc-bbut00012893"	exon_number "1"
13	lncRNA	exon	75955986	75956058	.	-	.	gene_id "lnc-bbug05437"	transcript_id "lnc-bbut00012893"	exon_number "2"
13	lncRNA	exon	75962881	75962998	.	-	.	gene_id "lnc-bbug05437"	transcript_id "lnc-bbut00012893"	exon_number "3"
13	lncRNA	exon	75963271	75963364	.	-	.	gene_id "lnc-bbug05437"	transcript_id "lnc-bbut00012893"	exon_number "4"
13	lncRNA	exon	75949269	75949848	.	-	.	gene_id "lnc-bbug05437"	transcript_id "lnc-bbut00012894"	exon_number "1"
13	lncRNA	exon	75955986	75956058	.	-	.	gene_id "lnc-bbug05437"	transcript_id "lnc-bbut00012894"	exon_number "2"
13	lncRNA	exon	75962881	75962998	.	-	.	gene_id "lnc-bbug05437"	transcript_id "lnc-bbut00012894"	exon_number "3"
13	lncRNA	exon	75963424	75963524	.	-	.	gene_id "lnc-bbug05437"	transcript_id "lnc-bbut00012894"	exon_number "4"
13	lncRNA	exon	76073104	76073756	.	-	.	gene_id "lnc-bbug05438"	transcript_id "lnc-bbut00012895"	exon_number "1"
13	lncRNA	exon	76082590	76083406	.	-	.	gene_id "lnc-bbug05438"	transcript_id "lnc-bbut00012895"	exon_number "2"
13	lncRNA	exon	78491180	78491442	.	-	.	gene_id "lnc-bbug05445"	transcript_id "lnc-bbut00012916"	exon_number "1"
13	lncRNA	exon	78498248	78498312	.	-	.	gene_id "lnc-bbug05445"	transcript_id "lnc-bbut00012916"	exon_number "2"
13	lncRNA	exon	79036497	79037202	.	-	.	gene_id "lnc-bbug05449"	transcript_id "lnc-bbut00012929"	exon_number "1"
13	lncRNA	exon	79040138	79043976	.	-	.	gene_id "lnc-bbug05449"	transcript_id "lnc-bbut00012929"	exon_number "2"
13	lncRNA	exon	83024574	83024676	.	-	.	gene_id "lnc-bbug05456"	transcript_id "lnc-bbut00012939"	exon_number "1"
13	lncRNA	exon	83026588	83026713	.	-	.	gene_id "lnc-bbug05456"	transcript_id "lnc-bbut00012939"	exon_number "2"
13	lncRNA	exon	83027209	83027460	.	-	.	gene_id "lnc-bbug05456"	transcript_id "lnc-bbut00012939"	exon_number "3"
13	lncRNA	exon	83027740	83028446	.	-	.	gene_id "lnc-bbug05456"	transcript_id "lnc-bbut00012939"	exon_number "4"
13	lncRNA	exon	83037414	83037484	.	-	.	gene_id "lnc-bbug05456"	transcript_id "lnc-bbut00012939"	exon_number "5"
13	lncRNA	exon	86501173	86501844	.	-	.	gene_id "lnc-bbug05457"	transcript_id "lnc-bbut00012942"	exon_number "1"
13	lncRNA	exon	86525761	86525883	.	-	.	gene_id "lnc-bbug05457"	transcript_id "lnc-bbut00012942"	exon_number "2"
13	lncRNA	exon	86526785	86526835	.	-	.	gene_id "lnc-bbug05457"	transcript_id "lnc-bbut00012942"	exon_number "3"
13	lncRNA	exon	86527712	86527907	.	-	.	gene_id "lnc-bbug05457"	transcript_id "lnc-bbut00012942"	exon_number "4"
13	lncRNA	exon	86533546	86533686	.	-	.	gene_id "lnc-bbug05457"	transcript_id "lnc-bbut00012942"	exon_number "5"
13	lncRNA	exon	86538220	86538381	.	-	.	gene_id "lnc-bbug05457"	transcript_id "lnc-bbut00012942"	exon_number "6"
13	lncRNA	exon	86539140	86539280	.	-	.	gene_id "lnc-bbug05457"	transcript_id "lnc-bbut00012942"	exon_number "7"
13	lncRNA	exon	86501173	86501844	.	-	.	gene_id "lnc-bbug05457"	transcript_id "lnc-bbut00012941"	exon_number "1"
13	lncRNA	exon	86525761	86525883	.	-	.	gene_id "lnc-bbug05457"	transcript_id "lnc-bbut00012941"	exon_number "2"
13	lncRNA	exon	86526785	86526835	.	-	.	gene_id "lnc-bbug05457"	transcript_id "lnc-bbut00012941"	exon_number "3"
13	lncRNA	exon	86527712	86527907	.	-	.	gene_id "lnc-bbug05457"	transcript_id "lnc-bbut00012941"	exon_number "4"
13	lncRNA	exon	86533546	86533686	.	-	.	gene_id "lnc-bbug05457"	transcript_id "lnc-bbut00012941"	exon_number "5"
13	lncRNA	exon	86537865	86537992	.	-	.	gene_id "lnc-bbug05457"	transcript_id "lnc-bbut00012941"	exon_number "6"
13	lncRNA	exon	86539140	86539280	.	-	.	gene_id "lnc-bbug05457"	transcript_id "lnc-bbut00012941"	exon_number "7"
13	lncRNA	exon	86501173	86501844	.	-	.	gene_id "lnc-bbug05457"	transcript_id "lnc-bbut00012940"	exon_number "1"
13	lncRNA	exon	86525761	86525883	.	-	.	gene_id "lnc-bbug05457"	transcript_id "lnc-bbut00012940"	exon_number "2"
13	lncRNA	exon	86526785	86526835	.	-	.	gene_id "lnc-bbug05457"	transcript_id "lnc-bbut00012940"	exon_number "3"
13	lncRNA	exon	86527712	86527907	.	-	.	gene_id "lnc-bbug05457"	transcript_id "lnc-bbut00012940"	exon_number "4"
13	lncRNA	exon	86533546	86533686	.	-	.	gene_id "lnc-bbug05457"	transcript_id "lnc-bbut00012940"	exon_number "5"
13	lncRNA	exon	86539140	86539280	.	-	.	gene_id "lnc-bbug05457"	transcript_id "lnc-bbut00012940"	exon_number "6"
13	lncRNA	exon	88410440	88411586	.	-	.	gene_id "lnc-bbug05459"	transcript_id "lnc-bbut00012950"	exon_number "1"
13	lncRNA	exon	89956633	89957605	.	-	.	gene_id "lnc-bbug05461"	transcript_id "lnc-bbut00012952"	exon_number "1"
13	lncRNA	exon	89995307	89995312	.	-	.	gene_id "lnc-bbug05461"	transcript_id "lnc-bbut00012952"	exon_number "2"
14	lncRNA	exon	574807	574901	.	+	.	gene_id "lnc-bbug05464"	transcript_id "lnc-bbut00012955"	exon_number "1"
14	lncRNA	exon	580072	580135	.	+	.	gene_id "lnc-bbug05464"	transcript_id "lnc-bbut00012955"	exon_number "2"
14	lncRNA	exon	580344	580394	.	+	.	gene_id "lnc-bbug05464"	transcript_id "lnc-bbut00012955"	exon_number "3"

14	lncRNA	exon	2903922	2907502	.	+	.	gene_id "lnc-bbug05470"	transcript_id "lnc-bbut00012969"	exon_number "1"
14	lncRNA	exon	2914728	2914870	.	+	.	gene_id "lnc-bbug05470"	transcript_id "lnc-bbut00012969"	exon_number "2"
14	lncRNA	exon	2915644	2915855	.	+	.	gene_id "lnc-bbug05470"	transcript_id "lnc-bbut00012969"	exon_number "3"
14	lncRNA	exon	3195084	3195399	.	+	.	gene_id "lnc-bbug05471"	transcript_id "lnc-bbut00012970"	exon_number "1"
14	lncRNA	exon	3198990	3199039	.	+	.	gene_id "lnc-bbug05471"	transcript_id "lnc-bbut00012970"	exon_number "2"
14	lncRNA	exon	3203365	3203874	.	+	.	gene_id "lnc-bbug05471"	transcript_id "lnc-bbut00012970"	exon_number "3"
14	lncRNA	exon	3533010	3533191	.	+	.	gene_id "lnc-bbug05472"	transcript_id "lnc-bbut00012971"	exon_number "1"
14	lncRNA	exon	3553618	3553727	.	+	.	gene_id "lnc-bbug05472"	transcript_id "lnc-bbut00012971"	exon_number "2"
14	lncRNA	exon	3558435	3558553	.	+	.	gene_id "lnc-bbug05472"	transcript_id "lnc-bbut00012971"	exon_number "3"
14	lncRNA	exon	3559021	3561056	.	+	.	gene_id "lnc-bbug05472"	transcript_id "lnc-bbut00012971"	exon_number "4"
14	lncRNA	exon	3533012	3533191	.	+	.	gene_id "lnc-bbug05472"	transcript_id "lnc-bbut00012972"	exon_number "1"
14	lncRNA	exon	3553618	3553727	.	+	.	gene_id "lnc-bbug05472"	transcript_id "lnc-bbut00012972"	exon_number "2"
14	lncRNA	exon	3558435	3561056	.	+	.	gene_id "lnc-bbug05472"	transcript_id "lnc-bbut00012972"	exon_number "3"
14	lncRNA	exon	3584737	3585482	.	+	.	gene_id "lnc-bbug05473"	transcript_id "lnc-bbut00012973"	exon_number "1"
14	lncRNA	exon	3586016	3587904	.	+	.	gene_id "lnc-bbug05473"	transcript_id "lnc-bbut00012973"	exon_number "2"
14	lncRNA	exon	4721995	4722395	.	+	.	gene_id "lnc-bbug05480"	transcript_id "lnc-bbut00012992"	exon_number "1"
14	lncRNA	exon	4723310	4723584	.	+	.	gene_id "lnc-bbug05480"	transcript_id "lnc-bbut00012992"	exon_number "2"
14	lncRNA	exon	4724649	4724906	.	+	.	gene_id "lnc-bbug05480"	transcript_id "lnc-bbut00012992"	exon_number "3"
14	lncRNA	exon	4726323	4726495	.	+	.	gene_id "lnc-bbug05480"	transcript_id "lnc-bbut00012992"	exon_number "4"
14	lncRNA	exon	4961092	4962026	.	+	.	gene_id "lnc-bbug05482"	transcript_id "lnc-bbut00013000"	exon_number "1"
14	lncRNA	exon	4968533	4970441	.	+	.	gene_id "lnc-bbug05482"	transcript_id "lnc-bbut00013000"	exon_number "2"
14	lncRNA	exon	5013095	5013309	.	+	.	gene_id "lnc-bbug05483"	transcript_id "lnc-bbut00013002"	exon_number "1"
14	lncRNA	exon	5013508	5014960	.	+	.	gene_id "lnc-bbug05483"	transcript_id "lnc-bbut00013002"	exon_number "2"
14	lncRNA	exon	5015740	5015949	.	+	.	gene_id "lnc-bbug05483"	transcript_id "lnc-bbut00013002"	exon_number "3"
14	lncRNA	exon	5013095	5013309	.	+	.	gene_id "lnc-bbug05483"	transcript_id "lnc-bbut00013001"	exon_number "1"
14	lncRNA	exon	5013508	5013687	.	+	.	gene_id "lnc-bbug05483"	transcript_id "lnc-bbut00013001"	exon_number "2"
14	lncRNA	exon	5015740	5015949	.	+	.	gene_id "lnc-bbug05483"	transcript_id "lnc-bbut00013001"	exon_number "3"
14	lncRNA	exon	5024380	5024740	.	+	.	gene_id "lnc-bbug05484"	transcript_id "lnc-bbut00013003"	exon_number "1"
14	lncRNA	exon	5025173	5025253	.	+	.	gene_id "lnc-bbug05484"	transcript_id "lnc-bbut00013003"	exon_number "2"
14	lncRNA	exon	5044273	5044467	.	+	.	gene_id "lnc-bbug05484"	transcript_id "lnc-bbut00013003"	exon_number "3"
14	lncRNA	exon	5057183	5057409	.	+	.	gene_id "lnc-bbug05484"	transcript_id "lnc-bbut00013003"	exon_number "4"
14	lncRNA	exon	5083604	5084694	.	+	.	gene_id "lnc-bbug05485"	transcript_id "lnc-bbut00013004"	exon_number "1"
14	lncRNA	exon	5087802	5087893	.	+	.	gene_id "lnc-bbug05485"	transcript_id "lnc-bbut00013004"	exon_number "2"
14	lncRNA	exon	5088011	5090861	.	+	.	gene_id "lnc-bbug05485"	transcript_id "lnc-bbut00013004"	exon_number "3"
14	lncRNA	exon	5087061	5087194	.	+	.	gene_id "lnc-bbug05485"	transcript_id "lnc-bbut00013005"	exon_number "1"
14	lncRNA	exon	5087802	5087893	.	+	.	gene_id "lnc-bbug05485"	transcript_id "lnc-bbut00013005"	exon_number "2"
14	lncRNA	exon	5088011	5090861	.	+	.	gene_id "lnc-bbug05485"	transcript_id "lnc-bbut00013005"	exon_number "3"
14	lncRNA	exon	5087094	5087151	.	+	.	gene_id "lnc-bbug05485"	transcript_id "lnc-bbut00013006"	exon_number "1"
14	lncRNA	exon	5087802	5087893	.	+	.	gene_id "lnc-bbug05485"	transcript_id "lnc-bbut00013006"	exon_number "2"
14	lncRNA	exon	5088011	5090861	.	+	.	gene_id "lnc-bbug05485"	transcript_id "lnc-bbut00013006"	exon_number "3"
14	lncRNA	exon	5190190	5191074	.	+	.	gene_id "lnc-bbug05486"	transcript_id "lnc-bbut00013007"	exon_number "1"
14	lncRNA	exon	5194741	5196778	.	+	.	gene_id "lnc-bbug05486"	transcript_id "lnc-bbut00013007"	exon_number "2"
14	lncRNA	exon	5227432	5244267	.	+	.	gene_id "lnc-bbug05487"	transcript_id "lnc-bbut00013008"	exon_number "1"
14	lncRNA	exon	5245278	5246349	.	+	.	gene_id "lnc-bbug05487"	transcript_id "lnc-bbut00013008"	exon_number "2"
14	lncRNA	exon	5356414	5357885	.	+	.	gene_id "lnc-bbug05489"	transcript_id "lnc-bbut00013010"	exon_number "1"
14	lncRNA	exon	5363502	5363727	.	+	.	gene_id "lnc-bbug05489"	transcript_id "lnc-bbut00013010"	exon_number "2"
14	lncRNA	exon	5357780	5357885	.	+	.	gene_id "lnc-bbug05489"	transcript_id "lnc-bbut00013011"	exon_number "1"
14	lncRNA	exon	5375429	5375577	.	+	.	gene_id "lnc-bbug05489"	transcript_id "lnc-bbut00013011"	exon_number "2"
14	lncRNA	exon	5384478	5386247	.	+	.	gene_id "lnc-bbug05489"	transcript_id "lnc-bbut00013011"	exon_number "3"
14	lncRNA	exon	5375746	5375764	.	+	.	gene_id "lnc-bbug05489"	transcript_id "lnc-bbut00013013"	exon_number "1"
14	lncRNA	exon	5382748	5382873	.	+	.	gene_id "lnc-bbug05489"	transcript_id "lnc-bbut00013013"	exon_number "2"
14	lncRNA	exon	5384478	5386247	.	+	.	gene_id "lnc-bbug05489"	transcript_id "lnc-bbut00013013"	exon_number "3"

14	lncRNA	exon	5409287	5413338	.	+	.	gene_id "lnc-bbug05490"	transcript_id "lnc-bbut00013014"	exon_number "1"
14	lncRNA	exon	5414434	5414711	.	+	.	gene_id "lnc-bbug05490"	transcript_id "lnc-bbut00013014"	exon_number "2"
14	lncRNA	exon	5618676	5619084	.	+	.	gene_id "lnc-bbug05493"	transcript_id "lnc-bbut00013020"	exon_number "1"
14	lncRNA	exon	5644956	5645133	.	+	.	gene_id "lnc-bbug05493"	transcript_id "lnc-bbut00013020"	exon_number "2"
14	lncRNA	exon	5645257	5646039	.	+	.	gene_id "lnc-bbug05493"	transcript_id "lnc-bbut00013020"	exon_number "3"
14	lncRNA	exon	5618681	5618980	.	+	.	gene_id "lnc-bbug05493"	transcript_id "lnc-bbut00013021"	exon_number "1"
14	lncRNA	exon	5644956	5645133	.	+	.	gene_id "lnc-bbug05493"	transcript_id "lnc-bbut00013021"	exon_number "2"
14	lncRNA	exon	5645257	5646039	.	+	.	gene_id "lnc-bbug05493"	transcript_id "lnc-bbut00013021"	exon_number "3"
14	lncRNA	exon	5785574	5786553	.	+	.	gene_id "lnc-bbug05495"	transcript_id "lnc-bbut00013026"	exon_number "1"
14	lncRNA	exon	5791321	5792683	.	+	.	gene_id "lnc-bbug05495"	transcript_id "lnc-bbut00013026"	exon_number "2"
14	lncRNA	exon	6196575	6196949	.	+	.	gene_id "lnc-bbug05501"	transcript_id "lnc-bbut00013035"	exon_number "1"
14	lncRNA	exon	7524494	7524803	.	+	.	gene_id "lnc-bbug05506"	transcript_id "lnc-bbut00013058"	exon_number "1"
14	lncRNA	exon	7524894	7524989	.	+	.	gene_id "lnc-bbug05506"	transcript_id "lnc-bbut00013058"	exon_number "2"
14	lncRNA	exon	7530992	7531038	.	+	.	gene_id "lnc-bbug05506"	transcript_id "lnc-bbut00013058"	exon_number "3"
14	lncRNA	exon	7748699	7752149	.	+	.	gene_id "lnc-bbug05509"	transcript_id "lnc-bbut00013077"	exon_number "1"
14	lncRNA	exon	7752820	7753002	.	+	.	gene_id "lnc-bbug05509"	transcript_id "lnc-bbut00013077"	exon_number "2"
14	lncRNA	exon	8296586	8296855	.	+	.	gene_id "lnc-bbug05516"	transcript_id "lnc-bbut00013084"	exon_number "1"
14	lncRNA	exon	8299487	8299537	.	+	.	gene_id "lnc-bbug05516"	transcript_id "lnc-bbut00013084"	exon_number "2"
14	lncRNA	exon	8302181	8303084	.	+	.	gene_id "lnc-bbug05516"	transcript_id "lnc-bbut00013084"	exon_number "3"
14	lncRNA	exon	8724709	8724818	.	+	.	gene_id "lnc-bbug05522"	transcript_id "lnc-bbut00013096"	exon_number "1"
14	lncRNA	exon	8725129	8725295	.	+	.	gene_id "lnc-bbug05522"	transcript_id "lnc-bbut00013096"	exon_number "2"
14	lncRNA	exon	8726734	8727085	.	+	.	gene_id "lnc-bbug05522"	transcript_id "lnc-bbut00013096"	exon_number "3"
14	lncRNA	exon	9040022	9040289	.	+	.	gene_id "lnc-bbug05530"	transcript_id "lnc-bbut00013118"	exon_number "1"
14	lncRNA	exon	9041227	9041540	.	+	.	gene_id "lnc-bbug05530"	transcript_id "lnc-bbut00013118"	exon_number "2"
14	lncRNA	exon	9054538	9054841	.	+	.	gene_id "lnc-bbug05531"	transcript_id "lnc-bbut00013119"	exon_number "1"
14	lncRNA	exon	9058666	9058774	.	+	.	gene_id "lnc-bbug05531"	transcript_id "lnc-bbut00013119"	exon_number "2"
14	lncRNA	exon	9059196	9059381	.	+	.	gene_id "lnc-bbug05531"	transcript_id "lnc-bbut00013119"	exon_number "3"
14	lncRNA	exon	9270479	9270501	.	+	.	gene_id "lnc-bbug05534"	transcript_id "lnc-bbut00013123"	exon_number "1"
14	lncRNA	exon	9270987	9271125	.	+	.	gene_id "lnc-bbug05534"	transcript_id "lnc-bbut00013123"	exon_number "2"
14	lncRNA	exon	9274873	9275064	.	+	.	gene_id "lnc-bbug05534"	transcript_id "lnc-bbut00013123"	exon_number "3"
14	lncRNA	exon	9275514	9276090	.	+	.	gene_id "lnc-bbug05534"	transcript_id "lnc-bbut00013123"	exon_number "4"
14	lncRNA	exon	9291269	9291283	.	+	.	gene_id "lnc-bbug05535"	transcript_id "lnc-bbut00013124"	exon_number "1"
14	lncRNA	exon	9291463	9291833	.	+	.	gene_id "lnc-bbug05535"	transcript_id "lnc-bbut00013124"	exon_number "2"
14	lncRNA	exon	9293531	9293572	.	+	.	gene_id "lnc-bbug05535"	transcript_id "lnc-bbut00013124"	exon_number "3"
14	lncRNA	exon	9296176	9296371	.	+	.	gene_id "lnc-bbug05535"	transcript_id "lnc-bbut00013124"	exon_number "4"
14	lncRNA	exon	9296817	9297068	.	+	.	gene_id "lnc-bbug05535"	transcript_id "lnc-bbut00013124"	exon_number "5"
14	lncRNA	exon	9291631	9291833	.	+	.	gene_id "lnc-bbug05535"	transcript_id "lnc-bbut00013125"	exon_number "1"
14	lncRNA	exon	9293531	9293572	.	+	.	gene_id "lnc-bbug05535"	transcript_id "lnc-bbut00013125"	exon_number "2"
14	lncRNA	exon	9296179	9296371	.	+	.	gene_id "lnc-bbug05535"	transcript_id "lnc-bbut00013125"	exon_number "3"
14	lncRNA	exon	9296817	9297068	.	+	.	gene_id "lnc-bbug05535"	transcript_id "lnc-bbut00013125"	exon_number "4"
14	lncRNA	exon	9995542	9995674	.	+	.	gene_id "lnc-bbug05545"	transcript_id "lnc-bbut00013141"	exon_number "1"
14	lncRNA	exon	9996318	9996476	.	+	.	gene_id "lnc-bbug05545"	transcript_id "lnc-bbut00013141"	exon_number "2"
14	lncRNA	exon	9996949	9997098	.	+	.	gene_id "lnc-bbug05545"	transcript_id "lnc-bbut00013141"	exon_number "3"
14	lncRNA	exon	9997680	9997868	.	+	.	gene_id "lnc-bbug05545"	transcript_id "lnc-bbut00013141"	exon_number "4"
14	lncRNA	exon	10165904	10166862	.	+	.	gene_id "lnc-bbug05546"	transcript_id "lnc-bbut00013142"	exon_number "1"
14	lncRNA	exon	10168734	10168874	.	+	.	gene_id "lnc-bbug05546"	transcript_id "lnc-bbut00013142"	exon_number "2"
14	lncRNA	exon	10168968	10169108	.	+	.	gene_id "lnc-bbug05546"	transcript_id "lnc-bbut00013142"	exon_number "3"
14	lncRNA	exon	10169482	10169652	.	+	.	gene_id "lnc-bbug05546"	transcript_id "lnc-bbut00013142"	exon_number "4"
14	lncRNA	exon	11258555	11259580	.	+	.	gene_id "lnc-bbug05556"	transcript_id "lnc-bbut00013160"	exon_number "1"
14	lncRNA	exon	11377097	11377931	.	+	.	gene_id "lnc-bbug05557"	transcript_id "lnc-bbut00013161"	exon_number "1"
14	lncRNA	exon	11699219	11699373	.	+	.	gene_id "lnc-bbug05558"	transcript_id "lnc-bbut00013163"	exon_number "1"
14	lncRNA	exon	11701947	11702077	.	+	.	gene_id "lnc-bbug05558"	transcript_id "lnc-bbut00013163"	exon_number "2"

14	lncRNA	exon	11706387	11706461	.	+	.	gene_id "lnc-bbug05558"	transcript_id "lnc-bbut00013163"	exon_number "3"
14	lncRNA	exon	11706546	11707835	.	+	.	gene_id "lnc-bbug05558"	transcript_id "lnc-bbut00013163"	exon_number "4"
14	lncRNA	exon	11699219	11699373	.	+	.	gene_id "lnc-bbug05558"	transcript_id "lnc-bbut00013162"	exon_number "1"
14	lncRNA	exon	11701947	11702077	.	+	.	gene_id "lnc-bbug05558"	transcript_id "lnc-bbut00013162"	exon_number "2"
14	lncRNA	exon	11706387	11706461	.	+	.	gene_id "lnc-bbug05558"	transcript_id "lnc-bbut00013162"	exon_number "3"
14	lncRNA	exon	11706533	11707835	.	+	.	gene_id "lnc-bbug05558"	transcript_id "lnc-bbut00013162"	exon_number "4"
14	lncRNA	exon	11699219	11699373	.	+	.	gene_id "lnc-bbug05558"	transcript_id "lnc-bbut00013164"	exon_number "1"
14	lncRNA	exon	11701947	11702077	.	+	.	gene_id "lnc-bbug05558"	transcript_id "lnc-bbut00013164"	exon_number "2"
14	lncRNA	exon	11706387	11706461	.	+	.	gene_id "lnc-bbug05558"	transcript_id "lnc-bbut00013164"	exon_number "3"
14	lncRNA	exon	11706546	11706671	.	+	.	gene_id "lnc-bbug05558"	transcript_id "lnc-bbut00013164"	exon_number "4"
14	lncRNA	exon	11741800	11744374	.	+	.	gene_id "lnc-bbug05558"	transcript_id "lnc-bbut00013164"	exon_number "5"
14	lncRNA	exon	11699219	11699373	.	+	.	gene_id "lnc-bbug05558"	transcript_id "lnc-bbut00013165"	exon_number "1"
14	lncRNA	exon	11701947	11702077	.	+	.	gene_id "lnc-bbug05558"	transcript_id "lnc-bbut00013165"	exon_number "2"
14	lncRNA	exon	11706387	11706461	.	+	.	gene_id "lnc-bbug05558"	transcript_id "lnc-bbut00013165"	exon_number "3"
14	lncRNA	exon	11706546	11706671	.	+	.	gene_id "lnc-bbug05558"	transcript_id "lnc-bbut00013165"	exon_number "4"
14	lncRNA	exon	11760979	11761792	.	+	.	gene_id "lnc-bbug05558"	transcript_id "lnc-bbut00013165"	exon_number "5"
14	lncRNA	exon	13000230	13000361	.	+	.	gene_id "lnc-bbug05559"	transcript_id "lnc-bbut00013166"	exon_number "1"
14	lncRNA	exon	13000463	13000612	.	+	.	gene_id "lnc-bbug05559"	transcript_id "lnc-bbut00013166"	exon_number "2"
14	lncRNA	exon	13830036	13830215	.	+	.	gene_id "lnc-bbug05563"	transcript_id "lnc-bbut00013180"	exon_number "1"
14	lncRNA	exon	13831307	13831446	.	+	.	gene_id "lnc-bbug05563"	transcript_id "lnc-bbut00013180"	exon_number "2"
14	lncRNA	exon	13833887	13833981	.	+	.	gene_id "lnc-bbug05563"	transcript_id "lnc-bbut00013180"	exon_number "3"
14	lncRNA	exon	13838675	13838770	.	+	.	gene_id "lnc-bbug05563"	transcript_id "lnc-bbut00013180"	exon_number "4"
14	lncRNA	exon	13850728	13851146	.	+	.	gene_id "lnc-bbug05563"	transcript_id "lnc-bbut00013180"	exon_number "5"
14	lncRNA	exon	14368805	14368901	.	+	.	gene_id "lnc-bbug05565"	transcript_id "lnc-bbut00013183"	exon_number "1"
14	lncRNA	exon	14371181	14371324	.	+	.	gene_id "lnc-bbug05565"	transcript_id "lnc-bbut00013183"	exon_number "2"
14	lncRNA	exon	14383165	14383803	.	+	.	gene_id "lnc-bbug05565"	transcript_id "lnc-bbut00013183"	exon_number "3"
14	lncRNA	exon	16245750	16245930	.	+	.	gene_id "lnc-bbug05567"	transcript_id "lnc-bbut00013185"	exon_number "1"
14	lncRNA	exon	16248840	16248955	.	+	.	gene_id "lnc-bbug05567"	transcript_id "lnc-bbut00013185"	exon_number "2"
14	lncRNA	exon	16250664	16250756	.	+	.	gene_id "lnc-bbug05567"	transcript_id "lnc-bbut00013185"	exon_number "3"
14	lncRNA	exon	16263375	16263612	.	+	.	gene_id "lnc-bbug05567"	transcript_id "lnc-bbut00013185"	exon_number "4"
14	lncRNA	exon	16245753	16245930	.	+	.	gene_id "lnc-bbug05567"	transcript_id "lnc-bbut00013186"	exon_number "1"
14	lncRNA	exon	16248840	16248955	.	+	.	gene_id "lnc-bbug05567"	transcript_id "lnc-bbut00013186"	exon_number "2"
14	lncRNA	exon	16250664	16250756	.	+	.	gene_id "lnc-bbug05567"	transcript_id "lnc-bbut00013186"	exon_number "3"
14	lncRNA	exon	16251834	16251883	.	+	.	gene_id "lnc-bbug05567"	transcript_id "lnc-bbut00013186"	exon_number "4"
14	lncRNA	exon	16245758	16245930	.	+	.	gene_id "lnc-bbug05567"	transcript_id "lnc-bbut00013187"	exon_number "1"
14	lncRNA	exon	16248840	16248955	.	+	.	gene_id "lnc-bbug05567"	transcript_id "lnc-bbut00013187"	exon_number "2"
14	lncRNA	exon	16250664	16250756	.	+	.	gene_id "lnc-bbug05567"	transcript_id "lnc-bbut00013187"	exon_number "3"
14	lncRNA	exon	16251070	16251121	.	+	.	gene_id "lnc-bbug05567"	transcript_id "lnc-bbut00013187"	exon_number "4"
14	lncRNA	exon	16245774	16245930	.	+	.	gene_id "lnc-bbug05567"	transcript_id "lnc-bbut00013188"	exon_number "1"
14	lncRNA	exon	16248840	16248955	.	+	.	gene_id "lnc-bbug05567"	transcript_id "lnc-bbut00013188"	exon_number "2"
14	lncRNA	exon	16250664	16251034	.	+	.	gene_id "lnc-bbug05567"	transcript_id "lnc-bbut00013188"	exon_number "3"
14	lncRNA	exon	16314106	16314291	.	+	.	gene_id "lnc-bbug05569"	transcript_id "lnc-bbut00013190"	exon_number "1"
14	lncRNA	exon	16325747	16325843	.	+	.	gene_id "lnc-bbug05569"	transcript_id "lnc-bbut00013190"	exon_number "2"
14	lncRNA	exon	16328901	16329046	.	+	.	gene_id "lnc-bbug05569"	transcript_id "lnc-bbut00013190"	exon_number "3"
14	lncRNA	exon	16330041	16331907	.	+	.	gene_id "lnc-bbug05569"	transcript_id "lnc-bbut00013190"	exon_number "4"
14	lncRNA	exon	16324360	16325843	.	+	.	gene_id "lnc-bbug05569"	transcript_id "lnc-bbut00013191"	exon_number "1"
14	lncRNA	exon	16328901	16329046	.	+	.	gene_id "lnc-bbug05569"	transcript_id "lnc-bbut00013191"	exon_number "2"
14	lncRNA	exon	16330041	16331907	.	+	.	gene_id "lnc-bbug05569"	transcript_id "lnc-bbut00013191"	exon_number "3"
14	lncRNA	exon	16378223	16378804	.	+	.	gene_id "lnc-bbug05570"	transcript_id "lnc-bbut00013192"	exon_number "1"
14	lncRNA	exon	16380374	16380711	.	+	.	gene_id "lnc-bbug05570"	transcript_id "lnc-bbut00013192"	exon_number "2"
14	lncRNA	exon	16385726	16385884	.	+	.	gene_id "lnc-bbug05570"	transcript_id "lnc-bbut00013192"	exon_number "3"
14	lncRNA	exon	17134071	17134359	.	+	.	gene_id "lnc-bbug05574"	transcript_id "lnc-bbut00013198"	exon_number "1"

14	lncRNA	exon	17136576	17138758	.	+	.	gene_id "lnc-bbug05574"	transcript_id "lnc-bbut00013198"	exon_number "2"
14	lncRNA	exon	17147231	17149158	.	+	.	gene_id "lnc-bbug05574"	transcript_id "lnc-bbut00013198"	exon_number "3"
14	lncRNA	exon	17134071	17134359	.	+	.	gene_id "lnc-bbug05574"	transcript_id "lnc-bbut00013197"	exon_number "1"
14	lncRNA	exon	17136026	17138758	.	+	.	gene_id "lnc-bbug05574"	transcript_id "lnc-bbut00013197"	exon_number "2"
14	lncRNA	exon	17147231	17149158	.	+	.	gene_id "lnc-bbug05574"	transcript_id "lnc-bbut00013197"	exon_number "3"
14	lncRNA	exon	17134073	17134252	.	+	.	gene_id "lnc-bbug05574"	transcript_id "lnc-bbut00013199"	exon_number "1"
14	lncRNA	exon	17136026	17136183	.	+	.	gene_id "lnc-bbug05574"	transcript_id "lnc-bbut00013199"	exon_number "2"
14	lncRNA	exon	17136576	17138758	.	+	.	gene_id "lnc-bbug05574"	transcript_id "lnc-bbut00013199"	exon_number "3"
14	lncRNA	exon	17147231	17149158	.	+	.	gene_id "lnc-bbug05574"	transcript_id "lnc-bbut00013199"	exon_number "4"
14	lncRNA	exon	17156773	17167248	.	+	.	gene_id "lnc-bbug05575"	transcript_id "lnc-bbut00013200"	exon_number "1"
14	lncRNA	exon	17169304	17169469	.	+	.	gene_id "lnc-bbug05575"	transcript_id "lnc-bbut00013200"	exon_number "2"
14	lncRNA	exon	17314250	17314374	.	+	.	gene_id "lnc-bbug05576"	transcript_id "lnc-bbut00013201"	exon_number "1"
14	lncRNA	exon	17315898	17316008	.	+	.	gene_id "lnc-bbug05576"	transcript_id "lnc-bbut00013201"	exon_number "2"
14	lncRNA	exon	17317129	17318148	.	+	.	gene_id "lnc-bbug05576"	transcript_id "lnc-bbut00013201"	exon_number "3"
14	lncRNA	exon	17314506	17314618	.	+	.	gene_id "lnc-bbug05576"	transcript_id "lnc-bbut00013202"	exon_number "1"
14	lncRNA	exon	17315898	17316008	.	+	.	gene_id "lnc-bbug05576"	transcript_id "lnc-bbut00013202"	exon_number "2"
14	lncRNA	exon	17317129	17318148	.	+	.	gene_id "lnc-bbug05576"	transcript_id "lnc-bbut00013202"	exon_number "3"
14	lncRNA	exon	17398580	17399003	.	+	.	gene_id "lnc-bbug05578"	transcript_id "lnc-bbut00013204"	exon_number "1"
14	lncRNA	exon	17399271	17399375	.	+	.	gene_id "lnc-bbug05578"	transcript_id "lnc-bbut00013204"	exon_number "2"
14	lncRNA	exon	17401278	17403027	.	+	.	gene_id "lnc-bbug05578"	transcript_id "lnc-bbut00013204"	exon_number "3"
14	lncRNA	exon	17417806	17417914	.	+	.	gene_id "lnc-bbug05579"	transcript_id "lnc-bbut00013205"	exon_number "1"
14	lncRNA	exon	17419229	17419412	.	+	.	gene_id "lnc-bbug05579"	transcript_id "lnc-bbut00013205"	exon_number "2"
14	lncRNA	exon	17420047	17420194	.	+	.	gene_id "lnc-bbug05579"	transcript_id "lnc-bbut00013205"	exon_number "3"
14	lncRNA	exon	17932681	17932987	.	+	.	gene_id "lnc-bbug05588"	transcript_id "lnc-bbut00013238"	exon_number "1"
14	lncRNA	exon	17933110	17933200	.	+	.	gene_id "lnc-bbug05588"	transcript_id "lnc-bbut00013238"	exon_number "2"
14	lncRNA	exon	17973430	17973553	.	+	.	gene_id "lnc-bbug05588"	transcript_id "lnc-bbut00013238"	exon_number "3"
14	lncRNA	exon	17974564	17975693	.	+	.	gene_id "lnc-bbug05588"	transcript_id "lnc-bbut00013238"	exon_number "4"
14	lncRNA	exon	17959691	17959881	.	+	.	gene_id "lnc-bbug05588"	transcript_id "lnc-bbut00013239"	exon_number "1"
14	lncRNA	exon	17973430	17973553	.	+	.	gene_id "lnc-bbug05588"	transcript_id "lnc-bbut00013239"	exon_number "2"
14	lncRNA	exon	17974564	17975693	.	+	.	gene_id "lnc-bbug05588"	transcript_id "lnc-bbut00013239"	exon_number "3"
14	lncRNA	exon	18424160	18424749	.	+	.	gene_id "lnc-bbug05589"	transcript_id "lnc-bbut00013240"	exon_number "1"
14	lncRNA	exon	18425160	18425222	.	+	.	gene_id "lnc-bbug05589"	transcript_id "lnc-bbut00013240"	exon_number "2"
14	lncRNA	exon	18887043	18887226	.	+	.	gene_id "lnc-bbug05597"	transcript_id "lnc-bbut00013272"	exon_number "1"
14	lncRNA	exon	18892747	18893060	.	+	.	gene_id "lnc-bbug05597"	transcript_id "lnc-bbut00013272"	exon_number "2"
14	lncRNA	exon	19067448	19067954	.	+	.	gene_id "lnc-bbug05602"	transcript_id "lnc-bbut00013278"	exon_number "1"
14	lncRNA	exon	19068039	19069599	.	+	.	gene_id "lnc-bbug05602"	transcript_id "lnc-bbut00013278"	exon_number "2"
14	lncRNA	exon	19580884	19581595	.	+	.	gene_id "lnc-bbug05609"	transcript_id "lnc-bbut00013308"	exon_number "1"
14	lncRNA	exon	19709540	19709767	.	+	.	gene_id "lnc-bbug05611"	transcript_id "lnc-bbut00013312"	exon_number "1"
14	lncRNA	exon	19710217	19710478	.	+	.	gene_id "lnc-bbug05611"	transcript_id "lnc-bbut00013312"	exon_number "2"
14	lncRNA	exon	21130078	21130252	.	+	.	gene_id "lnc-bbug05622"	transcript_id "lnc-bbut00013324"	exon_number "1"
14	lncRNA	exon	21130558	21130612	.	+	.	gene_id "lnc-bbug05622"	transcript_id "lnc-bbut00013324"	exon_number "2"
14	lncRNA	exon	21131979	21132675	.	+	.	gene_id "lnc-bbug05622"	transcript_id "lnc-bbut00013324"	exon_number "3"
14	lncRNA	exon	21204423	21205760	.	+	.	gene_id "lnc-bbug05623"	transcript_id "lnc-bbut00013325"	exon_number "1"
14	lncRNA	exon	21206942	21207077	.	+	.	gene_id "lnc-bbug05623"	transcript_id "lnc-bbut00013325"	exon_number "2"
14	lncRNA	exon	21208858	21209010	.	+	.	gene_id "lnc-bbug05623"	transcript_id "lnc-bbut00013325"	exon_number "3"
14	lncRNA	exon	21713226	21713758	.	+	.	gene_id "lnc-bbug05628"	transcript_id "lnc-bbut00013332"	exon_number "1"
14	lncRNA	exon	21955293	21957030	.	+	.	gene_id "lnc-bbug05630"	transcript_id "lnc-bbut00013337"	exon_number "1"
14	lncRNA	exon	21957169	21959101	.	+	.	gene_id "lnc-bbug05630"	transcript_id "lnc-bbut00013337"	exon_number "2"
14	lncRNA	exon	22014642	22016443	.	+	.	gene_id "lnc-bbug05631"	transcript_id "lnc-bbut00013338"	exon_number "1"
14	lncRNA	exon	22016550	22016732	.	+	.	gene_id "lnc-bbug05631"	transcript_id "lnc-bbut00013338"	exon_number "2"
14	lncRNA	exon	22121965	22122476	.	+	.	gene_id "lnc-bbug05633"	transcript_id "lnc-bbut00013341"	exon_number "1"
14	lncRNA	exon	22125472	22126135	.	+	.	gene_id "lnc-bbug05633"	transcript_id "lnc-bbut00013341"	exon_number "2"

14	lncRNA	exon	22167812	22168034	.	+	.	gene_id "lnc-bbug05634"	transcript_id "lnc-bbut00013346"	exon_number "1"
14	lncRNA	exon	22168750	22168783	.	+	.	gene_id "lnc-bbug05634"	transcript_id "lnc-bbut00013346"	exon_number "2"
14	lncRNA	exon	22169442	22169500	.	+	.	gene_id "lnc-bbug05634"	transcript_id "lnc-bbut00013346"	exon_number "3"
14	lncRNA	exon	22170377	22170459	.	+	.	gene_id "lnc-bbug05634"	transcript_id "lnc-bbut00013346"	exon_number "4"
14	lncRNA	exon	22172580	22172629	.	+	.	gene_id "lnc-bbug05634"	transcript_id "lnc-bbut00013346"	exon_number "5"
14	lncRNA	exon	22173609	22173683	.	+	.	gene_id "lnc-bbug05634"	transcript_id "lnc-bbut00013346"	exon_number "6"
14	lncRNA	exon	22174799	22174970	.	+	.	gene_id "lnc-bbug05634"	transcript_id "lnc-bbut00013346"	exon_number "7"
14	lncRNA	exon	22175988	22176964	.	+	.	gene_id "lnc-bbug05634"	transcript_id "lnc-bbut00013346"	exon_number "8"
14	lncRNA	exon	22179534	22179897	.	+	.	gene_id "lnc-bbug05635"	transcript_id "lnc-bbut00013348"	exon_number "1"
14	lncRNA	exon	22185516	22185706	.	+	.	gene_id "lnc-bbug05635"	transcript_id "lnc-bbut00013348"	exon_number "2"
14	lncRNA	exon	22186002	22186102	.	+	.	gene_id "lnc-bbug05635"	transcript_id "lnc-bbut00013348"	exon_number "3"
14	lncRNA	exon	22186931	22187027	.	+	.	gene_id "lnc-bbug05635"	transcript_id "lnc-bbut00013348"	exon_number "4"
14	lncRNA	exon	22187147	22187212	.	+	.	gene_id "lnc-bbug05635"	transcript_id "lnc-bbut00013348"	exon_number "5"
14	lncRNA	exon	22187983	22188354	.	+	.	gene_id "lnc-bbug05635"	transcript_id "lnc-bbut00013348"	exon_number "6"
14	lncRNA	exon	22188818	22188936	.	+	.	gene_id "lnc-bbug05635"	transcript_id "lnc-bbut00013348"	exon_number "7"
14	lncRNA	exon	22189170	22190442	.	+	.	gene_id "lnc-bbug05635"	transcript_id "lnc-bbut00013348"	exon_number "8"
14	lncRNA	exon	22500953	22501160	.	+	.	gene_id "lnc-bbug05638"	transcript_id "lnc-bbut00013355"	exon_number "1"
14	lncRNA	exon	22503757	22503954	.	+	.	gene_id "lnc-bbug05638"	transcript_id "lnc-bbut00013355"	exon_number "2"
14	lncRNA	exon	22536943	22537036	.	+	.	gene_id "lnc-bbug05639"	transcript_id "lnc-bbut00013356"	exon_number "1"
14	lncRNA	exon	22542996	22543233	.	+	.	gene_id "lnc-bbug05639"	transcript_id "lnc-bbut00013356"	exon_number "2"
14	lncRNA	exon	22582143	22582359	.	+	.	gene_id "lnc-bbug05641"	transcript_id "lnc-bbut00013358"	exon_number "1"
14	lncRNA	exon	22583537	22583824	.	+	.	gene_id "lnc-bbug05641"	transcript_id "lnc-bbut00013358"	exon_number "2"
14	lncRNA	exon	22582188	22582359	.	+	.	gene_id "lnc-bbug05641"	transcript_id "lnc-bbut00013359"	exon_number "1"
14	lncRNA	exon	22592239	22592495	.	+	.	gene_id "lnc-bbug05641"	transcript_id "lnc-bbut00013359"	exon_number "2"
14	lncRNA	exon	22649281	22649386	.	+	.	gene_id "lnc-bbug05642"	transcript_id "lnc-bbut00013360"	exon_number "1"
14	lncRNA	exon	22654175	22654493	.	+	.	gene_id "lnc-bbug05642"	transcript_id "lnc-bbut00013360"	exon_number "2"
14	lncRNA	exon	22776470	22776518	.	+	.	gene_id "lnc-bbug05643"	transcript_id "lnc-bbut00013361"	exon_number "1"
14	lncRNA	exon	22778433	22778768	.	+	.	gene_id "lnc-bbug05643"	transcript_id "lnc-bbut00013361"	exon_number "2"
14	lncRNA	exon	22867696	22869057	.	+	.	gene_id "lnc-bbug05646"	transcript_id "lnc-bbut00013364"	exon_number "1"
14	lncRNA	exon	22870891	22870968	.	+	.	gene_id "lnc-bbug05646"	transcript_id "lnc-bbut00013364"	exon_number "2"
14	lncRNA	exon	22873374	22873426	.	+	.	gene_id "lnc-bbug05646"	transcript_id "lnc-bbut00013364"	exon_number "3"
14	lncRNA	exon	23066298	23066524	.	+	.	gene_id "lnc-bbug05651"	transcript_id "lnc-bbut00013372"	exon_number "1"
14	lncRNA	exon	23070952	23071095	.	+	.	gene_id "lnc-bbug05651"	transcript_id "lnc-bbut00013372"	exon_number "2"
14	lncRNA	exon	23077487	23078165	.	+	.	gene_id "lnc-bbug05651"	transcript_id "lnc-bbut00013372"	exon_number "3"
14	lncRNA	exon	23066300	23066524	.	+	.	gene_id "lnc-bbug05651"	transcript_id "lnc-bbut00013373"	exon_number "1"
14	lncRNA	exon	23070952	23071095	.	+	.	gene_id "lnc-bbug05651"	transcript_id "lnc-bbut00013373"	exon_number "2"
14	lncRNA	exon	23077487	23077661	.	+	.	gene_id "lnc-bbug05651"	transcript_id "lnc-bbut00013373"	exon_number "3"
14	lncRNA	exon	23088527	23088638	.	+	.	gene_id "lnc-bbug05651"	transcript_id "lnc-bbut00013373"	exon_number "4"
14	lncRNA	exon	23223564	23223912	.	+	.	gene_id "lnc-bbug05652"	transcript_id "lnc-bbut00013374"	exon_number "1"
14	lncRNA	exon	23224786	23224856	.	+	.	gene_id "lnc-bbug05652"	transcript_id "lnc-bbut00013374"	exon_number "2"
14	lncRNA	exon	23227583	23230339	.	+	.	gene_id "lnc-bbug05652"	transcript_id "lnc-bbut00013374"	exon_number "3"
14	lncRNA	exon	23372671	23374453	.	+	.	gene_id "lnc-bbug05654"	transcript_id "lnc-bbut00013379"	exon_number "1"
14	lncRNA	exon	23377746	23378091	.	+	.	gene_id "lnc-bbug05654"	transcript_id "lnc-bbut00013379"	exon_number "2"
14	lncRNA	exon	23636972	23638019	.	+	.	gene_id "lnc-bbug05656"	transcript_id "lnc-bbut00013381"	exon_number "1"
14	lncRNA	exon	23639997	23645067	.	+	.	gene_id "lnc-bbug05656"	transcript_id "lnc-bbut00013381"	exon_number "2"
14	lncRNA	exon	23658491	23658753	.	+	.	gene_id "lnc-bbug05657"	transcript_id "lnc-bbut00013382"	exon_number "1"
14	lncRNA	exon	23665855	23666414	.	+	.	gene_id "lnc-bbug05657"	transcript_id "lnc-bbut00013382"	exon_number "2"
14	lncRNA	exon	23783098	23783358	.	+	.	gene_id "lnc-bbug05660"	transcript_id "lnc-bbut00013393"	exon_number "1"
14	lncRNA	exon	23794342	23794464	.	+	.	gene_id "lnc-bbug05660"	transcript_id "lnc-bbut00013393"	exon_number "2"
14	lncRNA	exon	23796437	23796540	.	+	.	gene_id "lnc-bbug05660"	transcript_id "lnc-bbut00013393"	exon_number "3"
14	lncRNA	exon	23799216	23799481	.	+	.	gene_id "lnc-bbug05660"	transcript_id "lnc-bbut00013393"	exon_number "4"
14	lncRNA	exon	24015390	24015769	.	+	.	gene_id "lnc-bbug05663"	transcript_id "lnc-bbut00013396"	exon_number "1"

14	lncRNA	exon	24016942	24017179	.	+	.	gene_id "lnc-bbug05663"	transcript_id "lnc-bbut00013396"	exon_number "2"
14	lncRNA	exon	24320004	24320751	.	+	.	gene_id "lnc-bbug05669"	transcript_id "lnc-bbut00013407"	exon_number "1"
14	lncRNA	exon	24322749	24322832	.	+	.	gene_id "lnc-bbug05669"	transcript_id "lnc-bbut00013407"	exon_number "2"
14	lncRNA	exon	24327184	24327815	.	+	.	gene_id "lnc-bbug05669"	transcript_id "lnc-bbut00013407"	exon_number "3"
14	lncRNA	exon	25289440	25289572	.	+	.	gene_id "lnc-bbug05674"	transcript_id "lnc-bbut00013419"	exon_number "1"
14	lncRNA	exon	25297478	25299131	.	+	.	gene_id "lnc-bbug05674"	transcript_id "lnc-bbut00013419"	exon_number "2"
14	lncRNA	exon	28515569	28517131	.	+	.	gene_id "lnc-bbug05689"	transcript_id "lnc-bbut00013471"	exon_number "1"
14	lncRNA	exon	28517289	28517982	.	+	.	gene_id "lnc-bbug05689"	transcript_id "lnc-bbut00013471"	exon_number "2"
14	lncRNA	exon	28748855	28748952	.	+	.	gene_id "lnc-bbug05694"	transcript_id "lnc-bbut00013487"	exon_number "1"
14	lncRNA	exon	28749199	28749261	.	+	.	gene_id "lnc-bbug05694"	transcript_id "lnc-bbut00013487"	exon_number "2"
14	lncRNA	exon	28749382	28749445	.	+	.	gene_id "lnc-bbug05694"	transcript_id "lnc-bbut00013487"	exon_number "3"
14	lncRNA	exon	28749584	28749655	.	+	.	gene_id "lnc-bbug05694"	transcript_id "lnc-bbut00013487"	exon_number "4"
14	lncRNA	exon	28750026	28750081	.	+	.	gene_id "lnc-bbug05694"	transcript_id "lnc-bbut00013487"	exon_number "5"
14	lncRNA	exon	28750161	28750224	.	+	.	gene_id "lnc-bbug05694"	transcript_id "lnc-bbut00013487"	exon_number "6"
14	lncRNA	exon	29477965	29478120	.	+	.	gene_id "lnc-bbug05705"	transcript_id "lnc-bbut00013506"	exon_number "1"
14	lncRNA	exon	29478587	29478667	.	+	.	gene_id "lnc-bbug05705"	transcript_id "lnc-bbut00013506"	exon_number "2"
14	lncRNA	exon	29478882	29478959	.	+	.	gene_id "lnc-bbug05705"	transcript_id "lnc-bbut00013506"	exon_number "3"
14	lncRNA	exon	29479041	29479137	.	+	.	gene_id "lnc-bbug05705"	transcript_id "lnc-bbut00013506"	exon_number "4"
14	lncRNA	exon	29479213	29479612	.	+	.	gene_id "lnc-bbug05705"	transcript_id "lnc-bbut00013506"	exon_number "5"
14	lncRNA	exon	29478220	29478438	.	+	.	gene_id "lnc-bbug05705"	transcript_id "lnc-bbut00013507"	exon_number "1"
14	lncRNA	exon	29478587	29478667	.	+	.	gene_id "lnc-bbug05705"	transcript_id "lnc-bbut00013507"	exon_number "2"
14	lncRNA	exon	29478882	29478959	.	+	.	gene_id "lnc-bbug05705"	transcript_id "lnc-bbut00013507"	exon_number "3"
14	lncRNA	exon	29479041	29479137	.	+	.	gene_id "lnc-bbug05705"	transcript_id "lnc-bbut00013507"	exon_number "4"
14	lncRNA	exon	29479213	29479612	.	+	.	gene_id "lnc-bbug05705"	transcript_id "lnc-bbut00013507"	exon_number "5"
14	lncRNA	exon	29520914	29521114	.	+	.	gene_id "lnc-bbug05707"	transcript_id "lnc-bbut00013511"	exon_number "1"
14	lncRNA	exon	29521232	29522692	.	+	.	gene_id "lnc-bbug05707"	transcript_id "lnc-bbut00013511"	exon_number "2"
14	lncRNA	exon	29778781	29780593	.	+	.	gene_id "lnc-bbug05715"	transcript_id "lnc-bbut00013535"	exon_number "1"
14	lncRNA	exon	29780737	29780865	.	+	.	gene_id "lnc-bbug05715"	transcript_id "lnc-bbut00013535"	exon_number "2"
14	lncRNA	exon	29782339	29782476	.	+	.	gene_id "lnc-bbug05715"	transcript_id "lnc-bbut00013535"	exon_number "3"
14	lncRNA	exon	29782559	29782711	.	+	.	gene_id "lnc-bbug05715"	transcript_id "lnc-bbut00013535"	exon_number "4"
14	lncRNA	exon	29782830	29783137	.	+	.	gene_id "lnc-bbug05715"	transcript_id "lnc-bbut00013535"	exon_number "5"
14	lncRNA	exon	29778781	29780593	.	+	.	gene_id "lnc-bbug05715"	transcript_id "lnc-bbut00013534"	exon_number "1"
14	lncRNA	exon	29780737	29780865	.	+	.	gene_id "lnc-bbug05715"	transcript_id "lnc-bbut00013534"	exon_number "2"
14	lncRNA	exon	29782330	29782476	.	+	.	gene_id "lnc-bbug05715"	transcript_id "lnc-bbut00013534"	exon_number "3"
14	lncRNA	exon	29782559	29782711	.	+	.	gene_id "lnc-bbug05715"	transcript_id "lnc-bbut00013534"	exon_number "4"
14	lncRNA	exon	29782830	29783137	.	+	.	gene_id "lnc-bbug05715"	transcript_id "lnc-bbut00013534"	exon_number "5"
14	lncRNA	exon	29778781	29780593	.	+	.	gene_id "lnc-bbug05715"	transcript_id "lnc-bbut00013533"	exon_number "1"
14	lncRNA	exon	29780737	29780865	.	+	.	gene_id "lnc-bbug05715"	transcript_id "lnc-bbut00013533"	exon_number "2"
14	lncRNA	exon	29782204	29782476	.	+	.	gene_id "lnc-bbug05715"	transcript_id "lnc-bbut00013533"	exon_number "3"
14	lncRNA	exon	29782559	29782711	.	+	.	gene_id "lnc-bbug05715"	transcript_id "lnc-bbut00013533"	exon_number "4"
14	lncRNA	exon	29782830	29783137	.	+	.	gene_id "lnc-bbug05715"	transcript_id "lnc-bbut00013533"	exon_number "5"
14	lncRNA	exon	29967278	29970761	.	+	.	gene_id "lnc-bbug05720"	transcript_id "lnc-bbut00013563"	exon_number "1"
14	lncRNA	exon	29994542	29995163	.	+	.	gene_id "lnc-bbug05720"	transcript_id "lnc-bbut00013563"	exon_number "2"
14	lncRNA	exon	30514347	30514802	.	+	.	gene_id "lnc-bbug05723"	transcript_id "lnc-bbut00013573"	exon_number "1"
14	lncRNA	exon	30516650	30516771	.	+	.	gene_id "lnc-bbug05723"	transcript_id "lnc-bbut00013573"	exon_number "2"
14	lncRNA	exon	30517352	30517499	.	+	.	gene_id "lnc-bbug05723"	transcript_id "lnc-bbut00013573"	exon_number "3"
14	lncRNA	exon	30518204	30518310	.	+	.	gene_id "lnc-bbug05723"	transcript_id "lnc-bbut00013573"	exon_number "4"
14	lncRNA	exon	31005783	31006049	.	+	.	gene_id "lnc-bbug05728"	transcript_id "lnc-bbut00013580"	exon_number "1"
14	lncRNA	exon	31307941	31308395	.	+	.	gene_id "lnc-bbug05734"	transcript_id "lnc-bbut00013596"	exon_number "1"
14	lncRNA	exon	31308530	31309012	.	+	.	gene_id "lnc-bbug05734"	transcript_id "lnc-bbut00013596"	exon_number "2"
14	lncRNA	exon	31539130	31541288	.	+	.	gene_id "lnc-bbug05738"	transcript_id "lnc-bbut00013610"	exon_number "1"
14	lncRNA	exon	31541412	31542244	.	+	.	gene_id "lnc-bbug05738"	transcript_id "lnc-bbut00013610"	exon_number "2"

14	lncRNA	exon	33131391	33132661	.	+	.	gene_id "lnc-bbug05752"	transcript_id "lnc-bbut00013656"	exon_number "1"
14	lncRNA	exon	33887717	33888860	.	+	.	gene_id "lnc-bbug05753"	transcript_id "lnc-bbut00013657"	exon_number "1"
14	lncRNA	exon	33889069	33889120	.	+	.	gene_id "lnc-bbug05753"	transcript_id "lnc-bbut00013657"	exon_number "2"
14	lncRNA	exon	35899813	35900365	.	+	.	gene_id "lnc-bbug05758"	transcript_id "lnc-bbut00013668"	exon_number "1"
14	lncRNA	exon	35903405	35903441	.	+	.	gene_id "lnc-bbug05758"	transcript_id "lnc-bbut00013668"	exon_number "2"
14	lncRNA	exon	35920946	35921535	.	+	.	gene_id "lnc-bbug05759"	transcript_id "lnc-bbut00013669"	exon_number "1"
14	lncRNA	exon	36246947	36247709	.	+	.	gene_id "lnc-bbug05763"	transcript_id "lnc-bbut00013677"	exon_number "1"
14	lncRNA	exon	36265204	36265211	.	+	.	gene_id "lnc-bbug05764"	transcript_id "lnc-bbut00013678"	exon_number "1"
14	lncRNA	exon	36271586	36272332	.	+	.	gene_id "lnc-bbug05764"	transcript_id "lnc-bbut00013678"	exon_number "2"
14	lncRNA	exon	36348974	36349204	.	+	.	gene_id "lnc-bbug05766"	transcript_id "lnc-bbut00013680"	exon_number "1"
14	lncRNA	exon	36351237	36351350	.	+	.	gene_id "lnc-bbug05766"	transcript_id "lnc-bbut00013680"	exon_number "2"
14	lncRNA	exon	36351716	36351794	.	+	.	gene_id "lnc-bbug05766"	transcript_id "lnc-bbut00013680"	exon_number "3"
14	lncRNA	exon	36352674	36352796	.	+	.	gene_id "lnc-bbug05766"	transcript_id "lnc-bbut00013680"	exon_number "4"
14	lncRNA	exon	36356134	36357220	.	+	.	gene_id "lnc-bbug05766"	transcript_id "lnc-bbut00013680"	exon_number "5"
14	lncRNA	exon	36348997	36349204	.	+	.	gene_id "lnc-bbug05766"	transcript_id "lnc-bbut00013681"	exon_number "1"
14	lncRNA	exon	36349318	36349379	.	+	.	gene_id "lnc-bbug05766"	transcript_id "lnc-bbut00013681"	exon_number "2"
14	lncRNA	exon	36351237	36351350	.	+	.	gene_id "lnc-bbug05766"	transcript_id "lnc-bbut00013681"	exon_number "3"
14	lncRNA	exon	36352674	36352796	.	+	.	gene_id "lnc-bbug05766"	transcript_id "lnc-bbut00013681"	exon_number "4"
14	lncRNA	exon	36356134	36357220	.	+	.	gene_id "lnc-bbug05766"	transcript_id "lnc-bbut00013681"	exon_number "5"
14	lncRNA	exon	36349012	36349204	.	+	.	gene_id "lnc-bbug05766"	transcript_id "lnc-bbut00013682"	exon_number "1"
14	lncRNA	exon	36351237	36351350	.	+	.	gene_id "lnc-bbug05766"	transcript_id "lnc-bbut00013682"	exon_number "2"
14	lncRNA	exon	36352674	36352796	.	+	.	gene_id "lnc-bbug05766"	transcript_id "lnc-bbut00013682"	exon_number "3"
14	lncRNA	exon	36356134	36357220	.	+	.	gene_id "lnc-bbug05766"	transcript_id "lnc-bbut00013682"	exon_number "4"
14	lncRNA	exon	36351237	36351350	.	+	.	gene_id "lnc-bbug05766"	transcript_id "lnc-bbut00013683"	exon_number "1"
14	lncRNA	exon	36351716	36351794	.	+	.	gene_id "lnc-bbug05766"	transcript_id "lnc-bbut00013683"	exon_number "2"
14	lncRNA	exon	36352674	36352796	.	+	.	gene_id "lnc-bbug05766"	transcript_id "lnc-bbut00013683"	exon_number "3"
14	lncRNA	exon	36352929	36352996	.	+	.	gene_id "lnc-bbug05766"	transcript_id "lnc-bbut00013683"	exon_number "4"
14	lncRNA	exon	36356134	36357220	.	+	.	gene_id "lnc-bbug05766"	transcript_id "lnc-bbut00013683"	exon_number "5"
14	lncRNA	exon	36380548	36380833	.	+	.	gene_id "lnc-bbug05767"	transcript_id "lnc-bbut00013685"	exon_number "1"
14	lncRNA	exon	36390019	36390140	.	+	.	gene_id "lnc-bbug05767"	transcript_id "lnc-bbut00013685"	exon_number "2"
14	lncRNA	exon	36419184	36419294	.	+	.	gene_id "lnc-bbug05767"	transcript_id "lnc-bbut00013685"	exon_number "3"
14	lncRNA	exon	36422226	36422311	.	+	.	gene_id "lnc-bbug05767"	transcript_id "lnc-bbut00013685"	exon_number "4"
14	lncRNA	exon	36429696	36429892	.	+	.	gene_id "lnc-bbug05767"	transcript_id "lnc-bbut00013685"	exon_number "5"
14	lncRNA	exon	36380548	36380833	.	+	.	gene_id "lnc-bbug05767"	transcript_id "lnc-bbut00013684"	exon_number "1"
14	lncRNA	exon	36390019	36390140	.	+	.	gene_id "lnc-bbug05767"	transcript_id "lnc-bbut00013684"	exon_number "2"
14	lncRNA	exon	36419184	36419294	.	+	.	gene_id "lnc-bbug05767"	transcript_id "lnc-bbut00013684"	exon_number "3"
14	lncRNA	exon	36419818	36422311	.	+	.	gene_id "lnc-bbug05767"	transcript_id "lnc-bbut00013684"	exon_number "4"
14	lncRNA	exon	36429696	36429892	.	+	.	gene_id "lnc-bbug05767"	transcript_id "lnc-bbut00013684"	exon_number "5"
14	lncRNA	exon	36670975	36671132	.	+	.	gene_id "lnc-bbug05770"	transcript_id "lnc-bbut00013693"	exon_number "1"
14	lncRNA	exon	36672021	36672060	.	+	.	gene_id "lnc-bbug05770"	transcript_id "lnc-bbut00013693"	exon_number "2"
14	lncRNA	exon	36673120	36673310	.	+	.	gene_id "lnc-bbug05770"	transcript_id "lnc-bbut00013693"	exon_number "3"
14	lncRNA	exon	37517445	37518931	.	+	.	gene_id "lnc-bbug05775"	transcript_id "lnc-bbut00013717"	exon_number "1"
14	lncRNA	exon	38844419	38844683	.	+	.	gene_id "lnc-bbug05777"	transcript_id "lnc-bbut00013720"	exon_number "1"
14	lncRNA	exon	38852876	38853275	.	+	.	gene_id "lnc-bbug05777"	transcript_id "lnc-bbut00013720"	exon_number "2"
14	lncRNA	exon	39253561	39253716	.	+	.	gene_id "lnc-bbug05778"	transcript_id "lnc-bbut00013721"	exon_number "1"
14	lncRNA	exon	39254378	39254544	.	+	.	gene_id "lnc-bbug05778"	transcript_id "lnc-bbut00013721"	exon_number "2"
14	lncRNA	exon	39254639	39254674	.	+	.	gene_id "lnc-bbug05778"	transcript_id "lnc-bbut00013721"	exon_number "3"
14	lncRNA	exon	39255895	39256149	.	+	.	gene_id "lnc-bbug05778"	transcript_id "lnc-bbut00013721"	exon_number "4"
14	lncRNA	exon	39253566	39253716	.	+	.	gene_id "lnc-bbug05778"	transcript_id "lnc-bbut00013722"	exon_number "1"
14	lncRNA	exon	39254434	39254544	.	+	.	gene_id "lnc-bbug05778"	transcript_id "lnc-bbut00013722"	exon_number "2"
14	lncRNA	exon	39254639	39254674	.	+	.	gene_id "lnc-bbug05778"	transcript_id "lnc-bbut00013722"	exon_number "3"
14	lncRNA	exon	39255895	39256149	.	+	.	gene_id "lnc-bbug05778"	transcript_id "lnc-bbut00013722"	exon_number "4"

14	lncRNA	exon	39264361	39264603	.	+	.	gene_id "lnc-bbug05779"	transcript_id "lnc-bbut00013725"	exon_number "1"
14	lncRNA	exon	39267835	39267891	.	+	.	gene_id "lnc-bbug05779"	transcript_id "lnc-bbut00013725"	exon_number "2"
14	lncRNA	exon	39269001	39269079	.	+	.	gene_id "lnc-bbug05779"	transcript_id "lnc-bbut00013725"	exon_number "3"
14	lncRNA	exon	39270535	39279405	.	+	.	gene_id "lnc-bbug05779"	transcript_id "lnc-bbut00013725"	exon_number "4"
14	lncRNA	exon	39317524	39317595	.	+	.	gene_id "lnc-bbug05780"	transcript_id "lnc-bbut00013726"	exon_number "1"
14	lncRNA	exon	39360372	39361795	.	+	.	gene_id "lnc-bbug05780"	transcript_id "lnc-bbut00013726"	exon_number "2"
14	lncRNA	exon	39322986	39323034	.	+	.	gene_id "lnc-bbug05780"	transcript_id "lnc-bbut00013727"	exon_number "1"
14	lncRNA	exon	39323703	39323815	.	+	.	gene_id "lnc-bbug05780"	transcript_id "lnc-bbut00013727"	exon_number "2"
14	lncRNA	exon	39346636	39347460	.	+	.	gene_id "lnc-bbug05780"	transcript_id "lnc-bbut00013727"	exon_number "3"
14	lncRNA	exon	39323024	39323175	.	+	.	gene_id "lnc-bbug05780"	transcript_id "lnc-bbut00013728"	exon_number "1"
14	lncRNA	exon	39323703	39323815	.	+	.	gene_id "lnc-bbug05780"	transcript_id "lnc-bbut00013728"	exon_number "2"
14	lncRNA	exon	39346636	39347460	.	+	.	gene_id "lnc-bbug05780"	transcript_id "lnc-bbut00013728"	exon_number "3"
14	lncRNA	exon	39323024	39323175	.	+	.	gene_id "lnc-bbug05780"	transcript_id "lnc-bbut00013730"	exon_number "1"
14	lncRNA	exon	39323703	39323815	.	+	.	gene_id "lnc-bbug05780"	transcript_id "lnc-bbut00013730"	exon_number "2"
14	lncRNA	exon	39360372	39361795	.	+	.	gene_id "lnc-bbug05780"	transcript_id "lnc-bbut00013730"	exon_number "3"
14	lncRNA	exon	39323024	39323034	.	+	.	gene_id "lnc-bbug05780"	transcript_id "lnc-bbut00013729"	exon_number "1"
14	lncRNA	exon	39323703	39323815	.	+	.	gene_id "lnc-bbug05780"	transcript_id "lnc-bbut00013729"	exon_number "2"
14	lncRNA	exon	39360372	39361795	.	+	.	gene_id "lnc-bbug05780"	transcript_id "lnc-bbut00013729"	exon_number "3"
14	lncRNA	exon	39323790	39323815	.	+	.	gene_id "lnc-bbug05780"	transcript_id "lnc-bbut00013731"	exon_number "1"
14	lncRNA	exon	39367844	39367981	.	+	.	gene_id "lnc-bbug05780"	transcript_id "lnc-bbut00013731"	exon_number "2"
14	lncRNA	exon	39368957	39369148	.	+	.	gene_id "lnc-bbug05780"	transcript_id "lnc-bbut00013731"	exon_number "3"
14	lncRNA	exon	39373311	39374121	.	+	.	gene_id "lnc-bbug05780"	transcript_id "lnc-bbut00013731"	exon_number "4"
14	lncRNA	exon	39423652	39423805	.	+	.	gene_id "lnc-bbug05781"	transcript_id "lnc-bbut00013734"	exon_number "1"
14	lncRNA	exon	39424031	39424121	.	+	.	gene_id "lnc-bbug05781"	transcript_id "lnc-bbut00013734"	exon_number "2"
14	lncRNA	exon	39439758	39440055	.	+	.	gene_id "lnc-bbug05781"	transcript_id "lnc-bbut00013734"	exon_number "3"
14	lncRNA	exon	39423652	39423805	.	+	.	gene_id "lnc-bbug05781"	transcript_id "lnc-bbut00013735"	exon_number "1"
14	lncRNA	exon	39424031	39424121	.	+	.	gene_id "lnc-bbug05781"	transcript_id "lnc-bbut00013735"	exon_number "2"
14	lncRNA	exon	39440662	39443455	.	+	.	gene_id "lnc-bbug05781"	transcript_id "lnc-bbut00013735"	exon_number "3"
14	lncRNA	exon	40990469	40990870	.	+	.	gene_id "lnc-bbug05787"	transcript_id "lnc-bbut00013760"	exon_number "1"
14	lncRNA	exon	40996696	40998587	.	+	.	gene_id "lnc-bbug05787"	transcript_id "lnc-bbut00013760"	exon_number "2"
14	lncRNA	exon	41001606	41001751	.	+	.	gene_id "lnc-bbug05787"	transcript_id "lnc-bbut00013760"	exon_number "3"
14	lncRNA	exon	40995057	40995874	.	+	.	gene_id "lnc-bbug05787"	transcript_id "lnc-bbut00013761"	exon_number "1"
14	lncRNA	exon	40996696	40998587	.	+	.	gene_id "lnc-bbug05787"	transcript_id "lnc-bbut00013761"	exon_number "2"
14	lncRNA	exon	41001606	41001751	.	+	.	gene_id "lnc-bbug05787"	transcript_id "lnc-bbut00013761"	exon_number "3"
14	lncRNA	exon	40995058	40996791	.	+	.	gene_id "lnc-bbug05787"	transcript_id "lnc-bbut00013762"	exon_number "1"
14	lncRNA	exon	40997258	40998587	.	+	.	gene_id "lnc-bbug05787"	transcript_id "lnc-bbut00013762"	exon_number "2"
14	lncRNA	exon	41001606	41001751	.	+	.	gene_id "lnc-bbug05787"	transcript_id "lnc-bbut00013762"	exon_number "3"
14	lncRNA	exon	41718189	41718435	.	+	.	gene_id "lnc-bbug05794"	transcript_id "lnc-bbut00013777"	exon_number "1"
14	lncRNA	exon	41719938	41720068	.	+	.	gene_id "lnc-bbug05794"	transcript_id "lnc-bbut00013777"	exon_number "2"
14	lncRNA	exon	41720839	41721075	.	+	.	gene_id "lnc-bbug05794"	transcript_id "lnc-bbut00013777"	exon_number "3"
14	lncRNA	exon	41921921	41922051	.	+	.	gene_id "lnc-bbug05797"	transcript_id "lnc-bbut00013783"	exon_number "1"
14	lncRNA	exon	41923462	41924359	.	+	.	gene_id "lnc-bbug05797"	transcript_id "lnc-bbut00013783"	exon_number "2"
14	lncRNA	exon	41956533	41956726	.	+	.	gene_id "lnc-bbug05798"	transcript_id "lnc-bbut00013784"	exon_number "1"
14	lncRNA	exon	41957408	41957593	.	+	.	gene_id "lnc-bbug05798"	transcript_id "lnc-bbut00013784"	exon_number "2"
14	lncRNA	exon	41974940	41975794	.	+	.	gene_id "lnc-bbug05798"	transcript_id "lnc-bbut00013784"	exon_number "3"
14	lncRNA	exon	42259726	42260409	.	+	.	gene_id "lnc-bbug05801"	transcript_id "lnc-bbut00013787"	exon_number "1"
14	lncRNA	exon	42324218	42324271	.	+	.	gene_id "lnc-bbug05802"	transcript_id "lnc-bbut00013789"	exon_number "1"
14	lncRNA	exon	42325266	42325335	.	+	.	gene_id "lnc-bbug05802"	transcript_id "lnc-bbut00013789"	exon_number "2"
14	lncRNA	exon	42342829	42349427	.	+	.	gene_id "lnc-bbug05802"	transcript_id "lnc-bbut00013789"	exon_number "3"
14	lncRNA	exon	42324247	42324271	.	+	.	gene_id "lnc-bbug05802"	transcript_id "lnc-bbut00013790"	exon_number "1"
14	lncRNA	exon	42325266	42325335	.	+	.	gene_id "lnc-bbug05802"	transcript_id "lnc-bbut00013790"	exon_number "2"
14	lncRNA	exon	42341764	42349427	.	+	.	gene_id "lnc-bbug05802"	transcript_id "lnc-bbut00013790"	exon_number "3"

14	lncRNA	exon	42360150	42361126	.	+	.	gene_id "lnc-bbug05803"	transcript_id "lnc-bbut00013793"	exon_number "1"
14	lncRNA	exon	42376895	42376968	.	+	.	gene_id "lnc-bbug05803"	transcript_id "lnc-bbut00013793"	exon_number "2"
14	lncRNA	exon	42378473	42381229	.	+	.	gene_id "lnc-bbug05803"	transcript_id "lnc-bbut00013793"	exon_number "3"
14	lncRNA	exon	42422863	42422960	.	+	.	gene_id "lnc-bbug05804"	transcript_id "lnc-bbut00013795"	exon_number "1"
14	lncRNA	exon	42438556	42438705	.	+	.	gene_id "lnc-bbug05804"	transcript_id "lnc-bbut00013795"	exon_number "2"
14	lncRNA	exon	42440531	42442441	.	+	.	gene_id "lnc-bbug05804"	transcript_id "lnc-bbut00013795"	exon_number "3"
14	lncRNA	exon	43266602	43267601	.	+	.	gene_id "lnc-bbug05807"	transcript_id "lnc-bbut00013799"	exon_number "1"
14	lncRNA	exon	43267896	43268325	.	+	.	gene_id "lnc-bbug05807"	transcript_id "lnc-bbut00013799"	exon_number "2"
14	lncRNA	exon	43670082	43670235	.	+	.	gene_id "lnc-bbug05809"	transcript_id "lnc-bbut00013801"	exon_number "1"
14	lncRNA	exon	43670389	43670511	.	+	.	gene_id "lnc-bbug05809"	transcript_id "lnc-bbut00013801"	exon_number "2"
14	lncRNA	exon	43675894	43676595	.	+	.	gene_id "lnc-bbug05809"	transcript_id "lnc-bbut00013801"	exon_number "3"
14	lncRNA	exon	44322453	44322561	.	+	.	gene_id "lnc-bbug05812"	transcript_id "lnc-bbut00013824"	exon_number "1"
14	lncRNA	exon	44323186	44323304	.	+	.	gene_id "lnc-bbug05812"	transcript_id "lnc-bbut00013824"	exon_number "2"
14	lncRNA	exon	44323966	44324252	.	+	.	gene_id "lnc-bbug05812"	transcript_id "lnc-bbut00013824"	exon_number "3"
14	lncRNA	exon	44511032	44511910	.	+	.	gene_id "lnc-bbug05813"	transcript_id "lnc-bbut00013826"	exon_number "1"
14	lncRNA	exon	44518633	44518741	.	+	.	gene_id "lnc-bbug05813"	transcript_id "lnc-bbut00013826"	exon_number "2"
14	lncRNA	exon	44532655	44533090	.	+	.	gene_id "lnc-bbug05813"	transcript_id "lnc-bbut00013826"	exon_number "3"
14	lncRNA	exon	44516218	44516603	.	+	.	gene_id "lnc-bbug05813"	transcript_id "lnc-bbut00013827"	exon_number "1"
14	lncRNA	exon	44518633	44518741	.	+	.	gene_id "lnc-bbug05813"	transcript_id "lnc-bbut00013827"	exon_number "2"
14	lncRNA	exon	44532655	44533090	.	+	.	gene_id "lnc-bbug05813"	transcript_id "lnc-bbut00013827"	exon_number "3"
14	lncRNA	exon	44973426	44973539	.	+	.	gene_id "lnc-bbug05814"	transcript_id "lnc-bbut00013828"	exon_number "1"
14	lncRNA	exon	44976590	44979551	.	+	.	gene_id "lnc-bbug05814"	transcript_id "lnc-bbut00013828"	exon_number "2"
14	lncRNA	exon	45911556	45911787	.	+	.	gene_id "lnc-bbug05824"	transcript_id "lnc-bbut00013857"	exon_number "1"
14	lncRNA	exon	45921672	45921823	.	+	.	gene_id "lnc-bbug05824"	transcript_id "lnc-bbut00013857"	exon_number "2"
14	lncRNA	exon	45924338	45924487	.	+	.	gene_id "lnc-bbug05824"	transcript_id "lnc-bbut00013857"	exon_number "3"
14	lncRNA	exon	45929723	45929936	.	+	.	gene_id "lnc-bbug05824"	transcript_id "lnc-bbut00013857"	exon_number "4"
14	lncRNA	exon	45911919	45912110	.	+	.	gene_id "lnc-bbug05824"	transcript_id "lnc-bbut00013858"	exon_number "1"
14	lncRNA	exon	45921672	45921823	.	+	.	gene_id "lnc-bbug05824"	transcript_id "lnc-bbut00013858"	exon_number "2"
14	lncRNA	exon	45924338	45924487	.	+	.	gene_id "lnc-bbug05824"	transcript_id "lnc-bbut00013858"	exon_number "3"
14	lncRNA	exon	45929723	45929936	.	+	.	gene_id "lnc-bbug05824"	transcript_id "lnc-bbut00013858"	exon_number "4"
14	lncRNA	exon	46122071	46123144	.	+	.	gene_id "lnc-bbug05827"	transcript_id "lnc-bbut00013874"	exon_number "1"
14	lncRNA	exon	46125696	46125970	.	+	.	gene_id "lnc-bbug05827"	transcript_id "lnc-bbut00013874"	exon_number "2"
14	lncRNA	exon	46784905	46785018	.	+	.	gene_id "lnc-bbug05830"	transcript_id "lnc-bbut00013877"	exon_number "1"
14	lncRNA	exon	46786180	46786757	.	+	.	gene_id "lnc-bbug05830"	transcript_id "lnc-bbut00013877"	exon_number "2"
14	lncRNA	exon	46865417	46866302	.	+	.	gene_id "lnc-bbug05832"	transcript_id "lnc-bbut00013879"	exon_number "1"
14	lncRNA	exon	46867089	46867504	.	+	.	gene_id "lnc-bbug05832"	transcript_id "lnc-bbut00013879"	exon_number "2"
14	lncRNA	exon	47217376	47217712	.	+	.	gene_id "lnc-bbug05833"	transcript_id "lnc-bbut00013880"	exon_number "1"
14	lncRNA	exon	47228563	47228614	.	+	.	gene_id "lnc-bbug05833"	transcript_id "lnc-bbut00013880"	exon_number "2"
14	lncRNA	exon	47232519	47232650	.	+	.	gene_id "lnc-bbug05834"	transcript_id "lnc-bbut00013881"	exon_number "1"
14	lncRNA	exon	47234398	47234514	.	+	.	gene_id "lnc-bbug05834"	transcript_id "lnc-bbut00013881"	exon_number "2"
14	lncRNA	exon	47315620	47315813	.	+	.	gene_id "lnc-bbug05834"	transcript_id "lnc-bbut00013881"	exon_number "3"
14	lncRNA	exon	47841896	47842014	.	+	.	gene_id "lnc-bbug05837"	transcript_id "lnc-bbut00013890"	exon_number "1"
14	lncRNA	exon	47854233	47854309	.	+	.	gene_id "lnc-bbug05837"	transcript_id "lnc-bbut00013890"	exon_number "2"
14	lncRNA	exon	47863945	47864399	.	+	.	gene_id "lnc-bbug05837"	transcript_id "lnc-bbut00013890"	exon_number "3"
14	lncRNA	exon	47888723	47889289	.	+	.	gene_id "lnc-bbug05838"	transcript_id "lnc-bbut00013891"	exon_number "1"
14	lncRNA	exon	47890400	47890581	.	+	.	gene_id "lnc-bbug05838"	transcript_id "lnc-bbut00013891"	exon_number "2"
14	lncRNA	exon	47906767	47907881	.	+	.	gene_id "lnc-bbug05838"	transcript_id "lnc-bbut00013891"	exon_number "3"
14	lncRNA	exon	47916053	47919260	.	+	.	gene_id "lnc-bbug05839"	transcript_id "lnc-bbut00013892"	exon_number "1"
14	lncRNA	exon	47921812	47921913	.	+	.	gene_id "lnc-bbug05839"	transcript_id "lnc-bbut00013892"	exon_number "2"
14	lncRNA	exon	47930254	47930305	.	+	.	gene_id "lnc-bbug05839"	transcript_id "lnc-bbut00013892"	exon_number "3"
14	lncRNA	exon	48319831	48319938	.	+	.	gene_id "lnc-bbug05840"	transcript_id "lnc-bbut00013893"	exon_number "1"
14	lncRNA	exon	48324496	48324658	.	+	.	gene_id "lnc-bbug05840"	transcript_id "lnc-bbut00013893"	exon_number "2"

14	lncRNA	exon	48325147	48325305	.	+	.	gene_id "lnc-bbug05840"	transcript_id "lnc-bbut00013893"	exon_number "3"
14	lncRNA	exon	48334639	48334717	.	+	.	gene_id "lnc-bbug05840"	transcript_id "lnc-bbut00013893"	exon_number "4"
14	lncRNA	exon	48335516	48336121	.	+	.	gene_id "lnc-bbug05840"	transcript_id "lnc-bbut00013893"	exon_number "5"
14	lncRNA	exon	48454642	48454741	.	+	.	gene_id "lnc-bbug05841"	transcript_id "lnc-bbut00013894"	exon_number "1"
14	lncRNA	exon	48455521	48455709	.	+	.	gene_id "lnc-bbug05841"	transcript_id "lnc-bbut00013894"	exon_number "2"
14	lncRNA	exon	48456519	48458091	.	+	.	gene_id "lnc-bbug05841"	transcript_id "lnc-bbut00013894"	exon_number "3"
14	lncRNA	exon	48454642	48454741	.	+	.	gene_id "lnc-bbug05841"	transcript_id "lnc-bbut00013895"	exon_number "1"
14	lncRNA	exon	48455521	48455709	.	+	.	gene_id "lnc-bbug05841"	transcript_id "lnc-bbut00013895"	exon_number "2"
14	lncRNA	exon	48458105	48460081	.	+	.	gene_id "lnc-bbug05841"	transcript_id "lnc-bbut00013895"	exon_number "3"
14	lncRNA	exon	48454642	48454741	.	+	.	gene_id "lnc-bbug05841"	transcript_id "lnc-bbut00013896"	exon_number "1"
14	lncRNA	exon	48455521	48455709	.	+	.	gene_id "lnc-bbug05841"	transcript_id "lnc-bbut00013896"	exon_number "2"
14	lncRNA	exon	48458105	48458839	.	+	.	gene_id "lnc-bbug05841"	transcript_id "lnc-bbut00013896"	exon_number "3"
14	lncRNA	exon	48460722	48461672	.	+	.	gene_id "lnc-bbug05841"	transcript_id "lnc-bbut00013896"	exon_number "4"
14	lncRNA	exon	48697627	48698686	.	+	.	gene_id "lnc-bbug05843"	transcript_id "lnc-bbut00013899"	exon_number "1"
14	lncRNA	exon	48700673	48700823	.	+	.	gene_id "lnc-bbug05843"	transcript_id "lnc-bbut00013899"	exon_number "2"
14	lncRNA	exon	48707370	48707707	.	+	.	gene_id "lnc-bbug05843"	transcript_id "lnc-bbut00013899"	exon_number "3"
14	lncRNA	exon	52886068	52886116	.	+	.	gene_id "lnc-bbug05854"	transcript_id "lnc-bbut00013956"	exon_number "1"
14	lncRNA	exon	52892590	52892811	.	+	.	gene_id "lnc-bbug05854"	transcript_id "lnc-bbut00013956"	exon_number "2"
14	lncRNA	exon	52900283	52900612	.	+	.	gene_id "lnc-bbug05854"	transcript_id "lnc-bbut00013956"	exon_number "3"
14	lncRNA	exon	52900918	52901019	.	+	.	gene_id "lnc-bbug05854"	transcript_id "lnc-bbut00013956"	exon_number "4"
14	lncRNA	exon	52906884	52907901	.	+	.	gene_id "lnc-bbug05854"	transcript_id "lnc-bbut00013956"	exon_number "5"
14	lncRNA	exon	52886068	52886116	.	+	.	gene_id "lnc-bbug05854"	transcript_id "lnc-bbut00013958"	exon_number "1"
14	lncRNA	exon	52892590	52892811	.	+	.	gene_id "lnc-bbug05854"	transcript_id "lnc-bbut00013958"	exon_number "2"
14	lncRNA	exon	52900283	52900612	.	+	.	gene_id "lnc-bbug05854"	transcript_id "lnc-bbut00013958"	exon_number "3"
14	lncRNA	exon	52900918	52901019	.	+	.	gene_id "lnc-bbug05854"	transcript_id "lnc-bbut00013958"	exon_number "4"
14	lncRNA	exon	52906884	52907071	.	+	.	gene_id "lnc-bbug05854"	transcript_id "lnc-bbut00013958"	exon_number "5"
14	lncRNA	exon	52910243	52912363	.	+	.	gene_id "lnc-bbug05854"	transcript_id "lnc-bbut00013958"	exon_number "6"
14	lncRNA	exon	52886068	52886116	.	+	.	gene_id "lnc-bbug05854"	transcript_id "lnc-bbut00013957"	exon_number "1"
14	lncRNA	exon	52892590	52892811	.	+	.	gene_id "lnc-bbug05854"	transcript_id "lnc-bbut00013957"	exon_number "2"
14	lncRNA	exon	52900283	52900612	.	+	.	gene_id "lnc-bbug05854"	transcript_id "lnc-bbut00013957"	exon_number "3"
14	lncRNA	exon	52900918	52901019	.	+	.	gene_id "lnc-bbug05854"	transcript_id "lnc-bbut00013957"	exon_number "4"
14	lncRNA	exon	52910243	52912363	.	+	.	gene_id "lnc-bbug05854"	transcript_id "lnc-bbut00013957"	exon_number "5"
14	lncRNA	exon	54091453	54092677	.	+	.	gene_id "lnc-bbug05859"	transcript_id "lnc-bbut00013971"	exon_number "1"
14	lncRNA	exon	54092734	54093642	.	+	.	gene_id "lnc-bbug05859"	transcript_id "lnc-bbut00013971"	exon_number "2"
14	lncRNA	exon	54141136	54141278	.	+	.	gene_id "lnc-bbug05860"	transcript_id "lnc-bbut00013972"	exon_number "1"
14	lncRNA	exon	54145893	54146010	.	+	.	gene_id "lnc-bbug05860"	transcript_id "lnc-bbut00013972"	exon_number "2"
14	lncRNA	exon	54146115	54146177	.	+	.	gene_id "lnc-bbug05860"	transcript_id "lnc-bbut00013972"	exon_number "3"
14	lncRNA	exon	54146261	54147688	.	+	.	gene_id "lnc-bbug05860"	transcript_id "lnc-bbut00013972"	exon_number "4"
14	lncRNA	exon	54609787	54616761	.	+	.	gene_id "lnc-bbug05865"	transcript_id "lnc-bbut00013982"	exon_number "1"
14	lncRNA	exon	54694142	54694361	.	+	.	gene_id "lnc-bbug05865"	transcript_id "lnc-bbut00013982"	exon_number "2"
14	lncRNA	exon	54699917	54699987	.	+	.	gene_id "lnc-bbug05865"	transcript_id "lnc-bbut00013982"	exon_number "3"
14	lncRNA	exon	54709371	54709503	.	+	.	gene_id "lnc-bbug05865"	transcript_id "lnc-bbut00013982"	exon_number "4"
14	lncRNA	exon	54762551	54762650	.	+	.	gene_id "lnc-bbug05865"	transcript_id "lnc-bbut00013982"	exon_number "5"
14	lncRNA	exon	54765131	54765164	.	+	.	gene_id "lnc-bbug05865"	transcript_id "lnc-bbut00013982"	exon_number "6"
14	lncRNA	exon	55904212	55905628	.	+	.	gene_id "lnc-bbug05872"	transcript_id "lnc-bbut00014004"	exon_number "1"
14	lncRNA	exon	55906096	55907291	.	+	.	gene_id "lnc-bbug05872"	transcript_id "lnc-bbut00014004"	exon_number "2"
14	lncRNA	exon	57503324	57503636	.	+	.	gene_id "lnc-bbug05875"	transcript_id "lnc-bbut00014009"	exon_number "1"
14	lncRNA	exon	57504641	57504814	.	+	.	gene_id "lnc-bbug05875"	transcript_id "lnc-bbut00014009"	exon_number "2"
14	lncRNA	exon	57506720	57506811	.	+	.	gene_id "lnc-bbug05875"	transcript_id "lnc-bbut00014009"	exon_number "3"
14	lncRNA	exon	57507389	57510210	.	+	.	gene_id "lnc-bbug05875"	transcript_id "lnc-bbut00014009"	exon_number "4"
14	lncRNA	exon	57503324	57503636	.	+	.	gene_id "lnc-bbug05875"	transcript_id "lnc-bbut00014008"	exon_number "1"
14	lncRNA	exon	57504641	57504814	.	+	.	gene_id "lnc-bbug05875"	transcript_id "lnc-bbut00014008"	exon_number "2"

14	lncRNA	exon	57506720	57510210	.	+	.	gene_id "lnc-bbug05875"	transcript_id "lnc-bbut00014008"	exon_number "3"
14	lncRNA	exon	58175756	58175812	.	+	.	gene_id "lnc-bbug05878"	transcript_id "lnc-bbut00014017"	exon_number "1"
14	lncRNA	exon	58177065	58177434	.	+	.	gene_id "lnc-bbug05878"	transcript_id "lnc-bbut00014017"	exon_number "2"
14	lncRNA	exon	58178249	58178786	.	+	.	gene_id "lnc-bbug05878"	transcript_id "lnc-bbut00014017"	exon_number "3"
14	lncRNA	exon	58735597	58735728	.	+	.	gene_id "lnc-bbug05880"	transcript_id "lnc-bbut00014026"	exon_number "1"
14	lncRNA	exon	58750874	58751047	.	+	.	gene_id "lnc-bbug05880"	transcript_id "lnc-bbut00014026"	exon_number "2"
14	lncRNA	exon	58756736	58756750	.	+	.	gene_id "lnc-bbug05880"	transcript_id "lnc-bbut00014026"	exon_number "3"
14	lncRNA	exon	60223468	60225015	.	+	.	gene_id "lnc-bbug05883"	transcript_id "lnc-bbut00014030"	exon_number "1"
14	lncRNA	exon	60225498	60225611	.	+	.	gene_id "lnc-bbug05883"	transcript_id "lnc-bbut00014030"	exon_number "2"
14	lncRNA	exon	60225971	60226028	.	+	.	gene_id "lnc-bbug05884"	transcript_id "lnc-bbut00014031"	exon_number "1"
14	lncRNA	exon	60231102	60231417	.	+	.	gene_id "lnc-bbug05884"	transcript_id "lnc-bbut00014031"	exon_number "2"
14	lncRNA	exon	60233363	60233521	.	+	.	gene_id "lnc-bbug05884"	transcript_id "lnc-bbut00014031"	exon_number "3"
14	lncRNA	exon	60236353	60236543	.	+	.	gene_id "lnc-bbug05884"	transcript_id "lnc-bbut00014031"	exon_number "4"
14	lncRNA	exon	60230454	60231417	.	+	.	gene_id "lnc-bbug05884"	transcript_id "lnc-bbut00014032"	exon_number "1"
14	lncRNA	exon	60233363	60233521	.	+	.	gene_id "lnc-bbug05884"	transcript_id "lnc-bbut00014032"	exon_number "2"
14	lncRNA	exon	60236353	60236543	.	+	.	gene_id "lnc-bbug05884"	transcript_id "lnc-bbut00014032"	exon_number "3"
14	lncRNA	exon	60301308	60301986	.	+	.	gene_id "lnc-bbug05885"	transcript_id "lnc-bbut00014033"	exon_number "1"
14	lncRNA	exon	60303017	60303165	.	+	.	gene_id "lnc-bbug05885"	transcript_id "lnc-bbut00014033"	exon_number "2"
14	lncRNA	exon	60472000	60472469	.	+	.	gene_id "lnc-bbug05886"	transcript_id "lnc-bbut00014034"	exon_number "1"
14	lncRNA	exon	60481516	60487486	.	+	.	gene_id "lnc-bbug05886"	transcript_id "lnc-bbut00014034"	exon_number "2"
14	lncRNA	exon	60549588	60551565	.	+	.	gene_id "lnc-bbug05887"	transcript_id "lnc-bbut00014035"	exon_number "1"
14	lncRNA	exon	61114793	61114851	.	+	.	gene_id "lnc-bbug05891"	transcript_id "lnc-bbut00014040"	exon_number "1"
14	lncRNA	exon	61115686	61119092	.	+	.	gene_id "lnc-bbug05891"	transcript_id "lnc-bbut00014040"	exon_number "2"
14	lncRNA	exon	62507628	62509139	.	+	.	gene_id "lnc-bbug05894"	transcript_id "lnc-bbut00014049"	exon_number "1"
14	lncRNA	exon	62509750	62509818	.	+	.	gene_id "lnc-bbug05894"	transcript_id "lnc-bbut00014049"	exon_number "2"
14	lncRNA	exon	62509927	62511805	.	+	.	gene_id "lnc-bbug05894"	transcript_id "lnc-bbut00014049"	exon_number "3"
14	lncRNA	exon	63902292	63902380	.	+	.	gene_id "lnc-bbug05896"	transcript_id "lnc-bbut00014062"	exon_number "1"
14	lncRNA	exon	63903082	63904671	.	+	.	gene_id "lnc-bbug05896"	transcript_id "lnc-bbut00014062"	exon_number "2"
14	lncRNA	exon	63902431	63902966	.	+	.	gene_id "lnc-bbug05896"	transcript_id "lnc-bbut00014063"	exon_number "1"
14	lncRNA	exon	63903082	63904671	.	+	.	gene_id "lnc-bbug05896"	transcript_id "lnc-bbut00014063"	exon_number "2"
14	lncRNA	exon	63964750	63964867	.	+	.	gene_id "lnc-bbug05897"	transcript_id "lnc-bbut00014064"	exon_number "1"
14	lncRNA	exon	63980703	63980887	.	+	.	gene_id "lnc-bbug05897"	transcript_id "lnc-bbut00014064"	exon_number "2"
14	lncRNA	exon	63985166	63986485	.	+	.	gene_id "lnc-bbug05897"	transcript_id "lnc-bbut00014064"	exon_number "3"
14	lncRNA	exon	64018914	64019439	.	+	.	gene_id "lnc-bbug05897"	transcript_id "lnc-bbut00014064"	exon_number "4"
14	lncRNA	exon	64021635	64022145	.	+	.	gene_id "lnc-bbug05897"	transcript_id "lnc-bbut00014064"	exon_number "5"
14	lncRNA	exon	64696427	64696525	.	+	.	gene_id "lnc-bbug05898"	transcript_id "lnc-bbut00014065"	exon_number "1"
14	lncRNA	exon	64699514	64700714	.	+	.	gene_id "lnc-bbug05898"	transcript_id "lnc-bbut00014065"	exon_number "2"
14	lncRNA	exon	64880600	64880730	.	+	.	gene_id "lnc-bbug05899"	transcript_id "lnc-bbut00014066"	exon_number "1"
14	lncRNA	exon	64880831	64880987	.	+	.	gene_id "lnc-bbug05899"	transcript_id "lnc-bbut00014066"	exon_number "2"
14	lncRNA	exon	64884480	64884588	.	+	.	gene_id "lnc-bbug05899"	transcript_id "lnc-bbut00014066"	exon_number "3"
14	lncRNA	exon	64886193	64886281	.	+	.	gene_id "lnc-bbug05899"	transcript_id "lnc-bbut00014066"	exon_number "4"
14	lncRNA	exon	64889324	64889844	.	+	.	gene_id "lnc-bbug05899"	transcript_id "lnc-bbut00014066"	exon_number "5"
14	lncRNA	exon	65647710	65648463	.	+	.	gene_id "lnc-bbug05900"	transcript_id "lnc-bbut00014078"	exon_number "1"
14	lncRNA	exon	65656613	65656769	.	+	.	gene_id "lnc-bbug05900"	transcript_id "lnc-bbut00014078"	exon_number "2"
14	lncRNA	exon	65660966	65661718	.	+	.	gene_id "lnc-bbug05900"	transcript_id "lnc-bbut00014078"	exon_number "3"
14	lncRNA	exon	65648896	65649201	.	+	.	gene_id "lnc-bbug05900"	transcript_id "lnc-bbut00014082"	exon_number "1"
14	lncRNA	exon	65656613	65656769	.	+	.	gene_id "lnc-bbug05900"	transcript_id "lnc-bbut00014082"	exon_number "2"
14	lncRNA	exon	65660545	65660849	.	+	.	gene_id "lnc-bbug05900"	transcript_id "lnc-bbut00014082"	exon_number "3"
14	lncRNA	exon	65648897	65649201	.	+	.	gene_id "lnc-bbug05900"	transcript_id "lnc-bbut00014083"	exon_number "1"
14	lncRNA	exon	65652767	65652802	.	+	.	gene_id "lnc-bbug05900"	transcript_id "lnc-bbut00014083"	exon_number "2"
14	lncRNA	exon	65656613	65656769	.	+	.	gene_id "lnc-bbug05900"	transcript_id "lnc-bbut00014083"	exon_number "3"
14	lncRNA	exon	65660545	65660849	.	+	.	gene_id "lnc-bbug05900"	transcript_id "lnc-bbut00014083"	exon_number "4"

14	lncRNA	exon	65612022	65612171	.	+	.	gene_id "lnc-bbug05901"	transcript_id "lnc-bbut00014073"	exon_number "1"
14	lncRNA	exon	65612636	65614296	.	+	.	gene_id "lnc-bbug05901"	transcript_id "lnc-bbut00014073"	exon_number "2"
14	lncRNA	exon	66334468	66334967	.	+	.	gene_id "lnc-bbug05909"	transcript_id "lnc-bbut00014113"	exon_number "1"
14	lncRNA	exon	66338466	66338511	.	+	.	gene_id "lnc-bbug05909"	transcript_id "lnc-bbut00014113"	exon_number "2"
14	lncRNA	exon	66346534	66347073	.	+	.	gene_id "lnc-bbug05909"	transcript_id "lnc-bbut00014113"	exon_number "3"
14	lncRNA	exon	66465882	66468834	.	+	.	gene_id "lnc-bbug05911"	transcript_id "lnc-bbut00014115"	exon_number "1"
14	lncRNA	exon	67277604	67278175	.	+	.	gene_id "lnc-bbug05914"	transcript_id "lnc-bbut00014126"	exon_number "1"
14	lncRNA	exon	67278297	67278649	.	+	.	gene_id "lnc-bbug05914"	transcript_id "lnc-bbut00014126"	exon_number "2"
14	lncRNA	exon	67354924	67355487	.	+	.	gene_id "lnc-bbug05915"	transcript_id "lnc-bbut00014127"	exon_number "1"
14	lncRNA	exon	67359276	67359329	.	+	.	gene_id "lnc-bbug05915"	transcript_id "lnc-bbut00014127"	exon_number "2"
14	lncRNA	exon	67366039	67366150	.	+	.	gene_id "lnc-bbug05915"	transcript_id "lnc-bbut00014127"	exon_number "3"
14	lncRNA	exon	67366731	67367123	.	+	.	gene_id "lnc-bbug05915"	transcript_id "lnc-bbut00014127"	exon_number "4"
14	lncRNA	exon	67372401	67372510	.	+	.	gene_id "lnc-bbug05916"	transcript_id "lnc-bbut00014128"	exon_number "1"
14	lncRNA	exon	67390152	67390538	.	+	.	gene_id "lnc-bbug05916"	transcript_id "lnc-bbut00014128"	exon_number "2"
14	lncRNA	exon	68436317	68436344	.	+	.	gene_id "lnc-bbug05918"	transcript_id "lnc-bbut00014135"	exon_number "1"
14	lncRNA	exon	68436730	68438365	.	+	.	gene_id "lnc-bbug05918"	transcript_id "lnc-bbut00014135"	exon_number "2"
14	lncRNA	exon	72557971	72558144	.	+	.	gene_id "lnc-bbug05927"	transcript_id "lnc-bbut00014168"	exon_number "1"
14	lncRNA	exon	72562114	72563228	.	+	.	gene_id "lnc-bbug05927"	transcript_id "lnc-bbut00014168"	exon_number "2"
14	lncRNA	exon	72568308	72568883	.	+	.	gene_id "lnc-bbug05927"	transcript_id "lnc-bbut00014168"	exon_number "3"
14	lncRNA	exon	72712523	72713816	.	+	.	gene_id "lnc-bbug05930"	transcript_id "lnc-bbut00014173"	exon_number "1"
14	lncRNA	exon	72735395	72736223	.	+	.	gene_id "lnc-bbug05931"	transcript_id "lnc-bbut00014174"	exon_number "1"
14	lncRNA	exon	72737168	72738318	.	+	.	gene_id "lnc-bbug05931"	transcript_id "lnc-bbut00014174"	exon_number "2"
14	lncRNA	exon	72742848	72742927	.	+	.	gene_id "lnc-bbug05931"	transcript_id "lnc-bbut00014174"	exon_number "3"
14	lncRNA	exon	72747117	72747246	.	+	.	gene_id "lnc-bbug05931"	transcript_id "lnc-bbut00014174"	exon_number "4"
14	lncRNA	exon	72748613	72748791	.	+	.	gene_id "lnc-bbug05931"	transcript_id "lnc-bbut00014174"	exon_number "5"
14	lncRNA	exon	72749384	72750275	.	+	.	gene_id "lnc-bbug05931"	transcript_id "lnc-bbut00014174"	exon_number "6"
14	lncRNA	exon	72736939	72737445	.	+	.	gene_id "lnc-bbug05931"	transcript_id "lnc-bbut00014178"	exon_number "1"
14	lncRNA	exon	72738164	72738318	.	+	.	gene_id "lnc-bbug05931"	transcript_id "lnc-bbut00014178"	exon_number "2"
14	lncRNA	exon	72742848	72742927	.	+	.	gene_id "lnc-bbug05931"	transcript_id "lnc-bbut00014178"	exon_number "3"
14	lncRNA	exon	72747117	72747246	.	+	.	gene_id "lnc-bbug05931"	transcript_id "lnc-bbut00014178"	exon_number "4"
14	lncRNA	exon	72748613	72748791	.	+	.	gene_id "lnc-bbug05931"	transcript_id "lnc-bbut00014178"	exon_number "5"
14	lncRNA	exon	72749384	72750275	.	+	.	gene_id "lnc-bbug05931"	transcript_id "lnc-bbut00014178"	exon_number "6"
14	lncRNA	exon	72736939	72737445	.	+	.	gene_id "lnc-bbug05931"	transcript_id "lnc-bbut00014177"	exon_number "1"
14	lncRNA	exon	72738149	72738318	.	+	.	gene_id "lnc-bbug05931"	transcript_id "lnc-bbut00014177"	exon_number "2"
14	lncRNA	exon	72742848	72742927	.	+	.	gene_id "lnc-bbug05931"	transcript_id "lnc-bbut00014177"	exon_number "3"
14	lncRNA	exon	72747117	72747246	.	+	.	gene_id "lnc-bbug05931"	transcript_id "lnc-bbut00014177"	exon_number "4"
14	lncRNA	exon	72748613	72748791	.	+	.	gene_id "lnc-bbug05931"	transcript_id "lnc-bbut00014177"	exon_number "5"
14	lncRNA	exon	72749384	72750275	.	+	.	gene_id "lnc-bbug05931"	transcript_id "lnc-bbut00014177"	exon_number "6"
14	lncRNA	exon	72736939	72738318	.	+	.	gene_id "lnc-bbug05931"	transcript_id "lnc-bbut00014176"	exon_number "1"
14	lncRNA	exon	72742848	72742927	.	+	.	gene_id "lnc-bbug05931"	transcript_id "lnc-bbut00014176"	exon_number "2"
14	lncRNA	exon	72747117	72747246	.	+	.	gene_id "lnc-bbug05931"	transcript_id "lnc-bbut00014176"	exon_number "3"
14	lncRNA	exon	72748613	72748791	.	+	.	gene_id "lnc-bbug05931"	transcript_id "lnc-bbut00014176"	exon_number "4"
14	lncRNA	exon	72749395	72750275	.	+	.	gene_id "lnc-bbug05931"	transcript_id "lnc-bbut00014176"	exon_number "5"
14	lncRNA	exon	72736939	72738318	.	+	.	gene_id "lnc-bbug05931"	transcript_id "lnc-bbut00014175"	exon_number "1"
14	lncRNA	exon	72742848	72742927	.	+	.	gene_id "lnc-bbug05931"	transcript_id "lnc-bbut00014175"	exon_number "2"
14	lncRNA	exon	72747117	72747246	.	+	.	gene_id "lnc-bbug05931"	transcript_id "lnc-bbut00014175"	exon_number "3"
14	lncRNA	exon	72748613	72748791	.	+	.	gene_id "lnc-bbug05931"	transcript_id "lnc-bbut00014175"	exon_number "4"
14	lncRNA	exon	72749384	72750275	.	+	.	gene_id "lnc-bbug05931"	transcript_id "lnc-bbut00014175"	exon_number "5"
14	lncRNA	exon	73011155	73011760	.	+	.	gene_id "lnc-bbug05933"	transcript_id "lnc-bbut00014186"	exon_number "1"
14	lncRNA	exon	73019338	73019660	.	+	.	gene_id "lnc-bbug05934"	transcript_id "lnc-bbut00014187"	exon_number "1"
14	lncRNA	exon	73023936	73024008	.	+	.	gene_id "lnc-bbug05934"	transcript_id "lnc-bbut00014187"	exon_number "2"
14	lncRNA	exon	73026051	73026090	.	+	.	gene_id "lnc-bbug05934"	transcript_id "lnc-bbut00014187"	exon_number "3"

14	lncRNA	exon	73080474	73080606	.	+	.	gene_id "lnc-bbug05935"	transcript_id "lnc-bbut00014189"	exon_number "1"
14	lncRNA	exon	73094912	73095160	.	+	.	gene_id "lnc-bbug05935"	transcript_id "lnc-bbut00014189"	exon_number "2"
14	lncRNA	exon	73201073	73201176	.	+	.	gene_id "lnc-bbug05935"	transcript_id "lnc-bbut00014189"	exon_number "3"
14	lncRNA	exon	73201673	73201761	.	+	.	gene_id "lnc-bbug05935"	transcript_id "lnc-bbut00014189"	exon_number "4"
14	lncRNA	exon	73201928	73205078	.	+	.	gene_id "lnc-bbug05935"	transcript_id "lnc-bbut00014189"	exon_number "5"
14	lncRNA	exon	73080474	73080606	.	+	.	gene_id "lnc-bbug05935"	transcript_id "lnc-bbut00014188"	exon_number "1"
14	lncRNA	exon	73094912	73095160	.	+	.	gene_id "lnc-bbug05935"	transcript_id "lnc-bbut00014188"	exon_number "2"
14	lncRNA	exon	73201073	73201176	.	+	.	gene_id "lnc-bbug05935"	transcript_id "lnc-bbut00014188"	exon_number "3"
14	lncRNA	exon	73201658	73201761	.	+	.	gene_id "lnc-bbug05935"	transcript_id "lnc-bbut00014188"	exon_number "4"
14	lncRNA	exon	73201928	73205078	.	+	.	gene_id "lnc-bbug05935"	transcript_id "lnc-bbut00014188"	exon_number "5"
14	lncRNA	exon	74293851	74293864	.	+	.	gene_id "lnc-bbug05937"	transcript_id "lnc-bbut00014197"	exon_number "1"
14	lncRNA	exon	74299187	74299189	.	+	.	gene_id "lnc-bbug05937"	transcript_id "lnc-bbut00014197"	exon_number "2"
14	lncRNA	exon	74311246	74311659	.	+	.	gene_id "lnc-bbug05937"	transcript_id "lnc-bbut00014197"	exon_number "3"
14	lncRNA	exon	77146861	77147622	.	+	.	gene_id "lnc-bbug05942"	transcript_id "lnc-bbut00014213"	exon_number "1"
14	lncRNA	exon	77147785	77147817	.	+	.	gene_id "lnc-bbug05942"	transcript_id "lnc-bbut00014213"	exon_number "2"
14	lncRNA	exon	77150694	77150745	.	+	.	gene_id "lnc-bbug05942"	transcript_id "lnc-bbut00014213"	exon_number "3"
14	lncRNA	exon	77250788	77250895	.	+	.	gene_id "lnc-bbug05943"	transcript_id "lnc-bbut00014214"	exon_number "1"
14	lncRNA	exon	77251824	77251933	.	+	.	gene_id "lnc-bbug05943"	transcript_id "lnc-bbut00014214"	exon_number "2"
14	lncRNA	exon	77278533	77279066	.	+	.	gene_id "lnc-bbug05943"	transcript_id "lnc-bbut00014214"	exon_number "3"
14	lncRNA	exon	77250793	77250922	.	+	.	gene_id "lnc-bbug05943"	transcript_id "lnc-bbut00014215"	exon_number "1"
14	lncRNA	exon	77251824	77251933	.	+	.	gene_id "lnc-bbug05943"	transcript_id "lnc-bbut00014215"	exon_number "2"
14	lncRNA	exon	77278533	77279066	.	+	.	gene_id "lnc-bbug05943"	transcript_id "lnc-bbut00014215"	exon_number "3"
14	lncRNA	exon	80713979	80714713	.	+	.	gene_id "lnc-bbug05947"	transcript_id "lnc-bbut00014227"	exon_number "1"
14	lncRNA	exon	81582446	81584379	.	+	.	gene_id "lnc-bbug05948"	transcript_id "lnc-bbut00014228"	exon_number "1"
14	lncRNA	exon	81589547	81589646	.	+	.	gene_id "lnc-bbug05948"	transcript_id "lnc-bbut00014228"	exon_number "2"
14	lncRNA	exon	81605836	81607352	.	+	.	gene_id "lnc-bbug05948"	transcript_id "lnc-bbut00014228"	exon_number "3"
14	lncRNA	exon	82595445	82595579	.	+	.	gene_id "lnc-bbug05949"	transcript_id "lnc-bbut00014229"	exon_number "1"
14	lncRNA	exon	82602171	82602420	.	+	.	gene_id "lnc-bbug05949"	transcript_id "lnc-bbut00014229"	exon_number "2"
14	lncRNA	exon	82616319	82616475	.	+	.	gene_id "lnc-bbug05949"	transcript_id "lnc-bbut00014229"	exon_number "3"
14	lncRNA	exon	82937574	82938486	.	+	.	gene_id "lnc-bbug05951"	transcript_id "lnc-bbut00014232"	exon_number "1"
14	lncRNA	exon	83244229	83245416	.	+	.	gene_id "lnc-bbug05957"	transcript_id "lnc-bbut00014238"	exon_number "1"
14	lncRNA	exon	83249151	83249303	.	+	.	gene_id "lnc-bbug05957"	transcript_id "lnc-bbut00014238"	exon_number "2"
14	lncRNA	exon	83249466	83249625	.	+	.	gene_id "lnc-bbug05957"	transcript_id "lnc-bbut00014238"	exon_number "3"
14	lncRNA	exon	83253937	83254043	.	+	.	gene_id "lnc-bbug05957"	transcript_id "lnc-bbut00014238"	exon_number "4"
14	lncRNA	exon	83262991	83263060	.	+	.	gene_id "lnc-bbug05957"	transcript_id "lnc-bbut00014238"	exon_number "5"
14	lncRNA	exon	83266159	83266351	.	+	.	gene_id "lnc-bbug05957"	transcript_id "lnc-bbut00014238"	exon_number "6"
14	lncRNA	exon	83278312	83278408	.	+	.	gene_id "lnc-bbug05957"	transcript_id "lnc-bbut00014238"	exon_number "7"
14	lncRNA	exon	83279137	83281552	.	+	.	gene_id "lnc-bbug05957"	transcript_id "lnc-bbut00014238"	exon_number "8"
14	lncRNA	exon	83248284	83249303	.	+	.	gene_id "lnc-bbug05957"	transcript_id "lnc-bbut00014242"	exon_number "1"
14	lncRNA	exon	83249466	83249625	.	+	.	gene_id "lnc-bbug05957"	transcript_id "lnc-bbut00014242"	exon_number "2"
14	lncRNA	exon	83253937	83254043	.	+	.	gene_id "lnc-bbug05957"	transcript_id "lnc-bbut00014242"	exon_number "3"
14	lncRNA	exon	83259018	83259082	.	+	.	gene_id "lnc-bbug05957"	transcript_id "lnc-bbut00014242"	exon_number "4"
14	lncRNA	exon	83262991	83263060	.	+	.	gene_id "lnc-bbug05957"	transcript_id "lnc-bbut00014242"	exon_number "5"
14	lncRNA	exon	83266159	83266351	.	+	.	gene_id "lnc-bbug05957"	transcript_id "lnc-bbut00014242"	exon_number "6"
14	lncRNA	exon	83278312	83278408	.	+	.	gene_id "lnc-bbug05957"	transcript_id "lnc-bbut00014242"	exon_number "7"
14	lncRNA	exon	83279137	83281552	.	+	.	gene_id "lnc-bbug05957"	transcript_id "lnc-bbut00014242"	exon_number "8"
14	lncRNA	exon	83248284	83249303	.	+	.	gene_id "lnc-bbug05957"	transcript_id "lnc-bbut00014241"	exon_number "1"
14	lncRNA	exon	83249466	83249625	.	+	.	gene_id "lnc-bbug05957"	transcript_id "lnc-bbut00014241"	exon_number "2"
14	lncRNA	exon	83253937	83254043	.	+	.	gene_id "lnc-bbug05957"	transcript_id "lnc-bbut00014241"	exon_number "3"
14	lncRNA	exon	83262991	83263060	.	+	.	gene_id "lnc-bbug05957"	transcript_id "lnc-bbut00014241"	exon_number "4"
14	lncRNA	exon	83266159	83266351	.	+	.	gene_id "lnc-bbug05957"	transcript_id "lnc-bbut00014241"	exon_number "5"
14	lncRNA	exon	83278315	83278408	.	+	.	gene_id "lnc-bbug05957"	transcript_id "lnc-bbut00014241"	exon_number "6"

14	lncRNA	exon	83279137	83281552	.	+	.	gene_id "lnc-bbug05957"	transcript_id "lnc-bbut00014241"	exon_number "7"
14	lncRNA	exon	83248284	83249303	.	+	.	gene_id "lnc-bbug05957"	transcript_id "lnc-bbut00014240"	exon_number "1"
14	lncRNA	exon	83249466	83249625	.	+	.	gene_id "lnc-bbug05957"	transcript_id "lnc-bbut00014240"	exon_number "2"
14	lncRNA	exon	83253937	83254043	.	+	.	gene_id "lnc-bbug05957"	transcript_id "lnc-bbut00014240"	exon_number "3"
14	lncRNA	exon	83262991	83263060	.	+	.	gene_id "lnc-bbug05957"	transcript_id "lnc-bbut00014240"	exon_number "4"
14	lncRNA	exon	83266159	83266351	.	+	.	gene_id "lnc-bbug05957"	transcript_id "lnc-bbut00014240"	exon_number "5"
14	lncRNA	exon	83278312	83278408	.	+	.	gene_id "lnc-bbug05957"	transcript_id "lnc-bbut00014240"	exon_number "6"
14	lncRNA	exon	83279140	83281552	.	+	.	gene_id "lnc-bbug05957"	transcript_id "lnc-bbut00014240"	exon_number "7"
14	lncRNA	exon	83248284	83249303	.	+	.	gene_id "lnc-bbug05957"	transcript_id "lnc-bbut00014239"	exon_number "1"
14	lncRNA	exon	83249466	83249625	.	+	.	gene_id "lnc-bbug05957"	transcript_id "lnc-bbut00014239"	exon_number "2"
14	lncRNA	exon	83253937	83254043	.	+	.	gene_id "lnc-bbug05957"	transcript_id "lnc-bbut00014239"	exon_number "3"
14	lncRNA	exon	83262991	83263060	.	+	.	gene_id "lnc-bbug05957"	transcript_id "lnc-bbut00014239"	exon_number "4"
14	lncRNA	exon	83266159	83266351	.	+	.	gene_id "lnc-bbug05957"	transcript_id "lnc-bbut00014239"	exon_number "5"
14	lncRNA	exon	83278312	83278408	.	+	.	gene_id "lnc-bbug05957"	transcript_id "lnc-bbut00014239"	exon_number "6"
14	lncRNA	exon	83279137	83281552	.	+	.	gene_id "lnc-bbug05957"	transcript_id "lnc-bbut00014239"	exon_number "7"
14	lncRNA	exon	83345614	83345671	.	+	.	gene_id "lnc-bbug05958"	transcript_id "lnc-bbut00014243"	exon_number "1"
14	lncRNA	exon	83354167	83354290	.	+	.	gene_id "lnc-bbug05958"	transcript_id "lnc-bbut00014243"	exon_number "2"
14	lncRNA	exon	83356260	83356366	.	+	.	gene_id "lnc-bbug05958"	transcript_id "lnc-bbut00014243"	exon_number "3"
14	lncRNA	exon	83356708	83359322	.	+	.	gene_id "lnc-bbug05958"	transcript_id "lnc-bbut00014243"	exon_number "4"
14	lncRNA	exon	83350388	83350446	.	+	.	gene_id "lnc-bbug05958"	transcript_id "lnc-bbut00014244"	exon_number "1"
14	lncRNA	exon	83354167	83354290	.	+	.	gene_id "lnc-bbug05958"	transcript_id "lnc-bbut00014244"	exon_number "2"
14	lncRNA	exon	83356260	83356366	.	+	.	gene_id "lnc-bbug05958"	transcript_id "lnc-bbut00014244"	exon_number "3"
14	lncRNA	exon	83356708	83359322	.	+	.	gene_id "lnc-bbug05958"	transcript_id "lnc-bbut00014244"	exon_number "4"
14	lncRNA	exon	83354028	83354290	.	+	.	gene_id "lnc-bbug05958"	transcript_id "lnc-bbut00014245"	exon_number "1"
14	lncRNA	exon	83356260	83356366	.	+	.	gene_id "lnc-bbug05958"	transcript_id "lnc-bbut00014245"	exon_number "2"
14	lncRNA	exon	83356708	83356763	.	+	.	gene_id "lnc-bbug05958"	transcript_id "lnc-bbut00014245"	exon_number "3"
14	lncRNA	exon	83360319	83360353	.	+	.	gene_id "lnc-bbug05958"	transcript_id "lnc-bbut00014245"	exon_number "4"
14	lncRNA	exon	1267116	1267888	.	-	.	gene_id "lnc-bbug05962"	transcript_id "lnc-bbut00014259"	exon_number "1"
14	lncRNA	exon	1276005	1276127	.	-	.	gene_id "lnc-bbug05962"	transcript_id "lnc-bbut00014259"	exon_number "2"
14	lncRNA	exon	1276541	1277569	.	-	.	gene_id "lnc-bbug05962"	transcript_id "lnc-bbut00014259"	exon_number "3"
14	lncRNA	exon	1845482	1845589	.	-	.	gene_id "lnc-bbug05964"	transcript_id "lnc-bbut00014263"	exon_number "1"
14	lncRNA	exon	1854857	1856683	.	-	.	gene_id "lnc-bbug05964"	transcript_id "lnc-bbut00014263"	exon_number "2"
14	lncRNA	exon	1857094	1857560	.	-	.	gene_id "lnc-bbug05965"	transcript_id "lnc-bbut00014264"	exon_number "1"
14	lncRNA	exon	1858296	1859775	.	-	.	gene_id "lnc-bbug05965"	transcript_id "lnc-bbut00014264"	exon_number "2"
14	lncRNA	exon	1866778	1867043	.	-	.	gene_id "lnc-bbug05965"	transcript_id "lnc-bbut00014264"	exon_number "3"
14	lncRNA	exon	1908540	1910923	.	-	.	gene_id "lnc-bbug05966"	transcript_id "lnc-bbut00014265"	exon_number "1"
14	lncRNA	exon	1930434	1930752	.	-	.	gene_id "lnc-bbug05966"	transcript_id "lnc-bbut00014265"	exon_number "2"
14	lncRNA	exon	1917460	1922605	.	-	.	gene_id "lnc-bbug05967"	transcript_id "lnc-bbut00014266"	exon_number "1"
14	lncRNA	exon	1924995	1928547	.	-	.	gene_id "lnc-bbug05967"	transcript_id "lnc-bbut00014266"	exon_number "2"
14	lncRNA	exon	2062975	2065427	.	-	.	gene_id "lnc-bbug05968"	transcript_id "lnc-bbut00014267"	exon_number "1"
14	lncRNA	exon	2070829	2070935	.	-	.	gene_id "lnc-bbug05968"	transcript_id "lnc-bbut00014267"	exon_number "2"
14	lncRNA	exon	2127904	2128099	.	-	.	gene_id "lnc-bbug05969"	transcript_id "lnc-bbut00014268"	exon_number "1"
14	lncRNA	exon	2128283	2128332	.	-	.	gene_id "lnc-bbug05969"	transcript_id "lnc-bbut00014268"	exon_number "2"
14	lncRNA	exon	3960077	3960325	.	-	.	gene_id "lnc-bbug05974"	transcript_id "lnc-bbut00014273"	exon_number "1"
14	lncRNA	exon	3960693	3961002	.	-	.	gene_id "lnc-bbug05974"	transcript_id "lnc-bbut00014273"	exon_number "2"
14	lncRNA	exon	4277126	4278229	.	-	.	gene_id "lnc-bbug05975"	transcript_id "lnc-bbut00014274"	exon_number "1"
14	lncRNA	exon	4278341	4278531	.	-	.	gene_id "lnc-bbug05975"	transcript_id "lnc-bbut00014274"	exon_number "2"
14	lncRNA	exon	4278630	4279357	.	-	.	gene_id "lnc-bbug05975"	transcript_id "lnc-bbut00014274"	exon_number "3"
14	lncRNA	exon	4565848	4569171	.	-	.	gene_id "lnc-bbug05976"	transcript_id "lnc-bbut00014275"	exon_number "1"
14	lncRNA	exon	4570836	4570898	.	-	.	gene_id "lnc-bbug05976"	transcript_id "lnc-bbut00014275"	exon_number "2"
14	lncRNA	exon	4571935	4572045	.	-	.	gene_id "lnc-bbug05976"	transcript_id "lnc-bbut00014275"	exon_number "3"
14	lncRNA	exon	4978807	4983299	.	-	.	gene_id "lnc-bbug05981"	transcript_id "lnc-bbut00014283"	exon_number "1"

14	lncRNA	exon	4984711	4985129	.	-	.	gene_id "lnc-bbug05981"	transcript_id "lnc-bbut00014283"	exon_number "2"
14	lncRNA	exon	5052999	5057057	.	-	.	gene_id "lnc-bbug05982"	transcript_id "lnc-bbut00014284"	exon_number "1"
14	lncRNA	exon	5063055	5063134	.	-	.	gene_id "lnc-bbug05982"	transcript_id "lnc-bbut00014284"	exon_number "2"
14	lncRNA	exon	5065620	5065692	.	-	.	gene_id "lnc-bbug05982"	transcript_id "lnc-bbut00014284"	exon_number "3"
14	lncRNA	exon	5065844	5066605	.	-	.	gene_id "lnc-bbug05982"	transcript_id "lnc-bbut00014284"	exon_number "4"
14	lncRNA	exon	5052999	5057057	.	-	.	gene_id "lnc-bbug05982"	transcript_id "lnc-bbut00014286"	exon_number "1"
14	lncRNA	exon	5063055	5063134	.	-	.	gene_id "lnc-bbug05982"	transcript_id "lnc-bbut00014286"	exon_number "2"
14	lncRNA	exon	5065620	5065692	.	-	.	gene_id "lnc-bbug05982"	transcript_id "lnc-bbut00014286"	exon_number "3"
14	lncRNA	exon	5067593	5068505	.	-	.	gene_id "lnc-bbug05982"	transcript_id "lnc-bbut00014286"	exon_number "4"
14	lncRNA	exon	5052999	5057057	.	-	.	gene_id "lnc-bbug05982"	transcript_id "lnc-bbut00014285"	exon_number "1"
14	lncRNA	exon	5063055	5063134	.	-	.	gene_id "lnc-bbug05982"	transcript_id "lnc-bbut00014285"	exon_number "2"
14	lncRNA	exon	5065620	5065692	.	-	.	gene_id "lnc-bbug05982"	transcript_id "lnc-bbut00014285"	exon_number "3"
14	lncRNA	exon	5067581	5068505	.	-	.	gene_id "lnc-bbug05982"	transcript_id "lnc-bbut00014285"	exon_number "4"
14	lncRNA	exon	5074375	5075021	.	-	.	gene_id "lnc-bbug05983"	transcript_id "lnc-bbut00014287"	exon_number "1"
14	lncRNA	exon	5077413	5079194	.	-	.	gene_id "lnc-bbug05983"	transcript_id "lnc-bbut00014287"	exon_number "2"
14	lncRNA	exon	5086480	5087418	.	-	.	gene_id "lnc-bbug05984"	transcript_id "lnc-bbut00014290"	exon_number "1"
14	lncRNA	exon	5088187	5088256	.	-	.	gene_id "lnc-bbug05984"	transcript_id "lnc-bbut00014290"	exon_number "2"
14	lncRNA	exon	5089586	5089663	.	-	.	gene_id "lnc-bbug05984"	transcript_id "lnc-bbut00014290"	exon_number "3"
14	lncRNA	exon	5169502	5171525	.	-	.	gene_id "lnc-bbug05984"	transcript_id "lnc-bbut00014290"	exon_number "4"
14	lncRNA	exon	5173818	5173975	.	-	.	gene_id "lnc-bbug05984"	transcript_id "lnc-bbut00014290"	exon_number "5"
14	lncRNA	exon	5174076	5175087	.	-	.	gene_id "lnc-bbug05984"	transcript_id "lnc-bbut00014290"	exon_number "6"
14	lncRNA	exon	5086480	5087418	.	-	.	gene_id "lnc-bbug05984"	transcript_id "lnc-bbut00014289"	exon_number "1"
14	lncRNA	exon	5088187	5088256	.	-	.	gene_id "lnc-bbug05984"	transcript_id "lnc-bbut00014289"	exon_number "2"
14	lncRNA	exon	5089586	5089663	.	-	.	gene_id "lnc-bbug05984"	transcript_id "lnc-bbut00014289"	exon_number "3"
14	lncRNA	exon	5169502	5171525	.	-	.	gene_id "lnc-bbug05984"	transcript_id "lnc-bbut00014289"	exon_number "4"
14	lncRNA	exon	5173818	5173971	.	-	.	gene_id "lnc-bbug05984"	transcript_id "lnc-bbut00014289"	exon_number "5"
14	lncRNA	exon	5174076	5175087	.	-	.	gene_id "lnc-bbug05984"	transcript_id "lnc-bbut00014289"	exon_number "6"
14	lncRNA	exon	5129329	5129373	.	-	.	gene_id "lnc-bbug05985"	transcript_id "lnc-bbut00014291"	exon_number "1"
14	lncRNA	exon	5134601	5134745	.	-	.	gene_id "lnc-bbug05985"	transcript_id "lnc-bbut00014291"	exon_number "2"
14	lncRNA	exon	5138271	5139487	.	-	.	gene_id "lnc-bbug05985"	transcript_id "lnc-bbut00014291"	exon_number "3"
14	lncRNA	exon	5187345	5187460	.	-	.	gene_id "lnc-bbug05986"	transcript_id "lnc-bbut00014292"	exon_number "1"
14	lncRNA	exon	5187939	5188320	.	-	.	gene_id "lnc-bbug05986"	transcript_id "lnc-bbut00014292"	exon_number "2"
14	lncRNA	exon	5213960	5218676	.	-	.	gene_id "lnc-bbug05988"	transcript_id "lnc-bbut00014295"	exon_number "1"
14	lncRNA	exon	5225841	5226398	.	-	.	gene_id "lnc-bbug05988"	transcript_id "lnc-bbut00014295"	exon_number "2"
14	lncRNA	exon	5226720	5226968	.	-	.	gene_id "lnc-bbug05988"	transcript_id "lnc-bbut00014295"	exon_number "3"
14	lncRNA	exon	5259279	5259574	.	-	.	gene_id "lnc-bbug05989"	transcript_id "lnc-bbut00014296"	exon_number "1"
14	lncRNA	exon	5265784	5267304	.	-	.	gene_id "lnc-bbug05989"	transcript_id "lnc-bbut00014296"	exon_number "2"
14	lncRNA	exon	5283876	5284088	.	-	.	gene_id "lnc-bbug05990"	transcript_id "lnc-bbut00014297"	exon_number "1"
14	lncRNA	exon	5284636	5286535	.	-	.	gene_id "lnc-bbug05990"	transcript_id "lnc-bbut00014297"	exon_number "2"
14	lncRNA	exon	5497427	5497516	.	-	.	gene_id "lnc-bbug05994"	transcript_id "lnc-bbut00014302"	exon_number "1"
14	lncRNA	exon	5507432	5507767	.	-	.	gene_id "lnc-bbug05994"	transcript_id "lnc-bbut00014302"	exon_number "2"
14	lncRNA	exon	5519534	5521920	.	-	.	gene_id "lnc-bbug05995"	transcript_id "lnc-bbut00014303"	exon_number "1"
14	lncRNA	exon	5525969	5526041	.	-	.	gene_id "lnc-bbug05995"	transcript_id "lnc-bbut00014303"	exon_number "2"
14	lncRNA	exon	5527510	5527533	.	-	.	gene_id "lnc-bbug05995"	transcript_id "lnc-bbut00014303"	exon_number "3"
14	lncRNA	exon	5986914	5988777	.	-	.	gene_id "lnc-bbug05997"	transcript_id "lnc-bbut00014308"	exon_number "1"
14	lncRNA	exon	5989557	5989841	.	-	.	gene_id "lnc-bbug05997"	transcript_id "lnc-bbut00014308"	exon_number "2"
14	lncRNA	exon	5992250	5992434	.	-	.	gene_id "lnc-bbug05997"	transcript_id "lnc-bbut00014308"	exon_number "3"
14	lncRNA	exon	5992886	5993242	.	-	.	gene_id "lnc-bbug05997"	transcript_id "lnc-bbut00014308"	exon_number "4"
14	lncRNA	exon	5995711	5995883	.	-	.	gene_id "lnc-bbug05997"	transcript_id "lnc-bbut00014308"	exon_number "5"
14	lncRNA	exon	5996746	5996841	.	-	.	gene_id "lnc-bbug05997"	transcript_id "lnc-bbut00014308"	exon_number "6"
14	lncRNA	exon	5997560	5998241	.	-	.	gene_id "lnc-bbug05997"	transcript_id "lnc-bbut00014308"	exon_number "7"
14	lncRNA	exon	5986914	5988777	.	-	.	gene_id "lnc-bbug05997"	transcript_id "lnc-bbut00014309"	exon_number "1"

14	lncRNA	exon	5989557	5989841	.	-	.	gene_id "lnc-bbug05997"	transcript_id "lnc-bbut00014309"	exon_number "2"
14	lncRNA	exon	5992250	5992434	.	-	.	gene_id "lnc-bbug05997"	transcript_id "lnc-bbut00014309"	exon_number "3"
14	lncRNA	exon	5992982	5993242	.	-	.	gene_id "lnc-bbug05997"	transcript_id "lnc-bbut00014309"	exon_number "4"
14	lncRNA	exon	5995711	5995883	.	-	.	gene_id "lnc-bbug05997"	transcript_id "lnc-bbut00014309"	exon_number "5"
14	lncRNA	exon	5996746	5996841	.	-	.	gene_id "lnc-bbug05997"	transcript_id "lnc-bbut00014309"	exon_number "6"
14	lncRNA	exon	5997560	5998241	.	-	.	gene_id "lnc-bbug05997"	transcript_id "lnc-bbut00014309"	exon_number "7"
14	lncRNA	exon	5986914	5988777	.	-	.	gene_id "lnc-bbug05997"	transcript_id "lnc-bbut00014307"	exon_number "1"
14	lncRNA	exon	5989557	5989841	.	-	.	gene_id "lnc-bbug05997"	transcript_id "lnc-bbut00014307"	exon_number "2"
14	lncRNA	exon	5992250	5993242	.	-	.	gene_id "lnc-bbug05997"	transcript_id "lnc-bbut00014307"	exon_number "3"
14	lncRNA	exon	5995711	5995883	.	-	.	gene_id "lnc-bbug05997"	transcript_id "lnc-bbut00014307"	exon_number "4"
14	lncRNA	exon	5996746	5996841	.	-	.	gene_id "lnc-bbug05997"	transcript_id "lnc-bbut00014307"	exon_number "5"
14	lncRNA	exon	5997586	5998241	.	-	.	gene_id "lnc-bbug05997"	transcript_id "lnc-bbut00014307"	exon_number "6"
14	lncRNA	exon	5986914	5988777	.	-	.	gene_id "lnc-bbug05997"	transcript_id "lnc-bbut00014306"	exon_number "1"
14	lncRNA	exon	5989557	5989841	.	-	.	gene_id "lnc-bbug05997"	transcript_id "lnc-bbut00014306"	exon_number "2"
14	lncRNA	exon	5992250	5993242	.	-	.	gene_id "lnc-bbug05997"	transcript_id "lnc-bbut00014306"	exon_number "3"
14	lncRNA	exon	5995711	5995883	.	-	.	gene_id "lnc-bbug05997"	transcript_id "lnc-bbut00014306"	exon_number "4"
14	lncRNA	exon	5996746	5996841	.	-	.	gene_id "lnc-bbug05997"	transcript_id "lnc-bbut00014306"	exon_number "5"
14	lncRNA	exon	5997560	5998241	.	-	.	gene_id "lnc-bbug05997"	transcript_id "lnc-bbut00014306"	exon_number "6"
14	lncRNA	exon	5986914	5988777	.	-	.	gene_id "lnc-bbug05997"	transcript_id "lnc-bbut00014310"	exon_number "1"
14	lncRNA	exon	5989557	5989841	.	-	.	gene_id "lnc-bbug05997"	transcript_id "lnc-bbut00014310"	exon_number "2"
14	lncRNA	exon	5992250	5993242	.	-	.	gene_id "lnc-bbug05997"	transcript_id "lnc-bbut00014310"	exon_number "3"
14	lncRNA	exon	5995711	5995883	.	-	.	gene_id "lnc-bbug05997"	transcript_id "lnc-bbut00014310"	exon_number "4"
14	lncRNA	exon	5996746	5996841	.	-	.	gene_id "lnc-bbug05997"	transcript_id "lnc-bbut00014310"	exon_number "5"
14	lncRNA	exon	5998434	5999381	.	-	.	gene_id "lnc-bbug05997"	transcript_id "lnc-bbut00014310"	exon_number "6"
14	lncRNA	exon	6322420	6322992	.	-	.	gene_id "lnc-bbug06003"	transcript_id "lnc-bbut00014316"	exon_number "1"
14	lncRNA	exon	8222275	8224723	.	-	.	gene_id "lnc-bbug06010"	transcript_id "lnc-bbut00014334"	exon_number "1"
14	lncRNA	exon	8224992	8225375	.	-	.	gene_id "lnc-bbug06010"	transcript_id "lnc-bbut00014334"	exon_number "2"
14	lncRNA	exon	8230458	8230551	.	-	.	gene_id "lnc-bbug06010"	transcript_id "lnc-bbut00014334"	exon_number "3"
14	lncRNA	exon	8237856	8238612	.	-	.	gene_id "lnc-bbug06010"	transcript_id "lnc-bbut00014334"	exon_number "4"
14	lncRNA	exon	8222275	8224723	.	-	.	gene_id "lnc-bbug06010"	transcript_id "lnc-bbut00014333"	exon_number "1"
14	lncRNA	exon	8224992	8225375	.	-	.	gene_id "lnc-bbug06010"	transcript_id "lnc-bbut00014333"	exon_number "2"
14	lncRNA	exon	8230458	8230551	.	-	.	gene_id "lnc-bbug06010"	transcript_id "lnc-bbut00014333"	exon_number "3"
14	lncRNA	exon	8237835	8238612	.	-	.	gene_id "lnc-bbug06010"	transcript_id "lnc-bbut00014333"	exon_number "4"
14	lncRNA	exon	8791821	8792795	.	-	.	gene_id "lnc-bbug06016"	transcript_id "lnc-bbut00014345"	exon_number "1"
14	lncRNA	exon	8798605	8799155	.	-	.	gene_id "lnc-bbug06016"	transcript_id "lnc-bbut00014345"	exon_number "2"
14	lncRNA	exon	8791821	8792792	.	-	.	gene_id "lnc-bbug06016"	transcript_id "lnc-bbut00014344"	exon_number "1"
14	lncRNA	exon	8798605	8799155	.	-	.	gene_id "lnc-bbug06016"	transcript_id "lnc-bbut00014344"	exon_number "2"
14	lncRNA	exon	8799629	8800015	.	-	.	gene_id "lnc-bbug06017"	transcript_id "lnc-bbut00014346"	exon_number "1"
14	lncRNA	exon	8801769	8802018	.	-	.	gene_id "lnc-bbug06017"	transcript_id "lnc-bbut00014346"	exon_number "2"
14	lncRNA	exon	9004197	9004370	.	-	.	gene_id "lnc-bbug06027"	transcript_id "lnc-bbut00014365"	exon_number "1"
14	lncRNA	exon	9005269	9005443	.	-	.	gene_id "lnc-bbug06027"	transcript_id "lnc-bbut00014365"	exon_number "2"
14	lncRNA	exon	9008042	9008174	.	-	.	gene_id "lnc-bbug06027"	transcript_id "lnc-bbut00014365"	exon_number "3"
14	lncRNA	exon	9030734	9031057	.	-	.	gene_id "lnc-bbug06028"	transcript_id "lnc-bbut00014366"	exon_number "1"
14	lncRNA	exon	9034418	9034482	.	-	.	gene_id "lnc-bbug06028"	transcript_id "lnc-bbut00014366"	exon_number "2"
14	lncRNA	exon	9035946	9036096	.	-	.	gene_id "lnc-bbug06028"	transcript_id "lnc-bbut00014366"	exon_number "3"
14	lncRNA	exon	9038666	9038825	.	-	.	gene_id "lnc-bbug06028"	transcript_id "lnc-bbut00014366"	exon_number "4"
14	lncRNA	exon	9044178	9044294	.	-	.	gene_id "lnc-bbug06029"	transcript_id "lnc-bbut00014367"	exon_number "1"
14	lncRNA	exon	9044824	9044965	.	-	.	gene_id "lnc-bbug06029"	transcript_id "lnc-bbut00014367"	exon_number "2"
14	lncRNA	exon	9046257	9046409	.	-	.	gene_id "lnc-bbug06029"	transcript_id "lnc-bbut00014367"	exon_number "3"
14	lncRNA	exon	9050793	9050900	.	-	.	gene_id "lnc-bbug06029"	transcript_id "lnc-bbut00014367"	exon_number "4"
14	lncRNA	exon	9169249	9169385	.	-	.	gene_id "lnc-bbug06032"	transcript_id "lnc-bbut00014370"	exon_number "1"
14	lncRNA	exon	9179697	9179888	.	-	.	gene_id "lnc-bbug06032"	transcript_id "lnc-bbut00014370"	exon_number "2"

14	lncRNA	exon	9191333	9191446	.	-	.	gene_id "lnc-bbug06032"	transcript_id "lnc-bbut00014370"	exon_number "3"
14	lncRNA	exon	9222232	9222808	.	-	.	gene_id "lnc-bbug06033"	transcript_id "lnc-bbut00014371"	exon_number "1"
14	lncRNA	exon	9223258	9223449	.	-	.	gene_id "lnc-bbug06033"	transcript_id "lnc-bbut00014371"	exon_number "2"
14	lncRNA	exon	9226221	9226448	.	-	.	gene_id "lnc-bbug06033"	transcript_id "lnc-bbut00014371"	exon_number "3"
14	lncRNA	exon	9825120	9825251	.	-	.	gene_id "lnc-bbug06041"	transcript_id "lnc-bbut00014380"	exon_number "1"
14	lncRNA	exon	9829568	9829694	.	-	.	gene_id "lnc-bbug06041"	transcript_id "lnc-bbut00014380"	exon_number "2"
14	lncRNA	exon	9874361	9874467	.	-	.	gene_id "lnc-bbug06043"	transcript_id "lnc-bbut00014386"	exon_number "1"
14	lncRNA	exon	9904194	9904261	.	-	.	gene_id "lnc-bbug06043"	transcript_id "lnc-bbut00014386"	exon_number "2"
14	lncRNA	exon	9905720	9905961	.	-	.	gene_id "lnc-bbug06043"	transcript_id "lnc-bbut00014386"	exon_number "3"
14	lncRNA	exon	9906177	9906358	.	-	.	gene_id "lnc-bbug06043"	transcript_id "lnc-bbut00014386"	exon_number "4"
14	lncRNA	exon	9888517	9889576	.	-	.	gene_id "lnc-bbug06043"	transcript_id "lnc-bbut00014388"	exon_number "1"
14	lncRNA	exon	9890104	9890381	.	-	.	gene_id "lnc-bbug06043"	transcript_id "lnc-bbut00014388"	exon_number "2"
14	lncRNA	exon	9904194	9904261	.	-	.	gene_id "lnc-bbug06043"	transcript_id "lnc-bbut00014388"	exon_number "3"
14	lncRNA	exon	9905720	9905961	.	-	.	gene_id "lnc-bbug06043"	transcript_id "lnc-bbut00014388"	exon_number "4"
14	lncRNA	exon	9906177	9906358	.	-	.	gene_id "lnc-bbug06043"	transcript_id "lnc-bbut00014388"	exon_number "5"
14	lncRNA	exon	9888517	9889576	.	-	.	gene_id "lnc-bbug06043"	transcript_id "lnc-bbut00014387"	exon_number "1"
14	lncRNA	exon	9904194	9904261	.	-	.	gene_id "lnc-bbug06043"	transcript_id "lnc-bbut00014387"	exon_number "2"
14	lncRNA	exon	9905720	9905961	.	-	.	gene_id "lnc-bbug06043"	transcript_id "lnc-bbut00014387"	exon_number "3"
14	lncRNA	exon	9906177	9906358	.	-	.	gene_id "lnc-bbug06043"	transcript_id "lnc-bbut00014387"	exon_number "4"
14	lncRNA	exon	9891813	9892929	.	-	.	gene_id "lnc-bbug06043"	transcript_id "lnc-bbut00014390"	exon_number "1"
14	lncRNA	exon	9904194	9904261	.	-	.	gene_id "lnc-bbug06043"	transcript_id "lnc-bbut00014390"	exon_number "2"
14	lncRNA	exon	9905720	9905961	.	-	.	gene_id "lnc-bbug06043"	transcript_id "lnc-bbut00014390"	exon_number "3"
14	lncRNA	exon	9906177	9906358	.	-	.	gene_id "lnc-bbug06043"	transcript_id "lnc-bbut00014390"	exon_number "4"
14	lncRNA	exon	9901765	9903088	.	-	.	gene_id "lnc-bbug06043"	transcript_id "lnc-bbut00014392"	exon_number "1"
14	lncRNA	exon	9904194	9904261	.	-	.	gene_id "lnc-bbug06043"	transcript_id "lnc-bbut00014392"	exon_number "2"
14	lncRNA	exon	9905720	9905961	.	-	.	gene_id "lnc-bbug06043"	transcript_id "lnc-bbut00014392"	exon_number "3"
14	lncRNA	exon	9906177	9906357	.	-	.	gene_id "lnc-bbug06043"	transcript_id "lnc-bbut00014392"	exon_number "4"
14	lncRNA	exon	10017605	10017861	.	-	.	gene_id "lnc-bbug06045"	transcript_id "lnc-bbut00014397"	exon_number "1"
14	lncRNA	exon	10018607	10018756	.	-	.	gene_id "lnc-bbug06045"	transcript_id "lnc-bbut00014397"	exon_number "2"
14	lncRNA	exon	10019173	10019334	.	-	.	gene_id "lnc-bbug06045"	transcript_id "lnc-bbut00014397"	exon_number "3"
14	lncRNA	exon	10020236	10020396	.	-	.	gene_id "lnc-bbug06045"	transcript_id "lnc-bbut00014397"	exon_number "4"
14	lncRNA	exon	10063463	10063656	.	-	.	gene_id "lnc-bbug06047"	transcript_id "lnc-bbut00014399"	exon_number "1"
14	lncRNA	exon	10064507	10064586	.	-	.	gene_id "lnc-bbug06047"	transcript_id "lnc-bbut00014399"	exon_number "2"
14	lncRNA	exon	10064793	10064987	.	-	.	gene_id "lnc-bbug06047"	transcript_id "lnc-bbut00014399"	exon_number "3"
14	lncRNA	exon	10067304	10067346	.	-	.	gene_id "lnc-bbug06047"	transcript_id "lnc-bbut00014399"	exon_number "4"
14	lncRNA	exon	10620851	10621596	.	-	.	gene_id "lnc-bbug06058"	transcript_id "lnc-bbut00014422"	exon_number "1"
14	lncRNA	exon	10625041	10625214	.	-	.	gene_id "lnc-bbug06058"	transcript_id "lnc-bbut00014422"	exon_number "2"
14	lncRNA	exon	10637206	10637313	.	-	.	gene_id "lnc-bbug06058"	transcript_id "lnc-bbut00014422"	exon_number "3"
14	lncRNA	exon	10620851	10621596	.	-	.	gene_id "lnc-bbug06058"	transcript_id "lnc-bbut00014423"	exon_number "1"
14	lncRNA	exon	10625041	10625214	.	-	.	gene_id "lnc-bbug06058"	transcript_id "lnc-bbut00014423"	exon_number "2"
14	lncRNA	exon	10645368	10645437	.	-	.	gene_id "lnc-bbug06058"	transcript_id "lnc-bbut00014423"	exon_number "3"
14	lncRNA	exon	10652449	10652559	.	-	.	gene_id "lnc-bbug06058"	transcript_id "lnc-bbut00014423"	exon_number "4"
14	lncRNA	exon	10620851	10621596	.	-	.	gene_id "lnc-bbug06058"	transcript_id "lnc-bbut00014424"	exon_number "1"
14	lncRNA	exon	10625041	10625214	.	-	.	gene_id "lnc-bbug06058"	transcript_id "lnc-bbut00014424"	exon_number "2"
14	lncRNA	exon	10645368	10645437	.	-	.	gene_id "lnc-bbug06058"	transcript_id "lnc-bbut00014424"	exon_number "3"
14	lncRNA	exon	10655009	10655296	.	-	.	gene_id "lnc-bbug06058"	transcript_id "lnc-bbut00014424"	exon_number "4"
14	lncRNA	exon	10620851	10621596	.	-	.	gene_id "lnc-bbug06058"	transcript_id "lnc-bbut00014425"	exon_number "1"
14	lncRNA	exon	10625041	10625214	.	-	.	gene_id "lnc-bbug06058"	transcript_id "lnc-bbut00014425"	exon_number "2"
14	lncRNA	exon	10645368	10645437	.	-	.	gene_id "lnc-bbug06058"	transcript_id "lnc-bbut00014425"	exon_number "3"
14	lncRNA	exon	10649888	10649962	.	-	.	gene_id "lnc-bbug06058"	transcript_id "lnc-bbut00014425"	exon_number "4"
14	lncRNA	exon	10655009	10655297	.	-	.	gene_id "lnc-bbug06058"	transcript_id "lnc-bbut00014425"	exon_number "5"
14	lncRNA	exon	10620851	10621596	.	-	.	gene_id "lnc-bbug06058"	transcript_id "lnc-bbut00014426"	exon_number "1"

14	lncRNA	exon	10625041	10625214	.	-	.	gene_id "lnc-bbug06058"	transcript_id "lnc-bbut00014426"	exon_number "2"
14	lncRNA	exon	10645368	10645437	.	-	.	gene_id "lnc-bbug06058"	transcript_id "lnc-bbut00014426"	exon_number "3"
14	lncRNA	exon	10657195	10657216	.	-	.	gene_id "lnc-bbug06058"	transcript_id "lnc-bbut00014426"	exon_number "4"
14	lncRNA	exon	10620851	10621596	.	-	.	gene_id "lnc-bbug06058"	transcript_id "lnc-bbut00014427"	exon_number "1"
14	lncRNA	exon	10625041	10625214	.	-	.	gene_id "lnc-bbug06058"	transcript_id "lnc-bbut00014427"	exon_number "2"
14	lncRNA	exon	10645368	10645437	.	-	.	gene_id "lnc-bbug06058"	transcript_id "lnc-bbut00014427"	exon_number "3"
14	lncRNA	exon	10696465	10696486	.	-	.	gene_id "lnc-bbug06058"	transcript_id "lnc-bbut00014427"	exon_number "4"
14	lncRNA	exon	10620851	10621596	.	-	.	gene_id "lnc-bbug06058"	transcript_id "lnc-bbut00014428"	exon_number "1"
14	lncRNA	exon	10625041	10625214	.	-	.	gene_id "lnc-bbug06058"	transcript_id "lnc-bbut00014428"	exon_number "2"
14	lncRNA	exon	10645368	10645437	.	-	.	gene_id "lnc-bbug06058"	transcript_id "lnc-bbut00014428"	exon_number "3"
14	lncRNA	exon	10700617	10700757	.	-	.	gene_id "lnc-bbug06058"	transcript_id "lnc-bbut00014428"	exon_number "4"
14	lncRNA	exon	11417470	11420518	.	-	.	gene_id "lnc-bbug06066"	transcript_id "lnc-bbut00014448"	exon_number "1"
14	lncRNA	exon	11421032	11421149	.	-	.	gene_id "lnc-bbug06066"	transcript_id "lnc-bbut00014448"	exon_number "2"
14	lncRNA	exon	11421884	11422301	.	-	.	gene_id "lnc-bbug06066"	transcript_id "lnc-bbut00014448"	exon_number "3"
14	lncRNA	exon	11785321	11785786	.	-	.	gene_id "lnc-bbug06072"	transcript_id "lnc-bbut00014465"	exon_number "1"
14	lncRNA	exon	11785931	11790172	.	-	.	gene_id "lnc-bbug06072"	transcript_id "lnc-bbut00014465"	exon_number "2"
14	lncRNA	exon	12923192	12926056	.	-	.	gene_id "lnc-bbug06073"	transcript_id "lnc-bbut00014466"	exon_number "1"
14	lncRNA	exon	12926929	12927021	.	-	.	gene_id "lnc-bbug06073"	transcript_id "lnc-bbut00014466"	exon_number "2"
14	lncRNA	exon	12927577	12927792	.	-	.	gene_id "lnc-bbug06073"	transcript_id "lnc-bbut00014466"	exon_number "3"
14	lncRNA	exon	12927918	12929506	.	-	.	gene_id "lnc-bbug06073"	transcript_id "lnc-bbut00014466"	exon_number "4"
14	lncRNA	exon	13350098	13350668	.	-	.	gene_id "lnc-bbug06074"	transcript_id "lnc-bbut00014467"	exon_number "1"
14	lncRNA	exon	13351736	13352048	.	-	.	gene_id "lnc-bbug06074"	transcript_id "lnc-bbut00014467"	exon_number "2"
14	lncRNA	exon	13352567	13352644	.	-	.	gene_id "lnc-bbug06074"	transcript_id "lnc-bbut00014467"	exon_number "3"
14	lncRNA	exon	13668416	13669350	.	-	.	gene_id "lnc-bbug06076"	transcript_id "lnc-bbut00014475"	exon_number "1"
14	lncRNA	exon	13672323	13672427	.	-	.	gene_id "lnc-bbug06076"	transcript_id "lnc-bbut00014475"	exon_number "2"
14	lncRNA	exon	13787585	13791798	.	-	.	gene_id "lnc-bbug06077"	transcript_id "lnc-bbut00014476"	exon_number "1"
14	lncRNA	exon	13792979	13794925	.	-	.	gene_id "lnc-bbug06077"	transcript_id "lnc-bbut00014476"	exon_number "2"
14	lncRNA	exon	13982042	13982455	.	-	.	gene_id "lnc-bbug06080"	transcript_id "lnc-bbut00014482"	exon_number "1"
14	lncRNA	exon	13984976	13985060	.	-	.	gene_id "lnc-bbug06080"	transcript_id "lnc-bbut00014482"	exon_number "2"
14	lncRNA	exon	14602772	14603007	.	-	.	gene_id "lnc-bbug06082"	transcript_id "lnc-bbut00014484"	exon_number "1"
14	lncRNA	exon	14607539	14607824	.	-	.	gene_id "lnc-bbug06082"	transcript_id "lnc-bbut00014484"	exon_number "2"
14	lncRNA	exon	15111690	15111806	.	-	.	gene_id "lnc-bbug06083"	transcript_id "lnc-bbut00014485"	exon_number "1"
14	lncRNA	exon	15112178	15112285	.	-	.	gene_id "lnc-bbug06083"	transcript_id "lnc-bbut00014485"	exon_number "2"
14	lncRNA	exon	15290119	15290556	.	-	.	gene_id "lnc-bbug06084"	transcript_id "lnc-bbut00014486"	exon_number "1"
14	lncRNA	exon	15291213	15291314	.	-	.	gene_id "lnc-bbug06084"	transcript_id "lnc-bbut00014486"	exon_number "2"
14	lncRNA	exon	15300943	15301045	.	-	.	gene_id "lnc-bbug06084"	transcript_id "lnc-bbut00014486"	exon_number "3"
14	lncRNA	exon	15587266	15587374	.	-	.	gene_id "lnc-bbug06085"	transcript_id "lnc-bbut00014487"	exon_number "1"
14	lncRNA	exon	15587483	15587799	.	-	.	gene_id "lnc-bbug06085"	transcript_id "lnc-bbut00014487"	exon_number "2"
14	lncRNA	exon	15636824	15636995	.	-	.	gene_id "lnc-bbug06085"	transcript_id "lnc-bbut00014487"	exon_number "3"
14	lncRNA	exon	15851687	15853671	.	-	.	gene_id "lnc-bbug06087"	transcript_id "lnc-bbut00014493"	exon_number "1"
14	lncRNA	exon	16140967	16143569	.	-	.	gene_id "lnc-bbug06093"	transcript_id "lnc-bbut00014501"	exon_number "1"
14	lncRNA	exon	16145799	16145931	.	-	.	gene_id "lnc-bbug06093"	transcript_id "lnc-bbut00014501"	exon_number "2"
14	lncRNA	exon	16152325	16153405	.	-	.	gene_id "lnc-bbug06093"	transcript_id "lnc-bbut00014501"	exon_number "3"
14	lncRNA	exon	16260242	16260649	.	-	.	gene_id "lnc-bbug06095"	transcript_id "lnc-bbut00014508"	exon_number "1"
14	lncRNA	exon	16260736	16262306	.	-	.	gene_id "lnc-bbug06095"	transcript_id "lnc-bbut00014508"	exon_number "2"
14	lncRNA	exon	16263441	16263785	.	-	.	gene_id "lnc-bbug06095"	transcript_id "lnc-bbut00014508"	exon_number "3"
14	lncRNA	exon	16264156	16264336	.	-	.	gene_id "lnc-bbug06095"	transcript_id "lnc-bbut00014508"	exon_number "4"
14	lncRNA	exon	16266190	16267046	.	-	.	gene_id "lnc-bbug06095"	transcript_id "lnc-bbut00014508"	exon_number "5"
14	lncRNA	exon	16267735	16267854	.	-	.	gene_id "lnc-bbug06095"	transcript_id "lnc-bbut00014508"	exon_number "6"
14	lncRNA	exon	16260242	16260649	.	-	.	gene_id "lnc-bbug06095"	transcript_id "lnc-bbut00014509"	exon_number "1"
14	lncRNA	exon	16260736	16260913	.	-	.	gene_id "lnc-bbug06095"	transcript_id "lnc-bbut00014509"	exon_number "2"
14	lncRNA	exon	16262234	16262306	.	-	.	gene_id "lnc-bbug06095"	transcript_id "lnc-bbut00014509"	exon_number "3"

14	lncRNA	exon	16263441	16263785	.	-	.	gene_id "lnc-bbug06095"	transcript_id "lnc-bbut00014509"	exon_number "4"
14	lncRNA	exon	16264156	16264336	.	-	.	gene_id "lnc-bbug06095"	transcript_id "lnc-bbut00014509"	exon_number "5"
14	lncRNA	exon	16266190	16267046	.	-	.	gene_id "lnc-bbug06095"	transcript_id "lnc-bbut00014509"	exon_number "6"
14	lncRNA	exon	16273070	16273811	.	-	.	gene_id "lnc-bbug06095"	transcript_id "lnc-bbut00014509"	exon_number "7"
14	lncRNA	exon	16260242	16260649	.	-	.	gene_id "lnc-bbug06095"	transcript_id "lnc-bbut00014510"	exon_number "1"
14	lncRNA	exon	16260736	16261957	.	-	.	gene_id "lnc-bbug06095"	transcript_id "lnc-bbut00014510"	exon_number "2"
14	lncRNA	exon	16262234	16262306	.	-	.	gene_id "lnc-bbug06095"	transcript_id "lnc-bbut00014510"	exon_number "3"
14	lncRNA	exon	16263441	16263785	.	-	.	gene_id "lnc-bbug06095"	transcript_id "lnc-bbut00014510"	exon_number "4"
14	lncRNA	exon	16264156	16264336	.	-	.	gene_id "lnc-bbug06095"	transcript_id "lnc-bbut00014510"	exon_number "5"
14	lncRNA	exon	16266190	16267046	.	-	.	gene_id "lnc-bbug06095"	transcript_id "lnc-bbut00014510"	exon_number "6"
14	lncRNA	exon	16273070	16273817	.	-	.	gene_id "lnc-bbug06095"	transcript_id "lnc-bbut00014510"	exon_number "7"
14	lncRNA	exon	16260242	16260649	.	-	.	gene_id "lnc-bbug06095"	transcript_id "lnc-bbut00014513"	exon_number "1"
14	lncRNA	exon	16260736	16260913	.	-	.	gene_id "lnc-bbug06095"	transcript_id "lnc-bbut00014513"	exon_number "2"
14	lncRNA	exon	16261804	16262306	.	-	.	gene_id "lnc-bbug06095"	transcript_id "lnc-bbut00014513"	exon_number "3"
14	lncRNA	exon	16263441	16263785	.	-	.	gene_id "lnc-bbug06095"	transcript_id "lnc-bbut00014513"	exon_number "4"
14	lncRNA	exon	16264156	16264336	.	-	.	gene_id "lnc-bbug06095"	transcript_id "lnc-bbut00014513"	exon_number "5"
14	lncRNA	exon	16266190	16267046	.	-	.	gene_id "lnc-bbug06095"	transcript_id "lnc-bbut00014513"	exon_number "6"
14	lncRNA	exon	16273070	16273818	.	-	.	gene_id "lnc-bbug06095"	transcript_id "lnc-bbut00014513"	exon_number "7"
14	lncRNA	exon	16260242	16260649	.	-	.	gene_id "lnc-bbug06095"	transcript_id "lnc-bbut00014512"	exon_number "1"
14	lncRNA	exon	16260736	16262306	.	-	.	gene_id "lnc-bbug06095"	transcript_id "lnc-bbut00014512"	exon_number "2"
14	lncRNA	exon	16263441	16263785	.	-	.	gene_id "lnc-bbug06095"	transcript_id "lnc-bbut00014512"	exon_number "3"
14	lncRNA	exon	16264156	16264336	.	-	.	gene_id "lnc-bbug06095"	transcript_id "lnc-bbut00014512"	exon_number "4"
14	lncRNA	exon	16266190	16267046	.	-	.	gene_id "lnc-bbug06095"	transcript_id "lnc-bbut00014512"	exon_number "5"
14	lncRNA	exon	16273382	16273818	.	-	.	gene_id "lnc-bbug06095"	transcript_id "lnc-bbut00014512"	exon_number "6"
14	lncRNA	exon	16260242	16260649	.	-	.	gene_id "lnc-bbug06095"	transcript_id "lnc-bbut00014511"	exon_number "1"
14	lncRNA	exon	16260736	16262306	.	-	.	gene_id "lnc-bbug06095"	transcript_id "lnc-bbut00014511"	exon_number "2"
14	lncRNA	exon	16263441	16263785	.	-	.	gene_id "lnc-bbug06095"	transcript_id "lnc-bbut00014511"	exon_number "3"
14	lncRNA	exon	16264156	16264336	.	-	.	gene_id "lnc-bbug06095"	transcript_id "lnc-bbut00014511"	exon_number "4"
14	lncRNA	exon	16266190	16267046	.	-	.	gene_id "lnc-bbug06095"	transcript_id "lnc-bbut00014511"	exon_number "5"
14	lncRNA	exon	16273070	16273818	.	-	.	gene_id "lnc-bbug06095"	transcript_id "lnc-bbut00014511"	exon_number "6"
14	lncRNA	exon	16490361	16490414	.	-	.	gene_id "lnc-bbug06099"	transcript_id "lnc-bbut00014518"	exon_number "1"
14	lncRNA	exon	16491584	16491674	.	-	.	gene_id "lnc-bbug06099"	transcript_id "lnc-bbut00014518"	exon_number "2"
14	lncRNA	exon	16504955	16505206	.	-	.	gene_id "lnc-bbug06099"	transcript_id "lnc-bbut00014518"	exon_number "3"
14	lncRNA	exon	16511352	16511464	.	-	.	gene_id "lnc-bbug06099"	transcript_id "lnc-bbut00014518"	exon_number "4"
14	lncRNA	exon	16504272	16504658	.	-	.	gene_id "lnc-bbug06099"	transcript_id "lnc-bbut00014519"	exon_number "1"
14	lncRNA	exon	16504955	16505206	.	-	.	gene_id "lnc-bbug06099"	transcript_id "lnc-bbut00014519"	exon_number "2"
14	lncRNA	exon	16507472	16507573	.	-	.	gene_id "lnc-bbug06099"	transcript_id "lnc-bbut00014519"	exon_number "3"
14	lncRNA	exon	16511352	16511460	.	-	.	gene_id "lnc-bbug06099"	transcript_id "lnc-bbut00014519"	exon_number "4"
14	lncRNA	exon	16504272	16504658	.	-	.	gene_id "lnc-bbug06099"	transcript_id "lnc-bbut00014520"	exon_number "1"
14	lncRNA	exon	16504955	16505206	.	-	.	gene_id "lnc-bbug06099"	transcript_id "lnc-bbut00014520"	exon_number "2"
14	lncRNA	exon	16511352	16511464	.	-	.	gene_id "lnc-bbug06099"	transcript_id "lnc-bbut00014520"	exon_number "3"
14	lncRNA	exon	16739751	16740018	.	-	.	gene_id "lnc-bbug06102"	transcript_id "lnc-bbut00014523"	exon_number "1"
14	lncRNA	exon	16740445	16740507	.	-	.	gene_id "lnc-bbug06102"	transcript_id "lnc-bbut00014523"	exon_number "2"
14	lncRNA	exon	16741839	16742064	.	-	.	gene_id "lnc-bbug06102"	transcript_id "lnc-bbut00014523"	exon_number "3"
14	lncRNA	exon	17143001	17144006	.	-	.	gene_id "lnc-bbug06105"	transcript_id "lnc-bbut00014527"	exon_number "1"
14	lncRNA	exon	17144232	17144312	.	-	.	gene_id "lnc-bbug06105"	transcript_id "lnc-bbut00014527"	exon_number "2"
14	lncRNA	exon	17145222	17145373	.	-	.	gene_id "lnc-bbug06105"	transcript_id "lnc-bbut00014527"	exon_number "3"
14	lncRNA	exon	17143001	17144006	.	-	.	gene_id "lnc-bbug06105"	transcript_id "lnc-bbut00014526"	exon_number "1"
14	lncRNA	exon	17145222	17145373	.	-	.	gene_id "lnc-bbug06105"	transcript_id "lnc-bbut00014526"	exon_number "2"
14	lncRNA	exon	17339658	17340539	.	-	.	gene_id "lnc-bbug06107"	transcript_id "lnc-bbut00014530"	exon_number "1"
14	lncRNA	exon	17358314	17358519	.	-	.	gene_id "lnc-bbug06107"	transcript_id "lnc-bbut00014530"	exon_number "2"
14	lncRNA	exon	17367860	17367892	.	-	.	gene_id "lnc-bbug06107"	transcript_id "lnc-bbut00014530"	exon_number "3"

14	lncRNA	exon	17339658	17340539	.	-	.	gene_id "lnc-bbug06107"	transcript_id "lnc-bbut00014531"	exon_number "1"
14	lncRNA	exon	17358314	17358522	.	-	.	gene_id "lnc-bbug06107"	transcript_id "lnc-bbut00014531"	exon_number "2"
14	lncRNA	exon	17367860	17367910	.	-	.	gene_id "lnc-bbug06107"	transcript_id "lnc-bbut00014531"	exon_number "3"
14	lncRNA	exon	17372656	17374640	.	-	.	gene_id "lnc-bbug06108"	transcript_id "lnc-bbut00014532"	exon_number "1"
14	lncRNA	exon	17381612	17381699	.	-	.	gene_id "lnc-bbug06108"	transcript_id "lnc-bbut00014532"	exon_number "2"
14	lncRNA	exon	17613017	17613635	.	-	.	gene_id "lnc-bbug06110"	transcript_id "lnc-bbut00014536"	exon_number "1"
14	lncRNA	exon	17615549	17615649	.	-	.	gene_id "lnc-bbug06110"	transcript_id "lnc-bbut00014536"	exon_number "2"
14	lncRNA	exon	17615855	17616614	.	-	.	gene_id "lnc-bbug06110"	transcript_id "lnc-bbut00014536"	exon_number "3"
14	lncRNA	exon	17613017	17613635	.	-	.	gene_id "lnc-bbug06110"	transcript_id "lnc-bbut00014537"	exon_number "1"
14	lncRNA	exon	17615549	17615649	.	-	.	gene_id "lnc-bbug06110"	transcript_id "lnc-bbut00014537"	exon_number "2"
14	lncRNA	exon	17626288	17626397	.	-	.	gene_id "lnc-bbug06110"	transcript_id "lnc-bbut00014537"	exon_number "3"
14	lncRNA	exon	17744353	17744577	.	-	.	gene_id "lnc-bbug06113"	transcript_id "lnc-bbut00014546"	exon_number "1"
14	lncRNA	exon	17744995	17745518	.	-	.	gene_id "lnc-bbug06113"	transcript_id "lnc-bbut00014546"	exon_number "2"
14	lncRNA	exon	18424181	18425390	.	-	.	gene_id "lnc-bbug06120"	transcript_id "lnc-bbut00014580"	exon_number "1"
14	lncRNA	exon	18425557	18428259	.	-	.	gene_id "lnc-bbug06120"	transcript_id "lnc-bbut00014580"	exon_number "2"
14	lncRNA	exon	18428439	18428457	.	-	.	gene_id "lnc-bbug06121"	transcript_id "lnc-bbut00014581"	exon_number "1"
14	lncRNA	exon	18428732	18429305	.	-	.	gene_id "lnc-bbug06121"	transcript_id "lnc-bbut00014581"	exon_number "2"
14	lncRNA	exon	18706322	18706519	.	-	.	gene_id "lnc-bbug06124"	transcript_id "lnc-bbut00014590"	exon_number "1"
14	lncRNA	exon	18707444	18707673	.	-	.	gene_id "lnc-bbug06124"	transcript_id "lnc-bbut00014590"	exon_number "2"
14	lncRNA	exon	18707765	18707872	.	-	.	gene_id "lnc-bbug06124"	transcript_id "lnc-bbut00014590"	exon_number "3"
14	lncRNA	exon	18706322	18706519	.	-	.	gene_id "lnc-bbug06124"	transcript_id "lnc-bbut00014591"	exon_number "1"
14	lncRNA	exon	18707444	18707574	.	-	.	gene_id "lnc-bbug06124"	transcript_id "lnc-bbut00014591"	exon_number "2"
14	lncRNA	exon	18707765	18707897	.	-	.	gene_id "lnc-bbug06124"	transcript_id "lnc-bbut00014591"	exon_number "3"
14	lncRNA	exon	18796795	18796900	.	-	.	gene_id "lnc-bbug06126"	transcript_id "lnc-bbut00014593"	exon_number "1"
14	lncRNA	exon	18800573	18800693	.	-	.	gene_id "lnc-bbug06126"	transcript_id "lnc-bbut00014593"	exon_number "2"
14	lncRNA	exon	18813791	18814280	.	-	.	gene_id "lnc-bbug06126"	transcript_id "lnc-bbut00014593"	exon_number "3"
14	lncRNA	exon	18943444	18943560	.	-	.	gene_id "lnc-bbug06129"	transcript_id "lnc-bbut00014603"	exon_number "1"
14	lncRNA	exon	18944675	18944856	.	-	.	gene_id "lnc-bbug06129"	transcript_id "lnc-bbut00014603"	exon_number "2"
14	lncRNA	exon	19590238	19591179	.	-	.	gene_id "lnc-bbug06137"	transcript_id "lnc-bbut00014615"	exon_number "1"
14	lncRNA	exon	19708228	19708573	.	-	.	gene_id "lnc-bbug06139"	transcript_id "lnc-bbut00014618"	exon_number "1"
14	lncRNA	exon	19712215	19712382	.	-	.	gene_id "lnc-bbug06139"	transcript_id "lnc-bbut00014618"	exon_number "2"
14	lncRNA	exon	19717441	19717516	.	-	.	gene_id "lnc-bbug06139"	transcript_id "lnc-bbut00014618"	exon_number "3"
14	lncRNA	exon	19725859	19725986	.	-	.	gene_id "lnc-bbug06139"	transcript_id "lnc-bbut00014618"	exon_number "4"
14	lncRNA	exon	19844276	19844842	.	-	.	gene_id "lnc-bbug06141"	transcript_id "lnc-bbut00014622"	exon_number "1"
14	lncRNA	exon	19851477	19851567	.	-	.	gene_id "lnc-bbug06141"	transcript_id "lnc-bbut00014622"	exon_number "2"
14	lncRNA	exon	19946809	19947495	.	-	.	gene_id "lnc-bbug06142"	transcript_id "lnc-bbut00014623"	exon_number "1"
14	lncRNA	exon	19951030	19951094	.	-	.	gene_id "lnc-bbug06142"	transcript_id "lnc-bbut00014623"	exon_number "2"
14	lncRNA	exon	19952408	19952577	.	-	.	gene_id "lnc-bbug06142"	transcript_id "lnc-bbut00014623"	exon_number "3"
14	lncRNA	exon	20022707	20022794	.	-	.	gene_id "lnc-bbug06142"	transcript_id "lnc-bbut00014623"	exon_number "4"
14	lncRNA	exon	20023154	20023802	.	-	.	gene_id "lnc-bbug06142"	transcript_id "lnc-bbut00014623"	exon_number "5"
14	lncRNA	exon	19946809	19947495	.	-	.	gene_id "lnc-bbug06142"	transcript_id "lnc-bbut00014624"	exon_number "1"
14	lncRNA	exon	19951030	19951094	.	-	.	gene_id "lnc-bbug06142"	transcript_id "lnc-bbut00014624"	exon_number "2"
14	lncRNA	exon	19952408	19952577	.	-	.	gene_id "lnc-bbug06142"	transcript_id "lnc-bbut00014624"	exon_number "3"
14	lncRNA	exon	20022707	20022794	.	-	.	gene_id "lnc-bbug06142"	transcript_id "lnc-bbut00014624"	exon_number "4"
14	lncRNA	exon	20024089	20024162	.	-	.	gene_id "lnc-bbug06142"	transcript_id "lnc-bbut00014624"	exon_number "5"
14	lncRNA	exon	19946809	19947495	.	-	.	gene_id "lnc-bbug06142"	transcript_id "lnc-bbut00014625"	exon_number "1"
14	lncRNA	exon	19951030	19951094	.	-	.	gene_id "lnc-bbug06142"	transcript_id "lnc-bbut00014625"	exon_number "2"
14	lncRNA	exon	19952408	19952577	.	-	.	gene_id "lnc-bbug06142"	transcript_id "lnc-bbut00014625"	exon_number "3"
14	lncRNA	exon	20022707	20022794	.	-	.	gene_id "lnc-bbug06142"	transcript_id "lnc-bbut00014625"	exon_number "4"
14	lncRNA	exon	20023840	20024013	.	-	.	gene_id "lnc-bbug06142"	transcript_id "lnc-bbut00014625"	exon_number "5"
14	lncRNA	exon	20025428	20025501	.	-	.	gene_id "lnc-bbug06142"	transcript_id "lnc-bbut00014625"	exon_number "6"
14	lncRNA	exon	20041769	20041824	.	-	.	gene_id "lnc-bbug06142"	transcript_id "lnc-bbut00014625"	exon_number "7"

14	lncRNA	exon	20043790	20043962	.	-	.	gene_id "lnc-bbug06142"	transcript_id "lnc-bbut00014625"	exon_number "8"
14	lncRNA	exon	20090670	20092579	.	-	.	gene_id "lnc-bbug06143"	transcript_id "lnc-bbut00014626"	exon_number "1"
14	lncRNA	exon	20110894	20110969	.	-	.	gene_id "lnc-bbug06143"	transcript_id "lnc-bbut00014626"	exon_number "2"
14	lncRNA	exon	20113065	20113139	.	-	.	gene_id "lnc-bbug06143"	transcript_id "lnc-bbut00014626"	exon_number "3"
14	lncRNA	exon	20722883	20723427	.	-	.	gene_id "lnc-bbug06149"	transcript_id "lnc-bbut00014645"	exon_number "1"
14	lncRNA	exon	20728799	20728912	.	-	.	gene_id "lnc-bbug06149"	transcript_id "lnc-bbut00014645"	exon_number "2"
14	lncRNA	exon	20729031	20729169	.	-	.	gene_id "lnc-bbug06149"	transcript_id "lnc-bbut00014645"	exon_number "3"
14	lncRNA	exon	20987091	20987267	.	-	.	gene_id "lnc-bbug06156"	transcript_id "lnc-bbut00014657"	exon_number "1"
14	lncRNA	exon	20987887	20987959	.	-	.	gene_id "lnc-bbug06156"	transcript_id "lnc-bbut00014657"	exon_number "2"
14	lncRNA	exon	20988861	20989160	.	-	.	gene_id "lnc-bbug06156"	transcript_id "lnc-bbut00014657"	exon_number "3"
14	lncRNA	exon	21480384	21480882	.	-	.	gene_id "lnc-bbug06169"	transcript_id "lnc-bbut00014682"	exon_number "1"
14	lncRNA	exon	21484106	21484157	.	-	.	gene_id "lnc-bbug06169"	transcript_id "lnc-bbut00014682"	exon_number "2"
14	lncRNA	exon	21485600	21487711	.	-	.	gene_id "lnc-bbug06170"	transcript_id "lnc-bbut00014683"	exon_number "1"
14	lncRNA	exon	21487927	21488449	.	-	.	gene_id "lnc-bbug06170"	transcript_id "lnc-bbut00014683"	exon_number "2"
14	lncRNA	exon	22008961	22009359	.	-	.	gene_id "lnc-bbug06176"	transcript_id "lnc-bbut00014710"	exon_number "1"
14	lncRNA	exon	22022050	22023146	.	-	.	gene_id "lnc-bbug06176"	transcript_id "lnc-bbut00014710"	exon_number "2"
14	lncRNA	exon	22024532	22026864	.	-	.	gene_id "lnc-bbug06177"	transcript_id "lnc-bbut00014711"	exon_number "1"
14	lncRNA	exon	22335753	22336904	.	-	.	gene_id "lnc-bbug06182"	transcript_id "lnc-bbut00014724"	exon_number "1"
14	lncRNA	exon	22342958	22343066	.	-	.	gene_id "lnc-bbug06182"	transcript_id "lnc-bbut00014724"	exon_number "2"
14	lncRNA	exon	22365228	22365471	.	-	.	gene_id "lnc-bbug06183"	transcript_id "lnc-bbut00014727"	exon_number "1"
14	lncRNA	exon	22366724	22366753	.	-	.	gene_id "lnc-bbug06183"	transcript_id "lnc-bbut00014727"	exon_number "2"
14	lncRNA	exon	22369463	22369544	.	-	.	gene_id "lnc-bbug06183"	transcript_id "lnc-bbut00014727"	exon_number "3"
14	lncRNA	exon	22370640	22373682	.	-	.	gene_id "lnc-bbug06183"	transcript_id "lnc-bbut00014727"	exon_number "4"
14	lncRNA	exon	22373870	22374221	.	-	.	gene_id "lnc-bbug06183"	transcript_id "lnc-bbut00014727"	exon_number "5"
14	lncRNA	exon	22365228	22365471	.	-	.	gene_id "lnc-bbug06183"	transcript_id "lnc-bbut00014728"	exon_number "1"
14	lncRNA	exon	22369463	22369544	.	-	.	gene_id "lnc-bbug06183"	transcript_id "lnc-bbut00014728"	exon_number "2"
14	lncRNA	exon	22370640	22373682	.	-	.	gene_id "lnc-bbug06183"	transcript_id "lnc-bbut00014728"	exon_number "3"
14	lncRNA	exon	22373870	22374222	.	-	.	gene_id "lnc-bbug06183"	transcript_id "lnc-bbut00014728"	exon_number "4"
14	lncRNA	exon	22519769	22520017	.	-	.	gene_id "lnc-bbug06187"	transcript_id "lnc-bbut00014738"	exon_number "1"
14	lncRNA	exon	22530196	22530394	.	-	.	gene_id "lnc-bbug06187"	transcript_id "lnc-bbut00014738"	exon_number "2"
14	lncRNA	exon	23019087	23020635	.	-	.	gene_id "lnc-bbug06193"	transcript_id "lnc-bbut00014751"	exon_number "1"
14	lncRNA	exon	23022469	23022911	.	-	.	gene_id "lnc-bbug06193"	transcript_id "lnc-bbut00014751"	exon_number "2"
14	lncRNA	exon	23164150	23166473	.	-	.	gene_id "lnc-bbug06195"	transcript_id "lnc-bbut00014753"	exon_number "1"
14	lncRNA	exon	23170968	23171411	.	-	.	gene_id "lnc-bbug06195"	transcript_id "lnc-bbut00014753"	exon_number "2"
14	lncRNA	exon	23704142	23705439	.	-	.	gene_id "lnc-bbug06201"	transcript_id "lnc-bbut00014761"	exon_number "1"
14	lncRNA	exon	23735377	23735512	.	-	.	gene_id "lnc-bbug06202"	transcript_id "lnc-bbut00014762"	exon_number "1"
14	lncRNA	exon	23742712	23742833	.	-	.	gene_id "lnc-bbug06202"	transcript_id "lnc-bbut00014762"	exon_number "2"
14	lncRNA	exon	23748183	23748292	.	-	.	gene_id "lnc-bbug06202"	transcript_id "lnc-bbut00014762"	exon_number "3"
14	lncRNA	exon	23809114	23811099	.	-	.	gene_id "lnc-bbug06204"	transcript_id "lnc-bbut00014765"	exon_number "1"
14	lncRNA	exon	23813131	23813295	.	-	.	gene_id "lnc-bbug06204"	transcript_id "lnc-bbut00014765"	exon_number "2"
14	lncRNA	exon	23817385	23817497	.	-	.	gene_id "lnc-bbug06204"	transcript_id "lnc-bbut00014765"	exon_number "3"
14	lncRNA	exon	23837214	23837261	.	-	.	gene_id "lnc-bbug06204"	transcript_id "lnc-bbut00014765"	exon_number "4"
14	lncRNA	exon	23837347	23837516	.	-	.	gene_id "lnc-bbug06204"	transcript_id "lnc-bbut00014765"	exon_number "5"
14	lncRNA	exon	24736168	24737526	.	-	.	gene_id "lnc-bbug06211"	transcript_id "lnc-bbut00014776"	exon_number "1"
14	lncRNA	exon	24743215	24743284	.	-	.	gene_id "lnc-bbug06211"	transcript_id "lnc-bbut00014776"	exon_number "2"
14	lncRNA	exon	24766899	24767008	.	-	.	gene_id "lnc-bbug06211"	transcript_id "lnc-bbut00014776"	exon_number "3"
14	lncRNA	exon	25100472	25101602	.	-	.	gene_id "lnc-bbug06215"	transcript_id "lnc-bbut00014782"	exon_number "1"
14	lncRNA	exon	25103774	25104013	.	-	.	gene_id "lnc-bbug06215"	transcript_id "lnc-bbut00014782"	exon_number "2"
14	lncRNA	exon	25204276	25204575	.	-	.	gene_id "lnc-bbug06216"	transcript_id "lnc-bbut00014783"	exon_number "1"
14	lncRNA	exon	25204700	25204840	.	-	.	gene_id "lnc-bbug06216"	transcript_id "lnc-bbut00014783"	exon_number "2"
14	lncRNA	exon	25205369	25205658	.	-	.	gene_id "lnc-bbug06216"	transcript_id "lnc-bbut00014783"	exon_number "3"
14	lncRNA	exon	25452284	25454188	.	-	.	gene_id "lnc-bbug06218"	transcript_id "lnc-bbut00014786"	exon_number "1"

14	lncRNA	exon	25458023	25458119	.	-	.	gene_id "lnc-bbug06218"	transcript_id "lnc-bbut00014786"	exon_number "2"
14	lncRNA	exon	25461097	25466999	.	-	.	gene_id "lnc-bbug06218"	transcript_id "lnc-bbut00014786"	exon_number "3"
14	lncRNA	exon	25470027	25470111	.	-	.	gene_id "lnc-bbug06218"	transcript_id "lnc-bbut00014786"	exon_number "4"
14	lncRNA	exon	25471281	25471525	.	-	.	gene_id "lnc-bbug06218"	transcript_id "lnc-bbut00014786"	exon_number "5"
14	lncRNA	exon	25492419	25492561	.	-	.	gene_id "lnc-bbug06218"	transcript_id "lnc-bbut00014786"	exon_number "6"
14	lncRNA	exon	25527579	25527775	.	-	.	gene_id "lnc-bbug06218"	transcript_id "lnc-bbut00014786"	exon_number "7"
14	lncRNA	exon	25452284	25454188	.	-	.	gene_id "lnc-bbug06218"	transcript_id "lnc-bbut00014785"	exon_number "1"
14	lncRNA	exon	25457986	25458119	.	-	.	gene_id "lnc-bbug06218"	transcript_id "lnc-bbut00014785"	exon_number "2"
14	lncRNA	exon	25461097	25466999	.	-	.	gene_id "lnc-bbug06218"	transcript_id "lnc-bbut00014785"	exon_number "3"
14	lncRNA	exon	25470027	25470111	.	-	.	gene_id "lnc-bbug06218"	transcript_id "lnc-bbut00014785"	exon_number "4"
14	lncRNA	exon	25471281	25471525	.	-	.	gene_id "lnc-bbug06218"	transcript_id "lnc-bbut00014785"	exon_number "5"
14	lncRNA	exon	25492419	25492561	.	-	.	gene_id "lnc-bbug06218"	transcript_id "lnc-bbut00014785"	exon_number "6"
14	lncRNA	exon	25527579	25527775	.	-	.	gene_id "lnc-bbug06218"	transcript_id "lnc-bbut00014785"	exon_number "7"
14	lncRNA	exon	25456554	25458119	.	-	.	gene_id "lnc-bbug06218"	transcript_id "lnc-bbut00014791"	exon_number "1"
14	lncRNA	exon	25461097	25461197	.	-	.	gene_id "lnc-bbug06218"	transcript_id "lnc-bbut00014791"	exon_number "2"
14	lncRNA	exon	25461738	25466999	.	-	.	gene_id "lnc-bbug06218"	transcript_id "lnc-bbut00014791"	exon_number "3"
14	lncRNA	exon	25470027	25470111	.	-	.	gene_id "lnc-bbug06218"	transcript_id "lnc-bbut00014791"	exon_number "4"
14	lncRNA	exon	25471281	25471525	.	-	.	gene_id "lnc-bbug06218"	transcript_id "lnc-bbut00014791"	exon_number "5"
14	lncRNA	exon	25492419	25492561	.	-	.	gene_id "lnc-bbug06218"	transcript_id "lnc-bbut00014791"	exon_number "6"
14	lncRNA	exon	25527579	25527775	.	-	.	gene_id "lnc-bbug06218"	transcript_id "lnc-bbut00014791"	exon_number "7"
14	lncRNA	exon	25456554	25458119	.	-	.	gene_id "lnc-bbug06218"	transcript_id "lnc-bbut00014793"	exon_number "1"
14	lncRNA	exon	25461097	25461882	.	-	.	gene_id "lnc-bbug06218"	transcript_id "lnc-bbut00014793"	exon_number "2"
14	lncRNA	exon	25462095	25466999	.	-	.	gene_id "lnc-bbug06218"	transcript_id "lnc-bbut00014793"	exon_number "3"
14	lncRNA	exon	25470027	25470111	.	-	.	gene_id "lnc-bbug06218"	transcript_id "lnc-bbut00014793"	exon_number "4"
14	lncRNA	exon	25471281	25471525	.	-	.	gene_id "lnc-bbug06218"	transcript_id "lnc-bbut00014793"	exon_number "5"
14	lncRNA	exon	25492419	25492561	.	-	.	gene_id "lnc-bbug06218"	transcript_id "lnc-bbut00014793"	exon_number "6"
14	lncRNA	exon	25527579	25527775	.	-	.	gene_id "lnc-bbug06218"	transcript_id "lnc-bbut00014793"	exon_number "7"
14	lncRNA	exon	25456554	25458119	.	-	.	gene_id "lnc-bbug06218"	transcript_id "lnc-bbut00014795"	exon_number "1"
14	lncRNA	exon	25461097	25466999	.	-	.	gene_id "lnc-bbug06218"	transcript_id "lnc-bbut00014795"	exon_number "2"
14	lncRNA	exon	25470027	25470111	.	-	.	gene_id "lnc-bbug06218"	transcript_id "lnc-bbut00014795"	exon_number "3"
14	lncRNA	exon	25471281	25471525	.	-	.	gene_id "lnc-bbug06218"	transcript_id "lnc-bbut00014795"	exon_number "4"
14	lncRNA	exon	25474475	25474570	.	-	.	gene_id "lnc-bbug06218"	transcript_id "lnc-bbut00014795"	exon_number "5"
14	lncRNA	exon	25492419	25492561	.	-	.	gene_id "lnc-bbug06218"	transcript_id "lnc-bbut00014795"	exon_number "6"
14	lncRNA	exon	25527579	25527775	.	-	.	gene_id "lnc-bbug06218"	transcript_id "lnc-bbut00014795"	exon_number "7"
14	lncRNA	exon	25456554	25458119	.	-	.	gene_id "lnc-bbug06218"	transcript_id "lnc-bbut00014794"	exon_number "1"
14	lncRNA	exon	25461097	25462670	.	-	.	gene_id "lnc-bbug06218"	transcript_id "lnc-bbut00014794"	exon_number "2"
14	lncRNA	exon	25466806	25466999	.	-	.	gene_id "lnc-bbug06218"	transcript_id "lnc-bbut00014794"	exon_number "3"
14	lncRNA	exon	25470027	25470111	.	-	.	gene_id "lnc-bbug06218"	transcript_id "lnc-bbut00014794"	exon_number "4"
14	lncRNA	exon	25471281	25471525	.	-	.	gene_id "lnc-bbug06218"	transcript_id "lnc-bbut00014794"	exon_number "5"
14	lncRNA	exon	25492419	25492561	.	-	.	gene_id "lnc-bbug06218"	transcript_id "lnc-bbut00014794"	exon_number "6"
14	lncRNA	exon	25527579	25527775	.	-	.	gene_id "lnc-bbug06218"	transcript_id "lnc-bbut00014794"	exon_number "7"
14	lncRNA	exon	25456554	25458119	.	-	.	gene_id "lnc-bbug06218"	transcript_id "lnc-bbut00014792"	exon_number "1"
14	lncRNA	exon	25461097	25461197	.	-	.	gene_id "lnc-bbug06218"	transcript_id "lnc-bbut00014792"	exon_number "2"
14	lncRNA	exon	25462095	25466999	.	-	.	gene_id "lnc-bbug06218"	transcript_id "lnc-bbut00014792"	exon_number "3"
14	lncRNA	exon	25470027	25470111	.	-	.	gene_id "lnc-bbug06218"	transcript_id "lnc-bbut00014792"	exon_number "4"
14	lncRNA	exon	25471281	25471525	.	-	.	gene_id "lnc-bbug06218"	transcript_id "lnc-bbut00014792"	exon_number "5"
14	lncRNA	exon	25492419	25492561	.	-	.	gene_id "lnc-bbug06218"	transcript_id "lnc-bbut00014792"	exon_number "6"
14	lncRNA	exon	25527579	25527775	.	-	.	gene_id "lnc-bbug06218"	transcript_id "lnc-bbut00014792"	exon_number "7"
14	lncRNA	exon	25456554	25458119	.	-	.	gene_id "lnc-bbug06218"	transcript_id "lnc-bbut00014790"	exon_number "1"
14	lncRNA	exon	25461097	25466999	.	-	.	gene_id "lnc-bbug06218"	transcript_id "lnc-bbut00014790"	exon_number "2"
14	lncRNA	exon	25470027	25470111	.	-	.	gene_id "lnc-bbug06218"	transcript_id "lnc-bbut00014790"	exon_number "3"
14	lncRNA	exon	25471281	25471579	.	-	.	gene_id "lnc-bbug06218"	transcript_id "lnc-bbut00014790"	exon_number "4"

14	lncRNA	exon	25492419	25492561	.	-	.	gene_id "lnc-bbug06218"	transcript_id "lnc-bbut00014790"	exon_number "5"
14	lncRNA	exon	25527579	25527775	.	-	.	gene_id "lnc-bbug06218"	transcript_id "lnc-bbut00014790"	exon_number "6"
14	lncRNA	exon	25456554	25458119	.	-	.	gene_id "lnc-bbug06218"	transcript_id "lnc-bbut00014789"	exon_number "1"
14	lncRNA	exon	25461097	25466999	.	-	.	gene_id "lnc-bbug06218"	transcript_id "lnc-bbut00014789"	exon_number "2"
14	lncRNA	exon	25470027	25470111	.	-	.	gene_id "lnc-bbug06218"	transcript_id "lnc-bbut00014789"	exon_number "3"
14	lncRNA	exon	25492419	25492561	.	-	.	gene_id "lnc-bbug06218"	transcript_id "lnc-bbut00014789"	exon_number "4"
14	lncRNA	exon	25527579	25527775	.	-	.	gene_id "lnc-bbug06218"	transcript_id "lnc-bbut00014789"	exon_number "5"
14	lncRNA	exon	25456554	25458119	.	-	.	gene_id "lnc-bbug06218"	transcript_id "lnc-bbut00014788"	exon_number "1"
14	lncRNA	exon	25461097	25466999	.	-	.	gene_id "lnc-bbug06218"	transcript_id "lnc-bbut00014788"	exon_number "2"
14	lncRNA	exon	25470027	25470111	.	-	.	gene_id "lnc-bbug06218"	transcript_id "lnc-bbut00014788"	exon_number "3"
14	lncRNA	exon	25471281	25471525	.	-	.	gene_id "lnc-bbug06218"	transcript_id "lnc-bbut00014788"	exon_number "4"
14	lncRNA	exon	25492419	25492561	.	-	.	gene_id "lnc-bbug06218"	transcript_id "lnc-bbut00014788"	exon_number "5"
14	lncRNA	exon	25503083	25503221	.	-	.	gene_id "lnc-bbug06218"	transcript_id "lnc-bbut00014788"	exon_number "6"
14	lncRNA	exon	25527579	25527775	.	-	.	gene_id "lnc-bbug06218"	transcript_id "lnc-bbut00014788"	exon_number "7"
14	lncRNA	exon	25456554	25458119	.	-	.	gene_id "lnc-bbug06218"	transcript_id "lnc-bbut00014787"	exon_number "1"
14	lncRNA	exon	25461097	25466999	.	-	.	gene_id "lnc-bbug06218"	transcript_id "lnc-bbut00014787"	exon_number "2"
14	lncRNA	exon	25470027	25470111	.	-	.	gene_id "lnc-bbug06218"	transcript_id "lnc-bbut00014787"	exon_number "3"
14	lncRNA	exon	25471281	25471525	.	-	.	gene_id "lnc-bbug06218"	transcript_id "lnc-bbut00014787"	exon_number "4"
14	lncRNA	exon	25492419	25492561	.	-	.	gene_id "lnc-bbug06218"	transcript_id "lnc-bbut00014787"	exon_number "5"
14	lncRNA	exon	25494845	25494947	.	-	.	gene_id "lnc-bbug06218"	transcript_id "lnc-bbut00014787"	exon_number "6"
14	lncRNA	exon	25527579	25527775	.	-	.	gene_id "lnc-bbug06218"	transcript_id "lnc-bbut00014787"	exon_number "7"
14	lncRNA	exon	25456554	25458119	.	-	.	gene_id "lnc-bbug06218"	transcript_id "lnc-bbut00014797"	exon_number "1"
14	lncRNA	exon	25461097	25466999	.	-	.	gene_id "lnc-bbug06218"	transcript_id "lnc-bbut00014797"	exon_number "2"
14	lncRNA	exon	25470027	25470111	.	-	.	gene_id "lnc-bbug06218"	transcript_id "lnc-bbut00014797"	exon_number "3"
14	lncRNA	exon	25471281	25471525	.	-	.	gene_id "lnc-bbug06218"	transcript_id "lnc-bbut00014797"	exon_number "4"
14	lncRNA	exon	25492419	25492561	.	-	.	gene_id "lnc-bbug06218"	transcript_id "lnc-bbut00014797"	exon_number "5"
14	lncRNA	exon	25527579	25527623	.	-	.	gene_id "lnc-bbug06218"	transcript_id "lnc-bbut00014797"	exon_number "6"
14	lncRNA	exon	25527782	25527881	.	-	.	gene_id "lnc-bbug06218"	transcript_id "lnc-bbut00014797"	exon_number "7"
14	lncRNA	exon	25456554	25458119	.	-	.	gene_id "lnc-bbug06218"	transcript_id "lnc-bbut00014796"	exon_number "1"
14	lncRNA	exon	25461097	25466999	.	-	.	gene_id "lnc-bbug06218"	transcript_id "lnc-bbut00014796"	exon_number "2"
14	lncRNA	exon	25470027	25470111	.	-	.	gene_id "lnc-bbug06218"	transcript_id "lnc-bbut00014796"	exon_number "3"
14	lncRNA	exon	25471281	25471525	.	-	.	gene_id "lnc-bbug06218"	transcript_id "lnc-bbut00014796"	exon_number "4"
14	lncRNA	exon	25492419	25492561	.	-	.	gene_id "lnc-bbug06218"	transcript_id "lnc-bbut00014796"	exon_number "5"
14	lncRNA	exon	25527579	25527623	.	-	.	gene_id "lnc-bbug06218"	transcript_id "lnc-bbut00014796"	exon_number "6"
14	lncRNA	exon	25527764	25527881	.	-	.	gene_id "lnc-bbug06218"	transcript_id "lnc-bbut00014796"	exon_number "7"
14	lncRNA	exon	26024446	26026530	.	-	.	gene_id "lnc-bbug06220"	transcript_id "lnc-bbut00014801"	exon_number "1"
14	lncRNA	exon	26026684	26027145	.	-	.	gene_id "lnc-bbug06220"	transcript_id "lnc-bbut00014801"	exon_number "2"
14	lncRNA	exon	26041916	26042049	.	-	.	gene_id "lnc-bbug06220"	transcript_id "lnc-bbut00014801"	exon_number "3"
14	lncRNA	exon	26052051	26052235	.	-	.	gene_id "lnc-bbug06220"	transcript_id "lnc-bbut00014801"	exon_number "4"
14	lncRNA	exon	26053783	26054709	.	-	.	gene_id "lnc-bbug06220"	transcript_id "lnc-bbut00014801"	exon_number "5"
14	lncRNA	exon	26024446	26026770	.	-	.	gene_id "lnc-bbug06220"	transcript_id "lnc-bbut00014803"	exon_number "1"
14	lncRNA	exon	26041916	26042049	.	-	.	gene_id "lnc-bbug06220"	transcript_id "lnc-bbut00014803"	exon_number "2"
14	lncRNA	exon	26052051	26052235	.	-	.	gene_id "lnc-bbug06220"	transcript_id "lnc-bbut00014803"	exon_number "3"
14	lncRNA	exon	26053783	26054709	.	-	.	gene_id "lnc-bbug06220"	transcript_id "lnc-bbut00014803"	exon_number "4"
14	lncRNA	exon	26024446	26027145	.	-	.	gene_id "lnc-bbug06220"	transcript_id "lnc-bbut00014800"	exon_number "1"
14	lncRNA	exon	26041916	26042049	.	-	.	gene_id "lnc-bbug06220"	transcript_id "lnc-bbut00014800"	exon_number "2"
14	lncRNA	exon	26045294	26045877	.	-	.	gene_id "lnc-bbug06220"	transcript_id "lnc-bbut00014800"	exon_number "3"
14	lncRNA	exon	26052051	26052235	.	-	.	gene_id "lnc-bbug06220"	transcript_id "lnc-bbut00014800"	exon_number "4"
14	lncRNA	exon	26053783	26054709	.	-	.	gene_id "lnc-bbug06220"	transcript_id "lnc-bbut00014800"	exon_number "5"
14	lncRNA	exon	26024446	26027145	.	-	.	gene_id "lnc-bbug06220"	transcript_id "lnc-bbut00014799"	exon_number "1"
14	lncRNA	exon	26041916	26042049	.	-	.	gene_id "lnc-bbug06220"	transcript_id "lnc-bbut00014799"	exon_number "2"
14	lncRNA	exon	26045759	26045877	.	-	.	gene_id "lnc-bbug06220"	transcript_id "lnc-bbut00014799"	exon_number "3"

14	lncRNA	exon	26052051	26052235	.	-	.	gene_id "lnc-bbug06220"	transcript_id "lnc-bbut00014799"	exon_number "4"
14	lncRNA	exon	26053783	26054709	.	-	.	gene_id "lnc-bbug06220"	transcript_id "lnc-bbut00014799"	exon_number "5"
14	lncRNA	exon	26087732	26088242	.	-	.	gene_id "lnc-bbug06221"	transcript_id "lnc-bbut00014804"	exon_number "1"
14	lncRNA	exon	26089107	26089554	.	-	.	gene_id "lnc-bbug06221"	transcript_id "lnc-bbut00014804"	exon_number "2"
14	lncRNA	exon	26216507	26216659	.	-	.	gene_id "lnc-bbug06223"	transcript_id "lnc-bbut00014806"	exon_number "1"
14	lncRNA	exon	26218082	26218208	.	-	.	gene_id "lnc-bbug06223"	transcript_id "lnc-bbut00014806"	exon_number "2"
14	lncRNA	exon	26219908	26220062	.	-	.	gene_id "lnc-bbug06223"	transcript_id "lnc-bbut00014806"	exon_number "3"
14	lncRNA	exon	26748976	26749743	.	-	.	gene_id "lnc-bbug06225"	transcript_id "lnc-bbut00014810"	exon_number "1"
14	lncRNA	exon	26749826	26749906	.	-	.	gene_id "lnc-bbug06225"	transcript_id "lnc-bbut00014810"	exon_number "2"
14	lncRNA	exon	26754185	26756436	.	-	.	gene_id "lnc-bbug06225"	transcript_id "lnc-bbut00014810"	exon_number "3"
14	lncRNA	exon	28439795	28439991	.	-	.	gene_id "lnc-bbug06228"	transcript_id "lnc-bbut00014825"	exon_number "1"
14	lncRNA	exon	28444105	28447733	.	-	.	gene_id "lnc-bbug06228"	transcript_id "lnc-bbut00014825"	exon_number "2"
14	lncRNA	exon	28863151	28863890	.	-	.	gene_id "lnc-bbug06236"	transcript_id "lnc-bbut00014838"	exon_number "1"
14	lncRNA	exon	28867949	28868068	.	-	.	gene_id "lnc-bbug06236"	transcript_id "lnc-bbut00014838"	exon_number "2"
14	lncRNA	exon	28901643	28903758	.	-	.	gene_id "lnc-bbug06237"	transcript_id "lnc-bbut00014839"	exon_number "1"
14	lncRNA	exon	28903881	28904103	.	-	.	gene_id "lnc-bbug06237"	transcript_id "lnc-bbut00014839"	exon_number "2"
14	lncRNA	exon	28901643	28903758	.	-	.	gene_id "lnc-bbug06237"	transcript_id "lnc-bbut00014840"	exon_number "1"
14	lncRNA	exon	28921648	28921985	.	-	.	gene_id "lnc-bbug06237"	transcript_id "lnc-bbut00014840"	exon_number "2"
14	lncRNA	exon	29149886	29151076	.	-	.	gene_id "lnc-bbug06240"	transcript_id "lnc-bbut00014853"	exon_number "1"
14	lncRNA	exon	29153866	29156577	.	-	.	gene_id "lnc-bbug06240"	transcript_id "lnc-bbut00014853"	exon_number "2"
14	lncRNA	exon	29149886	29151076	.	-	.	gene_id "lnc-bbug06240"	transcript_id "lnc-bbut00014854"	exon_number "1"
14	lncRNA	exon	29161467	29161558	.	-	.	gene_id "lnc-bbug06240"	transcript_id "lnc-bbut00014854"	exon_number "2"
14	lncRNA	exon	29288992	29289027	.	-	.	gene_id "lnc-bbug06243"	transcript_id "lnc-bbut00014859"	exon_number "1"
14	lncRNA	exon	29289616	29290376	.	-	.	gene_id "lnc-bbug06243"	transcript_id "lnc-bbut00014859"	exon_number "2"
14	lncRNA	exon	29990420	29994091	.	-	.	gene_id "lnc-bbug06261"	transcript_id "lnc-bbut00014916"	exon_number "1"
14	lncRNA	exon	29995755	29995829	.	-	.	gene_id "lnc-bbug06261"	transcript_id "lnc-bbut00014916"	exon_number "2"
14	lncRNA	exon	29996654	29996770	.	-	.	gene_id "lnc-bbug06261"	transcript_id "lnc-bbut00014916"	exon_number "3"
14	lncRNA	exon	29999978	30000068	.	-	.	gene_id "lnc-bbug06261"	transcript_id "lnc-bbut00014916"	exon_number "4"
14	lncRNA	exon	30000624	30000722	.	-	.	gene_id "lnc-bbug06261"	transcript_id "lnc-bbut00014916"	exon_number "5"
14	lncRNA	exon	30003157	30003221	.	-	.	gene_id "lnc-bbug06261"	transcript_id "lnc-bbut00014916"	exon_number "6"
14	lncRNA	exon	30006388	30006655	.	-	.	gene_id "lnc-bbug06261"	transcript_id "lnc-bbut00014916"	exon_number "7"
14	lncRNA	exon	30066056	30066898	.	-	.	gene_id "lnc-bbug06262"	transcript_id "lnc-bbut00014919"	exon_number "1"
14	lncRNA	exon	30067056	30067682	.	-	.	gene_id "lnc-bbug06262"	transcript_id "lnc-bbut00014919"	exon_number "2"
14	lncRNA	exon	30353150	30354066	.	-	.	gene_id "lnc-bbug06267"	transcript_id "lnc-bbut00014927"	exon_number "1"
14	lncRNA	exon	30359602	30359667	.	-	.	gene_id "lnc-bbug06267"	transcript_id "lnc-bbut00014927"	exon_number "2"
14	lncRNA	exon	30362280	30362714	.	-	.	gene_id "lnc-bbug06267"	transcript_id "lnc-bbut00014927"	exon_number "3"
14	lncRNA	exon	30820926	30821752	.	-	.	gene_id "lnc-bbug06269"	transcript_id "lnc-bbut00014930"	exon_number "1"
14	lncRNA	exon	32397802	32402073	.	-	.	gene_id "lnc-bbug06286"	transcript_id "lnc-bbut00015004"	exon_number "1"
14	lncRNA	exon	32402858	32402937	.	-	.	gene_id "lnc-bbug06286"	transcript_id "lnc-bbut00015004"	exon_number "2"
14	lncRNA	exon	32424451	32424492	.	-	.	gene_id "lnc-bbug06286"	transcript_id "lnc-bbut00015004"	exon_number "3"
14	lncRNA	exon	32426610	32426652	.	-	.	gene_id "lnc-bbug06286"	transcript_id "lnc-bbut00015004"	exon_number "4"
14	lncRNA	exon	32439848	32439997	.	-	.	gene_id "lnc-bbug06286"	transcript_id "lnc-bbut00015004"	exon_number "5"
14	lncRNA	exon	32446252	32446420	.	-	.	gene_id "lnc-bbug06286"	transcript_id "lnc-bbut00015004"	exon_number "6"
14	lncRNA	exon	32397802	32402073	.	-	.	gene_id "lnc-bbug06286"	transcript_id "lnc-bbut00015005"	exon_number "1"
14	lncRNA	exon	32402858	32402937	.	-	.	gene_id "lnc-bbug06286"	transcript_id "lnc-bbut00015005"	exon_number "2"
14	lncRNA	exon	32424451	32424492	.	-	.	gene_id "lnc-bbug06286"	transcript_id "lnc-bbut00015005"	exon_number "3"
14	lncRNA	exon	32426610	32426652	.	-	.	gene_id "lnc-bbug06286"	transcript_id "lnc-bbut00015005"	exon_number "4"
14	lncRNA	exon	32439848	32439997	.	-	.	gene_id "lnc-bbug06286"	transcript_id "lnc-bbut00015005"	exon_number "5"
14	lncRNA	exon	32505875	32506020	.	-	.	gene_id "lnc-bbug06286"	transcript_id "lnc-bbut00015005"	exon_number "6"
14	lncRNA	exon	34375952	34376233	.	-	.	gene_id "lnc-bbug06290"	transcript_id "lnc-bbut00015009"	exon_number "1"
14	lncRNA	exon	34377322	34378109	.	-	.	gene_id "lnc-bbug06290"	transcript_id "lnc-bbut00015009"	exon_number "2"
14	lncRNA	exon	35768302	35768576	.	-	.	gene_id "lnc-bbug06295"	transcript_id "lnc-bbut00015023"	exon_number "1"

14	lncRNA	exon	35824582	35824763	.	-	.	gene_id "lnc-bbug06295"	transcript_id "lnc-bbut00015023"	exon_number "2"
14	lncRNA	exon	35837159	35837286	.	-	.	gene_id "lnc-bbug06295"	transcript_id "lnc-bbut00015023"	exon_number "3"
14	lncRNA	exon	35785152	35786097	.	-	.	gene_id "lnc-bbug06295"	transcript_id "lnc-bbut00015024"	exon_number "1"
14	lncRNA	exon	35824582	35824763	.	-	.	gene_id "lnc-bbug06295"	transcript_id "lnc-bbut00015024"	exon_number "2"
14	lncRNA	exon	35837159	35837286	.	-	.	gene_id "lnc-bbug06295"	transcript_id "lnc-bbut00015024"	exon_number "3"
14	lncRNA	exon	35823390	35824490	.	-	.	gene_id "lnc-bbug06295"	transcript_id "lnc-bbut00015025"	exon_number "1"
14	lncRNA	exon	35824582	35824763	.	-	.	gene_id "lnc-bbug06295"	transcript_id "lnc-bbut00015025"	exon_number "2"
14	lncRNA	exon	35837159	35837286	.	-	.	gene_id "lnc-bbug06295"	transcript_id "lnc-bbut00015025"	exon_number "3"
14	lncRNA	exon	36426063	36426097	.	-	.	gene_id "lnc-bbug06298"	transcript_id "lnc-bbut00015028"	exon_number "1"
14	lncRNA	exon	36432077	36432285	.	-	.	gene_id "lnc-bbug06298"	transcript_id "lnc-bbut00015028"	exon_number "2"
14	lncRNA	exon	38603463	38603580	.	-	.	gene_id "lnc-bbug06307"	transcript_id "lnc-bbut00015058"	exon_number "1"
14	lncRNA	exon	38604014	38604248	.	-	.	gene_id "lnc-bbug06307"	transcript_id "lnc-bbut00015058"	exon_number "2"
14	lncRNA	exon	38609286	38609332	.	-	.	gene_id "lnc-bbug06307"	transcript_id "lnc-bbut00015058"	exon_number "3"
14	lncRNA	exon	38610054	38610490	.	-	.	gene_id "lnc-bbug06308"	transcript_id "lnc-bbut00015059"	exon_number "1"
14	lncRNA	exon	38614974	38615101	.	-	.	gene_id "lnc-bbug06308"	transcript_id "lnc-bbut00015059"	exon_number "2"
14	lncRNA	exon	38615438	38616478	.	-	.	gene_id "lnc-bbug06308"	transcript_id "lnc-bbut00015059"	exon_number "3"
14	lncRNA	exon	38610054	38610490	.	-	.	gene_id "lnc-bbug06308"	transcript_id "lnc-bbut00015061"	exon_number "1"
14	lncRNA	exon	38614974	38615101	.	-	.	gene_id "lnc-bbug06308"	transcript_id "lnc-bbut00015061"	exon_number "2"
14	lncRNA	exon	38615438	38615575	.	-	.	gene_id "lnc-bbug06308"	transcript_id "lnc-bbut00015061"	exon_number "3"
14	lncRNA	exon	38617424	38617530	.	-	.	gene_id "lnc-bbug06308"	transcript_id "lnc-bbut00015061"	exon_number "4"
14	lncRNA	exon	38618542	38620739	.	-	.	gene_id "lnc-bbug06308"	transcript_id "lnc-bbut00015061"	exon_number "5"
14	lncRNA	exon	38610054	38610490	.	-	.	gene_id "lnc-bbug06308"	transcript_id "lnc-bbut00015060"	exon_number "1"
14	lncRNA	exon	38614974	38615101	.	-	.	gene_id "lnc-bbug06308"	transcript_id "lnc-bbut00015060"	exon_number "2"
14	lncRNA	exon	38615438	38615575	.	-	.	gene_id "lnc-bbug06308"	transcript_id "lnc-bbut00015060"	exon_number "3"
14	lncRNA	exon	38618542	38620739	.	-	.	gene_id "lnc-bbug06308"	transcript_id "lnc-bbut00015060"	exon_number "4"
14	lncRNA	exon	38632338	38636002	.	-	.	gene_id "lnc-bbug06309"	transcript_id "lnc-bbut00015062"	exon_number "1"
14	lncRNA	exon	38638066	38638597	.	-	.	gene_id "lnc-bbug06309"	transcript_id "lnc-bbut00015062"	exon_number "2"
14	lncRNA	exon	39173334	39177060	.	-	.	gene_id "lnc-bbug06311"	transcript_id "lnc-bbut00015066"	exon_number "1"
14	lncRNA	exon	39183787	39184595	.	-	.	gene_id "lnc-bbug06311"	transcript_id "lnc-bbut00015066"	exon_number "2"
14	lncRNA	exon	39185681	39185719	.	-	.	gene_id "lnc-bbug06311"	transcript_id "lnc-bbut00015066"	exon_number "3"
14	lncRNA	exon	39173334	39183217	.	-	.	gene_id "lnc-bbug06311"	transcript_id "lnc-bbut00015068"	exon_number "1"
14	lncRNA	exon	39183787	39184595	.	-	.	gene_id "lnc-bbug06311"	transcript_id "lnc-bbut00015068"	exon_number "2"
14	lncRNA	exon	39185535	39185739	.	-	.	gene_id "lnc-bbug06311"	transcript_id "lnc-bbut00015068"	exon_number "3"
14	lncRNA	exon	39186069	39186152	.	-	.	gene_id "lnc-bbug06311"	transcript_id "lnc-bbut00015068"	exon_number "4"
14	lncRNA	exon	39173334	39177060	.	-	.	gene_id "lnc-bbug06311"	transcript_id "lnc-bbut00015069"	exon_number "1"
14	lncRNA	exon	39185606	39185739	.	-	.	gene_id "lnc-bbug06311"	transcript_id "lnc-bbut00015069"	exon_number "2"
14	lncRNA	exon	39186069	39186171	.	-	.	gene_id "lnc-bbug06311"	transcript_id "lnc-bbut00015069"	exon_number "3"
14	lncRNA	exon	39173334	39177060	.	-	.	gene_id "lnc-bbug06311"	transcript_id "lnc-bbut00015076"	exon_number "1"
14	lncRNA	exon	39178129	39185739	.	-	.	gene_id "lnc-bbug06311"	transcript_id "lnc-bbut00015076"	exon_number "2"
14	lncRNA	exon	39186069	39188954	.	-	.	gene_id "lnc-bbug06311"	transcript_id "lnc-bbut00015076"	exon_number "3"
14	lncRNA	exon	39173334	39177060	.	-	.	gene_id "lnc-bbug06311"	transcript_id "lnc-bbut00015078"	exon_number "1"
14	lncRNA	exon	39183787	39185739	.	-	.	gene_id "lnc-bbug06311"	transcript_id "lnc-bbut00015078"	exon_number "2"
14	lncRNA	exon	39186069	39188954	.	-	.	gene_id "lnc-bbug06311"	transcript_id "lnc-bbut00015078"	exon_number "3"
14	lncRNA	exon	39173334	39177060	.	-	.	gene_id "lnc-bbug06311"	transcript_id "lnc-bbut00015079"	exon_number "1"
14	lncRNA	exon	39185535	39185739	.	-	.	gene_id "lnc-bbug06311"	transcript_id "lnc-bbut00015079"	exon_number "2"
14	lncRNA	exon	39186069	39188954	.	-	.	gene_id "lnc-bbug06311"	transcript_id "lnc-bbut00015079"	exon_number "3"
14	lncRNA	exon	39173334	39177060	.	-	.	gene_id "lnc-bbug06311"	transcript_id "lnc-bbut00015077"	exon_number "1"
14	lncRNA	exon	39182288	39185739	.	-	.	gene_id "lnc-bbug06311"	transcript_id "lnc-bbut00015077"	exon_number "2"
14	lncRNA	exon	39186069	39188954	.	-	.	gene_id "lnc-bbug06311"	transcript_id "lnc-bbut00015077"	exon_number "3"
14	lncRNA	exon	39173334	39183217	.	-	.	gene_id "lnc-bbug06311"	transcript_id "lnc-bbut00015075"	exon_number "1"
14	lncRNA	exon	39183787	39188954	.	-	.	gene_id "lnc-bbug06311"	transcript_id "lnc-bbut00015075"	exon_number "2"
14	lncRNA	exon	39173334	39177060	.	-	.	gene_id "lnc-bbug06311"	transcript_id "lnc-bbut00015072"	exon_number "1"

14	lncRNA	exon	39178129	39184595	.	-	.	gene_id "lnc-bbug06311"	transcript_id "lnc-bbut00015072"	exon_number "2"
14	lncRNA	exon	39185535	39185739	.	-	.	gene_id "lnc-bbug06311"	transcript_id "lnc-bbut00015072"	exon_number "3"
14	lncRNA	exon	39186069	39188954	.	-	.	gene_id "lnc-bbug06311"	transcript_id "lnc-bbut00015072"	exon_number "4"
14	lncRNA	exon	39173334	39177060	.	-	.	gene_id "lnc-bbug06311"	transcript_id "lnc-bbut00015074"	exon_number "1"
14	lncRNA	exon	39183787	39185389	.	-	.	gene_id "lnc-bbug06311"	transcript_id "lnc-bbut00015074"	exon_number "2"
14	lncRNA	exon	39185606	39185739	.	-	.	gene_id "lnc-bbug06311"	transcript_id "lnc-bbut00015074"	exon_number "3"
14	lncRNA	exon	39186069	39188954	.	-	.	gene_id "lnc-bbug06311"	transcript_id "lnc-bbut00015074"	exon_number "4"
14	lncRNA	exon	39173334	39177060	.	-	.	gene_id "lnc-bbug06311"	transcript_id "lnc-bbut00015073"	exon_number "1"
14	lncRNA	exon	39178129	39185389	.	-	.	gene_id "lnc-bbug06311"	transcript_id "lnc-bbut00015073"	exon_number "2"
14	lncRNA	exon	39185606	39185739	.	-	.	gene_id "lnc-bbug06311"	transcript_id "lnc-bbut00015073"	exon_number "3"
14	lncRNA	exon	39186069	39188954	.	-	.	gene_id "lnc-bbug06311"	transcript_id "lnc-bbut00015073"	exon_number "4"
14	lncRNA	exon	39173334	39177060	.	-	.	gene_id "lnc-bbug06311"	transcript_id "lnc-bbut00015071"	exon_number "1"
14	lncRNA	exon	39178129	39183217	.	-	.	gene_id "lnc-bbug06311"	transcript_id "lnc-bbut00015071"	exon_number "2"
14	lncRNA	exon	39183787	39185739	.	-	.	gene_id "lnc-bbug06311"	transcript_id "lnc-bbut00015071"	exon_number "3"
14	lncRNA	exon	39186069	39188954	.	-	.	gene_id "lnc-bbug06311"	transcript_id "lnc-bbut00015071"	exon_number "4"
14	lncRNA	exon	39173334	39183217	.	-	.	gene_id "lnc-bbug06311"	transcript_id "lnc-bbut00015070"	exon_number "1"
14	lncRNA	exon	39183787	39185739	.	-	.	gene_id "lnc-bbug06311"	transcript_id "lnc-bbut00015070"	exon_number "2"
14	lncRNA	exon	39186069	39188954	.	-	.	gene_id "lnc-bbug06311"	transcript_id "lnc-bbut00015070"	exon_number "3"
14	lncRNA	exon	39234538	39235360	.	-	.	gene_id "lnc-bbug06313"	transcript_id "lnc-bbut00015082"	exon_number "1"
14	lncRNA	exon	39239906	39239948	.	-	.	gene_id "lnc-bbug06313"	transcript_id "lnc-bbut00015082"	exon_number "2"
14	lncRNA	exon	39234538	39235360	.	-	.	gene_id "lnc-bbug06313"	transcript_id "lnc-bbut00015083"	exon_number "1"
14	lncRNA	exon	39253230	39253440	.	-	.	gene_id "lnc-bbug06313"	transcript_id "lnc-bbut00015083"	exon_number "2"
14	lncRNA	exon	39948846	39949234	.	-	.	gene_id "lnc-bbug06316"	transcript_id "lnc-bbut00015086"	exon_number "1"
14	lncRNA	exon	39949813	39949978	.	-	.	gene_id "lnc-bbug06316"	transcript_id "lnc-bbut00015086"	exon_number "2"
14	lncRNA	exon	39954714	39954823	.	-	.	gene_id "lnc-bbug06316"	transcript_id "lnc-bbut00015086"	exon_number "3"
14	lncRNA	exon	39956457	39956579	.	-	.	gene_id "lnc-bbug06316"	transcript_id "lnc-bbut00015086"	exon_number "4"
14	lncRNA	exon	39958329	39958406	.	-	.	gene_id "lnc-bbug06316"	transcript_id "lnc-bbut00015086"	exon_number "5"
14	lncRNA	exon	39959342	39959467	.	-	.	gene_id "lnc-bbug06316"	transcript_id "lnc-bbut00015086"	exon_number "6"
14	lncRNA	exon	39961743	39961910	.	-	.	gene_id "lnc-bbug06316"	transcript_id "lnc-bbut00015086"	exon_number "7"
14	lncRNA	exon	39963868	39963951	.	-	.	gene_id "lnc-bbug06316"	transcript_id "lnc-bbut00015086"	exon_number "8"
14	lncRNA	exon	40229933	40229969	.	-	.	gene_id "lnc-bbug06323"	transcript_id "lnc-bbut00015099"	exon_number "1"
14	lncRNA	exon	40230501	40230583	.	-	.	gene_id "lnc-bbug06323"	transcript_id "lnc-bbut00015099"	exon_number "2"
14	lncRNA	exon	40233613	40233779	.	-	.	gene_id "lnc-bbug06323"	transcript_id "lnc-bbut00015099"	exon_number "3"
14	lncRNA	exon	40235016	40235124	.	-	.	gene_id "lnc-bbug06323"	transcript_id "lnc-bbut00015099"	exon_number "4"
14	lncRNA	exon	40235930	40236052	.	-	.	gene_id "lnc-bbug06323"	transcript_id "lnc-bbut00015099"	exon_number "5"
14	lncRNA	exon	40236477	40236554	.	-	.	gene_id "lnc-bbug06323"	transcript_id "lnc-bbut00015099"	exon_number "6"
14	lncRNA	exon	40247189	40247305	.	-	.	gene_id "lnc-bbug06323"	transcript_id "lnc-bbut00015099"	exon_number "7"
14	lncRNA	exon	40247577	40247716	.	-	.	gene_id "lnc-bbug06323"	transcript_id "lnc-bbut00015099"	exon_number "8"
14	lncRNA	exon	40249981	40250537	.	-	.	gene_id "lnc-bbug06323"	transcript_id "lnc-bbut00015099"	exon_number "9"
14	lncRNA	exon	40284843	40285009	.	-	.	gene_id "lnc-bbug06325"	transcript_id "lnc-bbut00015101"	exon_number "1"
14	lncRNA	exon	40286087	40286141	.	-	.	gene_id "lnc-bbug06325"	transcript_id "lnc-bbut00015101"	exon_number "2"
14	lncRNA	exon	40286291	40286500	.	-	.	gene_id "lnc-bbug06325"	transcript_id "lnc-bbut00015101"	exon_number "3"
14	lncRNA	exon	40297933	40299287	.	-	.	gene_id "lnc-bbug06327"	transcript_id "lnc-bbut00015103"	exon_number "1"
14	lncRNA	exon	40300232	40300402	.	-	.	gene_id "lnc-bbug06327"	transcript_id "lnc-bbut00015103"	exon_number "2"
14	lncRNA	exon	40592674	40596458	.	-	.	gene_id "lnc-bbug06337"	transcript_id "lnc-bbut00015132"	exon_number "1"
14	lncRNA	exon	40599459	40599765	.	-	.	gene_id "lnc-bbug06337"	transcript_id "lnc-bbut00015132"	exon_number "2"
14	lncRNA	exon	40607663	40608018	.	-	.	gene_id "lnc-bbug06337"	transcript_id "lnc-bbut00015132"	exon_number "3"
14	lncRNA	exon	41175943	41176056	.	-	.	gene_id "lnc-bbug06346"	transcript_id "lnc-bbut00015149"	exon_number "1"
14	lncRNA	exon	41176135	41176734	.	-	.	gene_id "lnc-bbug06346"	transcript_id "lnc-bbut00015149"	exon_number "2"
14	lncRNA	exon	41177814	41178867	.	-	.	gene_id "lnc-bbug06346"	transcript_id "lnc-bbut00015149"	exon_number "3"
14	lncRNA	exon	41744178	41745040	.	-	.	gene_id "lnc-bbug06351"	transcript_id "lnc-bbut00015159"	exon_number "1"
14	lncRNA	exon	41748537	41748650	.	-	.	gene_id "lnc-bbug06351"	transcript_id "lnc-bbut00015159"	exon_number "2"

14	lncRNA	exon	41752484	41753203	.	-	.	gene_id "lnc-bbug06351"	transcript_id "lnc-bbut00015159"	exon_number "3"
14	lncRNA	exon	41933098	41935577	.	-	.	gene_id "lnc-bbug06357"	transcript_id "lnc-bbut00015171"	exon_number "1"
14	lncRNA	exon	41946371	41946445	.	-	.	gene_id "lnc-bbug06357"	transcript_id "lnc-bbut00015171"	exon_number "2"
14	lncRNA	exon	41946575	41946810	.	-	.	gene_id "lnc-bbug06357"	transcript_id "lnc-bbut00015171"	exon_number "3"
14	lncRNA	exon	41942380	41946445	.	-	.	gene_id "lnc-bbug06357"	transcript_id "lnc-bbut00015172"	exon_number "1"
14	lncRNA	exon	41946575	41947033	.	-	.	gene_id "lnc-bbug06357"	transcript_id "lnc-bbut00015172"	exon_number "2"
14	lncRNA	exon	42603031	42603477	.	-	.	gene_id "lnc-bbug06360"	transcript_id "lnc-bbut00015175"	exon_number "1"
14	lncRNA	exon	42608543	42608703	.	-	.	gene_id "lnc-bbug06360"	transcript_id "lnc-bbut00015175"	exon_number "2"
14	lncRNA	exon	42610701	42610792	.	-	.	gene_id "lnc-bbug06360"	transcript_id "lnc-bbut00015175"	exon_number "3"
14	lncRNA	exon	42612064	42612241	.	-	.	gene_id "lnc-bbug06360"	transcript_id "lnc-bbut00015175"	exon_number "4"
14	lncRNA	exon	42631206	42631446	.	-	.	gene_id "lnc-bbug06360"	transcript_id "lnc-bbut00015175"	exon_number "5"
14	lncRNA	exon	45013617	45013971	.	-	.	gene_id "lnc-bbug06371"	transcript_id "lnc-bbut00015209"	exon_number "1"
14	lncRNA	exon	45023643	45024841	.	-	.	gene_id "lnc-bbug06371"	transcript_id "lnc-bbut00015209"	exon_number "2"
14	lncRNA	exon	45161432	45163935	.	-	.	gene_id "lnc-bbug06372"	transcript_id "lnc-bbut00015210"	exon_number "1"
14	lncRNA	exon	45172827	45173937	.	-	.	gene_id "lnc-bbug06373"	transcript_id "lnc-bbut00015211"	exon_number "1"
14	lncRNA	exon	45174099	45174736	.	-	.	gene_id "lnc-bbug06373"	transcript_id "lnc-bbut00015211"	exon_number "2"
14	lncRNA	exon	45272899	45273222	.	-	.	gene_id "lnc-bbug06375"	transcript_id "lnc-bbut00015213"	exon_number "1"
14	lncRNA	exon	45274343	45274537	.	-	.	gene_id "lnc-bbug06375"	transcript_id "lnc-bbut00015213"	exon_number "2"
14	lncRNA	exon	45529202	45529416	.	-	.	gene_id "lnc-bbug06382"	transcript_id "lnc-bbut00015231"	exon_number "1"
14	lncRNA	exon	45533024	45533217	.	-	.	gene_id "lnc-bbug06382"	transcript_id "lnc-bbut00015231"	exon_number "2"
14	lncRNA	exon	45664623	45665088	.	-	.	gene_id "lnc-bbug06384"	transcript_id "lnc-bbut00015234"	exon_number "1"
14	lncRNA	exon	45667080	45667205	.	-	.	gene_id "lnc-bbug06384"	transcript_id "lnc-bbut00015234"	exon_number "2"
14	lncRNA	exon	45703290	45703618	.	-	.	gene_id "lnc-bbug06385"	transcript_id "lnc-bbut00015235"	exon_number "1"
14	lncRNA	exon	45716510	45716681	.	-	.	gene_id "lnc-bbug06385"	transcript_id "lnc-bbut00015235"	exon_number "2"
14	lncRNA	exon	45787308	45787598	.	-	.	gene_id "lnc-bbug06386"	transcript_id "lnc-bbut00015236"	exon_number "1"
14	lncRNA	exon	45793119	45793250	.	-	.	gene_id "lnc-bbug06386"	transcript_id "lnc-bbut00015236"	exon_number "2"
14	lncRNA	exon	45813682	45814531	.	-	.	gene_id "lnc-bbug06386"	transcript_id "lnc-bbut00015236"	exon_number "3"
14	lncRNA	exon	45787308	45787598	.	-	.	gene_id "lnc-bbug06386"	transcript_id "lnc-bbut00015237"	exon_number "1"
14	lncRNA	exon	45793119	45793250	.	-	.	gene_id "lnc-bbug06386"	transcript_id "lnc-bbut00015237"	exon_number "2"
14	lncRNA	exon	45802347	45802417	.	-	.	gene_id "lnc-bbug06386"	transcript_id "lnc-bbut00015237"	exon_number "3"
14	lncRNA	exon	45813682	45814543	.	-	.	gene_id "lnc-bbug06386"	transcript_id "lnc-bbut00015237"	exon_number "4"
14	lncRNA	exon	46549894	46550216	.	-	.	gene_id "lnc-bbug06390"	transcript_id "lnc-bbut00015241"	exon_number "1"
14	lncRNA	exon	46552197	46552246	.	-	.	gene_id "lnc-bbug06390"	transcript_id "lnc-bbut00015241"	exon_number "2"
14	lncRNA	exon	46567694	46567785	.	-	.	gene_id "lnc-bbug06390"	transcript_id "lnc-bbut00015241"	exon_number "3"
14	lncRNA	exon	46588653	46588852	.	-	.	gene_id "lnc-bbug06390"	transcript_id "lnc-bbut00015241"	exon_number "4"
14	lncRNA	exon	46753144	46753904	.	-	.	gene_id "lnc-bbug06391"	transcript_id "lnc-bbut00015242"	exon_number "1"
14	lncRNA	exon	46754014	46754135	.	-	.	gene_id "lnc-bbug06391"	transcript_id "lnc-bbut00015242"	exon_number "2"
14	lncRNA	exon	46755662	46756488	.	-	.	gene_id "lnc-bbug06391"	transcript_id "lnc-bbut00015242"	exon_number "3"
14	lncRNA	exon	46757738	46757762	.	-	.	gene_id "lnc-bbug06391"	transcript_id "lnc-bbut00015242"	exon_number "4"
14	lncRNA	exon	46841270	46841748	.	-	.	gene_id "lnc-bbug06392"	transcript_id "lnc-bbut00015243"	exon_number "1"
14	lncRNA	exon	46851213	46851217	.	-	.	gene_id "lnc-bbug06392"	transcript_id "lnc-bbut00015243"	exon_number "2"
14	lncRNA	exon	48292552	48292763	.	-	.	gene_id "lnc-bbug06396"	transcript_id "lnc-bbut00015259"	exon_number "1"
14	lncRNA	exon	48294470	48294648	.	-	.	gene_id "lnc-bbug06396"	transcript_id "lnc-bbut00015259"	exon_number "2"
14	lncRNA	exon	48308611	48309106	.	-	.	gene_id "lnc-bbug06396"	transcript_id "lnc-bbut00015259"	exon_number "3"
14	lncRNA	exon	48364100	48364665	.	-	.	gene_id "lnc-bbug06397"	transcript_id "lnc-bbut00015260"	exon_number "1"
14	lncRNA	exon	48378084	48378237	.	-	.	gene_id "lnc-bbug06397"	transcript_id "lnc-bbut00015260"	exon_number "2"
14	lncRNA	exon	48413198	48413385	.	-	.	gene_id "lnc-bbug06397"	transcript_id "lnc-bbut00015260"	exon_number "3"
14	lncRNA	exon	48364100	48364665	.	-	.	gene_id "lnc-bbug06397"	transcript_id "lnc-bbut00015261"	exon_number "1"
14	lncRNA	exon	48378084	48378237	.	-	.	gene_id "lnc-bbug06397"	transcript_id "lnc-bbut00015261"	exon_number "2"
14	lncRNA	exon	48418441	48419025	.	-	.	gene_id "lnc-bbug06397"	transcript_id "lnc-bbut00015261"	exon_number "3"
14	lncRNA	exon	48403601	48403931	.	-	.	gene_id "lnc-bbug06398"	transcript_id "lnc-bbut00015262"	exon_number "1"
14	lncRNA	exon	48406058	48407111	.	-	.	gene_id "lnc-bbug06398"	transcript_id "lnc-bbut00015262"	exon_number "2"

14	lncRNA	exon	48419850	48420538	.	-	.	gene_id "lnc-bbug06399"	transcript_id "lnc-bbut00015264"	exon_number "1"
14	lncRNA	exon	48421747	48421901	.	-	.	gene_id "lnc-bbug06399"	transcript_id "lnc-bbut00015264"	exon_number "2"
14	lncRNA	exon	48423001	48423154	.	-	.	gene_id "lnc-bbug06399"	transcript_id "lnc-bbut00015264"	exon_number "3"
14	lncRNA	exon	48426743	48431799	.	-	.	gene_id "lnc-bbug06399"	transcript_id "lnc-bbut00015264"	exon_number "4"
14	lncRNA	exon	48455589	48455713	.	-	.	gene_id "lnc-bbug06399"	transcript_id "lnc-bbut00015264"	exon_number "5"
14	lncRNA	exon	48421218	48421901	.	-	.	gene_id "lnc-bbug06399"	transcript_id "lnc-bbut00015265"	exon_number "1"
14	lncRNA	exon	48423001	48423154	.	-	.	gene_id "lnc-bbug06399"	transcript_id "lnc-bbut00015265"	exon_number "2"
14	lncRNA	exon	48426743	48429699	.	-	.	gene_id "lnc-bbug06399"	transcript_id "lnc-bbut00015265"	exon_number "3"
14	lncRNA	exon	48455589	48455712	.	-	.	gene_id "lnc-bbug06399"	transcript_id "lnc-bbut00015265"	exon_number "4"
14	lncRNA	exon	48421218	48421921	.	-	.	gene_id "lnc-bbug06399"	transcript_id "lnc-bbut00015271"	exon_number "1"
14	lncRNA	exon	48423001	48423154	.	-	.	gene_id "lnc-bbug06399"	transcript_id "lnc-bbut00015271"	exon_number "2"
14	lncRNA	exon	48426743	48431799	.	-	.	gene_id "lnc-bbug06399"	transcript_id "lnc-bbut00015271"	exon_number "3"
14	lncRNA	exon	48455589	48455713	.	-	.	gene_id "lnc-bbug06399"	transcript_id "lnc-bbut00015271"	exon_number "4"
14	lncRNA	exon	48421218	48421901	.	-	.	gene_id "lnc-bbug06399"	transcript_id "lnc-bbut00015273"	exon_number "1"
14	lncRNA	exon	48423001	48423154	.	-	.	gene_id "lnc-bbug06399"	transcript_id "lnc-bbut00015273"	exon_number "2"
14	lncRNA	exon	48426743	48426816	.	-	.	gene_id "lnc-bbug06399"	transcript_id "lnc-bbut00015273"	exon_number "3"
14	lncRNA	exon	48429500	48431799	.	-	.	gene_id "lnc-bbug06399"	transcript_id "lnc-bbut00015273"	exon_number "4"
14	lncRNA	exon	48455589	48455713	.	-	.	gene_id "lnc-bbug06399"	transcript_id "lnc-bbut00015273"	exon_number "5"
14	lncRNA	exon	48421218	48421901	.	-	.	gene_id "lnc-bbug06399"	transcript_id "lnc-bbut00015274"	exon_number "1"
14	lncRNA	exon	48423001	48423154	.	-	.	gene_id "lnc-bbug06399"	transcript_id "lnc-bbut00015274"	exon_number "2"
14	lncRNA	exon	48426743	48429699	.	-	.	gene_id "lnc-bbug06399"	transcript_id "lnc-bbut00015274"	exon_number "3"
14	lncRNA	exon	48430966	48431799	.	-	.	gene_id "lnc-bbug06399"	transcript_id "lnc-bbut00015274"	exon_number "4"
14	lncRNA	exon	48455589	48455713	.	-	.	gene_id "lnc-bbug06399"	transcript_id "lnc-bbut00015274"	exon_number "5"
14	lncRNA	exon	48421218	48421901	.	-	.	gene_id "lnc-bbug06399"	transcript_id "lnc-bbut00015272"	exon_number "1"
14	lncRNA	exon	48423001	48423154	.	-	.	gene_id "lnc-bbug06399"	transcript_id "lnc-bbut00015272"	exon_number "2"
14	lncRNA	exon	48424666	48424806	.	-	.	gene_id "lnc-bbug06399"	transcript_id "lnc-bbut00015272"	exon_number "3"
14	lncRNA	exon	48426743	48431799	.	-	.	gene_id "lnc-bbug06399"	transcript_id "lnc-bbut00015272"	exon_number "4"
14	lncRNA	exon	48455589	48455713	.	-	.	gene_id "lnc-bbug06399"	transcript_id "lnc-bbut00015272"	exon_number "5"
14	lncRNA	exon	48421218	48421901	.	-	.	gene_id "lnc-bbug06399"	transcript_id "lnc-bbut00015270"	exon_number "1"
14	lncRNA	exon	48423001	48424806	.	-	.	gene_id "lnc-bbug06399"	transcript_id "lnc-bbut00015270"	exon_number "2"
14	lncRNA	exon	48426743	48431799	.	-	.	gene_id "lnc-bbug06399"	transcript_id "lnc-bbut00015270"	exon_number "3"
14	lncRNA	exon	48455589	48455713	.	-	.	gene_id "lnc-bbug06399"	transcript_id "lnc-bbut00015270"	exon_number "4"
14	lncRNA	exon	48421218	48421901	.	-	.	gene_id "lnc-bbug06399"	transcript_id "lnc-bbut00015268"	exon_number "1"
14	lncRNA	exon	48423001	48424806	.	-	.	gene_id "lnc-bbug06399"	transcript_id "lnc-bbut00015268"	exon_number "2"
14	lncRNA	exon	48426743	48426816	.	-	.	gene_id "lnc-bbug06399"	transcript_id "lnc-bbut00015268"	exon_number "3"
14	lncRNA	exon	48429500	48431799	.	-	.	gene_id "lnc-bbug06399"	transcript_id "lnc-bbut00015268"	exon_number "4"
14	lncRNA	exon	48455589	48455713	.	-	.	gene_id "lnc-bbug06399"	transcript_id "lnc-bbut00015268"	exon_number "5"
14	lncRNA	exon	48421218	48421901	.	-	.	gene_id "lnc-bbug06399"	transcript_id "lnc-bbut00015269"	exon_number "1"
14	lncRNA	exon	48423001	48423154	.	-	.	gene_id "lnc-bbug06399"	transcript_id "lnc-bbut00015269"	exon_number "2"
14	lncRNA	exon	48426743	48431799	.	-	.	gene_id "lnc-bbug06399"	transcript_id "lnc-bbut00015269"	exon_number "3"
14	lncRNA	exon	48455589	48455713	.	-	.	gene_id "lnc-bbug06399"	transcript_id "lnc-bbut00015269"	exon_number "4"
14	lncRNA	exon	48421218	48421901	.	-	.	gene_id "lnc-bbug06399"	transcript_id "lnc-bbut00015267"	exon_number "1"
14	lncRNA	exon	48423001	48424806	.	-	.	gene_id "lnc-bbug06399"	transcript_id "lnc-bbut00015267"	exon_number "2"
14	lncRNA	exon	48426743	48426805	.	-	.	gene_id "lnc-bbug06399"	transcript_id "lnc-bbut00015267"	exon_number "3"
14	lncRNA	exon	48429500	48431799	.	-	.	gene_id "lnc-bbug06399"	transcript_id "lnc-bbut00015267"	exon_number "4"
14	lncRNA	exon	48455589	48455713	.	-	.	gene_id "lnc-bbug06399"	transcript_id "lnc-bbut00015267"	exon_number "5"
14	lncRNA	exon	48421218	48421901	.	-	.	gene_id "lnc-bbug06399"	transcript_id "lnc-bbut00015266"	exon_number "1"
14	lncRNA	exon	48423001	48423154	.	-	.	gene_id "lnc-bbug06399"	transcript_id "lnc-bbut00015266"	exon_number "2"
14	lncRNA	exon	48426743	48429699	.	-	.	gene_id "lnc-bbug06399"	transcript_id "lnc-bbut00015266"	exon_number "3"
14	lncRNA	exon	48431665	48431799	.	-	.	gene_id "lnc-bbug06399"	transcript_id "lnc-bbut00015266"	exon_number "4"
14	lncRNA	exon	48455589	48455713	.	-	.	gene_id "lnc-bbug06399"	transcript_id "lnc-bbut00015266"	exon_number "5"
14	lncRNA	exon	48526678	48526761	.	-	.	gene_id "lnc-bbug06401"	transcript_id "lnc-bbut00015277"	exon_number "1"

14	lncRNA	exon	48532031	48532948	.	-	.	gene_id "lnc-bbug06401"	transcript_id "lnc-bbut00015277"	exon_number "2"
14	lncRNA	exon	48549083	48550961	.	-	.	gene_id "lnc-bbug06402"	transcript_id "lnc-bbut00015279"	exon_number "1"
14	lncRNA	exon	48555332	48556133	.	-	.	gene_id "lnc-bbug06402"	transcript_id "lnc-bbut00015279"	exon_number "2"
14	lncRNA	exon	48556523	48556600	.	-	.	gene_id "lnc-bbug06402"	transcript_id "lnc-bbut00015279"	exon_number "3"
14	lncRNA	exon	48560436	48560606	.	-	.	gene_id "lnc-bbug06402"	transcript_id "lnc-bbut00015279"	exon_number "4"
14	lncRNA	exon	48549083	48550961	.	-	.	gene_id "lnc-bbug06402"	transcript_id "lnc-bbut00015278"	exon_number "1"
14	lncRNA	exon	48556523	48556600	.	-	.	gene_id "lnc-bbug06402"	transcript_id "lnc-bbut00015278"	exon_number "2"
14	lncRNA	exon	48560436	48560606	.	-	.	gene_id "lnc-bbug06402"	transcript_id "lnc-bbut00015278"	exon_number "3"
14	lncRNA	exon	48947513	48949164	.	-	.	gene_id "lnc-bbug06403"	transcript_id "lnc-bbut00015280"	exon_number "1"
14	lncRNA	exon	48950121	48950206	.	-	.	gene_id "lnc-bbug06403"	transcript_id "lnc-bbut00015280"	exon_number "2"
14	lncRNA	exon	48950509	48950839	.	-	.	gene_id "lnc-bbug06403"	transcript_id "lnc-bbut00015280"	exon_number "3"
14	lncRNA	exon	49637204	49637738	.	-	.	gene_id "lnc-bbug06405"	transcript_id "lnc-bbut00015298"	exon_number "1"
14	lncRNA	exon	49658182	49658433	.	-	.	gene_id "lnc-bbug06405"	transcript_id "lnc-bbut00015298"	exon_number "2"
14	lncRNA	exon	49637204	49637738	.	-	.	gene_id "lnc-bbug06405"	transcript_id "lnc-bbut00015300"	exon_number "1"
14	lncRNA	exon	49677367	49677459	.	-	.	gene_id "lnc-bbug06405"	transcript_id "lnc-bbut00015300"	exon_number "2"
14	lncRNA	exon	49679943	49680037	.	-	.	gene_id "lnc-bbug06405"	transcript_id "lnc-bbut00015300"	exon_number "3"
14	lncRNA	exon	49717300	49717333	.	-	.	gene_id "lnc-bbug06405"	transcript_id "lnc-bbut00015300"	exon_number "4"
14	lncRNA	exon	49719967	49720074	.	-	.	gene_id "lnc-bbug06405"	transcript_id "lnc-bbut00015300"	exon_number "5"
14	lncRNA	exon	49725137	49725189	.	-	.	gene_id "lnc-bbug06405"	transcript_id "lnc-bbut00015300"	exon_number "6"
14	lncRNA	exon	49727028	49727347	.	-	.	gene_id "lnc-bbug06405"	transcript_id "lnc-bbut00015300"	exon_number "7"
14	lncRNA	exon	49637204	49637738	.	-	.	gene_id "lnc-bbug06405"	transcript_id "lnc-bbut00015299"	exon_number "1"
14	lncRNA	exon	49661590	49661760	.	-	.	gene_id "lnc-bbug06405"	transcript_id "lnc-bbut00015299"	exon_number "2"
14	lncRNA	exon	49677367	49677459	.	-	.	gene_id "lnc-bbug06405"	transcript_id "lnc-bbut00015299"	exon_number "3"
14	lncRNA	exon	49679943	49680037	.	-	.	gene_id "lnc-bbug06405"	transcript_id "lnc-bbut00015299"	exon_number "4"
14	lncRNA	exon	49717300	49717333	.	-	.	gene_id "lnc-bbug06405"	transcript_id "lnc-bbut00015299"	exon_number "5"
14	lncRNA	exon	49719967	49720074	.	-	.	gene_id "lnc-bbug06405"	transcript_id "lnc-bbut00015299"	exon_number "6"
14	lncRNA	exon	49725137	49725189	.	-	.	gene_id "lnc-bbug06405"	transcript_id "lnc-bbut00015299"	exon_number "7"
14	lncRNA	exon	49727028	49727347	.	-	.	gene_id "lnc-bbug06405"	transcript_id "lnc-bbut00015299"	exon_number "8"
14	lncRNA	exon	49672326	49672926	.	-	.	gene_id "lnc-bbug06405"	transcript_id "lnc-bbut00015301"	exon_number "1"
14	lncRNA	exon	49677367	49677459	.	-	.	gene_id "lnc-bbug06405"	transcript_id "lnc-bbut00015301"	exon_number "2"
14	lncRNA	exon	49679943	49680037	.	-	.	gene_id "lnc-bbug06405"	transcript_id "lnc-bbut00015301"	exon_number "3"
14	lncRNA	exon	49717300	49717333	.	-	.	gene_id "lnc-bbug06405"	transcript_id "lnc-bbut00015301"	exon_number "4"
14	lncRNA	exon	49719967	49720074	.	-	.	gene_id "lnc-bbug06405"	transcript_id "lnc-bbut00015301"	exon_number "5"
14	lncRNA	exon	49725137	49725189	.	-	.	gene_id "lnc-bbug06405"	transcript_id "lnc-bbut00015301"	exon_number "6"
14	lncRNA	exon	49727028	49727347	.	-	.	gene_id "lnc-bbug06405"	transcript_id "lnc-bbut00015301"	exon_number "7"
14	lncRNA	exon	49673761	49673977	.	-	.	gene_id "lnc-bbug06405"	transcript_id "lnc-bbut00015303"	exon_number "1"
14	lncRNA	exon	49677367	49677459	.	-	.	gene_id "lnc-bbug06405"	transcript_id "lnc-bbut00015303"	exon_number "2"
14	lncRNA	exon	49678440	49678501	.	-	.	gene_id "lnc-bbug06405"	transcript_id "lnc-bbut00015303"	exon_number "3"
14	lncRNA	exon	49679943	49680037	.	-	.	gene_id "lnc-bbug06405"	transcript_id "lnc-bbut00015303"	exon_number "4"
14	lncRNA	exon	49717300	49717333	.	-	.	gene_id "lnc-bbug06405"	transcript_id "lnc-bbut00015303"	exon_number "5"
14	lncRNA	exon	49719967	49720074	.	-	.	gene_id "lnc-bbug06405"	transcript_id "lnc-bbut00015303"	exon_number "6"
14	lncRNA	exon	49725137	49725189	.	-	.	gene_id "lnc-bbug06405"	transcript_id "lnc-bbut00015303"	exon_number "7"
14	lncRNA	exon	49727028	49727347	.	-	.	gene_id "lnc-bbug06405"	transcript_id "lnc-bbut00015303"	exon_number "8"
14	lncRNA	exon	49673761	49673977	.	-	.	gene_id "lnc-bbug06405"	transcript_id "lnc-bbut00015302"	exon_number "1"
14	lncRNA	exon	49677367	49677459	.	-	.	gene_id "lnc-bbug06405"	transcript_id "lnc-bbut00015302"	exon_number "2"
14	lncRNA	exon	49679943	49680037	.	-	.	gene_id "lnc-bbug06405"	transcript_id "lnc-bbut00015302"	exon_number "3"
14	lncRNA	exon	49717300	49717333	.	-	.	gene_id "lnc-bbug06405"	transcript_id "lnc-bbut00015302"	exon_number "4"
14	lncRNA	exon	49719967	49720074	.	-	.	gene_id "lnc-bbug06405"	transcript_id "lnc-bbut00015302"	exon_number "5"
14	lncRNA	exon	49725137	49725189	.	-	.	gene_id "lnc-bbug06405"	transcript_id "lnc-bbut00015302"	exon_number "6"
14	lncRNA	exon	49727028	49727347	.	-	.	gene_id "lnc-bbug06405"	transcript_id "lnc-bbut00015302"	exon_number "7"
14	lncRNA	exon	49675438	49676598	.	-	.	gene_id "lnc-bbug06405"	transcript_id "lnc-bbut00015304"	exon_number "1"
14	lncRNA	exon	49677367	49677459	.	-	.	gene_id "lnc-bbug06405"	transcript_id "lnc-bbut00015304"	exon_number "2"

14	lncRNA	exon	49679943	49680037	.	-	.	gene_id "lnc-bbug06405"	transcript_id "lnc-bbut00015304"	exon_number "3"
14	lncRNA	exon	49703455	49706771	.	-	.	gene_id "lnc-bbug06405"	transcript_id "lnc-bbut00015304"	exon_number "4"
14	lncRNA	exon	49675438	49676598	.	-	.	gene_id "lnc-bbug06405"	transcript_id "lnc-bbut00015305"	exon_number "1"
14	lncRNA	exon	49677367	49677459	.	-	.	gene_id "lnc-bbug06405"	transcript_id "lnc-bbut00015305"	exon_number "2"
14	lncRNA	exon	49679943	49680037	.	-	.	gene_id "lnc-bbug06405"	transcript_id "lnc-bbut00015305"	exon_number "3"
14	lncRNA	exon	49694869	49694949	.	-	.	gene_id "lnc-bbug06405"	transcript_id "lnc-bbut00015305"	exon_number "4"
14	lncRNA	exon	49703455	49706777	.	-	.	gene_id "lnc-bbug06405"	transcript_id "lnc-bbut00015305"	exon_number "5"
14	lncRNA	exon	49675438	49676598	.	-	.	gene_id "lnc-bbug06405"	transcript_id "lnc-bbut00015316"	exon_number "1"
14	lncRNA	exon	49677367	49677459	.	-	.	gene_id "lnc-bbug06405"	transcript_id "lnc-bbut00015316"	exon_number "2"
14	lncRNA	exon	49678440	49678501	.	-	.	gene_id "lnc-bbug06405"	transcript_id "lnc-bbut00015316"	exon_number "3"
14	lncRNA	exon	49679943	49680037	.	-	.	gene_id "lnc-bbug06405"	transcript_id "lnc-bbut00015316"	exon_number "4"
14	lncRNA	exon	49694869	49694949	.	-	.	gene_id "lnc-bbug06405"	transcript_id "lnc-bbut00015316"	exon_number "5"
14	lncRNA	exon	49717300	49717333	.	-	.	gene_id "lnc-bbug06405"	transcript_id "lnc-bbut00015316"	exon_number "6"
14	lncRNA	exon	49719967	49720074	.	-	.	gene_id "lnc-bbug06405"	transcript_id "lnc-bbut00015316"	exon_number "7"
14	lncRNA	exon	49725137	49725189	.	-	.	gene_id "lnc-bbug06405"	transcript_id "lnc-bbut00015316"	exon_number "8"
14	lncRNA	exon	49727028	49727347	.	-	.	gene_id "lnc-bbug06405"	transcript_id "lnc-bbut00015316"	exon_number "9"
14	lncRNA	exon	49675438	49676590	.	-	.	gene_id "lnc-bbug06405"	transcript_id "lnc-bbut00015315"	exon_number "1"
14	lncRNA	exon	49677367	49677459	.	-	.	gene_id "lnc-bbug06405"	transcript_id "lnc-bbut00015315"	exon_number "2"
14	lncRNA	exon	49679943	49680037	.	-	.	gene_id "lnc-bbug06405"	transcript_id "lnc-bbut00015315"	exon_number "3"
14	lncRNA	exon	49717300	49717333	.	-	.	gene_id "lnc-bbug06405"	transcript_id "lnc-bbut00015315"	exon_number "4"
14	lncRNA	exon	49719967	49720074	.	-	.	gene_id "lnc-bbug06405"	transcript_id "lnc-bbut00015315"	exon_number "5"
14	lncRNA	exon	49725137	49725189	.	-	.	gene_id "lnc-bbug06405"	transcript_id "lnc-bbut00015315"	exon_number "6"
14	lncRNA	exon	49727028	49727347	.	-	.	gene_id "lnc-bbug06405"	transcript_id "lnc-bbut00015315"	exon_number "7"
14	lncRNA	exon	49675438	49676598	.	-	.	gene_id "lnc-bbug06405"	transcript_id "lnc-bbut00015310"	exon_number "1"
14	lncRNA	exon	49677367	49677459	.	-	.	gene_id "lnc-bbug06405"	transcript_id "lnc-bbut00015310"	exon_number "2"
14	lncRNA	exon	49678440	49678501	.	-	.	gene_id "lnc-bbug06405"	transcript_id "lnc-bbut00015310"	exon_number "3"
14	lncRNA	exon	49679943	49680037	.	-	.	gene_id "lnc-bbug06405"	transcript_id "lnc-bbut00015310"	exon_number "4"
14	lncRNA	exon	49685078	49685227	.	-	.	gene_id "lnc-bbug06405"	transcript_id "lnc-bbut00015310"	exon_number "5"
14	lncRNA	exon	49694869	49694949	.	-	.	gene_id "lnc-bbug06405"	transcript_id "lnc-bbut00015310"	exon_number "6"
14	lncRNA	exon	49717300	49717333	.	-	.	gene_id "lnc-bbug06405"	transcript_id "lnc-bbut00015310"	exon_number "7"
14	lncRNA	exon	49719967	49720074	.	-	.	gene_id "lnc-bbug06405"	transcript_id "lnc-bbut00015310"	exon_number "8"
14	lncRNA	exon	49725137	49725189	.	-	.	gene_id "lnc-bbug06405"	transcript_id "lnc-bbut00015310"	exon_number "9"
14	lncRNA	exon	49727028	49727347	.	-	.	gene_id "lnc-bbug06405"	transcript_id "lnc-bbut00015310"	exon_number "10"
14	lncRNA	exon	49675438	49676598	.	-	.	gene_id "lnc-bbug06405"	transcript_id "lnc-bbut00015311"	exon_number "1"
14	lncRNA	exon	49677367	49677459	.	-	.	gene_id "lnc-bbug06405"	transcript_id "lnc-bbut00015311"	exon_number "2"
14	lncRNA	exon	49679943	49680037	.	-	.	gene_id "lnc-bbug06405"	transcript_id "lnc-bbut00015311"	exon_number "3"
14	lncRNA	exon	49685078	49685227	.	-	.	gene_id "lnc-bbug06405"	transcript_id "lnc-bbut00015311"	exon_number "4"
14	lncRNA	exon	49694869	49694949	.	-	.	gene_id "lnc-bbug06405"	transcript_id "lnc-bbut00015311"	exon_number "5"
14	lncRNA	exon	49717300	49717333	.	-	.	gene_id "lnc-bbug06405"	transcript_id "lnc-bbut00015311"	exon_number "6"
14	lncRNA	exon	49719967	49720074	.	-	.	gene_id "lnc-bbug06405"	transcript_id "lnc-bbut00015311"	exon_number "7"
14	lncRNA	exon	49725137	49725189	.	-	.	gene_id "lnc-bbug06405"	transcript_id "lnc-bbut00015311"	exon_number "8"
14	lncRNA	exon	49727028	49727347	.	-	.	gene_id "lnc-bbug06405"	transcript_id "lnc-bbut00015311"	exon_number "9"
14	lncRNA	exon	49675438	49676598	.	-	.	gene_id "lnc-bbug06405"	transcript_id "lnc-bbut00015312"	exon_number "1"
14	lncRNA	exon	49677367	49677459	.	-	.	gene_id "lnc-bbug06405"	transcript_id "lnc-bbut00015312"	exon_number "2"
14	lncRNA	exon	49679943	49680037	.	-	.	gene_id "lnc-bbug06405"	transcript_id "lnc-bbut00015312"	exon_number "3"
14	lncRNA	exon	49719967	49720074	.	-	.	gene_id "lnc-bbug06405"	transcript_id "lnc-bbut00015312"	exon_number "4"
14	lncRNA	exon	49725137	49725189	.	-	.	gene_id "lnc-bbug06405"	transcript_id "lnc-bbut00015312"	exon_number "5"
14	lncRNA	exon	49727028	49727347	.	-	.	gene_id "lnc-bbug06405"	transcript_id "lnc-bbut00015312"	exon_number "6"
14	lncRNA	exon	49675438	49676598	.	-	.	gene_id "lnc-bbug06405"	transcript_id "lnc-bbut00015314"	exon_number "1"
14	lncRNA	exon	49677367	49677459	.	-	.	gene_id "lnc-bbug06405"	transcript_id "lnc-bbut00015314"	exon_number "2"
14	lncRNA	exon	49679943	49680037	.	-	.	gene_id "lnc-bbug06405"	transcript_id "lnc-bbut00015314"	exon_number "3"
14	lncRNA	exon	49685078	49685227	.	-	.	gene_id "lnc-bbug06405"	transcript_id "lnc-bbut00015314"	exon_number "4"

14	lncRNA	exon	49694869	49694949	.	-	.	gene_id "lnc-bbug06405"	transcript_id "lnc-bbut00015314"	exon_number "5"
14	lncRNA	exon	49717300	49717333	.	-	.	gene_id "lnc-bbug06405"	transcript_id "lnc-bbut00015314"	exon_number "6"
14	lncRNA	exon	49719173	49719253	.	-	.	gene_id "lnc-bbug06405"	transcript_id "lnc-bbut00015314"	exon_number "7"
14	lncRNA	exon	49719967	49720074	.	-	.	gene_id "lnc-bbug06405"	transcript_id "lnc-bbut00015314"	exon_number "8"
14	lncRNA	exon	49725137	49725189	.	-	.	gene_id "lnc-bbug06405"	transcript_id "lnc-bbut00015314"	exon_number "9"
14	lncRNA	exon	49727028	49727347	.	-	.	gene_id "lnc-bbug06405"	transcript_id "lnc-bbut00015314"	exon_number "10"
14	lncRNA	exon	49675438	49676598	.	-	.	gene_id "lnc-bbug06405"	transcript_id "lnc-bbut00015313"	exon_number "1"
14	lncRNA	exon	49677367	49677459	.	-	.	gene_id "lnc-bbug06405"	transcript_id "lnc-bbut00015313"	exon_number "2"
14	lncRNA	exon	49679943	49680037	.	-	.	gene_id "lnc-bbug06405"	transcript_id "lnc-bbut00015313"	exon_number "3"
14	lncRNA	exon	49717300	49717333	.	-	.	gene_id "lnc-bbug06405"	transcript_id "lnc-bbut00015313"	exon_number "4"
14	lncRNA	exon	49719967	49720074	.	-	.	gene_id "lnc-bbug06405"	transcript_id "lnc-bbut00015313"	exon_number "5"
14	lncRNA	exon	49727028	49727347	.	-	.	gene_id "lnc-bbug06405"	transcript_id "lnc-bbut00015313"	exon_number "6"
14	lncRNA	exon	49675438	49676598	.	-	.	gene_id "lnc-bbug06405"	transcript_id "lnc-bbut00015309"	exon_number "1"
14	lncRNA	exon	49677367	49677459	.	-	.	gene_id "lnc-bbug06405"	transcript_id "lnc-bbut00015309"	exon_number "2"
14	lncRNA	exon	49679943	49680037	.	-	.	gene_id "lnc-bbug06405"	transcript_id "lnc-bbut00015309"	exon_number "3"
14	lncRNA	exon	49717300	49717333	.	-	.	gene_id "lnc-bbug06405"	transcript_id "lnc-bbut00015309"	exon_number "4"
14	lncRNA	exon	49719967	49720074	.	-	.	gene_id "lnc-bbug06405"	transcript_id "lnc-bbut00015309"	exon_number "5"
14	lncRNA	exon	49725137	49725189	.	-	.	gene_id "lnc-bbug06405"	transcript_id "lnc-bbut00015309"	exon_number "6"
14	lncRNA	exon	49727028	49727347	.	-	.	gene_id "lnc-bbug06405"	transcript_id "lnc-bbut00015309"	exon_number "7"
14	lncRNA	exon	49675438	49676598	.	-	.	gene_id "lnc-bbug06405"	transcript_id "lnc-bbut00015308"	exon_number "1"
14	lncRNA	exon	49677367	49677459	.	-	.	gene_id "lnc-bbug06405"	transcript_id "lnc-bbut00015308"	exon_number "2"
14	lncRNA	exon	49678440	49678501	.	-	.	gene_id "lnc-bbug06405"	transcript_id "lnc-bbut00015308"	exon_number "3"
14	lncRNA	exon	49679943	49680037	.	-	.	gene_id "lnc-bbug06405"	transcript_id "lnc-bbut00015308"	exon_number "4"
14	lncRNA	exon	49717300	49717333	.	-	.	gene_id "lnc-bbug06405"	transcript_id "lnc-bbut00015308"	exon_number "5"
14	lncRNA	exon	49719967	49720074	.	-	.	gene_id "lnc-bbug06405"	transcript_id "lnc-bbut00015308"	exon_number "6"
14	lncRNA	exon	49725137	49725189	.	-	.	gene_id "lnc-bbug06405"	transcript_id "lnc-bbut00015308"	exon_number "7"
14	lncRNA	exon	49727028	49727347	.	-	.	gene_id "lnc-bbug06405"	transcript_id "lnc-bbut00015308"	exon_number "8"
14	lncRNA	exon	49675438	49676598	.	-	.	gene_id "lnc-bbug06405"	transcript_id "lnc-bbut00015307"	exon_number "1"
14	lncRNA	exon	49677367	49677459	.	-	.	gene_id "lnc-bbug06405"	transcript_id "lnc-bbut00015307"	exon_number "2"
14	lncRNA	exon	49679943	49680037	.	-	.	gene_id "lnc-bbug06405"	transcript_id "lnc-bbut00015307"	exon_number "3"
14	lncRNA	exon	49694869	49694949	.	-	.	gene_id "lnc-bbug06405"	transcript_id "lnc-bbut00015307"	exon_number "4"
14	lncRNA	exon	49717300	49717333	.	-	.	gene_id "lnc-bbug06405"	transcript_id "lnc-bbut00015307"	exon_number "5"
14	lncRNA	exon	49719967	49720074	.	-	.	gene_id "lnc-bbug06405"	transcript_id "lnc-bbut00015307"	exon_number "6"
14	lncRNA	exon	49725137	49725189	.	-	.	gene_id "lnc-bbug06405"	transcript_id "lnc-bbut00015307"	exon_number "7"
14	lncRNA	exon	49727028	49727347	.	-	.	gene_id "lnc-bbug06405"	transcript_id "lnc-bbut00015307"	exon_number "8"
14	lncRNA	exon	49675438	49676598	.	-	.	gene_id "lnc-bbug06405"	transcript_id "lnc-bbut00015306"	exon_number "1"
14	lncRNA	exon	49677367	49677459	.	-	.	gene_id "lnc-bbug06405"	transcript_id "lnc-bbut00015306"	exon_number "2"
14	lncRNA	exon	49679943	49680037	.	-	.	gene_id "lnc-bbug06405"	transcript_id "lnc-bbut00015306"	exon_number "3"
14	lncRNA	exon	49717300	49717333	.	-	.	gene_id "lnc-bbug06405"	transcript_id "lnc-bbut00015306"	exon_number "4"
14	lncRNA	exon	49719173	49719253	.	-	.	gene_id "lnc-bbug06405"	transcript_id "lnc-bbut00015306"	exon_number "5"
14	lncRNA	exon	49719967	49720074	.	-	.	gene_id "lnc-bbug06405"	transcript_id "lnc-bbut00015306"	exon_number "6"
14	lncRNA	exon	49725137	49725189	.	-	.	gene_id "lnc-bbug06405"	transcript_id "lnc-bbut00015306"	exon_number "7"
14	lncRNA	exon	49727028	49727347	.	-	.	gene_id "lnc-bbug06405"	transcript_id "lnc-bbut00015306"	exon_number "8"
14	lncRNA	exon	49675438	49676598	.	-	.	gene_id "lnc-bbug06405"	transcript_id "lnc-bbut00015319"	exon_number "1"
14	lncRNA	exon	49677367	49677459	.	-	.	gene_id "lnc-bbug06405"	transcript_id "lnc-bbut00015319"	exon_number "2"
14	lncRNA	exon	49679943	49680037	.	-	.	gene_id "lnc-bbug06405"	transcript_id "lnc-bbut00015319"	exon_number "3"
14	lncRNA	exon	49831329	49831560	.	-	.	gene_id "lnc-bbug06405"	transcript_id "lnc-bbut00015319"	exon_number "4"
14	lncRNA	exon	49675438	49676598	.	-	.	gene_id "lnc-bbug06405"	transcript_id "lnc-bbut00015318"	exon_number "1"
14	lncRNA	exon	49677367	49677459	.	-	.	gene_id "lnc-bbug06405"	transcript_id "lnc-bbut00015318"	exon_number "2"
14	lncRNA	exon	49679943	49680037	.	-	.	gene_id "lnc-bbug06405"	transcript_id "lnc-bbut00015318"	exon_number "3"
14	lncRNA	exon	49717300	49717333	.	-	.	gene_id "lnc-bbug06405"	transcript_id "lnc-bbut00015318"	exon_number "4"
14	lncRNA	exon	49719967	49720074	.	-	.	gene_id "lnc-bbug06405"	transcript_id "lnc-bbut00015318"	exon_number "5"

14	lncRNA	exon	49831204	49831560	.	-	.	gene_id "lnc-bbug06405"	transcript_id "lnc-bbut00015318"	exon_number "6"
14	lncRNA	exon	49675438	49676598	.	-	.	gene_id "lnc-bbug06405"	transcript_id "lnc-bbut00015317"	exon_number "1"
14	lncRNA	exon	49677367	49677459	.	-	.	gene_id "lnc-bbug06405"	transcript_id "lnc-bbut00015317"	exon_number "2"
14	lncRNA	exon	49679943	49680037	.	-	.	gene_id "lnc-bbug06405"	transcript_id "lnc-bbut00015317"	exon_number "3"
14	lncRNA	exon	49717300	49717333	.	-	.	gene_id "lnc-bbug06405"	transcript_id "lnc-bbut00015317"	exon_number "4"
14	lncRNA	exon	49719967	49720074	.	-	.	gene_id "lnc-bbug06405"	transcript_id "lnc-bbut00015317"	exon_number "5"
14	lncRNA	exon	49831329	49831560	.	-	.	gene_id "lnc-bbug06405"	transcript_id "lnc-bbut00015317"	exon_number "6"
14	lncRNA	exon	50006824	50008314	.	-	.	gene_id "lnc-bbug06406"	transcript_id "lnc-bbut00015320"	exon_number "1"
14	lncRNA	exon	50013324	50013656	.	-	.	gene_id "lnc-bbug06406"	transcript_id "lnc-bbut00015320"	exon_number "2"
14	lncRNA	exon	50019007	50019075	.	-	.	gene_id "lnc-bbug06406"	transcript_id "lnc-bbut00015320"	exon_number "3"
14	lncRNA	exon	50068283	50068359	.	-	.	gene_id "lnc-bbug06406"	transcript_id "lnc-bbut00015320"	exon_number "4"
14	lncRNA	exon	50068523	50073526	.	-	.	gene_id "lnc-bbug06406"	transcript_id "lnc-bbut00015320"	exon_number "5"
14	lncRNA	exon	50074087	50074348	.	-	.	gene_id "lnc-bbug06406"	transcript_id "lnc-bbut00015320"	exon_number "6"
14	lncRNA	exon	50075432	50076439	.	-	.	gene_id "lnc-bbug06406"	transcript_id "lnc-bbut00015320"	exon_number "7"
14	lncRNA	exon	50079621	50079668	.	-	.	gene_id "lnc-bbug06407"	transcript_id "lnc-bbut00015321"	exon_number "1"
14	lncRNA	exon	50084237	50084331	.	-	.	gene_id "lnc-bbug06407"	transcript_id "lnc-bbut00015321"	exon_number "2"
14	lncRNA	exon	50085521	50085734	.	-	.	gene_id "lnc-bbug06407"	transcript_id "lnc-bbut00015321"	exon_number "3"
14	lncRNA	exon	50085883	50086903	.	-	.	gene_id "lnc-bbug06407"	transcript_id "lnc-bbut00015321"	exon_number "4"
14	lncRNA	exon	50079621	50079668	.	-	.	gene_id "lnc-bbug06407"	transcript_id "lnc-bbut00015322"	exon_number "1"
14	lncRNA	exon	50084237	50084331	.	-	.	gene_id "lnc-bbug06407"	transcript_id "lnc-bbut00015322"	exon_number "2"
14	lncRNA	exon	50085521	50085734	.	-	.	gene_id "lnc-bbug06407"	transcript_id "lnc-bbut00015322"	exon_number "3"
14	lncRNA	exon	50086947	50088429	.	-	.	gene_id "lnc-bbug06407"	transcript_id "lnc-bbut00015322"	exon_number "4"
14	lncRNA	exon	51640754	51640974	.	-	.	gene_id "lnc-bbug06416"	transcript_id "lnc-bbut00015356"	exon_number "1"
14	lncRNA	exon	51641302	51641342	.	-	.	gene_id "lnc-bbug06416"	transcript_id "lnc-bbut00015356"	exon_number "2"
14	lncRNA	exon	51706238	51706290	.	-	.	gene_id "lnc-bbug06418"	transcript_id "lnc-bbut00015361"	exon_number "1"
14	lncRNA	exon	51708069	51708213	.	-	.	gene_id "lnc-bbug06418"	transcript_id "lnc-bbut00015361"	exon_number "2"
14	lncRNA	exon	51708541	51708620	.	-	.	gene_id "lnc-bbug06418"	transcript_id "lnc-bbut00015361"	exon_number "3"
14	lncRNA	exon	51725210	51725352	.	-	.	gene_id "lnc-bbug06418"	transcript_id "lnc-bbut00015361"	exon_number "4"
14	lncRNA	exon	53094538	53094870	.	-	.	gene_id "lnc-bbug06421"	transcript_id "lnc-bbut00015368"	exon_number "1"
14	lncRNA	exon	53537948	53538248	.	-	.	gene_id "lnc-bbug06422"	transcript_id "lnc-bbut00015370"	exon_number "1"
14	lncRNA	exon	53539440	53539547	.	-	.	gene_id "lnc-bbug06422"	transcript_id "lnc-bbut00015370"	exon_number "2"
14	lncRNA	exon	53539903	53540015	.	-	.	gene_id "lnc-bbug06422"	transcript_id "lnc-bbut00015370"	exon_number "3"
14	lncRNA	exon	53540488	53541175	.	-	.	gene_id "lnc-bbug06422"	transcript_id "lnc-bbut00015370"	exon_number "4"
14	lncRNA	exon	53541656	53541860	.	-	.	gene_id "lnc-bbug06422"	transcript_id "lnc-bbut00015370"	exon_number "5"
14	lncRNA	exon	53537948	53538248	.	-	.	gene_id "lnc-bbug06422"	transcript_id "lnc-bbut00015369"	exon_number "1"
14	lncRNA	exon	53539440	53539547	.	-	.	gene_id "lnc-bbug06422"	transcript_id "lnc-bbut00015369"	exon_number "2"
14	lncRNA	exon	53539903	53540015	.	-	.	gene_id "lnc-bbug06422"	transcript_id "lnc-bbut00015369"	exon_number "3"
14	lncRNA	exon	53540488	53541860	.	-	.	gene_id "lnc-bbug06422"	transcript_id "lnc-bbut00015369"	exon_number "4"
14	lncRNA	exon	54202269	54202587	.	-	.	gene_id "lnc-bbug06425"	transcript_id "lnc-bbut00015373"	exon_number "1"
14	lncRNA	exon	54206741	54206907	.	-	.	gene_id "lnc-bbug06425"	transcript_id "lnc-bbut00015373"	exon_number "2"
14	lncRNA	exon	54218753	54218989	.	-	.	gene_id "lnc-bbug06425"	transcript_id "lnc-bbut00015373"	exon_number "3"
14	lncRNA	exon	54257788	54258848	.	-	.	gene_id "lnc-bbug06426"	transcript_id "lnc-bbut00015374"	exon_number "1"
14	lncRNA	exon	54259564	54259674	.	-	.	gene_id "lnc-bbug06426"	transcript_id "lnc-bbut00015374"	exon_number "2"
14	lncRNA	exon	55447746	55448658	.	-	.	gene_id "lnc-bbug06429"	transcript_id "lnc-bbut00015383"	exon_number "1"
14	lncRNA	exon	55449234	55449427	.	-	.	gene_id "lnc-bbug06429"	transcript_id "lnc-bbut00015383"	exon_number "2"
14	lncRNA	exon	55450957	55452161	.	-	.	gene_id "lnc-bbug06429"	transcript_id "lnc-bbut00015383"	exon_number "3"
14	lncRNA	exon	55455214	55455320	.	-	.	gene_id "lnc-bbug06429"	transcript_id "lnc-bbut00015383"	exon_number "4"
14	lncRNA	exon	55456412	55456654	.	-	.	gene_id "lnc-bbug06429"	transcript_id "lnc-bbut00015383"	exon_number "5"
14	lncRNA	exon	55458754	55458878	.	-	.	gene_id "lnc-bbug06429"	transcript_id "lnc-bbut00015383"	exon_number "6"
14	lncRNA	exon	56946108	56947042	.	-	.	gene_id "lnc-bbug06434"	transcript_id "lnc-bbut00015398"	exon_number "1"
14	lncRNA	exon	56947180	56947259	.	-	.	gene_id "lnc-bbug06434"	transcript_id "lnc-bbut00015398"	exon_number "2"
14	lncRNA	exon	56979316	56979435	.	-	.	gene_id "lnc-bbug06434"	transcript_id "lnc-bbut00015398"	exon_number "3"

14	lncRNA	exon	56990409	56990538	.	-	.	gene_id "lnc-bbug06434"	transcript_id "lnc-bbut00015398"	exon_number "4"
14	lncRNA	exon	57981677	57981727	.	-	.	gene_id "lnc-bbug06439"	transcript_id "lnc-bbut00015403"	exon_number "1"
14	lncRNA	exon	57989246	57989412	.	-	.	gene_id "lnc-bbug06439"	transcript_id "lnc-bbut00015403"	exon_number "2"
14	lncRNA	exon	57995106	57995282	.	-	.	gene_id "lnc-bbug06439"	transcript_id "lnc-bbut00015403"	exon_number "3"
14	lncRNA	exon	59301941	59305603	.	-	.	gene_id "lnc-bbug06442"	transcript_id "lnc-bbut00015424"	exon_number "1"
14	lncRNA	exon	59306768	59306863	.	-	.	gene_id "lnc-bbug06442"	transcript_id "lnc-bbut00015424"	exon_number "2"
14	lncRNA	exon	59321874	59322033	.	-	.	gene_id "lnc-bbug06442"	transcript_id "lnc-bbut00015424"	exon_number "3"
14	lncRNA	exon	59421772	59421842	.	-	.	gene_id "lnc-bbug06442"	transcript_id "lnc-bbut00015424"	exon_number "4"
14	lncRNA	exon	59426073	59426568	.	-	.	gene_id "lnc-bbug06442"	transcript_id "lnc-bbut00015424"	exon_number "5"
14	lncRNA	exon	59301941	59302932	.	-	.	gene_id "lnc-bbug06442"	transcript_id "lnc-bbut00015425"	exon_number "1"
14	lncRNA	exon	59303717	59305603	.	-	.	gene_id "lnc-bbug06442"	transcript_id "lnc-bbut00015425"	exon_number "2"
14	lncRNA	exon	59306768	59306863	.	-	.	gene_id "lnc-bbug06442"	transcript_id "lnc-bbut00015425"	exon_number "3"
14	lncRNA	exon	59321874	59322033	.	-	.	gene_id "lnc-bbug06442"	transcript_id "lnc-bbut00015425"	exon_number "4"
14	lncRNA	exon	59421772	59421842	.	-	.	gene_id "lnc-bbug06442"	transcript_id "lnc-bbut00015425"	exon_number "5"
14	lncRNA	exon	59426073	59427011	.	-	.	gene_id "lnc-bbug06442"	transcript_id "lnc-bbut00015425"	exon_number "6"
14	lncRNA	exon	59301941	59305603	.	-	.	gene_id "lnc-bbug06442"	transcript_id "lnc-bbut00015426"	exon_number "1"
14	lncRNA	exon	59306768	59306863	.	-	.	gene_id "lnc-bbug06442"	transcript_id "lnc-bbut00015426"	exon_number "2"
14	lncRNA	exon	59311964	59312005	.	-	.	gene_id "lnc-bbug06442"	transcript_id "lnc-bbut00015426"	exon_number "3"
14	lncRNA	exon	59321874	59322033	.	-	.	gene_id "lnc-bbug06442"	transcript_id "lnc-bbut00015426"	exon_number "4"
14	lncRNA	exon	59421772	59421842	.	-	.	gene_id "lnc-bbug06442"	transcript_id "lnc-bbut00015426"	exon_number "5"
14	lncRNA	exon	59426073	59427020	.	-	.	gene_id "lnc-bbug06442"	transcript_id "lnc-bbut00015426"	exon_number "6"
14	lncRNA	exon	59301941	59305603	.	-	.	gene_id "lnc-bbug06442"	transcript_id "lnc-bbut00015427"	exon_number "1"
14	lncRNA	exon	59306768	59306863	.	-	.	gene_id "lnc-bbug06442"	transcript_id "lnc-bbut00015427"	exon_number "2"
14	lncRNA	exon	59321874	59322033	.	-	.	gene_id "lnc-bbug06442"	transcript_id "lnc-bbut00015427"	exon_number "3"
14	lncRNA	exon	59421772	59421842	.	-	.	gene_id "lnc-bbug06442"	transcript_id "lnc-bbut00015427"	exon_number "4"
14	lncRNA	exon	59427446	59428179	.	-	.	gene_id "lnc-bbug06442"	transcript_id "lnc-bbut00015427"	exon_number "5"
14	lncRNA	exon	59301941	59305603	.	-	.	gene_id "lnc-bbug06442"	transcript_id "lnc-bbut00015428"	exon_number "1"
14	lncRNA	exon	59306768	59306863	.	-	.	gene_id "lnc-bbug06442"	transcript_id "lnc-bbut00015428"	exon_number "2"
14	lncRNA	exon	59321874	59322033	.	-	.	gene_id "lnc-bbug06442"	transcript_id "lnc-bbut00015428"	exon_number "3"
14	lncRNA	exon	59421772	59421842	.	-	.	gene_id "lnc-bbug06442"	transcript_id "lnc-bbut00015428"	exon_number "4"
14	lncRNA	exon	59427446	59427538	.	-	.	gene_id "lnc-bbug06442"	transcript_id "lnc-bbut00015428"	exon_number "5"
14	lncRNA	exon	59429841	59430202	.	-	.	gene_id "lnc-bbug06442"	transcript_id "lnc-bbut00015428"	exon_number "6"
14	lncRNA	exon	59301941	59305603	.	-	.	gene_id "lnc-bbug06442"	transcript_id "lnc-bbut00015429"	exon_number "1"
14	lncRNA	exon	59306768	59306863	.	-	.	gene_id "lnc-bbug06442"	transcript_id "lnc-bbut00015429"	exon_number "2"
14	lncRNA	exon	59321874	59322033	.	-	.	gene_id "lnc-bbug06442"	transcript_id "lnc-bbut00015429"	exon_number "3"
14	lncRNA	exon	59421772	59421842	.	-	.	gene_id "lnc-bbug06442"	transcript_id "lnc-bbut00015429"	exon_number "4"
14	lncRNA	exon	59447759	59448916	.	-	.	gene_id "lnc-bbug06442"	transcript_id "lnc-bbut00015429"	exon_number "5"
14	lncRNA	exon	59635693	59636144	.	-	.	gene_id "lnc-bbug06445"	transcript_id "lnc-bbut00015432"	exon_number "1"
14	lncRNA	exon	59636986	59637333	.	-	.	gene_id "lnc-bbug06445"	transcript_id "lnc-bbut00015432"	exon_number "2"
14	lncRNA	exon	59637611	59637739	.	-	.	gene_id "lnc-bbug06445"	transcript_id "lnc-bbut00015432"	exon_number "3"
14	lncRNA	exon	60174184	60174508	.	-	.	gene_id "lnc-bbug06449"	transcript_id "lnc-bbut00015450"	exon_number "1"
14	lncRNA	exon	60175715	60175859	.	-	.	gene_id "lnc-bbug06449"	transcript_id "lnc-bbut00015450"	exon_number "2"
14	lncRNA	exon	60190613	60190840	.	-	.	gene_id "lnc-bbug06449"	transcript_id "lnc-bbut00015450"	exon_number "3"
14	lncRNA	exon	60192862	60192911	.	-	.	gene_id "lnc-bbug06449"	transcript_id "lnc-bbut00015450"	exon_number "4"
14	lncRNA	exon	60202076	60202497	.	-	.	gene_id "lnc-bbug06449"	transcript_id "lnc-bbut00015450"	exon_number "5"
14	lncRNA	exon	60174184	60174508	.	-	.	gene_id "lnc-bbug06449"	transcript_id "lnc-bbut00015449"	exon_number "1"
14	lncRNA	exon	60175715	60175859	.	-	.	gene_id "lnc-bbug06449"	transcript_id "lnc-bbut00015449"	exon_number "2"
14	lncRNA	exon	60190613	60190840	.	-	.	gene_id "lnc-bbug06449"	transcript_id "lnc-bbut00015449"	exon_number "3"
14	lncRNA	exon	60202076	60202497	.	-	.	gene_id "lnc-bbug06449"	transcript_id "lnc-bbut00015449"	exon_number "4"
14	lncRNA	exon	60237465	60237574	.	-	.	gene_id "lnc-bbug06450"	transcript_id "lnc-bbut00015451"	exon_number "1"
14	lncRNA	exon	60242742	60242851	.	-	.	gene_id "lnc-bbug06450"	transcript_id "lnc-bbut00015451"	exon_number "2"
14	lncRNA	exon	60245779	60245808	.	-	.	gene_id "lnc-bbug06450"	transcript_id "lnc-bbut00015451"	exon_number "3"

14	lncRNA	exon	60250887	60250946	.	-	.	gene_id "lnc-bbug06450"	transcript_id "lnc-bbut00015451"	exon_number "4"
14	lncRNA	exon	60255101	60255933	.	-	.	gene_id "lnc-bbug06450"	transcript_id "lnc-bbut00015451"	exon_number "5"
14	lncRNA	exon	61693601	61694196	.	-	.	gene_id "lnc-bbug06456"	transcript_id "lnc-bbut00015466"	exon_number "1"
14	lncRNA	exon	63671700	63672557	.	-	.	gene_id "lnc-bbug06460"	transcript_id "lnc-bbut00015476"	exon_number "1"
14	lncRNA	exon	63980916	63984375	.	-	.	gene_id "lnc-bbug06462"	transcript_id "lnc-bbut00015480"	exon_number "1"
14	lncRNA	exon	64047792	64047953	.	-	.	gene_id "lnc-bbug06462"	transcript_id "lnc-bbut00015480"	exon_number "2"
14	lncRNA	exon	64084221	64084493	.	-	.	gene_id "lnc-bbug06462"	transcript_id "lnc-bbut00015480"	exon_number "3"
14	lncRNA	exon	63991178	63991427	.	-	.	gene_id "lnc-bbug06462"	transcript_id "lnc-bbut00015481"	exon_number "1"
14	lncRNA	exon	64047792	64047953	.	-	.	gene_id "lnc-bbug06462"	transcript_id "lnc-bbut00015481"	exon_number "2"
14	lncRNA	exon	64084221	64084492	.	-	.	gene_id "lnc-bbug06462"	transcript_id "lnc-bbut00015481"	exon_number "3"
14	lncRNA	exon	63991178	63991427	.	-	.	gene_id "lnc-bbug06462"	transcript_id "lnc-bbut00015482"	exon_number "1"
14	lncRNA	exon	63992128	63992299	.	-	.	gene_id "lnc-bbug06462"	transcript_id "lnc-bbut00015482"	exon_number "2"
14	lncRNA	exon	64047792	64047953	.	-	.	gene_id "lnc-bbug06462"	transcript_id "lnc-bbut00015482"	exon_number "3"
14	lncRNA	exon	64084221	64084495	.	-	.	gene_id "lnc-bbug06462"	transcript_id "lnc-bbut00015482"	exon_number "4"
14	lncRNA	exon	64577677	64578812	.	-	.	gene_id "lnc-bbug06464"	transcript_id "lnc-bbut00015490"	exon_number "1"
14	lncRNA	exon	64603399	64603571	.	-	.	gene_id "lnc-bbug06464"	transcript_id "lnc-bbut00015490"	exon_number "2"
14	lncRNA	exon	64604447	64604670	.	-	.	gene_id "lnc-bbug06464"	transcript_id "lnc-bbut00015490"	exon_number "3"
14	lncRNA	exon	64616736	64616843	.	-	.	gene_id "lnc-bbug06464"	transcript_id "lnc-bbut00015490"	exon_number "4"
14	lncRNA	exon	64577677	64578812	.	-	.	gene_id "lnc-bbug06464"	transcript_id "lnc-bbut00015489"	exon_number "1"
14	lncRNA	exon	64603399	64603571	.	-	.	gene_id "lnc-bbug06464"	transcript_id "lnc-bbut00015489"	exon_number "2"
14	lncRNA	exon	64604447	64604567	.	-	.	gene_id "lnc-bbug06464"	transcript_id "lnc-bbut00015489"	exon_number "3"
14	lncRNA	exon	64616736	64616843	.	-	.	gene_id "lnc-bbug06464"	transcript_id "lnc-bbut00015489"	exon_number "4"
14	lncRNA	exon	64577677	64578812	.	-	.	gene_id "lnc-bbug06464"	transcript_id "lnc-bbut00015488"	exon_number "1"
14	lncRNA	exon	64603399	64603514	.	-	.	gene_id "lnc-bbug06464"	transcript_id "lnc-bbut00015488"	exon_number "2"
14	lncRNA	exon	64604447	64604567	.	-	.	gene_id "lnc-bbug06464"	transcript_id "lnc-bbut00015488"	exon_number "3"
14	lncRNA	exon	64616736	64616843	.	-	.	gene_id "lnc-bbug06464"	transcript_id "lnc-bbut00015488"	exon_number "4"
14	lncRNA	exon	64600881	64602424	.	-	.	gene_id "lnc-bbug06464"	transcript_id "lnc-bbut00015491"	exon_number "1"
14	lncRNA	exon	64603399	64603571	.	-	.	gene_id "lnc-bbug06464"	transcript_id "lnc-bbut00015491"	exon_number "2"
14	lncRNA	exon	64604447	64604567	.	-	.	gene_id "lnc-bbug06464"	transcript_id "lnc-bbut00015491"	exon_number "3"
14	lncRNA	exon	64616736	64616843	.	-	.	gene_id "lnc-bbug06464"	transcript_id "lnc-bbut00015491"	exon_number "4"
14	lncRNA	exon	64735847	64735889	.	-	.	gene_id "lnc-bbug06465"	transcript_id "lnc-bbut00015492"	exon_number "1"
14	lncRNA	exon	64800523	64800694	.	-	.	gene_id "lnc-bbug06465"	transcript_id "lnc-bbut00015492"	exon_number "2"
14	lncRNA	exon	64802974	64803093	.	-	.	gene_id "lnc-bbug06465"	transcript_id "lnc-bbut00015492"	exon_number "3"
14	lncRNA	exon	65817545	65818311	.	-	.	gene_id "lnc-bbug06469"	transcript_id "lnc-bbut00015508"	exon_number "1"
14	lncRNA	exon	65822049	65822152	.	-	.	gene_id "lnc-bbug06469"	transcript_id "lnc-bbut00015508"	exon_number "2"
14	lncRNA	exon	66133367	66133933	.	-	.	gene_id "lnc-bbug06473"	transcript_id "lnc-bbut00015531"	exon_number "1"
14	lncRNA	exon	66140548	66141284	.	-	.	gene_id "lnc-bbug06474"	transcript_id "lnc-bbut00015532"	exon_number "1"
14	lncRNA	exon	67159963	67161906	.	-	.	gene_id "lnc-bbug06479"	transcript_id "lnc-bbut00015546"	exon_number "1"
14	lncRNA	exon	70458730	70459088	.	-	.	gene_id "lnc-bbug06484"	transcript_id "lnc-bbut00015561"	exon_number "1"
14	lncRNA	exon	70459475	70459587	.	-	.	gene_id "lnc-bbug06484"	transcript_id "lnc-bbut00015561"	exon_number "2"
14	lncRNA	exon	70460159	70460264	.	-	.	gene_id "lnc-bbug06484"	transcript_id "lnc-bbut00015561"	exon_number "3"
14	lncRNA	exon	70506593	70510117	.	-	.	gene_id "lnc-bbug06485"	transcript_id "lnc-bbut00015562"	exon_number "1"
14	lncRNA	exon	70510533	70510646	.	-	.	gene_id "lnc-bbug06485"	transcript_id "lnc-bbut00015562"	exon_number "2"
14	lncRNA	exon	70527044	70527682	.	-	.	gene_id "lnc-bbug06485"	transcript_id "lnc-bbut00015562"	exon_number "3"
14	lncRNA	exon	70695202	70695624	.	-	.	gene_id "lnc-bbug06486"	transcript_id "lnc-bbut00015563"	exon_number "1"
14	lncRNA	exon	70695887	70696010	.	-	.	gene_id "lnc-bbug06486"	transcript_id "lnc-bbut00015563"	exon_number "2"
14	lncRNA	exon	70699094	70699515	.	-	.	gene_id "lnc-bbug06486"	transcript_id "lnc-bbut00015563"	exon_number "3"
14	lncRNA	exon	71164684	71165206	.	-	.	gene_id "lnc-bbug06491"	transcript_id "lnc-bbut00015575"	exon_number "1"
14	lncRNA	exon	71168117	71168272	.	-	.	gene_id "lnc-bbug06491"	transcript_id "lnc-bbut00015575"	exon_number "2"
14	lncRNA	exon	71178404	71179047	.	-	.	gene_id "lnc-bbug06491"	transcript_id "lnc-bbut00015575"	exon_number "3"
14	lncRNA	exon	71913538	71913643	.	-	.	gene_id "lnc-bbug06493"	transcript_id "lnc-bbut00015577"	exon_number "1"
14	lncRNA	exon	71921517	71921742	.	-	.	gene_id "lnc-bbug06493"	transcript_id "lnc-bbut00015577"	exon_number "2"

14	lncRNA	exon	71925449	71925559	.	-	.	gene_id "lnc-bbug06493"	transcript_id "lnc-bbut00015577"	exon_number "3"
14	lncRNA	exon	71952556	71955455	.	-	.	gene_id "lnc-bbug06494"	transcript_id "lnc-bbut00015578"	exon_number "1"
14	lncRNA	exon	72020163	72020307	.	-	.	gene_id "lnc-bbug06494"	transcript_id "lnc-bbut00015578"	exon_number "2"
14	lncRNA	exon	72021780	72021857	.	-	.	gene_id "lnc-bbug06494"	transcript_id "lnc-bbut00015578"	exon_number "3"
14	lncRNA	exon	72024803	72024958	.	-	.	gene_id "lnc-bbug06494"	transcript_id "lnc-bbut00015578"	exon_number "4"
14	lncRNA	exon	72025163	72027028	.	-	.	gene_id "lnc-bbug06494"	transcript_id "lnc-bbut00015578"	exon_number "5"
14	lncRNA	exon	71975593	71975658	.	-	.	gene_id "lnc-bbug06494"	transcript_id "lnc-bbut00015579"	exon_number "1"
14	lncRNA	exon	72020163	72020307	.	-	.	gene_id "lnc-bbug06494"	transcript_id "lnc-bbut00015579"	exon_number "2"
14	lncRNA	exon	72021780	72021857	.	-	.	gene_id "lnc-bbug06494"	transcript_id "lnc-bbut00015579"	exon_number "3"
14	lncRNA	exon	72024803	72024958	.	-	.	gene_id "lnc-bbug06494"	transcript_id "lnc-bbut00015579"	exon_number "4"
14	lncRNA	exon	72025163	72027028	.	-	.	gene_id "lnc-bbug06494"	transcript_id "lnc-bbut00015579"	exon_number "5"
14	lncRNA	exon	72019853	72020307	.	-	.	gene_id "lnc-bbug06494"	transcript_id "lnc-bbut00015580"	exon_number "1"
14	lncRNA	exon	72021780	72021857	.	-	.	gene_id "lnc-bbug06494"	transcript_id "lnc-bbut00015580"	exon_number "2"
14	lncRNA	exon	72024803	72024958	.	-	.	gene_id "lnc-bbug06494"	transcript_id "lnc-bbut00015580"	exon_number "3"
14	lncRNA	exon	72025163	72027029	.	-	.	gene_id "lnc-bbug06494"	transcript_id "lnc-bbut00015580"	exon_number "4"
14	lncRNA	exon	72291082	72291987	.	-	.	gene_id "lnc-bbug06497"	transcript_id "lnc-bbut00015587"	exon_number "1"
14	lncRNA	exon	72293928	72293985	.	-	.	gene_id "lnc-bbug06497"	transcript_id "lnc-bbut00015587"	exon_number "2"
14	lncRNA	exon	72298734	72298785	.	-	.	gene_id "lnc-bbug06497"	transcript_id "lnc-bbut00015587"	exon_number "3"
14	lncRNA	exon	72291082	72291870	.	-	.	gene_id "lnc-bbug06497"	transcript_id "lnc-bbut00015588"	exon_number "1"
14	lncRNA	exon	72293928	72293985	.	-	.	gene_id "lnc-bbug06497"	transcript_id "lnc-bbut00015588"	exon_number "2"
14	lncRNA	exon	72298734	72298799	.	-	.	gene_id "lnc-bbug06497"	transcript_id "lnc-bbut00015588"	exon_number "3"
14	lncRNA	exon	72974557	72977203	.	-	.	gene_id "lnc-bbug06505"	transcript_id "lnc-bbut00015612"	exon_number "1"
14	lncRNA	exon	72986054	72986102	.	-	.	gene_id "lnc-bbug06506"	transcript_id "lnc-bbut00015616"	exon_number "1"
14	lncRNA	exon	72994514	72994607	.	-	.	gene_id "lnc-bbug06506"	transcript_id "lnc-bbut00015616"	exon_number "2"
14	lncRNA	exon	72995355	72995426	.	-	.	gene_id "lnc-bbug06506"	transcript_id "lnc-bbut00015616"	exon_number "3"
14	lncRNA	exon	72998337	72999538	.	-	.	gene_id "lnc-bbug06506"	transcript_id "lnc-bbut00015616"	exon_number "4"
14	lncRNA	exon	73016611	73017599	.	-	.	gene_id "lnc-bbug06506"	transcript_id "lnc-bbut00015616"	exon_number "5"
14	lncRNA	exon	73018671	73019005	.	-	.	gene_id "lnc-bbug06506"	transcript_id "lnc-bbut00015616"	exon_number "6"
14	lncRNA	exon	73586189	73586668	.	-	.	gene_id "lnc-bbug06509"	transcript_id "lnc-bbut00015621"	exon_number "1"
14	lncRNA	exon	77870657	77870990	.	-	.	gene_id "lnc-bbug06515"	transcript_id "lnc-bbut00015631"	exon_number "1"
14	lncRNA	exon	77871274	77871302	.	-	.	gene_id "lnc-bbug06515"	transcript_id "lnc-bbut00015631"	exon_number "2"
14	lncRNA	exon	79176676	79177475	.	-	.	gene_id "lnc-bbug06518"	transcript_id "lnc-bbut00015636"	exon_number "1"
14	lncRNA	exon	79177666	79177829	.	-	.	gene_id "lnc-bbug06518"	transcript_id "lnc-bbut00015636"	exon_number "2"
14	lncRNA	exon	79178201	79178306	.	-	.	gene_id "lnc-bbug06518"	transcript_id "lnc-bbut00015636"	exon_number "3"
14	lncRNA	exon	79195289	79196122	.	-	.	gene_id "lnc-bbug06518"	transcript_id "lnc-bbut00015636"	exon_number "4"
14	lncRNA	exon	79176676	79177475	.	-	.	gene_id "lnc-bbug06518"	transcript_id "lnc-bbut00015635"	exon_number "1"
14	lncRNA	exon	79177666	79177829	.	-	.	gene_id "lnc-bbug06518"	transcript_id "lnc-bbut00015635"	exon_number "2"
14	lncRNA	exon	79178201	79178306	.	-	.	gene_id "lnc-bbug06518"	transcript_id "lnc-bbut00015635"	exon_number "3"
14	lncRNA	exon	79195285	79196122	.	-	.	gene_id "lnc-bbug06518"	transcript_id "lnc-bbut00015635"	exon_number "4"
14	lncRNA	exon	79348428	79349931	.	-	.	gene_id "lnc-bbug06519"	transcript_id "lnc-bbut00015637"	exon_number "1"
14	lncRNA	exon	79449300	79449398	.	-	.	gene_id "lnc-bbug06519"	transcript_id "lnc-bbut00015637"	exon_number "2"
14	lncRNA	exon	79485654	79486891	.	-	.	gene_id "lnc-bbug06519"	transcript_id "lnc-bbut00015637"	exon_number "3"
14	lncRNA	exon	79348428	79349931	.	-	.	gene_id "lnc-bbug06519"	transcript_id "lnc-bbut00015638"	exon_number "1"
14	lncRNA	exon	79449300	79449398	.	-	.	gene_id "lnc-bbug06519"	transcript_id "lnc-bbut00015638"	exon_number "2"
14	lncRNA	exon	79502534	79502937	.	-	.	gene_id "lnc-bbug06519"	transcript_id "lnc-bbut00015638"	exon_number "3"
14	lncRNA	exon	79444299	79444946	.	-	.	gene_id "lnc-bbug06519"	transcript_id "lnc-bbut00015639"	exon_number "1"
14	lncRNA	exon	79449300	79449398	.	-	.	gene_id "lnc-bbug06519"	transcript_id "lnc-bbut00015639"	exon_number "2"
14	lncRNA	exon	79502534	79502937	.	-	.	gene_id "lnc-bbug06519"	transcript_id "lnc-bbut00015639"	exon_number "3"
14	lncRNA	exon	79523704	79523820	.	-	.	gene_id "lnc-bbug06520"	transcript_id "lnc-bbut00015640"	exon_number "1"
14	lncRNA	exon	79523943	79524173	.	-	.	gene_id "lnc-bbug06520"	transcript_id "lnc-bbut00015640"	exon_number "2"
14	lncRNA	exon	79538247	79538398	.	-	.	gene_id "lnc-bbug06520"	transcript_id "lnc-bbut00015640"	exon_number "3"
14	lncRNA	exon	79684688	79685088	.	-	.	gene_id "lnc-bbug06521"	transcript_id "lnc-bbut00015641"	exon_number "1"

14	lncRNA	exon	83073603	83074041	.	-	.	gene_id "lnc-bbug06529"	transcript_id "lnc-bbut00015661"	exon_number "1"
14	lncRNA	exon	83074847	83074851	.	-	.	gene_id "lnc-bbug06529"	transcript_id "lnc-bbut00015661"	exon_number "2"
14	lncRNA	exon	83080934	83081140	.	-	.	gene_id "lnc-bbug06529"	transcript_id "lnc-bbut00015661"	exon_number "3"
14	lncRNA	exon	83092894	83092929	.	-	.	gene_id "lnc-bbug06529"	transcript_id "lnc-bbut00015661"	exon_number "4"
14	lncRNA	exon	83100352	83101263	.	-	.	gene_id "lnc-bbug06529"	transcript_id "lnc-bbut00015661"	exon_number "5"
14	lncRNA	exon	83339283	83339527	.	-	.	gene_id "lnc-bbug06530"	transcript_id "lnc-bbut00015662"	exon_number "1"
14	lncRNA	exon	83356207	83356306	.	-	.	gene_id "lnc-bbug06530"	transcript_id "lnc-bbut00015662"	exon_number "2"
15	lncRNA	exon	730482	730807	.	+	.	gene_id "lnc-bbug06534"	transcript_id "lnc-bbut00015667"	exon_number "1"
15	lncRNA	exon	731377	731721	.	+	.	gene_id "lnc-bbug06534"	transcript_id "lnc-bbut00015667"	exon_number "2"
15	lncRNA	exon	1470317	1470723	.	+	.	gene_id "lnc-bbug06539"	transcript_id "lnc-bbut00015693"	exon_number "1"
15	lncRNA	exon	1519320	1519379	.	+	.	gene_id "lnc-bbug06539"	transcript_id "lnc-bbut00015693"	exon_number "2"
15	lncRNA	exon	1540024	1541371	.	+	.	gene_id "lnc-bbug06539"	transcript_id "lnc-bbut00015693"	exon_number "3"
15	lncRNA	exon	1698728	1698838	.	+	.	gene_id "lnc-bbug06540"	transcript_id "lnc-bbut00015694"	exon_number "1"
15	lncRNA	exon	1699356	1699441	.	+	.	gene_id "lnc-bbug06540"	transcript_id "lnc-bbut00015694"	exon_number "2"
15	lncRNA	exon	1714306	1714440	.	+	.	gene_id "lnc-bbug06540"	transcript_id "lnc-bbut00015694"	exon_number "3"
15	lncRNA	exon	1715123	1717443	.	+	.	gene_id "lnc-bbug06540"	transcript_id "lnc-bbut00015694"	exon_number "4"
15	lncRNA	exon	1777637	1777765	.	+	.	gene_id "lnc-bbug06541"	transcript_id "lnc-bbut00015695"	exon_number "1"
15	lncRNA	exon	1785523	1786122	.	+	.	gene_id "lnc-bbug06541"	transcript_id "lnc-bbut00015695"	exon_number "2"
15	lncRNA	exon	3942802	3947657	.	+	.	gene_id "lnc-bbug06544"	transcript_id "lnc-bbut00015698"	exon_number "1"
15	lncRNA	exon	4833531	4834006	.	+	.	gene_id "lnc-bbug06551"	transcript_id "lnc-bbut00015710"	exon_number "1"
15	lncRNA	exon	4834694	4834863	.	+	.	gene_id "lnc-bbug06551"	transcript_id "lnc-bbut00015710"	exon_number "2"
15	lncRNA	exon	4835217	4835627	.	+	.	gene_id "lnc-bbug06551"	transcript_id "lnc-bbut00015710"	exon_number "3"
15	lncRNA	exon	5286035	5286144	.	+	.	gene_id "lnc-bbug06556"	transcript_id "lnc-bbut00015715"	exon_number "1"
15	lncRNA	exon	5286221	5286273	.	+	.	gene_id "lnc-bbug06556"	transcript_id "lnc-bbut00015715"	exon_number "2"
15	lncRNA	exon	5286378	5286584	.	+	.	gene_id "lnc-bbug06556"	transcript_id "lnc-bbut00015715"	exon_number "3"
15	lncRNA	exon	5769418	5769539	.	+	.	gene_id "lnc-bbug06558"	transcript_id "lnc-bbut00015718"	exon_number "1"
15	lncRNA	exon	5769806	5770277	.	+	.	gene_id "lnc-bbug06558"	transcript_id "lnc-bbut00015718"	exon_number "2"
15	lncRNA	exon	5770426	5770936	.	+	.	gene_id "lnc-bbug06558"	transcript_id "lnc-bbut00015718"	exon_number "3"
15	lncRNA	exon	8521036	8521739	.	+	.	gene_id "lnc-bbug06567"	transcript_id "lnc-bbut00015740"	exon_number "1"
15	lncRNA	exon	8526281	8526361	.	+	.	gene_id "lnc-bbug06567"	transcript_id "lnc-bbut00015740"	exon_number "2"
15	lncRNA	exon	8528044	8528368	.	+	.	gene_id "lnc-bbug06567"	transcript_id "lnc-bbut00015740"	exon_number "3"
15	lncRNA	exon	8521036	8521739	.	+	.	gene_id "lnc-bbug06567"	transcript_id "lnc-bbut00015742"	exon_number "1"
15	lncRNA	exon	8526281	8528184	.	+	.	gene_id "lnc-bbug06567"	transcript_id "lnc-bbut00015742"	exon_number "2"
15	lncRNA	exon	8528484	8528901	.	+	.	gene_id "lnc-bbug06567"	transcript_id "lnc-bbut00015742"	exon_number "3"
15	lncRNA	exon	8521036	8521739	.	+	.	gene_id "lnc-bbug06567"	transcript_id "lnc-bbut00015741"	exon_number "1"
15	lncRNA	exon	8526281	8526361	.	+	.	gene_id "lnc-bbug06567"	transcript_id "lnc-bbut00015741"	exon_number "2"
15	lncRNA	exon	8528484	8528901	.	+	.	gene_id "lnc-bbug06567"	transcript_id "lnc-bbut00015741"	exon_number "3"
15	lncRNA	exon	8521036	8521739	.	+	.	gene_id "lnc-bbug06567"	transcript_id "lnc-bbut00015743"	exon_number "1"
15	lncRNA	exon	8526281	8528184	.	+	.	gene_id "lnc-bbug06567"	transcript_id "lnc-bbut00015743"	exon_number "2"
15	lncRNA	exon	8528484	8528658	.	+	.	gene_id "lnc-bbug06567"	transcript_id "lnc-bbut00015743"	exon_number "3"
15	lncRNA	exon	8530343	8532033	.	+	.	gene_id "lnc-bbug06567"	transcript_id "lnc-bbut00015743"	exon_number "4"
15	lncRNA	exon	8521036	8521739	.	+	.	gene_id "lnc-bbug06567"	transcript_id "lnc-bbut00015744"	exon_number "1"
15	lncRNA	exon	8526281	8528184	.	+	.	gene_id "lnc-bbug06567"	transcript_id "lnc-bbut00015744"	exon_number "2"
15	lncRNA	exon	8528484	8528658	.	+	.	gene_id "lnc-bbug06567"	transcript_id "lnc-bbut00015744"	exon_number "3"
15	lncRNA	exon	8533636	8533685	.	+	.	gene_id "lnc-bbug06567"	transcript_id "lnc-bbut00015744"	exon_number "4"
15	lncRNA	exon	8521036	8521739	.	+	.	gene_id "lnc-bbug06567"	transcript_id "lnc-bbut00015745"	exon_number "1"
15	lncRNA	exon	8526281	8528184	.	+	.	gene_id "lnc-bbug06567"	transcript_id "lnc-bbut00015745"	exon_number "2"
15	lncRNA	exon	8528484	8528658	.	+	.	gene_id "lnc-bbug06567"	transcript_id "lnc-bbut00015745"	exon_number "3"
15	lncRNA	exon	8547779	8547830	.	+	.	gene_id "lnc-bbug06567"	transcript_id "lnc-bbut00015745"	exon_number "4"
15	lncRNA	exon	9099632	9102270	.	+	.	gene_id "lnc-bbug06571"	transcript_id "lnc-bbut00015750"	exon_number "1"
15	lncRNA	exon	9338719	9338886	.	+	.	gene_id "lnc-bbug06572"	transcript_id "lnc-bbut00015751"	exon_number "1"
15	lncRNA	exon	9382048	9382720	.	+	.	gene_id "lnc-bbug06572"	transcript_id "lnc-bbut00015751"	exon_number "2"

15	lncRNA	exon	11817389	11817736	.	+	.	gene_id "lnc-bbug06576"	transcript_id "lnc-bbut00015757"	exon_number "1"
15	lncRNA	exon	11820148	11820239	.	+	.	gene_id "lnc-bbug06576"	transcript_id "lnc-bbut00015757"	exon_number "2"
15	lncRNA	exon	11820452	11822146	.	+	.	gene_id "lnc-bbug06576"	transcript_id "lnc-bbut00015757"	exon_number "3"
15	lncRNA	exon	12795151	12795275	.	+	.	gene_id "lnc-bbug06581"	transcript_id "lnc-bbut00015778"	exon_number "1"
15	lncRNA	exon	12848660	12849095	.	+	.	gene_id "lnc-bbug06581"	transcript_id "lnc-bbut00015778"	exon_number "2"
15	lncRNA	exon	12851123	12851898	.	+	.	gene_id "lnc-bbug06581"	transcript_id "lnc-bbut00015778"	exon_number "3"
15	lncRNA	exon	13122296	13122709	.	+	.	gene_id "lnc-bbug06584"	transcript_id "lnc-bbut00015786"	exon_number "1"
15	lncRNA	exon	13124220	13124855	.	+	.	gene_id "lnc-bbug06584"	transcript_id "lnc-bbut00015786"	exon_number "2"
15	lncRNA	exon	13811771	13811912	.	+	.	gene_id "lnc-bbug06585"	transcript_id "lnc-bbut00015787"	exon_number "1"
15	lncRNA	exon	13812668	13813066	.	+	.	gene_id "lnc-bbug06585"	transcript_id "lnc-bbut00015787"	exon_number "2"
15	lncRNA	exon	15360529	15360817	.	+	.	gene_id "lnc-bbug06589"	transcript_id "lnc-bbut00015794"	exon_number "1"
15	lncRNA	exon	15362410	15362461	.	+	.	gene_id "lnc-bbug06589"	transcript_id "lnc-bbut00015794"	exon_number "2"
15	lncRNA	exon	15362593	15364971	.	+	.	gene_id "lnc-bbug06589"	transcript_id "lnc-bbut00015794"	exon_number "3"
15	lncRNA	exon	16028995	16029330	.	+	.	gene_id "lnc-bbug06593"	transcript_id "lnc-bbut00015806"	exon_number "1"
15	lncRNA	exon	16037003	16037140	.	+	.	gene_id "lnc-bbug06593"	transcript_id "lnc-bbut00015806"	exon_number "2"
15	lncRNA	exon	16037503	16038177	.	+	.	gene_id "lnc-bbug06593"	transcript_id "lnc-bbut00015806"	exon_number "3"
15	lncRNA	exon	16028995	16029330	.	+	.	gene_id "lnc-bbug06593"	transcript_id "lnc-bbut00015807"	exon_number "1"
15	lncRNA	exon	16037003	16037140	.	+	.	gene_id "lnc-bbug06593"	transcript_id "lnc-bbut00015807"	exon_number "2"
15	lncRNA	exon	16038459	16040172	.	+	.	gene_id "lnc-bbug06593"	transcript_id "lnc-bbut00015807"	exon_number "3"
15	lncRNA	exon	16543695	16543771	.	+	.	gene_id "lnc-bbug06597"	transcript_id "lnc-bbut00015812"	exon_number "1"
15	lncRNA	exon	16543807	16543988	.	+	.	gene_id "lnc-bbug06597"	transcript_id "lnc-bbut00015812"	exon_number "2"
15	lncRNA	exon	16907568	16908798	.	+	.	gene_id "lnc-bbug06599"	transcript_id "lnc-bbut00015815"	exon_number "1"
15	lncRNA	exon	16919614	16919727	.	+	.	gene_id "lnc-bbug06599"	transcript_id "lnc-bbut00015815"	exon_number "2"
15	lncRNA	exon	16922986	16924944	.	+	.	gene_id "lnc-bbug06599"	transcript_id "lnc-bbut00015815"	exon_number "3"
15	lncRNA	exon	17777922	17778997	.	+	.	gene_id "lnc-bbug06602"	transcript_id "lnc-bbut00015830"	exon_number "1"
15	lncRNA	exon	17832762	17832850	.	+	.	gene_id "lnc-bbug06602"	transcript_id "lnc-bbut00015830"	exon_number "2"
15	lncRNA	exon	17832926	17834486	.	+	.	gene_id "lnc-bbug06602"	transcript_id "lnc-bbut00015830"	exon_number "3"
15	lncRNA	exon	17830817	17831155	.	+	.	gene_id "lnc-bbug06602"	transcript_id "lnc-bbut00015831"	exon_number "1"
15	lncRNA	exon	17832762	17832850	.	+	.	gene_id "lnc-bbug06602"	transcript_id "lnc-bbut00015831"	exon_number "2"
15	lncRNA	exon	17832926	17834486	.	+	.	gene_id "lnc-bbug06602"	transcript_id "lnc-bbut00015831"	exon_number "3"
15	lncRNA	exon	18003732	18003853	.	+	.	gene_id "lnc-bbug06603"	transcript_id "lnc-bbut00015832"	exon_number "1"
15	lncRNA	exon	18004920	18004988	.	+	.	gene_id "lnc-bbug06603"	transcript_id "lnc-bbut00015832"	exon_number "2"
15	lncRNA	exon	18005358	18005450	.	+	.	gene_id "lnc-bbug06603"	transcript_id "lnc-bbut00015832"	exon_number "3"
15	lncRNA	exon	18006126	18006815	.	+	.	gene_id "lnc-bbug06603"	transcript_id "lnc-bbut00015832"	exon_number "4"
15	lncRNA	exon	18003777	18003853	.	+	.	gene_id "lnc-bbug06603"	transcript_id "lnc-bbut00015833"	exon_number "1"
15	lncRNA	exon	18004791	18004988	.	+	.	gene_id "lnc-bbug06603"	transcript_id "lnc-bbut00015833"	exon_number "2"
15	lncRNA	exon	18005358	18005450	.	+	.	gene_id "lnc-bbug06603"	transcript_id "lnc-bbut00015833"	exon_number "3"
15	lncRNA	exon	18006126	18006815	.	+	.	gene_id "lnc-bbug06603"	transcript_id "lnc-bbut00015833"	exon_number "4"
15	lncRNA	exon	18348819	18349245	.	+	.	gene_id "lnc-bbug06605"	transcript_id "lnc-bbut00015836"	exon_number "1"
15	lncRNA	exon	18355749	18356096	.	+	.	gene_id "lnc-bbug06605"	transcript_id "lnc-bbut00015836"	exon_number "2"
15	lncRNA	exon	18419645	18420667	.	+	.	gene_id "lnc-bbug06606"	transcript_id "lnc-bbut00015837"	exon_number "1"
15	lncRNA	exon	18755272	18755511	.	+	.	gene_id "lnc-bbug06607"	transcript_id "lnc-bbut00015838"	exon_number "1"
15	lncRNA	exon	19017024	19017133	.	+	.	gene_id "lnc-bbug06609"	transcript_id "lnc-bbut00015841"	exon_number "1"
15	lncRNA	exon	19017582	19017716	.	+	.	gene_id "lnc-bbug06609"	transcript_id "lnc-bbut00015841"	exon_number "2"
15	lncRNA	exon	19022013	19022062	.	+	.	gene_id "lnc-bbug06609"	transcript_id "lnc-bbut00015841"	exon_number "3"
15	lncRNA	exon	19027939	19028167	.	+	.	gene_id "lnc-bbug06609"	transcript_id "lnc-bbut00015841"	exon_number "4"
15	lncRNA	exon	19017024	19017133	.	+	.	gene_id "lnc-bbug06609"	transcript_id "lnc-bbut00015840"	exon_number "1"
15	lncRNA	exon	19017531	19017716	.	+	.	gene_id "lnc-bbug06609"	transcript_id "lnc-bbut00015840"	exon_number "2"
15	lncRNA	exon	19022013	19022062	.	+	.	gene_id "lnc-bbug06609"	transcript_id "lnc-bbut00015840"	exon_number "3"
15	lncRNA	exon	19027939	19028167	.	+	.	gene_id "lnc-bbug06609"	transcript_id "lnc-bbut00015840"	exon_number "4"
15	lncRNA	exon	20520850	20520917	.	+	.	gene_id "lnc-bbug06613"	transcript_id "lnc-bbut00015849"	exon_number "1"
15	lncRNA	exon	20521002	20521194	.	+	.	gene_id "lnc-bbug06613"	transcript_id "lnc-bbut00015849"	exon_number "2"

15	lncRNA	exon	20521548	20521597	.	+	.	gene_id "lnc-bbug06613"	transcript_id "lnc-bbut00015849"	exon_number "3"
15	lncRNA	exon	20524708	20524818	.	+	.	gene_id "lnc-bbug06613"	transcript_id "lnc-bbut00015849"	exon_number "4"
15	lncRNA	exon	20525991	20526150	.	+	.	gene_id "lnc-bbug06613"	transcript_id "lnc-bbut00015849"	exon_number "5"
15	lncRNA	exon	20531017	20531740	.	+	.	gene_id "lnc-bbug06613"	transcript_id "lnc-bbut00015849"	exon_number "6"
15	lncRNA	exon	20520998	20521597	.	+	.	gene_id "lnc-bbug06613"	transcript_id "lnc-bbut00015850"	exon_number "1"
15	lncRNA	exon	20524708	20524818	.	+	.	gene_id "lnc-bbug06613"	transcript_id "lnc-bbut00015850"	exon_number "2"
15	lncRNA	exon	20525991	20526150	.	+	.	gene_id "lnc-bbug06613"	transcript_id "lnc-bbut00015850"	exon_number "3"
15	lncRNA	exon	20531017	20531740	.	+	.	gene_id "lnc-bbug06613"	transcript_id "lnc-bbut00015850"	exon_number "4"
15	lncRNA	exon	20525369	20525806	.	+	.	gene_id "lnc-bbug06613"	transcript_id "lnc-bbut00015851"	exon_number "1"
15	lncRNA	exon	20525991	20526150	.	+	.	gene_id "lnc-bbug06613"	transcript_id "lnc-bbut00015851"	exon_number "2"
15	lncRNA	exon	20531017	20531740	.	+	.	gene_id "lnc-bbug06613"	transcript_id "lnc-bbut00015851"	exon_number "3"
15	lncRNA	exon	20530467	20530547	.	+	.	gene_id "lnc-bbug06613"	transcript_id "lnc-bbut00015852"	exon_number "1"
15	lncRNA	exon	20531017	20531740	.	+	.	gene_id "lnc-bbug06613"	transcript_id "lnc-bbut00015852"	exon_number "2"
15	lncRNA	exon	20659143	20660242	.	+	.	gene_id "lnc-bbug06614"	transcript_id "lnc-bbut00015853"	exon_number "1"
15	lncRNA	exon	20663769	20663772	.	+	.	gene_id "lnc-bbug06614"	transcript_id "lnc-bbut00015853"	exon_number "2"
15	lncRNA	exon	20879967	20880229	.	+	.	gene_id "lnc-bbug06616"	transcript_id "lnc-bbut00015857"	exon_number "1"
15	lncRNA	exon	20950642	20950809	.	+	.	gene_id "lnc-bbug06616"	transcript_id "lnc-bbut00015857"	exon_number "2"
15	lncRNA	exon	20965916	20967201	.	+	.	gene_id "lnc-bbug06616"	transcript_id "lnc-bbut00015857"	exon_number "3"
15	lncRNA	exon	22545641	22545713	.	+	.	gene_id "lnc-bbug06623"	transcript_id "lnc-bbut00015916"	exon_number "1"
15	lncRNA	exon	22547072	22547208	.	+	.	gene_id "lnc-bbug06623"	transcript_id "lnc-bbut00015916"	exon_number "2"
15	lncRNA	exon	22557657	22557701	.	+	.	gene_id "lnc-bbug06623"	transcript_id "lnc-bbut00015916"	exon_number "3"
15	lncRNA	exon	24416889	24420735	.	+	.	gene_id "lnc-bbug06626"	transcript_id "lnc-bbut00015933"	exon_number "1"
15	lncRNA	exon	24455121	24455256	.	+	.	gene_id "lnc-bbug06627"	transcript_id "lnc-bbut00015934"	exon_number "1"
15	lncRNA	exon	24552830	24552902	.	+	.	gene_id "lnc-bbug06627"	transcript_id "lnc-bbut00015934"	exon_number "2"
15	lncRNA	exon	24565128	24565214	.	+	.	gene_id "lnc-bbug06627"	transcript_id "lnc-bbut00015934"	exon_number "3"
15	lncRNA	exon	24565795	24566096	.	+	.	gene_id "lnc-bbug06627"	transcript_id "lnc-bbut00015934"	exon_number "4"
15	lncRNA	exon	27125789	27125906	.	+	.	gene_id "lnc-bbug06632"	transcript_id "lnc-bbut00015939"	exon_number "1"
15	lncRNA	exon	27127326	27127402	.	+	.	gene_id "lnc-bbug06632"	transcript_id "lnc-bbut00015939"	exon_number "2"
15	lncRNA	exon	27131163	27131233	.	+	.	gene_id "lnc-bbug06632"	transcript_id "lnc-bbut00015939"	exon_number "3"
15	lncRNA	exon	27132442	27132516	.	+	.	gene_id "lnc-bbug06632"	transcript_id "lnc-bbut00015939"	exon_number "4"
15	lncRNA	exon	27135155	27136295	.	+	.	gene_id "lnc-bbug06632"	transcript_id "lnc-bbut00015939"	exon_number "5"
15	lncRNA	exon	27125821	27125978	.	+	.	gene_id "lnc-bbug06632"	transcript_id "lnc-bbut00015940"	exon_number "1"
15	lncRNA	exon	27127326	27127402	.	+	.	gene_id "lnc-bbug06632"	transcript_id "lnc-bbut00015940"	exon_number "2"
15	lncRNA	exon	27131163	27131233	.	+	.	gene_id "lnc-bbug06632"	transcript_id "lnc-bbut00015940"	exon_number "3"
15	lncRNA	exon	27132442	27132516	.	+	.	gene_id "lnc-bbug06632"	transcript_id "lnc-bbut00015940"	exon_number "4"
15	lncRNA	exon	27135155	27136295	.	+	.	gene_id "lnc-bbug06632"	transcript_id "lnc-bbut00015940"	exon_number "5"
15	lncRNA	exon	27525868	27526476	.	+	.	gene_id "lnc-bbug06635"	transcript_id "lnc-bbut00015944"	exon_number "1"
15	lncRNA	exon	28757161	28757697	.	+	.	gene_id "lnc-bbug06637"	transcript_id "lnc-bbut00015946"	exon_number "1"
15	lncRNA	exon	28760225	28760282	.	+	.	gene_id "lnc-bbug06637"	transcript_id "lnc-bbut00015946"	exon_number "2"
15	lncRNA	exon	28761404	28761699	.	+	.	gene_id "lnc-bbug06637"	transcript_id "lnc-bbut00015946"	exon_number "3"
15	lncRNA	exon	33162375	33163149	.	+	.	gene_id "lnc-bbug06639"	transcript_id "lnc-bbut00015948"	exon_number "1"
15	lncRNA	exon	33792608	33792919	.	+	.	gene_id "lnc-bbug06640"	transcript_id "lnc-bbut00015949"	exon_number "1"
15	lncRNA	exon	36676515	36676832	.	+	.	gene_id "lnc-bbug06645"	transcript_id "lnc-bbut00015958"	exon_number "1"
15	lncRNA	exon	36750716	36750789	.	+	.	gene_id "lnc-bbug06645"	transcript_id "lnc-bbut00015958"	exon_number "2"
15	lncRNA	exon	36802115	36802224	.	+	.	gene_id "lnc-bbug06645"	transcript_id "lnc-bbut00015958"	exon_number "3"
15	lncRNA	exon	36850582	36850727	.	+	.	gene_id "lnc-bbug06645"	transcript_id "lnc-bbut00015958"	exon_number "4"
15	lncRNA	exon	36979362	36979483	.	+	.	gene_id "lnc-bbug06645"	transcript_id "lnc-bbut00015958"	exon_number "5"
15	lncRNA	exon	37009323	37009414	.	+	.	gene_id "lnc-bbug06645"	transcript_id "lnc-bbut00015958"	exon_number "6"
15	lncRNA	exon	37021967	37022017	.	+	.	gene_id "lnc-bbug06645"	transcript_id "lnc-bbut00015958"	exon_number "7"
15	lncRNA	exon	36676515	36676832	.	+	.	gene_id "lnc-bbug06645"	transcript_id "lnc-bbut00015957"	exon_number "1"
15	lncRNA	exon	36802115	36802224	.	+	.	gene_id "lnc-bbug06645"	transcript_id "lnc-bbut00015957"	exon_number "2"
15	lncRNA	exon	36850582	36850727	.	+	.	gene_id "lnc-bbug06645"	transcript_id "lnc-bbut00015957"	exon_number "3"

15	lncRNA	exon	36979362	36979483	.	+	.	gene_id "lnc-bbug06645"	transcript_id "lnc-bbut00015957"	exon_number "4"
15	lncRNA	exon	37009323	37009414	.	+	.	gene_id "lnc-bbug06645"	transcript_id "lnc-bbut00015957"	exon_number "5"
15	lncRNA	exon	37021967	37022017	.	+	.	gene_id "lnc-bbug06645"	transcript_id "lnc-bbut00015957"	exon_number "6"
15	lncRNA	exon	36676515	36676832	.	+	.	gene_id "lnc-bbug06645"	transcript_id "lnc-bbut00015956"	exon_number "1"
15	lncRNA	exon	36750716	36750789	.	+	.	gene_id "lnc-bbug06645"	transcript_id "lnc-bbut00015956"	exon_number "2"
15	lncRNA	exon	36850582	36850727	.	+	.	gene_id "lnc-bbug06645"	transcript_id "lnc-bbut00015956"	exon_number "3"
15	lncRNA	exon	36979362	36979483	.	+	.	gene_id "lnc-bbug06645"	transcript_id "lnc-bbut00015956"	exon_number "4"
15	lncRNA	exon	37009323	37009414	.	+	.	gene_id "lnc-bbug06645"	transcript_id "lnc-bbut00015956"	exon_number "5"
15	lncRNA	exon	37021967	37022017	.	+	.	gene_id "lnc-bbug06645"	transcript_id "lnc-bbut00015956"	exon_number "6"
15	lncRNA	exon	36704345	36704887	.	+	.	gene_id "lnc-bbug06645"	transcript_id "lnc-bbut00015960"	exon_number "1"
15	lncRNA	exon	36750716	36750789	.	+	.	gene_id "lnc-bbug06645"	transcript_id "lnc-bbut00015960"	exon_number "2"
15	lncRNA	exon	36802115	36802224	.	+	.	gene_id "lnc-bbug06645"	transcript_id "lnc-bbut00015960"	exon_number "3"
15	lncRNA	exon	36850582	36850727	.	+	.	gene_id "lnc-bbug06645"	transcript_id "lnc-bbut00015960"	exon_number "4"
15	lncRNA	exon	36979362	36979483	.	+	.	gene_id "lnc-bbug06645"	transcript_id "lnc-bbut00015960"	exon_number "5"
15	lncRNA	exon	37009323	37009414	.	+	.	gene_id "lnc-bbug06645"	transcript_id "lnc-bbut00015960"	exon_number "6"
15	lncRNA	exon	37021967	37022017	.	+	.	gene_id "lnc-bbug06645"	transcript_id "lnc-bbut00015960"	exon_number "7"
15	lncRNA	exon	36704345	36704887	.	+	.	gene_id "lnc-bbug06645"	transcript_id "lnc-bbut00015959"	exon_number "1"
15	lncRNA	exon	36802115	36802224	.	+	.	gene_id "lnc-bbug06645"	transcript_id "lnc-bbut00015959"	exon_number "2"
15	lncRNA	exon	36850582	36850727	.	+	.	gene_id "lnc-bbug06645"	transcript_id "lnc-bbut00015959"	exon_number "3"
15	lncRNA	exon	36979362	36979483	.	+	.	gene_id "lnc-bbug06645"	transcript_id "lnc-bbut00015959"	exon_number "4"
15	lncRNA	exon	37009323	37009414	.	+	.	gene_id "lnc-bbug06645"	transcript_id "lnc-bbut00015959"	exon_number "5"
15	lncRNA	exon	37021967	37022017	.	+	.	gene_id "lnc-bbug06645"	transcript_id "lnc-bbut00015959"	exon_number "6"
15	lncRNA	exon	36776544	36777261	.	+	.	gene_id "lnc-bbug06645"	transcript_id "lnc-bbut00015961"	exon_number "1"
15	lncRNA	exon	36802115	36802224	.	+	.	gene_id "lnc-bbug06645"	transcript_id "lnc-bbut00015961"	exon_number "2"
15	lncRNA	exon	36850582	36850727	.	+	.	gene_id "lnc-bbug06645"	transcript_id "lnc-bbut00015961"	exon_number "3"
15	lncRNA	exon	36979362	36979483	.	+	.	gene_id "lnc-bbug06645"	transcript_id "lnc-bbut00015961"	exon_number "4"
15	lncRNA	exon	37009323	37009414	.	+	.	gene_id "lnc-bbug06645"	transcript_id "lnc-bbut00015961"	exon_number "5"
15	lncRNA	exon	37021967	37022017	.	+	.	gene_id "lnc-bbug06645"	transcript_id "lnc-bbut00015961"	exon_number "6"
15	lncRNA	exon	36846878	36847028	.	+	.	gene_id "lnc-bbug06645"	transcript_id "lnc-bbut00015962"	exon_number "1"
15	lncRNA	exon	36850582	36850727	.	+	.	gene_id "lnc-bbug06645"	transcript_id "lnc-bbut00015962"	exon_number "2"
15	lncRNA	exon	36979362	36979483	.	+	.	gene_id "lnc-bbug06645"	transcript_id "lnc-bbut00015962"	exon_number "3"
15	lncRNA	exon	37009323	37009414	.	+	.	gene_id "lnc-bbug06645"	transcript_id "lnc-bbut00015962"	exon_number "4"
15	lncRNA	exon	37021967	37022017	.	+	.	gene_id "lnc-bbug06645"	transcript_id "lnc-bbut00015962"	exon_number "5"
15	lncRNA	exon	37022159	37022330	.	+	.	gene_id "lnc-bbug06646"	transcript_id "lnc-bbut00015963"	exon_number "1"
15	lncRNA	exon	37127663	37127758	.	+	.	gene_id "lnc-bbug06646"	transcript_id "lnc-bbut00015963"	exon_number "2"
15	lncRNA	exon	37139761	37139922	.	+	.	gene_id "lnc-bbug06646"	transcript_id "lnc-bbut00015963"	exon_number "3"
15	lncRNA	exon	37305262	37305409	.	+	.	gene_id "lnc-bbug06648"	transcript_id "lnc-bbut00015966"	exon_number "1"
15	lncRNA	exon	37321284	37321455	.	+	.	gene_id "lnc-bbug06648"	transcript_id "lnc-bbut00015966"	exon_number "2"
15	lncRNA	exon	37333472	37333627	.	+	.	gene_id "lnc-bbug06648"	transcript_id "lnc-bbut00015966"	exon_number "3"
15	lncRNA	exon	37336249	37338531	.	+	.	gene_id "lnc-bbug06648"	transcript_id "lnc-bbut00015966"	exon_number "4"
15	lncRNA	exon	37333634	37334530	.	+	.	gene_id "lnc-bbug06649"	transcript_id "lnc-bbut00015967"	exon_number "1"
15	lncRNA	exon	37335807	37336127	.	+	.	gene_id "lnc-bbug06649"	transcript_id "lnc-bbut00015967"	exon_number "2"
15	lncRNA	exon	37683775	37683926	.	+	.	gene_id "lnc-bbug06654"	transcript_id "lnc-bbut00015972"	exon_number "1"
15	lncRNA	exon	37686091	37686263	.	+	.	gene_id "lnc-bbug06654"	transcript_id "lnc-bbut00015972"	exon_number "2"
15	lncRNA	exon	37686474	37686575	.	+	.	gene_id "lnc-bbug06654"	transcript_id "lnc-bbut00015972"	exon_number "3"
15	lncRNA	exon	37687836	37688911	.	+	.	gene_id "lnc-bbug06654"	transcript_id "lnc-bbut00015972"	exon_number "4"
15	lncRNA	exon	37784651	37785120	.	+	.	gene_id "lnc-bbug06655"	transcript_id "lnc-bbut00015973"	exon_number "1"
15	lncRNA	exon	37909280	37909336	.	+	.	gene_id "lnc-bbug06656"	transcript_id "lnc-bbut00015974"	exon_number "1"
15	lncRNA	exon	37909448	37909615	.	+	.	gene_id "lnc-bbug06656"	transcript_id "lnc-bbut00015974"	exon_number "2"
15	lncRNA	exon	37930311	37930393	.	+	.	gene_id "lnc-bbug06656"	transcript_id "lnc-bbut00015974"	exon_number "3"
15	lncRNA	exon	37933205	37933766	.	+	.	gene_id "lnc-bbug06656"	transcript_id "lnc-bbut00015974"	exon_number "4"
15	lncRNA	exon	37944154	37945677	.	+	.	gene_id "lnc-bbug06657"	transcript_id "lnc-bbut00015975"	exon_number "1"

15	lncRNA	exon	37947463	37947716	.	+	.	gene_id "lnc-bbug06657"	transcript_id "lnc-bbut00015975"	exon_number "2"
15	lncRNA	exon	37944168	37945646	.	+	.	gene_id "lnc-bbug06657"	transcript_id "lnc-bbut00015976"	exon_number "1"
15	lncRNA	exon	37945755	37946061	.	+	.	gene_id "lnc-bbug06657"	transcript_id "lnc-bbut00015976"	exon_number "2"
15	lncRNA	exon	37947463	37947716	.	+	.	gene_id "lnc-bbug06657"	transcript_id "lnc-bbut00015976"	exon_number "3"
15	lncRNA	exon	38761231	38761549	.	+	.	gene_id "lnc-bbug06661"	transcript_id "lnc-bbut00015983"	exon_number "1"
15	lncRNA	exon	38969850	38971897	.	+	.	gene_id "lnc-bbug06663"	transcript_id "lnc-bbut00015985"	exon_number "1"
15	lncRNA	exon	38980518	38980642	.	+	.	gene_id "lnc-bbug06663"	transcript_id "lnc-bbut00015985"	exon_number "2"
15	lncRNA	exon	39385187	39385257	.	+	.	gene_id "lnc-bbug06666"	transcript_id "lnc-bbut00015991"	exon_number "1"
15	lncRNA	exon	39385352	39385439	.	+	.	gene_id "lnc-bbug06666"	transcript_id "lnc-bbut00015991"	exon_number "2"
15	lncRNA	exon	39387805	39387932	.	+	.	gene_id "lnc-bbug06666"	transcript_id "lnc-bbut00015991"	exon_number "3"
15	lncRNA	exon	42161000	42161111	.	+	.	gene_id "lnc-bbug06673"	transcript_id "lnc-bbut00016000"	exon_number "1"
15	lncRNA	exon	42163724	42163889	.	+	.	gene_id "lnc-bbug06673"	transcript_id "lnc-bbut00016000"	exon_number "2"
15	lncRNA	exon	42164812	42166038	.	+	.	gene_id "lnc-bbug06673"	transcript_id "lnc-bbut00016000"	exon_number "3"
15	lncRNA	exon	42454420	42457790	.	+	.	gene_id "lnc-bbug06674"	transcript_id "lnc-bbut00016003"	exon_number "1"
15	lncRNA	exon	42462566	42462763	.	+	.	gene_id "lnc-bbug06674"	transcript_id "lnc-bbut00016003"	exon_number "2"
15	lncRNA	exon	42473564	42473738	.	+	.	gene_id "lnc-bbug06674"	transcript_id "lnc-bbut00016003"	exon_number "3"
15	lncRNA	exon	42475500	42475574	.	+	.	gene_id "lnc-bbug06674"	transcript_id "lnc-bbut00016003"	exon_number "4"
15	lncRNA	exon	42485212	42485368	.	+	.	gene_id "lnc-bbug06674"	transcript_id "lnc-bbut00016003"	exon_number "5"
15	lncRNA	exon	42492096	42499225	.	+	.	gene_id "lnc-bbug06674"	transcript_id "lnc-bbut00016003"	exon_number "6"
15	lncRNA	exon	42454420	42457790	.	+	.	gene_id "lnc-bbug06674"	transcript_id "lnc-bbut00016005"	exon_number "1"
15	lncRNA	exon	42462566	42473738	.	+	.	gene_id "lnc-bbug06674"	transcript_id "lnc-bbut00016005"	exon_number "2"
15	lncRNA	exon	42475500	42475574	.	+	.	gene_id "lnc-bbug06674"	transcript_id "lnc-bbut00016005"	exon_number "3"
15	lncRNA	exon	42485212	42485368	.	+	.	gene_id "lnc-bbug06674"	transcript_id "lnc-bbut00016005"	exon_number "4"
15	lncRNA	exon	42492096	42492298	.	+	.	gene_id "lnc-bbug06674"	transcript_id "lnc-bbut00016005"	exon_number "5"
15	lncRNA	exon	42492391	42492471	.	+	.	gene_id "lnc-bbug06674"	transcript_id "lnc-bbut00016005"	exon_number "6"
15	lncRNA	exon	42492588	42499225	.	+	.	gene_id "lnc-bbug06674"	transcript_id "lnc-bbut00016005"	exon_number "7"
15	lncRNA	exon	42454420	42457790	.	+	.	gene_id "lnc-bbug06674"	transcript_id "lnc-bbut00016004"	exon_number "1"
15	lncRNA	exon	42462566	42468850	.	+	.	gene_id "lnc-bbug06674"	transcript_id "lnc-bbut00016004"	exon_number "2"
15	lncRNA	exon	42470720	42473738	.	+	.	gene_id "lnc-bbug06674"	transcript_id "lnc-bbut00016004"	exon_number "3"
15	lncRNA	exon	42475500	42475574	.	+	.	gene_id "lnc-bbug06674"	transcript_id "lnc-bbut00016004"	exon_number "4"
15	lncRNA	exon	42485212	42485368	.	+	.	gene_id "lnc-bbug06674"	transcript_id "lnc-bbut00016004"	exon_number "5"
15	lncRNA	exon	42492096	42499225	.	+	.	gene_id "lnc-bbug06674"	transcript_id "lnc-bbut00016004"	exon_number "6"
15	lncRNA	exon	42454420	42457790	.	+	.	gene_id "lnc-bbug06674"	transcript_id "lnc-bbut00016002"	exon_number "1"
15	lncRNA	exon	42462566	42462763	.	+	.	gene_id "lnc-bbug06674"	transcript_id "lnc-bbut00016002"	exon_number "2"
15	lncRNA	exon	42468717	42473738	.	+	.	gene_id "lnc-bbug06674"	transcript_id "lnc-bbut00016002"	exon_number "3"
15	lncRNA	exon	42475500	42475574	.	+	.	gene_id "lnc-bbug06674"	transcript_id "lnc-bbut00016002"	exon_number "4"
15	lncRNA	exon	42485212	42485368	.	+	.	gene_id "lnc-bbug06674"	transcript_id "lnc-bbut00016002"	exon_number "5"
15	lncRNA	exon	42492096	42499225	.	+	.	gene_id "lnc-bbug06674"	transcript_id "lnc-bbut00016002"	exon_number "6"
15	lncRNA	exon	42454420	42457790	.	+	.	gene_id "lnc-bbug06674"	transcript_id "lnc-bbut00016001"	exon_number "1"
15	lncRNA	exon	42462566	42473738	.	+	.	gene_id "lnc-bbug06674"	transcript_id "lnc-bbut00016001"	exon_number "2"
15	lncRNA	exon	42485212	42485368	.	+	.	gene_id "lnc-bbug06674"	transcript_id "lnc-bbut00016001"	exon_number "3"
15	lncRNA	exon	42492096	42499225	.	+	.	gene_id "lnc-bbug06674"	transcript_id "lnc-bbut00016001"	exon_number "4"
15	lncRNA	exon	42454420	42457790	.	+	.	gene_id "lnc-bbug06674"	transcript_id "lnc-bbut00016006"	exon_number "1"
15	lncRNA	exon	42462566	42473738	.	+	.	gene_id "lnc-bbug06674"	transcript_id "lnc-bbut00016006"	exon_number "2"
15	lncRNA	exon	42475500	42475574	.	+	.	gene_id "lnc-bbug06674"	transcript_id "lnc-bbut00016006"	exon_number "3"
15	lncRNA	exon	42485212	42485368	.	+	.	gene_id "lnc-bbug06674"	transcript_id "lnc-bbut00016006"	exon_number "4"
15	lncRNA	exon	42492096	42492471	.	+	.	gene_id "lnc-bbug06674"	transcript_id "lnc-bbut00016006"	exon_number "5"
15	lncRNA	exon	42499260	42499910	.	+	.	gene_id "lnc-bbug06674"	transcript_id "lnc-bbut00016006"	exon_number "6"
15	lncRNA	exon	43891799	43891831	.	+	.	gene_id "lnc-bbug06675"	transcript_id "lnc-bbut00016007"	exon_number "1"
15	lncRNA	exon	43892772	43892888	.	+	.	gene_id "lnc-bbug06675"	transcript_id "lnc-bbut00016007"	exon_number "2"
15	lncRNA	exon	43896337	43896362	.	+	.	gene_id "lnc-bbug06675"	transcript_id "lnc-bbut00016007"	exon_number "3"
15	lncRNA	exon	43897269	43897963	.	+	.	gene_id "lnc-bbug06675"	transcript_id "lnc-bbut00016007"	exon_number "4"

15	lncRNA	exon	44916817	44917369	.	+	.	gene_id "lnc-bbug06677"	transcript_id "lnc-bbut00016014"	exon_number "1"
15	lncRNA	exon	45124549	45125270	.	+	.	gene_id "lnc-bbug06679"	transcript_id "lnc-bbut00016017"	exon_number "1"
15	lncRNA	exon	45127294	45127576	.	+	.	gene_id "lnc-bbug06679"	transcript_id "lnc-bbut00016017"	exon_number "2"
15	lncRNA	exon	46544475	46544606	.	+	.	gene_id "lnc-bbug06686"	transcript_id "lnc-bbut00016034"	exon_number "1"
15	lncRNA	exon	46545356	46545506	.	+	.	gene_id "lnc-bbug06686"	transcript_id "lnc-bbut00016034"	exon_number "2"
15	lncRNA	exon	48021617	48026070	.	+	.	gene_id "lnc-bbug06691"	transcript_id "lnc-bbut00016049"	exon_number "1"
15	lncRNA	exon	48027776	48027837	.	+	.	gene_id "lnc-bbug06691"	transcript_id "lnc-bbut00016049"	exon_number "2"
15	lncRNA	exon	48030463	48031386	.	+	.	gene_id "lnc-bbug06691"	transcript_id "lnc-bbut00016049"	exon_number "3"
15	lncRNA	exon	48089988	48090067	.	+	.	gene_id "lnc-bbug06693"	transcript_id "lnc-bbut00016053"	exon_number "1"
15	lncRNA	exon	48093046	48093148	.	+	.	gene_id "lnc-bbug06693"	transcript_id "lnc-bbut00016053"	exon_number "2"
15	lncRNA	exon	48100729	48101925	.	+	.	gene_id "lnc-bbug06693"	transcript_id "lnc-bbut00016053"	exon_number "3"
15	lncRNA	exon	51230632	51231025	.	+	.	gene_id "lnc-bbug06703"	transcript_id "lnc-bbut00016084"	exon_number "1"
15	lncRNA	exon	51234108	51234221	.	+	.	gene_id "lnc-bbug06703"	transcript_id "lnc-bbut00016084"	exon_number "2"
15	lncRNA	exon	51234470	51234538	.	+	.	gene_id "lnc-bbug06703"	transcript_id "lnc-bbut00016084"	exon_number "3"
15	lncRNA	exon	51234716	51234894	.	+	.	gene_id "lnc-bbug06703"	transcript_id "lnc-bbut00016084"	exon_number "4"
15	lncRNA	exon	51383914	51384262	.	+	.	gene_id "lnc-bbug06704"	transcript_id "lnc-bbut00016085"	exon_number "1"
15	lncRNA	exon	51580390	51581393	.	+	.	gene_id "lnc-bbug06708"	transcript_id "lnc-bbut00016094"	exon_number "1"
15	lncRNA	exon	51586895	51586969	.	+	.	gene_id "lnc-bbug06708"	transcript_id "lnc-bbut00016094"	exon_number "2"
15	lncRNA	exon	51631807	51632610	.	+	.	gene_id "lnc-bbug06708"	transcript_id "lnc-bbut00016094"	exon_number "3"
15	lncRNA	exon	51724966	51726894	.	+	.	gene_id "lnc-bbug06709"	transcript_id "lnc-bbut00016095"	exon_number "1"
15	lncRNA	exon	51731046	51731196	.	+	.	gene_id "lnc-bbug06709"	transcript_id "lnc-bbut00016095"	exon_number "2"
15	lncRNA	exon	51731822	51731896	.	+	.	gene_id "lnc-bbug06709"	transcript_id "lnc-bbut00016095"	exon_number "3"
15	lncRNA	exon	51732767	51733936	.	+	.	gene_id "lnc-bbug06709"	transcript_id "lnc-bbut00016095"	exon_number "4"
15	lncRNA	exon	51724969	51726894	.	+	.	gene_id "lnc-bbug06709"	transcript_id "lnc-bbut00016096"	exon_number "1"
15	lncRNA	exon	51731046	51731196	.	+	.	gene_id "lnc-bbug06709"	transcript_id "lnc-bbut00016096"	exon_number "2"
15	lncRNA	exon	51731822	51733936	.	+	.	gene_id "lnc-bbug06709"	transcript_id "lnc-bbut00016096"	exon_number "3"
15	lncRNA	exon	52042783	52043163	.	+	.	gene_id "lnc-bbug06711"	transcript_id "lnc-bbut00016098"	exon_number "1"
15	lncRNA	exon	55504859	55505501	.	+	.	gene_id "lnc-bbug06720"	transcript_id "lnc-bbut00016119"	exon_number "1"
15	lncRNA	exon	55516736	55516787	.	+	.	gene_id "lnc-bbug06720"	transcript_id "lnc-bbut00016119"	exon_number "2"
15	lncRNA	exon	55517394	55517514	.	+	.	gene_id "lnc-bbug06720"	transcript_id "lnc-bbut00016119"	exon_number "3"
15	lncRNA	exon	55801239	55801587	.	+	.	gene_id "lnc-bbug06721"	transcript_id "lnc-bbut00016120"	exon_number "1"
15	lncRNA	exon	55802266	55802421	.	+	.	gene_id "lnc-bbug06721"	transcript_id "lnc-bbut00016120"	exon_number "2"
15	lncRNA	exon	56140790	56140896	.	+	.	gene_id "lnc-bbug06723"	transcript_id "lnc-bbut00016122"	exon_number "1"
15	lncRNA	exon	56142948	56142996	.	+	.	gene_id "lnc-bbug06723"	transcript_id "lnc-bbut00016122"	exon_number "2"
15	lncRNA	exon	56145983	56146197	.	+	.	gene_id "lnc-bbug06723"	transcript_id "lnc-bbut00016122"	exon_number "3"
15	lncRNA	exon	56148104	56148320	.	+	.	gene_id "lnc-bbug06723"	transcript_id "lnc-bbut00016122"	exon_number "4"
15	lncRNA	exon	58628703	58629433	.	+	.	gene_id "lnc-bbug06729"	transcript_id "lnc-bbut00016139"	exon_number "1"
15	lncRNA	exon	60295079	60295148	.	+	.	gene_id "lnc-bbug06737"	transcript_id "lnc-bbut00016154"	exon_number "1"
15	lncRNA	exon	60295563	60295711	.	+	.	gene_id "lnc-bbug06737"	transcript_id "lnc-bbut00016154"	exon_number "2"
15	lncRNA	exon	60296534	60296568	.	+	.	gene_id "lnc-bbug06737"	transcript_id "lnc-bbut00016154"	exon_number "3"
15	lncRNA	exon	60296892	60296989	.	+	.	gene_id "lnc-bbug06737"	transcript_id "lnc-bbut00016154"	exon_number "4"
15	lncRNA	exon	60309840	60311067	.	+	.	gene_id "lnc-bbug06737"	transcript_id "lnc-bbut00016154"	exon_number "5"
15	lncRNA	exon	60865647	60867211	.	+	.	gene_id "lnc-bbug06743"	transcript_id "lnc-bbut00016175"	exon_number "1"
15	lncRNA	exon	60867320	60868097	.	+	.	gene_id "lnc-bbug06743"	transcript_id "lnc-bbut00016175"	exon_number "2"
15	lncRNA	exon	61251443	61251867	.	+	.	gene_id "lnc-bbug06747"	transcript_id "lnc-bbut00016181"	exon_number "1"
15	lncRNA	exon	61259824	61260014	.	+	.	gene_id "lnc-bbug06747"	transcript_id "lnc-bbut00016181"	exon_number "2"
15	lncRNA	exon	61260452	61262768	.	+	.	gene_id "lnc-bbug06747"	transcript_id "lnc-bbut00016181"	exon_number "3"
15	lncRNA	exon	62063729	62069093	.	+	.	gene_id "lnc-bbug06750"	transcript_id "lnc-bbut00016191"	exon_number "1"
15	lncRNA	exon	62081145	62081615	.	+	.	gene_id "lnc-bbug06751"	transcript_id "lnc-bbut00016192"	exon_number "1"
15	lncRNA	exon	62085212	62085261	.	+	.	gene_id "lnc-bbug06751"	transcript_id "lnc-bbut00016192"	exon_number "2"
15	lncRNA	exon	62086278	62087058	.	+	.	gene_id "lnc-bbug06751"	transcript_id "lnc-bbut00016192"	exon_number "3"
15	lncRNA	exon	62976878	62979365	.	+	.	gene_id "lnc-bbug06756"	transcript_id "lnc-bbut00016204"	exon_number "1"

15	lncRNA	exon	62979498	62979861	.	+	.	gene_id "lnc-bbug06756"	transcript_id "lnc-bbut00016204"	exon_number "2"
15	lncRNA	exon	63212776	63214274	.	+	.	gene_id "lnc-bbug06758"	transcript_id "lnc-bbut00016206"	exon_number "1"
15	lncRNA	exon	63215057	63215642	.	+	.	gene_id "lnc-bbug06758"	transcript_id "lnc-bbut00016206"	exon_number "2"
15	lncRNA	exon	63321374	63321605	.	+	.	gene_id "lnc-bbug06759"	transcript_id "lnc-bbut00016207"	exon_number "1"
15	lncRNA	exon	63328091	63331386	.	+	.	gene_id "lnc-bbug06759"	transcript_id "lnc-bbut00016207"	exon_number "2"
15	lncRNA	exon	63375985	63376362	.	+	.	gene_id "lnc-bbug06760"	transcript_id "lnc-bbut00016208"	exon_number "1"
15	lncRNA	exon	63376646	63376695	.	+	.	gene_id "lnc-bbug06760"	transcript_id "lnc-bbut00016208"	exon_number "2"
15	lncRNA	exon	63376787	63377040	.	+	.	gene_id "lnc-bbug06760"	transcript_id "lnc-bbut00016208"	exon_number "3"
15	lncRNA	exon	65528473	65528524	.	+	.	gene_id "lnc-bbug06764"	transcript_id "lnc-bbut00016212"	exon_number "1"
15	lncRNA	exon	65528659	65530011	.	+	.	gene_id "lnc-bbug06764"	transcript_id "lnc-bbut00016212"	exon_number "2"
15	lncRNA	exon	66685239	66685841	.	+	.	gene_id "lnc-bbug06771"	transcript_id "lnc-bbut00016233"	exon_number "1"
15	lncRNA	exon	66686810	66686920	.	+	.	gene_id "lnc-bbug06771"	transcript_id "lnc-bbut00016233"	exon_number "2"
15	lncRNA	exon	66703926	66704821	.	+	.	gene_id "lnc-bbug06771"	transcript_id "lnc-bbut00016233"	exon_number "3"
15	lncRNA	exon	67753945	67754807	.	+	.	gene_id "lnc-bbug06780"	transcript_id "lnc-bbut00016257"	exon_number "1"
15	lncRNA	exon	67756182	67756542	.	+	.	gene_id "lnc-bbug06780"	transcript_id "lnc-bbut00016257"	exon_number "2"
15	lncRNA	exon	67757472	67759041	.	+	.	gene_id "lnc-bbug06780"	transcript_id "lnc-bbut00016257"	exon_number "3"
15	lncRNA	exon	67842045	67844125	.	+	.	gene_id "lnc-bbug06781"	transcript_id "lnc-bbut00016260"	exon_number "1"
15	lncRNA	exon	67845360	67845520	.	+	.	gene_id "lnc-bbug06781"	transcript_id "lnc-bbut00016260"	exon_number "2"
15	lncRNA	exon	67847025	67847302	.	+	.	gene_id "lnc-bbug06781"	transcript_id "lnc-bbut00016260"	exon_number "3"
15	lncRNA	exon	67847652	67848105	.	+	.	gene_id "lnc-bbug06781"	transcript_id "lnc-bbut00016260"	exon_number "4"
15	lncRNA	exon	67842045	67844125	.	+	.	gene_id "lnc-bbug06781"	transcript_id "lnc-bbut00016259"	exon_number "1"
15	lncRNA	exon	67847025	67847302	.	+	.	gene_id "lnc-bbug06781"	transcript_id "lnc-bbut00016259"	exon_number "2"
15	lncRNA	exon	67847652	67848105	.	+	.	gene_id "lnc-bbug06781"	transcript_id "lnc-bbut00016259"	exon_number "3"
15	lncRNA	exon	67842045	67844125	.	+	.	gene_id "lnc-bbug06781"	transcript_id "lnc-bbut00016258"	exon_number "1"
15	lncRNA	exon	67847025	67848105	.	+	.	gene_id "lnc-bbug06781"	transcript_id "lnc-bbut00016258"	exon_number "2"
15	lncRNA	exon	67846330	67847302	.	+	.	gene_id "lnc-bbug06781"	transcript_id "lnc-bbut00016261"	exon_number "1"
15	lncRNA	exon	67847652	67848105	.	+	.	gene_id "lnc-bbug06781"	transcript_id "lnc-bbut00016261"	exon_number "2"
15	lncRNA	exon	67848539	67850193	.	+	.	gene_id "lnc-bbug06782"	transcript_id "lnc-bbut00016262"	exon_number "1"
15	lncRNA	exon	67851238	67855025	.	+	.	gene_id "lnc-bbug06782"	transcript_id "lnc-bbut00016262"	exon_number "2"
15	lncRNA	exon	67849508	67850116	.	+	.	gene_id "lnc-bbug06782"	transcript_id "lnc-bbut00016263"	exon_number "1"
15	lncRNA	exon	67851238	67855025	.	+	.	gene_id "lnc-bbug06782"	transcript_id "lnc-bbut00016263"	exon_number "2"
15	lncRNA	exon	68838019	68838328	.	+	.	gene_id "lnc-bbug06785"	transcript_id "lnc-bbut00016266"	exon_number "1"
15	lncRNA	exon	68839164	68839351	.	+	.	gene_id "lnc-bbug06785"	transcript_id "lnc-bbut00016266"	exon_number "2"
15	lncRNA	exon	68843870	68844273	.	+	.	gene_id "lnc-bbug06785"	transcript_id "lnc-bbut00016266"	exon_number "3"
15	lncRNA	exon	69694205	69694421	.	+	.	gene_id "lnc-bbug06787"	transcript_id "lnc-bbut00016268"	exon_number "1"
15	lncRNA	exon	69761148	69761609	.	+	.	gene_id "lnc-bbug06787"	transcript_id "lnc-bbut00016268"	exon_number "2"
15	lncRNA	exon	69835528	69835624	.	+	.	gene_id "lnc-bbug06787"	transcript_id "lnc-bbut00016268"	exon_number "3"
15	lncRNA	exon	69694205	69694421	.	+	.	gene_id "lnc-bbug06787"	transcript_id "lnc-bbut00016269"	exon_number "1"
15	lncRNA	exon	69761148	69761609	.	+	.	gene_id "lnc-bbug06787"	transcript_id "lnc-bbut00016269"	exon_number "2"
15	lncRNA	exon	69837373	69837505	.	+	.	gene_id "lnc-bbug06787"	transcript_id "lnc-bbut00016269"	exon_number "3"
15	lncRNA	exon	69856407	69856691	.	+	.	gene_id "lnc-bbug06787"	transcript_id "lnc-bbut00016269"	exon_number "4"
15	lncRNA	exon	69694205	69694421	.	+	.	gene_id "lnc-bbug06787"	transcript_id "lnc-bbut00016270"	exon_number "1"
15	lncRNA	exon	69761148	69761609	.	+	.	gene_id "lnc-bbug06787"	transcript_id "lnc-bbut00016270"	exon_number "2"
15	lncRNA	exon	69837373	69837505	.	+	.	gene_id "lnc-bbug06787"	transcript_id "lnc-bbut00016270"	exon_number "3"
15	lncRNA	exon	69868388	69873425	.	+	.	gene_id "lnc-bbug06787"	transcript_id "lnc-bbut00016270"	exon_number "4"
15	lncRNA	exon	69695454	69695684	.	+	.	gene_id "lnc-bbug06787"	transcript_id "lnc-bbut00016271"	exon_number "1"
15	lncRNA	exon	69761148	69761609	.	+	.	gene_id "lnc-bbug06787"	transcript_id "lnc-bbut00016271"	exon_number "2"
15	lncRNA	exon	69837373	69837505	.	+	.	gene_id "lnc-bbug06787"	transcript_id "lnc-bbut00016271"	exon_number "3"
15	lncRNA	exon	69868388	69873425	.	+	.	gene_id "lnc-bbug06787"	transcript_id "lnc-bbut00016271"	exon_number "4"
15	lncRNA	exon	69695468	69695684	.	+	.	gene_id "lnc-bbug06787"	transcript_id "lnc-bbut00016272"	exon_number "1"
15	lncRNA	exon	69761148	69761609	.	+	.	gene_id "lnc-bbug06787"	transcript_id "lnc-bbut00016272"	exon_number "2"
15	lncRNA	exon	69837373	69837505	.	+	.	gene_id "lnc-bbug06787"	transcript_id "lnc-bbut00016272"	exon_number "3"

15	lncRNA	exon	69842292	69842336	.	+	.	gene_id "lnc-bbug06787"	transcript_id "lnc-bbut00016272"	exon_number "4"
15	lncRNA	exon	69695498	69695684	.	+	.	gene_id "lnc-bbug06787"	transcript_id "lnc-bbut00016273"	exon_number "1"
15	lncRNA	exon	69753302	69753466	.	+	.	gene_id "lnc-bbug06787"	transcript_id "lnc-bbut00016273"	exon_number "2"
15	lncRNA	exon	69873492	69875827	.	+	.	gene_id "lnc-bbug06788"	transcript_id "lnc-bbut00016274"	exon_number "1"
15	lncRNA	exon	69878076	69879299	.	+	.	gene_id "lnc-bbug06788"	transcript_id "lnc-bbut00016274"	exon_number "2"
15	lncRNA	exon	69890901	69891699	.	+	.	gene_id "lnc-bbug06789"	transcript_id "lnc-bbut00016277"	exon_number "1"
15	lncRNA	exon	69892296	69892445	.	+	.	gene_id "lnc-bbug06789"	transcript_id "lnc-bbut00016277"	exon_number "2"
15	lncRNA	exon	69914278	69914459	.	+	.	gene_id "lnc-bbug06789"	transcript_id "lnc-bbut00016277"	exon_number "3"
15	lncRNA	exon	69916630	69917230	.	+	.	gene_id "lnc-bbug06789"	transcript_id "lnc-bbut00016277"	exon_number "4"
15	lncRNA	exon	69891763	69892215	.	+	.	gene_id "lnc-bbug06789"	transcript_id "lnc-bbut00016278"	exon_number "1"
15	lncRNA	exon	69892296	69892445	.	+	.	gene_id "lnc-bbug06789"	transcript_id "lnc-bbut00016278"	exon_number "2"
15	lncRNA	exon	69914278	69914459	.	+	.	gene_id "lnc-bbug06789"	transcript_id "lnc-bbut00016278"	exon_number "3"
15	lncRNA	exon	69916630	69917230	.	+	.	gene_id "lnc-bbug06789"	transcript_id "lnc-bbut00016278"	exon_number "4"
15	lncRNA	exon	69955853	69961519	.	+	.	gene_id "lnc-bbug06790"	transcript_id "lnc-bbut00016279"	exon_number "1"
15	lncRNA	exon	69961615	69961970	.	+	.	gene_id "lnc-bbug06790"	transcript_id "lnc-bbut00016279"	exon_number "2"
15	lncRNA	exon	70013405	70015754	.	+	.	gene_id "lnc-bbug06791"	transcript_id "lnc-bbut00016280"	exon_number "1"
15	lncRNA	exon	70020401	70023564	.	+	.	gene_id "lnc-bbug06791"	transcript_id "lnc-bbut00016280"	exon_number "2"
15	lncRNA	exon	70030692	70031227	.	+	.	gene_id "lnc-bbug06791"	transcript_id "lnc-bbut00016280"	exon_number "3"
15	lncRNA	exon	70927789	70927829	.	+	.	gene_id "lnc-bbug06792"	transcript_id "lnc-bbut00016281"	exon_number "1"
15	lncRNA	exon	70928353	70928417	.	+	.	gene_id "lnc-bbug06792"	transcript_id "lnc-bbut00016281"	exon_number "2"
15	lncRNA	exon	70938317	70939696	.	+	.	gene_id "lnc-bbug06792"	transcript_id "lnc-bbut00016281"	exon_number "3"
15	lncRNA	exon	72659911	72660138	.	+	.	gene_id "lnc-bbug06797"	transcript_id "lnc-bbut00016286"	exon_number "1"
15	lncRNA	exon	72685741	72685856	.	+	.	gene_id "lnc-bbug06797"	transcript_id "lnc-bbut00016286"	exon_number "2"
15	lncRNA	exon	72686154	72686272	.	+	.	gene_id "lnc-bbug06797"	transcript_id "lnc-bbut00016286"	exon_number "3"
15	lncRNA	exon	72742248	72743401	.	+	.	gene_id "lnc-bbug06797"	transcript_id "lnc-bbut00016286"	exon_number "4"
15	lncRNA	exon	74069219	74069357	.	+	.	gene_id "lnc-bbug06801"	transcript_id "lnc-bbut00016309"	exon_number "1"
15	lncRNA	exon	74075906	74076065	.	+	.	gene_id "lnc-bbug06801"	transcript_id "lnc-bbut00016309"	exon_number "2"
15	lncRNA	exon	75328561	75330518	.	+	.	gene_id "lnc-bbug06804"	transcript_id "lnc-bbut00016312"	exon_number "1"
15	lncRNA	exon	75331786	75331946	.	+	.	gene_id "lnc-bbug06804"	transcript_id "lnc-bbut00016312"	exon_number "2"
15	lncRNA	exon	75332169	75332220	.	+	.	gene_id "lnc-bbug06804"	transcript_id "lnc-bbut00016312"	exon_number "3"
15	lncRNA	exon	75340520	75340582	.	+	.	gene_id "lnc-bbug06804"	transcript_id "lnc-bbut00016312"	exon_number "4"
15	lncRNA	exon	75341523	75341891	.	+	.	gene_id "lnc-bbug06804"	transcript_id "lnc-bbut00016312"	exon_number "5"
15	lncRNA	exon	75343803	75343891	.	+	.	gene_id "lnc-bbug06804"	transcript_id "lnc-bbut00016312"	exon_number "6"
15	lncRNA	exon	75344339	75344598	.	+	.	gene_id "lnc-bbug06804"	transcript_id "lnc-bbut00016312"	exon_number "7"
15	lncRNA	exon	78245044	78245176	.	+	.	gene_id "lnc-bbug06806"	transcript_id "lnc-bbut00016314"	exon_number "1"
15	lncRNA	exon	78256188	78256246	.	+	.	gene_id "lnc-bbug06806"	transcript_id "lnc-bbut00016314"	exon_number "2"
15	lncRNA	exon	78259467	78259573	.	+	.	gene_id "lnc-bbug06806"	transcript_id "lnc-bbut00016314"	exon_number "3"
15	lncRNA	exon	78260685	78260850	.	+	.	gene_id "lnc-bbug06806"	transcript_id "lnc-bbut00016314"	exon_number "4"
15	lncRNA	exon	79887766	79888297	.	+	.	gene_id "lnc-bbug06810"	transcript_id "lnc-bbut00016325"	exon_number "1"
15	lncRNA	exon	79890518	79890601	.	+	.	gene_id "lnc-bbug06810"	transcript_id "lnc-bbut00016325"	exon_number "2"
15	lncRNA	exon	79891084	79891297	.	+	.	gene_id "lnc-bbug06810"	transcript_id "lnc-bbut00016325"	exon_number "3"
15	lncRNA	exon	80570744	80571031	.	+	.	gene_id "lnc-bbug06813"	transcript_id "lnc-bbut00016328"	exon_number "1"
15	lncRNA	exon	80571657	80571975	.	+	.	gene_id "lnc-bbug06813"	transcript_id "lnc-bbut00016328"	exon_number "2"
15	lncRNA	exon	81281501	81282069	.	+	.	gene_id "lnc-bbug06829"	transcript_id "lnc-bbut00016356"	exon_number "1"
15	lncRNA	exon	81283011	81287193	.	+	.	gene_id "lnc-bbug06829"	transcript_id "lnc-bbut00016356"	exon_number "2"
15	lncRNA	exon	81530612	81531041	.	+	.	gene_id "lnc-bbug06833"	transcript_id "lnc-bbut00016363"	exon_number "1"
15	lncRNA	exon	81534585	81535481	.	+	.	gene_id "lnc-bbug06833"	transcript_id "lnc-bbut00016363"	exon_number "2"
15	lncRNA	exon	81877744	81878054	.	+	.	gene_id "lnc-bbug06850"	transcript_id "lnc-bbut00016421"	exon_number "1"
15	lncRNA	exon	81878343	81878998	.	+	.	gene_id "lnc-bbug06850"	transcript_id "lnc-bbut00016421"	exon_number "2"
15	lncRNA	exon	82043253	82043331	.	+	.	gene_id "lnc-bbug06855"	transcript_id "lnc-bbut00016436"	exon_number "1"
15	lncRNA	exon	82049379	82049471	.	+	.	gene_id "lnc-bbug06855"	transcript_id "lnc-bbut00016436"	exon_number "2"
15	lncRNA	exon	82049691	82049817	.	+	.	gene_id "lnc-bbug06855"	transcript_id "lnc-bbut00016436"	exon_number "3"

15	lncRNA	exon	82049906	82050019	.	+	.	gene_id "lnc-bbug06855"	transcript_id "lnc-bbut00016436"	exon_number "4"
15	lncRNA	exon	82050495	82051392	.	+	.	gene_id "lnc-bbug06855"	transcript_id "lnc-bbut00016436"	exon_number "5"
15	lncRNA	exon	82065039	82065545	.	+	.	gene_id "lnc-bbug06856"	transcript_id "lnc-bbut00016440"	exon_number "1"
15	lncRNA	exon	82074239	82074384	.	+	.	gene_id "lnc-bbug06856"	transcript_id "lnc-bbut00016440"	exon_number "2"
15	lncRNA	exon	82082689	82082749	.	+	.	gene_id "lnc-bbug06856"	transcript_id "lnc-bbut00016440"	exon_number "3"
15	lncRNA	exon	82082870	82083821	.	+	.	gene_id "lnc-bbug06856"	transcript_id "lnc-bbut00016440"	exon_number "4"
15	lncRNA	exon	1109834	1113703	.	-	.	gene_id "lnc-bbug06860"	transcript_id "lnc-bbut00016453"	exon_number "1"
15	lncRNA	exon	1114602	1114736	.	-	.	gene_id "lnc-bbug06860"	transcript_id "lnc-bbut00016453"	exon_number "2"
15	lncRNA	exon	1492406	1492512	.	-	.	gene_id "lnc-bbug06865"	transcript_id "lnc-bbut00016467"	exon_number "1"
15	lncRNA	exon	1499825	1499915	.	-	.	gene_id "lnc-bbug06865"	transcript_id "lnc-bbut00016467"	exon_number "2"
15	lncRNA	exon	1501472	1502742	.	-	.	gene_id "lnc-bbug06865"	transcript_id "lnc-bbut00016467"	exon_number "3"
15	lncRNA	exon	1772291	1772528	.	-	.	gene_id "lnc-bbug06866"	transcript_id "lnc-bbut00016468"	exon_number "1"
15	lncRNA	exon	1774164	1774400	.	-	.	gene_id "lnc-bbug06866"	transcript_id "lnc-bbut00016468"	exon_number "2"
15	lncRNA	exon	1777381	1777590	.	-	.	gene_id "lnc-bbug06866"	transcript_id "lnc-bbut00016468"	exon_number "3"
15	lncRNA	exon	4951450	4952142	.	-	.	gene_id "lnc-bbug06870"	transcript_id "lnc-bbut00016476"	exon_number "1"
15	lncRNA	exon	4953012	4953167	.	-	.	gene_id "lnc-bbug06870"	transcript_id "lnc-bbut00016476"	exon_number "2"
15	lncRNA	exon	4955629	4955691	.	-	.	gene_id "lnc-bbug06870"	transcript_id "lnc-bbut00016476"	exon_number "3"
15	lncRNA	exon	4956412	4956507	.	-	.	gene_id "lnc-bbug06870"	transcript_id "lnc-bbut00016476"	exon_number "4"
15	lncRNA	exon	4959262	4959381	.	-	.	gene_id "lnc-bbug06870"	transcript_id "lnc-bbut00016476"	exon_number "5"
15	lncRNA	exon	4960855	4961052	.	-	.	gene_id "lnc-bbug06870"	transcript_id "lnc-bbut00016476"	exon_number "6"
15	lncRNA	exon	4961537	4961671	.	-	.	gene_id "lnc-bbug06870"	transcript_id "lnc-bbut00016476"	exon_number "7"
15	lncRNA	exon	5199634	5201330	.	-	.	gene_id "lnc-bbug06872"	transcript_id "lnc-bbut00016479"	exon_number "1"
15	lncRNA	exon	5202293	5202439	.	-	.	gene_id "lnc-bbug06872"	transcript_id "lnc-bbut00016479"	exon_number "2"
15	lncRNA	exon	5209946	5210158	.	-	.	gene_id "lnc-bbug06872"	transcript_id "lnc-bbut00016479"	exon_number "3"
15	lncRNA	exon	5471906	5472217	.	-	.	gene_id "lnc-bbug06873"	transcript_id "lnc-bbut00016480"	exon_number "1"
15	lncRNA	exon	5472319	5472614	.	-	.	gene_id "lnc-bbug06873"	transcript_id "lnc-bbut00016480"	exon_number "2"
15	lncRNA	exon	5481476	5481623	.	-	.	gene_id "lnc-bbug06873"	transcript_id "lnc-bbut00016480"	exon_number "3"
15	lncRNA	exon	5520657	5520959	.	-	.	gene_id "lnc-bbug06874"	transcript_id "lnc-bbut00016481"	exon_number "1"
15	lncRNA	exon	5528952	5529607	.	-	.	gene_id "lnc-bbug06874"	transcript_id "lnc-bbut00016481"	exon_number "2"
15	lncRNA	exon	5535008	5535072	.	-	.	gene_id "lnc-bbug06874"	transcript_id "lnc-bbut00016481"	exon_number "3"
15	lncRNA	exon	5606644	5606916	.	-	.	gene_id "lnc-bbug06875"	transcript_id "lnc-bbut00016482"	exon_number "1"
15	lncRNA	exon	5606997	5607109	.	-	.	gene_id "lnc-bbug06875"	transcript_id "lnc-bbut00016482"	exon_number "2"
15	lncRNA	exon	5614018	5615369	.	-	.	gene_id "lnc-bbug06875"	transcript_id "lnc-bbut00016482"	exon_number "3"
15	lncRNA	exon	5703316	5704118	.	-	.	gene_id "lnc-bbug06877"	transcript_id "lnc-bbut00016484"	exon_number "1"
15	lncRNA	exon	5706492	5706586	.	-	.	gene_id "lnc-bbug06877"	transcript_id "lnc-bbut00016484"	exon_number "2"
15	lncRNA	exon	8061610	8061856	.	-	.	gene_id "lnc-bbug06883"	transcript_id "lnc-bbut00016496"	exon_number "1"
15	lncRNA	exon	8062159	8062388	.	-	.	gene_id "lnc-bbug06883"	transcript_id "lnc-bbut00016496"	exon_number "2"
15	lncRNA	exon	8711190	8712032	.	-	.	gene_id "lnc-bbug06888"	transcript_id "lnc-bbut00016504"	exon_number "1"
15	lncRNA	exon	8723080	8723186	.	-	.	gene_id "lnc-bbug06888"	transcript_id "lnc-bbut00016504"	exon_number "2"
15	lncRNA	exon	8727680	8727752	.	-	.	gene_id "lnc-bbug06888"	transcript_id "lnc-bbut00016504"	exon_number "3"
15	lncRNA	exon	8736516	8736589	.	-	.	gene_id "lnc-bbug06888"	transcript_id "lnc-bbut00016504"	exon_number "4"
15	lncRNA	exon	8739898	8739996	.	-	.	gene_id "lnc-bbug06888"	transcript_id "lnc-bbut00016504"	exon_number "5"
15	lncRNA	exon	8747607	8747797	.	-	.	gene_id "lnc-bbug06888"	transcript_id "lnc-bbut00016504"	exon_number "6"
15	lncRNA	exon	8716959	8717008	.	-	.	gene_id "lnc-bbug06888"	transcript_id "lnc-bbut00016506"	exon_number "1"
15	lncRNA	exon	8723080	8723186	.	-	.	gene_id "lnc-bbug06888"	transcript_id "lnc-bbut00016506"	exon_number "2"
15	lncRNA	exon	8727680	8727752	.	-	.	gene_id "lnc-bbug06888"	transcript_id "lnc-bbut00016506"	exon_number "3"
15	lncRNA	exon	8736516	8736589	.	-	.	gene_id "lnc-bbug06888"	transcript_id "lnc-bbut00016506"	exon_number "4"
15	lncRNA	exon	8739898	8739996	.	-	.	gene_id "lnc-bbug06888"	transcript_id "lnc-bbut00016506"	exon_number "5"
15	lncRNA	exon	8747607	8748020	.	-	.	gene_id "lnc-bbug06888"	transcript_id "lnc-bbut00016506"	exon_number "6"
15	lncRNA	exon	8867312	8868587	.	-	.	gene_id "lnc-bbug06890"	transcript_id "lnc-bbut00016508"	exon_number "1"
15	lncRNA	exon	8868696	8868830	.	-	.	gene_id "lnc-bbug06890"	transcript_id "lnc-bbut00016508"	exon_number "2"
15	lncRNA	exon	10786747	10789873	.	-	.	gene_id "lnc-bbug06893"	transcript_id "lnc-bbut00016519"	exon_number "1"

15	lncRNA	exon	10795200	10795285	.	-	.	gene_id "lnc-bbug06893"	transcript_id "lnc-bbut00016519"	exon_number "2"
15	lncRNA	exon	10829832	10829913	.	-	.	gene_id "lnc-bbug06893"	transcript_id "lnc-bbut00016519"	exon_number "3"
15	lncRNA	exon	10871047	10871201	.	-	.	gene_id "lnc-bbug06893"	transcript_id "lnc-bbut00016519"	exon_number "4"
15	lncRNA	exon	10879035	10879885	.	-	.	gene_id "lnc-bbug06893"	transcript_id "lnc-bbut00016519"	exon_number "5"
15	lncRNA	exon	10786747	10789873	.	-	.	gene_id "lnc-bbug06893"	transcript_id "lnc-bbut00016520"	exon_number "1"
15	lncRNA	exon	10795200	10795285	.	-	.	gene_id "lnc-bbug06893"	transcript_id "lnc-bbut00016520"	exon_number "2"
15	lncRNA	exon	10829832	10829910	.	-	.	gene_id "lnc-bbug06893"	transcript_id "lnc-bbut00016520"	exon_number "3"
15	lncRNA	exon	10871047	10871201	.	-	.	gene_id "lnc-bbug06893"	transcript_id "lnc-bbut00016520"	exon_number "4"
15	lncRNA	exon	10879035	10879897	.	-	.	gene_id "lnc-bbug06893"	transcript_id "lnc-bbut00016520"	exon_number "5"
15	lncRNA	exon	10786747	10789873	.	-	.	gene_id "lnc-bbug06893"	transcript_id "lnc-bbut00016521"	exon_number "1"
15	lncRNA	exon	10795200	10795285	.	-	.	gene_id "lnc-bbug06893"	transcript_id "lnc-bbut00016521"	exon_number "2"
15	lncRNA	exon	10829832	10829913	.	-	.	gene_id "lnc-bbug06893"	transcript_id "lnc-bbut00016521"	exon_number "3"
15	lncRNA	exon	10889999	10890097	.	-	.	gene_id "lnc-bbug06893"	transcript_id "lnc-bbut00016521"	exon_number "4"
15	lncRNA	exon	10786747	10789873	.	-	.	gene_id "lnc-bbug06893"	transcript_id "lnc-bbut00016522"	exon_number "1"
15	lncRNA	exon	10795200	10795285	.	-	.	gene_id "lnc-bbug06893"	transcript_id "lnc-bbut00016522"	exon_number "2"
15	lncRNA	exon	10829832	10829913	.	-	.	gene_id "lnc-bbug06893"	transcript_id "lnc-bbut00016522"	exon_number "3"
15	lncRNA	exon	10832474	10832567	.	-	.	gene_id "lnc-bbug06893"	transcript_id "lnc-bbut00016522"	exon_number "4"
15	lncRNA	exon	10871047	10871201	.	-	.	gene_id "lnc-bbug06893"	transcript_id "lnc-bbut00016522"	exon_number "5"
15	lncRNA	exon	10889999	10890121	.	-	.	gene_id "lnc-bbug06893"	transcript_id "lnc-bbut00016522"	exon_number "6"
15	lncRNA	exon	11077811	11078227	.	-	.	gene_id "lnc-bbug06895"	transcript_id "lnc-bbut00016524"	exon_number "1"
15	lncRNA	exon	11080380	11080536	.	-	.	gene_id "lnc-bbug06895"	transcript_id "lnc-bbut00016524"	exon_number "2"
15	lncRNA	exon	11090639	11090905	.	-	.	gene_id "lnc-bbug06895"	transcript_id "lnc-bbut00016524"	exon_number "3"
15	lncRNA	exon	11579573	11579622	.	-	.	gene_id "lnc-bbug06897"	transcript_id "lnc-bbut00016527"	exon_number "1"
15	lncRNA	exon	11581938	11582038	.	-	.	gene_id "lnc-bbug06897"	transcript_id "lnc-bbut00016527"	exon_number "2"
15	lncRNA	exon	11602014	11602156	.	-	.	gene_id "lnc-bbug06897"	transcript_id "lnc-bbut00016527"	exon_number "3"
15	lncRNA	exon	11602603	11602761	.	-	.	gene_id "lnc-bbug06897"	transcript_id "lnc-bbut00016527"	exon_number "4"
15	lncRNA	exon	11608049	11608371	.	-	.	gene_id "lnc-bbug06897"	transcript_id "lnc-bbut00016527"	exon_number "5"
15	lncRNA	exon	11579573	11579622	.	-	.	gene_id "lnc-bbug06897"	transcript_id "lnc-bbut00016526"	exon_number "1"
15	lncRNA	exon	11581938	11582038	.	-	.	gene_id "lnc-bbug06897"	transcript_id "lnc-bbut00016526"	exon_number "2"
15	lncRNA	exon	11602014	11602156	.	-	.	gene_id "lnc-bbug06897"	transcript_id "lnc-bbut00016526"	exon_number "3"
15	lncRNA	exon	11608049	11608371	.	-	.	gene_id "lnc-bbug06897"	transcript_id "lnc-bbut00016526"	exon_number "4"
15	lncRNA	exon	12508666	12510005	.	-	.	gene_id "lnc-bbug06905"	transcript_id "lnc-bbut00016551"	exon_number "1"
15	lncRNA	exon	12512374	12512518	.	-	.	gene_id "lnc-bbug06905"	transcript_id "lnc-bbut00016551"	exon_number "2"
15	lncRNA	exon	12514504	12514618	.	-	.	gene_id "lnc-bbug06905"	transcript_id "lnc-bbut00016551"	exon_number "3"
15	lncRNA	exon	12742651	12743483	.	-	.	gene_id "lnc-bbug06907"	transcript_id "lnc-bbut00016555"	exon_number "1"
15	lncRNA	exon	12744250	12744385	.	-	.	gene_id "lnc-bbug06907"	transcript_id "lnc-bbut00016555"	exon_number "2"
15	lncRNA	exon	12744861	12744908	.	-	.	gene_id "lnc-bbug06907"	transcript_id "lnc-bbut00016555"	exon_number "3"
15	lncRNA	exon	12742651	12743483	.	-	.	gene_id "lnc-bbug06907"	transcript_id "lnc-bbut00016556"	exon_number "1"
15	lncRNA	exon	12744250	12744385	.	-	.	gene_id "lnc-bbug06907"	transcript_id "lnc-bbut00016556"	exon_number "2"
15	lncRNA	exon	12745571	12745661	.	-	.	gene_id "lnc-bbug06907"	transcript_id "lnc-bbut00016556"	exon_number "3"
15	lncRNA	exon	12742651	12743483	.	-	.	gene_id "lnc-bbug06907"	transcript_id "lnc-bbut00016557"	exon_number "1"
15	lncRNA	exon	12744250	12745838	.	-	.	gene_id "lnc-bbug06907"	transcript_id "lnc-bbut00016557"	exon_number "2"
15	lncRNA	exon	12789097	12789296	.	-	.	gene_id "lnc-bbug06909"	transcript_id "lnc-bbut00016561"	exon_number "1"
15	lncRNA	exon	12793892	12794051	.	-	.	gene_id "lnc-bbug06909"	transcript_id "lnc-bbut00016561"	exon_number "2"
15	lncRNA	exon	12794357	12794474	.	-	.	gene_id "lnc-bbug06909"	transcript_id "lnc-bbut00016561"	exon_number "3"
15	lncRNA	exon	13060680	13063722	.	-	.	gene_id "lnc-bbug06910"	transcript_id "lnc-bbut00016562"	exon_number "1"
15	lncRNA	exon	13064019	13064155	.	-	.	gene_id "lnc-bbug06910"	transcript_id "lnc-bbut00016562"	exon_number "2"
15	lncRNA	exon	13077561	13080001	.	-	.	gene_id "lnc-bbug06910"	transcript_id "lnc-bbut00016562"	exon_number "3"
15	lncRNA	exon	14487350	14488705	.	-	.	gene_id "lnc-bbug06917"	transcript_id "lnc-bbut00016571"	exon_number "1"
15	lncRNA	exon	14491575	14491685	.	-	.	gene_id "lnc-bbug06917"	transcript_id "lnc-bbut00016571"	exon_number "2"
15	lncRNA	exon	14491969	14492139	.	-	.	gene_id "lnc-bbug06917"	transcript_id "lnc-bbut00016571"	exon_number "3"
15	lncRNA	exon	15465747	15466021	.	-	.	gene_id "lnc-bbug06919"	transcript_id "lnc-bbut00016574"	exon_number "1"

15	lncRNA	exon	15466960	15467043	.	-	.	gene_id "lnc-bbug06919"	transcript_id "lnc-bbut00016574"	exon_number "2"
15	lncRNA	exon	15487100	15487387	.	-	.	gene_id "lnc-bbug06919"	transcript_id "lnc-bbut00016574"	exon_number "3"
15	lncRNA	exon	15493298	15493675	.	-	.	gene_id "lnc-bbug06920"	transcript_id "lnc-bbut00016575"	exon_number "1"
15	lncRNA	exon	15494317	15494644	.	-	.	gene_id "lnc-bbug06920"	transcript_id "lnc-bbut00016575"	exon_number "2"
15	lncRNA	exon	15500504	15500839	.	-	.	gene_id "lnc-bbug06921"	transcript_id "lnc-bbut00016576"	exon_number "1"
15	lncRNA	exon	15500971	15501026	.	-	.	gene_id "lnc-bbug06921"	transcript_id "lnc-bbut00016576"	exon_number "2"
15	lncRNA	exon	15501516	15501809	.	-	.	gene_id "lnc-bbug06921"	transcript_id "lnc-bbut00016576"	exon_number "3"
15	lncRNA	exon	15517249	15518015	.	-	.	gene_id "lnc-bbug06922"	transcript_id "lnc-bbut00016577"	exon_number "1"
15	lncRNA	exon	18351614	18352739	.	-	.	gene_id "lnc-bbug06930"	transcript_id "lnc-bbut00016594"	exon_number "1"
15	lncRNA	exon	18353166	18353318	.	-	.	gene_id "lnc-bbug06930"	transcript_id "lnc-bbut00016594"	exon_number "2"
15	lncRNA	exon	18704349	18704536	.	-	.	gene_id "lnc-bbug06934"	transcript_id "lnc-bbut00016602"	exon_number "1"
15	lncRNA	exon	18705703	18705821	.	-	.	gene_id "lnc-bbug06934"	transcript_id "lnc-bbut00016602"	exon_number "2"
15	lncRNA	exon	18745921	18746086	.	-	.	gene_id "lnc-bbug06934"	transcript_id "lnc-bbut00016602"	exon_number "3"
15	lncRNA	exon	18795160	18796419	.	-	.	gene_id "lnc-bbug06935"	transcript_id "lnc-bbut00016603"	exon_number "1"
15	lncRNA	exon	18796504	18796839	.	-	.	gene_id "lnc-bbug06935"	transcript_id "lnc-bbut00016603"	exon_number "2"
15	lncRNA	exon	19066140	19068340	.	-	.	gene_id "lnc-bbug06937"	transcript_id "lnc-bbut00016606"	exon_number "1"
15	lncRNA	exon	19069017	19069124	.	-	.	gene_id "lnc-bbug06937"	transcript_id "lnc-bbut00016606"	exon_number "2"
15	lncRNA	exon	19071623	19071759	.	-	.	gene_id "lnc-bbug06937"	transcript_id "lnc-bbut00016606"	exon_number "3"
15	lncRNA	exon	19074953	19074976	.	-	.	gene_id "lnc-bbug06937"	transcript_id "lnc-bbut00016606"	exon_number "4"
15	lncRNA	exon	19066140	19068340	.	-	.	gene_id "lnc-bbug06937"	transcript_id "lnc-bbut00016607"	exon_number "1"
15	lncRNA	exon	19069017	19069124	.	-	.	gene_id "lnc-bbug06937"	transcript_id "lnc-bbut00016607"	exon_number "2"
15	lncRNA	exon	19071623	19071759	.	-	.	gene_id "lnc-bbug06937"	transcript_id "lnc-bbut00016607"	exon_number "3"
15	lncRNA	exon	19075351	19075558	.	-	.	gene_id "lnc-bbug06937"	transcript_id "lnc-bbut00016607"	exon_number "4"
15	lncRNA	exon	19066140	19068340	.	-	.	gene_id "lnc-bbug06937"	transcript_id "lnc-bbut00016608"	exon_number "1"
15	lncRNA	exon	19069017	19069124	.	-	.	gene_id "lnc-bbug06937"	transcript_id "lnc-bbut00016608"	exon_number "2"
15	lncRNA	exon	19071623	19071759	.	-	.	gene_id "lnc-bbug06937"	transcript_id "lnc-bbut00016608"	exon_number "3"
15	lncRNA	exon	19075964	19076088	.	-	.	gene_id "lnc-bbug06937"	transcript_id "lnc-bbut00016608"	exon_number "4"
15	lncRNA	exon	19294475	19294741	.	-	.	gene_id "lnc-bbug06938"	transcript_id "lnc-bbut00016609"	exon_number "1"
15	lncRNA	exon	19302332	19303636	.	-	.	gene_id "lnc-bbug06938"	transcript_id "lnc-bbut00016609"	exon_number "2"
15	lncRNA	exon	19308048	19308327	.	-	.	gene_id "lnc-bbug06938"	transcript_id "lnc-bbut00016609"	exon_number "3"
15	lncRNA	exon	19337701	19337808	.	-	.	gene_id "lnc-bbug06938"	transcript_id "lnc-bbut00016609"	exon_number "4"
15	lncRNA	exon	19301711	19303636	.	-	.	gene_id "lnc-bbug06938"	transcript_id "lnc-bbut00016610"	exon_number "1"
15	lncRNA	exon	19308048	19308327	.	-	.	gene_id "lnc-bbug06938"	transcript_id "lnc-bbut00016610"	exon_number "2"
15	lncRNA	exon	19337701	19337808	.	-	.	gene_id "lnc-bbug06938"	transcript_id "lnc-bbut00016610"	exon_number "3"
15	lncRNA	exon	19307204	19307254	.	-	.	gene_id "lnc-bbug06938"	transcript_id "lnc-bbut00016611"	exon_number "1"
15	lncRNA	exon	19308048	19308327	.	-	.	gene_id "lnc-bbug06938"	transcript_id "lnc-bbut00016611"	exon_number "2"
15	lncRNA	exon	19337701	19337808	.	-	.	gene_id "lnc-bbug06938"	transcript_id "lnc-bbut00016611"	exon_number "3"
15	lncRNA	exon	20288059	20289769	.	-	.	gene_id "lnc-bbug06944"	transcript_id "lnc-bbut00016624"	exon_number "1"
15	lncRNA	exon	20353110	20353199	.	-	.	gene_id "lnc-bbug06944"	transcript_id "lnc-bbut00016624"	exon_number "2"
15	lncRNA	exon	20354851	20355072	.	-	.	gene_id "lnc-bbug06944"	transcript_id "lnc-bbut00016624"	exon_number "3"
15	lncRNA	exon	20355665	20355781	.	-	.	gene_id "lnc-bbug06944"	transcript_id "lnc-bbut00016624"	exon_number "4"
15	lncRNA	exon	20360207	20360373	.	-	.	gene_id "lnc-bbug06944"	transcript_id "lnc-bbut00016624"	exon_number "5"
15	lncRNA	exon	20375050	20376230	.	-	.	gene_id "lnc-bbug06944"	transcript_id "lnc-bbut00016624"	exon_number "6"
15	lncRNA	exon	20343617	20345307	.	-	.	gene_id "lnc-bbug06944"	transcript_id "lnc-bbut00016626"	exon_number "1"
15	lncRNA	exon	20353110	20353199	.	-	.	gene_id "lnc-bbug06944"	transcript_id "lnc-bbut00016626"	exon_number "2"
15	lncRNA	exon	20354851	20355072	.	-	.	gene_id "lnc-bbug06944"	transcript_id "lnc-bbut00016626"	exon_number "3"
15	lncRNA	exon	20355665	20355781	.	-	.	gene_id "lnc-bbug06944"	transcript_id "lnc-bbut00016626"	exon_number "4"
15	lncRNA	exon	20360207	20360373	.	-	.	gene_id "lnc-bbug06944"	transcript_id "lnc-bbut00016626"	exon_number "5"
15	lncRNA	exon	20375050	20376230	.	-	.	gene_id "lnc-bbug06944"	transcript_id "lnc-bbut00016626"	exon_number "6"
15	lncRNA	exon	20343617	20345307	.	-	.	gene_id "lnc-bbug06944"	transcript_id "lnc-bbut00016625"	exon_number "1"
15	lncRNA	exon	20353110	20353199	.	-	.	gene_id "lnc-bbug06944"	transcript_id "lnc-bbut00016625"	exon_number "2"
15	lncRNA	exon	20354851	20355072	.	-	.	gene_id "lnc-bbug06944"	transcript_id "lnc-bbut00016625"	exon_number "3"

15	lncRNA	exon	20355665	20355781	.	-	.	gene_id "lnc-bbug06944"	transcript_id "lnc-bbut00016625"	exon_number "4"
15	lncRNA	exon	20375050	20376230	.	-	.	gene_id "lnc-bbug06944"	transcript_id "lnc-bbut00016625"	exon_number "5"
15	lncRNA	exon	20581370	20582480	.	-	.	gene_id "lnc-bbug06946"	transcript_id "lnc-bbut00016630"	exon_number "1"
15	lncRNA	exon	20585802	20586015	.	-	.	gene_id "lnc-bbug06946"	transcript_id "lnc-bbut00016630"	exon_number "2"
15	lncRNA	exon	20594989	20595229	.	-	.	gene_id "lnc-bbug06946"	transcript_id "lnc-bbut00016630"	exon_number "3"
15	lncRNA	exon	20595870	20596482	.	-	.	gene_id "lnc-bbug06946"	transcript_id "lnc-bbut00016630"	exon_number "4"
15	lncRNA	exon	20836941	20837524	.	-	.	gene_id "lnc-bbug06948"	transcript_id "lnc-bbut00016637"	exon_number "1"
15	lncRNA	exon	21001713	21002223	.	-	.	gene_id "lnc-bbug06949"	transcript_id "lnc-bbut00016638"	exon_number "1"
15	lncRNA	exon	25991710	25992685	.	-	.	gene_id "lnc-bbug06957"	transcript_id "lnc-bbut00016652"	exon_number "1"
15	lncRNA	exon	25992813	25994995	.	-	.	gene_id "lnc-bbug06957"	transcript_id "lnc-bbut00016652"	exon_number "2"
15	lncRNA	exon	26089822	26090109	.	-	.	gene_id "lnc-bbug06958"	transcript_id "lnc-bbut00016653"	exon_number "1"
15	lncRNA	exon	26220323	26220469	.	-	.	gene_id "lnc-bbug06958"	transcript_id "lnc-bbut00016653"	exon_number "2"
15	lncRNA	exon	30975800	30977632	.	-	.	gene_id "lnc-bbug06964"	transcript_id "lnc-bbut00016669"	exon_number "1"
15	lncRNA	exon	30977712	30977783	.	-	.	gene_id "lnc-bbug06964"	transcript_id "lnc-bbut00016669"	exon_number "2"
15	lncRNA	exon	33368377	33368521	.	-	.	gene_id "lnc-bbug06967"	transcript_id "lnc-bbut00016676"	exon_number "1"
15	lncRNA	exon	33368698	33369315	.	-	.	gene_id "lnc-bbug06967"	transcript_id "lnc-bbut00016676"	exon_number "2"
15	lncRNA	exon	34437163	34438770	.	-	.	gene_id "lnc-bbug06968"	transcript_id "lnc-bbut00016677"	exon_number "1"
15	lncRNA	exon	34440678	34440808	.	-	.	gene_id "lnc-bbug06968"	transcript_id "lnc-bbut00016677"	exon_number "2"
15	lncRNA	exon	34443503	34443652	.	-	.	gene_id "lnc-bbug06968"	transcript_id "lnc-bbut00016677"	exon_number "3"
15	lncRNA	exon	34457716	34460196	.	-	.	gene_id "lnc-bbug06968"	transcript_id "lnc-bbut00016677"	exon_number "4"
15	lncRNA	exon	34492189	34492752	.	-	.	gene_id "lnc-bbug06969"	transcript_id "lnc-bbut00016678"	exon_number "1"
15	lncRNA	exon	34501587	34501694	.	-	.	gene_id "lnc-bbug06969"	transcript_id "lnc-bbut00016678"	exon_number "2"
15	lncRNA	exon	34538783	34539623	.	-	.	gene_id "lnc-bbug06969"	transcript_id "lnc-bbut00016678"	exon_number "3"
15	lncRNA	exon	36215463	36223907	.	-	.	gene_id "lnc-bbug06973"	transcript_id "lnc-bbut00016683"	exon_number "1"
15	lncRNA	exon	36408563	36408703	.	-	.	gene_id "lnc-bbug06973"	transcript_id "lnc-bbut00016683"	exon_number "2"
15	lncRNA	exon	36467698	36467827	.	-	.	gene_id "lnc-bbug06973"	transcript_id "lnc-bbut00016683"	exon_number "3"
15	lncRNA	exon	36624167	36624345	.	-	.	gene_id "lnc-bbug06973"	transcript_id "lnc-bbut00016683"	exon_number "4"
15	lncRNA	exon	36676376	36676503	.	-	.	gene_id "lnc-bbug06973"	transcript_id "lnc-bbut00016683"	exon_number "5"
15	lncRNA	exon	37230985	37231964	.	-	.	gene_id "lnc-bbug06975"	transcript_id "lnc-bbut00016687"	exon_number "1"
15	lncRNA	exon	37232779	37232851	.	-	.	gene_id "lnc-bbug06975"	transcript_id "lnc-bbut00016687"	exon_number "2"
15	lncRNA	exon	37302783	37303286	.	-	.	gene_id "lnc-bbug06976"	transcript_id "lnc-bbut00016688"	exon_number "1"
15	lncRNA	exon	37304558	37305409	.	-	.	gene_id "lnc-bbug06976"	transcript_id "lnc-bbut00016688"	exon_number "2"
15	lncRNA	exon	38166023	38167335	.	-	.	gene_id "lnc-bbug06978"	transcript_id "lnc-bbut00016690"	exon_number "1"
15	lncRNA	exon	38193342	38193408	.	-	.	gene_id "lnc-bbug06978"	transcript_id "lnc-bbut00016690"	exon_number "2"
15	lncRNA	exon	38201045	38201148	.	-	.	gene_id "lnc-bbug06978"	transcript_id "lnc-bbut00016690"	exon_number "3"
15	lncRNA	exon	38422430	38422695	.	-	.	gene_id "lnc-bbug06980"	transcript_id "lnc-bbut00016692"	exon_number "1"
15	lncRNA	exon	38499779	38499884	.	-	.	gene_id "lnc-bbug06980"	transcript_id "lnc-bbut00016692"	exon_number "2"
15	lncRNA	exon	38527454	38530926	.	-	.	gene_id "lnc-bbug06980"	transcript_id "lnc-bbut00016692"	exon_number "3"
15	lncRNA	exon	38497928	38498047	.	-	.	gene_id "lnc-bbug06980"	transcript_id "lnc-bbut00016693"	exon_number "1"
15	lncRNA	exon	38499779	38499884	.	-	.	gene_id "lnc-bbug06980"	transcript_id "lnc-bbut00016693"	exon_number "2"
15	lncRNA	exon	38527454	38530926	.	-	.	gene_id "lnc-bbug06980"	transcript_id "lnc-bbut00016693"	exon_number "3"
15	lncRNA	exon	38553999	38554277	.	-	.	gene_id "lnc-bbug06981"	transcript_id "lnc-bbut00016694"	exon_number "1"
15	lncRNA	exon	38555507	38555648	.	-	.	gene_id "lnc-bbug06981"	transcript_id "lnc-bbut00016694"	exon_number "2"
15	lncRNA	exon	38555854	38557563	.	-	.	gene_id "lnc-bbug06981"	transcript_id "lnc-bbut00016694"	exon_number "3"
15	lncRNA	exon	38818347	38819018	.	-	.	gene_id "lnc-bbug06983"	transcript_id "lnc-bbut00016696"	exon_number "1"
15	lncRNA	exon	39265187	39265366	.	-	.	gene_id "lnc-bbug06984"	transcript_id "lnc-bbut00016697"	exon_number "1"
15	lncRNA	exon	39270396	39270988	.	-	.	gene_id "lnc-bbug06984"	transcript_id "lnc-bbut00016697"	exon_number "2"
15	lncRNA	exon	39271079	39272719	.	-	.	gene_id "lnc-bbug06984"	transcript_id "lnc-bbut00016697"	exon_number "3"
15	lncRNA	exon	39590562	39590702	.	-	.	gene_id "lnc-bbug06987"	transcript_id "lnc-bbut00016700"	exon_number "1"
15	lncRNA	exon	39596343	39596899	.	-	.	gene_id "lnc-bbug06987"	transcript_id "lnc-bbut00016700"	exon_number "2"
15	lncRNA	exon	40303049	40303726	.	-	.	gene_id "lnc-bbug06989"	transcript_id "lnc-bbut00016702"	exon_number "1"
15	lncRNA	exon	40304434	40304580	.	-	.	gene_id "lnc-bbug06989"	transcript_id "lnc-bbut00016702"	exon_number "2"

15	lncRNA	exon	40371597	40372218	.	-	.	gene_id "lnc-bbug06990"	transcript_id "lnc-bbut00016703"	exon_number "1"
15	lncRNA	exon	40374095	40374449	.	-	.	gene_id "lnc-bbug06990"	transcript_id "lnc-bbut00016703"	exon_number "2"
15	lncRNA	exon	40479249	40482730	.	-	.	gene_id "lnc-bbug06992"	transcript_id "lnc-bbut00016707"	exon_number "1"
15	lncRNA	exon	40486513	40486690	.	-	.	gene_id "lnc-bbug06992"	transcript_id "lnc-bbut00016707"	exon_number "2"
15	lncRNA	exon	40520446	40520573	.	-	.	gene_id "lnc-bbug06992"	transcript_id "lnc-bbut00016707"	exon_number "3"
15	lncRNA	exon	40578487	40578607	.	-	.	gene_id "lnc-bbug06992"	transcript_id "lnc-bbut00016707"	exon_number "4"
15	lncRNA	exon	42091395	42092873	.	-	.	gene_id "lnc-bbug06994"	transcript_id "lnc-bbut00016709"	exon_number "1"
15	lncRNA	exon	42101775	42101903	.	-	.	gene_id "lnc-bbug06994"	transcript_id "lnc-bbut00016709"	exon_number "2"
15	lncRNA	exon	42103019	42103304	.	-	.	gene_id "lnc-bbug06994"	transcript_id "lnc-bbut00016709"	exon_number "3"
15	lncRNA	exon	44977283	44977729	.	-	.	gene_id "lnc-bbug07003"	transcript_id "lnc-bbut00016730"	exon_number "1"
15	lncRNA	exon	44981086	44981206	.	-	.	gene_id "lnc-bbug07003"	transcript_id "lnc-bbut00016730"	exon_number "2"
15	lncRNA	exon	44981927	44982146	.	-	.	gene_id "lnc-bbug07003"	transcript_id "lnc-bbut00016730"	exon_number "3"
15	lncRNA	exon	44990618	44992017	.	-	.	gene_id "lnc-bbug07004"	transcript_id "lnc-bbut00016731"	exon_number "1"
15	lncRNA	exon	44997091	44997911	.	-	.	gene_id "lnc-bbug07004"	transcript_id "lnc-bbut00016731"	exon_number "2"
15	lncRNA	exon	46693537	46694394	.	-	.	gene_id "lnc-bbug07009"	transcript_id "lnc-bbut00016737"	exon_number "1"
15	lncRNA	exon	46863072	46864232	.	-	.	gene_id "lnc-bbug07010"	transcript_id "lnc-bbut00016738"	exon_number "1"
15	lncRNA	exon	46883099	46883148	.	-	.	gene_id "lnc-bbug07011"	transcript_id "lnc-bbut00016739"	exon_number "1"
15	lncRNA	exon	46891430	46891520	.	-	.	gene_id "lnc-bbug07011"	transcript_id "lnc-bbut00016739"	exon_number "2"
15	lncRNA	exon	46893958	46894806	.	-	.	gene_id "lnc-bbug07011"	transcript_id "lnc-bbut00016739"	exon_number "3"
15	lncRNA	exon	46899776	46900322	.	-	.	gene_id "lnc-bbug07011"	transcript_id "lnc-bbut00016739"	exon_number "4"
15	lncRNA	exon	47819495	47820253	.	-	.	gene_id "lnc-bbug07013"	transcript_id "lnc-bbut00016741"	exon_number "1"
15	lncRNA	exon	49963061	49963740	.	-	.	gene_id "lnc-bbug07018"	transcript_id "lnc-bbut00016753"	exon_number "1"
15	lncRNA	exon	49965218	49965226	.	-	.	gene_id "lnc-bbug07018"	transcript_id "lnc-bbut00016753"	exon_number "2"
15	lncRNA	exon	49965822	49965883	.	-	.	gene_id "lnc-bbug07018"	transcript_id "lnc-bbut00016753"	exon_number "3"
15	lncRNA	exon	49966015	49966074	.	-	.	gene_id "lnc-bbug07018"	transcript_id "lnc-bbut00016753"	exon_number "4"
15	lncRNA	exon	50017715	50018001	.	-	.	gene_id "lnc-bbug07019"	transcript_id "lnc-bbut00016754"	exon_number "1"
15	lncRNA	exon	50019256	50019389	.	-	.	gene_id "lnc-bbug07019"	transcript_id "lnc-bbut00016754"	exon_number "2"
15	lncRNA	exon	50610567	50612429	.	-	.	gene_id "lnc-bbug07021"	transcript_id "lnc-bbut00016756"	exon_number "1"
15	lncRNA	exon	50718280	50718416	.	-	.	gene_id "lnc-bbug07021"	transcript_id "lnc-bbut00016756"	exon_number "2"
15	lncRNA	exon	50790160	50791090	.	-	.	gene_id "lnc-bbug07021"	transcript_id "lnc-bbut00016756"	exon_number "3"
15	lncRNA	exon	50610567	50612429	.	-	.	gene_id "lnc-bbug07021"	transcript_id "lnc-bbut00016757"	exon_number "1"
15	lncRNA	exon	50718280	50718416	.	-	.	gene_id "lnc-bbug07021"	transcript_id "lnc-bbut00016757"	exon_number "2"
15	lncRNA	exon	50801331	50802144	.	-	.	gene_id "lnc-bbug07021"	transcript_id "lnc-bbut00016757"	exon_number "3"
15	lncRNA	exon	51434574	51434814	.	-	.	gene_id "lnc-bbug07026"	transcript_id "lnc-bbut00016777"	exon_number "1"
15	lncRNA	exon	51445639	51445989	.	-	.	gene_id "lnc-bbug07026"	transcript_id "lnc-bbut00016777"	exon_number "2"
15	lncRNA	exon	52105799	52105906	.	-	.	gene_id "lnc-bbug07034"	transcript_id "lnc-bbut00016793"	exon_number "1"
15	lncRNA	exon	52107324	52107471	.	-	.	gene_id "lnc-bbug07034"	transcript_id "lnc-bbut00016793"	exon_number "2"
15	lncRNA	exon	52108213	52108852	.	-	.	gene_id "lnc-bbug07034"	transcript_id "lnc-bbut00016793"	exon_number "3"
15	lncRNA	exon	52821265	52821606	.	-	.	gene_id "lnc-bbug07036"	transcript_id "lnc-bbut00016798"	exon_number "1"
15	lncRNA	exon	52823909	52824005	.	-	.	gene_id "lnc-bbug07036"	transcript_id "lnc-bbut00016798"	exon_number "2"
15	lncRNA	exon	52824122	52824218	.	-	.	gene_id "lnc-bbug07036"	transcript_id "lnc-bbut00016798"	exon_number "3"
15	lncRNA	exon	52830818	52830944	.	-	.	gene_id "lnc-bbug07036"	transcript_id "lnc-bbut00016798"	exon_number "4"
15	lncRNA	exon	52875480	52875570	.	-	.	gene_id "lnc-bbug07036"	transcript_id "lnc-bbut00016798"	exon_number "5"
15	lncRNA	exon	52883020	52883416	.	-	.	gene_id "lnc-bbug07036"	transcript_id "lnc-bbut00016798"	exon_number "6"
15	lncRNA	exon	53210161	53217593	.	-	.	gene_id "lnc-bbug07038"	transcript_id "lnc-bbut00016800"	exon_number "1"
15	lncRNA	exon	53222155	53222514	.	-	.	gene_id "lnc-bbug07038"	transcript_id "lnc-bbut00016800"	exon_number "2"
15	lncRNA	exon	54285644	54285827	.	-	.	gene_id "lnc-bbug07040"	transcript_id "lnc-bbut00016815"	exon_number "1"
15	lncRNA	exon	54286645	54287927	.	-	.	gene_id "lnc-bbug07040"	transcript_id "lnc-bbut00016815"	exon_number "2"
15	lncRNA	exon	55151179	55151497	.	-	.	gene_id "lnc-bbug07043"	transcript_id "lnc-bbut00016819"	exon_number "1"
15	lncRNA	exon	55152954	55153060	.	-	.	gene_id "lnc-bbug07043"	transcript_id "lnc-bbut00016819"	exon_number "2"
15	lncRNA	exon	55612665	55613010	.	-	.	gene_id "lnc-bbug07046"	transcript_id "lnc-bbut00016822"	exon_number "1"
15	lncRNA	exon	55616918	55617203	.	-	.	gene_id "lnc-bbug07046"	transcript_id "lnc-bbut00016822"	exon_number "2"

15	lncRNA	exon	55722626	55723063	.	-	.	gene_id "lnc-bbug07048"	transcript_id "lnc-bbut00016824"	exon_number "1"
15	lncRNA	exon	55728481	55728588	.	-	.	gene_id "lnc-bbug07048"	transcript_id "lnc-bbut00016824"	exon_number "2"
15	lncRNA	exon	55730716	55731045	.	-	.	gene_id "lnc-bbug07048"	transcript_id "lnc-bbut00016824"	exon_number "3"
15	lncRNA	exon	55796480	55796912	.	-	.	gene_id "lnc-bbug07049"	transcript_id "lnc-bbut00016825"	exon_number "1"
15	lncRNA	exon	55802090	55802207	.	-	.	gene_id "lnc-bbug07049"	transcript_id "lnc-bbut00016825"	exon_number "2"
15	lncRNA	exon	56061523	56062668	.	-	.	gene_id "lnc-bbug07051"	transcript_id "lnc-bbut00016829"	exon_number "1"
15	lncRNA	exon	56063007	56063151	.	-	.	gene_id "lnc-bbug07051"	transcript_id "lnc-bbut00016829"	exon_number "2"
15	lncRNA	exon	56269856	56270431	.	-	.	gene_id "lnc-bbug07053"	transcript_id "lnc-bbut00016832"	exon_number "1"
15	lncRNA	exon	56278435	56278762	.	-	.	gene_id "lnc-bbug07053"	transcript_id "lnc-bbut00016832"	exon_number "2"
15	lncRNA	exon	56284743	56284892	.	-	.	gene_id "lnc-bbug07053"	transcript_id "lnc-bbut00016832"	exon_number "3"
15	lncRNA	exon	56286037	56286163	.	-	.	gene_id "lnc-bbug07053"	transcript_id "lnc-bbut00016832"	exon_number "4"
15	lncRNA	exon	56296157	56297056	.	-	.	gene_id "lnc-bbug07053"	transcript_id "lnc-bbut00016832"	exon_number "5"
15	lncRNA	exon	57619731	57620547	.	-	.	gene_id "lnc-bbug07055"	transcript_id "lnc-bbut00016834"	exon_number "1"
15	lncRNA	exon	57620859	57622184	.	-	.	gene_id "lnc-bbug07055"	transcript_id "lnc-bbut00016834"	exon_number "2"
15	lncRNA	exon	57723198	57723455	.	-	.	gene_id "lnc-bbug07056"	transcript_id "lnc-bbut00016835"	exon_number "1"
15	lncRNA	exon	57724435	57724541	.	-	.	gene_id "lnc-bbug07056"	transcript_id "lnc-bbut00016835"	exon_number "2"
15	lncRNA	exon	57742909	57743322	.	-	.	gene_id "lnc-bbug07056"	transcript_id "lnc-bbut00016835"	exon_number "3"
15	lncRNA	exon	58987595	58989888	.	-	.	gene_id "lnc-bbug07059"	transcript_id "lnc-bbut00016843"	exon_number "1"
15	lncRNA	exon	58991289	58992450	.	-	.	gene_id "lnc-bbug07059"	transcript_id "lnc-bbut00016843"	exon_number "2"
15	lncRNA	exon	58987595	58989888	.	-	.	gene_id "lnc-bbug07059"	transcript_id "lnc-bbut00016842"	exon_number "1"
15	lncRNA	exon	58990949	58992450	.	-	.	gene_id "lnc-bbug07059"	transcript_id "lnc-bbut00016842"	exon_number "2"
15	lncRNA	exon	58993881	58995114	.	-	.	gene_id "lnc-bbug07060"	transcript_id "lnc-bbut00016844"	exon_number "1"
15	lncRNA	exon	58996073	58996171	.	-	.	gene_id "lnc-bbug07060"	transcript_id "lnc-bbut00016844"	exon_number "2"
15	lncRNA	exon	58997738	58997807	.	-	.	gene_id "lnc-bbug07060"	transcript_id "lnc-bbut00016844"	exon_number "3"
15	lncRNA	exon	58999503	58999769	.	-	.	gene_id "lnc-bbug07060"	transcript_id "lnc-bbut00016844"	exon_number "4"
15	lncRNA	exon	59000900	59000980	.	-	.	gene_id "lnc-bbug07060"	transcript_id "lnc-bbut00016844"	exon_number "5"
15	lncRNA	exon	58993881	58995114	.	-	.	gene_id "lnc-bbug07060"	transcript_id "lnc-bbut00016845"	exon_number "1"
15	lncRNA	exon	58996073	58996171	.	-	.	gene_id "lnc-bbug07060"	transcript_id "lnc-bbut00016845"	exon_number "2"
15	lncRNA	exon	58997738	58997807	.	-	.	gene_id "lnc-bbug07060"	transcript_id "lnc-bbut00016845"	exon_number "3"
15	lncRNA	exon	59000900	59001027	.	-	.	gene_id "lnc-bbug07060"	transcript_id "lnc-bbut00016845"	exon_number "4"
15	lncRNA	exon	59269431	59269538	.	-	.	gene_id "lnc-bbug07063"	transcript_id "lnc-bbut00016851"	exon_number "1"
15	lncRNA	exon	59269629	59270352	.	-	.	gene_id "lnc-bbug07063"	transcript_id "lnc-bbut00016851"	exon_number "2"
15	lncRNA	exon	61300171	61300449	.	-	.	gene_id "lnc-bbug07072"	transcript_id "lnc-bbut00016872"	exon_number "1"
15	lncRNA	exon	61308637	61308857	.	-	.	gene_id "lnc-bbug07072"	transcript_id "lnc-bbut00016872"	exon_number "2"
15	lncRNA	exon	62364677	62367696	.	-	.	gene_id "lnc-bbug07074"	transcript_id "lnc-bbut00016874"	exon_number "1"
15	lncRNA	exon	62367943	62368042	.	-	.	gene_id "lnc-bbug07074"	transcript_id "lnc-bbut00016874"	exon_number "2"
15	lncRNA	exon	62368327	62368404	.	-	.	gene_id "lnc-bbug07074"	transcript_id "lnc-bbut00016874"	exon_number "3"
15	lncRNA	exon	62369726	62369820	.	-	.	gene_id "lnc-bbug07074"	transcript_id "lnc-bbut00016874"	exon_number "4"
15	lncRNA	exon	62370090	62370396	.	-	.	gene_id "lnc-bbug07074"	transcript_id "lnc-bbut00016874"	exon_number "5"
15	lncRNA	exon	62513143	62513463	.	-	.	gene_id "lnc-bbug07075"	transcript_id "lnc-bbut00016876"	exon_number "1"
15	lncRNA	exon	62520952	62521036	.	-	.	gene_id "lnc-bbug07075"	transcript_id "lnc-bbut00016876"	exon_number "2"
15	lncRNA	exon	62521233	62521369	.	-	.	gene_id "lnc-bbug07075"	transcript_id "lnc-bbut00016876"	exon_number "3"
15	lncRNA	exon	62523397	62523522	.	-	.	gene_id "lnc-bbug07075"	transcript_id "lnc-bbut00016876"	exon_number "4"
15	lncRNA	exon	62520420	62520774	.	-	.	gene_id "lnc-bbug07075"	transcript_id "lnc-bbut00016877"	exon_number "1"
15	lncRNA	exon	62520900	62521036	.	-	.	gene_id "lnc-bbug07075"	transcript_id "lnc-bbut00016877"	exon_number "2"
15	lncRNA	exon	62521233	62521369	.	-	.	gene_id "lnc-bbug07075"	transcript_id "lnc-bbut00016877"	exon_number "3"
15	lncRNA	exon	62523293	62523399	.	-	.	gene_id "lnc-bbug07075"	transcript_id "lnc-bbut00016877"	exon_number "4"
15	lncRNA	exon	62520420	62520774	.	-	.	gene_id "lnc-bbug07075"	transcript_id "lnc-bbut00016878"	exon_number "1"
15	lncRNA	exon	62520952	62521036	.	-	.	gene_id "lnc-bbug07075"	transcript_id "lnc-bbut00016878"	exon_number "2"
15	lncRNA	exon	62521233	62521369	.	-	.	gene_id "lnc-bbug07075"	transcript_id "lnc-bbut00016878"	exon_number "3"
15	lncRNA	exon	62523397	62523522	.	-	.	gene_id "lnc-bbug07075"	transcript_id "lnc-bbut00016878"	exon_number "4"
15	lncRNA	exon	62520420	62520774	.	-	.	gene_id "lnc-bbug07075"	transcript_id "lnc-bbut00016879"	exon_number "1"

15	lncRNA	exon	62520900	62521036	.	-	.	gene_id "lnc-bbug07075"	transcript_id "lnc-bbut00016879"	exon_number "2"
15	lncRNA	exon	62521233	62521369	.	-	.	gene_id "lnc-bbug07075"	transcript_id "lnc-bbut00016879"	exon_number "3"
15	lncRNA	exon	62523397	62523523	.	-	.	gene_id "lnc-bbug07075"	transcript_id "lnc-bbut00016879"	exon_number "4"
15	lncRNA	exon	62578211	62578405	.	-	.	gene_id "lnc-bbug07076"	transcript_id "lnc-bbut00016880"	exon_number "1"
15	lncRNA	exon	62578771	62578849	.	-	.	gene_id "lnc-bbug07076"	transcript_id "lnc-bbut00016880"	exon_number "2"
15	lncRNA	exon	62580788	62580880	.	-	.	gene_id "lnc-bbug07076"	transcript_id "lnc-bbut00016880"	exon_number "3"
15	lncRNA	exon	62585144	62585342	.	-	.	gene_id "lnc-bbug07076"	transcript_id "lnc-bbut00016880"	exon_number "4"
15	lncRNA	exon	62586609	62586662	.	-	.	gene_id "lnc-bbug07076"	transcript_id "lnc-bbut00016880"	exon_number "5"
15	lncRNA	exon	62594606	62595726	.	-	.	gene_id "lnc-bbug07076"	transcript_id "lnc-bbut00016880"	exon_number "6"
15	lncRNA	exon	62578211	62578405	.	-	.	gene_id "lnc-bbug07076"	transcript_id "lnc-bbut00016883"	exon_number "1"
15	lncRNA	exon	62578771	62578849	.	-	.	gene_id "lnc-bbug07076"	transcript_id "lnc-bbut00016883"	exon_number "2"
15	lncRNA	exon	62580788	62580880	.	-	.	gene_id "lnc-bbug07076"	transcript_id "lnc-bbut00016883"	exon_number "3"
15	lncRNA	exon	62585144	62585342	.	-	.	gene_id "lnc-bbug07076"	transcript_id "lnc-bbut00016883"	exon_number "4"
15	lncRNA	exon	62586609	62587245	.	-	.	gene_id "lnc-bbug07076"	transcript_id "lnc-bbut00016883"	exon_number "5"
15	lncRNA	exon	62595733	62596141	.	-	.	gene_id "lnc-bbug07076"	transcript_id "lnc-bbut00016883"	exon_number "6"
15	lncRNA	exon	62660438	62661054	.	-	.	gene_id "lnc-bbug07077"	transcript_id "lnc-bbut00016884"	exon_number "1"
15	lncRNA	exon	62664736	62665118	.	-	.	gene_id "lnc-bbug07077"	transcript_id "lnc-bbut00016884"	exon_number "2"
15	lncRNA	exon	62690672	62691422	.	-	.	gene_id "lnc-bbug07077"	transcript_id "lnc-bbut00016884"	exon_number "3"
15	lncRNA	exon	62781580	62784842	.	-	.	gene_id "lnc-bbug07078"	transcript_id "lnc-bbut00016886"	exon_number "1"
15	lncRNA	exon	62786028	62786153	.	-	.	gene_id "lnc-bbug07078"	transcript_id "lnc-bbut00016886"	exon_number "2"
15	lncRNA	exon	62790433	62790581	.	-	.	gene_id "lnc-bbug07078"	transcript_id "lnc-bbut00016886"	exon_number "3"
15	lncRNA	exon	62803682	62803703	.	-	.	gene_id "lnc-bbug07078"	transcript_id "lnc-bbut00016886"	exon_number "4"
15	lncRNA	exon	62824443	62824949	.	-	.	gene_id "lnc-bbug07079"	transcript_id "lnc-bbut00016888"	exon_number "1"
15	lncRNA	exon	62828104	62828726	.	-	.	gene_id "lnc-bbug07079"	transcript_id "lnc-bbut00016888"	exon_number "2"
15	lncRNA	exon	62832618	62832696	.	-	.	gene_id "lnc-bbug07079"	transcript_id "lnc-bbut00016888"	exon_number "3"
15	lncRNA	exon	63871111	63871488	.	-	.	gene_id "lnc-bbug07084"	transcript_id "lnc-bbut00016895"	exon_number "1"
15	lncRNA	exon	63871868	63872024	.	-	.	gene_id "lnc-bbug07084"	transcript_id "lnc-bbut00016895"	exon_number "2"
15	lncRNA	exon	63877448	63877556	.	-	.	gene_id "lnc-bbug07084"	transcript_id "lnc-bbut00016895"	exon_number "3"
15	lncRNA	exon	63958573	63958953	.	-	.	gene_id "lnc-bbug07085"	transcript_id "lnc-bbut00016898"	exon_number "1"
15	lncRNA	exon	63961373	63961425	.	-	.	gene_id "lnc-bbug07085"	transcript_id "lnc-bbut00016898"	exon_number "2"
15	lncRNA	exon	63997716	63997784	.	-	.	gene_id "lnc-bbug07085"	transcript_id "lnc-bbut00016898"	exon_number "3"
15	lncRNA	exon	63997913	63998099	.	-	.	gene_id "lnc-bbug07085"	transcript_id "lnc-bbut00016898"	exon_number "4"
15	lncRNA	exon	64021173	64021268	.	-	.	gene_id "lnc-bbug07085"	transcript_id "lnc-bbut00016898"	exon_number "5"
15	lncRNA	exon	64037686	64037929	.	-	.	gene_id "lnc-bbug07085"	transcript_id "lnc-bbut00016898"	exon_number "6"
15	lncRNA	exon	63958573	63960142	.	-	.	gene_id "lnc-bbug07085"	transcript_id "lnc-bbut00016900"	exon_number "1"
15	lncRNA	exon	63961373	63961425	.	-	.	gene_id "lnc-bbug07085"	transcript_id "lnc-bbut00016900"	exon_number "2"
15	lncRNA	exon	63997716	63997784	.	-	.	gene_id "lnc-bbug07085"	transcript_id "lnc-bbut00016900"	exon_number "3"
15	lncRNA	exon	63997913	63998099	.	-	.	gene_id "lnc-bbug07085"	transcript_id "lnc-bbut00016900"	exon_number "4"
15	lncRNA	exon	64021173	64021268	.	-	.	gene_id "lnc-bbug07085"	transcript_id "lnc-bbut00016900"	exon_number "5"
15	lncRNA	exon	64037686	64037929	.	-	.	gene_id "lnc-bbug07085"	transcript_id "lnc-bbut00016900"	exon_number "6"
15	lncRNA	exon	63958573	63960142	.	-	.	gene_id "lnc-bbug07085"	transcript_id "lnc-bbut00016901"	exon_number "1"
15	lncRNA	exon	63961373	63961425	.	-	.	gene_id "lnc-bbug07085"	transcript_id "lnc-bbut00016901"	exon_number "2"
15	lncRNA	exon	63994767	63994843	.	-	.	gene_id "lnc-bbug07085"	transcript_id "lnc-bbut00016901"	exon_number "3"
15	lncRNA	exon	63997716	63997784	.	-	.	gene_id "lnc-bbug07085"	transcript_id "lnc-bbut00016901"	exon_number "4"
15	lncRNA	exon	63997913	63998099	.	-	.	gene_id "lnc-bbug07085"	transcript_id "lnc-bbut00016901"	exon_number "5"
15	lncRNA	exon	64021173	64021268	.	-	.	gene_id "lnc-bbug07085"	transcript_id "lnc-bbut00016901"	exon_number "6"
15	lncRNA	exon	64037686	64037929	.	-	.	gene_id "lnc-bbug07085"	transcript_id "lnc-bbut00016901"	exon_number "7"
15	lncRNA	exon	63958573	63960142	.	-	.	gene_id "lnc-bbug07085"	transcript_id "lnc-bbut00016899"	exon_number "1"
15	lncRNA	exon	63961373	63961425	.	-	.	gene_id "lnc-bbug07085"	transcript_id "lnc-bbut00016899"	exon_number "2"
15	lncRNA	exon	63994767	63994843	.	-	.	gene_id "lnc-bbug07085"	transcript_id "lnc-bbut00016899"	exon_number "3"
15	lncRNA	exon	63997913	63998099	.	-	.	gene_id "lnc-bbug07085"	transcript_id "lnc-bbut00016899"	exon_number "4"
15	lncRNA	exon	64021173	64021268	.	-	.	gene_id "lnc-bbug07085"	transcript_id "lnc-bbut00016899"	exon_number "5"

15	lncRNA	exon	64037686	64037929	.	-	.	gene_id "lnc-bbug07085"	transcript_id "lnc-bbut00016899"	exon_number "6"
15	lncRNA	exon	63958573	63960142	.	-	.	gene_id "lnc-bbug07085"	transcript_id "lnc-bbut00016897"	exon_number "1"
15	lncRNA	exon	63961373	63961425	.	-	.	gene_id "lnc-bbug07085"	transcript_id "lnc-bbut00016897"	exon_number "2"
15	lncRNA	exon	63997913	63998099	.	-	.	gene_id "lnc-bbug07085"	transcript_id "lnc-bbut00016897"	exon_number "3"
15	lncRNA	exon	64021173	64021268	.	-	.	gene_id "lnc-bbug07085"	transcript_id "lnc-bbut00016897"	exon_number "4"
15	lncRNA	exon	64037686	64037929	.	-	.	gene_id "lnc-bbug07085"	transcript_id "lnc-bbut00016897"	exon_number "5"
15	lncRNA	exon	63958573	63958953	.	-	.	gene_id "lnc-bbug07085"	transcript_id "lnc-bbut00016896"	exon_number "1"
15	lncRNA	exon	63961373	63961425	.	-	.	gene_id "lnc-bbug07085"	transcript_id "lnc-bbut00016896"	exon_number "2"
15	lncRNA	exon	63997913	63998099	.	-	.	gene_id "lnc-bbug07085"	transcript_id "lnc-bbut00016896"	exon_number "3"
15	lncRNA	exon	64021173	64021268	.	-	.	gene_id "lnc-bbug07085"	transcript_id "lnc-bbut00016896"	exon_number "4"
15	lncRNA	exon	64037686	64037929	.	-	.	gene_id "lnc-bbug07085"	transcript_id "lnc-bbut00016896"	exon_number "5"
15	lncRNA	exon	64475182	64475590	.	-	.	gene_id "lnc-bbug07088"	transcript_id "lnc-bbut00016905"	exon_number "1"
15	lncRNA	exon	65527314	65528100	.	-	.	gene_id "lnc-bbug07090"	transcript_id "lnc-bbut00016907"	exon_number "1"
15	lncRNA	exon	65529498	65529604	.	-	.	gene_id "lnc-bbug07090"	transcript_id "lnc-bbut00016907"	exon_number "2"
15	lncRNA	exon	65714382	65716692	.	-	.	gene_id "lnc-bbug07091"	transcript_id "lnc-bbut00016908"	exon_number "1"
15	lncRNA	exon	65720535	65720645	.	-	.	gene_id "lnc-bbug07091"	transcript_id "lnc-bbut00016908"	exon_number "2"
15	lncRNA	exon	65722084	65722136	.	-	.	gene_id "lnc-bbug07091"	transcript_id "lnc-bbut00016908"	exon_number "3"
15	lncRNA	exon	65723628	65723723	.	-	.	gene_id "lnc-bbug07091"	transcript_id "lnc-bbut00016908"	exon_number "4"
15	lncRNA	exon	65724303	65724329	.	-	.	gene_id "lnc-bbug07091"	transcript_id "lnc-bbut00016908"	exon_number "5"
15	lncRNA	exon	65725341	65725405	.	-	.	gene_id "lnc-bbug07091"	transcript_id "lnc-bbut00016908"	exon_number "6"
15	lncRNA	exon	65729309	65729420	.	-	.	gene_id "lnc-bbug07091"	transcript_id "lnc-bbut00016908"	exon_number "7"
15	lncRNA	exon	65738705	65738868	.	-	.	gene_id "lnc-bbug07091"	transcript_id "lnc-bbut00016908"	exon_number "8"
15	lncRNA	exon	65775482	65775765	.	-	.	gene_id "lnc-bbug07092"	transcript_id "lnc-bbut00016910"	exon_number "1"
15	lncRNA	exon	65820952	65821059	.	-	.	gene_id "lnc-bbug07092"	transcript_id "lnc-bbut00016910"	exon_number "2"
15	lncRNA	exon	65825192	65825333	.	-	.	gene_id "lnc-bbug07092"	transcript_id "lnc-bbut00016910"	exon_number "3"
15	lncRNA	exon	66083095	66083567	.	-	.	gene_id "lnc-bbug07097"	transcript_id "lnc-bbut00016922"	exon_number "1"
15	lncRNA	exon	66204000	66205086	.	-	.	gene_id "lnc-bbug07099"	transcript_id "lnc-bbut00016924"	exon_number "1"
15	lncRNA	exon	66538179	66538682	.	-	.	gene_id "lnc-bbug07102"	transcript_id "lnc-bbut00016930"	exon_number "1"
15	lncRNA	exon	66540339	66540466	.	-	.	gene_id "lnc-bbug07102"	transcript_id "lnc-bbut00016930"	exon_number "2"
15	lncRNA	exon	66544170	66544239	.	-	.	gene_id "lnc-bbug07102"	transcript_id "lnc-bbut00016930"	exon_number "3"
15	lncRNA	exon	66557374	66557676	.	-	.	gene_id "lnc-bbug07102"	transcript_id "lnc-bbut00016930"	exon_number "4"
15	lncRNA	exon	66846987	66847246	.	-	.	gene_id "lnc-bbug07104"	transcript_id "lnc-bbut00016934"	exon_number "1"
15	lncRNA	exon	66847363	66847419	.	-	.	gene_id "lnc-bbug07104"	transcript_id "lnc-bbut00016934"	exon_number "2"
15	lncRNA	exon	66847788	66847895	.	-	.	gene_id "lnc-bbug07104"	transcript_id "lnc-bbut00016934"	exon_number "3"
15	lncRNA	exon	67068555	67069027	.	-	.	gene_id "lnc-bbug07108"	transcript_id "lnc-bbut00016947"	exon_number "1"
15	lncRNA	exon	67070232	67070370	.	-	.	gene_id "lnc-bbug07108"	transcript_id "lnc-bbut00016947"	exon_number "2"
15	lncRNA	exon	67071112	67071248	.	-	.	gene_id "lnc-bbug07108"	transcript_id "lnc-bbut00016947"	exon_number "3"
15	lncRNA	exon	67071346	67071418	.	-	.	gene_id "lnc-bbug07108"	transcript_id "lnc-bbut00016947"	exon_number "4"
15	lncRNA	exon	67073152	67073645	.	-	.	gene_id "lnc-bbug07108"	transcript_id "lnc-bbut00016947"	exon_number "5"
15	lncRNA	exon	67068555	67069027	.	-	.	gene_id "lnc-bbug07108"	transcript_id "lnc-bbut00016946"	exon_number "1"
15	lncRNA	exon	67070232	67070356	.	-	.	gene_id "lnc-bbug07108"	transcript_id "lnc-bbut00016946"	exon_number "2"
15	lncRNA	exon	67071112	67071248	.	-	.	gene_id "lnc-bbug07108"	transcript_id "lnc-bbut00016946"	exon_number "3"
15	lncRNA	exon	67071346	67071418	.	-	.	gene_id "lnc-bbug07108"	transcript_id "lnc-bbut00016946"	exon_number "4"
15	lncRNA	exon	67073152	67073645	.	-	.	gene_id "lnc-bbug07108"	transcript_id "lnc-bbut00016946"	exon_number "5"
15	lncRNA	exon	67185457	67186090	.	-	.	gene_id "lnc-bbug07109"	transcript_id "lnc-bbut00016948"	exon_number "1"
15	lncRNA	exon	67188572	67188791	.	-	.	gene_id "lnc-bbug07109"	transcript_id "lnc-bbut00016948"	exon_number "2"
15	lncRNA	exon	67726698	67727666	.	-	.	gene_id "lnc-bbug07111"	transcript_id "lnc-bbut00016953"	exon_number "1"
15	lncRNA	exon	67731274	67731482	.	-	.	gene_id "lnc-bbug07111"	transcript_id "lnc-bbut00016953"	exon_number "2"
15	lncRNA	exon	67731578	67733317	.	-	.	gene_id "lnc-bbug07111"	transcript_id "lnc-bbut00016953"	exon_number "3"
15	lncRNA	exon	67726698	67727666	.	-	.	gene_id "lnc-bbug07111"	transcript_id "lnc-bbut00016954"	exon_number "1"
15	lncRNA	exon	67731274	67731482	.	-	.	gene_id "lnc-bbug07111"	transcript_id "lnc-bbut00016954"	exon_number "2"
15	lncRNA	exon	67733960	67735493	.	-	.	gene_id "lnc-bbug07111"	transcript_id "lnc-bbut00016954"	exon_number "3"

15	lncRNA	exon	67739075	67739146	.	-	.	gene_id "lnc-bbug07112"	transcript_id "lnc-bbut00016955"	exon_number "1"
15	lncRNA	exon	67751925	67756400	.	-	.	gene_id "lnc-bbug07112"	transcript_id "lnc-bbut00016955"	exon_number "2"
15	lncRNA	exon	67829118	67833355	.	-	.	gene_id "lnc-bbug07113"	transcript_id "lnc-bbut00016956"	exon_number "1"
15	lncRNA	exon	67846972	67847308	.	-	.	gene_id "lnc-bbug07113"	transcript_id "lnc-bbut00016956"	exon_number "2"
15	lncRNA	exon	67847537	67848105	.	-	.	gene_id "lnc-bbug07113"	transcript_id "lnc-bbut00016956"	exon_number "3"
15	lncRNA	exon	67841860	67844901	.	-	.	gene_id "lnc-bbug07113"	transcript_id "lnc-bbut00016957"	exon_number "1"
15	lncRNA	exon	67846972	67847308	.	-	.	gene_id "lnc-bbug07113"	transcript_id "lnc-bbut00016957"	exon_number "2"
15	lncRNA	exon	67847537	67847950	.	-	.	gene_id "lnc-bbug07113"	transcript_id "lnc-bbut00016957"	exon_number "3"
15	lncRNA	exon	67841860	67844182	.	-	.	gene_id "lnc-bbug07113"	transcript_id "lnc-bbut00016958"	exon_number "1"
15	lncRNA	exon	67847537	67847970	.	-	.	gene_id "lnc-bbug07113"	transcript_id "lnc-bbut00016958"	exon_number "2"
15	lncRNA	exon	67841860	67844182	.	-	.	gene_id "lnc-bbug07113"	transcript_id "lnc-bbut00016959"	exon_number "1"
15	lncRNA	exon	67846972	67847308	.	-	.	gene_id "lnc-bbug07113"	transcript_id "lnc-bbut00016959"	exon_number "2"
15	lncRNA	exon	67847537	67848106	.	-	.	gene_id "lnc-bbug07113"	transcript_id "lnc-bbut00016959"	exon_number "3"
15	lncRNA	exon	67841860	67844182	.	-	.	gene_id "lnc-bbug07113"	transcript_id "lnc-bbut00016960"	exon_number "1"
15	lncRNA	exon	67849103	67849252	.	-	.	gene_id "lnc-bbug07113"	transcript_id "lnc-bbut00016960"	exon_number "2"
15	lncRNA	exon	67841860	67844182	.	-	.	gene_id "lnc-bbug07113"	transcript_id "lnc-bbut00016961"	exon_number "1"
15	lncRNA	exon	67846972	67847308	.	-	.	gene_id "lnc-bbug07113"	transcript_id "lnc-bbut00016961"	exon_number "2"
15	lncRNA	exon	67849103	67849427	.	-	.	gene_id "lnc-bbug07113"	transcript_id "lnc-bbut00016961"	exon_number "3"
15	lncRNA	exon	67846330	67847308	.	-	.	gene_id "lnc-bbug07113"	transcript_id "lnc-bbut00016962"	exon_number "1"
15	lncRNA	exon	67847537	67847950	.	-	.	gene_id "lnc-bbug07113"	transcript_id "lnc-bbut00016962"	exon_number "2"
15	lncRNA	exon	68427363	68428354	.	-	.	gene_id "lnc-bbug07116"	transcript_id "lnc-bbut00016965"	exon_number "1"
15	lncRNA	exon	68431476	68431586	.	-	.	gene_id "lnc-bbug07116"	transcript_id "lnc-bbut00016965"	exon_number "2"
15	lncRNA	exon	68433364	68433458	.	-	.	gene_id "lnc-bbug07116"	transcript_id "lnc-bbut00016965"	exon_number "3"
15	lncRNA	exon	68443946	68444131	.	-	.	gene_id "lnc-bbug07116"	transcript_id "lnc-bbut00016965"	exon_number "4"
15	lncRNA	exon	68430321	68431586	.	-	.	gene_id "lnc-bbug07116"	transcript_id "lnc-bbut00016966"	exon_number "1"
15	lncRNA	exon	68433364	68433458	.	-	.	gene_id "lnc-bbug07116"	transcript_id "lnc-bbut00016966"	exon_number "2"
15	lncRNA	exon	68443946	68444131	.	-	.	gene_id "lnc-bbug07116"	transcript_id "lnc-bbut00016966"	exon_number "3"
15	lncRNA	exon	68614852	68615370	.	-	.	gene_id "lnc-bbug07119"	transcript_id "lnc-bbut00016971"	exon_number "1"
15	lncRNA	exon	68617393	68617467	.	-	.	gene_id "lnc-bbug07119"	transcript_id "lnc-bbut00016971"	exon_number "2"
15	lncRNA	exon	68624043	68624282	.	-	.	gene_id "lnc-bbug07119"	transcript_id "lnc-bbut00016971"	exon_number "3"
15	lncRNA	exon	68711622	68712310	.	-	.	gene_id "lnc-bbug07121"	transcript_id "lnc-bbut00016973"	exon_number "1"
15	lncRNA	exon	68712694	68712819	.	-	.	gene_id "lnc-bbug07121"	transcript_id "lnc-bbut00016973"	exon_number "2"
15	lncRNA	exon	68726745	68726940	.	-	.	gene_id "lnc-bbug07121"	transcript_id "lnc-bbut00016973"	exon_number "3"
15	lncRNA	exon	69598089	69598657	.	-	.	gene_id "lnc-bbug07123"	transcript_id "lnc-bbut00016976"	exon_number "1"
15	lncRNA	exon	69598753	69598859	.	-	.	gene_id "lnc-bbug07123"	transcript_id "lnc-bbut00016976"	exon_number "2"
15	lncRNA	exon	69599491	69602280	.	-	.	gene_id "lnc-bbug07123"	transcript_id "lnc-bbut00016976"	exon_number "3"
15	lncRNA	exon	69603648	69603751	.	-	.	gene_id "lnc-bbug07123"	transcript_id "lnc-bbut00016976"	exon_number "4"
15	lncRNA	exon	69633233	69633272	.	-	.	gene_id "lnc-bbug07123"	transcript_id "lnc-bbut00016976"	exon_number "5"
15	lncRNA	exon	69636193	69637312	.	-	.	gene_id "lnc-bbug07123"	transcript_id "lnc-bbut00016976"	exon_number "6"
15	lncRNA	exon	69598089	69598657	.	-	.	gene_id "lnc-bbug07123"	transcript_id "lnc-bbut00016975"	exon_number "1"
15	lncRNA	exon	69598753	69602280	.	-	.	gene_id "lnc-bbug07123"	transcript_id "lnc-bbut00016975"	exon_number "2"
15	lncRNA	exon	69603648	69603751	.	-	.	gene_id "lnc-bbug07123"	transcript_id "lnc-bbut00016975"	exon_number "3"
15	lncRNA	exon	69633233	69633272	.	-	.	gene_id "lnc-bbug07123"	transcript_id "lnc-bbut00016975"	exon_number "4"
15	lncRNA	exon	69636193	69637312	.	-	.	gene_id "lnc-bbug07123"	transcript_id "lnc-bbut00016975"	exon_number "5"
15	lncRNA	exon	69622278	69622319	.	-	.	gene_id "lnc-bbug07123"	transcript_id "lnc-bbut00016977"	exon_number "1"
15	lncRNA	exon	69633069	69633272	.	-	.	gene_id "lnc-bbug07123"	transcript_id "lnc-bbut00016977"	exon_number "2"
15	lncRNA	exon	69636193	69637333	.	-	.	gene_id "lnc-bbug07123"	transcript_id "lnc-bbut00016977"	exon_number "3"
15	lncRNA	exon	70191597	70191791	.	-	.	gene_id "lnc-bbug07125"	transcript_id "lnc-bbut00016979"	exon_number "1"
15	lncRNA	exon	70208522	70208584	.	-	.	gene_id "lnc-bbug07125"	transcript_id "lnc-bbut00016979"	exon_number "2"
15	lncRNA	exon	70224043	70224192	.	-	.	gene_id "lnc-bbug07125"	transcript_id "lnc-bbut00016979"	exon_number "3"
15	lncRNA	exon	70198404	70198958	.	-	.	gene_id "lnc-bbug07125"	transcript_id "lnc-bbut00016980"	exon_number "1"
15	lncRNA	exon	70208522	70208584	.	-	.	gene_id "lnc-bbug07125"	transcript_id "lnc-bbut00016980"	exon_number "2"

15	lncRNA	exon	70224043	70224192	.	-	.	gene_id "lnc-bbug07125"	transcript_id "lnc-bbut00016980"	exon_number "3"
15	lncRNA	exon	70202528	70202569	.	-	.	gene_id "lnc-bbug07125"	transcript_id "lnc-bbut00016981"	exon_number "1"
15	lncRNA	exon	70208522	70208584	.	-	.	gene_id "lnc-bbug07125"	transcript_id "lnc-bbut00016981"	exon_number "2"
15	lncRNA	exon	70224043	70224192	.	-	.	gene_id "lnc-bbug07125"	transcript_id "lnc-bbut00016981"	exon_number "3"
15	lncRNA	exon	71129857	71130012	.	-	.	gene_id "lnc-bbug07126"	transcript_id "lnc-bbut00016982"	exon_number "1"
15	lncRNA	exon	71141167	71141353	.	-	.	gene_id "lnc-bbug07126"	transcript_id "lnc-bbut00016982"	exon_number "2"
15	lncRNA	exon	73024559	73024878	.	-	.	gene_id "lnc-bbug07131"	transcript_id "lnc-bbut00017003"	exon_number "1"
15	lncRNA	exon	73025326	73025403	.	-	.	gene_id "lnc-bbug07131"	transcript_id "lnc-bbut00017003"	exon_number "2"
15	lncRNA	exon	73028621	73028783	.	-	.	gene_id "lnc-bbug07131"	transcript_id "lnc-bbut00017003"	exon_number "3"
15	lncRNA	exon	73041656	73041836	.	-	.	gene_id "lnc-bbug07131"	transcript_id "lnc-bbut00017003"	exon_number "4"
15	lncRNA	exon	73024559	73024878	.	-	.	gene_id "lnc-bbug07131"	transcript_id "lnc-bbut00017004"	exon_number "1"
15	lncRNA	exon	73025326	73025403	.	-	.	gene_id "lnc-bbug07131"	transcript_id "lnc-bbut00017004"	exon_number "2"
15	lncRNA	exon	73028621	73028783	.	-	.	gene_id "lnc-bbug07131"	transcript_id "lnc-bbut00017004"	exon_number "3"
15	lncRNA	exon	73041662	73041837	.	-	.	gene_id "lnc-bbug07131"	transcript_id "lnc-bbut00017004"	exon_number "4"
15	lncRNA	exon	73024559	73024878	.	-	.	gene_id "lnc-bbug07131"	transcript_id "lnc-bbut00017005"	exon_number "1"
15	lncRNA	exon	73025326	73025403	.	-	.	gene_id "lnc-bbug07131"	transcript_id "lnc-bbut00017005"	exon_number "2"
15	lncRNA	exon	73041323	73041512	.	-	.	gene_id "lnc-bbug07131"	transcript_id "lnc-bbut00017005"	exon_number "3"
15	lncRNA	exon	73041662	73041841	.	-	.	gene_id "lnc-bbug07131"	transcript_id "lnc-bbut00017005"	exon_number "4"
15	lncRNA	exon	73024559	73024878	.	-	.	gene_id "lnc-bbug07131"	transcript_id "lnc-bbut00017006"	exon_number "1"
15	lncRNA	exon	73025326	73025403	.	-	.	gene_id "lnc-bbug07131"	transcript_id "lnc-bbut00017006"	exon_number "2"
15	lncRNA	exon	73028621	73028783	.	-	.	gene_id "lnc-bbug07131"	transcript_id "lnc-bbut00017006"	exon_number "3"
15	lncRNA	exon	73041323	73041512	.	-	.	gene_id "lnc-bbug07131"	transcript_id "lnc-bbut00017006"	exon_number "4"
15	lncRNA	exon	73041662	73041844	.	-	.	gene_id "lnc-bbug07131"	transcript_id "lnc-bbut00017006"	exon_number "5"
15	lncRNA	exon	73024559	73024878	.	-	.	gene_id "lnc-bbug07131"	transcript_id "lnc-bbut00017007"	exon_number "1"
15	lncRNA	exon	73025326	73025403	.	-	.	gene_id "lnc-bbug07131"	transcript_id "lnc-bbut00017007"	exon_number "2"
15	lncRNA	exon	73028621	73028783	.	-	.	gene_id "lnc-bbug07131"	transcript_id "lnc-bbut00017007"	exon_number "3"
15	lncRNA	exon	73041323	73041512	.	-	.	gene_id "lnc-bbug07131"	transcript_id "lnc-bbut00017007"	exon_number "4"
15	lncRNA	exon	73041656	73041847	.	-	.	gene_id "lnc-bbug07131"	transcript_id "lnc-bbut00017007"	exon_number "5"
15	lncRNA	exon	74065611	74065716	.	-	.	gene_id "lnc-bbug07138"	transcript_id "lnc-bbut00017041"	exon_number "1"
15	lncRNA	exon	74066803	74066955	.	-	.	gene_id "lnc-bbug07138"	transcript_id "lnc-bbut00017041"	exon_number "2"
15	lncRNA	exon	74070152	74070910	.	-	.	gene_id "lnc-bbug07138"	transcript_id "lnc-bbut00017041"	exon_number "3"
15	lncRNA	exon	74075092	74075935	.	-	.	gene_id "lnc-bbug07139"	transcript_id "lnc-bbut00017042"	exon_number "1"
15	lncRNA	exon	74079556	74079647	.	-	.	gene_id "lnc-bbug07139"	transcript_id "lnc-bbut00017042"	exon_number "2"
15	lncRNA	exon	74080260	74080570	.	-	.	gene_id "lnc-bbug07139"	transcript_id "lnc-bbut00017042"	exon_number "3"
15	lncRNA	exon	74215231	74215434	.	-	.	gene_id "lnc-bbug07141"	transcript_id "lnc-bbut00017046"	exon_number "1"
15	lncRNA	exon	74217412	74217864	.	-	.	gene_id "lnc-bbug07141"	transcript_id "lnc-bbut00017046"	exon_number "2"
15	lncRNA	exon	74288286	74288459	.	-	.	gene_id "lnc-bbug07142"	transcript_id "lnc-bbut00017047"	exon_number "1"
15	lncRNA	exon	74293855	74294734	.	-	.	gene_id "lnc-bbug07142"	transcript_id "lnc-bbut00017047"	exon_number "2"
15	lncRNA	exon	74854265	74854514	.	-	.	gene_id "lnc-bbug07144"	transcript_id "lnc-bbut00017053"	exon_number "1"
15	lncRNA	exon	74882770	74882853	.	-	.	gene_id "lnc-bbug07144"	transcript_id "lnc-bbut00017053"	exon_number "2"
15	lncRNA	exon	74882978	74883495	.	-	.	gene_id "lnc-bbug07144"	transcript_id "lnc-bbut00017053"	exon_number "3"
15	lncRNA	exon	75263734	75272685	.	-	.	gene_id "lnc-bbug07146"	transcript_id "lnc-bbut00017056"	exon_number "1"
15	lncRNA	exon	75282760	75294760	.	-	.	gene_id "lnc-bbug07146"	transcript_id "lnc-bbut00017056"	exon_number "2"
15	lncRNA	exon	75294966	75295186	.	-	.	gene_id "lnc-bbug07147"	transcript_id "lnc-bbut00017057"	exon_number "1"
15	lncRNA	exon	75304270	75304654	.	-	.	gene_id "lnc-bbug07147"	transcript_id "lnc-bbut00017057"	exon_number "2"
15	lncRNA	exon	75582054	75582412	.	-	.	gene_id "lnc-bbug07148"	transcript_id "lnc-bbut00017058"	exon_number "1"
15	lncRNA	exon	75584248	75584339	.	-	.	gene_id "lnc-bbug07148"	transcript_id "lnc-bbut00017058"	exon_number "2"
15	lncRNA	exon	75584975	75585052	.	-	.	gene_id "lnc-bbug07148"	transcript_id "lnc-bbut00017058"	exon_number "3"
15	lncRNA	exon	75635749	75636014	.	-	.	gene_id "lnc-bbug07148"	transcript_id "lnc-bbut00017058"	exon_number "4"
15	lncRNA	exon	76480081	76480241	.	-	.	gene_id "lnc-bbug07150"	transcript_id "lnc-bbut00017060"	exon_number "1"
15	lncRNA	exon	76480601	76480729	.	-	.	gene_id "lnc-bbug07150"	transcript_id "lnc-bbut00017060"	exon_number "2"
15	lncRNA	exon	76510168	76510256	.	-	.	gene_id "lnc-bbug07150"	transcript_id "lnc-bbut00017060"	exon_number "3"

15	lncRNA	exon	79102454	79102870	.	-	.	gene_id "lnc-bbug07155"	transcript_id "lnc-bbut00017070"	exon_number "1"
15	lncRNA	exon	79107257	79108521	.	-	.	gene_id "lnc-bbug07155"	transcript_id "lnc-bbut00017070"	exon_number "2"
15	lncRNA	exon	79102454	79102870	.	-	.	gene_id "lnc-bbug07155"	transcript_id "lnc-bbut00017071"	exon_number "1"
15	lncRNA	exon	79107257	79108013	.	-	.	gene_id "lnc-bbug07155"	transcript_id "lnc-bbut00017071"	exon_number "2"
15	lncRNA	exon	79115566	79116082	.	-	.	gene_id "lnc-bbug07155"	transcript_id "lnc-bbut00017071"	exon_number "3"
15	lncRNA	exon	79801886	79802074	.	-	.	gene_id "lnc-bbug07161"	transcript_id "lnc-bbut00017108"	exon_number "1"
15	lncRNA	exon	79803220	79803368	.	-	.	gene_id "lnc-bbug07161"	transcript_id "lnc-bbut00017108"	exon_number "2"
15	lncRNA	exon	79805060	79805494	.	-	.	gene_id "lnc-bbug07161"	transcript_id "lnc-bbut00017108"	exon_number "3"
15	lncRNA	exon	79889747	79890589	.	-	.	gene_id "lnc-bbug07162"	transcript_id "lnc-bbut00017109"	exon_number "1"
15	lncRNA	exon	79890879	79891058	.	-	.	gene_id "lnc-bbug07162"	transcript_id "lnc-bbut00017109"	exon_number "2"
15	lncRNA	exon	79891293	79892420	.	-	.	gene_id "lnc-bbug07162"	transcript_id "lnc-bbut00017109"	exon_number "3"
15	lncRNA	exon	79893242	79893350	.	-	.	gene_id "lnc-bbug07162"	transcript_id "lnc-bbut00017109"	exon_number "4"
15	lncRNA	exon	79890601	79891058	.	-	.	gene_id "lnc-bbug07162"	transcript_id "lnc-bbut00017110"	exon_number "1"
15	lncRNA	exon	79891293	79892420	.	-	.	gene_id "lnc-bbug07162"	transcript_id "lnc-bbut00017110"	exon_number "2"
15	lncRNA	exon	79893242	79893350	.	-	.	gene_id "lnc-bbug07162"	transcript_id "lnc-bbut00017110"	exon_number "3"
15	lncRNA	exon	80596855	80600755	.	-	.	gene_id "lnc-bbug07169"	transcript_id "lnc-bbut00017143"	exon_number "1"
15	lncRNA	exon	80600962	80601063	.	-	.	gene_id "lnc-bbug07169"	transcript_id "lnc-bbut00017143"	exon_number "2"
15	lncRNA	exon	80601255	80601456	.	-	.	gene_id "lnc-bbug07169"	transcript_id "lnc-bbut00017143"	exon_number "3"
15	lncRNA	exon	80602401	80602532	.	-	.	gene_id "lnc-bbug07169"	transcript_id "lnc-bbut00017143"	exon_number "4"
15	lncRNA	exon	80649509	80651863	.	-	.	gene_id "lnc-bbug07171"	transcript_id "lnc-bbut00017146"	exon_number "1"
15	lncRNA	exon	80653067	80653171	.	-	.	gene_id "lnc-bbug07171"	transcript_id "lnc-bbut00017146"	exon_number "2"
15	lncRNA	exon	80654085	80654181	.	-	.	gene_id "lnc-bbug07171"	transcript_id "lnc-bbut00017146"	exon_number "3"
15	lncRNA	exon	80658484	80659924	.	-	.	gene_id "lnc-bbug07171"	transcript_id "lnc-bbut00017146"	exon_number "4"
15	lncRNA	exon	80660832	80661853	.	-	.	gene_id "lnc-bbug07172"	transcript_id "lnc-bbut00017147"	exon_number "1"
15	lncRNA	exon	80665601	80665720	.	-	.	gene_id "lnc-bbug07172"	transcript_id "lnc-bbut00017147"	exon_number "2"
15	lncRNA	exon	80666021	80666334	.	-	.	gene_id "lnc-bbug07172"	transcript_id "lnc-bbut00017147"	exon_number "3"
15	lncRNA	exon	80762328	80763405	.	-	.	gene_id "lnc-bbug07175"	transcript_id "lnc-bbut00017152"	exon_number "1"
15	lncRNA	exon	80763602	80764227	.	-	.	gene_id "lnc-bbug07175"	transcript_id "lnc-bbut00017152"	exon_number "2"
15	lncRNA	exon	80918926	80920665	.	-	.	gene_id "lnc-bbug07177"	transcript_id "lnc-bbut00017156"	exon_number "1"
15	lncRNA	exon	80920964	80921083	.	-	.	gene_id "lnc-bbug07177"	transcript_id "lnc-bbut00017156"	exon_number "2"
15	lncRNA	exon	80921800	80921927	.	-	.	gene_id "lnc-bbug07177"	transcript_id "lnc-bbut00017156"	exon_number "3"
15	lncRNA	exon	80922410	80922529	.	-	.	gene_id "lnc-bbug07177"	transcript_id "lnc-bbut00017156"	exon_number "4"
15	lncRNA	exon	80926313	80926347	.	-	.	gene_id "lnc-bbug07177"	transcript_id "lnc-bbut00017156"	exon_number "5"
15	lncRNA	exon	80926512	80926800	.	-	.	gene_id "lnc-bbug07177"	transcript_id "lnc-bbut00017156"	exon_number "6"
15	lncRNA	exon	80983627	80984983	.	-	.	gene_id "lnc-bbug07178"	transcript_id "lnc-bbut00017160"	exon_number "1"
15	lncRNA	exon	80985407	80986127	.	-	.	gene_id "lnc-bbug07178"	transcript_id "lnc-bbut00017160"	exon_number "2"
15	lncRNA	exon	80986347	80986494	.	-	.	gene_id "lnc-bbug07178"	transcript_id "lnc-bbut00017160"	exon_number "3"
15	lncRNA	exon	80983627	80984983	.	-	.	gene_id "lnc-bbug07178"	transcript_id "lnc-bbut00017159"	exon_number "1"
15	lncRNA	exon	80985407	80985534	.	-	.	gene_id "lnc-bbug07178"	transcript_id "lnc-bbut00017159"	exon_number "2"
15	lncRNA	exon	80985949	80986494	.	-	.	gene_id "lnc-bbug07178"	transcript_id "lnc-bbut00017159"	exon_number "3"
15	lncRNA	exon	81230886	81232012	.	-	.	gene_id "lnc-bbug07189"	transcript_id "lnc-bbut00017188"	exon_number "1"
15	lncRNA	exon	81232804	81233374	.	-	.	gene_id "lnc-bbug07189"	transcript_id "lnc-bbut00017188"	exon_number "2"
15	lncRNA	exon	81233504	81233833	.	-	.	gene_id "lnc-bbug07189"	transcript_id "lnc-bbut00017188"	exon_number "3"
15	lncRNA	exon	81533085	81534003	.	-	.	gene_id "lnc-bbug07198"	transcript_id "lnc-bbut00017242"	exon_number "1"
15	lncRNA	exon	81538270	81538410	.	-	.	gene_id "lnc-bbug07198"	transcript_id "lnc-bbut00017242"	exon_number "2"
15	lncRNA	exon	81539286	81539839	.	-	.	gene_id "lnc-bbug07199"	transcript_id "lnc-bbut00017243"	exon_number "1"
15	lncRNA	exon	81541076	81541495	.	-	.	gene_id "lnc-bbug07199"	transcript_id "lnc-bbut00017243"	exon_number "2"
15	lncRNA	exon	81568253	81568388	.	-	.	gene_id "lnc-bbug07201"	transcript_id "lnc-bbut00017245"	exon_number "1"
15	lncRNA	exon	81568482	81568597	.	-	.	gene_id "lnc-bbug07201"	transcript_id "lnc-bbut00017245"	exon_number "2"
15	lncRNA	exon	81569407	81569507	.	-	.	gene_id "lnc-bbug07201"	transcript_id "lnc-bbut00017245"	exon_number "3"
15	lncRNA	exon	81572357	81572748	.	-	.	gene_id "lnc-bbug07201"	transcript_id "lnc-bbut00017245"	exon_number "4"
15	lncRNA	exon	81572839	81572959	.	-	.	gene_id "lnc-bbug07201"	transcript_id "lnc-bbut00017245"	exon_number "5"

15	lncRNA	exon	81568951	81569081	.	-	.	gene_id "lnc-bbug07201"	transcript_id "lnc-bbut00017246"	exon_number "1"
15	lncRNA	exon	81569407	81569603	.	-	.	gene_id "lnc-bbug07201"	transcript_id "lnc-bbut00017246"	exon_number "2"
15	lncRNA	exon	81572357	81572748	.	-	.	gene_id "lnc-bbug07201"	transcript_id "lnc-bbut00017246"	exon_number "3"
15	lncRNA	exon	81572839	81572959	.	-	.	gene_id "lnc-bbug07201"	transcript_id "lnc-bbut00017246"	exon_number "4"
15	lncRNA	exon	81876092	81876665	.	-	.	gene_id "lnc-bbug07217"	transcript_id "lnc-bbut00017284"	exon_number "1"
15	lncRNA	exon	81876945	81876993	.	-	.	gene_id "lnc-bbug07217"	transcript_id "lnc-bbut00017284"	exon_number "2"
15	lncRNA	exon	81877524	81877728	.	-	.	gene_id "lnc-bbug07217"	transcript_id "lnc-bbut00017284"	exon_number "3"
15	lncRNA	exon	81942128	81942670	.	-	.	gene_id "lnc-bbug07219"	transcript_id "lnc-bbut00017286"	exon_number "1"
15	lncRNA	exon	81944251	81944380	.	-	.	gene_id "lnc-bbug07219"	transcript_id "lnc-bbut00017286"	exon_number "2"
16	lncRNA	exon	818674	818713	.	+	.	gene_id "lnc-bbug07227"	transcript_id "lnc-bbut00017306"	exon_number "1"
16	lncRNA	exon	822076	822750	.	+	.	gene_id "lnc-bbug07227"	transcript_id "lnc-bbut00017306"	exon_number "2"
16	lncRNA	exon	1168289	1170971	.	+	.	gene_id "lnc-bbug07235"	transcript_id "lnc-bbut00017315"	exon_number "1"
16	lncRNA	exon	1172107	1173716	.	+	.	gene_id "lnc-bbug07235"	transcript_id "lnc-bbut00017315"	exon_number "2"
16	lncRNA	exon	1243878	1244316	.	+	.	gene_id "lnc-bbug07237"	transcript_id "lnc-bbut00017317"	exon_number "1"
16	lncRNA	exon	1249052	1251150	.	+	.	gene_id "lnc-bbug07237"	transcript_id "lnc-bbut00017317"	exon_number "2"
16	lncRNA	exon	1463608	1464640	.	+	.	gene_id "lnc-bbug07244"	transcript_id "lnc-bbut00017324"	exon_number "1"
16	lncRNA	exon	1685947	1686146	.	+	.	gene_id "lnc-bbug07250"	transcript_id "lnc-bbut00017330"	exon_number "1"
16	lncRNA	exon	1690388	1690854	.	+	.	gene_id "lnc-bbug07250"	transcript_id "lnc-bbut00017330"	exon_number "2"
16	lncRNA	exon	2080507	2080838	.	+	.	gene_id "lnc-bbug07256"	transcript_id "lnc-bbut00017343"	exon_number "1"
16	lncRNA	exon	2109100	2109271	.	+	.	gene_id "lnc-bbug07256"	transcript_id "lnc-bbut00017343"	exon_number "2"
16	lncRNA	exon	2109527	2110194	.	+	.	gene_id "lnc-bbug07256"	transcript_id "lnc-bbut00017343"	exon_number "3"
16	lncRNA	exon	2992422	2992612	.	+	.	gene_id "lnc-bbug07288"	transcript_id "lnc-bbut00017376"	exon_number "1"
16	lncRNA	exon	2994863	2995187	.	+	.	gene_id "lnc-bbug07288"	transcript_id "lnc-bbut00017376"	exon_number "2"
16	lncRNA	exon	2995374	2995417	.	+	.	gene_id "lnc-bbug07288"	transcript_id "lnc-bbut00017376"	exon_number "3"
16	lncRNA	exon	2995757	2995865	.	+	.	gene_id "lnc-bbug07288"	transcript_id "lnc-bbut00017376"	exon_number "4"
16	lncRNA	exon	2996074	2997093	.	+	.	gene_id "lnc-bbug07288"	transcript_id "lnc-bbut00017376"	exon_number "5"
16	lncRNA	exon	3007164	3010384	.	+	.	gene_id "lnc-bbug07289"	transcript_id "lnc-bbut00017377"	exon_number "1"
16	lncRNA	exon	3012326	3012385	.	+	.	gene_id "lnc-bbug07289"	transcript_id "lnc-bbut00017377"	exon_number "2"
16	lncRNA	exon	3021688	3022098	.	+	.	gene_id "lnc-bbug07289"	transcript_id "lnc-bbut00017377"	exon_number "3"
16	lncRNA	exon	3101840	3101960	.	+	.	gene_id "lnc-bbug07291"	transcript_id "lnc-bbut00017379"	exon_number "1"
16	lncRNA	exon	3102444	3102560	.	+	.	gene_id "lnc-bbug07291"	transcript_id "lnc-bbut00017379"	exon_number "2"
16	lncRNA	exon	3104106	3104520	.	+	.	gene_id "lnc-bbug07291"	transcript_id "lnc-bbut00017379"	exon_number "3"
16	lncRNA	exon	3101992	3102319	.	+	.	gene_id "lnc-bbug07291"	transcript_id "lnc-bbut00017380"	exon_number "1"
16	lncRNA	exon	3102444	3102560	.	+	.	gene_id "lnc-bbug07291"	transcript_id "lnc-bbut00017380"	exon_number "2"
16	lncRNA	exon	3104106	3104520	.	+	.	gene_id "lnc-bbug07291"	transcript_id "lnc-bbut00017380"	exon_number "3"
16	lncRNA	exon	3895887	3896821	.	+	.	gene_id "lnc-bbug07305"	transcript_id "lnc-bbut00017407"	exon_number "1"
16	lncRNA	exon	3905712	3906453	.	+	.	gene_id "lnc-bbug07307"	transcript_id "lnc-bbut00017409"	exon_number "1"
16	lncRNA	exon	4047409	4048128	.	+	.	gene_id "lnc-bbug07313"	transcript_id "lnc-bbut00017415"	exon_number "1"
16	lncRNA	exon	4048353	4048376	.	+	.	gene_id "lnc-bbug07313"	transcript_id "lnc-bbut00017415"	exon_number "2"
16	lncRNA	exon	4317874	4318795	.	+	.	gene_id "lnc-bbug07319"	transcript_id "lnc-bbut00017421"	exon_number "1"
16	lncRNA	exon	4996997	4998032	.	+	.	gene_id "lnc-bbug07331"	transcript_id "lnc-bbut00017433"	exon_number "1"
16	lncRNA	exon	5567508	5568261	.	+	.	gene_id "lnc-bbug07344"	transcript_id "lnc-bbut00017446"	exon_number "1"
16	lncRNA	exon	5859478	5860447	.	+	.	gene_id "lnc-bbug07357"	transcript_id "lnc-bbut00017459"	exon_number "1"
16	lncRNA	exon	5885570	5885665	.	+	.	gene_id "lnc-bbug07361"	transcript_id "lnc-bbut00017463"	exon_number "1"
16	lncRNA	exon	5887007	5888036	.	+	.	gene_id "lnc-bbug07361"	transcript_id "lnc-bbut00017463"	exon_number "2"
16	lncRNA	exon	6064415	6065098	.	+	.	gene_id "lnc-bbug07368"	transcript_id "lnc-bbut00017470"	exon_number "1"
16	lncRNA	exon	6830628	6831547	.	+	.	gene_id "lnc-bbug07382"	transcript_id "lnc-bbut00017484"	exon_number "1"
16	lncRNA	exon	6994406	6994535	.	+	.	gene_id "lnc-bbug07383"	transcript_id "lnc-bbut00017485"	exon_number "1"
16	lncRNA	exon	6996843	6996991	.	+	.	gene_id "lnc-bbug07383"	transcript_id "lnc-bbut00017485"	exon_number "2"
16	lncRNA	exon	6999249	6999370	.	+	.	gene_id "lnc-bbug07383"	transcript_id "lnc-bbut00017485"	exon_number "3"
16	lncRNA	exon	7076594	7077081	.	+	.	gene_id "lnc-bbug07384"	transcript_id "lnc-bbut00017486"	exon_number "1"
16	lncRNA	exon	7089893	7090137	.	+	.	gene_id "lnc-bbug07384"	transcript_id "lnc-bbut00017486"	exon_number "2"

16	lncRNA	exon	7090926	7092017	.	+	.	gene_id "lnc-bbug07384"	transcript_id "lnc-bbut00017486"	exon_number "3"
16	lncRNA	exon	8784243	8784414	.	+	.	gene_id "lnc-bbug07408"	transcript_id "lnc-bbut00017583"	exon_number "1"
16	lncRNA	exon	8784540	8784613	.	+	.	gene_id "lnc-bbug07408"	transcript_id "lnc-bbut00017583"	exon_number "2"
16	lncRNA	exon	8784784	8784932	.	+	.	gene_id "lnc-bbug07408"	transcript_id "lnc-bbut00017583"	exon_number "3"
16	lncRNA	exon	9129174	9129308	.	+	.	gene_id "lnc-bbug07410"	transcript_id "lnc-bbut00017594"	exon_number "1"
16	lncRNA	exon	9129624	9129723	.	+	.	gene_id "lnc-bbug07410"	transcript_id "lnc-bbut00017594"	exon_number "2"
16	lncRNA	exon	9129912	9130135	.	+	.	gene_id "lnc-bbug07410"	transcript_id "lnc-bbut00017594"	exon_number "3"
16	lncRNA	exon	9131473	9131533	.	+	.	gene_id "lnc-bbug07410"	transcript_id "lnc-bbut00017594"	exon_number "4"
16	lncRNA	exon	9136512	9136726	.	+	.	gene_id "lnc-bbug07410"	transcript_id "lnc-bbut00017594"	exon_number "5"
16	lncRNA	exon	9138379	9138530	.	+	.	gene_id "lnc-bbug07410"	transcript_id "lnc-bbut00017594"	exon_number "6"
16	lncRNA	exon	9139248	9139411	.	+	.	gene_id "lnc-bbug07410"	transcript_id "lnc-bbut00017594"	exon_number "7"
16	lncRNA	exon	9139837	9139964	.	+	.	gene_id "lnc-bbug07410"	transcript_id "lnc-bbut00017594"	exon_number "8"
16	lncRNA	exon	9129174	9129308	.	+	.	gene_id "lnc-bbug07410"	transcript_id "lnc-bbut00017595"	exon_number "1"
16	lncRNA	exon	9129624	9129723	.	+	.	gene_id "lnc-bbug07410"	transcript_id "lnc-bbut00017595"	exon_number "2"
16	lncRNA	exon	9129912	9130135	.	+	.	gene_id "lnc-bbug07410"	transcript_id "lnc-bbut00017595"	exon_number "3"
16	lncRNA	exon	9131519	9131533	.	+	.	gene_id "lnc-bbug07410"	transcript_id "lnc-bbut00017595"	exon_number "4"
16	lncRNA	exon	9136512	9136726	.	+	.	gene_id "lnc-bbug07410"	transcript_id "lnc-bbut00017595"	exon_number "5"
16	lncRNA	exon	9138379	9138530	.	+	.	gene_id "lnc-bbug07410"	transcript_id "lnc-bbut00017595"	exon_number "6"
16	lncRNA	exon	9139248	9139411	.	+	.	gene_id "lnc-bbug07410"	transcript_id "lnc-bbut00017595"	exon_number "7"
16	lncRNA	exon	9139837	9139964	.	+	.	gene_id "lnc-bbug07410"	transcript_id "lnc-bbut00017595"	exon_number "8"
16	lncRNA	exon	9129174	9129308	.	+	.	gene_id "lnc-bbug07410"	transcript_id "lnc-bbut00017593"	exon_number "1"
16	lncRNA	exon	9129624	9129723	.	+	.	gene_id "lnc-bbug07410"	transcript_id "lnc-bbut00017593"	exon_number "2"
16	lncRNA	exon	9131519	9131533	.	+	.	gene_id "lnc-bbug07410"	transcript_id "lnc-bbut00017593"	exon_number "3"
16	lncRNA	exon	9136512	9136726	.	+	.	gene_id "lnc-bbug07410"	transcript_id "lnc-bbut00017593"	exon_number "4"
16	lncRNA	exon	9138379	9138530	.	+	.	gene_id "lnc-bbug07410"	transcript_id "lnc-bbut00017593"	exon_number "5"
16	lncRNA	exon	9139248	9139411	.	+	.	gene_id "lnc-bbug07410"	transcript_id "lnc-bbut00017593"	exon_number "6"
16	lncRNA	exon	9139837	9139964	.	+	.	gene_id "lnc-bbug07410"	transcript_id "lnc-bbut00017593"	exon_number "7"
16	lncRNA	exon	9129174	9129308	.	+	.	gene_id "lnc-bbug07410"	transcript_id "lnc-bbut00017592"	exon_number "1"
16	lncRNA	exon	9129624	9129723	.	+	.	gene_id "lnc-bbug07410"	transcript_id "lnc-bbut00017592"	exon_number "2"
16	lncRNA	exon	9129912	9130135	.	+	.	gene_id "lnc-bbug07410"	transcript_id "lnc-bbut00017592"	exon_number "3"
16	lncRNA	exon	9131519	9131533	.	+	.	gene_id "lnc-bbug07410"	transcript_id "lnc-bbut00017592"	exon_number "4"
16	lncRNA	exon	9136512	9136726	.	+	.	gene_id "lnc-bbug07410"	transcript_id "lnc-bbut00017592"	exon_number "5"
16	lncRNA	exon	9139248	9139411	.	+	.	gene_id "lnc-bbug07410"	transcript_id "lnc-bbut00017592"	exon_number "6"
16	lncRNA	exon	9139837	9139964	.	+	.	gene_id "lnc-bbug07410"	transcript_id "lnc-bbut00017592"	exon_number "7"
16	lncRNA	exon	9186229	9186327	.	+	.	gene_id "lnc-bbug07411"	transcript_id "lnc-bbut00017597"	exon_number "1"
16	lncRNA	exon	9186537	9186775	.	+	.	gene_id "lnc-bbug07411"	transcript_id "lnc-bbut00017597"	exon_number "2"
16	lncRNA	exon	9186986	9187194	.	+	.	gene_id "lnc-bbug07411"	transcript_id "lnc-bbut00017597"	exon_number "3"
16	lncRNA	exon	9187582	9189960	.	+	.	gene_id "lnc-bbug07411"	transcript_id "lnc-bbut00017597"	exon_number "4"
16	lncRNA	exon	9186229	9186327	.	+	.	gene_id "lnc-bbug07411"	transcript_id "lnc-bbut00017596"	exon_number "1"
16	lncRNA	exon	9186537	9186775	.	+	.	gene_id "lnc-bbug07411"	transcript_id "lnc-bbut00017596"	exon_number "2"
16	lncRNA	exon	9186986	9189960	.	+	.	gene_id "lnc-bbug07411"	transcript_id "lnc-bbut00017596"	exon_number "3"
16	lncRNA	exon	9186229	9186327	.	+	.	gene_id "lnc-bbug07411"	transcript_id "lnc-bbut00017598"	exon_number "1"
16	lncRNA	exon	9186537	9186775	.	+	.	gene_id "lnc-bbug07411"	transcript_id "lnc-bbut00017598"	exon_number "2"
16	lncRNA	exon	9189988	9190295	.	+	.	gene_id "lnc-bbug07411"	transcript_id "lnc-bbut00017598"	exon_number "3"
16	lncRNA	exon	9271033	9271808	.	+	.	gene_id "lnc-bbug07412"	transcript_id "lnc-bbut00017599"	exon_number "1"
16	lncRNA	exon	9277327	9277512	.	+	.	gene_id "lnc-bbug07412"	transcript_id "lnc-bbut00017599"	exon_number "2"
16	lncRNA	exon	9282297	9283622	.	+	.	gene_id "lnc-bbug07412"	transcript_id "lnc-bbut00017599"	exon_number "3"
16	lncRNA	exon	9720702	9722180	.	+	.	gene_id "lnc-bbug07414"	transcript_id "lnc-bbut00017601"	exon_number "1"
16	lncRNA	exon	9722352	9722882	.	+	.	gene_id "lnc-bbug07414"	transcript_id "lnc-bbut00017601"	exon_number "2"
16	lncRNA	exon	9743127	9743166	.	+	.	gene_id "lnc-bbug07415"	transcript_id "lnc-bbut00017607"	exon_number "1"
16	lncRNA	exon	9743356	9743404	.	+	.	gene_id "lnc-bbug07415"	transcript_id "lnc-bbut00017607"	exon_number "2"
16	lncRNA	exon	9743736	9743755	.	+	.	gene_id "lnc-bbug07415"	transcript_id "lnc-bbut00017607"	exon_number "3"

16	lncRNA	exon	9744529	9744630	.	+	.	gene_id "lnc-bbug07415"	transcript_id "lnc-bbut00017607"	exon_number "4"
16	lncRNA	exon	9745121	9747208	.	+	.	gene_id "lnc-bbug07415"	transcript_id "lnc-bbut00017607"	exon_number "5"
16	lncRNA	exon	10103721	10104368	.	+	.	gene_id "lnc-bbug07417"	transcript_id "lnc-bbut00017609"	exon_number "1"
16	lncRNA	exon	10106423	10106514	.	+	.	gene_id "lnc-bbug07417"	transcript_id "lnc-bbut00017609"	exon_number "2"
16	lncRNA	exon	10117858	10118155	.	+	.	gene_id "lnc-bbug07417"	transcript_id "lnc-bbut00017609"	exon_number "3"
16	lncRNA	exon	10103721	10104368	.	+	.	gene_id "lnc-bbug07417"	transcript_id "lnc-bbut00017610"	exon_number "1"
16	lncRNA	exon	10106423	10106514	.	+	.	gene_id "lnc-bbug07417"	transcript_id "lnc-bbut00017610"	exon_number "2"
16	lncRNA	exon	10118388	10118796	.	+	.	gene_id "lnc-bbug07417"	transcript_id "lnc-bbut00017610"	exon_number "3"
16	lncRNA	exon	10103721	10104368	.	+	.	gene_id "lnc-bbug07417"	transcript_id "lnc-bbut00017611"	exon_number "1"
16	lncRNA	exon	10106423	10106514	.	+	.	gene_id "lnc-bbug07417"	transcript_id "lnc-bbut00017611"	exon_number "2"
16	lncRNA	exon	10122293	10122722	.	+	.	gene_id "lnc-bbug07417"	transcript_id "lnc-bbut00017611"	exon_number "3"
16	lncRNA	exon	11108822	11109300	.	+	.	gene_id "lnc-bbug07419"	transcript_id "lnc-bbut00017613"	exon_number "1"
16	lncRNA	exon	11159314	11159509	.	+	.	gene_id "lnc-bbug07419"	transcript_id "lnc-bbut00017613"	exon_number "2"
16	lncRNA	exon	11165442	11165535	.	+	.	gene_id "lnc-bbug07419"	transcript_id "lnc-bbut00017613"	exon_number "3"
16	lncRNA	exon	11168641	11168748	.	+	.	gene_id "lnc-bbug07419"	transcript_id "lnc-bbut00017613"	exon_number "4"
16	lncRNA	exon	11270377	11270495	.	+	.	gene_id "lnc-bbug07420"	transcript_id "lnc-bbut00017616"	exon_number "1"
16	lncRNA	exon	11282247	11282322	.	+	.	gene_id "lnc-bbug07420"	transcript_id "lnc-bbut00017616"	exon_number "2"
16	lncRNA	exon	11284312	11284352	.	+	.	gene_id "lnc-bbug07420"	transcript_id "lnc-bbut00017616"	exon_number "3"
16	lncRNA	exon	11285003	11285565	.	+	.	gene_id "lnc-bbug07420"	transcript_id "lnc-bbut00017616"	exon_number "4"
16	lncRNA	exon	11270377	11270495	.	+	.	gene_id "lnc-bbug07420"	transcript_id "lnc-bbut00017617"	exon_number "1"
16	lncRNA	exon	11282247	11282322	.	+	.	gene_id "lnc-bbug07420"	transcript_id "lnc-bbut00017617"	exon_number "2"
16	lncRNA	exon	11284312	11284352	.	+	.	gene_id "lnc-bbug07420"	transcript_id "lnc-bbut00017617"	exon_number "3"
16	lncRNA	exon	11285036	11285565	.	+	.	gene_id "lnc-bbug07420"	transcript_id "lnc-bbut00017617"	exon_number "4"
16	lncRNA	exon	11270377	11270495	.	+	.	gene_id "lnc-bbug07420"	transcript_id "lnc-bbut00017615"	exon_number "1"
16	lncRNA	exon	11282247	11282322	.	+	.	gene_id "lnc-bbug07420"	transcript_id "lnc-bbut00017615"	exon_number "2"
16	lncRNA	exon	11285036	11285565	.	+	.	gene_id "lnc-bbug07420"	transcript_id "lnc-bbut00017615"	exon_number "3"
16	lncRNA	exon	11270377	11270495	.	+	.	gene_id "lnc-bbug07420"	transcript_id "lnc-bbut00017614"	exon_number "1"
16	lncRNA	exon	11282247	11282322	.	+	.	gene_id "lnc-bbug07420"	transcript_id "lnc-bbut00017614"	exon_number "2"
16	lncRNA	exon	11285003	11285565	.	+	.	gene_id "lnc-bbug07420"	transcript_id "lnc-bbut00017614"	exon_number "3"
16	lncRNA	exon	12900145	12901145	.	+	.	gene_id "lnc-bbug07421"	transcript_id "lnc-bbut00017618"	exon_number "1"
16	lncRNA	exon	13175726	13175915	.	+	.	gene_id "lnc-bbug07421"	transcript_id "lnc-bbut00017618"	exon_number "2"
16	lncRNA	exon	13459074	13459155	.	+	.	gene_id "lnc-bbug07421"	transcript_id "lnc-bbut00017618"	exon_number "3"
16	lncRNA	exon	13750782	13750918	.	+	.	gene_id "lnc-bbug07421"	transcript_id "lnc-bbut00017618"	exon_number "4"
16	lncRNA	exon	14017274	14017376	.	+	.	gene_id "lnc-bbug07421"	transcript_id "lnc-bbut00017618"	exon_number "5"
16	lncRNA	exon	14114131	14114227	.	+	.	gene_id "lnc-bbug07421"	transcript_id "lnc-bbut00017618"	exon_number "6"
16	lncRNA	exon	14134788	14136480	.	+	.	gene_id "lnc-bbug07421"	transcript_id "lnc-bbut00017618"	exon_number "7"
16	lncRNA	exon	16165566	16165599	.	+	.	gene_id "lnc-bbug07422"	transcript_id "lnc-bbut00017624"	exon_number "1"
16	lncRNA	exon	16325190	16325677	.	+	.	gene_id "lnc-bbug07422"	transcript_id "lnc-bbut00017624"	exon_number "2"
16	lncRNA	exon	16618799	16621457	.	+	.	gene_id "lnc-bbug07423"	transcript_id "lnc-bbut00017625"	exon_number "1"
16	lncRNA	exon	18202995	18203177	.	+	.	gene_id "lnc-bbug07428"	transcript_id "lnc-bbut00017631"	exon_number "1"
16	lncRNA	exon	18203350	18203410	.	+	.	gene_id "lnc-bbug07428"	transcript_id "lnc-bbut00017631"	exon_number "2"
16	lncRNA	exon	18204527	18204760	.	+	.	gene_id "lnc-bbug07428"	transcript_id "lnc-bbut00017631"	exon_number "3"
16	lncRNA	exon	18202995	18203177	.	+	.	gene_id "lnc-bbug07428"	transcript_id "lnc-bbut00017632"	exon_number "1"
16	lncRNA	exon	18203350	18203410	.	+	.	gene_id "lnc-bbug07428"	transcript_id "lnc-bbut00017632"	exon_number "2"
16	lncRNA	exon	18204778	18205106	.	+	.	gene_id "lnc-bbug07428"	transcript_id "lnc-bbut00017632"	exon_number "3"
16	lncRNA	exon	18267858	18268260	.	+	.	gene_id "lnc-bbug07429"	transcript_id "lnc-bbut00017633"	exon_number "1"
16	lncRNA	exon	18271729	18273589	.	+	.	gene_id "lnc-bbug07429"	transcript_id "lnc-bbut00017633"	exon_number "2"
16	lncRNA	exon	18640779	18641418	.	+	.	gene_id "lnc-bbug07430"	transcript_id "lnc-bbut00017634"	exon_number "1"
16	lncRNA	exon	18644815	18644917	.	+	.	gene_id "lnc-bbug07430"	transcript_id "lnc-bbut00017634"	exon_number "2"
16	lncRNA	exon	18650973	18652257	.	+	.	gene_id "lnc-bbug07430"	transcript_id "lnc-bbut00017634"	exon_number "3"
16	lncRNA	exon	19199458	19200069	.	+	.	gene_id "lnc-bbug07433"	transcript_id "lnc-bbut00017640"	exon_number "1"
16	lncRNA	exon	19232904	19233137	.	+	.	gene_id "lnc-bbug07433"	transcript_id "lnc-bbut00017640"	exon_number "2"

16	lncRNA	exon	19233639	19233842	.	+	.	gene_id "lnc-bbug07433"	transcript_id "lnc-bbut00017640"	exon_number "3"
16	lncRNA	exon	19234011	19236432	.	+	.	gene_id "lnc-bbug07433"	transcript_id "lnc-bbut00017640"	exon_number "4"
16	lncRNA	exon	19242850	19242901	.	+	.	gene_id "lnc-bbug07434"	transcript_id "lnc-bbut00017641"	exon_number "1"
16	lncRNA	exon	19245687	19245772	.	+	.	gene_id "lnc-bbug07434"	transcript_id "lnc-bbut00017641"	exon_number "2"
16	lncRNA	exon	19286455	19286965	.	+	.	gene_id "lnc-bbug07434"	transcript_id "lnc-bbut00017641"	exon_number "3"
16	lncRNA	exon	20350651	20350863	.	+	.	gene_id "lnc-bbug07438"	transcript_id "lnc-bbut00017648"	exon_number "1"
16	lncRNA	exon	20358424	20358488	.	+	.	gene_id "lnc-bbug07438"	transcript_id "lnc-bbut00017648"	exon_number "2"
16	lncRNA	exon	20486214	20486337	.	+	.	gene_id "lnc-bbug07441"	transcript_id "lnc-bbut00017652"	exon_number "1"
16	lncRNA	exon	20499273	20499357	.	+	.	gene_id "lnc-bbug07441"	transcript_id "lnc-bbut00017652"	exon_number "2"
16	lncRNA	exon	20503055	20503153	.	+	.	gene_id "lnc-bbug07441"	transcript_id "lnc-bbut00017652"	exon_number "3"
16	lncRNA	exon	20509397	20510943	.	+	.	gene_id "lnc-bbug07441"	transcript_id "lnc-bbut00017652"	exon_number "4"
16	lncRNA	exon	20620781	20620918	.	+	.	gene_id "lnc-bbug07443"	transcript_id "lnc-bbut00017655"	exon_number "1"
16	lncRNA	exon	20622263	20622335	.	+	.	gene_id "lnc-bbug07443"	transcript_id "lnc-bbut00017655"	exon_number "2"
16	lncRNA	exon	20630586	20632776	.	+	.	gene_id "lnc-bbug07443"	transcript_id "lnc-bbut00017655"	exon_number "3"
16	lncRNA	exon	20620781	20620918	.	+	.	gene_id "lnc-bbug07443"	transcript_id "lnc-bbut00017654"	exon_number "1"
16	lncRNA	exon	20622263	20622331	.	+	.	gene_id "lnc-bbug07443"	transcript_id "lnc-bbut00017654"	exon_number "2"
16	lncRNA	exon	20630586	20632776	.	+	.	gene_id "lnc-bbug07443"	transcript_id "lnc-bbut00017654"	exon_number "3"
16	lncRNA	exon	21344606	21344729	.	+	.	gene_id "lnc-bbug07446"	transcript_id "lnc-bbut00017660"	exon_number "1"
16	lncRNA	exon	21348373	21348483	.	+	.	gene_id "lnc-bbug07446"	transcript_id "lnc-bbut00017660"	exon_number "2"
16	lncRNA	exon	21360617	21361098	.	+	.	gene_id "lnc-bbug07446"	transcript_id "lnc-bbut00017660"	exon_number "3"
16	lncRNA	exon	21802318	21809706	.	+	.	gene_id "lnc-bbug07450"	transcript_id "lnc-bbut00017668"	exon_number "1"
16	lncRNA	exon	22037271	22039680	.	+	.	gene_id "lnc-bbug07451"	transcript_id "lnc-bbut00017673"	exon_number "1"
16	lncRNA	exon	22049325	22049395	.	+	.	gene_id "lnc-bbug07451"	transcript_id "lnc-bbut00017673"	exon_number "2"
16	lncRNA	exon	22050879	22052429	.	+	.	gene_id "lnc-bbug07451"	transcript_id "lnc-bbut00017673"	exon_number "3"
16	lncRNA	exon	24326709	24327220	.	+	.	gene_id "lnc-bbug07458"	transcript_id "lnc-bbut00017691"	exon_number "1"
16	lncRNA	exon	24332755	24332845	.	+	.	gene_id "lnc-bbug07458"	transcript_id "lnc-bbut00017691"	exon_number "2"
16	lncRNA	exon	24334027	24334106	.	+	.	gene_id "lnc-bbug07458"	transcript_id "lnc-bbut00017691"	exon_number "3"
16	lncRNA	exon	24339149	24339311	.	+	.	gene_id "lnc-bbug07458"	transcript_id "lnc-bbut00017691"	exon_number "4"
16	lncRNA	exon	25120485	25121037	.	+	.	gene_id "lnc-bbug07459"	transcript_id "lnc-bbut00017692"	exon_number "1"
16	lncRNA	exon	27109017	27109423	.	+	.	gene_id "lnc-bbug07467"	transcript_id "lnc-bbut00017707"	exon_number "1"
16	lncRNA	exon	27113476	27113557	.	+	.	gene_id "lnc-bbug07467"	transcript_id "lnc-bbut00017707"	exon_number "2"
16	lncRNA	exon	27114243	27115188	.	+	.	gene_id "lnc-bbug07467"	transcript_id "lnc-bbut00017707"	exon_number "3"
16	lncRNA	exon	28430308	28432312	.	+	.	gene_id "lnc-bbug07471"	transcript_id "lnc-bbut00017719"	exon_number "1"
16	lncRNA	exon	28697984	28698651	.	+	.	gene_id "lnc-bbug07472"	transcript_id "lnc-bbut00017720"	exon_number "1"
16	lncRNA	exon	28700137	28700293	.	+	.	gene_id "lnc-bbug07472"	transcript_id "lnc-bbut00017720"	exon_number "2"
16	lncRNA	exon	28702691	28706478	.	+	.	gene_id "lnc-bbug07472"	transcript_id "lnc-bbut00017720"	exon_number "3"
16	lncRNA	exon	28774082	28774706	.	+	.	gene_id "lnc-bbug07475"	transcript_id "lnc-bbut00017723"	exon_number "1"
16	lncRNA	exon	28780537	28780777	.	+	.	gene_id "lnc-bbug07475"	transcript_id "lnc-bbut00017723"	exon_number "2"
16	lncRNA	exon	28781189	28781606	.	+	.	gene_id "lnc-bbug07475"	transcript_id "lnc-bbut00017723"	exon_number "3"
16	lncRNA	exon	29203765	29204444	.	+	.	gene_id "lnc-bbug07478"	transcript_id "lnc-bbut00017731"	exon_number "1"
16	lncRNA	exon	29204910	29205042	.	+	.	gene_id "lnc-bbug07478"	transcript_id "lnc-bbut00017731"	exon_number "2"
16	lncRNA	exon	29205652	29205710	.	+	.	gene_id "lnc-bbug07478"	transcript_id "lnc-bbut00017731"	exon_number "3"
16	lncRNA	exon	29205917	29208294	.	+	.	gene_id "lnc-bbug07478"	transcript_id "lnc-bbut00017731"	exon_number "4"
16	lncRNA	exon	29203765	29204444	.	+	.	gene_id "lnc-bbug07478"	transcript_id "lnc-bbut00017732"	exon_number "1"
16	lncRNA	exon	29204910	29205042	.	+	.	gene_id "lnc-bbug07478"	transcript_id "lnc-bbut00017732"	exon_number "2"
16	lncRNA	exon	29205652	29205710	.	+	.	gene_id "lnc-bbug07478"	transcript_id "lnc-bbut00017732"	exon_number "3"
16	lncRNA	exon	29210066	29210929	.	+	.	gene_id "lnc-bbug07478"	transcript_id "lnc-bbut00017732"	exon_number "4"
16	lncRNA	exon	29400959	29401027	.	+	.	gene_id "lnc-bbug07480"	transcript_id "lnc-bbut00017735"	exon_number "1"
16	lncRNA	exon	29401651	29402104	.	+	.	gene_id "lnc-bbug07480"	transcript_id "lnc-bbut00017735"	exon_number "2"
16	lncRNA	exon	29694637	29697123	.	+	.	gene_id "lnc-bbug07481"	transcript_id "lnc-bbut00017737"	exon_number "1"
16	lncRNA	exon	29697687	29697831	.	+	.	gene_id "lnc-bbug07481"	transcript_id "lnc-bbut00017737"	exon_number "2"
16	lncRNA	exon	29699161	29699294	.	+	.	gene_id "lnc-bbug07481"	transcript_id "lnc-bbut00017737"	exon_number "3"

16	lncRNA	exon	29717411	29717824	.	+	.	gene_id "lnc-bbug07481"	transcript_id "lnc-bbut00017737"	exon_number "4"
16	lncRNA	exon	29694638	29694952	.	+	.	gene_id "lnc-bbug07481"	transcript_id "lnc-bbut00017738"	exon_number "1"
16	lncRNA	exon	29697687	29697831	.	+	.	gene_id "lnc-bbug07481"	transcript_id "lnc-bbut00017738"	exon_number "2"
16	lncRNA	exon	29699161	29699294	.	+	.	gene_id "lnc-bbug07481"	transcript_id "lnc-bbut00017738"	exon_number "3"
16	lncRNA	exon	29717411	29717824	.	+	.	gene_id "lnc-bbug07481"	transcript_id "lnc-bbut00017738"	exon_number "4"
16	lncRNA	exon	29710043	29711840	.	+	.	gene_id "lnc-bbug07482"	transcript_id "lnc-bbut00017739"	exon_number "1"
16	lncRNA	exon	30045758	30046062	.	+	.	gene_id "lnc-bbug07485"	transcript_id "lnc-bbut00017742"	exon_number "1"
16	lncRNA	exon	30051212	30051305	.	+	.	gene_id "lnc-bbug07485"	transcript_id "lnc-bbut00017742"	exon_number "2"
16	lncRNA	exon	30053482	30054155	.	+	.	gene_id "lnc-bbug07485"	transcript_id "lnc-bbut00017742"	exon_number "3"
16	lncRNA	exon	30989025	30989212	.	+	.	gene_id "lnc-bbug07495"	transcript_id "lnc-bbut00017772"	exon_number "1"
16	lncRNA	exon	30990660	30990754	.	+	.	gene_id "lnc-bbug07495"	transcript_id "lnc-bbut00017772"	exon_number "2"
16	lncRNA	exon	30999265	30999888	.	+	.	gene_id "lnc-bbug07495"	transcript_id "lnc-bbut00017772"	exon_number "3"
16	lncRNA	exon	31006112	31009092	.	+	.	gene_id "lnc-bbug07496"	transcript_id "lnc-bbut00017773"	exon_number "1"
16	lncRNA	exon	31010941	31011128	.	+	.	gene_id "lnc-bbug07496"	transcript_id "lnc-bbut00017773"	exon_number "2"
16	lncRNA	exon	31188858	31189338	.	+	.	gene_id "lnc-bbug07499"	transcript_id "lnc-bbut00017778"	exon_number "1"
16	lncRNA	exon	31387121	31387869	.	+	.	gene_id "lnc-bbug07502"	transcript_id "lnc-bbut00017782"	exon_number "1"
16	lncRNA	exon	31410890	31411693	.	+	.	gene_id "lnc-bbug07504"	transcript_id "lnc-bbut00017784"	exon_number "1"
16	lncRNA	exon	31412726	31414275	.	+	.	gene_id "lnc-bbug07504"	transcript_id "lnc-bbut00017784"	exon_number "2"
16	lncRNA	exon	31471853	31471974	.	+	.	gene_id "lnc-bbug07505"	transcript_id "lnc-bbut00017785"	exon_number "1"
16	lncRNA	exon	31473752	31474375	.	+	.	gene_id "lnc-bbug07505"	transcript_id "lnc-bbut00017785"	exon_number "2"
16	lncRNA	exon	31471907	31472058	.	+	.	gene_id "lnc-bbug07505"	transcript_id "lnc-bbut00017786"	exon_number "1"
16	lncRNA	exon	31473752	31474375	.	+	.	gene_id "lnc-bbug07505"	transcript_id "lnc-bbut00017786"	exon_number "2"
16	lncRNA	exon	31594622	31597189	.	+	.	gene_id "lnc-bbug07507"	transcript_id "lnc-bbut00017789"	exon_number "1"
16	lncRNA	exon	32457715	32458465	.	+	.	gene_id "lnc-bbug07510"	transcript_id "lnc-bbut00017798"	exon_number "1"
16	lncRNA	exon	32460116	32460260	.	+	.	gene_id "lnc-bbug07510"	transcript_id "lnc-bbut00017798"	exon_number "2"
16	lncRNA	exon	33083721	33084054	.	+	.	gene_id "lnc-bbug07519"	transcript_id "lnc-bbut00017829"	exon_number "1"
16	lncRNA	exon	33088140	33090092	.	+	.	gene_id "lnc-bbug07519"	transcript_id "lnc-bbut00017829"	exon_number "2"
16	lncRNA	exon	33712732	33715365	.	+	.	gene_id "lnc-bbug07526"	transcript_id "lnc-bbut00017855"	exon_number "1"
16	lncRNA	exon	33905884	33906465	.	+	.	gene_id "lnc-bbug07531"	transcript_id "lnc-bbut00017860"	exon_number "1"
16	lncRNA	exon	33938168	33939111	.	+	.	gene_id "lnc-bbug07532"	transcript_id "lnc-bbut00017861"	exon_number "1"
16	lncRNA	exon	33958539	33958547	.	+	.	gene_id "lnc-bbug07533"	transcript_id "lnc-bbut00017862"	exon_number "1"
16	lncRNA	exon	33962366	33962905	.	+	.	gene_id "lnc-bbug07533"	transcript_id "lnc-bbut00017862"	exon_number "2"
16	lncRNA	exon	33963019	33963355	.	+	.	gene_id "lnc-bbug07533"	transcript_id "lnc-bbut00017862"	exon_number "3"
16	lncRNA	exon	34123127	34123144	.	+	.	gene_id "lnc-bbug07541"	transcript_id "lnc-bbut00017870"	exon_number "1"
16	lncRNA	exon	34127756	34128698	.	+	.	gene_id "lnc-bbug07541"	transcript_id "lnc-bbut00017870"	exon_number "2"
16	lncRNA	exon	34563840	34564148	.	+	.	gene_id "lnc-bbug07551"	transcript_id "lnc-bbut00017881"	exon_number "1"
16	lncRNA	exon	34581241	34582174	.	+	.	gene_id "lnc-bbug07552"	transcript_id "lnc-bbut00017882"	exon_number "1"
16	lncRNA	exon	34619880	34620830	.	+	.	gene_id "lnc-bbug07555"	transcript_id "lnc-bbut00017885"	exon_number "1"
16	lncRNA	exon	34730943	34731269	.	+	.	gene_id "lnc-bbug07558"	transcript_id "lnc-bbut00017888"	exon_number "1"
16	lncRNA	exon	35134949	35135523	.	+	.	gene_id "lnc-bbug07574"	transcript_id "lnc-bbut00017904"	exon_number "1"
16	lncRNA	exon	35143349	35143368	.	+	.	gene_id "lnc-bbug07574"	transcript_id "lnc-bbut00017904"	exon_number "2"
16	lncRNA	exon	35516008	35519840	.	+	.	gene_id "lnc-bbug07590"	transcript_id "lnc-bbut00017920"	exon_number "1"
16	lncRNA	exon	35610328	35611710	.	+	.	gene_id "lnc-bbug07594"	transcript_id "lnc-bbut00017924"	exon_number "1"
16	lncRNA	exon	35986153	35986485	.	+	.	gene_id "lnc-bbug07597"	transcript_id "lnc-bbut00017927"	exon_number "1"
16	lncRNA	exon	36142790	36143722	.	+	.	gene_id "lnc-bbug07603"	transcript_id "lnc-bbut00017933"	exon_number "1"
16	lncRNA	exon	36387134	36388082	.	+	.	gene_id "lnc-bbug07613"	transcript_id "lnc-bbut00017943"	exon_number "1"
16	lncRNA	exon	36781784	36782560	.	+	.	gene_id "lnc-bbug07618"	transcript_id "lnc-bbut00017948"	exon_number "1"
16	lncRNA	exon	37163159	37165672	.	+	.	gene_id "lnc-bbug07632"	transcript_id "lnc-bbut00017971"	exon_number "1"
16	lncRNA	exon	37166474	37166569	.	+	.	gene_id "lnc-bbug07632"	transcript_id "lnc-bbut00017971"	exon_number "2"
16	lncRNA	exon	37168457	37168928	.	+	.	gene_id "lnc-bbug07632"	transcript_id "lnc-bbut00017971"	exon_number "3"
16	lncRNA	exon	37396945	37397920	.	+	.	gene_id "lnc-bbug07645"	transcript_id "lnc-bbut00017987"	exon_number "1"
16	lncRNA	exon	37522630	37523590	.	+	.	gene_id "lnc-bbug07652"	transcript_id "lnc-bbut00017994"	exon_number "1"

16	lncRNA	exon	37587057	37587795	.	+	.	gene_id "lnc-bbug07656"	transcript_id "lnc-bbut00017998"	exon_number "1"
16	lncRNA	exon	37588077	37588301	.	+	.	gene_id "lnc-bbug07656"	transcript_id "lnc-bbut00017998"	exon_number "2"
16	lncRNA	exon	37713692	37714600	.	+	.	gene_id "lnc-bbug07662"	transcript_id "lnc-bbut00018004"	exon_number "1"
16	lncRNA	exon	37719150	37719158	.	+	.	gene_id "lnc-bbug07662"	transcript_id "lnc-bbut00018004"	exon_number "2"
16	lncRNA	exon	37951547	37951885	.	+	.	gene_id "lnc-bbug07671"	transcript_id "lnc-bbut00018013"	exon_number "1"
16	lncRNA	exon	37952030	37952772	.	+	.	gene_id "lnc-bbug07671"	transcript_id "lnc-bbut00018013"	exon_number "2"
16	lncRNA	exon	38235744	38236022	.	+	.	gene_id "lnc-bbug07676"	transcript_id "lnc-bbut00018018"	exon_number "1"
16	lncRNA	exon	38236483	38236658	.	+	.	gene_id "lnc-bbug07676"	transcript_id "lnc-bbut00018018"	exon_number "2"
16	lncRNA	exon	38243971	38244386	.	+	.	gene_id "lnc-bbug07676"	transcript_id "lnc-bbut00018018"	exon_number "3"
16	lncRNA	exon	38244908	38245088	.	+	.	gene_id "lnc-bbug07676"	transcript_id "lnc-bbut00018018"	exon_number "4"
16	lncRNA	exon	38237711	38237766	.	+	.	gene_id "lnc-bbug07677"	transcript_id "lnc-bbut00018019"	exon_number "1"
16	lncRNA	exon	38238943	38239126	.	+	.	gene_id "lnc-bbug07677"	transcript_id "lnc-bbut00018019"	exon_number "2"
16	lncRNA	exon	38295310	38295427	.	+	.	gene_id "lnc-bbug07678"	transcript_id "lnc-bbut00018020"	exon_number "1"
16	lncRNA	exon	38295627	38295722	.	+	.	gene_id "lnc-bbug07678"	transcript_id "lnc-bbut00018020"	exon_number "2"
16	lncRNA	exon	38295910	38296714	.	+	.	gene_id "lnc-bbug07678"	transcript_id "lnc-bbut00018020"	exon_number "3"
16	lncRNA	exon	39020797	39021855	.	+	.	gene_id "lnc-bbug07686"	transcript_id "lnc-bbut00018034"	exon_number "1"
16	lncRNA	exon	41392821	41393087	.	+	.	gene_id "lnc-bbug07713"	transcript_id "lnc-bbut00018091"	exon_number "1"
16	lncRNA	exon	41393555	41393686	.	+	.	gene_id "lnc-bbug07713"	transcript_id "lnc-bbut00018091"	exon_number "2"
16	lncRNA	exon	41395651	41395799	.	+	.	gene_id "lnc-bbug07713"	transcript_id "lnc-bbut00018091"	exon_number "3"
16	lncRNA	exon	41415738	41415902	.	+	.	gene_id "lnc-bbug07713"	transcript_id "lnc-bbut00018091"	exon_number "4"
16	lncRNA	exon	41417389	41418502	.	+	.	gene_id "lnc-bbug07713"	transcript_id "lnc-bbut00018091"	exon_number "5"
16	lncRNA	exon	41593940	41596067	.	+	.	gene_id "lnc-bbug07716"	transcript_id "lnc-bbut00018094"	exon_number "1"
16	lncRNA	exon	41831629	41832719	.	+	.	gene_id "lnc-bbug07720"	transcript_id "lnc-bbut00018103"	exon_number "1"
16	lncRNA	exon	41879893	41879972	.	+	.	gene_id "lnc-bbug07720"	transcript_id "lnc-bbut00018103"	exon_number "2"
16	lncRNA	exon	41893073	41894174	.	+	.	gene_id "lnc-bbug07720"	transcript_id "lnc-bbut00018103"	exon_number "3"
16	lncRNA	exon	41831635	41832719	.	+	.	gene_id "lnc-bbug07720"	transcript_id "lnc-bbut00018104"	exon_number "1"
16	lncRNA	exon	41879893	41879972	.	+	.	gene_id "lnc-bbug07720"	transcript_id "lnc-bbut00018104"	exon_number "2"
16	lncRNA	exon	41886148	41886296	.	+	.	gene_id "lnc-bbug07720"	transcript_id "lnc-bbut00018104"	exon_number "3"
16	lncRNA	exon	42646828	42646984	.	+	.	gene_id "lnc-bbug07723"	transcript_id "lnc-bbut00018110"	exon_number "1"
16	lncRNA	exon	42660356	42660475	.	+	.	gene_id "lnc-bbug07723"	transcript_id "lnc-bbut00018110"	exon_number "2"
16	lncRNA	exon	42663339	42663431	.	+	.	gene_id "lnc-bbug07723"	transcript_id "lnc-bbut00018110"	exon_number "3"
16	lncRNA	exon	42670128	42671015	.	+	.	gene_id "lnc-bbug07723"	transcript_id "lnc-bbut00018110"	exon_number "4"
16	lncRNA	exon	42971033	42972219	.	+	.	gene_id "lnc-bbug07725"	transcript_id "lnc-bbut00018112"	exon_number "1"
16	lncRNA	exon	44266081	44266281	.	+	.	gene_id "lnc-bbug07729"	transcript_id "lnc-bbut00018119"	exon_number "1"
16	lncRNA	exon	44268163	44268480	.	+	.	gene_id "lnc-bbug07729"	transcript_id "lnc-bbut00018119"	exon_number "2"
16	lncRNA	exon	44272927	44273698	.	+	.	gene_id "lnc-bbug07729"	transcript_id "lnc-bbut00018119"	exon_number "3"
16	lncRNA	exon	46420544	46425237	.	+	.	gene_id "lnc-bbug07739"	transcript_id "lnc-bbut00018155"	exon_number "1"
16	lncRNA	exon	46796148	46796315	.	+	.	gene_id "lnc-bbug07740"	transcript_id "lnc-bbut00018156"	exon_number "1"
16	lncRNA	exon	46804367	46807121	.	+	.	gene_id "lnc-bbug07740"	transcript_id "lnc-bbut00018156"	exon_number "2"
16	lncRNA	exon	46985057	46985117	.	+	.	gene_id "lnc-bbug07742"	transcript_id "lnc-bbut00018159"	exon_number "1"
16	lncRNA	exon	46987205	46987452	.	+	.	gene_id "lnc-bbug07742"	transcript_id "lnc-bbut00018159"	exon_number "2"
16	lncRNA	exon	48117362	48117477	.	+	.	gene_id "lnc-bbug07746"	transcript_id "lnc-bbut00018165"	exon_number "1"
16	lncRNA	exon	48131388	48133340	.	+	.	gene_id "lnc-bbug07746"	transcript_id "lnc-bbut00018165"	exon_number "2"
16	lncRNA	exon	50752425	50752633	.	+	.	gene_id "lnc-bbug07757"	transcript_id "lnc-bbut00018185"	exon_number "1"
16	lncRNA	exon	50752732	50752912	.	+	.	gene_id "lnc-bbug07757"	transcript_id "lnc-bbut00018185"	exon_number "2"
16	lncRNA	exon	50876561	50877279	.	+	.	gene_id "lnc-bbug07758"	transcript_id "lnc-bbut00018186"	exon_number "1"
16	lncRNA	exon	50885874	50886357	.	+	.	gene_id "lnc-bbug07758"	transcript_id "lnc-bbut00018186"	exon_number "2"
16	lncRNA	exon	51114536	51114726	.	+	.	gene_id "lnc-bbug07759"	transcript_id "lnc-bbut00018187"	exon_number "1"
16	lncRNA	exon	51114820	51114955	.	+	.	gene_id "lnc-bbug07759"	transcript_id "lnc-bbut00018187"	exon_number "2"
16	lncRNA	exon	51116577	51116800	.	+	.	gene_id "lnc-bbug07759"	transcript_id "lnc-bbut00018187"	exon_number "3"
16	lncRNA	exon	51241901	51244518	.	+	.	gene_id "lnc-bbug07762"	transcript_id "lnc-bbut00018190"	exon_number "1"
16	lncRNA	exon	51482067	51483062	.	+	.	gene_id "lnc-bbug07765"	transcript_id "lnc-bbut00018193"	exon_number "1"

16	lncRNA	exon	51484154	51484333	.	+	.	gene_id "lnc-bbug07765"	transcript_id "lnc-bbut00018193"	exon_number "2"
16	lncRNA	exon	51484425	51485338	.	+	.	gene_id "lnc-bbug07765"	transcript_id "lnc-bbut00018193"	exon_number "3"
16	lncRNA	exon	51485633	51485853	.	+	.	gene_id "lnc-bbug07765"	transcript_id "lnc-bbut00018193"	exon_number "4"
16	lncRNA	exon	51487582	51488022	.	+	.	gene_id "lnc-bbug07765"	transcript_id "lnc-bbut00018193"	exon_number "5"
16	lncRNA	exon	51483645	51484333	.	+	.	gene_id "lnc-bbug07765"	transcript_id "lnc-bbut00018195"	exon_number "1"
16	lncRNA	exon	51484425	51485338	.	+	.	gene_id "lnc-bbug07765"	transcript_id "lnc-bbut00018195"	exon_number "2"
16	lncRNA	exon	51485633	51485853	.	+	.	gene_id "lnc-bbug07765"	transcript_id "lnc-bbut00018195"	exon_number "3"
16	lncRNA	exon	51487582	51488022	.	+	.	gene_id "lnc-bbug07765"	transcript_id "lnc-bbut00018195"	exon_number "4"
16	lncRNA	exon	51483645	51484333	.	+	.	gene_id "lnc-bbug07765"	transcript_id "lnc-bbut00018194"	exon_number "1"
16	lncRNA	exon	51484425	51485084	.	+	.	gene_id "lnc-bbug07765"	transcript_id "lnc-bbut00018194"	exon_number "2"
16	lncRNA	exon	51485633	51485853	.	+	.	gene_id "lnc-bbug07765"	transcript_id "lnc-bbut00018194"	exon_number "3"
16	lncRNA	exon	51487582	51488022	.	+	.	gene_id "lnc-bbug07765"	transcript_id "lnc-bbut00018194"	exon_number "4"
16	lncRNA	exon	51861394	51861829	.	+	.	gene_id "lnc-bbug07766"	transcript_id "lnc-bbut00018196"	exon_number "1"
16	lncRNA	exon	51941916	51942032	.	+	.	gene_id "lnc-bbug07766"	transcript_id "lnc-bbut00018196"	exon_number "2"
16	lncRNA	exon	52040862	52041013	.	+	.	gene_id "lnc-bbug07766"	transcript_id "lnc-bbut00018196"	exon_number "3"
16	lncRNA	exon	52067152	52067214	.	+	.	gene_id "lnc-bbug07766"	transcript_id "lnc-bbut00018196"	exon_number "4"
16	lncRNA	exon	52093342	52093428	.	+	.	gene_id "lnc-bbug07766"	transcript_id "lnc-bbut00018196"	exon_number "5"
16	lncRNA	exon	52096141	52097235	.	+	.	gene_id "lnc-bbug07766"	transcript_id "lnc-bbut00018196"	exon_number "6"
16	lncRNA	exon	52232320	52235810	.	+	.	gene_id "lnc-bbug07768"	transcript_id "lnc-bbut00018199"	exon_number "1"
16	lncRNA	exon	52236874	52237007	.	+	.	gene_id "lnc-bbug07768"	transcript_id "lnc-bbut00018199"	exon_number "2"
16	lncRNA	exon	52242157	52242263	.	+	.	gene_id "lnc-bbug07768"	transcript_id "lnc-bbut00018199"	exon_number "3"
16	lncRNA	exon	52309083	52309198	.	+	.	gene_id "lnc-bbug07768"	transcript_id "lnc-bbut00018199"	exon_number "4"
16	lncRNA	exon	52344450	52344554	.	+	.	gene_id "lnc-bbug07768"	transcript_id "lnc-bbut00018199"	exon_number "5"
16	lncRNA	exon	52347434	52349053	.	+	.	gene_id "lnc-bbug07768"	transcript_id "lnc-bbut00018199"	exon_number "6"
16	lncRNA	exon	54927191	54927338	.	+	.	gene_id "lnc-bbug07773"	transcript_id "lnc-bbut00018205"	exon_number "1"
16	lncRNA	exon	54930102	54930162	.	+	.	gene_id "lnc-bbug07773"	transcript_id "lnc-bbut00018205"	exon_number "2"
16	lncRNA	exon	54930651	54930986	.	+	.	gene_id "lnc-bbug07773"	transcript_id "lnc-bbut00018205"	exon_number "3"
16	lncRNA	exon	54931737	54933219	.	+	.	gene_id "lnc-bbug07773"	transcript_id "lnc-bbut00018205"	exon_number "4"
16	lncRNA	exon	55232698	55234610	.	+	.	gene_id "lnc-bbug07780"	transcript_id "lnc-bbut00018222"	exon_number "1"
16	lncRNA	exon	55446327	55446439	.	+	.	gene_id "lnc-bbug07785"	transcript_id "lnc-bbut00018239"	exon_number "1"
16	lncRNA	exon	55446997	55447074	.	+	.	gene_id "lnc-bbug07785"	transcript_id "lnc-bbut00018239"	exon_number "2"
16	lncRNA	exon	55470439	55470966	.	+	.	gene_id "lnc-bbug07785"	transcript_id "lnc-bbut00018239"	exon_number "3"
16	lncRNA	exon	55485447	55485823	.	+	.	gene_id "lnc-bbug07786"	transcript_id "lnc-bbut00018240"	exon_number "1"
16	lncRNA	exon	56187145	56187194	.	+	.	gene_id "lnc-bbug07797"	transcript_id "lnc-bbut00018266"	exon_number "1"
16	lncRNA	exon	56235917	56236209	.	+	.	gene_id "lnc-bbug07797"	transcript_id "lnc-bbut00018266"	exon_number "2"
16	lncRNA	exon	56237471	56237504	.	+	.	gene_id "lnc-bbug07797"	transcript_id "lnc-bbut00018266"	exon_number "3"
16	lncRNA	exon	56238213	56238347	.	+	.	gene_id "lnc-bbug07797"	transcript_id "lnc-bbut00018266"	exon_number "4"
16	lncRNA	exon	56239209	56241932	.	+	.	gene_id "lnc-bbug07797"	transcript_id "lnc-bbut00018266"	exon_number "5"
16	lncRNA	exon	56242628	56242987	.	+	.	gene_id "lnc-bbug07798"	transcript_id "lnc-bbut00018267"	exon_number "1"
16	lncRNA	exon	56249181	56250833	.	+	.	gene_id "lnc-bbug07798"	transcript_id "lnc-bbut00018267"	exon_number "2"
16	lncRNA	exon	56392548	56392640	.	+	.	gene_id "lnc-bbug07800"	transcript_id "lnc-bbut00018270"	exon_number "1"
16	lncRNA	exon	56404045	56404266	.	+	.	gene_id "lnc-bbug07800"	transcript_id "lnc-bbut00018270"	exon_number "2"
16	lncRNA	exon	56422295	56422357	.	+	.	gene_id "lnc-bbug07800"	transcript_id "lnc-bbut00018270"	exon_number "3"
16	lncRNA	exon	56422985	56423023	.	+	.	gene_id "lnc-bbug07800"	transcript_id "lnc-bbut00018270"	exon_number "4"
16	lncRNA	exon	56423130	56423205	.	+	.	gene_id "lnc-bbug07800"	transcript_id "lnc-bbut00018270"	exon_number "5"
16	lncRNA	exon	56423781	56423817	.	+	.	gene_id "lnc-bbug07800"	transcript_id "lnc-bbut00018270"	exon_number "6"
16	lncRNA	exon	56424528	56424577	.	+	.	gene_id "lnc-bbug07800"	transcript_id "lnc-bbut00018270"	exon_number "7"
16	lncRNA	exon	56425125	56425175	.	+	.	gene_id "lnc-bbug07800"	transcript_id "lnc-bbut00018270"	exon_number "8"
16	lncRNA	exon	56427060	56428247	.	+	.	gene_id "lnc-bbug07800"	transcript_id "lnc-bbut00018270"	exon_number "9"
16	lncRNA	exon	56438023	56438208	.	+	.	gene_id "lnc-bbug07801"	transcript_id "lnc-bbut00018271"	exon_number "1"
16	lncRNA	exon	56442634	56442672	.	+	.	gene_id "lnc-bbug07801"	transcript_id "lnc-bbut00018271"	exon_number "2"
16	lncRNA	exon	56442875	56442949	.	+	.	gene_id "lnc-bbug07801"	transcript_id "lnc-bbut00018271"	exon_number "3"

16	lncRNA	exon	56444546	56444582	.	+	.	gene_id "lnc-bbug07801"	transcript_id "lnc-bbut00018271"	exon_number "4"
16	lncRNA	exon	56444776	56444806	.	+	.	gene_id "lnc-bbug07801"	transcript_id "lnc-bbut00018271"	exon_number "5"
16	lncRNA	exon	56445387	56445586	.	+	.	gene_id "lnc-bbug07801"	transcript_id "lnc-bbut00018271"	exon_number "6"
16	lncRNA	exon	57046091	57047082	.	+	.	gene_id "lnc-bbug07806"	transcript_id "lnc-bbut00018280"	exon_number "1"
16	lncRNA	exon	57047795	57048122	.	+	.	gene_id "lnc-bbug07806"	transcript_id "lnc-bbut00018280"	exon_number "2"
16	lncRNA	exon	57647028	57649620	.	+	.	gene_id "lnc-bbug07814"	transcript_id "lnc-bbut00018297"	exon_number "1"
16	lncRNA	exon	57733477	57733583	.	+	.	gene_id "lnc-bbug07814"	transcript_id "lnc-bbut00018297"	exon_number "2"
16	lncRNA	exon	57922180	57922364	.	+	.	gene_id "lnc-bbug07814"	transcript_id "lnc-bbut00018297"	exon_number "3"
16	lncRNA	exon	57799568	57800645	.	+	.	gene_id "lnc-bbug07815"	transcript_id "lnc-bbut00018298"	exon_number "1"
16	lncRNA	exon	57804341	57804508	.	+	.	gene_id "lnc-bbug07815"	transcript_id "lnc-bbut00018298"	exon_number "2"
16	lncRNA	exon	57805670	57806930	.	+	.	gene_id "lnc-bbug07815"	transcript_id "lnc-bbut00018298"	exon_number "3"
16	lncRNA	exon	57877297	57878750	.	+	.	gene_id "lnc-bbug07816"	transcript_id "lnc-bbut00018299"	exon_number "1"
16	lncRNA	exon	57878979	57879023	.	+	.	gene_id "lnc-bbug07816"	transcript_id "lnc-bbut00018299"	exon_number "2"
16	lncRNA	exon	57880422	57880487	.	+	.	gene_id "lnc-bbug07816"	transcript_id "lnc-bbut00018299"	exon_number "3"
16	lncRNA	exon	57890007	57890497	.	+	.	gene_id "lnc-bbug07816"	transcript_id "lnc-bbut00018299"	exon_number "4"
16	lncRNA	exon	57890589	57890827	.	+	.	gene_id "lnc-bbug07816"	transcript_id "lnc-bbut00018299"	exon_number "5"
16	lncRNA	exon	57891662	57891917	.	+	.	gene_id "lnc-bbug07816"	transcript_id "lnc-bbut00018299"	exon_number "6"
16	lncRNA	exon	58881401	58889866	.	+	.	gene_id "lnc-bbug07819"	transcript_id "lnc-bbut00018307"	exon_number "1"
16	lncRNA	exon	58894858	58897697	.	+	.	gene_id "lnc-bbug07819"	transcript_id "lnc-bbut00018307"	exon_number "2"
16	lncRNA	exon	59892732	59893609	.	+	.	gene_id "lnc-bbug07821"	transcript_id "lnc-bbut00018310"	exon_number "1"
16	lncRNA	exon	60013892	60014538	.	+	.	gene_id "lnc-bbug07822"	transcript_id "lnc-bbut00018311"	exon_number "1"
16	lncRNA	exon	60033101	60033436	.	+	.	gene_id "lnc-bbug07823"	transcript_id "lnc-bbut00018312"	exon_number "1"
16	lncRNA	exon	60035721	60035754	.	+	.	gene_id "lnc-bbug07823"	transcript_id "lnc-bbut00018312"	exon_number "2"
16	lncRNA	exon	60036813	60037158	.	+	.	gene_id "lnc-bbug07823"	transcript_id "lnc-bbut00018312"	exon_number "3"
16	lncRNA	exon	60263894	60264017	.	+	.	gene_id "lnc-bbug07824"	transcript_id "lnc-bbut00018313"	exon_number "1"
16	lncRNA	exon	60264834	60264965	.	+	.	gene_id "lnc-bbug07824"	transcript_id "lnc-bbut00018313"	exon_number "2"
16	lncRNA	exon	60265751	60265859	.	+	.	gene_id "lnc-bbug07824"	transcript_id "lnc-bbut00018313"	exon_number "3"
16	lncRNA	exon	60268206	60268431	.	+	.	gene_id "lnc-bbug07824"	transcript_id "lnc-bbut00018313"	exon_number "4"
16	lncRNA	exon	60264552	60264725	.	+	.	gene_id "lnc-bbug07824"	transcript_id "lnc-bbut00018314"	exon_number "1"
16	lncRNA	exon	60264834	60264965	.	+	.	gene_id "lnc-bbug07824"	transcript_id "lnc-bbut00018314"	exon_number "2"
16	lncRNA	exon	60265751	60265859	.	+	.	gene_id "lnc-bbug07824"	transcript_id "lnc-bbut00018314"	exon_number "3"
16	lncRNA	exon	60268206	60268431	.	+	.	gene_id "lnc-bbug07824"	transcript_id "lnc-bbut00018314"	exon_number "4"
16	lncRNA	exon	62564970	62565099	.	+	.	gene_id "lnc-bbug07833"	transcript_id "lnc-bbut00018337"	exon_number "1"
16	lncRNA	exon	62566590	62566986	.	+	.	gene_id "lnc-bbug07833"	transcript_id "lnc-bbut00018337"	exon_number "2"
16	lncRNA	exon	62599010	62599316	.	+	.	gene_id "lnc-bbug07835"	transcript_id "lnc-bbut00018339"	exon_number "1"
16	lncRNA	exon	63393072	63394136	.	+	.	gene_id "lnc-bbug07842"	transcript_id "lnc-bbut00018349"	exon_number "1"
16	lncRNA	exon	67353921	67354117	.	+	.	gene_id "lnc-bbug07850"	transcript_id "lnc-bbut00018384"	exon_number "1"
16	lncRNA	exon	67358020	67358149	.	+	.	gene_id "lnc-bbug07850"	transcript_id "lnc-bbut00018384"	exon_number "2"
16	lncRNA	exon	67359130	67359352	.	+	.	gene_id "lnc-bbug07850"	transcript_id "lnc-bbut00018384"	exon_number "3"
16	lncRNA	exon	67731787	67731903	.	+	.	gene_id "lnc-bbug07852"	transcript_id "lnc-bbut00018386"	exon_number "1"
16	lncRNA	exon	67735789	67736381	.	+	.	gene_id "lnc-bbug07852"	transcript_id "lnc-bbut00018386"	exon_number "2"
16	lncRNA	exon	67882636	67883593	.	+	.	gene_id "lnc-bbug07853"	transcript_id "lnc-bbut00018387"	exon_number "1"
16	lncRNA	exon	68916367	68916591	.	+	.	gene_id "lnc-bbug07856"	transcript_id "lnc-bbut00018390"	exon_number "1"
16	lncRNA	exon	68922511	68922615	.	+	.	gene_id "lnc-bbug07856"	transcript_id "lnc-bbut00018390"	exon_number "2"
16	lncRNA	exon	68936370	68936495	.	+	.	gene_id "lnc-bbug07856"	transcript_id "lnc-bbut00018390"	exon_number "3"
16	lncRNA	exon	68949728	68950660	.	+	.	gene_id "lnc-bbug07856"	transcript_id "lnc-bbut00018390"	exon_number "4"
16	lncRNA	exon	70022510	70023565	.	+	.	gene_id "lnc-bbug07864"	transcript_id "lnc-bbut00018408"	exon_number "1"
16	lncRNA	exon	70024649	70024738	.	+	.	gene_id "lnc-bbug07864"	transcript_id "lnc-bbut00018408"	exon_number "2"
16	lncRNA	exon	70087395	70088416	.	+	.	gene_id "lnc-bbug07864"	transcript_id "lnc-bbut00018408"	exon_number "3"
16	lncRNA	exon	70614903	70615444	.	+	.	gene_id "lnc-bbug07866"	transcript_id "lnc-bbut00018412"	exon_number "1"
16	lncRNA	exon	70615548	70615729	.	+	.	gene_id "lnc-bbug07866"	transcript_id "lnc-bbut00018412"	exon_number "2"
16	lncRNA	exon	70616816	70616948	.	+	.	gene_id "lnc-bbug07866"	transcript_id "lnc-bbut00018412"	exon_number "3"

16	lncRNA	exon	71125403	71126028	.	+	.	gene_id "lnc-bbug07868"	transcript_id "lnc-bbut00018414"	exon_number "1"
16	lncRNA	exon	71147653	71147820	.	+	.	gene_id "lnc-bbug07868"	transcript_id "lnc-bbut00018414"	exon_number "2"
16	lncRNA	exon	71171245	71171309	.	+	.	gene_id "lnc-bbug07868"	transcript_id "lnc-bbut00018414"	exon_number "3"
16	lncRNA	exon	71172974	71173189	.	+	.	gene_id "lnc-bbug07868"	transcript_id "lnc-bbut00018414"	exon_number "4"
16	lncRNA	exon	71131654	71132247	.	+	.	gene_id "lnc-bbug07868"	transcript_id "lnc-bbut00018415"	exon_number "1"
16	lncRNA	exon	71147653	71147820	.	+	.	gene_id "lnc-bbug07868"	transcript_id "lnc-bbut00018415"	exon_number "2"
16	lncRNA	exon	71171245	71171309	.	+	.	gene_id "lnc-bbug07868"	transcript_id "lnc-bbut00018415"	exon_number "3"
16	lncRNA	exon	71172974	71173189	.	+	.	gene_id "lnc-bbug07868"	transcript_id "lnc-bbut00018415"	exon_number "4"
16	lncRNA	exon	71146594	71146695	.	+	.	gene_id "lnc-bbug07868"	transcript_id "lnc-bbut00018416"	exon_number "1"
16	lncRNA	exon	71147653	71147820	.	+	.	gene_id "lnc-bbug07868"	transcript_id "lnc-bbut00018416"	exon_number "2"
16	lncRNA	exon	71171245	71171309	.	+	.	gene_id "lnc-bbug07868"	transcript_id "lnc-bbut00018416"	exon_number "3"
16	lncRNA	exon	71172974	71173189	.	+	.	gene_id "lnc-bbug07868"	transcript_id "lnc-bbut00018416"	exon_number "4"
16	lncRNA	exon	72525911	72526383	.	+	.	gene_id "lnc-bbug07869"	transcript_id "lnc-bbut00018417"	exon_number "1"
16	lncRNA	exon	74404015	74404291	.	+	.	gene_id "lnc-bbug07870"	transcript_id "lnc-bbut00018418"	exon_number "1"
16	lncRNA	exon	74405394	74405656	.	+	.	gene_id "lnc-bbug07870"	transcript_id "lnc-bbut00018418"	exon_number "2"
16	lncRNA	exon	74405691	74405986	.	+	.	gene_id "lnc-bbug07870"	transcript_id "lnc-bbut00018418"	exon_number "3"
16	lncRNA	exon	77527421	77527565	.	+	.	gene_id "lnc-bbug07872"	transcript_id "lnc-bbut00018421"	exon_number "1"
16	lncRNA	exon	77527725	77527809	.	+	.	gene_id "lnc-bbug07872"	transcript_id "lnc-bbut00018421"	exon_number "2"
16	lncRNA	exon	77544053	77544236	.	+	.	gene_id "lnc-bbug07872"	transcript_id "lnc-bbut00018421"	exon_number "3"
16	lncRNA	exon	77561308	77561629	.	+	.	gene_id "lnc-bbug07872"	transcript_id "lnc-bbut00018421"	exon_number "4"
16	lncRNA	exon	77527421	77527538	.	+	.	gene_id "lnc-bbug07872"	transcript_id "lnc-bbut00018420"	exon_number "1"
16	lncRNA	exon	77527725	77527809	.	+	.	gene_id "lnc-bbug07872"	transcript_id "lnc-bbut00018420"	exon_number "2"
16	lncRNA	exon	77544053	77544236	.	+	.	gene_id "lnc-bbug07872"	transcript_id "lnc-bbut00018420"	exon_number "3"
16	lncRNA	exon	77561308	77561629	.	+	.	gene_id "lnc-bbug07872"	transcript_id "lnc-bbut00018420"	exon_number "4"
16	lncRNA	exon	78551533	78552736	.	+	.	gene_id "lnc-bbug07876"	transcript_id "lnc-bbut00018444"	exon_number "1"
16	lncRNA	exon	79134376	79134533	.	+	.	gene_id "lnc-bbug07879"	transcript_id "lnc-bbut00018453"	exon_number "1"
16	lncRNA	exon	79135252	79135356	.	+	.	gene_id "lnc-bbug07879"	transcript_id "lnc-bbut00018453"	exon_number "2"
16	lncRNA	exon	79136426	79136826	.	+	.	gene_id "lnc-bbug07879"	transcript_id "lnc-bbut00018453"	exon_number "3"
16	lncRNA	exon	79477286	79479350	.	+	.	gene_id "lnc-bbug07881"	transcript_id "lnc-bbut00018455"	exon_number "1"
16	lncRNA	exon	80370409	80371352	.	+	.	gene_id "lnc-bbug07883"	transcript_id "lnc-bbut00018457"	exon_number "1"
16	lncRNA	exon	80777053	80777206	.	+	.	gene_id "lnc-bbug07885"	transcript_id "lnc-bbut00018459"	exon_number "1"
16	lncRNA	exon	80782217	80782286	.	+	.	gene_id "lnc-bbug07885"	transcript_id "lnc-bbut00018459"	exon_number "2"
16	lncRNA	exon	80784375	80784422	.	+	.	gene_id "lnc-bbug07885"	transcript_id "lnc-bbut00018459"	exon_number "3"
16	lncRNA	exon	80784839	80785586	.	+	.	gene_id "lnc-bbug07885"	transcript_id "lnc-bbut00018459"	exon_number "4"
16	lncRNA	exon	80777970	80778023	.	+	.	gene_id "lnc-bbug07885"	transcript_id "lnc-bbut00018460"	exon_number "1"
16	lncRNA	exon	80782217	80782286	.	+	.	gene_id "lnc-bbug07885"	transcript_id "lnc-bbut00018460"	exon_number "2"
16	lncRNA	exon	80784375	80784422	.	+	.	gene_id "lnc-bbug07885"	transcript_id "lnc-bbut00018460"	exon_number "3"
16	lncRNA	exon	80784839	80785586	.	+	.	gene_id "lnc-bbug07885"	transcript_id "lnc-bbut00018460"	exon_number "4"
16	lncRNA	exon	80778764	80780537	.	+	.	gene_id "lnc-bbug07885"	transcript_id "lnc-bbut00018461"	exon_number "1"
16	lncRNA	exon	80782217	80782286	.	+	.	gene_id "lnc-bbug07885"	transcript_id "lnc-bbut00018461"	exon_number "2"
16	lncRNA	exon	80784375	80784422	.	+	.	gene_id "lnc-bbug07885"	transcript_id "lnc-bbut00018461"	exon_number "3"
16	lncRNA	exon	80784839	80785586	.	+	.	gene_id "lnc-bbug07885"	transcript_id "lnc-bbut00018461"	exon_number "4"
16	lncRNA	exon	83127192	83127227	.	+	.	gene_id "lnc-bbug07889"	transcript_id "lnc-bbut00018472"	exon_number "1"
16	lncRNA	exon	83127553	83128582	.	+	.	gene_id "lnc-bbug07889"	transcript_id "lnc-bbut00018472"	exon_number "2"
16	lncRNA	exon	83129114	83129434	.	+	.	gene_id "lnc-bbug07889"	transcript_id "lnc-bbut00018472"	exon_number "3"
16	lncRNA	exon	84011982	84012888	.	+	.	gene_id "lnc-bbug07898"	transcript_id "lnc-bbut00018485"	exon_number "1"
16	lncRNA	exon	507249	507459	.	-	.	gene_id "lnc-bbug07910"	transcript_id "lnc-bbut00018511"	exon_number "1"
16	lncRNA	exon	510780	510897	.	-	.	gene_id "lnc-bbug07910"	transcript_id "lnc-bbut00018511"	exon_number "2"
16	lncRNA	exon	520972	521028	.	-	.	gene_id "lnc-bbug07910"	transcript_id "lnc-bbut00018511"	exon_number "3"
16	lncRNA	exon	524759	524902	.	-	.	gene_id "lnc-bbug07910"	transcript_id "lnc-bbut00018511"	exon_number "4"
16	lncRNA	exon	525987	526084	.	-	.	gene_id "lnc-bbug07910"	transcript_id "lnc-bbut00018511"	exon_number "5"
16	lncRNA	exon	528652	528772	.	-	.	gene_id "lnc-bbug07910"	transcript_id "lnc-bbut00018511"	exon_number "6"

16	lncRNA	exon	529908	530012	.	-	.	gene_id "lnc-bbug07910"	transcript_id "lnc-bbut00018511"	exon_number "7"
16	lncRNA	exon	515307	515421	.	-	.	gene_id "lnc-bbug07910"	transcript_id "lnc-bbut00018512"	exon_number "1"
16	lncRNA	exon	520972	521028	.	-	.	gene_id "lnc-bbug07910"	transcript_id "lnc-bbut00018512"	exon_number "2"
16	lncRNA	exon	524759	524902	.	-	.	gene_id "lnc-bbug07910"	transcript_id "lnc-bbut00018512"	exon_number "3"
16	lncRNA	exon	525987	526084	.	-	.	gene_id "lnc-bbug07910"	transcript_id "lnc-bbut00018512"	exon_number "4"
16	lncRNA	exon	528652	528772	.	-	.	gene_id "lnc-bbug07910"	transcript_id "lnc-bbut00018512"	exon_number "5"
16	lncRNA	exon	529908	530012	.	-	.	gene_id "lnc-bbug07910"	transcript_id "lnc-bbut00018512"	exon_number "6"
16	lncRNA	exon	768873	769084	.	-	.	gene_id "lnc-bbug07917"	transcript_id "lnc-bbut00018523"	exon_number "1"
16	lncRNA	exon	769700	769816	.	-	.	gene_id "lnc-bbug07917"	transcript_id "lnc-bbut00018523"	exon_number "2"
16	lncRNA	exon	770976	771024	.	-	.	gene_id "lnc-bbug07917"	transcript_id "lnc-bbut00018523"	exon_number "3"
16	lncRNA	exon	772489	772620	.	-	.	gene_id "lnc-bbug07917"	transcript_id "lnc-bbut00018523"	exon_number "4"
16	lncRNA	exon	812092	812199	.	-	.	gene_id "lnc-bbug07920"	transcript_id "lnc-bbut00018527"	exon_number "1"
16	lncRNA	exon	813481	813656	.	-	.	gene_id "lnc-bbug07920"	transcript_id "lnc-bbut00018527"	exon_number "2"
16	lncRNA	exon	818544	818559	.	-	.	gene_id "lnc-bbug07920"	transcript_id "lnc-bbut00018527"	exon_number "3"
16	lncRNA	exon	991657	992023	.	-	.	gene_id "lnc-bbug07923"	transcript_id "lnc-bbut00018530"	exon_number "1"
16	lncRNA	exon	992219	992390	.	-	.	gene_id "lnc-bbug07923"	transcript_id "lnc-bbut00018530"	exon_number "2"
16	lncRNA	exon	996029	996352	.	-	.	gene_id "lnc-bbug07923"	transcript_id "lnc-bbut00018530"	exon_number "3"
16	lncRNA	exon	1653004	1653053	.	-	.	gene_id "lnc-bbug07938"	transcript_id "lnc-bbut00018549"	exon_number "1"
16	lncRNA	exon	1656225	1656328	.	-	.	gene_id "lnc-bbug07938"	transcript_id "lnc-bbut00018549"	exon_number "2"
16	lncRNA	exon	1658501	1659431	.	-	.	gene_id "lnc-bbug07938"	transcript_id "lnc-bbut00018549"	exon_number "3"
16	lncRNA	exon	1949329	1950717	.	-	.	gene_id "lnc-bbug07941"	transcript_id "lnc-bbut00018552"	exon_number "1"
16	lncRNA	exon	1950947	1951584	.	-	.	gene_id "lnc-bbug07941"	transcript_id "lnc-bbut00018552"	exon_number "2"
16	lncRNA	exon	2936777	2936961	.	-	.	gene_id "lnc-bbug07955"	transcript_id "lnc-bbut00018582"	exon_number "1"
16	lncRNA	exon	2937683	2937812	.	-	.	gene_id "lnc-bbug07955"	transcript_id "lnc-bbut00018582"	exon_number "2"
16	lncRNA	exon	2937936	2938081	.	-	.	gene_id "lnc-bbug07955"	transcript_id "lnc-bbut00018582"	exon_number "3"
16	lncRNA	exon	2938159	2938430	.	-	.	gene_id "lnc-bbug07955"	transcript_id "lnc-bbut00018582"	exon_number "4"
16	lncRNA	exon	2936777	2936961	.	-	.	gene_id "lnc-bbug07955"	transcript_id "lnc-bbut00018583"	exon_number "1"
16	lncRNA	exon	2937583	2937812	.	-	.	gene_id "lnc-bbug07955"	transcript_id "lnc-bbut00018583"	exon_number "2"
16	lncRNA	exon	2937936	2938081	.	-	.	gene_id "lnc-bbug07955"	transcript_id "lnc-bbut00018583"	exon_number "3"
16	lncRNA	exon	2938159	2938435	.	-	.	gene_id "lnc-bbug07955"	transcript_id "lnc-bbut00018583"	exon_number "4"
16	lncRNA	exon	3038293	3039372	.	-	.	gene_id "lnc-bbug07960"	transcript_id "lnc-bbut00018592"	exon_number "1"
16	lncRNA	exon	3039869	3042089	.	-	.	gene_id "lnc-bbug07960"	transcript_id "lnc-bbut00018592"	exon_number "2"
16	lncRNA	exon	3092915	3097280	.	-	.	gene_id "lnc-bbug07962"	transcript_id "lnc-bbut00018597"	exon_number "1"
16	lncRNA	exon	3104785	3106245	.	-	.	gene_id "lnc-bbug07963"	transcript_id "lnc-bbut00018598"	exon_number "1"
16	lncRNA	exon	3106444	3106889	.	-	.	gene_id "lnc-bbug07963"	transcript_id "lnc-bbut00018598"	exon_number "2"
16	lncRNA	exon	3118707	3118970	.	-	.	gene_id "lnc-bbug07963"	transcript_id "lnc-bbut00018598"	exon_number "3"
16	lncRNA	exon	3104785	3106245	.	-	.	gene_id "lnc-bbug07963"	transcript_id "lnc-bbut00018599"	exon_number "1"
16	lncRNA	exon	3106444	3106889	.	-	.	gene_id "lnc-bbug07963"	transcript_id "lnc-bbut00018599"	exon_number "2"
16	lncRNA	exon	3107062	3107293	.	-	.	gene_id "lnc-bbug07963"	transcript_id "lnc-bbut00018599"	exon_number "3"
16	lncRNA	exon	3118707	3118974	.	-	.	gene_id "lnc-bbug07963"	transcript_id "lnc-bbut00018599"	exon_number "4"
16	lncRNA	exon	4335497	4336415	.	-	.	gene_id "lnc-bbug07990"	transcript_id "lnc-bbut00018629"	exon_number "1"
16	lncRNA	exon	4406454	4406573	.	-	.	gene_id "lnc-bbug07996"	transcript_id "lnc-bbut00018635"	exon_number "1"
16	lncRNA	exon	4409925	4410577	.	-	.	gene_id "lnc-bbug07996"	transcript_id "lnc-bbut00018635"	exon_number "2"
16	lncRNA	exon	4411555	4411790	.	-	.	gene_id "lnc-bbug07996"	transcript_id "lnc-bbut00018635"	exon_number "3"
16	lncRNA	exon	4412372	4412390	.	-	.	gene_id "lnc-bbug07996"	transcript_id "lnc-bbut00018635"	exon_number "4"
16	lncRNA	exon	5099799	5099828	.	-	.	gene_id "lnc-bbug08025"	transcript_id "lnc-bbut00018664"	exon_number "1"
16	lncRNA	exon	5103768	5104486	.	-	.	gene_id "lnc-bbug08025"	transcript_id "lnc-bbut00018664"	exon_number "2"
16	lncRNA	exon	5659876	5660559	.	-	.	gene_id "lnc-bbug08050"	transcript_id "lnc-bbut00018689"	exon_number "1"
16	lncRNA	exon	6485151	6485999	.	-	.	gene_id "lnc-bbug08086"	transcript_id "lnc-bbut00018725"	exon_number "1"
16	lncRNA	exon	6547306	6548226	.	-	.	gene_id "lnc-bbug08092"	transcript_id "lnc-bbut00018731"	exon_number "1"
16	lncRNA	exon	6838219	6842371	.	-	.	gene_id "lnc-bbug08108"	transcript_id "lnc-bbut00018747"	exon_number "1"
16	lncRNA	exon	6846706	6850266	.	-	.	gene_id "lnc-bbug08108"	transcript_id "lnc-bbut00018747"	exon_number "2"

16	lncRNA	exon	7123806	7127851	.	-	.	gene_id "lnc-bbug08110"	transcript_id "lnc-bbut00018749"	exon_number "1"
16	lncRNA	exon	7157616	7157619	.	-	.	gene_id "lnc-bbug08111"	transcript_id "lnc-bbut00018750"	exon_number "1"
16	lncRNA	exon	7157786	7158126	.	-	.	gene_id "lnc-bbug08111"	transcript_id "lnc-bbut00018750"	exon_number "2"
16	lncRNA	exon	8470140	8470486	.	-	.	gene_id "lnc-bbug08125"	transcript_id "lnc-bbut00018805"	exon_number "1"
16	lncRNA	exon	8470944	8471323	.	-	.	gene_id "lnc-bbug08125"	transcript_id "lnc-bbut00018805"	exon_number "2"
16	lncRNA	exon	8942703	8943138	.	-	.	gene_id "lnc-bbug08129"	transcript_id "lnc-bbut00018818"	exon_number "1"
16	lncRNA	exon	8943934	8944027	.	-	.	gene_id "lnc-bbug08129"	transcript_id "lnc-bbut00018818"	exon_number "2"
16	lncRNA	exon	8944727	8944865	.	-	.	gene_id "lnc-bbug08129"	transcript_id "lnc-bbut00018818"	exon_number "3"
16	lncRNA	exon	9720091	9720757	.	-	.	gene_id "lnc-bbug08132"	transcript_id "lnc-bbut00018824"	exon_number "1"
16	lncRNA	exon	9731354	9731557	.	-	.	gene_id "lnc-bbug08132"	transcript_id "lnc-bbut00018824"	exon_number "2"
16	lncRNA	exon	9981819	9982402	.	-	.	gene_id "lnc-bbug08134"	transcript_id "lnc-bbut00018832"	exon_number "1"
16	lncRNA	exon	9983221	9983654	.	-	.	gene_id "lnc-bbug08134"	transcript_id "lnc-bbut00018832"	exon_number "2"
16	lncRNA	exon	9987794	9988651	.	-	.	gene_id "lnc-bbug08134"	transcript_id "lnc-bbut00018832"	exon_number "3"
16	lncRNA	exon	11012721	11013045	.	-	.	gene_id "lnc-bbug08143"	transcript_id "lnc-bbut00018855"	exon_number "1"
16	lncRNA	exon	11056811	11056925	.	-	.	gene_id "lnc-bbug08143"	transcript_id "lnc-bbut00018855"	exon_number "2"
16	lncRNA	exon	11064989	11065002	.	-	.	gene_id "lnc-bbug08143"	transcript_id "lnc-bbut00018855"	exon_number "3"
16	lncRNA	exon	11088692	11089032	.	-	.	gene_id "lnc-bbug08143"	transcript_id "lnc-bbut00018855"	exon_number "4"
16	lncRNA	exon	11071512	11072138	.	-	.	gene_id "lnc-bbug08144"	transcript_id "lnc-bbut00018856"	exon_number "1"
16	lncRNA	exon	11072331	11072863	.	-	.	gene_id "lnc-bbug08144"	transcript_id "lnc-bbut00018856"	exon_number "2"
16	lncRNA	exon	13723111	13723491	.	-	.	gene_id "lnc-bbug08147"	transcript_id "lnc-bbut00018865"	exon_number "1"
16	lncRNA	exon	13724371	13724460	.	-	.	gene_id "lnc-bbug08147"	transcript_id "lnc-bbut00018865"	exon_number "2"
16	lncRNA	exon	13730062	13731683	.	-	.	gene_id "lnc-bbug08147"	transcript_id "lnc-bbut00018865"	exon_number "3"
16	lncRNA	exon	16686739	16687155	.	-	.	gene_id "lnc-bbug08148"	transcript_id "lnc-bbut00018866"	exon_number "1"
16	lncRNA	exon	16700680	16701144	.	-	.	gene_id "lnc-bbug08148"	transcript_id "lnc-bbut00018866"	exon_number "2"
16	lncRNA	exon	16695563	16695598	.	-	.	gene_id "lnc-bbug08148"	transcript_id "lnc-bbut00018867"	exon_number "1"
16	lncRNA	exon	16700680	16701069	.	-	.	gene_id "lnc-bbug08148"	transcript_id "lnc-bbut00018867"	exon_number "2"
16	lncRNA	exon	17896760	17902503	.	-	.	gene_id "lnc-bbug08151"	transcript_id "lnc-bbut00018875"	exon_number "1"
16	lncRNA	exon	17904174	17905511	.	-	.	gene_id "lnc-bbug08151"	transcript_id "lnc-bbut00018875"	exon_number "2"
16	lncRNA	exon	18048121	18049580	.	-	.	gene_id "lnc-bbug08153"	transcript_id "lnc-bbut00018887"	exon_number "1"
16	lncRNA	exon	18050968	18051054	.	-	.	gene_id "lnc-bbug08153"	transcript_id "lnc-bbut00018887"	exon_number "2"
16	lncRNA	exon	18066065	18066395	.	-	.	gene_id "lnc-bbug08153"	transcript_id "lnc-bbut00018887"	exon_number "3"
16	lncRNA	exon	18050149	18050197	.	-	.	gene_id "lnc-bbug08153"	transcript_id "lnc-bbut00018888"	exon_number "1"
16	lncRNA	exon	18050968	18051054	.	-	.	gene_id "lnc-bbug08153"	transcript_id "lnc-bbut00018888"	exon_number "2"
16	lncRNA	exon	18066065	18066394	.	-	.	gene_id "lnc-bbug08153"	transcript_id "lnc-bbut00018888"	exon_number "3"
16	lncRNA	exon	18946084	18946963	.	-	.	gene_id "lnc-bbug08157"	transcript_id "lnc-bbut00018900"	exon_number "1"
16	lncRNA	exon	18953149	18954928	.	-	.	gene_id "lnc-bbug08157"	transcript_id "lnc-bbut00018900"	exon_number "2"
16	lncRNA	exon	19496101	19496469	.	-	.	gene_id "lnc-bbug08160"	transcript_id "lnc-bbut00018920"	exon_number "1"
16	lncRNA	exon	19500463	19500602	.	-	.	gene_id "lnc-bbug08160"	transcript_id "lnc-bbut00018920"	exon_number "2"
16	lncRNA	exon	19909687	19909745	.	-	.	gene_id "lnc-bbug08163"	transcript_id "lnc-bbut00018924"	exon_number "1"
16	lncRNA	exon	19913336	19913502	.	-	.	gene_id "lnc-bbug08163"	transcript_id "lnc-bbut00018924"	exon_number "2"
16	lncRNA	exon	19913687	19914035	.	-	.	gene_id "lnc-bbug08163"	transcript_id "lnc-bbut00018924"	exon_number "3"
16	lncRNA	exon	21665862	21666135	.	-	.	gene_id "lnc-bbug08173"	transcript_id "lnc-bbut00018958"	exon_number "1"
16	lncRNA	exon	21687137	21687486	.	-	.	gene_id "lnc-bbug08173"	transcript_id "lnc-bbut00018958"	exon_number "2"
16	lncRNA	exon	22608955	22609014	.	-	.	gene_id "lnc-bbug08176"	transcript_id "lnc-bbut00018961"	exon_number "1"
16	lncRNA	exon	22621777	22621889	.	-	.	gene_id "lnc-bbug08176"	transcript_id "lnc-bbut00018961"	exon_number "2"
16	lncRNA	exon	22623135	22623190	.	-	.	gene_id "lnc-bbug08176"	transcript_id "lnc-bbut00018961"	exon_number "3"
16	lncRNA	exon	22627397	22628107	.	-	.	gene_id "lnc-bbug08176"	transcript_id "lnc-bbut00018961"	exon_number "4"
16	lncRNA	exon	22621520	22621889	.	-	.	gene_id "lnc-bbug08176"	transcript_id "lnc-bbut00018962"	exon_number "1"
16	lncRNA	exon	22623135	22623190	.	-	.	gene_id "lnc-bbug08176"	transcript_id "lnc-bbut00018962"	exon_number "2"
16	lncRNA	exon	22627397	22628107	.	-	.	gene_id "lnc-bbug08176"	transcript_id "lnc-bbut00018962"	exon_number "3"
16	lncRNA	exon	23255762	23255980	.	-	.	gene_id "lnc-bbug08178"	transcript_id "lnc-bbut00018965"	exon_number "1"
16	lncRNA	exon	23728557	23728952	.	-	.	gene_id "lnc-bbug08179"	transcript_id "lnc-bbut00018966"	exon_number "1"

16	lncRNA	exon	23739425	23739533	.	-	.	gene_id "lnc-bbug08179"	transcript_id "lnc-bbut00018966"	exon_number "2"
16	lncRNA	exon	23740087	23740384	.	-	.	gene_id "lnc-bbug08179"	transcript_id "lnc-bbut00018966"	exon_number "3"
16	lncRNA	exon	23862712	23864228	.	-	.	gene_id "lnc-bbug08181"	transcript_id "lnc-bbut00018969"	exon_number "1"
16	lncRNA	exon	23865792	23865988	.	-	.	gene_id "lnc-bbug08181"	transcript_id "lnc-bbut00018969"	exon_number "2"
16	lncRNA	exon	23866593	23866649	.	-	.	gene_id "lnc-bbug08181"	transcript_id "lnc-bbut00018969"	exon_number "3"
16	lncRNA	exon	27825829	27826723	.	-	.	gene_id "lnc-bbug08187"	transcript_id "lnc-bbut00018988"	exon_number "1"
16	lncRNA	exon	27842742	27842838	.	-	.	gene_id "lnc-bbug08187"	transcript_id "lnc-bbut00018988"	exon_number "2"
16	lncRNA	exon	27842933	27842990	.	-	.	gene_id "lnc-bbug08187"	transcript_id "lnc-bbut00018988"	exon_number "3"
16	lncRNA	exon	27853786	27854500	.	-	.	gene_id "lnc-bbug08187"	transcript_id "lnc-bbut00018988"	exon_number "4"
16	lncRNA	exon	28451845	28453805	.	-	.	gene_id "lnc-bbug08192"	transcript_id "lnc-bbut00019002"	exon_number "1"
16	lncRNA	exon	28454291	28454336	.	-	.	gene_id "lnc-bbug08192"	transcript_id "lnc-bbut00019002"	exon_number "2"
16	lncRNA	exon	28463577	28463681	.	-	.	gene_id "lnc-bbug08192"	transcript_id "lnc-bbut00019002"	exon_number "3"
16	lncRNA	exon	28468273	28468374	.	-	.	gene_id "lnc-bbug08192"	transcript_id "lnc-bbut00019002"	exon_number "4"
16	lncRNA	exon	28490595	28490653	.	-	.	gene_id "lnc-bbug08192"	transcript_id "lnc-bbut00019002"	exon_number "5"
16	lncRNA	exon	28507000	28507035	.	-	.	gene_id "lnc-bbug08192"	transcript_id "lnc-bbut00019002"	exon_number "6"
16	lncRNA	exon	28509815	28509896	.	-	.	gene_id "lnc-bbug08192"	transcript_id "lnc-bbut00019002"	exon_number "7"
16	lncRNA	exon	28512647	28512699	.	-	.	gene_id "lnc-bbug08192"	transcript_id "lnc-bbut00019002"	exon_number "8"
16	lncRNA	exon	28553608	28553718	.	-	.	gene_id "lnc-bbug08192"	transcript_id "lnc-bbut00019002"	exon_number "9"
16	lncRNA	exon	28807752	28807946	.	-	.	gene_id "lnc-bbug08194"	transcript_id "lnc-bbut00019006"	exon_number "1"
16	lncRNA	exon	28808029	28808332	.	-	.	gene_id "lnc-bbug08194"	transcript_id "lnc-bbut00019006"	exon_number "2"
16	lncRNA	exon	28896993	28897407	.	-	.	gene_id "lnc-bbug08196"	transcript_id "lnc-bbut00019008"	exon_number "1"
16	lncRNA	exon	28898559	28898601	.	-	.	gene_id "lnc-bbug08196"	transcript_id "lnc-bbut00019008"	exon_number "2"
16	lncRNA	exon	29054224	29062282	.	-	.	gene_id "lnc-bbug08198"	transcript_id "lnc-bbut00019026"	exon_number "1"
16	lncRNA	exon	29062745	29063731	.	-	.	gene_id "lnc-bbug08198"	transcript_id "lnc-bbut00019026"	exon_number "2"
16	lncRNA	exon	29879832	29880068	.	-	.	gene_id "lnc-bbug08207"	transcript_id "lnc-bbut00019039"	exon_number "1"
16	lncRNA	exon	29880390	29880590	.	-	.	gene_id "lnc-bbug08207"	transcript_id "lnc-bbut00019039"	exon_number "2"
16	lncRNA	exon	29883460	29883621	.	-	.	gene_id "lnc-bbug08207"	transcript_id "lnc-bbut00019039"	exon_number "3"
16	lncRNA	exon	29891483	29891573	.	-	.	gene_id "lnc-bbug08207"	transcript_id "lnc-bbut00019039"	exon_number "4"
16	lncRNA	exon	29926647	29927378	.	-	.	gene_id "lnc-bbug08209"	transcript_id "lnc-bbut00019041"	exon_number "1"
16	lncRNA	exon	30699970	30702208	.	-	.	gene_id "lnc-bbug08217"	transcript_id "lnc-bbut00019054"	exon_number "1"
16	lncRNA	exon	30706466	30706505	.	-	.	gene_id "lnc-bbug08217"	transcript_id "lnc-bbut00019054"	exon_number "2"
16	lncRNA	exon	31554402	31557080	.	-	.	gene_id "lnc-bbug08224"	transcript_id "lnc-bbut00019068"	exon_number "1"
16	lncRNA	exon	32087075	32092165	.	-	.	gene_id "lnc-bbug08229"	transcript_id "lnc-bbut00019096"	exon_number "1"
16	lncRNA	exon	32099515	32099790	.	-	.	gene_id "lnc-bbug08229"	transcript_id "lnc-bbut00019096"	exon_number "2"
16	lncRNA	exon	32108201	32108339	.	-	.	gene_id "lnc-bbug08229"	transcript_id "lnc-bbut00019096"	exon_number "3"
16	lncRNA	exon	32568683	32569024	.	-	.	gene_id "lnc-bbug08231"	transcript_id "lnc-bbut00019103"	exon_number "1"
16	lncRNA	exon	32572973	32573107	.	-	.	gene_id "lnc-bbug08231"	transcript_id "lnc-bbut00019103"	exon_number "2"
16	lncRNA	exon	32573981	32574198	.	-	.	gene_id "lnc-bbug08231"	transcript_id "lnc-bbut00019103"	exon_number "3"
16	lncRNA	exon	32961106	32963316	.	-	.	gene_id "lnc-bbug08232"	transcript_id "lnc-bbut00019104"	exon_number "1"
16	lncRNA	exon	32964850	32964966	.	-	.	gene_id "lnc-bbug08232"	transcript_id "lnc-bbut00019104"	exon_number "2"
16	lncRNA	exon	34709735	34710043	.	-	.	gene_id "lnc-bbug08280"	transcript_id "lnc-bbut00019186"	exon_number "1"
16	lncRNA	exon	35811317	35811625	.	-	.	gene_id "lnc-bbug08318"	transcript_id "lnc-bbut00019224"	exon_number "1"
16	lncRNA	exon	35812749	35812901	.	-	.	gene_id "lnc-bbug08318"	transcript_id "lnc-bbut00019224"	exon_number "2"
16	lncRNA	exon	35813124	35813588	.	-	.	gene_id "lnc-bbug08318"	transcript_id "lnc-bbut00019224"	exon_number "3"
16	lncRNA	exon	35829765	35830699	.	-	.	gene_id "lnc-bbug08320"	transcript_id "lnc-bbut00019226"	exon_number "1"
16	lncRNA	exon	35889132	35889385	.	-	.	gene_id "lnc-bbug08325"	transcript_id "lnc-bbut00019231"	exon_number "1"
16	lncRNA	exon	35889966	35890172	.	-	.	gene_id "lnc-bbug08325"	transcript_id "lnc-bbut00019231"	exon_number "2"
16	lncRNA	exon	35890301	35890402	.	-	.	gene_id "lnc-bbug08325"	transcript_id "lnc-bbut00019231"	exon_number "3"
16	lncRNA	exon	35912637	35913591	.	-	.	gene_id "lnc-bbug08327"	transcript_id "lnc-bbut00019233"	exon_number "1"
16	lncRNA	exon	36265026	36265762	.	-	.	gene_id "lnc-bbug08342"	transcript_id "lnc-bbut00019248"	exon_number "1"
16	lncRNA	exon	36267494	36267548	.	-	.	gene_id "lnc-bbug08342"	transcript_id "lnc-bbut00019248"	exon_number "2"
16	lncRNA	exon	36436801	36437111	.	-	.	gene_id "lnc-bbug08347"	transcript_id "lnc-bbut00019253"	exon_number "1"

16	lncRNA	exon	36438232	36438382	.	-	.	gene_id "lnc-bbug08347"	transcript_id "lnc-bbut00019253"	exon_number "2"
16	lncRNA	exon	36438605	36439068	.	-	.	gene_id "lnc-bbug08347"	transcript_id "lnc-bbut00019253"	exon_number "3"
16	lncRNA	exon	36483166	36483276	.	-	.	gene_id "lnc-bbug08351"	transcript_id "lnc-bbut00019257"	exon_number "1"
16	lncRNA	exon	36483380	36484245	.	-	.	gene_id "lnc-bbug08351"	transcript_id "lnc-bbut00019257"	exon_number "2"
16	lncRNA	exon	36485330	36485343	.	-	.	gene_id "lnc-bbug08351"	transcript_id "lnc-bbut00019257"	exon_number "3"
16	lncRNA	exon	36669573	36670524	.	-	.	gene_id "lnc-bbug08368"	transcript_id "lnc-bbut00019274"	exon_number "1"
16	lncRNA	exon	36750674	36751622	.	-	.	gene_id "lnc-bbug08370"	transcript_id "lnc-bbut00019276"	exon_number "1"
16	lncRNA	exon	37970569	37971567	.	-	.	gene_id "lnc-bbug08399"	transcript_id "lnc-bbut00019305"	exon_number "1"
16	lncRNA	exon	37972124	37972729	.	-	.	gene_id "lnc-bbug08399"	transcript_id "lnc-bbut00019305"	exon_number "2"
16	lncRNA	exon	37988196	37990175	.	-	.	gene_id "lnc-bbug08400"	transcript_id "lnc-bbut00019306"	exon_number "1"
16	lncRNA	exon	37990333	37990624	.	-	.	gene_id "lnc-bbug08400"	transcript_id "lnc-bbut00019306"	exon_number "2"
16	lncRNA	exon	38071017	38071921	.	-	.	gene_id "lnc-bbug08403"	transcript_id "lnc-bbut00019318"	exon_number "1"
16	lncRNA	exon	38073924	38073984	.	-	.	gene_id "lnc-bbug08403"	transcript_id "lnc-bbut00019318"	exon_number "2"
16	lncRNA	exon	38118948	38122667	.	-	.	gene_id "lnc-bbug08404"	transcript_id "lnc-bbut00019319"	exon_number "1"
16	lncRNA	exon	38124960	38124987	.	-	.	gene_id "lnc-bbug08404"	transcript_id "lnc-bbut00019319"	exon_number "2"
16	lncRNA	exon	38648818	38648852	.	-	.	gene_id "lnc-bbug08416"	transcript_id "lnc-bbut00019337"	exon_number "1"
16	lncRNA	exon	38664054	38664181	.	-	.	gene_id "lnc-bbug08416"	transcript_id "lnc-bbut00019337"	exon_number "2"
16	lncRNA	exon	38665823	38665896	.	-	.	gene_id "lnc-bbug08416"	transcript_id "lnc-bbut00019337"	exon_number "3"
16	lncRNA	exon	38726387	38726456	.	-	.	gene_id "lnc-bbug08416"	transcript_id "lnc-bbut00019337"	exon_number "4"
16	lncRNA	exon	38744411	38744538	.	-	.	gene_id "lnc-bbug08416"	transcript_id "lnc-bbut00019337"	exon_number "5"
16	lncRNA	exon	38746182	38746260	.	-	.	gene_id "lnc-bbug08416"	transcript_id "lnc-bbut00019337"	exon_number "6"
16	lncRNA	exon	39196686	39197528	.	-	.	gene_id "lnc-bbug08428"	transcript_id "lnc-bbut00019355"	exon_number "1"
16	lncRNA	exon	40209585	40211181	.	-	.	gene_id "lnc-bbug08437"	transcript_id "lnc-bbut00019365"	exon_number "1"
16	lncRNA	exon	40220121	40220292	.	-	.	gene_id "lnc-bbug08437"	transcript_id "lnc-bbut00019365"	exon_number "2"
16	lncRNA	exon	40220551	40220596	.	-	.	gene_id "lnc-bbug08437"	transcript_id "lnc-bbut00019365"	exon_number "3"
16	lncRNA	exon	40576334	40576717	.	-	.	gene_id "lnc-bbug08446"	transcript_id "lnc-bbut00019377"	exon_number "1"
16	lncRNA	exon	40578182	40578307	.	-	.	gene_id "lnc-bbug08446"	transcript_id "lnc-bbut00019377"	exon_number "2"
16	lncRNA	exon	40580833	40581046	.	-	.	gene_id "lnc-bbug08446"	transcript_id "lnc-bbut00019377"	exon_number "3"
16	lncRNA	exon	40584640	40592534	.	-	.	gene_id "lnc-bbug08446"	transcript_id "lnc-bbut00019377"	exon_number "4"
16	lncRNA	exon	40997108	40997240	.	-	.	gene_id "lnc-bbug08449"	transcript_id "lnc-bbut00019389"	exon_number "1"
16	lncRNA	exon	41002068	41003221	.	-	.	gene_id "lnc-bbug08449"	transcript_id "lnc-bbut00019389"	exon_number "2"
16	lncRNA	exon	41009743	41012381	.	-	.	gene_id "lnc-bbug08449"	transcript_id "lnc-bbut00019389"	exon_number "3"
16	lncRNA	exon	41000212	41002189	.	-	.	gene_id "lnc-bbug08449"	transcript_id "lnc-bbut00019390"	exon_number "1"
16	lncRNA	exon	41003103	41003221	.	-	.	gene_id "lnc-bbug08449"	transcript_id "lnc-bbut00019390"	exon_number "2"
16	lncRNA	exon	41009743	41012381	.	-	.	gene_id "lnc-bbug08449"	transcript_id "lnc-bbut00019390"	exon_number "3"
16	lncRNA	exon	41209048	41209096	.	-	.	gene_id "lnc-bbug08451"	transcript_id "lnc-bbut00019395"	exon_number "1"
16	lncRNA	exon	41209995	41210065	.	-	.	gene_id "lnc-bbug08451"	transcript_id "lnc-bbut00019395"	exon_number "2"
16	lncRNA	exon	41211570	41213109	.	-	.	gene_id "lnc-bbug08451"	transcript_id "lnc-bbut00019395"	exon_number "3"
16	lncRNA	exon	41246681	41247973	.	-	.	gene_id "lnc-bbug08455"	transcript_id "lnc-bbut00019399"	exon_number "1"
16	lncRNA	exon	41252369	41252503	.	-	.	gene_id "lnc-bbug08455"	transcript_id "lnc-bbut00019399"	exon_number "2"
16	lncRNA	exon	41254661	41254769	.	-	.	gene_id "lnc-bbug08455"	transcript_id "lnc-bbut00019399"	exon_number "3"
16	lncRNA	exon	41260842	41260933	.	-	.	gene_id "lnc-bbug08455"	transcript_id "lnc-bbut00019399"	exon_number "4"
16	lncRNA	exon	41261149	41261254	.	-	.	gene_id "lnc-bbug08455"	transcript_id "lnc-bbut00019399"	exon_number "5"
16	lncRNA	exon	41264395	41264532	.	-	.	gene_id "lnc-bbug08455"	transcript_id "lnc-bbut00019399"	exon_number "6"
16	lncRNA	exon	41282305	41282535	.	-	.	gene_id "lnc-bbug08457"	transcript_id "lnc-bbut00019403"	exon_number "1"
16	lncRNA	exon	41502077	41502971	.	-	.	gene_id "lnc-bbug08460"	transcript_id "lnc-bbut00019417"	exon_number "1"
16	lncRNA	exon	41789223	41789798	.	-	.	gene_id "lnc-bbug08462"	transcript_id "lnc-bbut00019419"	exon_number "1"
16	lncRNA	exon	41796071	41798153	.	-	.	gene_id "lnc-bbug08462"	transcript_id "lnc-bbut00019419"	exon_number "2"
16	lncRNA	exon	45073104	45073333	.	-	.	gene_id "lnc-bbug08473"	transcript_id "lnc-bbut00019447"	exon_number "1"
16	lncRNA	exon	45075218	45075369	.	-	.	gene_id "lnc-bbug08473"	transcript_id "lnc-bbut00019447"	exon_number "2"
16	lncRNA	exon	45713887	45716246	.	-	.	gene_id "lnc-bbug08475"	transcript_id "lnc-bbut00019460"	exon_number "1"
16	lncRNA	exon	45716366	45716456	.	-	.	gene_id "lnc-bbug08475"	transcript_id "lnc-bbut00019460"	exon_number "2"

16	lncRNA	exon	45718147	45718272	.	-	.	gene_id "lnc-bbug08475"	transcript_id "lnc-bbut00019460"	exon_number "3"
16	lncRNA	exon	45858123	45858760	.	-	.	gene_id "lnc-bbug08477"	transcript_id "lnc-bbut00019463"	exon_number "1"
16	lncRNA	exon	45861284	45861329	.	-	.	gene_id "lnc-bbug08477"	transcript_id "lnc-bbut00019463"	exon_number "2"
16	lncRNA	exon	46692695	46693424	.	-	.	gene_id "lnc-bbug08481"	transcript_id "lnc-bbut00019469"	exon_number "1"
16	lncRNA	exon	46694451	46694539	.	-	.	gene_id "lnc-bbug08481"	transcript_id "lnc-bbut00019469"	exon_number "2"
16	lncRNA	exon	46725447	46725557	.	-	.	gene_id "lnc-bbug08481"	transcript_id "lnc-bbut00019469"	exon_number "3"
16	lncRNA	exon	46730962	46731258	.	-	.	gene_id "lnc-bbug08481"	transcript_id "lnc-bbut00019469"	exon_number "4"
16	lncRNA	exon	46693927	46694539	.	-	.	gene_id "lnc-bbug08481"	transcript_id "lnc-bbut00019470"	exon_number "1"
16	lncRNA	exon	46725447	46725557	.	-	.	gene_id "lnc-bbug08481"	transcript_id "lnc-bbut00019470"	exon_number "2"
16	lncRNA	exon	46730962	46731254	.	-	.	gene_id "lnc-bbug08481"	transcript_id "lnc-bbut00019470"	exon_number "3"
16	lncRNA	exon	47181973	47182734	.	-	.	gene_id "lnc-bbug08485"	transcript_id "lnc-bbut00019477"	exon_number "1"
16	lncRNA	exon	47183618	47183766	.	-	.	gene_id "lnc-bbug08485"	transcript_id "lnc-bbut00019477"	exon_number "2"
16	lncRNA	exon	47191302	47191992	.	-	.	gene_id "lnc-bbug08485"	transcript_id "lnc-bbut00019477"	exon_number "3"
16	lncRNA	exon	49700596	49701164	.	-	.	gene_id "lnc-bbug08493"	transcript_id "lnc-bbut00019506"	exon_number "1"
16	lncRNA	exon	49701673	49701744	.	-	.	gene_id "lnc-bbug08493"	transcript_id "lnc-bbut00019506"	exon_number "2"
16	lncRNA	exon	49704295	49704404	.	-	.	gene_id "lnc-bbug08493"	transcript_id "lnc-bbut00019506"	exon_number "3"
16	lncRNA	exon	50579832	50580303	.	-	.	gene_id "lnc-bbug08500"	transcript_id "lnc-bbut00019517"	exon_number "1"
16	lncRNA	exon	50580528	50580629	.	-	.	gene_id "lnc-bbug08500"	transcript_id "lnc-bbut00019517"	exon_number "2"
16	lncRNA	exon	50584240	50584680	.	-	.	gene_id "lnc-bbug08501"	transcript_id "lnc-bbut00019518"	exon_number "1"
16	lncRNA	exon	50590459	50590663	.	-	.	gene_id "lnc-bbug08501"	transcript_id "lnc-bbut00019518"	exon_number "2"
16	lncRNA	exon	50594716	50594827	.	-	.	gene_id "lnc-bbug08501"	transcript_id "lnc-bbut00019518"	exon_number "3"
16	lncRNA	exon	50584240	50584680	.	-	.	gene_id "lnc-bbug08501"	transcript_id "lnc-bbut00019519"	exon_number "1"
16	lncRNA	exon	50590459	50590533	.	-	.	gene_id "lnc-bbug08501"	transcript_id "lnc-bbut00019519"	exon_number "2"
16	lncRNA	exon	50594716	50594829	.	-	.	gene_id "lnc-bbug08501"	transcript_id "lnc-bbut00019519"	exon_number "3"
16	lncRNA	exon	50842051	50842428	.	-	.	gene_id "lnc-bbug08503"	transcript_id "lnc-bbut00019532"	exon_number "1"
16	lncRNA	exon	50851277	50852666	.	-	.	gene_id "lnc-bbug08503"	transcript_id "lnc-bbut00019532"	exon_number "2"
16	lncRNA	exon	51541642	51544053	.	-	.	gene_id "lnc-bbug08507"	transcript_id "lnc-bbut00019538"	exon_number "1"
16	lncRNA	exon	51572895	51574603	.	-	.	gene_id "lnc-bbug08507"	transcript_id "lnc-bbut00019538"	exon_number "2"
16	lncRNA	exon	52670860	52672465	.	-	.	gene_id "lnc-bbug08508"	transcript_id "lnc-bbut00019539"	exon_number "1"
16	lncRNA	exon	52672614	52672709	.	-	.	gene_id "lnc-bbug08508"	transcript_id "lnc-bbut00019539"	exon_number "2"
16	lncRNA	exon	52679743	52680278	.	-	.	gene_id "lnc-bbug08508"	transcript_id "lnc-bbut00019539"	exon_number "3"
16	lncRNA	exon	52670860	52672465	.	-	.	gene_id "lnc-bbug08508"	transcript_id "lnc-bbut00019540"	exon_number "1"
16	lncRNA	exon	52672614	52683444	.	-	.	gene_id "lnc-bbug08508"	transcript_id "lnc-bbut00019540"	exon_number "2"
16	lncRNA	exon	52948767	52948981	.	-	.	gene_id "lnc-bbug08510"	transcript_id "lnc-bbut00019542"	exon_number "1"
16	lncRNA	exon	52955424	52955477	.	-	.	gene_id "lnc-bbug08510"	transcript_id "lnc-bbut00019542"	exon_number "2"
16	lncRNA	exon	52957970	52958091	.	-	.	gene_id "lnc-bbug08510"	transcript_id "lnc-bbut00019542"	exon_number "3"
16	lncRNA	exon	55321073	55321418	.	-	.	gene_id "lnc-bbug08530"	transcript_id "lnc-bbut00019602"	exon_number "1"
16	lncRNA	exon	55321696	55321822	.	-	.	gene_id "lnc-bbug08530"	transcript_id "lnc-bbut00019602"	exon_number "2"
16	lncRNA	exon	55323871	55324006	.	-	.	gene_id "lnc-bbug08530"	transcript_id "lnc-bbut00019602"	exon_number "3"
16	lncRNA	exon	55496476	55498449	.	-	.	gene_id "lnc-bbug08535"	transcript_id "lnc-bbut00019610"	exon_number "1"
16	lncRNA	exon	55816109	55816483	.	-	.	gene_id "lnc-bbug08542"	transcript_id "lnc-bbut00019621"	exon_number "1"
16	lncRNA	exon	55816628	55816781	.	-	.	gene_id "lnc-bbug08542"	transcript_id "lnc-bbut00019621"	exon_number "2"
16	lncRNA	exon	55818528	55818650	.	-	.	gene_id "lnc-bbug08542"	transcript_id "lnc-bbut00019621"	exon_number "3"
16	lncRNA	exon	55819810	55820926	.	-	.	gene_id "lnc-bbug08542"	transcript_id "lnc-bbut00019621"	exon_number "4"
16	lncRNA	exon	55816557	55816781	.	-	.	gene_id "lnc-bbug08542"	transcript_id "lnc-bbut00019622"	exon_number "1"
16	lncRNA	exon	55818528	55818650	.	-	.	gene_id "lnc-bbug08542"	transcript_id "lnc-bbut00019622"	exon_number "2"
16	lncRNA	exon	55820093	55820926	.	-	.	gene_id "lnc-bbug08542"	transcript_id "lnc-bbut00019622"	exon_number "3"
16	lncRNA	exon	55831580	55831782	.	-	.	gene_id "lnc-bbug08543"	transcript_id "lnc-bbut00019623"	exon_number "1"
16	lncRNA	exon	55833219	55833379	.	-	.	gene_id "lnc-bbug08543"	transcript_id "lnc-bbut00019623"	exon_number "2"
16	lncRNA	exon	55839424	55839573	.	-	.	gene_id "lnc-bbug08543"	transcript_id "lnc-bbut00019623"	exon_number "3"
16	lncRNA	exon	56029991	56030184	.	-	.	gene_id "lnc-bbug08547"	transcript_id "lnc-bbut00019632"	exon_number "1"
16	lncRNA	exon	56047960	56048092	.	-	.	gene_id "lnc-bbug08547"	transcript_id "lnc-bbut00019632"	exon_number "2"

16	lncRNA	exon	56074094	56074814	.	-	.	gene_id "lnc-bbug08547"	transcript_id "lnc-bbut00019632"	exon_number "3"
16	lncRNA	exon	57437483	57437791	.	-	.	gene_id "lnc-bbug08558"	transcript_id "lnc-bbut00019658"	exon_number "1"
16	lncRNA	exon	57439078	57439174	.	-	.	gene_id "lnc-bbug08558"	transcript_id "lnc-bbut00019658"	exon_number "2"
16	lncRNA	exon	57439322	57439389	.	-	.	gene_id "lnc-bbug08558"	transcript_id "lnc-bbut00019658"	exon_number "3"
16	lncRNA	exon	57440318	57440341	.	-	.	gene_id "lnc-bbug08558"	transcript_id "lnc-bbut00019658"	exon_number "4"
16	lncRNA	exon	57833130	57837746	.	-	.	gene_id "lnc-bbug08561"	transcript_id "lnc-bbut00019661"	exon_number "1"
16	lncRNA	exon	57839278	57839336	.	-	.	gene_id "lnc-bbug08561"	transcript_id "lnc-bbut00019661"	exon_number "2"
16	lncRNA	exon	57844436	57844735	.	-	.	gene_id "lnc-bbug08561"	transcript_id "lnc-bbut00019661"	exon_number "3"
16	lncRNA	exon	58035398	58037565	.	-	.	gene_id "lnc-bbug08562"	transcript_id "lnc-bbut00019662"	exon_number "1"
16	lncRNA	exon	58038349	58038448	.	-	.	gene_id "lnc-bbug08562"	transcript_id "lnc-bbut00019662"	exon_number "2"
16	lncRNA	exon	58038535	58038812	.	-	.	gene_id "lnc-bbug08562"	transcript_id "lnc-bbut00019662"	exon_number "3"
16	lncRNA	exon	58038962	58039127	.	-	.	gene_id "lnc-bbug08562"	transcript_id "lnc-bbut00019662"	exon_number "4"
16	lncRNA	exon	58562663	58563156	.	-	.	gene_id "lnc-bbug08563"	transcript_id "lnc-bbut00019664"	exon_number "1"
16	lncRNA	exon	58591877	58591973	.	-	.	gene_id "lnc-bbug08563"	transcript_id "lnc-bbut00019664"	exon_number "2"
16	lncRNA	exon	58594742	58595077	.	-	.	gene_id "lnc-bbug08563"	transcript_id "lnc-bbut00019664"	exon_number "3"
16	lncRNA	exon	58660789	58661000	.	-	.	gene_id "lnc-bbug08564"	transcript_id "lnc-bbut00019666"	exon_number "1"
16	lncRNA	exon	58661812	58661955	.	-	.	gene_id "lnc-bbug08564"	transcript_id "lnc-bbut00019666"	exon_number "2"
16	lncRNA	exon	58662693	58662789	.	-	.	gene_id "lnc-bbug08564"	transcript_id "lnc-bbut00019666"	exon_number "3"
16	lncRNA	exon	58670129	58670652	.	-	.	gene_id "lnc-bbug08564"	transcript_id "lnc-bbut00019666"	exon_number "4"
16	lncRNA	exon	58660789	58661000	.	-	.	gene_id "lnc-bbug08564"	transcript_id "lnc-bbut00019665"	exon_number "1"
16	lncRNA	exon	58661812	58662789	.	-	.	gene_id "lnc-bbug08564"	transcript_id "lnc-bbut00019665"	exon_number "2"
16	lncRNA	exon	58670129	58670652	.	-	.	gene_id "lnc-bbug08564"	transcript_id "lnc-bbut00019665"	exon_number "3"
16	lncRNA	exon	58660789	58661000	.	-	.	gene_id "lnc-bbug08564"	transcript_id "lnc-bbut00019667"	exon_number "1"
16	lncRNA	exon	58661812	58662789	.	-	.	gene_id "lnc-bbug08564"	transcript_id "lnc-bbut00019667"	exon_number "2"
16	lncRNA	exon	58670129	58670297	.	-	.	gene_id "lnc-bbug08564"	transcript_id "lnc-bbut00019667"	exon_number "3"
16	lncRNA	exon	58674496	58675413	.	-	.	gene_id "lnc-bbug08564"	transcript_id "lnc-bbut00019667"	exon_number "4"
16	lncRNA	exon	58660789	58661000	.	-	.	gene_id "lnc-bbug08564"	transcript_id "lnc-bbut00019668"	exon_number "1"
16	lncRNA	exon	58661812	58662789	.	-	.	gene_id "lnc-bbug08564"	transcript_id "lnc-bbut00019668"	exon_number "2"
16	lncRNA	exon	58670129	58670297	.	-	.	gene_id "lnc-bbug08564"	transcript_id "lnc-bbut00019668"	exon_number "3"
16	lncRNA	exon	58701036	58701461	.	-	.	gene_id "lnc-bbug08564"	transcript_id "lnc-bbut00019668"	exon_number "4"
16	lncRNA	exon	60092825	60093251	.	-	.	gene_id "lnc-bbug08569"	transcript_id "lnc-bbut00019674"	exon_number "1"
16	lncRNA	exon	60093771	60093776	.	-	.	gene_id "lnc-bbug08569"	transcript_id "lnc-bbut00019674"	exon_number "2"
16	lncRNA	exon	60126966	60127959	.	-	.	gene_id "lnc-bbug08570"	transcript_id "lnc-bbut00019675"	exon_number "1"
16	lncRNA	exon	60132175	60132298	.	-	.	gene_id "lnc-bbug08570"	transcript_id "lnc-bbut00019675"	exon_number "2"
16	lncRNA	exon	61768374	61768622	.	-	.	gene_id "lnc-bbug08579"	transcript_id "lnc-bbut00019710"	exon_number "1"
16	lncRNA	exon	61769952	61770578	.	-	.	gene_id "lnc-bbug08579"	transcript_id "lnc-bbut00019710"	exon_number "2"
16	lncRNA	exon	61774428	61774645	.	-	.	gene_id "lnc-bbug08579"	transcript_id "lnc-bbut00019710"	exon_number "3"
16	lncRNA	exon	61800642	61803960	.	-	.	gene_id "lnc-bbug08580"	transcript_id "lnc-bbut00019711"	exon_number "1"
16	lncRNA	exon	61805034	61805124	.	-	.	gene_id "lnc-bbug08580"	transcript_id "lnc-bbut00019711"	exon_number "2"
16	lncRNA	exon	61805510	61805676	.	-	.	gene_id "lnc-bbug08580"	transcript_id "lnc-bbut00019711"	exon_number "3"
16	lncRNA	exon	61888066	61889604	.	-	.	gene_id "lnc-bbug08581"	transcript_id "lnc-bbut00019712"	exon_number "1"
16	lncRNA	exon	61890296	61890435	.	-	.	gene_id "lnc-bbug08581"	transcript_id "lnc-bbut00019712"	exon_number "2"
16	lncRNA	exon	61890517	61890583	.	-	.	gene_id "lnc-bbug08581"	transcript_id "lnc-bbut00019712"	exon_number "3"
16	lncRNA	exon	61892423	61892794	.	-	.	gene_id "lnc-bbug08581"	transcript_id "lnc-bbut00019712"	exon_number "4"
16	lncRNA	exon	62113127	62113167	.	-	.	gene_id "lnc-bbug08582"	transcript_id "lnc-bbut00019713"	exon_number "1"
16	lncRNA	exon	62114665	62114877	.	-	.	gene_id "lnc-bbug08582"	transcript_id "lnc-bbut00019713"	exon_number "2"
16	lncRNA	exon	62115933	62116640	.	-	.	gene_id "lnc-bbug08582"	transcript_id "lnc-bbut00019713"	exon_number "3"
16	lncRNA	exon	62116727	62118258	.	-	.	gene_id "lnc-bbug08583"	transcript_id "lnc-bbut00019714"	exon_number "1"
16	lncRNA	exon	62121016	62121142	.	-	.	gene_id "lnc-bbug08583"	transcript_id "lnc-bbut00019714"	exon_number "2"
16	lncRNA	exon	62123308	62123589	.	-	.	gene_id "lnc-bbug08583"	transcript_id "lnc-bbut00019714"	exon_number "3"
16	lncRNA	exon	62136813	62136924	.	-	.	gene_id "lnc-bbug08584"	transcript_id "lnc-bbut00019715"	exon_number "1"
16	lncRNA	exon	62143489	62143577	.	-	.	gene_id "lnc-bbug08584"	transcript_id "lnc-bbut00019715"	exon_number "2"

16	lncRNA	exon	62160275	62160977	.	-	.	gene_id "lnc-bbug08584"	transcript_id "lnc-bbut00019715"	exon_number "3"
16	lncRNA	exon	62553145	62554124	.	-	.	gene_id "lnc-bbug08588"	transcript_id "lnc-bbut00019721"	exon_number "1"
16	lncRNA	exon	62561866	62561982	.	-	.	gene_id "lnc-bbug08588"	transcript_id "lnc-bbut00019721"	exon_number "2"
16	lncRNA	exon	62564783	62564940	.	-	.	gene_id "lnc-bbug08588"	transcript_id "lnc-bbut00019721"	exon_number "3"
16	lncRNA	exon	62683135	62683949	.	-	.	gene_id "lnc-bbug08592"	transcript_id "lnc-bbut00019729"	exon_number "1"
16	lncRNA	exon	64931744	64932009	.	-	.	gene_id "lnc-bbug08607"	transcript_id "lnc-bbut00019759"	exon_number "1"
16	lncRNA	exon	64933286	64933395	.	-	.	gene_id "lnc-bbug08607"	transcript_id "lnc-bbut00019759"	exon_number "2"
16	lncRNA	exon	64944557	64945104	.	-	.	gene_id "lnc-bbug08607"	transcript_id "lnc-bbut00019759"	exon_number "3"
16	lncRNA	exon	64945659	64946310	.	-	.	gene_id "lnc-bbug08607"	transcript_id "lnc-bbut00019759"	exon_number "4"
16	lncRNA	exon	66218266	66219258	.	-	.	gene_id "lnc-bbug08610"	transcript_id "lnc-bbut00019763"	exon_number "1"
16	lncRNA	exon	66219621	66219684	.	-	.	gene_id "lnc-bbug08610"	transcript_id "lnc-bbut00019763"	exon_number "2"
16	lncRNA	exon	66242304	66242382	.	-	.	gene_id "lnc-bbug08610"	transcript_id "lnc-bbut00019763"	exon_number "3"
16	lncRNA	exon	66257244	66258017	.	-	.	gene_id "lnc-bbug08610"	transcript_id "lnc-bbut00019763"	exon_number "4"
16	lncRNA	exon	67569045	67571471	.	-	.	gene_id "lnc-bbug08617"	transcript_id "lnc-bbut00019778"	exon_number "1"
16	lncRNA	exon	69364609	69364714	.	-	.	gene_id "lnc-bbug08622"	transcript_id "lnc-bbut00019790"	exon_number "1"
16	lncRNA	exon	69367199	69367326	.	-	.	gene_id "lnc-bbug08622"	transcript_id "lnc-bbut00019790"	exon_number "2"
16	lncRNA	exon	69367897	69367989	.	-	.	gene_id "lnc-bbug08622"	transcript_id "lnc-bbut00019790"	exon_number "3"
16	lncRNA	exon	69364609	69364714	.	-	.	gene_id "lnc-bbug08622"	transcript_id "lnc-bbut00019791"	exon_number "1"
16	lncRNA	exon	69367199	69367299	.	-	.	gene_id "lnc-bbug08622"	transcript_id "lnc-bbut00019791"	exon_number "2"
16	lncRNA	exon	69367897	69368009	.	-	.	gene_id "lnc-bbug08622"	transcript_id "lnc-bbut00019791"	exon_number "3"
16	lncRNA	exon	70066515	70067000	.	-	.	gene_id "lnc-bbug08625"	transcript_id "lnc-bbut00019798"	exon_number "1"
16	lncRNA	exon	70067290	70068666	.	-	.	gene_id "lnc-bbug08625"	transcript_id "lnc-bbut00019798"	exon_number "2"
16	lncRNA	exon	70130952	70131096	.	-	.	gene_id "lnc-bbug08626"	transcript_id "lnc-bbut00019799"	exon_number "1"
16	lncRNA	exon	70133741	70133933	.	-	.	gene_id "lnc-bbug08626"	transcript_id "lnc-bbut00019799"	exon_number "2"
16	lncRNA	exon	70159613	70160030	.	-	.	gene_id "lnc-bbug08626"	transcript_id "lnc-bbut00019799"	exon_number "3"
16	lncRNA	exon	70932532	70932864	.	-	.	gene_id "lnc-bbug08630"	transcript_id "lnc-bbut00019812"	exon_number "1"
16	lncRNA	exon	70932964	70935783	.	-	.	gene_id "lnc-bbug08630"	transcript_id "lnc-bbut00019812"	exon_number "2"
16	lncRNA	exon	73066822	73068298	.	-	.	gene_id "lnc-bbug08632"	transcript_id "lnc-bbut00019820"	exon_number "1"
16	lncRNA	exon	78575210	78576164	.	-	.	gene_id "lnc-bbug08639"	transcript_id "lnc-bbut00019827"	exon_number "1"
16	lncRNA	exon	78576635	78576807	.	-	.	gene_id "lnc-bbug08639"	transcript_id "lnc-bbut00019827"	exon_number "2"
16	lncRNA	exon	78610059	78613066	.	-	.	gene_id "lnc-bbug08640"	transcript_id "lnc-bbut00019828"	exon_number "1"
16	lncRNA	exon	81721959	81722456	.	-	.	gene_id "lnc-bbug08655"	transcript_id "lnc-bbut00019848"	exon_number "1"
16	lncRNA	exon	81723070	81725987	.	-	.	gene_id "lnc-bbug08655"	transcript_id "lnc-bbut00019848"	exon_number "2"
16	lncRNA	exon	81742724	81743889	.	-	.	gene_id "lnc-bbug08656"	transcript_id "lnc-bbut00019850"	exon_number "1"
16	lncRNA	exon	81745037	81745303	.	-	.	gene_id "lnc-bbug08656"	transcript_id "lnc-bbut00019850"	exon_number "2"
16	lncRNA	exon	81745917	81745987	.	-	.	gene_id "lnc-bbug08656"	transcript_id "lnc-bbut00019850"	exon_number "3"
16	lncRNA	exon	83269377	83272513	.	-	.	gene_id "lnc-bbug08658"	transcript_id "lnc-bbut00019858"	exon_number "1"
16	lncRNA	exon	83520769	83521770	.	-	.	gene_id "lnc-bbug08661"	transcript_id "lnc-bbut00019864"	exon_number "1"
16	lncRNA	exon	83535000	83535134	.	-	.	gene_id "lnc-bbug08661"	transcript_id "lnc-bbut00019864"	exon_number "2"
16	lncRNA	exon	83920359	83921276	.	-	.	gene_id "lnc-bbug08664"	transcript_id "lnc-bbut00019867"	exon_number "1"
16	lncRNA	exon	84491576	84491680	.	-	.	gene_id "lnc-bbug08683"	transcript_id "lnc-bbut00019886"	exon_number "1"
16	lncRNA	exon	84499207	84500010	.	-	.	gene_id "lnc-bbug08683"	transcript_id "lnc-bbut00019886"	exon_number "2"
17	lncRNA	exon	150554	150689	.	+	.	gene_id "lnc-bbug08689"	transcript_id "lnc-bbut00019897"	exon_number "1"
17	lncRNA	exon	150917	151134	.	+	.	gene_id "lnc-bbug08689"	transcript_id "lnc-bbut00019897"	exon_number "2"
17	lncRNA	exon	152120	152272	.	+	.	gene_id "lnc-bbug08689"	transcript_id "lnc-bbut00019897"	exon_number "3"
17	lncRNA	exon	439761	440142	.	+	.	gene_id "lnc-bbug08695"	transcript_id "lnc-bbut00019916"	exon_number "1"
17	lncRNA	exon	440250	440417	.	+	.	gene_id "lnc-bbug08695"	transcript_id "lnc-bbut00019916"	exon_number "2"
17	lncRNA	exon	441828	444489	.	+	.	gene_id "lnc-bbug08695"	transcript_id "lnc-bbut00019916"	exon_number "3"
17	lncRNA	exon	645288	646001	.	+	.	gene_id "lnc-bbug08702"	transcript_id "lnc-bbut00019930"	exon_number "1"
17	lncRNA	exon	648274	648923	.	+	.	gene_id "lnc-bbug08702"	transcript_id "lnc-bbut00019930"	exon_number "2"
17	lncRNA	exon	1913270	1913594	.	+	.	gene_id "lnc-bbug08729"	transcript_id "lnc-bbut00019996"	exon_number "1"
17	lncRNA	exon	1914285	1914521	.	+	.	gene_id "lnc-bbug08729"	transcript_id "lnc-bbut00019996"	exon_number "2"

17	lncRNA	exon	2599154	2599633	.	+	.	gene_id "lnc-bbug08736"	transcript_id "lnc-bbut00020006"	exon_number "1"
17	lncRNA	exon	3202903	3203209	.	+	.	gene_id "lnc-bbug08743"	transcript_id "lnc-bbut00020032"	exon_number "1"
17	lncRNA	exon	3204662	3204697	.	+	.	gene_id "lnc-bbug08743"	transcript_id "lnc-bbut00020032"	exon_number "2"
17	lncRNA	exon	3204895	3204929	.	+	.	gene_id "lnc-bbug08743"	transcript_id "lnc-bbut00020032"	exon_number "3"
17	lncRNA	exon	3205289	3205367	.	+	.	gene_id "lnc-bbug08743"	transcript_id "lnc-bbut00020032"	exon_number "4"
17	lncRNA	exon	3205456	3205805	.	+	.	gene_id "lnc-bbug08743"	transcript_id "lnc-bbut00020032"	exon_number "5"
17	lncRNA	exon	3242867	3246868	.	+	.	gene_id "lnc-bbug08745"	transcript_id "lnc-bbut00020034"	exon_number "1"
17	lncRNA	exon	3247312	3247429	.	+	.	gene_id "lnc-bbug08745"	transcript_id "lnc-bbut00020034"	exon_number "2"
17	lncRNA	exon	3248206	3248287	.	+	.	gene_id "lnc-bbug08745"	transcript_id "lnc-bbut00020034"	exon_number "3"
17	lncRNA	exon	3249085	3249571	.	+	.	gene_id "lnc-bbug08745"	transcript_id "lnc-bbut00020034"	exon_number "4"
17	lncRNA	exon	3515899	3518651	.	+	.	gene_id "lnc-bbug08747"	transcript_id "lnc-bbut00020047"	exon_number "1"
17	lncRNA	exon	3877134	3877873	.	+	.	gene_id "lnc-bbug08759"	transcript_id "lnc-bbut00020078"	exon_number "1"
17	lncRNA	exon	3878410	3878903	.	+	.	gene_id "lnc-bbug08759"	transcript_id "lnc-bbut00020078"	exon_number "2"
17	lncRNA	exon	3880149	3880278	.	+	.	gene_id "lnc-bbug08760"	transcript_id "lnc-bbut00020079"	exon_number "1"
17	lncRNA	exon	3881871	3882004	.	+	.	gene_id "lnc-bbug08760"	transcript_id "lnc-bbut00020079"	exon_number "2"
17	lncRNA	exon	3882575	3884165	.	+	.	gene_id "lnc-bbug08760"	transcript_id "lnc-bbut00020079"	exon_number "3"
17	lncRNA	exon	4478215	4478343	.	+	.	gene_id "lnc-bbug08768"	transcript_id "lnc-bbut00020104"	exon_number "1"
17	lncRNA	exon	4480133	4480269	.	+	.	gene_id "lnc-bbug08768"	transcript_id "lnc-bbut00020104"	exon_number "2"
17	lncRNA	exon	4486585	4486667	.	+	.	gene_id "lnc-bbug08768"	transcript_id "lnc-bbut00020104"	exon_number "3"
17	lncRNA	exon	4493466	4493564	.	+	.	gene_id "lnc-bbug08768"	transcript_id "lnc-bbut00020104"	exon_number "4"
17	lncRNA	exon	4496676	4498555	.	+	.	gene_id "lnc-bbug08768"	transcript_id "lnc-bbut00020104"	exon_number "5"
17	lncRNA	exon	4478215	4478343	.	+	.	gene_id "lnc-bbug08768"	transcript_id "lnc-bbut00020103"	exon_number "1"
17	lncRNA	exon	4480133	4480269	.	+	.	gene_id "lnc-bbug08768"	transcript_id "lnc-bbut00020103"	exon_number "2"
17	lncRNA	exon	4486585	4486667	.	+	.	gene_id "lnc-bbug08768"	transcript_id "lnc-bbut00020103"	exon_number "3"
17	lncRNA	exon	4493463	4493564	.	+	.	gene_id "lnc-bbug08768"	transcript_id "lnc-bbut00020103"	exon_number "4"
17	lncRNA	exon	4496676	4498555	.	+	.	gene_id "lnc-bbug08768"	transcript_id "lnc-bbut00020103"	exon_number "5"
17	lncRNA	exon	4689110	4689221	.	+	.	gene_id "lnc-bbug08772"	transcript_id "lnc-bbut00020122"	exon_number "1"
17	lncRNA	exon	4689776	4689896	.	+	.	gene_id "lnc-bbug08772"	transcript_id "lnc-bbut00020122"	exon_number "2"
17	lncRNA	exon	4690075	4690412	.	+	.	gene_id "lnc-bbug08772"	transcript_id "lnc-bbut00020122"	exon_number "3"
17	lncRNA	exon	4931796	4934384	.	+	.	gene_id "lnc-bbug08774"	transcript_id "lnc-bbut00020124"	exon_number "1"
17	lncRNA	exon	4935154	4935320	.	+	.	gene_id "lnc-bbug08774"	transcript_id "lnc-bbut00020124"	exon_number "2"
17	lncRNA	exon	5306410	5311585	.	+	.	gene_id "lnc-bbug08778"	transcript_id "lnc-bbut00020134"	exon_number "1"
17	lncRNA	exon	5313102	5314027	.	+	.	gene_id "lnc-bbug08778"	transcript_id "lnc-bbut00020134"	exon_number "2"
17	lncRNA	exon	6373103	6373335	.	+	.	gene_id "lnc-bbug08781"	transcript_id "lnc-bbut00020140"	exon_number "1"
17	lncRNA	exon	6685143	6686252	.	+	.	gene_id "lnc-bbug08782"	transcript_id "lnc-bbut00020141"	exon_number "1"
17	lncRNA	exon	6688502	6688636	.	+	.	gene_id "lnc-bbug08782"	transcript_id "lnc-bbut00020141"	exon_number "2"
17	lncRNA	exon	6828505	6830184	.	+	.	gene_id "lnc-bbug08786"	transcript_id "lnc-bbut00020147"	exon_number "1"
17	lncRNA	exon	6830263	6831180	.	+	.	gene_id "lnc-bbug08786"	transcript_id "lnc-bbut00020147"	exon_number "2"
17	lncRNA	exon	7357374	7357595	.	+	.	gene_id "lnc-bbug08788"	transcript_id "lnc-bbut00020154"	exon_number "1"
17	lncRNA	exon	7358034	7358113	.	+	.	gene_id "lnc-bbug08788"	transcript_id "lnc-bbut00020154"	exon_number "2"
17	lncRNA	exon	7362063	7362174	.	+	.	gene_id "lnc-bbug08788"	transcript_id "lnc-bbut00020154"	exon_number "3"
17	lncRNA	exon	8484331	8485151	.	+	.	gene_id "lnc-bbug08794"	transcript_id "lnc-bbut00020167"	exon_number "1"
17	lncRNA	exon	8564356	8564536	.	+	.	gene_id "lnc-bbug08794"	transcript_id "lnc-bbut00020167"	exon_number "2"
17	lncRNA	exon	8566029	8566933	.	+	.	gene_id "lnc-bbug08794"	transcript_id "lnc-bbut00020167"	exon_number "3"
17	lncRNA	exon	8641455	8642948	.	+	.	gene_id "lnc-bbug08797"	transcript_id "lnc-bbut00020170"	exon_number "1"
17	lncRNA	exon	8644069	8644345	.	+	.	gene_id "lnc-bbug08797"	transcript_id "lnc-bbut00020170"	exon_number "2"
17	lncRNA	exon	8737703	8738196	.	+	.	gene_id "lnc-bbug08798"	transcript_id "lnc-bbut00020171"	exon_number "1"
17	lncRNA	exon	9536970	9537083	.	+	.	gene_id "lnc-bbug08806"	transcript_id "lnc-bbut00020191"	exon_number "1"
17	lncRNA	exon	9538001	9539664	.	+	.	gene_id "lnc-bbug08806"	transcript_id "lnc-bbut00020191"	exon_number "2"
17	lncRNA	exon	9539793	9539947	.	+	.	gene_id "lnc-bbug08806"	transcript_id "lnc-bbut00020191"	exon_number "3"
17	lncRNA	exon	9536970	9537083	.	+	.	gene_id "lnc-bbug08806"	transcript_id "lnc-bbut00020190"	exon_number "1"
17	lncRNA	exon	9538001	9538778	.	+	.	gene_id "lnc-bbug08806"	transcript_id "lnc-bbut00020190"	exon_number "2"

17	lncRNA	exon	9539438	9539947	.	+	.	gene_id "lnc-bbug08806"	transcript_id "lnc-bbut00020190"	exon_number "3"
17	lncRNA	exon	9605946	9606386	.	+	.	gene_id "lnc-bbug08807"	transcript_id "lnc-bbut00020195"	exon_number "1"
17	lncRNA	exon	9609055	9609135	.	+	.	gene_id "lnc-bbug08807"	transcript_id "lnc-bbut00020195"	exon_number "2"
17	lncRNA	exon	9610113	9610634	.	+	.	gene_id "lnc-bbug08807"	transcript_id "lnc-bbut00020195"	exon_number "3"
17	lncRNA	exon	10007816	10008560	.	+	.	gene_id "lnc-bbug08815"	transcript_id "lnc-bbut00020212"	exon_number "1"
17	lncRNA	exon	10016476	10016868	.	+	.	gene_id "lnc-bbug08815"	transcript_id "lnc-bbut00020212"	exon_number "2"
17	lncRNA	exon	10134297	10134750	.	+	.	gene_id "lnc-bbug08816"	transcript_id "lnc-bbut00020213"	exon_number "1"
17	lncRNA	exon	10140513	10140637	.	+	.	gene_id "lnc-bbug08816"	transcript_id "lnc-bbut00020213"	exon_number "2"
17	lncRNA	exon	10140917	10141803	.	+	.	gene_id "lnc-bbug08816"	transcript_id "lnc-bbut00020213"	exon_number "3"
17	lncRNA	exon	10137485	10140637	.	+	.	gene_id "lnc-bbug08816"	transcript_id "lnc-bbut00020214"	exon_number "1"
17	lncRNA	exon	10140917	10141803	.	+	.	gene_id "lnc-bbug08816"	transcript_id "lnc-bbut00020214"	exon_number "2"
17	lncRNA	exon	10235917	10236102	.	+	.	gene_id "lnc-bbug08819"	transcript_id "lnc-bbut00020218"	exon_number "1"
17	lncRNA	exon	10242921	10243026	.	+	.	gene_id "lnc-bbug08819"	transcript_id "lnc-bbut00020218"	exon_number "2"
17	lncRNA	exon	10248848	10249496	.	+	.	gene_id "lnc-bbug08819"	transcript_id "lnc-bbut00020218"	exon_number "3"
17	lncRNA	exon	10275615	10275833	.	+	.	gene_id "lnc-bbug08821"	transcript_id "lnc-bbut00020220"	exon_number "1"
17	lncRNA	exon	10276980	10278511	.	+	.	gene_id "lnc-bbug08821"	transcript_id "lnc-bbut00020220"	exon_number "2"
17	lncRNA	exon	11252590	11253095	.	+	.	gene_id "lnc-bbug08835"	transcript_id "lnc-bbut00020246"	exon_number "1"
17	lncRNA	exon	11961184	11961731	.	+	.	gene_id "lnc-bbug08846"	transcript_id "lnc-bbut00020277"	exon_number "1"
17	lncRNA	exon	11962184	11963185	.	+	.	gene_id "lnc-bbug08846"	transcript_id "lnc-bbut00020277"	exon_number "2"
17	lncRNA	exon	12750287	12751053	.	+	.	gene_id "lnc-bbug08848"	transcript_id "lnc-bbut00020281"	exon_number "1"
17	lncRNA	exon	12751129	12751680	.	+	.	gene_id "lnc-bbug08848"	transcript_id "lnc-bbut00020281"	exon_number "2"
17	lncRNA	exon	12751885	12752316	.	+	.	gene_id "lnc-bbug08848"	transcript_id "lnc-bbut00020281"	exon_number "3"
17	lncRNA	exon	12753513	12754323	.	+	.	gene_id "lnc-bbug08848"	transcript_id "lnc-bbut00020281"	exon_number "4"
17	lncRNA	exon	12750287	12751053	.	+	.	gene_id "lnc-bbug08848"	transcript_id "lnc-bbut00020280"	exon_number "1"
17	lncRNA	exon	12751129	12752451	.	+	.	gene_id "lnc-bbug08848"	transcript_id "lnc-bbut00020280"	exon_number "2"
17	lncRNA	exon	12753513	12754323	.	+	.	gene_id "lnc-bbug08848"	transcript_id "lnc-bbut00020280"	exon_number "3"
17	lncRNA	exon	12750287	12751053	.	+	.	gene_id "lnc-bbug08848"	transcript_id "lnc-bbut00020279"	exon_number "1"
17	lncRNA	exon	12751129	12752316	.	+	.	gene_id "lnc-bbug08848"	transcript_id "lnc-bbut00020279"	exon_number "2"
17	lncRNA	exon	12753513	12754323	.	+	.	gene_id "lnc-bbug08848"	transcript_id "lnc-bbut00020279"	exon_number "3"
17	lncRNA	exon	12925932	12926062	.	+	.	gene_id "lnc-bbug08849"	transcript_id "lnc-bbut00020282"	exon_number "1"
17	lncRNA	exon	12950913	12951325	.	+	.	gene_id "lnc-bbug08849"	transcript_id "lnc-bbut00020282"	exon_number "2"
17	lncRNA	exon	12973202	12973459	.	+	.	gene_id "lnc-bbug08850"	transcript_id "lnc-bbut00020284"	exon_number "1"
17	lncRNA	exon	12982406	12982619	.	+	.	gene_id "lnc-bbug08850"	transcript_id "lnc-bbut00020284"	exon_number "2"
17	lncRNA	exon	13008182	13008865	.	+	.	gene_id "lnc-bbug08850"	transcript_id "lnc-bbut00020284"	exon_number "3"
17	lncRNA	exon	12980212	12981582	.	+	.	gene_id "lnc-bbug08850"	transcript_id "lnc-bbut00020285"	exon_number "1"
17	lncRNA	exon	12981708	12981812	.	+	.	gene_id "lnc-bbug08850"	transcript_id "lnc-bbut00020285"	exon_number "2"
17	lncRNA	exon	12982406	12982619	.	+	.	gene_id "lnc-bbug08850"	transcript_id "lnc-bbut00020285"	exon_number "3"
17	lncRNA	exon	13008182	13008865	.	+	.	gene_id "lnc-bbug08850"	transcript_id "lnc-bbut00020285"	exon_number "4"
17	lncRNA	exon	13039795	13041296	.	+	.	gene_id "lnc-bbug08851"	transcript_id "lnc-bbut00020286"	exon_number "1"
17	lncRNA	exon	13045087	13045373	.	+	.	gene_id "lnc-bbug08851"	transcript_id "lnc-bbut00020286"	exon_number "2"
17	lncRNA	exon	13039849	13041296	.	+	.	gene_id "lnc-bbug08851"	transcript_id "lnc-bbut00020287"	exon_number "1"
17	lncRNA	exon	13045153	13045373	.	+	.	gene_id "lnc-bbug08851"	transcript_id "lnc-bbut00020287"	exon_number "2"
17	lncRNA	exon	13165612	13165729	.	+	.	gene_id "lnc-bbug08852"	transcript_id "lnc-bbut00020292"	exon_number "1"
17	lncRNA	exon	13186247	13186403	.	+	.	gene_id "lnc-bbug08852"	transcript_id "lnc-bbut00020292"	exon_number "2"
17	lncRNA	exon	13220858	13220939	.	+	.	gene_id "lnc-bbug08852"	transcript_id "lnc-bbut00020292"	exon_number "3"
17	lncRNA	exon	13232723	13232826	.	+	.	gene_id "lnc-bbug08852"	transcript_id "lnc-bbut00020292"	exon_number "4"
17	lncRNA	exon	13233659	13233761	.	+	.	gene_id "lnc-bbug08852"	transcript_id "lnc-bbut00020292"	exon_number "5"
17	lncRNA	exon	13234544	13235594	.	+	.	gene_id "lnc-bbug08852"	transcript_id "lnc-bbut00020292"	exon_number "6"
17	lncRNA	exon	13165612	13165729	.	+	.	gene_id "lnc-bbug08852"	transcript_id "lnc-bbut00020291"	exon_number "1"
17	lncRNA	exon	13186247	13186403	.	+	.	gene_id "lnc-bbug08852"	transcript_id "lnc-bbut00020291"	exon_number "2"
17	lncRNA	exon	13232723	13232826	.	+	.	gene_id "lnc-bbug08852"	transcript_id "lnc-bbut00020291"	exon_number "3"
17	lncRNA	exon	13233659	13233761	.	+	.	gene_id "lnc-bbug08852"	transcript_id "lnc-bbut00020291"	exon_number "4"

17	lncRNA	exon	13234544	13235594	.	+	.	gene_id "lnc-bbug08852"	transcript_id "lnc-bbut00020291"	exon_number "5"
17	lncRNA	exon	13180124	13181194	.	+	.	gene_id "lnc-bbug08852"	transcript_id "lnc-bbut00020294"	exon_number "1"
17	lncRNA	exon	13184974	13185109	.	+	.	gene_id "lnc-bbug08852"	transcript_id "lnc-bbut00020294"	exon_number "2"
17	lncRNA	exon	13186247	13186403	.	+	.	gene_id "lnc-bbug08852"	transcript_id "lnc-bbut00020294"	exon_number "3"
17	lncRNA	exon	13220858	13220939	.	+	.	gene_id "lnc-bbug08852"	transcript_id "lnc-bbut00020294"	exon_number "4"
17	lncRNA	exon	13232723	13232826	.	+	.	gene_id "lnc-bbug08852"	transcript_id "lnc-bbut00020294"	exon_number "5"
17	lncRNA	exon	13233659	13233761	.	+	.	gene_id "lnc-bbug08852"	transcript_id "lnc-bbut00020294"	exon_number "6"
17	lncRNA	exon	13234544	13235594	.	+	.	gene_id "lnc-bbug08852"	transcript_id "lnc-bbut00020294"	exon_number "7"
17	lncRNA	exon	15040221	15041357	.	+	.	gene_id "lnc-bbug08855"	transcript_id "lnc-bbut00020297"	exon_number "1"
17	lncRNA	exon	17193324	17193618	.	+	.	gene_id "lnc-bbug08863"	transcript_id "lnc-bbut00020311"	exon_number "1"
17	lncRNA	exon	17227676	17227816	.	+	.	gene_id "lnc-bbug08863"	transcript_id "lnc-bbut00020311"	exon_number "2"
17	lncRNA	exon	17239185	17241185	.	+	.	gene_id "lnc-bbug08863"	transcript_id "lnc-bbut00020311"	exon_number "3"
17	lncRNA	exon	17282887	17282990	.	+	.	gene_id "lnc-bbug08865"	transcript_id "lnc-bbut00020313"	exon_number "1"
17	lncRNA	exon	17283924	17284216	.	+	.	gene_id "lnc-bbug08865"	transcript_id "lnc-bbut00020313"	exon_number "2"
17	lncRNA	exon	17284453	17284607	.	+	.	gene_id "lnc-bbug08865"	transcript_id "lnc-bbut00020313"	exon_number "3"
17	lncRNA	exon	17285393	17289568	.	+	.	gene_id "lnc-bbug08865"	transcript_id "lnc-bbut00020313"	exon_number "4"
17	lncRNA	exon	17628321	17628567	.	+	.	gene_id "lnc-bbug08866"	transcript_id "lnc-bbut00020315"	exon_number "1"
17	lncRNA	exon	17629044	17629200	.	+	.	gene_id "lnc-bbug08866"	transcript_id "lnc-bbut00020315"	exon_number "2"
17	lncRNA	exon	17631238	17631399	.	+	.	gene_id "lnc-bbug08866"	transcript_id "lnc-bbut00020315"	exon_number "3"
17	lncRNA	exon	17634145	17634349	.	+	.	gene_id "lnc-bbug08866"	transcript_id "lnc-bbut00020315"	exon_number "4"
17	lncRNA	exon	17636410	17636989	.	+	.	gene_id "lnc-bbug08866"	transcript_id "lnc-bbut00020315"	exon_number "5"
17	lncRNA	exon	17637251	17638198	.	+	.	gene_id "lnc-bbug08866"	transcript_id "lnc-bbut00020315"	exon_number "6"
17	lncRNA	exon	17628321	17628567	.	+	.	gene_id "lnc-bbug08866"	transcript_id "lnc-bbut00020314"	exon_number "1"
17	lncRNA	exon	17629040	17629200	.	+	.	gene_id "lnc-bbug08866"	transcript_id "lnc-bbut00020314"	exon_number "2"
17	lncRNA	exon	17631238	17631399	.	+	.	gene_id "lnc-bbug08866"	transcript_id "lnc-bbut00020314"	exon_number "3"
17	lncRNA	exon	17634145	17634349	.	+	.	gene_id "lnc-bbug08866"	transcript_id "lnc-bbut00020314"	exon_number "4"
17	lncRNA	exon	17636410	17636989	.	+	.	gene_id "lnc-bbug08866"	transcript_id "lnc-bbut00020314"	exon_number "5"
17	lncRNA	exon	17637251	17638198	.	+	.	gene_id "lnc-bbug08866"	transcript_id "lnc-bbut00020314"	exon_number "6"
17	lncRNA	exon	17628329	17628567	.	+	.	gene_id "lnc-bbug08866"	transcript_id "lnc-bbut00020317"	exon_number "1"
17	lncRNA	exon	17631238	17631399	.	+	.	gene_id "lnc-bbug08866"	transcript_id "lnc-bbut00020317"	exon_number "2"
17	lncRNA	exon	17634145	17634349	.	+	.	gene_id "lnc-bbug08866"	transcript_id "lnc-bbut00020317"	exon_number "3"
17	lncRNA	exon	17636410	17636989	.	+	.	gene_id "lnc-bbug08866"	transcript_id "lnc-bbut00020317"	exon_number "4"
17	lncRNA	exon	17637251	17638198	.	+	.	gene_id "lnc-bbug08866"	transcript_id "lnc-bbut00020317"	exon_number "5"
17	lncRNA	exon	17628329	17628567	.	+	.	gene_id "lnc-bbug08866"	transcript_id "lnc-bbut00020316"	exon_number "1"
17	lncRNA	exon	17631238	17631399	.	+	.	gene_id "lnc-bbug08866"	transcript_id "lnc-bbut00020316"	exon_number "2"
17	lncRNA	exon	17634145	17634349	.	+	.	gene_id "lnc-bbug08866"	transcript_id "lnc-bbut00020316"	exon_number "3"
17	lncRNA	exon	17636410	17636989	.	+	.	gene_id "lnc-bbug08866"	transcript_id "lnc-bbut00020316"	exon_number "4"
17	lncRNA	exon	17637246	17638198	.	+	.	gene_id "lnc-bbug08866"	transcript_id "lnc-bbut00020316"	exon_number "5"
17	lncRNA	exon	18824628	18824824	.	+	.	gene_id "lnc-bbug08878"	transcript_id "lnc-bbut00020349"	exon_number "1"
17	lncRNA	exon	18825040	18827200	.	+	.	gene_id "lnc-bbug08878"	transcript_id "lnc-bbut00020349"	exon_number "2"
17	lncRNA	exon	19380091	19380601	.	+	.	gene_id "lnc-bbug08883"	transcript_id "lnc-bbut00020358"	exon_number "1"
17	lncRNA	exon	19402756	19402894	.	+	.	gene_id "lnc-bbug08883"	transcript_id "lnc-bbut00020358"	exon_number "2"
17	lncRNA	exon	19405309	19405858	.	+	.	gene_id "lnc-bbug08883"	transcript_id "lnc-bbut00020358"	exon_number "3"
17	lncRNA	exon	19861172	19861301	.	+	.	gene_id "lnc-bbug08893"	transcript_id "lnc-bbut00020387"	exon_number "1"
17	lncRNA	exon	19868807	19869054	.	+	.	gene_id "lnc-bbug08893"	transcript_id "lnc-bbut00020387"	exon_number "2"
17	lncRNA	exon	19870838	19870914	.	+	.	gene_id "lnc-bbug08893"	transcript_id "lnc-bbut00020387"	exon_number "3"
17	lncRNA	exon	19871100	19871214	.	+	.	gene_id "lnc-bbug08893"	transcript_id "lnc-bbut00020387"	exon_number "4"
17	lncRNA	exon	19861172	19861301	.	+	.	gene_id "lnc-bbug08893"	transcript_id "lnc-bbut00020388"	exon_number "1"
17	lncRNA	exon	19868807	19869054	.	+	.	gene_id "lnc-bbug08893"	transcript_id "lnc-bbut00020388"	exon_number "2"
17	lncRNA	exon	19870838	19870914	.	+	.	gene_id "lnc-bbug08893"	transcript_id "lnc-bbut00020388"	exon_number "3"
17	lncRNA	exon	19876995	19877040	.	+	.	gene_id "lnc-bbug08893"	transcript_id "lnc-bbut00020388"	exon_number "4"
17	lncRNA	exon	20442731	20443208	.	+	.	gene_id "lnc-bbug08898"	transcript_id "lnc-bbut00020405"	exon_number "1"

17	lncRNA	exon	20444698	20444904	.	+	.	gene_id "lnc-bbug08898"	transcript_id "lnc-bbut00020405"	exon_number "2"
17	lncRNA	exon	20604689	20604937	.	+	.	gene_id "lnc-bbug08901"	transcript_id "lnc-bbut00020412"	exon_number "1"
17	lncRNA	exon	20605312	20605778	.	+	.	gene_id "lnc-bbug08901"	transcript_id "lnc-bbut00020412"	exon_number "2"
17	lncRNA	exon	20798528	20798742	.	+	.	gene_id "lnc-bbug08903"	transcript_id "lnc-bbut00020416"	exon_number "1"
17	lncRNA	exon	20802758	20803656	.	+	.	gene_id "lnc-bbug08903"	transcript_id "lnc-bbut00020416"	exon_number "2"
17	lncRNA	exon	21391793	21393458	.	+	.	gene_id "lnc-bbug08913"	transcript_id "lnc-bbut00020439"	exon_number "1"
17	lncRNA	exon	21394044	21394154	.	+	.	gene_id "lnc-bbug08913"	transcript_id "lnc-bbut00020439"	exon_number "2"
17	lncRNA	exon	21397206	21397316	.	+	.	gene_id "lnc-bbug08913"	transcript_id "lnc-bbut00020439"	exon_number "3"
17	lncRNA	exon	21399334	21402361	.	+	.	gene_id "lnc-bbug08913"	transcript_id "lnc-bbut00020439"	exon_number "4"
17	lncRNA	exon	21410561	21410618	.	+	.	gene_id "lnc-bbug08914"	transcript_id "lnc-bbut00020440"	exon_number "1"
17	lncRNA	exon	21411420	21411511	.	+	.	gene_id "lnc-bbug08914"	transcript_id "lnc-bbut00020440"	exon_number "2"
17	lncRNA	exon	21440697	21440857	.	+	.	gene_id "lnc-bbug08914"	transcript_id "lnc-bbut00020440"	exon_number "3"
17	lncRNA	exon	21444922	21445097	.	+	.	gene_id "lnc-bbug08915"	transcript_id "lnc-bbut00020441"	exon_number "1"
17	lncRNA	exon	21445203	21448898	.	+	.	gene_id "lnc-bbug08915"	transcript_id "lnc-bbut00020441"	exon_number "2"
17	lncRNA	exon	22106169	22106296	.	+	.	gene_id "lnc-bbug08917"	transcript_id "lnc-bbut00020443"	exon_number "1"
17	lncRNA	exon	22109377	22109491	.	+	.	gene_id "lnc-bbug08917"	transcript_id "lnc-bbut00020443"	exon_number "2"
17	lncRNA	exon	22148007	22148538	.	+	.	gene_id "lnc-bbug08918"	transcript_id "lnc-bbut00020444"	exon_number "1"
17	lncRNA	exon	22153408	22153474	.	+	.	gene_id "lnc-bbug08918"	transcript_id "lnc-bbut00020444"	exon_number "2"
17	lncRNA	exon	22154811	22155497	.	+	.	gene_id "lnc-bbug08918"	transcript_id "lnc-bbut00020444"	exon_number "3"
17	lncRNA	exon	23042494	23042743	.	+	.	gene_id "lnc-bbug08922"	transcript_id "lnc-bbut00020454"	exon_number "1"
17	lncRNA	exon	23047091	23047156	.	+	.	gene_id "lnc-bbug08922"	transcript_id "lnc-bbut00020454"	exon_number "2"
17	lncRNA	exon	23052273	23052928	.	+	.	gene_id "lnc-bbug08922"	transcript_id "lnc-bbut00020454"	exon_number "3"
17	lncRNA	exon	23359527	23360276	.	+	.	gene_id "lnc-bbug08923"	transcript_id "lnc-bbut00020455"	exon_number "1"
17	lncRNA	exon	23365469	23365592	.	+	.	gene_id "lnc-bbug08923"	transcript_id "lnc-bbut00020455"	exon_number "2"
17	lncRNA	exon	23367888	23368951	.	+	.	gene_id "lnc-bbug08923"	transcript_id "lnc-bbut00020455"	exon_number "3"
17	lncRNA	exon	26677790	26677839	.	+	.	gene_id "lnc-bbug08929"	transcript_id "lnc-bbut00020470"	exon_number "1"
17	lncRNA	exon	26680928	26681179	.	+	.	gene_id "lnc-bbug08929"	transcript_id "lnc-bbut00020470"	exon_number "2"
17	lncRNA	exon	28650263	28650277	.	+	.	gene_id "lnc-bbug08944"	transcript_id "lnc-bbut00020541"	exon_number "1"
17	lncRNA	exon	28657692	28657892	.	+	.	gene_id "lnc-bbug08944"	transcript_id "lnc-bbut00020541"	exon_number "2"
17	lncRNA	exon	28664451	28667319	.	+	.	gene_id "lnc-bbug08944"	transcript_id "lnc-bbut00020541"	exon_number "3"
17	lncRNA	exon	28681771	28682851	.	+	.	gene_id "lnc-bbug08945"	transcript_id "lnc-bbut00020542"	exon_number "1"
17	lncRNA	exon	32576237	32576297	.	+	.	gene_id "lnc-bbug08958"	transcript_id "lnc-bbut00020592"	exon_number "1"
17	lncRNA	exon	32577614	32578188	.	+	.	gene_id "lnc-bbug08958"	transcript_id "lnc-bbut00020592"	exon_number "2"
17	lncRNA	exon	32578221	32580348	.	+	.	gene_id "lnc-bbug08958"	transcript_id "lnc-bbut00020592"	exon_number "3"
17	lncRNA	exon	32695739	32695857	.	+	.	gene_id "lnc-bbug08960"	transcript_id "lnc-bbut00020595"	exon_number "1"
17	lncRNA	exon	32697954	32698637	.	+	.	gene_id "lnc-bbug08960"	transcript_id "lnc-bbut00020595"	exon_number "2"
17	lncRNA	exon	32702219	32703371	.	+	.	gene_id "lnc-bbug08961"	transcript_id "lnc-bbut00020597"	exon_number "1"
17	lncRNA	exon	37908417	37908577	.	+	.	gene_id "lnc-bbug08969"	transcript_id "lnc-bbut00020635"	exon_number "1"
17	lncRNA	exon	37974448	37974516	.	+	.	gene_id "lnc-bbug08969"	transcript_id "lnc-bbut00020635"	exon_number "2"
17	lncRNA	exon	37976705	37976897	.	+	.	gene_id "lnc-bbug08969"	transcript_id "lnc-bbut00020635"	exon_number "3"
17	lncRNA	exon	37977282	37977403	.	+	.	gene_id "lnc-bbug08969"	transcript_id "lnc-bbut00020635"	exon_number "4"
17	lncRNA	exon	37980441	37980634	.	+	.	gene_id "lnc-bbug08969"	transcript_id "lnc-bbut00020635"	exon_number "5"
17	lncRNA	exon	37985734	37986761	.	+	.	gene_id "lnc-bbug08969"	transcript_id "lnc-bbut00020635"	exon_number "6"
17	lncRNA	exon	37908425	37908577	.	+	.	gene_id "lnc-bbug08969"	transcript_id "lnc-bbut00020638"	exon_number "1"
17	lncRNA	exon	37974448	37974516	.	+	.	gene_id "lnc-bbug08969"	transcript_id "lnc-bbut00020638"	exon_number "2"
17	lncRNA	exon	37976705	37976897	.	+	.	gene_id "lnc-bbug08969"	transcript_id "lnc-bbut00020638"	exon_number "3"
17	lncRNA	exon	37980441	37980634	.	+	.	gene_id "lnc-bbug08969"	transcript_id "lnc-bbut00020638"	exon_number "4"
17	lncRNA	exon	37980982	37981035	.	+	.	gene_id "lnc-bbug08969"	transcript_id "lnc-bbut00020638"	exon_number "5"
17	lncRNA	exon	37985734	37986761	.	+	.	gene_id "lnc-bbug08969"	transcript_id "lnc-bbut00020638"	exon_number "6"
17	lncRNA	exon	37908425	37908577	.	+	.	gene_id "lnc-bbug08969"	transcript_id "lnc-bbut00020637"	exon_number "1"
17	lncRNA	exon	37943429	37943537	.	+	.	gene_id "lnc-bbug08969"	transcript_id "lnc-bbut00020637"	exon_number "2"
17	lncRNA	exon	37974448	37974516	.	+	.	gene_id "lnc-bbug08969"	transcript_id "lnc-bbut00020637"	exon_number "3"

17	lncRNA	exon	37976705	37976897	.	+	.	gene_id "lnc-bbug08969"	transcript_id "lnc-bbut00020637"	exon_number "4"
17	lncRNA	exon	37980441	37980634	.	+	.	gene_id "lnc-bbug08969"	transcript_id "lnc-bbut00020637"	exon_number "5"
17	lncRNA	exon	37985734	37986761	.	+	.	gene_id "lnc-bbug08969"	transcript_id "lnc-bbut00020637"	exon_number "6"
17	lncRNA	exon	37908425	37908577	.	+	.	gene_id "lnc-bbug08969"	transcript_id "lnc-bbut00020636"	exon_number "1"
17	lncRNA	exon	37956085	37956236	.	+	.	gene_id "lnc-bbug08969"	transcript_id "lnc-bbut00020636"	exon_number "2"
17	lncRNA	exon	37976705	37976897	.	+	.	gene_id "lnc-bbug08969"	transcript_id "lnc-bbut00020636"	exon_number "3"
17	lncRNA	exon	37980441	37980634	.	+	.	gene_id "lnc-bbug08969"	transcript_id "lnc-bbut00020636"	exon_number "4"
17	lncRNA	exon	37985734	37986761	.	+	.	gene_id "lnc-bbug08969"	transcript_id "lnc-bbut00020636"	exon_number "5"
17	lncRNA	exon	37908433	37908577	.	+	.	gene_id "lnc-bbug08969"	transcript_id "lnc-bbut00020639"	exon_number "1"
17	lncRNA	exon	37974448	37974516	.	+	.	gene_id "lnc-bbug08969"	transcript_id "lnc-bbut00020639"	exon_number "2"
17	lncRNA	exon	37976705	37976897	.	+	.	gene_id "lnc-bbug08969"	transcript_id "lnc-bbut00020639"	exon_number "3"
17	lncRNA	exon	37980441	37980634	.	+	.	gene_id "lnc-bbug08969"	transcript_id "lnc-bbut00020639"	exon_number "4"
17	lncRNA	exon	37985734	37986680	.	+	.	gene_id "lnc-bbug08969"	transcript_id "lnc-bbut00020639"	exon_number "5"
17	lncRNA	exon	37989845	37990106	.	+	.	gene_id "lnc-bbug08969"	transcript_id "lnc-bbut00020639"	exon_number "6"
17	lncRNA	exon	37908439	37908577	.	+	.	gene_id "lnc-bbug08969"	transcript_id "lnc-bbut00020640"	exon_number "1"
17	lncRNA	exon	37976705	37976897	.	+	.	gene_id "lnc-bbug08969"	transcript_id "lnc-bbut00020640"	exon_number "2"
17	lncRNA	exon	37980441	37980634	.	+	.	gene_id "lnc-bbug08969"	transcript_id "lnc-bbut00020640"	exon_number "3"
17	lncRNA	exon	37985734	37986761	.	+	.	gene_id "lnc-bbug08969"	transcript_id "lnc-bbut00020640"	exon_number "4"
17	lncRNA	exon	38878201	38879662	.	+	.	gene_id "lnc-bbug08974"	transcript_id "lnc-bbut00020656"	exon_number "1"
17	lncRNA	exon	38924378	38924430	.	+	.	gene_id "lnc-bbug08974"	transcript_id "lnc-bbut00020656"	exon_number "2"
17	lncRNA	exon	38972368	38973744	.	+	.	gene_id "lnc-bbug08974"	transcript_id "lnc-bbut00020656"	exon_number "3"
17	lncRNA	exon	39237506	39238538	.	+	.	gene_id "lnc-bbug08976"	transcript_id "lnc-bbut00020658"	exon_number "1"
17	lncRNA	exon	39238672	39238753	.	+	.	gene_id "lnc-bbug08976"	transcript_id "lnc-bbut00020658"	exon_number "2"
17	lncRNA	exon	39238901	39238952	.	+	.	gene_id "lnc-bbug08976"	transcript_id "lnc-bbut00020658"	exon_number "3"
17	lncRNA	exon	39239560	39240080	.	+	.	gene_id "lnc-bbug08976"	transcript_id "lnc-bbut00020658"	exon_number "4"
17	lncRNA	exon	41161823	41162832	.	+	.	gene_id "lnc-bbug08978"	transcript_id "lnc-bbut00020660"	exon_number "1"
17	lncRNA	exon	41809415	41809745	.	+	.	gene_id "lnc-bbug08979"	transcript_id "lnc-bbut00020661"	exon_number "1"
17	lncRNA	exon	41809885	41810189	.	+	.	gene_id "lnc-bbug08979"	transcript_id "lnc-bbut00020661"	exon_number "2"
17	lncRNA	exon	43324178	43327977	.	+	.	gene_id "lnc-bbug08987"	transcript_id "lnc-bbut00020675"	exon_number "1"
17	lncRNA	exon	43798095	43798202	.	+	.	gene_id "lnc-bbug08992"	transcript_id "lnc-bbut00020687"	exon_number "1"
17	lncRNA	exon	43835614	43835745	.	+	.	gene_id "lnc-bbug08992"	transcript_id "lnc-bbut00020687"	exon_number "2"
17	lncRNA	exon	43870919	43870981	.	+	.	gene_id "lnc-bbug08992"	transcript_id "lnc-bbut00020687"	exon_number "3"
17	lncRNA	exon	43874554	43876633	.	+	.	gene_id "lnc-bbug08992"	transcript_id "lnc-bbut00020687"	exon_number "4"
17	lncRNA	exon	44007517	44008074	.	+	.	gene_id "lnc-bbug08994"	transcript_id "lnc-bbut00020690"	exon_number "1"
17	lncRNA	exon	44429269	44429413	.	+	.	gene_id "lnc-bbug08997"	transcript_id "lnc-bbut00020703"	exon_number "1"
17	lncRNA	exon	44430186	44430233	.	+	.	gene_id "lnc-bbug08997"	transcript_id "lnc-bbut00020703"	exon_number "2"
17	lncRNA	exon	44432326	44432707	.	+	.	gene_id "lnc-bbug08997"	transcript_id "lnc-bbut00020703"	exon_number "3"
17	lncRNA	exon	44990254	44990309	.	+	.	gene_id "lnc-bbug08999"	transcript_id "lnc-bbut00020705"	exon_number "1"
17	lncRNA	exon	45023580	45023601	.	+	.	gene_id "lnc-bbug08999"	transcript_id "lnc-bbut00020705"	exon_number "2"
17	lncRNA	exon	45067575	45067733	.	+	.	gene_id "lnc-bbug08999"	transcript_id "lnc-bbut00020705"	exon_number "3"
17	lncRNA	exon	45079056	45079105	.	+	.	gene_id "lnc-bbug08999"	transcript_id "lnc-bbut00020705"	exon_number "4"
17	lncRNA	exon	45028943	45029563	.	+	.	gene_id "lnc-bbug08999"	transcript_id "lnc-bbut00020706"	exon_number "1"
17	lncRNA	exon	45067575	45067733	.	+	.	gene_id "lnc-bbug08999"	transcript_id "lnc-bbut00020706"	exon_number "2"
17	lncRNA	exon	45079056	45079105	.	+	.	gene_id "lnc-bbug08999"	transcript_id "lnc-bbut00020706"	exon_number "3"
17	lncRNA	exon	52094268	52094551	.	+	.	gene_id "lnc-bbug09003"	transcript_id "lnc-bbut00020711"	exon_number "1"
17	lncRNA	exon	52094584	52095766	.	+	.	gene_id "lnc-bbug09003"	transcript_id "lnc-bbut00020711"	exon_number "2"
17	lncRNA	exon	54765022	54766578	.	+	.	gene_id "lnc-bbug09009"	transcript_id "lnc-bbut00020723"	exon_number "1"
17	lncRNA	exon	54767143	54767205	.	+	.	gene_id "lnc-bbug09009"	transcript_id "lnc-bbut00020723"	exon_number "2"
17	lncRNA	exon	54804657	54805037	.	+	.	gene_id "lnc-bbug09011"	transcript_id "lnc-bbut00020727"	exon_number "1"
17	lncRNA	exon	55405929	55406136	.	+	.	gene_id "lnc-bbug09013"	transcript_id "lnc-bbut00020730"	exon_number "1"
17	lncRNA	exon	55408166	55408230	.	+	.	gene_id "lnc-bbug09013"	transcript_id "lnc-bbut00020730"	exon_number "2"
17	lncRNA	exon	55424890	55425350	.	+	.	gene_id "lnc-bbug09013"	transcript_id "lnc-bbut00020730"	exon_number "3"

17	lncRNA	exon	55590004	55590561	.	+	.	gene_id "lnc-bbug09014"	transcript_id "lnc-bbut00020731"	exon_number "1"
17	lncRNA	exon	55590759	55591050	.	+	.	gene_id "lnc-bbug09014"	transcript_id "lnc-bbut00020731"	exon_number "2"
17	lncRNA	exon	55591129	55591512	.	+	.	gene_id "lnc-bbug09014"	transcript_id "lnc-bbut00020731"	exon_number "3"
17	lncRNA	exon	55592633	55592682	.	+	.	gene_id "lnc-bbug09014"	transcript_id "lnc-bbut00020731"	exon_number "4"
17	lncRNA	exon	55592769	55593525	.	+	.	gene_id "lnc-bbug09014"	transcript_id "lnc-bbut00020731"	exon_number "5"
17	lncRNA	exon	55726447	55726754	.	+	.	gene_id "lnc-bbug09015"	transcript_id "lnc-bbut00020736"	exon_number "1"
17	lncRNA	exon	55734129	55734200	.	+	.	gene_id "lnc-bbug09015"	transcript_id "lnc-bbut00020736"	exon_number "2"
17	lncRNA	exon	55734566	55734649	.	+	.	gene_id "lnc-bbug09015"	transcript_id "lnc-bbut00020736"	exon_number "3"
17	lncRNA	exon	55736075	55737630	.	+	.	gene_id "lnc-bbug09015"	transcript_id "lnc-bbut00020736"	exon_number "4"
17	lncRNA	exon	56106087	56106222	.	+	.	gene_id "lnc-bbug09017"	transcript_id "lnc-bbut00020739"	exon_number "1"
17	lncRNA	exon	56112981	56113168	.	+	.	gene_id "lnc-bbug09017"	transcript_id "lnc-bbut00020739"	exon_number "2"
17	lncRNA	exon	56456767	56456837	.	+	.	gene_id "lnc-bbug09019"	transcript_id "lnc-bbut00020741"	exon_number "1"
17	lncRNA	exon	56464094	56464812	.	+	.	gene_id "lnc-bbug09019"	transcript_id "lnc-bbut00020741"	exon_number "2"
17	lncRNA	exon	56466035	56466261	.	+	.	gene_id "lnc-bbug09019"	transcript_id "lnc-bbut00020741"	exon_number "3"
17	lncRNA	exon	57356602	57356679	.	+	.	gene_id "lnc-bbug09022"	transcript_id "lnc-bbut00020755"	exon_number "1"
17	lncRNA	exon	57357049	57357841	.	+	.	gene_id "lnc-bbug09022"	transcript_id "lnc-bbut00020755"	exon_number "2"
17	lncRNA	exon	58079630	58079758	.	+	.	gene_id "lnc-bbug09023"	transcript_id "lnc-bbut00020756"	exon_number "1"
17	lncRNA	exon	58079898	58080398	.	+	.	gene_id "lnc-bbug09023"	transcript_id "lnc-bbut00020756"	exon_number "2"
17	lncRNA	exon	58452460	58452610	.	+	.	gene_id "lnc-bbug09025"	transcript_id "lnc-bbut00020758"	exon_number "1"
17	lncRNA	exon	58456941	58457305	.	+	.	gene_id "lnc-bbug09025"	transcript_id "lnc-bbut00020758"	exon_number "2"
17	lncRNA	exon	58804765	58804973	.	+	.	gene_id "lnc-bbug09028"	transcript_id "lnc-bbut00020765"	exon_number "1"
17	lncRNA	exon	58809759	58810813	.	+	.	gene_id "lnc-bbug09028"	transcript_id "lnc-bbut00020765"	exon_number "2"
17	lncRNA	exon	59970442	59970842	.	+	.	gene_id "lnc-bbug09032"	transcript_id "lnc-bbut00020771"	exon_number "1"
17	lncRNA	exon	59971473	59971739	.	+	.	gene_id "lnc-bbug09032"	transcript_id "lnc-bbut00020771"	exon_number "2"
17	lncRNA	exon	60907822	60907972	.	+	.	gene_id "lnc-bbug09037"	transcript_id "lnc-bbut00020785"	exon_number "1"
17	lncRNA	exon	60915989	60916104	.	+	.	gene_id "lnc-bbug09037"	transcript_id "lnc-bbut00020785"	exon_number "2"
17	lncRNA	exon	60919461	60919574	.	+	.	gene_id "lnc-bbug09037"	transcript_id "lnc-bbut00020785"	exon_number "3"
17	lncRNA	exon	60922548	60922938	.	+	.	gene_id "lnc-bbug09037"	transcript_id "lnc-bbut00020785"	exon_number "4"
17	lncRNA	exon	60908236	60908259	.	+	.	gene_id "lnc-bbug09037"	transcript_id "lnc-bbut00020786"	exon_number "1"
17	lncRNA	exon	60915989	60916104	.	+	.	gene_id "lnc-bbug09037"	transcript_id "lnc-bbut00020786"	exon_number "2"
17	lncRNA	exon	60919461	60919574	.	+	.	gene_id "lnc-bbug09037"	transcript_id "lnc-bbut00020786"	exon_number "3"
17	lncRNA	exon	60922548	60922938	.	+	.	gene_id "lnc-bbug09037"	transcript_id "lnc-bbut00020786"	exon_number "4"
17	lncRNA	exon	60978721	60979152	.	+	.	gene_id "lnc-bbug09039"	transcript_id "lnc-bbut00020788"	exon_number "1"
17	lncRNA	exon	60989768	60990788	.	+	.	gene_id "lnc-bbug09039"	transcript_id "lnc-bbut00020788"	exon_number "2"
17	lncRNA	exon	61294687	61295247	.	+	.	gene_id "lnc-bbug09040"	transcript_id "lnc-bbut00020789"	exon_number "1"
17	lncRNA	exon	61295339	61295451	.	+	.	gene_id "lnc-bbug09040"	transcript_id "lnc-bbut00020789"	exon_number "2"
17	lncRNA	exon	61819375	61819708	.	+	.	gene_id "lnc-bbug09041"	transcript_id "lnc-bbut00020792"	exon_number "1"
17	lncRNA	exon	61820549	61820725	.	+	.	gene_id "lnc-bbug09041"	transcript_id "lnc-bbut00020792"	exon_number "2"
17	lncRNA	exon	61821022	61826356	.	+	.	gene_id "lnc-bbug09041"	transcript_id "lnc-bbut00020792"	exon_number "3"
17	lncRNA	exon	61819375	61820109	.	+	.	gene_id "lnc-bbug09041"	transcript_id "lnc-bbut00020793"	exon_number "1"
17	lncRNA	exon	61820549	61820725	.	+	.	gene_id "lnc-bbug09041"	transcript_id "lnc-bbut00020793"	exon_number "2"
17	lncRNA	exon	61821022	61826356	.	+	.	gene_id "lnc-bbug09041"	transcript_id "lnc-bbut00020793"	exon_number "3"
17	lncRNA	exon	61819375	61820109	.	+	.	gene_id "lnc-bbug09041"	transcript_id "lnc-bbut00020791"	exon_number "1"
17	lncRNA	exon	61821022	61826356	.	+	.	gene_id "lnc-bbug09041"	transcript_id "lnc-bbut00020791"	exon_number "2"
17	lncRNA	exon	61819375	61819708	.	+	.	gene_id "lnc-bbug09041"	transcript_id "lnc-bbut00020790"	exon_number "1"
17	lncRNA	exon	61821022	61826356	.	+	.	gene_id "lnc-bbug09041"	transcript_id "lnc-bbut00020790"	exon_number "2"
17	lncRNA	exon	62569291	62569837	.	+	.	gene_id "lnc-bbug09044"	transcript_id "lnc-bbut00020797"	exon_number "1"
17	lncRNA	exon	62571880	62572032	.	+	.	gene_id "lnc-bbug09044"	transcript_id "lnc-bbut00020797"	exon_number "2"
17	lncRNA	exon	62577850	62578639	.	+	.	gene_id "lnc-bbug09044"	transcript_id "lnc-bbut00020797"	exon_number "3"
17	lncRNA	exon	62589313	62589902	.	+	.	gene_id "lnc-bbug09045"	transcript_id "lnc-bbut00020800"	exon_number "1"
17	lncRNA	exon	62593434	62593551	.	+	.	gene_id "lnc-bbug09045"	transcript_id "lnc-bbut00020800"	exon_number "2"
17	lncRNA	exon	62600614	62600735	.	+	.	gene_id "lnc-bbug09045"	transcript_id "lnc-bbut00020800"	exon_number "3"

17	lncRNA	exon	62602336	62603320	.	+	.	gene_id "lnc-bbug09045"	transcript_id "lnc-bbut00020800"	exon_number "4"
17	lncRNA	exon	62604434	62604487	.	+	.	gene_id "lnc-bbug09045"	transcript_id "lnc-bbut00020800"	exon_number "5"
17	lncRNA	exon	62605257	62605305	.	+	.	gene_id "lnc-bbug09045"	transcript_id "lnc-bbut00020800"	exon_number "6"
17	lncRNA	exon	62607064	62607312	.	+	.	gene_id "lnc-bbug09045"	transcript_id "lnc-bbut00020800"	exon_number "7"
17	lncRNA	exon	62589313	62589902	.	+	.	gene_id "lnc-bbug09045"	transcript_id "lnc-bbut00020799"	exon_number "1"
17	lncRNA	exon	62593434	62593551	.	+	.	gene_id "lnc-bbug09045"	transcript_id "lnc-bbut00020799"	exon_number "2"
17	lncRNA	exon	62600614	62600735	.	+	.	gene_id "lnc-bbug09045"	transcript_id "lnc-bbut00020799"	exon_number "3"
17	lncRNA	exon	62602336	62603070	.	+	.	gene_id "lnc-bbug09045"	transcript_id "lnc-bbut00020799"	exon_number "4"
17	lncRNA	exon	62603171	62604487	.	+	.	gene_id "lnc-bbug09045"	transcript_id "lnc-bbut00020799"	exon_number "5"
17	lncRNA	exon	62605257	62605305	.	+	.	gene_id "lnc-bbug09045"	transcript_id "lnc-bbut00020799"	exon_number "6"
17	lncRNA	exon	62607064	62607312	.	+	.	gene_id "lnc-bbug09045"	transcript_id "lnc-bbut00020799"	exon_number "7"
17	lncRNA	exon	62589313	62589902	.	+	.	gene_id "lnc-bbug09045"	transcript_id "lnc-bbut00020798"	exon_number "1"
17	lncRNA	exon	62593434	62593551	.	+	.	gene_id "lnc-bbug09045"	transcript_id "lnc-bbut00020798"	exon_number "2"
17	lncRNA	exon	62600614	62600735	.	+	.	gene_id "lnc-bbug09045"	transcript_id "lnc-bbut00020798"	exon_number "3"
17	lncRNA	exon	62602336	62602478	.	+	.	gene_id "lnc-bbug09045"	transcript_id "lnc-bbut00020798"	exon_number "4"
17	lncRNA	exon	62602906	62604487	.	+	.	gene_id "lnc-bbug09045"	transcript_id "lnc-bbut00020798"	exon_number "5"
17	lncRNA	exon	62605257	62605305	.	+	.	gene_id "lnc-bbug09045"	transcript_id "lnc-bbut00020798"	exon_number "6"
17	lncRNA	exon	62607064	62607312	.	+	.	gene_id "lnc-bbug09045"	transcript_id "lnc-bbut00020798"	exon_number "7"
17	lncRNA	exon	62608342	62608527	.	+	.	gene_id "lnc-bbug09046"	transcript_id "lnc-bbut00020801"	exon_number "1"
17	lncRNA	exon	62611561	62611747	.	+	.	gene_id "lnc-bbug09046"	transcript_id "lnc-bbut00020801"	exon_number "2"
17	lncRNA	exon	62616665	62617160	.	+	.	gene_id "lnc-bbug09046"	transcript_id "lnc-bbut00020801"	exon_number "3"
17	lncRNA	exon	63446606	63446843	.	+	.	gene_id "lnc-bbug09048"	transcript_id "lnc-bbut00020806"	exon_number "1"
17	lncRNA	exon	63447902	63448303	.	+	.	gene_id "lnc-bbug09048"	transcript_id "lnc-bbut00020806"	exon_number "2"
17	lncRNA	exon	64095900	64095957	.	+	.	gene_id "lnc-bbug09049"	transcript_id "lnc-bbut00020807"	exon_number "1"
17	lncRNA	exon	64171159	64171393	.	+	.	gene_id "lnc-bbug09049"	transcript_id "lnc-bbut00020807"	exon_number "2"
17	lncRNA	exon	67351863	67352679	.	+	.	gene_id "lnc-bbug09056"	transcript_id "lnc-bbut00020824"	exon_number "1"
17	lncRNA	exon	67354091	67354278	.	+	.	gene_id "lnc-bbug09056"	transcript_id "lnc-bbut00020824"	exon_number "2"
17	lncRNA	exon	67355699	67355982	.	+	.	gene_id "lnc-bbug09056"	transcript_id "lnc-bbut00020824"	exon_number "3"
17	lncRNA	exon	68037389	68038604	.	+	.	gene_id "lnc-bbug09058"	transcript_id "lnc-bbut00020826"	exon_number "1"
17	lncRNA	exon	68042607	68051951	.	+	.	gene_id "lnc-bbug09058"	transcript_id "lnc-bbut00020826"	exon_number "2"
17	lncRNA	exon	68055021	68055134	.	+	.	gene_id "lnc-bbug09059"	transcript_id "lnc-bbut00020827"	exon_number "1"
17	lncRNA	exon	68067282	68067469	.	+	.	gene_id "lnc-bbug09059"	transcript_id "lnc-bbut00020827"	exon_number "2"
17	lncRNA	exon	68072731	68074309	.	+	.	gene_id "lnc-bbug09059"	transcript_id "lnc-bbut00020827"	exon_number "3"
17	lncRNA	exon	68055032	68055134	.	+	.	gene_id "lnc-bbug09059"	transcript_id "lnc-bbut00020828"	exon_number "1"
17	lncRNA	exon	68072731	68074309	.	+	.	gene_id "lnc-bbug09059"	transcript_id "lnc-bbut00020828"	exon_number "2"
17	lncRNA	exon	68055197	68055236	.	+	.	gene_id "lnc-bbug09059"	transcript_id "lnc-bbut00020829"	exon_number "1"
17	lncRNA	exon	68067282	68067469	.	+	.	gene_id "lnc-bbug09059"	transcript_id "lnc-bbut00020829"	exon_number "2"
17	lncRNA	exon	68073245	68074309	.	+	.	gene_id "lnc-bbug09059"	transcript_id "lnc-bbut00020829"	exon_number "3"
17	lncRNA	exon	68273644	68274357	.	+	.	gene_id "lnc-bbug09061"	transcript_id "lnc-bbut00020838"	exon_number "1"
17	lncRNA	exon	68274614	68274770	.	+	.	gene_id "lnc-bbug09061"	transcript_id "lnc-bbut00020838"	exon_number "2"
17	lncRNA	exon	69107732	69108753	.	+	.	gene_id "lnc-bbug09067"	transcript_id "lnc-bbut00020867"	exon_number "1"
17	lncRNA	exon	69191096	69191187	.	+	.	gene_id "lnc-bbug09067"	transcript_id "lnc-bbut00020867"	exon_number "2"
17	lncRNA	exon	69192806	69193087	.	+	.	gene_id "lnc-bbug09067"	transcript_id "lnc-bbut00020867"	exon_number "3"
17	lncRNA	exon	69401838	69403889	.	+	.	gene_id "lnc-bbug09068"	transcript_id "lnc-bbut00020868"	exon_number "1"
17	lncRNA	exon	69407588	69407789	.	+	.	gene_id "lnc-bbug09068"	transcript_id "lnc-bbut00020868"	exon_number "2"
17	lncRNA	exon	69408050	69408168	.	+	.	gene_id "lnc-bbug09068"	transcript_id "lnc-bbut00020868"	exon_number "3"
17	lncRNA	exon	69437317	69437966	.	+	.	gene_id "lnc-bbug09069"	transcript_id "lnc-bbut00020869"	exon_number "1"
17	lncRNA	exon	69450147	69450328	.	+	.	gene_id "lnc-bbug09069"	transcript_id "lnc-bbut00020869"	exon_number "2"
17	lncRNA	exon	69450977	69451083	.	+	.	gene_id "lnc-bbug09069"	transcript_id "lnc-bbut00020869"	exon_number "3"
17	lncRNA	exon	70179801	70180364	.	+	.	gene_id "lnc-bbug09072"	transcript_id "lnc-bbut00020872"	exon_number "1"
17	lncRNA	exon	70180967	70181547	.	+	.	gene_id "lnc-bbug09072"	transcript_id "lnc-bbut00020872"	exon_number "2"
17	lncRNA	exon	70183890	70186800	.	+	.	gene_id "lnc-bbug09072"	transcript_id "lnc-bbut00020872"	exon_number "3"

17	lncRNA	exon	71005900	71006280	.	+	.	gene_id "lnc-bbug09076"	transcript_id "lnc-bbut00020882"	exon_number "1"
17	lncRNA	exon	71006533	71006725	.	+	.	gene_id "lnc-bbug09076"	transcript_id "lnc-bbut00020882"	exon_number "2"
17	lncRNA	exon	72394122	72394267	.	+	.	gene_id "lnc-bbug09077"	transcript_id "lnc-bbut00020883"	exon_number "1"
17	lncRNA	exon	72397889	72398885	.	+	.	gene_id "lnc-bbug09077"	transcript_id "lnc-bbut00020883"	exon_number "2"
17	lncRNA	exon	72644639	72644977	.	+	.	gene_id "lnc-bbug09078"	transcript_id "lnc-bbut00020884"	exon_number "1"
17	lncRNA	exon	72869012	72869956	.	+	.	gene_id "lnc-bbug09084"	transcript_id "lnc-bbut00020893"	exon_number "1"
17	lncRNA	exon	72876739	72876749	.	+	.	gene_id "lnc-bbug09084"	transcript_id "lnc-bbut00020893"	exon_number "2"
17	lncRNA	exon	72963250	72963418	.	+	.	gene_id "lnc-bbug09090"	transcript_id "lnc-bbut00020899"	exon_number "1"
17	lncRNA	exon	72965917	72966195	.	+	.	gene_id "lnc-bbug09090"	transcript_id "lnc-bbut00020899"	exon_number "2"
17	lncRNA	exon	73160468	73160803	.	+	.	gene_id "lnc-bbug09092"	transcript_id "lnc-bbut00020901"	exon_number "1"
17	lncRNA	exon	73163032	73163171	.	+	.	gene_id "lnc-bbug09092"	transcript_id "lnc-bbut00020901"	exon_number "2"
17	lncRNA	exon	73163386	73163493	.	+	.	gene_id "lnc-bbug09092"	transcript_id "lnc-bbut00020901"	exon_number "3"
17	lncRNA	exon	73163621	73163829	.	+	.	gene_id "lnc-bbug09092"	transcript_id "lnc-bbut00020901"	exon_number "4"
17	lncRNA	exon	73168202	73168689	.	+	.	gene_id "lnc-bbug09092"	transcript_id "lnc-bbut00020901"	exon_number "5"
17	lncRNA	exon	73197037	73197173	.	+	.	gene_id "lnc-bbug09093"	transcript_id "lnc-bbut00020902"	exon_number "1"
17	lncRNA	exon	73199894	73199922	.	+	.	gene_id "lnc-bbug09093"	transcript_id "lnc-bbut00020902"	exon_number "2"
17	lncRNA	exon	73211664	73211747	.	+	.	gene_id "lnc-bbug09093"	transcript_id "lnc-bbut00020902"	exon_number "3"
17	lncRNA	exon	73214723	73216868	.	+	.	gene_id "lnc-bbug09093"	transcript_id "lnc-bbut00020902"	exon_number "4"
17	lncRNA	exon	111802	114492	.	-	.	gene_id "lnc-bbug09096"	transcript_id "lnc-bbut00020907"	exon_number "1"
17	lncRNA	exon	114775	114953	.	-	.	gene_id "lnc-bbug09096"	transcript_id "lnc-bbut00020907"	exon_number "2"
17	lncRNA	exon	118008	118118	.	-	.	gene_id "lnc-bbug09096"	transcript_id "lnc-bbut00020907"	exon_number "3"
17	lncRNA	exon	399288	402334	.	-	.	gene_id "lnc-bbug09105"	transcript_id "lnc-bbut00020933"	exon_number "1"
17	lncRNA	exon	403246	403373	.	-	.	gene_id "lnc-bbug09105"	transcript_id "lnc-bbut00020933"	exon_number "2"
17	lncRNA	exon	516425	517689	.	-	.	gene_id "lnc-bbug09108"	transcript_id "lnc-bbut00020942"	exon_number "1"
17	lncRNA	exon	518129	518453	.	-	.	gene_id "lnc-bbug09108"	transcript_id "lnc-bbut00020942"	exon_number "2"
17	lncRNA	exon	516425	517689	.	-	.	gene_id "lnc-bbug09108"	transcript_id "lnc-bbut00020941"	exon_number "1"
17	lncRNA	exon	518120	518453	.	-	.	gene_id "lnc-bbug09108"	transcript_id "lnc-bbut00020941"	exon_number "2"
17	lncRNA	exon	759953	764692	.	-	.	gene_id "lnc-bbug09116"	transcript_id "lnc-bbut00020960"	exon_number "1"
17	lncRNA	exon	764950	765355	.	-	.	gene_id "lnc-bbug09116"	transcript_id "lnc-bbut00020960"	exon_number "2"
17	lncRNA	exon	792408	794234	.	-	.	gene_id "lnc-bbug09118"	transcript_id "lnc-bbut00020962"	exon_number "1"
17	lncRNA	exon	794923	795210	.	-	.	gene_id "lnc-bbug09118"	transcript_id "lnc-bbut00020962"	exon_number "2"
17	lncRNA	exon	1025090	1025166	.	-	.	gene_id "lnc-bbug09128"	transcript_id "lnc-bbut00020981"	exon_number "1"
17	lncRNA	exon	1026830	1026913	.	-	.	gene_id "lnc-bbug09128"	transcript_id "lnc-bbut00020981"	exon_number "2"
17	lncRNA	exon	1027042	1027409	.	-	.	gene_id "lnc-bbug09128"	transcript_id "lnc-bbut00020981"	exon_number "3"
17	lncRNA	exon	1059891	1061713	.	-	.	gene_id "lnc-bbug09129"	transcript_id "lnc-bbut00020983"	exon_number "1"
17	lncRNA	exon	1062268	1062376	.	-	.	gene_id "lnc-bbug09129"	transcript_id "lnc-bbut00020983"	exon_number "2"
17	lncRNA	exon	1062663	1062706	.	-	.	gene_id "lnc-bbug09129"	transcript_id "lnc-bbut00020983"	exon_number "3"
17	lncRNA	exon	1063464	1064277	.	-	.	gene_id "lnc-bbug09129"	transcript_id "lnc-bbut00020983"	exon_number "4"
17	lncRNA	exon	1064410	1066298	.	-	.	gene_id "lnc-bbug09129"	transcript_id "lnc-bbut00020983"	exon_number "5"
17	lncRNA	exon	1059891	1061713	.	-	.	gene_id "lnc-bbug09129"	transcript_id "lnc-bbut00020982"	exon_number "1"
17	lncRNA	exon	1062268	1062376	.	-	.	gene_id "lnc-bbug09129"	transcript_id "lnc-bbut00020982"	exon_number "2"
17	lncRNA	exon	1062663	1062706	.	-	.	gene_id "lnc-bbug09129"	transcript_id "lnc-bbut00020982"	exon_number "3"
17	lncRNA	exon	1063464	1063741	.	-	.	gene_id "lnc-bbug09129"	transcript_id "lnc-bbut00020982"	exon_number "4"
17	lncRNA	exon	1064410	1066298	.	-	.	gene_id "lnc-bbug09129"	transcript_id "lnc-bbut00020982"	exon_number "5"
17	lncRNA	exon	1059891	1061768	.	-	.	gene_id "lnc-bbug09129"	transcript_id "lnc-bbut00020984"	exon_number "1"
17	lncRNA	exon	1062268	1062376	.	-	.	gene_id "lnc-bbug09129"	transcript_id "lnc-bbut00020984"	exon_number "2"
17	lncRNA	exon	1062663	1062706	.	-	.	gene_id "lnc-bbug09129"	transcript_id "lnc-bbut00020984"	exon_number "3"
17	lncRNA	exon	1063464	1066312	.	-	.	gene_id "lnc-bbug09129"	transcript_id "lnc-bbut00020984"	exon_number "4"
17	lncRNA	exon	1059891	1061713	.	-	.	gene_id "lnc-bbug09129"	transcript_id "lnc-bbut00020985"	exon_number "1"
17	lncRNA	exon	1062268	1062376	.	-	.	gene_id "lnc-bbug09129"	transcript_id "lnc-bbut00020985"	exon_number "2"
17	lncRNA	exon	1062663	1062706	.	-	.	gene_id "lnc-bbug09129"	transcript_id "lnc-bbut00020985"	exon_number "3"
17	lncRNA	exon	1063464	1063741	.	-	.	gene_id "lnc-bbug09129"	transcript_id "lnc-bbut00020985"	exon_number "4"

17	lncRNA	exon	1068634	1069480	.	-	.	gene_id "lnc-bbug09129"	transcript_id "lnc-bbut00020985"	exon_number "5"
17	lncRNA	exon	1059891	1061713	.	-	.	gene_id "lnc-bbug09129"	transcript_id "lnc-bbut00020987"	exon_number "1"
17	lncRNA	exon	1062268	1062376	.	-	.	gene_id "lnc-bbug09129"	transcript_id "lnc-bbut00020987"	exon_number "2"
17	lncRNA	exon	1062663	1062706	.	-	.	gene_id "lnc-bbug09129"	transcript_id "lnc-bbut00020987"	exon_number "3"
17	lncRNA	exon	1063464	1063741	.	-	.	gene_id "lnc-bbug09129"	transcript_id "lnc-bbut00020987"	exon_number "4"
17	lncRNA	exon	1073467	1073618	.	-	.	gene_id "lnc-bbug09129"	transcript_id "lnc-bbut00020987"	exon_number "5"
17	lncRNA	exon	1059891	1061713	.	-	.	gene_id "lnc-bbug09129"	transcript_id "lnc-bbut00020988"	exon_number "1"
17	lncRNA	exon	1062268	1062376	.	-	.	gene_id "lnc-bbug09129"	transcript_id "lnc-bbut00020988"	exon_number "2"
17	lncRNA	exon	1062663	1062706	.	-	.	gene_id "lnc-bbug09129"	transcript_id "lnc-bbut00020988"	exon_number "3"
17	lncRNA	exon	1063464	1063741	.	-	.	gene_id "lnc-bbug09129"	transcript_id "lnc-bbut00020988"	exon_number "4"
17	lncRNA	exon	1077650	1077666	.	-	.	gene_id "lnc-bbug09129"	transcript_id "lnc-bbut00020988"	exon_number "5"
17	lncRNA	exon	1580682	1580893	.	-	.	gene_id "lnc-bbug09135"	transcript_id "lnc-bbut00020997"	exon_number "1"
17	lncRNA	exon	1581313	1581440	.	-	.	gene_id "lnc-bbug09135"	transcript_id "lnc-bbut00020997"	exon_number "2"
17	lncRNA	exon	1604117	1604356	.	-	.	gene_id "lnc-bbug09135"	transcript_id "lnc-bbut00020997"	exon_number "3"
17	lncRNA	exon	1672301	1673130	.	-	.	gene_id "lnc-bbug09137"	transcript_id "lnc-bbut00021005"	exon_number "1"
17	lncRNA	exon	1673386	1673483	.	-	.	gene_id "lnc-bbug09137"	transcript_id "lnc-bbut00021005"	exon_number "2"
17	lncRNA	exon	1673981	1674229	.	-	.	gene_id "lnc-bbug09137"	transcript_id "lnc-bbut00021005"	exon_number "3"
17	lncRNA	exon	1814280	1814739	.	-	.	gene_id "lnc-bbug09143"	transcript_id "lnc-bbut00021017"	exon_number "1"
17	lncRNA	exon	1819463	1819550	.	-	.	gene_id "lnc-bbug09143"	transcript_id "lnc-bbut00021017"	exon_number "2"
17	lncRNA	exon	1823125	1823336	.	-	.	gene_id "lnc-bbug09143"	transcript_id "lnc-bbut00021017"	exon_number "3"
17	lncRNA	exon	1814280	1814739	.	-	.	gene_id "lnc-bbug09143"	transcript_id "lnc-bbut00021018"	exon_number "1"
17	lncRNA	exon	1823125	1823358	.	-	.	gene_id "lnc-bbug09143"	transcript_id "lnc-bbut00021018"	exon_number "2"
17	lncRNA	exon	2151118	2151584	.	-	.	gene_id "lnc-bbug09151"	transcript_id "lnc-bbut00021039"	exon_number "1"
17	lncRNA	exon	2153715	2153889	.	-	.	gene_id "lnc-bbug09151"	transcript_id "lnc-bbut00021039"	exon_number "2"
17	lncRNA	exon	2174271	2174470	.	-	.	gene_id "lnc-bbug09151"	transcript_id "lnc-bbut00021040"	exon_number "1"
17	lncRNA	exon	2174883	2176048	.	-	.	gene_id "lnc-bbug09151"	transcript_id "lnc-bbut00021040"	exon_number "2"
17	lncRNA	exon	2423129	2423646	.	-	.	gene_id "lnc-bbug09154"	transcript_id "lnc-bbut00021045"	exon_number "1"
17	lncRNA	exon	2440727	2440822	.	-	.	gene_id "lnc-bbug09154"	transcript_id "lnc-bbut00021045"	exon_number "2"
17	lncRNA	exon	2481390	2481819	.	-	.	gene_id "lnc-bbug09163"	transcript_id "lnc-bbut00021054"	exon_number "1"
17	lncRNA	exon	2481929	2481989	.	-	.	gene_id "lnc-bbug09163"	transcript_id "lnc-bbut00021054"	exon_number "2"
17	lncRNA	exon	2740186	2740386	.	-	.	gene_id "lnc-bbug09167"	transcript_id "lnc-bbut00021059"	exon_number "1"
17	lncRNA	exon	3048822	3050478	.	-	.	gene_id "lnc-bbug09170"	transcript_id "lnc-bbut00021064"	exon_number "1"
17	lncRNA	exon	3062187	3062400	.	-	.	gene_id "lnc-bbug09170"	transcript_id "lnc-bbut00021064"	exon_number "2"
17	lncRNA	exon	3705995	3706239	.	-	.	gene_id "lnc-bbug09180"	transcript_id "lnc-bbut00021089"	exon_number "1"
17	lncRNA	exon	3707241	3707272	.	-	.	gene_id "lnc-bbug09180"	transcript_id "lnc-bbut00021089"	exon_number "2"
17	lncRNA	exon	3707491	3707629	.	-	.	gene_id "lnc-bbug09180"	transcript_id "lnc-bbut00021089"	exon_number "3"
17	lncRNA	exon	4272659	4272922	.	-	.	gene_id "lnc-bbug09186"	transcript_id "lnc-bbut00021119"	exon_number "1"
17	lncRNA	exon	4274666	4274849	.	-	.	gene_id "lnc-bbug09186"	transcript_id "lnc-bbut00021119"	exon_number "2"
17	lncRNA	exon	4272659	4272922	.	-	.	gene_id "lnc-bbug09186"	transcript_id "lnc-bbut00021120"	exon_number "1"
17	lncRNA	exon	4274606	4274864	.	-	.	gene_id "lnc-bbug09186"	transcript_id "lnc-bbut00021120"	exon_number "2"
17	lncRNA	exon	4773572	4775112	.	-	.	gene_id "lnc-bbug09195"	transcript_id "lnc-bbut00021142"	exon_number "1"
17	lncRNA	exon	4776725	4778381	.	-	.	gene_id "lnc-bbug09195"	transcript_id "lnc-bbut00021142"	exon_number "2"
17	lncRNA	exon	4936309	4937021	.	-	.	gene_id "lnc-bbug09196"	transcript_id "lnc-bbut00021143"	exon_number "1"
17	lncRNA	exon	4937139	4937209	.	-	.	gene_id "lnc-bbug09196"	transcript_id "lnc-bbut00021143"	exon_number "2"
17	lncRNA	exon	4945720	4945807	.	-	.	gene_id "lnc-bbug09196"	transcript_id "lnc-bbut00021143"	exon_number "3"
17	lncRNA	exon	5638938	5639417	.	-	.	gene_id "lnc-bbug09199"	transcript_id "lnc-bbut00021150"	exon_number "1"
17	lncRNA	exon	5641358	5641787	.	-	.	gene_id "lnc-bbug09199"	transcript_id "lnc-bbut00021150"	exon_number "2"
17	lncRNA	exon	5668670	5668807	.	-	.	gene_id "lnc-bbug09199"	transcript_id "lnc-bbut00021150"	exon_number "3"
17	lncRNA	exon	5890123	5892362	.	-	.	gene_id "lnc-bbug09200"	transcript_id "lnc-bbut00021151"	exon_number "1"
17	lncRNA	exon	5893032	5893272	.	-	.	gene_id "lnc-bbug09200"	transcript_id "lnc-bbut00021151"	exon_number "2"
17	lncRNA	exon	5893352	5894373	.	-	.	gene_id "lnc-bbug09200"	transcript_id "lnc-bbut00021151"	exon_number "3"
17	lncRNA	exon	6687811	6689689	.	-	.	gene_id "lnc-bbug09201"	transcript_id "lnc-bbut00021152"	exon_number "1"

17	lncRNA	exon	6690119	6690203	.	-	.	gene_id "lnc-bbug09201"	transcript_id "lnc-bbut00021152"	exon_number "2"
17	lncRNA	exon	6690443	6692014	.	-	.	gene_id "lnc-bbug09201"	transcript_id "lnc-bbut00021152"	exon_number "3"
17	lncRNA	exon	6693530	6693616	.	-	.	gene_id "lnc-bbug09201"	transcript_id "lnc-bbut00021152"	exon_number "4"
17	lncRNA	exon	6697605	6697674	.	-	.	gene_id "lnc-bbug09201"	transcript_id "lnc-bbut00021152"	exon_number "5"
17	lncRNA	exon	6698332	6698457	.	-	.	gene_id "lnc-bbug09201"	transcript_id "lnc-bbut00021152"	exon_number "6"
17	lncRNA	exon	6687811	6689689	.	-	.	gene_id "lnc-bbug09201"	transcript_id "lnc-bbut00021153"	exon_number "1"
17	lncRNA	exon	6690119	6690203	.	-	.	gene_id "lnc-bbug09201"	transcript_id "lnc-bbut00021153"	exon_number "2"
17	lncRNA	exon	6690443	6692014	.	-	.	gene_id "lnc-bbug09201"	transcript_id "lnc-bbut00021153"	exon_number "3"
17	lncRNA	exon	6693530	6693616	.	-	.	gene_id "lnc-bbug09201"	transcript_id "lnc-bbut00021153"	exon_number "4"
17	lncRNA	exon	6697529	6697674	.	-	.	gene_id "lnc-bbug09201"	transcript_id "lnc-bbut00021153"	exon_number "5"
17	lncRNA	exon	6698332	6698458	.	-	.	gene_id "lnc-bbug09201"	transcript_id "lnc-bbut00021153"	exon_number "6"
17	lncRNA	exon	6687811	6689689	.	-	.	gene_id "lnc-bbug09201"	transcript_id "lnc-bbut00021157"	exon_number "1"
17	lncRNA	exon	6690119	6690208	.	-	.	gene_id "lnc-bbug09201"	transcript_id "lnc-bbut00021157"	exon_number "2"
17	lncRNA	exon	6690443	6690496	.	-	.	gene_id "lnc-bbug09201"	transcript_id "lnc-bbut00021157"	exon_number "3"
17	lncRNA	exon	6691131	6692014	.	-	.	gene_id "lnc-bbug09201"	transcript_id "lnc-bbut00021157"	exon_number "4"
17	lncRNA	exon	6693530	6693616	.	-	.	gene_id "lnc-bbug09201"	transcript_id "lnc-bbut00021157"	exon_number "5"
17	lncRNA	exon	6698332	6698463	.	-	.	gene_id "lnc-bbug09201"	transcript_id "lnc-bbut00021157"	exon_number "6"
17	lncRNA	exon	6687811	6689689	.	-	.	gene_id "lnc-bbug09201"	transcript_id "lnc-bbut00021156"	exon_number "1"
17	lncRNA	exon	6690119	6690203	.	-	.	gene_id "lnc-bbug09201"	transcript_id "lnc-bbut00021156"	exon_number "2"
17	lncRNA	exon	6690443	6690496	.	-	.	gene_id "lnc-bbug09201"	transcript_id "lnc-bbut00021156"	exon_number "3"
17	lncRNA	exon	6691131	6692014	.	-	.	gene_id "lnc-bbug09201"	transcript_id "lnc-bbut00021156"	exon_number "4"
17	lncRNA	exon	6693530	6693616	.	-	.	gene_id "lnc-bbug09201"	transcript_id "lnc-bbut00021156"	exon_number "5"
17	lncRNA	exon	6698332	6698463	.	-	.	gene_id "lnc-bbug09201"	transcript_id "lnc-bbut00021156"	exon_number "6"
17	lncRNA	exon	6687811	6689689	.	-	.	gene_id "lnc-bbug09201"	transcript_id "lnc-bbut00021155"	exon_number "1"
17	lncRNA	exon	6690119	6690208	.	-	.	gene_id "lnc-bbug09201"	transcript_id "lnc-bbut00021155"	exon_number "2"
17	lncRNA	exon	6690443	6692014	.	-	.	gene_id "lnc-bbug09201"	transcript_id "lnc-bbut00021155"	exon_number "3"
17	lncRNA	exon	6693530	6693616	.	-	.	gene_id "lnc-bbug09201"	transcript_id "lnc-bbut00021155"	exon_number "4"
17	lncRNA	exon	6698332	6698463	.	-	.	gene_id "lnc-bbug09201"	transcript_id "lnc-bbut00021155"	exon_number "5"
17	lncRNA	exon	6687811	6689689	.	-	.	gene_id "lnc-bbug09201"	transcript_id "lnc-bbut00021154"	exon_number "1"
17	lncRNA	exon	6690119	6690203	.	-	.	gene_id "lnc-bbug09201"	transcript_id "lnc-bbut00021154"	exon_number "2"
17	lncRNA	exon	6690443	6692014	.	-	.	gene_id "lnc-bbug09201"	transcript_id "lnc-bbut00021154"	exon_number "3"
17	lncRNA	exon	6693530	6693616	.	-	.	gene_id "lnc-bbug09201"	transcript_id "lnc-bbut00021154"	exon_number "4"
17	lncRNA	exon	6698332	6698463	.	-	.	gene_id "lnc-bbug09201"	transcript_id "lnc-bbut00021154"	exon_number "5"
17	lncRNA	exon	6687811	6689689	.	-	.	gene_id "lnc-bbug09201"	transcript_id "lnc-bbut00021158"	exon_number "1"
17	lncRNA	exon	6690119	6690203	.	-	.	gene_id "lnc-bbug09201"	transcript_id "lnc-bbut00021158"	exon_number "2"
17	lncRNA	exon	6690443	6692014	.	-	.	gene_id "lnc-bbug09201"	transcript_id "lnc-bbut00021158"	exon_number "3"
17	lncRNA	exon	6693530	6693772	.	-	.	gene_id "lnc-bbug09201"	transcript_id "lnc-bbut00021158"	exon_number "4"
17	lncRNA	exon	6698332	6698468	.	-	.	gene_id "lnc-bbug09201"	transcript_id "lnc-bbut00021158"	exon_number "5"
17	lncRNA	exon	7891443	7892659	.	-	.	gene_id "lnc-bbug09212"	transcript_id "lnc-bbut00021178"	exon_number "1"
17	lncRNA	exon	7895094	7896205	.	-	.	gene_id "lnc-bbug09212"	transcript_id "lnc-bbut00021178"	exon_number "2"
17	lncRNA	exon	7896325	7896915	.	-	.	gene_id "lnc-bbug09212"	transcript_id "lnc-bbut00021178"	exon_number "3"
17	lncRNA	exon	7897443	7897513	.	-	.	gene_id "lnc-bbug09212"	transcript_id "lnc-bbut00021178"	exon_number "4"
17	lncRNA	exon	7901821	7901973	.	-	.	gene_id "lnc-bbug09212"	transcript_id "lnc-bbut00021178"	exon_number "5"
17	lncRNA	exon	7907970	7908977	.	-	.	gene_id "lnc-bbug09212"	transcript_id "lnc-bbut00021178"	exon_number "6"
17	lncRNA	exon	7990191	7990340	.	-	.	gene_id "lnc-bbug09214"	transcript_id "lnc-bbut00021185"	exon_number "1"
17	lncRNA	exon	7991528	7991597	.	-	.	gene_id "lnc-bbug09214"	transcript_id "lnc-bbut00021185"	exon_number "2"
17	lncRNA	exon	8011937	8012003	.	-	.	gene_id "lnc-bbug09214"	transcript_id "lnc-bbut00021185"	exon_number "3"
17	lncRNA	exon	8026027	8027658	.	-	.	gene_id "lnc-bbug09214"	transcript_id "lnc-bbut00021185"	exon_number "4"
17	lncRNA	exon	8698447	8698868	.	-	.	gene_id "lnc-bbug09218"	transcript_id "lnc-bbut00021202"	exon_number "1"
17	lncRNA	exon	8699131	8699208	.	-	.	gene_id "lnc-bbug09218"	transcript_id "lnc-bbut00021202"	exon_number "2"
17	lncRNA	exon	8700735	8700868	.	-	.	gene_id "lnc-bbug09218"	transcript_id "lnc-bbut00021202"	exon_number "3"
17	lncRNA	exon	8740347	8741397	.	-	.	gene_id "lnc-bbug09220"	transcript_id "lnc-bbut00021204"	exon_number "1"

17	lncRNA	exon	8742553	8743140	.	-	.	gene_id "lnc-bbug09220"	transcript_id "lnc-bbut00021204"	exon_number "2"
17	lncRNA	exon	8774824	8781730	.	-	.	gene_id "lnc-bbug09221"	transcript_id "lnc-bbut00021205"	exon_number "1"
17	lncRNA	exon	9543966	9544211	.	-	.	gene_id "lnc-bbug09228"	transcript_id "lnc-bbut00021221"	exon_number "1"
17	lncRNA	exon	9544413	9544621	.	-	.	gene_id "lnc-bbug09228"	transcript_id "lnc-bbut00021221"	exon_number "2"
17	lncRNA	exon	9545686	9545921	.	-	.	gene_id "lnc-bbug09228"	transcript_id "lnc-bbut00021221"	exon_number "3"
17	lncRNA	exon	9547034	9547739	.	-	.	gene_id "lnc-bbug09228"	transcript_id "lnc-bbut00021221"	exon_number "4"
17	lncRNA	exon	9543966	9544211	.	-	.	gene_id "lnc-bbug09228"	transcript_id "lnc-bbut00021220"	exon_number "1"
17	lncRNA	exon	9544413	9545921	.	-	.	gene_id "lnc-bbug09228"	transcript_id "lnc-bbut00021220"	exon_number "2"
17	lncRNA	exon	9547034	9547739	.	-	.	gene_id "lnc-bbug09228"	transcript_id "lnc-bbut00021220"	exon_number "3"
17	lncRNA	exon	9543966	9544211	.	-	.	gene_id "lnc-bbug09228"	transcript_id "lnc-bbut00021222"	exon_number "1"
17	lncRNA	exon	9544413	9545921	.	-	.	gene_id "lnc-bbug09228"	transcript_id "lnc-bbut00021222"	exon_number "2"
17	lncRNA	exon	9547034	9547194	.	-	.	gene_id "lnc-bbug09228"	transcript_id "lnc-bbut00021222"	exon_number "3"
17	lncRNA	exon	9569680	9570456	.	-	.	gene_id "lnc-bbug09228"	transcript_id "lnc-bbut00021222"	exon_number "4"
17	lncRNA	exon	9997244	9997467	.	-	.	gene_id "lnc-bbug09235"	transcript_id "lnc-bbut00021243"	exon_number "1"
17	lncRNA	exon	9998324	9998506	.	-	.	gene_id "lnc-bbug09235"	transcript_id "lnc-bbut00021243"	exon_number "2"
17	lncRNA	exon	10016035	10016481	.	-	.	gene_id "lnc-bbug09235"	transcript_id "lnc-bbut00021243"	exon_number "3"
17	lncRNA	exon	10008557	10010209	.	-	.	gene_id "lnc-bbug09235"	transcript_id "lnc-bbut00021244"	exon_number "1"
17	lncRNA	exon	10011303	10011377	.	-	.	gene_id "lnc-bbug09235"	transcript_id "lnc-bbut00021244"	exon_number "2"
17	lncRNA	exon	10016035	10016481	.	-	.	gene_id "lnc-bbug09235"	transcript_id "lnc-bbut00021244"	exon_number "3"
17	lncRNA	exon	10233240	10233667	.	-	.	gene_id "lnc-bbug09241"	transcript_id "lnc-bbut00021256"	exon_number "1"
17	lncRNA	exon	10235244	10235381	.	-	.	gene_id "lnc-bbug09241"	transcript_id "lnc-bbut00021256"	exon_number "2"
17	lncRNA	exon	10235587	10235737	.	-	.	gene_id "lnc-bbug09241"	transcript_id "lnc-bbut00021256"	exon_number "3"
17	lncRNA	exon	10434306	10436142	.	-	.	gene_id "lnc-bbug09247"	transcript_id "lnc-bbut00021267"	exon_number "1"
17	lncRNA	exon	10436804	10437799	.	-	.	gene_id "lnc-bbug09247"	transcript_id "lnc-bbut00021267"	exon_number "2"
17	lncRNA	exon	10661202	10661323	.	-	.	gene_id "lnc-bbug09250"	transcript_id "lnc-bbut00021275"	exon_number "1"
17	lncRNA	exon	10671162	10671375	.	-	.	gene_id "lnc-bbug09250"	transcript_id "lnc-bbut00021275"	exon_number "2"
17	lncRNA	exon	10683438	10683775	.	-	.	gene_id "lnc-bbug09250"	transcript_id "lnc-bbut00021275"	exon_number "3"
17	lncRNA	exon	10691712	10691921	.	-	.	gene_id "lnc-bbug09250"	transcript_id "lnc-bbut00021275"	exon_number "4"
17	lncRNA	exon	10715333	10718288	.	-	.	gene_id "lnc-bbug09250"	transcript_id "lnc-bbut00021275"	exon_number "5"
17	lncRNA	exon	10718613	10718699	.	-	.	gene_id "lnc-bbug09250"	transcript_id "lnc-bbut00021275"	exon_number "6"
17	lncRNA	exon	10730491	10730599	.	-	.	gene_id "lnc-bbug09250"	transcript_id "lnc-bbut00021275"	exon_number "7"
17	lncRNA	exon	10735058	10735146	.	-	.	gene_id "lnc-bbug09250"	transcript_id "lnc-bbut00021275"	exon_number "8"
17	lncRNA	exon	10735254	10735456	.	-	.	gene_id "lnc-bbug09250"	transcript_id "lnc-bbut00021275"	exon_number "9"
17	lncRNA	exon	10661202	10661323	.	-	.	gene_id "lnc-bbug09250"	transcript_id "lnc-bbut00021276"	exon_number "1"
17	lncRNA	exon	10671162	10671375	.	-	.	gene_id "lnc-bbug09250"	transcript_id "lnc-bbut00021276"	exon_number "2"
17	lncRNA	exon	10683438	10683775	.	-	.	gene_id "lnc-bbug09250"	transcript_id "lnc-bbut00021276"	exon_number "3"
17	lncRNA	exon	10691712	10691921	.	-	.	gene_id "lnc-bbug09250"	transcript_id "lnc-bbut00021276"	exon_number "4"
17	lncRNA	exon	10715333	10718288	.	-	.	gene_id "lnc-bbug09250"	transcript_id "lnc-bbut00021276"	exon_number "5"
17	lncRNA	exon	10718613	10718699	.	-	.	gene_id "lnc-bbug09250"	transcript_id "lnc-bbut00021276"	exon_number "6"
17	lncRNA	exon	10735058	10735146	.	-	.	gene_id "lnc-bbug09250"	transcript_id "lnc-bbut00021276"	exon_number "7"
17	lncRNA	exon	10735304	10735457	.	-	.	gene_id "lnc-bbug09250"	transcript_id "lnc-bbut00021276"	exon_number "8"
17	lncRNA	exon	10727815	10728764	.	-	.	gene_id "lnc-bbug09250"	transcript_id "lnc-bbut00021277"	exon_number "1"
17	lncRNA	exon	10730491	10730599	.	-	.	gene_id "lnc-bbug09250"	transcript_id "lnc-bbut00021277"	exon_number "2"
17	lncRNA	exon	10735058	10735146	.	-	.	gene_id "lnc-bbug09250"	transcript_id "lnc-bbut00021277"	exon_number "3"
17	lncRNA	exon	10735304	10735487	.	-	.	gene_id "lnc-bbug09250"	transcript_id "lnc-bbut00021277"	exon_number "4"
17	lncRNA	exon	10955759	10956713	.	-	.	gene_id "lnc-bbug09253"	transcript_id "lnc-bbut00021287"	exon_number "1"
17	lncRNA	exon	11950386	11950889	.	-	.	gene_id "lnc-bbug09261"	transcript_id "lnc-bbut00021308"	exon_number "1"
17	lncRNA	exon	11959412	11959833	.	-	.	gene_id "lnc-bbug09261"	transcript_id "lnc-bbut00021308"	exon_number "2"
17	lncRNA	exon	11960502	11960667	.	-	.	gene_id "lnc-bbug09261"	transcript_id "lnc-bbut00021308"	exon_number "3"
17	lncRNA	exon	12107262	12108336	.	-	.	gene_id "lnc-bbug09264"	transcript_id "lnc-bbut00021311"	exon_number "1"
17	lncRNA	exon	12109296	12109389	.	-	.	gene_id "lnc-bbug09264"	transcript_id "lnc-bbut00021311"	exon_number "2"
17	lncRNA	exon	12111067	12111212	.	-	.	gene_id "lnc-bbug09264"	transcript_id "lnc-bbut00021311"	exon_number "3"

17	lncRNA	exon	12114078	12114188	.	-	.	gene_id "lnc-bbug09264"	transcript_id "lnc-bbut00021311"	exon_number "4"
17	lncRNA	exon	12256347	12256983	.	-	.	gene_id "lnc-bbug09265"	transcript_id "lnc-bbut00021312"	exon_number "1"
17	lncRNA	exon	13284760	13290208	.	-	.	gene_id "lnc-bbug09269"	transcript_id "lnc-bbut00021318"	exon_number "1"
17	lncRNA	exon	13291343	13291526	.	-	.	gene_id "lnc-bbug09269"	transcript_id "lnc-bbut00021318"	exon_number "2"
17	lncRNA	exon	13346527	13346593	.	-	.	gene_id "lnc-bbug09269"	transcript_id "lnc-bbut00021318"	exon_number "3"
17	lncRNA	exon	13374671	13375049	.	-	.	gene_id "lnc-bbug09269"	transcript_id "lnc-bbut00021318"	exon_number "4"
17	lncRNA	exon	13284760	13290208	.	-	.	gene_id "lnc-bbug09269"	transcript_id "lnc-bbut00021319"	exon_number "1"
17	lncRNA	exon	13291343	13291526	.	-	.	gene_id "lnc-bbug09269"	transcript_id "lnc-bbut00021319"	exon_number "2"
17	lncRNA	exon	13346527	13346593	.	-	.	gene_id "lnc-bbug09269"	transcript_id "lnc-bbut00021319"	exon_number "3"
17	lncRNA	exon	13354947	13355034	.	-	.	gene_id "lnc-bbug09269"	transcript_id "lnc-bbut00021319"	exon_number "4"
17	lncRNA	exon	13374671	13375052	.	-	.	gene_id "lnc-bbug09269"	transcript_id "lnc-bbut00021319"	exon_number "5"
17	lncRNA	exon	14039020	14039135	.	-	.	gene_id "lnc-bbug09270"	transcript_id "lnc-bbut00021320"	exon_number "1"
17	lncRNA	exon	14039772	14040042	.	-	.	gene_id "lnc-bbug09270"	transcript_id "lnc-bbut00021320"	exon_number "2"
17	lncRNA	exon	14040235	14040283	.	-	.	gene_id "lnc-bbug09271"	transcript_id "lnc-bbut00021322"	exon_number "1"
17	lncRNA	exon	14042259	14042348	.	-	.	gene_id "lnc-bbug09271"	transcript_id "lnc-bbut00021322"	exon_number "2"
17	lncRNA	exon	14042903	14043109	.	-	.	gene_id "lnc-bbug09271"	transcript_id "lnc-bbut00021322"	exon_number "3"
17	lncRNA	exon	14045376	14045601	.	-	.	gene_id "lnc-bbug09271"	transcript_id "lnc-bbut00021322"	exon_number "4"
17	lncRNA	exon	14045693	14047085	.	-	.	gene_id "lnc-bbug09271"	transcript_id "lnc-bbut00021322"	exon_number "5"
17	lncRNA	exon	14048113	14048261	.	-	.	gene_id "lnc-bbug09271"	transcript_id "lnc-bbut00021322"	exon_number "6"
17	lncRNA	exon	14040235	14040283	.	-	.	gene_id "lnc-bbug09271"	transcript_id "lnc-bbut00021321"	exon_number "1"
17	lncRNA	exon	14042259	14042348	.	-	.	gene_id "lnc-bbug09271"	transcript_id "lnc-bbut00021321"	exon_number "2"
17	lncRNA	exon	14042903	14043109	.	-	.	gene_id "lnc-bbug09271"	transcript_id "lnc-bbut00021321"	exon_number "3"
17	lncRNA	exon	14046963	14047085	.	-	.	gene_id "lnc-bbug09271"	transcript_id "lnc-bbut00021321"	exon_number "4"
17	lncRNA	exon	14048113	14048261	.	-	.	gene_id "lnc-bbug09271"	transcript_id "lnc-bbut00021321"	exon_number "5"
17	lncRNA	exon	14836560	14841372	.	-	.	gene_id "lnc-bbug09274"	transcript_id "lnc-bbut00021333"	exon_number "1"
17	lncRNA	exon	14860854	14861347	.	-	.	gene_id "lnc-bbug09274"	transcript_id "lnc-bbut00021333"	exon_number "2"
17	lncRNA	exon	17792020	17792792	.	-	.	gene_id "lnc-bbug09285"	transcript_id "lnc-bbut00021363"	exon_number "1"
17	lncRNA	exon	17792861	17792877	.	-	.	gene_id "lnc-bbug09285"	transcript_id "lnc-bbut00021363"	exon_number "2"
17	lncRNA	exon	18230777	18230885	.	-	.	gene_id "lnc-bbug09288"	transcript_id "lnc-bbut00021367"	exon_number "1"
17	lncRNA	exon	18231438	18231707	.	-	.	gene_id "lnc-bbug09288"	transcript_id "lnc-bbut00021367"	exon_number "2"
17	lncRNA	exon	19654559	19654904	.	-	.	gene_id "lnc-bbug09301"	transcript_id "lnc-bbut00021417"	exon_number "1"
17	lncRNA	exon	19655000	19655078	.	-	.	gene_id "lnc-bbug09301"	transcript_id "lnc-bbut00021417"	exon_number "2"
17	lncRNA	exon	19655933	19655988	.	-	.	gene_id "lnc-bbug09301"	transcript_id "lnc-bbut00021417"	exon_number "3"
17	lncRNA	exon	19659635	19660071	.	-	.	gene_id "lnc-bbug09301"	transcript_id "lnc-bbut00021417"	exon_number "4"
17	lncRNA	exon	19691105	19692675	.	-	.	gene_id "lnc-bbug09302"	transcript_id "lnc-bbut00021418"	exon_number "1"
17	lncRNA	exon	19695053	19695119	.	-	.	gene_id "lnc-bbug09302"	transcript_id "lnc-bbut00021418"	exon_number "2"
17	lncRNA	exon	19695975	19697457	.	-	.	gene_id "lnc-bbug09302"	transcript_id "lnc-bbut00021418"	exon_number "3"
17	lncRNA	exon	19778332	19779031	.	-	.	gene_id "lnc-bbug09303"	transcript_id "lnc-bbut00021419"	exon_number "1"
17	lncRNA	exon	19781877	19782004	.	-	.	gene_id "lnc-bbug09303"	transcript_id "lnc-bbut00021419"	exon_number "2"
17	lncRNA	exon	19782574	19782638	.	-	.	gene_id "lnc-bbug09303"	transcript_id "lnc-bbut00021419"	exon_number "3"
17	lncRNA	exon	19857754	19858514	.	-	.	gene_id "lnc-bbug09304"	transcript_id "lnc-bbut00021420"	exon_number "1"
17	lncRNA	exon	19859156	19859249	.	-	.	gene_id "lnc-bbug09304"	transcript_id "lnc-bbut00021420"	exon_number "2"
17	lncRNA	exon	20118175	20118603	.	-	.	gene_id "lnc-bbug09310"	transcript_id "lnc-bbut00021448"	exon_number "1"
17	lncRNA	exon	20126008	20126559	.	-	.	gene_id "lnc-bbug09310"	transcript_id "lnc-bbut00021448"	exon_number "2"
17	lncRNA	exon	20158484	20158595	.	-	.	gene_id "lnc-bbug09311"	transcript_id "lnc-bbut00021449"	exon_number "1"
17	lncRNA	exon	20162598	20162722	.	-	.	gene_id "lnc-bbug09311"	transcript_id "lnc-bbut00021449"	exon_number "2"
17	lncRNA	exon	20180761	20180871	.	-	.	gene_id "lnc-bbug09311"	transcript_id "lnc-bbut00021449"	exon_number "3"
17	lncRNA	exon	20223402	20224409	.	-	.	gene_id "lnc-bbug09312"	transcript_id "lnc-bbut00021450"	exon_number "1"
17	lncRNA	exon	20718440	20719269	.	-	.	gene_id "lnc-bbug09319"	transcript_id "lnc-bbut00021490"	exon_number "1"
17	lncRNA	exon	20721977	20722103	.	-	.	gene_id "lnc-bbug09319"	transcript_id "lnc-bbut00021490"	exon_number "2"
17	lncRNA	exon	20723768	20723865	.	-	.	gene_id "lnc-bbug09319"	transcript_id "lnc-bbut00021490"	exon_number "3"
17	lncRNA	exon	20728659	20729009	.	-	.	gene_id "lnc-bbug09319"	transcript_id "lnc-bbut00021490"	exon_number "4"

17	lncRNA	exon	20807724	20807843	.	-	.	gene_id "lnc-bbug09321"	transcript_id "lnc-bbut00021492"	exon_number "1"
17	lncRNA	exon	20808267	20808656	.	-	.	gene_id "lnc-bbug09321"	transcript_id "lnc-bbut00021492"	exon_number "2"
17	lncRNA	exon	21913798	21914323	.	-	.	gene_id "lnc-bbug09328"	transcript_id "lnc-bbut00021523"	exon_number "1"
17	lncRNA	exon	21918467	21918834	.	-	.	gene_id "lnc-bbug09328"	transcript_id "lnc-bbut00021523"	exon_number "2"
17	lncRNA	exon	21913798	21914320	.	-	.	gene_id "lnc-bbug09328"	transcript_id "lnc-bbut00021522"	exon_number "1"
17	lncRNA	exon	21918467	21918834	.	-	.	gene_id "lnc-bbug09328"	transcript_id "lnc-bbut00021522"	exon_number "2"
17	lncRNA	exon	21941563	21941897	.	-	.	gene_id "lnc-bbug09329"	transcript_id "lnc-bbut00021525"	exon_number "1"
17	lncRNA	exon	21945316	21945496	.	-	.	gene_id "lnc-bbug09329"	transcript_id "lnc-bbut00021525"	exon_number "2"
17	lncRNA	exon	21946902	21947539	.	-	.	gene_id "lnc-bbug09329"	transcript_id "lnc-bbut00021525"	exon_number "3"
17	lncRNA	exon	22135870	22139127	.	-	.	gene_id "lnc-bbug09331"	transcript_id "lnc-bbut00021528"	exon_number "1"
17	lncRNA	exon	22140121	22143114	.	-	.	gene_id "lnc-bbug09331"	transcript_id "lnc-bbut00021528"	exon_number "2"
17	lncRNA	exon	22158863	22159427	.	-	.	gene_id "lnc-bbug09333"	transcript_id "lnc-bbut00021530"	exon_number "1"
17	lncRNA	exon	22160005	22160337	.	-	.	gene_id "lnc-bbug09333"	transcript_id "lnc-bbut00021530"	exon_number "2"
17	lncRNA	exon	23285067	23286887	.	-	.	gene_id "lnc-bbug09336"	transcript_id "lnc-bbut00021534"	exon_number "1"
17	lncRNA	exon	26198014	26198502	.	-	.	gene_id "lnc-bbug09341"	transcript_id "lnc-bbut00021539"	exon_number "1"
17	lncRNA	exon	26199239	26199329	.	-	.	gene_id "lnc-bbug09341"	transcript_id "lnc-bbut00021539"	exon_number "2"
17	lncRNA	exon	26230235	26230562	.	-	.	gene_id "lnc-bbug09341"	transcript_id "lnc-bbut00021539"	exon_number "3"
17	lncRNA	exon	27485334	27486949	.	-	.	gene_id "lnc-bbug09344"	transcript_id "lnc-bbut00021554"	exon_number "1"
17	lncRNA	exon	27573575	27573886	.	-	.	gene_id "lnc-bbug09345"	transcript_id "lnc-bbut00021555"	exon_number "1"
17	lncRNA	exon	27576774	27576869	.	-	.	gene_id "lnc-bbug09345"	transcript_id "lnc-bbut00021555"	exon_number "2"
17	lncRNA	exon	27580606	27580905	.	-	.	gene_id "lnc-bbug09345"	transcript_id "lnc-bbut00021555"	exon_number "3"
17	lncRNA	exon	28239596	28240376	.	-	.	gene_id "lnc-bbug09348"	transcript_id "lnc-bbut00021560"	exon_number "1"
17	lncRNA	exon	28241831	28241938	.	-	.	gene_id "lnc-bbug09348"	transcript_id "lnc-bbut00021560"	exon_number "2"
17	lncRNA	exon	28243001	28243089	.	-	.	gene_id "lnc-bbug09348"	transcript_id "lnc-bbut00021560"	exon_number "3"
17	lncRNA	exon	28266574	28266772	.	-	.	gene_id "lnc-bbug09348"	transcript_id "lnc-bbut00021560"	exon_number "4"
17	lncRNA	exon	28271150	28271278	.	-	.	gene_id "lnc-bbug09348"	transcript_id "lnc-bbut00021560"	exon_number "5"
17	lncRNA	exon	32639677	32640256	.	-	.	gene_id "lnc-bbug09365"	transcript_id "lnc-bbut00021612"	exon_number "1"
17	lncRNA	exon	32695761	32695849	.	-	.	gene_id "lnc-bbug09367"	transcript_id "lnc-bbut00021614"	exon_number "1"
17	lncRNA	exon	32698342	32698435	.	-	.	gene_id "lnc-bbug09367"	transcript_id "lnc-bbut00021614"	exon_number "2"
17	lncRNA	exon	32707904	32708016	.	-	.	gene_id "lnc-bbug09367"	transcript_id "lnc-bbut00021614"	exon_number "3"
17	lncRNA	exon	32711697	32712250	.	-	.	gene_id "lnc-bbug09367"	transcript_id "lnc-bbut00021614"	exon_number "4"
17	lncRNA	exon	33029941	33030261	.	-	.	gene_id "lnc-bbug09369"	transcript_id "lnc-bbut00021616"	exon_number "1"
17	lncRNA	exon	34007704	34007964	.	-	.	gene_id "lnc-bbug09370"	transcript_id "lnc-bbut00021617"	exon_number "1"
17	lncRNA	exon	34042677	34042798	.	-	.	gene_id "lnc-bbug09370"	transcript_id "lnc-bbut00021617"	exon_number "2"
17	lncRNA	exon	34045888	34046076	.	-	.	gene_id "lnc-bbug09370"	transcript_id "lnc-bbut00021617"	exon_number "3"
17	lncRNA	exon	34033461	34033518	.	-	.	gene_id "lnc-bbug09370"	transcript_id "lnc-bbut00021618"	exon_number "1"
17	lncRNA	exon	34033679	34033729	.	-	.	gene_id "lnc-bbug09370"	transcript_id "lnc-bbut00021618"	exon_number "2"
17	lncRNA	exon	34042677	34042798	.	-	.	gene_id "lnc-bbug09370"	transcript_id "lnc-bbut00021618"	exon_number "3"
17	lncRNA	exon	34045888	34046076	.	-	.	gene_id "lnc-bbug09370"	transcript_id "lnc-bbut00021618"	exon_number "4"
17	lncRNA	exon	34646380	34646984	.	-	.	gene_id "lnc-bbug09371"	transcript_id "lnc-bbut00021619"	exon_number "1"
17	lncRNA	exon	35819306	35819671	.	-	.	gene_id "lnc-bbug09372"	transcript_id "lnc-bbut00021620"	exon_number "1"
17	lncRNA	exon	35819710	35819930	.	-	.	gene_id "lnc-bbug09372"	transcript_id "lnc-bbut00021620"	exon_number "2"
17	lncRNA	exon	35820058	35820171	.	-	.	gene_id "lnc-bbug09372"	transcript_id "lnc-bbut00021620"	exon_number "3"
17	lncRNA	exon	37527449	37528615	.	-	.	gene_id "lnc-bbug09375"	transcript_id "lnc-bbut00021626"	exon_number "1"
17	lncRNA	exon	37530134	37530287	.	-	.	gene_id "lnc-bbug09375"	transcript_id "lnc-bbut00021626"	exon_number "2"
17	lncRNA	exon	38250234	38256282	.	-	.	gene_id "lnc-bbug09380"	transcript_id "lnc-bbut00021635"	exon_number "1"
17	lncRNA	exon	38256703	38257270	.	-	.	gene_id "lnc-bbug09380"	transcript_id "lnc-bbut00021635"	exon_number "2"
17	lncRNA	exon	40413772	40414535	.	-	.	gene_id "lnc-bbug09384"	transcript_id "lnc-bbut00021641"	exon_number "1"
17	lncRNA	exon	40418004	40418100	.	-	.	gene_id "lnc-bbug09384"	transcript_id "lnc-bbut00021641"	exon_number "2"
17	lncRNA	exon	40514184	40514695	.	-	.	gene_id "lnc-bbug09384"	transcript_id "lnc-bbut00021641"	exon_number "3"
17	lncRNA	exon	40413772	40414535	.	-	.	gene_id "lnc-bbug09384"	transcript_id "lnc-bbut00021642"	exon_number "1"
17	lncRNA	exon	40418004	40418100	.	-	.	gene_id "lnc-bbug09384"	transcript_id "lnc-bbut00021642"	exon_number "2"

17	lncRNA	exon	40460705	40460739	.	-	.	gene_id "lnc-bbug09384"	transcript_id "lnc-bbut00021642"	exon_number "3"
17	lncRNA	exon	40514184	40514697	.	-	.	gene_id "lnc-bbug09384"	transcript_id "lnc-bbut00021642"	exon_number "4"
17	lncRNA	exon	40413772	40414535	.	-	.	gene_id "lnc-bbug09384"	transcript_id "lnc-bbut00021643"	exon_number "1"
17	lncRNA	exon	40418004	40418100	.	-	.	gene_id "lnc-bbug09384"	transcript_id "lnc-bbut00021643"	exon_number "2"
17	lncRNA	exon	40460705	40460739	.	-	.	gene_id "lnc-bbug09384"	transcript_id "lnc-bbut00021643"	exon_number "3"
17	lncRNA	exon	40514184	40514326	.	-	.	gene_id "lnc-bbug09384"	transcript_id "lnc-bbut00021643"	exon_number "4"
17	lncRNA	exon	40514727	40515297	.	-	.	gene_id "lnc-bbug09384"	transcript_id "lnc-bbut00021643"	exon_number "5"
17	lncRNA	exon	43134355	43136599	.	-	.	gene_id "lnc-bbug09385"	transcript_id "lnc-bbut00021644"	exon_number "1"
17	lncRNA	exon	43480001	43480542	.	-	.	gene_id "lnc-bbug09387"	transcript_id "lnc-bbut00021647"	exon_number "1"
17	lncRNA	exon	43493462	43493580	.	-	.	gene_id "lnc-bbug09387"	transcript_id "lnc-bbut00021647"	exon_number "2"
17	lncRNA	exon	43509890	43509939	.	-	.	gene_id "lnc-bbug09387"	transcript_id "lnc-bbut00021647"	exon_number "3"
17	lncRNA	exon	43524549	43526188	.	-	.	gene_id "lnc-bbug09388"	transcript_id "lnc-bbut00021648"	exon_number "1"
17	lncRNA	exon	43538524	43539565	.	-	.	gene_id "lnc-bbug09389"	transcript_id "lnc-bbut00021649"	exon_number "1"
17	lncRNA	exon	43983405	43984045	.	-	.	gene_id "lnc-bbug09391"	transcript_id "lnc-bbut00021651"	exon_number "1"
17	lncRNA	exon	43984240	43984516	.	-	.	gene_id "lnc-bbug09391"	transcript_id "lnc-bbut00021651"	exon_number "2"
17	lncRNA	exon	43985166	43986148	.	-	.	gene_id "lnc-bbug09391"	transcript_id "lnc-bbut00021651"	exon_number "3"
17	lncRNA	exon	43997651	43999311	.	-	.	gene_id "lnc-bbug09392"	transcript_id "lnc-bbut00021652"	exon_number "1"
17	lncRNA	exon	44001786	44001974	.	-	.	gene_id "lnc-bbug09392"	transcript_id "lnc-bbut00021652"	exon_number "2"
17	lncRNA	exon	44074379	44074658	.	-	.	gene_id "lnc-bbug09393"	transcript_id "lnc-bbut00021653"	exon_number "1"
17	lncRNA	exon	44076459	44081661	.	-	.	gene_id "lnc-bbug09393"	transcript_id "lnc-bbut00021653"	exon_number "2"
17	lncRNA	exon	44087171	44090538	.	-	.	gene_id "lnc-bbug09393"	transcript_id "lnc-bbut00021653"	exon_number "3"
17	lncRNA	exon	44487519	44487616	.	-	.	gene_id "lnc-bbug09396"	transcript_id "lnc-bbut00021659"	exon_number "1"
17	lncRNA	exon	44488231	44488400	.	-	.	gene_id "lnc-bbug09396"	transcript_id "lnc-bbut00021659"	exon_number "2"
17	lncRNA	exon	44503255	44503483	.	-	.	gene_id "lnc-bbug09396"	transcript_id "lnc-bbut00021659"	exon_number "3"
17	lncRNA	exon	47926075	47926187	.	-	.	gene_id "lnc-bbug09398"	transcript_id "lnc-bbut00021661"	exon_number "1"
17	lncRNA	exon	47990141	47990333	.	-	.	gene_id "lnc-bbug09398"	transcript_id "lnc-bbut00021661"	exon_number "2"
17	lncRNA	exon	47990591	47992047	.	-	.	gene_id "lnc-bbug09398"	transcript_id "lnc-bbut00021661"	exon_number "3"
17	lncRNA	exon	47994271	47999038	.	-	.	gene_id "lnc-bbug09398"	transcript_id "lnc-bbut00021661"	exon_number "4"
17	lncRNA	exon	47999467	48000924	.	-	.	gene_id "lnc-bbug09398"	transcript_id "lnc-bbut00021661"	exon_number "5"
17	lncRNA	exon	47982746	47985593	.	-	.	gene_id "lnc-bbug09398"	transcript_id "lnc-bbut00021662"	exon_number "1"
17	lncRNA	exon	47990141	47990333	.	-	.	gene_id "lnc-bbug09398"	transcript_id "lnc-bbut00021662"	exon_number "2"
17	lncRNA	exon	47990591	47992047	.	-	.	gene_id "lnc-bbug09398"	transcript_id "lnc-bbut00021662"	exon_number "3"
17	lncRNA	exon	47994271	47999038	.	-	.	gene_id "lnc-bbug09398"	transcript_id "lnc-bbut00021662"	exon_number "4"
17	lncRNA	exon	47999467	48000924	.	-	.	gene_id "lnc-bbug09398"	transcript_id "lnc-bbut00021662"	exon_number "5"
17	lncRNA	exon	47986743	47986793	.	-	.	gene_id "lnc-bbug09398"	transcript_id "lnc-bbut00021663"	exon_number "1"
17	lncRNA	exon	47990141	47990333	.	-	.	gene_id "lnc-bbug09398"	transcript_id "lnc-bbut00021663"	exon_number "2"
17	lncRNA	exon	47990591	47992047	.	-	.	gene_id "lnc-bbug09398"	transcript_id "lnc-bbut00021663"	exon_number "3"
17	lncRNA	exon	47994271	47994357	.	-	.	gene_id "lnc-bbug09398"	transcript_id "lnc-bbut00021663"	exon_number "4"
17	lncRNA	exon	47998959	47999038	.	-	.	gene_id "lnc-bbug09398"	transcript_id "lnc-bbut00021663"	exon_number "5"
17	lncRNA	exon	47999467	48000902	.	-	.	gene_id "lnc-bbug09398"	transcript_id "lnc-bbut00021663"	exon_number "6"
17	lncRNA	exon	47986743	47986793	.	-	.	gene_id "lnc-bbug09398"	transcript_id "lnc-bbut00021665"	exon_number "1"
17	lncRNA	exon	47990141	47990333	.	-	.	gene_id "lnc-bbug09398"	transcript_id "lnc-bbut00021665"	exon_number "2"
17	lncRNA	exon	47990591	47992047	.	-	.	gene_id "lnc-bbug09398"	transcript_id "lnc-bbut00021665"	exon_number "3"
17	lncRNA	exon	47994271	47999038	.	-	.	gene_id "lnc-bbug09398"	transcript_id "lnc-bbut00021665"	exon_number "4"
17	lncRNA	exon	47999467	48000924	.	-	.	gene_id "lnc-bbug09398"	transcript_id "lnc-bbut00021665"	exon_number "5"
17	lncRNA	exon	47986743	47986793	.	-	.	gene_id "lnc-bbug09398"	transcript_id "lnc-bbut00021664"	exon_number "1"
17	lncRNA	exon	47990141	47990333	.	-	.	gene_id "lnc-bbug09398"	transcript_id "lnc-bbut00021664"	exon_number "2"
17	lncRNA	exon	47990591	47991844	.	-	.	gene_id "lnc-bbug09398"	transcript_id "lnc-bbut00021664"	exon_number "3"
17	lncRNA	exon	47994271	47999038	.	-	.	gene_id "lnc-bbug09398"	transcript_id "lnc-bbut00021664"	exon_number "4"
17	lncRNA	exon	47999467	48000924	.	-	.	gene_id "lnc-bbug09398"	transcript_id "lnc-bbut00021664"	exon_number "5"
17	lncRNA	exon	48946588	48947578	.	-	.	gene_id "lnc-bbug09399"	transcript_id "lnc-bbut00021666"	exon_number "1"
17	lncRNA	exon	48972615	48972668	.	-	.	gene_id "lnc-bbug09399"	transcript_id "lnc-bbut00021666"	exon_number "2"

17	lncRNA	exon	51870339	51870985	.	-	.	gene_id "lnc-bbug09401"	transcript_id "lnc-bbut00021668"	exon_number "1"
17	lncRNA	exon	52504401	52507919	.	-	.	gene_id "lnc-bbug09402"	transcript_id "lnc-bbut00021669"	exon_number "1"
17	lncRNA	exon	52512200	52512469	.	-	.	gene_id "lnc-bbug09402"	transcript_id "lnc-bbut00021669"	exon_number "2"
17	lncRNA	exon	52547513	52547775	.	-	.	gene_id "lnc-bbug09402"	transcript_id "lnc-bbut00021669"	exon_number "3"
17	lncRNA	exon	54196387	54196843	.	-	.	gene_id "lnc-bbug09406"	transcript_id "lnc-bbut00021676"	exon_number "1"
17	lncRNA	exon	54203202	54203215	.	-	.	gene_id "lnc-bbug09406"	transcript_id "lnc-bbut00021676"	exon_number "2"
17	lncRNA	exon	54246191	54246838	.	-	.	gene_id "lnc-bbug09407"	transcript_id "lnc-bbut00021677"	exon_number "1"
17	lncRNA	exon	54251154	54251234	.	-	.	gene_id "lnc-bbug09407"	transcript_id "lnc-bbut00021677"	exon_number "2"
17	lncRNA	exon	54267538	54271334	.	-	.	gene_id "lnc-bbug09407"	transcript_id "lnc-bbut00021677"	exon_number "3"
17	lncRNA	exon	54551102	54552195	.	-	.	gene_id "lnc-bbug09409"	transcript_id "lnc-bbut00021689"	exon_number "1"
17	lncRNA	exon	54552481	54553704	.	-	.	gene_id "lnc-bbug09409"	transcript_id "lnc-bbut00021689"	exon_number "2"
17	lncRNA	exon	54663146	54666195	.	-	.	gene_id "lnc-bbug09411"	transcript_id "lnc-bbut00021691"	exon_number "1"
17	lncRNA	exon	54667104	54667183	.	-	.	gene_id "lnc-bbug09411"	transcript_id "lnc-bbut00021691"	exon_number "2"
17	lncRNA	exon	54669253	54669356	.	-	.	gene_id "lnc-bbug09411"	transcript_id "lnc-bbut00021691"	exon_number "3"
17	lncRNA	exon	54669890	54670035	.	-	.	gene_id "lnc-bbug09411"	transcript_id "lnc-bbut00021691"	exon_number "4"
17	lncRNA	exon	55007177	55008044	.	-	.	gene_id "lnc-bbug09413"	transcript_id "lnc-bbut00021693"	exon_number "1"
17	lncRNA	exon	55008127	55008130	.	-	.	gene_id "lnc-bbug09413"	transcript_id "lnc-bbut00021693"	exon_number "2"
17	lncRNA	exon	55551715	55552071	.	-	.	gene_id "lnc-bbug09415"	transcript_id "lnc-bbut00021696"	exon_number "1"
17	lncRNA	exon	55580056	55580182	.	-	.	gene_id "lnc-bbug09415"	transcript_id "lnc-bbut00021696"	exon_number "2"
17	lncRNA	exon	55594822	55595040	.	-	.	gene_id "lnc-bbug09416"	transcript_id "lnc-bbut00021697"	exon_number "1"
17	lncRNA	exon	55595133	55597076	.	-	.	gene_id "lnc-bbug09416"	transcript_id "lnc-bbut00021697"	exon_number "2"
17	lncRNA	exon	55713358	55713934	.	-	.	gene_id "lnc-bbug09417"	transcript_id "lnc-bbut00021698"	exon_number "1"
17	lncRNA	exon	55717348	55717388	.	-	.	gene_id "lnc-bbug09417"	transcript_id "lnc-bbut00021698"	exon_number "2"
17	lncRNA	exon	55725546	55725693	.	-	.	gene_id "lnc-bbug09417"	transcript_id "lnc-bbut00021698"	exon_number "3"
17	lncRNA	exon	55921858	55921926	.	-	.	gene_id "lnc-bbug09421"	transcript_id "lnc-bbut00021708"	exon_number "1"
17	lncRNA	exon	55922214	55923984	.	-	.	gene_id "lnc-bbug09421"	transcript_id "lnc-bbut00021708"	exon_number "2"
17	lncRNA	exon	55959775	55960426	.	-	.	gene_id "lnc-bbug09423"	transcript_id "lnc-bbut00021713"	exon_number "1"
17	lncRNA	exon	55960556	55960853	.	-	.	gene_id "lnc-bbug09423"	transcript_id "lnc-bbut00021713"	exon_number "2"
17	lncRNA	exon	56032501	56038657	.	-	.	gene_id "lnc-bbug09424"	transcript_id "lnc-bbut00021714"	exon_number "1"
17	lncRNA	exon	56039469	56039518	.	-	.	gene_id "lnc-bbug09424"	transcript_id "lnc-bbut00021714"	exon_number "2"
17	lncRNA	exon	56482045	56483970	.	-	.	gene_id "lnc-bbug09427"	transcript_id "lnc-bbut00021719"	exon_number "1"
17	lncRNA	exon	56485308	56485398	.	-	.	gene_id "lnc-bbug09427"	transcript_id "lnc-bbut00021719"	exon_number "2"
17	lncRNA	exon	58629823	58630283	.	-	.	gene_id "lnc-bbug09430"	transcript_id "lnc-bbut00021732"	exon_number "1"
17	lncRNA	exon	58674345	58674539	.	-	.	gene_id "lnc-bbug09430"	transcript_id "lnc-bbut00021732"	exon_number "2"
17	lncRNA	exon	60043811	60044299	.	-	.	gene_id "lnc-bbug09435"	transcript_id "lnc-bbut00021744"	exon_number "1"
17	lncRNA	exon	60474599	60474842	.	-	.	gene_id "lnc-bbug09436"	transcript_id "lnc-bbut00021745"	exon_number "1"
17	lncRNA	exon	60475219	60475259	.	-	.	gene_id "lnc-bbug09436"	transcript_id "lnc-bbut00021745"	exon_number "2"
17	lncRNA	exon	60476526	60476918	.	-	.	gene_id "lnc-bbug09436"	transcript_id "lnc-bbut00021745"	exon_number "3"
17	lncRNA	exon	61047587	61047670	.	-	.	gene_id "lnc-bbug09438"	transcript_id "lnc-bbut00021758"	exon_number "1"
17	lncRNA	exon	61072307	61072342	.	-	.	gene_id "lnc-bbug09438"	transcript_id "lnc-bbut00021758"	exon_number "2"
17	lncRNA	exon	61193045	61193083	.	-	.	gene_id "lnc-bbug09438"	transcript_id "lnc-bbut00021758"	exon_number "3"
17	lncRNA	exon	61266929	61267004	.	-	.	gene_id "lnc-bbug09438"	transcript_id "lnc-bbut00021758"	exon_number "4"
17	lncRNA	exon	61286382	61286464	.	-	.	gene_id "lnc-bbug09438"	transcript_id "lnc-bbut00021758"	exon_number "5"
17	lncRNA	exon	61293997	61294656	.	-	.	gene_id "lnc-bbug09438"	transcript_id "lnc-bbut00021758"	exon_number "6"
17	lncRNA	exon	61047587	61047670	.	-	.	gene_id "lnc-bbug09438"	transcript_id "lnc-bbut00021757"	exon_number "1"
17	lncRNA	exon	61193045	61193083	.	-	.	gene_id "lnc-bbug09438"	transcript_id "lnc-bbut00021757"	exon_number "2"
17	lncRNA	exon	61266929	61267004	.	-	.	gene_id "lnc-bbug09438"	transcript_id "lnc-bbut00021757"	exon_number "3"
17	lncRNA	exon	61286382	61286464	.	-	.	gene_id "lnc-bbug09438"	transcript_id "lnc-bbut00021757"	exon_number "4"
17	lncRNA	exon	61293997	61294656	.	-	.	gene_id "lnc-bbug09438"	transcript_id "lnc-bbut00021757"	exon_number "5"
17	lncRNA	exon	61136998	61139076	.	-	.	gene_id "lnc-bbug09438"	transcript_id "lnc-bbut00021760"	exon_number "1"
17	lncRNA	exon	61193045	61193083	.	-	.	gene_id "lnc-bbug09438"	transcript_id "lnc-bbut00021760"	exon_number "2"
17	lncRNA	exon	61266929	61267004	.	-	.	gene_id "lnc-bbug09438"	transcript_id "lnc-bbut00021760"	exon_number "3"

17	lncRNA	exon	61286382	61286464	.	-	.	gene_id "lnc-bbug09438"	transcript_id "lnc-bbut00021760"	exon_number "4"
17	lncRNA	exon	61293997	61294656	.	-	.	gene_id "lnc-bbug09438"	transcript_id "lnc-bbut00021760"	exon_number "5"
17	lncRNA	exon	61166025	61166622	.	-	.	gene_id "lnc-bbug09438"	transcript_id "lnc-bbut00021762"	exon_number "1"
17	lncRNA	exon	61193045	61193083	.	-	.	gene_id "lnc-bbug09438"	transcript_id "lnc-bbut00021762"	exon_number "2"
17	lncRNA	exon	61266929	61267004	.	-	.	gene_id "lnc-bbug09438"	transcript_id "lnc-bbut00021762"	exon_number "3"
17	lncRNA	exon	61286382	61286464	.	-	.	gene_id "lnc-bbug09438"	transcript_id "lnc-bbut00021762"	exon_number "4"
17	lncRNA	exon	61293997	61294656	.	-	.	gene_id "lnc-bbug09438"	transcript_id "lnc-bbut00021762"	exon_number "5"
17	lncRNA	exon	61854536	61854702	.	-	.	gene_id "lnc-bbug09440"	transcript_id "lnc-bbut00021768"	exon_number "1"
17	lncRNA	exon	61871632	61871781	.	-	.	gene_id "lnc-bbug09440"	transcript_id "lnc-bbut00021768"	exon_number "2"
17	lncRNA	exon	61924942	61925162	.	-	.	gene_id "lnc-bbug09440"	transcript_id "lnc-bbut00021768"	exon_number "3"
17	lncRNA	exon	67290457	67290957	.	-	.	gene_id "lnc-bbug09451"	transcript_id "lnc-bbut00021799"	exon_number "1"
17	lncRNA	exon	67291834	67292242	.	-	.	gene_id "lnc-bbug09451"	transcript_id "lnc-bbut00021799"	exon_number "2"
17	lncRNA	exon	67298758	67298893	.	-	.	gene_id "lnc-bbug09451"	transcript_id "lnc-bbut00021799"	exon_number "3"
17	lncRNA	exon	67319310	67319461	.	-	.	gene_id "lnc-bbug09451"	transcript_id "lnc-bbut00021799"	exon_number "4"
17	lncRNA	exon	67320660	67321793	.	-	.	gene_id "lnc-bbug09451"	transcript_id "lnc-bbut00021799"	exon_number "5"
17	lncRNA	exon	67342618	67342717	.	-	.	gene_id "lnc-bbug09451"	transcript_id "lnc-bbut00021799"	exon_number "6"
17	lncRNA	exon	67352830	67354060	.	-	.	gene_id "lnc-bbug09451"	transcript_id "lnc-bbut00021799"	exon_number "7"
17	lncRNA	exon	67904113	67905398	.	-	.	gene_id "lnc-bbug09453"	transcript_id "lnc-bbut00021809"	exon_number "1"
17	lncRNA	exon	67907282	67907373	.	-	.	gene_id "lnc-bbug09453"	transcript_id "lnc-bbut00021809"	exon_number "2"
17	lncRNA	exon	67908417	67908483	.	-	.	gene_id "lnc-bbug09453"	transcript_id "lnc-bbut00021809"	exon_number "3"
17	lncRNA	exon	67911989	67912298	.	-	.	gene_id "lnc-bbug09453"	transcript_id "lnc-bbut00021809"	exon_number "4"
17	lncRNA	exon	67971473	67980142	.	-	.	gene_id "lnc-bbug09455"	transcript_id "lnc-bbut00021811"	exon_number "1"
17	lncRNA	exon	67981825	67983016	.	-	.	gene_id "lnc-bbug09455"	transcript_id "lnc-bbut00021811"	exon_number "2"
17	lncRNA	exon	68242950	68243251	.	-	.	gene_id "lnc-bbug09458"	transcript_id "lnc-bbut00021820"	exon_number "1"
17	lncRNA	exon	68244838	68244981	.	-	.	gene_id "lnc-bbug09458"	transcript_id "lnc-bbut00021820"	exon_number "2"
17	lncRNA	exon	68246619	68246798	.	-	.	gene_id "lnc-bbug09458"	transcript_id "lnc-bbut00021820"	exon_number "3"
17	lncRNA	exon	68252039	68252189	.	-	.	gene_id "lnc-bbug09459"	transcript_id "lnc-bbut00021821"	exon_number "1"
17	lncRNA	exon	68256769	68256909	.	-	.	gene_id "lnc-bbug09459"	transcript_id "lnc-bbut00021821"	exon_number "2"
17	lncRNA	exon	68267049	68267133	.	-	.	gene_id "lnc-bbug09459"	transcript_id "lnc-bbut00021821"	exon_number "3"
17	lncRNA	exon	68268614	68268711	.	-	.	gene_id "lnc-bbug09459"	transcript_id "lnc-bbut00021821"	exon_number "4"
17	lncRNA	exon	68271311	68271421	.	-	.	gene_id "lnc-bbug09459"	transcript_id "lnc-bbut00021821"	exon_number "5"
17	lncRNA	exon	68256478	68256909	.	-	.	gene_id "lnc-bbug09459"	transcript_id "lnc-bbut00021822"	exon_number "1"
17	lncRNA	exon	68267049	68267133	.	-	.	gene_id "lnc-bbug09459"	transcript_id "lnc-bbut00021822"	exon_number "2"
17	lncRNA	exon	68268614	68268711	.	-	.	gene_id "lnc-bbug09459"	transcript_id "lnc-bbut00021822"	exon_number "3"
17	lncRNA	exon	68271311	68271421	.	-	.	gene_id "lnc-bbug09459"	transcript_id "lnc-bbut00021822"	exon_number "4"
17	lncRNA	exon	68621975	68622743	.	-	.	gene_id "lnc-bbug09460"	transcript_id "lnc-bbut00021823"	exon_number "1"
17	lncRNA	exon	68625516	68627255	.	-	.	gene_id "lnc-bbug09460"	transcript_id "lnc-bbut00021823"	exon_number "2"
17	lncRNA	exon	70059890	70059986	.	-	.	gene_id "lnc-bbug09466"	transcript_id "lnc-bbut00021831"	exon_number "1"
17	lncRNA	exon	70079589	70079711	.	-	.	gene_id "lnc-bbug09466"	transcript_id "lnc-bbut00021831"	exon_number "2"
17	lncRNA	exon	70089728	70089864	.	-	.	gene_id "lnc-bbug09466"	transcript_id "lnc-bbut00021831"	exon_number "3"
17	lncRNA	exon	72510136	72511886	.	-	.	gene_id "lnc-bbug09478"	transcript_id "lnc-bbut00021853"	exon_number "1"
17	lncRNA	exon	72965623	72966444	.	-	.	gene_id "lnc-bbug09479"	transcript_id "lnc-bbut00021854"	exon_number "1"
17	lncRNA	exon	72966793	72966878	.	-	.	gene_id "lnc-bbug09479"	transcript_id "lnc-bbut00021854"	exon_number "2"
17	lncRNA	exon	73006844	73006953	.	-	.	gene_id "lnc-bbug09480"	transcript_id "lnc-bbut00021855"	exon_number "1"
17	lncRNA	exon	73007552	73007608	.	-	.	gene_id "lnc-bbug09480"	transcript_id "lnc-bbut00021855"	exon_number "2"
17	lncRNA	exon	73008334	73008528	.	-	.	gene_id "lnc-bbug09480"	transcript_id "lnc-bbut00021855"	exon_number "3"
17	lncRNA	exon	73090673	73091241	.	-	.	gene_id "lnc-bbug09481"	transcript_id "lnc-bbut00021856"	exon_number "1"
18	lncRNA	exon	1624982	1626965	.	+	.	gene_id "lnc-bbug09492"	transcript_id "lnc-bbut00021874"	exon_number "1"
18	lncRNA	exon	1628531	1629917	.	+	.	gene_id "lnc-bbug09492"	transcript_id "lnc-bbut00021874"	exon_number "2"
18	lncRNA	exon	1757413	1757747	.	+	.	gene_id "lnc-bbug09493"	transcript_id "lnc-bbut00021875"	exon_number "1"
18	lncRNA	exon	1762556	1762688	.	+	.	gene_id "lnc-bbug09493"	transcript_id "lnc-bbut00021875"	exon_number "2"
18	lncRNA	exon	1776371	1776517	.	+	.	gene_id "lnc-bbug09493"	transcript_id "lnc-bbut00021875"	exon_number "3"

18	lncRNA	exon	1781325	1781481	.	+	.	gene_id "lnc-bbug09493"	transcript_id "lnc-bbut00021875"	exon_number "4"
18	lncRNA	exon	1864437	1864888	.	+	.	gene_id "lnc-bbug09494"	transcript_id "lnc-bbut00021876"	exon_number "1"
18	lncRNA	exon	1864977	1865050	.	+	.	gene_id "lnc-bbug09494"	transcript_id "lnc-bbut00021876"	exon_number "2"
18	lncRNA	exon	1872316	1872482	.	+	.	gene_id "lnc-bbug09494"	transcript_id "lnc-bbut00021876"	exon_number "3"
18	lncRNA	exon	1949571	1950125	.	+	.	gene_id "lnc-bbug09495"	transcript_id "lnc-bbut00021877"	exon_number "1"
18	lncRNA	exon	2451235	2451389	.	+	.	gene_id "lnc-bbug09502"	transcript_id "lnc-bbut00021885"	exon_number "1"
18	lncRNA	exon	2451462	2451588	.	+	.	gene_id "lnc-bbug09502"	transcript_id "lnc-bbut00021885"	exon_number "2"
18	lncRNA	exon	2565975	2566135	.	+	.	gene_id "lnc-bbug09503"	transcript_id "lnc-bbut00021886"	exon_number "1"
18	lncRNA	exon	2566462	2566517	.	+	.	gene_id "lnc-bbug09503"	transcript_id "lnc-bbut00021886"	exon_number "2"
18	lncRNA	exon	2567654	2567826	.	+	.	gene_id "lnc-bbug09503"	transcript_id "lnc-bbut00021886"	exon_number "3"
18	lncRNA	exon	2571203	2573507	.	+	.	gene_id "lnc-bbug09503"	transcript_id "lnc-bbut00021886"	exon_number "4"
18	lncRNA	exon	2565975	2566135	.	+	.	gene_id "lnc-bbug09503"	transcript_id "lnc-bbut00021887"	exon_number "1"
18	lncRNA	exon	2566462	2566517	.	+	.	gene_id "lnc-bbug09503"	transcript_id "lnc-bbut00021887"	exon_number "2"
18	lncRNA	exon	2567654	2567826	.	+	.	gene_id "lnc-bbug09503"	transcript_id "lnc-bbut00021887"	exon_number "3"
18	lncRNA	exon	2575490	2576118	.	+	.	gene_id "lnc-bbug09503"	transcript_id "lnc-bbut00021887"	exon_number "4"
18	lncRNA	exon	2565975	2566135	.	+	.	gene_id "lnc-bbug09503"	transcript_id "lnc-bbut00021888"	exon_number "1"
18	lncRNA	exon	2566462	2566517	.	+	.	gene_id "lnc-bbug09503"	transcript_id "lnc-bbut00021888"	exon_number "2"
18	lncRNA	exon	2567654	2567826	.	+	.	gene_id "lnc-bbug09503"	transcript_id "lnc-bbut00021888"	exon_number "3"
18	lncRNA	exon	2579049	2579103	.	+	.	gene_id "lnc-bbug09503"	transcript_id "lnc-bbut00021888"	exon_number "4"
18	lncRNA	exon	2579291	2579481	.	+	.	gene_id "lnc-bbug09503"	transcript_id "lnc-bbut00021888"	exon_number "5"
18	lncRNA	exon	2616373	2616555	.	+	.	gene_id "lnc-bbug09504"	transcript_id "lnc-bbut00021890"	exon_number "1"
18	lncRNA	exon	2617350	2617384	.	+	.	gene_id "lnc-bbug09504"	transcript_id "lnc-bbut00021890"	exon_number "2"
18	lncRNA	exon	2617958	2618610	.	+	.	gene_id "lnc-bbug09504"	transcript_id "lnc-bbut00021890"	exon_number "3"
18	lncRNA	exon	2758875	2759587	.	+	.	gene_id "lnc-bbug09506"	transcript_id "lnc-bbut00021892"	exon_number "1"
18	lncRNA	exon	2760302	2762022	.	+	.	gene_id "lnc-bbug09506"	transcript_id "lnc-bbut00021892"	exon_number "2"
18	lncRNA	exon	5432948	5434351	.	+	.	gene_id "lnc-bbug09514"	transcript_id "lnc-bbut00021909"	exon_number "1"
18	lncRNA	exon	5434702	5440868	.	+	.	gene_id "lnc-bbug09514"	transcript_id "lnc-bbut00021909"	exon_number "2"
18	lncRNA	exon	5716180	5719095	.	+	.	gene_id "lnc-bbug09515"	transcript_id "lnc-bbut00021910"	exon_number "1"
18	lncRNA	exon	5720154	5721610	.	+	.	gene_id "lnc-bbug09515"	transcript_id "lnc-bbut00021910"	exon_number "2"
18	lncRNA	exon	6205394	6205727	.	+	.	gene_id "lnc-bbug09516"	transcript_id "lnc-bbut00021911"	exon_number "1"
18	lncRNA	exon	6206484	6207181	.	+	.	gene_id "lnc-bbug09516"	transcript_id "lnc-bbut00021911"	exon_number "2"
18	lncRNA	exon	6214876	6215063	.	+	.	gene_id "lnc-bbug09517"	transcript_id "lnc-bbut00021912"	exon_number "1"
18	lncRNA	exon	6217975	6218826	.	+	.	gene_id "lnc-bbug09517"	transcript_id "lnc-bbut00021912"	exon_number "2"
18	lncRNA	exon	6991729	6991844	.	+	.	gene_id "lnc-bbug09518"	transcript_id "lnc-bbut00021913"	exon_number "1"
18	lncRNA	exon	6994292	6994367	.	+	.	gene_id "lnc-bbug09518"	transcript_id "lnc-bbut00021913"	exon_number "2"
18	lncRNA	exon	7000471	7000638	.	+	.	gene_id "lnc-bbug09518"	transcript_id "lnc-bbut00021913"	exon_number "3"
18	lncRNA	exon	7001665	7001918	.	+	.	gene_id "lnc-bbug09518"	transcript_id "lnc-bbut00021913"	exon_number "4"
18	lncRNA	exon	6991792	6991810	.	+	.	gene_id "lnc-bbug09518"	transcript_id "lnc-bbut00021914"	exon_number "1"
18	lncRNA	exon	6994292	6994367	.	+	.	gene_id "lnc-bbug09518"	transcript_id "lnc-bbut00021914"	exon_number "2"
18	lncRNA	exon	7000471	7000638	.	+	.	gene_id "lnc-bbug09518"	transcript_id "lnc-bbut00021914"	exon_number "3"
18	lncRNA	exon	7001665	7001918	.	+	.	gene_id "lnc-bbug09518"	transcript_id "lnc-bbut00021914"	exon_number "4"
18	lncRNA	exon	7191421	7192390	.	+	.	gene_id "lnc-bbug09519"	transcript_id "lnc-bbut00021915"	exon_number "1"
18	lncRNA	exon	7689226	7689353	.	+	.	gene_id "lnc-bbug09525"	transcript_id "lnc-bbut00021925"	exon_number "1"
18	lncRNA	exon	7702098	7702217	.	+	.	gene_id "lnc-bbug09525"	transcript_id "lnc-bbut00021925"	exon_number "2"
18	lncRNA	exon	7702645	7703428	.	+	.	gene_id "lnc-bbug09525"	transcript_id "lnc-bbut00021925"	exon_number "3"
18	lncRNA	exon	8388456	8388768	.	+	.	gene_id "lnc-bbug09531"	transcript_id "lnc-bbut00021934"	exon_number "1"
18	lncRNA	exon	8405758	8405861	.	+	.	gene_id "lnc-bbug09531"	transcript_id "lnc-bbut00021934"	exon_number "2"
18	lncRNA	exon	8409297	8410298	.	+	.	gene_id "lnc-bbug09531"	transcript_id "lnc-bbut00021934"	exon_number "3"
18	lncRNA	exon	8904961	8910933	.	+	.	gene_id "lnc-bbug09533"	transcript_id "lnc-bbut00021937"	exon_number "1"
18	lncRNA	exon	9801917	9802072	.	+	.	gene_id "lnc-bbug09534"	transcript_id "lnc-bbut00021938"	exon_number "1"
18	lncRNA	exon	9802609	9802675	.	+	.	gene_id "lnc-bbug09534"	transcript_id "lnc-bbut00021938"	exon_number "2"
18	lncRNA	exon	9803231	9803351	.	+	.	gene_id "lnc-bbug09534"	transcript_id "lnc-bbut00021938"	exon_number "3"

18	lncRNA	exon	9804849	9806364	.	+	.	gene_id "lnc-bbug09534"	transcript_id "lnc-bbut00021938"	exon_number "4"
18	lncRNA	exon	11402301	11402594	.	+	.	gene_id "lnc-bbug09547"	transcript_id "lnc-bbut00021968"	exon_number "1"
18	lncRNA	exon	11404595	11405107	.	+	.	gene_id "lnc-bbug09547"	transcript_id "lnc-bbut00021968"	exon_number "2"
18	lncRNA	exon	11405845	11406029	.	+	.	gene_id "lnc-bbug09547"	transcript_id "lnc-bbut00021968"	exon_number "3"
18	lncRNA	exon	11406295	11407130	.	+	.	gene_id "lnc-bbug09547"	transcript_id "lnc-bbut00021968"	exon_number "4"
18	lncRNA	exon	11404320	11405107	.	+	.	gene_id "lnc-bbug09547"	transcript_id "lnc-bbut00021969"	exon_number "1"
18	lncRNA	exon	11405845	11406029	.	+	.	gene_id "lnc-bbug09547"	transcript_id "lnc-bbut00021969"	exon_number "2"
18	lncRNA	exon	11406295	11407130	.	+	.	gene_id "lnc-bbug09547"	transcript_id "lnc-bbut00021969"	exon_number "3"
18	lncRNA	exon	11703137	11704715	.	+	.	gene_id "lnc-bbug09549"	transcript_id "lnc-bbut00021973"	exon_number "1"
18	lncRNA	exon	11705779	11708121	.	+	.	gene_id "lnc-bbug09549"	transcript_id "lnc-bbut00021973"	exon_number "2"
18	lncRNA	exon	11774130	11774236	.	+	.	gene_id "lnc-bbug09550"	transcript_id "lnc-bbut00021974"	exon_number "1"
18	lncRNA	exon	11779084	11779970	.	+	.	gene_id "lnc-bbug09550"	transcript_id "lnc-bbut00021974"	exon_number "2"
18	lncRNA	exon	12358470	12359822	.	+	.	gene_id "lnc-bbug09555"	transcript_id "lnc-bbut00021979"	exon_number "1"
18	lncRNA	exon	12360457	12360705	.	+	.	gene_id "lnc-bbug09555"	transcript_id "lnc-bbut00021979"	exon_number "2"
18	lncRNA	exon	12361585	12361698	.	+	.	gene_id "lnc-bbug09555"	transcript_id "lnc-bbut00021979"	exon_number "3"
18	lncRNA	exon	12609601	12609760	.	+	.	gene_id "lnc-bbug09557"	transcript_id "lnc-bbut00021981"	exon_number "1"
18	lncRNA	exon	12617753	12617808	.	+	.	gene_id "lnc-bbug09557"	transcript_id "lnc-bbut00021981"	exon_number "2"
18	lncRNA	exon	12620411	12620517	.	+	.	gene_id "lnc-bbug09557"	transcript_id "lnc-bbut00021981"	exon_number "3"
18	lncRNA	exon	12621354	12623147	.	+	.	gene_id "lnc-bbug09557"	transcript_id "lnc-bbut00021981"	exon_number "4"
18	lncRNA	exon	12674174	12674926	.	+	.	gene_id "lnc-bbug09558"	transcript_id "lnc-bbut00021982"	exon_number "1"
18	lncRNA	exon	12677805	12677904	.	+	.	gene_id "lnc-bbug09558"	transcript_id "lnc-bbut00021982"	exon_number "2"
18	lncRNA	exon	12682890	12683062	.	+	.	gene_id "lnc-bbug09558"	transcript_id "lnc-bbut00021982"	exon_number "3"
18	lncRNA	exon	12684400	12687263	.	+	.	gene_id "lnc-bbug09558"	transcript_id "lnc-bbut00021982"	exon_number "4"
18	lncRNA	exon	12675143	12675466	.	+	.	gene_id "lnc-bbug09558"	transcript_id "lnc-bbut00021983"	exon_number "1"
18	lncRNA	exon	12677805	12677904	.	+	.	gene_id "lnc-bbug09558"	transcript_id "lnc-bbut00021983"	exon_number "2"
18	lncRNA	exon	12682890	12683062	.	+	.	gene_id "lnc-bbug09558"	transcript_id "lnc-bbut00021983"	exon_number "3"
18	lncRNA	exon	12684400	12687263	.	+	.	gene_id "lnc-bbug09558"	transcript_id "lnc-bbut00021983"	exon_number "4"
18	lncRNA	exon	12877099	12877657	.	+	.	gene_id "lnc-bbug09560"	transcript_id "lnc-bbut00021985"	exon_number "1"
18	lncRNA	exon	12977638	12979971	.	+	.	gene_id "lnc-bbug09561"	transcript_id "lnc-bbut00021986"	exon_number "1"
18	lncRNA	exon	12980209	12980350	.	+	.	gene_id "lnc-bbug09561"	transcript_id "lnc-bbut00021986"	exon_number "2"
18	lncRNA	exon	12980719	12981708	.	+	.	gene_id "lnc-bbug09561"	transcript_id "lnc-bbut00021986"	exon_number "3"
18	lncRNA	exon	12979866	12979971	.	+	.	gene_id "lnc-bbug09561"	transcript_id "lnc-bbut00021987"	exon_number "1"
18	lncRNA	exon	12980209	12980350	.	+	.	gene_id "lnc-bbug09561"	transcript_id "lnc-bbut00021987"	exon_number "2"
18	lncRNA	exon	12982032	12982141	.	+	.	gene_id "lnc-bbug09561"	transcript_id "lnc-bbut00021987"	exon_number "3"
18	lncRNA	exon	12983319	12983395	.	+	.	gene_id "lnc-bbug09561"	transcript_id "lnc-bbut00021987"	exon_number "4"
18	lncRNA	exon	13430400	13431035	.	+	.	gene_id "lnc-bbug09565"	transcript_id "lnc-bbut00021993"	exon_number "1"
18	lncRNA	exon	13432561	13433003	.	+	.	gene_id "lnc-bbug09565"	transcript_id "lnc-bbut00021993"	exon_number "2"
18	lncRNA	exon	13551455	13551692	.	+	.	gene_id "lnc-bbug09568"	transcript_id "lnc-bbut00022000"	exon_number "1"
18	lncRNA	exon	13553063	13553235	.	+	.	gene_id "lnc-bbug09568"	transcript_id "lnc-bbut00022000"	exon_number "2"
18	lncRNA	exon	13556000	13556107	.	+	.	gene_id "lnc-bbug09568"	transcript_id "lnc-bbut00022000"	exon_number "3"
18	lncRNA	exon	13708277	13708383	.	+	.	gene_id "lnc-bbug09572"	transcript_id "lnc-bbut00022006"	exon_number "1"
18	lncRNA	exon	13709844	13709945	.	+	.	gene_id "lnc-bbug09572"	transcript_id "lnc-bbut00022006"	exon_number "2"
18	lncRNA	exon	13710242	13710440	.	+	.	gene_id "lnc-bbug09572"	transcript_id "lnc-bbut00022006"	exon_number "3"
18	lncRNA	exon	13938380	13938625	.	+	.	gene_id "lnc-bbug09575"	transcript_id "lnc-bbut00022010"	exon_number "1"
18	lncRNA	exon	13938854	13939227	.	+	.	gene_id "lnc-bbug09575"	transcript_id "lnc-bbut00022010"	exon_number "2"
18	lncRNA	exon	13939738	13939794	.	+	.	gene_id "lnc-bbug09575"	transcript_id "lnc-bbut00022010"	exon_number "3"
18	lncRNA	exon	13940967	13941603	.	+	.	gene_id "lnc-bbug09575"	transcript_id "lnc-bbut00022010"	exon_number "4"
18	lncRNA	exon	14149570	14149670	.	+	.	gene_id "lnc-bbug09580"	transcript_id "lnc-bbut00022018"	exon_number "1"
18	lncRNA	exon	14149921	14150088	.	+	.	gene_id "lnc-bbug09580"	transcript_id "lnc-bbut00022018"	exon_number "2"
18	lncRNA	exon	14157813	14158978	.	+	.	gene_id "lnc-bbug09580"	transcript_id "lnc-bbut00022018"	exon_number "3"
18	lncRNA	exon	14274814	14276676	.	+	.	gene_id "lnc-bbug09584"	transcript_id "lnc-bbut00022040"	exon_number "1"
18	lncRNA	exon	14276776	14278005	.	+	.	gene_id "lnc-bbug09584"	transcript_id "lnc-bbut00022040"	exon_number "2"

18	lncRNA	exon	14801906	14802680	.	+	.	gene_id "lnc-bbug09590"	transcript_id "lnc-bbut00022058"	exon_number "1"
18	lncRNA	exon	14803180	14803325	.	+	.	gene_id "lnc-bbug09590"	transcript_id "lnc-bbut00022058"	exon_number "2"
18	lncRNA	exon	14819730	14819774	.	+	.	gene_id "lnc-bbug09590"	transcript_id "lnc-bbut00022058"	exon_number "3"
18	lncRNA	exon	14821647	14824787	.	+	.	gene_id "lnc-bbug09591"	transcript_id "lnc-bbut00022059"	exon_number "1"
18	lncRNA	exon	15129390	15129753	.	+	.	gene_id "lnc-bbug09593"	transcript_id "lnc-bbut00022062"	exon_number "1"
18	lncRNA	exon	15175668	15175820	.	+	.	gene_id "lnc-bbug09593"	transcript_id "lnc-bbut00022062"	exon_number "2"
18	lncRNA	exon	15177997	15178096	.	+	.	gene_id "lnc-bbug09593"	transcript_id "lnc-bbut00022062"	exon_number "3"
18	lncRNA	exon	15209624	15209858	.	+	.	gene_id "lnc-bbug09593"	transcript_id "lnc-bbut00022062"	exon_number "4"
18	lncRNA	exon	15129390	15129753	.	+	.	gene_id "lnc-bbug09593"	transcript_id "lnc-bbut00022061"	exon_number "1"
18	lncRNA	exon	15175668	15175820	.	+	.	gene_id "lnc-bbug09593"	transcript_id "lnc-bbut00022061"	exon_number "2"
18	lncRNA	exon	15209624	15209858	.	+	.	gene_id "lnc-bbug09593"	transcript_id "lnc-bbut00022061"	exon_number "3"
18	lncRNA	exon	16074567	16074651	.	+	.	gene_id "lnc-bbug09596"	transcript_id "lnc-bbut00022073"	exon_number "1"
18	lncRNA	exon	16077251	16077352	.	+	.	gene_id "lnc-bbug09596"	transcript_id "lnc-bbut00022073"	exon_number "2"
18	lncRNA	exon	16080806	16080886	.	+	.	gene_id "lnc-bbug09596"	transcript_id "lnc-bbut00022073"	exon_number "3"
18	lncRNA	exon	16083827	16084341	.	+	.	gene_id "lnc-bbug09596"	transcript_id "lnc-bbut00022073"	exon_number "4"
18	lncRNA	exon	16076291	16077352	.	+	.	gene_id "lnc-bbug09596"	transcript_id "lnc-bbut00022074"	exon_number "1"
18	lncRNA	exon	16080806	16080886	.	+	.	gene_id "lnc-bbug09596"	transcript_id "lnc-bbut00022074"	exon_number "2"
18	lncRNA	exon	16083827	16084341	.	+	.	gene_id "lnc-bbug09596"	transcript_id "lnc-bbut00022074"	exon_number "3"
18	lncRNA	exon	18046616	18046754	.	+	.	gene_id "lnc-bbug09599"	transcript_id "lnc-bbut00022080"	exon_number "1"
18	lncRNA	exon	18047808	18047879	.	+	.	gene_id "lnc-bbug09599"	transcript_id "lnc-bbut00022080"	exon_number "2"
18	lncRNA	exon	18050073	18050146	.	+	.	gene_id "lnc-bbug09599"	transcript_id "lnc-bbut00022080"	exon_number "3"
18	lncRNA	exon	18054803	18054912	.	+	.	gene_id "lnc-bbug09599"	transcript_id "lnc-bbut00022080"	exon_number "4"
18	lncRNA	exon	18055028	18055082	.	+	.	gene_id "lnc-bbug09599"	transcript_id "lnc-bbut00022080"	exon_number "5"
18	lncRNA	exon	18059852	18061209	.	+	.	gene_id "lnc-bbug09599"	transcript_id "lnc-bbut00022080"	exon_number "6"
18	lncRNA	exon	19182636	19183527	.	+	.	gene_id "lnc-bbug09607"	transcript_id "lnc-bbut00022117"	exon_number "1"
18	lncRNA	exon	19183680	19186761	.	+	.	gene_id "lnc-bbug09607"	transcript_id "lnc-bbut00022117"	exon_number "2"
18	lncRNA	exon	19194393	19195437	.	+	.	gene_id "lnc-bbug09607"	transcript_id "lnc-bbut00022117"	exon_number "3"
18	lncRNA	exon	21626286	21627531	.	+	.	gene_id "lnc-bbug09612"	transcript_id "lnc-bbut00022140"	exon_number "1"
18	lncRNA	exon	21631513	21632791	.	+	.	gene_id "lnc-bbug09612"	transcript_id "lnc-bbut00022140"	exon_number "2"
18	lncRNA	exon	23015659	23015690	.	+	.	gene_id "lnc-bbug09616"	transcript_id "lnc-bbut00022145"	exon_number "1"
18	lncRNA	exon	23064195	23064535	.	+	.	gene_id "lnc-bbug09616"	transcript_id "lnc-bbut00022145"	exon_number "2"
18	lncRNA	exon	23208916	23209254	.	+	.	gene_id "lnc-bbug09619"	transcript_id "lnc-bbut00022148"	exon_number "1"
18	lncRNA	exon	23209678	23209785	.	+	.	gene_id "lnc-bbug09619"	transcript_id "lnc-bbut00022148"	exon_number "2"
18	lncRNA	exon	23231632	23232803	.	+	.	gene_id "lnc-bbug09619"	transcript_id "lnc-bbut00022148"	exon_number "3"
18	lncRNA	exon	24267729	24267899	.	+	.	gene_id "lnc-bbug09630"	transcript_id "lnc-bbut00022173"	exon_number "1"
18	lncRNA	exon	24270283	24270366	.	+	.	gene_id "lnc-bbug09630"	transcript_id "lnc-bbut00022173"	exon_number "2"
18	lncRNA	exon	24271036	24271767	.	+	.	gene_id "lnc-bbug09630"	transcript_id "lnc-bbut00022173"	exon_number "3"
18	lncRNA	exon	24295048	24295138	.	+	.	gene_id "lnc-bbug09631"	transcript_id "lnc-bbut00022174"	exon_number "1"
18	lncRNA	exon	24297605	24297809	.	+	.	gene_id "lnc-bbug09631"	transcript_id "lnc-bbut00022174"	exon_number "2"
18	lncRNA	exon	24301396	24301540	.	+	.	gene_id "lnc-bbug09631"	transcript_id "lnc-bbut00022174"	exon_number "3"
18	lncRNA	exon	24303879	24304012	.	+	.	gene_id "lnc-bbug09631"	transcript_id "lnc-bbut00022174"	exon_number "4"
18	lncRNA	exon	24306094	24306247	.	+	.	gene_id "lnc-bbug09631"	transcript_id "lnc-bbut00022174"	exon_number "5"
18	lncRNA	exon	24307944	24308048	.	+	.	gene_id "lnc-bbug09631"	transcript_id "lnc-bbut00022174"	exon_number "6"
18	lncRNA	exon	24310270	24310374	.	+	.	gene_id "lnc-bbug09631"	transcript_id "lnc-bbut00022174"	exon_number "7"
18	lncRNA	exon	24312708	24312746	.	+	.	gene_id "lnc-bbug09631"	transcript_id "lnc-bbut00022174"	exon_number "8"
18	lncRNA	exon	24315258	24315398	.	+	.	gene_id "lnc-bbug09631"	transcript_id "lnc-bbut00022174"	exon_number "9"
18	lncRNA	exon	24317195	24317275	.	+	.	gene_id "lnc-bbug09631"	transcript_id "lnc-bbut00022174"	exon_number "10"
18	lncRNA	exon	24317556	24317703	.	+	.	gene_id "lnc-bbug09631"	transcript_id "lnc-bbut00022174"	exon_number "11"
18	lncRNA	exon	24321767	24321898	.	+	.	gene_id "lnc-bbug09631"	transcript_id "lnc-bbut00022174"	exon_number "12"
18	lncRNA	exon	24322888	24322960	.	+	.	gene_id "lnc-bbug09631"	transcript_id "lnc-bbut00022174"	exon_number "13"
18	lncRNA	exon	24323225	24323407	.	+	.	gene_id "lnc-bbug09631"	transcript_id "lnc-bbut00022174"	exon_number "14"
18	lncRNA	exon	24326132	24326250	.	+	.	gene_id "lnc-bbug09632"	transcript_id "lnc-bbut00022175"	exon_number "1"

18	lncRNA	exon	24328139	24328303	.	+	.	gene_id "lnc-bbug09632"	transcript_id "lnc-bbut00022175"	exon_number "2"
18	lncRNA	exon	24330868	24331104	.	+	.	gene_id "lnc-bbug09632"	transcript_id "lnc-bbut00022175"	exon_number "3"
18	lncRNA	exon	24529821	24531818	.	+	.	gene_id "lnc-bbug09636"	transcript_id "lnc-bbut00022187"	exon_number "1"
18	lncRNA	exon	24533125	24533896	.	+	.	gene_id "lnc-bbug09636"	transcript_id "lnc-bbut00022187"	exon_number "2"
18	lncRNA	exon	24909161	24909292	.	+	.	gene_id "lnc-bbug09643"	transcript_id "lnc-bbut00022198"	exon_number "1"
18	lncRNA	exon	24910679	24910802	.	+	.	gene_id "lnc-bbug09643"	transcript_id "lnc-bbut00022198"	exon_number "2"
18	lncRNA	exon	24915439	24917219	.	+	.	gene_id "lnc-bbug09643"	transcript_id "lnc-bbut00022198"	exon_number "3"
18	lncRNA	exon	25025975	25026124	.	+	.	gene_id "lnc-bbug09648"	transcript_id "lnc-bbut00022204"	exon_number "1"
18	lncRNA	exon	25026594	25026653	.	+	.	gene_id "lnc-bbug09648"	transcript_id "lnc-bbut00022204"	exon_number "2"
18	lncRNA	exon	25027821	25028076	.	+	.	gene_id "lnc-bbug09648"	transcript_id "lnc-bbut00022204"	exon_number "3"
18	lncRNA	exon	25529441	25530590	.	+	.	gene_id "lnc-bbug09657"	transcript_id "lnc-bbut00022240"	exon_number "1"
18	lncRNA	exon	25531578	25531841	.	+	.	gene_id "lnc-bbug09657"	transcript_id "lnc-bbut00022240"	exon_number "2"
18	lncRNA	exon	25568409	25568718	.	+	.	gene_id "lnc-bbug09658"	transcript_id "lnc-bbut00022241"	exon_number "1"
18	lncRNA	exon	25571557	25571693	.	+	.	gene_id "lnc-bbug09658"	transcript_id "lnc-bbut00022241"	exon_number "2"
18	lncRNA	exon	25575607	25575732	.	+	.	gene_id "lnc-bbug09658"	transcript_id "lnc-bbut00022241"	exon_number "3"
18	lncRNA	exon	25587349	25587463	.	+	.	gene_id "lnc-bbug09658"	transcript_id "lnc-bbut00022241"	exon_number "4"
18	lncRNA	exon	25588298	25588573	.	+	.	gene_id "lnc-bbug09658"	transcript_id "lnc-bbut00022241"	exon_number "5"
18	lncRNA	exon	28474093	28474236	.	+	.	gene_id "lnc-bbug09664"	transcript_id "lnc-bbut00022257"	exon_number "1"
18	lncRNA	exon	28481787	28481823	.	+	.	gene_id "lnc-bbug09664"	transcript_id "lnc-bbut00022257"	exon_number "2"
18	lncRNA	exon	28484705	28488384	.	+	.	gene_id "lnc-bbug09664"	transcript_id "lnc-bbut00022257"	exon_number "3"
18	lncRNA	exon	32416068	32416480	.	+	.	gene_id "lnc-bbug09666"	transcript_id "lnc-bbut00022260"	exon_number "1"
18	lncRNA	exon	32452332	32452491	.	+	.	gene_id "lnc-bbug09666"	transcript_id "lnc-bbut00022260"	exon_number "2"
18	lncRNA	exon	32452817	32452956	.	+	.	gene_id "lnc-bbug09666"	transcript_id "lnc-bbut00022260"	exon_number "3"
18	lncRNA	exon	32454424	32454886	.	+	.	gene_id "lnc-bbug09666"	transcript_id "lnc-bbut00022260"	exon_number "4"
18	lncRNA	exon	32416068	32416480	.	+	.	gene_id "lnc-bbug09666"	transcript_id "lnc-bbut00022259"	exon_number "1"
18	lncRNA	exon	32452332	32452491	.	+	.	gene_id "lnc-bbug09666"	transcript_id "lnc-bbut00022259"	exon_number "2"
18	lncRNA	exon	32454424	32454886	.	+	.	gene_id "lnc-bbug09666"	transcript_id "lnc-bbut00022259"	exon_number "3"
18	lncRNA	exon	32416068	32416480	.	+	.	gene_id "lnc-bbug09666"	transcript_id "lnc-bbut00022261"	exon_number "1"
18	lncRNA	exon	32452332	32452491	.	+	.	gene_id "lnc-bbug09666"	transcript_id "lnc-bbut00022261"	exon_number "2"
18	lncRNA	exon	32452817	32452956	.	+	.	gene_id "lnc-bbug09666"	transcript_id "lnc-bbut00022261"	exon_number "3"
18	lncRNA	exon	32454424	32454628	.	+	.	gene_id "lnc-bbug09666"	transcript_id "lnc-bbut00022261"	exon_number "4"
18	lncRNA	exon	32459236	32460147	.	+	.	gene_id "lnc-bbug09666"	transcript_id "lnc-bbut00022261"	exon_number "5"
18	lncRNA	exon	32451655	32452491	.	+	.	gene_id "lnc-bbug09666"	transcript_id "lnc-bbut00022262"	exon_number "1"
18	lncRNA	exon	32452817	32452956	.	+	.	gene_id "lnc-bbug09666"	transcript_id "lnc-bbut00022262"	exon_number "2"
18	lncRNA	exon	32563939	32564323	.	+	.	gene_id "lnc-bbug09667"	transcript_id "lnc-bbut00022263"	exon_number "1"
18	lncRNA	exon	32570272	32570363	.	+	.	gene_id "lnc-bbug09667"	transcript_id "lnc-bbut00022263"	exon_number "2"
18	lncRNA	exon	32570645	32571215	.	+	.	gene_id "lnc-bbug09667"	transcript_id "lnc-bbut00022263"	exon_number "3"
18	lncRNA	exon	32569136	32570168	.	+	.	gene_id "lnc-bbug09667"	transcript_id "lnc-bbut00022264"	exon_number "1"
18	lncRNA	exon	32570276	32570363	.	+	.	gene_id "lnc-bbug09667"	transcript_id "lnc-bbut00022264"	exon_number "2"
18	lncRNA	exon	32570645	32571215	.	+	.	gene_id "lnc-bbug09667"	transcript_id "lnc-bbut00022264"	exon_number "3"
18	lncRNA	exon	34392742	34393029	.	+	.	gene_id "lnc-bbug09681"	transcript_id "lnc-bbut00022292"	exon_number "1"
18	lncRNA	exon	34393253	34393345	.	+	.	gene_id "lnc-bbug09681"	transcript_id "lnc-bbut00022292"	exon_number "2"
18	lncRNA	exon	34395938	34396074	.	+	.	gene_id "lnc-bbug09681"	transcript_id "lnc-bbut00022292"	exon_number "3"
18	lncRNA	exon	34953424	34953628	.	+	.	gene_id "lnc-bbug09700"	transcript_id "lnc-bbut00022355"	exon_number "1"
18	lncRNA	exon	34953761	34953863	.	+	.	gene_id "lnc-bbug09700"	transcript_id "lnc-bbut00022355"	exon_number "2"
18	lncRNA	exon	34953986	34954174	.	+	.	gene_id "lnc-bbug09700"	transcript_id "lnc-bbut00022355"	exon_number "3"
18	lncRNA	exon	35430358	35430640	.	+	.	gene_id "lnc-bbug09713"	transcript_id "lnc-bbut00022392"	exon_number "1"
18	lncRNA	exon	35434112	35434332	.	+	.	gene_id "lnc-bbug09713"	transcript_id "lnc-bbut00022392"	exon_number "2"
18	lncRNA	exon	35439428	35439577	.	+	.	gene_id "lnc-bbug09713"	transcript_id "lnc-bbut00022392"	exon_number "3"
18	lncRNA	exon	35442196	35442378	.	+	.	gene_id "lnc-bbug09713"	transcript_id "lnc-bbut00022392"	exon_number "4"
18	lncRNA	exon	35447420	35447635	.	+	.	gene_id "lnc-bbug09713"	transcript_id "lnc-bbut00022392"	exon_number "5"
18	lncRNA	exon	35450238	35450524	.	+	.	gene_id "lnc-bbug09713"	transcript_id "lnc-bbut00022392"	exon_number "6"

18	lncRNA	exon	35452252	35452524	.	+	.	gene_id "lnc-bbug09713"	transcript_id "lnc-bbut00022392"	exon_number "7"
18	lncRNA	exon	35453166	35453336	.	+	.	gene_id "lnc-bbug09713"	transcript_id "lnc-bbut00022392"	exon_number "8"
18	lncRNA	exon	35468051	35468754	.	+	.	gene_id "lnc-bbug09713"	transcript_id "lnc-bbut00022392"	exon_number "9"
18	lncRNA	exon	35470953	35471700	.	+	.	gene_id "lnc-bbug09713"	transcript_id "lnc-bbut00022392"	exon_number "10"
18	lncRNA	exon	35433101	35433430	.	+	.	gene_id "lnc-bbug09713"	transcript_id "lnc-bbut00022393"	exon_number "1"
18	lncRNA	exon	35434112	35434332	.	+	.	gene_id "lnc-bbug09713"	transcript_id "lnc-bbut00022393"	exon_number "2"
18	lncRNA	exon	35439428	35439577	.	+	.	gene_id "lnc-bbug09713"	transcript_id "lnc-bbut00022393"	exon_number "3"
18	lncRNA	exon	35442196	35442378	.	+	.	gene_id "lnc-bbug09713"	transcript_id "lnc-bbut00022393"	exon_number "4"
18	lncRNA	exon	35447420	35447635	.	+	.	gene_id "lnc-bbug09713"	transcript_id "lnc-bbut00022393"	exon_number "5"
18	lncRNA	exon	35450238	35450524	.	+	.	gene_id "lnc-bbug09713"	transcript_id "lnc-bbut00022393"	exon_number "6"
18	lncRNA	exon	35451177	35451324	.	+	.	gene_id "lnc-bbug09713"	transcript_id "lnc-bbut00022393"	exon_number "7"
18	lncRNA	exon	35452252	35452524	.	+	.	gene_id "lnc-bbug09713"	transcript_id "lnc-bbut00022393"	exon_number "8"
18	lncRNA	exon	35453166	35453336	.	+	.	gene_id "lnc-bbug09713"	transcript_id "lnc-bbut00022393"	exon_number "9"
18	lncRNA	exon	35468051	35468754	.	+	.	gene_id "lnc-bbug09713"	transcript_id "lnc-bbut00022393"	exon_number "10"
18	lncRNA	exon	35470953	35471700	.	+	.	gene_id "lnc-bbug09713"	transcript_id "lnc-bbut00022393"	exon_number "11"
18	lncRNA	exon	36683297	36683395	.	+	.	gene_id "lnc-bbug09723"	transcript_id "lnc-bbut00022437"	exon_number "1"
18	lncRNA	exon	36684765	36684851	.	+	.	gene_id "lnc-bbug09723"	transcript_id "lnc-bbut00022437"	exon_number "2"
18	lncRNA	exon	36696216	36696263	.	+	.	gene_id "lnc-bbug09723"	transcript_id "lnc-bbut00022437"	exon_number "3"
18	lncRNA	exon	37749917	37750028	.	+	.	gene_id "lnc-bbug09724"	transcript_id "lnc-bbut00022438"	exon_number "1"
18	lncRNA	exon	37750221	37750300	.	+	.	gene_id "lnc-bbug09724"	transcript_id "lnc-bbut00022438"	exon_number "2"
18	lncRNA	exon	37786886	37788238	.	+	.	gene_id "lnc-bbug09724"	transcript_id "lnc-bbut00022438"	exon_number "3"
18	lncRNA	exon	39224888	39225761	.	+	.	gene_id "lnc-bbug09732"	transcript_id "lnc-bbut00022462"	exon_number "1"
18	lncRNA	exon	39228445	39228785	.	+	.	gene_id "lnc-bbug09732"	transcript_id "lnc-bbut00022462"	exon_number "2"
18	lncRNA	exon	39388808	39389246	.	+	.	gene_id "lnc-bbug09736"	transcript_id "lnc-bbut00022468"	exon_number "1"
18	lncRNA	exon	39390063	39390212	.	+	.	gene_id "lnc-bbug09736"	transcript_id "lnc-bbut00022468"	exon_number "2"
18	lncRNA	exon	39390979	39391009	.	+	.	gene_id "lnc-bbug09736"	transcript_id "lnc-bbut00022468"	exon_number "3"
18	lncRNA	exon	39394346	39397983	.	+	.	gene_id "lnc-bbug09736"	transcript_id "lnc-bbut00022468"	exon_number "4"
18	lncRNA	exon	39527814	39529011	.	+	.	gene_id "lnc-bbug09738"	transcript_id "lnc-bbut00022472"	exon_number "1"
18	lncRNA	exon	40454699	40454888	.	+	.	gene_id "lnc-bbug09743"	transcript_id "lnc-bbut00022482"	exon_number "1"
18	lncRNA	exon	40459353	40459450	.	+	.	gene_id "lnc-bbug09743"	transcript_id "lnc-bbut00022482"	exon_number "2"
18	lncRNA	exon	40460473	40460889	.	+	.	gene_id "lnc-bbug09743"	transcript_id "lnc-bbut00022482"	exon_number "3"
18	lncRNA	exon	42228650	42228883	.	+	.	gene_id "lnc-bbug09747"	transcript_id "lnc-bbut00022492"	exon_number "1"
18	lncRNA	exon	42230801	42232589	.	+	.	gene_id "lnc-bbug09747"	transcript_id "lnc-bbut00022492"	exon_number "2"
18	lncRNA	exon	42232995	42235181	.	+	.	gene_id "lnc-bbug09747"	transcript_id "lnc-bbut00022492"	exon_number "3"
18	lncRNA	exon	42861847	42861996	.	+	.	gene_id "lnc-bbug09751"	transcript_id "lnc-bbut00022499"	exon_number "1"
18	lncRNA	exon	42862442	42862733	.	+	.	gene_id "lnc-bbug09751"	transcript_id "lnc-bbut00022499"	exon_number "2"
18	lncRNA	exon	42943283	42945118	.	+	.	gene_id "lnc-bbug09754"	transcript_id "lnc-bbut00022502"	exon_number "1"
18	lncRNA	exon	43555375	43555555	.	+	.	gene_id "lnc-bbug09761"	transcript_id "lnc-bbut00022518"	exon_number "1"
18	lncRNA	exon	43561522	43565054	.	+	.	gene_id "lnc-bbug09761"	transcript_id "lnc-bbut00022518"	exon_number "2"
18	lncRNA	exon	45065092	45065194	.	+	.	gene_id "lnc-bbug09775"	transcript_id "lnc-bbut00022544"	exon_number "1"
18	lncRNA	exon	45065707	45065890	.	+	.	gene_id "lnc-bbug09775"	transcript_id "lnc-bbut00022544"	exon_number "2"
18	lncRNA	exon	45067093	45067320	.	+	.	gene_id "lnc-bbug09775"	transcript_id "lnc-bbut00022544"	exon_number "3"
18	lncRNA	exon	45396822	45397111	.	+	.	gene_id "lnc-bbug09777"	transcript_id "lnc-bbut00022550"	exon_number "1"
18	lncRNA	exon	45399341	45399457	.	+	.	gene_id "lnc-bbug09777"	transcript_id "lnc-bbut00022550"	exon_number "2"
18	lncRNA	exon	45403412	45405537	.	+	.	gene_id "lnc-bbug09777"	transcript_id "lnc-bbut00022550"	exon_number "3"
18	lncRNA	exon	45421615	45424259	.	+	.	gene_id "lnc-bbug09778"	transcript_id "lnc-bbut00022551"	exon_number "1"
18	lncRNA	exon	45607199	45607309	.	+	.	gene_id "lnc-bbug09783"	transcript_id "lnc-bbut00022562"	exon_number "1"
18	lncRNA	exon	45610344	45610406	.	+	.	gene_id "lnc-bbug09783"	transcript_id "lnc-bbut00022562"	exon_number "2"
18	lncRNA	exon	45611771	45611803	.	+	.	gene_id "lnc-bbug09783"	transcript_id "lnc-bbut00022562"	exon_number "3"
18	lncRNA	exon	45611911	45611934	.	+	.	gene_id "lnc-bbug09783"	transcript_id "lnc-bbut00022562"	exon_number "4"
18	lncRNA	exon	45612497	45612571	.	+	.	gene_id "lnc-bbug09783"	transcript_id "lnc-bbut00022562"	exon_number "5"
18	lncRNA	exon	45612658	45612694	.	+	.	gene_id "lnc-bbug09783"	transcript_id "lnc-bbut00022562"	exon_number "6"

18	lncRNA	exon	45613015	45613052	.	+	.	gene_id "lnc-bbug09783"	transcript_id "lnc-bbut00022562"	exon_number "7"
18	lncRNA	exon	45613205	45613413	.	+	.	gene_id "lnc-bbug09783"	transcript_id "lnc-bbut00022562"	exon_number "8"
18	lncRNA	exon	45607207	45607309	.	+	.	gene_id "lnc-bbug09783"	transcript_id "lnc-bbut00022563"	exon_number "1"
18	lncRNA	exon	45610214	45610406	.	+	.	gene_id "lnc-bbug09783"	transcript_id "lnc-bbut00022563"	exon_number "2"
18	lncRNA	exon	45611771	45611803	.	+	.	gene_id "lnc-bbug09783"	transcript_id "lnc-bbut00022563"	exon_number "3"
18	lncRNA	exon	45611911	45611934	.	+	.	gene_id "lnc-bbug09783"	transcript_id "lnc-bbut00022563"	exon_number "4"
18	lncRNA	exon	45612497	45612571	.	+	.	gene_id "lnc-bbug09783"	transcript_id "lnc-bbut00022563"	exon_number "5"
18	lncRNA	exon	45612658	45612694	.	+	.	gene_id "lnc-bbug09783"	transcript_id "lnc-bbut00022563"	exon_number "6"
18	lncRNA	exon	45613015	45613052	.	+	.	gene_id "lnc-bbug09783"	transcript_id "lnc-bbut00022563"	exon_number "7"
18	lncRNA	exon	45613205	45613413	.	+	.	gene_id "lnc-bbug09783"	transcript_id "lnc-bbut00022563"	exon_number "8"
18	lncRNA	exon	45625287	45625439	.	+	.	gene_id "lnc-bbug09784"	transcript_id "lnc-bbut00022564"	exon_number "1"
18	lncRNA	exon	45625704	45625783	.	+	.	gene_id "lnc-bbug09784"	transcript_id "lnc-bbut00022564"	exon_number "2"
18	lncRNA	exon	45628930	45628962	.	+	.	gene_id "lnc-bbug09784"	transcript_id "lnc-bbut00022564"	exon_number "3"
18	lncRNA	exon	45629517	45629591	.	+	.	gene_id "lnc-bbug09784"	transcript_id "lnc-bbut00022564"	exon_number "4"
18	lncRNA	exon	45630076	45630112	.	+	.	gene_id "lnc-bbug09784"	transcript_id "lnc-bbut00022564"	exon_number "5"
18	lncRNA	exon	45631016	45631065	.	+	.	gene_id "lnc-bbug09784"	transcript_id "lnc-bbut00022564"	exon_number "6"
18	lncRNA	exon	45631246	45631545	.	+	.	gene_id "lnc-bbug09784"	transcript_id "lnc-bbut00022564"	exon_number "7"
18	lncRNA	exon	45625290	45625439	.	+	.	gene_id "lnc-bbug09784"	transcript_id "lnc-bbut00022565"	exon_number "1"
18	lncRNA	exon	45625704	45625783	.	+	.	gene_id "lnc-bbug09784"	transcript_id "lnc-bbut00022565"	exon_number "2"
18	lncRNA	exon	45628409	45628473	.	+	.	gene_id "lnc-bbug09784"	transcript_id "lnc-bbut00022565"	exon_number "3"
18	lncRNA	exon	45628930	45628962	.	+	.	gene_id "lnc-bbug09784"	transcript_id "lnc-bbut00022565"	exon_number "4"
18	lncRNA	exon	45629517	45629591	.	+	.	gene_id "lnc-bbug09784"	transcript_id "lnc-bbut00022565"	exon_number "5"
18	lncRNA	exon	45630076	45630112	.	+	.	gene_id "lnc-bbug09784"	transcript_id "lnc-bbut00022565"	exon_number "6"
18	lncRNA	exon	45631016	45631065	.	+	.	gene_id "lnc-bbug09784"	transcript_id "lnc-bbut00022565"	exon_number "7"
18	lncRNA	exon	45631246	45631545	.	+	.	gene_id "lnc-bbug09784"	transcript_id "lnc-bbut00022565"	exon_number "8"
18	lncRNA	exon	45625314	45625439	.	+	.	gene_id "lnc-bbug09784"	transcript_id "lnc-bbut00022566"	exon_number "1"
18	lncRNA	exon	45625645	45625783	.	+	.	gene_id "lnc-bbug09784"	transcript_id "lnc-bbut00022566"	exon_number "2"
18	lncRNA	exon	45628930	45628962	.	+	.	gene_id "lnc-bbug09784"	transcript_id "lnc-bbut00022566"	exon_number "3"
18	lncRNA	exon	45629517	45629591	.	+	.	gene_id "lnc-bbug09784"	transcript_id "lnc-bbut00022566"	exon_number "4"
18	lncRNA	exon	45630076	45630112	.	+	.	gene_id "lnc-bbug09784"	transcript_id "lnc-bbut00022566"	exon_number "5"
18	lncRNA	exon	45631016	45631065	.	+	.	gene_id "lnc-bbug09784"	transcript_id "lnc-bbut00022566"	exon_number "6"
18	lncRNA	exon	45631246	45631545	.	+	.	gene_id "lnc-bbug09784"	transcript_id "lnc-bbut00022566"	exon_number "7"
18	lncRNA	exon	45627093	45627236	.	+	.	gene_id "lnc-bbug09784"	transcript_id "lnc-bbut00022567"	exon_number "1"
18	lncRNA	exon	45628409	45628473	.	+	.	gene_id "lnc-bbug09784"	transcript_id "lnc-bbut00022567"	exon_number "2"
18	lncRNA	exon	45628930	45628962	.	+	.	gene_id "lnc-bbug09784"	transcript_id "lnc-bbut00022567"	exon_number "3"
18	lncRNA	exon	45629517	45629591	.	+	.	gene_id "lnc-bbug09784"	transcript_id "lnc-bbut00022567"	exon_number "4"
18	lncRNA	exon	45630076	45630112	.	+	.	gene_id "lnc-bbug09784"	transcript_id "lnc-bbut00022567"	exon_number "5"
18	lncRNA	exon	45631016	45631065	.	+	.	gene_id "lnc-bbug09784"	transcript_id "lnc-bbut00022567"	exon_number "6"
18	lncRNA	exon	45631246	45631545	.	+	.	gene_id "lnc-bbug09784"	transcript_id "lnc-bbut00022567"	exon_number "7"
18	lncRNA	exon	45627206	45627321	.	+	.	gene_id "lnc-bbug09784"	transcript_id "lnc-bbut00022568"	exon_number "1"
18	lncRNA	exon	45628409	45628473	.	+	.	gene_id "lnc-bbug09784"	transcript_id "lnc-bbut00022568"	exon_number "2"
18	lncRNA	exon	45628930	45628962	.	+	.	gene_id "lnc-bbug09784"	transcript_id "lnc-bbut00022568"	exon_number "3"
18	lncRNA	exon	45629517	45629591	.	+	.	gene_id "lnc-bbug09784"	transcript_id "lnc-bbut00022568"	exon_number "4"
18	lncRNA	exon	45630076	45630112	.	+	.	gene_id "lnc-bbug09784"	transcript_id "lnc-bbut00022568"	exon_number "5"
18	lncRNA	exon	45631016	45631065	.	+	.	gene_id "lnc-bbug09784"	transcript_id "lnc-bbut00022568"	exon_number "6"
18	lncRNA	exon	45631246	45631545	.	+	.	gene_id "lnc-bbug09784"	transcript_id "lnc-bbut00022568"	exon_number "7"
18	lncRNA	exon	45627969	45628110	.	+	.	gene_id "lnc-bbug09784"	transcript_id "lnc-bbut00022570"	exon_number "1"
18	lncRNA	exon	45628409	45628473	.	+	.	gene_id "lnc-bbug09784"	transcript_id "lnc-bbut00022570"	exon_number "2"
18	lncRNA	exon	45628930	45628962	.	+	.	gene_id "lnc-bbug09784"	transcript_id "lnc-bbut00022570"	exon_number "3"
18	lncRNA	exon	45629517	45629591	.	+	.	gene_id "lnc-bbug09784"	transcript_id "lnc-bbut00022570"	exon_number "4"
18	lncRNA	exon	45630076	45630112	.	+	.	gene_id "lnc-bbug09784"	transcript_id "lnc-bbut00022570"	exon_number "5"
18	lncRNA	exon	45631016	45631065	.	+	.	gene_id "lnc-bbug09784"	transcript_id "lnc-bbut00022570"	exon_number "6"

18	lncRNA	exon	45631246	45631545	.	+	.	gene_id "lnc-bbug09784"	transcript_id "lnc-bbut00022570"	exon_number "7"
18	lncRNA	exon	45631675	45631865	.	+	.	gene_id "lnc-bbug09785"	transcript_id "lnc-bbut00022573"	exon_number "1"
18	lncRNA	exon	45636382	45636411	.	+	.	gene_id "lnc-bbug09785"	transcript_id "lnc-bbut00022573"	exon_number "2"
18	lncRNA	exon	45638344	45638418	.	+	.	gene_id "lnc-bbug09785"	transcript_id "lnc-bbut00022573"	exon_number "3"
18	lncRNA	exon	45638560	45638593	.	+	.	gene_id "lnc-bbug09785"	transcript_id "lnc-bbut00022573"	exon_number "4"
18	lncRNA	exon	45638765	45638781	.	+	.	gene_id "lnc-bbug09785"	transcript_id "lnc-bbut00022573"	exon_number "5"
18	lncRNA	exon	45639025	45639137	.	+	.	gene_id "lnc-bbug09785"	transcript_id "lnc-bbut00022573"	exon_number "6"
18	lncRNA	exon	45640314	45640698	.	+	.	gene_id "lnc-bbug09785"	transcript_id "lnc-bbut00022573"	exon_number "7"
18	lncRNA	exon	45631675	45631865	.	+	.	gene_id "lnc-bbug09785"	transcript_id "lnc-bbut00022575"	exon_number "1"
18	lncRNA	exon	45636382	45636411	.	+	.	gene_id "lnc-bbug09785"	transcript_id "lnc-bbut00022575"	exon_number "2"
18	lncRNA	exon	45638344	45638418	.	+	.	gene_id "lnc-bbug09785"	transcript_id "lnc-bbut00022575"	exon_number "3"
18	lncRNA	exon	45638560	45638593	.	+	.	gene_id "lnc-bbug09785"	transcript_id "lnc-bbut00022575"	exon_number "4"
18	lncRNA	exon	45638765	45638805	.	+	.	gene_id "lnc-bbug09785"	transcript_id "lnc-bbut00022575"	exon_number "5"
18	lncRNA	exon	45639047	45639137	.	+	.	gene_id "lnc-bbug09785"	transcript_id "lnc-bbut00022575"	exon_number "6"
18	lncRNA	exon	45640314	45640698	.	+	.	gene_id "lnc-bbug09785"	transcript_id "lnc-bbut00022575"	exon_number "7"
18	lncRNA	exon	45631675	45631865	.	+	.	gene_id "lnc-bbug09785"	transcript_id "lnc-bbut00022577"	exon_number "1"
18	lncRNA	exon	45636382	45636411	.	+	.	gene_id "lnc-bbug09785"	transcript_id "lnc-bbut00022577"	exon_number "2"
18	lncRNA	exon	45638344	45638418	.	+	.	gene_id "lnc-bbug09785"	transcript_id "lnc-bbut00022577"	exon_number "3"
18	lncRNA	exon	45638560	45638602	.	+	.	gene_id "lnc-bbug09785"	transcript_id "lnc-bbut00022577"	exon_number "4"
18	lncRNA	exon	45638765	45638805	.	+	.	gene_id "lnc-bbug09785"	transcript_id "lnc-bbut00022577"	exon_number "5"
18	lncRNA	exon	45639025	45639137	.	+	.	gene_id "lnc-bbug09785"	transcript_id "lnc-bbut00022577"	exon_number "6"
18	lncRNA	exon	45640314	45640698	.	+	.	gene_id "lnc-bbug09785"	transcript_id "lnc-bbut00022577"	exon_number "7"
18	lncRNA	exon	45631675	45631865	.	+	.	gene_id "lnc-bbug09785"	transcript_id "lnc-bbut00022578"	exon_number "1"
18	lncRNA	exon	45636382	45636411	.	+	.	gene_id "lnc-bbug09785"	transcript_id "lnc-bbut00022578"	exon_number "2"
18	lncRNA	exon	45638344	45638418	.	+	.	gene_id "lnc-bbug09785"	transcript_id "lnc-bbut00022578"	exon_number "3"
18	lncRNA	exon	45638560	45638602	.	+	.	gene_id "lnc-bbug09785"	transcript_id "lnc-bbut00022578"	exon_number "4"
18	lncRNA	exon	45638765	45638805	.	+	.	gene_id "lnc-bbug09785"	transcript_id "lnc-bbut00022578"	exon_number "5"
18	lncRNA	exon	45639047	45639137	.	+	.	gene_id "lnc-bbug09785"	transcript_id "lnc-bbut00022578"	exon_number "6"
18	lncRNA	exon	45640314	45640698	.	+	.	gene_id "lnc-bbug09785"	transcript_id "lnc-bbut00022578"	exon_number "7"
18	lncRNA	exon	45631675	45631865	.	+	.	gene_id "lnc-bbug09785"	transcript_id "lnc-bbut00022576"	exon_number "1"
18	lncRNA	exon	45636382	45636411	.	+	.	gene_id "lnc-bbug09785"	transcript_id "lnc-bbut00022576"	exon_number "2"
18	lncRNA	exon	45638344	45638418	.	+	.	gene_id "lnc-bbug09785"	transcript_id "lnc-bbut00022576"	exon_number "3"
18	lncRNA	exon	45638560	45638602	.	+	.	gene_id "lnc-bbug09785"	transcript_id "lnc-bbut00022576"	exon_number "4"
18	lncRNA	exon	45638765	45638781	.	+	.	gene_id "lnc-bbug09785"	transcript_id "lnc-bbut00022576"	exon_number "5"
18	lncRNA	exon	45639025	45639137	.	+	.	gene_id "lnc-bbug09785"	transcript_id "lnc-bbut00022576"	exon_number "6"
18	lncRNA	exon	45640314	45640698	.	+	.	gene_id "lnc-bbug09785"	transcript_id "lnc-bbut00022576"	exon_number "7"
18	lncRNA	exon	45631675	45631865	.	+	.	gene_id "lnc-bbug09785"	transcript_id "lnc-bbut00022574"	exon_number "1"
18	lncRNA	exon	45636382	45636411	.	+	.	gene_id "lnc-bbug09785"	transcript_id "lnc-bbut00022574"	exon_number "2"
18	lncRNA	exon	45638344	45638418	.	+	.	gene_id "lnc-bbug09785"	transcript_id "lnc-bbut00022574"	exon_number "3"
18	lncRNA	exon	45638560	45638593	.	+	.	gene_id "lnc-bbug09785"	transcript_id "lnc-bbut00022574"	exon_number "4"
18	lncRNA	exon	45638765	45638805	.	+	.	gene_id "lnc-bbug09785"	transcript_id "lnc-bbut00022574"	exon_number "5"
18	lncRNA	exon	45639025	45639137	.	+	.	gene_id "lnc-bbug09785"	transcript_id "lnc-bbut00022574"	exon_number "6"
18	lncRNA	exon	45640314	45640698	.	+	.	gene_id "lnc-bbug09785"	transcript_id "lnc-bbut00022574"	exon_number "7"
18	lncRNA	exon	45631675	45631865	.	+	.	gene_id "lnc-bbug09785"	transcript_id "lnc-bbut00022572"	exon_number "1"
18	lncRNA	exon	45636382	45636411	.	+	.	gene_id "lnc-bbug09785"	transcript_id "lnc-bbut00022572"	exon_number "2"
18	lncRNA	exon	45638344	45638418	.	+	.	gene_id "lnc-bbug09785"	transcript_id "lnc-bbut00022572"	exon_number "3"
18	lncRNA	exon	45638560	45638602	.	+	.	gene_id "lnc-bbug09785"	transcript_id "lnc-bbut00022572"	exon_number "4"
18	lncRNA	exon	45638765	45638805	.	+	.	gene_id "lnc-bbug09785"	transcript_id "lnc-bbut00022572"	exon_number "5"
18	lncRNA	exon	45640314	45640698	.	+	.	gene_id "lnc-bbug09785"	transcript_id "lnc-bbut00022572"	exon_number "6"
18	lncRNA	exon	45631675	45631865	.	+	.	gene_id "lnc-bbug09785"	transcript_id "lnc-bbut00022571"	exon_number "1"
18	lncRNA	exon	45636382	45636411	.	+	.	gene_id "lnc-bbug09785"	transcript_id "lnc-bbut00022571"	exon_number "2"
18	lncRNA	exon	45638344	45638418	.	+	.	gene_id "lnc-bbug09785"	transcript_id "lnc-bbut00022571"	exon_number "3"

18	lncRNA	exon	45638560	45638593	.	+	.	gene_id "lnc-bbug09785"	transcript_id "lnc-bbut00022571"	exon_number "4"
18	lncRNA	exon	45638765	45638805	.	+	.	gene_id "lnc-bbug09785"	transcript_id "lnc-bbut00022571"	exon_number "5"
18	lncRNA	exon	45640314	45640698	.	+	.	gene_id "lnc-bbug09785"	transcript_id "lnc-bbut00022571"	exon_number "6"
18	lncRNA	exon	45659631	45659841	.	+	.	gene_id "lnc-bbug09787"	transcript_id "lnc-bbut00022586"	exon_number "1"
18	lncRNA	exon	45660329	45660420	.	+	.	gene_id "lnc-bbug09787"	transcript_id "lnc-bbut00022586"	exon_number "2"
18	lncRNA	exon	45660983	45661812	.	+	.	gene_id "lnc-bbug09787"	transcript_id "lnc-bbut00022586"	exon_number "3"
18	lncRNA	exon	45758314	45758470	.	+	.	gene_id "lnc-bbug09790"	transcript_id "lnc-bbut00022593"	exon_number "1"
18	lncRNA	exon	45759415	45759474	.	+	.	gene_id "lnc-bbug09790"	transcript_id "lnc-bbut00022593"	exon_number "2"
18	lncRNA	exon	45759565	45759784	.	+	.	gene_id "lnc-bbug09790"	transcript_id "lnc-bbut00022593"	exon_number "3"
18	lncRNA	exon	45814107	45814566	.	+	.	gene_id "lnc-bbug09792"	transcript_id "lnc-bbut00022608"	exon_number "1"
18	lncRNA	exon	45815012	45815873	.	+	.	gene_id "lnc-bbug09792"	transcript_id "lnc-bbut00022608"	exon_number "2"
18	lncRNA	exon	45826476	45827105	.	+	.	gene_id "lnc-bbug09795"	transcript_id "lnc-bbut00022611"	exon_number "1"
18	lncRNA	exon	45829534	45830413	.	+	.	gene_id "lnc-bbug09795"	transcript_id "lnc-bbut00022611"	exon_number "2"
18	lncRNA	exon	45829030	45829078	.	+	.	gene_id "lnc-bbug09795"	transcript_id "lnc-bbut00022612"	exon_number "1"
18	lncRNA	exon	45829534	45830413	.	+	.	gene_id "lnc-bbug09795"	transcript_id "lnc-bbut00022612"	exon_number "2"
18	lncRNA	exon	45898230	45898810	.	+	.	gene_id "lnc-bbug09798"	transcript_id "lnc-bbut00022616"	exon_number "1"
18	lncRNA	exon	45899643	45899699	.	+	.	gene_id "lnc-bbug09798"	transcript_id "lnc-bbut00022616"	exon_number "2"
18	lncRNA	exon	45911366	45911766	.	+	.	gene_id "lnc-bbug09798"	transcript_id "lnc-bbut00022616"	exon_number "3"
18	lncRNA	exon	46053493	46053974	.	+	.	gene_id "lnc-bbug09801"	transcript_id "lnc-bbut00022619"	exon_number "1"
18	lncRNA	exon	46066914	46068090	.	+	.	gene_id "lnc-bbug09802"	transcript_id "lnc-bbut00022620"	exon_number "1"
18	lncRNA	exon	46068488	46068633	.	+	.	gene_id "lnc-bbug09802"	transcript_id "lnc-bbut00022620"	exon_number "2"
18	lncRNA	exon	46070288	46070441	.	+	.	gene_id "lnc-bbug09802"	transcript_id "lnc-bbut00022620"	exon_number "3"
18	lncRNA	exon	46070811	46071628	.	+	.	gene_id "lnc-bbug09803"	transcript_id "lnc-bbut00022621"	exon_number "1"
18	lncRNA	exon	46072418	46072448	.	+	.	gene_id "lnc-bbug09803"	transcript_id "lnc-bbut00022621"	exon_number "2"
18	lncRNA	exon	46160485	46160640	.	+	.	gene_id "lnc-bbug09808"	transcript_id "lnc-bbut00022629"	exon_number "1"
18	lncRNA	exon	46162206	46162322	.	+	.	gene_id "lnc-bbug09808"	transcript_id "lnc-bbut00022629"	exon_number "2"
18	lncRNA	exon	46163627	46163727	.	+	.	gene_id "lnc-bbug09808"	transcript_id "lnc-bbut00022629"	exon_number "3"
18	lncRNA	exon	46168800	46168991	.	+	.	gene_id "lnc-bbug09808"	transcript_id "lnc-bbut00022629"	exon_number "4"
18	lncRNA	exon	46160519	46160640	.	+	.	gene_id "lnc-bbug09808"	transcript_id "lnc-bbut00022630"	exon_number "1"
18	lncRNA	exon	46162123	46162322	.	+	.	gene_id "lnc-bbug09808"	transcript_id "lnc-bbut00022630"	exon_number "2"
18	lncRNA	exon	46163627	46163727	.	+	.	gene_id "lnc-bbug09808"	transcript_id "lnc-bbut00022630"	exon_number "3"
18	lncRNA	exon	46168800	46168991	.	+	.	gene_id "lnc-bbug09808"	transcript_id "lnc-bbut00022630"	exon_number "4"
18	lncRNA	exon	46245134	46245285	.	+	.	gene_id "lnc-bbug09812"	transcript_id "lnc-bbut00022637"	exon_number "1"
18	lncRNA	exon	46245419	46245571	.	+	.	gene_id "lnc-bbug09812"	transcript_id "lnc-bbut00022637"	exon_number "2"
18	lncRNA	exon	46245989	46246093	.	+	.	gene_id "lnc-bbug09812"	transcript_id "lnc-bbut00022637"	exon_number "3"
18	lncRNA	exon	46246280	46246548	.	+	.	gene_id "lnc-bbug09812"	transcript_id "lnc-bbut00022637"	exon_number "4"
18	lncRNA	exon	46245153	46245285	.	+	.	gene_id "lnc-bbug09812"	transcript_id "lnc-bbut00022638"	exon_number "1"
18	lncRNA	exon	46245457	46245571	.	+	.	gene_id "lnc-bbug09812"	transcript_id "lnc-bbut00022638"	exon_number "2"
18	lncRNA	exon	46245989	46246093	.	+	.	gene_id "lnc-bbug09812"	transcript_id "lnc-bbut00022638"	exon_number "3"
18	lncRNA	exon	46246280	46246548	.	+	.	gene_id "lnc-bbug09812"	transcript_id "lnc-bbut00022638"	exon_number "4"
18	lncRNA	exon	46372759	46372839	.	+	.	gene_id "lnc-bbug09818"	transcript_id "lnc-bbut00022647"	exon_number "1"
18	lncRNA	exon	46374841	46374936	.	+	.	gene_id "lnc-bbug09818"	transcript_id "lnc-bbut00022647"	exon_number "2"
18	lncRNA	exon	46375327	46375350	.	+	.	gene_id "lnc-bbug09818"	transcript_id "lnc-bbut00022647"	exon_number "3"
18	lncRNA	exon	46375454	46375560	.	+	.	gene_id "lnc-bbug09818"	transcript_id "lnc-bbut00022647"	exon_number "4"
18	lncRNA	exon	46375764	46375894	.	+	.	gene_id "lnc-bbug09818"	transcript_id "lnc-bbut00022647"	exon_number "5"
18	lncRNA	exon	46372788	46372839	.	+	.	gene_id "lnc-bbug09818"	transcript_id "lnc-bbut00022648"	exon_number "1"
18	lncRNA	exon	46374841	46374936	.	+	.	gene_id "lnc-bbug09818"	transcript_id "lnc-bbut00022648"	exon_number "2"
18	lncRNA	exon	46375327	46375350	.	+	.	gene_id "lnc-bbug09818"	transcript_id "lnc-bbut00022648"	exon_number "3"
18	lncRNA	exon	46375764	46375895	.	+	.	gene_id "lnc-bbug09818"	transcript_id "lnc-bbut00022648"	exon_number "4"
18	lncRNA	exon	46376123	46376283	.	+	.	gene_id "lnc-bbug09818"	transcript_id "lnc-bbut00022648"	exon_number "5"
18	lncRNA	exon	46372887	46374936	.	+	.	gene_id "lnc-bbug09818"	transcript_id "lnc-bbut00022649"	exon_number "1"
18	lncRNA	exon	46375327	46375350	.	+	.	gene_id "lnc-bbug09818"	transcript_id "lnc-bbut00022649"	exon_number "2"

18	lncRNA	exon	46375454	46375560	.	+	.	gene_id "lnc-bbug09818"	transcript_id "lnc-bbut00022649"	exon_number "3"
18	lncRNA	exon	46375764	46375894	.	+	.	gene_id "lnc-bbug09818"	transcript_id "lnc-bbut00022649"	exon_number "4"
18	lncRNA	exon	46380464	46380485	.	+	.	gene_id "lnc-bbug09819"	transcript_id "lnc-bbut00022650"	exon_number "1"
18	lncRNA	exon	46395879	46396235	.	+	.	gene_id "lnc-bbug09819"	transcript_id "lnc-bbut00022650"	exon_number "2"
18	lncRNA	exon	46452507	46453620	.	+	.	gene_id "lnc-bbug09821"	transcript_id "lnc-bbut00022656"	exon_number "1"
18	lncRNA	exon	47178879	47180957	.	+	.	gene_id "lnc-bbug09836"	transcript_id "lnc-bbut00022710"	exon_number "1"
18	lncRNA	exon	47468033	47468125	.	+	.	gene_id "lnc-bbug09840"	transcript_id "lnc-bbut00022721"	exon_number "1"
18	lncRNA	exon	47492284	47494185	.	+	.	gene_id "lnc-bbug09840"	transcript_id "lnc-bbut00022721"	exon_number "2"
18	lncRNA	exon	47495427	47495550	.	+	.	gene_id "lnc-bbug09840"	transcript_id "lnc-bbut00022721"	exon_number "3"
18	lncRNA	exon	47496620	47496872	.	+	.	gene_id "lnc-bbug09840"	transcript_id "lnc-bbut00022721"	exon_number "4"
18	lncRNA	exon	47541074	47541329	.	+	.	gene_id "lnc-bbug09841"	transcript_id "lnc-bbut00022723"	exon_number "1"
18	lncRNA	exon	47546449	47546679	.	+	.	gene_id "lnc-bbug09841"	transcript_id "lnc-bbut00022723"	exon_number "2"
18	lncRNA	exon	47547706	47547799	.	+	.	gene_id "lnc-bbug09841"	transcript_id "lnc-bbut00022723"	exon_number "3"
18	lncRNA	exon	47548683	47548782	.	+	.	gene_id "lnc-bbug09841"	transcript_id "lnc-bbut00022723"	exon_number "4"
18	lncRNA	exon	47549134	47549207	.	+	.	gene_id "lnc-bbug09841"	transcript_id "lnc-bbut00022723"	exon_number "5"
18	lncRNA	exon	47555218	47555396	.	+	.	gene_id "lnc-bbug09841"	transcript_id "lnc-bbut00022723"	exon_number "6"
18	lncRNA	exon	47560451	47561430	.	+	.	gene_id "lnc-bbug09841"	transcript_id "lnc-bbut00022723"	exon_number "7"
18	lncRNA	exon	47541074	47541329	.	+	.	gene_id "lnc-bbug09841"	transcript_id "lnc-bbut00022722"	exon_number "1"
18	lncRNA	exon	47547706	47547799	.	+	.	gene_id "lnc-bbug09841"	transcript_id "lnc-bbut00022722"	exon_number "2"
18	lncRNA	exon	47548683	47548782	.	+	.	gene_id "lnc-bbug09841"	transcript_id "lnc-bbut00022722"	exon_number "3"
18	lncRNA	exon	47549134	47549207	.	+	.	gene_id "lnc-bbug09841"	transcript_id "lnc-bbut00022722"	exon_number "4"
18	lncRNA	exon	47555218	47555396	.	+	.	gene_id "lnc-bbug09841"	transcript_id "lnc-bbut00022722"	exon_number "5"
18	lncRNA	exon	47560451	47561430	.	+	.	gene_id "lnc-bbug09841"	transcript_id "lnc-bbut00022722"	exon_number "6"
18	lncRNA	exon	47863497	47863893	.	+	.	gene_id "lnc-bbug09843"	transcript_id "lnc-bbut00022733"	exon_number "1"
18	lncRNA	exon	47864538	47864579	.	+	.	gene_id "lnc-bbug09843"	transcript_id "lnc-bbut00022733"	exon_number "2"
18	lncRNA	exon	47864953	47865168	.	+	.	gene_id "lnc-bbug09843"	transcript_id "lnc-bbut00022733"	exon_number "3"
18	lncRNA	exon	47914844	47914954	.	+	.	gene_id "lnc-bbug09845"	transcript_id "lnc-bbut00022735"	exon_number "1"
18	lncRNA	exon	47915071	47915171	.	+	.	gene_id "lnc-bbug09845"	transcript_id "lnc-bbut00022735"	exon_number "2"
18	lncRNA	exon	47915299	47915379	.	+	.	gene_id "lnc-bbug09845"	transcript_id "lnc-bbut00022735"	exon_number "3"
18	lncRNA	exon	47915513	47915654	.	+	.	gene_id "lnc-bbug09845"	transcript_id "lnc-bbut00022735"	exon_number "4"
18	lncRNA	exon	48309826	48309876	.	+	.	gene_id "lnc-bbug09854"	transcript_id "lnc-bbut00022766"	exon_number "1"
18	lncRNA	exon	48310011	48310835	.	+	.	gene_id "lnc-bbug09854"	transcript_id "lnc-bbut00022766"	exon_number "2"
18	lncRNA	exon	48439001	48439297	.	+	.	gene_id "lnc-bbug09857"	transcript_id "lnc-bbut00022771"	exon_number "1"
18	lncRNA	exon	48439726	48439793	.	+	.	gene_id "lnc-bbug09857"	transcript_id "lnc-bbut00022771"	exon_number "2"
18	lncRNA	exon	48440439	48441248	.	+	.	gene_id "lnc-bbug09857"	transcript_id "lnc-bbut00022771"	exon_number "3"
18	lncRNA	exon	48439001	48439281	.	+	.	gene_id "lnc-bbug09857"	transcript_id "lnc-bbut00022770"	exon_number "1"
18	lncRNA	exon	48439726	48439793	.	+	.	gene_id "lnc-bbug09857"	transcript_id "lnc-bbut00022770"	exon_number "2"
18	lncRNA	exon	48440439	48441248	.	+	.	gene_id "lnc-bbug09857"	transcript_id "lnc-bbut00022770"	exon_number "3"
18	lncRNA	exon	48439001	48439227	.	+	.	gene_id "lnc-bbug09857"	transcript_id "lnc-bbut00022769"	exon_number "1"
18	lncRNA	exon	48439726	48439793	.	+	.	gene_id "lnc-bbug09857"	transcript_id "lnc-bbut00022769"	exon_number "2"
18	lncRNA	exon	48440439	48441248	.	+	.	gene_id "lnc-bbug09857"	transcript_id "lnc-bbut00022769"	exon_number "3"
18	lncRNA	exon	48796161	48796263	.	+	.	gene_id "lnc-bbug09864"	transcript_id "lnc-bbut00022779"	exon_number "1"
18	lncRNA	exon	48797459	48797496	.	+	.	gene_id "lnc-bbug09864"	transcript_id "lnc-bbut00022779"	exon_number "2"
18	lncRNA	exon	48810055	48810132	.	+	.	gene_id "lnc-bbug09864"	transcript_id "lnc-bbut00022779"	exon_number "3"
18	lncRNA	exon	48810800	48811130	.	+	.	gene_id "lnc-bbug09864"	transcript_id "lnc-bbut00022779"	exon_number "4"
18	lncRNA	exon	48811241	48812840	.	+	.	gene_id "lnc-bbug09864"	transcript_id "lnc-bbut00022779"	exon_number "5"
18	lncRNA	exon	49036561	49037241	.	+	.	gene_id "lnc-bbug09874"	transcript_id "lnc-bbut00022798"	exon_number "1"
18	lncRNA	exon	49237987	49238106	.	+	.	gene_id "lnc-bbug09877"	transcript_id "lnc-bbut00022801"	exon_number "1"
18	lncRNA	exon	49239505	49240172	.	+	.	gene_id "lnc-bbug09877"	transcript_id "lnc-bbut00022801"	exon_number "2"
18	lncRNA	exon	49539592	49541534	.	+	.	gene_id "lnc-bbug09882"	transcript_id "lnc-bbut00022813"	exon_number "1"
18	lncRNA	exon	49541620	49541727	.	+	.	gene_id "lnc-bbug09882"	transcript_id "lnc-bbut00022813"	exon_number "2"
18	lncRNA	exon	49812016	49814526	.	+	.	gene_id "lnc-bbug09888"	transcript_id "lnc-bbut00022825"	exon_number "1"

18	lncRNA	exon	49814635	49814768	.	+	.	gene_id "lnc-bbug09888" transcript_id "lnc-bbut00022825"	exon_number "2"
18	lncRNA	exon	49815241	49815351	.	+	.	gene_id "lnc-bbug09888" transcript_id "lnc-bbut00022825"	exon_number "3"
18	lncRNA	exon	49815670	49815761	.	+	.	gene_id "lnc-bbug09888" transcript_id "lnc-bbut00022825"	exon_number "4"
18	lncRNA	exon	49857787	49857791	.	+	.	gene_id "lnc-bbug09892" transcript_id "lnc-bbut00022833"	exon_number "1"
18	lncRNA	exon	49859007	49859103	.	+	.	gene_id "lnc-bbug09892" transcript_id "lnc-bbut00022833"	exon_number "2"
18	lncRNA	exon	49875855	49877497	.	+	.	gene_id "lnc-bbug09892" transcript_id "lnc-bbut00022833"	exon_number "3"
18	lncRNA	exon	49977096	49978794	.	+	.	gene_id "lnc-bbug09893" transcript_id "lnc-bbut00022834"	exon_number "1"
18	lncRNA	exon	49984774	49984831	.	+	.	gene_id "lnc-bbug09893" transcript_id "lnc-bbut00022834"	exon_number "2"
18	lncRNA	exon	49985977	49986107	.	+	.	gene_id "lnc-bbug09893" transcript_id "lnc-bbut00022834"	exon_number "3"
18	lncRNA	exon	49988901	49989081	.	+	.	gene_id "lnc-bbug09893" transcript_id "lnc-bbut00022834"	exon_number "4"
18	lncRNA	exon	49991481	49991945	.	+	.	gene_id "lnc-bbug09893" transcript_id "lnc-bbut00022834"	exon_number "5"
18	lncRNA	exon	50335128	50336486	.	+	.	gene_id "lnc-bbug09899" transcript_id "lnc-bbut00022847"	exon_number "1"
18	lncRNA	exon	50337692	50337915	.	+	.	gene_id "lnc-bbug09899" transcript_id "lnc-bbut00022847"	exon_number "2"
18	lncRNA	exon	50339969	50340119	.	+	.	gene_id "lnc-bbug09899" transcript_id "lnc-bbut00022847"	exon_number "3"
18	lncRNA	exon	50340650	50340938	.	+	.	gene_id "lnc-bbug09899" transcript_id "lnc-bbut00022847"	exon_number "4"
18	lncRNA	exon	50487191	50487634	.	+	.	gene_id "lnc-bbug09903" transcript_id "lnc-bbut00022852"	exon_number "1"
18	lncRNA	exon	50487775	50487885	.	+	.	gene_id "lnc-bbug09903" transcript_id "lnc-bbut00022852"	exon_number "2"
18	lncRNA	exon	50489479	50490849	.	+	.	gene_id "lnc-bbug09903" transcript_id "lnc-bbut00022852"	exon_number "3"
18	lncRNA	exon	50487191	50487634	.	+	.	gene_id "lnc-bbug09903" transcript_id "lnc-bbut00022853"	exon_number "1"
18	lncRNA	exon	50487775	50487885	.	+	.	gene_id "lnc-bbug09903" transcript_id "lnc-bbut00022853"	exon_number "2"
18	lncRNA	exon	50569974	50570465	.	+	.	gene_id "lnc-bbug09903" transcript_id "lnc-bbut00022853"	exon_number "3"
18	lncRNA	exon	50595464	50596334	.	+	.	gene_id "lnc-bbug09904" transcript_id "lnc-bbut00022854"	exon_number "1"
18	lncRNA	exon	50609441	50609775	.	+	.	gene_id "lnc-bbug09904" transcript_id "lnc-bbut00022854"	exon_number "2"
18	lncRNA	exon	50611250	50612171	.	+	.	gene_id "lnc-bbug09904" transcript_id "lnc-bbut00022854"	exon_number "3"
18	lncRNA	exon	50640869	50640960	.	+	.	gene_id "lnc-bbug09906" transcript_id "lnc-bbut00022856"	exon_number "1"
18	lncRNA	exon	50643110	50650933	.	+	.	gene_id "lnc-bbug09906" transcript_id "lnc-bbut00022856"	exon_number "2"
18	lncRNA	exon	50640871	50640960	.	+	.	gene_id "lnc-bbug09906" transcript_id "lnc-bbut00022857"	exon_number "1"
18	lncRNA	exon	50643107	50650933	.	+	.	gene_id "lnc-bbug09906" transcript_id "lnc-bbut00022857"	exon_number "2"
18	lncRNA	exon	50640874	50640960	.	+	.	gene_id "lnc-bbug09906" transcript_id "lnc-bbut00022858"	exon_number "1"
18	lncRNA	exon	50643107	50643202	.	+	.	gene_id "lnc-bbug09906" transcript_id "lnc-bbut00022858"	exon_number "2"
18	lncRNA	exon	50646514	50650933	.	+	.	gene_id "lnc-bbug09906" transcript_id "lnc-bbut00022858"	exon_number "3"
18	lncRNA	exon	50640876	50640960	.	+	.	gene_id "lnc-bbug09906" transcript_id "lnc-bbut00022859"	exon_number "1"
18	lncRNA	exon	50643110	50643202	.	+	.	gene_id "lnc-bbug09906" transcript_id "lnc-bbut00022859"	exon_number "2"
18	lncRNA	exon	50645686	50650933	.	+	.	gene_id "lnc-bbug09906" transcript_id "lnc-bbut00022859"	exon_number "3"
18	lncRNA	exon	50640877	50640960	.	+	.	gene_id "lnc-bbug09906" transcript_id "lnc-bbut00022860"	exon_number "1"
18	lncRNA	exon	50643107	50643202	.	+	.	gene_id "lnc-bbug09906" transcript_id "lnc-bbut00022860"	exon_number "2"
18	lncRNA	exon	50645686	50650933	.	+	.	gene_id "lnc-bbug09906" transcript_id "lnc-bbut00022860"	exon_number "3"
18	lncRNA	exon	50659460	50659639	.	+	.	gene_id "lnc-bbug09907" transcript_id "lnc-bbut00022861"	exon_number "1"
18	lncRNA	exon	50661124	50661166	.	+	.	gene_id "lnc-bbug09907" transcript_id "lnc-bbut00022861"	exon_number "2"
18	lncRNA	exon	50662536	50664473	.	+	.	gene_id "lnc-bbug09907" transcript_id "lnc-bbut00022861"	exon_number "3"
18	lncRNA	exon	50686109	50690681	.	+	.	gene_id "lnc-bbug09909" transcript_id "lnc-bbut00022866"	exon_number "1"
18	lncRNA	exon	50692140	50692182	.	+	.	gene_id "lnc-bbug09909" transcript_id "lnc-bbut00022866"	exon_number "2"
18	lncRNA	exon	50693567	50695517	.	+	.	gene_id "lnc-bbug09909" transcript_id "lnc-bbut00022866"	exon_number "3"
18	lncRNA	exon	50686109	50690681	.	+	.	gene_id "lnc-bbug09909" transcript_id "lnc-bbut00022868"	exon_number "1"
18	lncRNA	exon	50692140	50692182	.	+	.	gene_id "lnc-bbug09909" transcript_id "lnc-bbut00022868"	exon_number "2"
18	lncRNA	exon	50692826	50692887	.	+	.	gene_id "lnc-bbug09909" transcript_id "lnc-bbut00022868"	exon_number "3"
18	lncRNA	exon	50693567	50695517	.	+	.	gene_id "lnc-bbug09909" transcript_id "lnc-bbut00022868"	exon_number "4"
18	lncRNA	exon	50686109	50690681	.	+	.	gene_id "lnc-bbug09909" transcript_id "lnc-bbut00022870"	exon_number "1"
18	lncRNA	exon	50692140	50692182	.	+	.	gene_id "lnc-bbug09909" transcript_id "lnc-bbut00022870"	exon_number "2"
18	lncRNA	exon	50692936	50692971	.	+	.	gene_id "lnc-bbug09909" transcript_id "lnc-bbut00022870"	exon_number "3"
18	lncRNA	exon	50693567	50695517	.	+	.	gene_id "lnc-bbug09909" transcript_id "lnc-bbut00022870"	exon_number "4"
18	lncRNA	exon	50686109	50690681	.	+	.	gene_id "lnc-bbug09909" transcript_id "lnc-bbut00022871"	exon_number "1"

18	lncRNA	exon	50692140	50692182	.	+	.	gene_id "lnc-bbug09909"	transcript_id "lnc-bbut00022871"	exon_number "2"
18	lncRNA	exon	50692936	50692971	.	+	.	gene_id "lnc-bbug09909"	transcript_id "lnc-bbut00022871"	exon_number "3"
18	lncRNA	exon	50693838	50695517	.	+	.	gene_id "lnc-bbug09909"	transcript_id "lnc-bbut00022871"	exon_number "4"
18	lncRNA	exon	50686109	50690681	.	+	.	gene_id "lnc-bbug09909"	transcript_id "lnc-bbut00022869"	exon_number "1"
18	lncRNA	exon	50692140	50692182	.	+	.	gene_id "lnc-bbug09909"	transcript_id "lnc-bbut00022869"	exon_number "2"
18	lncRNA	exon	50692826	50692971	.	+	.	gene_id "lnc-bbug09909"	transcript_id "lnc-bbut00022869"	exon_number "3"
18	lncRNA	exon	50693567	50695517	.	+	.	gene_id "lnc-bbug09909"	transcript_id "lnc-bbut00022869"	exon_number "4"
18	lncRNA	exon	50686109	50689888	.	+	.	gene_id "lnc-bbug09909"	transcript_id "lnc-bbut00022867"	exon_number "1"
18	lncRNA	exon	50690460	50690681	.	+	.	gene_id "lnc-bbug09909"	transcript_id "lnc-bbut00022867"	exon_number "2"
18	lncRNA	exon	50692140	50692182	.	+	.	gene_id "lnc-bbug09909"	transcript_id "lnc-bbut00022867"	exon_number "3"
18	lncRNA	exon	50692826	50695517	.	+	.	gene_id "lnc-bbug09909"	transcript_id "lnc-bbut00022867"	exon_number "4"
18	lncRNA	exon	50686109	50690681	.	+	.	gene_id "lnc-bbug09909"	transcript_id "lnc-bbut00022865"	exon_number "1"
18	lncRNA	exon	50692140	50692182	.	+	.	gene_id "lnc-bbug09909"	transcript_id "lnc-bbut00022865"	exon_number "2"
18	lncRNA	exon	50692936	50695517	.	+	.	gene_id "lnc-bbug09909"	transcript_id "lnc-bbut00022865"	exon_number "3"
18	lncRNA	exon	50686109	50690681	.	+	.	gene_id "lnc-bbug09909"	transcript_id "lnc-bbut00022864"	exon_number "1"
18	lncRNA	exon	50692140	50692182	.	+	.	gene_id "lnc-bbug09909"	transcript_id "lnc-bbut00022864"	exon_number "2"
18	lncRNA	exon	50692826	50695517	.	+	.	gene_id "lnc-bbug09909"	transcript_id "lnc-bbut00022864"	exon_number "3"
18	lncRNA	exon	50686110	50689888	.	+	.	gene_id "lnc-bbug09909"	transcript_id "lnc-bbut00022876"	exon_number "1"
18	lncRNA	exon	50692140	50692182	.	+	.	gene_id "lnc-bbug09909"	transcript_id "lnc-bbut00022876"	exon_number "2"
18	lncRNA	exon	50692826	50695517	.	+	.	gene_id "lnc-bbug09909"	transcript_id "lnc-bbut00022876"	exon_number "3"
18	lncRNA	exon	50686110	50689888	.	+	.	gene_id "lnc-bbug09909"	transcript_id "lnc-bbut00022875"	exon_number "1"
18	lncRNA	exon	50692140	50692182	.	+	.	gene_id "lnc-bbug09909"	transcript_id "lnc-bbut00022875"	exon_number "2"
18	lncRNA	exon	50692936	50692971	.	+	.	gene_id "lnc-bbug09909"	transcript_id "lnc-bbut00022875"	exon_number "3"
18	lncRNA	exon	50693838	50695517	.	+	.	gene_id "lnc-bbug09909"	transcript_id "lnc-bbut00022875"	exon_number "4"
18	lncRNA	exon	50686110	50689888	.	+	.	gene_id "lnc-bbug09909"	transcript_id "lnc-bbut00022874"	exon_number "1"
18	lncRNA	exon	50692140	50692182	.	+	.	gene_id "lnc-bbug09909"	transcript_id "lnc-bbut00022874"	exon_number "2"
18	lncRNA	exon	50692936	50692971	.	+	.	gene_id "lnc-bbug09909"	transcript_id "lnc-bbut00022874"	exon_number "3"
18	lncRNA	exon	50693567	50695517	.	+	.	gene_id "lnc-bbug09909"	transcript_id "lnc-bbut00022874"	exon_number "4"
18	lncRNA	exon	50686110	50689888	.	+	.	gene_id "lnc-bbug09909"	transcript_id "lnc-bbut00022873"	exon_number "1"
18	lncRNA	exon	50690499	50690681	.	+	.	gene_id "lnc-bbug09909"	transcript_id "lnc-bbut00022873"	exon_number "2"
18	lncRNA	exon	50692140	50692182	.	+	.	gene_id "lnc-bbug09909"	transcript_id "lnc-bbut00022873"	exon_number "3"
18	lncRNA	exon	50692826	50695517	.	+	.	gene_id "lnc-bbug09909"	transcript_id "lnc-bbut00022873"	exon_number "4"
18	lncRNA	exon	50686110	50689888	.	+	.	gene_id "lnc-bbug09909"	transcript_id "lnc-bbut00022872"	exon_number "1"
18	lncRNA	exon	50692140	50692182	.	+	.	gene_id "lnc-bbug09909"	transcript_id "lnc-bbut00022872"	exon_number "2"
18	lncRNA	exon	50692936	50695517	.	+	.	gene_id "lnc-bbug09909"	transcript_id "lnc-bbut00022872"	exon_number "3"
18	lncRNA	exon	50702361	50702518	.	+	.	gene_id "lnc-bbug09910"	transcript_id "lnc-bbut00022877"	exon_number "1"
18	lncRNA	exon	50705582	50705821	.	+	.	gene_id "lnc-bbug09910"	transcript_id "lnc-bbut00022877"	exon_number "2"
18	lncRNA	exon	50787072	50787201	.	+	.	gene_id "lnc-bbug09912"	transcript_id "lnc-bbut00022886"	exon_number "1"
18	lncRNA	exon	50797887	50797955	.	+	.	gene_id "lnc-bbug09912"	transcript_id "lnc-bbut00022886"	exon_number "2"
18	lncRNA	exon	50799066	50799397	.	+	.	gene_id "lnc-bbug09912"	transcript_id "lnc-bbut00022886"	exon_number "3"
18	lncRNA	exon	50980924	50981201	.	+	.	gene_id "lnc-bbug09919"	transcript_id "lnc-bbut00022902"	exon_number "1"
18	lncRNA	exon	50981254	50983035	.	+	.	gene_id "lnc-bbug09919"	transcript_id "lnc-bbut00022902"	exon_number "2"
18	lncRNA	exon	51092189	51092323	.	+	.	gene_id "lnc-bbug09921"	transcript_id "lnc-bbut00022920"	exon_number "1"
18	lncRNA	exon	51093994	51094319	.	+	.	gene_id "lnc-bbug09921"	transcript_id "lnc-bbut00022920"	exon_number "2"
18	lncRNA	exon	51474613	51474866	.	+	.	gene_id "lnc-bbug09927"	transcript_id "lnc-bbut00022933"	exon_number "1"
18	lncRNA	exon	51478153	51478305	.	+	.	gene_id "lnc-bbug09927"	transcript_id "lnc-bbut00022933"	exon_number "2"
18	lncRNA	exon	51481195	51481307	.	+	.	gene_id "lnc-bbug09927"	transcript_id "lnc-bbut00022933"	exon_number "3"
18	lncRNA	exon	51495483	51495949	.	+	.	gene_id "lnc-bbug09928"	transcript_id "lnc-bbut00022934"	exon_number "1"
18	lncRNA	exon	51504867	51504915	.	+	.	gene_id "lnc-bbug09928"	transcript_id "lnc-bbut00022934"	exon_number "2"
18	lncRNA	exon	51506512	51507119	.	+	.	gene_id "lnc-bbug09928"	transcript_id "lnc-bbut00022934"	exon_number "3"
18	lncRNA	exon	51740338	51741387	.	+	.	gene_id "lnc-bbug09936"	transcript_id "lnc-bbut00022945"	exon_number "1"
18	lncRNA	exon	51748421	51748537	.	+	.	gene_id "lnc-bbug09936"	transcript_id "lnc-bbut00022945"	exon_number "2"

18	lncRNA	exon	51749898	51750050	.	+	.	gene_id "lnc-bbug09936"	transcript_id "lnc-bbut00022945"	exon_number "3"
18	lncRNA	exon	51750439	51750582	.	+	.	gene_id "lnc-bbug09936"	transcript_id "lnc-bbut00022945"	exon_number "4"
18	lncRNA	exon	51750777	51751021	.	+	.	gene_id "lnc-bbug09936"	transcript_id "lnc-bbut00022945"	exon_number "5"
18	lncRNA	exon	51831204	51832147	.	+	.	gene_id "lnc-bbug09940"	transcript_id "lnc-bbut00022950"	exon_number "1"
18	lncRNA	exon	51833011	51833603	.	+	.	gene_id "lnc-bbug09940"	transcript_id "lnc-bbut00022950"	exon_number "2"
18	lncRNA	exon	51831204	51832147	.	+	.	gene_id "lnc-bbug09940"	transcript_id "lnc-bbut00022952"	exon_number "1"
18	lncRNA	exon	51835741	51835912	.	+	.	gene_id "lnc-bbug09940"	transcript_id "lnc-bbut00022952"	exon_number "2"
18	lncRNA	exon	51836569	51836648	.	+	.	gene_id "lnc-bbug09940"	transcript_id "lnc-bbut00022952"	exon_number "3"
18	lncRNA	exon	51837219	51837310	.	+	.	gene_id "lnc-bbug09940"	transcript_id "lnc-bbut00022952"	exon_number "4"
18	lncRNA	exon	51839843	51840073	.	+	.	gene_id "lnc-bbug09940"	transcript_id "lnc-bbut00022952"	exon_number "5"
18	lncRNA	exon	51840178	51840367	.	+	.	gene_id "lnc-bbug09940"	transcript_id "lnc-bbut00022952"	exon_number "6"
18	lncRNA	exon	51831204	51832147	.	+	.	gene_id "lnc-bbug09940"	transcript_id "lnc-bbut00022951"	exon_number "1"
18	lncRNA	exon	51835741	51835912	.	+	.	gene_id "lnc-bbug09940"	transcript_id "lnc-bbut00022951"	exon_number "2"
18	lncRNA	exon	51836569	51836648	.	+	.	gene_id "lnc-bbug09940"	transcript_id "lnc-bbut00022951"	exon_number "3"
18	lncRNA	exon	51837219	51837310	.	+	.	gene_id "lnc-bbug09940"	transcript_id "lnc-bbut00022951"	exon_number "4"
18	lncRNA	exon	51838260	51838377	.	+	.	gene_id "lnc-bbug09940"	transcript_id "lnc-bbut00022951"	exon_number "5"
18	lncRNA	exon	51839843	51840367	.	+	.	gene_id "lnc-bbug09940"	transcript_id "lnc-bbut00022951"	exon_number "6"
18	lncRNA	exon	51831205	51832147	.	+	.	gene_id "lnc-bbug09940"	transcript_id "lnc-bbut00022953"	exon_number "1"
18	lncRNA	exon	51835741	51835912	.	+	.	gene_id "lnc-bbug09940"	transcript_id "lnc-bbut00022953"	exon_number "2"
18	lncRNA	exon	51837219	51837310	.	+	.	gene_id "lnc-bbug09940"	transcript_id "lnc-bbut00022953"	exon_number "3"
18	lncRNA	exon	51838260	51838377	.	+	.	gene_id "lnc-bbug09940"	transcript_id "lnc-bbut00022953"	exon_number "4"
18	lncRNA	exon	51839843	51840367	.	+	.	gene_id "lnc-bbug09940"	transcript_id "lnc-bbut00022953"	exon_number "5"
18	lncRNA	exon	51831212	51832147	.	+	.	gene_id "lnc-bbug09940"	transcript_id "lnc-bbut00022954"	exon_number "1"
18	lncRNA	exon	51835741	51835912	.	+	.	gene_id "lnc-bbug09940"	transcript_id "lnc-bbut00022954"	exon_number "2"
18	lncRNA	exon	51839843	51840367	.	+	.	gene_id "lnc-bbug09940"	transcript_id "lnc-bbut00022954"	exon_number "3"
18	lncRNA	exon	51831214	51832147	.	+	.	gene_id "lnc-bbug09940"	transcript_id "lnc-bbut00022955"	exon_number "1"
18	lncRNA	exon	51833548	51833656	.	+	.	gene_id "lnc-bbug09940"	transcript_id "lnc-bbut00022955"	exon_number "2"
18	lncRNA	exon	51833759	51834484	.	+	.	gene_id "lnc-bbug09940"	transcript_id "lnc-bbut00022955"	exon_number "3"
18	lncRNA	exon	51849197	51849308	.	+	.	gene_id "lnc-bbug09942"	transcript_id "lnc-bbut00022962"	exon_number "1"
18	lncRNA	exon	51851627	51851786	.	+	.	gene_id "lnc-bbug09942"	transcript_id "lnc-bbut00022962"	exon_number "2"
18	lncRNA	exon	51853618	51854217	.	+	.	gene_id "lnc-bbug09942"	transcript_id "lnc-bbut00022962"	exon_number "3"
18	lncRNA	exon	52098543	52098853	.	+	.	gene_id "lnc-bbug09947"	transcript_id "lnc-bbut00022976"	exon_number "1"
18	lncRNA	exon	52100049	52100557	.	+	.	gene_id "lnc-bbug09947"	transcript_id "lnc-bbut00022976"	exon_number "2"
18	lncRNA	exon	52600705	52611398	.	+	.	gene_id "lnc-bbug09958"	transcript_id "lnc-bbut00023011"	exon_number "1"
18	lncRNA	exon	52671914	52673190	.	+	.	gene_id "lnc-bbug09962"	transcript_id "lnc-bbut00023018"	exon_number "1"
18	lncRNA	exon	52673371	52673530	.	+	.	gene_id "lnc-bbug09962"	transcript_id "lnc-bbut00023018"	exon_number "2"
18	lncRNA	exon	52673834	52674082	.	+	.	gene_id "lnc-bbug09962"	transcript_id "lnc-bbut00023018"	exon_number "3"
18	lncRNA	exon	52773061	52773312	.	+	.	gene_id "lnc-bbug09966"	transcript_id "lnc-bbut00023036"	exon_number "1"
18	lncRNA	exon	52774151	52774287	.	+	.	gene_id "lnc-bbug09966"	transcript_id "lnc-bbut00023036"	exon_number "2"
18	lncRNA	exon	52775842	52775930	.	+	.	gene_id "lnc-bbug09966"	transcript_id "lnc-bbut00023036"	exon_number "3"
18	lncRNA	exon	52779590	52780396	.	+	.	gene_id "lnc-bbug09966"	transcript_id "lnc-bbut00023036"	exon_number "4"
18	lncRNA	exon	52896795	52897478	.	+	.	gene_id "lnc-bbug09970"	transcript_id "lnc-bbut00023043"	exon_number "1"
18	lncRNA	exon	53325425	53325559	.	+	.	gene_id "lnc-bbug09980"	transcript_id "lnc-bbut00023063"	exon_number "1"
18	lncRNA	exon	53325767	53325856	.	+	.	gene_id "lnc-bbug09980"	transcript_id "lnc-bbut00023063"	exon_number "2"
18	lncRNA	exon	53326570	53326874	.	+	.	gene_id "lnc-bbug09980"	transcript_id "lnc-bbut00023063"	exon_number "3"
18	lncRNA	exon	53326983	53327037	.	+	.	gene_id "lnc-bbug09980"	transcript_id "lnc-bbut00023063"	exon_number "4"
18	lncRNA	exon	53328066	53328655	.	+	.	gene_id "lnc-bbug09980"	transcript_id "lnc-bbut00023063"	exon_number "5"
18	lncRNA	exon	53325425	53325559	.	+	.	gene_id "lnc-bbug09980"	transcript_id "lnc-bbut00023064"	exon_number "1"
18	lncRNA	exon	53325767	53325856	.	+	.	gene_id "lnc-bbug09980"	transcript_id "lnc-bbut00023064"	exon_number "2"
18	lncRNA	exon	53326570	53326874	.	+	.	gene_id "lnc-bbug09980"	transcript_id "lnc-bbut00023064"	exon_number "3"
18	lncRNA	exon	53329506	53329818	.	+	.	gene_id "lnc-bbug09980"	transcript_id "lnc-bbut00023064"	exon_number "4"
18	lncRNA	exon	53673049	53673685	.	+	.	gene_id "lnc-bbug09986"	transcript_id "lnc-bbut00023081"	exon_number "1"

18	lncRNA	exon	53747877	53748098	.	+	.	gene_id "lnc-bbug09990"	transcript_id "lnc-bbut00023085"	exon_number "1"
18	lncRNA	exon	53749602	53750189	.	+	.	gene_id "lnc-bbug09990"	transcript_id "lnc-bbut00023085"	exon_number "2"
18	lncRNA	exon	54153531	54153879	.	+	.	gene_id "lnc-bbug09995"	transcript_id "lnc-bbut00023094"	exon_number "1"
18	lncRNA	exon	54160282	54160587	.	+	.	gene_id "lnc-bbug09995"	transcript_id "lnc-bbut00023094"	exon_number "2"
18	lncRNA	exon	54281808	54282047	.	+	.	gene_id "lnc-bbug09998"	transcript_id "lnc-bbut00023098"	exon_number "1"
18	lncRNA	exon	54282160	54282209	.	+	.	gene_id "lnc-bbug09998"	transcript_id "lnc-bbut00023098"	exon_number "2"
18	lncRNA	exon	54282775	54284556	.	+	.	gene_id "lnc-bbug09998"	transcript_id "lnc-bbut00023098"	exon_number "3"
18	lncRNA	exon	54282227	54282363	.	+	.	gene_id "lnc-bbug09998"	transcript_id "lnc-bbut00023099"	exon_number "1"
18	lncRNA	exon	54282775	54284556	.	+	.	gene_id "lnc-bbug09998"	transcript_id "lnc-bbut00023099"	exon_number "2"
18	lncRNA	exon	54673933	54674088	.	+	.	gene_id "lnc-bbug10004"	transcript_id "lnc-bbut00023114"	exon_number "1"
18	lncRNA	exon	54675871	54675895	.	+	.	gene_id "lnc-bbug10004"	transcript_id "lnc-bbut00023114"	exon_number "2"
18	lncRNA	exon	54676015	54676068	.	+	.	gene_id "lnc-bbug10004"	transcript_id "lnc-bbut00023114"	exon_number "3"
18	lncRNA	exon	54676242	54676316	.	+	.	gene_id "lnc-bbug10004"	transcript_id "lnc-bbut00023114"	exon_number "4"
18	lncRNA	exon	54676419	54676517	.	+	.	gene_id "lnc-bbug10004"	transcript_id "lnc-bbut00023114"	exon_number "5"
18	lncRNA	exon	54677669	54678046	.	+	.	gene_id "lnc-bbug10004"	transcript_id "lnc-bbut00023114"	exon_number "6"
18	lncRNA	exon	55160990	55161125	.	+	.	gene_id "lnc-bbug10019"	transcript_id "lnc-bbut00023146"	exon_number "1"
18	lncRNA	exon	55169924	55169992	.	+	.	gene_id "lnc-bbug10019"	transcript_id "lnc-bbut00023146"	exon_number "2"
18	lncRNA	exon	55171457	55171941	.	+	.	gene_id "lnc-bbug10019"	transcript_id "lnc-bbut00023146"	exon_number "3"
18	lncRNA	exon	55428964	55429089	.	+	.	gene_id "lnc-bbug10026"	transcript_id "lnc-bbut00023167"	exon_number "1"
18	lncRNA	exon	55429791	55429907	.	+	.	gene_id "lnc-bbug10026"	transcript_id "lnc-bbut00023167"	exon_number "2"
18	lncRNA	exon	55431604	55431690	.	+	.	gene_id "lnc-bbug10026"	transcript_id "lnc-bbut00023167"	exon_number "3"
18	lncRNA	exon	55432077	55432497	.	+	.	gene_id "lnc-bbug10026"	transcript_id "lnc-bbut00023167"	exon_number "4"
18	lncRNA	exon	55432813	55434249	.	+	.	gene_id "lnc-bbug10026"	transcript_id "lnc-bbut00023167"	exon_number "5"
18	lncRNA	exon	55428965	55429089	.	+	.	gene_id "lnc-bbug10026"	transcript_id "lnc-bbut00023168"	exon_number "1"
18	lncRNA	exon	55429791	55429907	.	+	.	gene_id "lnc-bbug10026"	transcript_id "lnc-bbut00023168"	exon_number "2"
18	lncRNA	exon	55431604	55431690	.	+	.	gene_id "lnc-bbug10026"	transcript_id "lnc-bbut00023168"	exon_number "3"
18	lncRNA	exon	55432077	55434249	.	+	.	gene_id "lnc-bbug10026"	transcript_id "lnc-bbut00023168"	exon_number "4"
18	lncRNA	exon	56125975	56126070	.	+	.	gene_id "lnc-bbug10055"	transcript_id "lnc-bbut00023250"	exon_number "1"
18	lncRNA	exon	56127491	56127550	.	+	.	gene_id "lnc-bbug10055"	transcript_id "lnc-bbut00023250"	exon_number "2"
18	lncRNA	exon	56129415	56131015	.	+	.	gene_id "lnc-bbug10055"	transcript_id "lnc-bbut00023250"	exon_number "3"
18	lncRNA	exon	56171111	56171423	.	+	.	gene_id "lnc-bbug10057"	transcript_id "lnc-bbut00023253"	exon_number "1"
18	lncRNA	exon	56171567	56171791	.	+	.	gene_id "lnc-bbug10057"	transcript_id "lnc-bbut00023253"	exon_number "2"
18	lncRNA	exon	56517352	56517559	.	+	.	gene_id "lnc-bbug10065"	transcript_id "lnc-bbut00023265"	exon_number "1"
18	lncRNA	exon	56520962	56521632	.	+	.	gene_id "lnc-bbug10065"	transcript_id "lnc-bbut00023265"	exon_number "2"
18	lncRNA	exon	57467511	57467645	.	+	.	gene_id "lnc-bbug10090"	transcript_id "lnc-bbut00023303"	exon_number "1"
18	lncRNA	exon	57467781	57467995	.	+	.	gene_id "lnc-bbug10090"	transcript_id "lnc-bbut00023303"	exon_number "2"
18	lncRNA	exon	57473636	57473792	.	+	.	gene_id "lnc-bbug10090"	transcript_id "lnc-bbut00023303"	exon_number "3"
18	lncRNA	exon	57482902	57483253	.	+	.	gene_id "lnc-bbug10090"	transcript_id "lnc-bbut00023303"	exon_number "4"
18	lncRNA	exon	57489017	57489489	.	+	.	gene_id "lnc-bbug10091"	transcript_id "lnc-bbut00023305"	exon_number "1"
18	lncRNA	exon	57492464	57492967	.	+	.	gene_id "lnc-bbug10091"	transcript_id "lnc-bbut00023305"	exon_number "2"
18	lncRNA	exon	57493083	57493853	.	+	.	gene_id "lnc-bbug10091"	transcript_id "lnc-bbut00023305"	exon_number "3"
18	lncRNA	exon	57489017	57489489	.	+	.	gene_id "lnc-bbug10091"	transcript_id "lnc-bbut00023304"	exon_number "1"
18	lncRNA	exon	57492464	57492940	.	+	.	gene_id "lnc-bbug10091"	transcript_id "lnc-bbut00023304"	exon_number "2"
18	lncRNA	exon	57493083	57493853	.	+	.	gene_id "lnc-bbug10091"	transcript_id "lnc-bbut00023304"	exon_number "3"
18	lncRNA	exon	57533235	57533347	.	+	.	gene_id "lnc-bbug10092"	transcript_id "lnc-bbut00023307"	exon_number "1"
18	lncRNA	exon	57534172	57534414	.	+	.	gene_id "lnc-bbug10092"	transcript_id "lnc-bbut00023307"	exon_number "2"
18	lncRNA	exon	57535761	57536280	.	+	.	gene_id "lnc-bbug10092"	transcript_id "lnc-bbut00023307"	exon_number "3"
18	lncRNA	exon	57536664	57538073	.	+	.	gene_id "lnc-bbug10092"	transcript_id "lnc-bbut00023307"	exon_number "4"
18	lncRNA	exon	57533235	57533347	.	+	.	gene_id "lnc-bbug10092"	transcript_id "lnc-bbut00023306"	exon_number "1"
18	lncRNA	exon	57535761	57536280	.	+	.	gene_id "lnc-bbug10092"	transcript_id "lnc-bbut00023306"	exon_number "2"
18	lncRNA	exon	57536664	57538073	.	+	.	gene_id "lnc-bbug10092"	transcript_id "lnc-bbut00023306"	exon_number "3"
18	lncRNA	exon	57535535	57535673	.	+	.	gene_id "lnc-bbug10092"	transcript_id "lnc-bbut00023308"	exon_number "1"

18	lncRNA	exon	57535761	57536280	.	+	.	gene_id "lnc-bbug10092"	transcript_id "lnc-bbut00023308"	exon_number "2"
18	lncRNA	exon	57536664	57538073	.	+	.	gene_id "lnc-bbug10092"	transcript_id "lnc-bbut00023308"	exon_number "3"
18	lncRNA	exon	58183896	58184817	.	+	.	gene_id "lnc-bbug10106"	transcript_id "lnc-bbut00023324"	exon_number "1"
18	lncRNA	exon	59965680	59965718	.	+	.	gene_id "lnc-bbug10123"	transcript_id "lnc-bbut00023376"	exon_number "1"
18	lncRNA	exon	59967597	59967660	.	+	.	gene_id "lnc-bbug10123"	transcript_id "lnc-bbut00023376"	exon_number "2"
18	lncRNA	exon	59971965	59972091	.	+	.	gene_id "lnc-bbug10123"	transcript_id "lnc-bbut00023376"	exon_number "3"
18	lncRNA	exon	59978053	59978163	.	+	.	gene_id "lnc-bbug10123"	transcript_id "lnc-bbut00023376"	exon_number "4"
18	lncRNA	exon	60758256	60761913	.	+	.	gene_id "lnc-bbug10129"	transcript_id "lnc-bbut00023386"	exon_number "1"
18	lncRNA	exon	60762015	60763812	.	+	.	gene_id "lnc-bbug10129"	transcript_id "lnc-bbut00023386"	exon_number "2"
18	lncRNA	exon	60758492	60759746	.	+	.	gene_id "lnc-bbug10129"	transcript_id "lnc-bbut00023387"	exon_number "1"
18	lncRNA	exon	60762015	60763812	.	+	.	gene_id "lnc-bbug10129"	transcript_id "lnc-bbut00023387"	exon_number "2"
18	lncRNA	exon	61006467	61006573	.	+	.	gene_id "lnc-bbug10139"	transcript_id "lnc-bbut00023432"	exon_number "1"
18	lncRNA	exon	61007136	61007331	.	+	.	gene_id "lnc-bbug10139"	transcript_id "lnc-bbut00023432"	exon_number "2"
18	lncRNA	exon	61007530	61007692	.	+	.	gene_id "lnc-bbug10139"	transcript_id "lnc-bbut00023432"	exon_number "3"
18	lncRNA	exon	61007961	61008069	.	+	.	gene_id "lnc-bbug10139"	transcript_id "lnc-bbut00023432"	exon_number "4"
18	lncRNA	exon	61008232	61009792	.	+	.	gene_id "lnc-bbug10139"	transcript_id "lnc-bbut00023432"	exon_number "5"
18	lncRNA	exon	61683242	61683262	.	+	.	gene_id "lnc-bbug10164"	transcript_id "lnc-bbut00023476"	exon_number "1"
18	lncRNA	exon	61685166	61685727	.	+	.	gene_id "lnc-bbug10164"	transcript_id "lnc-bbut00023476"	exon_number "2"
18	lncRNA	exon	61797888	61798122	.	+	.	gene_id "lnc-bbug10165"	transcript_id "lnc-bbut00023477"	exon_number "1"
18	lncRNA	exon	61800439	61801703	.	+	.	gene_id "lnc-bbug10165"	transcript_id "lnc-bbut00023477"	exon_number "2"
18	lncRNA	exon	62072697	62072766	.	+	.	gene_id "lnc-bbug10175"	transcript_id "lnc-bbut00023498"	exon_number "1"
18	lncRNA	exon	62073113	62073486	.	+	.	gene_id "lnc-bbug10175"	transcript_id "lnc-bbut00023498"	exon_number "2"
18	lncRNA	exon	62448280	62448378	.	+	.	gene_id "lnc-bbug10191"	transcript_id "lnc-bbut00023530"	exon_number "1"
18	lncRNA	exon	62448786	62448908	.	+	.	gene_id "lnc-bbug10191"	transcript_id "lnc-bbut00023530"	exon_number "2"
18	lncRNA	exon	62451215	62451326	.	+	.	gene_id "lnc-bbug10191"	transcript_id "lnc-bbut00023530"	exon_number "3"
18	lncRNA	exon	62770641	62770700	.	+	.	gene_id "lnc-bbug10193"	transcript_id "lnc-bbut00023532"	exon_number "1"
18	lncRNA	exon	62772046	62772162	.	+	.	gene_id "lnc-bbug10193"	transcript_id "lnc-bbut00023532"	exon_number "2"
18	lncRNA	exon	62774699	62774742	.	+	.	gene_id "lnc-bbug10193"	transcript_id "lnc-bbut00023532"	exon_number "3"
18	lncRNA	exon	62827667	62827930	.	+	.	gene_id "lnc-bbug10194"	transcript_id "lnc-bbut00023533"	exon_number "1"
18	lncRNA	exon	62829968	62830132	.	+	.	gene_id "lnc-bbug10194"	transcript_id "lnc-bbut00023533"	exon_number "2"
18	lncRNA	exon	62844031	62844600	.	+	.	gene_id "lnc-bbug10194"	transcript_id "lnc-bbut00023533"	exon_number "3"
18	lncRNA	exon	62925410	62926769	.	+	.	gene_id "lnc-bbug10199"	transcript_id "lnc-bbut00023546"	exon_number "1"
18	lncRNA	exon	62926924	62927040	.	+	.	gene_id "lnc-bbug10199"	transcript_id "lnc-bbut00023546"	exon_number "2"
18	lncRNA	exon	62927476	62929659	.	+	.	gene_id "lnc-bbug10199"	transcript_id "lnc-bbut00023546"	exon_number "3"
18	lncRNA	exon	62939802	62940087	.	+	.	gene_id "lnc-bbug10200"	transcript_id "lnc-bbut00023547"	exon_number "1"
18	lncRNA	exon	62940489	62942729	.	+	.	gene_id "lnc-bbug10200"	transcript_id "lnc-bbut00023547"	exon_number "2"
18	lncRNA	exon	62942949	62944950	.	+	.	gene_id "lnc-bbug10200"	transcript_id "lnc-bbut00023547"	exon_number "3"
18	lncRNA	exon	63163120	63163247	.	+	.	gene_id "lnc-bbug10208"	transcript_id "lnc-bbut00023560"	exon_number "1"
18	lncRNA	exon	63164941	63165164	.	+	.	gene_id "lnc-bbug10208"	transcript_id "lnc-bbut00023560"	exon_number "2"
18	lncRNA	exon	63166430	63166659	.	+	.	gene_id "lnc-bbug10208"	transcript_id "lnc-bbut00023560"	exon_number "3"
18	lncRNA	exon	63167163	63168469	.	+	.	gene_id "lnc-bbug10208"	transcript_id "lnc-bbut00023560"	exon_number "4"
18	lncRNA	exon	63164324	63164492	.	+	.	gene_id "lnc-bbug10208"	transcript_id "lnc-bbut00023561"	exon_number "1"
18	lncRNA	exon	63164941	63165164	.	+	.	gene_id "lnc-bbug10208"	transcript_id "lnc-bbut00023561"	exon_number "2"
18	lncRNA	exon	63166430	63166659	.	+	.	gene_id "lnc-bbug10208"	transcript_id "lnc-bbut00023561"	exon_number "3"
18	lncRNA	exon	63167163	63168469	.	+	.	gene_id "lnc-bbug10208"	transcript_id "lnc-bbut00023561"	exon_number "4"
18	lncRNA	exon	63164590	63165164	.	+	.	gene_id "lnc-bbug10208"	transcript_id "lnc-bbut00023562"	exon_number "1"
18	lncRNA	exon	63166430	63166659	.	+	.	gene_id "lnc-bbug10208"	transcript_id "lnc-bbut00023562"	exon_number "2"
18	lncRNA	exon	63167163	63168469	.	+	.	gene_id "lnc-bbug10208"	transcript_id "lnc-bbut00023562"	exon_number "3"
18	lncRNA	exon	63215918	63216147	.	+	.	gene_id "lnc-bbug10212"	transcript_id "lnc-bbut00023573"	exon_number "1"
18	lncRNA	exon	63216249	63216991	.	+	.	gene_id "lnc-bbug10212"	transcript_id "lnc-bbut00023573"	exon_number "2"
18	lncRNA	exon	63225157	63227751	.	+	.	gene_id "lnc-bbug10213"	transcript_id "lnc-bbut00023574"	exon_number "1"
18	lncRNA	exon	63375885	63376224	.	+	.	gene_id "lnc-bbug10221"	transcript_id "lnc-bbut00023582"	exon_number "1"

18	lncRNA	exon	63376586	63376718	.	+	.	gene_id "lnc-bbug10221"	transcript_id "lnc-bbut00023582"	exon_number "2"
18	lncRNA	exon	63377740	63378131	.	+	.	gene_id "lnc-bbug10221"	transcript_id "lnc-bbut00023582"	exon_number "3"
18	lncRNA	exon	63386377	63386492	.	+	.	gene_id "lnc-bbug10222"	transcript_id "lnc-bbut00023583"	exon_number "1"
18	lncRNA	exon	63387044	63387165	.	+	.	gene_id "lnc-bbug10222"	transcript_id "lnc-bbut00023583"	exon_number "2"
18	lncRNA	exon	63387484	63387538	.	+	.	gene_id "lnc-bbug10222"	transcript_id "lnc-bbut00023583"	exon_number "3"
18	lncRNA	exon	63491792	63492911	.	+	.	gene_id "lnc-bbug10225"	transcript_id "lnc-bbut00023587"	exon_number "1"
18	lncRNA	exon	63532326	63532956	.	+	.	gene_id "lnc-bbug10233"	transcript_id "lnc-bbut00023595"	exon_number "1"
18	lncRNA	exon	63670557	63671327	.	+	.	gene_id "lnc-bbug10239"	transcript_id "lnc-bbut00023601"	exon_number "1"
18	lncRNA	exon	63685861	63686334	.	+	.	gene_id "lnc-bbug10241"	transcript_id "lnc-bbut00023603"	exon_number "1"
18	lncRNA	exon	64456975	64459522	.	+	.	gene_id "lnc-bbug10254"	transcript_id "lnc-bbut00023627"	exon_number "1"
18	lncRNA	exon	64460347	64460387	.	+	.	gene_id "lnc-bbug10254"	transcript_id "lnc-bbut00023627"	exon_number "2"
18	lncRNA	exon	64650313	64651519	.	+	.	gene_id "lnc-bbug10258"	transcript_id "lnc-bbut00023648"	exon_number "1"
18	lncRNA	exon	64652101	64654861	.	+	.	gene_id "lnc-bbug10258"	transcript_id "lnc-bbut00023648"	exon_number "2"
18	lncRNA	exon	65287584	65289225	.	+	.	gene_id "lnc-bbug10272"	transcript_id "lnc-bbut00023663"	exon_number "1"
18	lncRNA	exon	65753978	65754304	.	+	.	gene_id "lnc-bbug10282"	transcript_id "lnc-bbut00023679"	exon_number "1"
18	lncRNA	exon	65754950	65755024	.	+	.	gene_id "lnc-bbug10282"	transcript_id "lnc-bbut00023679"	exon_number "2"
18	lncRNA	exon	65800361	65800675	.	+	.	gene_id "lnc-bbug10286"	transcript_id "lnc-bbut00023686"	exon_number "1"
18	lncRNA	exon	65804262	65804460	.	+	.	gene_id "lnc-bbug10286"	transcript_id "lnc-bbut00023686"	exon_number "2"
18	lncRNA	exon	65804763	65805055	.	+	.	gene_id "lnc-bbug10286"	transcript_id "lnc-bbut00023686"	exon_number "3"
18	lncRNA	exon	65807901	65809182	.	+	.	gene_id "lnc-bbug10286"	transcript_id "lnc-bbut00023686"	exon_number "4"
18	lncRNA	exon	65800361	65800675	.	+	.	gene_id "lnc-bbug10286"	transcript_id "lnc-bbut00023685"	exon_number "1"
18	lncRNA	exon	65804262	65804460	.	+	.	gene_id "lnc-bbug10286"	transcript_id "lnc-bbut00023685"	exon_number "2"
18	lncRNA	exon	65804760	65805055	.	+	.	gene_id "lnc-bbug10286"	transcript_id "lnc-bbut00023685"	exon_number "3"
18	lncRNA	exon	65807901	65809182	.	+	.	gene_id "lnc-bbug10286"	transcript_id "lnc-bbut00023685"	exon_number "4"
18	lncRNA	exon	65800361	65800675	.	+	.	gene_id "lnc-bbug10286"	transcript_id "lnc-bbut00023684"	exon_number "1"
18	lncRNA	exon	65804760	65805055	.	+	.	gene_id "lnc-bbug10286"	transcript_id "lnc-bbut00023684"	exon_number "2"
18	lncRNA	exon	65807901	65809182	.	+	.	gene_id "lnc-bbug10286"	transcript_id "lnc-bbut00023684"	exon_number "3"
18	lncRNA	exon	65844121	65844210	.	+	.	gene_id "lnc-bbug10289"	transcript_id "lnc-bbut00023698"	exon_number "1"
18	lncRNA	exon	65844679	65845141	.	+	.	gene_id "lnc-bbug10289"	transcript_id "lnc-bbut00023698"	exon_number "2"
18	lncRNA	exon	65864705	65865425	.	+	.	gene_id "lnc-bbug10290"	transcript_id "lnc-bbut00023699"	exon_number "1"
18	lncRNA	exon	65865625	65865913	.	+	.	gene_id "lnc-bbug10290"	transcript_id "lnc-bbut00023699"	exon_number "2"
18	lncRNA	exon	65881733	65882719	.	+	.	gene_id "lnc-bbug10292"	transcript_id "lnc-bbut00023702"	exon_number "1"
18	lncRNA	exon	65890211	65890370	.	+	.	gene_id "lnc-bbug10292"	transcript_id "lnc-bbut00023702"	exon_number "2"
18	lncRNA	exon	65890631	65890859	.	+	.	gene_id "lnc-bbug10292"	transcript_id "lnc-bbut00023702"	exon_number "3"
18	lncRNA	exon	65881733	65882719	.	+	.	gene_id "lnc-bbug10292"	transcript_id "lnc-bbut00023701"	exon_number "1"
18	lncRNA	exon	65889267	65890370	.	+	.	gene_id "lnc-bbug10292"	transcript_id "lnc-bbut00023701"	exon_number "2"
18	lncRNA	exon	65890631	65890859	.	+	.	gene_id "lnc-bbug10292"	transcript_id "lnc-bbut00023701"	exon_number "3"
18	lncRNA	exon	65895847	65898569	.	+	.	gene_id "lnc-bbug10293"	transcript_id "lnc-bbut00023703"	exon_number "1"
18	lncRNA	exon	1174502	1174705	.	-	.	gene_id "lnc-bbug10300"	transcript_id "lnc-bbut00023722"	exon_number "1"
18	lncRNA	exon	1175632	1175710	.	-	.	gene_id "lnc-bbug10300"	transcript_id "lnc-bbut00023722"	exon_number "2"
18	lncRNA	exon	1180155	1180489	.	-	.	gene_id "lnc-bbug10300"	transcript_id "lnc-bbut00023722"	exon_number "3"
18	lncRNA	exon	1180646	1180887	.	-	.	gene_id "lnc-bbug10300"	transcript_id "lnc-bbut00023722"	exon_number "4"
18	lncRNA	exon	1233273	1235718	.	-	.	gene_id "lnc-bbug10302"	transcript_id "lnc-bbut00023729"	exon_number "1"
18	lncRNA	exon	1706322	1706520	.	-	.	gene_id "lnc-bbug10311"	transcript_id "lnc-bbut00023752"	exon_number "1"
18	lncRNA	exon	1710864	1712408	.	-	.	gene_id "lnc-bbug10311"	transcript_id "lnc-bbut00023752"	exon_number "2"
18	lncRNA	exon	2389682	2390084	.	-	.	gene_id "lnc-bbug10320"	transcript_id "lnc-bbut00023771"	exon_number "1"
18	lncRNA	exon	2504355	2507444	.	-	.	gene_id "lnc-bbug10323"	transcript_id "lnc-bbut00023778"	exon_number "1"
18	lncRNA	exon	2510008	2510109	.	-	.	gene_id "lnc-bbug10323"	transcript_id "lnc-bbut00023778"	exon_number "2"
18	lncRNA	exon	2517694	2518005	.	-	.	gene_id "lnc-bbug10323"	transcript_id "lnc-bbut00023778"	exon_number "3"
18	lncRNA	exon	2504355	2507444	.	-	.	gene_id "lnc-bbug10323"	transcript_id "lnc-bbut00023777"	exon_number "1"
18	lncRNA	exon	2509939	2510109	.	-	.	gene_id "lnc-bbug10323"	transcript_id "lnc-bbut00023777"	exon_number "2"
18	lncRNA	exon	2517694	2518005	.	-	.	gene_id "lnc-bbug10323"	transcript_id "lnc-bbut00023777"	exon_number "3"

18	lncRNA	exon	3125705	3127899	.	-	.	gene_id "lnc-bbug10329"	transcript_id "lnc-bbut00023798"	exon_number "1"
18	lncRNA	exon	3129011	3129091	.	-	.	gene_id "lnc-bbug10329"	transcript_id "lnc-bbut00023798"	exon_number "2"
18	lncRNA	exon	3136794	3137143	.	-	.	gene_id "lnc-bbug10329"	transcript_id "lnc-bbut00023798"	exon_number "3"
18	lncRNA	exon	3505541	3505879	.	-	.	gene_id "lnc-bbug10330"	transcript_id "lnc-bbut00023799"	exon_number "1"
18	lncRNA	exon	3517462	3517731	.	-	.	gene_id "lnc-bbug10331"	transcript_id "lnc-bbut00023800"	exon_number "1"
18	lncRNA	exon	3518890	3519159	.	-	.	gene_id "lnc-bbug10331"	transcript_id "lnc-bbut00023800"	exon_number "2"
18	lncRNA	exon	3519408	3519485	.	-	.	gene_id "lnc-bbug10331"	transcript_id "lnc-bbut00023800"	exon_number "3"
18	lncRNA	exon	3520424	3520537	.	-	.	gene_id "lnc-bbug10331"	transcript_id "lnc-bbut00023800"	exon_number "4"
18	lncRNA	exon	3522955	3523089	.	-	.	gene_id "lnc-bbug10331"	transcript_id "lnc-bbut00023800"	exon_number "5"
18	lncRNA	exon	3544793	3544868	.	-	.	gene_id "lnc-bbug10331"	transcript_id "lnc-bbut00023800"	exon_number "6"
18	lncRNA	exon	3545378	3545490	.	-	.	gene_id "lnc-bbug10331"	transcript_id "lnc-bbut00023800"	exon_number "7"
18	lncRNA	exon	3546230	3550638	.	-	.	gene_id "lnc-bbug10331"	transcript_id "lnc-bbut00023800"	exon_number "8"
18	lncRNA	exon	3613444	3613629	.	-	.	gene_id "lnc-bbug10331"	transcript_id "lnc-bbut00023800"	exon_number "9"
18	lncRNA	exon	3810860	3811124	.	-	.	gene_id "lnc-bbug10331"	transcript_id "lnc-bbut00023800"	exon_number "10"
18	lncRNA	exon	3518625	3519159	.	-	.	gene_id "lnc-bbug10331"	transcript_id "lnc-bbut00023801"	exon_number "1"
18	lncRNA	exon	3519408	3519485	.	-	.	gene_id "lnc-bbug10331"	transcript_id "lnc-bbut00023801"	exon_number "2"
18	lncRNA	exon	3520424	3520537	.	-	.	gene_id "lnc-bbug10331"	transcript_id "lnc-bbut00023801"	exon_number "3"
18	lncRNA	exon	3522955	3523089	.	-	.	gene_id "lnc-bbug10331"	transcript_id "lnc-bbut00023801"	exon_number "4"
18	lncRNA	exon	3544793	3544868	.	-	.	gene_id "lnc-bbug10331"	transcript_id "lnc-bbut00023801"	exon_number "5"
18	lncRNA	exon	3545378	3545490	.	-	.	gene_id "lnc-bbug10331"	transcript_id "lnc-bbut00023801"	exon_number "6"
18	lncRNA	exon	3546230	3546320	.	-	.	gene_id "lnc-bbug10331"	transcript_id "lnc-bbut00023801"	exon_number "7"
18	lncRNA	exon	3550565	3550638	.	-	.	gene_id "lnc-bbug10331"	transcript_id "lnc-bbut00023801"	exon_number "8"
18	lncRNA	exon	3613444	3613629	.	-	.	gene_id "lnc-bbug10331"	transcript_id "lnc-bbut00023801"	exon_number "9"
18	lncRNA	exon	3810860	3811117	.	-	.	gene_id "lnc-bbug10331"	transcript_id "lnc-bbut00023801"	exon_number "10"
18	lncRNA	exon	3518625	3519159	.	-	.	gene_id "lnc-bbug10331"	transcript_id "lnc-bbut00023802"	exon_number "1"
18	lncRNA	exon	3519408	3519485	.	-	.	gene_id "lnc-bbug10331"	transcript_id "lnc-bbut00023802"	exon_number "2"
18	lncRNA	exon	3520424	3520537	.	-	.	gene_id "lnc-bbug10331"	transcript_id "lnc-bbut00023802"	exon_number "3"
18	lncRNA	exon	3522955	3523089	.	-	.	gene_id "lnc-bbug10331"	transcript_id "lnc-bbut00023802"	exon_number "4"
18	lncRNA	exon	3544793	3544868	.	-	.	gene_id "lnc-bbug10331"	transcript_id "lnc-bbut00023802"	exon_number "5"
18	lncRNA	exon	3545378	3545490	.	-	.	gene_id "lnc-bbug10331"	transcript_id "lnc-bbut00023802"	exon_number "6"
18	lncRNA	exon	3546230	3548542	.	-	.	gene_id "lnc-bbug10331"	transcript_id "lnc-bbut00023802"	exon_number "7"
18	lncRNA	exon	3550565	3550638	.	-	.	gene_id "lnc-bbug10331"	transcript_id "lnc-bbut00023802"	exon_number "8"
18	lncRNA	exon	3613444	3613629	.	-	.	gene_id "lnc-bbug10331"	transcript_id "lnc-bbut00023802"	exon_number "9"
18	lncRNA	exon	3810860	3811123	.	-	.	gene_id "lnc-bbug10331"	transcript_id "lnc-bbut00023802"	exon_number "10"
18	lncRNA	exon	3518625	3519159	.	-	.	gene_id "lnc-bbug10331"	transcript_id "lnc-bbut00023808"	exon_number "1"
18	lncRNA	exon	3519408	3519485	.	-	.	gene_id "lnc-bbug10331"	transcript_id "lnc-bbut00023808"	exon_number "2"
18	lncRNA	exon	3520424	3520537	.	-	.	gene_id "lnc-bbug10331"	transcript_id "lnc-bbut00023808"	exon_number "3"
18	lncRNA	exon	3522955	3523089	.	-	.	gene_id "lnc-bbug10331"	transcript_id "lnc-bbut00023808"	exon_number "4"
18	lncRNA	exon	3544793	3544868	.	-	.	gene_id "lnc-bbug10331"	transcript_id "lnc-bbut00023808"	exon_number "5"
18	lncRNA	exon	3545378	3545486	.	-	.	gene_id "lnc-bbug10331"	transcript_id "lnc-bbut00023808"	exon_number "6"
18	lncRNA	exon	3546230	3546320	.	-	.	gene_id "lnc-bbug10331"	transcript_id "lnc-bbut00023808"	exon_number "7"
18	lncRNA	exon	3546814	3550638	.	-	.	gene_id "lnc-bbug10331"	transcript_id "lnc-bbut00023808"	exon_number "8"
18	lncRNA	exon	3613444	3613629	.	-	.	gene_id "lnc-bbug10331"	transcript_id "lnc-bbut00023808"	exon_number "9"
18	lncRNA	exon	3810860	3811125	.	-	.	gene_id "lnc-bbug10331"	transcript_id "lnc-bbut00023808"	exon_number "10"
18	lncRNA	exon	3518625	3519159	.	-	.	gene_id "lnc-bbug10331"	transcript_id "lnc-bbut00023810"	exon_number "1"
18	lncRNA	exon	3519408	3519485	.	-	.	gene_id "lnc-bbug10331"	transcript_id "lnc-bbut00023810"	exon_number "2"
18	lncRNA	exon	3520424	3520537	.	-	.	gene_id "lnc-bbug10331"	transcript_id "lnc-bbut00023810"	exon_number "3"
18	lncRNA	exon	3522955	3523089	.	-	.	gene_id "lnc-bbug10331"	transcript_id "lnc-bbut00023810"	exon_number "4"
18	lncRNA	exon	3544793	3544868	.	-	.	gene_id "lnc-bbug10331"	transcript_id "lnc-bbut00023810"	exon_number "5"
18	lncRNA	exon	3545378	3545490	.	-	.	gene_id "lnc-bbug10331"	transcript_id "lnc-bbut00023810"	exon_number "6"
18	lncRNA	exon	3546230	3546320	.	-	.	gene_id "lnc-bbug10331"	transcript_id "lnc-bbut00023810"	exon_number "7"
18	lncRNA	exon	3548400	3550638	.	-	.	gene_id "lnc-bbug10331"	transcript_id "lnc-bbut00023810"	exon_number "8"

18	lncRNA	exon	3613444	3613629	.	-	.	gene_id "lnc-bbug10331"	transcript_id "lnc-bbut00023810"	exon_number "9"
18	lncRNA	exon	3810860	3811125	.	-	.	gene_id "lnc-bbug10331"	transcript_id "lnc-bbut00023810"	exon_number "10"
18	lncRNA	exon	3518625	3519159	.	-	.	gene_id "lnc-bbug10331"	transcript_id "lnc-bbut00023809"	exon_number "1"
18	lncRNA	exon	3519408	3519485	.	-	.	gene_id "lnc-bbug10331"	transcript_id "lnc-bbut00023809"	exon_number "2"
18	lncRNA	exon	3520424	3520537	.	-	.	gene_id "lnc-bbug10331"	transcript_id "lnc-bbut00023809"	exon_number "3"
18	lncRNA	exon	3522955	3523089	.	-	.	gene_id "lnc-bbug10331"	transcript_id "lnc-bbut00023809"	exon_number "4"
18	lncRNA	exon	3544793	3544868	.	-	.	gene_id "lnc-bbug10331"	transcript_id "lnc-bbut00023809"	exon_number "5"
18	lncRNA	exon	3545378	3545490	.	-	.	gene_id "lnc-bbug10331"	transcript_id "lnc-bbut00023809"	exon_number "6"
18	lncRNA	exon	3546230	3546320	.	-	.	gene_id "lnc-bbug10331"	transcript_id "lnc-bbut00023809"	exon_number "7"
18	lncRNA	exon	3546814	3550638	.	-	.	gene_id "lnc-bbug10331"	transcript_id "lnc-bbut00023809"	exon_number "8"
18	lncRNA	exon	3613444	3613629	.	-	.	gene_id "lnc-bbug10331"	transcript_id "lnc-bbut00023809"	exon_number "9"
18	lncRNA	exon	3810860	3811125	.	-	.	gene_id "lnc-bbug10331"	transcript_id "lnc-bbut00023809"	exon_number "10"
18	lncRNA	exon	3518625	3519159	.	-	.	gene_id "lnc-bbug10331"	transcript_id "lnc-bbut00023807"	exon_number "1"
18	lncRNA	exon	3519408	3519485	.	-	.	gene_id "lnc-bbug10331"	transcript_id "lnc-bbut00023807"	exon_number "2"
18	lncRNA	exon	3520424	3520537	.	-	.	gene_id "lnc-bbug10331"	transcript_id "lnc-bbut00023807"	exon_number "3"
18	lncRNA	exon	3522955	3523089	.	-	.	gene_id "lnc-bbug10331"	transcript_id "lnc-bbut00023807"	exon_number "4"
18	lncRNA	exon	3544793	3544868	.	-	.	gene_id "lnc-bbug10331"	transcript_id "lnc-bbut00023807"	exon_number "5"
18	lncRNA	exon	3545378	3545490	.	-	.	gene_id "lnc-bbug10331"	transcript_id "lnc-bbut00023807"	exon_number "6"
18	lncRNA	exon	3546230	3550638	.	-	.	gene_id "lnc-bbug10331"	transcript_id "lnc-bbut00023807"	exon_number "7"
18	lncRNA	exon	3613444	3613629	.	-	.	gene_id "lnc-bbug10331"	transcript_id "lnc-bbut00023807"	exon_number "8"
18	lncRNA	exon	3810860	3811125	.	-	.	gene_id "lnc-bbug10331"	transcript_id "lnc-bbut00023807"	exon_number "9"
18	lncRNA	exon	3518625	3519159	.	-	.	gene_id "lnc-bbug10331"	transcript_id "lnc-bbut00023805"	exon_number "1"
18	lncRNA	exon	3519408	3519485	.	-	.	gene_id "lnc-bbug10331"	transcript_id "lnc-bbut00023805"	exon_number "2"
18	lncRNA	exon	3520424	3520537	.	-	.	gene_id "lnc-bbug10331"	transcript_id "lnc-bbut00023805"	exon_number "3"
18	lncRNA	exon	3522955	3523089	.	-	.	gene_id "lnc-bbug10331"	transcript_id "lnc-bbut00023805"	exon_number "4"
18	lncRNA	exon	3544793	3544868	.	-	.	gene_id "lnc-bbug10331"	transcript_id "lnc-bbut00023805"	exon_number "5"
18	lncRNA	exon	3546230	3550638	.	-	.	gene_id "lnc-bbug10331"	transcript_id "lnc-bbut00023805"	exon_number "6"
18	lncRNA	exon	3613444	3613629	.	-	.	gene_id "lnc-bbug10331"	transcript_id "lnc-bbut00023805"	exon_number "7"
18	lncRNA	exon	3810860	3811125	.	-	.	gene_id "lnc-bbug10331"	transcript_id "lnc-bbut00023805"	exon_number "8"
18	lncRNA	exon	3518625	3519159	.	-	.	gene_id "lnc-bbug10331"	transcript_id "lnc-bbut00023806"	exon_number "1"
18	lncRNA	exon	3519408	3519485	.	-	.	gene_id "lnc-bbug10331"	transcript_id "lnc-bbut00023806"	exon_number "2"
18	lncRNA	exon	3520424	3520537	.	-	.	gene_id "lnc-bbug10331"	transcript_id "lnc-bbut00023806"	exon_number "3"
18	lncRNA	exon	3522955	3523089	.	-	.	gene_id "lnc-bbug10331"	transcript_id "lnc-bbut00023806"	exon_number "4"
18	lncRNA	exon	3544793	3544868	.	-	.	gene_id "lnc-bbug10331"	transcript_id "lnc-bbut00023806"	exon_number "5"
18	lncRNA	exon	3545378	3545486	.	-	.	gene_id "lnc-bbug10331"	transcript_id "lnc-bbut00023806"	exon_number "6"
18	lncRNA	exon	3546230	3550638	.	-	.	gene_id "lnc-bbug10331"	transcript_id "lnc-bbut00023806"	exon_number "7"
18	lncRNA	exon	3613444	3613629	.	-	.	gene_id "lnc-bbug10331"	transcript_id "lnc-bbut00023806"	exon_number "8"
18	lncRNA	exon	3810860	3811125	.	-	.	gene_id "lnc-bbug10331"	transcript_id "lnc-bbut00023806"	exon_number "9"
18	lncRNA	exon	3518625	3519159	.	-	.	gene_id "lnc-bbug10331"	transcript_id "lnc-bbut00023804"	exon_number "1"
18	lncRNA	exon	3519408	3519485	.	-	.	gene_id "lnc-bbug10331"	transcript_id "lnc-bbut00023804"	exon_number "2"
18	lncRNA	exon	3520424	3520537	.	-	.	gene_id "lnc-bbug10331"	transcript_id "lnc-bbut00023804"	exon_number "3"
18	lncRNA	exon	3522955	3523089	.	-	.	gene_id "lnc-bbug10331"	transcript_id "lnc-bbut00023804"	exon_number "4"
18	lncRNA	exon	3544793	3544868	.	-	.	gene_id "lnc-bbug10331"	transcript_id "lnc-bbut00023804"	exon_number "5"
18	lncRNA	exon	3545378	3545490	.	-	.	gene_id "lnc-bbug10331"	transcript_id "lnc-bbut00023804"	exon_number "6"
18	lncRNA	exon	3546230	3546320	.	-	.	gene_id "lnc-bbug10331"	transcript_id "lnc-bbut00023804"	exon_number "7"
18	lncRNA	exon	3546814	3546872	.	-	.	gene_id "lnc-bbug10331"	transcript_id "lnc-bbut00023804"	exon_number "8"
18	lncRNA	exon	3548400	3550638	.	-	.	gene_id "lnc-bbug10331"	transcript_id "lnc-bbut00023804"	exon_number "9"
18	lncRNA	exon	3613444	3613629	.	-	.	gene_id "lnc-bbug10331"	transcript_id "lnc-bbut00023804"	exon_number "10"
18	lncRNA	exon	3810860	3811125	.	-	.	gene_id "lnc-bbug10331"	transcript_id "lnc-bbut00023804"	exon_number "11"
18	lncRNA	exon	3518625	3519159	.	-	.	gene_id "lnc-bbug10331"	transcript_id "lnc-bbut00023803"	exon_number "1"
18	lncRNA	exon	3519408	3519485	.	-	.	gene_id "lnc-bbug10331"	transcript_id "lnc-bbut00023803"	exon_number "2"
18	lncRNA	exon	3520424	3520537	.	-	.	gene_id "lnc-bbug10331"	transcript_id "lnc-bbut00023803"	exon_number "3"

18	lncRNA	exon	3522955	3523089	.	-	.	gene_id "lnc-bbug10331"	transcript_id "lnc-bbut00023803"	exon_number "4"
18	lncRNA	exon	3544793	3544868	.	-	.	gene_id "lnc-bbug10331"	transcript_id "lnc-bbut00023803"	exon_number "5"
18	lncRNA	exon	3545378	3545490	.	-	.	gene_id "lnc-bbug10331"	transcript_id "lnc-bbut00023803"	exon_number "6"
18	lncRNA	exon	3546230	3546872	.	-	.	gene_id "lnc-bbug10331"	transcript_id "lnc-bbut00023803"	exon_number "7"
18	lncRNA	exon	3548400	3550638	.	-	.	gene_id "lnc-bbug10331"	transcript_id "lnc-bbut00023803"	exon_number "8"
18	lncRNA	exon	3613444	3613629	.	-	.	gene_id "lnc-bbug10331"	transcript_id "lnc-bbut00023803"	exon_number "9"
18	lncRNA	exon	3810860	3811125	.	-	.	gene_id "lnc-bbug10331"	transcript_id "lnc-bbut00023803"	exon_number "10"
18	lncRNA	exon	3834956	3835153	.	-	.	gene_id "lnc-bbug10332"	transcript_id "lnc-bbut00023813"	exon_number "1"
18	lncRNA	exon	3850063	3850199	.	-	.	gene_id "lnc-bbug10332"	transcript_id "lnc-bbut00023813"	exon_number "2"
18	lncRNA	exon	3851481	3851540	.	-	.	gene_id "lnc-bbug10332"	transcript_id "lnc-bbut00023813"	exon_number "3"
18	lncRNA	exon	3854039	3854088	.	-	.	gene_id "lnc-bbug10332"	transcript_id "lnc-bbut00023813"	exon_number "4"
18	lncRNA	exon	3855159	3856118	.	-	.	gene_id "lnc-bbug10332"	transcript_id "lnc-bbut00023813"	exon_number "5"
18	lncRNA	exon	3834956	3835153	.	-	.	gene_id "lnc-bbug10332"	transcript_id "lnc-bbut00023812"	exon_number "1"
18	lncRNA	exon	3850063	3850199	.	-	.	gene_id "lnc-bbug10332"	transcript_id "lnc-bbut00023812"	exon_number "2"
18	lncRNA	exon	3851393	3851540	.	-	.	gene_id "lnc-bbug10332"	transcript_id "lnc-bbut00023812"	exon_number "3"
18	lncRNA	exon	3854039	3854088	.	-	.	gene_id "lnc-bbug10332"	transcript_id "lnc-bbut00023812"	exon_number "4"
18	lncRNA	exon	3855159	3856118	.	-	.	gene_id "lnc-bbug10332"	transcript_id "lnc-bbut00023812"	exon_number "5"
18	lncRNA	exon	3834956	3835153	.	-	.	gene_id "lnc-bbug10332"	transcript_id "lnc-bbut00023811"	exon_number "1"
18	lncRNA	exon	3850063	3850199	.	-	.	gene_id "lnc-bbug10332"	transcript_id "lnc-bbut00023811"	exon_number "2"
18	lncRNA	exon	3854039	3854088	.	-	.	gene_id "lnc-bbug10332"	transcript_id "lnc-bbut00023811"	exon_number "3"
18	lncRNA	exon	3855159	3856118	.	-	.	gene_id "lnc-bbug10332"	transcript_id "lnc-bbut00023811"	exon_number "4"
18	lncRNA	exon	4017897	4018040	.	-	.	gene_id "lnc-bbug10333"	transcript_id "lnc-bbut00023814"	exon_number "1"
18	lncRNA	exon	4022052	4022729	.	-	.	gene_id "lnc-bbug10333"	transcript_id "lnc-bbut00023814"	exon_number "2"
18	lncRNA	exon	4552982	4553369	.	-	.	gene_id "lnc-bbug10335"	transcript_id "lnc-bbut00023817"	exon_number "1"
18	lncRNA	exon	4572793	4575011	.	-	.	gene_id "lnc-bbug10335"	transcript_id "lnc-bbut00023817"	exon_number "2"
18	lncRNA	exon	4555854	4556659	.	-	.	gene_id "lnc-bbug10335"	transcript_id "lnc-bbut00023818"	exon_number "1"
18	lncRNA	exon	4560575	4561644	.	-	.	gene_id "lnc-bbug10335"	transcript_id "lnc-bbut00023818"	exon_number "2"
18	lncRNA	exon	4555854	4556659	.	-	.	gene_id "lnc-bbug10335"	transcript_id "lnc-bbut00023819"	exon_number "1"
18	lncRNA	exon	4565434	4565455	.	-	.	gene_id "lnc-bbug10335"	transcript_id "lnc-bbut00023819"	exon_number "2"
18	lncRNA	exon	4593816	4594205	.	-	.	gene_id "lnc-bbug10336"	transcript_id "lnc-bbut00023821"	exon_number "1"
18	lncRNA	exon	4596840	4597949	.	-	.	gene_id "lnc-bbug10336"	transcript_id "lnc-bbut00023821"	exon_number "2"
18	lncRNA	exon	5824022	5825501	.	-	.	gene_id "lnc-bbug10339"	transcript_id "lnc-bbut00023824"	exon_number "1"
18	lncRNA	exon	5825758	5829583	.	-	.	gene_id "lnc-bbug10339"	transcript_id "lnc-bbut00023824"	exon_number "2"
18	lncRNA	exon	5833850	5834026	.	-	.	gene_id "lnc-bbug10340"	transcript_id "lnc-bbut00023825"	exon_number "1"
18	lncRNA	exon	5952071	5952308	.	-	.	gene_id "lnc-bbug10340"	transcript_id "lnc-bbut00023825"	exon_number "2"
18	lncRNA	exon	6192431	6193923	.	-	.	gene_id "lnc-bbug10342"	transcript_id "lnc-bbut00023828"	exon_number "1"
18	lncRNA	exon	6201845	6202269	.	-	.	gene_id "lnc-bbug10342"	transcript_id "lnc-bbut00023828"	exon_number "2"
18	lncRNA	exon	6202446	6202615	.	-	.	gene_id "lnc-bbug10342"	transcript_id "lnc-bbut00023828"	exon_number "3"
18	lncRNA	exon	6319177	6319387	.	-	.	gene_id "lnc-bbug10343"	transcript_id "lnc-bbut00023829"	exon_number "1"
18	lncRNA	exon	6330980	6331103	.	-	.	gene_id "lnc-bbug10343"	transcript_id "lnc-bbut00023829"	exon_number "2"
18	lncRNA	exon	6333078	6333156	.	-	.	gene_id "lnc-bbug10343"	transcript_id "lnc-bbut00023829"	exon_number "3"
18	lncRNA	exon	6333819	6334143	.	-	.	gene_id "lnc-bbug10343"	transcript_id "lnc-bbut00023829"	exon_number "4"
18	lncRNA	exon	6421225	6421451	.	-	.	gene_id "lnc-bbug10344"	transcript_id "lnc-bbut00023831"	exon_number "1"
18	lncRNA	exon	6426710	6426820	.	-	.	gene_id "lnc-bbug10344"	transcript_id "lnc-bbut00023831"	exon_number "2"
18	lncRNA	exon	6569060	6569116	.	-	.	gene_id "lnc-bbug10344"	transcript_id "lnc-bbut00023831"	exon_number "3"
18	lncRNA	exon	6633339	6633426	.	-	.	gene_id "lnc-bbug10344"	transcript_id "lnc-bbut00023831"	exon_number "4"
18	lncRNA	exon	6716399	6716482	.	-	.	gene_id "lnc-bbug10344"	transcript_id "lnc-bbut00023831"	exon_number "5"
18	lncRNA	exon	6806284	6806465	.	-	.	gene_id "lnc-bbug10344"	transcript_id "lnc-bbut00023831"	exon_number "6"
18	lncRNA	exon	6905291	6905326	.	-	.	gene_id "lnc-bbug10344"	transcript_id "lnc-bbut00023831"	exon_number "7"
18	lncRNA	exon	6991530	6991694	.	-	.	gene_id "lnc-bbug10344"	transcript_id "lnc-bbut00023831"	exon_number "8"
18	lncRNA	exon	6421225	6421451	.	-	.	gene_id "lnc-bbug10344"	transcript_id "lnc-bbut00023830"	exon_number "1"
18	lncRNA	exon	6426710	6426820	.	-	.	gene_id "lnc-bbug10344"	transcript_id "lnc-bbut00023830"	exon_number "2"

18	lncRNA	exon	6569060	6569116	.	-	.	gene_id "lnc-bbug10344"	transcript_id "lnc-bbut00023830"	exon_number "3"
18	lncRNA	exon	6716399	6716482	.	-	.	gene_id "lnc-bbug10344"	transcript_id "lnc-bbut00023830"	exon_number "4"
18	lncRNA	exon	6806284	6806465	.	-	.	gene_id "lnc-bbug10344"	transcript_id "lnc-bbut00023830"	exon_number "5"
18	lncRNA	exon	6905291	6905326	.	-	.	gene_id "lnc-bbug10344"	transcript_id "lnc-bbut00023830"	exon_number "6"
18	lncRNA	exon	6991530	6991694	.	-	.	gene_id "lnc-bbug10344"	transcript_id "lnc-bbut00023830"	exon_number "7"
18	lncRNA	exon	7349285	7349665	.	-	.	gene_id "lnc-bbug10346"	transcript_id "lnc-bbut00023833"	exon_number "1"
18	lncRNA	exon	7352206	7352277	.	-	.	gene_id "lnc-bbug10346"	transcript_id "lnc-bbut00023833"	exon_number "2"
18	lncRNA	exon	7356387	7356499	.	-	.	gene_id "lnc-bbug10346"	transcript_id "lnc-bbut00023833"	exon_number "3"
18	lncRNA	exon	7363387	7363538	.	-	.	gene_id "lnc-bbug10346"	transcript_id "lnc-bbut00023833"	exon_number "4"
18	lncRNA	exon	7606649	7606758	.	-	.	gene_id "lnc-bbug10350"	transcript_id "lnc-bbut00023840"	exon_number "1"
18	lncRNA	exon	7608379	7608774	.	-	.	gene_id "lnc-bbug10350"	transcript_id "lnc-bbut00023840"	exon_number "2"
18	lncRNA	exon	7859800	7859907	.	-	.	gene_id "lnc-bbug10351"	transcript_id "lnc-bbut00023841"	exon_number "1"
18	lncRNA	exon	7862302	7862488	.	-	.	gene_id "lnc-bbug10351"	transcript_id "lnc-bbut00023841"	exon_number "2"
18	lncRNA	exon	7862669	7862863	.	-	.	gene_id "lnc-bbug10351"	transcript_id "lnc-bbut00023841"	exon_number "3"
18	lncRNA	exon	8064104	8064641	.	-	.	gene_id "lnc-bbug10352"	transcript_id "lnc-bbut00023842"	exon_number "1"
18	lncRNA	exon	8065267	8067941	.	-	.	gene_id "lnc-bbug10352"	transcript_id "lnc-bbut00023842"	exon_number "2"
18	lncRNA	exon	8237882	8238433	.	-	.	gene_id "lnc-bbug10354"	transcript_id "lnc-bbut00023848"	exon_number "1"
18	lncRNA	exon	8241116	8242478	.	-	.	gene_id "lnc-bbug10354"	transcript_id "lnc-bbut00023848"	exon_number "2"
18	lncRNA	exon	8237882	8238430	.	-	.	gene_id "lnc-bbug10354"	transcript_id "lnc-bbut00023847"	exon_number "1"
18	lncRNA	exon	8241116	8242478	.	-	.	gene_id "lnc-bbug10354"	transcript_id "lnc-bbut00023847"	exon_number "2"
18	lncRNA	exon	8237882	8238433	.	-	.	gene_id "lnc-bbug10354"	transcript_id "lnc-bbut00023849"	exon_number "1"
18	lncRNA	exon	8241116	8242046	.	-	.	gene_id "lnc-bbug10354"	transcript_id "lnc-bbut00023849"	exon_number "2"
18	lncRNA	exon	8242485	8242662	.	-	.	gene_id "lnc-bbug10354"	transcript_id "lnc-bbut00023849"	exon_number "3"
18	lncRNA	exon	8254731	8255910	.	-	.	gene_id "lnc-bbug10354"	transcript_id "lnc-bbut00023849"	exon_number "4"
18	lncRNA	exon	8244729	8246196	.	-	.	gene_id "lnc-bbug10355"	transcript_id "lnc-bbut00023850"	exon_number "1"
18	lncRNA	exon	8247249	8247697	.	-	.	gene_id "lnc-bbug10355"	transcript_id "lnc-bbut00023850"	exon_number "2"
18	lncRNA	exon	8344389	8344533	.	-	.	gene_id "lnc-bbug10356"	transcript_id "lnc-bbut00023851"	exon_number "1"
18	lncRNA	exon	8349951	8351418	.	-	.	gene_id "lnc-bbug10356"	transcript_id "lnc-bbut00023851"	exon_number "2"
18	lncRNA	exon	8361434	8364806	.	-	.	gene_id "lnc-bbug10357"	transcript_id "lnc-bbut00023853"	exon_number "1"
18	lncRNA	exon	8366418	8366614	.	-	.	gene_id "lnc-bbug10357"	transcript_id "lnc-bbut00023853"	exon_number "2"
18	lncRNA	exon	8361434	8364806	.	-	.	gene_id "lnc-bbug10357"	transcript_id "lnc-bbut00023852"	exon_number "1"
18	lncRNA	exon	8366376	8366614	.	-	.	gene_id "lnc-bbug10357"	transcript_id "lnc-bbut00023852"	exon_number "2"
18	lncRNA	exon	8527102	8527951	.	-	.	gene_id "lnc-bbug10359"	transcript_id "lnc-bbut00023855"	exon_number "1"
18	lncRNA	exon	9844843	9845042	.	-	.	gene_id "lnc-bbug10360"	transcript_id "lnc-bbut00023856"	exon_number "1"
18	lncRNA	exon	9845340	9851947	.	-	.	gene_id "lnc-bbug10360"	transcript_id "lnc-bbut00023856"	exon_number "2"
18	lncRNA	exon	9855552	9856607	.	-	.	gene_id "lnc-bbug10360"	transcript_id "lnc-bbut00023856"	exon_number "3"
18	lncRNA	exon	9876947	9878393	.	-	.	gene_id "lnc-bbug10361"	transcript_id "lnc-bbut00023857"	exon_number "1"
18	lncRNA	exon	9878799	9878938	.	-	.	gene_id "lnc-bbug10361"	transcript_id "lnc-bbut00023857"	exon_number "2"
18	lncRNA	exon	9879158	9879314	.	-	.	gene_id "lnc-bbug10361"	transcript_id "lnc-bbut00023857"	exon_number "3"
18	lncRNA	exon	9879621	9879756	.	-	.	gene_id "lnc-bbug10361"	transcript_id "lnc-bbut00023857"	exon_number "4"
18	lncRNA	exon	9876947	9878393	.	-	.	gene_id "lnc-bbug10361"	transcript_id "lnc-bbut00023858"	exon_number "1"
18	lncRNA	exon	9878799	9878938	.	-	.	gene_id "lnc-bbug10361"	transcript_id "lnc-bbut00023858"	exon_number "2"
18	lncRNA	exon	9879158	9879314	.	-	.	gene_id "lnc-bbug10361"	transcript_id "lnc-bbut00023858"	exon_number "3"
18	lncRNA	exon	9880505	9880794	.	-	.	gene_id "lnc-bbug10361"	transcript_id "lnc-bbut00023858"	exon_number "4"
18	lncRNA	exon	10280574	10280991	.	-	.	gene_id "lnc-bbug10367"	transcript_id "lnc-bbut00023868"	exon_number "1"
18	lncRNA	exon	10283976	10287639	.	-	.	gene_id "lnc-bbug10367"	transcript_id "lnc-bbut00023868"	exon_number "2"
18	lncRNA	exon	10310918	10311144	.	-	.	gene_id "lnc-bbug10368"	transcript_id "lnc-bbut00023869"	exon_number "1"
18	lncRNA	exon	10311604	10312846	.	-	.	gene_id "lnc-bbug10368"	transcript_id "lnc-bbut00023869"	exon_number "2"
18	lncRNA	exon	10313192	10313330	.	-	.	gene_id "lnc-bbug10368"	transcript_id "lnc-bbut00023869"	exon_number "3"
18	lncRNA	exon	10391284	10402748	.	-	.	gene_id "lnc-bbug10370"	transcript_id "lnc-bbut00023875"	exon_number "1"
18	lncRNA	exon	10402821	10404577	.	-	.	gene_id "lnc-bbug10370"	transcript_id "lnc-bbut00023875"	exon_number "2"
18	lncRNA	exon	10450221	10450538	.	-	.	gene_id "lnc-bbug10372"	transcript_id "lnc-bbut00023877"	exon_number "1"

18	lncRNA	exon	10451341	10451386	.	-	.	gene_id "lnc-bbug10372"	transcript_id "lnc-bbut00023877"	exon_number "2"
18	lncRNA	exon	10468441	10468571	.	-	.	gene_id "lnc-bbug10372"	transcript_id "lnc-bbut00023877"	exon_number "3"
18	lncRNA	exon	10770193	10770990	.	-	.	gene_id "lnc-bbug10375"	transcript_id "lnc-bbut00023882"	exon_number "1"
18	lncRNA	exon	10773924	10774325	.	-	.	gene_id "lnc-bbug10375"	transcript_id "lnc-bbut00023882"	exon_number "2"
18	lncRNA	exon	10770193	10770987	.	-	.	gene_id "lnc-bbug10375"	transcript_id "lnc-bbut00023881"	exon_number "1"
18	lncRNA	exon	10773924	10774325	.	-	.	gene_id "lnc-bbug10375"	transcript_id "lnc-bbut00023881"	exon_number "2"
18	lncRNA	exon	10931850	10932120	.	-	.	gene_id "lnc-bbug10377"	transcript_id "lnc-bbut00023885"	exon_number "1"
18	lncRNA	exon	10936120	10936204	.	-	.	gene_id "lnc-bbug10377"	transcript_id "lnc-bbut00023885"	exon_number "2"
18	lncRNA	exon	10942997	10943109	.	-	.	gene_id "lnc-bbug10377"	transcript_id "lnc-bbut00023885"	exon_number "3"
18	lncRNA	exon	10931850	10932120	.	-	.	gene_id "lnc-bbug10377"	transcript_id "lnc-bbut00023886"	exon_number "1"
18	lncRNA	exon	10936120	10936204	.	-	.	gene_id "lnc-bbug10377"	transcript_id "lnc-bbut00023886"	exon_number "2"
18	lncRNA	exon	10942903	10943113	.	-	.	gene_id "lnc-bbug10377"	transcript_id "lnc-bbut00023886"	exon_number "3"
18	lncRNA	exon	11074044	11074279	.	-	.	gene_id "lnc-bbug10378"	transcript_id "lnc-bbut00023887"	exon_number "1"
18	lncRNA	exon	11074578	11074797	.	-	.	gene_id "lnc-bbug10378"	transcript_id "lnc-bbut00023887"	exon_number "2"
18	lncRNA	exon	11076544	11076837	.	-	.	gene_id "lnc-bbug10378"	transcript_id "lnc-bbut00023887"	exon_number "3"
18	lncRNA	exon	11283442	11283584	.	-	.	gene_id "lnc-bbug10379"	transcript_id "lnc-bbut00023888"	exon_number "1"
18	lncRNA	exon	11284235	11285701	.	-	.	gene_id "lnc-bbug10379"	transcript_id "lnc-bbut00023888"	exon_number "2"
18	lncRNA	exon	11324671	11325202	.	-	.	gene_id "lnc-bbug10381"	transcript_id "lnc-bbut00023890"	exon_number "1"
18	lncRNA	exon	11327444	11327584	.	-	.	gene_id "lnc-bbug10381"	transcript_id "lnc-bbut00023890"	exon_number "2"
18	lncRNA	exon	11346398	11346442	.	-	.	gene_id "lnc-bbug10381"	transcript_id "lnc-bbut00023890"	exon_number "3"
18	lncRNA	exon	11354372	11354553	.	-	.	gene_id "lnc-bbug10381"	transcript_id "lnc-bbut00023890"	exon_number "4"
18	lncRNA	exon	11324671	11325202	.	-	.	gene_id "lnc-bbug10381"	transcript_id "lnc-bbut00023891"	exon_number "1"
18	lncRNA	exon	11327444	11327584	.	-	.	gene_id "lnc-bbug10381"	transcript_id "lnc-bbut00023891"	exon_number "2"
18	lncRNA	exon	11346398	11346442	.	-	.	gene_id "lnc-bbug10381"	transcript_id "lnc-bbut00023891"	exon_number "3"
18	lncRNA	exon	11352926	11352989	.	-	.	gene_id "lnc-bbug10381"	transcript_id "lnc-bbut00023891"	exon_number "4"
18	lncRNA	exon	11354372	11354554	.	-	.	gene_id "lnc-bbug10381"	transcript_id "lnc-bbut00023891"	exon_number "5"
18	lncRNA	exon	11499438	11499577	.	-	.	gene_id "lnc-bbug10383"	transcript_id "lnc-bbut00023894"	exon_number "1"
18	lncRNA	exon	11526046	11526329	.	-	.	gene_id "lnc-bbug10383"	transcript_id "lnc-bbut00023894"	exon_number "2"
18	lncRNA	exon	11526901	11527032	.	-	.	gene_id "lnc-bbug10383"	transcript_id "lnc-bbut00023894"	exon_number "3"
18	lncRNA	exon	11534107	11534271	.	-	.	gene_id "lnc-bbug10383"	transcript_id "lnc-bbut00023894"	exon_number "4"
18	lncRNA	exon	11689649	11689697	.	-	.	gene_id "lnc-bbug10383"	transcript_id "lnc-bbut00023894"	exon_number "5"
18	lncRNA	exon	11707072	11707206	.	-	.	gene_id "lnc-bbug10383"	transcript_id "lnc-bbut00023894"	exon_number "6"
18	lncRNA	exon	11771891	11772398	.	-	.	gene_id "lnc-bbug10383"	transcript_id "lnc-bbut00023894"	exon_number "7"
18	lncRNA	exon	11773818	11773929	.	-	.	gene_id "lnc-bbug10383"	transcript_id "lnc-bbut00023894"	exon_number "8"
18	lncRNA	exon	11590459	11590935	.	-	.	gene_id "lnc-bbug10383"	transcript_id "lnc-bbut00023898"	exon_number "1"
18	lncRNA	exon	11684398	11684636	.	-	.	gene_id "lnc-bbug10383"	transcript_id "lnc-bbut00023898"	exon_number "2"
18	lncRNA	exon	11689649	11689697	.	-	.	gene_id "lnc-bbug10383"	transcript_id "lnc-bbut00023898"	exon_number "3"
18	lncRNA	exon	11707072	11707206	.	-	.	gene_id "lnc-bbug10383"	transcript_id "lnc-bbut00023898"	exon_number "4"
18	lncRNA	exon	11771891	11772398	.	-	.	gene_id "lnc-bbug10383"	transcript_id "lnc-bbut00023898"	exon_number "5"
18	lncRNA	exon	11773818	11773929	.	-	.	gene_id "lnc-bbug10383"	transcript_id "lnc-bbut00023898"	exon_number "6"
18	lncRNA	exon	11590459	11590935	.	-	.	gene_id "lnc-bbug10383"	transcript_id "lnc-bbut00023897"	exon_number "1"
18	lncRNA	exon	11689649	11689697	.	-	.	gene_id "lnc-bbug10383"	transcript_id "lnc-bbut00023897"	exon_number "2"
18	lncRNA	exon	11707072	11707206	.	-	.	gene_id "lnc-bbug10383"	transcript_id "lnc-bbut00023897"	exon_number "3"
18	lncRNA	exon	11771891	11772398	.	-	.	gene_id "lnc-bbug10383"	transcript_id "lnc-bbut00023897"	exon_number "4"
18	lncRNA	exon	11773818	11773929	.	-	.	gene_id "lnc-bbug10383"	transcript_id "lnc-bbut00023897"	exon_number "5"
18	lncRNA	exon	11675235	11677688	.	-	.	gene_id "lnc-bbug10383"	transcript_id "lnc-bbut00023899"	exon_number "1"
18	lncRNA	exon	11684398	11684636	.	-	.	gene_id "lnc-bbug10383"	transcript_id "lnc-bbut00023899"	exon_number "2"
18	lncRNA	exon	11689649	11689697	.	-	.	gene_id "lnc-bbug10383"	transcript_id "lnc-bbut00023899"	exon_number "3"
18	lncRNA	exon	11707072	11707206	.	-	.	gene_id "lnc-bbug10383"	transcript_id "lnc-bbut00023899"	exon_number "4"
18	lncRNA	exon	11771891	11772398	.	-	.	gene_id "lnc-bbug10383"	transcript_id "lnc-bbut00023899"	exon_number "5"
18	lncRNA	exon	11773818	11773929	.	-	.	gene_id "lnc-bbug10383"	transcript_id "lnc-bbut00023899"	exon_number "6"
18	lncRNA	exon	11585334	11588555	.	-	.	gene_id "lnc-bbug10384"	transcript_id "lnc-bbut00023896"	exon_number "1"

18	lncRNA	exon	11589390	11589445	.	-	.	gene_id "lnc-bbug10384"	transcript_id "lnc-bbut00023896"	exon_number "2"
18	lncRNA	exon	12362687	12362924	.	-	.	gene_id "lnc-bbug10386"	transcript_id "lnc-bbut00023908"	exon_number "1"
18	lncRNA	exon	12366859	12366968	.	-	.	gene_id "lnc-bbug10386"	transcript_id "lnc-bbut00023908"	exon_number "2"
18	lncRNA	exon	12369893	12370029	.	-	.	gene_id "lnc-bbug10386"	transcript_id "lnc-bbut00023908"	exon_number "3"
18	lncRNA	exon	12409119	12409168	.	-	.	gene_id "lnc-bbug10386"	transcript_id "lnc-bbut00023908"	exon_number "4"
18	lncRNA	exon	12366589	12367720	.	-	.	gene_id "lnc-bbug10386"	transcript_id "lnc-bbut00023909"	exon_number "1"
18	lncRNA	exon	12369893	12370029	.	-	.	gene_id "lnc-bbug10386"	transcript_id "lnc-bbut00023909"	exon_number "2"
18	lncRNA	exon	12371611	12371719	.	-	.	gene_id "lnc-bbug10386"	transcript_id "lnc-bbut00023909"	exon_number "3"
18	lncRNA	exon	12468339	12468408	.	-	.	gene_id "lnc-bbug10386"	transcript_id "lnc-bbut00023909"	exon_number "4"
18	lncRNA	exon	12476973	12477051	.	-	.	gene_id "lnc-bbug10386"	transcript_id "lnc-bbut00023909"	exon_number "5"
18	lncRNA	exon	12366589	12367720	.	-	.	gene_id "lnc-bbug10386"	transcript_id "lnc-bbut00023910"	exon_number "1"
18	lncRNA	exon	12369893	12370029	.	-	.	gene_id "lnc-bbug10386"	transcript_id "lnc-bbut00023910"	exon_number "2"
18	lncRNA	exon	12468339	12468408	.	-	.	gene_id "lnc-bbug10386"	transcript_id "lnc-bbut00023910"	exon_number "3"
18	lncRNA	exon	12476973	12477062	.	-	.	gene_id "lnc-bbug10386"	transcript_id "lnc-bbut00023910"	exon_number "4"
18	lncRNA	exon	12366589	12367720	.	-	.	gene_id "lnc-bbug10386"	transcript_id "lnc-bbut00023911"	exon_number "1"
18	lncRNA	exon	12369893	12370029	.	-	.	gene_id "lnc-bbug10386"	transcript_id "lnc-bbut00023911"	exon_number "2"
18	lncRNA	exon	12415785	12415843	.	-	.	gene_id "lnc-bbug10386"	transcript_id "lnc-bbut00023911"	exon_number "3"
18	lncRNA	exon	12468339	12468408	.	-	.	gene_id "lnc-bbug10386"	transcript_id "lnc-bbut00023911"	exon_number "4"
18	lncRNA	exon	12476973	12477088	.	-	.	gene_id "lnc-bbug10386"	transcript_id "lnc-bbut00023911"	exon_number "5"
18	lncRNA	exon	12366589	12367720	.	-	.	gene_id "lnc-bbug10386"	transcript_id "lnc-bbut00023912"	exon_number "1"
18	lncRNA	exon	12369893	12370029	.	-	.	gene_id "lnc-bbug10386"	transcript_id "lnc-bbut00023912"	exon_number "2"
18	lncRNA	exon	12415785	12415843	.	-	.	gene_id "lnc-bbug10386"	transcript_id "lnc-bbut00023912"	exon_number "3"
18	lncRNA	exon	12438239	12438313	.	-	.	gene_id "lnc-bbug10386"	transcript_id "lnc-bbut00023912"	exon_number "4"
18	lncRNA	exon	12468339	12468408	.	-	.	gene_id "lnc-bbug10386"	transcript_id "lnc-bbut00023912"	exon_number "5"
18	lncRNA	exon	12476973	12477167	.	-	.	gene_id "lnc-bbug10386"	transcript_id "lnc-bbut00023912"	exon_number "6"
18	lncRNA	exon	12369175	12369813	.	-	.	gene_id "lnc-bbug10386"	transcript_id "lnc-bbut00023913"	exon_number "1"
18	lncRNA	exon	12369893	12370029	.	-	.	gene_id "lnc-bbug10386"	transcript_id "lnc-bbut00023913"	exon_number "2"
18	lncRNA	exon	12468339	12468408	.	-	.	gene_id "lnc-bbug10386"	transcript_id "lnc-bbut00023913"	exon_number "3"
18	lncRNA	exon	12475475	12475791	.	-	.	gene_id "lnc-bbug10386"	transcript_id "lnc-bbut00023913"	exon_number "4"
18	lncRNA	exon	12476973	12477074	.	-	.	gene_id "lnc-bbug10386"	transcript_id "lnc-bbut00023913"	exon_number "5"
18	lncRNA	exon	12369175	12369813	.	-	.	gene_id "lnc-bbug10386"	transcript_id "lnc-bbut00023915"	exon_number "1"
18	lncRNA	exon	12369893	12370029	.	-	.	gene_id "lnc-bbug10386"	transcript_id "lnc-bbut00023915"	exon_number "2"
18	lncRNA	exon	12468339	12468408	.	-	.	gene_id "lnc-bbug10386"	transcript_id "lnc-bbut00023915"	exon_number "3"
18	lncRNA	exon	12476973	12477088	.	-	.	gene_id "lnc-bbug10386"	transcript_id "lnc-bbut00023915"	exon_number "4"
18	lncRNA	exon	12369175	12369299	.	-	.	gene_id "lnc-bbug10386"	transcript_id "lnc-bbut00023914"	exon_number "1"
18	lncRNA	exon	12369893	12370029	.	-	.	gene_id "lnc-bbug10386"	transcript_id "lnc-bbut00023914"	exon_number "2"
18	lncRNA	exon	12468339	12468408	.	-	.	gene_id "lnc-bbug10386"	transcript_id "lnc-bbut00023914"	exon_number "3"
18	lncRNA	exon	12476973	12477088	.	-	.	gene_id "lnc-bbug10386"	transcript_id "lnc-bbut00023914"	exon_number "4"
18	lncRNA	exon	12407813	12407870	.	-	.	gene_id "lnc-bbug10386"	transcript_id "lnc-bbut00023916"	exon_number "1"
18	lncRNA	exon	12409119	12409172	.	-	.	gene_id "lnc-bbug10386"	transcript_id "lnc-bbut00023916"	exon_number "2"
18	lncRNA	exon	12414017	12414319	.	-	.	gene_id "lnc-bbug10386"	transcript_id "lnc-bbut00023916"	exon_number "3"
18	lncRNA	exon	12415785	12415843	.	-	.	gene_id "lnc-bbug10386"	transcript_id "lnc-bbut00023916"	exon_number "4"
18	lncRNA	exon	12438239	12438313	.	-	.	gene_id "lnc-bbug10386"	transcript_id "lnc-bbut00023916"	exon_number "5"
18	lncRNA	exon	12468339	12468408	.	-	.	gene_id "lnc-bbug10386"	transcript_id "lnc-bbut00023916"	exon_number "6"
18	lncRNA	exon	12475475	12475791	.	-	.	gene_id "lnc-bbug10386"	transcript_id "lnc-bbut00023916"	exon_number "7"
18	lncRNA	exon	12476973	12477074	.	-	.	gene_id "lnc-bbug10386"	transcript_id "lnc-bbut00023916"	exon_number "8"
18	lncRNA	exon	12407813	12407870	.	-	.	gene_id "lnc-bbug10386"	transcript_id "lnc-bbut00023917"	exon_number "1"
18	lncRNA	exon	12409119	12409172	.	-	.	gene_id "lnc-bbug10386"	transcript_id "lnc-bbut00023917"	exon_number "2"
18	lncRNA	exon	12414017	12414319	.	-	.	gene_id "lnc-bbug10386"	transcript_id "lnc-bbut00023917"	exon_number "3"
18	lncRNA	exon	12415785	12415843	.	-	.	gene_id "lnc-bbug10386"	transcript_id "lnc-bbut00023917"	exon_number "4"
18	lncRNA	exon	12438239	12438313	.	-	.	gene_id "lnc-bbug10386"	transcript_id "lnc-bbut00023917"	exon_number "5"
18	lncRNA	exon	12468339	12468408	.	-	.	gene_id "lnc-bbug10386"	transcript_id "lnc-bbut00023917"	exon_number "6"

18	lncRNA	exon	12476973	12477080	.	-	.	gene_id "lnc-bbug10386"	transcript_id "lnc-bbut00023917"	exon_number "7"
18	lncRNA	exon	12723220	12723992	.	-	.	gene_id "lnc-bbug10390"	transcript_id "lnc-bbut00023924"	exon_number "1"
18	lncRNA	exon	12725739	12726604	.	-	.	gene_id "lnc-bbug10390"	transcript_id "lnc-bbut00023924"	exon_number "2"
18	lncRNA	exon	12928810	12932622	.	-	.	gene_id "lnc-bbug10393"	transcript_id "lnc-bbut00023933"	exon_number "1"
18	lncRNA	exon	12933534	12934387	.	-	.	gene_id "lnc-bbug10393"	transcript_id "lnc-bbut00023933"	exon_number "2"
18	lncRNA	exon	13500438	13500958	.	-	.	gene_id "lnc-bbug10397"	transcript_id "lnc-bbut00023944"	exon_number "1"
18	lncRNA	exon	13502346	13502450	.	-	.	gene_id "lnc-bbug10397"	transcript_id "lnc-bbut00023944"	exon_number "2"
18	lncRNA	exon	14377906	14377950	.	-	.	gene_id "lnc-bbug10415"	transcript_id "lnc-bbut00023996"	exon_number "1"
18	lncRNA	exon	14378500	14378660	.	-	.	gene_id "lnc-bbug10415"	transcript_id "lnc-bbut00023996"	exon_number "2"
18	lncRNA	exon	14378883	14379000	.	-	.	gene_id "lnc-bbug10415"	transcript_id "lnc-bbut00023996"	exon_number "3"
18	lncRNA	exon	14381373	14381442	.	-	.	gene_id "lnc-bbug10415"	transcript_id "lnc-bbut00023996"	exon_number "4"
18	lncRNA	exon	14382245	14382339	.	-	.	gene_id "lnc-bbug10415"	transcript_id "lnc-bbut00023996"	exon_number "5"
18	lncRNA	exon	14382738	14384486	.	-	.	gene_id "lnc-bbug10415"	transcript_id "lnc-bbut00023996"	exon_number "6"
18	lncRNA	exon	14717812	14719857	.	-	.	gene_id "lnc-bbug10420"	transcript_id "lnc-bbut00024019"	exon_number "1"
18	lncRNA	exon	14727845	14728027	.	-	.	gene_id "lnc-bbug10420"	transcript_id "lnc-bbut00024019"	exon_number "2"
18	lncRNA	exon	14746638	14746734	.	-	.	gene_id "lnc-bbug10420"	transcript_id "lnc-bbut00024019"	exon_number "3"
18	lncRNA	exon	14751660	14751790	.	-	.	gene_id "lnc-bbug10420"	transcript_id "lnc-bbut00024019"	exon_number "4"
18	lncRNA	exon	15005413	15006779	.	-	.	gene_id "lnc-bbug10423"	transcript_id "lnc-bbut00024022"	exon_number "1"
18	lncRNA	exon	15007849	15007974	.	-	.	gene_id "lnc-bbug10423"	transcript_id "lnc-bbut00024022"	exon_number "2"
18	lncRNA	exon	15011466	15015225	.	-	.	gene_id "lnc-bbug10423"	transcript_id "lnc-bbut00024022"	exon_number "3"
18	lncRNA	exon	16405617	16405662	.	-	.	gene_id "lnc-bbug10429"	transcript_id "lnc-bbut00024031"	exon_number "1"
18	lncRNA	exon	16406706	16406839	.	-	.	gene_id "lnc-bbug10429"	transcript_id "lnc-bbut00024031"	exon_number "2"
18	lncRNA	exon	16409089	16409136	.	-	.	gene_id "lnc-bbug10429"	transcript_id "lnc-bbut00024031"	exon_number "3"
18	lncRNA	exon	18116347	18118449	.	-	.	gene_id "lnc-bbug10433"	transcript_id "lnc-bbut00024047"	exon_number "1"
18	lncRNA	exon	18118538	18118543	.	-	.	gene_id "lnc-bbug10433"	transcript_id "lnc-bbut00024047"	exon_number "2"
18	lncRNA	exon	19781756	19782492	.	-	.	gene_id "lnc-bbug10437"	transcript_id "lnc-bbut00024056"	exon_number "1"
18	lncRNA	exon	19783261	19783373	.	-	.	gene_id "lnc-bbug10437"	transcript_id "lnc-bbut00024056"	exon_number "2"
18	lncRNA	exon	19792104	19792419	.	-	.	gene_id "lnc-bbug10437"	transcript_id "lnc-bbut00024056"	exon_number "3"
18	lncRNA	exon	20130145	20130572	.	-	.	gene_id "lnc-bbug10438"	transcript_id "lnc-bbut00024057"	exon_number "1"
18	lncRNA	exon	20131102	20131230	.	-	.	gene_id "lnc-bbug10438"	transcript_id "lnc-bbut00024057"	exon_number "2"
18	lncRNA	exon	20416240	20419054	.	-	.	gene_id "lnc-bbug10439"	transcript_id "lnc-bbut00024058"	exon_number "1"
18	lncRNA	exon	20420677	20420896	.	-	.	gene_id "lnc-bbug10439"	transcript_id "lnc-bbut00024058"	exon_number "2"
18	lncRNA	exon	20421332	20421772	.	-	.	gene_id "lnc-bbug10439"	transcript_id "lnc-bbut00024058"	exon_number "3"
18	lncRNA	exon	21108920	21110010	.	-	.	gene_id "lnc-bbug10441"	transcript_id "lnc-bbut00024061"	exon_number "1"
18	lncRNA	exon	21119687	21121534	.	-	.	gene_id "lnc-bbug10441"	transcript_id "lnc-bbut00024061"	exon_number "2"
18	lncRNA	exon	21121623	21121869	.	-	.	gene_id "lnc-bbug10441"	transcript_id "lnc-bbut00024061"	exon_number "3"
18	lncRNA	exon	21494672	21494985	.	-	.	gene_id "lnc-bbug10443"	transcript_id "lnc-bbut00024066"	exon_number "1"
18	lncRNA	exon	21498869	21499034	.	-	.	gene_id "lnc-bbug10443"	transcript_id "lnc-bbut00024066"	exon_number "2"
18	lncRNA	exon	22732246	22733279	.	-	.	gene_id "lnc-bbug10446"	transcript_id "lnc-bbut00024074"	exon_number "1"
18	lncRNA	exon	22734687	22734903	.	-	.	gene_id "lnc-bbug10446"	transcript_id "lnc-bbut00024074"	exon_number "2"
18	lncRNA	exon	22737783	22737913	.	-	.	gene_id "lnc-bbug10446"	transcript_id "lnc-bbut00024074"	exon_number "3"
18	lncRNA	exon	22742542	22742759	.	-	.	gene_id "lnc-bbug10446"	transcript_id "lnc-bbut00024074"	exon_number "4"
18	lncRNA	exon	22743086	22744150	.	-	.	gene_id "lnc-bbug10446"	transcript_id "lnc-bbut00024074"	exon_number "5"
18	lncRNA	exon	22732246	22733279	.	-	.	gene_id "lnc-bbug10446"	transcript_id "lnc-bbut00024073"	exon_number "1"
18	lncRNA	exon	22734687	22734903	.	-	.	gene_id "lnc-bbug10446"	transcript_id "lnc-bbut00024073"	exon_number "2"
18	lncRNA	exon	22737783	22737913	.	-	.	gene_id "lnc-bbug10446"	transcript_id "lnc-bbut00024073"	exon_number "3"
18	lncRNA	exon	22742542	22744150	.	-	.	gene_id "lnc-bbug10446"	transcript_id "lnc-bbut00024073"	exon_number "4"
18	lncRNA	exon	22732246	22733279	.	-	.	gene_id "lnc-bbug10446"	transcript_id "lnc-bbut00024075"	exon_number "1"
18	lncRNA	exon	22734687	22734903	.	-	.	gene_id "lnc-bbug10446"	transcript_id "lnc-bbut00024075"	exon_number "2"
18	lncRNA	exon	22737783	22737913	.	-	.	gene_id "lnc-bbug10446"	transcript_id "lnc-bbut00024075"	exon_number "3"
18	lncRNA	exon	22742542	22742759	.	-	.	gene_id "lnc-bbug10446"	transcript_id "lnc-bbut00024075"	exon_number "4"
18	lncRNA	exon	22744157	22744205	.	-	.	gene_id "lnc-bbug10446"	transcript_id "lnc-bbut00024075"	exon_number "5"

18	lncRNA	exon	22773526	22775570	.	-	.	gene_id "lnc-bbug10447"	transcript_id "lnc-bbut00024076"	exon_number "1"
18	lncRNA	exon	22780145	22780210	.	-	.	gene_id "lnc-bbug10447"	transcript_id "lnc-bbut00024076"	exon_number "2"
18	lncRNA	exon	22781598	22781715	.	-	.	gene_id "lnc-bbug10447"	transcript_id "lnc-bbut00024076"	exon_number "3"
18	lncRNA	exon	22782944	22783248	.	-	.	gene_id "lnc-bbug10447"	transcript_id "lnc-bbut00024076"	exon_number "4"
18	lncRNA	exon	22773526	22775570	.	-	.	gene_id "lnc-bbug10447"	transcript_id "lnc-bbut00024077"	exon_number "1"
18	lncRNA	exon	22780145	22780210	.	-	.	gene_id "lnc-bbug10447"	transcript_id "lnc-bbut00024077"	exon_number "2"
18	lncRNA	exon	22781598	22781715	.	-	.	gene_id "lnc-bbug10447"	transcript_id "lnc-bbut00024077"	exon_number "3"
18	lncRNA	exon	22784995	22785106	.	-	.	gene_id "lnc-bbug10447"	transcript_id "lnc-bbut00024077"	exon_number "4"
18	lncRNA	exon	22773526	22775570	.	-	.	gene_id "lnc-bbug10447"	transcript_id "lnc-bbut00024078"	exon_number "1"
18	lncRNA	exon	22780145	22780210	.	-	.	gene_id "lnc-bbug10447"	transcript_id "lnc-bbut00024078"	exon_number "2"
18	lncRNA	exon	22781598	22781715	.	-	.	gene_id "lnc-bbug10447"	transcript_id "lnc-bbut00024078"	exon_number "3"
18	lncRNA	exon	22785150	22785395	.	-	.	gene_id "lnc-bbug10447"	transcript_id "lnc-bbut00024078"	exon_number "4"
18	lncRNA	exon	22773526	22775570	.	-	.	gene_id "lnc-bbug10447"	transcript_id "lnc-bbut00024079"	exon_number "1"
18	lncRNA	exon	22780145	22780210	.	-	.	gene_id "lnc-bbug10447"	transcript_id "lnc-bbut00024079"	exon_number "2"
18	lncRNA	exon	22781598	22781715	.	-	.	gene_id "lnc-bbug10447"	transcript_id "lnc-bbut00024079"	exon_number "3"
18	lncRNA	exon	22785282	22785673	.	-	.	gene_id "lnc-bbug10447"	transcript_id "lnc-bbut00024079"	exon_number "4"
18	lncRNA	exon	23151553	23151878	.	-	.	gene_id "lnc-bbug10448"	transcript_id "lnc-bbut00024080"	exon_number "1"
18	lncRNA	exon	23153677	23153785	.	-	.	gene_id "lnc-bbug10448"	transcript_id "lnc-bbut00024080"	exon_number "2"
18	lncRNA	exon	23261650	23261701	.	-	.	gene_id "lnc-bbug10449"	transcript_id "lnc-bbut00024081"	exon_number "1"
18	lncRNA	exon	23264845	23264953	.	-	.	gene_id "lnc-bbug10449"	transcript_id "lnc-bbut00024081"	exon_number "2"
18	lncRNA	exon	23265347	23265567	.	-	.	gene_id "lnc-bbug10449"	transcript_id "lnc-bbut00024081"	exon_number "3"
18	lncRNA	exon	23485829	23486518	.	-	.	gene_id "lnc-bbug10450"	transcript_id "lnc-bbut00024082"	exon_number "1"
18	lncRNA	exon	23578629	23578861	.	-	.	gene_id "lnc-bbug10453"	transcript_id "lnc-bbut00024085"	exon_number "1"
18	lncRNA	exon	23579802	23579866	.	-	.	gene_id "lnc-bbug10453"	transcript_id "lnc-bbut00024085"	exon_number "2"
18	lncRNA	exon	23580558	23580893	.	-	.	gene_id "lnc-bbug10453"	transcript_id "lnc-bbut00024085"	exon_number "3"
18	lncRNA	exon	23583712	23583936	.	-	.	gene_id "lnc-bbug10454"	transcript_id "lnc-bbut00024086"	exon_number "1"
18	lncRNA	exon	23584308	23584373	.	-	.	gene_id "lnc-bbug10454"	transcript_id "lnc-bbut00024086"	exon_number "2"
18	lncRNA	exon	23584977	23585132	.	-	.	gene_id "lnc-bbug10454"	transcript_id "lnc-bbut00024086"	exon_number "3"
18	lncRNA	exon	23591684	23591903	.	-	.	gene_id "lnc-bbug10455"	transcript_id "lnc-bbut00024087"	exon_number "1"
18	lncRNA	exon	23592162	23592227	.	-	.	gene_id "lnc-bbug10455"	transcript_id "lnc-bbut00024087"	exon_number "2"
18	lncRNA	exon	23592520	23592680	.	-	.	gene_id "lnc-bbug10455"	transcript_id "lnc-bbut00024087"	exon_number "3"
18	lncRNA	exon	23629694	23629960	.	-	.	gene_id "lnc-bbug10456"	transcript_id "lnc-bbut00024088"	exon_number "1"
18	lncRNA	exon	23630478	23630543	.	-	.	gene_id "lnc-bbug10456"	transcript_id "lnc-bbut00024088"	exon_number "2"
18	lncRNA	exon	23631934	23632035	.	-	.	gene_id "lnc-bbug10456"	transcript_id "lnc-bbut00024088"	exon_number "3"
18	lncRNA	exon	24217303	24217642	.	-	.	gene_id "lnc-bbug10459"	transcript_id "lnc-bbut00024094"	exon_number "1"
18	lncRNA	exon	24298451	24298901	.	-	.	gene_id "lnc-bbug10460"	transcript_id "lnc-bbut00024095"	exon_number "1"
18	lncRNA	exon	24300979	24301149	.	-	.	gene_id "lnc-bbug10460"	transcript_id "lnc-bbut00024095"	exon_number "2"
18	lncRNA	exon	24305733	24306083	.	-	.	gene_id "lnc-bbug10460"	transcript_id "lnc-bbut00024095"	exon_number "3"
18	lncRNA	exon	24550877	24552143	.	-	.	gene_id "lnc-bbug10461"	transcript_id "lnc-bbut00024096"	exon_number "1"
18	lncRNA	exon	24555284	24559039	.	-	.	gene_id "lnc-bbug10461"	transcript_id "lnc-bbut00024096"	exon_number "2"
18	lncRNA	exon	25419892	25420172	.	-	.	gene_id "lnc-bbug10472"	transcript_id "lnc-bbut00024125"	exon_number "1"
18	lncRNA	exon	25424068	25424255	.	-	.	gene_id "lnc-bbug10472"	transcript_id "lnc-bbut00024125"	exon_number "2"
18	lncRNA	exon	26062694	26065553	.	-	.	gene_id "lnc-bbug10481"	transcript_id "lnc-bbut00024160"	exon_number "1"
18	lncRNA	exon	26253964	26254723	.	-	.	gene_id "lnc-bbug10482"	transcript_id "lnc-bbut00024161"	exon_number "1"
18	lncRNA	exon	26257122	26257127	.	-	.	gene_id "lnc-bbug10482"	transcript_id "lnc-bbut00024161"	exon_number "2"
18	lncRNA	exon	29718458	29719108	.	-	.	gene_id "lnc-bbug10487"	transcript_id "lnc-bbut00024166"	exon_number "1"
18	lncRNA	exon	31906399	31906560	.	-	.	gene_id "lnc-bbug10489"	transcript_id "lnc-bbut00024168"	exon_number "1"
18	lncRNA	exon	31935886	31936095	.	-	.	gene_id "lnc-bbug10489"	transcript_id "lnc-bbut00024168"	exon_number "2"
18	lncRNA	exon	31937088	31937253	.	-	.	gene_id "lnc-bbug10489"	transcript_id "lnc-bbut00024168"	exon_number "3"
18	lncRNA	exon	33287767	33287938	.	-	.	gene_id "lnc-bbug10492"	transcript_id "lnc-bbut00024171"	exon_number "1"
18	lncRNA	exon	33289272	33289317	.	-	.	gene_id "lnc-bbug10492"	transcript_id "lnc-bbut00024171"	exon_number "2"
18	lncRNA	exon	33410803	33410883	.	-	.	gene_id "lnc-bbug10492"	transcript_id "lnc-bbut00024171"	exon_number "3"

18	lncRNA	exon	33476386	33476593	.	-	.	gene_id "lnc-bbug10492"	transcript_id "lnc-bbut00024171"	exon_number "4"
18	lncRNA	exon	33326917	33327538	.	-	.	gene_id "lnc-bbug10493"	transcript_id "lnc-bbut00024172"	exon_number "1"
18	lncRNA	exon	34334295	34334882	.	-	.	gene_id "lnc-bbug10503"	transcript_id "lnc-bbut00024195"	exon_number "1"
18	lncRNA	exon	34543217	34544028	.	-	.	gene_id "lnc-bbug10509"	transcript_id "lnc-bbut00024216"	exon_number "1"
18	lncRNA	exon	34547404	34547424	.	-	.	gene_id "lnc-bbug10509"	transcript_id "lnc-bbut00024216"	exon_number "2"
18	lncRNA	exon	34788153	34789165	.	-	.	gene_id "lnc-bbug10516"	transcript_id "lnc-bbut00024239"	exon_number "1"
18	lncRNA	exon	34789699	34789785	.	-	.	gene_id "lnc-bbug10516"	transcript_id "lnc-bbut00024239"	exon_number "2"
18	lncRNA	exon	34794844	34794970	.	-	.	gene_id "lnc-bbug10516"	transcript_id "lnc-bbut00024239"	exon_number "3"
18	lncRNA	exon	34796980	34797062	.	-	.	gene_id "lnc-bbug10516"	transcript_id "lnc-bbut00024239"	exon_number "4"
18	lncRNA	exon	34806073	34806250	.	-	.	gene_id "lnc-bbug10516"	transcript_id "lnc-bbut00024239"	exon_number "5"
18	lncRNA	exon	34788153	34789165	.	-	.	gene_id "lnc-bbug10516"	transcript_id "lnc-bbut00024240"	exon_number "1"
18	lncRNA	exon	34789699	34789785	.	-	.	gene_id "lnc-bbug10516"	transcript_id "lnc-bbut00024240"	exon_number "2"
18	lncRNA	exon	34794844	34794970	.	-	.	gene_id "lnc-bbug10516"	transcript_id "lnc-bbut00024240"	exon_number "3"
18	lncRNA	exon	34796980	34797062	.	-	.	gene_id "lnc-bbug10516"	transcript_id "lnc-bbut00024240"	exon_number "4"
18	lncRNA	exon	34811683	34812180	.	-	.	gene_id "lnc-bbug10516"	transcript_id "lnc-bbut00024240"	exon_number "5"
18	lncRNA	exon	34823437	34823567	.	-	.	gene_id "lnc-bbug10517"	transcript_id "lnc-bbut00024241"	exon_number "1"
18	lncRNA	exon	34825604	34828329	.	-	.	gene_id "lnc-bbug10517"	transcript_id "lnc-bbut00024241"	exon_number "2"
18	lncRNA	exon	34951909	34952228	.	-	.	gene_id "lnc-bbug10521"	transcript_id "lnc-bbut00024261"	exon_number "1"
18	lncRNA	exon	35162799	35163753	.	-	.	gene_id "lnc-bbug10529"	transcript_id "lnc-bbut00024284"	exon_number "1"
18	lncRNA	exon	35165096	35167836	.	-	.	gene_id "lnc-bbug10529"	transcript_id "lnc-bbut00024284"	exon_number "2"
18	lncRNA	exon	35318743	35319406	.	-	.	gene_id "lnc-bbug10532"	transcript_id "lnc-bbut00024292"	exon_number "1"
18	lncRNA	exon	35524387	35524832	.	-	.	gene_id "lnc-bbug10535"	transcript_id "lnc-bbut00024299"	exon_number "1"
18	lncRNA	exon	37668686	37671604	.	-	.	gene_id "lnc-bbug10546"	transcript_id "lnc-bbut00024314"	exon_number "1"
18	lncRNA	exon	37671873	37672044	.	-	.	gene_id "lnc-bbug10546"	transcript_id "lnc-bbut00024314"	exon_number "2"
18	lncRNA	exon	37672637	37675335	.	-	.	gene_id "lnc-bbug10546"	transcript_id "lnc-bbut00024314"	exon_number "3"
18	lncRNA	exon	37801153	37802847	.	-	.	gene_id "lnc-bbug10547"	transcript_id "lnc-bbut00024315"	exon_number "1"
18	lncRNA	exon	37803427	37813273	.	-	.	gene_id "lnc-bbug10547"	transcript_id "lnc-bbut00024315"	exon_number "2"
18	lncRNA	exon	38052602	38053965	.	-	.	gene_id "lnc-bbug10548"	transcript_id "lnc-bbut00024316"	exon_number "1"
18	lncRNA	exon	38054237	38054598	.	-	.	gene_id "lnc-bbug10548"	transcript_id "lnc-bbut00024316"	exon_number "2"
18	lncRNA	exon	38055178	38055262	.	-	.	gene_id "lnc-bbug10548"	transcript_id "lnc-bbut00024316"	exon_number "3"
18	lncRNA	exon	38057411	38057516	.	-	.	gene_id "lnc-bbug10548"	transcript_id "lnc-bbut00024316"	exon_number "4"
18	lncRNA	exon	38062061	38062360	.	-	.	gene_id "lnc-bbug10549"	transcript_id "lnc-bbut00024317"	exon_number "1"
18	lncRNA	exon	38076258	38076395	.	-	.	gene_id "lnc-bbug10549"	transcript_id "lnc-bbut00024317"	exon_number "2"
18	lncRNA	exon	38160143	38160218	.	-	.	gene_id "lnc-bbug10549"	transcript_id "lnc-bbut00024317"	exon_number "3"
18	lncRNA	exon	38232120	38232310	.	-	.	gene_id "lnc-bbug10549"	transcript_id "lnc-bbut00024317"	exon_number "4"
18	lncRNA	exon	38062061	38062360	.	-	.	gene_id "lnc-bbug10549"	transcript_id "lnc-bbut00024318"	exon_number "1"
18	lncRNA	exon	38076258	38076395	.	-	.	gene_id "lnc-bbug10549"	transcript_id "lnc-bbut00024318"	exon_number "2"
18	lncRNA	exon	38160143	38160218	.	-	.	gene_id "lnc-bbug10549"	transcript_id "lnc-bbut00024318"	exon_number "3"
18	lncRNA	exon	38189120	38189279	.	-	.	gene_id "lnc-bbug10549"	transcript_id "lnc-bbut00024318"	exon_number "4"
18	lncRNA	exon	38232120	38232319	.	-	.	gene_id "lnc-bbug10549"	transcript_id "lnc-bbut00024318"	exon_number "5"
18	lncRNA	exon	38887316	38887679	.	-	.	gene_id "lnc-bbug10554"	transcript_id "lnc-bbut00024324"	exon_number "1"
18	lncRNA	exon	38889983	38891741	.	-	.	gene_id "lnc-bbug10554"	transcript_id "lnc-bbut00024324"	exon_number "2"
18	lncRNA	exon	39218652	39218853	.	-	.	gene_id "lnc-bbug10558"	transcript_id "lnc-bbut00024332"	exon_number "1"
18	lncRNA	exon	39220766	39221052	.	-	.	gene_id "lnc-bbug10558"	transcript_id "lnc-bbut00024332"	exon_number "2"
18	lncRNA	exon	39979187	39979244	.	-	.	gene_id "lnc-bbug10562"	transcript_id "lnc-bbut00024339"	exon_number "1"
18	lncRNA	exon	39979853	39980070	.	-	.	gene_id "lnc-bbug10562"	transcript_id "lnc-bbut00024339"	exon_number "2"
18	lncRNA	exon	39981038	39982610	.	-	.	gene_id "lnc-bbug10562"	transcript_id "lnc-bbut00024339"	exon_number "3"
18	lncRNA	exon	39979187	39979244	.	-	.	gene_id "lnc-bbug10562"	transcript_id "lnc-bbut00024338"	exon_number "1"
18	lncRNA	exon	39979633	39980070	.	-	.	gene_id "lnc-bbug10562"	transcript_id "lnc-bbut00024338"	exon_number "2"
18	lncRNA	exon	39981038	39982610	.	-	.	gene_id "lnc-bbug10562"	transcript_id "lnc-bbut00024338"	exon_number "3"
18	lncRNA	exon	39979187	39979228	.	-	.	gene_id "lnc-bbug10562"	transcript_id "lnc-bbut00024337"	exon_number "1"
18	lncRNA	exon	39979853	39980070	.	-	.	gene_id "lnc-bbug10562"	transcript_id "lnc-bbut00024337"	exon_number "2"

18	lncRNA	exon	39981038	39982610	.	-	.	gene_id "lnc-bbug10562"	transcript_id "lnc-bbut00024337"	exon_number "3"
18	lncRNA	exon	39979517	39980070	.	-	.	gene_id "lnc-bbug10562"	transcript_id "lnc-bbut00024340"	exon_number "1"
18	lncRNA	exon	39981038	39981198	.	-	.	gene_id "lnc-bbug10562"	transcript_id "lnc-bbut00024340"	exon_number "2"
18	lncRNA	exon	39983430	39985001	.	-	.	gene_id "lnc-bbug10562"	transcript_id "lnc-bbut00024340"	exon_number "3"
18	lncRNA	exon	39985120	39986115	.	-	.	gene_id "lnc-bbug10562"	transcript_id "lnc-bbut00024340"	exon_number "4"
18	lncRNA	exon	40075031	40077236	.	-	.	gene_id "lnc-bbug10563"	transcript_id "lnc-bbut00024341"	exon_number "1"
18	lncRNA	exon	40087343	40087432	.	-	.	gene_id "lnc-bbug10563"	transcript_id "lnc-bbut00024341"	exon_number "2"
18	lncRNA	exon	40196135	40197174	.	-	.	gene_id "lnc-bbug10564"	transcript_id "lnc-bbut00024344"	exon_number "1"
18	lncRNA	exon	40198545	40198635	.	-	.	gene_id "lnc-bbug10564"	transcript_id "lnc-bbut00024344"	exon_number "2"
18	lncRNA	exon	40201247	40201337	.	-	.	gene_id "lnc-bbug10564"	transcript_id "lnc-bbut00024344"	exon_number "3"
18	lncRNA	exon	40205259	40205429	.	-	.	gene_id "lnc-bbug10564"	transcript_id "lnc-bbut00024344"	exon_number "4"
18	lncRNA	exon	40196135	40197174	.	-	.	gene_id "lnc-bbug10564"	transcript_id "lnc-bbut00024343"	exon_number "1"
18	lncRNA	exon	40201247	40201337	.	-	.	gene_id "lnc-bbug10564"	transcript_id "lnc-bbut00024343"	exon_number "2"
18	lncRNA	exon	40205259	40205429	.	-	.	gene_id "lnc-bbug10564"	transcript_id "lnc-bbut00024343"	exon_number "3"
18	lncRNA	exon	40577043	40578081	.	-	.	gene_id "lnc-bbug10565"	transcript_id "lnc-bbut00024345"	exon_number "1"
18	lncRNA	exon	40584512	40584825	.	-	.	gene_id "lnc-bbug10565"	transcript_id "lnc-bbut00024345"	exon_number "2"
18	lncRNA	exon	40885080	40885943	.	-	.	gene_id "lnc-bbug10566"	transcript_id "lnc-bbut00024346"	exon_number "1"
18	lncRNA	exon	40887520	40887557	.	-	.	gene_id "lnc-bbug10566"	transcript_id "lnc-bbut00024346"	exon_number "2"
18	lncRNA	exon	40888696	40888803	.	-	.	gene_id "lnc-bbug10566"	transcript_id "lnc-bbut00024346"	exon_number "3"
18	lncRNA	exon	40889062	40890600	.	-	.	gene_id "lnc-bbug10566"	transcript_id "lnc-bbut00024346"	exon_number "4"
18	lncRNA	exon	40885080	40885943	.	-	.	gene_id "lnc-bbug10566"	transcript_id "lnc-bbut00024347"	exon_number "1"
18	lncRNA	exon	40887520	40887557	.	-	.	gene_id "lnc-bbug10566"	transcript_id "lnc-bbut00024347"	exon_number "2"
18	lncRNA	exon	40888696	40890605	.	-	.	gene_id "lnc-bbug10566"	transcript_id "lnc-bbut00024347"	exon_number "3"
18	lncRNA	exon	40973089	40976388	.	-	.	gene_id "lnc-bbug10567"	transcript_id "lnc-bbut00024348"	exon_number "1"
18	lncRNA	exon	40986877	40987127	.	-	.	gene_id "lnc-bbug10567"	transcript_id "lnc-bbut00024348"	exon_number "2"
18	lncRNA	exon	40996448	40996629	.	-	.	gene_id "lnc-bbug10568"	transcript_id "lnc-bbut00024349"	exon_number "1"
18	lncRNA	exon	41005091	41005185	.	-	.	gene_id "lnc-bbug10568"	transcript_id "lnc-bbut00024349"	exon_number "2"
18	lncRNA	exon	41030499	41030656	.	-	.	gene_id "lnc-bbug10568"	transcript_id "lnc-bbut00024349"	exon_number "3"
18	lncRNA	exon	41030777	41031010	.	-	.	gene_id "lnc-bbug10568"	transcript_id "lnc-bbut00024349"	exon_number "4"
18	lncRNA	exon	41594082	41595119	.	-	.	gene_id "lnc-bbug10569"	transcript_id "lnc-bbut00024350"	exon_number "1"
18	lncRNA	exon	41602273	41602307	.	-	.	gene_id "lnc-bbug10569"	transcript_id "lnc-bbut00024350"	exon_number "2"
18	lncRNA	exon	41603990	41604268	.	-	.	gene_id "lnc-bbug10569"	transcript_id "lnc-bbut00024350"	exon_number "3"
18	lncRNA	exon	42925438	42925947	.	-	.	gene_id "lnc-bbug10574"	transcript_id "lnc-bbut00024358"	exon_number "1"
18	lncRNA	exon	42926931	42927111	.	-	.	gene_id "lnc-bbug10574"	transcript_id "lnc-bbut00024358"	exon_number "2"
18	lncRNA	exon	42929724	42929861	.	-	.	gene_id "lnc-bbug10574"	transcript_id "lnc-bbut00024358"	exon_number "3"
18	lncRNA	exon	44028724	44029963	.	-	.	gene_id "lnc-bbug10581"	transcript_id "lnc-bbut00024371"	exon_number "1"
18	lncRNA	exon	44038924	44039235	.	-	.	gene_id "lnc-bbug10581"	transcript_id "lnc-bbut00024371"	exon_number "2"
18	lncRNA	exon	44040472	44042312	.	-	.	gene_id "lnc-bbug10581"	transcript_id "lnc-bbut00024371"	exon_number "3"
18	lncRNA	exon	44087938	44088213	.	-	.	gene_id "lnc-bbug10582"	transcript_id "lnc-bbut00024372"	exon_number "1"
18	lncRNA	exon	44089447	44089703	.	-	.	gene_id "lnc-bbug10582"	transcript_id "lnc-bbut00024372"	exon_number "2"
18	lncRNA	exon	44090859	44091301	.	-	.	gene_id "lnc-bbug10582"	transcript_id "lnc-bbut00024372"	exon_number "3"
18	lncRNA	exon	44310519	44311521	.	-	.	gene_id "lnc-bbug10583"	transcript_id "lnc-bbut00024373"	exon_number "1"
18	lncRNA	exon	44311679	44312486	.	-	.	gene_id "lnc-bbug10583"	transcript_id "lnc-bbut00024373"	exon_number "2"
18	lncRNA	exon	44328549	44328613	.	-	.	gene_id "lnc-bbug10583"	transcript_id "lnc-bbut00024373"	exon_number "3"
18	lncRNA	exon	44643224	44644618	.	-	.	gene_id "lnc-bbug10584"	transcript_id "lnc-bbut00024374"	exon_number "1"
18	lncRNA	exon	45436769	45437179	.	-	.	gene_id "lnc-bbug10591"	transcript_id "lnc-bbut00024385"	exon_number "1"
18	lncRNA	exon	45441565	45441749	.	-	.	gene_id "lnc-bbug10591"	transcript_id "lnc-bbut00024385"	exon_number "2"
18	lncRNA	exon	45444766	45445348	.	-	.	gene_id "lnc-bbug10591"	transcript_id "lnc-bbut00024385"	exon_number "3"
18	lncRNA	exon	45770047	45770214	.	-	.	gene_id "lnc-bbug10595"	transcript_id "lnc-bbut00024394"	exon_number "1"
18	lncRNA	exon	45770809	45770935	.	-	.	gene_id "lnc-bbug10595"	transcript_id "lnc-bbut00024394"	exon_number "2"
18	lncRNA	exon	45836149	45836612	.	-	.	gene_id "lnc-bbug10596"	transcript_id "lnc-bbut00024395"	exon_number "1"
18	lncRNA	exon	45838329	45838374	.	-	.	gene_id "lnc-bbug10596"	transcript_id "lnc-bbut00024395"	exon_number "2"

18	lncRNA	exon	45918264	45918413	.	-	.	gene_id "lnc-bbug10597"	transcript_id "lnc-bbut00024397"	exon_number "1"
18	lncRNA	exon	45919094	45919141	.	-	.	gene_id "lnc-bbug10597"	transcript_id "lnc-bbut00024397"	exon_number "2"
18	lncRNA	exon	45919941	45919985	.	-	.	gene_id "lnc-bbug10597"	transcript_id "lnc-bbut00024397"	exon_number "3"
18	lncRNA	exon	45920300	45920338	.	-	.	gene_id "lnc-bbug10597"	transcript_id "lnc-bbut00024397"	exon_number "4"
18	lncRNA	exon	45921598	45921751	.	-	.	gene_id "lnc-bbug10597"	transcript_id "lnc-bbut00024397"	exon_number "5"
18	lncRNA	exon	46051198	46053840	.	-	.	gene_id "lnc-bbug10601"	transcript_id "lnc-bbut00024401"	exon_number "1"
18	lncRNA	exon	46376392	46376627	.	-	.	gene_id "lnc-bbug10610"	transcript_id "lnc-bbut00024425"	exon_number "1"
18	lncRNA	exon	46378256	46378302	.	-	.	gene_id "lnc-bbug10610"	transcript_id "lnc-bbut00024425"	exon_number "2"
18	lncRNA	exon	46378457	46378591	.	-	.	gene_id "lnc-bbug10610"	transcript_id "lnc-bbut00024425"	exon_number "3"
18	lncRNA	exon	46378752	46378778	.	-	.	gene_id "lnc-bbug10610"	transcript_id "lnc-bbut00024425"	exon_number "4"
18	lncRNA	exon	46379156	46379306	.	-	.	gene_id "lnc-bbug10610"	transcript_id "lnc-bbut00024425"	exon_number "5"
18	lncRNA	exon	46376392	46376627	.	-	.	gene_id "lnc-bbug10610"	transcript_id "lnc-bbut00024424"	exon_number "1"
18	lncRNA	exon	46378256	46378299	.	-	.	gene_id "lnc-bbug10610"	transcript_id "lnc-bbut00024424"	exon_number "2"
18	lncRNA	exon	46378457	46378591	.	-	.	gene_id "lnc-bbug10610"	transcript_id "lnc-bbut00024424"	exon_number "3"
18	lncRNA	exon	46378752	46378778	.	-	.	gene_id "lnc-bbug10610"	transcript_id "lnc-bbut00024424"	exon_number "4"
18	lncRNA	exon	46379156	46379306	.	-	.	gene_id "lnc-bbug10610"	transcript_id "lnc-bbut00024424"	exon_number "5"
18	lncRNA	exon	46590660	46590798	.	-	.	gene_id "lnc-bbug10616"	transcript_id "lnc-bbut00024435"	exon_number "1"
18	lncRNA	exon	46591173	46591257	.	-	.	gene_id "lnc-bbug10616"	transcript_id "lnc-bbut00024435"	exon_number "2"
18	lncRNA	exon	46591392	46591478	.	-	.	gene_id "lnc-bbug10616"	transcript_id "lnc-bbut00024435"	exon_number "3"
18	lncRNA	exon	46592175	46592280	.	-	.	gene_id "lnc-bbug10616"	transcript_id "lnc-bbut00024435"	exon_number "4"
18	lncRNA	exon	46807075	46807393	.	-	.	gene_id "lnc-bbug10620"	transcript_id "lnc-bbut00024452"	exon_number "1"
18	lncRNA	exon	46807658	46807832	.	-	.	gene_id "lnc-bbug10620"	transcript_id "lnc-bbut00024452"	exon_number "2"
18	lncRNA	exon	46867750	46868104	.	-	.	gene_id "lnc-bbug10621"	transcript_id "lnc-bbut00024453"	exon_number "1"
18	lncRNA	exon	46870504	46870602	.	-	.	gene_id "lnc-bbug10621"	transcript_id "lnc-bbut00024453"	exon_number "2"
18	lncRNA	exon	46871759	46872043	.	-	.	gene_id "lnc-bbug10621"	transcript_id "lnc-bbut00024453"	exon_number "3"
18	lncRNA	exon	46900626	46902023	.	-	.	gene_id "lnc-bbug10622"	transcript_id "lnc-bbut00024454"	exon_number "1"
18	lncRNA	exon	46902606	46903281	.	-	.	gene_id "lnc-bbug10622"	transcript_id "lnc-bbut00024454"	exon_number "2"
18	lncRNA	exon	47080728	47086231	.	-	.	gene_id "lnc-bbug10626"	transcript_id "lnc-bbut00024471"	exon_number "1"
18	lncRNA	exon	47301516	47301836	.	-	.	gene_id "lnc-bbug10629"	transcript_id "lnc-bbut00024474"	exon_number "1"
18	lncRNA	exon	47302231	47302315	.	-	.	gene_id "lnc-bbug10629"	transcript_id "lnc-bbut00024474"	exon_number "2"
18	lncRNA	exon	47307239	47307356	.	-	.	gene_id "lnc-bbug10629"	transcript_id "lnc-bbut00024474"	exon_number "3"
18	lncRNA	exon	48221751	48222354	.	-	.	gene_id "lnc-bbug10641"	transcript_id "lnc-bbut00024513"	exon_number "1"
18	lncRNA	exon	48227477	48227602	.	-	.	gene_id "lnc-bbug10641"	transcript_id "lnc-bbut00024513"	exon_number "2"
18	lncRNA	exon	48228635	48228709	.	-	.	gene_id "lnc-bbug10641"	transcript_id "lnc-bbut00024513"	exon_number "3"
18	lncRNA	exon	48233203	48233313	.	-	.	gene_id "lnc-bbug10641"	transcript_id "lnc-bbut00024513"	exon_number "4"
18	lncRNA	exon	48488556	48488925	.	-	.	gene_id "lnc-bbug10652"	transcript_id "lnc-bbut00024534"	exon_number "1"
18	lncRNA	exon	48489345	48489429	.	-	.	gene_id "lnc-bbug10652"	transcript_id "lnc-bbut00024534"	exon_number "2"
18	lncRNA	exon	48505374	48505379	.	-	.	gene_id "lnc-bbug10652"	transcript_id "lnc-bbut00024534"	exon_number "3"
18	lncRNA	exon	48609943	48610452	.	-	.	gene_id "lnc-bbug10659"	transcript_id "lnc-bbut00024541"	exon_number "1"
18	lncRNA	exon	48719494	48719658	.	-	.	gene_id "lnc-bbug10660"	transcript_id "lnc-bbut00024542"	exon_number "1"
18	lncRNA	exon	48720505	48720922	.	-	.	gene_id "lnc-bbug10660"	transcript_id "lnc-bbut00024542"	exon_number "2"
18	lncRNA	exon	48778348	48778468	.	-	.	gene_id "lnc-bbug10663"	transcript_id "lnc-bbut00024545"	exon_number "1"
18	lncRNA	exon	48793331	48793393	.	-	.	gene_id "lnc-bbug10663"	transcript_id "lnc-bbut00024545"	exon_number "2"
18	lncRNA	exon	48796011	48796254	.	-	.	gene_id "lnc-bbug10663"	transcript_id "lnc-bbut00024545"	exon_number "3"
18	lncRNA	exon	49145492	49153372	.	-	.	gene_id "lnc-bbug10672"	transcript_id "lnc-bbut00024561"	exon_number "1"
18	lncRNA	exon	49153413	49156318	.	-	.	gene_id "lnc-bbug10672"	transcript_id "lnc-bbut00024561"	exon_number "2"
18	lncRNA	exon	49274414	49275789	.	-	.	gene_id "lnc-bbug10677"	transcript_id "lnc-bbut00024569"	exon_number "1"
18	lncRNA	exon	49281252	49281442	.	-	.	gene_id "lnc-bbug10677"	transcript_id "lnc-bbut00024569"	exon_number "2"
18	lncRNA	exon	49283998	49284212	.	-	.	gene_id "lnc-bbug10677"	transcript_id "lnc-bbut00024569"	exon_number "3"
18	lncRNA	exon	49274414	49275789	.	-	.	gene_id "lnc-bbug10677"	transcript_id "lnc-bbut00024570"	exon_number "1"
18	lncRNA	exon	49278951	49279052	.	-	.	gene_id "lnc-bbug10677"	transcript_id "lnc-bbut00024570"	exon_number "2"
18	lncRNA	exon	49281252	49281442	.	-	.	gene_id "lnc-bbug10677"	transcript_id "lnc-bbut00024570"	exon_number "3"

18	lncRNA	exon	49283998	49284217	.	-	.	gene_id "lnc-bbug10677"	transcript_id "lnc-bbut00024570"	exon_number "4"
18	lncRNA	exon	49274414	49275789	.	-	.	gene_id "lnc-bbug10677"	transcript_id "lnc-bbut00024572"	exon_number "1"
18	lncRNA	exon	49278951	49279052	.	-	.	gene_id "lnc-bbug10677"	transcript_id "lnc-bbut00024572"	exon_number "2"
18	lncRNA	exon	49281252	49281442	.	-	.	gene_id "lnc-bbug10677"	transcript_id "lnc-bbut00024572"	exon_number "3"
18	lncRNA	exon	49284318	49284475	.	-	.	gene_id "lnc-bbug10677"	transcript_id "lnc-bbut00024572"	exon_number "4"
18	lncRNA	exon	49284886	49285062	.	-	.	gene_id "lnc-bbug10677"	transcript_id "lnc-bbut00024572"	exon_number "5"
18	lncRNA	exon	49293816	49293917	.	-	.	gene_id "lnc-bbug10677"	transcript_id "lnc-bbut00024572"	exon_number "6"
18	lncRNA	exon	49297212	49297328	.	-	.	gene_id "lnc-bbug10677"	transcript_id "lnc-bbut00024572"	exon_number "7"
18	lncRNA	exon	49297716	49297831	.	-	.	gene_id "lnc-bbug10677"	transcript_id "lnc-bbut00024572"	exon_number "8"
18	lncRNA	exon	49274414	49275789	.	-	.	gene_id "lnc-bbug10677"	transcript_id "lnc-bbut00024571"	exon_number "1"
18	lncRNA	exon	49278951	49279052	.	-	.	gene_id "lnc-bbug10677"	transcript_id "lnc-bbut00024571"	exon_number "2"
18	lncRNA	exon	49281252	49281442	.	-	.	gene_id "lnc-bbug10677"	transcript_id "lnc-bbut00024571"	exon_number "3"
18	lncRNA	exon	49284886	49285062	.	-	.	gene_id "lnc-bbug10677"	transcript_id "lnc-bbut00024571"	exon_number "4"
18	lncRNA	exon	49293816	49293917	.	-	.	gene_id "lnc-bbug10677"	transcript_id "lnc-bbut00024571"	exon_number "5"
18	lncRNA	exon	49297212	49297328	.	-	.	gene_id "lnc-bbug10677"	transcript_id "lnc-bbut00024571"	exon_number "6"
18	lncRNA	exon	49297716	49297831	.	-	.	gene_id "lnc-bbug10677"	transcript_id "lnc-bbut00024571"	exon_number "7"
18	lncRNA	exon	49340856	49341952	.	-	.	gene_id "lnc-bbug10678"	transcript_id "lnc-bbut00024574"	exon_number "1"
18	lncRNA	exon	49343026	49343097	.	-	.	gene_id "lnc-bbug10678"	transcript_id "lnc-bbut00024574"	exon_number "2"
18	lncRNA	exon	49355529	49355730	.	-	.	gene_id "lnc-bbug10678"	transcript_id "lnc-bbut00024574"	exon_number "3"
18	lncRNA	exon	49356220	49356339	.	-	.	gene_id "lnc-bbug10678"	transcript_id "lnc-bbut00024574"	exon_number "4"
18	lncRNA	exon	49358805	49358924	.	-	.	gene_id "lnc-bbug10678"	transcript_id "lnc-bbut00024574"	exon_number "5"
18	lncRNA	exon	49376695	49376804	.	-	.	gene_id "lnc-bbug10678"	transcript_id "lnc-bbut00024574"	exon_number "6"
18	lncRNA	exon	49340856	49341952	.	-	.	gene_id "lnc-bbug10678"	transcript_id "lnc-bbut00024573"	exon_number "1"
18	lncRNA	exon	49355529	49355730	.	-	.	gene_id "lnc-bbug10678"	transcript_id "lnc-bbut00024573"	exon_number "2"
18	lncRNA	exon	49356220	49356339	.	-	.	gene_id "lnc-bbug10678"	transcript_id "lnc-bbut00024573"	exon_number "3"
18	lncRNA	exon	49358805	49358924	.	-	.	gene_id "lnc-bbug10678"	transcript_id "lnc-bbut00024573"	exon_number "4"
18	lncRNA	exon	49376695	49376804	.	-	.	gene_id "lnc-bbug10678"	transcript_id "lnc-bbut00024573"	exon_number "5"
18	lncRNA	exon	49352498	49355730	.	-	.	gene_id "lnc-bbug10678"	transcript_id "lnc-bbut00024575"	exon_number "1"
18	lncRNA	exon	49356220	49356339	.	-	.	gene_id "lnc-bbug10678"	transcript_id "lnc-bbut00024575"	exon_number "2"
18	lncRNA	exon	49358805	49358924	.	-	.	gene_id "lnc-bbug10678"	transcript_id "lnc-bbut00024575"	exon_number "3"
18	lncRNA	exon	49376695	49376804	.	-	.	gene_id "lnc-bbug10678"	transcript_id "lnc-bbut00024575"	exon_number "4"
18	lncRNA	exon	49650010	49650557	.	-	.	gene_id "lnc-bbug10687"	transcript_id "lnc-bbut00024591"	exon_number "1"
18	lncRNA	exon	49653756	49653873	.	-	.	gene_id "lnc-bbug10687"	transcript_id "lnc-bbut00024591"	exon_number "2"
18	lncRNA	exon	49655412	49655524	.	-	.	gene_id "lnc-bbug10687"	transcript_id "lnc-bbut00024591"	exon_number "3"
18	lncRNA	exon	49837278	49837646	.	-	.	gene_id "lnc-bbug10692"	transcript_id "lnc-bbut00024607"	exon_number "1"
18	lncRNA	exon	49838397	49838563	.	-	.	gene_id "lnc-bbug10692"	transcript_id "lnc-bbut00024607"	exon_number "2"
18	lncRNA	exon	49995784	49996039	.	-	.	gene_id "lnc-bbug10696"	transcript_id "lnc-bbut00024611"	exon_number "1"
18	lncRNA	exon	49999628	49999760	.	-	.	gene_id "lnc-bbug10696"	transcript_id "lnc-bbut00024611"	exon_number "2"
18	lncRNA	exon	50159972	50160493	.	-	.	gene_id "lnc-bbug10701"	transcript_id "lnc-bbut00024618"	exon_number "1"
18	lncRNA	exon	50246610	50247019	.	-	.	gene_id "lnc-bbug10702"	transcript_id "lnc-bbut00024619"	exon_number "1"
18	lncRNA	exon	50250855	50251003	.	-	.	gene_id "lnc-bbug10702"	transcript_id "lnc-bbut00024619"	exon_number "2"
18	lncRNA	exon	50251684	50252077	.	-	.	gene_id "lnc-bbug10702"	transcript_id "lnc-bbut00024619"	exon_number "3"
18	lncRNA	exon	50275465	50275550	.	-	.	gene_id "lnc-bbug10703"	transcript_id "lnc-bbut00024620"	exon_number "1"
18	lncRNA	exon	50283710	50283806	.	-	.	gene_id "lnc-bbug10703"	transcript_id "lnc-bbut00024620"	exon_number "2"
18	lncRNA	exon	50288477	50288540	.	-	.	gene_id "lnc-bbug10703"	transcript_id "lnc-bbut00024620"	exon_number "3"
18	lncRNA	exon	50364036	50364493	.	-	.	gene_id "lnc-bbug10707"	transcript_id "lnc-bbut00024628"	exon_number "1"
18	lncRNA	exon	50365146	50365281	.	-	.	gene_id "lnc-bbug10707"	transcript_id "lnc-bbut00024628"	exon_number "2"
18	lncRNA	exon	50365564	50366001	.	-	.	gene_id "lnc-bbug10707"	transcript_id "lnc-bbut00024628"	exon_number "3"
18	lncRNA	exon	50368064	50369304	.	-	.	gene_id "lnc-bbug10707"	transcript_id "lnc-bbut00024628"	exon_number "4"
18	lncRNA	exon	50422907	50424791	.	-	.	gene_id "lnc-bbug10710"	transcript_id "lnc-bbut00024633"	exon_number "1"
18	lncRNA	exon	50425215	50425675	.	-	.	gene_id "lnc-bbug10710"	transcript_id "lnc-bbut00024633"	exon_number "2"
18	lncRNA	exon	50426148	50426410	.	-	.	gene_id "lnc-bbug10711"	transcript_id "lnc-bbut00024634"	exon_number "1"

18	lncRNA	exon	50449374	50449469	.	-	.	gene_id "lnc-bbug10711"	transcript_id "lnc-bbut00024634"	exon_number "2"
18	lncRNA	exon	50451575	50451707	.	-	.	gene_id "lnc-bbug10711"	transcript_id "lnc-bbut00024634"	exon_number "3"
18	lncRNA	exon	50441397	50442013	.	-	.	gene_id "lnc-bbug10711"	transcript_id "lnc-bbut00024635"	exon_number "1"
18	lncRNA	exon	50449374	50449469	.	-	.	gene_id "lnc-bbug10711"	transcript_id "lnc-bbut00024635"	exon_number "2"
18	lncRNA	exon	50451575	50451707	.	-	.	gene_id "lnc-bbug10711"	transcript_id "lnc-bbut00024635"	exon_number "3"
18	lncRNA	exon	50470937	50471649	.	-	.	gene_id "lnc-bbug10712"	transcript_id "lnc-bbut00024636"	exon_number "1"
18	lncRNA	exon	50471762	50471988	.	-	.	gene_id "lnc-bbug10712"	transcript_id "lnc-bbut00024636"	exon_number "2"
18	lncRNA	exon	50472124	50475600	.	-	.	gene_id "lnc-bbug10712"	transcript_id "lnc-bbut00024636"	exon_number "3"
18	lncRNA	exon	50470937	50471649	.	-	.	gene_id "lnc-bbug10712"	transcript_id "lnc-bbut00024637"	exon_number "1"
18	lncRNA	exon	50475529	50475621	.	-	.	gene_id "lnc-bbug10712"	transcript_id "lnc-bbut00024637"	exon_number "2"
18	lncRNA	exon	50478253	50478360	.	-	.	gene_id "lnc-bbug10712"	transcript_id "lnc-bbut00024637"	exon_number "3"
18	lncRNA	exon	50470937	50471649	.	-	.	gene_id "lnc-bbug10712"	transcript_id "lnc-bbut00024638"	exon_number "1"
18	lncRNA	exon	50475529	50475624	.	-	.	gene_id "lnc-bbug10712"	transcript_id "lnc-bbut00024638"	exon_number "2"
18	lncRNA	exon	50478253	50478369	.	-	.	gene_id "lnc-bbug10712"	transcript_id "lnc-bbut00024638"	exon_number "3"
18	lncRNA	exon	50517845	50519915	.	-	.	gene_id "lnc-bbug10713"	transcript_id "lnc-bbut00024640"	exon_number "1"
18	lncRNA	exon	50521178	50521220	.	-	.	gene_id "lnc-bbug10713"	transcript_id "lnc-bbut00024640"	exon_number "2"
18	lncRNA	exon	50523546	50523640	.	-	.	gene_id "lnc-bbug10713"	transcript_id "lnc-bbut00024640"	exon_number "3"
18	lncRNA	exon	50529290	50529786	.	-	.	gene_id "lnc-bbug10713"	transcript_id "lnc-bbut00024640"	exon_number "4"
18	lncRNA	exon	50517845	50519528	.	-	.	gene_id "lnc-bbug10713"	transcript_id "lnc-bbut00024639"	exon_number "1"
18	lncRNA	exon	50521178	50521220	.	-	.	gene_id "lnc-bbug10713"	transcript_id "lnc-bbut00024639"	exon_number "2"
18	lncRNA	exon	50523546	50523640	.	-	.	gene_id "lnc-bbug10713"	transcript_id "lnc-bbut00024639"	exon_number "3"
18	lncRNA	exon	50529290	50529786	.	-	.	gene_id "lnc-bbug10713"	transcript_id "lnc-bbut00024639"	exon_number "4"
18	lncRNA	exon	50517845	50519528	.	-	.	gene_id "lnc-bbug10713"	transcript_id "lnc-bbut00024641"	exon_number "1"
18	lncRNA	exon	50520477	50520538	.	-	.	gene_id "lnc-bbug10713"	transcript_id "lnc-bbut00024641"	exon_number "2"
18	lncRNA	exon	50521178	50521220	.	-	.	gene_id "lnc-bbug10713"	transcript_id "lnc-bbut00024641"	exon_number "3"
18	lncRNA	exon	50523546	50523640	.	-	.	gene_id "lnc-bbug10713"	transcript_id "lnc-bbut00024641"	exon_number "4"
18	lncRNA	exon	50529290	50529787	.	-	.	gene_id "lnc-bbug10713"	transcript_id "lnc-bbut00024641"	exon_number "5"
18	lncRNA	exon	50517845	50519528	.	-	.	gene_id "lnc-bbug10713"	transcript_id "lnc-bbut00024642"	exon_number "1"
18	lncRNA	exon	50520477	50520538	.	-	.	gene_id "lnc-bbug10713"	transcript_id "lnc-bbut00024642"	exon_number "2"
18	lncRNA	exon	50521178	50521220	.	-	.	gene_id "lnc-bbug10713"	transcript_id "lnc-bbut00024642"	exon_number "3"
18	lncRNA	exon	50523546	50523640	.	-	.	gene_id "lnc-bbug10713"	transcript_id "lnc-bbut00024642"	exon_number "4"
18	lncRNA	exon	50523742	50523847	.	-	.	gene_id "lnc-bbug10713"	transcript_id "lnc-bbut00024642"	exon_number "5"
18	lncRNA	exon	50529290	50529791	.	-	.	gene_id "lnc-bbug10713"	transcript_id "lnc-bbut00024642"	exon_number "6"
18	lncRNA	exon	50517845	50519528	.	-	.	gene_id "lnc-bbug10713"	transcript_id "lnc-bbut00024643"	exon_number "1"
18	lncRNA	exon	50520477	50520538	.	-	.	gene_id "lnc-bbug10713"	transcript_id "lnc-bbut00024643"	exon_number "2"
18	lncRNA	exon	50521178	50521220	.	-	.	gene_id "lnc-bbug10713"	transcript_id "lnc-bbut00024643"	exon_number "3"
18	lncRNA	exon	50523546	50523640	.	-	.	gene_id "lnc-bbug10713"	transcript_id "lnc-bbut00024643"	exon_number "4"
18	lncRNA	exon	50529036	50529792	.	-	.	gene_id "lnc-bbug10713"	transcript_id "lnc-bbut00024643"	exon_number "5"
18	lncRNA	exon	50517845	50519528	.	-	.	gene_id "lnc-bbug10713"	transcript_id "lnc-bbut00024644"	exon_number "1"
18	lncRNA	exon	50520477	50520538	.	-	.	gene_id "lnc-bbug10713"	transcript_id "lnc-bbut00024644"	exon_number "2"
18	lncRNA	exon	50521178	50521220	.	-	.	gene_id "lnc-bbug10713"	transcript_id "lnc-bbut00024644"	exon_number "3"
18	lncRNA	exon	50523546	50523640	.	-	.	gene_id "lnc-bbug10713"	transcript_id "lnc-bbut00024644"	exon_number "4"
18	lncRNA	exon	50523742	50523847	.	-	.	gene_id "lnc-bbug10713"	transcript_id "lnc-bbut00024644"	exon_number "5"
18	lncRNA	exon	50529036	50529794	.	-	.	gene_id "lnc-bbug10713"	transcript_id "lnc-bbut00024644"	exon_number "6"
18	lncRNA	exon	50530226	50530488	.	-	.	gene_id "lnc-bbug10714"	transcript_id "lnc-bbut00024645"	exon_number "1"
18	lncRNA	exon	50530913	50532116	.	-	.	gene_id "lnc-bbug10714"	transcript_id "lnc-bbut00024645"	exon_number "2"
18	lncRNA	exon	50551516	50551714	.	-	.	gene_id "lnc-bbug10714"	transcript_id "lnc-bbut00024645"	exon_number "3"
18	lncRNA	exon	50573385	50573667	.	-	.	gene_id "lnc-bbug10715"	transcript_id "lnc-bbut00024646"	exon_number "1"
18	lncRNA	exon	50576416	50576700	.	-	.	gene_id "lnc-bbug10715"	transcript_id "lnc-bbut00024646"	exon_number "2"
18	lncRNA	exon	50576127	50576304	.	-	.	gene_id "lnc-bbug10715"	transcript_id "lnc-bbut00024647"	exon_number "1"
18	lncRNA	exon	50576416	50576700	.	-	.	gene_id "lnc-bbug10715"	transcript_id "lnc-bbut00024647"	exon_number "2"
18	lncRNA	exon	50628467	50628517	.	-	.	gene_id "lnc-bbug10716"	transcript_id "lnc-bbut00024648"	exon_number "1"

18	lncRNA	exon	50631325	50631435	.	-	.	gene_id "lnc-bbug10716"	transcript_id "lnc-bbut00024648"	exon_number "2"
18	lncRNA	exon	50632157	50632488	.	-	.	gene_id "lnc-bbug10716"	transcript_id "lnc-bbut00024648"	exon_number "3"
18	lncRNA	exon	50787131	50787219	.	-	.	gene_id "lnc-bbug10717"	transcript_id "lnc-bbut00024649"	exon_number "1"
18	lncRNA	exon	50794474	50794525	.	-	.	gene_id "lnc-bbug10717"	transcript_id "lnc-bbut00024649"	exon_number "2"
18	lncRNA	exon	50795549	50795611	.	-	.	gene_id "lnc-bbug10717"	transcript_id "lnc-bbut00024649"	exon_number "3"
18	lncRNA	exon	50799395	50799441	.	-	.	gene_id "lnc-bbug10717"	transcript_id "lnc-bbut00024649"	exon_number "4"
18	lncRNA	exon	50823253	50823373	.	-	.	gene_id "lnc-bbug10717"	transcript_id "lnc-bbut00024649"	exon_number "5"
18	lncRNA	exon	50793491	50794525	.	-	.	gene_id "lnc-bbug10717"	transcript_id "lnc-bbut00024650"	exon_number "1"
18	lncRNA	exon	50795549	50795611	.	-	.	gene_id "lnc-bbug10717"	transcript_id "lnc-bbut00024650"	exon_number "2"
18	lncRNA	exon	50799395	50799441	.	-	.	gene_id "lnc-bbug10717"	transcript_id "lnc-bbut00024650"	exon_number "3"
18	lncRNA	exon	50823253	50823373	.	-	.	gene_id "lnc-bbug10717"	transcript_id "lnc-bbut00024650"	exon_number "4"
18	lncRNA	exon	50980089	50980199	.	-	.	gene_id "lnc-bbug10723"	transcript_id "lnc-bbut00024665"	exon_number "1"
18	lncRNA	exon	50981056	50981214	.	-	.	gene_id "lnc-bbug10723"	transcript_id "lnc-bbut00024665"	exon_number "2"
18	lncRNA	exon	50981267	50981339	.	-	.	gene_id "lnc-bbug10723"	transcript_id "lnc-bbut00024665"	exon_number "3"
18	lncRNA	exon	51041048	51041598	.	-	.	gene_id "lnc-bbug10724"	transcript_id "lnc-bbut00024666"	exon_number "1"
18	lncRNA	exon	51045846	51045984	.	-	.	gene_id "lnc-bbug10724"	transcript_id "lnc-bbut00024666"	exon_number "2"
18	lncRNA	exon	51046270	51046383	.	-	.	gene_id "lnc-bbug10724"	transcript_id "lnc-bbut00024666"	exon_number "3"
18	lncRNA	exon	51227904	51232949	.	-	.	gene_id "lnc-bbug10726"	transcript_id "lnc-bbut00024674"	exon_number "1"
18	lncRNA	exon	51240283	51242455	.	-	.	gene_id "lnc-bbug10726"	transcript_id "lnc-bbut00024674"	exon_number "2"
18	lncRNA	exon	51355106	51355627	.	-	.	gene_id "lnc-bbug10733"	transcript_id "lnc-bbut00024688"	exon_number "1"
18	lncRNA	exon	51356503	51356781	.	-	.	gene_id "lnc-bbug10733"	transcript_id "lnc-bbut00024688"	exon_number "2"
18	lncRNA	exon	51357971	51358331	.	-	.	gene_id "lnc-bbug10733"	transcript_id "lnc-bbut00024688"	exon_number "3"
18	lncRNA	exon	51359232	51359498	.	-	.	gene_id "lnc-bbug10733"	transcript_id "lnc-bbut00024688"	exon_number "4"
18	lncRNA	exon	51420841	51422010	.	-	.	gene_id "lnc-bbug10736"	transcript_id "lnc-bbut00024693"	exon_number "1"
18	lncRNA	exon	51439117	51440508	.	-	.	gene_id "lnc-bbug10737"	transcript_id "lnc-bbut00024694"	exon_number "1"
18	lncRNA	exon	51497955	51498613	.	-	.	gene_id "lnc-bbug10740"	transcript_id "lnc-bbut00024698"	exon_number "1"
18	lncRNA	exon	51503636	51503744	.	-	.	gene_id "lnc-bbug10740"	transcript_id "lnc-bbut00024698"	exon_number "2"
18	lncRNA	exon	51519534	51519751	.	-	.	gene_id "lnc-bbug10740"	transcript_id "lnc-bbut00024698"	exon_number "3"
18	lncRNA	exon	51917615	51917666	.	-	.	gene_id "lnc-bbug10750"	transcript_id "lnc-bbut00024718"	exon_number "1"
18	lncRNA	exon	51917757	51917924	.	-	.	gene_id "lnc-bbug10750"	transcript_id "lnc-bbut00024718"	exon_number "2"
18	lncRNA	exon	51918617	51918811	.	-	.	gene_id "lnc-bbug10750"	transcript_id "lnc-bbut00024718"	exon_number "3"
18	lncRNA	exon	52242148	52242452	.	-	.	gene_id "lnc-bbug10756"	transcript_id "lnc-bbut00024729"	exon_number "1"
18	lncRNA	exon	52245524	52245853	.	-	.	gene_id "lnc-bbug10756"	transcript_id "lnc-bbut00024729"	exon_number "2"
18	lncRNA	exon	52246159	52247045	.	-	.	gene_id "lnc-bbug10756"	transcript_id "lnc-bbut00024729"	exon_number "3"
18	lncRNA	exon	52293851	52294362	.	-	.	gene_id "lnc-bbug10758"	transcript_id "lnc-bbut00024731"	exon_number "1"
18	lncRNA	exon	52294993	52295119	.	-	.	gene_id "lnc-bbug10758"	transcript_id "lnc-bbut00024731"	exon_number "2"
18	lncRNA	exon	52295786	52295843	.	-	.	gene_id "lnc-bbug10758"	transcript_id "lnc-bbut00024731"	exon_number "3"
18	lncRNA	exon	52320170	52320244	.	-	.	gene_id "lnc-bbug10758"	transcript_id "lnc-bbut00024731"	exon_number "4"
18	lncRNA	exon	52323788	52323961	.	-	.	gene_id "lnc-bbug10758"	transcript_id "lnc-bbut00024731"	exon_number "5"
18	lncRNA	exon	52328940	52329029	.	-	.	gene_id "lnc-bbug10758"	transcript_id "lnc-bbut00024731"	exon_number "6"
18	lncRNA	exon	52330470	52330506	.	-	.	gene_id "lnc-bbug10758"	transcript_id "lnc-bbut00024731"	exon_number "7"
18	lncRNA	exon	52297949	52300896	.	-	.	gene_id "lnc-bbug10759"	transcript_id "lnc-bbut00024732"	exon_number "1"
18	lncRNA	exon	52301147	52301590	.	-	.	gene_id "lnc-bbug10759"	transcript_id "lnc-bbut00024732"	exon_number "2"
18	lncRNA	exon	52333253	52334187	.	-	.	gene_id "lnc-bbug10760"	transcript_id "lnc-bbut00024736"	exon_number "1"
18	lncRNA	exon	52507940	52508249	.	-	.	gene_id "lnc-bbug10764"	transcript_id "lnc-bbut00024740"	exon_number "1"
18	lncRNA	exon	52510982	52511292	.	-	.	gene_id "lnc-bbug10764"	transcript_id "lnc-bbut00024740"	exon_number "2"
18	lncRNA	exon	52521325	52521804	.	-	.	gene_id "lnc-bbug10765"	transcript_id "lnc-bbut00024741"	exon_number "1"
18	lncRNA	exon	52522432	52522644	.	-	.	gene_id "lnc-bbug10765"	transcript_id "lnc-bbut00024741"	exon_number "2"
18	lncRNA	exon	52662149	52662424	.	-	.	gene_id "lnc-bbug10766"	transcript_id "lnc-bbut00024742"	exon_number "1"
18	lncRNA	exon	52662981	52663451	.	-	.	gene_id "lnc-bbug10766"	transcript_id "lnc-bbut00024742"	exon_number "2"
18	lncRNA	exon	52667165	52667207	.	-	.	gene_id "lnc-bbug10766"	transcript_id "lnc-bbut00024742"	exon_number "3"
18	lncRNA	exon	52698784	52698987	.	-	.	gene_id "lnc-bbug10767"	transcript_id "lnc-bbut00024743"	exon_number "1"

18	lncRNA	exon	52699862	52701745	.	-	.	gene_id "lnc-bbug10767"	transcript_id "lnc-bbut00024743"	exon_number "2"
18	lncRNA	exon	52846967	52847058	.	-	.	gene_id "lnc-bbug10770"	transcript_id "lnc-bbut00024758"	exon_number "1"
18	lncRNA	exon	52849117	52849381	.	-	.	gene_id "lnc-bbug10770"	transcript_id "lnc-bbut00024758"	exon_number "2"
18	lncRNA	exon	53431733	53435977	.	-	.	gene_id "lnc-bbug10789"	transcript_id "lnc-bbut00024800"	exon_number "1"
18	lncRNA	exon	53567123	53567357	.	-	.	gene_id "lnc-bbug10793"	transcript_id "lnc-bbut00024804"	exon_number "1"
18	lncRNA	exon	53567549	53567604	.	-	.	gene_id "lnc-bbug10793"	transcript_id "lnc-bbut00024804"	exon_number "2"
18	lncRNA	exon	53569263	53569368	.	-	.	gene_id "lnc-bbug10793"	transcript_id "lnc-bbut00024804"	exon_number "3"
18	lncRNA	exon	53689573	53690246	.	-	.	gene_id "lnc-bbug10794"	transcript_id "lnc-bbut00024805"	exon_number "1"
18	lncRNA	exon	53690873	53691629	.	-	.	gene_id "lnc-bbug10794"	transcript_id "lnc-bbut00024805"	exon_number "2"
18	lncRNA	exon	53696874	53698611	.	-	.	gene_id "lnc-bbug10795"	transcript_id "lnc-bbut00024806"	exon_number "1"
18	lncRNA	exon	53698756	53700699	.	-	.	gene_id "lnc-bbug10795"	transcript_id "lnc-bbut00024806"	exon_number "2"
18	lncRNA	exon	53763131	53763385	.	-	.	gene_id "lnc-bbug10797"	transcript_id "lnc-bbut00024810"	exon_number "1"
18	lncRNA	exon	53763758	53763822	.	-	.	gene_id "lnc-bbug10797"	transcript_id "lnc-bbut00024810"	exon_number "2"
18	lncRNA	exon	53765029	53766439	.	-	.	gene_id "lnc-bbug10797"	transcript_id "lnc-bbut00024810"	exon_number "3"
18	lncRNA	exon	53763131	53763385	.	-	.	gene_id "lnc-bbug10797"	transcript_id "lnc-bbut00024809"	exon_number "1"
18	lncRNA	exon	53763737	53763822	.	-	.	gene_id "lnc-bbug10797"	transcript_id "lnc-bbut00024809"	exon_number "2"
18	lncRNA	exon	53765029	53766439	.	-	.	gene_id "lnc-bbug10797"	transcript_id "lnc-bbut00024809"	exon_number "3"
18	lncRNA	exon	54790361	54790470	.	-	.	gene_id "lnc-bbug10815"	transcript_id "lnc-bbut00024855"	exon_number "1"
18	lncRNA	exon	54792575	54792722	.	-	.	gene_id "lnc-bbug10815"	transcript_id "lnc-bbut00024855"	exon_number "2"
18	lncRNA	exon	54793182	54793264	.	-	.	gene_id "lnc-bbug10815"	transcript_id "lnc-bbut00024855"	exon_number "3"
18	lncRNA	exon	54794891	54794923	.	-	.	gene_id "lnc-bbug10815"	transcript_id "lnc-bbut00024855"	exon_number "4"
18	lncRNA	exon	54800744	54800905	.	-	.	gene_id "lnc-bbug10815"	transcript_id "lnc-bbut00024855"	exon_number "5"
18	lncRNA	exon	54816459	54816718	.	-	.	gene_id "lnc-bbug10816"	transcript_id "lnc-bbut00024858"	exon_number "1"
18	lncRNA	exon	54817119	54817250	.	-	.	gene_id "lnc-bbug10816"	transcript_id "lnc-bbut00024858"	exon_number "2"
18	lncRNA	exon	54818041	54818070	.	-	.	gene_id "lnc-bbug10816"	transcript_id "lnc-bbut00024858"	exon_number "3"
18	lncRNA	exon	54819346	54819366	.	-	.	gene_id "lnc-bbug10816"	transcript_id "lnc-bbut00024858"	exon_number "4"
18	lncRNA	exon	54824758	54825065	.	-	.	gene_id "lnc-bbug10816"	transcript_id "lnc-bbut00024858"	exon_number "5"
18	lncRNA	exon	55356919	55357980	.	-	.	gene_id "lnc-bbug10830"	transcript_id "lnc-bbut00024883"	exon_number "1"
18	lncRNA	exon	55358277	55358326	.	-	.	gene_id "lnc-bbug10830"	transcript_id "lnc-bbut00024883"	exon_number "2"
18	lncRNA	exon	55637224	55637787	.	-	.	gene_id "lnc-bbug10844"	transcript_id "lnc-bbut00024917"	exon_number "1"
18	lncRNA	exon	56080999	56082508	.	-	.	gene_id "lnc-bbug10856"	transcript_id "lnc-bbut00024948"	exon_number "1"
18	lncRNA	exon	56082701	56082794	.	-	.	gene_id "lnc-bbug10856"	transcript_id "lnc-bbut00024948"	exon_number "2"
18	lncRNA	exon	56084352	56085040	.	-	.	gene_id "lnc-bbug10856"	transcript_id "lnc-bbut00024948"	exon_number "3"
18	lncRNA	exon	56080999	56082508	.	-	.	gene_id "lnc-bbug10856"	transcript_id "lnc-bbut00024949"	exon_number "1"
18	lncRNA	exon	56082701	56082794	.	-	.	gene_id "lnc-bbug10856"	transcript_id "lnc-bbut00024949"	exon_number "2"
18	lncRNA	exon	56084562	56085040	.	-	.	gene_id "lnc-bbug10856"	transcript_id "lnc-bbut00024949"	exon_number "3"
18	lncRNA	exon	56080999	56081755	.	-	.	gene_id "lnc-bbug10856"	transcript_id "lnc-bbut00024947"	exon_number "1"
18	lncRNA	exon	56082342	56082508	.	-	.	gene_id "lnc-bbug10856"	transcript_id "lnc-bbut00024947"	exon_number "2"
18	lncRNA	exon	56082701	56085040	.	-	.	gene_id "lnc-bbug10856"	transcript_id "lnc-bbut00024947"	exon_number "3"
18	lncRNA	exon	56080999	56081365	.	-	.	gene_id "lnc-bbug10856"	transcript_id "lnc-bbut00024946"	exon_number "1"
18	lncRNA	exon	56081492	56082508	.	-	.	gene_id "lnc-bbug10856"	transcript_id "lnc-bbut00024946"	exon_number "2"
18	lncRNA	exon	56082701	56085040	.	-	.	gene_id "lnc-bbug10856"	transcript_id "lnc-bbut00024946"	exon_number "3"
18	lncRNA	exon	56118099	56118631	.	-	.	gene_id "lnc-bbug10857"	transcript_id "lnc-bbut00024950"	exon_number "1"
18	lncRNA	exon	56286895	56287334	.	-	.	gene_id "lnc-bbug10864"	transcript_id "lnc-bbut00024976"	exon_number "1"
18	lncRNA	exon	56288697	56288770	.	-	.	gene_id "lnc-bbug10864"	transcript_id "lnc-bbut00024976"	exon_number "2"
18	lncRNA	exon	56289707	56289967	.	-	.	gene_id "lnc-bbug10864"	transcript_id "lnc-bbut00024976"	exon_number "3"
18	lncRNA	exon	56344856	56345605	.	-	.	gene_id "lnc-bbug10866"	transcript_id "lnc-bbut00024980"	exon_number "1"
18	lncRNA	exon	56347624	56348078	.	-	.	gene_id "lnc-bbug10866"	transcript_id "lnc-bbut00024980"	exon_number "2"
18	lncRNA	exon	56417482	56418298	.	-	.	gene_id "lnc-bbug10869"	transcript_id "lnc-bbut00024984"	exon_number "1"
18	lncRNA	exon	56418695	56418773	.	-	.	gene_id "lnc-bbug10869"	transcript_id "lnc-bbut00024984"	exon_number "2"
18	lncRNA	exon	56419130	56419193	.	-	.	gene_id "lnc-bbug10869"	transcript_id "lnc-bbut00024984"	exon_number "3"
18	lncRNA	exon	56420407	56420687	.	-	.	gene_id "lnc-bbug10869"	transcript_id "lnc-bbut00024984"	exon_number "4"

18	lncRNA	exon	56417482	56418298	.	-	.	gene_id "lnc-bbug10869"	transcript_id "lnc-bbut00024983"	exon_number "1"
18	lncRNA	exon	56418695	56418773	.	-	.	gene_id "lnc-bbug10869"	transcript_id "lnc-bbut00024983"	exon_number "2"
18	lncRNA	exon	56420407	56420687	.	-	.	gene_id "lnc-bbug10869"	transcript_id "lnc-bbut00024983"	exon_number "3"
18	lncRNA	exon	56629830	56630691	.	-	.	gene_id "lnc-bbug10873"	transcript_id "lnc-bbut00024999"	exon_number "1"
18	lncRNA	exon	56630834	56631900	.	-	.	gene_id "lnc-bbug10873"	transcript_id "lnc-bbut00024999"	exon_number "2"
18	lncRNA	exon	56854533	56854635	.	-	.	gene_id "lnc-bbug10879"	transcript_id "lnc-bbut00025012"	exon_number "1"
18	lncRNA	exon	56854760	56854819	.	-	.	gene_id "lnc-bbug10879"	transcript_id "lnc-bbut00025012"	exon_number "2"
18	lncRNA	exon	56854916	56855053	.	-	.	gene_id "lnc-bbug10879"	transcript_id "lnc-bbut00025012"	exon_number "3"
18	lncRNA	exon	57010288	57010741	.	-	.	gene_id "lnc-bbug10892"	transcript_id "lnc-bbut00025041"	exon_number "1"
18	lncRNA	exon	57011629	57011762	.	-	.	gene_id "lnc-bbug10892"	transcript_id "lnc-bbut00025041"	exon_number "2"
18	lncRNA	exon	57013761	57013920	.	-	.	gene_id "lnc-bbug10892"	transcript_id "lnc-bbut00025041"	exon_number "3"
18	lncRNA	exon	57014517	57014572	.	-	.	gene_id "lnc-bbug10892"	transcript_id "lnc-bbut00025041"	exon_number "4"
18	lncRNA	exon	57020069	57020235	.	-	.	gene_id "lnc-bbug10892"	transcript_id "lnc-bbut00025041"	exon_number "5"
18	lncRNA	exon	57615945	57616552	.	-	.	gene_id "lnc-bbug10909"	transcript_id "lnc-bbut00025096"	exon_number "1"
18	lncRNA	exon	57617508	57617555	.	-	.	gene_id "lnc-bbug10909"	transcript_id "lnc-bbut00025096"	exon_number "2"
18	lncRNA	exon	57617793	57617977	.	-	.	gene_id "lnc-bbug10909"	transcript_id "lnc-bbut00025096"	exon_number "3"
18	lncRNA	exon	57618464	57618884	.	-	.	gene_id "lnc-bbug10909"	transcript_id "lnc-bbut00025096"	exon_number "4"
18	lncRNA	exon	57634748	57635355	.	-	.	gene_id "lnc-bbug10911"	transcript_id "lnc-bbut00025100"	exon_number "1"
18	lncRNA	exon	57636311	57636358	.	-	.	gene_id "lnc-bbug10911"	transcript_id "lnc-bbut00025100"	exon_number "2"
18	lncRNA	exon	57636599	57636783	.	-	.	gene_id "lnc-bbug10911"	transcript_id "lnc-bbut00025100"	exon_number "3"
18	lncRNA	exon	57637270	57637690	.	-	.	gene_id "lnc-bbug10911"	transcript_id "lnc-bbut00025100"	exon_number "4"
18	lncRNA	exon	57752002	57755310	.	-	.	gene_id "lnc-bbug10915"	transcript_id "lnc-bbut00025106"	exon_number "1"
18	lncRNA	exon	57757972	57758161	.	-	.	gene_id "lnc-bbug10915"	transcript_id "lnc-bbut00025106"	exon_number "2"
18	lncRNA	exon	58329208	58329506	.	-	.	gene_id "lnc-bbug10919"	transcript_id "lnc-bbut00025110"	exon_number "1"
18	lncRNA	exon	58332871	58332962	.	-	.	gene_id "lnc-bbug10919"	transcript_id "lnc-bbut00025110"	exon_number "2"
18	lncRNA	exon	58335237	58335411	.	-	.	gene_id "lnc-bbug10919"	transcript_id "lnc-bbut00025110"	exon_number "3"
18	lncRNA	exon	58407746	58408040	.	-	.	gene_id "lnc-bbug10920"	transcript_id "lnc-bbut00025111"	exon_number "1"
18	lncRNA	exon	58410722	58410798	.	-	.	gene_id "lnc-bbug10920"	transcript_id "lnc-bbut00025111"	exon_number "2"
18	lncRNA	exon	58411660	58411829	.	-	.	gene_id "lnc-bbug10920"	transcript_id "lnc-bbut00025111"	exon_number "3"
18	lncRNA	exon	58412473	58413955	.	-	.	gene_id "lnc-bbug10920"	transcript_id "lnc-bbut00025111"	exon_number "4"
18	lncRNA	exon	58673051	58673669	.	-	.	gene_id "lnc-bbug10924"	transcript_id "lnc-bbut00025115"	exon_number "1"
18	lncRNA	exon	58710703	58711033	.	-	.	gene_id "lnc-bbug10925"	transcript_id "lnc-bbut00025116"	exon_number "1"
18	lncRNA	exon	58721398	58721514	.	-	.	gene_id "lnc-bbug10925"	transcript_id "lnc-bbut00025116"	exon_number "2"
18	lncRNA	exon	58725757	58725833	.	-	.	gene_id "lnc-bbug10925"	transcript_id "lnc-bbut00025116"	exon_number "3"
18	lncRNA	exon	58730015	58730114	.	-	.	gene_id "lnc-bbug10925"	transcript_id "lnc-bbut00025116"	exon_number "4"
18	lncRNA	exon	58773589	58776865	.	-	.	gene_id "lnc-bbug10926"	transcript_id "lnc-bbut00025117"	exon_number "1"
18	lncRNA	exon	58811250	58811289	.	-	.	gene_id "lnc-bbug10927"	transcript_id "lnc-bbut00025119"	exon_number "1"
18	lncRNA	exon	58824105	58824181	.	-	.	gene_id "lnc-bbug10927"	transcript_id "lnc-bbut00025119"	exon_number "2"
18	lncRNA	exon	58827925	58827998	.	-	.	gene_id "lnc-bbug10927"	transcript_id "lnc-bbut00025119"	exon_number "3"
18	lncRNA	exon	58828615	58829027	.	-	.	gene_id "lnc-bbug10927"	transcript_id "lnc-bbut00025119"	exon_number "4"
18	lncRNA	exon	58811250	58811289	.	-	.	gene_id "lnc-bbug10927"	transcript_id "lnc-bbut00025118"	exon_number "1"
18	lncRNA	exon	58824105	58824181	.	-	.	gene_id "lnc-bbug10927"	transcript_id "lnc-bbut00025118"	exon_number "2"
18	lncRNA	exon	58825913	58825998	.	-	.	gene_id "lnc-bbug10927"	transcript_id "lnc-bbut00025118"	exon_number "3"
18	lncRNA	exon	58828615	58829027	.	-	.	gene_id "lnc-bbug10927"	transcript_id "lnc-bbut00025118"	exon_number "4"
18	lncRNA	exon	58818850	58819921	.	-	.	gene_id "lnc-bbug10927"	transcript_id "lnc-bbut00025125"	exon_number "1"
18	lncRNA	exon	58824105	58824181	.	-	.	gene_id "lnc-bbug10927"	transcript_id "lnc-bbut00025125"	exon_number "2"
18	lncRNA	exon	58825913	58825998	.	-	.	gene_id "lnc-bbug10927"	transcript_id "lnc-bbut00025125"	exon_number "3"
18	lncRNA	exon	58827925	58827998	.	-	.	gene_id "lnc-bbug10927"	transcript_id "lnc-bbut00025125"	exon_number "4"
18	lncRNA	exon	58828615	58829027	.	-	.	gene_id "lnc-bbug10927"	transcript_id "lnc-bbut00025125"	exon_number "5"
18	lncRNA	exon	58818850	58819921	.	-	.	gene_id "lnc-bbug10927"	transcript_id "lnc-bbut00025124"	exon_number "1"
18	lncRNA	exon	58824105	58824181	.	-	.	gene_id "lnc-bbug10927"	transcript_id "lnc-bbut00025124"	exon_number "2"
18	lncRNA	exon	58827925	58827998	.	-	.	gene_id "lnc-bbug10927"	transcript_id "lnc-bbut00025124"	exon_number "3"

18	lncRNA	exon	58828615	58829027	.	-	.	gene_id "lnc-bbug10927"	transcript_id "lnc-bbut00025124"	exon_number "4"
18	lncRNA	exon	58823588	58824181	.	-	.	gene_id "lnc-bbug10927"	transcript_id "lnc-bbut00025126"	exon_number "1"
18	lncRNA	exon	58825913	58825998	.	-	.	gene_id "lnc-bbug10927"	transcript_id "lnc-bbut00025126"	exon_number "2"
18	lncRNA	exon	58828615	58829027	.	-	.	gene_id "lnc-bbug10927"	transcript_id "lnc-bbut00025126"	exon_number "3"
18	lncRNA	exon	59072500	59072845	.	-	.	gene_id "lnc-bbug10933"	transcript_id "lnc-bbut00025141"	exon_number "1"
18	lncRNA	exon	59073666	59075288	.	-	.	gene_id "lnc-bbug10933"	transcript_id "lnc-bbut00025141"	exon_number "2"
18	lncRNA	exon	59408853	59409143	.	-	.	gene_id "lnc-bbug10940"	transcript_id "lnc-bbut00025169"	exon_number "1"
18	lncRNA	exon	59411393	59411452	.	-	.	gene_id "lnc-bbug10940"	transcript_id "lnc-bbut00025169"	exon_number "2"
18	lncRNA	exon	59415512	59415629	.	-	.	gene_id "lnc-bbug10940"	transcript_id "lnc-bbut00025169"	exon_number "3"
18	lncRNA	exon	59415717	59416915	.	-	.	gene_id "lnc-bbug10940"	transcript_id "lnc-bbut00025169"	exon_number "4"
18	lncRNA	exon	59459831	59460721	.	-	.	gene_id "lnc-bbug10942"	transcript_id "lnc-bbut00025172"	exon_number "1"
18	lncRNA	exon	59465121	59465242	.	-	.	gene_id "lnc-bbug10942"	transcript_id "lnc-bbut00025172"	exon_number "2"
18	lncRNA	exon	59470468	59470551	.	-	.	gene_id "lnc-bbug10942"	transcript_id "lnc-bbut00025172"	exon_number "3"
18	lncRNA	exon	59471241	59471593	.	-	.	gene_id "lnc-bbug10942"	transcript_id "lnc-bbut00025172"	exon_number "4"
18	lncRNA	exon	59459831	59460721	.	-	.	gene_id "lnc-bbug10942"	transcript_id "lnc-bbut00025173"	exon_number "1"
18	lncRNA	exon	59465121	59465242	.	-	.	gene_id "lnc-bbug10942"	transcript_id "lnc-bbut00025173"	exon_number "2"
18	lncRNA	exon	59470468	59470551	.	-	.	gene_id "lnc-bbug10942"	transcript_id "lnc-bbut00025173"	exon_number "3"
18	lncRNA	exon	59471602	59472235	.	-	.	gene_id "lnc-bbug10942"	transcript_id "lnc-bbut00025173"	exon_number "4"
18	lncRNA	exon	59545994	59546044	.	-	.	gene_id "lnc-bbug10946"	transcript_id "lnc-bbut00025177"	exon_number "1"
18	lncRNA	exon	59551026	59551108	.	-	.	gene_id "lnc-bbug10946"	transcript_id "lnc-bbut00025177"	exon_number "2"
18	lncRNA	exon	59556463	59556521	.	-	.	gene_id "lnc-bbug10946"	transcript_id "lnc-bbut00025177"	exon_number "3"
18	lncRNA	exon	59558693	59558818	.	-	.	gene_id "lnc-bbug10946"	transcript_id "lnc-bbut00025177"	exon_number "4"
18	lncRNA	exon	59548813	59550930	.	-	.	gene_id "lnc-bbug10946"	transcript_id "lnc-bbut00025178"	exon_number "1"
18	lncRNA	exon	59551026	59551108	.	-	.	gene_id "lnc-bbug10946"	transcript_id "lnc-bbut00025178"	exon_number "2"
18	lncRNA	exon	59556463	59556521	.	-	.	gene_id "lnc-bbug10946"	transcript_id "lnc-bbut00025178"	exon_number "3"
18	lncRNA	exon	59558693	59558818	.	-	.	gene_id "lnc-bbug10946"	transcript_id "lnc-bbut00025178"	exon_number "4"
18	lncRNA	exon	59594526	59594555	.	-	.	gene_id "lnc-bbug10947"	transcript_id "lnc-bbut00025179"	exon_number "1"
18	lncRNA	exon	59608924	59609227	.	-	.	gene_id "lnc-bbug10947"	transcript_id "lnc-bbut00025179"	exon_number "2"
18	lncRNA	exon	59629007	59629235	.	-	.	gene_id "lnc-bbug10949"	transcript_id "lnc-bbut00025181"	exon_number "1"
18	lncRNA	exon	59638697	59638780	.	-	.	gene_id "lnc-bbug10949"	transcript_id "lnc-bbut00025181"	exon_number "2"
18	lncRNA	exon	59639189	59639780	.	-	.	gene_id "lnc-bbug10949"	transcript_id "lnc-bbut00025181"	exon_number "3"
18	lncRNA	exon	59811097	59812170	.	-	.	gene_id "lnc-bbug10953"	transcript_id "lnc-bbut00025192"	exon_number "1"
18	lncRNA	exon	59813424	59813550	.	-	.	gene_id "lnc-bbug10953"	transcript_id "lnc-bbut00025192"	exon_number "2"
18	lncRNA	exon	59817330	59817415	.	-	.	gene_id "lnc-bbug10953"	transcript_id "lnc-bbut00025192"	exon_number "3"
18	lncRNA	exon	59832959	59833085	.	-	.	gene_id "lnc-bbug10953"	transcript_id "lnc-bbut00025192"	exon_number "4"
18	lncRNA	exon	59839080	59839169	.	-	.	gene_id "lnc-bbug10953"	transcript_id "lnc-bbut00025192"	exon_number "5"
18	lncRNA	exon	59839254	59842914	.	-	.	gene_id "lnc-bbug10953"	transcript_id "lnc-bbut00025192"	exon_number "6"
18	lncRNA	exon	60136239	60137719	.	-	.	gene_id "lnc-bbug10957"	transcript_id "lnc-bbut00025201"	exon_number "1"
18	lncRNA	exon	60138346	60138393	.	-	.	gene_id "lnc-bbug10957"	transcript_id "lnc-bbut00025201"	exon_number "2"
18	lncRNA	exon	60179925	60181989	.	-	.	gene_id "lnc-bbug10960"	transcript_id "lnc-bbut00025204"	exon_number "1"
18	lncRNA	exon	60365762	60365862	.	-	.	gene_id "lnc-bbug10964"	transcript_id "lnc-bbut00025217"	exon_number "1"
18	lncRNA	exon	60366085	60366175	.	-	.	gene_id "lnc-bbug10964"	transcript_id "lnc-bbut00025217"	exon_number "2"
18	lncRNA	exon	60369095	60370555	.	-	.	gene_id "lnc-bbug10964"	transcript_id "lnc-bbut00025217"	exon_number "3"
18	lncRNA	exon	60365762	60366175	.	-	.	gene_id "lnc-bbug10964"	transcript_id "lnc-bbut00025216"	exon_number "1"
18	lncRNA	exon	60369095	60370555	.	-	.	gene_id "lnc-bbug10964"	transcript_id "lnc-bbut00025216"	exon_number "2"
18	lncRNA	exon	61482606	61483047	.	-	.	gene_id "lnc-bbug10980"	transcript_id "lnc-bbut00025253"	exon_number "1"
18	lncRNA	exon	61491139	61491501	.	-	.	gene_id "lnc-bbug10980"	transcript_id "lnc-bbut00025253"	exon_number "2"
18	lncRNA	exon	61527728	61528304	.	-	.	gene_id "lnc-bbug10981"	transcript_id "lnc-bbut00025254"	exon_number "1"
18	lncRNA	exon	61544037	61544132	.	-	.	gene_id "lnc-bbug10981"	transcript_id "lnc-bbut00025254"	exon_number "2"
18	lncRNA	exon	61544283	61544738	.	-	.	gene_id "lnc-bbug10981"	transcript_id "lnc-bbut00025254"	exon_number "3"
18	lncRNA	exon	61531742	61533090	.	-	.	gene_id "lnc-bbug10981"	transcript_id "lnc-bbut00025258"	exon_number "1"
18	lncRNA	exon	61544037	61544132	.	-	.	gene_id "lnc-bbug10981"	transcript_id "lnc-bbut00025258"	exon_number "2"

18	lncRNA	exon	61547211	61547337	.	-	.	gene_id "lnc-bbug10981"	transcript_id "lnc-bbut00025258"	exon_number "3"
18	lncRNA	exon	61542401	61543496	.	-	.	gene_id "lnc-bbug10981"	transcript_id "lnc-bbut00025260"	exon_number "1"
18	lncRNA	exon	61544037	61544132	.	-	.	gene_id "lnc-bbug10981"	transcript_id "lnc-bbut00025260"	exon_number "2"
18	lncRNA	exon	61544363	61544478	.	-	.	gene_id "lnc-bbug10981"	transcript_id "lnc-bbut00025260"	exon_number "3"
18	lncRNA	exon	61542401	61543496	.	-	.	gene_id "lnc-bbug10981"	transcript_id "lnc-bbut00025263"	exon_number "1"
18	lncRNA	exon	61544037	61544132	.	-	.	gene_id "lnc-bbug10981"	transcript_id "lnc-bbut00025263"	exon_number "2"
18	lncRNA	exon	61547211	61547337	.	-	.	gene_id "lnc-bbug10981"	transcript_id "lnc-bbut00025263"	exon_number "3"
18	lncRNA	exon	61542401	61543796	.	-	.	gene_id "lnc-bbug10981"	transcript_id "lnc-bbut00025262"	exon_number "1"
18	lncRNA	exon	61544037	61544132	.	-	.	gene_id "lnc-bbug10981"	transcript_id "lnc-bbut00025262"	exon_number "2"
18	lncRNA	exon	61547211	61547337	.	-	.	gene_id "lnc-bbug10981"	transcript_id "lnc-bbut00025262"	exon_number "3"
18	lncRNA	exon	61862211	61862924	.	-	.	gene_id "lnc-bbug10987"	transcript_id "lnc-bbut00025276"	exon_number "1"
18	lncRNA	exon	61864276	61865180	.	-	.	gene_id "lnc-bbug10987"	transcript_id "lnc-bbut00025276"	exon_number "2"
18	lncRNA	exon	61865260	61865355	.	-	.	gene_id "lnc-bbug10987"	transcript_id "lnc-bbut00025276"	exon_number "3"
18	lncRNA	exon	61870681	61870782	.	-	.	gene_id "lnc-bbug10987"	transcript_id "lnc-bbut00025276"	exon_number "4"
18	lncRNA	exon	61871137	61872920	.	-	.	gene_id "lnc-bbug10987"	transcript_id "lnc-bbut00025276"	exon_number "5"
18	lncRNA	exon	62398177	62399064	.	-	.	gene_id "lnc-bbug11001"	transcript_id "lnc-bbut00025299"	exon_number "1"
18	lncRNA	exon	62444770	62444819	.	-	.	gene_id "lnc-bbug11004"	transcript_id "lnc-bbut00025305"	exon_number "1"
18	lncRNA	exon	62446516	62447021	.	-	.	gene_id "lnc-bbug11004"	transcript_id "lnc-bbut00025305"	exon_number "2"
18	lncRNA	exon	62694312	62694436	.	-	.	gene_id "lnc-bbug11017"	transcript_id "lnc-bbut00025333"	exon_number "1"
18	lncRNA	exon	62697803	62698099	.	-	.	gene_id "lnc-bbug11017"	transcript_id "lnc-bbut00025333"	exon_number "2"
18	lncRNA	exon	62696493	62696572	.	-	.	gene_id "lnc-bbug11017"	transcript_id "lnc-bbut00025334"	exon_number "1"
18	lncRNA	exon	62697803	62698095	.	-	.	gene_id "lnc-bbug11017"	transcript_id "lnc-bbut00025334"	exon_number "2"
18	lncRNA	exon	63094816	63095655	.	-	.	gene_id "lnc-bbug11027"	transcript_id "lnc-bbut00025356"	exon_number "1"
18	lncRNA	exon	63096911	63097314	.	-	.	gene_id "lnc-bbug11027"	transcript_id "lnc-bbut00025356"	exon_number "2"
18	lncRNA	exon	63182916	63184001	.	-	.	gene_id "lnc-bbug11032"	transcript_id "lnc-bbut00025365"	exon_number "1"
18	lncRNA	exon	63184428	63185291	.	-	.	gene_id "lnc-bbug11032"	transcript_id "lnc-bbut00025365"	exon_number "2"
18	lncRNA	exon	63185412	63185695	.	-	.	gene_id "lnc-bbug11032"	transcript_id "lnc-bbut00025365"	exon_number "3"
18	lncRNA	exon	63186560	63186712	.	-	.	gene_id "lnc-bbug11033"	transcript_id "lnc-bbut00025366"	exon_number "1"
18	lncRNA	exon	63187600	63187677	.	-	.	gene_id "lnc-bbug11033"	transcript_id "lnc-bbut00025366"	exon_number "2"
18	lncRNA	exon	63189498	63189572	.	-	.	gene_id "lnc-bbug11033"	transcript_id "lnc-bbut00025366"	exon_number "3"
18	lncRNA	exon	63189792	63190215	.	-	.	gene_id "lnc-bbug11033"	transcript_id "lnc-bbut00025366"	exon_number "4"
18	lncRNA	exon	63191986	63192251	.	-	.	gene_id "lnc-bbug11033"	transcript_id "lnc-bbut00025366"	exon_number "5"
18	lncRNA	exon	63358712	63359716	.	-	.	gene_id "lnc-bbug11035"	transcript_id "lnc-bbut00025368"	exon_number "1"
18	lncRNA	exon	63359869	63360010	.	-	.	gene_id "lnc-bbug11035"	transcript_id "lnc-bbut00025368"	exon_number "2"
18	lncRNA	exon	63434203	63434265	.	-	.	gene_id "lnc-bbug11038"	transcript_id "lnc-bbut00025373"	exon_number "1"
18	lncRNA	exon	63434298	63434410	.	-	.	gene_id "lnc-bbug11038"	transcript_id "lnc-bbut00025373"	exon_number "2"
18	lncRNA	exon	63434689	63434787	.	-	.	gene_id "lnc-bbug11038"	transcript_id "lnc-bbut00025373"	exon_number "3"
18	lncRNA	exon	63445574	63445727	.	-	.	gene_id "lnc-bbug11038"	transcript_id "lnc-bbut00025373"	exon_number "4"
18	lncRNA	exon	63446663	63446867	.	-	.	gene_id "lnc-bbug11038"	transcript_id "lnc-bbut00025373"	exon_number "5"
18	lncRNA	exon	63447157	63447605	.	-	.	gene_id "lnc-bbug11038"	transcript_id "lnc-bbut00025373"	exon_number "6"
18	lncRNA	exon	63449779	63450499	.	-	.	gene_id "lnc-bbug11040"	transcript_id "lnc-bbut00025375"	exon_number "1"
18	lncRNA	exon	63471141	63471220	.	-	.	gene_id "lnc-bbug11042"	transcript_id "lnc-bbut00025377"	exon_number "1"
18	lncRNA	exon	63472854	63473246	.	-	.	gene_id "lnc-bbug11042"	transcript_id "lnc-bbut00025377"	exon_number "2"
18	lncRNA	exon	63593960	63594875	.	-	.	gene_id "lnc-bbug11048"	transcript_id "lnc-bbut00025383"	exon_number "1"
18	lncRNA	exon	63612170	63612786	.	-	.	gene_id "lnc-bbug11051"	transcript_id "lnc-bbut00025386"	exon_number "1"
18	lncRNA	exon	63803048	63804016	.	-	.	gene_id "lnc-bbug11060"	transcript_id "lnc-bbut00025396"	exon_number "1"
18	lncRNA	exon	63806774	63806864	.	-	.	gene_id "lnc-bbug11060"	transcript_id "lnc-bbut00025396"	exon_number "2"
18	lncRNA	exon	63851647	63852319	.	-	.	gene_id "lnc-bbug11062"	transcript_id "lnc-bbut00025407"	exon_number "1"
18	lncRNA	exon	63853268	63853371	.	-	.	gene_id "lnc-bbug11062"	transcript_id "lnc-bbut00025407"	exon_number "2"
18	lncRNA	exon	63865868	63866005	.	-	.	gene_id "lnc-bbug11063"	transcript_id "lnc-bbut00025408"	exon_number "1"
18	lncRNA	exon	63867194	63867267	.	-	.	gene_id "lnc-bbug11063"	transcript_id "lnc-bbut00025408"	exon_number "2"
18	lncRNA	exon	63879424	63879572	.	-	.	gene_id "lnc-bbug11063"	transcript_id "lnc-bbut00025408"	exon_number "3"

18	lncRNA	exon	63912110	63912438	.	-	.	gene_id "lnc-bbug11064"	transcript_id "lnc-bbut00025410"	exon_number "1"
18	lncRNA	exon	63915681	63915756	.	-	.	gene_id "lnc-bbug11064"	transcript_id "lnc-bbut00025410"	exon_number "2"
18	lncRNA	exon	63917259	63917519	.	-	.	gene_id "lnc-bbug11064"	transcript_id "lnc-bbut00025410"	exon_number "3"
18	lncRNA	exon	63937375	63937433	.	-	.	gene_id "lnc-bbug11065"	transcript_id "lnc-bbut00025412"	exon_number "1"
18	lncRNA	exon	63938908	63938979	.	-	.	gene_id "lnc-bbug11065"	transcript_id "lnc-bbut00025412"	exon_number "2"
18	lncRNA	exon	63940805	63941359	.	-	.	gene_id "lnc-bbug11065"	transcript_id "lnc-bbut00025412"	exon_number "3"
18	lncRNA	exon	64807324	64811305	.	-	.	gene_id "lnc-bbug11085"	transcript_id "lnc-bbut00025465"	exon_number "1"
18	lncRNA	exon	65442416	65442828	.	-	.	gene_id "lnc-bbug11098"	transcript_id "lnc-bbut00025486"	exon_number "1"
18	lncRNA	exon	65621676	65621812	.	-	.	gene_id "lnc-bbug11101"	transcript_id "lnc-bbut00025495"	exon_number "1"
18	lncRNA	exon	65623365	65623495	.	-	.	gene_id "lnc-bbug11101"	transcript_id "lnc-bbut00025495"	exon_number "2"
18	lncRNA	exon	65626571	65626889	.	-	.	gene_id "lnc-bbug11101"	transcript_id "lnc-bbut00025495"	exon_number "3"
18	lncRNA	exon	65633673	65633896	.	-	.	gene_id "lnc-bbug11102"	transcript_id "lnc-bbut00025498"	exon_number "1"
18	lncRNA	exon	65634495	65634682	.	-	.	gene_id "lnc-bbug11102"	transcript_id "lnc-bbut00025498"	exon_number "2"
18	lncRNA	exon	65651381	65652139	.	-	.	gene_id "lnc-bbug11102"	transcript_id "lnc-bbut00025498"	exon_number "3"
18	lncRNA	exon	65652850	65653125	.	-	.	gene_id "lnc-bbug11102"	transcript_id "lnc-bbut00025498"	exon_number "4"
18	lncRNA	exon	65633673	65633896	.	-	.	gene_id "lnc-bbug11102"	transcript_id "lnc-bbut00025497"	exon_number "1"
18	lncRNA	exon	65634413	65634682	.	-	.	gene_id "lnc-bbug11102"	transcript_id "lnc-bbut00025497"	exon_number "2"
18	lncRNA	exon	65651381	65652139	.	-	.	gene_id "lnc-bbug11102"	transcript_id "lnc-bbut00025497"	exon_number "3"
18	lncRNA	exon	65652850	65653125	.	-	.	gene_id "lnc-bbug11102"	transcript_id "lnc-bbut00025497"	exon_number "4"
18	lncRNA	exon	65633673	65633896	.	-	.	gene_id "lnc-bbug11102"	transcript_id "lnc-bbut00025496"	exon_number "1"
18	lncRNA	exon	65634413	65634678	.	-	.	gene_id "lnc-bbug11102"	transcript_id "lnc-bbut00025496"	exon_number "2"
18	lncRNA	exon	65651381	65652139	.	-	.	gene_id "lnc-bbug11102"	transcript_id "lnc-bbut00025496"	exon_number "3"
18	lncRNA	exon	65652850	65653125	.	-	.	gene_id "lnc-bbug11102"	transcript_id "lnc-bbut00025496"	exon_number "4"
18	lncRNA	exon	65638230	65638624	.	-	.	gene_id "lnc-bbug11102"	transcript_id "lnc-bbut00025500"	exon_number "1"
18	lncRNA	exon	65651381	65652139	.	-	.	gene_id "lnc-bbug11102"	transcript_id "lnc-bbut00025500"	exon_number "2"
18	lncRNA	exon	65652850	65653122	.	-	.	gene_id "lnc-bbug11102"	transcript_id "lnc-bbut00025500"	exon_number "3"
18	lncRNA	exon	65638230	65638597	.	-	.	gene_id "lnc-bbug11102"	transcript_id "lnc-bbut00025499"	exon_number "1"
18	lncRNA	exon	65651381	65652139	.	-	.	gene_id "lnc-bbug11102"	transcript_id "lnc-bbut00025499"	exon_number "2"
18	lncRNA	exon	65652850	65653122	.	-	.	gene_id "lnc-bbug11102"	transcript_id "lnc-bbut00025499"	exon_number "3"
18	lncRNA	exon	65650568	65651118	.	-	.	gene_id "lnc-bbug11102"	transcript_id "lnc-bbut00025501"	exon_number "1"
18	lncRNA	exon	65651381	65652139	.	-	.	gene_id "lnc-bbug11102"	transcript_id "lnc-bbut00025501"	exon_number "2"
18	lncRNA	exon	65652850	65653122	.	-	.	gene_id "lnc-bbug11102"	transcript_id "lnc-bbut00025501"	exon_number "3"
18	lncRNA	exon	65650568	65651486	.	-	.	gene_id "lnc-bbug11102"	transcript_id "lnc-bbut00025502"	exon_number "1"
18	lncRNA	exon	65651716	65652139	.	-	.	gene_id "lnc-bbug11102"	transcript_id "lnc-bbut00025502"	exon_number "2"
18	lncRNA	exon	65652850	65653124	.	-	.	gene_id "lnc-bbug11102"	transcript_id "lnc-bbut00025502"	exon_number "3"
18	lncRNA	exon	65650568	65652139	.	-	.	gene_id "lnc-bbug11102"	transcript_id "lnc-bbut00025506"	exon_number "1"
18	lncRNA	exon	65652850	65652912	.	-	.	gene_id "lnc-bbug11102"	transcript_id "lnc-bbut00025506"	exon_number "2"
18	lncRNA	exon	65653261	65653297	.	-	.	gene_id "lnc-bbug11102"	transcript_id "lnc-bbut00025506"	exon_number "3"
18	lncRNA	exon	65654091	65654276	.	-	.	gene_id "lnc-bbug11102"	transcript_id "lnc-bbut00025506"	exon_number "4"
18	lncRNA	exon	65650568	65652139	.	-	.	gene_id "lnc-bbug11102"	transcript_id "lnc-bbut00025508"	exon_number "1"
18	lncRNA	exon	65652850	65652975	.	-	.	gene_id "lnc-bbug11102"	transcript_id "lnc-bbut00025508"	exon_number "2"
18	lncRNA	exon	65653261	65653498	.	-	.	gene_id "lnc-bbug11102"	transcript_id "lnc-bbut00025508"	exon_number "3"
18	lncRNA	exon	65654091	65654276	.	-	.	gene_id "lnc-bbug11102"	transcript_id "lnc-bbut00025508"	exon_number "4"
18	lncRNA	exon	65650568	65651486	.	-	.	gene_id "lnc-bbug11102"	transcript_id "lnc-bbut00025509"	exon_number "1"
18	lncRNA	exon	65652850	65652912	.	-	.	gene_id "lnc-bbug11102"	transcript_id "lnc-bbut00025509"	exon_number "2"
18	lncRNA	exon	65653261	65654276	.	-	.	gene_id "lnc-bbug11102"	transcript_id "lnc-bbut00025509"	exon_number "3"
18	lncRNA	exon	65650568	65651486	.	-	.	gene_id "lnc-bbug11102"	transcript_id "lnc-bbut00025510"	exon_number "1"
18	lncRNA	exon	65652850	65652912	.	-	.	gene_id "lnc-bbug11102"	transcript_id "lnc-bbut00025510"	exon_number "2"
18	lncRNA	exon	65654091	65654276	.	-	.	gene_id "lnc-bbug11102"	transcript_id "lnc-bbut00025510"	exon_number "3"
18	lncRNA	exon	65650568	65652139	.	-	.	gene_id "lnc-bbug11102"	transcript_id "lnc-bbut00025507"	exon_number "1"
18	lncRNA	exon	65652850	65652975	.	-	.	gene_id "lnc-bbug11102"	transcript_id "lnc-bbut00025507"	exon_number "2"
18	lncRNA	exon	65653261	65653297	.	-	.	gene_id "lnc-bbug11102"	transcript_id "lnc-bbut00025507"	exon_number "3"

18	lncRNA	exon	65654091	65654276	.	-	.	gene_id "lnc-bbug11102"	transcript_id "lnc-bbut00025507"	exon_number "4"
18	lncRNA	exon	65650568	65652139	.	-	.	gene_id "lnc-bbug11102"	transcript_id "lnc-bbut00025505"	exon_number "1"
18	lncRNA	exon	65652850	65652975	.	-	.	gene_id "lnc-bbug11102"	transcript_id "lnc-bbut00025505"	exon_number "2"
18	lncRNA	exon	65653261	65654276	.	-	.	gene_id "lnc-bbug11102"	transcript_id "lnc-bbut00025505"	exon_number "3"
18	lncRNA	exon	65650568	65652139	.	-	.	gene_id "lnc-bbug11102"	transcript_id "lnc-bbut00025504"	exon_number "1"
18	lncRNA	exon	65652850	65652912	.	-	.	gene_id "lnc-bbug11102"	transcript_id "lnc-bbut00025504"	exon_number "2"
18	lncRNA	exon	65654091	65654276	.	-	.	gene_id "lnc-bbug11102"	transcript_id "lnc-bbut00025504"	exon_number "3"
18	lncRNA	exon	65650568	65652139	.	-	.	gene_id "lnc-bbug11102"	transcript_id "lnc-bbut00025503"	exon_number "1"
18	lncRNA	exon	65652850	65652912	.	-	.	gene_id "lnc-bbug11102"	transcript_id "lnc-bbut00025503"	exon_number "2"
18	lncRNA	exon	65653261	65654276	.	-	.	gene_id "lnc-bbug11102"	transcript_id "lnc-bbut00025503"	exon_number "3"
18	lncRNA	exon	65664495	65664690	.	-	.	gene_id "lnc-bbug11103"	transcript_id "lnc-bbut00025511"	exon_number "1"
18	lncRNA	exon	65667159	65667258	.	-	.	gene_id "lnc-bbug11103"	transcript_id "lnc-bbut00025511"	exon_number "2"
18	lncRNA	exon	65674795	65674980	.	-	.	gene_id "lnc-bbug11103"	transcript_id "lnc-bbut00025511"	exon_number "3"
18	lncRNA	exon	65755994	65757587	.	-	.	gene_id "lnc-bbug11105"	transcript_id "lnc-bbut00025514"	exon_number "1"
18	lncRNA	exon	65757996	65758118	.	-	.	gene_id "lnc-bbug11105"	transcript_id "lnc-bbut00025514"	exon_number "2"
18	lncRNA	exon	65794020	65794082	.	-	.	gene_id "lnc-bbug11106"	transcript_id "lnc-bbut00025519"	exon_number "1"
18	lncRNA	exon	65797047	65797215	.	-	.	gene_id "lnc-bbug11106"	transcript_id "lnc-bbut00025519"	exon_number "2"
18	lncRNA	exon	65797371	65797451	.	-	.	gene_id "lnc-bbug11106"	transcript_id "lnc-bbut00025519"	exon_number "3"
18	lncRNA	exon	65799886	65800276	.	-	.	gene_id "lnc-bbug11106"	transcript_id "lnc-bbut00025519"	exon_number "4"
19	lncRNA	exon	1898704	1898822	.	+	.	gene_id "lnc-bbug11116"	transcript_id "lnc-bbut00025541"	exon_number "1"
19	lncRNA	exon	1901190	1901283	.	+	.	gene_id "lnc-bbug11116"	transcript_id "lnc-bbut00025541"	exon_number "2"
19	lncRNA	exon	1910728	1911001	.	+	.	gene_id "lnc-bbug11116"	transcript_id "lnc-bbut00025541"	exon_number "3"
19	lncRNA	exon	1898717	1898822	.	+	.	gene_id "lnc-bbug11116"	transcript_id "lnc-bbut00025542"	exon_number "1"
19	lncRNA	exon	1899855	1899928	.	+	.	gene_id "lnc-bbug11116"	transcript_id "lnc-bbut00025542"	exon_number "2"
19	lncRNA	exon	1901190	1901283	.	+	.	gene_id "lnc-bbug11116"	transcript_id "lnc-bbut00025542"	exon_number "3"
19	lncRNA	exon	1910728	1911001	.	+	.	gene_id "lnc-bbug11116"	transcript_id "lnc-bbut00025542"	exon_number "4"
19	lncRNA	exon	1898991	1899012	.	+	.	gene_id "lnc-bbug11116"	transcript_id "lnc-bbut00025543"	exon_number "1"
19	lncRNA	exon	1899855	1899928	.	+	.	gene_id "lnc-bbug11116"	transcript_id "lnc-bbut00025543"	exon_number "2"
19	lncRNA	exon	1901190	1901283	.	+	.	gene_id "lnc-bbug11116"	transcript_id "lnc-bbut00025543"	exon_number "3"
19	lncRNA	exon	1910728	1911001	.	+	.	gene_id "lnc-bbug11116"	transcript_id "lnc-bbut00025543"	exon_number "4"
19	lncRNA	exon	1899061	1899239	.	+	.	gene_id "lnc-bbug11116"	transcript_id "lnc-bbut00025544"	exon_number "1"
19	lncRNA	exon	1899855	1899928	.	+	.	gene_id "lnc-bbug11116"	transcript_id "lnc-bbut00025544"	exon_number "2"
19	lncRNA	exon	1901190	1901283	.	+	.	gene_id "lnc-bbug11116"	transcript_id "lnc-bbut00025544"	exon_number "3"
19	lncRNA	exon	1910728	1911001	.	+	.	gene_id "lnc-bbug11116"	transcript_id "lnc-bbut00025544"	exon_number "4"
19	lncRNA	exon	1963302	1964157	.	+	.	gene_id "lnc-bbug11117"	transcript_id "lnc-bbut00025545"	exon_number "1"
19	lncRNA	exon	1966264	1966409	.	+	.	gene_id "lnc-bbug11117"	transcript_id "lnc-bbut00025545"	exon_number "2"
19	lncRNA	exon	1995066	1999496	.	+	.	gene_id "lnc-bbug11117"	transcript_id "lnc-bbut00025545"	exon_number "3"
19	lncRNA	exon	1963302	1964157	.	+	.	gene_id "lnc-bbug11117"	transcript_id "lnc-bbut00025546"	exon_number "1"
19	lncRNA	exon	1966264	1966409	.	+	.	gene_id "lnc-bbug11117"	transcript_id "lnc-bbut00025546"	exon_number "2"
19	lncRNA	exon	1995066	1995169	.	+	.	gene_id "lnc-bbug11117"	transcript_id "lnc-bbut00025546"	exon_number "3"
19	lncRNA	exon	2013341	2014068	.	+	.	gene_id "lnc-bbug11117"	transcript_id "lnc-bbut00025546"	exon_number "4"
19	lncRNA	exon	1963952	1964157	.	+	.	gene_id "lnc-bbug11117"	transcript_id "lnc-bbut00025547"	exon_number "1"
19	lncRNA	exon	1966264	1967009	.	+	.	gene_id "lnc-bbug11117"	transcript_id "lnc-bbut00025547"	exon_number "2"
19	lncRNA	exon	1963952	1964157	.	+	.	gene_id "lnc-bbug11117"	transcript_id "lnc-bbut00025549"	exon_number "1"
19	lncRNA	exon	1966264	1966405	.	+	.	gene_id "lnc-bbug11117"	transcript_id "lnc-bbut00025549"	exon_number "2"
19	lncRNA	exon	1995066	1995169	.	+	.	gene_id "lnc-bbug11117"	transcript_id "lnc-bbut00025549"	exon_number "3"
19	lncRNA	exon	1996872	1999496	.	+	.	gene_id "lnc-bbug11117"	transcript_id "lnc-bbut00025549"	exon_number "4"
19	lncRNA	exon	1963952	1964157	.	+	.	gene_id "lnc-bbug11117"	transcript_id "lnc-bbut00025548"	exon_number "1"
19	lncRNA	exon	1966264	1966405	.	+	.	gene_id "lnc-bbug11117"	transcript_id "lnc-bbut00025548"	exon_number "2"
19	lncRNA	exon	1995066	1999496	.	+	.	gene_id "lnc-bbug11117"	transcript_id "lnc-bbut00025548"	exon_number "3"
19	lncRNA	exon	1964114	1964157	.	+	.	gene_id "lnc-bbug11117"	transcript_id "lnc-bbut00025550"	exon_number "1"
19	lncRNA	exon	1965026	1965628	.	+	.	gene_id "lnc-bbug11117"	transcript_id "lnc-bbut00025550"	exon_number "2"

19	lncRNA	exon	1965895	1966409	.	+	.	gene_id "lnc-bbug11117"	transcript_id "lnc-bbut00025551"	exon_number "1"
19	lncRNA	exon	1995066	1995169	.	+	.	gene_id "lnc-bbug11117"	transcript_id "lnc-bbut00025551"	exon_number "2"
19	lncRNA	exon	2013341	2014068	.	+	.	gene_id "lnc-bbug11117"	transcript_id "lnc-bbut00025551"	exon_number "3"
19	lncRNA	exon	3740729	3741096	.	+	.	gene_id "lnc-bbug11125"	transcript_id "lnc-bbut00025564"	exon_number "1"
19	lncRNA	exon	3741252	3741858	.	+	.	gene_id "lnc-bbug11125"	transcript_id "lnc-bbut00025564"	exon_number "2"
19	lncRNA	exon	4356020	4366252	.	+	.	gene_id "lnc-bbug11128"	transcript_id "lnc-bbut00025567"	exon_number "1"
19	lncRNA	exon	4370290	4370418	.	+	.	gene_id "lnc-bbug11128"	transcript_id "lnc-bbut00025567"	exon_number "2"
19	lncRNA	exon	4374062	4375320	.	+	.	gene_id "lnc-bbug11128"	transcript_id "lnc-bbut00025567"	exon_number "3"
19	lncRNA	exon	4376109	4376305	.	+	.	gene_id "lnc-bbug11128"	transcript_id "lnc-bbut00025567"	exon_number "4"
19	lncRNA	exon	4486498	4491163	.	+	.	gene_id "lnc-bbug11130"	transcript_id "lnc-bbut00025570"	exon_number "1"
19	lncRNA	exon	4522879	4523063	.	+	.	gene_id "lnc-bbug11132"	transcript_id "lnc-bbut00025572"	exon_number "1"
19	lncRNA	exon	4529733	4529780	.	+	.	gene_id "lnc-bbug11132"	transcript_id "lnc-bbut00025572"	exon_number "2"
19	lncRNA	exon	4546366	4546495	.	+	.	gene_id "lnc-bbug11132"	transcript_id "lnc-bbut00025572"	exon_number "3"
19	lncRNA	exon	4553808	4554299	.	+	.	gene_id "lnc-bbug11132"	transcript_id "lnc-bbut00025572"	exon_number "4"
19	lncRNA	exon	4730679	4730728	.	+	.	gene_id "lnc-bbug11135"	transcript_id "lnc-bbut00025577"	exon_number "1"
19	lncRNA	exon	4732335	4732500	.	+	.	gene_id "lnc-bbug11135"	transcript_id "lnc-bbut00025577"	exon_number "2"
19	lncRNA	exon	4733377	4734047	.	+	.	gene_id "lnc-bbug11135"	transcript_id "lnc-bbut00025577"	exon_number "3"
19	lncRNA	exon	4732206	4732500	.	+	.	gene_id "lnc-bbug11135"	transcript_id "lnc-bbut00025578"	exon_number "1"
19	lncRNA	exon	4733377	4733417	.	+	.	gene_id "lnc-bbug11135"	transcript_id "lnc-bbut00025578"	exon_number "2"
19	lncRNA	exon	4737159	4737623	.	+	.	gene_id "lnc-bbug11135"	transcript_id "lnc-bbut00025578"	exon_number "3"
19	lncRNA	exon	5101078	5101130	.	+	.	gene_id "lnc-bbug11136"	transcript_id "lnc-bbut00025579"	exon_number "1"
19	lncRNA	exon	5101260	5101502	.	+	.	gene_id "lnc-bbug11136"	transcript_id "lnc-bbut00025579"	exon_number "2"
19	lncRNA	exon	5117340	5117552	.	+	.	gene_id "lnc-bbug11137"	transcript_id "lnc-bbut00025580"	exon_number "1"
19	lncRNA	exon	5118196	5118342	.	+	.	gene_id "lnc-bbug11137"	transcript_id "lnc-bbut00025580"	exon_number "2"
19	lncRNA	exon	5130208	5132588	.	+	.	gene_id "lnc-bbug11137"	transcript_id "lnc-bbut00025580"	exon_number "3"
19	lncRNA	exon	5145138	5146070	.	+	.	gene_id "lnc-bbug11138"	transcript_id "lnc-bbut00025581"	exon_number "1"
19	lncRNA	exon	5153479	5153613	.	+	.	gene_id "lnc-bbug11138"	transcript_id "lnc-bbut00025581"	exon_number "2"
19	lncRNA	exon	5153744	5153934	.	+	.	gene_id "lnc-bbug11138"	transcript_id "lnc-bbut00025581"	exon_number "3"
19	lncRNA	exon	5154852	5154909	.	+	.	gene_id "lnc-bbug11138"	transcript_id "lnc-bbut00025581"	exon_number "4"
19	lncRNA	exon	5158911	5159739	.	+	.	gene_id "lnc-bbug11138"	transcript_id "lnc-bbut00025581"	exon_number "5"
19	lncRNA	exon	5145138	5146070	.	+	.	gene_id "lnc-bbug11138"	transcript_id "lnc-bbut00025582"	exon_number "1"
19	lncRNA	exon	5153479	5153613	.	+	.	gene_id "lnc-bbug11138"	transcript_id "lnc-bbut00025582"	exon_number "2"
19	lncRNA	exon	5153744	5153934	.	+	.	gene_id "lnc-bbug11138"	transcript_id "lnc-bbut00025582"	exon_number "3"
19	lncRNA	exon	5154852	5154909	.	+	.	gene_id "lnc-bbug11138"	transcript_id "lnc-bbut00025582"	exon_number "4"
19	lncRNA	exon	5163819	5164109	.	+	.	gene_id "lnc-bbug11138"	transcript_id "lnc-bbut00025582"	exon_number "5"
19	lncRNA	exon	5274373	5274900	.	+	.	gene_id "lnc-bbug11139"	transcript_id "lnc-bbut00025583"	exon_number "1"
19	lncRNA	exon	5275312	5275450	.	+	.	gene_id "lnc-bbug11139"	transcript_id "lnc-bbut00025583"	exon_number "2"
19	lncRNA	exon	5276255	5277162	.	+	.	gene_id "lnc-bbug11139"	transcript_id "lnc-bbut00025583"	exon_number "3"
19	lncRNA	exon	5486037	5487270	.	+	.	gene_id "lnc-bbug11142"	transcript_id "lnc-bbut00025589"	exon_number "1"
19	lncRNA	exon	5496947	5497032	.	+	.	gene_id "lnc-bbug11142"	transcript_id "lnc-bbut00025589"	exon_number "2"
19	lncRNA	exon	5501067	5501105	.	+	.	gene_id "lnc-bbug11142"	transcript_id "lnc-bbut00025589"	exon_number "3"
19	lncRNA	exon	5503255	5503376	.	+	.	gene_id "lnc-bbug11142"	transcript_id "lnc-bbut00025589"	exon_number "4"
19	lncRNA	exon	5504677	5504858	.	+	.	gene_id "lnc-bbug11142"	transcript_id "lnc-bbut00025589"	exon_number "5"
19	lncRNA	exon	7096366	7097207	.	+	.	gene_id "lnc-bbug11149"	transcript_id "lnc-bbut00025606"	exon_number "1"
19	lncRNA	exon	7098788	7098921	.	+	.	gene_id "lnc-bbug11149"	transcript_id "lnc-bbut00025606"	exon_number "2"
19	lncRNA	exon	7102763	7103270	.	+	.	gene_id "lnc-bbug11149"	transcript_id "lnc-bbut00025606"	exon_number "3"
19	lncRNA	exon	7109342	7111082	.	+	.	gene_id "lnc-bbug11149"	transcript_id "lnc-bbut00025606"	exon_number "4"
19	lncRNA	exon	7409191	7409291	.	+	.	gene_id "lnc-bbug11151"	transcript_id "lnc-bbut00025608"	exon_number "1"
19	lncRNA	exon	7409914	7410155	.	+	.	gene_id "lnc-bbug11151"	transcript_id "lnc-bbut00025608"	exon_number "2"
19	lncRNA	exon	7410847	7411109	.	+	.	gene_id "lnc-bbug11151"	transcript_id "lnc-bbut00025608"	exon_number "3"
19	lncRNA	exon	7411868	7411973	.	+	.	gene_id "lnc-bbug11151"	transcript_id "lnc-bbut00025608"	exon_number "4"
19	lncRNA	exon	7439984	7442035	.	+	.	gene_id "lnc-bbug11151"	transcript_id "lnc-bbut00025608"	exon_number "5"

19	lncRNA	exon	7487779	7487948	.	+	.	gene_id "lnc-bbug11152"	transcript_id "lnc-bbut00025609"	exon_number "1"
19	lncRNA	exon	7488119	7488373	.	+	.	gene_id "lnc-bbug11152"	transcript_id "lnc-bbut00025609"	exon_number "2"
19	lncRNA	exon	7490314	7490513	.	+	.	gene_id "lnc-bbug11152"	transcript_id "lnc-bbut00025609"	exon_number "3"
19	lncRNA	exon	8204161	8204281	.	+	.	gene_id "lnc-bbug11156"	transcript_id "lnc-bbut00025616"	exon_number "1"
19	lncRNA	exon	8204516	8204913	.	+	.	gene_id "lnc-bbug11156"	transcript_id "lnc-bbut00025616"	exon_number "2"
19	lncRNA	exon	8210013	8210287	.	+	.	gene_id "lnc-bbug11156"	transcript_id "lnc-bbut00025616"	exon_number "3"
19	lncRNA	exon	8618414	8618621	.	+	.	gene_id "lnc-bbug11157"	transcript_id "lnc-bbut00025617"	exon_number "1"
19	lncRNA	exon	8619875	8619974	.	+	.	gene_id "lnc-bbug11157"	transcript_id "lnc-bbut00025617"	exon_number "2"
19	lncRNA	exon	8631736	8632363	.	+	.	gene_id "lnc-bbug11157"	transcript_id "lnc-bbut00025617"	exon_number "3"
19	lncRNA	exon	8618416	8618621	.	+	.	gene_id "lnc-bbug11157"	transcript_id "lnc-bbut00025618"	exon_number "1"
19	lncRNA	exon	8619875	8619974	.	+	.	gene_id "lnc-bbug11157"	transcript_id "lnc-bbut00025618"	exon_number "2"
19	lncRNA	exon	8621127	8621344	.	+	.	gene_id "lnc-bbug11157"	transcript_id "lnc-bbut00025618"	exon_number "3"
19	lncRNA	exon	9844245	9844416	.	+	.	gene_id "lnc-bbug11161"	transcript_id "lnc-bbut00025625"	exon_number "1"
19	lncRNA	exon	9845418	9845526	.	+	.	gene_id "lnc-bbug11161"	transcript_id "lnc-bbut00025625"	exon_number "2"
19	lncRNA	exon	9847802	9848068	.	+	.	gene_id "lnc-bbug11161"	transcript_id "lnc-bbut00025625"	exon_number "3"
19	lncRNA	exon	10512145	10512251	.	+	.	gene_id "lnc-bbug11165"	transcript_id "lnc-bbut00025637"	exon_number "1"
19	lncRNA	exon	10533543	10533843	.	+	.	gene_id "lnc-bbug11165"	transcript_id "lnc-bbut00025637"	exon_number "2"
19	lncRNA	exon	14078213	14078367	.	+	.	gene_id "lnc-bbug11173"	transcript_id "lnc-bbut00025654"	exon_number "1"
19	lncRNA	exon	14080026	14081724	.	+	.	gene_id "lnc-bbug11173"	transcript_id "lnc-bbut00025654"	exon_number "2"
19	lncRNA	exon	14093288	14093558	.	+	.	gene_id "lnc-bbug11174"	transcript_id "lnc-bbut00025655"	exon_number "1"
19	lncRNA	exon	14108354	14108534	.	+	.	gene_id "lnc-bbug11174"	transcript_id "lnc-bbut00025655"	exon_number "2"
19	lncRNA	exon	14129617	14130150	.	+	.	gene_id "lnc-bbug11174"	transcript_id "lnc-bbut00025655"	exon_number "3"
19	lncRNA	exon	14794432	14794582	.	+	.	gene_id "lnc-bbug11176"	transcript_id "lnc-bbut00025657"	exon_number "1"
19	lncRNA	exon	14810840	14810987	.	+	.	gene_id "lnc-bbug11176"	transcript_id "lnc-bbut00025657"	exon_number "2"
19	lncRNA	exon	14812710	14813791	.	+	.	gene_id "lnc-bbug11176"	transcript_id "lnc-bbut00025657"	exon_number "3"
19	lncRNA	exon	16937263	16937663	.	+	.	gene_id "lnc-bbug11180"	transcript_id "lnc-bbut00025663"	exon_number "1"
19	lncRNA	exon	17117978	17118171	.	+	.	gene_id "lnc-bbug11180"	transcript_id "lnc-bbut00025663"	exon_number "2"
19	lncRNA	exon	17186619	17186716	.	+	.	gene_id "lnc-bbug11180"	transcript_id "lnc-bbut00025663"	exon_number "3"
19	lncRNA	exon	17188392	17188494	.	+	.	gene_id "lnc-bbug11180"	transcript_id "lnc-bbut00025663"	exon_number "4"
19	lncRNA	exon	17190877	17190994	.	+	.	gene_id "lnc-bbug11180"	transcript_id "lnc-bbut00025663"	exon_number "5"
19	lncRNA	exon	17192815	17192959	.	+	.	gene_id "lnc-bbug11180"	transcript_id "lnc-bbut00025663"	exon_number "6"
19	lncRNA	exon	17193758	17193993	.	+	.	gene_id "lnc-bbug11180"	transcript_id "lnc-bbut00025663"	exon_number "7"
19	lncRNA	exon	17206701	17208417	.	+	.	gene_id "lnc-bbug11180"	transcript_id "lnc-bbut00025663"	exon_number "8"
19	lncRNA	exon	16937263	16937663	.	+	.	gene_id "lnc-bbug11180"	transcript_id "lnc-bbut00025662"	exon_number "1"
19	lncRNA	exon	17117978	17118171	.	+	.	gene_id "lnc-bbug11180"	transcript_id "lnc-bbut00025662"	exon_number "2"
19	lncRNA	exon	17186619	17186716	.	+	.	gene_id "lnc-bbug11180"	transcript_id "lnc-bbut00025662"	exon_number "3"
19	lncRNA	exon	17188392	17188494	.	+	.	gene_id "lnc-bbug11180"	transcript_id "lnc-bbut00025662"	exon_number "4"
19	lncRNA	exon	17190877	17190994	.	+	.	gene_id "lnc-bbug11180"	transcript_id "lnc-bbut00025662"	exon_number "5"
19	lncRNA	exon	17193758	17193993	.	+	.	gene_id "lnc-bbug11180"	transcript_id "lnc-bbut00025662"	exon_number "6"
19	lncRNA	exon	17206701	17208417	.	+	.	gene_id "lnc-bbug11180"	transcript_id "lnc-bbut00025662"	exon_number "7"
19	lncRNA	exon	17914487	17914911	.	+	.	gene_id "lnc-bbug11182"	transcript_id "lnc-bbut00025665"	exon_number "1"
19	lncRNA	exon	17915755	17915888	.	+	.	gene_id "lnc-bbug11182"	transcript_id "lnc-bbut00025665"	exon_number "2"
19	lncRNA	exon	17916956	17918506	.	+	.	gene_id "lnc-bbug11182"	transcript_id "lnc-bbut00025665"	exon_number "3"
19	lncRNA	exon	20076390	20078060	.	+	.	gene_id "lnc-bbug11188"	transcript_id "lnc-bbut00025684"	exon_number "1"
19	lncRNA	exon	20083163	20083259	.	+	.	gene_id "lnc-bbug11188"	transcript_id "lnc-bbut00025684"	exon_number "2"
19	lncRNA	exon	20083366	20084211	.	+	.	gene_id "lnc-bbug11188"	transcript_id "lnc-bbut00025684"	exon_number "3"
19	lncRNA	exon	20076390	20078060	.	+	.	gene_id "lnc-bbug11188"	transcript_id "lnc-bbut00025686"	exon_number "1"
19	lncRNA	exon	20083163	20083259	.	+	.	gene_id "lnc-bbug11188"	transcript_id "lnc-bbut00025686"	exon_number "2"
19	lncRNA	exon	20083366	20083570	.	+	.	gene_id "lnc-bbug11188"	transcript_id "lnc-bbut00025686"	exon_number "3"
19	lncRNA	exon	20084793	20084999	.	+	.	gene_id "lnc-bbug11188"	transcript_id "lnc-bbut00025686"	exon_number "4"
19	lncRNA	exon	20076390	20078060	.	+	.	gene_id "lnc-bbug11188"	transcript_id "lnc-bbut00025685"	exon_number "1"
19	lncRNA	exon	20083163	20083259	.	+	.	gene_id "lnc-bbug11188"	transcript_id "lnc-bbut00025685"	exon_number "2"

19	lncRNA	exon	20083366	20083455	.	+	.	gene_id "lnc-bbug11188"	transcript_id "lnc-bbut00025685"	exon_number "3"
19	lncRNA	exon	20084793	20084999	.	+	.	gene_id "lnc-bbug11188"	transcript_id "lnc-bbut00025685"	exon_number "4"
19	lncRNA	exon	22168959	22169168	.	+	.	gene_id "lnc-bbug11196"	transcript_id "lnc-bbut00025695"	exon_number "1"
19	lncRNA	exon	22169641	22171340	.	+	.	gene_id "lnc-bbug11196"	transcript_id "lnc-bbut00025695"	exon_number "2"
19	lncRNA	exon	22235342	22235477	.	+	.	gene_id "lnc-bbug11197"	transcript_id "lnc-bbut00025696"	exon_number "1"
19	lncRNA	exon	22235952	22236282	.	+	.	gene_id "lnc-bbug11197"	transcript_id "lnc-bbut00025696"	exon_number "2"
19	lncRNA	exon	22992945	22995188	.	+	.	gene_id "lnc-bbug11201"	transcript_id "lnc-bbut00025701"	exon_number "1"
19	lncRNA	exon	23628888	23630411	.	+	.	gene_id "lnc-bbug11205"	transcript_id "lnc-bbut00025713"	exon_number "1"
19	lncRNA	exon	25374142	25374485	.	+	.	gene_id "lnc-bbug11213"	transcript_id "lnc-bbut00025724"	exon_number "1"
19	lncRNA	exon	25375044	25375559	.	+	.	gene_id "lnc-bbug11213"	transcript_id "lnc-bbut00025724"	exon_number "2"
19	lncRNA	exon	25635900	25635999	.	+	.	gene_id "lnc-bbug11215"	transcript_id "lnc-bbut00025726"	exon_number "1"
19	lncRNA	exon	25643601	25643751	.	+	.	gene_id "lnc-bbug11215"	transcript_id "lnc-bbut00025726"	exon_number "2"
19	lncRNA	exon	25655858	25657375	.	+	.	gene_id "lnc-bbug11215"	transcript_id "lnc-bbut00025726"	exon_number "3"
19	lncRNA	exon	25770109	25771712	.	+	.	gene_id "lnc-bbug11217"	transcript_id "lnc-bbut00025728"	exon_number "1"
19	lncRNA	exon	25774005	25778128	.	+	.	gene_id "lnc-bbug11217"	transcript_id "lnc-bbut00025728"	exon_number "2"
19	lncRNA	exon	26420326	26420563	.	+	.	gene_id "lnc-bbug11218"	transcript_id "lnc-bbut00025730"	exon_number "1"
19	lncRNA	exon	26437977	26438030	.	+	.	gene_id "lnc-bbug11218"	transcript_id "lnc-bbut00025730"	exon_number "2"
19	lncRNA	exon	26467522	26467572	.	+	.	gene_id "lnc-bbug11218"	transcript_id "lnc-bbut00025730"	exon_number "3"
19	lncRNA	exon	26478256	26478450	.	+	.	gene_id "lnc-bbug11218"	transcript_id "lnc-bbut00025730"	exon_number "4"
19	lncRNA	exon	26420326	26420563	.	+	.	gene_id "lnc-bbug11218"	transcript_id "lnc-bbut00025729"	exon_number "1"
19	lncRNA	exon	26437977	26438030	.	+	.	gene_id "lnc-bbug11218"	transcript_id "lnc-bbut00025729"	exon_number "2"
19	lncRNA	exon	26478256	26478450	.	+	.	gene_id "lnc-bbug11218"	transcript_id "lnc-bbut00025729"	exon_number "3"
19	lncRNA	exon	27491670	27493211	.	+	.	gene_id "lnc-bbug11219"	transcript_id "lnc-bbut00025731"	exon_number "1"
19	lncRNA	exon	27494200	27494309	.	+	.	gene_id "lnc-bbug11219"	transcript_id "lnc-bbut00025731"	exon_number "2"
19	lncRNA	exon	27495006	27495117	.	+	.	gene_id "lnc-bbug11219"	transcript_id "lnc-bbut00025731"	exon_number "3"
19	lncRNA	exon	28864871	28864896	.	+	.	gene_id "lnc-bbug11222"	transcript_id "lnc-bbut00025740"	exon_number "1"
19	lncRNA	exon	28864947	28865223	.	+	.	gene_id "lnc-bbug11222"	transcript_id "lnc-bbut00025740"	exon_number "2"
19	lncRNA	exon	28865425	28865504	.	+	.	gene_id "lnc-bbug11222"	transcript_id "lnc-bbut00025740"	exon_number "3"
19	lncRNA	exon	28865580	28865727	.	+	.	gene_id "lnc-bbug11222"	transcript_id "lnc-bbut00025740"	exon_number "4"
19	lncRNA	exon	28888349	28888504	.	+	.	gene_id "lnc-bbug11222"	transcript_id "lnc-bbut00025740"	exon_number "5"
19	lncRNA	exon	30030382	30030679	.	+	.	gene_id "lnc-bbug11224"	transcript_id "lnc-bbut00025742"	exon_number "1"
19	lncRNA	exon	31027453	31029069	.	+	.	gene_id "lnc-bbug11228"	transcript_id "lnc-bbut00025748"	exon_number "1"
19	lncRNA	exon	31111212	31111363	.	+	.	gene_id "lnc-bbug11231"	transcript_id "lnc-bbut00025756"	exon_number "1"
19	lncRNA	exon	31123599	31123725	.	+	.	gene_id "lnc-bbug11231"	transcript_id "lnc-bbut00025756"	exon_number "2"
19	lncRNA	exon	31127164	31127361	.	+	.	gene_id "lnc-bbug11231"	transcript_id "lnc-bbut00025756"	exon_number "3"
19	lncRNA	exon	31134803	31135233	.	+	.	gene_id "lnc-bbug11231"	transcript_id "lnc-bbut00025756"	exon_number "4"
19	lncRNA	exon	31499956	31500112	.	+	.	gene_id "lnc-bbug11234"	transcript_id "lnc-bbut00025762"	exon_number "1"
19	lncRNA	exon	31503082	31503297	.	+	.	gene_id "lnc-bbug11234"	transcript_id "lnc-bbut00025762"	exon_number "2"
19	lncRNA	exon	31504037	31504249	.	+	.	gene_id "lnc-bbug11234"	transcript_id "lnc-bbut00025762"	exon_number "3"
19	lncRNA	exon	31506610	31506727	.	+	.	gene_id "lnc-bbug11234"	transcript_id "lnc-bbut00025762"	exon_number "4"
19	lncRNA	exon	31508511	31510031	.	+	.	gene_id "lnc-bbug11234"	transcript_id "lnc-bbut00025762"	exon_number "5"
19	lncRNA	exon	31562885	31563540	.	+	.	gene_id "lnc-bbug11235"	transcript_id "lnc-bbut00025763"	exon_number "1"
19	lncRNA	exon	31673467	31673547	.	+	.	gene_id "lnc-bbug11235"	transcript_id "lnc-bbut00025763"	exon_number "2"
19	lncRNA	exon	31674141	31674784	.	+	.	gene_id "lnc-bbug11235"	transcript_id "lnc-bbut00025763"	exon_number "3"
19	lncRNA	exon	33388609	33388760	.	+	.	gene_id "lnc-bbug11241"	transcript_id "lnc-bbut00025770"	exon_number "1"
19	lncRNA	exon	33389377	33389512	.	+	.	gene_id "lnc-bbug11241"	transcript_id "lnc-bbut00025770"	exon_number "2"
19	lncRNA	exon	33390027	33390111	.	+	.	gene_id "lnc-bbug11241"	transcript_id "lnc-bbut00025770"	exon_number "3"
19	lncRNA	exon	33391209	33391326	.	+	.	gene_id "lnc-bbug11241"	transcript_id "lnc-bbut00025770"	exon_number "4"
19	lncRNA	exon	34152758	34152792	.	+	.	gene_id "lnc-bbug11244"	transcript_id "lnc-bbut00025778"	exon_number "1"
19	lncRNA	exon	34153727	34154205	.	+	.	gene_id "lnc-bbug11244"	transcript_id "lnc-bbut00025778"	exon_number "2"
19	lncRNA	exon	34154252	34154391	.	+	.	gene_id "lnc-bbug11244"	transcript_id "lnc-bbut00025778"	exon_number "3"
19	lncRNA	exon	34154463	34154883	.	+	.	gene_id "lnc-bbug11244"	transcript_id "lnc-bbut00025778"	exon_number "4"

19	lncRNA	exon	34155409	34155498	.	+	.	gene_id "lnc-bbug11244"	transcript_id "lnc-bbut00025778"	exon_number "5"
19	lncRNA	exon	34778432	34781231	.	+	.	gene_id "lnc-bbug11248"	transcript_id "lnc-bbut00025785"	exon_number "1"
19	lncRNA	exon	34781340	34781492	.	+	.	gene_id "lnc-bbug11248"	transcript_id "lnc-bbut00025785"	exon_number "2"
19	lncRNA	exon	35300627	35300826	.	+	.	gene_id "lnc-bbug11252"	transcript_id "lnc-bbut00025796"	exon_number "1"
19	lncRNA	exon	35304000	35304129	.	+	.	gene_id "lnc-bbug11252"	transcript_id "lnc-bbut00025796"	exon_number "2"
19	lncRNA	exon	35304762	35306597	.	+	.	gene_id "lnc-bbug11252"	transcript_id "lnc-bbut00025796"	exon_number "3"
19	lncRNA	exon	35300627	35300813	.	+	.	gene_id "lnc-bbug11252"	transcript_id "lnc-bbut00025795"	exon_number "1"
19	lncRNA	exon	35304000	35304129	.	+	.	gene_id "lnc-bbug11252"	transcript_id "lnc-bbut00025795"	exon_number "2"
19	lncRNA	exon	35304762	35306597	.	+	.	gene_id "lnc-bbug11252"	transcript_id "lnc-bbut00025795"	exon_number "3"
19	lncRNA	exon	35336938	35337938	.	+	.	gene_id "lnc-bbug11253"	transcript_id "lnc-bbut00025797"	exon_number "1"
19	lncRNA	exon	35409540	35409656	.	+	.	gene_id "lnc-bbug11253"	transcript_id "lnc-bbut00025797"	exon_number "2"
19	lncRNA	exon	35409767	35411847	.	+	.	gene_id "lnc-bbug11253"	transcript_id "lnc-bbut00025797"	exon_number "3"
19	lncRNA	exon	37327842	37328057	.	+	.	gene_id "lnc-bbug11259"	transcript_id "lnc-bbut00025824"	exon_number "1"
19	lncRNA	exon	37330983	37331060	.	+	.	gene_id "lnc-bbug11259"	transcript_id "lnc-bbut00025824"	exon_number "2"
19	lncRNA	exon	37336598	37336749	.	+	.	gene_id "lnc-bbug11259"	transcript_id "lnc-bbut00025824"	exon_number "3"
19	lncRNA	exon	37345737	37345841	.	+	.	gene_id "lnc-bbug11259"	transcript_id "lnc-bbut00025824"	exon_number "4"
19	lncRNA	exon	38332913	38333012	.	+	.	gene_id "lnc-bbug11266"	transcript_id "lnc-bbut00025846"	exon_number "1"
19	lncRNA	exon	38345877	38345953	.	+	.	gene_id "lnc-bbug11266"	transcript_id "lnc-bbut00025846"	exon_number "2"
19	lncRNA	exon	38347385	38347488	.	+	.	gene_id "lnc-bbug11266"	transcript_id "lnc-bbut00025846"	exon_number "3"
19	lncRNA	exon	38351939	38352103	.	+	.	gene_id "lnc-bbug11266"	transcript_id "lnc-bbut00025846"	exon_number "4"
19	lncRNA	exon	38332931	38333016	.	+	.	gene_id "lnc-bbug11266"	transcript_id "lnc-bbut00025847"	exon_number "1"
19	lncRNA	exon	38345877	38345953	.	+	.	gene_id "lnc-bbug11266"	transcript_id "lnc-bbut00025847"	exon_number "2"
19	lncRNA	exon	38347385	38347488	.	+	.	gene_id "lnc-bbug11266"	transcript_id "lnc-bbut00025847"	exon_number "3"
19	lncRNA	exon	38351939	38352103	.	+	.	gene_id "lnc-bbug11266"	transcript_id "lnc-bbut00025847"	exon_number "4"
19	lncRNA	exon	40160241	40160367	.	+	.	gene_id "lnc-bbug11274"	transcript_id "lnc-bbut00025866"	exon_number "1"
19	lncRNA	exon	40163234	40163297	.	+	.	gene_id "lnc-bbug11274"	transcript_id "lnc-bbut00025866"	exon_number "2"
19	lncRNA	exon	40163390	40163764	.	+	.	gene_id "lnc-bbug11274"	transcript_id "lnc-bbut00025866"	exon_number "3"
19	lncRNA	exon	40774487	40775267	.	+	.	gene_id "lnc-bbug11275"	transcript_id "lnc-bbut00025867"	exon_number "1"
19	lncRNA	exon	40775449	40775670	.	+	.	gene_id "lnc-bbug11275"	transcript_id "lnc-bbut00025867"	exon_number "2"
19	lncRNA	exon	40826108	40826623	.	+	.	gene_id "lnc-bbug11276"	transcript_id "lnc-bbut00025868"	exon_number "1"
19	lncRNA	exon	40831468	40831579	.	+	.	gene_id "lnc-bbug11276"	transcript_id "lnc-bbut00025868"	exon_number "2"
19	lncRNA	exon	40834456	40834608	.	+	.	gene_id "lnc-bbug11276"	transcript_id "lnc-bbut00025868"	exon_number "3"
19	lncRNA	exon	41224796	41225874	.	+	.	gene_id "lnc-bbug11280"	transcript_id "lnc-bbut00025876"	exon_number "1"
19	lncRNA	exon	41227626	41230124	.	+	.	gene_id "lnc-bbug11280"	transcript_id "lnc-bbut00025876"	exon_number "2"
19	lncRNA	exon	42437769	42438321	.	+	.	gene_id "lnc-bbug11282"	transcript_id "lnc-bbut00025882"	exon_number "1"
19	lncRNA	exon	42562440	42562493	.	+	.	gene_id "lnc-bbug11282"	transcript_id "lnc-bbut00025882"	exon_number "2"
19	lncRNA	exon	42693639	42694775	.	+	.	gene_id "lnc-bbug11282"	transcript_id "lnc-bbut00025882"	exon_number "3"
19	lncRNA	exon	42705837	42705849	.	+	.	gene_id "lnc-bbug11283"	transcript_id "lnc-bbut00025883"	exon_number "1"
19	lncRNA	exon	42707526	42709925	.	+	.	gene_id "lnc-bbug11283"	transcript_id "lnc-bbut00025883"	exon_number "2"
19	lncRNA	exon	43367565	43367643	.	+	.	gene_id "lnc-bbug11284"	transcript_id "lnc-bbut00025884"	exon_number "1"
19	lncRNA	exon	43463723	43463743	.	+	.	gene_id "lnc-bbug11284"	transcript_id "lnc-bbut00025884"	exon_number "2"
19	lncRNA	exon	43465338	43466178	.	+	.	gene_id "lnc-bbug11284"	transcript_id "lnc-bbut00025884"	exon_number "3"
19	lncRNA	exon	48871986	48872126	.	+	.	gene_id "lnc-bbug11290"	transcript_id "lnc-bbut00025890"	exon_number "1"
19	lncRNA	exon	48872648	48873439	.	+	.	gene_id "lnc-bbug11290"	transcript_id "lnc-bbut00025890"	exon_number "2"
19	lncRNA	exon	50058612	50059732	.	+	.	gene_id "lnc-bbug11291"	transcript_id "lnc-bbut00025891"	exon_number "1"
19	lncRNA	exon	50063278	50063348	.	+	.	gene_id "lnc-bbug11291"	transcript_id "lnc-bbut00025891"	exon_number "2"
19	lncRNA	exon	50068713	50069029	.	+	.	gene_id "lnc-bbug11291"	transcript_id "lnc-bbut00025891"	exon_number "3"
19	lncRNA	exon	50359176	50360038	.	+	.	gene_id "lnc-bbug11292"	transcript_id "lnc-bbut00025892"	exon_number "1"
19	lncRNA	exon	52818048	52818391	.	+	.	gene_id "lnc-bbug11293"	transcript_id "lnc-bbut00025893"	exon_number "1"
19	lncRNA	exon	52822091	52822152	.	+	.	gene_id "lnc-bbug11293"	transcript_id "lnc-bbut00025893"	exon_number "2"
19	lncRNA	exon	52825970	52826344	.	+	.	gene_id "lnc-bbug11293"	transcript_id "lnc-bbut00025893"	exon_number "3"
19	lncRNA	exon	54529270	54529465	.	+	.	gene_id "lnc-bbug11295"	transcript_id "lnc-bbut00025898"	exon_number "1"

19	lncRNA	exon	54532307	54532407	.	+	.	gene_id "lnc-bbug11295"	transcript_id "lnc-bbut00025898"	exon_number "2"
19	lncRNA	exon	54567887	54568415	.	+	.	gene_id "lnc-bbug11295"	transcript_id "lnc-bbut00025898"	exon_number "3"
19	lncRNA	exon	55486063	55486723	.	+	.	gene_id "lnc-bbug11297"	transcript_id "lnc-bbut00025900"	exon_number "1"
19	lncRNA	exon	55487195	55488073	.	+	.	gene_id "lnc-bbug11297"	transcript_id "lnc-bbut00025900"	exon_number "2"
19	lncRNA	exon	55579593	55580145	.	+	.	gene_id "lnc-bbug11298"	transcript_id "lnc-bbut00025901"	exon_number "1"
19	lncRNA	exon	55580382	55580514	.	+	.	gene_id "lnc-bbug11298"	transcript_id "lnc-bbut00025901"	exon_number "2"
19	lncRNA	exon	57433367	57433610	.	+	.	gene_id "lnc-bbug11304"	transcript_id "lnc-bbut00025914"	exon_number "1"
19	lncRNA	exon	57435374	57435433	.	+	.	gene_id "lnc-bbug11304"	transcript_id "lnc-bbut00025914"	exon_number "2"
19	lncRNA	exon	57456807	57456955	.	+	.	gene_id "lnc-bbug11304"	transcript_id "lnc-bbut00025914"	exon_number "3"
19	lncRNA	exon	57994772	57994816	.	+	.	gene_id "lnc-bbug11305"	transcript_id "lnc-bbut00025915"	exon_number "1"
19	lncRNA	exon	57995572	57996642	.	+	.	gene_id "lnc-bbug11305"	transcript_id "lnc-bbut00025915"	exon_number "2"
19	lncRNA	exon	62250162	62250356	.	+	.	gene_id "lnc-bbug11311"	transcript_id "lnc-bbut00025925"	exon_number "1"
19	lncRNA	exon	62265583	62265679	.	+	.	gene_id "lnc-bbug11311"	transcript_id "lnc-bbut00025925"	exon_number "2"
19	lncRNA	exon	62314482	62314524	.	+	.	gene_id "lnc-bbug11311"	transcript_id "lnc-bbut00025925"	exon_number "3"
19	lncRNA	exon	62315548	62317437	.	+	.	gene_id "lnc-bbug11311"	transcript_id "lnc-bbut00025925"	exon_number "4"
19	lncRNA	exon	62440868	62440964	.	+	.	gene_id "lnc-bbug11312"	transcript_id "lnc-bbut00025926"	exon_number "1"
19	lncRNA	exon	62442264	62442309	.	+	.	gene_id "lnc-bbug11312"	transcript_id "lnc-bbut00025926"	exon_number "2"
19	lncRNA	exon	62445086	62445895	.	+	.	gene_id "lnc-bbug11312"	transcript_id "lnc-bbut00025926"	exon_number "3"
19	lncRNA	exon	62443963	62444014	.	+	.	gene_id "lnc-bbug11312"	transcript_id "lnc-bbut00025935"	exon_number "1"
19	lncRNA	exon	62445086	62445895	.	+	.	gene_id "lnc-bbug11312"	transcript_id "lnc-bbut00025935"	exon_number "2"
19	lncRNA	exon	62443963	62444014	.	+	.	gene_id "lnc-bbug11312"	transcript_id "lnc-bbut00025934"	exon_number "1"
19	lncRNA	exon	62445086	62445173	.	+	.	gene_id "lnc-bbug11312"	transcript_id "lnc-bbut00025934"	exon_number "2"
19	lncRNA	exon	62445519	62445895	.	+	.	gene_id "lnc-bbug11312"	transcript_id "lnc-bbut00025934"	exon_number "3"
19	lncRNA	exon	62552491	62552918	.	+	.	gene_id "lnc-bbug11313"	transcript_id "lnc-bbut00025936"	exon_number "1"
19	lncRNA	exon	62553254	62553751	.	+	.	gene_id "lnc-bbug11313"	transcript_id "lnc-bbut00025936"	exon_number "2"
19	lncRNA	exon	64048286	64048540	.	+	.	gene_id "lnc-bbug11318"	transcript_id "lnc-bbut00025948"	exon_number "1"
19	lncRNA	exon	64074689	64074804	.	+	.	gene_id "lnc-bbug11318"	transcript_id "lnc-bbut00025948"	exon_number "2"
19	lncRNA	exon	64076767	64076818	.	+	.	gene_id "lnc-bbug11318"	transcript_id "lnc-bbut00025948"	exon_number "3"
19	lncRNA	exon	65155099	65155554	.	+	.	gene_id "lnc-bbug11320"	transcript_id "lnc-bbut00025952"	exon_number "1"
19	lncRNA	exon	65168396	65168512	.	+	.	gene_id "lnc-bbug11320"	transcript_id "lnc-bbut00025952"	exon_number "2"
19	lncRNA	exon	65170686	65171183	.	+	.	gene_id "lnc-bbug11320"	transcript_id "lnc-bbut00025952"	exon_number "3"
19	lncRNA	exon	67964763	67964959	.	+	.	gene_id "lnc-bbug11325"	transcript_id "lnc-bbut00025957"	exon_number "1"
19	lncRNA	exon	67966573	67967610	.	+	.	gene_id "lnc-bbug11325"	transcript_id "lnc-bbut00025957"	exon_number "2"
19	lncRNA	exon	67964849	67964959	.	+	.	gene_id "lnc-bbug11325"	transcript_id "lnc-bbut00025958"	exon_number "1"
19	lncRNA	exon	67967230	67967610	.	+	.	gene_id "lnc-bbug11325"	transcript_id "lnc-bbut00025958"	exon_number "2"
19	lncRNA	exon	68794212	68794531	.	+	.	gene_id "lnc-bbug11326"	transcript_id "lnc-bbut00025959"	exon_number "1"
19	lncRNA	exon	68798666	68798997	.	+	.	gene_id "lnc-bbug11326"	transcript_id "lnc-bbut00025959"	exon_number "2"
19	lncRNA	exon	68803073	68805199	.	+	.	gene_id "lnc-bbug11326"	transcript_id "lnc-bbut00025959"	exon_number "3"
19	lncRNA	exon	68806135	68806348	.	+	.	gene_id "lnc-bbug11326"	transcript_id "lnc-bbut00025959"	exon_number "4"
19	lncRNA	exon	68807133	68807774	.	+	.	gene_id "lnc-bbug11326"	transcript_id "lnc-bbut00025959"	exon_number "5"
19	lncRNA	exon	68794390	68794531	.	+	.	gene_id "lnc-bbug11326"	transcript_id "lnc-bbut00025960"	exon_number "1"
19	lncRNA	exon	68798666	68799434	.	+	.	gene_id "lnc-bbug11326"	transcript_id "lnc-bbut00025960"	exon_number "2"
19	lncRNA	exon	68794481	68794531	.	+	.	gene_id "lnc-bbug11326"	transcript_id "lnc-bbut00025961"	exon_number "1"
19	lncRNA	exon	68798712	68798997	.	+	.	gene_id "lnc-bbug11326"	transcript_id "lnc-bbut00025961"	exon_number "2"
19	lncRNA	exon	68803073	68803112	.	+	.	gene_id "lnc-bbug11326"	transcript_id "lnc-bbut00025961"	exon_number "3"
19	lncRNA	exon	68805641	68806348	.	+	.	gene_id "lnc-bbug11326"	transcript_id "lnc-bbut00025962"	exon_number "1"
19	lncRNA	exon	68807133	68807774	.	+	.	gene_id "lnc-bbug11326"	transcript_id "lnc-bbut00025962"	exon_number "2"
19	lncRNA	exon	69861662	69861680	.	+	.	gene_id "lnc-bbug11327"	transcript_id "lnc-bbut00025963"	exon_number "1"
19	lncRNA	exon	69863554	69864391	.	+	.	gene_id "lnc-bbug11327"	transcript_id "lnc-bbut00025963"	exon_number "2"
19	lncRNA	exon	69866268	69867094	.	+	.	gene_id "lnc-bbug11328"	transcript_id "lnc-bbut00025964"	exon_number "1"
19	lncRNA	exon	69867357	69867548	.	+	.	gene_id "lnc-bbug11328"	transcript_id "lnc-bbut00025964"	exon_number "2"
19	lncRNA	exon	69928285	69928307	.	+	.	gene_id "lnc-bbug11330"	transcript_id "lnc-bbut00025967"	exon_number "1"

19	lncRNA	exon	69942857	69943156	.	+	.	gene_id "lnc-bbug11330"	transcript_id "lnc-bbut00025967"	exon_number "2"
19	lncRNA	exon	70022001	70022499	.	+	.	gene_id "lnc-bbug11331"	transcript_id "lnc-bbut00025968"	exon_number "1"
19	lncRNA	exon	70023115	70023290	.	+	.	gene_id "lnc-bbug11331"	transcript_id "lnc-bbut00025968"	exon_number "2"
19	lncRNA	exon	70024158	70026336	.	+	.	gene_id "lnc-bbug11331"	transcript_id "lnc-bbut00025968"	exon_number "3"
19	lncRNA	exon	70680189	70680267	.	+	.	gene_id "lnc-bbug11333"	transcript_id "lnc-bbut00025971"	exon_number "1"
19	lncRNA	exon	70680553	70681328	.	+	.	gene_id "lnc-bbug11333"	transcript_id "lnc-bbut00025971"	exon_number "2"
19	lncRNA	exon	70752419	70754444	.	+	.	gene_id "lnc-bbug11334"	transcript_id "lnc-bbut00025974"	exon_number "1"
19	lncRNA	exon	70754579	70754752	.	+	.	gene_id "lnc-bbug11334"	transcript_id "lnc-bbut00025974"	exon_number "2"
19	lncRNA	exon	70754960	70756524	.	+	.	gene_id "lnc-bbug11334"	transcript_id "lnc-bbut00025974"	exon_number "3"
19	lncRNA	exon	70756652	70757122	.	+	.	gene_id "lnc-bbug11334"	transcript_id "lnc-bbut00025974"	exon_number "4"
19	lncRNA	exon	70757274	70760503	.	+	.	gene_id "lnc-bbug11334"	transcript_id "lnc-bbut00025974"	exon_number "5"
19	lncRNA	exon	70752419	70753339	.	+	.	gene_id "lnc-bbug11334"	transcript_id "lnc-bbut00025973"	exon_number "1"
19	lncRNA	exon	70753983	70754444	.	+	.	gene_id "lnc-bbug11334"	transcript_id "lnc-bbut00025973"	exon_number "2"
19	lncRNA	exon	70754579	70756524	.	+	.	gene_id "lnc-bbug11334"	transcript_id "lnc-bbut00025973"	exon_number "3"
19	lncRNA	exon	70756652	70757122	.	+	.	gene_id "lnc-bbug11334"	transcript_id "lnc-bbut00025973"	exon_number "4"
19	lncRNA	exon	70757274	70760503	.	+	.	gene_id "lnc-bbug11334"	transcript_id "lnc-bbut00025973"	exon_number "5"
19	lncRNA	exon	70752419	70754444	.	+	.	gene_id "lnc-bbug11334"	transcript_id "lnc-bbut00025972"	exon_number "1"
19	lncRNA	exon	70754579	70756524	.	+	.	gene_id "lnc-bbug11334"	transcript_id "lnc-bbut00025972"	exon_number "2"
19	lncRNA	exon	70756652	70757122	.	+	.	gene_id "lnc-bbug11334"	transcript_id "lnc-bbut00025972"	exon_number "3"
19	lncRNA	exon	70757274	70760503	.	+	.	gene_id "lnc-bbug11334"	transcript_id "lnc-bbut00025972"	exon_number "4"
19	lncRNA	exon	71788416	71788508	.	+	.	gene_id "lnc-bbug11346"	transcript_id "lnc-bbut00026009"	exon_number "1"
19	lncRNA	exon	71788591	71789207	.	+	.	gene_id "lnc-bbug11346"	transcript_id "lnc-bbut00026009"	exon_number "2"
19	lncRNA	exon	71796434	71796543	.	+	.	gene_id "lnc-bbug11347"	transcript_id "lnc-bbut00026010"	exon_number "1"
19	lncRNA	exon	71796756	71796846	.	+	.	gene_id "lnc-bbug11347"	transcript_id "lnc-bbut00026010"	exon_number "2"
19	lncRNA	exon	71798027	71798067	.	+	.	gene_id "lnc-bbug11347"	transcript_id "lnc-bbut00026010"	exon_number "3"
19	lncRNA	exon	71800028	71800119	.	+	.	gene_id "lnc-bbug11347"	transcript_id "lnc-bbut00026010"	exon_number "4"
19	lncRNA	exon	71800203	71800817	.	+	.	gene_id "lnc-bbug11347"	transcript_id "lnc-bbut00026010"	exon_number "5"
19	lncRNA	exon	71815517	71816260	.	+	.	gene_id "lnc-bbug11349"	transcript_id "lnc-bbut00026012"	exon_number "1"
19	lncRNA	exon	71816343	71816987	.	+	.	gene_id "lnc-bbug11349"	transcript_id "lnc-bbut00026012"	exon_number "2"
19	lncRNA	exon	999236	999952	.	-	.	gene_id "lnc-bbug11355"	transcript_id "lnc-bbut00026019"	exon_number "1"
19	lncRNA	exon	1184844	1184920	.	-	.	gene_id "lnc-bbug11355"	transcript_id "lnc-bbut00026019"	exon_number "2"
19	lncRNA	exon	1223113	1223218	.	-	.	gene_id "lnc-bbug11355"	transcript_id "lnc-bbut00026019"	exon_number "3"
19	lncRNA	exon	1243875	1243960	.	-	.	gene_id "lnc-bbug11355"	transcript_id "lnc-bbut00026019"	exon_number "4"
19	lncRNA	exon	1258234	1258280	.	-	.	gene_id "lnc-bbug11355"	transcript_id "lnc-bbut00026019"	exon_number "5"
19	lncRNA	exon	1259899	1260019	.	-	.	gene_id "lnc-bbug11355"	transcript_id "lnc-bbut00026019"	exon_number "6"
19	lncRNA	exon	1152511	1152688	.	-	.	gene_id "lnc-bbug11355"	transcript_id "lnc-bbut00026022"	exon_number "1"
19	lncRNA	exon	1184844	1184920	.	-	.	gene_id "lnc-bbug11355"	transcript_id "lnc-bbut00026022"	exon_number "2"
19	lncRNA	exon	1223113	1223218	.	-	.	gene_id "lnc-bbug11355"	transcript_id "lnc-bbut00026022"	exon_number "3"
19	lncRNA	exon	1243875	1243960	.	-	.	gene_id "lnc-bbug11355"	transcript_id "lnc-bbut00026022"	exon_number "4"
19	lncRNA	exon	1258234	1258280	.	-	.	gene_id "lnc-bbug11355"	transcript_id "lnc-bbut00026022"	exon_number "5"
19	lncRNA	exon	1259899	1260019	.	-	.	gene_id "lnc-bbug11355"	transcript_id "lnc-bbut00026022"	exon_number "6"
19	lncRNA	exon	1152511	1152688	.	-	.	gene_id "lnc-bbug11355"	transcript_id "lnc-bbut00026021"	exon_number "1"
19	lncRNA	exon	1184844	1184920	.	-	.	gene_id "lnc-bbug11355"	transcript_id "lnc-bbut00026021"	exon_number "2"
19	lncRNA	exon	1243875	1243960	.	-	.	gene_id "lnc-bbug11355"	transcript_id "lnc-bbut00026021"	exon_number "3"
19	lncRNA	exon	1258234	1258280	.	-	.	gene_id "lnc-bbug11355"	transcript_id "lnc-bbut00026021"	exon_number "4"
19	lncRNA	exon	1259899	1260019	.	-	.	gene_id "lnc-bbug11355"	transcript_id "lnc-bbut00026021"	exon_number "5"
19	lncRNA	exon	1152511	1152688	.	-	.	gene_id "lnc-bbug11355"	transcript_id "lnc-bbut00026020"	exon_number "1"
19	lncRNA	exon	1184844	1184920	.	-	.	gene_id "lnc-bbug11355"	transcript_id "lnc-bbut00026020"	exon_number "2"
19	lncRNA	exon	1243847	1243960	.	-	.	gene_id "lnc-bbug11355"	transcript_id "lnc-bbut00026020"	exon_number "3"
19	lncRNA	exon	1258234	1258280	.	-	.	gene_id "lnc-bbug11355"	transcript_id "lnc-bbut00026020"	exon_number "4"
19	lncRNA	exon	1259899	1260019	.	-	.	gene_id "lnc-bbug11355"	transcript_id "lnc-bbut00026020"	exon_number "5"
19	lncRNA	exon	1181511	1181559	.	-	.	gene_id "lnc-bbug11355"	transcript_id "lnc-bbut00026023"	exon_number "1"

19	lncRNA	exon	1184844	1184920	.	-	.	gene_id "lnc-bbug11355"	transcript_id "lnc-bbut00026023"	exon_number "2"
19	lncRNA	exon	1223113	1223218	.	-	.	gene_id "lnc-bbug11355"	transcript_id "lnc-bbut00026023"	exon_number "3"
19	lncRNA	exon	1243875	1243960	.	-	.	gene_id "lnc-bbug11355"	transcript_id "lnc-bbut00026023"	exon_number "4"
19	lncRNA	exon	1258234	1258280	.	-	.	gene_id "lnc-bbug11355"	transcript_id "lnc-bbut00026023"	exon_number "5"
19	lncRNA	exon	1259899	1260019	.	-	.	gene_id "lnc-bbug11355"	transcript_id "lnc-bbut00026023"	exon_number "6"
19	lncRNA	exon	1849932	1853919	.	-	.	gene_id "lnc-bbug11357"	transcript_id "lnc-bbut00026025"	exon_number "1"
19	lncRNA	exon	1862823	1862871	.	-	.	gene_id "lnc-bbug11357"	transcript_id "lnc-bbut00026025"	exon_number "2"
19	lncRNA	exon	1863582	1864220	.	-	.	gene_id "lnc-bbug11357"	transcript_id "lnc-bbut00026025"	exon_number "3"
19	lncRNA	exon	3598810	3598961	.	-	.	gene_id "lnc-bbug11362"	transcript_id "lnc-bbut00026043"	exon_number "1"
19	lncRNA	exon	3602542	3603019	.	-	.	gene_id "lnc-bbug11362"	transcript_id "lnc-bbut00026043"	exon_number "2"
19	lncRNA	exon	4104220	4105167	.	-	.	gene_id "lnc-bbug11366"	transcript_id "lnc-bbut00026048"	exon_number "1"
19	lncRNA	exon	4116187	4116594	.	-	.	gene_id "lnc-bbug11366"	transcript_id "lnc-bbut00026048"	exon_number "2"
19	lncRNA	exon	6069817	6071050	.	-	.	gene_id "lnc-bbug11372"	transcript_id "lnc-bbut00026054"	exon_number "1"
19	lncRNA	exon	6653373	6653839	.	-	.	gene_id "lnc-bbug11375"	transcript_id "lnc-bbut00026058"	exon_number "1"
19	lncRNA	exon	6654286	6654674	.	-	.	gene_id "lnc-bbug11375"	transcript_id "lnc-bbut00026058"	exon_number "2"
19	lncRNA	exon	6653373	6653839	.	-	.	gene_id "lnc-bbug11375"	transcript_id "lnc-bbut00026059"	exon_number "1"
19	lncRNA	exon	6654286	6654468	.	-	.	gene_id "lnc-bbug11375"	transcript_id "lnc-bbut00026059"	exon_number "2"
19	lncRNA	exon	6662116	6662154	.	-	.	gene_id "lnc-bbug11375"	transcript_id "lnc-bbut00026059"	exon_number "3"
19	lncRNA	exon	7953865	7956973	.	-	.	gene_id "lnc-bbug11381"	transcript_id "lnc-bbut00026067"	exon_number "1"
19	lncRNA	exon	7975584	7975822	.	-	.	gene_id "lnc-bbug11381"	transcript_id "lnc-bbut00026067"	exon_number "2"
19	lncRNA	exon	8187464	8188821	.	-	.	gene_id "lnc-bbug11383"	transcript_id "lnc-bbut00026069"	exon_number "1"
19	lncRNA	exon	8190147	8190953	.	-	.	gene_id "lnc-bbug11383"	transcript_id "lnc-bbut00026069"	exon_number "2"
19	lncRNA	exon	8477708	8478615	.	-	.	gene_id "lnc-bbug11387"	transcript_id "lnc-bbut00026075"	exon_number "1"
19	lncRNA	exon	8482825	8482964	.	-	.	gene_id "lnc-bbug11387"	transcript_id "lnc-bbut00026075"	exon_number "2"
19	lncRNA	exon	8483074	8484174	.	-	.	gene_id "lnc-bbug11387"	transcript_id "lnc-bbut00026075"	exon_number "3"
19	lncRNA	exon	8652212	8652888	.	-	.	gene_id "lnc-bbug11389"	transcript_id "lnc-bbut00026079"	exon_number "1"
19	lncRNA	exon	9619518	9620063	.	-	.	gene_id "lnc-bbug11392"	transcript_id "lnc-bbut00026086"	exon_number "1"
19	lncRNA	exon	9620922	9621005	.	-	.	gene_id "lnc-bbug11392"	transcript_id "lnc-bbut00026086"	exon_number "2"
19	lncRNA	exon	9621431	9621930	.	-	.	gene_id "lnc-bbug11392"	transcript_id "lnc-bbut00026086"	exon_number "3"
19	lncRNA	exon	10206899	10208195	.	-	.	gene_id "lnc-bbug11402"	transcript_id "lnc-bbut00026107"	exon_number "1"
19	lncRNA	exon	10208239	10208282	.	-	.	gene_id "lnc-bbug11402"	transcript_id "lnc-bbut00026107"	exon_number "2"
19	lncRNA	exon	10218475	10219460	.	-	.	gene_id "lnc-bbug11403"	transcript_id "lnc-bbut00026108"	exon_number "1"
19	lncRNA	exon	10220171	10220358	.	-	.	gene_id "lnc-bbug11403"	transcript_id "lnc-bbut00026108"	exon_number "2"
19	lncRNA	exon	10221303	10221366	.	-	.	gene_id "lnc-bbug11403"	transcript_id "lnc-bbut00026108"	exon_number "3"
19	lncRNA	exon	10225843	10225958	.	-	.	gene_id "lnc-bbug11403"	transcript_id "lnc-bbut00026108"	exon_number "4"
19	lncRNA	exon	10218475	10219460	.	-	.	gene_id "lnc-bbug11403"	transcript_id "lnc-bbut00026109"	exon_number "1"
19	lncRNA	exon	10220171	10220358	.	-	.	gene_id "lnc-bbug11403"	transcript_id "lnc-bbut00026109"	exon_number "2"
19	lncRNA	exon	10221303	10221366	.	-	.	gene_id "lnc-bbug11403"	transcript_id "lnc-bbut00026109"	exon_number "3"
19	lncRNA	exon	10227101	10227242	.	-	.	gene_id "lnc-bbug11403"	transcript_id "lnc-bbut00026109"	exon_number "4"
19	lncRNA	exon	11203133	11204018	.	-	.	gene_id "lnc-bbug11409"	transcript_id "lnc-bbut00026119"	exon_number "1"
19	lncRNA	exon	11204643	11220168	.	-	.	gene_id "lnc-bbug11409"	transcript_id "lnc-bbut00026119"	exon_number "2"
19	lncRNA	exon	11583989	11585160	.	-	.	gene_id "lnc-bbug11410"	transcript_id "lnc-bbut00026120"	exon_number "1"
19	lncRNA	exon	11585795	11585899	.	-	.	gene_id "lnc-bbug11410"	transcript_id "lnc-bbut00026120"	exon_number "2"
19	lncRNA	exon	11645577	11645687	.	-	.	gene_id "lnc-bbug11410"	transcript_id "lnc-bbut00026120"	exon_number "3"
19	lncRNA	exon	11773370	11773440	.	-	.	gene_id "lnc-bbug11410"	transcript_id "lnc-bbut00026120"	exon_number "4"
19	lncRNA	exon	11787181	11787590	.	-	.	gene_id "lnc-bbug11410"	transcript_id "lnc-bbut00026120"	exon_number "5"
19	lncRNA	exon	11899680	11899953	.	-	.	gene_id "lnc-bbug11411"	transcript_id "lnc-bbut00026121"	exon_number "1"
19	lncRNA	exon	12083803	12083883	.	-	.	gene_id "lnc-bbug11411"	transcript_id "lnc-bbut00026121"	exon_number "2"
19	lncRNA	exon	12092879	12094174	.	-	.	gene_id "lnc-bbug11411"	transcript_id "lnc-bbut00026121"	exon_number "3"
19	lncRNA	exon	12094324	12094825	.	-	.	gene_id "lnc-bbug11411"	transcript_id "lnc-bbut00026121"	exon_number "4"
19	lncRNA	exon	11943598	11944907	.	-	.	gene_id "lnc-bbug11412"	transcript_id "lnc-bbut00026122"	exon_number "1"
19	lncRNA	exon	13606260	13607166	.	-	.	gene_id "lnc-bbug11418"	transcript_id "lnc-bbut00026144"	exon_number "1"

19	lncRNA	exon	14400573	14400998	.	-	.	gene_id "lnc-bbug11423"	transcript_id "lnc-bbut00026159"	exon_number "1"
19	lncRNA	exon	16435243	16437651	.	-	.	gene_id "lnc-bbug11427"	transcript_id "lnc-bbut00026165"	exon_number "1"
19	lncRNA	exon	16439680	16440656	.	-	.	gene_id "lnc-bbug11427"	transcript_id "lnc-bbut00026165"	exon_number "2"
19	lncRNA	exon	16505592	16508176	.	-	.	gene_id "lnc-bbug11429"	transcript_id "lnc-bbut00026169"	exon_number "1"
19	lncRNA	exon	16509475	16510775	.	-	.	gene_id "lnc-bbug11429"	transcript_id "lnc-bbut00026169"	exon_number "2"
19	lncRNA	exon	17771962	17774158	.	-	.	gene_id "lnc-bbug11432"	transcript_id "lnc-bbut00026178"	exon_number "1"
19	lncRNA	exon	17774586	17774757	.	-	.	gene_id "lnc-bbug11432"	transcript_id "lnc-bbut00026178"	exon_number "2"
19	lncRNA	exon	17776456	17776613	.	-	.	gene_id "lnc-bbug11432"	transcript_id "lnc-bbut00026178"	exon_number "3"
19	lncRNA	exon	17777438	17777641	.	-	.	gene_id "lnc-bbug11432"	transcript_id "lnc-bbut00026178"	exon_number "4"
19	lncRNA	exon	17853826	17854815	.	-	.	gene_id "lnc-bbug11433"	transcript_id "lnc-bbut00026179"	exon_number "1"
19	lncRNA	exon	17855040	17855855	.	-	.	gene_id "lnc-bbug11433"	transcript_id "lnc-bbut00026179"	exon_number "2"
19	lncRNA	exon	17974319	17975428	.	-	.	gene_id "lnc-bbug11434"	transcript_id "lnc-bbut00026182"	exon_number "1"
19	lncRNA	exon	17978687	17978773	.	-	.	gene_id "lnc-bbug11434"	transcript_id "lnc-bbut00026182"	exon_number "2"
19	lncRNA	exon	17981066	17981269	.	-	.	gene_id "lnc-bbug11434"	transcript_id "lnc-bbut00026182"	exon_number "3"
19	lncRNA	exon	17997567	17997656	.	-	.	gene_id "lnc-bbug11434"	transcript_id "lnc-bbut00026182"	exon_number "4"
19	lncRNA	exon	18108110	18108324	.	-	.	gene_id "lnc-bbug11434"	transcript_id "lnc-bbut00026182"	exon_number "5"
19	lncRNA	exon	17974319	17975428	.	-	.	gene_id "lnc-bbug11434"	transcript_id "lnc-bbut00026181"	exon_number "1"
19	lncRNA	exon	17981066	17981269	.	-	.	gene_id "lnc-bbug11434"	transcript_id "lnc-bbut00026181"	exon_number "2"
19	lncRNA	exon	17997567	17997656	.	-	.	gene_id "lnc-bbug11434"	transcript_id "lnc-bbut00026181"	exon_number "3"
19	lncRNA	exon	18108110	18108324	.	-	.	gene_id "lnc-bbug11434"	transcript_id "lnc-bbut00026181"	exon_number "4"
19	lncRNA	exon	18219596	18223435	.	-	.	gene_id "lnc-bbug11435"	transcript_id "lnc-bbut00026183"	exon_number "1"
19	lncRNA	exon	18375980	18376300	.	-	.	gene_id "lnc-bbug11436"	transcript_id "lnc-bbut00026184"	exon_number "1"
19	lncRNA	exon	18488732	18491455	.	-	.	gene_id "lnc-bbug11437"	transcript_id "lnc-bbut00026185"	exon_number "1"
19	lncRNA	exon	18492751	18492878	.	-	.	gene_id "lnc-bbug11437"	transcript_id "lnc-bbut00026185"	exon_number "2"
19	lncRNA	exon	18494076	18494343	.	-	.	gene_id "lnc-bbug11437"	transcript_id "lnc-bbut00026185"	exon_number "3"
19	lncRNA	exon	19752663	19753564	.	-	.	gene_id "lnc-bbug11439"	transcript_id "lnc-bbut00026187"	exon_number "1"
19	lncRNA	exon	19754014	19754091	.	-	.	gene_id "lnc-bbug11439"	transcript_id "lnc-bbut00026187"	exon_number "2"
19	lncRNA	exon	19776828	19776927	.	-	.	gene_id "lnc-bbug11439"	transcript_id "lnc-bbut00026187"	exon_number "3"
19	lncRNA	exon	20116374	20116719	.	-	.	gene_id "lnc-bbug11440"	transcript_id "lnc-bbut00026188"	exon_number "1"
19	lncRNA	exon	20117637	20117775	.	-	.	gene_id "lnc-bbug11440"	transcript_id "lnc-bbut00026188"	exon_number "2"
19	lncRNA	exon	20118110	20119225	.	-	.	gene_id "lnc-bbug11440"	transcript_id "lnc-bbut00026188"	exon_number "3"
19	lncRNA	exon	21492544	21495178	.	-	.	gene_id "lnc-bbug11443"	transcript_id "lnc-bbut00026198"	exon_number "1"
19	lncRNA	exon	21495266	21495360	.	-	.	gene_id "lnc-bbug11443"	transcript_id "lnc-bbut00026198"	exon_number "2"
19	lncRNA	exon	21499539	21499592	.	-	.	gene_id "lnc-bbug11443"	transcript_id "lnc-bbut00026198"	exon_number "3"
19	lncRNA	exon	21511180	21511279	.	-	.	gene_id "lnc-bbug11443"	transcript_id "lnc-bbut00026198"	exon_number "4"
19	lncRNA	exon	21579306	21579413	.	-	.	gene_id "lnc-bbug11444"	transcript_id "lnc-bbut00026199"	exon_number "1"
19	lncRNA	exon	21591092	21591201	.	-	.	gene_id "lnc-bbug11444"	transcript_id "lnc-bbut00026199"	exon_number "2"
19	lncRNA	exon	21591928	21592182	.	-	.	gene_id "lnc-bbug11444"	transcript_id "lnc-bbut00026199"	exon_number "3"
19	lncRNA	exon	23661883	23662739	.	-	.	gene_id "lnc-bbug11452"	transcript_id "lnc-bbut00026219"	exon_number "1"
19	lncRNA	exon	23663432	23663847	.	-	.	gene_id "lnc-bbug11452"	transcript_id "lnc-bbut00026219"	exon_number "2"
19	lncRNA	exon	23675575	23676145	.	-	.	gene_id "lnc-bbug11453"	transcript_id "lnc-bbut00026220"	exon_number "1"
19	lncRNA	exon	23678311	23678423	.	-	.	gene_id "lnc-bbug11453"	transcript_id "lnc-bbut00026220"	exon_number "2"
19	lncRNA	exon	23688625	23688728	.	-	.	gene_id "lnc-bbug11453"	transcript_id "lnc-bbut00026220"	exon_number "3"
19	lncRNA	exon	23694792	23694936	.	-	.	gene_id "lnc-bbug11453"	transcript_id "lnc-bbut00026220"	exon_number "4"
19	lncRNA	exon	23709131	23709728	.	-	.	gene_id "lnc-bbug11454"	transcript_id "lnc-bbut00026222"	exon_number "1"
19	lncRNA	exon	23713726	23713972	.	-	.	gene_id "lnc-bbug11454"	transcript_id "lnc-bbut00026222"	exon_number "2"
19	lncRNA	exon	28423806	28423886	.	-	.	gene_id "lnc-bbug11467"	transcript_id "lnc-bbut00026240"	exon_number "1"
19	lncRNA	exon	28424483	28424544	.	-	.	gene_id "lnc-bbug11467"	transcript_id "lnc-bbut00026240"	exon_number "2"
19	lncRNA	exon	28427281	28427465	.	-	.	gene_id "lnc-bbug11467"	transcript_id "lnc-bbut00026240"	exon_number "3"
19	lncRNA	exon	28442537	28442783	.	-	.	gene_id "lnc-bbug11467"	transcript_id "lnc-bbut00026240"	exon_number "4"
19	lncRNA	exon	28423806	28423907	.	-	.	gene_id "lnc-bbug11467"	transcript_id "lnc-bbut00026241"	exon_number "1"
19	lncRNA	exon	28424483	28424544	.	-	.	gene_id "lnc-bbug11467"	transcript_id "lnc-bbut00026241"	exon_number "2"

19	lncRNA	exon	28427281	28427465	.	-	.	gene_id "lnc-bbug11467"	transcript_id "lnc-bbut00026241"	exon_number "3"
19	lncRNA	exon	28442537	28442784	.	-	.	gene_id "lnc-bbug11467"	transcript_id "lnc-bbut00026241"	exon_number "4"
19	lncRNA	exon	28596457	28597839	.	-	.	gene_id "lnc-bbug11468"	transcript_id "lnc-bbut00026242"	exon_number "1"
19	lncRNA	exon	31377304	31378224	.	-	.	gene_id "lnc-bbug11474"	transcript_id "lnc-bbut00026258"	exon_number "1"
19	lncRNA	exon	34115355	34115849	.	-	.	gene_id "lnc-bbug11483"	transcript_id "lnc-bbut00026279"	exon_number "1"
19	lncRNA	exon	34118121	34118710	.	-	.	gene_id "lnc-bbug11483"	transcript_id "lnc-bbut00026279"	exon_number "2"
19	lncRNA	exon	34889791	34891657	.	-	.	gene_id "lnc-bbug11485"	transcript_id "lnc-bbut00026281"	exon_number "1"
19	lncRNA	exon	34934191	34934358	.	-	.	gene_id "lnc-bbug11485"	transcript_id "lnc-bbut00026281"	exon_number "2"
19	lncRNA	exon	34938939	34939050	.	-	.	gene_id "lnc-bbug11485"	transcript_id "lnc-bbut00026281"	exon_number "3"
19	lncRNA	exon	35396181	35397881	.	-	.	gene_id "lnc-bbug11487"	transcript_id "lnc-bbut00026291"	exon_number "1"
19	lncRNA	exon	35400834	35400970	.	-	.	gene_id "lnc-bbug11487"	transcript_id "lnc-bbut00026291"	exon_number "2"
19	lncRNA	exon	35401684	35401801	.	-	.	gene_id "lnc-bbug11487"	transcript_id "lnc-bbut00026291"	exon_number "3"
19	lncRNA	exon	35396181	35397881	.	-	.	gene_id "lnc-bbug11487"	transcript_id "lnc-bbut00026290"	exon_number "1"
19	lncRNA	exon	35400808	35400970	.	-	.	gene_id "lnc-bbug11487"	transcript_id "lnc-bbut00026290"	exon_number "2"
19	lncRNA	exon	35401684	35401801	.	-	.	gene_id "lnc-bbug11487"	transcript_id "lnc-bbut00026290"	exon_number "3"
19	lncRNA	exon	40027661	40028542	.	-	.	gene_id "lnc-bbug11502"	transcript_id "lnc-bbut00026331"	exon_number "1"
19	lncRNA	exon	40029358	40029448	.	-	.	gene_id "lnc-bbug11502"	transcript_id "lnc-bbut00026331"	exon_number "2"
19	lncRNA	exon	40672381	40673252	.	-	.	gene_id "lnc-bbug11504"	transcript_id "lnc-bbut00026334"	exon_number "1"
19	lncRNA	exon	40678277	40678406	.	-	.	gene_id "lnc-bbug11504"	transcript_id "lnc-bbut00026334"	exon_number "2"
19	lncRNA	exon	40685967	40686310	.	-	.	gene_id "lnc-bbug11504"	transcript_id "lnc-bbut00026334"	exon_number "3"
19	lncRNA	exon	40803897	40804436	.	-	.	gene_id "lnc-bbug11506"	transcript_id "lnc-bbut00026337"	exon_number "1"
19	lncRNA	exon	40804778	40806382	.	-	.	gene_id "lnc-bbug11506"	transcript_id "lnc-bbut00026337"	exon_number "2"
19	lncRNA	exon	40849062	40852065	.	-	.	gene_id "lnc-bbug11507"	transcript_id "lnc-bbut00026338"	exon_number "1"
19	lncRNA	exon	40853751	40853859	.	-	.	gene_id "lnc-bbug11507"	transcript_id "lnc-bbut00026338"	exon_number "2"
19	lncRNA	exon	40864739	40864861	.	-	.	gene_id "lnc-bbug11507"	transcript_id "lnc-bbut00026338"	exon_number "3"
19	lncRNA	exon	40868367	40868439	.	-	.	gene_id "lnc-bbug11507"	transcript_id "lnc-bbut00026338"	exon_number "4"
19	lncRNA	exon	40870996	40871122	.	-	.	gene_id "lnc-bbug11507"	transcript_id "lnc-bbut00026338"	exon_number "5"
19	lncRNA	exon	41020448	41020655	.	-	.	gene_id "lnc-bbug11508"	transcript_id "lnc-bbut00026339"	exon_number "1"
19	lncRNA	exon	41024377	41024684	.	-	.	gene_id "lnc-bbug11508"	transcript_id "lnc-bbut00026339"	exon_number "2"
19	lncRNA	exon	41222187	41222635	.	-	.	gene_id "lnc-bbug11510"	transcript_id "lnc-bbut00026341"	exon_number "1"
19	lncRNA	exon	41223680	41223803	.	-	.	gene_id "lnc-bbug11510"	transcript_id "lnc-bbut00026341"	exon_number "2"
19	lncRNA	exon	43929076	43929596	.	-	.	gene_id "lnc-bbug11515"	transcript_id "lnc-bbut00026357"	exon_number "1"
19	lncRNA	exon	44779757	44780489	.	-	.	gene_id "lnc-bbug11516"	transcript_id "lnc-bbut00026358"	exon_number "1"
19	lncRNA	exon	48855676	48856521	.	-	.	gene_id "lnc-bbug11518"	transcript_id "lnc-bbut00026360"	exon_number "1"
19	lncRNA	exon	49658927	49659947	.	-	.	gene_id "lnc-bbug11519"	transcript_id "lnc-bbut00026361"	exon_number "1"
19	lncRNA	exon	54471526	54472464	.	-	.	gene_id "lnc-bbug11523"	transcript_id "lnc-bbut00026365"	exon_number "1"
19	lncRNA	exon	55409106	55410597	.	-	.	gene_id "lnc-bbug11526"	transcript_id "lnc-bbut00026368"	exon_number "1"
19	lncRNA	exon	55421082	55421160	.	-	.	gene_id "lnc-bbug11526"	transcript_id "lnc-bbut00026368"	exon_number "2"
19	lncRNA	exon	55498965	55502803	.	-	.	gene_id "lnc-bbug11528"	transcript_id "lnc-bbut00026371"	exon_number "1"
19	lncRNA	exon	55504647	55508733	.	-	.	gene_id "lnc-bbug11528"	transcript_id "lnc-bbut00026371"	exon_number "2"
19	lncRNA	exon	55498965	55502423	.	-	.	gene_id "lnc-bbug11528"	transcript_id "lnc-bbut00026370"	exon_number "1"
19	lncRNA	exon	55502523	55508733	.	-	.	gene_id "lnc-bbug11528"	transcript_id "lnc-bbut00026370"	exon_number "2"
19	lncRNA	exon	55518499	55518605	.	-	.	gene_id "lnc-bbug11529"	transcript_id "lnc-bbut00026372"	exon_number "1"
19	lncRNA	exon	55523074	55523189	.	-	.	gene_id "lnc-bbug11529"	transcript_id "lnc-bbut00026372"	exon_number "2"
19	lncRNA	exon	55523645	55523872	.	-	.	gene_id "lnc-bbug11529"	transcript_id "lnc-bbut00026372"	exon_number "3"
19	lncRNA	exon	56697581	56697974	.	-	.	gene_id "lnc-bbug11531"	transcript_id "lnc-bbut00026377"	exon_number "1"
19	lncRNA	exon	56702100	56703840	.	-	.	gene_id "lnc-bbug11531"	transcript_id "lnc-bbut00026377"	exon_number "2"
19	lncRNA	exon	56703997	56704308	.	-	.	gene_id "lnc-bbug11531"	transcript_id "lnc-bbut00026377"	exon_number "3"
19	lncRNA	exon	58319559	58321449	.	-	.	gene_id "lnc-bbug11534"	transcript_id "lnc-bbut00026384"	exon_number "1"
19	lncRNA	exon	58321898	58325202	.	-	.	gene_id "lnc-bbug11534"	transcript_id "lnc-bbut00026384"	exon_number "2"
19	lncRNA	exon	62188316	62188698	.	-	.	gene_id "lnc-bbug11537"	transcript_id "lnc-bbut00026389"	exon_number "1"
19	lncRNA	exon	62233061	62233143	.	-	.	gene_id "lnc-bbug11537"	transcript_id "lnc-bbut00026389"	exon_number "2"

19	lncRNA	exon	62249918	62249946	.	-	.	gene_id "lnc-bbug11537"	transcript_id "lnc-bbut00026389"	exon_number "3"
19	lncRNA	exon	62519688	62520484	.	-	.	gene_id "lnc-bbug11539"	transcript_id "lnc-bbut00026392"	exon_number "1"
19	lncRNA	exon	62521337	62521479	.	-	.	gene_id "lnc-bbug11539"	transcript_id "lnc-bbut00026392"	exon_number "2"
19	lncRNA	exon	62530345	62532562	.	-	.	gene_id "lnc-bbug11539"	transcript_id "lnc-bbut00026392"	exon_number "3"
19	lncRNA	exon	63212024	63212754	.	-	.	gene_id "lnc-bbug11543"	transcript_id "lnc-bbut00026396"	exon_number "1"
19	lncRNA	exon	63213477	63213634	.	-	.	gene_id "lnc-bbug11543"	transcript_id "lnc-bbut00026396"	exon_number "2"
19	lncRNA	exon	63214933	63215074	.	-	.	gene_id "lnc-bbug11543"	transcript_id "lnc-bbut00026396"	exon_number "3"
19	lncRNA	exon	63408321	63409710	.	-	.	gene_id "lnc-bbug11544"	transcript_id "lnc-bbut00026397"	exon_number "1"
19	lncRNA	exon	63412849	63413186	.	-	.	gene_id "lnc-bbug11544"	transcript_id "lnc-bbut00026397"	exon_number "2"
19	lncRNA	exon	64167095	64167462	.	-	.	gene_id "lnc-bbug11547"	transcript_id "lnc-bbut00026400"	exon_number "1"
19	lncRNA	exon	66703794	66704401	.	-	.	gene_id "lnc-bbug11553"	transcript_id "lnc-bbut00026413"	exon_number "1"
19	lncRNA	exon	66706525	66706645	.	-	.	gene_id "lnc-bbug11553"	transcript_id "lnc-bbut00026413"	exon_number "2"
19	lncRNA	exon	66707557	66707719	.	-	.	gene_id "lnc-bbug11553"	transcript_id "lnc-bbut00026413"	exon_number "3"
19	lncRNA	exon	70198872	70199319	.	-	.	gene_id "lnc-bbug11559"	transcript_id "lnc-bbut00026422"	exon_number "1"
19	lncRNA	exon	70657595	70657786	.	-	.	gene_id "lnc-bbug11561"	transcript_id "lnc-bbut00026424"	exon_number "1"
19	lncRNA	exon	70658149	70658685	.	-	.	gene_id "lnc-bbug11561"	transcript_id "lnc-bbut00026424"	exon_number "2"
19	lncRNA	exon	70669132	70669880	.	-	.	gene_id "lnc-bbug11562"	transcript_id "lnc-bbut00026425"	exon_number "1"
19	lncRNA	exon	70670698	70670888	.	-	.	gene_id "lnc-bbug11562"	transcript_id "lnc-bbut00026425"	exon_number "2"
19	lncRNA	exon	70670976	70671410	.	-	.	gene_id "lnc-bbug11563"	transcript_id "lnc-bbut00026426"	exon_number "1"
19	lncRNA	exon	70671937	70672059	.	-	.	gene_id "lnc-bbug11563"	transcript_id "lnc-bbut00026426"	exon_number "2"
19	lncRNA	exon	70675273	70675326	.	-	.	gene_id "lnc-bbug11563"	transcript_id "lnc-bbut00026426"	exon_number "3"
19	lncRNA	exon	70676189	70676360	.	-	.	gene_id "lnc-bbug11563"	transcript_id "lnc-bbut00026426"	exon_number "4"
19	lncRNA	exon	71159316	71159426	.	-	.	gene_id "lnc-bbug11568"	transcript_id "lnc-bbut00026434"	exon_number "1"
19	lncRNA	exon	71160084	71160860	.	-	.	gene_id "lnc-bbug11568"	transcript_id "lnc-bbut00026434"	exon_number "2"
19	lncRNA	exon	71161338	71161537	.	-	.	gene_id "lnc-bbug11568"	transcript_id "lnc-bbut00026434"	exon_number "3"
19	lncRNA	exon	71169134	71169273	.	-	.	gene_id "lnc-bbug11568"	transcript_id "lnc-bbut00026434"	exon_number "4"
19	lncRNA	exon	71159316	71160860	.	-	.	gene_id "lnc-bbug11568"	transcript_id "lnc-bbut00026433"	exon_number "1"
19	lncRNA	exon	71161338	71161537	.	-	.	gene_id "lnc-bbug11568"	transcript_id "lnc-bbut00026433"	exon_number "2"
19	lncRNA	exon	71169134	71169273	.	-	.	gene_id "lnc-bbug11568"	transcript_id "lnc-bbut00026433"	exon_number "3"
19	lncRNA	exon	71212680	71213364	.	-	.	gene_id "lnc-bbug11570"	transcript_id "lnc-bbut00026436"	exon_number "1"
19	lncRNA	exon	71214695	71215461	.	-	.	gene_id "lnc-bbug11570"	transcript_id "lnc-bbut00026436"	exon_number "2"
19	lncRNA	exon	71682208	71682230	.	-	.	gene_id "lnc-bbug11579"	transcript_id "lnc-bbut00026464"	exon_number "1"
19	lncRNA	exon	71684374	71685388	.	-	.	gene_id "lnc-bbug11579"	transcript_id "lnc-bbut00026464"	exon_number "2"
2	lncRNA	exon	134390	134979	.	+	.	gene_id "lnc-bbug11581"	transcript_id "lnc-bbut00026469"	exon_number "1"
2	lncRNA	exon	145444	145477	.	+	.	gene_id "lnc-bbug11581"	transcript_id "lnc-bbut00026469"	exon_number "2"
2	lncRNA	exon	150209	150291	.	+	.	gene_id "lnc-bbug11581"	transcript_id "lnc-bbut00026469"	exon_number "3"
2	lncRNA	exon	191201	191392	.	+	.	gene_id "lnc-bbug11581"	transcript_id "lnc-bbut00026469"	exon_number "4"
2	lncRNA	exon	191499	200808	.	+	.	gene_id "lnc-bbug11581"	transcript_id "lnc-bbut00026469"	exon_number "5"
2	lncRNA	exon	416144	416235	.	+	.	gene_id "lnc-bbug11583"	transcript_id "lnc-bbut00026482"	exon_number "1"
2	lncRNA	exon	416835	416893	.	+	.	gene_id "lnc-bbug11583"	transcript_id "lnc-bbut00026482"	exon_number "2"
2	lncRNA	exon	423592	424994	.	+	.	gene_id "lnc-bbug11583"	transcript_id "lnc-bbut00026482"	exon_number "3"
2	lncRNA	exon	923823	924300	.	+	.	gene_id "lnc-bbug11587"	transcript_id "lnc-bbut00026486"	exon_number "1"
2	lncRNA	exon	924514	924812	.	+	.	gene_id "lnc-bbug11587"	transcript_id "lnc-bbut00026486"	exon_number "2"
2	lncRNA	exon	1708417	1708635	.	+	.	gene_id "lnc-bbug11590"	transcript_id "lnc-bbut00026489"	exon_number "1"
2	lncRNA	exon	1711046	1711154	.	+	.	gene_id "lnc-bbug11590"	transcript_id "lnc-bbut00026489"	exon_number "2"
2	lncRNA	exon	1711551	1712878	.	+	.	gene_id "lnc-bbug11590"	transcript_id "lnc-bbut00026489"	exon_number "3"
2	lncRNA	exon	1713527	1714654	.	+	.	gene_id "lnc-bbug11590"	transcript_id "lnc-bbut00026489"	exon_number "4"
2	lncRNA	exon	1740056	1740295	.	+	.	gene_id "lnc-bbug11591"	transcript_id "lnc-bbut00026490"	exon_number "1"
2	lncRNA	exon	1740582	1744029	.	+	.	gene_id "lnc-bbug11591"	transcript_id "lnc-bbut00026490"	exon_number "2"
2	lncRNA	exon	1745370	1746968	.	+	.	gene_id "lnc-bbug11591"	transcript_id "lnc-bbut00026490"	exon_number "3"
2	lncRNA	exon	1740273	1740389	.	+	.	gene_id "lnc-bbug11591"	transcript_id "lnc-bbut00026494"	exon_number "1"
2	lncRNA	exon	1740582	1744029	.	+	.	gene_id "lnc-bbug11591"	transcript_id "lnc-bbut00026494"	exon_number "2"

2	lncRNA	exon	1745370	1745821	.	+	.	gene_id "lnc-bbug11591"	transcript_id "lnc-bbut00026494"	exon_number "3"
2	lncRNA	exon	1746177	1746968	.	+	.	gene_id "lnc-bbug11591"	transcript_id "lnc-bbut00026494"	exon_number "4"
2	lncRNA	exon	1740273	1740389	.	+	.	gene_id "lnc-bbug11591"	transcript_id "lnc-bbut00026493"	exon_number "1"
2	lncRNA	exon	1740582	1744029	.	+	.	gene_id "lnc-bbug11591"	transcript_id "lnc-bbut00026493"	exon_number "2"
2	lncRNA	exon	1745370	1745661	.	+	.	gene_id "lnc-bbug11591"	transcript_id "lnc-bbut00026493"	exon_number "3"
2	lncRNA	exon	1746177	1746968	.	+	.	gene_id "lnc-bbug11591"	transcript_id "lnc-bbut00026493"	exon_number "4"
2	lncRNA	exon	1740273	1740389	.	+	.	gene_id "lnc-bbug11591"	transcript_id "lnc-bbut00026492"	exon_number "1"
2	lncRNA	exon	1740582	1744029	.	+	.	gene_id "lnc-bbug11591"	transcript_id "lnc-bbut00026492"	exon_number "2"
2	lncRNA	exon	1745489	1746968	.	+	.	gene_id "lnc-bbug11591"	transcript_id "lnc-bbut00026492"	exon_number "3"
2	lncRNA	exon	1740273	1740389	.	+	.	gene_id "lnc-bbug11591"	transcript_id "lnc-bbut00026491"	exon_number "1"
2	lncRNA	exon	1740582	1744029	.	+	.	gene_id "lnc-bbug11591"	transcript_id "lnc-bbut00026491"	exon_number "2"
2	lncRNA	exon	1745370	1746968	.	+	.	gene_id "lnc-bbug11591"	transcript_id "lnc-bbut00026491"	exon_number "3"
2	lncRNA	exon	1858051	1859166	.	+	.	gene_id "lnc-bbug11596"	transcript_id "lnc-bbut00026500"	exon_number "1"
2	lncRNA	exon	1870411	1870672	.	+	.	gene_id "lnc-bbug11596"	transcript_id "lnc-bbut00026500"	exon_number "2"
2	lncRNA	exon	1871225	1873791	.	+	.	gene_id "lnc-bbug11596"	transcript_id "lnc-bbut00026500"	exon_number "3"
2	lncRNA	exon	2183940	2184274	.	+	.	gene_id "lnc-bbug11600"	transcript_id "lnc-bbut00026507"	exon_number "1"
2	lncRNA	exon	2184405	2185352	.	+	.	gene_id "lnc-bbug11600"	transcript_id "lnc-bbut00026507"	exon_number "2"
2	lncRNA	exon	2189497	2190588	.	+	.	gene_id "lnc-bbug11600"	transcript_id "lnc-bbut00026507"	exon_number "3"
2	lncRNA	exon	2183940	2184274	.	+	.	gene_id "lnc-bbug11600"	transcript_id "lnc-bbut00026506"	exon_number "1"
2	lncRNA	exon	2184405	2185322	.	+	.	gene_id "lnc-bbug11600"	transcript_id "lnc-bbut00026506"	exon_number "2"
2	lncRNA	exon	2189497	2190588	.	+	.	gene_id "lnc-bbug11600"	transcript_id "lnc-bbut00026506"	exon_number "3"
2	lncRNA	exon	2187371	2188849	.	+	.	gene_id "lnc-bbug11600"	transcript_id "lnc-bbut00026509"	exon_number "1"
2	lncRNA	exon	2189497	2190588	.	+	.	gene_id "lnc-bbug11600"	transcript_id "lnc-bbut00026509"	exon_number "2"
2	lncRNA	exon	2187371	2188052	.	+	.	gene_id "lnc-bbug11600"	transcript_id "lnc-bbut00026508"	exon_number "1"
2	lncRNA	exon	2189497	2190588	.	+	.	gene_id "lnc-bbug11600"	transcript_id "lnc-bbut00026508"	exon_number "2"
2	lncRNA	exon	2392180	2392333	.	+	.	gene_id "lnc-bbug11603"	transcript_id "lnc-bbut00026528"	exon_number "1"
2	lncRNA	exon	2398004	2398544	.	+	.	gene_id "lnc-bbug11603"	transcript_id "lnc-bbut00026528"	exon_number "2"
2	lncRNA	exon	2399537	2399957	.	+	.	gene_id "lnc-bbug11604"	transcript_id "lnc-bbut00026529"	exon_number "1"
2	lncRNA	exon	2410402	2410851	.	+	.	gene_id "lnc-bbug11604"	transcript_id "lnc-bbut00026529"	exon_number "2"
2	lncRNA	exon	2510826	2511269	.	+	.	gene_id "lnc-bbug11606"	transcript_id "lnc-bbut00026531"	exon_number "1"
2	lncRNA	exon	2706857	2707209	.	+	.	gene_id "lnc-bbug11607"	transcript_id "lnc-bbut00026532"	exon_number "1"
2	lncRNA	exon	2713626	2713950	.	+	.	gene_id "lnc-bbug11607"	transcript_id "lnc-bbut00026532"	exon_number "2"
2	lncRNA	exon	2714485	2715375	.	+	.	gene_id "lnc-bbug11607"	transcript_id "lnc-bbut00026532"	exon_number "3"
2	lncRNA	exon	2941963	2942615	.	+	.	gene_id "lnc-bbug11610"	transcript_id "lnc-bbut00026537"	exon_number "1"
2	lncRNA	exon	2944857	2947492	.	+	.	gene_id "lnc-bbug11610"	transcript_id "lnc-bbut00026537"	exon_number "2"
2	lncRNA	exon	3134686	3135565	.	+	.	gene_id "lnc-bbug11612"	transcript_id "lnc-bbut00026547"	exon_number "1"
2	lncRNA	exon	3135847	3138779	.	+	.	gene_id "lnc-bbug11612"	transcript_id "lnc-bbut00026547"	exon_number "2"
2	lncRNA	exon	4078120	4078358	.	+	.	gene_id "lnc-bbug11615"	transcript_id "lnc-bbut00026552"	exon_number "1"
2	lncRNA	exon	4079414	4080233	.	+	.	gene_id "lnc-bbug11615"	transcript_id "lnc-bbut00026552"	exon_number "2"
2	lncRNA	exon	4132744	4132896	.	+	.	gene_id "lnc-bbug11616"	transcript_id "lnc-bbut00026553"	exon_number "1"
2	lncRNA	exon	4134132	4134553	.	+	.	gene_id "lnc-bbug11616"	transcript_id "lnc-bbut00026553"	exon_number "2"
2	lncRNA	exon	6520664	6520953	.	+	.	gene_id "lnc-bbug11622"	transcript_id "lnc-bbut00026567"	exon_number "1"
2	lncRNA	exon	6537517	6537589	.	+	.	gene_id "lnc-bbug11622"	transcript_id "lnc-bbut00026567"	exon_number "2"
2	lncRNA	exon	6900360	6900790	.	+	.	gene_id "lnc-bbug11623"	transcript_id "lnc-bbut00026568"	exon_number "1"
2	lncRNA	exon	6901017	6901381	.	+	.	gene_id "lnc-bbug11623"	transcript_id "lnc-bbut00026568"	exon_number "2"
2	lncRNA	exon	7070511	7070622	.	+	.	gene_id "lnc-bbug11626"	transcript_id "lnc-bbut00026577"	exon_number "1"
2	lncRNA	exon	7071627	7071684	.	+	.	gene_id "lnc-bbug11626"	transcript_id "lnc-bbut00026577"	exon_number "2"
2	lncRNA	exon	7071992	7072068	.	+	.	gene_id "lnc-bbug11626"	transcript_id "lnc-bbut00026577"	exon_number "3"
2	lncRNA	exon	7072912	7073013	.	+	.	gene_id "lnc-bbug11626"	transcript_id "lnc-bbut00026577"	exon_number "4"
2	lncRNA	exon	7075735	7075822	.	+	.	gene_id "lnc-bbug11626"	transcript_id "lnc-bbut00026577"	exon_number "5"
2	lncRNA	exon	7076721	7077252	.	+	.	gene_id "lnc-bbug11626"	transcript_id "lnc-bbut00026577"	exon_number "6"
2	lncRNA	exon	7225195	7225384	.	+	.	gene_id "lnc-bbug11628"	transcript_id "lnc-bbut00026579"	exon_number "1"

2	lncRNA	exon	7226994	7227036	.	+	.	gene_id "lnc-bbug11628"	transcript_id "lnc-bbut00026579"	exon_number "2"
2	lncRNA	exon	7228627	7229256	.	+	.	gene_id "lnc-bbug11628"	transcript_id "lnc-bbut00026579"	exon_number "3"
2	lncRNA	exon	7296119	7296272	.	+	.	gene_id "lnc-bbug11629"	transcript_id "lnc-bbut00026580"	exon_number "1"
2	lncRNA	exon	7311615	7311975	.	+	.	gene_id "lnc-bbug11629"	transcript_id "lnc-bbut00026580"	exon_number "2"
2	lncRNA	exon	7571750	7572360	.	+	.	gene_id "lnc-bbug11630"	transcript_id "lnc-bbut00026581"	exon_number "1"
2	lncRNA	exon	7583795	7583994	.	+	.	gene_id "lnc-bbug11630"	transcript_id "lnc-bbut00026581"	exon_number "2"
2	lncRNA	exon	7584951	7585165	.	+	.	gene_id "lnc-bbug11630"	transcript_id "lnc-bbut00026581"	exon_number "3"
2	lncRNA	exon	7571750	7572360	.	+	.	gene_id "lnc-bbug11630"	transcript_id "lnc-bbut00026582"	exon_number "1"
2	lncRNA	exon	7583795	7583994	.	+	.	gene_id "lnc-bbug11630"	transcript_id "lnc-bbut00026582"	exon_number "2"
2	lncRNA	exon	7588181	7588230	.	+	.	gene_id "lnc-bbug11630"	transcript_id "lnc-bbut00026582"	exon_number "3"
2	lncRNA	exon	7649426	7649557	.	+	.	gene_id "lnc-bbug11631"	transcript_id "lnc-bbut00026584"	exon_number "1"
2	lncRNA	exon	7667479	7667649	.	+	.	gene_id "lnc-bbug11631"	transcript_id "lnc-bbut00026584"	exon_number "2"
2	lncRNA	exon	7681143	7682960	.	+	.	gene_id "lnc-bbug11631"	transcript_id "lnc-bbut00026584"	exon_number "3"
2	lncRNA	exon	7684684	7689341	.	+	.	gene_id "lnc-bbug11631"	transcript_id "lnc-bbut00026584"	exon_number "4"
2	lncRNA	exon	7649426	7649557	.	+	.	gene_id "lnc-bbug11631"	transcript_id "lnc-bbut00026583"	exon_number "1"
2	lncRNA	exon	7667479	7667649	.	+	.	gene_id "lnc-bbug11631"	transcript_id "lnc-bbut00026583"	exon_number "2"
2	lncRNA	exon	7681143	7689341	.	+	.	gene_id "lnc-bbug11631"	transcript_id "lnc-bbut00026583"	exon_number "3"
2	lncRNA	exon	7781655	7783956	.	+	.	gene_id "lnc-bbug11632"	transcript_id "lnc-bbut00026586"	exon_number "1"
2	lncRNA	exon	7791623	7792584	.	+	.	gene_id "lnc-bbug11632"	transcript_id "lnc-bbut00026586"	exon_number "2"
2	lncRNA	exon	7868706	7870014	.	+	.	gene_id "lnc-bbug11633"	transcript_id "lnc-bbut00026587"	exon_number "1"
2	lncRNA	exon	7880387	7880432	.	+	.	gene_id "lnc-bbug11633"	transcript_id "lnc-bbut00026587"	exon_number "2"
2	lncRNA	exon	7894141	7894259	.	+	.	gene_id "lnc-bbug11633"	transcript_id "lnc-bbut00026587"	exon_number "3"
2	lncRNA	exon	7896947	7897014	.	+	.	gene_id "lnc-bbug11633"	transcript_id "lnc-bbut00026587"	exon_number "4"
2	lncRNA	exon	7962353	7962529	.	+	.	gene_id "lnc-bbug11634"	transcript_id "lnc-bbut00026588"	exon_number "1"
2	lncRNA	exon	7963012	7964425	.	+	.	gene_id "lnc-bbug11634"	transcript_id "lnc-bbut00026588"	exon_number "2"
2	lncRNA	exon	8009388	8009493	.	+	.	gene_id "lnc-bbug11635"	transcript_id "lnc-bbut00026589"	exon_number "1"
2	lncRNA	exon	8009728	8009816	.	+	.	gene_id "lnc-bbug11635"	transcript_id "lnc-bbut00026589"	exon_number "2"
2	lncRNA	exon	8011070	8012028	.	+	.	gene_id "lnc-bbug11635"	transcript_id "lnc-bbut00026589"	exon_number "3"
2	lncRNA	exon	9389618	9390060	.	+	.	gene_id "lnc-bbug11639"	transcript_id "lnc-bbut00026608"	exon_number "1"
2	lncRNA	exon	9402455	9403044	.	+	.	gene_id "lnc-bbug11639"	transcript_id "lnc-bbut00026608"	exon_number "2"
2	lncRNA	exon	9786179	9789041	.	+	.	gene_id "lnc-bbug11644"	transcript_id "lnc-bbut00026620"	exon_number "1"
2	lncRNA	exon	9790050	9790441	.	+	.	gene_id "lnc-bbug11644"	transcript_id "lnc-bbut00026620"	exon_number "2"
2	lncRNA	exon	9867445	9867760	.	+	.	gene_id "lnc-bbug11645"	transcript_id "lnc-bbut00026621"	exon_number "1"
2	lncRNA	exon	9867962	9869919	.	+	.	gene_id "lnc-bbug11645"	transcript_id "lnc-bbut00026621"	exon_number "2"
2	lncRNA	exon	9879067	9879172	.	+	.	gene_id "lnc-bbug11645"	transcript_id "lnc-bbut00026621"	exon_number "3"
2	lncRNA	exon	9879498	9879584	.	+	.	gene_id "lnc-bbug11645"	transcript_id "lnc-bbut00026621"	exon_number "4"
2	lncRNA	exon	9882803	9883625	.	+	.	gene_id "lnc-bbug11645"	transcript_id "lnc-bbut00026621"	exon_number "5"
2	lncRNA	exon	9867445	9867760	.	+	.	gene_id "lnc-bbug11645"	transcript_id "lnc-bbut00026622"	exon_number "1"
2	lncRNA	exon	9867962	9869919	.	+	.	gene_id "lnc-bbug11645"	transcript_id "lnc-bbut00026622"	exon_number "2"
2	lncRNA	exon	9907763	9907825	.	+	.	gene_id "lnc-bbug11645"	transcript_id "lnc-bbut00026622"	exon_number "3"
2	lncRNA	exon	9919625	9919817	.	+	.	gene_id "lnc-bbug11645"	transcript_id "lnc-bbut00026622"	exon_number "4"
2	lncRNA	exon	9920457	9920654	.	+	.	gene_id "lnc-bbug11645"	transcript_id "lnc-bbut00026622"	exon_number "5"
2	lncRNA	exon	10108392	10108432	.	+	.	gene_id "lnc-bbug11646"	transcript_id "lnc-bbut00026623"	exon_number "1"
2	lncRNA	exon	10109157	10111956	.	+	.	gene_id "lnc-bbug11646"	transcript_id "lnc-bbut00026623"	exon_number "2"
2	lncRNA	exon	10297883	10299318	.	+	.	gene_id "lnc-bbug11647"	transcript_id "lnc-bbut00026624"	exon_number "1"
2	lncRNA	exon	10299954	10300021	.	+	.	gene_id "lnc-bbug11647"	transcript_id "lnc-bbut00026624"	exon_number "2"
2	lncRNA	exon	10303813	10304818	.	+	.	gene_id "lnc-bbug11647"	transcript_id "lnc-bbut00026624"	exon_number "3"
2	lncRNA	exon	11077576	11077704	.	+	.	gene_id "lnc-bbug11649"	transcript_id "lnc-bbut00026628"	exon_number "1"
2	lncRNA	exon	11085817	11085889	.	+	.	gene_id "lnc-bbug11649"	transcript_id "lnc-bbut00026628"	exon_number "2"
2	lncRNA	exon	11086060	11087141	.	+	.	gene_id "lnc-bbug11649"	transcript_id "lnc-bbut00026628"	exon_number "3"
2	lncRNA	exon	11078266	11078459	.	+	.	gene_id "lnc-bbug11649"	transcript_id "lnc-bbut00026629"	exon_number "1"
2	lncRNA	exon	11085817	11085889	.	+	.	gene_id "lnc-bbug11649"	transcript_id "lnc-bbut00026629"	exon_number "2"

2	lncRNA	exon	11086060	11087141	.	+	.	gene_id "lnc-bbug11649"	transcript_id "lnc-bbut00026629"	exon_number "3"
2	lncRNA	exon	11087205	11087834	.	+	.	gene_id "lnc-bbug11650"	transcript_id "lnc-bbut00026630"	exon_number "1"
2	lncRNA	exon	11096357	11096790	.	+	.	gene_id "lnc-bbug11650"	transcript_id "lnc-bbut00026630"	exon_number "2"
2	lncRNA	exon	11097032	11097105	.	+	.	gene_id "lnc-bbug11650"	transcript_id "lnc-bbut00026630"	exon_number "3"
2	lncRNA	exon	11097777	11097968	.	+	.	gene_id "lnc-bbug11650"	transcript_id "lnc-bbut00026630"	exon_number "4"
2	lncRNA	exon	12132371	12132448	.	+	.	gene_id "lnc-bbug11653"	transcript_id "lnc-bbut00026645"	exon_number "1"
2	lncRNA	exon	12133060	12133461	.	+	.	gene_id "lnc-bbug11653"	transcript_id "lnc-bbut00026645"	exon_number "2"
2	lncRNA	exon	12144396	12144694	.	+	.	gene_id "lnc-bbug11653"	transcript_id "lnc-bbut00026645"	exon_number "3"
2	lncRNA	exon	12145507	12146375	.	+	.	gene_id "lnc-bbug11653"	transcript_id "lnc-bbut00026645"	exon_number "4"
2	lncRNA	exon	12132532	12133461	.	+	.	gene_id "lnc-bbug11653"	transcript_id "lnc-bbut00026646"	exon_number "1"
2	lncRNA	exon	12144396	12144694	.	+	.	gene_id "lnc-bbug11653"	transcript_id "lnc-bbut00026646"	exon_number "2"
2	lncRNA	exon	12145507	12146375	.	+	.	gene_id "lnc-bbug11653"	transcript_id "lnc-bbut00026646"	exon_number "3"
2	lncRNA	exon	12132532	12133461	.	+	.	gene_id "lnc-bbug11653"	transcript_id "lnc-bbut00026647"	exon_number "1"
2	lncRNA	exon	12144396	12144694	.	+	.	gene_id "lnc-bbug11653"	transcript_id "lnc-bbut00026647"	exon_number "2"
2	lncRNA	exon	12145507	12145679	.	+	.	gene_id "lnc-bbug11653"	transcript_id "lnc-bbut00026647"	exon_number "3"
2	lncRNA	exon	12158234	12158972	.	+	.	gene_id "lnc-bbug11653"	transcript_id "lnc-bbut00026647"	exon_number "4"
2	lncRNA	exon	13388815	13389705	.	+	.	gene_id "lnc-bbug11660"	transcript_id "lnc-bbut00026656"	exon_number "1"
2	lncRNA	exon	13407169	13407297	.	+	.	gene_id "lnc-bbug11660"	transcript_id "lnc-bbut00026656"	exon_number "2"
2	lncRNA	exon	13491750	13493347	.	+	.	gene_id "lnc-bbug11660"	transcript_id "lnc-bbut00026656"	exon_number "3"
2	lncRNA	exon	14409601	14409619	.	+	.	gene_id "lnc-bbug11661"	transcript_id "lnc-bbut00026657"	exon_number "1"
2	lncRNA	exon	14409792	14410183	.	+	.	gene_id "lnc-bbug11661"	transcript_id "lnc-bbut00026657"	exon_number "2"
2	lncRNA	exon	14952657	14953876	.	+	.	gene_id "lnc-bbug11664"	transcript_id "lnc-bbut00026661"	exon_number "1"
2	lncRNA	exon	14957564	14958696	.	+	.	gene_id "lnc-bbug11664"	transcript_id "lnc-bbut00026661"	exon_number "2"
2	lncRNA	exon	14972891	14976474	.	+	.	gene_id "lnc-bbug11665"	transcript_id "lnc-bbut00026662"	exon_number "1"
2	lncRNA	exon	14977481	14978405	.	+	.	gene_id "lnc-bbug11665"	transcript_id "lnc-bbut00026662"	exon_number "2"
2	lncRNA	exon	15772071	15776655	.	+	.	gene_id "lnc-bbug11667"	transcript_id "lnc-bbut00026670"	exon_number "1"
2	lncRNA	exon	15778070	15778191	.	+	.	gene_id "lnc-bbug11667"	transcript_id "lnc-bbut00026670"	exon_number "2"
2	lncRNA	exon	15778911	15781691	.	+	.	gene_id "lnc-bbug11667"	transcript_id "lnc-bbut00026670"	exon_number "3"
2	lncRNA	exon	15839008	15839131	.	+	.	gene_id "lnc-bbug11668"	transcript_id "lnc-bbut00026671"	exon_number "1"
2	lncRNA	exon	15842259	15842368	.	+	.	gene_id "lnc-bbug11668"	transcript_id "lnc-bbut00026671"	exon_number "2"
2	lncRNA	exon	15842732	15843779	.	+	.	gene_id "lnc-bbug11668"	transcript_id "lnc-bbut00026671"	exon_number "3"
2	lncRNA	exon	16063833	16064091	.	+	.	gene_id "lnc-bbug11670"	transcript_id "lnc-bbut00026673"	exon_number "1"
2	lncRNA	exon	16065768	16066014	.	+	.	gene_id "lnc-bbug11670"	transcript_id "lnc-bbut00026673"	exon_number "2"
2	lncRNA	exon	16122436	16122563	.	+	.	gene_id "lnc-bbug11670"	transcript_id "lnc-bbut00026673"	exon_number "3"
2	lncRNA	exon	16163157	16163200	.	+	.	gene_id "lnc-bbug11670"	transcript_id "lnc-bbut00026673"	exon_number "4"
2	lncRNA	exon	16176584	16179621	.	+	.	gene_id "lnc-bbug11670"	transcript_id "lnc-bbut00026673"	exon_number "5"
2	lncRNA	exon	16063833	16064091	.	+	.	gene_id "lnc-bbug11670"	transcript_id "lnc-bbut00026674"	exon_number "1"
2	lncRNA	exon	16065768	16066014	.	+	.	gene_id "lnc-bbug11670"	transcript_id "lnc-bbut00026674"	exon_number "2"
2	lncRNA	exon	16122436	16122563	.	+	.	gene_id "lnc-bbug11670"	transcript_id "lnc-bbut00026674"	exon_number "3"
2	lncRNA	exon	16163157	16163200	.	+	.	gene_id "lnc-bbug11670"	transcript_id "lnc-bbut00026674"	exon_number "4"
2	lncRNA	exon	16245428	16246188	.	+	.	gene_id "lnc-bbug11670"	transcript_id "lnc-bbut00026674"	exon_number "5"
2	lncRNA	exon	17248991	17248996	.	+	.	gene_id "lnc-bbug11674"	transcript_id "lnc-bbut00026679"	exon_number "1"
2	lncRNA	exon	17253122	17253242	.	+	.	gene_id "lnc-bbug11674"	transcript_id "lnc-bbut00026679"	exon_number "2"
2	lncRNA	exon	17257637	17257827	.	+	.	gene_id "lnc-bbug11674"	transcript_id "lnc-bbut00026679"	exon_number "3"
2	lncRNA	exon	17260950	17261057	.	+	.	gene_id "lnc-bbug11674"	transcript_id "lnc-bbut00026679"	exon_number "4"
2	lncRNA	exon	17263214	17263392	.	+	.	gene_id "lnc-bbug11674"	transcript_id "lnc-bbut00026679"	exon_number "5"
2	lncRNA	exon	17265675	17265981	.	+	.	gene_id "lnc-bbug11674"	transcript_id "lnc-bbut00026679"	exon_number "6"
2	lncRNA	exon	17308783	17308934	.	+	.	gene_id "lnc-bbug11675"	transcript_id "lnc-bbut00026680"	exon_number "1"
2	lncRNA	exon	17384819	17384934	.	+	.	gene_id "lnc-bbug11675"	transcript_id "lnc-bbut00026680"	exon_number "2"
2	lncRNA	exon	17387284	17387666	.	+	.	gene_id "lnc-bbug11675"	transcript_id "lnc-bbut00026680"	exon_number "3"
2	lncRNA	exon	18991813	18991944	.	+	.	gene_id "lnc-bbug11691"	transcript_id "lnc-bbut00026701"	exon_number "1"
2	lncRNA	exon	18993632	18993704	.	+	.	gene_id "lnc-bbug11691"	transcript_id "lnc-bbut00026701"	exon_number "2"

2	lncRNA	exon	18998993	18999191	.	+	.	gene_id "lnc-bbug11691"	transcript_id "lnc-bbut00026701"	exon_number "3"
2	lncRNA	exon	19010555	19012205	.	+	.	gene_id "lnc-bbug11691"	transcript_id "lnc-bbut00026701"	exon_number "4"
2	lncRNA	exon	19306710	19310322	.	+	.	gene_id "lnc-bbug11693"	transcript_id "lnc-bbut00026705"	exon_number "1"
2	lncRNA	exon	19310745	19311659	.	+	.	gene_id "lnc-bbug11693"	transcript_id "lnc-bbut00026705"	exon_number "2"
2	lncRNA	exon	19312413	19312494	.	+	.	gene_id "lnc-bbug11693"	transcript_id "lnc-bbut00026705"	exon_number "3"
2	lncRNA	exon	19464270	19464568	.	+	.	gene_id "lnc-bbug11695"	transcript_id "lnc-bbut00026707"	exon_number "1"
2	lncRNA	exon	19465832	19466035	.	+	.	gene_id "lnc-bbug11695"	transcript_id "lnc-bbut00026707"	exon_number "2"
2	lncRNA	exon	19469926	19470384	.	+	.	gene_id "lnc-bbug11695"	transcript_id "lnc-bbut00026707"	exon_number "3"
2	lncRNA	exon	20375051	20375858	.	+	.	gene_id "lnc-bbug11701"	transcript_id "lnc-bbut00026722"	exon_number "1"
2	lncRNA	exon	20498028	20498363	.	+	.	gene_id "lnc-bbug11703"	transcript_id "lnc-bbut00026736"	exon_number "1"
2	lncRNA	exon	20512274	20512399	.	+	.	gene_id "lnc-bbug11703"	transcript_id "lnc-bbut00026736"	exon_number "2"
2	lncRNA	exon	20527484	20527565	.	+	.	gene_id "lnc-bbug11703"	transcript_id "lnc-bbut00026736"	exon_number "3"
2	lncRNA	exon	20528311	20528433	.	+	.	gene_id "lnc-bbug11703"	transcript_id "lnc-bbut00026736"	exon_number "4"
2	lncRNA	exon	20547542	20547724	.	+	.	gene_id "lnc-bbug11703"	transcript_id "lnc-bbut00026736"	exon_number "5"
2	lncRNA	exon	20498126	20498363	.	+	.	gene_id "lnc-bbug11703"	transcript_id "lnc-bbut00026737"	exon_number "1"
2	lncRNA	exon	20512274	20512399	.	+	.	gene_id "lnc-bbug11703"	transcript_id "lnc-bbut00026737"	exon_number "2"
2	lncRNA	exon	20512782	20512969	.	+	.	gene_id "lnc-bbug11703"	transcript_id "lnc-bbut00026737"	exon_number "3"
2	lncRNA	exon	20610279	20610456	.	+	.	gene_id "lnc-bbug11705"	transcript_id "lnc-bbut00026739"	exon_number "1"
2	lncRNA	exon	20611464	20611749	.	+	.	gene_id "lnc-bbug11705"	transcript_id "lnc-bbut00026739"	exon_number "2"
2	lncRNA	exon	20611984	20612049	.	+	.	gene_id "lnc-bbug11705"	transcript_id "lnc-bbut00026739"	exon_number "3"
2	lncRNA	exon	20615010	20615969	.	+	.	gene_id "lnc-bbug11705"	transcript_id "lnc-bbut00026739"	exon_number "4"
2	lncRNA	exon	20651691	20653064	.	+	.	gene_id "lnc-bbug11708"	transcript_id "lnc-bbut00026742"	exon_number "1"
2	lncRNA	exon	20662217	20662946	.	+	.	gene_id "lnc-bbug11709"	transcript_id "lnc-bbut00026743"	exon_number "1"
2	lncRNA	exon	20663691	20664079	.	+	.	gene_id "lnc-bbug11709"	transcript_id "lnc-bbut00026743"	exon_number "2"
2	lncRNA	exon	20708700	20712023	.	+	.	gene_id "lnc-bbug11712"	transcript_id "lnc-bbut00026747"	exon_number "1"
2	lncRNA	exon	20714896	20720008	.	+	.	gene_id "lnc-bbug11712"	transcript_id "lnc-bbut00026747"	exon_number "2"
2	lncRNA	exon	20720276	20721470	.	+	.	gene_id "lnc-bbug11713"	transcript_id "lnc-bbut00026749"	exon_number "1"
2	lncRNA	exon	20725973	20726068	.	+	.	gene_id "lnc-bbug11713"	transcript_id "lnc-bbut00026749"	exon_number "2"
2	lncRNA	exon	20726369	20726601	.	+	.	gene_id "lnc-bbug11713"	transcript_id "lnc-bbut00026749"	exon_number "3"
2	lncRNA	exon	20727257	20727482	.	+	.	gene_id "lnc-bbug11713"	transcript_id "lnc-bbut00026749"	exon_number "4"
2	lncRNA	exon	20728151	20729706	.	+	.	gene_id "lnc-bbug11713"	transcript_id "lnc-bbut00026749"	exon_number "5"
2	lncRNA	exon	20720276	20721470	.	+	.	gene_id "lnc-bbug11713"	transcript_id "lnc-bbut00026748"	exon_number "1"
2	lncRNA	exon	20725973	20726068	.	+	.	gene_id "lnc-bbug11713"	transcript_id "lnc-bbut00026748"	exon_number "2"
2	lncRNA	exon	20726369	20726601	.	+	.	gene_id "lnc-bbug11713"	transcript_id "lnc-bbut00026748"	exon_number "3"
2	lncRNA	exon	20727257	20727449	.	+	.	gene_id "lnc-bbug11713"	transcript_id "lnc-bbut00026748"	exon_number "4"
2	lncRNA	exon	20728151	20729706	.	+	.	gene_id "lnc-bbug11713"	transcript_id "lnc-bbut00026748"	exon_number "5"
2	lncRNA	exon	20797966	20797969	.	+	.	gene_id "lnc-bbug11722"	transcript_id "lnc-bbut00026758"	exon_number "1"
2	lncRNA	exon	20798563	20798952	.	+	.	gene_id "lnc-bbug11722"	transcript_id "lnc-bbut00026758"	exon_number "2"
2	lncRNA	exon	20830856	20832407	.	+	.	gene_id "lnc-bbug11725"	transcript_id "lnc-bbut00026762"	exon_number "1"
2	lncRNA	exon	20832502	20833062	.	+	.	gene_id "lnc-bbug11725"	transcript_id "lnc-bbut00026762"	exon_number "2"
2	lncRNA	exon	20830856	20831438	.	+	.	gene_id "lnc-bbug11725"	transcript_id "lnc-bbut00026761"	exon_number "1"
2	lncRNA	exon	20832502	20833062	.	+	.	gene_id "lnc-bbug11725"	transcript_id "lnc-bbut00026761"	exon_number "2"
2	lncRNA	exon	20955313	20955445	.	+	.	gene_id "lnc-bbug11740"	transcript_id "lnc-bbut00026794"	exon_number "1"
2	lncRNA	exon	20961070	20962308	.	+	.	gene_id "lnc-bbug11740"	transcript_id "lnc-bbut00026794"	exon_number "2"
2	lncRNA	exon	21545591	21545890	.	+	.	gene_id "lnc-bbug11794"	transcript_id "lnc-bbut00026852"	exon_number "1"
2	lncRNA	exon	21547457	21547562	.	+	.	gene_id "lnc-bbug11794"	transcript_id "lnc-bbut00026852"	exon_number "2"
2	lncRNA	exon	21547942	21549186	.	+	.	gene_id "lnc-bbug11794"	transcript_id "lnc-bbut00026852"	exon_number "3"
2	lncRNA	exon	21597156	21597538	.	+	.	gene_id "lnc-bbug11802"	transcript_id "lnc-bbut00026860"	exon_number "1"
2	lncRNA	exon	21641171	21641614	.	+	.	gene_id "lnc-bbug11803"	transcript_id "lnc-bbut00026861"	exon_number "1"
2	lncRNA	exon	21642101	21642498	.	+	.	gene_id "lnc-bbug11804"	transcript_id "lnc-bbut00026862"	exon_number "1"
2	lncRNA	exon	21646054	21646176	.	+	.	gene_id "lnc-bbug11804"	transcript_id "lnc-bbut00026862"	exon_number "2"
2	lncRNA	exon	21647865	21648047	.	+	.	gene_id "lnc-bbug11804"	transcript_id "lnc-bbut00026862"	exon_number "3"

2	lncRNA	exon	22132289	22132834	.	+	.	gene_id "lnc-bbug11818"	transcript_id "lnc-bbut00026877"	exon_number "1"
2	lncRNA	exon	22316250	22316345	.	+	.	gene_id "lnc-bbug11825"	transcript_id "lnc-bbut00026887"	exon_number "1"
2	lncRNA	exon	22318273	22318377	.	+	.	gene_id "lnc-bbug11825"	transcript_id "lnc-bbut00026887"	exon_number "2"
2	lncRNA	exon	22321122	22321892	.	+	.	gene_id "lnc-bbug11825"	transcript_id "lnc-bbut00026887"	exon_number "3"
2	lncRNA	exon	22316497	22316607	.	+	.	gene_id "lnc-bbug11825"	transcript_id "lnc-bbut00026888"	exon_number "1"
2	lncRNA	exon	22318273	22318377	.	+	.	gene_id "lnc-bbug11825"	transcript_id "lnc-bbut00026888"	exon_number "2"
2	lncRNA	exon	22321122	22321892	.	+	.	gene_id "lnc-bbug11825"	transcript_id "lnc-bbut00026888"	exon_number "3"
2	lncRNA	exon	22521779	22521922	.	+	.	gene_id "lnc-bbug11835"	transcript_id "lnc-bbut00026898"	exon_number "1"
2	lncRNA	exon	22522315	22522634	.	+	.	gene_id "lnc-bbug11835"	transcript_id "lnc-bbut00026898"	exon_number "2"
2	lncRNA	exon	22522792	22523034	.	+	.	gene_id "lnc-bbug11835"	transcript_id "lnc-bbut00026898"	exon_number "3"
2	lncRNA	exon	22525696	22526862	.	+	.	gene_id "lnc-bbug11835"	transcript_id "lnc-bbut00026898"	exon_number "4"
2	lncRNA	exon	22555249	22555471	.	+	.	gene_id "lnc-bbug11837"	transcript_id "lnc-bbut00026900"	exon_number "1"
2	lncRNA	exon	22556050	22556263	.	+	.	gene_id "lnc-bbug11837"	transcript_id "lnc-bbut00026900"	exon_number "2"
2	lncRNA	exon	22556444	22558798	.	+	.	gene_id "lnc-bbug11837"	transcript_id "lnc-bbut00026900"	exon_number "3"
2	lncRNA	exon	22612326	22615222	.	+	.	gene_id "lnc-bbug11840"	transcript_id "lnc-bbut00026903"	exon_number "1"
2	lncRNA	exon	22617531	22617688	.	+	.	gene_id "lnc-bbug11840"	transcript_id "lnc-bbut00026903"	exon_number "2"
2	lncRNA	exon	23459602	23460530	.	+	.	gene_id "lnc-bbug11860"	transcript_id "lnc-bbut00026923"	exon_number "1"
2	lncRNA	exon	23599804	23600756	.	+	.	gene_id "lnc-bbug11874"	transcript_id "lnc-bbut00026937"	exon_number "1"
2	lncRNA	exon	23613357	23614284	.	+	.	gene_id "lnc-bbug11876"	transcript_id "lnc-bbut00026939"	exon_number "1"
2	lncRNA	exon	23911293	23911462	.	+	.	gene_id "lnc-bbug11891"	transcript_id "lnc-bbut00026960"	exon_number "1"
2	lncRNA	exon	23912296	23912404	.	+	.	gene_id "lnc-bbug11891"	transcript_id "lnc-bbut00026960"	exon_number "2"
2	lncRNA	exon	23912842	23914094	.	+	.	gene_id "lnc-bbug11891"	transcript_id "lnc-bbut00026960"	exon_number "3"
2	lncRNA	exon	24069831	24070761	.	+	.	gene_id "lnc-bbug11894"	transcript_id "lnc-bbut00026964"	exon_number "1"
2	lncRNA	exon	24071765	24071797	.	+	.	gene_id "lnc-bbug11894"	transcript_id "lnc-bbut00026964"	exon_number "2"
2	lncRNA	exon	24073008	24073708	.	+	.	gene_id "lnc-bbug11894"	transcript_id "lnc-bbut00026964"	exon_number "3"
2	lncRNA	exon	24069835	24070761	.	+	.	gene_id "lnc-bbug11894"	transcript_id "lnc-bbut00026965"	exon_number "1"
2	lncRNA	exon	24071765	24072429	.	+	.	gene_id "lnc-bbug11894"	transcript_id "lnc-bbut00026965"	exon_number "2"
2	lncRNA	exon	24073008	24073708	.	+	.	gene_id "lnc-bbug11894"	transcript_id "lnc-bbut00026965"	exon_number "3"
2	lncRNA	exon	24234047	24234319	.	+	.	gene_id "lnc-bbug11898"	transcript_id "lnc-bbut00026971"	exon_number "1"
2	lncRNA	exon	24244201	24244444	.	+	.	gene_id "lnc-bbug11898"	transcript_id "lnc-bbut00026971"	exon_number "2"
2	lncRNA	exon	24248691	24248846	.	+	.	gene_id "lnc-bbug11898"	transcript_id "lnc-bbut00026971"	exon_number "3"
2	lncRNA	exon	24242851	24242956	.	+	.	gene_id "lnc-bbug11898"	transcript_id "lnc-bbut00026972"	exon_number "1"
2	lncRNA	exon	24244201	24244518	.	+	.	gene_id "lnc-bbug11898"	transcript_id "lnc-bbut00026972"	exon_number "2"
2	lncRNA	exon	24304912	24305551	.	+	.	gene_id "lnc-bbug11900"	transcript_id "lnc-bbut00026974"	exon_number "1"
2	lncRNA	exon	24305749	24305779	.	+	.	gene_id "lnc-bbug11900"	transcript_id "lnc-bbut00026974"	exon_number "2"
2	lncRNA	exon	24310482	24310576	.	+	.	gene_id "lnc-bbug11900"	transcript_id "lnc-bbut00026974"	exon_number "3"
2	lncRNA	exon	24310954	24311134	.	+	.	gene_id "lnc-bbug11900"	transcript_id "lnc-bbut00026974"	exon_number "4"
2	lncRNA	exon	24334795	24334899	.	+	.	gene_id "lnc-bbug11902"	transcript_id "lnc-bbut00026981"	exon_number "1"
2	lncRNA	exon	24335981	24336129	.	+	.	gene_id "lnc-bbug11902"	transcript_id "lnc-bbut00026981"	exon_number "2"
2	lncRNA	exon	24337143	24338000	.	+	.	gene_id "lnc-bbug11902"	transcript_id "lnc-bbut00026981"	exon_number "3"
2	lncRNA	exon	24338417	24339594	.	+	.	gene_id "lnc-bbug11902"	transcript_id "lnc-bbut00026981"	exon_number "4"
2	lncRNA	exon	24452997	24454930	.	+	.	gene_id "lnc-bbug11905"	transcript_id "lnc-bbut00026990"	exon_number "1"
2	lncRNA	exon	24455048	24455152	.	+	.	gene_id "lnc-bbug11905"	transcript_id "lnc-bbut00026990"	exon_number "2"
2	lncRNA	exon	24455567	24455599	.	+	.	gene_id "lnc-bbug11905"	transcript_id "lnc-bbut00026990"	exon_number "3"
2	lncRNA	exon	24455739	24455786	.	+	.	gene_id "lnc-bbug11905"	transcript_id "lnc-bbut00026990"	exon_number "4"
2	lncRNA	exon	24456062	24456066	.	+	.	gene_id "lnc-bbug11905"	transcript_id "lnc-bbut00026990"	exon_number "5"
2	lncRNA	exon	24701963	24702340	.	+	.	gene_id "lnc-bbug11914"	transcript_id "lnc-bbut00027012"	exon_number "1"
2	lncRNA	exon	24703851	24704883	.	+	.	gene_id "lnc-bbug11914"	transcript_id "lnc-bbut00027012"	exon_number "2"
2	lncRNA	exon	24760585	24762020	.	+	.	gene_id "lnc-bbug11916"	transcript_id "lnc-bbut00027014"	exon_number "1"
2	lncRNA	exon	24762664	24762825	.	+	.	gene_id "lnc-bbug11916"	transcript_id "lnc-bbut00027014"	exon_number "2"
2	lncRNA	exon	24762914	24764774	.	+	.	gene_id "lnc-bbug11916"	transcript_id "lnc-bbut00027014"	exon_number "3"
2	lncRNA	exon	24789902	24790482	.	+	.	gene_id "lnc-bbug11917"	transcript_id "lnc-bbut00027015"	exon_number "1"

2	lncRNA	exon	24790586	24790904	.	+	.	gene_id "lnc-bbug11917"	transcript_id "lnc-bbut00027015"	exon_number "2"
2	lncRNA	exon	24928660	24929221	.	+	.	gene_id "lnc-bbug11924"	transcript_id "lnc-bbut00027028"	exon_number "1"
2	lncRNA	exon	24930762	24930836	.	+	.	gene_id "lnc-bbug11925"	transcript_id "lnc-bbut00027029"	exon_number "1"
2	lncRNA	exon	24930972	24931101	.	+	.	gene_id "lnc-bbug11925"	transcript_id "lnc-bbut00027029"	exon_number "2"
2	lncRNA	exon	24933072	24933297	.	+	.	gene_id "lnc-bbug11925"	transcript_id "lnc-bbut00027029"	exon_number "3"
2	lncRNA	exon	24930762	24930836	.	+	.	gene_id "lnc-bbug11925"	transcript_id "lnc-bbut00027030"	exon_number "1"
2	lncRNA	exon	24930972	24931101	.	+	.	gene_id "lnc-bbug11925"	transcript_id "lnc-bbut00027030"	exon_number "2"
2	lncRNA	exon	24933587	24933962	.	+	.	gene_id "lnc-bbug11925"	transcript_id "lnc-bbut00027030"	exon_number "3"
2	lncRNA	exon	24958863	24958893	.	+	.	gene_id "lnc-bbug11927"	transcript_id "lnc-bbut00027032"	exon_number "1"
2	lncRNA	exon	24960038	24961301	.	+	.	gene_id "lnc-bbug11927"	transcript_id "lnc-bbut00027032"	exon_number "2"
2	lncRNA	exon	25073527	25073708	.	+	.	gene_id "lnc-bbug11930"	transcript_id "lnc-bbut00027036"	exon_number "1"
2	lncRNA	exon	25075711	25076134	.	+	.	gene_id "lnc-bbug11930"	transcript_id "lnc-bbut00027036"	exon_number "2"
2	lncRNA	exon	25129559	25129600	.	+	.	gene_id "lnc-bbug11932"	transcript_id "lnc-bbut00027040"	exon_number "1"
2	lncRNA	exon	25130382	25131544	.	+	.	gene_id "lnc-bbug11932"	transcript_id "lnc-bbut00027040"	exon_number "2"
2	lncRNA	exon	25199169	25199226	.	+	.	gene_id "lnc-bbug11934"	transcript_id "lnc-bbut00027042"	exon_number "1"
2	lncRNA	exon	25199333	25199456	.	+	.	gene_id "lnc-bbug11934"	transcript_id "lnc-bbut00027042"	exon_number "2"
2	lncRNA	exon	25201817	25203798	.	+	.	gene_id "lnc-bbug11934"	transcript_id "lnc-bbut00027042"	exon_number "3"
2	lncRNA	exon	25277146	25277164	.	+	.	gene_id "lnc-bbug11940"	transcript_id "lnc-bbut00027053"	exon_number "1"
2	lncRNA	exon	25278036	25278077	.	+	.	gene_id "lnc-bbug11940"	transcript_id "lnc-bbut00027053"	exon_number "2"
2	lncRNA	exon	25278414	25278576	.	+	.	gene_id "lnc-bbug11940"	transcript_id "lnc-bbut00027053"	exon_number "3"
2	lncRNA	exon	25278884	25279090	.	+	.	gene_id "lnc-bbug11940"	transcript_id "lnc-bbut00027053"	exon_number "4"
2	lncRNA	exon	25446375	25447191	.	+	.	gene_id "lnc-bbug11949"	transcript_id "lnc-bbut00027074"	exon_number "1"
2	lncRNA	exon	25447289	25447727	.	+	.	gene_id "lnc-bbug11949"	transcript_id "lnc-bbut00027074"	exon_number "2"
2	lncRNA	exon	25457791	25458363	.	+	.	gene_id "lnc-bbug11950"	transcript_id "lnc-bbut00027075"	exon_number "1"
2	lncRNA	exon	25460155	25460307	.	+	.	gene_id "lnc-bbug11951"	transcript_id "lnc-bbut00027076"	exon_number "1"
2	lncRNA	exon	25460490	25460528	.	+	.	gene_id "lnc-bbug11951"	transcript_id "lnc-bbut00027076"	exon_number "2"
2	lncRNA	exon	25460749	25460788	.	+	.	gene_id "lnc-bbug11951"	transcript_id "lnc-bbut00027076"	exon_number "3"
2	lncRNA	exon	25461250	25461452	.	+	.	gene_id "lnc-bbug11951"	transcript_id "lnc-bbut00027076"	exon_number "4"
2	lncRNA	exon	25461808	25462694	.	+	.	gene_id "lnc-bbug11951"	transcript_id "lnc-bbut00027076"	exon_number "5"
2	lncRNA	exon	25705545	25705691	.	+	.	gene_id "lnc-bbug11960"	transcript_id "lnc-bbut00027100"	exon_number "1"
2	lncRNA	exon	25713752	25713937	.	+	.	gene_id "lnc-bbug11960"	transcript_id "lnc-bbut00027100"	exon_number "2"
2	lncRNA	exon	25714978	25715078	.	+	.	gene_id "lnc-bbug11960"	transcript_id "lnc-bbut00027100"	exon_number "3"
2	lncRNA	exon	25715782	25715899	.	+	.	gene_id "lnc-bbug11960"	transcript_id "lnc-bbut00027100"	exon_number "4"
2	lncRNA	exon	25717385	25720193	.	+	.	gene_id "lnc-bbug11960"	transcript_id "lnc-bbut00027100"	exon_number "5"
2	lncRNA	exon	25715240	25715375	.	+	.	gene_id "lnc-bbug11960"	transcript_id "lnc-bbut00027102"	exon_number "1"
2	lncRNA	exon	25715782	25715899	.	+	.	gene_id "lnc-bbug11960"	transcript_id "lnc-bbut00027102"	exon_number "2"
2	lncRNA	exon	25717385	25720193	.	+	.	gene_id "lnc-bbug11960"	transcript_id "lnc-bbut00027102"	exon_number "3"
2	lncRNA	exon	26437982	26438700	.	+	.	gene_id "lnc-bbug11965"	transcript_id "lnc-bbut00027118"	exon_number "1"
2	lncRNA	exon	26453701	26453718	.	+	.	gene_id "lnc-bbug11965"	transcript_id "lnc-bbut00027118"	exon_number "2"
2	lncRNA	exon	26459215	26460625	.	+	.	gene_id "lnc-bbug11965"	transcript_id "lnc-bbut00027118"	exon_number "3"
2	lncRNA	exon	26462912	26464574	.	+	.	gene_id "lnc-bbug11966"	transcript_id "lnc-bbut00027119"	exon_number "1"
2	lncRNA	exon	26466878	26466898	.	+	.	gene_id "lnc-bbug11966"	transcript_id "lnc-bbut00027119"	exon_number "2"
2	lncRNA	exon	26469656	26469749	.	+	.	gene_id "lnc-bbug11966"	transcript_id "lnc-bbut00027119"	exon_number "3"
2	lncRNA	exon	26494743	26495954	.	+	.	gene_id "lnc-bbug11967"	transcript_id "lnc-bbut00027120"	exon_number "1"
2	lncRNA	exon	26498255	26498275	.	+	.	gene_id "lnc-bbug11967"	transcript_id "lnc-bbut00027120"	exon_number "2"
2	lncRNA	exon	26500996	26501772	.	+	.	gene_id "lnc-bbug11967"	transcript_id "lnc-bbut00027120"	exon_number "3"
2	lncRNA	exon	26650603	26650739	.	+	.	gene_id "lnc-bbug11969"	transcript_id "lnc-bbut00027123"	exon_number "1"
2	lncRNA	exon	26655008	26655061	.	+	.	gene_id "lnc-bbug11969"	transcript_id "lnc-bbut00027123"	exon_number "2"
2	lncRNA	exon	26656554	26656662	.	+	.	gene_id "lnc-bbug11969"	transcript_id "lnc-bbut00027123"	exon_number "3"
2	lncRNA	exon	27282598	27283568	.	+	.	gene_id "lnc-bbug11977"	transcript_id "lnc-bbut00027134"	exon_number "1"
2	lncRNA	exon	27284002	27284160	.	+	.	gene_id "lnc-bbug11977"	transcript_id "lnc-bbut00027134"	exon_number "2"
2	lncRNA	exon	27285524	27285807	.	+	.	gene_id "lnc-bbug11977"	transcript_id "lnc-bbut00027134"	exon_number "3"

2	lncRNA	exon	27889650	27890347	.	+	.	gene_id "lnc-bbug11987"	transcript_id "lnc-bbut00027151"	exon_number "1"
2	lncRNA	exon	27906795	27906870	.	+	.	gene_id "lnc-bbug11987"	transcript_id "lnc-bbut00027151"	exon_number "2"
2	lncRNA	exon	27908322	27908799	.	+	.	gene_id "lnc-bbug11987"	transcript_id "lnc-bbut00027151"	exon_number "3"
2	lncRNA	exon	30037784	30038170	.	+	.	gene_id "lnc-bbug11991"	transcript_id "lnc-bbut00027156"	exon_number "1"
2	lncRNA	exon	30040014	30040154	.	+	.	gene_id "lnc-bbug11991"	transcript_id "lnc-bbut00027156"	exon_number "2"
2	lncRNA	exon	30058249	30058305	.	+	.	gene_id "lnc-bbug11991"	transcript_id "lnc-bbut00027156"	exon_number "3"
2	lncRNA	exon	30066031	30066332	.	+	.	gene_id "lnc-bbug11991"	transcript_id "lnc-bbut00027156"	exon_number "4"
2	lncRNA	exon	30037983	30038170	.	+	.	gene_id "lnc-bbug11991"	transcript_id "lnc-bbut00027157"	exon_number "1"
2	lncRNA	exon	30040014	30040154	.	+	.	gene_id "lnc-bbug11991"	transcript_id "lnc-bbut00027157"	exon_number "2"
2	lncRNA	exon	30058249	30058305	.	+	.	gene_id "lnc-bbug11991"	transcript_id "lnc-bbut00027157"	exon_number "3"
2	lncRNA	exon	30058392	30058569	.	+	.	gene_id "lnc-bbug11991"	transcript_id "lnc-bbut00027157"	exon_number "4"
2	lncRNA	exon	30037989	30038170	.	+	.	gene_id "lnc-bbug11991"	transcript_id "lnc-bbut00027158"	exon_number "1"
2	lncRNA	exon	30040014	30040154	.	+	.	gene_id "lnc-bbug11991"	transcript_id "lnc-bbut00027158"	exon_number "2"
2	lncRNA	exon	30066031	30066332	.	+	.	gene_id "lnc-bbug11991"	transcript_id "lnc-bbut00027158"	exon_number "3"
2	lncRNA	exon	30038007	30038170	.	+	.	gene_id "lnc-bbug11991"	transcript_id "lnc-bbut00027159"	exon_number "1"
2	lncRNA	exon	30040014	30040154	.	+	.	gene_id "lnc-bbug11991"	transcript_id "lnc-bbut00027159"	exon_number "2"
2	lncRNA	exon	30046193	30046871	.	+	.	gene_id "lnc-bbug11991"	transcript_id "lnc-bbut00027159"	exon_number "3"
2	lncRNA	exon	30053350	30053499	.	+	.	gene_id "lnc-bbug11991"	transcript_id "lnc-bbut00027160"	exon_number "1"
2	lncRNA	exon	30066031	30066332	.	+	.	gene_id "lnc-bbug11991"	transcript_id "lnc-bbut00027160"	exon_number "2"
2	lncRNA	exon	30053353	30053499	.	+	.	gene_id "lnc-bbug11991"	transcript_id "lnc-bbut00027161"	exon_number "1"
2	lncRNA	exon	30058249	30058305	.	+	.	gene_id "lnc-bbug11991"	transcript_id "lnc-bbut00027161"	exon_number "2"
2	lncRNA	exon	30066031	30066332	.	+	.	gene_id "lnc-bbug11991"	transcript_id "lnc-bbut00027161"	exon_number "3"
2	lncRNA	exon	30053355	30053499	.	+	.	gene_id "lnc-bbug11991"	transcript_id "lnc-bbut00027162"	exon_number "1"
2	lncRNA	exon	30058249	30058305	.	+	.	gene_id "lnc-bbug11991"	transcript_id "lnc-bbut00027162"	exon_number "2"
2	lncRNA	exon	30058392	30058569	.	+	.	gene_id "lnc-bbug11991"	transcript_id "lnc-bbut00027162"	exon_number "3"
2	lncRNA	exon	30094935	30095001	.	+	.	gene_id "lnc-bbug11993"	transcript_id "lnc-bbut00027164"	exon_number "1"
2	lncRNA	exon	30097307	30097443	.	+	.	gene_id "lnc-bbug11993"	transcript_id "lnc-bbut00027164"	exon_number "2"
2	lncRNA	exon	31201434	31201780	.	+	.	gene_id "lnc-bbug12001"	transcript_id "lnc-bbut00027175"	exon_number "1"
2	lncRNA	exon	31205609	31205710	.	+	.	gene_id "lnc-bbug12001"	transcript_id "lnc-bbut00027175"	exon_number "2"
2	lncRNA	exon	31215485	31215641	.	+	.	gene_id "lnc-bbug12001"	transcript_id "lnc-bbut00027175"	exon_number "3"
2	lncRNA	exon	31216553	31216859	.	+	.	gene_id "lnc-bbug12001"	transcript_id "lnc-bbut00027175"	exon_number "4"
2	lncRNA	exon	31279876	31281310	.	+	.	gene_id "lnc-bbug12002"	transcript_id "lnc-bbut00027176"	exon_number "1"
2	lncRNA	exon	32378822	32379552	.	+	.	gene_id "lnc-bbug12007"	transcript_id "lnc-bbut00027182"	exon_number "1"
2	lncRNA	exon	32380385	32382448	.	+	.	gene_id "lnc-bbug12007"	transcript_id "lnc-bbut00027182"	exon_number "2"
2	lncRNA	exon	32667807	32667865	.	+	.	gene_id "lnc-bbug12010"	transcript_id "lnc-bbut00027195"	exon_number "1"
2	lncRNA	exon	32669157	32669305	.	+	.	gene_id "lnc-bbug12010"	transcript_id "lnc-bbut00027195"	exon_number "2"
2	lncRNA	exon	32684348	32684524	.	+	.	gene_id "lnc-bbug12010"	transcript_id "lnc-bbut00027195"	exon_number "3"
2	lncRNA	exon	32690394	32690573	.	+	.	gene_id "lnc-bbug12010"	transcript_id "lnc-bbut00027195"	exon_number "4"
2	lncRNA	exon	33053755	33053862	.	+	.	gene_id "lnc-bbug12013"	transcript_id "lnc-bbut00027202"	exon_number "1"
2	lncRNA	exon	33077516	33077712	.	+	.	gene_id "lnc-bbug12013"	transcript_id "lnc-bbut00027202"	exon_number "2"
2	lncRNA	exon	33080873	33080984	.	+	.	gene_id "lnc-bbug12013"	transcript_id "lnc-bbut00027202"	exon_number "3"
2	lncRNA	exon	33084563	33084725	.	+	.	gene_id "lnc-bbug12013"	transcript_id "lnc-bbut00027202"	exon_number "4"
2	lncRNA	exon	33053755	33053862	.	+	.	gene_id "lnc-bbug12013"	transcript_id "lnc-bbut00027201"	exon_number "1"
2	lncRNA	exon	33077516	33077712	.	+	.	gene_id "lnc-bbug12013"	transcript_id "lnc-bbut00027201"	exon_number "2"
2	lncRNA	exon	33080873	33084725	.	+	.	gene_id "lnc-bbug12013"	transcript_id "lnc-bbut00027201"	exon_number "3"
2	lncRNA	exon	34285311	34285841	.	+	.	gene_id "lnc-bbug12016"	transcript_id "lnc-bbut00027209"	exon_number "1"
2	lncRNA	exon	34297395	34297741	.	+	.	gene_id "lnc-bbug12016"	transcript_id "lnc-bbut00027209"	exon_number "2"
2	lncRNA	exon	34412746	34413162	.	+	.	gene_id "lnc-bbug12017"	transcript_id "lnc-bbut00027210"	exon_number "1"
2	lncRNA	exon	34413442	34413734	.	+	.	gene_id "lnc-bbug12017"	transcript_id "lnc-bbut00027210"	exon_number "2"
2	lncRNA	exon	34742541	34743165	.	+	.	gene_id "lnc-bbug12022"	transcript_id "lnc-bbut00027219"	exon_number "1"
2	lncRNA	exon	35600868	35601071	.	+	.	gene_id "lnc-bbug12033"	transcript_id "lnc-bbut00027243"	exon_number "1"
2	lncRNA	exon	35602629	35602697	.	+	.	gene_id "lnc-bbug12033"	transcript_id "lnc-bbut00027243"	exon_number "2"

2	lncRNA	exon	35607006	35608143	.	+	.	gene_id "lnc-bbug12033"	transcript_id "lnc-bbut00027243"	exon_number "3"
2	lncRNA	exon	35878599	35879718	.	+	.	gene_id "lnc-bbug12040"	transcript_id "lnc-bbut00027252"	exon_number "1"
2	lncRNA	exon	35879965	35880074	.	+	.	gene_id "lnc-bbug12040"	transcript_id "lnc-bbut00027252"	exon_number "2"
2	lncRNA	exon	35880361	35880463	.	+	.	gene_id "lnc-bbug12040"	transcript_id "lnc-bbut00027252"	exon_number "3"
2	lncRNA	exon	35881909	35882198	.	+	.	gene_id "lnc-bbug12040"	transcript_id "lnc-bbut00027252"	exon_number "4"
2	lncRNA	exon	35878599	35880074	.	+	.	gene_id "lnc-bbug12040"	transcript_id "lnc-bbut00027251"	exon_number "1"
2	lncRNA	exon	35880361	35880463	.	+	.	gene_id "lnc-bbug12040"	transcript_id "lnc-bbut00027251"	exon_number "2"
2	lncRNA	exon	35881909	35882198	.	+	.	gene_id "lnc-bbug12040"	transcript_id "lnc-bbut00027251"	exon_number "3"
2	lncRNA	exon	36149394	36149470	.	+	.	gene_id "lnc-bbug12043"	transcript_id "lnc-bbut00027255"	exon_number "1"
2	lncRNA	exon	36187631	36187746	.	+	.	gene_id "lnc-bbug12043"	transcript_id "lnc-bbut00027255"	exon_number "2"
2	lncRNA	exon	36187842	36187976	.	+	.	gene_id "lnc-bbug12043"	transcript_id "lnc-bbut00027255"	exon_number "3"
2	lncRNA	exon	36192584	36193688	.	+	.	gene_id "lnc-bbug12043"	transcript_id "lnc-bbut00027255"	exon_number "4"
2	lncRNA	exon	36243644	36245144	.	+	.	gene_id "lnc-bbug12044"	transcript_id "lnc-bbut00027256"	exon_number "1"
2	lncRNA	exon	36528116	36528217	.	+	.	gene_id "lnc-bbug12048"	transcript_id "lnc-bbut00027261"	exon_number "1"
2	lncRNA	exon	36529885	36529993	.	+	.	gene_id "lnc-bbug12048"	transcript_id "lnc-bbut00027261"	exon_number "2"
2	lncRNA	exon	36537663	36539274	.	+	.	gene_id "lnc-bbug12048"	transcript_id "lnc-bbut00027261"	exon_number "3"
2	lncRNA	exon	36528890	36529678	.	+	.	gene_id "lnc-bbug12048"	transcript_id "lnc-bbut00027262"	exon_number "1"
2	lncRNA	exon	36529885	36529993	.	+	.	gene_id "lnc-bbug12048"	transcript_id "lnc-bbut00027262"	exon_number "2"
2	lncRNA	exon	36537663	36539274	.	+	.	gene_id "lnc-bbug12048"	transcript_id "lnc-bbut00027262"	exon_number "3"
2	lncRNA	exon	37128616	37128889	.	+	.	gene_id "lnc-bbug12056"	transcript_id "lnc-bbut00027285"	exon_number "1"
2	lncRNA	exon	37132930	37133176	.	+	.	gene_id "lnc-bbug12056"	transcript_id "lnc-bbut00027285"	exon_number "2"
2	lncRNA	exon	37134258	37135646	.	+	.	gene_id "lnc-bbug12056"	transcript_id "lnc-bbut00027285"	exon_number "3"
2	lncRNA	exon	37128617	37128889	.	+	.	gene_id "lnc-bbug12056"	transcript_id "lnc-bbut00027286"	exon_number "1"
2	lncRNA	exon	37133035	37133176	.	+	.	gene_id "lnc-bbug12056"	transcript_id "lnc-bbut00027286"	exon_number "2"
2	lncRNA	exon	37134258	37135646	.	+	.	gene_id "lnc-bbug12056"	transcript_id "lnc-bbut00027286"	exon_number "3"
2	lncRNA	exon	37151285	37152702	.	+	.	gene_id "lnc-bbug12057"	transcript_id "lnc-bbut00027288"	exon_number "1"
2	lncRNA	exon	37153492	37153589	.	+	.	gene_id "lnc-bbug12057"	transcript_id "lnc-bbut00027288"	exon_number "2"
2	lncRNA	exon	37154927	37156033	.	+	.	gene_id "lnc-bbug12057"	transcript_id "lnc-bbut00027288"	exon_number "3"
2	lncRNA	exon	37151285	37152702	.	+	.	gene_id "lnc-bbug12057"	transcript_id "lnc-bbut00027287"	exon_number "1"
2	lncRNA	exon	37153468	37153589	.	+	.	gene_id "lnc-bbug12057"	transcript_id "lnc-bbut00027287"	exon_number "2"
2	lncRNA	exon	37154927	37156033	.	+	.	gene_id "lnc-bbug12057"	transcript_id "lnc-bbut00027287"	exon_number "3"
2	lncRNA	exon	37416754	37416888	.	+	.	gene_id "lnc-bbug12063"	transcript_id "lnc-bbut00027303"	exon_number "1"
2	lncRNA	exon	37417497	37417576	.	+	.	gene_id "lnc-bbug12063"	transcript_id "lnc-bbut00027303"	exon_number "2"
2	lncRNA	exon	37417938	37418082	.	+	.	gene_id "lnc-bbug12063"	transcript_id "lnc-bbut00027303"	exon_number "3"
2	lncRNA	exon	37419122	37419180	.	+	.	gene_id "lnc-bbug12063"	transcript_id "lnc-bbut00027303"	exon_number "4"
2	lncRNA	exon	37420176	37420288	.	+	.	gene_id "lnc-bbug12063"	transcript_id "lnc-bbut00027303"	exon_number "5"
2	lncRNA	exon	37420827	37421790	.	+	.	gene_id "lnc-bbug12063"	transcript_id "lnc-bbut00027303"	exon_number "6"
2	lncRNA	exon	37416754	37416888	.	+	.	gene_id "lnc-bbug12063"	transcript_id "lnc-bbut00027304"	exon_number "1"
2	lncRNA	exon	37417497	37417576	.	+	.	gene_id "lnc-bbug12063"	transcript_id "lnc-bbut00027304"	exon_number "2"
2	lncRNA	exon	37417938	37418082	.	+	.	gene_id "lnc-bbug12063"	transcript_id "lnc-bbut00027304"	exon_number "3"
2	lncRNA	exon	37419122	37419180	.	+	.	gene_id "lnc-bbug12063"	transcript_id "lnc-bbut00027304"	exon_number "4"
2	lncRNA	exon	37421797	37422430	.	+	.	gene_id "lnc-bbug12063"	transcript_id "lnc-bbut00027304"	exon_number "5"
2	lncRNA	exon	38178887	38179048	.	+	.	gene_id "lnc-bbug12067"	transcript_id "lnc-bbut00027316"	exon_number "1"
2	lncRNA	exon	38198931	38199029	.	+	.	gene_id "lnc-bbug12067"	transcript_id "lnc-bbut00027316"	exon_number "2"
2	lncRNA	exon	38202492	38204107	.	+	.	gene_id "lnc-bbug12067"	transcript_id "lnc-bbut00027316"	exon_number "3"
2	lncRNA	exon	38204168	38204205	.	+	.	gene_id "lnc-bbug12068"	transcript_id "lnc-bbut00027317"	exon_number "1"
2	lncRNA	exon	38204747	38204856	.	+	.	gene_id "lnc-bbug12068"	transcript_id "lnc-bbut00027317"	exon_number "2"
2	lncRNA	exon	38205210	38205495	.	+	.	gene_id "lnc-bbug12068"	transcript_id "lnc-bbut00027317"	exon_number "3"
2	lncRNA	exon	38239465	38240542	.	+	.	gene_id "lnc-bbug12070"	transcript_id "lnc-bbut00027319"	exon_number "1"
2	lncRNA	exon	38241522	38241770	.	+	.	gene_id "lnc-bbug12070"	transcript_id "lnc-bbut00027319"	exon_number "2"
2	lncRNA	exon	38331208	38334438	.	+	.	gene_id "lnc-bbug12070"	transcript_id "lnc-bbut00027319"	exon_number "3"
2	lncRNA	exon	40712198	40715016	.	+	.	gene_id "lnc-bbug12082"	transcript_id "lnc-bbut00027346"	exon_number "1"

2	lncRNA	exon	40715097	40715988	.	+	.	gene_id "lnc-bbug12082"	transcript_id "lnc-bbut00027346"	exon_number "2"
2	lncRNA	exon	40712198	40713495	.	+	.	gene_id "lnc-bbug12082"	transcript_id "lnc-bbut00027345"	exon_number "1"
2	lncRNA	exon	40715097	40715988	.	+	.	gene_id "lnc-bbug12082"	transcript_id "lnc-bbut00027345"	exon_number "2"
2	lncRNA	exon	41047285	41047547	.	+	.	gene_id "lnc-bbug12084"	transcript_id "lnc-bbut00027348"	exon_number "1"
2	lncRNA	exon	41060865	41060959	.	+	.	gene_id "lnc-bbug12084"	transcript_id "lnc-bbut00027348"	exon_number "2"
2	lncRNA	exon	41061151	41061203	.	+	.	gene_id "lnc-bbug12084"	transcript_id "lnc-bbut00027348"	exon_number "3"
2	lncRNA	exon	41062571	41062923	.	+	.	gene_id "lnc-bbug12084"	transcript_id "lnc-bbut00027348"	exon_number "4"
2	lncRNA	exon	41355304	41356742	.	+	.	gene_id "lnc-bbug12087"	transcript_id "lnc-bbut00027356"	exon_number "1"
2	lncRNA	exon	41364273	41364743	.	+	.	gene_id "lnc-bbug12087"	transcript_id "lnc-bbut00027356"	exon_number "2"
2	lncRNA	exon	41465900	41465988	.	+	.	gene_id "lnc-bbug12088"	transcript_id "lnc-bbut00027357"	exon_number "1"
2	lncRNA	exon	41468695	41468779	.	+	.	gene_id "lnc-bbug12088"	transcript_id "lnc-bbut00027357"	exon_number "2"
2	lncRNA	exon	41469801	41477079	.	+	.	gene_id "lnc-bbug12088"	transcript_id "lnc-bbut00027357"	exon_number "3"
2	lncRNA	exon	41468556	41469020	.	+	.	gene_id "lnc-bbug12088"	transcript_id "lnc-bbut00027358"	exon_number "1"
2	lncRNA	exon	41469801	41477079	.	+	.	gene_id "lnc-bbug12088"	transcript_id "lnc-bbut00027358"	exon_number "2"
2	lncRNA	exon	41991676	41991805	.	+	.	gene_id "lnc-bbug12096"	transcript_id "lnc-bbut00027377"	exon_number "1"
2	lncRNA	exon	41991902	41993637	.	+	.	gene_id "lnc-bbug12096"	transcript_id "lnc-bbut00027377"	exon_number "2"
2	lncRNA	exon	42063523	42064177	.	+	.	gene_id "lnc-bbug12097"	transcript_id "lnc-bbut00027378"	exon_number "1"
2	lncRNA	exon	42075021	42075151	.	+	.	gene_id "lnc-bbug12097"	transcript_id "lnc-bbut00027378"	exon_number "2"
2	lncRNA	exon	42080063	42081157	.	+	.	gene_id "lnc-bbug12097"	transcript_id "lnc-bbut00027378"	exon_number "3"
2	lncRNA	exon	42301124	42301163	.	+	.	gene_id "lnc-bbug12099"	transcript_id "lnc-bbut00027381"	exon_number "1"
2	lncRNA	exon	42324849	42324898	.	+	.	gene_id "lnc-bbug12099"	transcript_id "lnc-bbut00027381"	exon_number "2"
2	lncRNA	exon	42348623	42349326	.	+	.	gene_id "lnc-bbug12099"	transcript_id "lnc-bbut00027381"	exon_number "3"
2	lncRNA	exon	42710435	42710577	.	+	.	gene_id "lnc-bbug12103"	transcript_id "lnc-bbut00027392"	exon_number "1"
2	lncRNA	exon	42711268	42711636	.	+	.	gene_id "lnc-bbug12103"	transcript_id "lnc-bbut00027392"	exon_number "2"
2	lncRNA	exon	43178408	43178558	.	+	.	gene_id "lnc-bbug12107"	transcript_id "lnc-bbut00027403"	exon_number "1"
2	lncRNA	exon	43205057	43205219	.	+	.	gene_id "lnc-bbug12107"	transcript_id "lnc-bbut00027403"	exon_number "2"
2	lncRNA	exon	43213986	43214407	.	+	.	gene_id "lnc-bbug12107"	transcript_id "lnc-bbut00027403"	exon_number "3"
2	lncRNA	exon	43605600	43605952	.	+	.	gene_id "lnc-bbug12110"	transcript_id "lnc-bbut00027407"	exon_number "1"
2	lncRNA	exon	43609094	43609244	.	+	.	gene_id "lnc-bbug12110"	transcript_id "lnc-bbut00027407"	exon_number "2"
2	lncRNA	exon	43613771	43613820	.	+	.	gene_id "lnc-bbug12110"	transcript_id "lnc-bbut00027407"	exon_number "3"
2	lncRNA	exon	43605600	43605952	.	+	.	gene_id "lnc-bbug12110"	transcript_id "lnc-bbut00027408"	exon_number "1"
2	lncRNA	exon	43609094	43609244	.	+	.	gene_id "lnc-bbug12110"	transcript_id "lnc-bbut00027408"	exon_number "2"
2	lncRNA	exon	43614305	43614587	.	+	.	gene_id "lnc-bbug12110"	transcript_id "lnc-bbut00027408"	exon_number "3"
2	lncRNA	exon	43931003	43931453	.	+	.	gene_id "lnc-bbug12114"	transcript_id "lnc-bbut00027416"	exon_number "1"
2	lncRNA	exon	44088877	44088995	.	+	.	gene_id "lnc-bbug12116"	transcript_id "lnc-bbut00027419"	exon_number "1"
2	lncRNA	exon	44090292	44090475	.	+	.	gene_id "lnc-bbug12116"	transcript_id "lnc-bbut00027419"	exon_number "2"
2	lncRNA	exon	44090658	44090683	.	+	.	gene_id "lnc-bbug12116"	transcript_id "lnc-bbut00027419"	exon_number "3"
2	lncRNA	exon	44091009	44091075	.	+	.	gene_id "lnc-bbug12116"	transcript_id "lnc-bbut00027419"	exon_number "4"
2	lncRNA	exon	44091236	44091668	.	+	.	gene_id "lnc-bbug12116"	transcript_id "lnc-bbut00027419"	exon_number "5"
2	lncRNA	exon	44088880	44088995	.	+	.	gene_id "lnc-bbug12116"	transcript_id "lnc-bbut00027420"	exon_number "1"
2	lncRNA	exon	44090292	44090415	.	+	.	gene_id "lnc-bbug12116"	transcript_id "lnc-bbut00027420"	exon_number "2"
2	lncRNA	exon	44090658	44090683	.	+	.	gene_id "lnc-bbug12116"	transcript_id "lnc-bbut00027420"	exon_number "3"
2	lncRNA	exon	44090970	44091075	.	+	.	gene_id "lnc-bbug12116"	transcript_id "lnc-bbut00027420"	exon_number "4"
2	lncRNA	exon	44091236	44091668	.	+	.	gene_id "lnc-bbug12116"	transcript_id "lnc-bbut00027420"	exon_number "5"
2	lncRNA	exon	44489531	44489648	.	+	.	gene_id "lnc-bbug12118"	transcript_id "lnc-bbut00027432"	exon_number "1"
2	lncRNA	exon	44491938	44492051	.	+	.	gene_id "lnc-bbug12118"	transcript_id "lnc-bbut00027432"	exon_number "2"
2	lncRNA	exon	44493446	44493554	.	+	.	gene_id "lnc-bbug12118"	transcript_id "lnc-bbut00027432"	exon_number "3"
2	lncRNA	exon	44494351	44494464	.	+	.	gene_id "lnc-bbug12118"	transcript_id "lnc-bbut00027432"	exon_number "4"
2	lncRNA	exon	45167533	45168302	.	+	.	gene_id "lnc-bbug12135"	transcript_id "lnc-bbut00027471"	exon_number "1"
2	lncRNA	exon	45175270	45175392	.	+	.	gene_id "lnc-bbug12135"	transcript_id "lnc-bbut00027471"	exon_number "2"
2	lncRNA	exon	45175479	45175638	.	+	.	gene_id "lnc-bbug12135"	transcript_id "lnc-bbut00027471"	exon_number "3"
2	lncRNA	exon	45170366	45170879	.	+	.	gene_id "lnc-bbug12135"	transcript_id "lnc-bbut00027472"	exon_number "1"

2	lncRNA	exon	45175270	45175392	.	+	.	gene_id "lnc-bbug12135"	transcript_id "lnc-bbut00027472"	exon_number "2"
2	lncRNA	exon	45175479	45175638	.	+	.	gene_id "lnc-bbug12135"	transcript_id "lnc-bbut00027472"	exon_number "3"
2	lncRNA	exon	45171579	45173888	.	+	.	gene_id "lnc-bbug12135"	transcript_id "lnc-bbut00027473"	exon_number "1"
2	lncRNA	exon	45175270	45175392	.	+	.	gene_id "lnc-bbug12135"	transcript_id "lnc-bbut00027473"	exon_number "2"
2	lncRNA	exon	45175479	45175638	.	+	.	gene_id "lnc-bbug12135"	transcript_id "lnc-bbut00027473"	exon_number "3"
2	lncRNA	exon	45195283	45195590	.	+	.	gene_id "lnc-bbug12136"	transcript_id "lnc-bbut00027474"	exon_number "1"
2	lncRNA	exon	45217405	45218656	.	+	.	gene_id "lnc-bbug12136"	transcript_id "lnc-bbut00027474"	exon_number "2"
2	lncRNA	exon	45590118	45591212	.	+	.	gene_id "lnc-bbug12142"	transcript_id "lnc-bbut00027485"	exon_number "1"
2	lncRNA	exon	45592838	45592946	.	+	.	gene_id "lnc-bbug12142"	transcript_id "lnc-bbut00027485"	exon_number "2"
2	lncRNA	exon	45594393	45594452	.	+	.	gene_id "lnc-bbug12142"	transcript_id "lnc-bbut00027485"	exon_number "3"
2	lncRNA	exon	45840560	45841231	.	+	.	gene_id "lnc-bbug12145"	transcript_id "lnc-bbut00027488"	exon_number "1"
2	lncRNA	exon	45844960	45845764	.	+	.	gene_id "lnc-bbug12145"	transcript_id "lnc-bbut00027488"	exon_number "2"
2	lncRNA	exon	46220642	46221097	.	+	.	gene_id "lnc-bbug12147"	transcript_id "lnc-bbut00027508"	exon_number "1"
2	lncRNA	exon	46222250	46222725	.	+	.	gene_id "lnc-bbug12147"	transcript_id "lnc-bbut00027508"	exon_number "2"
2	lncRNA	exon	46223587	46224145	.	+	.	gene_id "lnc-bbug12147"	transcript_id "lnc-bbut00027508"	exon_number "3"
2	lncRNA	exon	47561678	47563934	.	+	.	gene_id "lnc-bbug12152"	transcript_id "lnc-bbut00027516"	exon_number "1"
2	lncRNA	exon	47581682	47581872	.	+	.	gene_id "lnc-bbug12152"	transcript_id "lnc-bbut00027516"	exon_number "2"
2	lncRNA	exon	47587955	47588135	.	+	.	gene_id "lnc-bbug12152"	transcript_id "lnc-bbut00027516"	exon_number "3"
2	lncRNA	exon	47799532	47799615	.	+	.	gene_id "lnc-bbug12153"	transcript_id "lnc-bbut00027517"	exon_number "1"
2	lncRNA	exon	47801573	47801652	.	+	.	gene_id "lnc-bbug12153"	transcript_id "lnc-bbut00027517"	exon_number "2"
2	lncRNA	exon	47804670	47805436	.	+	.	gene_id "lnc-bbug12153"	transcript_id "lnc-bbut00027517"	exon_number "3"
2	lncRNA	exon	49129897	49130852	.	+	.	gene_id "lnc-bbug12156"	transcript_id "lnc-bbut00027520"	exon_number "1"
2	lncRNA	exon	49188583	49188748	.	+	.	gene_id "lnc-bbug12157"	transcript_id "lnc-bbut00027522"	exon_number "1"
2	lncRNA	exon	49189059	49189143	.	+	.	gene_id "lnc-bbug12157"	transcript_id "lnc-bbut00027522"	exon_number "2"
2	lncRNA	exon	49190962	49191177	.	+	.	gene_id "lnc-bbug12157"	transcript_id "lnc-bbut00027522"	exon_number "3"
2	lncRNA	exon	49191712	49192571	.	+	.	gene_id "lnc-bbug12157"	transcript_id "lnc-bbut00027522"	exon_number "4"
2	lncRNA	exon	49188583	49188748	.	+	.	gene_id "lnc-bbug12157"	transcript_id "lnc-bbut00027523"	exon_number "1"
2	lncRNA	exon	49189082	49189143	.	+	.	gene_id "lnc-bbug12157"	transcript_id "lnc-bbut00027523"	exon_number "2"
2	lncRNA	exon	49190962	49191177	.	+	.	gene_id "lnc-bbug12157"	transcript_id "lnc-bbut00027523"	exon_number "3"
2	lncRNA	exon	49193947	49194194	.	+	.	gene_id "lnc-bbug12157"	transcript_id "lnc-bbut00027523"	exon_number "4"
2	lncRNA	exon	49740957	49745443	.	+	.	gene_id "lnc-bbug12162"	transcript_id "lnc-bbut00027532"	exon_number "1"
2	lncRNA	exon	49827445	49829075	.	+	.	gene_id "lnc-bbug12163"	transcript_id "lnc-bbut00027533"	exon_number "1"
2	lncRNA	exon	50873378	50873491	.	+	.	gene_id "lnc-bbug12165"	transcript_id "lnc-bbut00027535"	exon_number "1"
2	lncRNA	exon	50874631	50874761	.	+	.	gene_id "lnc-bbug12165"	transcript_id "lnc-bbut00027535"	exon_number "2"
2	lncRNA	exon	50874822	50875117	.	+	.	gene_id "lnc-bbug12165"	transcript_id "lnc-bbut00027535"	exon_number "3"
2	lncRNA	exon	50876283	50877028	.	+	.	gene_id "lnc-bbug12165"	transcript_id "lnc-bbut00027535"	exon_number "4"
2	lncRNA	exon	50877196	50877587	.	+	.	gene_id "lnc-bbug12165"	transcript_id "lnc-bbut00027535"	exon_number "5"
2	lncRNA	exon	52820460	52821486	.	+	.	gene_id "lnc-bbug12171"	transcript_id "lnc-bbut00027549"	exon_number "1"
2	lncRNA	exon	52881680	52882653	.	+	.	gene_id "lnc-bbug12172"	transcript_id "lnc-bbut00027550"	exon_number "1"
2	lncRNA	exon	53986921	53987155	.	+	.	gene_id "lnc-bbug12178"	transcript_id "lnc-bbut00027565"	exon_number "1"
2	lncRNA	exon	54576102	54576184	.	+	.	gene_id "lnc-bbug12182"	transcript_id "lnc-bbut00027575"	exon_number "1"
2	lncRNA	exon	54583886	54583971	.	+	.	gene_id "lnc-bbug12182"	transcript_id "lnc-bbut00027575"	exon_number "2"
2	lncRNA	exon	54584075	54584121	.	+	.	gene_id "lnc-bbug12182"	transcript_id "lnc-bbut00027575"	exon_number "3"
2	lncRNA	exon	55432223	55432344	.	+	.	gene_id "lnc-bbug12183"	transcript_id "lnc-bbut00027576"	exon_number "1"
2	lncRNA	exon	55433162	55433260	.	+	.	gene_id "lnc-bbug12183"	transcript_id "lnc-bbut00027576"	exon_number "2"
2	lncRNA	exon	55439501	55439637	.	+	.	gene_id "lnc-bbug12183"	transcript_id "lnc-bbut00027576"	exon_number "3"
2	lncRNA	exon	55923724	55923768	.	+	.	gene_id "lnc-bbug12185"	transcript_id "lnc-bbut00027578"	exon_number "1"
2	lncRNA	exon	55926918	55927012	.	+	.	gene_id "lnc-bbug12185"	transcript_id "lnc-bbut00027578"	exon_number "2"
2	lncRNA	exon	55928729	55929705	.	+	.	gene_id "lnc-bbug12185"	transcript_id "lnc-bbut00027578"	exon_number "3"
2	lncRNA	exon	56392051	56392941	.	+	.	gene_id "lnc-bbug12186"	transcript_id "lnc-bbut00027579"	exon_number "1"
2	lncRNA	exon	56393266	56393400	.	+	.	gene_id "lnc-bbug12186"	transcript_id "lnc-bbut00027579"	exon_number "2"
2	lncRNA	exon	56399821	56401111	.	+	.	gene_id "lnc-bbug12186"	transcript_id "lnc-bbut00027579"	exon_number "3"

2	lncRNA	exon	57115419	57115567	.	+	.	gene_id "lnc-bbug12191"	transcript_id "lnc-bbut00027594"	exon_number "1"
2	lncRNA	exon	57117319	57117500	.	+	.	gene_id "lnc-bbug12191"	transcript_id "lnc-bbut00027594"	exon_number "2"
2	lncRNA	exon	57123451	57123546	.	+	.	gene_id "lnc-bbug12191"	transcript_id "lnc-bbut00027594"	exon_number "3"
2	lncRNA	exon	57126488	57127763	.	+	.	gene_id "lnc-bbug12191"	transcript_id "lnc-bbut00027594"	exon_number "4"
2	lncRNA	exon	57608246	57608776	.	+	.	gene_id "lnc-bbug12195"	transcript_id "lnc-bbut00027618"	exon_number "1"
2	lncRNA	exon	57608989	57609368	.	+	.	gene_id "lnc-bbug12195"	transcript_id "lnc-bbut00027618"	exon_number "2"
2	lncRNA	exon	58202018	58202958	.	+	.	gene_id "lnc-bbug12202"	transcript_id "lnc-bbut00027649"	exon_number "1"
2	lncRNA	exon	58220531	58220867	.	+	.	gene_id "lnc-bbug12202"	transcript_id "lnc-bbut00027649"	exon_number "2"
2	lncRNA	exon	58577305	58577396	.	+	.	gene_id "lnc-bbug12205"	transcript_id "lnc-bbut00027661"	exon_number "1"
2	lncRNA	exon	58582397	58582728	.	+	.	gene_id "lnc-bbug12205"	transcript_id "lnc-bbut00027661"	exon_number "2"
2	lncRNA	exon	59369956	59370512	.	+	.	gene_id "lnc-bbug12210"	transcript_id "lnc-bbut00027668"	exon_number "1"
2	lncRNA	exon	59373519	59373631	.	+	.	gene_id "lnc-bbug12210"	transcript_id "lnc-bbut00027668"	exon_number "2"
2	lncRNA	exon	59374750	59374790	.	+	.	gene_id "lnc-bbug12210"	transcript_id "lnc-bbut00027668"	exon_number "3"
2	lncRNA	exon	59375015	59375319	.	+	.	gene_id "lnc-bbug12210"	transcript_id "lnc-bbut00027668"	exon_number "4"
2	lncRNA	exon	59375500	59375704	.	+	.	gene_id "lnc-bbug12210"	transcript_id "lnc-bbut00027668"	exon_number "5"
2	lncRNA	exon	59386176	59386384	.	+	.	gene_id "lnc-bbug12211"	transcript_id "lnc-bbut00027669"	exon_number "1"
2	lncRNA	exon	59390154	59390279	.	+	.	gene_id "lnc-bbug12211"	transcript_id "lnc-bbut00027669"	exon_number "2"
2	lncRNA	exon	59390559	59391520	.	+	.	gene_id "lnc-bbug12211"	transcript_id "lnc-bbut00027669"	exon_number "3"
2	lncRNA	exon	59391857	59392920	.	+	.	gene_id "lnc-bbug12211"	transcript_id "lnc-bbut00027669"	exon_number "4"
2	lncRNA	exon	59454470	59454795	.	+	.	gene_id "lnc-bbug12212"	transcript_id "lnc-bbut00027670"	exon_number "1"
2	lncRNA	exon	59490323	59490441	.	+	.	gene_id "lnc-bbug12212"	transcript_id "lnc-bbut00027670"	exon_number "2"
2	lncRNA	exon	59495227	59495281	.	+	.	gene_id "lnc-bbug12212"	transcript_id "lnc-bbut00027670"	exon_number "3"
2	lncRNA	exon	59534926	59536334	.	+	.	gene_id "lnc-bbug12213"	transcript_id "lnc-bbut00027671"	exon_number "1"
2	lncRNA	exon	59543398	59543517	.	+	.	gene_id "lnc-bbug12213"	transcript_id "lnc-bbut00027671"	exon_number "2"
2	lncRNA	exon	59544135	59544671	.	+	.	gene_id "lnc-bbug12213"	transcript_id "lnc-bbut00027671"	exon_number "3"
2	lncRNA	exon	59534926	59536334	.	+	.	gene_id "lnc-bbug12213"	transcript_id "lnc-bbut00027672"	exon_number "1"
2	lncRNA	exon	59543398	59543517	.	+	.	gene_id "lnc-bbug12213"	transcript_id "lnc-bbut00027672"	exon_number "2"
2	lncRNA	exon	59544135	59544188	.	+	.	gene_id "lnc-bbug12213"	transcript_id "lnc-bbut00027672"	exon_number "3"
2	lncRNA	exon	59545051	59545110	.	+	.	gene_id "lnc-bbug12213"	transcript_id "lnc-bbut00027672"	exon_number "4"
2	lncRNA	exon	60271966	60272133	.	+	.	gene_id "lnc-bbug12215"	transcript_id "lnc-bbut00027674"	exon_number "1"
2	lncRNA	exon	60278395	60278492	.	+	.	gene_id "lnc-bbug12215"	transcript_id "lnc-bbut00027674"	exon_number "2"
2	lncRNA	exon	60282399	60282447	.	+	.	gene_id "lnc-bbug12215"	transcript_id "lnc-bbut00027674"	exon_number "3"
2	lncRNA	exon	62277719	62278048	.	+	.	gene_id "lnc-bbug12221"	transcript_id "lnc-bbut00027681"	exon_number "1"
2	lncRNA	exon	66690657	66690870	.	+	.	gene_id "lnc-bbug12230"	transcript_id "lnc-bbut00027695"	exon_number "1"
2	lncRNA	exon	66691011	66692053	.	+	.	gene_id "lnc-bbug12230"	transcript_id "lnc-bbut00027695"	exon_number "2"
2	lncRNA	exon	67209079	67212240	.	+	.	gene_id "lnc-bbug12231"	transcript_id "lnc-bbut00027698"	exon_number "1"
2	lncRNA	exon	67217559	67217650	.	+	.	gene_id "lnc-bbug12231"	transcript_id "lnc-bbut00027698"	exon_number "2"
2	lncRNA	exon	67222816	67223803	.	+	.	gene_id "lnc-bbug12231"	transcript_id "lnc-bbut00027698"	exon_number "3"
2	lncRNA	exon	67224555	67225330	.	+	.	gene_id "lnc-bbug12232"	transcript_id "lnc-bbut00027699"	exon_number "1"
2	lncRNA	exon	67229289	67229445	.	+	.	gene_id "lnc-bbug12232"	transcript_id "lnc-bbut00027699"	exon_number "2"
2	lncRNA	exon	67232096	67232147	.	+	.	gene_id "lnc-bbug12232"	transcript_id "lnc-bbut00027699"	exon_number "3"
2	lncRNA	exon	67823854	67823960	.	+	.	gene_id "lnc-bbug12235"	transcript_id "lnc-bbut00027704"	exon_number "1"
2	lncRNA	exon	67824473	67825317	.	+	.	gene_id "lnc-bbug12235"	transcript_id "lnc-bbut00027704"	exon_number "2"
2	lncRNA	exon	67858138	67858516	.	+	.	gene_id "lnc-bbug12236"	transcript_id "lnc-bbut00027705"	exon_number "1"
2	lncRNA	exon	67878090	67879387	.	+	.	gene_id "lnc-bbug12236"	transcript_id "lnc-bbut00027705"	exon_number "2"
2	lncRNA	exon	67872250	67872312	.	+	.	gene_id "lnc-bbug12236"	transcript_id "lnc-bbut00027706"	exon_number "1"
2	lncRNA	exon	67878090	67879387	.	+	.	gene_id "lnc-bbug12236"	transcript_id "lnc-bbut00027706"	exon_number "2"
2	lncRNA	exon	67985182	67985462	.	+	.	gene_id "lnc-bbug12237"	transcript_id "lnc-bbut00027707"	exon_number "1"
2	lncRNA	exon	67985874	67986065	.	+	.	gene_id "lnc-bbug12237"	transcript_id "lnc-bbut00027707"	exon_number "2"
2	lncRNA	exon	67991287	67991315	.	+	.	gene_id "lnc-bbug12238"	transcript_id "lnc-bbut00027708"	exon_number "1"
2	lncRNA	exon	67996906	68001455	.	+	.	gene_id "lnc-bbug12238"	transcript_id "lnc-bbut00027708"	exon_number "2"
2	lncRNA	exon	69775798	69777300	.	+	.	gene_id "lnc-bbug12241"	transcript_id "lnc-bbut00027718"	exon_number "1"

2	lncRNA	exon	69777468	69781745	.	+	.	gene_id "lnc-bbug12241"	transcript_id "lnc-bbut00027718"	exon_number "2"
2	lncRNA	exon	69982824	69983299	.	+	.	gene_id "lnc-bbug12242"	transcript_id "lnc-bbut00027720"	exon_number "1"
2	lncRNA	exon	69992741	69992965	.	+	.	gene_id "lnc-bbug12242"	transcript_id "lnc-bbut00027720"	exon_number "2"
2	lncRNA	exon	69993687	69993833	.	+	.	gene_id "lnc-bbug12242"	transcript_id "lnc-bbut00027720"	exon_number "3"
2	lncRNA	exon	70007078	70007139	.	+	.	gene_id "lnc-bbug12242"	transcript_id "lnc-bbut00027720"	exon_number "4"
2	lncRNA	exon	70040111	70040233	.	+	.	gene_id "lnc-bbug12242"	transcript_id "lnc-bbut00027720"	exon_number "5"
2	lncRNA	exon	70045540	70045590	.	+	.	gene_id "lnc-bbug12242"	transcript_id "lnc-bbut00027720"	exon_number "6"
2	lncRNA	exon	69982835	69983299	.	+	.	gene_id "lnc-bbug12242"	transcript_id "lnc-bbut00027721"	exon_number "1"
2	lncRNA	exon	69992741	69992965	.	+	.	gene_id "lnc-bbug12242"	transcript_id "lnc-bbut00027721"	exon_number "2"
2	lncRNA	exon	70007078	70007139	.	+	.	gene_id "lnc-bbug12242"	transcript_id "lnc-bbut00027721"	exon_number "3"
2	lncRNA	exon	70040111	70040233	.	+	.	gene_id "lnc-bbug12242"	transcript_id "lnc-bbut00027721"	exon_number "4"
2	lncRNA	exon	70040465	70044264	.	+	.	gene_id "lnc-bbug12242"	transcript_id "lnc-bbut00027721"	exon_number "5"
2	lncRNA	exon	69982843	69983299	.	+	.	gene_id "lnc-bbug12242"	transcript_id "lnc-bbut00027722"	exon_number "1"
2	lncRNA	exon	69992741	69992965	.	+	.	gene_id "lnc-bbug12242"	transcript_id "lnc-bbut00027722"	exon_number "2"
2	lncRNA	exon	70007078	70007139	.	+	.	gene_id "lnc-bbug12242"	transcript_id "lnc-bbut00027722"	exon_number "3"
2	lncRNA	exon	70040111	70040233	.	+	.	gene_id "lnc-bbug12242"	transcript_id "lnc-bbut00027722"	exon_number "4"
2	lncRNA	exon	70045540	70045590	.	+	.	gene_id "lnc-bbug12242"	transcript_id "lnc-bbut00027722"	exon_number "5"
2	lncRNA	exon	70853774	70854052	.	+	.	gene_id "lnc-bbug12249"	transcript_id "lnc-bbut00027735"	exon_number "1"
2	lncRNA	exon	71199460	71199667	.	+	.	gene_id "lnc-bbug12252"	transcript_id "lnc-bbut00027740"	exon_number "1"
2	lncRNA	exon	71233993	71234333	.	+	.	gene_id "lnc-bbug12253"	transcript_id "lnc-bbut00027741"	exon_number "1"
2	lncRNA	exon	71239689	71239863	.	+	.	gene_id "lnc-bbug12253"	transcript_id "lnc-bbut00027741"	exon_number "2"
2	lncRNA	exon	71252888	71253131	.	+	.	gene_id "lnc-bbug12253"	transcript_id "lnc-bbut00027741"	exon_number "3"
2	lncRNA	exon	71913154	71913612	.	+	.	gene_id "lnc-bbug12258"	transcript_id "lnc-bbut00027746"	exon_number "1"
2	lncRNA	exon	71913844	71914223	.	+	.	gene_id "lnc-bbug12258"	transcript_id "lnc-bbut00027746"	exon_number "2"
2	lncRNA	exon	71917958	71918638	.	+	.	gene_id "lnc-bbug12258"	transcript_id "lnc-bbut00027746"	exon_number "3"
2	lncRNA	exon	72006926	72007053	.	+	.	gene_id "lnc-bbug12259"	transcript_id "lnc-bbut00027747"	exon_number "1"
2	lncRNA	exon	72012989	72014440	.	+	.	gene_id "lnc-bbug12259"	transcript_id "lnc-bbut00027747"	exon_number "2"
2	lncRNA	exon	72517964	72517977	.	+	.	gene_id "lnc-bbug12261"	transcript_id "lnc-bbut00027752"	exon_number "1"
2	lncRNA	exon	72518250	72521058	.	+	.	gene_id "lnc-bbug12261"	transcript_id "lnc-bbut00027752"	exon_number "2"
2	lncRNA	exon	72811194	72811348	.	+	.	gene_id "lnc-bbug12262"	transcript_id "lnc-bbut00027753"	exon_number "1"
2	lncRNA	exon	72812386	72812520	.	+	.	gene_id "lnc-bbug12262"	transcript_id "lnc-bbut00027753"	exon_number "2"
2	lncRNA	exon	72812692	72813482	.	+	.	gene_id "lnc-bbug12262"	transcript_id "lnc-bbut00027753"	exon_number "3"
2	lncRNA	exon	73901399	73901876	.	+	.	gene_id "lnc-bbug12265"	transcript_id "lnc-bbut00027762"	exon_number "1"
2	lncRNA	exon	74109577	74109797	.	+	.	gene_id "lnc-bbug12266"	transcript_id "lnc-bbut00027763"	exon_number "1"
2	lncRNA	exon	74895825	74895924	.	+	.	gene_id "lnc-bbug12274"	transcript_id "lnc-bbut00027783"	exon_number "1"
2	lncRNA	exon	74896269	74896404	.	+	.	gene_id "lnc-bbug12274"	transcript_id "lnc-bbut00027783"	exon_number "2"
2	lncRNA	exon	74896538	74896681	.	+	.	gene_id "lnc-bbug12274"	transcript_id "lnc-bbut00027783"	exon_number "3"
2	lncRNA	exon	74896816	74897000	.	+	.	gene_id "lnc-bbug12274"	transcript_id "lnc-bbut00027783"	exon_number "4"
2	lncRNA	exon	74895984	74896404	.	+	.	gene_id "lnc-bbug12274"	transcript_id "lnc-bbut00027784"	exon_number "1"
2	lncRNA	exon	74896538	74896681	.	+	.	gene_id "lnc-bbug12274"	transcript_id "lnc-bbut00027784"	exon_number "2"
2	lncRNA	exon	74896816	74897000	.	+	.	gene_id "lnc-bbug12274"	transcript_id "lnc-bbut00027784"	exon_number "3"
2	lncRNA	exon	75027296	75029623	.	+	.	gene_id "lnc-bbug12276"	transcript_id "lnc-bbut00027787"	exon_number "1"
2	lncRNA	exon	75029727	75032126	.	+	.	gene_id "lnc-bbug12276"	transcript_id "lnc-bbut00027787"	exon_number "2"
2	lncRNA	exon	75049733	75052637	.	+	.	gene_id "lnc-bbug12277"	transcript_id "lnc-bbut00027788"	exon_number "1"
2	lncRNA	exon	75135408	75136985	.	+	.	gene_id "lnc-bbug12278"	transcript_id "lnc-bbut00027789"	exon_number "1"
2	lncRNA	exon	75151830	75152708	.	+	.	gene_id "lnc-bbug12279"	transcript_id "lnc-bbut00027790"	exon_number "1"
2	lncRNA	exon	75152834	75152964	.	+	.	gene_id "lnc-bbug12279"	transcript_id "lnc-bbut00027790"	exon_number "2"
2	lncRNA	exon	75169049	75170546	.	+	.	gene_id "lnc-bbug12279"	transcript_id "lnc-bbut00027790"	exon_number "3"
2	lncRNA	exon	75173309	75174152	.	+	.	gene_id "lnc-bbug12280"	transcript_id "lnc-bbut00027791"	exon_number "1"
2	lncRNA	exon	75191510	75192769	.	+	.	gene_id "lnc-bbug12281"	transcript_id "lnc-bbut00027792"	exon_number "1"
2	lncRNA	exon	75193837	75194327	.	+	.	gene_id "lnc-bbug12281"	transcript_id "lnc-bbut00027792"	exon_number "2"
2	lncRNA	exon	76522864	76523432	.	+	.	gene_id "lnc-bbug12283"	transcript_id "lnc-bbut00027794"	exon_number "1"

2	lncRNA	exon	76523891	76524467	.	+	.	gene_id "lnc-bbug12283"	transcript_id "lnc-bbut00027794"	exon_number "2"
2	lncRNA	exon	77428503	77429322	.	+	.	gene_id "lnc-bbug12286"	transcript_id "lnc-bbut00027799"	exon_number "1"
2	lncRNA	exon	77429705	77430885	.	+	.	gene_id "lnc-bbug12286"	transcript_id "lnc-bbut00027799"	exon_number "2"
2	lncRNA	exon	77433300	77433421	.	+	.	gene_id "lnc-bbug12286"	transcript_id "lnc-bbut00027799"	exon_number "3"
2	lncRNA	exon	77434654	77435313	.	+	.	gene_id "lnc-bbug12286"	transcript_id "lnc-bbut00027799"	exon_number "4"
2	lncRNA	exon	77433469	77434570	.	+	.	gene_id "lnc-bbug12287"	transcript_id "lnc-bbut00027800"	exon_number "1"
2	lncRNA	exon	77679938	77681741	.	+	.	gene_id "lnc-bbug12289"	transcript_id "lnc-bbut00027809"	exon_number "1"
2	lncRNA	exon	77704923	77705007	.	+	.	gene_id "lnc-bbug12289"	transcript_id "lnc-bbut00027809"	exon_number "2"
2	lncRNA	exon	77706623	77706798	.	+	.	gene_id "lnc-bbug12289"	transcript_id "lnc-bbut00027809"	exon_number "3"
2	lncRNA	exon	77679938	77681741	.	+	.	gene_id "lnc-bbug12289"	transcript_id "lnc-bbut00027808"	exon_number "1"
2	lncRNA	exon	77704923	77706798	.	+	.	gene_id "lnc-bbug12289"	transcript_id "lnc-bbut00027808"	exon_number "2"
2	lncRNA	exon	77927038	77930474	.	+	.	gene_id "lnc-bbug12291"	transcript_id "lnc-bbut00027814"	exon_number "1"
2	lncRNA	exon	80087432	80087747	.	+	.	gene_id "lnc-bbug12298"	transcript_id "lnc-bbut00027839"	exon_number "1"
2	lncRNA	exon	80090326	80090689	.	+	.	gene_id "lnc-bbug12298"	transcript_id "lnc-bbut00027839"	exon_number "2"
2	lncRNA	exon	80735990	80738028	.	+	.	gene_id "lnc-bbug12300"	transcript_id "lnc-bbut00027844"	exon_number "1"
2	lncRNA	exon	82274640	82275208	.	+	.	gene_id "lnc-bbug12301"	transcript_id "lnc-bbut00027845"	exon_number "1"
2	lncRNA	exon	83221385	83222546	.	+	.	gene_id "lnc-bbug12307"	transcript_id "lnc-bbut00027855"	exon_number "1"
2	lncRNA	exon	85142281	85143203	.	+	.	gene_id "lnc-bbug12313"	transcript_id "lnc-bbut00027871"	exon_number "1"
2	lncRNA	exon	85143783	85149485	.	+	.	gene_id "lnc-bbug12313"	transcript_id "lnc-bbut00027871"	exon_number "2"
2	lncRNA	exon	85336404	85338111	.	+	.	gene_id "lnc-bbug12314"	transcript_id "lnc-bbut00027872"	exon_number "1"
2	lncRNA	exon	85351704	85351768	.	+	.	gene_id "lnc-bbug12314"	transcript_id "lnc-bbut00027872"	exon_number "2"
2	lncRNA	exon	85353576	85354098	.	+	.	gene_id "lnc-bbug12314"	transcript_id "lnc-bbut00027872"	exon_number "3"
2	lncRNA	exon	87060692	87062337	.	+	.	gene_id "lnc-bbug12320"	transcript_id "lnc-bbut00027883"	exon_number "1"
2	lncRNA	exon	87637676	87643619	.	+	.	gene_id "lnc-bbug12321"	transcript_id "lnc-bbut00027884"	exon_number "1"
2	lncRNA	exon	87644181	87644243	.	+	.	gene_id "lnc-bbug12321"	transcript_id "lnc-bbut00027884"	exon_number "2"
2	lncRNA	exon	87725894	87725996	.	+	.	gene_id "lnc-bbug12322"	transcript_id "lnc-bbut00027885"	exon_number "1"
2	lncRNA	exon	87726623	87726679	.	+	.	gene_id "lnc-bbug12322"	transcript_id "lnc-bbut00027885"	exon_number "2"
2	lncRNA	exon	87728701	87728747	.	+	.	gene_id "lnc-bbug12322"	transcript_id "lnc-bbut00027885"	exon_number "3"
2	lncRNA	exon	87744559	87746611	.	+	.	gene_id "lnc-bbug12323"	transcript_id "lnc-bbut00027886"	exon_number "1"
2	lncRNA	exon	87746916	87746982	.	+	.	gene_id "lnc-bbug12323"	transcript_id "lnc-bbut00027886"	exon_number "2"
2	lncRNA	exon	87747079	87747298	.	+	.	gene_id "lnc-bbug12323"	transcript_id "lnc-bbut00027886"	exon_number "3"
2	lncRNA	exon	87768093	87769710	.	+	.	gene_id "lnc-bbug12323"	transcript_id "lnc-bbut00027886"	exon_number "4"
2	lncRNA	exon	87744559	87746611	.	+	.	gene_id "lnc-bbug12323"	transcript_id "lnc-bbut00027887"	exon_number "1"
2	lncRNA	exon	87746916	87746982	.	+	.	gene_id "lnc-bbug12323"	transcript_id "lnc-bbut00027887"	exon_number "2"
2	lncRNA	exon	87747079	87747298	.	+	.	gene_id "lnc-bbug12323"	transcript_id "lnc-bbut00027887"	exon_number "3"
2	lncRNA	exon	87768093	87768217	.	+	.	gene_id "lnc-bbug12323"	transcript_id "lnc-bbut00027887"	exon_number "4"
2	lncRNA	exon	87792669	87793111	.	+	.	gene_id "lnc-bbug12323"	transcript_id "lnc-bbut00027887"	exon_number "5"
2	lncRNA	exon	87971753	87972179	.	+	.	gene_id "lnc-bbug12324"	transcript_id "lnc-bbut00027888"	exon_number "1"
2	lncRNA	exon	87972395	87972520	.	+	.	gene_id "lnc-bbug12324"	transcript_id "lnc-bbut00027888"	exon_number "2"
2	lncRNA	exon	89802250	89802490	.	+	.	gene_id "lnc-bbug12334"	transcript_id "lnc-bbut00027944"	exon_number "1"
2	lncRNA	exon	89808162	89810135	.	+	.	gene_id "lnc-bbug12334"	transcript_id "lnc-bbut00027944"	exon_number "2"
2	lncRNA	exon	90109999	90111215	.	+	.	gene_id "lnc-bbug12335"	transcript_id "lnc-bbut00027945"	exon_number "1"
2	lncRNA	exon	90117670	90118148	.	+	.	gene_id "lnc-bbug12335"	transcript_id "lnc-bbut00027945"	exon_number "2"
2	lncRNA	exon	92319333	92319704	.	+	.	gene_id "lnc-bbug12343"	transcript_id "lnc-bbut00027956"	exon_number "1"
2	lncRNA	exon	92381512	92383438	.	+	.	gene_id "lnc-bbug12344"	transcript_id "lnc-bbut00027957"	exon_number "1"
2	lncRNA	exon	92389273	92389399	.	+	.	gene_id "lnc-bbug12344"	transcript_id "lnc-bbut00027957"	exon_number "2"
2	lncRNA	exon	92389517	92389847	.	+	.	gene_id "lnc-bbug12344"	transcript_id "lnc-bbut00027957"	exon_number "3"
2	lncRNA	exon	94239498	94240244	.	+	.	gene_id "lnc-bbug12349"	transcript_id "lnc-bbut00027962"	exon_number "1"
2	lncRNA	exon	94241315	94241409	.	+	.	gene_id "lnc-bbug12349"	transcript_id "lnc-bbut00027962"	exon_number "2"
2	lncRNA	exon	94242238	94242298	.	+	.	gene_id "lnc-bbug12349"	transcript_id "lnc-bbut00027962"	exon_number "3"
2	lncRNA	exon	94244155	94244213	.	+	.	gene_id "lnc-bbug12349"	transcript_id "lnc-bbut00027962"	exon_number "4"
2	lncRNA	exon	94245158	94245366	.	+	.	gene_id "lnc-bbug12349"	transcript_id "lnc-bbut00027962"	exon_number "5"

2	lncRNA	exon	95259924	95260180	.	+	.	gene_id "lnc-bbug12351"	transcript_id "lnc-bbut00027964"	exon_number "1"
2	lncRNA	exon	95275939	95276288	.	+	.	gene_id "lnc-bbug12351"	transcript_id "lnc-bbut00027964"	exon_number "2"
2	lncRNA	exon	103103204	103103595	.	+	.	gene_id "lnc-bbug12365"	transcript_id "lnc-bbut00028021"	exon_number "1"
2	lncRNA	exon	104792841	104793949	.	+	.	gene_id "lnc-bbug12366"	transcript_id "lnc-bbut00028022"	exon_number "1"
2	lncRNA	exon	104794454	104794697	.	+	.	gene_id "lnc-bbug12366"	transcript_id "lnc-bbut00028022"	exon_number "2"
2	lncRNA	exon	105568700	105568997	.	+	.	gene_id "lnc-bbug12369"	transcript_id "lnc-bbut00028042"	exon_number "1"
2	lncRNA	exon	105624137	105624324	.	+	.	gene_id "lnc-bbug12369"	transcript_id "lnc-bbut00028042"	exon_number "2"
2	lncRNA	exon	105630577	105630692	.	+	.	gene_id "lnc-bbug12369"	transcript_id "lnc-bbut00028042"	exon_number "3"
2	lncRNA	exon	105632682	105632845	.	+	.	gene_id "lnc-bbug12369"	transcript_id "lnc-bbut00028042"	exon_number "4"
2	lncRNA	exon	105638698	105638826	.	+	.	gene_id "lnc-bbug12369"	transcript_id "lnc-bbut00028042"	exon_number "5"
2	lncRNA	exon	105698082	105698139	.	+	.	gene_id "lnc-bbug12369"	transcript_id "lnc-bbut00028042"	exon_number "6"
2	lncRNA	exon	105709881	105711305	.	+	.	gene_id "lnc-bbug12369"	transcript_id "lnc-bbut00028042"	exon_number "7"
2	lncRNA	exon	105624004	105624324	.	+	.	gene_id "lnc-bbug12369"	transcript_id "lnc-bbut00028043"	exon_number "1"
2	lncRNA	exon	105630577	105630692	.	+	.	gene_id "lnc-bbug12369"	transcript_id "lnc-bbut00028043"	exon_number "2"
2	lncRNA	exon	105632682	105632845	.	+	.	gene_id "lnc-bbug12369"	transcript_id "lnc-bbut00028043"	exon_number "3"
2	lncRNA	exon	105638698	105638826	.	+	.	gene_id "lnc-bbug12369"	transcript_id "lnc-bbut00028043"	exon_number "4"
2	lncRNA	exon	105666672	105667758	.	+	.	gene_id "lnc-bbug12369"	transcript_id "lnc-bbut00028043"	exon_number "5"
2	lncRNA	exon	105624004	105624324	.	+	.	gene_id "lnc-bbug12369"	transcript_id "lnc-bbut00028045"	exon_number "1"
2	lncRNA	exon	105630580	105630692	.	+	.	gene_id "lnc-bbug12369"	transcript_id "lnc-bbut00028045"	exon_number "2"
2	lncRNA	exon	105632682	105632845	.	+	.	gene_id "lnc-bbug12369"	transcript_id "lnc-bbut00028045"	exon_number "3"
2	lncRNA	exon	105638698	105638826	.	+	.	gene_id "lnc-bbug12369"	transcript_id "lnc-bbut00028045"	exon_number "4"
2	lncRNA	exon	105698082	105698139	.	+	.	gene_id "lnc-bbug12369"	transcript_id "lnc-bbut00028045"	exon_number "5"
2	lncRNA	exon	105709881	105711305	.	+	.	gene_id "lnc-bbug12369"	transcript_id "lnc-bbut00028045"	exon_number "6"
2	lncRNA	exon	105624004	105624324	.	+	.	gene_id "lnc-bbug12369"	transcript_id "lnc-bbut00028044"	exon_number "1"
2	lncRNA	exon	105630577	105630692	.	+	.	gene_id "lnc-bbug12369"	transcript_id "lnc-bbut00028044"	exon_number "2"
2	lncRNA	exon	105632682	105632845	.	+	.	gene_id "lnc-bbug12369"	transcript_id "lnc-bbut00028044"	exon_number "3"
2	lncRNA	exon	105638698	105638826	.	+	.	gene_id "lnc-bbug12369"	transcript_id "lnc-bbut00028044"	exon_number "4"
2	lncRNA	exon	105709881	105711305	.	+	.	gene_id "lnc-bbug12369"	transcript_id "lnc-bbut00028044"	exon_number "5"
2	lncRNA	exon	112178007	112178339	.	+	.	gene_id "lnc-bbug12374"	transcript_id "lnc-bbut00028050"	exon_number "1"
2	lncRNA	exon	114343943	114344574	.	+	.	gene_id "lnc-bbug12380"	transcript_id "lnc-bbut00028057"	exon_number "1"
2	lncRNA	exon	114874807	114875040	.	+	.	gene_id "lnc-bbug12382"	transcript_id "lnc-bbut00028068"	exon_number "1"
2	lncRNA	exon	114875373	114875617	.	+	.	gene_id "lnc-bbug12382"	transcript_id "lnc-bbut00028068"	exon_number "2"
2	lncRNA	exon	115078815	115078885	.	+	.	gene_id "lnc-bbug12384"	transcript_id "lnc-bbut00028071"	exon_number "1"
2	lncRNA	exon	115078925	115079084	.	+	.	gene_id "lnc-bbug12384"	transcript_id "lnc-bbut00028071"	exon_number "2"
2	lncRNA	exon	115169629	115169791	.	+	.	gene_id "lnc-bbug12384"	transcript_id "lnc-bbut00028071"	exon_number "3"
2	lncRNA	exon	118207345	118207469	.	+	.	gene_id "lnc-bbug12390"	transcript_id "lnc-bbut00028087"	exon_number "1"
2	lncRNA	exon	118212264	118212373	.	+	.	gene_id "lnc-bbug12390"	transcript_id "lnc-bbut00028087"	exon_number "2"
2	lncRNA	exon	118244326	118244448	.	+	.	gene_id "lnc-bbug12390"	transcript_id "lnc-bbut00028087"	exon_number "3"
2	lncRNA	exon	118330081	118330687	.	+	.	gene_id "lnc-bbug12392"	transcript_id "lnc-bbut00028089"	exon_number "1"
2	lncRNA	exon	118332784	118333576	.	+	.	gene_id "lnc-bbug12392"	transcript_id "lnc-bbut00028089"	exon_number "2"
2	lncRNA	exon	118334535	118335564	.	+	.	gene_id "lnc-bbug12393"	transcript_id "lnc-bbut00028090"	exon_number "1"
2	lncRNA	exon	118338845	118338864	.	+	.	gene_id "lnc-bbug12393"	transcript_id "lnc-bbut00028090"	exon_number "2"
2	lncRNA	exon	122907201	122907659	.	+	.	gene_id "lnc-bbug12401"	transcript_id "lnc-bbut00028104"	exon_number "1"
2	lncRNA	exon	122908199	122908245	.	+	.	gene_id "lnc-bbug12401"	transcript_id "lnc-bbut00028104"	exon_number "2"
2	lncRNA	exon	122916592	122919081	.	+	.	gene_id "lnc-bbug12401"	transcript_id "lnc-bbut00028104"	exon_number "3"
2	lncRNA	exon	124044705	124044853	.	+	.	gene_id "lnc-bbug12404"	transcript_id "lnc-bbut00028112"	exon_number "1"
2	lncRNA	exon	124045891	124046008	.	+	.	gene_id "lnc-bbug12404"	transcript_id "lnc-bbut00028112"	exon_number "2"
2	lncRNA	exon	124047877	124047939	.	+	.	gene_id "lnc-bbug12404"	transcript_id "lnc-bbut00028112"	exon_number "3"
2	lncRNA	exon	124049265	124049553	.	+	.	gene_id "lnc-bbug12404"	transcript_id "lnc-bbut00028112"	exon_number "4"
2	lncRNA	exon	124607416	124609939	.	+	.	gene_id "lnc-bbug12411"	transcript_id "lnc-bbut00028126"	exon_number "1"
2	lncRNA	exon	124809520	124809940	.	+	.	gene_id "lnc-bbug12412"	transcript_id "lnc-bbut00028132"	exon_number "1"
2	lncRNA	exon	124817473	124819873	.	+	.	gene_id "lnc-bbug12412"	transcript_id "lnc-bbut00028132"	exon_number "2"

2	lncRNA	exon	125764182	125764248	.	+	.	gene_id "lnc-bbug12416"	transcript_id "lnc-bbut00028139"	exon_number "1"
2	lncRNA	exon	125765434	125765883	.	+	.	gene_id "lnc-bbug12416"	transcript_id "lnc-bbut00028139"	exon_number "2"
2	lncRNA	exon	126267767	126269923	.	+	.	gene_id "lnc-bbug12421"	transcript_id "lnc-bbut00028145"	exon_number "1"
2	lncRNA	exon	127610781	127611442	.	+	.	gene_id "lnc-bbug12422"	transcript_id "lnc-bbut00028146"	exon_number "1"
2	lncRNA	exon	128622295	128622538	.	+	.	gene_id "lnc-bbug12424"	transcript_id "lnc-bbut00028148"	exon_number "1"
2	lncRNA	exon	132477762	132478610	.	+	.	gene_id "lnc-bbug12432"	transcript_id "lnc-bbut00028170"	exon_number "1"
2	lncRNA	exon	132485446	132485584	.	+	.	gene_id "lnc-bbug12432"	transcript_id "lnc-bbut00028170"	exon_number "2"
2	lncRNA	exon	132486326	132486445	.	+	.	gene_id "lnc-bbug12432"	transcript_id "lnc-bbut00028170"	exon_number "3"
2	lncRNA	exon	133199696	133200186	.	+	.	gene_id "lnc-bbug12435"	transcript_id "lnc-bbut00028176"	exon_number "1"
2	lncRNA	exon	133200791	133200928	.	+	.	gene_id "lnc-bbug12435"	transcript_id "lnc-bbut00028176"	exon_number "2"
2	lncRNA	exon	136107351	136107787	.	+	.	gene_id "lnc-bbug12436"	transcript_id "lnc-bbut00028183"	exon_number "1"
2	lncRNA	exon	137608332	137608550	.	+	.	gene_id "lnc-bbug12438"	transcript_id "lnc-bbut00028188"	exon_number "1"
2	lncRNA	exon	137609010	137609113	.	+	.	gene_id "lnc-bbug12438"	transcript_id "lnc-bbut00028188"	exon_number "2"
2	lncRNA	exon	138803562	138803565	.	+	.	gene_id "lnc-bbug12440"	transcript_id "lnc-bbut00028196"	exon_number "1"
2	lncRNA	exon	138805184	138805828	.	+	.	gene_id "lnc-bbug12440"	transcript_id "lnc-bbut00028196"	exon_number "2"
2	lncRNA	exon	141981816	141982024	.	+	.	gene_id "lnc-bbug12450"	transcript_id "lnc-bbut00028221"	exon_number "1"
2	lncRNA	exon	141989285	141989342	.	+	.	gene_id "lnc-bbug12450"	transcript_id "lnc-bbut00028221"	exon_number "2"
2	lncRNA	exon	141991519	141991615	.	+	.	gene_id "lnc-bbug12450"	transcript_id "lnc-bbut00028221"	exon_number "3"
2	lncRNA	exon	141993351	141993585	.	+	.	gene_id "lnc-bbug12450"	transcript_id "lnc-bbut00028221"	exon_number "4"
2	lncRNA	exon	141996942	141997031	.	+	.	gene_id "lnc-bbug12450"	transcript_id "lnc-bbut00028221"	exon_number "5"
2	lncRNA	exon	141981816	141982024	.	+	.	gene_id "lnc-bbug12450"	transcript_id "lnc-bbut00028220"	exon_number "1"
2	lncRNA	exon	141989285	141989342	.	+	.	gene_id "lnc-bbug12450"	transcript_id "lnc-bbut00028220"	exon_number "2"
2	lncRNA	exon	141991519	141991615	.	+	.	gene_id "lnc-bbug12450"	transcript_id "lnc-bbut00028220"	exon_number "3"
2	lncRNA	exon	141993351	141993582	.	+	.	gene_id "lnc-bbug12450"	transcript_id "lnc-bbut00028220"	exon_number "4"
2	lncRNA	exon	141996939	141997031	.	+	.	gene_id "lnc-bbug12450"	transcript_id "lnc-bbut00028220"	exon_number "5"
2	lncRNA	exon	142490238	142490350	.	+	.	gene_id "lnc-bbug12457"	transcript_id "lnc-bbut00028232"	exon_number "1"
2	lncRNA	exon	142497335	142497476	.	+	.	gene_id "lnc-bbug12457"	transcript_id "lnc-bbut00028232"	exon_number "2"
2	lncRNA	exon	142500532	142500822	.	+	.	gene_id "lnc-bbug12457"	transcript_id "lnc-bbut00028232"	exon_number "3"
2	lncRNA	exon	142490384	142490552	.	+	.	gene_id "lnc-bbug12457"	transcript_id "lnc-bbut00028233"	exon_number "1"
2	lncRNA	exon	142497335	142497476	.	+	.	gene_id "lnc-bbug12457"	transcript_id "lnc-bbut00028233"	exon_number "2"
2	lncRNA	exon	142500532	142500822	.	+	.	gene_id "lnc-bbug12457"	transcript_id "lnc-bbut00028233"	exon_number "3"
2	lncRNA	exon	144040441	144040548	.	+	.	gene_id "lnc-bbug12469"	transcript_id "lnc-bbut00028270"	exon_number "1"
2	lncRNA	exon	144041692	144041742	.	+	.	gene_id "lnc-bbug12469"	transcript_id "lnc-bbut00028270"	exon_number "2"
2	lncRNA	exon	144043267	144043524	.	+	.	gene_id "lnc-bbug12469"	transcript_id "lnc-bbut00028270"	exon_number "3"
2	lncRNA	exon	148466763	148466858	.	+	.	gene_id "lnc-bbug12486"	transcript_id "lnc-bbut00028326"	exon_number "1"
2	lncRNA	exon	148495484	148495629	.	+	.	gene_id "lnc-bbug12486"	transcript_id "lnc-bbut00028326"	exon_number "2"
2	lncRNA	exon	148496502	148496744	.	+	.	gene_id "lnc-bbug12486"	transcript_id "lnc-bbut00028326"	exon_number "3"
2	lncRNA	exon	148498361	148500274	.	+	.	gene_id "lnc-bbug12486"	transcript_id "lnc-bbut00028326"	exon_number "4"
2	lncRNA	exon	148466763	148466858	.	+	.	gene_id "lnc-bbug12486"	transcript_id "lnc-bbut00028325"	exon_number "1"
2	lncRNA	exon	148495484	148495629	.	+	.	gene_id "lnc-bbug12486"	transcript_id "lnc-bbut00028325"	exon_number "2"
2	lncRNA	exon	148498361	148500274	.	+	.	gene_id "lnc-bbug12486"	transcript_id "lnc-bbut00028325"	exon_number "3"
2	lncRNA	exon	148745007	148745525	.	+	.	gene_id "lnc-bbug12488"	transcript_id "lnc-bbut00028333"	exon_number "1"
2	lncRNA	exon	148760555	148760904	.	+	.	gene_id "lnc-bbug12488"	transcript_id "lnc-bbut00028333"	exon_number "2"
2	lncRNA	exon	148745007	148745525	.	+	.	gene_id "lnc-bbug12488"	transcript_id "lnc-bbut00028334"	exon_number "1"
2	lncRNA	exon	148760555	148760700	.	+	.	gene_id "lnc-bbug12488"	transcript_id "lnc-bbut00028334"	exon_number "2"
2	lncRNA	exon	148761070	148762516	.	+	.	gene_id "lnc-bbug12488"	transcript_id "lnc-bbut00028334"	exon_number "3"
2	lncRNA	exon	148762619	148764729	.	+	.	gene_id "lnc-bbug12489"	transcript_id "lnc-bbut00028335"	exon_number "1"
2	lncRNA	exon	148892123	148892190	.	+	.	gene_id "lnc-bbug12491"	transcript_id "lnc-bbut00028338"	exon_number "1"
2	lncRNA	exon	148892756	148892797	.	+	.	gene_id "lnc-bbug12491"	transcript_id "lnc-bbut00028338"	exon_number "2"
2	lncRNA	exon	148892900	148893562	.	+	.	gene_id "lnc-bbug12491"	transcript_id "lnc-bbut00028338"	exon_number "3"
2	lncRNA	exon	149008012	149008251	.	+	.	gene_id "lnc-bbug12494"	transcript_id "lnc-bbut00028343"	exon_number "1"
2	lncRNA	exon	149054226	149054471	.	+	.	gene_id "lnc-bbug12494"	transcript_id "lnc-bbut00028343"	exon_number "2"

2	lncRNA	exon	149062083	149062487	.	+	.	gene_id "lnc-bbug12494"	transcript_id "lnc-bbut00028343"	exon_number "3"
2	lncRNA	exon	149340739	149340835	.	+	.	gene_id "lnc-bbug12495"	transcript_id "lnc-bbut00028344"	exon_number "1"
2	lncRNA	exon	149340923	149341074	.	+	.	gene_id "lnc-bbug12495"	transcript_id "lnc-bbut00028344"	exon_number "2"
2	lncRNA	exon	153950522	153952016	.	+	.	gene_id "lnc-bbug12504"	transcript_id "lnc-bbut00028405"	exon_number "1"
2	lncRNA	exon	153952594	153952726	.	+	.	gene_id "lnc-bbug12504"	transcript_id "lnc-bbut00028405"	exon_number "2"
2	lncRNA	exon	153962803	153963591	.	+	.	gene_id "lnc-bbug12504"	transcript_id "lnc-bbut00028405"	exon_number "3"
2	lncRNA	exon	154236132	154237071	.	+	.	gene_id "lnc-bbug12505"	transcript_id "lnc-bbut00028408"	exon_number "1"
2	lncRNA	exon	154239407	154239538	.	+	.	gene_id "lnc-bbug12505"	transcript_id "lnc-bbut00028408"	exon_number "2"
2	lncRNA	exon	154251411	154251460	.	+	.	gene_id "lnc-bbug12505"	transcript_id "lnc-bbut00028408"	exon_number "3"
2	lncRNA	exon	154236132	154237062	.	+	.	gene_id "lnc-bbug12505"	transcript_id "lnc-bbut00028410"	exon_number "1"
2	lncRNA	exon	154239410	154239538	.	+	.	gene_id "lnc-bbug12505"	transcript_id "lnc-bbut00028410"	exon_number "2"
2	lncRNA	exon	154251411	154251460	.	+	.	gene_id "lnc-bbug12505"	transcript_id "lnc-bbut00028410"	exon_number "3"
2	lncRNA	exon	154236132	154237071	.	+	.	gene_id "lnc-bbug12505"	transcript_id "lnc-bbut00028409"	exon_number "1"
2	lncRNA	exon	154239368	154239538	.	+	.	gene_id "lnc-bbug12505"	transcript_id "lnc-bbut00028409"	exon_number "2"
2	lncRNA	exon	154251411	154251460	.	+	.	gene_id "lnc-bbug12505"	transcript_id "lnc-bbut00028409"	exon_number "3"
2	lncRNA	exon	154236132	154237071	.	+	.	gene_id "lnc-bbug12505"	transcript_id "lnc-bbut00028407"	exon_number "1"
2	lncRNA	exon	154239410	154239538	.	+	.	gene_id "lnc-bbug12505"	transcript_id "lnc-bbut00028407"	exon_number "2"
2	lncRNA	exon	154251411	154251460	.	+	.	gene_id "lnc-bbug12505"	transcript_id "lnc-bbut00028407"	exon_number "3"
2	lncRNA	exon	154236132	154237062	.	+	.	gene_id "lnc-bbug12505"	transcript_id "lnc-bbut00028406"	exon_number "1"
2	lncRNA	exon	154239407	154239538	.	+	.	gene_id "lnc-bbug12505"	transcript_id "lnc-bbut00028406"	exon_number "2"
2	lncRNA	exon	154251411	154251460	.	+	.	gene_id "lnc-bbug12505"	transcript_id "lnc-bbut00028406"	exon_number "3"
2	lncRNA	exon	155893182	155893291	.	+	.	gene_id "lnc-bbug12507"	transcript_id "lnc-bbut00028413"	exon_number "1"
2	lncRNA	exon	155910049	155910254	.	+	.	gene_id "lnc-bbug12507"	transcript_id "lnc-bbut00028413"	exon_number "2"
2	lncRNA	exon	155912047	155912095	.	+	.	gene_id "lnc-bbug12507"	transcript_id "lnc-bbut00028413"	exon_number "3"
2	lncRNA	exon	156270498	156270674	.	+	.	gene_id "lnc-bbug12509"	transcript_id "lnc-bbut00028415"	exon_number "1"
2	lncRNA	exon	156278941	156279072	.	+	.	gene_id "lnc-bbug12509"	transcript_id "lnc-bbut00028415"	exon_number "2"
2	lncRNA	exon	156884195	156884258	.	+	.	gene_id "lnc-bbug12511"	transcript_id "lnc-bbut00028417"	exon_number "1"
2	lncRNA	exon	156906242	156906474	.	+	.	gene_id "lnc-bbug12511"	transcript_id "lnc-bbut00028417"	exon_number "2"
2	lncRNA	exon	156908121	156909291	.	+	.	gene_id "lnc-bbug12511"	transcript_id "lnc-bbut00028417"	exon_number "3"
2	lncRNA	exon	157539673	157539692	.	+	.	gene_id "lnc-bbug12516"	transcript_id "lnc-bbut00028425"	exon_number "1"
2	lncRNA	exon	157540059	157540187	.	+	.	gene_id "lnc-bbug12516"	transcript_id "lnc-bbut00028425"	exon_number "2"
2	lncRNA	exon	157540753	157540835	.	+	.	gene_id "lnc-bbug12516"	transcript_id "lnc-bbut00028425"	exon_number "3"
2	lncRNA	exon	157541840	157541958	.	+	.	gene_id "lnc-bbug12516"	transcript_id "lnc-bbut00028425"	exon_number "4"
2	lncRNA	exon	157667155	157667293	.	+	.	gene_id "lnc-bbug12517"	transcript_id "lnc-bbut00028426"	exon_number "1"
2	lncRNA	exon	157670743	157671836	.	+	.	gene_id "lnc-bbug12517"	transcript_id "lnc-bbut00028426"	exon_number "2"
2	lncRNA	exon	157811301	157811359	.	+	.	gene_id "lnc-bbug12519"	transcript_id "lnc-bbut00028428"	exon_number "1"
2	lncRNA	exon	157832484	157832513	.	+	.	gene_id "lnc-bbug12519"	transcript_id "lnc-bbut00028428"	exon_number "2"
2	lncRNA	exon	157847074	157847281	.	+	.	gene_id "lnc-bbug12519"	transcript_id "lnc-bbut00028428"	exon_number "3"
2	lncRNA	exon	157848065	157851080	.	+	.	gene_id "lnc-bbug12519"	transcript_id "lnc-bbut00028428"	exon_number "4"
2	lncRNA	exon	157811301	157811359	.	+	.	gene_id "lnc-bbug12519"	transcript_id "lnc-bbut00028429"	exon_number "1"
2	lncRNA	exon	157832484	157832513	.	+	.	gene_id "lnc-bbug12519"	transcript_id "lnc-bbut00028429"	exon_number "2"
2	lncRNA	exon	157847074	157847281	.	+	.	gene_id "lnc-bbug12519"	transcript_id "lnc-bbut00028429"	exon_number "3"
2	lncRNA	exon	157848065	157848243	.	+	.	gene_id "lnc-bbug12519"	transcript_id "lnc-bbut00028429"	exon_number "4"
2	lncRNA	exon	157868421	157869609	.	+	.	gene_id "lnc-bbug12519"	transcript_id "lnc-bbut00028429"	exon_number "5"
2	lncRNA	exon	158905412	158905773	.	+	.	gene_id "lnc-bbug12520"	transcript_id "lnc-bbut00028430"	exon_number "1"
2	lncRNA	exon	159293237	159294978	.	+	.	gene_id "lnc-bbug12523"	transcript_id "lnc-bbut00028435"	exon_number "1"
2	lncRNA	exon	159295132	159295817	.	+	.	gene_id "lnc-bbug12523"	transcript_id "lnc-bbut00028435"	exon_number "2"
2	lncRNA	exon	159340257	159340523	.	+	.	gene_id "lnc-bbug12525"	transcript_id "lnc-bbut00028437"	exon_number "1"
2	lncRNA	exon	159341880	159344629	.	+	.	gene_id "lnc-bbug12525"	transcript_id "lnc-bbut00028437"	exon_number "2"
2	lncRNA	exon	159346734	159347482	.	+	.	gene_id "lnc-bbug12526"	transcript_id "lnc-bbut00028438"	exon_number "1"
2	lncRNA	exon	159347966	159350311	.	+	.	gene_id "lnc-bbug12526"	transcript_id "lnc-bbut00028438"	exon_number "2"
2	lncRNA	exon	159378948	159379039	.	+	.	gene_id "lnc-bbug12528"	transcript_id "lnc-bbut00028440"	exon_number "1"

2	lncRNA	exon	159379795	159379963	.	+	.	gene_id "lnc-bbug12528"	transcript_id "lnc-bbut00028440"	exon_number "2"
2	lncRNA	exon	159381079	159381689	.	+	.	gene_id "lnc-bbug12528"	transcript_id "lnc-bbut00028440"	exon_number "3"
2	lncRNA	exon	159989971	159990274	.	+	.	gene_id "lnc-bbug12542"	transcript_id "lnc-bbut00028476"	exon_number "1"
2	lncRNA	exon	159999224	160001212	.	+	.	gene_id "lnc-bbug12542"	transcript_id "lnc-bbut00028476"	exon_number "2"
2	lncRNA	exon	161070889	161071150	.	+	.	gene_id "lnc-bbug12558"	transcript_id "lnc-bbut00028539"	exon_number "1"
2	lncRNA	exon	161072395	161072557	.	+	.	gene_id "lnc-bbug12558"	transcript_id "lnc-bbut00028539"	exon_number "2"
2	lncRNA	exon	161078938	161079071	.	+	.	gene_id "lnc-bbug12558"	transcript_id "lnc-bbut00028539"	exon_number "3"
2	lncRNA	exon	161079547	161079735	.	+	.	gene_id "lnc-bbug12558"	transcript_id "lnc-bbut00028539"	exon_number "4"
2	lncRNA	exon	161080195	161080654	.	+	.	gene_id "lnc-bbug12558"	transcript_id "lnc-bbut00028539"	exon_number "5"
2	lncRNA	exon	161071736	161071803	.	+	.	gene_id "lnc-bbug12558"	transcript_id "lnc-bbut00028542"	exon_number "1"
2	lncRNA	exon	161072395	161072557	.	+	.	gene_id "lnc-bbug12558"	transcript_id "lnc-bbut00028542"	exon_number "2"
2	lncRNA	exon	161077110	161077234	.	+	.	gene_id "lnc-bbug12558"	transcript_id "lnc-bbut00028542"	exon_number "3"
2	lncRNA	exon	161078938	161079071	.	+	.	gene_id "lnc-bbug12558"	transcript_id "lnc-bbut00028542"	exon_number "4"
2	lncRNA	exon	161079547	161079735	.	+	.	gene_id "lnc-bbug12558"	transcript_id "lnc-bbut00028542"	exon_number "5"
2	lncRNA	exon	161080195	161080654	.	+	.	gene_id "lnc-bbug12558"	transcript_id "lnc-bbut00028542"	exon_number "6"
2	lncRNA	exon	161071736	161071803	.	+	.	gene_id "lnc-bbug12558"	transcript_id "lnc-bbut00028541"	exon_number "1"
2	lncRNA	exon	161072395	161072557	.	+	.	gene_id "lnc-bbug12558"	transcript_id "lnc-bbut00028541"	exon_number "2"
2	lncRNA	exon	161078938	161079071	.	+	.	gene_id "lnc-bbug12558"	transcript_id "lnc-bbut00028541"	exon_number "3"
2	lncRNA	exon	161079547	161079735	.	+	.	gene_id "lnc-bbug12558"	transcript_id "lnc-bbut00028541"	exon_number "4"
2	lncRNA	exon	161080195	161080654	.	+	.	gene_id "lnc-bbug12558"	transcript_id "lnc-bbut00028541"	exon_number "5"
2	lncRNA	exon	161071736	161071803	.	+	.	gene_id "lnc-bbug12558"	transcript_id "lnc-bbut00028540"	exon_number "1"
2	lncRNA	exon	161072395	161072553	.	+	.	gene_id "lnc-bbug12558"	transcript_id "lnc-bbut00028540"	exon_number "2"
2	lncRNA	exon	161078938	161079071	.	+	.	gene_id "lnc-bbug12558"	transcript_id "lnc-bbut00028540"	exon_number "3"
2	lncRNA	exon	161079547	161079735	.	+	.	gene_id "lnc-bbug12558"	transcript_id "lnc-bbut00028540"	exon_number "4"
2	lncRNA	exon	161080195	161080654	.	+	.	gene_id "lnc-bbug12558"	transcript_id "lnc-bbut00028540"	exon_number "5"
2	lncRNA	exon	161208403	161209407	.	+	.	gene_id "lnc-bbug12559"	transcript_id "lnc-bbut00028543"	exon_number "1"
2	lncRNA	exon	161313230	161313894	.	+	.	gene_id "lnc-bbug12560"	transcript_id "lnc-bbut00028544"	exon_number "1"
2	lncRNA	exon	161578786	161578829	.	+	.	gene_id "lnc-bbug12561"	transcript_id "lnc-bbut00028545"	exon_number "1"
2	lncRNA	exon	161579254	161579776	.	+	.	gene_id "lnc-bbug12561"	transcript_id "lnc-bbut00028545"	exon_number "2"
2	lncRNA	exon	161580581	161580754	.	+	.	gene_id "lnc-bbug12561"	transcript_id "lnc-bbut00028545"	exon_number "3"
2	lncRNA	exon	164027127	164028030	.	+	.	gene_id "lnc-bbug12566"	transcript_id "lnc-bbut00028551"	exon_number "1"
2	lncRNA	exon	164196833	164197033	.	+	.	gene_id "lnc-bbug12568"	transcript_id "lnc-bbut00028554"	exon_number "1"
2	lncRNA	exon	164198098	164198479	.	+	.	gene_id "lnc-bbug12568"	transcript_id "lnc-bbut00028554"	exon_number "2"
2	lncRNA	exon	164199865	164200305	.	+	.	gene_id "lnc-bbug12568"	transcript_id "lnc-bbut00028554"	exon_number "3"
2	lncRNA	exon	164196833	164197033	.	+	.	gene_id "lnc-bbug12568"	transcript_id "lnc-bbut00028553"	exon_number "1"
2	lncRNA	exon	164198098	164198231	.	+	.	gene_id "lnc-bbug12568"	transcript_id "lnc-bbut00028553"	exon_number "2"
2	lncRNA	exon	164199865	164200305	.	+	.	gene_id "lnc-bbug12568"	transcript_id "lnc-bbut00028553"	exon_number "3"
2	lncRNA	exon	164400832	164400890	.	+	.	gene_id "lnc-bbug12570"	transcript_id "lnc-bbut00028556"	exon_number "1"
2	lncRNA	exon	164401340	164401405	.	+	.	gene_id "lnc-bbug12570"	transcript_id "lnc-bbut00028556"	exon_number "2"
2	lncRNA	exon	164499347	164501454	.	+	.	gene_id "lnc-bbug12570"	transcript_id "lnc-bbut00028556"	exon_number "3"
2	lncRNA	exon	164766351	164766501	.	+	.	gene_id "lnc-bbug12571"	transcript_id "lnc-bbut00028557"	exon_number "1"
2	lncRNA	exon	164776424	164776513	.	+	.	gene_id "lnc-bbug12571"	transcript_id "lnc-bbut00028557"	exon_number "2"
2	lncRNA	exon	164779513	164779685	.	+	.	gene_id "lnc-bbug12571"	transcript_id "lnc-bbut00028557"	exon_number "3"
2	lncRNA	exon	165756117	165756230	.	+	.	gene_id "lnc-bbug12573"	transcript_id "lnc-bbut00028559"	exon_number "1"
2	lncRNA	exon	165756673	165756715	.	+	.	gene_id "lnc-bbug12573"	transcript_id "lnc-bbut00028559"	exon_number "2"
2	lncRNA	exon	165758403	165761656	.	+	.	gene_id "lnc-bbug12573"	transcript_id "lnc-bbut00028559"	exon_number "3"
2	lncRNA	exon	168023965	168024087	.	+	.	gene_id "lnc-bbug12575"	transcript_id "lnc-bbut00028563"	exon_number "1"
2	lncRNA	exon	168028309	168030546	.	+	.	gene_id "lnc-bbug12575"	transcript_id "lnc-bbut00028563"	exon_number "2"
2	lncRNA	exon	168772178	168772569	.	+	.	gene_id "lnc-bbug12581"	transcript_id "lnc-bbut00028596"	exon_number "1"
2	lncRNA	exon	168775581	168775675	.	+	.	gene_id "lnc-bbug12581"	transcript_id "lnc-bbut00028596"	exon_number "2"
2	lncRNA	exon	168776948	168777051	.	+	.	gene_id "lnc-bbug12581"	transcript_id "lnc-bbut00028596"	exon_number "3"
2	lncRNA	exon	168777651	168777813	.	+	.	gene_id "lnc-bbug12581"	transcript_id "lnc-bbut00028596"	exon_number "4"

2	lncRNA	exon	168780928	168781036	.	+	.	gene_id "lnc-bbug12581"	transcript_id "lnc-bbut00028596"	exon_number "5"
2	lncRNA	exon	168782430	168782501	.	+	.	gene_id "lnc-bbug12581"	transcript_id "lnc-bbut00028596"	exon_number "6"
2	lncRNA	exon	168783012	168784447	.	+	.	gene_id "lnc-bbug12581"	transcript_id "lnc-bbut00028596"	exon_number "7"
2	lncRNA	exon	168772178	168772569	.	+	.	gene_id "lnc-bbug12581"	transcript_id "lnc-bbut00028595"	exon_number "1"
2	lncRNA	exon	168775581	168775675	.	+	.	gene_id "lnc-bbug12581"	transcript_id "lnc-bbut00028595"	exon_number "2"
2	lncRNA	exon	168776948	168777051	.	+	.	gene_id "lnc-bbug12581"	transcript_id "lnc-bbut00028595"	exon_number "3"
2	lncRNA	exon	168777651	168777813	.	+	.	gene_id "lnc-bbug12581"	transcript_id "lnc-bbut00028595"	exon_number "4"
2	lncRNA	exon	168780928	168781036	.	+	.	gene_id "lnc-bbug12581"	transcript_id "lnc-bbut00028595"	exon_number "5"
2	lncRNA	exon	168782430	168784447	.	+	.	gene_id "lnc-bbug12581"	transcript_id "lnc-bbut00028595"	exon_number "6"
2	lncRNA	exon	168974704	168974872	.	+	.	gene_id "lnc-bbug12583"	transcript_id "lnc-bbut00028598"	exon_number "1"
2	lncRNA	exon	168976007	168976121	.	+	.	gene_id "lnc-bbug12583"	transcript_id "lnc-bbut00028598"	exon_number "2"
2	lncRNA	exon	168976837	168976914	.	+	.	gene_id "lnc-bbug12583"	transcript_id "lnc-bbut00028598"	exon_number "3"
2	lncRNA	exon	168977740	168978216	.	+	.	gene_id "lnc-bbug12583"	transcript_id "lnc-bbut00028598"	exon_number "4"
2	lncRNA	exon	168974705	168974872	.	+	.	gene_id "lnc-bbug12583"	transcript_id "lnc-bbut00028599"	exon_number "1"
2	lncRNA	exon	168976010	168976121	.	+	.	gene_id "lnc-bbug12583"	transcript_id "lnc-bbut00028599"	exon_number "2"
2	lncRNA	exon	168976837	168976914	.	+	.	gene_id "lnc-bbug12583"	transcript_id "lnc-bbut00028599"	exon_number "3"
2	lncRNA	exon	168977740	168978216	.	+	.	gene_id "lnc-bbug12583"	transcript_id "lnc-bbut00028599"	exon_number "4"
2	lncRNA	exon	172411521	172412769	.	+	.	gene_id "lnc-bbug12600"	transcript_id "lnc-bbut00028666"	exon_number "1"
2	lncRNA	exon	172412845	172413001	.	+	.	gene_id "lnc-bbug12600"	transcript_id "lnc-bbut00028666"	exon_number "2"
2	lncRNA	exon	172414176	172414675	.	+	.	gene_id "lnc-bbug12600"	transcript_id "lnc-bbut00028666"	exon_number "3"
2	lncRNA	exon	172579337	172579783	.	+	.	gene_id "lnc-bbug12603"	transcript_id "lnc-bbut00028672"	exon_number "1"
2	lncRNA	exon	173683093	173684749	.	+	.	gene_id "lnc-bbug12618"	transcript_id "lnc-bbut00028704"	exon_number "1"
2	lncRNA	exon	173685449	173685492	.	+	.	gene_id "lnc-bbug12618"	transcript_id "lnc-bbut00028704"	exon_number "2"
2	lncRNA	exon	173687474	173687649	.	+	.	gene_id "lnc-bbug12618"	transcript_id "lnc-bbut00028704"	exon_number "3"
2	lncRNA	exon	173689652	173692354	.	+	.	gene_id "lnc-bbug12618"	transcript_id "lnc-bbut00028704"	exon_number "4"
2	lncRNA	exon	173805666	173809218	.	+	.	gene_id "lnc-bbug12621"	transcript_id "lnc-bbut00028710"	exon_number "1"
2	lncRNA	exon	174564619	174564759	.	+	.	gene_id "lnc-bbug12631"	transcript_id "lnc-bbut00028729"	exon_number "1"
2	lncRNA	exon	174579249	174579948	.	+	.	gene_id "lnc-bbug12631"	transcript_id "lnc-bbut00028729"	exon_number "2"
2	lncRNA	exon	174582145	174582237	.	+	.	gene_id "lnc-bbug12631"	transcript_id "lnc-bbut00028729"	exon_number "3"
2	lncRNA	exon	174586123	174586253	.	+	.	gene_id "lnc-bbug12631"	transcript_id "lnc-bbut00028729"	exon_number "4"
2	lncRNA	exon	174588629	174591175	.	+	.	gene_id "lnc-bbug12631"	transcript_id "lnc-bbut00028729"	exon_number "5"
2	lncRNA	exon	174610978	174611363	.	+	.	gene_id "lnc-bbug12632"	transcript_id "lnc-bbut00028731"	exon_number "1"
2	lncRNA	exon	174635198	174635361	.	+	.	gene_id "lnc-bbug12632"	transcript_id "lnc-bbut00028731"	exon_number "2"
2	lncRNA	exon	174636518	174637860	.	+	.	gene_id "lnc-bbug12632"	transcript_id "lnc-bbut00028731"	exon_number "3"
2	lncRNA	exon	174610978	174611363	.	+	.	gene_id "lnc-bbug12632"	transcript_id "lnc-bbut00028732"	exon_number "1"
2	lncRNA	exon	174635198	174635361	.	+	.	gene_id "lnc-bbug12632"	transcript_id "lnc-bbut00028732"	exon_number "2"
2	lncRNA	exon	174636518	174636618	.	+	.	gene_id "lnc-bbug12632"	transcript_id "lnc-bbut00028732"	exon_number "3"
2	lncRNA	exon	174653930	174654462	.	+	.	gene_id "lnc-bbug12632"	transcript_id "lnc-bbut00028732"	exon_number "4"
2	lncRNA	exon	174631856	174632583	.	+	.	gene_id "lnc-bbug12632"	transcript_id "lnc-bbut00028733"	exon_number "1"
2	lncRNA	exon	174635198	174635361	.	+	.	gene_id "lnc-bbug12632"	transcript_id "lnc-bbut00028733"	exon_number "2"
2	lncRNA	exon	174636518	174637860	.	+	.	gene_id "lnc-bbug12632"	transcript_id "lnc-bbut00028733"	exon_number "3"
2	lncRNA	exon	175093682	175093972	.	+	.	gene_id "lnc-bbug12639"	transcript_id "lnc-bbut00028760"	exon_number "1"
2	lncRNA	exon	175111506	175111765	.	+	.	gene_id "lnc-bbug12639"	transcript_id "lnc-bbut00028760"	exon_number "2"
2	lncRNA	exon	176521770	176522710	.	+	.	gene_id "lnc-bbug12646"	transcript_id "lnc-bbut00028769"	exon_number "1"
2	lncRNA	exon	176523264	176524902	.	+	.	gene_id "lnc-bbug12646"	transcript_id "lnc-bbut00028769"	exon_number "2"
2	lncRNA	exon	178062970	178066553	.	+	.	gene_id "lnc-bbug12660"	transcript_id "lnc-bbut00028797"	exon_number "1"
2	lncRNA	exon	178215971	178216109	.	+	.	gene_id "lnc-bbug12662"	transcript_id "lnc-bbut00028805"	exon_number "1"
2	lncRNA	exon	178217666	178217764	.	+	.	gene_id "lnc-bbug12662"	transcript_id "lnc-bbut00028805"	exon_number "2"
2	lncRNA	exon	178224430	178225135	.	+	.	gene_id "lnc-bbug12662"	transcript_id "lnc-bbut00028805"	exon_number "3"
2	lncRNA	exon	179194274	179194475	.	+	.	gene_id "lnc-bbug12677"	transcript_id "lnc-bbut00028843"	exon_number "1"
2	lncRNA	exon	179200540	179202543	.	+	.	gene_id "lnc-bbug12677"	transcript_id "lnc-bbut00028843"	exon_number "2"
2	lncRNA	exon	179437286	179438757	.	+	.	gene_id "lnc-bbug12678"	transcript_id "lnc-bbut00028844"	exon_number "1"

2	lncRNA	exon	179480249	179480865	.	+	.	gene_id "lnc-bbug12679"	transcript_id "lnc-bbut00028845"	exon_number "1"
2	lncRNA	exon	180051826	180052149	.	+	.	gene_id "lnc-bbug12689"	transcript_id "lnc-bbut00028874"	exon_number "1"
2	lncRNA	exon	180053519	180053624	.	+	.	gene_id "lnc-bbug12689"	transcript_id "lnc-bbut00028874"	exon_number "2"
2	lncRNA	exon	180302120	180303137	.	+	.	gene_id "lnc-bbug12691"	transcript_id "lnc-bbut00028879"	exon_number "1"
2	lncRNA	exon	180303693	180306005	.	+	.	gene_id "lnc-bbug12691"	transcript_id "lnc-bbut00028879"	exon_number "2"
2	lncRNA	exon	180302120	180303128	.	+	.	gene_id "lnc-bbug12691"	transcript_id "lnc-bbut00028878"	exon_number "1"
2	lncRNA	exon	180303693	180306005	.	+	.	gene_id "lnc-bbug12691"	transcript_id "lnc-bbut00028878"	exon_number "2"
2	lncRNA	exon	180338038	180338393	.	+	.	gene_id "lnc-bbug12692"	transcript_id "lnc-bbut00028880"	exon_number "1"
2	lncRNA	exon	180338609	180339015	.	+	.	gene_id "lnc-bbug12692"	transcript_id "lnc-bbut00028880"	exon_number "2"
2	lncRNA	exon	180929663	180930631	.	+	.	gene_id "lnc-bbug12697"	transcript_id "lnc-bbut00028891"	exon_number "1"
2	lncRNA	exon	180930743	180930787	.	+	.	gene_id "lnc-bbug12697"	transcript_id "lnc-bbut00028891"	exon_number "2"
2	lncRNA	exon	180931766	180931803	.	+	.	gene_id "lnc-bbug12697"	transcript_id "lnc-bbut00028891"	exon_number "3"
2	lncRNA	exon	181223948	181224021	.	+	.	gene_id "lnc-bbug12698"	transcript_id "lnc-bbut00028892"	exon_number "1"
2	lncRNA	exon	181226378	181226470	.	+	.	gene_id "lnc-bbug12698"	transcript_id "lnc-bbut00028892"	exon_number "2"
2	lncRNA	exon	181237722	181238172	.	+	.	gene_id "lnc-bbug12698"	transcript_id "lnc-bbut00028892"	exon_number "3"
2	lncRNA	exon	181429555	181429666	.	+	.	gene_id "lnc-bbug12700"	transcript_id "lnc-bbut00028895"	exon_number "1"
2	lncRNA	exon	181438052	181438306	.	+	.	gene_id "lnc-bbug12700"	transcript_id "lnc-bbut00028895"	exon_number "2"
2	lncRNA	exon	181455224	181455273	.	+	.	gene_id "lnc-bbug12700"	transcript_id "lnc-bbut00028895"	exon_number "3"
2	lncRNA	exon	181864152	181865821	.	+	.	gene_id "lnc-bbug12706"	transcript_id "lnc-bbut00028913"	exon_number "1"
2	lncRNA	exon	182536668	182539947	.	+	.	gene_id "lnc-bbug12718"	transcript_id "lnc-bbut00028949"	exon_number "1"
2	lncRNA	exon	182650837	182651136	.	+	.	gene_id "lnc-bbug12719"	transcript_id "lnc-bbut00028950"	exon_number "1"
2	lncRNA	exon	182653055	182653980	.	+	.	gene_id "lnc-bbug12719"	transcript_id "lnc-bbut00028950"	exon_number "2"
2	lncRNA	exon	183628078	183628710	.	+	.	gene_id "lnc-bbug12722"	transcript_id "lnc-bbut00028955"	exon_number "1"
2	lncRNA	exon	183628846	183630157	.	+	.	gene_id "lnc-bbug12722"	transcript_id "lnc-bbut00028955"	exon_number "2"
2	lncRNA	exon	184844284	184844391	.	+	.	gene_id "lnc-bbug12733"	transcript_id "lnc-bbut00029015"	exon_number "1"
2	lncRNA	exon	184844533	184845090	.	+	.	gene_id "lnc-bbug12733"	transcript_id "lnc-bbut00029015"	exon_number "2"
[truncated: 2,844,598 more chars]
